# Next-generation IEDB tools: a platform for epitope prediction and analysis

**DOI:** 10.1093/nar/gkae407

**Published:** 2024-05-23

**Authors:** Zhen Yan, Kevin Kim, Haeuk Kim, Brendan Ha, Anaïs Gambiez, Jason Bennett, Marcus Fabiano de Almeida Mendes, Raphael Trevizani, Jarjapu Mahita, Eve Richardson, Daniel Marrama, Nina Blazeska, Zeynep Koşaloğlu-Yalçın, Morten Nielsen, Alessandro Sette, Bjoern Peters, Jason A Greenbaum

**Affiliations:** Bioinformatics Core, La Jolla Institute for Immunology, La Jolla, CA 92037, USA; Information Technology, La Jolla Institute for Immunology, La Jolla, CA 92037, USA; Bioinformatics Core, La Jolla Institute for Immunology, La Jolla, CA 92037, USA; Bioinformatics Core, La Jolla Institute for Immunology, La Jolla, CA 92037, USA; Bioinformatics Core, La Jolla Institute for Immunology, La Jolla, CA 92037, USA; Center for Vaccine Innovation, La Jolla Institute for Immunology, La Jolla, CA 92037, USA; Center for Vaccine Innovation, La Jolla Institute for Immunology, La Jolla, CA 92037, USA; Center for Vaccine Innovation, La Jolla Institute for Immunology, La Jolla, CA 92037, USA; Fiocruz Ceará, Fundação Oswaldo Cruz, Rua São José s/n, Precabura, Eusébio/CE, Brazil; Center for Vaccine Innovation, La Jolla Institute for Immunology, La Jolla, CA 92037, USA; Center for Vaccine Innovation, La Jolla Institute for Immunology, La Jolla, CA 92037, USA; Center for Vaccine Innovation, La Jolla Institute for Immunology, La Jolla, CA 92037, USA; Center for Vaccine Innovation, La Jolla Institute for Immunology, La Jolla, CA 92037, USA; Center for Vaccine Innovation, La Jolla Institute for Immunology, La Jolla, CA 92037, USA; Department of Health Technology, Technical University of Denmark, DK-2800 Kgs, Lyngby, Denmark; Instituto de Investigaciones Biotecnológicas, Universidad Nacional de San Martín, B1650 Buenos Aires, Argentina; Center for Vaccine Innovation, La Jolla Institute for Immunology, La Jolla, CA 92037, USA; Department of Medicine, University of California San Diego, La Jolla, CA 92093, USA; Center for Vaccine Innovation, La Jolla Institute for Immunology, La Jolla, CA 92037, USA; Department of Medicine, University of California San Diego, La Jolla, CA 92093, USA; Bioinformatics Core, La Jolla Institute for Immunology, La Jolla, CA 92037, USA

## Abstract

The Next-Generation (NG) IEDB Tools website (https://nextgen-tools.iedb.org) provides users with a redesigned interface to many of the algorithms for epitope prediction and analysis that were originally released on the legacy IEDB Tools website. The initial release focuses on consolidation of all tools related to HLA class I epitopes (MHC binding, elution, immunogenicity, and processing), making all of these predictions accessible from a single application and allowing for their simultaneous execution with minimal user inputs. Additionally, the PEPMatch tool for identifying highly similar epitopes in a set of curated proteomes, as well as a tool for epitope clustering, are available on the site. The NG Tools site allows users to build data pipelines by sending the output of one tool as input for the next. Over the next several years, all pre-existing IEDB Tools, and any newly developed tools, will be integrated into this new site. Here we describe the philosophy behind the redesign and demonstrate the utility and productivity enhancements that are enabled by the new interface.

## Graphical Abstract

**Graphical Abstract:**
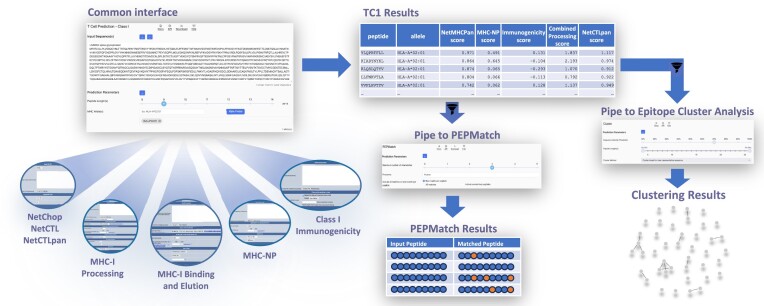

## Introduction

The Immune Epitope Database-Analysis Resource (IEDB-AR) (1) provides tools to the immunology community related to the prediction and analysis of T and B cell epitopes. It serves as a companion site to the Immune Epitope Database (IEDB) (2), which catalogs experimental data that is used to train the tools in the IEDB-AR. The IEDB-AR has grown in scope and usage over ∼20 years and now serves over 600 users daily. Here, we present a complete redesign of the website, dubbed the Next Generation IEDB Tools (NG Tools, https://nextgen-tools.iedb.org), which addresses frequent user requests to make the tools more consistent in their interface and reduce the number of user interactions (clicks) it takes to run common predictions across multiple tools and combine their results. The NG Tools site focuses on reimplementing the interfaces (web, API, and standalone) to the pre-existing algorithms rather than developing novel ones. This website is free and open to all users, and there is no login requirement.

### Motivations and goals

A recurring challenge for IEDB-AR users has been the need for an integrated tool set with improved navigation and functionality, including the ability to share workflows for publications or otherwise. To date, users have had to use different tools across multiple web pages to integrate prediction results and complete their analysis objectives. Once completed, there is no direct way to share the results. To address these issues, the Next-Generation Tools site focuses on creating a streamlined, consistent, and intuitive user interface. The NG Tools enables users to create reusable workflows to run datasets through several tools in succession, where the output of a given tool can be filtered and sent to the next tool as input. These data pipelines can be configured from any of the available tools with a consistent interface across each. Additionally, the platform has improved capabilities to accommodate large-scale datasets and achieve results in a reasonable time frame. Apart from some minor differences in rounding precision, results obtained by tools that exist on both the legacy and NG Tools sites are consistent (Supplemental Table S2).

## The approach

### Overall vision

The NG Tools should allow new users, and those without a bioinformatics background, to get to work instantly and also provide the functionality needed for power users to perform complex workflows. Therefore, it has been imperative to strike an appropriate balance between streamlined usability and high functionality. We set out to achieve this through utilizing already familiar and reliable systems for the bioinformatics community, such as REST APIs (representational state transfer application programming interfaces) and JSON (JavaScript Object Notation) data formats. Although not directly used by the NG Tools, some inspiration was drawn from the way Jupyter (3) notebooks function under Python. Overall, we believe the NG Tools improve significantly upon the legacy Analysis Resource, as significant effort was placed on simplicity while extending functionality.

### Interface design

The NG Tools interface has been designed with the assistance of external usability experts to ensure that our goal of intuitive simplicity is achieved. NG Tools require minimal input, and a prediction can be run with a single click from the home page using pre-populated example inputs. All user inputs are validated as early in the process as possible and, if issues are discovered, user-friendly warning messages are returned to ensure the prediction will run smoothly. NG Tools also provides input flexibility, allowing users to run analyses on proteins or peptide lists in different formats, such as a FASTA or JSON file. Uniformity is key to NG; therefore, the input formats and validations are consistent across all NG Tools applications, from the website to the API and standalone tools.

Once a prediction has been completed, NG Tools returns outputs in an intuitive and interactive format. Users are able to review their results in tables where each column is described, and sorting and filtering capabilities are provided. Other tool-specific graphical visualizations are available for users, such as an interactive plot for the Epitope Cluster Tool.

### Key features

#### Integration and consolidation of tools

The legacy IEDB Tools site has tools spread across almost 30 different pages, many of which include numerous prediction and analysis methods. While designing NG Tools, we took this opportunity to create tool suites in which related tools with similar inputs and outputs are consolidated. For example, the T Cell Prediction, Class I (TC1) tools suite, which was made available in the first release of NG Tools, is a set of tools related to peptide:MHC binding, elution, immunogenicity, and processing. These tools were separate webpages on the legacy site, with varying user interfaces. They have now been consolidated in NG Tools to simplify the interface for users and allow for simultaneous prediction against a common input data set.

#### Reduction in input complexity

**Figure 1.**
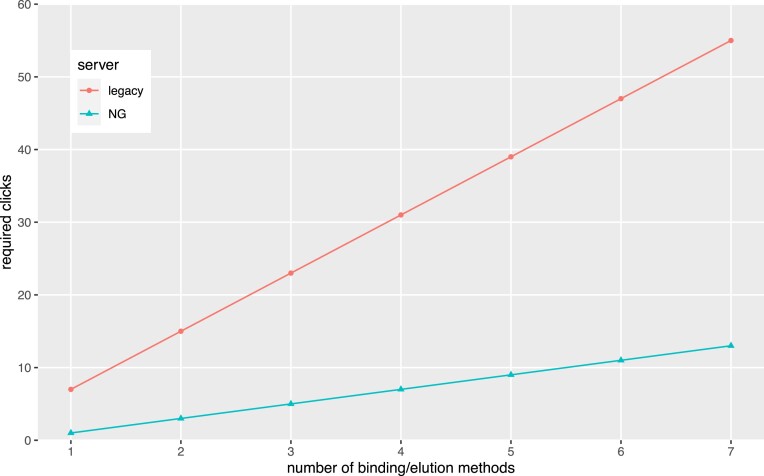
Required clicks as a function of the number of selected binding/elution methods on the legacy and NG sites.

An important goal for NG Tools is that users could get from the landing page to prediction results with minimal interaction. This was partially enabled by the consolidation effort mentioned above as a user can provide one set of inputs to run predictions with multiple tools. To further reduce complexity, input controls were simplified and sensible defaults have been pre-populated. This allows a user to go from the landing page to prediction results in a single click with the example sequence. Once users are on the pipeline/results page, more controls are available to fine-tune predictions. Despite providing more controls to users on this page, it is remarkably efficient when compared to the legacy site. In most cases, the number of clicks to run any of the individual tools that are common between the sites has been reduced by half or more (Supplemental Table S1). Figure 1 compares the numbers of clicks required on the legacy and NG sites as a function of the number of selected MHC I binding/elution methods in the TC1 tool and illustrates that the discrepancy becomes more pronounced as more methods are added.

Adding MHC processing and immunogenicity predictors, as well as additional peptide lengths, further highlights the difference. For instance, a prediction of the example sequence with NetMHCPan EL 4.1 for peptide lengths 8 through 11 requires 19 clicks on the legacy site as opposed to just 3 on NG Tools. Further, whereas one would need to navigate through multiple pages and make 23 additional clicks on the legacy site to add MHC class I processing and immunogenicity predictions, it requires only 4 additional clicks on NG Tools without navigating away from the TC1 page. In addition to the time savings and reduction in number of necessary interactions, the laborious and error-prone effort of consolidating outputs from different tools is eliminated.

#### Prediction pipelines

Perhaps the most impactful feature of the NG tools, in terms of productivity, is the ability to create data pipelines by sending the output from one tool as the input to the next. Below, we demonstrate the ability to make such user-created pipelines reusable and shareable, and also highlight how the IEDB team can host curated pipelines reflecting best practices in a transparent fashion.

The idea of running a set of predictors in series on a given dataset to form a pipeline is not novel. However, before the implementation of the NG Tools, sending data through the IEDB tools would have been a manual, error-prone process and/or would have required a programmer. In contrast, in the NG Tools, a graphical interface provides user-friendly access to immuno-informatics pipelines. This design paradigm will allow greater control for users to customize their analyses to their specific needs and eventually alleviate the need for less flexible, multi-step form-based applications such as TepiTool (4).

#### Sharable links to results and parameter configurations

In order to assist with sharing of prediction workflows and enhance reproducibility, the NG Tools include two types of shareable links. A ‘pipeline’ link is available that will correspond to the current state of the pipeline, including all parameters, pipeline steps, inputs, and outputs. As each change to a pipeline (e.g., step added or removed, table filtered) generates a new URL, all intermediate pipeline states are saved and can be shared. These links enable sharing a snapshot of the entire pipeline, including their input data and results. Anyone that accesses this link can load the pipeline and will see the same view as the pipeline creator.

A separate link to the ‘pipeline configuration’ is also available and includes only the pipeline parameters and steps, without any data (input or output) attached. These links can be shared to repeat workflows on new datasets. Both types of links are available from the clipboard in the ‘Pipeline Map’ on the left side of the application. It should be noted that email addresses will never be revealed when these links are shared, regardless of whether an email was supplied upon pipeline submission.

These two links further reduce the number of user interactions required to obtain a meaningful prediction, as all of the work to build the pipeline needs to be done only once. When starting from one of these links, the user simply needs to type or paste their input sequence(s) and click ‘Run All Above’.

#### Interactive results

In addition to the thousands of rows that may be returned for a given set of inputs, many of the methods include a multitude of columns in their results tables, which can be overwhelming. We have attempted to address these issues by reducing the number of columns that are displayed by default and embedding several interactive controls in the tables. Users can filter based on their column(s) of interest, choose which columns to display for each prediction method, save new table states for any changes that have been made, or reset the table to its original state. Rows can be filtered by clicking the ‘filter’ icons in the column header where numeric fields can be limited to a range and text fields can be limited to specific strings. The 'Display Columns' button opens a window with controls to show or hide columns, as well as a description of each. For the antigen processing methods, as well as the Cluster tool, interactive plots are also available on a separate results tab. The results can be downloaded as a TSV, CSV, or JSON file, or shared via a link to enable collaboration in the scientific community. Users who provide an email address will receive an email once the predictions have been completed with a link back to the pipeline where they can review the results.

#### Documentation system

NG Tools has implemented a documentation system using Sphinx (5), which provides an overview of site usage, an in-depth guide of each available tool, a description of pipeline creation and sharing, details on API usage, as well as troubleshooting details and a privacy notice. The help documents will be continually updated as new tools are implemented and new user questions arise. The NG Tools API is documented as an OpenAPI page rendered via Swagger UI (6), allowing users an interactive and guided approach to accessing the API.

#### Standalone tools

Each of the tools that are implemented into NG Tools have a corresponding command line (i.e., standalone) version. Through whichever route the prediction request is acquired, it ultimately runs through the standalone code. This system helps to support the goal of running consistent predictions on the website, API, and standalone. Each of the standalone tools has a common interface, with several input parameters, formats, and validations that are consistent across all tools. Each tool may extend that interface with additional, tool-specific options.

Wrapping up the scientific code into a standalone tool with a common interface has several benefits. First, it affords freedoms for the scientific developers to implement algorithms with few restrictions on programming languages or libraries used. Second, it helps to minimize the cognitive overhead of users who wish to use the tool by providing consistent command-line parameters and input/output file formats across the entire suite of tools. By enforcing a common JSON input format, the same inputs that are used by the web interface and API may also be used with the standalones, which assists with reproducibility across the different systems.

### Anatomy of a prediction request

**Figure 2.**
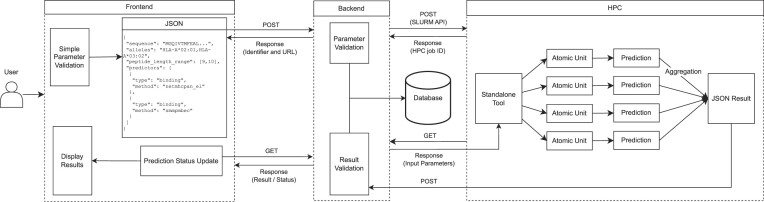
Anatomy of a prediction request. This schematic depicts the flow of data for a prediction request that begins through the frontend web interface. Beginning the moment that the user submits the job, the frontend periodically checks the prediction status until it has completed.

All prediction requests run through the same process (Figure 2). Before they are sent to the backend, the frontend runs basic parameter validation. Next, the parameters are formatted as JSON and sent through the backend API. The caller, the frontend in this case, immediately receives a response with an identifier and a URL. As the backend receives the request, a more robust set of parameter checks occur. If an error or warning is generated, the frontend will receive the response quickly to convey to the user. Rather than directly returning the results of the prediction request, the caller is provided with an identifier and a URL where the prediction results will be located once it is complete. This workflow allows for submitting large prediction requests without the risk of HTTP connection timeouts.

After the initial validation passes, the NG API sends a request to the High Performance Cluster (HPC) system through the Slurm API to begin processing the job. A custom library on the HPC will retrieve all necessary inputs for data processing from a central database. If the request is parallelizable, it will be split into chunks that are efficient for processing and distributed across the cluster by logic that is built into each of the standalone tools. Once the individual predictions have been completed, an aggregation job pulls together the individual outputs and re-composes them into a JSON result object that is inserted into the database. Once the job is marked as completed in the database, the results will be available at the URL provided in the initial POST response. If an error occurred during runtime, this will also be returned to the caller.

## Release 1.2

The NG Tools website went public in March 2023 and has seen two successive minor releases since that time. In this section, we briefly discuss the latest release, version 1.2. The latest release of NG Tools builds upon the initial release (1.0) and its successor (1.1), which pushed all computation to the HPC. This release includes three major tool suites, each of which is described in more detail below. Results from any of the current tool suites can be filtered and piped as input to any of the other tools to build data pipelines.

### T cell prediction, class I

The T Cell Prediction, Class I (TC1) application bundles all tools related to class I peptide:MHC binding, elution, immunogenicity, and processing predictions into a single, unified interface. As most of the individual tools can accept similar inputs (e.g., protein or peptide sequences) and provide similarly shaped outputs (e.g., one row per peptide), they were well-suited for consolidation. There are 8 methods available for peptide:MHC binding/elution prediction and, as with the legacy tools site, NetMHCPan 4.1 (7) is the current recommendation. MHCFlurry 2.0 (8), which is not available on the legacy site, has also been integrated into this tool. Class I immunogenicity (9) and antigen processing methods (Basic, NetChop, NetCTL, NetCTLPan) (10–13) can be individually selected as well and a prediction can be run simultaneously for all predictors.

Global inputs that affect each of the selected predictors are found near the top of the page. This design pattern is consistent across the NG Tools site. For TC1, these include the input sequences (proteins or peptides in various formats), peptide lengths, and MHC alleles. In contrast to the legacy tools site, the selection of peptide lengths and MHC alleles is decoupled from the method selection. As described above, this allows for parameter selection with many fewer user interactions, particularly as more parameter combinations are selected. The noted downside to this approach is that parameter validation, in terms of whether the selected parameters are compatible, is delayed until the user clicks ‘run’. However, users will receive warnings nearly instantly upon an invalid parameter submission and we believe that this tradeoff is acceptable as it addresses one of the most frequently cited frustrations with the legacy site.

As far as data pipelines go, this tool can accept peptide lists as input from Cluster and PEPMatch and can pass peptide lists for these tools to consume. The TC1 tool will be used to generate binding, elution, immunogenicity, and processing predictions for each peptide followed by filtering and sending to downstream tools for post-processing/redundancy removal.

### PEPMatch

PEPMatch (14) is a tool to efficiently and exhaustively compare a list of peptides to a database and identify hits with high sequence identity. In contrast to tools like BLAST (15), it has been optimized for short peptides and has 100% sensitivity. Unlike TC1, this application provides only one method at present. Inputs include the peptide list, a proteome selector, and the maximum number of mismatches to report as a hit. Eight mammalian proteomes are selectable with plans to incorporate curated viral and bacterial proteomes in the future, as well as the ability for users to upload custom proteomes.

As a step in a data pipeline, PEPMatch can be used to prefilter a peptide list for those that share high similarity with a given species or conversely to filter for those that do not. This tool can accept peptide lists as piped input and can pipe its peptide lists to downstream tools.

### Cluster

The Cluster tool (16) has been ported from the legacy site into NG Tools as a separate application. This takes a list of peptides as input, along with a sequence identity threshold and will group the peptides into clusters based on one of three different levels of stringency. In addition to the tabular output, an interactive visualization of the clustered peptides is available in a separate tab.

In a data pipeline, the cluster tool could be used as a redundancy removal step to prefilter a peptide list or downstream to consolidate results. The tool will accept piped peptide data from any tool that exports that data type and can pipe peptides or consensus sequences to downstream tools.

### Solving biological-scale problems

One of the goals of the NG Tools project was to ensure the site could handle real-world, biological-scale problems and complete them in an acceptable time frame. Several design decisions are directed at achieving this goal. For instance, the decision for the API to provide pointers to results as a response rather than the results themselves allows for the submission of large-scale datasets without the risk of HTTP timeouts. The standalone tools that parallelize jobs and use the HPC backend help to ensure that jobs are broken down into smaller units that are most efficient for processing. Additionally, the ability to submit an email address to be notified when results are complete is helpful for those predictions that might run for more than a few minutes.

With all of these pieces in place, the NG site is capable of handling much larger inputs and outputs than the legacy site. For instance, the legacy site is unable to display results in tabular format for >10,000 peptides and will not return any results for inputs of >100,000 amino acids. In contrast, the NG Tools site supports running and displaying results for inputs of up to 1,000,000 amino acids. Additionally, since multiple predictors can be run simultaneously, particularly in the case of the TC1 tool, users do not need to spend time manually merging outputs from different tools. Aside from the significant time savings, this also reduces the likelihood of errors stemming from human data manipulation.

Although at the time of writing the NG Tools site generally lags the legacy site in terms of speed of returning results (data not shown), particularly when input sequences are short and the number of alleles is low, we are currently evaluating inefficiencies that can be addressed to close the performance gap.

### An example pipeline for epitope prioritization

**Figure 3.**
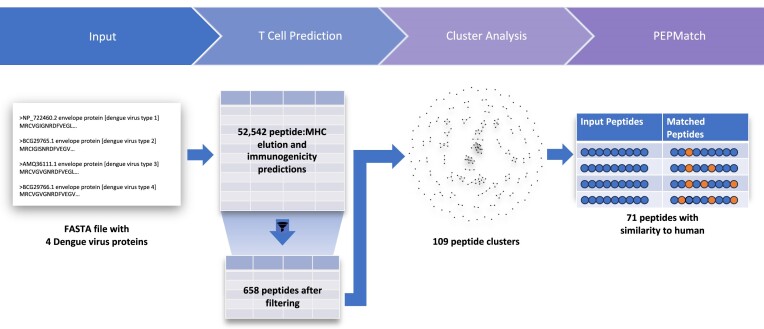
Example epitope prioritization pipeline. A high-level overview of the data flow through the example pipeline.

Here we illustrate the value of pipelines with a simple use case. Imagine we are tasked with predicting a set of broadly recognized T cell epitopes derived from envelope proteins of four Dengue serotypes. This workflow (Figure 3) would naturally start with the TC1 tool by entering the four protein sequences and selecting NetMHCPan 4.1 EL to predict elution and Immunogenicity to predict the likelihood of T cell recognition. From the ‘allele selector’, the reference panel of 27 HLA class I alleles (17) would be selected. After clicking ‘run’, the results table contains 52,542 rows and we can filter for NetMHCPan EL percentile rank ⇐ 1 and Immunogenicity score > 0. The 658 rows that remain after filtering are next piped to the Cluster tool to group the peptides into clusters and assist with redundancy removal. Next, the Cluster results table is filtered down to 109 cluster consensus sequences and singletons that are then piped to PEPMatch. PEPMatch yields 71 sequences that are similar to human with three or less mismatches. Depending upon the goals of this study, this information can be used to stratify the Dengue peptides by their similarity to human, which may have an effect on observed immunogenicity (18). This example workflow, particularly the TC1 portion can be extended to include additional binding, elution, or processing methods with filters set appropriately on each column to ultimately arrive at a consensus prediction. Once the workflow has been completed, the researcher could save the URL to recover the results, rerun after adjusting settings, or repeat the workflow on an entirely new dataset. For reference, the example workflow above is available at https://nextgen-tools.iedb.org/nar-2024-example.

## Summary and future plans

NG Tools is a multi-stage project with the goal of re-implementing all of the IEDB’s most heavily used tools with an interface that addresses frequent user requests and allows for higher productivity. Looking forward, the next release will include a migration of PepX (19) from the legacy site, a new tool to compare MHC binding predictions of wild-type and mutant peptides, and a new tool to predict peptides that may exist as a result of somatic variants. Following releases will focus on implementing the T cell class II suite and migrating the most utilized B cell tools available on the legacy site including BepiPred (20), Discotope (21) and Lyra (22). Intervening releases targeting bug fixes and performance improvements will make their way to our production servers as issues are addressed.

Additional planned functionality includes maintaining a set of curated pipelines to serve as templates for best-practices, developing client-side libraries for interacting with the NG Tools APIs, and providing an export of the complete NG Tools system for licensees to run on their own hardware. With its streamlined interface, new functionalities, and the broad array of tools and features planned for implementation, the NG Tools aims to build on the established algorithms of the legacy IEDB Tools site and serve as a centralized analysis resource for the immuno-informatics community.

## Supplementary Material

gkae407_Supplemental_File

## Data Availability

The Next-Generation IEDB Tools (web, API, and standalone) are freely available at https://nextgen-tools.iedb.org.
