# Supplementary material for: Next-generation IEDB tools: a platform for epitope prediction and analysis
Source: Nucleic Acids Res. 2024 May 23;52(W1):W526–32. doi: 10.1093/nar/gkae407 (PMC11223806; doi:10.1093/nar/gkae407)
Supplement: gkae407_Supplemental_File [file gkae407_supplemental_file.pdf]

SUPPLEMENTAL TECHNICAL DETAILS

Application Architecture and Technology Stack

Separation of Frontend and Backend. The operation of the NG Tools is based on the coordination between a variety of components, each of which serves a particular purpose. For instance, the web interface is the main entry point for users but offloads all incoming requests through the API. The API handles requests from both the web interface as well as API users and submits jobs for execution on the high-performance cluster (HPC) backend. There, a custom library executes each of the standalone tools, splitting parallelizable jobs into smaller components before aggregating the individual outputs and posting the results back to the database. All the systems are based upon open-source software and are technologies that are widely used in the field. Here, we describe each of the individual components that make up the NG Tools in a bit more detail.

Web Interface. The user-facing frontend interface is implemented as an Angular (1) web app. Angular is an open source, Typescript-based frontend framework that allows us to easily develop, build, and test our frontend code. The framework is led by the Angular Team at Google and by a large community of developers. Using a frontend framework like Angular helps to separate out backend and frontend logic thus helping developers to scale and update their apps more efficiently. Results tables are rendered with DataTables (2), which is a JavaScript library for adding interactive features and optimizing the performance of HTML tables. Using DataTables, we are able to filter and sort thousands of rows that contain multiple columns nearly instantly. The built-in sorting, filtering, and table state functionalities helped us to develop the Pipeline feature so that users can send results as inputs to the next tool in the pipeline. Additional technologies used by the frontend are Bootstrap 5 (3) for styling, D3 (4) for visualizations, and Google Analytics for monitoring usage patterns for users that opt in.

Python/Django API. To support both the web application and API users, the Django REST framework (5) was used to build out the backend system. By serving as a common interface for all predictions, we are able to ensure a consistent experience for all users, regardless of which method is used to submit a request. The Django REST framework adheres to RESTful principles,

offering a standardized approach for interacting with the NG Tools through HTTP methods (e.g., GET, POST). The body of each request and their responses, both formatted as JSON, aim to be consistent across all endpoints. Upon submission of a request, initial validations are run to ensure the data are formatted properly and all required fields are present. If this validation fails, the caller will receive a response that includes an actionable error message. As requests pass validation, the submitted data structures are automatically serialized into JSON representations, keyed with a request ID, and stored in a centralized database. The Django REST framework also supports authentication and authorization through OAuth2. Although API users will never need to provide credentials, these capabilities are used internally to prevent unauthorized users from accessing restricted endpoints. All of the endpoints are documented for end users using the drf-yasg (6) package to produce an OpenAPI 2.0 document that is served through Swagger UI.

The entire backend is supported by a Postgres database that is managed through Django's Object-Relational Mapper (ORM). The system is built around the concept of 'objects', which are stored in the 'object' table. A variety of object types are currently supported, including 'peptide list', 'protein list', and 'results table'. Each object can be linked to one or more requests / predictions and undergoes object-specific validation.

High Performance Compute Cluster Backend. An HPC, composed of 10 nodes, each with 24 cores and 72GB of memory supports all computation. It is configured with the Slurm scheduling software (7) and a common fileystem. Jobs are sent to this system by the NG API server through the Slurm REST API. Once received, a custom module will pull all needed inputs to run the job and employ the standalone tools to break them up into atomic units, submit for execution, and aggregate results.

Testing and Quality Assurance

For the NG Tools project, tests are widely used to help identify and fix errors, improve code quality, and ensure that the end product meets the requirements and expectations of the users.

Unit tests are used to check small components to ensure functionality. Django also provides a test-execution framework and assorted utilities that allow us to test at a higher level by simulating requests, inserting test data, and inspecting the application's output. End-to-end tests are automated with Playwright (8), which is a cross-platform web test automation library and is used to test the IEDB NG Tools web and API interfaces. All the different levels of testing were integrated with GitLab CI/CD (9). The testing and its automation aid in discovering issues before they make their way to our production systems.

In addition to code testing, we validated that we were able to obtain identical results between the two sites for all tools that are in common (Supplemental Table 2). The test datasets for each method are listed below. In all cases, default settings were used and outputs are reported in a supplemental spreadsheet. Of the 14 methods tested, 11 matched exactly between the two sites. The remaining 3 (Comblib_Sidney2008, MHC-NP, and Basic Processing) differed only very slightly due to rounding differences between the reports produced on the two sites.

Test datasets

Binding and elution test input:

```
>SARS Spike protein
MFIFLLFLTLTSGSDLDRCTTFDDVQAPNYTQHTSSMRGVYYPDEIFRSDTLYLTQDLFL
PFYSNVTGFHTINHTFGNPVIPFKDGIYFAATEKSNVVRGWVFGSTMNNKSQSVIIINNS
TNVVIRACNFELCDNPFFAVSKPMGTQTHTMIFDNAFNCTFEYISDAFSLDVSEKSGNFK
HLREFVFKNKDGFLYVYKGYQPIDVVRDLPSGFNTLKPIFKLPLGINITNFRAILTAFSP
AQDIWGTSAAAYFVGYLKPTTFMLKYDENGTITDAVDCSQNPLAELKCSVKSFEIDKGIY
QTSNFRVVPSGDVVRFPNITNLCPFGEVFNATKFPSVYAWERKKISNCVADYSVLYNSTF
FSTFKCYGVSATKLNDLCFSNVYADSFVVKGDDVRQIAPGQTGVIADYNYKLPDDFMGCV
LAWNTRNIDATSTGNYNYKYRYLRHGKLRPFERDISNVPFSPDGKPCTPPALNCYWPLND
YGFYTTTGIGYQPYRVVVLSFELLNAPATVCGPKLSTDLIKNQCVNFNFNGLTGTGVLTP
SSKRFQPFQQFGRDVSDFTDSVRDPKTSEILDISPCSFGGVSVITPGTNASSEVAVLYQD
VNCTDVSTAIHADQLTPAWRIYSTGNNVFQTQAGCLIGAEHVDTSYECDIPIGAGICASY
HTVSLLRSTSQKSIVAYTMSLGADSSIAYSNNTIAIPTNFSISITTEVMPVSMAKTSVDC
NMYICGDSTECANLLLQYGSFCTQLNRALSGIAAEQDRNTREVFAQVKQMYKTPTLKYFG
GFNFSQILPDPLKPTKRSFIEDLLFNKVTLADAGFMKQYGECLGDINARDLICAQKFNGL
TVLPPLLTDDMIAAYTAALVSGTATAGWTFGAGAALQIPFAMQMAYRFNGIGVTQNVLYE
NQKQIANQFNKAISQIQESLTTTSTALGKLQDVVNQNAQALNTLVKQLSSNFGAISSVLN
DILSRLDKVEAEVQIDRLITGRLQSLQTYVTQQLIRAAEIRASANLAATKMSECVLGQSK
RVDFCGKGYHLMSFPQAAPHGVVFLHVTYVPSQERNFTTAPAICHEGKAYFPREGVFVFN
GTSWFITQRNFFSPQIITTDNTFVSGNCDVVIGIINNTVYDPLQPELDSFKEELDKYFKN
HTSPDVDLGDISGINASVVNIQKEIDRLNEVAKNLNESLIDLQELGKYEQYIKWPWYVWL
GFIAGLIAIVMVTILLCCMTSCCSCLKGACSCGSCCKFDEDDSEPVLKGVKLHYT
```

Immunogenicity test input:
FIAGLIAIV
LITGRLQSL
RLNEVAKNL
KAVYNFATC
FQPQNGQFI

Cluster test input:
>Mus Pep1
LEQIHVLENSLVL
>Mus Pep2
FVEHIHVLENSLAFK
>Mus Pep3
GLYGREPDLSSDIKERFA
>Mus Pep4
EWFSILLASDKREKI
>Mus Pep5
DGFNTFTILKTDYDN
>Mus Pep6
NEKDGETFQLMGLY
>Mus Pep7
YGREPDLSLDIKEK
>Mus Pep8
LENSLVLKFHTVRDE
>Mus Pep9
AHLINEKDGETFQLM
>Mus Pep10
EEASSTGRNFNVQKINGEWHTIIL
>Mus Pep11
EKALVSSVRQRMKCS
>Mus Pep12
SRPFIFQEVIDLGGE
>Mus Pep13
NVEKINGEWHTIIL
>Mus Pep14
GIYYLNYDGFNTFTI
>Mus Pep15
DDVVASEALNSVWSGF
>Mus Pep16
GSMRVFVEHIHVLEN
>Mus Pep17
ENIIDLTKTNRCLKA
>Mus Pep18
ILGKLVKDYHLQFHR
>Mus Pep19
IGGPDDGVITPWQSSF
>Mus Pep20
DKETLSLEELKALLL
>Mus Pep21
LQSGFYSLSSLVTVP

>Mus Pep22
EELRRLAPITSDPTE
>Mus Pep23
TIFISLFLLSVCYSA
>Rat Pep1
ASNKREKIEENGSMRVFMQH
>Rat Pep2
DIKEKFAKLCEAHGITRDNI
>Rat Pep3
EEASSTRGNLDVAKLNGDWF
>Rat Pep4
EENGSMRVFMQHIDVLENSL
>Rat Pep5
ENSLGFKFRIKENGECRELY
>Rat Pep6
FMQHIDVLENSLGFKFRIKE
>Rat Pep7
FVEYDGGNTFTILKTDYDRY
>Rat Pep8
GDWFSIVVASNKREKIEENG
>Rat Pep9
GRTKDLSSDIKEKFAKLCEA
>Rat Pep10
KTPEDGEYFVEYDGGNTFTI
>Rat Pep11
LCEAHGITRDNIIDLTKTDR
>Rat Pep12
LINFKNGETFQLMVLYGRTK
>Rat Pep13
NLDVAKLNGDWFSIVVASNK
>Rat Pep14
RDNIIDLTKTDRCLQARG
>Rat Pep15
RELYLVAYKTPEDGEYFVEY
>Rat Pep16
RIKENGECRELYLVAYKTPE
>Rat Pep17
TFQLMVLYGRTKDLSSDIKE
>Rat Pep18
TFTILKTDYDRYVMFHLINF
>Rat Pep19
YDRYVMFHLINFKNGETFQL

References

1. Angular Development Team Angular.

2. DataTables Development Team DataTables.

3. Bootstrap Development Team Bootstrap.

4. D3 Development Team D3.

5. Django REST Framework Development Team Django REST Framework.

6. drf-yasg Development Team drf-yasg.

7. Jette,M.A. and Wickberg,T. (2023) Architecture of the Slurm Workload Manager. *Lecture Notes in Computer Science (including subseries Lecture Notes in Artificial Intelligence and Lecture Notes in Bioinformatics)*, **14283 LNCS**, 3–23.

https://doi.org/10.1007/978-3-031-43943-8_1/FIGURES/6

8. Playwright Development Team Playwright.

9. Gitlab Development Team Gitlab.

SUPPLEMENTAL TABLES

Supplemental Table 1. Minimum number of clicks to run the example data through each of the tools that are common between the legacy and NG websites.

Tool	Legacy clicks	NG clicks	Net difference
MHC I binding/elution	7	1	-6
MHC I processing	7	3	-4
NetChop/NetCTL/NetCTLPan	3	5	2
MHC-NP	7	3	-4
Immunogenicity	3	3	0
Cluster	5	1	-4
**median**	6	3	-4

Supplemental Table 2 (on following pages). Validating consistency in results between the legacy and NG sites. Results from running the test datasets through each of the 14 methods on both the legacy and NG sites are included in this multi-schema table. In addition to the method outputs, columns are added that compare the values between the two result sets. For 11 of the 14 methods, the values are exactly equivalent. For the remaining 3 (Comblib_Sidney2008, MHC-NP, and Basic Processing) some of the values differ very slightly due to rounding differences between the reporting formats.

seq #	peptide	start	end	peptide length	allele	peptide index	median binding	netmhcpan_el	netmhcpan_el	netmhcpan_el	netmhcpan_el	legacy score	legacy rank	scores equal	percentiles equal
1	VLNDILSRL	958	966	9	HLA-A*02:01	958	0.03	VLNDILSRL	VLNDILSRL	0.938498	0.03	0.938498	0.03	TRUE	TRUE
1	YLKPTTFML	256	264	9	HLA-A*02:01	256	0.03	YLKPTTFML	YLKPTTFML	0.925604	0.03	0.925604	0.03	TRUE	TRUE
1	RLQSLQTYV	982	990	9	HLA-A*02:01	982	0.05	RLQSLQTYV	RLQSLQTYV	0.87376	0.05	0.87376	0.05	TRUE	TRUE
1	RLDKVEAEV	965	973	9	HLA-A*02:01	965	0.06	RLDKVEAEV	RLDKVEAEV	0.825045	0.06	0.825045	0.06	TRUE	TRUE
1	LLFNKVTLA	803	811	9	HLA-A*02:01	803	0.08	LLFNKVTLA	LLFNKVTLA	0.803506	0.08	0.803506	0.08	TRUE	TRUE
1	VIADYNYKL	404	412	9	HLA-A*02:01	404	0.09	VIADYNYKL	VIADYNYKL	0.77259	0.09	0.77259	0.09	TRUE	TRUE
1	VVFLHVTYV	1042	1050	9	HLA-A*02:01	1042	0.11	VVFLHVTYV	VVFLHVTYV	0.74167	0.11	0.74167	0.11	TRUE	TRUE
1	VLYENQKQI	897	905	9	HLA-A*02:01	897	0.13	VLYENQKQI	VLYENQKQI	0.698692	0.13	0.698692	0.13	TRUE	TRUE
1	HLMSFPQAA	1030	1038	9	HLA-A*02:01	1030	0.13	HLMSFPQAA	HLMSFPQAA	0.693337	0.13	0.693337	0.13	TRUE	TRUE
1	KQMYKTPTL	768	776	9	HLA-A*02:01	768	0.14	KQMYKTPTL	KQMYKTPTL	0.679794	0.14	0.679794	0.14	TRUE	TRUE
1	ALNTLVKQL	940	948	9	HLA-A*02:01	940	0.16	ALNTLVKQL	ALNTLVKQL	0.657403	0.16	0.657403	0.16	TRUE	TRUE
1	RLNEVAKNL	1167	1175	9	HLA-A*02:01	1167	0.16	RLNEVAKNL	RLNEVAKNL	0.652653	0.16	0.652653	0.16	TRUE	TRUE
1	FIAGLIAIV	1202	1210	9	HLA-A*02:01	1202	0.17	FIAGLIAIV	FIAGLIAIV	0.641405	0.17	0.641405	0.17	TRUE	TRUE
1	GLTVLPPLL	839	847	9	HLA-A*02:01	839	0.18	GLTVLPPLL	GLTVLPPLL	0.622173	0.18	0.622173	0.18	TRUE	TRUE
1	NLNESLIDL	1174	1182	9	HLA-A*02:01	1174	0.18	NLNESLIDL	NLNESLIDL	0.618877	0.18	0.618877	0.18	TRUE	TRUE
1	ALVSGTATA	858	866	9	HLA-A*02:01	858	0.26	ALVSGTATA	ALVSGTATA	0.492248	0.26	0.492248	0.26	TRUE	TRUE
1	FSISITTEV	700	708	9	HLA-A*02:01	700	0.27	FSISITTEV	FSISITTEV	0.490881	0.27	0.490881	0.27	TRUE	TRUE
1	TMNNKSQSV	106	114	9	HLA-A*02:01	106	0.27	TMNNKSQSV	TMNNKSQSV	0.484911	0.27	0.484911	0.27	TRUE	TRUE
1	LLTDDMIAA	846	854	9	HLA-A*02:01	846	0.32	LLTDDMIAA	LLTDDMIAA	0.443474	0.32	0.443474	0.32	TRUE	TRUE
1	KLPDDFMGC	411	419	9	HLA-A*02:01	411	0.35	KLPDDFMGC	KLPDDFMGC	0.408859	0.35	0.408859	0.35	TRUE	TRUE
1	ELLNAPATV	502	510	9	HLA-A*02:01	502	0.44	ELLNAPATV	ELLNAPATV	0.355502	0.44	0.355502	0.44	TRUE	TRUE
1	QIAPGQTGV	396	404	9	HLA-A*02:01	396	0.44	QIAPGQTGV	QIAPGQTGV	0.353593	0.44	0.353593	0.44	TRUE	TRUE
1	IIINNSTNV	115	123	9	HLA-A*02:01	115	0.48	IIINNSTNV	IIINNSTNV	0.335589	0.48	0.335589	0.48	TRUE	TRUE
1	SIVAYTMSL	673	681	9	HLA-A*02:01	673	0.5	SIVAYTMSL	SIVAYTMSL	0.325955	0.5	0.325955	0.5	TRUE	TRUE
1	AISQIQESL	912	920	9	HLA-A*02:01	912	0.58	AISQIQESL	AISQIQESL	0.280929	0.58	0.280929	0.58	TRUE	TRUE
1	VLSFELLNA	498	506	9	HLA-A*02:01	498	0.61	VLSFELLNA	VLSFELLNA	0.268326	0.61	0.268326	0.61	TRUE	TRUE
1	SLTTTSTAL	919	927	9	HLA-A*02:01	919	0.61	SLTTTSTAL	SLTTTSTAL	0.26725	0.61	0.26725	0.61	TRUE	TRUE
1	YVYKGYQPI	195	203	9	HLA-A*02:01	195	0.68	YVYKGYQPI	YVYKGYQPI	0.235587	0.68	0.235587	0.68	TRUE	TRUE
1	MIAAYTAAL	851	859	9	HLA-A*02:01	851	0.68	MIAAYTAAL	MIAAYTAAL	0.234789	0.68	0.234789	0.68	TRUE	TRUE
1	NVYADSFVV	381	389	9	HLA-A*02:01	381	0.74	NVYADSFVV	NVYADSFVV	0.215408	0.74	0.215408	0.74	TRUE	TRUE
1	RIYSTGNNV	620	628	9	HLA-A*02:01	620	0.75	RIYSTGNNV	RIYSTGNNV	0.21518	0.75	0.21518	0.75	TRUE	TRUE
1	SITTEVMPV	703	711	9	HLA-A*02:01	703	0.75	SITTEVMPV	SITTEVMPV	0.214767	0.75	0.214767	0.75	TRUE	TRUE
1	NTLKPIFKL	214	222	9	HLA-A*02:01	214	0.75	NTLKPIFKL	NTLKPIFKL	0.212951	0.75	0.212951	0.75	TRUE	TRUE
1	VYDPLQPEL	1119	1127	9	HLA-A*02:01	1119	0.8	VYDPLQPEL	VYDPLQPEL	0.196623	0.8	0.196623	0.8	TRUE	TRUE
1	YQPYRVVVL	491	499	9	HLA-A*02:01	491	0.84	YQPYRVVVL	YQPYRVVVL	0.189014	0.84	0.189014	0.84	TRUE	TRUE
1	ALGKLQDVV	926	934	9	HLA-A*02:01	926	0.91	ALGKLQDVV	ALGKLQDVV	0.173341	0.91	0.173341	0.91	TRUE	TRUE
1	LQIPFAMQM	876	884	9	HLA-A*02:01	876	0.99	LQIPFAMQM	LQIPFAMQM	0.157345	0.99	0.157345	0.99	TRUE	TRUE
1	ELDSFKEEL	1126	1134	9	HLA-A*02:01	1126	1.1	ELDSFKEEL	ELDSFKEEL	0.14897	1.1	0.14897	1.1	TRUE	TRUE
1	ILPDPLKPT	787	795	9	HLA-A*02:01	787	1.1	ILPDPLKPT	ILPDPLKPT	0.142014	1.1	0.142014	1.1	TRUE	TRUE
1	FMKQYGECL	815	823	9	HLA-A*02:01	815	1.1	FMKQYGECL	FMKQYGECL	0.138019	1.1	0.138019	1.1	TRUE	TRUE
1	GINASVVNI	1153	1161	9	HLA-A*02:01	1153	1.1	GINASVVNI	GINASVVNI	0.137188	1.1	0.137188	1.1	TRUE	TRUE
1	GIGYQPYRV	488	496	9	HLA-A*02:01	488	1.2	GIGYQPYRV	GIGYQPYRV	0.133399	1.2	0.133399	1.2	TRUE	TRUE
1	ILDISPCSF	570	578	9	HLA-A*02:01	570	1.3	ILDISPCSF	ILDISPCSF	0.112157	1.3	0.112157	1.3	TRUE	TRUE
1	VLYQDVNCT	596	604	9	HLA-A*02:01	596	1.3	VLYQDVNCT	VLYQDVNCT	0.11128	1.3	0.11128	1.3	TRUE	TRUE
1	MIFDNAFNC	151	159	9	HLA-A*02:01	151	1.4	MIFDNAFNC	MIFDNAFNC	0.103791	1.4	0.103791	1.4	TRUE	TRUE
1	ELCDNPFFA	131	139	9	HLA-A*02:01	131	1.4	ELCDNPFFA	ELCDNPFFA	0.101191	1.4	0.101191	1.4	TRUE	TRUE
1	VIGIINNTV	1111	1119	9	HLA-A*02:01	1111	1.4	VIGIINNTV	VIGIINNTV	0.100297	1.4	0.100297	1.4	TRUE	TRUE
1	LITGRLQSL	978	986	9	HLA-A*02:01	978	1.4	LITGRLQSL	LITGRLQSL	0.098152	1.4	0.098152	1.4	TRUE	TRUE
1	VLYNSTFFS	354	362	9	HLA-A*02:01	354	1.4	VLYNSTFFS	VLYNSTFFS	0.097551	1.4	0.097551	1.4	TRUE	TRUE
1	GICASYHTV	655	663	9	HLA-A*02:01	655	1.5	GICASYHTV	GICASYHTV	0.094521	1.5	0.094521	1.5	TRUE	TRUE
1	YVTQQLIRA	989	997	9	HLA-A*02:01	989	1.5	YVTQQLIRA	YVTQQLIRA	0.091708	1.5	0.091708	1.5	TRUE	TRUE
1	IITTDNTFV	1096	1104	9	HLA-A*02:01	1096	1.5	IITTDNTFV	IITTDNTFV	0.088901	1.5	0.088901	1.5	TRUE	TRUE
1	KEIDRLNEV	1163	1171	9	HLA-A*02:01	1163	1.5	KEIDRLNEV	KEIDRLNEV	0.087213	1.5	0.087213	1.5	TRUE	TRUE
1	ATAGWTFGA	864	872	9	HLA-A*02:01	864	1.5	ATAGWTFGA	ATAGWTFGA	0.086897	1.5	0.086897	1.5	TRUE	TRUE
1	YQTSNFRVV	300	308	9	HLA-A*02:01	300	1.6	YQTSNFRVV	YQTSNFRVV	0.08234	1.6	0.08234	1.6	TRUE	TRUE
1	LQTYVTQQL	986	994	9	HLA-A*02:01	986	1.6	LQTYVTQQL	LQTYVTQQL	0.077489	1.6	0.077489	1.6	TRUE	TRUE
1	GLIAIVMVT	1205	1213	9	HLA-A*02:01	1205	1.6	GLIAIVMVT	GLIAIVMVT	0.076364	1.6	0.076364	1.6	TRUE	TRUE
1	FIFLLFLTL	2	10	9	HLA-A*02:01	2	1.6	FIFLLFLTL	FIFLLFLTL	0.07501	1.6	0.07501	1.6	TRUE	TRUE
1	PLAELKCSV	282	290	9	HLA-A*02:01	282	1.7	PLAELKCSV	PLAELKCSV	0.074482	1.7	0.074482	1.7	TRUE	TRUE
1	FNATKFPSV	329	337	9	HLA-A*02:01	329	1.7	FNATKFPSV	FNATKFPSV	0.071268	1.7	0.071268	1.7	TRUE	TRUE
1	IAIPTNFSI	694	702	9	HLA-A*02:01	694	1.8	IAIPTNFSI	IAIPTNFSI	0.065559	1.8	0.065559	1.8	TRUE	TRUE
1	VLAWNTRNI	420	428	9	HLA-A*02:01	420	1.9	VLAWNTRNI	VLAWNTRNI	0.058034	1.9	0.058034	1.9	TRUE	TRUE
1	RVVVLSFEL	495	503	9	HLA-A*02:01	495	1.9	RVVVLSFEL	RVVVLSFEL	0.057848	1.9	0.057848	1.9	TRUE	TRUE
1	QYIKWPWYV	1190	1198	9	HLA-A*02:01	1190	2	QYIKWPWYV	QYIKWPWYV	0.056776	2	0.056776	2	TRUE	TRUE
1	IINNSTNVV	116	124	9	HLA-A*02:01	116	2.1	IINNSTNVV	IINNSTNVV	0.051177	2.1	0.051177	2.1	TRUE	TRUE
1	YQDVNCTDV	598	606	9	HLA-A*02:01	598	2.1	YQDVNCTDV	YQDVNCTDV	0.048786	2.1	0.048786	2.1	TRUE	TRUE
1	AIVMVTILL	1208	1216	9	HLA-A*02:01	1208	2.2	AIVMVTILL	AIVMVTILL	0.047718	2.2	0.047718	2.2	TRUE	TRUE
1	AISSVLNDI	954	962	9	HLA-A*02:01	954	2.2	AISSVLNDI	AISSVLNDI	0.046737	2.2	0.046737	2.2	TRUE	TRUE
1	FVSGNCDVV	1103	1111	9	HLA-A*02:01	1103	2.2	FVSGNCDVV	FVSGNCDVV	0.046521	2.2	0.046521	2.2	TRUE	TRUE
1	FLLFLTLTS	4	12	9	HLA-A*02:01	4	2.2	FLLFLTLTS	FLLFLTLTS	0.046034	2.2	0.046034	2.2	TRUE	TRUE
1	TQAGCLIGA	631	639	9	HLA-A*02:01	631	2.3	TQAGCLIGA	TQAGCLIGA	0.042522	2.3	0.042522	2.3	TRUE	TRUE
1	AAPHGVVFL	1037	1045	9	HLA-A*02:01	1037	2.3	AAPHGVVFL	AAPHGVVFL	0.042404	2.3	0.042404	2.3	TRUE	TRUE
1	QLNRALSGI	744	752	9	HLA-A*02:01	744	2.4	QLNRALSGI	QLNRALSGI	0.042058	2.4	0.042058	2.4	TRUE	TRUE
1	DLPSGFNTL	208	216	9	HLA-A*02:01	208	2.4	DLPSGFNTL	DLPSGFNTL	0.042007	2.4	0.042007	2.4	TRUE	TRUE
1	YLTQDLFLP	53	61	9	HLA-A*02:01	53	2.4	YLTQDLFLP	YLTQDLFLP	0.040783	2.4	0.040783	2.4	TRUE	TRUE
1	KQLSSNFGA	946	954	9	HLA-A*02:01	946	2.5	KQLSSNFGA	KQLSSNFGA	0.037259	2.5	0.037259	2.5	TRUE	TRUE
1	TALGKLQDV	925	933	9	HLA-A*02:01	925	2.6	TALGKLQDV	TALGKLQDV	0.036029	2.6	0.036029	2.6	TRUE	TRUE
1	NTREVFAQV	759	767	9	HLA-A*02:01	759	2.6	NTREVFAQV	NTREVFAQV	0.035739	2.6	0.035739	2.6	TRUE	TRUE
1	AQKFNGLTV	834	842	9	HLA-A*02:01	834	2.6	AQKFNGLTV	AQKFNGLTV	0.035721	2.6	0.035721	2.6	TRUE	TRUE
1	GTSAAAYFV	246	254	9	HLA-A*02:01	246	2.6	GTSAAAYFV	GTSAAAYFV	0.03495	2.6	0.03495	2.6	TRUE	TRUE
1	DLLFNKVTL	802	810	9	HLA-A*02:01	802	2.6	DLLFNKVTL	DLLFNKVTL	0.034579	2.6	0.034579	2.6	TRUE	TRUE
1	FLPFYSNVT	59	67	9	HLA-A*02:01	59	2.7	FLPFYSNVT	FLPFYSNVT	0.032313	2.7	0.032313	2.7	TRUE	TRUE
1	DVSDFTDSV	554	562	9	HLA-A*02:01	554	2.7	DVSDFTDSV	DVSDFTDSV	0.032199	2.7	0.032199	2.7	TRUE	TRUE
1	LCDNPFFAV	132	140	9	HLA-A*02:01	132	2.7	LCDNPFFAV	LCDNPFFAV	0.032001	2.7	0.032001	2.7	TRUE	TRUE
1	VVIGIINNT	1110	1118	9	HLA-A*02:01	1110	2.8	VVIGIINNT	VVIGIINNT	0.031298	2.8	0.031298	2.8	TRUE	TRUE
1	LIAIVMVTI	1206	1214	9	HLA-A*02:01	1206	2.8	LIAIVMVTI	LIAIVMVTI	0.030623	2.8	0.030623	2.8	TRUE	TRUE
1	SVITPGTNA	582	590	9	HLA-A*02:01	582	2.8	SVITPGTNA	SVITPGTNA	0.030392	2.8	0.030392	2.8	TRUE	TRUE
1	AQDIWGTSA	241	249	9	HLA-A*02:01	241	2.9	AQDIWGTSA	AQDIWGTSA	0.029698	2.9	0.029698	2.9	TRUE	TRUE
1	SVVNIQKEI	1157	1165	9	HLA-A*02:01	1157	2.9	SVVNIQKEI	SVVNIQKEI	0.029493	2.9	0.029493	2.9	TRUE	TRUE
1	AILTAFSPA	233	241	9	HLA-A*02:01	233	3	AILTAFSPA	AILTAFSPA	0.027161	3	0.027161	3	TRUE	TRUE
1	KAYFPREGV	1068	1076	9	HLA-A*02:01	1068	3	KAYFPREGV	KAYFPREGV	0.026617	3	0.026617	3	TRUE	TRUE
1	WTFGAGAAL	868	876	9	HLA-A*02:01	868	3.1	WTFGAGAAL	WTFGAGAAL	0.026042	3.1	0.026042	3.1	TRUE	TRUE
1	VVNQNAQAL	933	941	9	HLA-A*02:01	933	3.2	VVNQNAQAL	VVNQNAQAL	0.024928	3.2	0.024928	3.2	TRUE	TRUE
1	SIAYSNNTI	686	694	9	HLA-A*02:01	686	3.2	SIAYSNNTI	SIAYSNNTI	0.023996	3.2	0.023996	3.2	TRUE	TRUE
1	DILSRLDKV	961	969	9	HLA-A*02:01	961	3.2	DILSRLDKV	DILSRLDKV	0.023845	3.2	0.023845	3.2	TRUE	TRUE
1	AALQIPFAM	874	882	9	HLA-A*02:01	874	3.3	AALQIPFAM	AALQIPFAM	0.023375	3.3	0.023375	3.3	TRUE	TRUE
1	VVVLSFELL	496	504	9	HLA-A*02:01	496	3.3	VVVLSFELL	VVVLSFELL	0.023315	3.3	0.023315	3.3	TRUE	TRUE
1	ASYHTVSLL	658	666	9	HLA-A*02:01	658	3.3	ASYHTVSLL	ASYHTVSLL	0.02288	3.3	0.02288	3.3	TRUE	TRUE
1	ELGKYEQYI	1184	1192	9	HLA-A*02:01	1184	3.3	ELGKYEQYI	ELGKYEQYI	0.022851	3.3	0.022851	3.3	TRUE	TRUE
1	FVFNGTSWF	1077	1085	9	HLA-A*02:01	1077	3.3	FVFNGTSWF	FVFNGTSWF	0.022813	3.3	0.022813	3.3	TRUE	TRUE
1	RVVPSGDVV	306	314	9	HLA-A*02:01	306	3.4	RVVPSGDVV	RVVPSGDVV	0.022151	3.4	0.022151	3.4	TRUE	TRUE
1	SNFGAISSV	950	958	9	HLA-A*02:01	950	3.4	SNFGAISSV	SNFGAISSV	0.021855	3.4	0.021855	3.4	TRUE	TRUE
1	FFSPQIITT	1091	1099	9	HLA-A*02:01	1091	3.5	FFSPQIITT	FFSPQIITT	0.020859	3.5	0.020859	3.5	TRUE	TRUE
1	VLTPSSKRF	537	545	9	HLA-A*02:01	537	3.5	VLTPSSKRF	VLTPSSKRF	0.020051	3.5	0.020051	3.5	TRUE	TRUE
1	DISGINASV	1150	1158	9	HLA-A*02:01	1150	3.6	DISGINASV	DISGINASV	0.019632	3.6	0.019632	3.6	TRUE	TRUE
1	TLYLTQDLF	51	59	9	HLA-A*02:01	51	3.6	TLYLTQDLF	TLYLTQDLF	0.019466	3.6	0.019466	3.6	TRUE	TRUE
1	EQDRNTREV	755	763	9	HLA-A*02:01	755	3.6	EQDRNTREV	EQDRNTREV	0.019216	3.6	0.019216	3.6	TRUE	TRUE
1	VRFPNITNL	314	322	9	HLA-A*02:01	314	3.6	VRFPNITNL	VRFPNITNL	0.019052	3.6	0.019052	3.6	TRUE	TRUE
1	KLPLGINIT	221	229	9	HLA-A*02:01	221	3.6	KLPLGINIT	KLPLGINIT	0.018646	3.6	0.018646	3.6	TRUE	TRUE
1	SLIDLQELG	1178	1186	9	HLA-A*02:01	1178	3.9	SLIDLQELG	SLIDLQELG	0.016217	3.9	0.016217	3.9	TRUE	TRUE
1	LLLQYGSFC	734	742	9	HLA-A*02:01	734	4	LLLQYGSFC	LLLQYGSFC	0.015968	4	0.015968	4	TRUE	TRUE
1	KGYQPIDVV	198	206	9	HLA-A*02:01	198	4	KGYQPIDVV	KGYQPIDVV	0.01587	4	0.01587	4	TRUE	TRUE
1	KLRPFERDI	447	455	9	HLA-A*02:01	447	4	KLRPFERDI	KLRPFERDI	0.01563	4	0.01563	4	TRUE	TRUE
1	NIQKEIDRL	1160	1168	9	HLA-A*02:01	1160	4	NIQKEIDRL	NIQKEIDRL	0.015623	4	0.015623	4	TRUE	TRUE
1	SQIQESLTT	914	922	9	HLA-A*02:01	914	4	SQIQESLTT	SQIQESLTT	0.015508	4	0.015508	4	TRUE	TRUE

seq #	peptide	start	end	peptide length	allele	peptide index	median binding	netmhcpan_el	netmhcpan_el	netmhcpan_el	netmhcpan_el	legacy score	legacy rank	scores equal	percentiles equal
1	KNKDGFLYV	188	196	9	HLA-A*02:01	188	4	KNKDGFLYV	KNKDGFLYV	0.015334	4	0.015334	4	TRUE	TRUE
1	LLNAPATVC	503	511	9	HLA-A*02:01	503	4.2	LLNAPATVC	LLNAPATVC	0.014064	4.2	0.014064	4.2	TRUE	TRUE
1	LFLPFYSNV	58	66	9	HLA-A*02:01	58	4.4	LFLPFYSNV	LFLPFYSNV	0.013126	4.4	0.013126	4.4	TRUE	TRUE
1	QIQESLTTT	915	923	9	HLA-A*02:01	915	4.4	QIQESLTTT	QIQESLTTT	0.012978	4.4	0.012978	4.4	TRUE	TRUE
1	VIRACNFEL	124	132	9	HLA-A*02:01	124	4.4	VIRACNFEL	VIRACNFEL	0.01275	4.4	0.01275	4.4	TRUE	TRUE
1	NGIGVTQNV	889	897	9	HLA-A*02:01	889	4.5	NGIGVTQNV	NGIGVTQNV	0.012498	4.5	0.012498	4.5	TRUE	TRUE
1	GLTGTGVLT	531	539	9	HLA-A*02:01	531	4.5	GLTGTGVLT	GLTGTGVLT	0.012351	4.5	0.012351	4.5	TRUE	TRUE
1	GAALQIPFA	873	881	9	HLA-A*02:01	873	4.5	GAALQIPFA	GAALQIPFA	0.012319	4.5	0.012319	4.5	TRUE	TRUE
1	FAATEKSNV	89	97	9	HLA-A*02:01	89	4.6	FAATEKSNV	FAATEKSNV	0.011666	4.6	0.011666	4.6	TRUE	TRUE
1	AVDCSQNPL	275	283	9	HLA-A*02:01	275	4.6	AVDCSQNPL	AVDCSQNPL	0.01155	4.6	0.01155	4.6	TRUE	TRUE
1	SQKSIVAYT	670	678	9	HLA-A*02:01	670	4.7	SQKSIVAYT	SQKSIVAYT	0.011395	4.7	0.011395	4.7	TRUE	TRUE
1	NLCPFGEVF	321	329	9	HLA-A*02:01	321	4.7	NLCPFGEVF	NLCPFGEVF	0.011141	4.7	0.011141	4.7	TRUE	TRUE
1	CTFEYISDA	159	167	9	HLA-A*02:01	159	4.8	CTFEYISDA	CTFEYISDA	0.010949	4.8	0.010949	4.8	TRUE	TRUE
1	KSFEIDKGI	291	299	9	HLA-A*02:01	291	4.8	KSFEIDKGI	KSFEIDKGI	0.01088	4.8	0.01088	4.8	TRUE	TRUE
1	QIANQFNKA	904	912	9	HLA-A*02:01	904	4.8	QIANQFNKA	QIANQFNKA	0.010863	4.8	0.010863	4.8	TRUE	TRUE
1	QLSSNFGAI	947	955	9	HLA-A*02:01	947	4.8	QLSSNFGAI	QLSSNFGAI	0.010857	4.8	0.010857	4.8	TRUE	TRUE
1	AQVKQMYKT	765	773	9	HLA-A*02:01	765	4.9	AQVKQMYKT	AQVKQMYKT	0.010562	4.9	0.010562	4.9	TRUE	TRUE
1	SMAKTSVDC	712	720	9	HLA-A*02:01	712	4.9	SMAKTSVDC	SMAKTSVDC	0.01046	4.9	0.01046	4.9	TRUE	TRUE
1	TLKPIFKLP	215	223	9	HLA-A*02:01	215	4.9	TLKPIFKLP	TLKPIFKLP	0.010384	4.9	0.010384	4.9	TRUE	TRUE
1	QMAYRFNGI	883	891	9	HLA-A*02:01	883	4.9	QMAYRFNGI	QMAYRFNGI	0.010361	4.9	0.010361	4.9	TRUE	TRUE
1	RAAEIRASA	996	1004	9	HLA-A*02:01	996	4.9	RAAEIRASA	RAAEIRASA	0.010269	4.9	0.010269	4.9	TRUE	TRUE
1	ISDAFSLDV	164	172	9	HLA-A*02:01	164	4.9	ISDAFSLDV	ISDAFSLDV	0.010108	4.9	0.010108	4.9	TRUE	TRUE
1	GFIAGLIAI	1201	1209	9	HLA-A*02:01	1201	5	GFIAGLIAI	GFIAGLIAI	0.009798	5	0.009798	5	TRUE	TRUE
1	ATKFPSVYA	331	339	9	HLA-A*02:01	331	5	ATKFPSVYA	ATKFPSVYA	0.009712	5	0.009712	5	TRUE	TRUE
1	SLGADSSIA	680	688	9	HLA-A*02:01	680	5.1	SLGADSSIA	SLGADSSIA	0.009616	5.1	0.009616	5.1	TRUE	TRUE
1	STFFSTFKC	358	366	9	HLA-A*02:01	358	5.1	STFFSTFKC	STFFSTFKC	0.009461	5.1	0.009461	5.1	TRUE	TRUE
1	RLITGRLQS	977	985	9	HLA-A*02:01	977	5.1	RLITGRLQS	RLITGRLQS	0.009392	5.1	0.009392	5.1	TRUE	TRUE
1	FQTQAGCLI	629	637	9	HLA-A*02:01	629	5.1	FQTQAGCLI	FQTQAGCLI	0.009382	5.1	0.009382	5.1	TRUE	TRUE
1	EIDKGIYQT	294	302	9	HLA-A*02:01	294	5.3	EIDKGIYQT	EIDKGIYQT	0.008686	5.3	0.008686	5.3	TRUE	TRUE
1	PIFKLPLGI	218	226	9	HLA-A*02:01	218	5.4	PIFKLPLGI	PIFKLPLGI	0.008452	5.4	0.008452	5.4	TRUE	TRUE
1	AATEKSNVV	90	98	9	HLA-A*02:01	90	5.4	AATEKSNVV	AATEKSNVV	0.008385	5.4	0.008385	5.4	TRUE	TRUE
1	QTYVTQQLI	987	995	9	HLA-A*02:01	987	5.5	QTYVTQQLI	QTYVTQQLI	0.008154	5.5	0.008154	5.5	TRUE	TRUE
1	YFPREGVFV	1070	1078	9	HLA-A*02:01	1070	5.5	YFPREGVFV	YFPREGVFV	0.008122	5.5	0.008122	5.5	TRUE	TRUE
1	LTDDMIAAY	847	855	9	HLA-A*02:01	847	5.5	LTDDMIAAY	LTDDMIAAY	0.008033	5.5	0.008033	5.5	TRUE	TRUE
1	LLQYGSFCT	735	743	9	HLA-A*02:01	735	5.6	LLQYGSFCT	LLQYGSFCT	0.007895	5.6	0.007895	5.6	TRUE	TRUE
1	IVMVTILLC	1209	1217	9	HLA-A*02:01	1209	5.7	IVMVTILLC	IVMVTILLC	0.007563	5.7	0.007563	5.7	TRUE	TRUE
1	LQDVVNQNA	930	938	9	HLA-A*02:01	930	5.7	LQDVVNQNA	LQDVVNQNA	0.007548	5.7	0.007548	5.7	TRUE	TRUE
1	SISITTEVM	701	709	9	HLA-A*02:01	701	5.7	SISITTEVM	SISITTEVM	0.007473	5.7	0.007473	5.7	TRUE	TRUE
1	IAAYTAALV	852	860	9	HLA-A*02:01	852	5.7	IAAYTAALV	IAAYTAALV	0.007472	5.7	0.007472	5.7	TRUE	TRUE
1	VWLGFIAGL	1198	1206	9	HLA-A*02:01	1198	5.7	VWLGFIAGL	VWLGFIAGL	0.007452	5.7	0.007452	5.7	TRUE	TRUE
1	FLHVTYVPS	1044	1052	9	HLA-A*02:01	1044	5.8	FLHVTYVPS	FLHVTYVPS	0.007378	5.8	0.007378	5.8	TRUE	TRUE
1	SQERNFTTA	1052	1060	9	HLA-A*02:01	1052	5.8	SQERNFTTA	SQERNFTTA	0.007354	5.8	0.007354	5.8	TRUE	TRUE
1	DQLTPAWRI	613	621	9	HLA-A*02:01	613	5.8	DQLTPAWRI	DQLTPAWRI	0.007329	5.8	0.007329	5.8	TRUE	TRUE
1	PLNDYGFYT	477	485	9	HLA-A*02:01	477	5.8	PLNDYGFYT	PLNDYGFYT	0.007265	5.8	0.007265	5.8	TRUE	TRUE
1	HADQLTPAW	611	619	9	HLA-A*02:01	611	5.9	HADQLTPAW	HADQLTPAW	0.007111	5.9	0.007111	5.9	TRUE	TRUE
1	VRDPKTSEI	562	570	9	HLA-A*02:01	562	6	VRDPKTSEI	VRDPKTSEI	0.006853	6	0.006853	6	TRUE	TRUE
1	ALSGIAAEQ	748	756	9	HLA-A*02:01	748	6.1	ALSGIAAEQ	ALSGIAAEQ	0.006665	6.1	0.006665	6.1	TRUE	TRUE
1	FGAGAALQI	870	878	9	HLA-A*02:01	870	6.1	FGAGAALQI	FGAGAALQI	0.006599	6.1	0.006599	6.1	TRUE	TRUE
1	FPQAAPHGV	1034	1042	9	HLA-A*02:01	1034	6.1	FPQAAPHGV	FPQAAPHGV	0.006584	6.1	0.006584	6.1	TRUE	TRUE
1	VFNGTSWFI	1078	1086	9	HLA-A*02:01	1078	6.1	VFNGTSWFI	VFNGTSWFI	0.006461	6.1	0.006461	6.1	TRUE	TRUE
1	CVLGQSKRV	1014	1022	9	HLA-A*02:01	1014	6.2	CVLGQSKRV	CVLGQSKRV	0.006416	6.2	0.006416	6.2	TRUE	TRUE
1	AGLIAIVMV	1204	1212	9	HLA-A*02:01	1204	6.2	AGLIAIVMV	AGLIAIVMV	0.006388	6.2	0.006388	6.2	TRUE	TRUE
1	QQLIRAAEI	992	1000	9	HLA-A*02:01	992	6.2	QQLIRAAEI	QQLIRAAEI	0.006297	6.2	0.006297	6.2	TRUE	TRUE
1	QMYKTPTLK	769	777	9	HLA-A*02:01	769	6.2	QMYKTPTLK	QMYKTPTLK	0.006251	6.2	0.006251	6.2	TRUE	TRUE
1	IKWPWYVWL	1192	1200	9	HLA-A*02:01	1192	6.3	IKWPWYVWL	IKWPWYVWL	0.006196	6.3	0.006196	6.3	TRUE	TRUE
1	GIGVTQNVL	890	898	9	HLA-A*02:01	890	6.3	GIGVTQNVL	GIGVTQNVL	0.006051	6.3	0.006051	6.3	TRUE	TRUE
1	TSYECDIPI	644	652	9	HLA-A*02:01	644	6.4	TSYECDIPI	TSYECDIPI	0.005881	6.4	0.005881	6.4	TRUE	TRUE
1	YICGDSTEC	723	731	9	HLA-A*02:01	723	6.5	YICGDSTEC	YICGDSTEC	0.005754	6.5	0.005754	6.5	TRUE	TRUE
1	VLKGVKLHY	1246	1254	9	HLA-A*02:01	1246	6.5	VLKGVKLHY	VLKGVKLHY	0.00571	6.5	0.00571	6.5	TRUE	TRUE
1	AAAYFVGYL	249	257	9	HLA-A*02:01	249	6.5	AAAYFVGYL	AAAYFVGYL	0.005701	6.5	0.005701	6.5	TRUE	TRUE
1	SLQTYVTQQ	985	993	9	HLA-A*02:01	985	6.5	SLQTYVTQQ	SLQTYVTQQ	0.005645	6.5	0.005645	6.5	TRUE	TRUE
1	KRFQPFQQF	543	551	9	HLA-A*02:01	543	6.5	KRFQPFQQF	KRFQPFQQF	0.00564	6.5	0.00564	6.5	TRUE	TRUE
1	IEDLLFNKV	800	808	9	HLA-A*02:01	800	6.6	IEDLLFNKV	IEDLLFNKV	0.005606	6.6	0.005606	6.6	TRUE	TRUE
1	QILPDPLKP	786	794	9	HLA-A*02:01	786	6.6	QILPDPLKP	QILPDPLKP	0.005526	6.6	0.005526	6.6	TRUE	TRUE
1	TLADAGFMK	809	817	9	HLA-A*02:01	809	6.7	TLADAGFMK	TLADAGFMK	0.005381	6.7	0.005381	6.7	TRUE	TRUE
1	TVYDPLQPE	1118	1126	9	HLA-A*02:01	1118	6.7	TVYDPLQPE	TVYDPLQPE	0.005319	6.7	0.005319	6.7	TRUE	TRUE
1	VMPVSMAKT	708	716	9	HLA-A*02:01	708	6.7	VMPVSMAKT	VMPVSMAKT	0.005279	6.7	0.005279	6.7	TRUE	TRUE
1	FSTFKCYGV	361	369	9	HLA-A*02:01	361	6.8	FSTFKCYGV	FSTFKCYGV	0.005212	6.8	0.005212	6.8	TRUE	TRUE
1	SVLNDILSR	957	965	9	HLA-A*02:01	957	7	SVLNDILSR	SVLNDILSR	0.004883	7	0.004883	7	TRUE	TRUE
1	EVFAQVKQM	762	770	9	HLA-A*02:01	762	7.1	EVFAQVKQM	EVFAQVKQM	0.004799	7.1	0.004799	7.1	TRUE	TRUE
1	LMSFPQAAP	1031	1039	9	HLA-A*02:01	1031	7.1	LMSFPQAAP	LMSFPQAAP	0.004754	7.1	0.004754	7.1	TRUE	TRUE
1	GINITNFRA	225	233	9	HLA-A*02:01	225	7.2	GINITNFRA	GINITNFRA	0.004651	7.2	0.004651	7.2	TRUE	TRUE
1	NVFQTQAGC	627	635	9	HLA-A*02:01	627	7.2	NVFQTQAGC	NVFQTQAGC	0.004636	7.2	0.004636	7.2	TRUE	TRUE
1	STAIHADQL	607	615	9	HLA-A*02:01	607	7.2	STAIHADQL	STAIHADQL	0.004632	7.2	0.004632	7.2	TRUE	TRUE
1	TSVDCNMYI	716	724	9	HLA-A*02:01	716	7.2	TSVDCNMYI	TSVDCNMYI	0.004583	7.2	0.004583	7.2	TRUE	TRUE
1	TQDLFLPFY	55	63	9	HLA-A*02:01	55	7.2	TQDLFLPFY	TQDLFLPFY	0.004557	7.2	0.004557	7.2	TRUE	TRUE
1	QIPFAMQMA	877	885	9	HLA-A*02:01	877	7.2	QIPFAMQMA	QIPFAMQMA	0.004536	7.2	0.004536	7.2	TRUE	TRUE
1	STSQKSIVA	668	676	9	HLA-A*02:01	668	7.2	STSQKSIVA	STSQKSIVA	0.004525	7.2	0.004525	7.2	TRUE	TRUE
1	VTQQLIRAA	990	998	9	HLA-A*02:01	990	7.3	VTQQLIRAA	VTQQLIRAA	0.004453	7.3	0.004453	7.3	TRUE	TRUE
1	GVYYPDEIF	39	47	9	HLA-A*02:01	39	7.3	GVYYPDEIF	GVYYPDEIF	0.004451	7.3	0.004451	7.3	TRUE	TRUE
1	FVGYLKPTT	253	261	9	HLA-A*02:01	253	7.4	FVGYLKPTT	FVGYLKPTT	0.004349	7.4	0.004349	7.4	TRUE	TRUE
1	KLQDVVNQN	929	937	9	HLA-A*02:01	929	7.4	KLQDVVNQN	KLQDVVNQN	0.004311	7.4	0.004311	7.4	TRUE	TRUE
1	YGFYTTTGI	481	489	9	HLA-A*02:01	481	7.4	YGFYTTTGI	YGFYTTTGI	0.004264	7.4	0.004264	7.4	TRUE	TRUE
1	FVFKNKDGF	185	193	9	HLA-A*02:01	185	7.5	FVFKNKDGF	FVFKNKDGF	0.00421	7.5	0.00421	7.5	TRUE	TRUE
1	CSFGGVSVI	576	584	9	HLA-A*02:01	576	7.5	CSFGGVSVI	CSFGGVSVI	0.004136	7.5	0.004136	7.5	TRUE	TRUE
1	IFRSDTLYL	46	54	9	HLA-A*02:01	46	7.5	IFRSDTLYL	IFRSDTLYL	0.004119	7.5	0.004119	7.5	TRUE	TRUE
1	NASSEVAVL	589	597	9	HLA-A*02:01	589	7.6	NASSEVAVL	NASSEVAVL	0.004049	7.6	0.004049	7.6	TRUE	TRUE
1	VMVTILLCC	1210	1218	9	HLA-A*02:01	1210	7.6	VMVTILLCC	VMVTILLCC	0.004039	7.6	0.004039	7.6	TRUE	TRUE
1	ILLCCMTSC	1214	1222	9	HLA-A*02:01	1214	7.8	ILLCCMTSC	ILLCCMTSC	0.003815	7.8	0.003815	7.8	TRUE	TRUE
1	FAVSKPMGT	138	146	9	HLA-A*02:01	138	7.9	FAVSKPMGT	FAVSKPMGT	0.003757	7.9	0.003757	7.9	TRUE	TRUE
1	GQSKRVDFC	1017	1025	9	HLA-A*02:01	1017	7.9	GQSKRVDFC	GQSKRVDFC	0.003698	7.9	0.003698	7.9	TRUE	TRUE
1	CSQNPLAEL	278	286	9	HLA-A*02:01	278	8	CSQNPLAEL	CSQNPLAEL	0.003587	8	0.003587	8	TRUE	TRUE
1	DIWGTSAAA	243	251	9	HLA-A*02:01	243	8.1	DIWGTSAAA	DIWGTSAAA	0.003554	8.1	0.003554	8.1	TRUE	TRUE
1	FAMQMAYRF	880	888	9	HLA-A*02:01	880	8.1	FAMQMAYRF	FAMQMAYRF	0.003517	8.1	0.003517	8.1	TRUE	TRUE
1	LLFLTLTSG	5	13	9	HLA-A*02:01	5	8.1	LLFLTLTSG	LLFLTLTSG	0.003501	8.1	0.003501	8.1	TRUE	TRUE
1	NAQALNTLV	937	945	9	HLA-A*02:01	937	8.1	NAQALNTLV	NAQALNTLV	0.003495	8.1	0.003495	8.1	TRUE	TRUE
1	GNCDVVIGI	1106	1114	9	HLA-A*02:01	1106	8.1	GNCDVVIGI	GNCDVVIGI	0.003479	8.1	0.003479	8.1	TRUE	TRUE
1	DLCFSNVYA	376	384	9	HLA-A*02:01	376	8.2	DLCFSNVYA	DLCFSNVYA	0.003459	8.2	0.003459	8.2	TRUE	TRUE
1	TTFDDVQAP	20	28	9	HLA-A*02:01	20	8.2	TTFDDVQAP	TTFDDVQAP	0.003452	8.2	0.003452	8.2	TRUE	TRUE
1	VAYTMSLGA	675	683	9	HLA-A*02:01	675	8.2	VAYTMSLGA	VAYTMSLGA	0.003445	8.2	0.003445	8.2	TRUE	TRUE
1	SVLYNSTFF	353	361	9	HLA-A*02:01	353	8.2	SVLYNSTFF	SVLYNSTFF	0.003402	8.2	0.003402	8.2	TRUE	TRUE
1	FKDGIYFAA	83	91	9	HLA-A*02:01	83	8.3	FKDGIYFAA	FKDGIYFAA	0.003336	8.3	0.003336	8.3	TRUE	TRUE
1	SNVTGFHTI	64	72	9	HLA-A*02:01	64	8.3	SNVTGFHTI	SNVTGFHTI	0.00333	8.3	0.00333	8.3	TRUE	TRUE
1	KMSECVLGQ	1010	1018	9	HLA-A*02:01	1010	8.4	KMSECVLGQ	KMSECVLGQ	0.003197	8.4	0.003197	8.4	TRUE	TRUE
1	STDLIKNQC	516	524	9	HLA-A*02:01	516	8.4	STDLIKNQC	STDLIKNQC	0.003197	8.4	0.003197	8.4	TRUE	TRUE
1	IAIVMVTIL	1207	1215	9	HLA-A*02:01	1207	8.5	IAIVMVTIL	IAIVMVTIL	0.003109	8.5	0.003109	8.5	TRUE	TRUE
1	KQIANQFNK	903	911	9	HLA-A*02:01	903	8.5	KQIANQFNK	KQIANQFNK	0.003104	8.5	0.003104	8.5	TRUE	TRUE
1	NITNFRAIL	227	235	9	HLA-A*02:01	227	8.5	NITNFRAIL	NITNFRAIL	0.003102	8.5	0.003102	8.5	TRUE	TRUE
1	VQIDRLITG	973	981	9	HLA-A*02:01	973	8.6	VQIDRLITG	VQIDRLITG	0.003028	8.6	0.003028	8.6	TRUE	TRUE
1	DEDDSEPVL	1239	1247	9	HLA-A*02:01	1239	8.6	DEDDSEPVL	DEDDSEPVL	0.003024	8.6	0.003024	8.6	TRUE	TRUE
1	QFNKAISQI	908	916	9	HLA-A*02:01	908	8.6	QFNKAISQI	QFNKAISQI	0.003009	8.6	0.003009	8.6	TRUE	TRUE
1	ESLIDLQEL	1177	1185	9	HLA-A*02:01	1177	8.6	ESLIDLQEL	ESLIDLQEL	0.002996	8.6	0.002996	8.6	TRUE	TRUE
1	VGYLKPTTF	254	262	9	HLA-A*02:01	254	8.8	VGYLKPTTF	VGYLKPTTF	0.002884	8.8	0.002884	8.8	TRUE	TRUE
1	KCSVKSFEI	287	295	9	HLA-A*02:01	287	8.8	KCSVKSFEI	KCSVKSFEI	0.002882	8.8	0.002882	8.8	TRUE	TRUE
1	DMIAAYTAA	850	858	9	HLA-A*02:01	850	8.9	DMIAAYTAA	DMIAAYTAA	0.002846	8.9	0.002846	8.9	TRUE	TRUE

seq #	peptide	start	end	peptide length	allele	peptide index	median binding	netmhcpan_el	netmhcpan_el	netmhcpan_el	netmhcpan_el	legacy score	legacy rank	scores equal	percentiles equal
1	VAKNLNESL	1171	1179	9	HLA-A*02:01	1171	8.9	VAKNLNESL	VAKNLNESL	0.002833	8.9	0.002833	8.9	TRUE	TRUE
1	NSTNVVIRA	119	127	9	HLA-A*02:01	119	8.9	NSTNVVIRA	NSTNVVIRA	0.002821	8.9	0.002821	8.9	TRUE	TRUE
1	YTAALVSGT	855	863	9	HLA-A*02:01	855	9	YTAALVSGT	YTAALVSGT	0.002786	9	0.002786	9	TRUE	TRUE
1	QIITTDNTF	1095	1103	9	HLA-A*02:01	1095	9	QIITTDNTF	QIITTDNTF	0.002755	9	0.002755	9	TRUE	TRUE
1	ICAQKFNGL	832	840	9	HLA-A*02:01	832	9	ICAQKFNGL	ICAQKFNGL	0.002752	9	0.002752	9	TRUE	TRUE
1	STGNNVFQT	623	631	9	HLA-A*02:01	623	9	STGNNVFQT	STGNNVFQT	0.002738	9	0.002738	9	TRUE	TRUE
1	CASYHTVSL	657	665	9	HLA-A*02:01	657	9.1	CASYHTVSL	CASYHTVSL	0.002696	9.1	0.002696	9.1	TRUE	TRUE
1	IGYQPYRVV	489	497	9	HLA-A*02:01	489	9.2	IGYQPYRVV	IGYQPYRVV	0.002626	9.2	0.002626	9.2	TRUE	TRUE
1	QPIDVVRDL	201	209	9	HLA-A*02:01	201	9.2	QPIDVVRDL	QPIDVVRDL	0.002613	9.2	0.002613	9.2	TRUE	TRUE
1	MSLGADSSI	679	687	9	HLA-A*02:01	679	9.2	MSLGADSSI	MSLGADSSI	0.002593	9.2	0.002593	9.2	TRUE	TRUE
1	AEIRASANL	998	1006	9	HLA-A*02:01	998	9.3	AEIRASANL	AEIRASANL	0.002573	9.3	0.002573	9.3	TRUE	TRUE
1	NTIAIPTNF	692	700	9	HLA-A*02:01	692	9.3	NTIAIPTNF	NTIAIPTNF	0.002498	9.3	0.002498	9.3	TRUE	TRUE
1	SVIIINNST	113	121	9	HLA-A*02:01	113	9.4	SVIIINNST	SVIIINNST	0.002436	9.4	0.002436	9.4	TRUE	TRUE
1	STNVVIRAC	120	128	9	HLA-A*02:01	120	9.5	STNVVIRAC	STNVVIRAC	0.002417	9.5	0.002417	9.5	TRUE	TRUE
1	GFHTINHTF	68	76	9	HLA-A*02:01	68	9.5	GFHTINHTF	GFHTINHTF	0.002394	9.5	0.002394	9.5	TRUE	TRUE
1	FCTQLNRAL	741	749	9	HLA-A*02:01	741	9.5	FCTQLNRAL	FCTQLNRAL	0.002387	9.5	0.002387	9.5	TRUE	TRUE
1	PLQPELDSF	1122	1130	9	HLA-A*02:01	1122	9.6	PLQPELDSF	PLQPELDSF	0.00237	9.6	0.00237	9.6	TRUE	TRUE
1	EYISDAFSL	162	170	9	HLA-A*02:01	162	9.6	EYISDAFSL	EYISDAFSL	0.002337	9.6	0.002337	9.6	TRUE	TRUE
1	WLGFIAGLI	1199	1207	9	HLA-A*02:01	1199	9.6	WLGFIAGLI	WLGFIAGLI	0.002337	9.6	0.002337	9.6	TRUE	TRUE
1	FIEDLLFNK	799	807	9	HLA-A*02:01	799	9.6	FIEDLLFNK	FIEDLLFNK	0.002335	9.6	0.002335	9.6	TRUE	TRUE
1	VVRDLPSGF	205	213	9	HLA-A*02:01	205	9.6	VVRDLPSGF	VVRDLPSGF	0.002331	9.6	0.002331	9.6	TRUE	TRUE
1	GCLIGAEHV	634	642	9	HLA-A*02:01	634	9.7	GCLIGAEHV	GCLIGAEHV	0.002308	9.7	0.002308	9.7	TRUE	TRUE
1	GGFNFSQIL	780	788	9	HLA-A*02:01	780	9.7	GGFNFSQIL	GGFNFSQIL	0.002304	9.7	0.002304	9.7	TRUE	TRUE
1	CVNFNFNGL	524	532	9	HLA-A*02:01	524	9.7	CVNFNFNGL	CVNFNFNGL	0.002278	9.7	0.002278	9.7	TRUE	TRUE
1	SQNPLAELK	279	287	9	HLA-A*02:01	279	9.8	SQNPLAELK	SQNPLAELK	0.002234	9.8	0.002234	9.8	TRUE	TRUE
1	QAAPHGVVF	1036	1044	9	HLA-A*02:01	1036	9.9	QAAPHGVVF	QAAPHGVVF	0.002215	9.9	0.002215	9.9	TRUE	TRUE
1	FSQILPDPL	784	792	9	HLA-A*02:01	784	9.9	FSQILPDPL	FSQILPDPL	0.002166	9.9	0.002166	9.9	TRUE	TRUE
1	GVSVITPGT	580	588	9	HLA-A*02:01	580	10	GVSVITPGT	GVSVITPGT	0.002146	10	0.002146	10	TRUE	TRUE
1	RNFTTAPAI	1055	1063	9	HLA-A*02:01	1055	10	RNFTTAPAI	RNFTTAPAI	0.002128	10	0.002128	10	TRUE	TRUE
1	FCGKGYHLM	1024	1032	9	HLA-A*02:01	1024	11	FCGKGYHLM	FCGKGYHLM	0.002098	11	0.002098	11	TRUE	TRUE
1	TVCGPKLST	509	517	9	HLA-A*02:01	509	11	TVCGPKLST	TVCGPKLST	0.002098	11	0.002098	11	TRUE	TRUE
1	NGLTVLPPL	838	846	9	HLA-A*02:01	838	11	NGLTVLPPL	NGLTVLPPL	0.002092	11	0.002092	11	TRUE	TRUE
1	AVLYQDVNC	595	603	9	HLA-A*02:01	595	11	AVLYQDVNC	AVLYQDVNC	0.00204	11	0.00204	11	TRUE	TRUE
1	VYYPDEIFR	40	48	9	HLA-A*02:01	40	11	VYYPDEIFR	VYYPDEIFR	0.002034	11	0.002034	11	TRUE	TRUE
1	LTQDLFLPF	54	62	9	HLA-A*02:01	54	11	LTQDLFLPF	LTQDLFLPF	0.001994	11	0.001994	11	TRUE	TRUE
1	AATKMSECV	1007	1015	9	HLA-A*02:01	1007	11	AATKMSECV	AATKMSECV	0.001991	11	0.001991	11	TRUE	TRUE
1	LYLTQDLFL	52	60	9	HLA-A*02:01	52	11	LYLTQDLFL	LYLTQDLFL	0.00199	11	0.00199	11	TRUE	TRUE
1	LTVLPPLLT	840	848	9	HLA-A*02:01	840	11	LTVLPPLLT	LTVLPPLLT	0.001982	11	0.001982	11	TRUE	TRUE
1	TAFSPAQDI	236	244	9	HLA-A*02:01	236	11	TAFSPAQDI	TAFSPAQDI	0.001958	11	0.001958	11	TRUE	TRUE
1	PMGTQTHTM	143	151	9	HLA-A*02:01	143	11	PMGTQTHTM	PMGTQTHTM	0.00195	11	0.00195	11	TRUE	TRUE
1	EVMPVSMAK	707	715	9	HLA-A*02:01	707	11	EVMPVSMAK	EVMPVSMAK	0.001945	11	0.001945	11	TRUE	TRUE
1	ASSEVAVLY	590	598	9	HLA-A*02:01	590	11	ASSEVAVLY	ASSEVAVLY	0.001944	11	0.001944	11	TRUE	TRUE
1	IAYSNNTIA	687	695	9	HLA-A*02:01	687	11	IAYSNNTIA	IAYSNNTIA	0.001941	11	0.001941	11	TRUE	TRUE
1	ILTAFSPAQ	234	242	9	HLA-A*02:01	234	11	ILTAFSPAQ	ILTAFSPAQ	0.001919	11	0.001919	11	TRUE	TRUE
1	KCYGVSATK	365	373	9	HLA-A*02:01	365	11	KCYGVSATK	KCYGVSATK	0.001877	11	0.001877	11	TRUE	TRUE
1	FKLPLGINI	220	228	9	HLA-A*02:01	220	11	FKLPLGINI	FKLPLGINI	0.001874	11	0.001874	11	TRUE	TRUE
1	AIHADQLTP	609	617	9	HLA-A*02:01	609	11	AIHADQLTP	AIHADQLTP	0.001865	11	0.001865	11	TRUE	TRUE
1	TTEVMPVSM	705	713	9	HLA-A*02:01	705	11	TTEVMPVSM	TTEVMPVSM	0.001846	11	0.001846	11	TRUE	TRUE
1	GQTGVIADY	400	408	9	HLA-A*02:01	400	11	GQTGVIADY	GQTGVIADY	0.001775	11	0.001775	11	TRUE	TRUE
1	NQKQIANQF	901	909	9	HLA-A*02:01	901	11	NQKQIANQF	NQKQIANQF	0.00177	11	0.00177	11	TRUE	TRUE
1	GYLKPTTFM	255	263	9	HLA-A*02:01	255	11	GYLKPTTFM	GYLKPTTFM	0.001762	11	0.001762	11	TRUE	TRUE
1	NCTDVSTAI	602	610	9	HLA-A*02:01	602	11	NCTDVSTAI	NCTDVSTAI	0.00174	11	0.00174	11	TRUE	TRUE
1	FSPDGKPCT	460	468	9	HLA-A*02:01	460	11	FSPDGKPCT	FSPDGKPCT	0.001718	11	0.001718	11	TRUE	TRUE
1	ELDKYFKNH	1133	1141	9	HLA-A*02:01	1133	12	ELDKYFKNH	ELDKYFKNH	0.001692	12	0.001692	12	TRUE	TRUE
1	HTFGNPVIP	74	82	9	HLA-A*02:01	74	12	HTFGNPVIP	HTFGNPVIP	0.001667	12	0.001667	12	TRUE	TRUE
1	IYQTSNFRV	299	307	9	HLA-A*02:01	299	12	IYQTSNFRV	IYQTSNFRV	0.001663	12	0.001663	12	TRUE	TRUE
1	FLYVYKGYQ	193	201	9	HLA-A*02:01	193	12	FLYVYKGYQ	FLYVYKGYQ	0.001625	12	0.001625	12	TRUE	TRUE
1	VQAPNYTQH	25	33	9	HLA-A*02:01	25	12	VQAPNYTQH	VQAPNYTQH	0.001613	12	0.001613	12	TRUE	TRUE
1	LAATKMSEC	1006	1014	9	HLA-A*02:01	1006	12	LAATKMSEC	LAATKMSEC	0.001593	12	0.001593	12	TRUE	TRUE
1	SQILPDPLK	785	793	9	HLA-A*02:01	785	12	SQILPDPLK	SQILPDPLK	0.001587	12	0.001587	12	TRUE	TRUE
1	AGWTFGAGA	866	874	9	HLA-A*02:01	866	12	AGWTFGAGA	AGWTFGAGA	0.001584	12	0.001584	12	TRUE	TRUE
1	QKFNGLTVL	835	843	9	HLA-A*02:01	835	12	QKFNGLTVL	QKFNGLTVL	0.001578	12	0.001578	12	TRUE	TRUE
1	SGFNTLKPI	211	219	9	HLA-A*02:01	211	12	SGFNTLKPI	SGFNTLKPI	0.001543	12	0.001543	12	TRUE	TRUE
1	YVPSQERNF	1049	1057	9	HLA-A*02:01	1049	12	YVPSQERNF	YVPSQERNF	0.001543	12	0.001543	12	TRUE	TRUE
1	RSFIEDLLF	797	805	9	HLA-A*02:01	797	12	RSFIEDLLF	RSFIEDLLF	0.001536	12	0.001536	12	TRUE	TRUE
1	GTSWFITQR	1081	1089	9	HLA-A*02:01	1081	12	GTSWFITQR	GTSWFITQR	0.001523	12	0.001523	12	TRUE	TRUE
1	GVVFLHVTY	1041	1049	9	HLA-A*02:01	1041	12	GVVFLHVTY	GVVFLHVTY	0.001519	12	0.001519	12	TRUE	TRUE
1	MLKYDENGT	263	271	9	HLA-A*02:01	263	12	MLKYDENGT	MLKYDENGT	0.001516	12	0.001516	12	TRUE	TRUE
1	SLLRSTSQK	664	672	9	HLA-A*02:01	664	12	SLLRSTSQK	SLLRSTSQK	0.001492	12	0.001492	12	TRUE	TRUE
1	TVLPPLLTD	841	849	9	HLA-A*02:01	841	12	TVLPPLLTD	TVLPPLLTD	0.001486	12	0.001486	12	TRUE	TRUE
1	FSLDVSEKS	168	176	9	HLA-A*02:01	168	12	FSLDVSEKS	FSLDVSEKS	0.001461	12	0.001461	12	TRUE	TRUE
1	YLRHGKLRP	442	450	9	HLA-A*02:01	442	12	YLRHGKLRP	YLRHGKLRP	0.001458	12	0.001458	12	TRUE	TRUE
1	ALQIPFAMQ	875	883	9	HLA-A*02:01	875	12	ALQIPFAMQ	ALQIPFAMQ	0.001447	12	0.001447	12	TRUE	TRUE
1	CVADYSVLY	348	356	9	HLA-A*02:01	348	12	CVADYSVLY	CVADYSVLY	0.001439	12	0.001439	12	TRUE	TRUE
1	FITQRNFFS	1085	1093	9	HLA-A*02:01	1085	12	FITQRNFFS	FITQRNFFS	0.001435	12	0.001435	12	TRUE	TRUE
1	SVDCNMYIC	717	725	9	HLA-A*02:01	717	12	SVDCNMYIC	SVDCNMYIC	0.001427	12	0.001427	12	TRUE	TRUE
1	KAISQIQES	911	919	9	HLA-A*02:01	911	12	KAISQIQES	KAISQIQES	0.001421	12	0.001421	12	TRUE	TRUE
1	GYQPYRVVV	490	498	9	HLA-A*02:01	490	12	GYQPYRVVV	GYQPYRVVV	0.001419	12	0.001419	12	TRUE	TRUE
1	LIRAAEIRA	994	1002	9	HLA-A*02:01	994	13	LIRAAEIRA	LIRAAEIRA	0.001374	13	0.001374	13	TRUE	TRUE
1	TNASSEVAV	588	596	9	HLA-A*02:01	588	13	TNASSEVAV	TNASSEVAV	0.001366	13	0.001366	13	TRUE	TRUE
1	SVYAWERKK	336	344	9	HLA-A*02:01	336	13	SVYAWERKK	SVYAWERKK	0.001355	13	0.001355	13	TRUE	TRUE
1	TNFRAILTA	229	237	9	HLA-A*02:01	229	13	TNFRAILTA	TNFRAILTA	0.001349	13	0.001349	13	TRUE	TRUE
1	FMLKYDENG	262	270	9	HLA-A*02:01	262	13	FMLKYDENG	FMLKYDENG	0.001333	13	0.001333	13	TRUE	TRUE
1	IANQFNKAI	905	913	9	HLA-A*02:01	905	13	IANQFNKAI	IANQFNKAI	0.001331	13	0.001331	13	TRUE	TRUE
1	STECANLLL	728	736	9	HLA-A*02:01	728	13	STECANLLL	STECANLLL	0.001299	13	0.001299	13	TRUE	TRUE
1	CPFGEVFNA	323	331	9	HLA-A*02:01	323	13	CPFGEVFNA	CPFGEVFNA	0.001277	13	0.001277	13	TRUE	TRUE
1	GIYFAATEK	86	94	9	HLA-A*02:01	86	13	GIYFAATEK	GIYFAATEK	0.001273	13	0.001273	13	TRUE	TRUE
1	DSEPVLKGV	1242	1250	9	HLA-A*02:01	1242	13	DSEPVLKGV	DSEPVLKGV	0.001266	13	0.001266	13	TRUE	TRUE
1	IPTNFSISI	696	704	9	HLA-A*02:01	696	13	IPTNFSISI	IPTNFSISI	0.001261	13	0.001261	13	TRUE	TRUE
1	EVAVLYQDV	593	601	9	HLA-A*02:01	593	13	EVAVLYQDV	EVAVLYQDV	0.001226	13	0.001226	13	TRUE	TRUE
1	TTSTALGKL	922	930	9	HLA-A*02:01	922	13	TTSTALGKL	TTSTALGKL	0.001208	13	0.001208	13	TRUE	TRUE
1	HTVSLLRST	661	669	9	HLA-A*02:01	661	13	HTVSLLRST	HTVSLLRST	0.001199	13	0.001199	13	TRUE	TRUE
1	ITTEVMPVS	704	712	9	HLA-A*02:01	704	13	ITTEVMPVS	ITTEVMPVS	0.001188	13	0.001188	13	TRUE	TRUE
1	GTATAGWTF	862	870	9	HLA-A*02:01	862	13	GTATAGWTF	GTATAGWTF	0.001178	13	0.001178	13	TRUE	TRUE
1	YHLMSFPQA	1029	1037	9	HLA-A*02:01	1029	13	YHLMSFPQA	YHLMSFPQA	0.00117	13	0.00117	13	TRUE	TRUE
1	FNFNGLTGT	527	535	9	HLA-A*02:01	527	14	FNFNGLTGT	FNFNGLTGT	0.001131	14	0.001131	14	TRUE	TRUE
1	RQIAPGQTG	395	403	9	HLA-A*02:01	395	14	RQIAPGQTG	RQIAPGQTG	0.001126	14	0.001126	14	TRUE	TRUE
1	YECDIPIGA	646	654	9	HLA-A*02:01	646	14	YECDIPIGA	YECDIPIGA	0.001124	14	0.001124	14	TRUE	TRUE
1	VTYVPSQER	1047	1055	9	HLA-A*02:01	1047	14	VTYVPSQER	VTYVPSQER	0.001101	14	0.001101	14	TRUE	TRUE
1	FGNPVIPFK	76	84	9	HLA-A*02:01	76	14	FGNPVIPFK	FGNPVIPFK	0.00108	14	0.00108	14	TRUE	TRUE
1	FPREGVFVF	1071	1079	9	HLA-A*02:01	1071	14	FPREGVFVF	FPREGVFVF	0.001063	14	0.001063	14	TRUE	TRUE
1	RGWVFGSTM	99	107	9	HLA-A*02:01	99	14	RGWVFGSTM	RGWVFGSTM	0.001059	14	0.001059	14	TRUE	TRUE
1	LTLTSGSDL	8	16	9	HLA-A*02:01	8	14	LTLTSGSDL	LTLTSGSDL	0.001036	14	0.001036	14	TRUE	TRUE
1	ISSVLNDIL	955	963	9	HLA-A*02:01	955	14	ISSVLNDIL	ISSVLNDIL	0.001032	14	0.001032	14	TRUE	TRUE
1	ISPCSFGGV	573	581	9	HLA-A*02:01	573	14	ISPCSFGGV	ISPCSFGGV	0.00102	14	0.00102	14	TRUE	TRUE
1	GRLQSLQTY	981	989	9	HLA-A*02:01	981	14	GRLQSLQTY	GRLQSLQTY	0.001018	14	0.001018	14	TRUE	TRUE
1	AQALNTLVK	938	946	9	HLA-A*02:01	938	14	AQALNTLVK	AQALNTLVK	0.00101	14	0.00101	14	TRUE	TRUE
1	GVLTPSSKR	536	544	9	HLA-A*02:01	536	14	GVLTPSSKR	GVLTPSSKR	0.00099	14	0.00099	14	TRUE	TRUE
1	KRSFIEDLL	796	804	9	HLA-A*02:01	796	15	KRSFIEDLL	KRSFIEDLL	0.000965	15	0.000965	15	TRUE	TRUE
1	IPFKDGIYF	81	89	9	HLA-A*02:01	81	15	IPFKDGIYF	IPFKDGIYF	0.00096	15	0.00096	15	TRUE	TRUE
1	LLRSTSQKS	665	673	9	HLA-A*02:01	665	15	LLRSTSQKS	LLRSTSQKS	0.000959	15	0.000959	15	TRUE	TRUE
1	NFFSPQIIT	1090	1098	9	HLA-A*02:01	1090	15	NFFSPQIIT	NFFSPQIIT	0.000952	15	0.000952	15	TRUE	TRUE
1	VSATKLNDL	369	377	9	HLA-A*02:01	369	15	VSATKLNDL	VSATKLNDL	0.000951	15	0.000951	15	TRUE	TRUE
1	SSMRGVYYP	35	43	9	HLA-A*02:01	35	15	SSMRGVYYP	SSMRGVYYP	0.000947	15	0.000947	15	TRUE	TRUE
1	LPDDFMGCV	412	420	9	HLA-A*02:01	412	15	LPDDFMGCV	LPDDFMGCV	0.000928	15	0.000928	15	TRUE	TRUE

seq #	peptide	start	end	peptide length	allele	peptide index	median binding	netmhcpan_el	netmhcpan_el	netmhcpan_el	netmhcpan_el	legacy score	legacy rank	scores equal	percentiles equal	
1	SANLAATKM	1003	1011	9	HLA-A*02:01	1003	15	SANLAATKM	SANLAATKM	0.000916	15	0.000916	15	TRUE	TRUE	
1	RFPNITNLC	315	323	9	HLA-A*02:01	315	15	RFPNITNLC	RFPNITNLC	0.000914	15	0.000914	15	TRUE	TRUE	
1	AEVQIDRLI	971	979	9	HLA-A*02:01	971	15	AEVQIDRLI	AEVQIDRLI	0.000912	15	0.000912	15	TRUE	TRUE	
1	NMYICGDST	721	729	9	HLA-A*02:01	721	15	NMYICGDST	NMYICGDST	0.000902	15	0.000902	15	TRUE	TRUE	
1	VFAQVKQMY	763	771	9	HLA-A*02:01	763	15	VFAQVKQMY	VFAQVKQMY	0.000879	15	0.000879	15	TRUE	TRUE	
1	QNAQALNTL	936	944	9	HLA-A*02:01	936	15	QNAQALNTL	QNAQALNTL	0.000863	15	0.000863	15	TRUE	TRUE	
1	GIYQTSNFR	298	306	9	HLA-A*02:01	298	15	GIYQTSNFR	GIYQTSNFR	0.000859	15	0.000859	15	TRUE	TRUE	
1	PLLTDDMIA	845	853	9	HLA-A*02:01	845	15	PLLTDDMIA	PLLTDDMIA	0.000857	15	0.000857	15	TRUE	TRUE	
1	YVWLGFIAG	1197	1205	9	HLA-A*02:01	1197	15	YVWLGFIAG	YVWLGFIAG	0.000855	15	0.000855	15	TRUE	TRUE	
1	LVKQLSSNF	944	952	9	HLA-A*02:01	944	15	LVKQLSSNF	LVKQLSSNF	0.000851	15	0.000851	15	TRUE	TRUE	
1	IAGLIAIVM	1203	1211	9	HLA-A*02:01	1203	15	IAGLIAIVM	IAGLIAIVM	0.000847	15	0.000847	15	TRUE	TRUE	
1	NAFNCTFEY	155	163	9	HLA-A*02:01	155	15	NAFNCTFEY	NAFNCTFEY	0.000845	15	0.000845	15	TRUE	TRUE	
1	NLLLQYGSF	733	741	9	HLA-A*02:01	733	15	NLLLQYGSF	NLLLQYGSF	0.00084	15	0.00084	15	TRUE	TRUE	
1	VDLGDISGI	1146	1154	9	HLA-A*02:01	1146	15	VDLGDISGI	VDLGDISGI	0.000837	15	0.000837	15	TRUE	TRUE	
1	NLAATKMSE	1005	1013	9	HLA-A*02:01	1005	15	NLAATKMSE	NLAATKMSE	0.000835	15	0.000835	15	TRUE	TRUE	
1	ESLTTTSTA	918	926	9	HLA-A*02:01	918	15	ESLTTTSTA	ESLTTTSTA	0.000829	15	0.000829	15	TRUE	TRUE	
1	NQFNKAISQ	907	915	9	HLA-A*02:01	907	15	NQFNKAISQ	NQFNKAISQ	0.000825	15	0.000825	15	TRUE	TRUE	
1	FNGLTGTGV	529	537	9	HLA-A*02:01	529	16	FNGLTGTGV	FNGLTGTGV	0.000817	16	0.000817	16	TRUE	TRUE	
1	KGDDVRQIA	390	398	9	HLA-A*02:01	390	16	KGDDVRQIA	KGDDVRQIA	0.000809	16	0.000809	16	TRUE	TRUE	
1	FGGFNFSQI	779	787	9	HLA-A*02:01	779	16	FGGFNFSQI	FGGFNFSQI	0.000788	16	0.000788	16	TRUE	TRUE	
1	KYDENGTIT	265	273	9	HLA-A*02:01	265	16	KYDENGTIT	KYDENGTIT	0.000784	16	0.000784	16	TRUE	TRUE	
1	PVIPFKDGI	79	87	9	HLA-A*02:01	79	16	PVIPFKDGI	PVIPFKDGI	0.000783	16	0.000783	16	TRUE	TRUE	
1	LSFELLNAP	499	507	9	HLA-A*02:01	499	16	LSFELLNAP	LSFELLNAP	0.000781	16	0.000781	16	TRUE	TRUE	
1	GDVVRFPNI	311	319	9	HLA-A*02:01	311	16	GDVVRFPNI	GDVVRFPNI	0.000762	16	0.000762	16	TRUE	TRUE	
1	VVRGWVFGS	97	105	9	HLA-A*02:01	97	16	VVRGWVFGS	VVRGWVFGS	0.000754	16	0.000754	16	TRUE	TRUE	
1	VTLADAGFM	808	816	9	HLA-A*02:01	808	16	VTLADAGFM	VTLADAGFM	0.000751	16	0.000751	16	TRUE	TRUE	
1	YYPDEIFRS	41	49	9	HLA-A*02:01	41	16	YYPDEIFRS	YYPDEIFRS	0.000751	16	0.000751	16	TRUE	TRUE	
1	QLTPAWRIY	614	622	9	HLA-A*02:01	614	16	QLTPAWRIY	QLTPAWRIY	0.00075	16	0.00075	16	TRUE	TRUE	
1	QRNFFSPQI	1088	1096	9	HLA-A*02:01	1088	16	QRNFFSPQI	QRNFFSPQI	0.00075	16	0.00075	16	TRUE	TRUE	
1	KVTLADAGF	807	815	9	HLA-A*02:01	807	16	KVTLADAGF	KVTLADAGF	0.000747	16	0.000747	16	TRUE	TRUE	
1	GTQTHTMIF	145	153	9	HLA-A*02:01	145	16	GTQTHTMIF	GTQTHTMIF	0.000744	16	0.000744	16	TRUE	TRUE	
1	AYSNNTIAI	688	696	9	HLA-A*02:01	688	16	AYSNNTIAI	AYSNNTIAI	0.000727	16	0.000727	16	TRUE	TRUE	
1	MGTQTHTMI	144	152	9	HLA-A*02:01	144	16	MGTQTHTMI	MGTQTHTMI	0.000718	16	0.000718	16	TRUE	TRUE	
1	NFGAISSVL	951	959	9	HLA-A*02:01	951	17	NFGAISSVL	NFGAISSVL	0.000696	17	0.000696	17	TRUE	TRUE	
1	LQSLQTYVT	983	991	9	HLA-A*02:01	983	17	LQSLQTYVT	LQSLQTYVT	0.000694	17	0.000694	17	TRUE	TRUE	
1	IAPGQTGVI	397	405	9	HLA-A*02:01	397	17	IAPGQTGVI	IAPGQTGVI	0.000692	17	0.000692	17	TRUE	TRUE	
1	KFNGLTVLP	836	844	9	HLA-A*02:01	836	17	KFNGLTVLP	KFNGLTVLP	0.000692	17	0.000692	17	TRUE	TRUE	
1	VYADSFVVK	382	390	9	HLA-A*02:01	382	17	VYADSFVVK	VYADSFVVK	0.00069	17	0.00069	17	TRUE	TRUE	
1	YADSFVVKG	383	391	9	HLA-A*02:01	383	17	YADSFVVKG	YADSFVVKG	0.000685	17	0.000685	17	TRUE	TRUE	
1	ICHEGKAYF	1063	1071	9	HLA-A*02:01	1063	17	ICHEGKAYF	ICHEGKAYF	0.000684	17	0.000684	17	TRUE	TRUE	
1	ATKMSECVL	1008	1016	9	HLA-A*02:01	1008	17	ATKMSECVL	ATKMSECVL	0.000682	17	0.000682	17	TRUE	TRUE	
1	EILDISPCS	569	577	9	HLA-A*02:01	569	17	EILDISPCS	EILDISPCS	0.000673	17	0.000673	17	TRUE	TRUE	
1	SAAAYFVGY	248	256	9	HLA-A*02:01	248	17	SAAAYFVGY	SAAAYFVGY	0.000671	17	0.000671	17	TRUE	TRUE	
1	EIDRLNEVA	1164	1172	9	HLA-A*02:01	1164	17	EIDRLNEVA	EIDRLNEVA	0.000668	17	0.000668	17	TRUE	TRUE	
1	FDEDDSEPV	1238	1246	9	HLA-A*02:01	1238	17	FDEDDSEPV	FDEDDSEPV	0.000666	17	0.000666	17	TRUE	TRUE	
1	HLREFVFKN	181	189	9	HLA-A*02:01	181	17	HLREFVFKN	HLREFVFKN	0.000656	17	0.000656	17	TRUE	TRUE	
1	SDLDRCTTF	14	22	9	HLA-A*02:01	14	17	SDLDRCTTF	SDLDRCTTF	0.000653	17	0.000653	17	TRUE	TRUE	
1	GTNASSEVA	587	595	9	HLA-A*02:01	587	17	GTNASSEVA	GTNASSEVA	0.000651	17	0.000651	17	TRUE	TRUE	
1	CMTSCCSCL	1218	1226	9	HLA-A*02:01	1218	17	CMTSCCSCL	CMTSCCSCL	0.000649	17	0.000649	17	TRUE	TRUE	
1	YTQHTSSMR	30	38	9	HLA-A*02:01	30	17	YTQHTSSMR	YTQHTSSMR	0.000646	17	0.000646	17	TRUE	TRUE	
1	PVSMAKTSV	710	718	9	HLA-A*02:01	710	17	PVSMAKTSV	PVSMAKTSV	0.000636	17	0.000636	17	TRUE	TRUE	
1	YRYLRHGKL	440	448	9	HLA-A*02:01	440	17	YRYLRHGKL	YRYLRHGKL	0.00063	17	0.00063	17	TRUE	TRUE	
1	RAILTAFSP	232	240	9	HLA-A*02:01	232	17	RAILTAFSP	RAILTAFSP	0.000629	17	0.000629	17	TRUE	TRUE	
1	TIAIPTNFS	693	701	9	HLA-A*02:01	693	17	TIAIPTNFS	TIAIPTNFS	0.000624	17	0.000624	17	TRUE	TRUE	
1	GKPCTPPAL	464	472	9	HLA-A*02:01	464	17	GKPCTPPAL	GKPCTPPAL	0.000619	17	0.000619	17	TRUE	TRUE	
1	DISNVPFSP	454	462	9	HLA-A*02:01	454	17	DISNVPFSP	DISNVPFSP	0.000618	17	0.000618	17	TRUE	TRUE	
1	KLNDLCFSN	373	381	9	HLA-A*02:01	373	17	KLNDLCFSN	KLNDLCFSN	0.000609	17	0.000609	17	TRUE	TRUE	
1	KLSTDLIKN	514	522	9	HLA-A*02:01	514	17	KLSTDLIKN	KLSTDLIKN	0.000609	17	0.000609	17	TRUE	TRUE	
1	FQPFQQFGR	545	553	9	HLA-A*02:01	545	18	FQPFQQFGR	FQPFQQFGR	0.000601	18	0.000601	18	TRUE	TRUE	
1	VPSGDVVRF	308	316	9	HLA-A*02:01	308	18	VPSGDVVRF	VPSGDVVRF	0.000598	18	0.000598	18	TRUE	TRUE	
1	YSVLYNSTF	352	360	9	HLA-A*02:01	352	18	YSVLYNSTF	YSVLYNSTF	0.000595	18	0.000595	18	TRUE	TRUE	
1	FRSDTLYLT	47	55	9	HLA-A*02:01	47	18	FRSDTLYLT	FRSDTLYLT	0.000594	18	0.000594	18	TRUE	TRUE	
1	TAALVSGTA	856	864	9	HLA-A*02:01	856	18	TAALVSGTA	TAALVSGTA	0.000587	18	0.000587	18	TRUE	TRUE	
1	VVPSGDVVR	307	315	9	HLA-A*02:01	307	18	VVPSGDVVR	VVPSGDVVR	0.000587	18	0.000587	18	TRUE	TRUE	
1	RASANLAAT	1001	1009	9	HLA-A*02:01	1001	18	RASANLAAT	RASANLAAT	0.000575	18	0.000575	18	TRUE	TRUE	
1	IHADQLTPA	610	618	9	HLA-A*02:01	610	18	IHADQLTPA	IHADQLTPA	0.000573	18	0.000573	18	TRUE	TRUE	
1	YAWERKKIS	338	346	9	HLA-A*02:01	338	18	YAWERKKIS	YAWERKKIS	0.000571	18	0.000571	18	TRUE	TRUE	
1	TTTGIGYQP	485	493	9	HLA-A*02:01	485	18	TTTGIGYQP	TTTGIGYQP	0.000568	18	0.000568	18	TRUE	TRUE	
1	ATSTGNYNY	430	438	9	HLA-A*02:01	430	18	ATSTGNYNY	ATSTGNYNY	0.000567	18	0.000567	18	TRUE	TRUE	
1	SFKEELDKY	1129	1137	9	HLA-A*02:01	1129	18	SFKEELDKY	SFKEELDKY	0.000565	18	0.000565	18	TRUE	TRUE	
1	VITPGTNAS	583	591	9	HLA-A*02:01	583	18	VITPGTNAS	VITPGTNAS	0.000557	18	0.000557	18	TRUE	TRUE	
1	YKTPTLKYF	771	779	9	HLA-A*02:01	771	18	YKTPTLKYF	YKTPTLKYF	0.000557	18	0.000557	18	TRUE	TRUE	
1	FKNHTSPDV	1138	1146	9	HLA-A*02:01	1138	18	FKNHTSPDV	FKNHTSPDV	0.000547	18	0.000547	18	TRUE	TRUE	
1	RVDFCGKGY	1021	1029	9	HLA-A*02:01	1021	18	RVDFCGKGY	RVDFCGKGY	0.000545	18	0.000545	18	TRUE	TRUE	
1	YFVGYLKPT	252	260	9	HLA-A*02:01	252	18	YFVGYLKPT	YFVGYLKPT	0.000542	18	0.000542	18	TRUE	TRUE	
1	EPVLKGVKL	1244	1252	9	HLA-A*02:01	1244	18	EPVLKGVKL	EPVLKGVKL	0.000539	18	0.000539	18	TRUE	TRUE	
1	LPDPLKPTK	788	796	9	HLA-A*02:01	788	18	LPDPLKPTK	LPDPLKPTK	0.000537	18	0.000537	18	TRUE	TRUE	
1	RNFFSPQII	1089	1097	9	HLA-A*02:01	1089	18	RNFFSPQII	RNFFSPQII	0.000534	18	0.000534	18	TRUE	TRUE	
1	ISGINASVV	1151	1159	9	HLA-A*02:01	1151	18	ISGINASVV	ISGINASVV	0.000529	18	0.000529	18	TRUE	TRUE	
1	QIDRLITGR	974	982	9	HLA-A*02:01	974	18	QIDRLITGR	QIDRLITGR	0.000529	18	0.000529	18	TRUE	TRUE	
1	QLIRAAEIR	993	1001	9	HLA-A*02:01	993	18	QLIRAAEIR	QLIRAAEIR	0.000529	18	0.000529	18	TRUE	TRUE	
1	LIKNQCVNF	519	527	9	HLA-A*02:01	519	18	LIKNQCVNF	LIKNQCVNF	0.000528	18	0.000528	18	TRUE	TRUE	
1	INNSTNVVI	117	125	9	HLA-A*02:01	117	19	INNSTNVVI	INNSTNVVI	0.000521	19	0.000521	19	TRUE	TRUE	
1	TDLIKNQCV	517	525	9	HLA-A*02:01	517	19	TDLIKNQCV	TDLIKNQCV	0.000521	19	0.000521	19	TRUE	TRUE	
1	TFGNPVIPF	75	83	9	HLA-A*02:01	75	19	TFGNPVIPF	TFGNPVIPF	0.000519	19	0.000519	19	TRUE	TRUE	
1	KISNCVADY	344	352	9	HLA-A*02:01	344	19	KISNCVADY	KISNCVADY	0.000496	19	0.000496	19	TRUE	TRUE	
1	TKFPSVYAW	332	340	9	HLA-A*02:01	332	19	TKFPSVYAW	TKFPSVYAW	0.000496	19	0.000496	19	TRUE	TRUE	
1	EIFRSDTLY	45	53	9	HLA-A*02:01	45	19	EIFRSDTLY	EIFRSDTLY	0.000493	19	0.000493	19	TRUE	TRUE	
1	CDIPIGAGI	648	656	9	HLA-A*02:01	648	19	CDIPIGAGI	CDIPIGAGI	0.00049	19	0.00049	19	TRUE	TRUE	
1	LTGTGVLTP	532	540	9	HLA-A*02:01	532	19	LTGTGVLTP	LTGTGVLTP	0.000476	19	0.000476	19	TRUE	TRUE	
1	MYKTPTLKY	770	778	9	HLA-A*02:01	770	19	MYKTPTLKY	MYKTPTLKY	0.000463	19	0.000463	19	TRUE	TRUE	
1	FKNKDGFLY	187	195	9	HLA-A*02:01	187	20	FKNKDGFLY	FKNKDGFLY	0.000457	20	0.000457	20	TRUE	TRUE	
1	STGNYNYKY	432	440	9	HLA-A*02:01	432	20	STGNYNYKY	STGNYNYKY	0.000457	20	0.000457	20	TRUE	TRUE	
1	SYHTVSLLR	659	667	9	HLA-A*02:01	659	20	SYHTVSLLR	SYHTVSLLR	0.000457	20	0.000457	20	TRUE	TRUE	
1	FVVKGDDVR	387	395	9	HLA-A*02:01	387	20	FVVKGDDVR	FVVKGDDVR	0.000453	20	0.000453	20	TRUE	TRUE	
1	TGFHTINHT	67	75	9	HLA-A*02:01	67	20	TGFHTINHT	TGFHTINHT	0.00045	20	0.00045	20	TRUE	TRUE	
1	AFNCTFEYI	156	164	9	HLA-A*02:01	156	20	AFNCTFEYI	AFNCTFEYI	0.000449	20	0.000449	20	TRUE	TRUE	
1	HTMIFDNAF	149	157	9	HLA-A*02:01	149	20	HTMIFDNAF	HTMIFDNAF	0.000446	20	0.000446	20	TRUE	TRUE	
1	INITNFRAI	226	234	9	HLA-A*02:01	226	20	INITNFRAI	INITNFRAI	0.000446	20	0.000446	20	TRUE	TRUE	
1	TSQKSIVAY	669	677	9	HLA-A*02:01	669	20	TSQKSIVAY	TSQKSIVAY	0.000445	20	0.000445	20	TRUE	TRUE	
1	SPAQDIWGT	239	247	9	HLA-A*02:01	239	20	SPAQDIWGT	SPAQDIWGT	0.000441	20	0.000441	20	TRUE	TRUE	
1	DTLYLTQDL	50	58	9	HLA-A*02:01	50	20	DTLYLTQDL	DTLYLTQDL	0.00044	20	0.00044	20	TRUE	TRUE	
1	AIPTNFSIS	695	703	9	HLA-A*02:01	695	20	AIPTNFSIS	AIPTNFSIS	0.000439	20	0.000439	20	TRUE	TRUE	
1	NQNAQALNT	935	943	9	HLA-A*02:01	935	20	NQNAQALNT	NQNAQALNT	0.000437	20	0.000437	20	TRUE	TRUE	
1	EAEVQIDRL	970	978	9	HLA-A*02:01	970	20	EAEVQIDRL	EAEVQIDRL	0.000436	20	0.000436	20	TRUE	TRUE	
1	PLGINITNF	223	231	9	HLA-A*02:01	223	20	PLGINITNF	PLGINITNF	0.000435	20	0.000435	20	TRUE	TRUE	
1	ITQRNFFSP	1086	1094	9	HLA-A*02:01	1086	20	ITQRNFFSP	ITQRNFFSP	0.000431	20	0.000431	20	TRUE	TRUE	
1	LQYGSFCTQ	736	744	9	HLA-A*02:01	736	20	LQYGSFCTQ	LQYGSFCTQ	0.00043	20	0.00043	20	TRUE	TRUE	
1	TNFSISITT	698	706	9	HLA-A*02:01	698	20	TNFSISITT	TNFSISITT	0.00043	20	0.00043	20	TRUE	TRUE	
1	LQPELDSFK	1123	1131	9	HLA-A*02:01	1123	20	LQPELDSFK	LQPELDSFK	0.000425	20	0.000425	20	TRUE	TRUE	
1	DFCGKGYHL	1023	1031	9	HLA-A*02:01	1023	20	DFCGKGYHL	DFCGKGYHL	0.000424	20	0.000424	20	TRUE	TRUE	
1	QAPNYTQHT	26	34	9	HLA-A*02:01	26	20	QAPNYTQHT	QAPNYTQHT	0.000424	20	0.000424	20	TRUE	TRUE	
1	DVVNQNAQA	932	940	9	HLA-A*02:01	932	20	DVVNQNAQA	DVVNQNAQA	0.000422	20	0.000422	20	TRUE	TRUE	
1	FAQVKQMYK	764	772	9	HLA-A*02:01	764	20	FAQVKQMYK	FAQVKQMYK	0.000416	20	0.000416	20	TRUE	TRUE	
1	FTTAPAICH	1057	1065	9	HLA-A*02:01	1057	20	FTTAPAICH	FTTAPAICH	0.000416	20	0.000416	20	TRUE	TRUE	

seq #	peptide	start	end	peptide length	allele	peptide index	median binding	netmhcpan_el	netmhcpan_el	netmhcpan_el	netmhcpan_el	legacy score	legacy rank	scores equal	percentiles equal
1	YWPLNDYGF	475	483	9	HLA-A*02:01	475	20	YWPLNDYGF	YWPLNDYGF	0.000416	20	0.000416	20	TRUE	TRUE
1	IINNTVYDP	1114	1122	9	HLA-A*02:01	1114	20	IINNTVYDP	IINNTVYDP	0.000415	20	0.000415	20	TRUE	TRUE
1	GYQPIDVVR	199	207	9	HLA-A*02:01	199	20	GYQPIDVVR	GYQPIDVVR	0.000407	20	0.000407	20	TRUE	TRUE
1	SVRDPKTSE	561	569	9	HLA-A*02:01	561	20	SVRDPKTSE	SVRDPKTSE	0.000406	20	0.000406	20	TRUE	TRUE
1	ELKCSVKSF	285	293	9	HLA-A*02:01	285	21	ELKCSVKSF	ELKCSVKSF	0.000403	21	0.000403	21	TRUE	TRUE
1	YNSTFFSTF	356	364	9	HLA-A*02:01	356	21	YNSTFFSTF	YNSTFFSTF	0.0004	21	0.0004	21	TRUE	TRUE
1	ITNFRAILT	228	236	9	HLA-A*02:01	228	21	ITNFRAILT	ITNFRAILT	0.000397	21	0.000397	21	TRUE	TRUE
1	SFELLNAPA	500	508	9	HLA-A*02:01	500	21	SFELLNAPA	SFELLNAPA	0.000393	21	0.000393	21	TRUE	TRUE
1	CTPPALNCY	467	475	9	HLA-A*02:01	467	21	CTPPALNCY	CTPPALNCY	0.000391	21	0.000391	21	TRUE	TRUE
1	VSDFTDSVR	555	563	9	HLA-A*02:01	555	21	VSDFTDSVR	VSDFTDSVR	0.00039	21	0.00039	21	TRUE	TRUE
1	LGDISGINA	1148	1156	9	HLA-A*02:01	1148	21	LGDISGINA	LGDISGINA	0.000386	21	0.000386	21	TRUE	TRUE
1	ITTDNTFVS	1097	1105	9	HLA-A*02:01	1097	21	ITTDNTFVS	ITTDNTFVS	0.000378	21	0.000378	21	TRUE	TRUE
1	GADSSIAYS	682	690	9	HLA-A*02:01	682	21	GADSSIAYS	GADSSIAYS	0.000375	21	0.000375	21	TRUE	TRUE
1	IPFAMQMAY	878	886	9	HLA-A*02:01	878	21	IPFAMQMAY	IPFAMQMAY	0.000375	21	0.000375	21	TRUE	TRUE
1	EVAKNLNES	1170	1178	9	HLA-A*02:01	1170	21	EVAKNLNES	EVAKNLNES	0.000372	21	0.000372	21	TRUE	TRUE
1	AAYTAALVS	853	861	9	HLA-A*02:01	853	21	AAYTAALVS	AAYTAALVS	0.000371	21	0.000371	21	TRUE	TRUE
1	NYTQHTSSM	29	37	9	HLA-A*02:01	29	21	NYTQHTSSM	NYTQHTSSM	0.000361	21	0.000361	21	TRUE	TRUE
1	LGDINARDL	823	831	9	HLA-A*02:01	823	21	LGDINARDL	LGDINARDL	0.000359	21	0.000359	21	TRUE	TRUE
1	TFVSGNCDV	1102	1110	9	HLA-A*02:01	1102	21	TFVSGNCDV	TFVSGNCDV	0.000357	21	0.000357	21	TRUE	TRUE
1	FKEELDKYF	1130	1138	9	HLA-A*02:01	1130	22	FKEELDKYF	FKEELDKYF	0.000355	22	0.000355	22	TRUE	TRUE
1	RDPKTSEIL	563	571	9	HLA-A*02:01	563	22	RDPKTSEIL	RDPKTSEIL	0.000354	22	0.000354	22	TRUE	TRUE
1	TLVKQLSSN	943	951	9	HLA-A*02:01	943	22	TLVKQLSSN	TLVKQLSSN	0.000354	22	0.000354	22	TRUE	TRUE
1	KTSEILDIS	566	574	9	HLA-A*02:01	566	22	KTSEILDIS	KTSEILDIS	0.000351	22	0.000351	22	TRUE	TRUE
1	LNDLCFSNV	374	382	9	HLA-A*02:01	374	22	LNDLCFSNV	LNDLCFSNV	0.000351	22	0.000351	22	TRUE	TRUE
1	YSNVTGFHT	63	71	9	HLA-A*02:01	63	22	YSNVTGFHT	YSNVTGFHT	0.00035	22	0.00035	22	TRUE	TRUE
1	SFGGVSVIT	577	585	9	HLA-A*02:01	577	22	SFGGVSVIT	SFGGVSVIT	0.000349	22	0.000349	22	TRUE	TRUE
1	AAYFVGYLK	250	258	9	HLA-A*02:01	250	22	AAYFVGYLK	AAYFVGYLK	0.000348	22	0.000348	22	TRUE	TRUE
1	YIKWPWYVW	1191	1199	9	HLA-A*02:01	1191	22	YIKWPWYVW	YIKWPWYVW	0.000347	22	0.000347	22	TRUE	TRUE
1	QYGSFCTQL	737	745	9	HLA-A*02:01	737	22	QYGSFCTQL	QYGSFCTQL	0.000346	22	0.000346	22	TRUE	TRUE
1	DSTECANLL	727	735	9	HLA-A*02:01	727	22	DSTECANLL	DSTECANLL	0.000342	22	0.000342	22	TRUE	TRUE
1	GVIADYNYK	403	411	9	HLA-A*02:01	403	22	GVIADYNYK	GVIADYNYK	0.00034	22	0.00034	22	TRUE	TRUE
1	LGADSSIAY	681	689	9	HLA-A*02:01	681	22	LGADSSIAY	LGADSSIAY	0.000336	22	0.000336	22	TRUE	TRUE
1	TILLCCMTS	1213	1221	9	HLA-A*02:01	1213	22	TILLCCMTS	TILLCCMTS	0.000336	22	0.000336	22	TRUE	TRUE
1	SLDVSEKSG	169	177	9	HLA-A*02:01	169	22	SLDVSEKSG	SLDVSEKSG	0.000332	22	0.000332	22	TRUE	TRUE
1	FELCDNPFF	130	138	9	HLA-A*02:01	130	22	FELCDNPFF	FELCDNPFF	0.000331	22	0.000331	22	TRUE	TRUE
1	NFKHLREFV	178	186	9	HLA-A*02:01	178	22	NFKHLREFV	NFKHLREFV	0.000331	22	0.000331	22	TRUE	TRUE
1	PLKPTKRSF	791	799	9	HLA-A*02:01	791	22	PLKPTKRSF	PLKPTKRSF	0.000331	22	0.000331	22	TRUE	TRUE
1	AFSPAQDIW	237	245	9	HLA-A*02:01	237	22	AFSPAQDIW	AFSPAQDIW	0.000329	22	0.000329	22	TRUE	TRUE
1	RGVYYPDEI	38	46	9	HLA-A*02:01	38	22	RGVYYPDEI	RGVYYPDEI	0.000328	22	0.000328	22	TRUE	TRUE
1	RSTSQKSIV	667	675	9	HLA-A*02:01	667	22	RSTSQKSIV	RSTSQKSIV	0.000328	22	0.000328	22	TRUE	TRUE
1	VKGDDVRQI	389	397	9	HLA-A*02:01	389	22	VKGDDVRQI	VKGDDVRQI	0.000327	22	0.000327	22	TRUE	TRUE
1	FTDSVRDPK	558	566	9	HLA-A*02:01	558	22	FTDSVRDPK	FTDSVRDPK	0.000323	22	0.000323	22	TRUE	TRUE
1	GKYEQYIKW	1186	1194	9	HLA-A*02:01	1186	22	GKYEQYIKW	GKYEQYIKW	0.000323	22	0.000323	22	TRUE	TRUE
1	FSNVYADSF	379	387	9	HLA-A*02:01	379	22	FSNVYADSF	FSNVYADSF	0.000322	22	0.000322	22	TRUE	TRUE
1	IFDNAFNCT	152	160	9	HLA-A*02:01	152	22	IFDNAFNCT	IFDNAFNCT	0.000321	22	0.000321	22	TRUE	TRUE
1	GAEHVDTSY	638	646	9	HLA-A*02:01	638	22	GAEHVDTSY	GAEHVDTSY	0.000316	22	0.000316	22	TRUE	TRUE
1	VSKPMGTQT	140	148	9	HLA-A*02:01	140	22	VSKPMGTQT	VSKPMGTQT	0.000316	22	0.000316	22	TRUE	TRUE
1	AICHEGKAY	1062	1070	9	HLA-A*02:01	1062	22	AICHEGKAY	AICHEGKAY	0.000314	22	0.000314	22	TRUE	TRUE
1	AYFPREGVF	1069	1077	9	HLA-A*02:01	1069	22	AYFPREGVF	AYFPREGVF	0.000314	22	0.000314	22	TRUE	TRUE
1	KFDEDDSEP	1237	1245	9	HLA-A*02:01	1237	22	KFDEDDSEP	KFDEDDSEP	0.000314	22	0.000314	22	TRUE	TRUE
1	LIDLQELGK	1179	1187	9	HLA-A*02:01	1179	23	LIDLQELGK	LIDLQELGK	0.000309	23	0.000309	23	TRUE	TRUE
1	AVSKPMGTQ	139	147	9	HLA-A*02:01	139	23	AVSKPMGTQ	AVSKPMGTQ	0.000308	23	0.000308	23	TRUE	TRUE
1	NCVADYSVL	347	355	9	HLA-A*02:01	347	23	NCVADYSVL	NCVADYSVL	0.000307	23	0.000307	23	TRUE	TRUE
1	AYFVGYLKP	251	259	9	HLA-A*02:01	251	23	AYFVGYLKP	AYFVGYLKP	0.000305	23	0.000305	23	TRUE	TRUE
1	SSIAYSNNT	685	693	9	HLA-A*02:01	685	23	SSIAYSNNT	SSIAYSNNT	0.000304	23	0.000304	23	TRUE	TRUE
1	IFLLFLTLT	3	11	9	HLA-A*02:01	3	23	IFLLFLTLT	IFLLFLTLT	0.000303	23	0.000303	23	TRUE	TRUE
1	TTAPAICHE	1058	1066	9	HLA-A*02:01	1058	23	TTAPAICHE	TTAPAICHE	0.0003	23	0.0003	23	TRUE	TRUE
1	EIRASANLA	999	1007	9	HLA-A*02:01	999	23	EIRASANLA	EIRASANLA	0.000299	23	0.000299	23	TRUE	TRUE
1	EVFNATKFP	327	335	9	HLA-A*02:01	327	23	EVFNATKFP	EVFNATKFP	0.000299	23	0.000299	23	TRUE	TRUE
1	AGAALQIPF	872	880	9	HLA-A*02:01	872	23	AGAALQIPF	AGAALQIPF	0.000298	23	0.000298	23	TRUE	TRUE
1	NTLVKQLSS	942	950	9	HLA-A*02:01	942	23	NTLVKQLSS	NTLVKQLSS	0.000295	23	0.000295	23	TRUE	TRUE
1	GVTQNVLYE	892	900	9	HLA-A*02:01	892	23	GVTQNVLYE	GVTQNVLYE	0.000294	23	0.000294	23	TRUE	TRUE
1	TINHTFGNP	71	79	9	HLA-A*02:01	71	23	TINHTFGNP	TINHTFGNP	0.000294	23	0.000294	23	TRUE	TRUE
1	IADYNYKLP	405	413	9	HLA-A*02:01	405	23	IADYNYKLP	IADYNYKLP	0.000292	23	0.000292	23	TRUE	TRUE
1	FEIDKGIYQ	293	301	9	HLA-A*02:01	293	23	FEIDKGIYQ	FEIDKGIYQ	0.000291	23	0.000291	23	TRUE	TRUE
1	KYFGGFNFS	777	785	9	HLA-A*02:01	777	23	KYFGGFNFS	KYFGGFNFS	0.000291	23	0.000291	23	TRUE	TRUE
1	PQAAPHGVV	1035	1043	9	HLA-A*02:01	1035	23	PQAAPHGVV	PQAAPHGVV	0.000288	23	0.000288	23	TRUE	TRUE
1	ASANLAATK	1002	1010	9	HLA-A*02:01	1002	23	ASANLAATK	ASANLAATK	0.000285	23	0.000285	23	TRUE	TRUE
1	SVKSFEIDK	289	297	9	HLA-A*02:01	289	23	SVKSFEIDK	SVKSFEIDK	0.00028	23	0.00028	23	TRUE	TRUE
1	TFFSTFKCY	359	367	9	HLA-A*02:01	359	23	TFFSTFKCY	TFFSTFKCY	0.00028	23	0.00028	23	TRUE	TRUE
1	INHTFGNPV	72	80	9	HLA-A*02:01	72	23	INHTFGNPV	INHTFGNPV	0.000279	23	0.000279	23	TRUE	TRUE
1	MFIFLLFLT	1	9	9	HLA-A*02:01	1	23	MFIFLLFLT	MFIFLLFLT	0.000279	23	0.000279	23	TRUE	TRUE
1	RALSGIAAE	747	755	9	HLA-A*02:01	747	23	RALSGIAAE	RALSGIAAE	0.000279	23	0.000279	23	TRUE	TRUE
1	FKHLREFVF	179	187	9	HLA-A*02:01	179	24	FKHLREFVF	FKHLREFVF	0.000276	24	0.000276	24	TRUE	TRUE
1	NATKFPSVY	330	338	9	HLA-A*02:01	330	24	NATKFPSVY	NATKFPSVY	0.000272	24	0.000272	24	TRUE	TRUE
1	GNFKHLREF	177	185	9	HLA-A*02:01	177	24	GNFKHLREF	GNFKHLREF	0.000271	24	0.000271	24	TRUE	TRUE
1	RSDTLYLTQ	48	56	9	HLA-A*02:01	48	24	RSDTLYLTQ	RSDTLYLTQ	0.000271	24	0.000271	24	TRUE	TRUE
1	ECANLLLQY	730	738	9	HLA-A*02:01	730	24	ECANLLLQY	ECANLLLQY	0.00027	24	0.00027	24	TRUE	TRUE
1	TMSLGADSS	678	686	9	HLA-A*02:01	678	24	TMSLGADSS	TMSLGADSS	0.000268	24	0.000268	24	TRUE	TRUE
1	DEIFRSDTL	44	52	9	HLA-A*02:01	44	24	DEIFRSDTL	DEIFRSDTL	0.000267	24	0.000267	24	TRUE	TRUE
1	PFKDGIYFA	82	90	9	HLA-A*02:01	82	24	PFKDGIYFA	PFKDGIYFA	0.000264	24	0.000264	24	TRUE	TRUE
1	AALVSGTAT	857	865	9	HLA-A*02:01	857	24	AALVSGTAT	AALVSGTAT	0.000263	24	0.000263	24	TRUE	TRUE
1	FYTTTGIGY	483	491	9	HLA-A*02:01	483	24	FYTTTGIGY	FYTTTGIGY	0.000262	24	0.000262	24	TRUE	TRUE
1	SNCVADYSV	346	354	9	HLA-A*02:01	346	24	SNCVADYSV	SNCVADYSV	0.00026	24	0.00026	24	TRUE	TRUE
1	GTITDAVDC	270	278	9	HLA-A*02:01	270	24	GTITDAVDC	GTITDAVDC	0.000256	24	0.000256	24	TRUE	TRUE
1	ITPGTNASS	584	592	9	HLA-A*02:01	584	24	ITPGTNASS	ITPGTNASS	0.000253	24	0.000253	24	TRUE	TRUE
1	SEILDISPC	568	576	9	HLA-A*02:01	568	24	SEILDISPC	SEILDISPC	0.000253	24	0.000253	24	TRUE	TRUE
1	SWFITQRNF	1083	1091	9	HLA-A*02:01	1083	24	SWFITQRNF	SWFITQRNF	0.000253	24	0.000253	24	TRUE	TRUE
1	KPTTFMLKY	258	266	9	HLA-A*02:01	258	24	KPTTFMLKY	KPTTFMLKY	0.000251	24	0.000251	24	TRUE	TRUE
1	MNNKSQSVI	107	115	9	HLA-A*02:01	107	24	MNNKSQSVI	MNNKSQSVI	0.000251	24	0.000251	24	TRUE	TRUE
1	VFLHVTYVP	1043	1051	9	HLA-A*02:01	1043	24	VFLHVTYVP	VFLHVTYVP	0.000251	24	0.000251	24	TRUE	TRUE
1	GFNFSQILP	781	789	9	HLA-A*02:01	781	24	GFNFSQILP	GFNFSQILP	0.000249	24	0.000249	24	TRUE	TRUE
1	SFCTQLNRA	740	748	9	HLA-A*02:01	740	24	SFCTQLNRA	SFCTQLNRA	0.000249	24	0.000249	24	TRUE	TRUE
1	FGGVSVITP	578	586	9	HLA-A*02:01	578	25	FGGVSVITP	FGGVSVITP	0.000248	25	0.000248	25	TRUE	TRUE
1	IGAGICASY	652	660	9	HLA-A*02:01	652	25	IGAGICASY	IGAGICASY	0.000247	25	0.000247	25	TRUE	TRUE
1	CTTFDDVQA	19	27	9	HLA-A*02:01	19	25	CTTFDDVQA	CTTFDDVQA	0.000246	25	0.000246	25	TRUE	TRUE
1	AYRFNGIGV	885	893	9	HLA-A*02:01	885	25	AYRFNGIGV	AYRFNGIGV	0.000242	25	0.000242	25	TRUE	TRUE
1	NKDGFLYVY	189	197	9	HLA-A*02:01	189	25	NKDGFLYVY	NKDGFLYVY	0.000242	25	0.000242	25	TRUE	TRUE
1	IGVTQNVLY	891	899	9	HLA-A*02:01	891	25	IGVTQNVLY	IGVTQNVLY	0.000239	25	0.000239	25	TRUE	TRUE
1	KSNVVRGWV	94	102	9	HLA-A*02:01	94	25	KSNVVRGWV	KSNVVRGWV	0.000238	25	0.000238	25	TRUE	TRUE
1	LSRLDKVEA	963	971	9	HLA-A*02:01	963	25	LSRLDKVEA	LSRLDKVEA	0.000235	25	0.000235	25	TRUE	TRUE
1	MQMAYRFNG	882	890	9	HLA-A*02:01	882	25	MQMAYRFNG	MQMAYRFNG	0.000234	25	0.000234	25	TRUE	TRUE
1	KYFKNHTSP	1136	1144	9	HLA-A*02:01	1136	25	KYFKNHTSP	KYFKNHTSP	0.000231	25	0.000231	25	TRUE	TRUE
1	GVFVFNGTS	1075	1083	9	HLA-A*02:01	1075	25	GVFVFNGTS	GVFVFNGTS	0.00023	25	0.00023	25	TRUE	TRUE
1	NTVYDPLQP	1117	1125	9	HLA-A*02:01	1117	25	NTVYDPLQP	NTVYDPLQP	0.00023	25	0.00023	25	TRUE	TRUE
1	VFVFNGTSW	1076	1084	9	HLA-A*02:01	1076	25	VFVFNGTSW	VFVFNGTSW	0.00023	25	0.00023	25	TRUE	TRUE
1	EQYIKWPWY	1189	1197	9	HLA-A*02:01	1189	25	EQYIKWPWY	EQYIKWPWY	0.000227	25	0.000227	25	TRUE	TRUE
1	TFEYISDAF	160	168	9	HLA-A*02:01	160	25	TFEYISDAF	TFEYISDAF	0.000226	25	0.000226	25	TRUE	TRUE
1	ILSRLDKVE	962	970	9	HLA-A*02:01	962	25	ILSRLDKVE	ILSRLDKVE	0.000225	25	0.000225	25	TRUE	TRUE
1	YISDAFSLD	163	171	9	HLA-A*02:01	163	25	YISDAFSLD	YISDAFSLD	0.000225	25	0.000225	25	TRUE	TRUE
1	HGVVFLHVT	1040	1048	9	HLA-A*02:01	1040	25	HGVVFLHVT	HGVVFLHVT	0.000223	25	0.000223	25	TRUE	TRUE
1	HVTYVPSQE	1046	1054	9	HLA-A*02:01	1046	25	HVTYVPSQE	HVTYVPSQE	0.000222	25	0.000222	25	TRUE	TRUE
1	LGFIAGLIA	1200	1208	9	HLA-A*02:01	1200	25	LGFIAGLIA	LGFIAGLIA	0.000222	25	0.000222	25	TRUE	TRUE
1	ATKLNDLCF	371	379	9	HLA-A*02:01	371	25	ATKLNDLCF	ATKLNDLCF	0.000221	25	0.000221	25	TRUE	TRUE
1	IQESLTTTS	916	924	9	HLA-A*02:01	916	26	IQESLTTTS	IQESLTTTS	0.00022	26	0.00022	26	TRUE	TRUE

seq #	peptide	start	end	peptide length	allele	peptide index	median binding	netmhcpan_el	netmhcpan_el	netmhcpan_el	netmhcpan_el	legacy score	legacy rank	scores equal	percentiles equal	
1	KSGNFKHLR	175	183	9	HLA-A*02:01	175	26	KSGNFKHLR	KSGNFKHLR	0.00022	26	0.00022	26	TRUE	TRUE	
1	DKVEAEVQI	967	975	9	HLA-A*02:01	967	26	DKVEAEVQI	DKVEAEVQI	0.000219	26	0.000219	26	TRUE	TRUE	
1	SNVYADSFV	380	388	9	HLA-A*02:01	380	26	SNVYADSFV	SNVYADSFV	0.000219	26	0.000219	26	TRUE	TRUE	
1	TPAWRIYST	616	624	9	HLA-A*02:01	616	26	TPAWRIYST	TPAWRIYST	0.000217	26	0.000217	26	TRUE	TRUE	
1	MSFPQAAPH	1032	1040	9	HLA-A*02:01	1032	26	MSFPQAAPH	MSFPQAAPH	0.000215	26	0.000215	26	TRUE	TRUE	
1	ISITTEVMP	702	710	9	HLA-A*02:01	702	26	ISITTEVMP	ISITTEVMP	0.000213	26	0.000213	26	TRUE	TRUE	
1	NCDVVIGII	1107	1115	9	HLA-A*02:01	1107	26	NCDVVIGII	NCDVVIGII	0.000213	26	0.000213	26	TRUE	TRUE	
1	ADYSVLYNS	350	358	9	HLA-A*02:01	350	26	ADYSVLYNS	ADYSVLYNS	0.00021	26	0.00021	26	TRUE	TRUE	
1	KPMGTQTHT	142	150	9	HLA-A*02:01	142	26	KPMGTQTHT	KPMGTQTHT	0.00021	26	0.00021	26	TRUE	TRUE	
1	LADAGFMKQ	810	818	9	HLA-A*02:01	810	26	LADAGFMKQ	LADAGFMKQ	0.00021	26	0.00021	26	TRUE	TRUE	
1	NDYGFYTTT	479	487	9	HLA-A*02:01	479	26	NDYGFYTTT	NDYGFYTTT	0.00021	26	0.00021	26	TRUE	TRUE	
1	RFNGIGVTQ	887	895	9	HLA-A*02:01	887	26	RFNGIGVTQ	RFNGIGVTQ	0.000209	26	0.000209	26	TRUE	TRUE	
1	ADAGFMKQY	811	819	9	HLA-A*02:01	811	26	ADAGFMKQY	ADAGFMKQY	0.000206	26	0.000206	26	TRUE	TRUE	
1	FELLNAPAT	501	509	9	HLA-A*02:01	501	26	FELLNAPAT	FELLNAPAT	0.000205	26	0.000205	26	TRUE	TRUE	
1	VSGNCDVVI	1104	1112	9	HLA-A*02:01	1104	26	VSGNCDVVI	VSGNCDVVI	0.000203	26	0.000203	26	TRUE	TRUE	
1	YNYKYRYLR	436	444	9	HLA-A*02:01	436	27	YNYKYRYLR	YNYKYRYLR	0.000198	27	0.000198	27	TRUE	TRUE	
1	YSNNTIAIP	689	697	9	HLA-A*02:01	689	27	YSNNTIAIP	YSNNTIAIP	0.000198	27	0.000198	27	TRUE	TRUE	
1	NFRAILTAF	230	238	9	HLA-A*02:01	230	27	NFRAILTAF	NFRAILTAF	0.000196	27	0.000196	27	TRUE	TRUE	
1	IPIGAGICA	650	658	9	HLA-A*02:01	650	27	IPIGAGICA	IPIGAGICA	0.000195	27	0.000195	27	TRUE	TRUE	
1	NCYWPLNDY	473	481	9	HLA-A*02:01	473	27	NCYWPLNDY	NCYWPLNDY	0.000195	27	0.000195	27	TRUE	TRUE	
1	GSFCTQLNR	739	747	9	HLA-A*02:01	739	27	GSFCTQLNR	GSFCTQLNR	0.000193	27	0.000193	27	TRUE	TRUE	
1	TEVMPVSMA	706	714	9	HLA-A*02:01	706	27	TEVMPVSMA	TEVMPVSMA	0.000193	27	0.000193	27	TRUE	TRUE	
1	VTILLCCMT	1212	1220	9	HLA-A*02:01	1212	27	VTILLCCMT	VTILLCCMT	0.000192	27	0.000192	27	TRUE	TRUE	
1	LTPAWRIYS	615	623	9	HLA-A*02:01	615	27	LTPAWRIYS	LTPAWRIYS	0.000191	27	0.000191	27	TRUE	TRUE	
1	KHLREFVFK	180	188	9	HLA-A*02:01	180	27	KHLREFVFK	KHLREFVFK	0.00019	27	0.00019	27	TRUE	TRUE	
1	SFVVKGDDV	386	394	9	HLA-A*02:01	386	27	SFVVKGDDV	SFVVKGDDV	0.00019	27	0.00019	27	TRUE	TRUE	
1	TNLCPFGEV	320	328	9	HLA-A*02:01	320	27	TNLCPFGEV	TNLCPFGEV	0.000187	27	0.000187	27	TRUE	TRUE	
1	TFKCYGVSA	363	371	9	HLA-A*02:01	363	27	TFKCYGVSA	TFKCYGVSA	0.000185	27	0.000185	27	TRUE	TRUE	
1	TGIGYQPYR	487	495	9	HLA-A*02:01	487	27	TGIGYQPYR	TGIGYQPYR	0.000184	27	0.000184	27	TRUE	TRUE	
1	CLIGAEHVD	635	643	9	HLA-A*02:01	635	27	CLIGAEHVD	CLIGAEHVD	0.000182	27	0.000182	27	TRUE	TRUE	
1	ATEKSNVVR	91	99	9	HLA-A*02:01	91	28	ATEKSNVVR	ATEKSNVVR	0.000177	28	0.000177	28	TRUE	TRUE	
1	GFNTLKPIF	212	220	9	HLA-A*02:01	212	28	GFNTLKPIF	GFNTLKPIF	0.000177	28	0.000177	28	TRUE	TRUE	
1	GPKLSTDLI	512	520	9	HLA-A*02:01	512	28	GPKLSTDLI	GPKLSTDLI	0.000177	28	0.000177	28	TRUE	TRUE	
1	QTSNFRVVP	301	309	9	HLA-A*02:01	301	28	QTSNFRVVP	QTSNFRVVP	0.000177	28	0.000177	28	TRUE	TRUE	
1	YEQYIKWPW	1188	1196	9	HLA-A*02:01	1188	28	YEQYIKWPW	YEQYIKWPW	0.000176	28	0.000176	28	TRUE	TRUE	
1	IGAEHVDTS	637	645	9	HLA-A*02:01	637	28	IGAEHVDTS	IGAEHVDTS	0.000174	28	0.000174	28	TRUE	TRUE	
1	IGIINNTVY	1112	1120	9	HLA-A*02:01	1112	28	IGIINNTVY	IGIINNTVY	0.000174	28	0.000174	28	TRUE	TRUE	
1	APHGVVFLH	1038	1046	9	HLA-A*02:01	1038	28	APHGVVFLH	APHGVVFLH	0.000171	28	0.000171	28	TRUE	TRUE	
1	FDDVQAPNY	22	30	9	HLA-A*02:01	22	28	FDDVQAPNY	FDDVQAPNY	0.00017	28	0.00017	28	TRUE	TRUE	
1	GEVFNATKF	326	334	9	HLA-A*02:01	326	28	GEVFNATKF	GEVFNATKF	0.000169	28	0.000169	28	TRUE	TRUE	
1	INARDLICA	826	834	9	HLA-A*02:01	826	28	INARDLICA	INARDLICA	0.000168	28	0.000168	28	TRUE	TRUE	
1	KGIYQTSNF	297	305	9	HLA-A*02:01	297	28	KGIYQTSNF	KGIYQTSNF	0.000168	28	0.000168	28	TRUE	TRUE	
1	KGYHLMSFP	1027	1035	9	HLA-A*02:01	1027	28	KGYHLMSFP	KGYHLMSFP	0.000167	28	0.000167	28	TRUE	TRUE	
1	KTSVDCNMY	715	723	9	HLA-A*02:01	715	28	KTSVDCNMY	KTSVDCNMY	0.000167	28	0.000167	28	TRUE	TRUE	
1	VFKNKDGFL	186	194	9	HLA-A*02:01	186	28	VFKNKDGFL	VFKNKDGFL	0.000167	28	0.000167	28	TRUE	TRUE	
1	FDNAFNCTF	153	161	9	HLA-A*02:01	153	28	FDNAFNCTF	FDNAFNCTF	0.000165	28	0.000165	28	TRUE	TRUE	
1	SCLKGACSC	1224	1232	9	HLA-A*02:01	1224	29	SCLKGACSC	SCLKGACSC	0.000164	29	0.000164	29	TRUE	TRUE	
1	TTDNTFVSG	1098	1106	9	HLA-A*02:01	1098	29	TTDNTFVSG	TTDNTFVSG	0.000164	29	0.000164	29	TRUE	TRUE	
1	IYSTGNNVF	621	629	9	HLA-A*02:01	621	29	IYSTGNNVF	IYSTGNNVF	0.000163	29	0.000163	29	TRUE	TRUE	
1	VEAEVQIDR	969	977	9	HLA-A*02:01	969	29	VEAEVQIDR	VEAEVQIDR	0.000163	29	0.000163	29	TRUE	TRUE	
1	ARDLICAQK	828	836	9	HLA-A*02:01	828	29	ARDLICAQK	ARDLICAQK	0.000161	29	0.000161	29	TRUE	TRUE	
1	QVKQMYKTP	766	774	9	HLA-A*02:01	766	29	QVKQMYKTP	QVKQMYKTP	0.000161	29	0.000161	29	TRUE	TRUE	
1	DVSEKSGNF	171	179	9	HLA-A*02:01	171	29	DVSEKSGNF	DVSEKSGNF	0.00016	29	0.00016	29	TRUE	TRUE	
1	GTGVLTPSS	534	542	9	HLA-A*02:01	534	29	GTGVLTPSS	GTGVLTPSS	0.000158	29	0.000158	29	TRUE	TRUE	
1	AFSLDVSEK	167	175	9	HLA-A*02:01	167	29	AFSLDVSEK	AFSLDVSEK	0.000154	29	0.000154	29	TRUE	TRUE	
1	GNNVFQTQA	625	633	9	HLA-A*02:01	625	29	GNNVFQTQA	GNNVFQTQA	0.000153	29	0.000153	29	TRUE	TRUE	
1	SPDGKPCTP	461	469	9	HLA-A*02:01	461	29	SPDGKPCTP	SPDGKPCTP	0.000153	29	0.000153	29	TRUE	TRUE	
1	TVSLLRSTS	662	670	9	HLA-A*02:01	662	29	TVSLLRSTS	TVSLLRSTS	0.000151	29	0.000151	29	TRUE	TRUE	
1	TDVSTAIHA	604	612	9	HLA-A*02:01	604	29	TDVSTAIHA	TDVSTAIHA	0.00015	29	0.00015	29	TRUE	TRUE	
1	MVTILLCCM	1211	1219	9	HLA-A*02:01	1211	29	MVTILLCCM	MVTILLCCM	0.000149	29	0.000149	29	TRUE	TRUE	
1	LLCCMTSCC	1215	1223	9	HLA-A*02:01	1215	30	LLCCMTSCC	LLCCMTSCC	0.000148	30	0.000148	30	TRUE	TRUE	
1	QELGKYEQY	1183	1191	9	HLA-A*02:01	1183	30	QELGKYEQY	QELGKYEQY	0.000148	30	0.000148	30	TRUE	TRUE	
1	LKYDENGTI	264	272	9	HLA-A*02:01	264	30	LKYDENGTI	LKYDENGTI	0.000145	30	0.000145	30	TRUE	TRUE	
1	PFYSNVTGF	61	69	9	HLA-A*02:01	61	30	PFYSNVTGF	PFYSNVTGF	0.000145	30	0.000145	30	TRUE	TRUE	
1	TTTSTALGK	921	929	9	HLA-A*02:01	921	30	TTTSTALGK	TTTSTALGK	0.000145	30	0.000145	30	TRUE	TRUE	
1	GNYNYKYRY	434	442	9	HLA-A*02:01	434	30	GNYNYKYRY	GNYNYKYRY	0.000144	30	0.000144	30	TRUE	TRUE	
1	VLPPLLTDD	842	850	9	HLA-A*02:01	842	30	VLPPLLTDD	VLPPLLTDD	0.000144	30	0.000144	30	TRUE	TRUE	
1	INNTVYDPL	1115	1123	9	HLA-A*02:01	1115	30	INNTVYDPL	INNTVYDPL	0.000143	30	0.000143	30	TRUE	TRUE	
1	RDISNVPFS	453	461	9	HLA-A*02:01	453	30	RDISNVPFS	RDISNVPFS	0.000143	30	0.000143	30	TRUE	TRUE	
1	VIPFKDGIY	80	88	9	HLA-A*02:01	80	30	VIPFKDGIY	VIPFKDGIY	0.000143	30	0.000143	30	TRUE	TRUE	
1	VTGFHTINH	66	74	9	HLA-A*02:01	66	30	VTGFHTINH	VTGFHTINH	0.000142	30	0.000142	30	TRUE	TRUE	
1	NHTSPDVDL	1140	1148	9	HLA-A*02:01	1140	30	NHTSPDVDL	NHTSPDVDL	0.000141	30	0.000141	30	TRUE	TRUE	
1	YTMSLGADS	677	685	9	HLA-A*02:01	677	30	YTMSLGADS	YTMSLGADS	0.000141	30	0.000141	30	TRUE	TRUE	
1	LKPIFKLPL	216	224	9	HLA-A*02:01	216	30	LKPIFKLPL	LKPIFKLPL	0.00014	30	0.00014	30	TRUE	TRUE	
1	QDIWGTSAA	242	250	9	HLA-A*02:01	242	30	QDIWGTSAA	QDIWGTSAA	0.000138	30	0.000138	30	TRUE	TRUE	
1	RDLPSGFNT	207	215	9	HLA-A*02:01	207	30	RDLPSGFNT	RDLPSGFNT	0.000136	30	0.000136	30	TRUE	TRUE	
1	RFQPFQQFG	544	552	9	HLA-A*02:01	544	31	RFQPFQQFG	RFQPFQQFG	0.000133	31	0.000133	31	TRUE	TRUE	
1	TQQLIRAAE	991	999	9	HLA-A*02:01	991	31	TQQLIRAAE	TQQLIRAAE	0.000133	31	0.000133	31	TRUE	TRUE	
1	IDRLITGRL	975	983	9	HLA-A*02:01	975	31	IDRLITGRL	IDRLITGRL	0.000132	31	0.000132	31	TRUE	TRUE	
1	TSSMRGVYY	34	42	9	HLA-A*02:01	34	31	TSSMRGVYY	TSSMRGVYY	0.000132	31	0.000132	31	TRUE	TRUE	
1	KQYGECLGD	817	825	9	HLA-A*02:01	817	31	KQYGECLGD	KQYGECLGD	0.000131	31	0.000131	31	TRUE	TRUE	
1	QALNTLVKQ	939	947	9	HLA-A*02:01	939	31	QALNTLVKQ	QALNTLVKQ	0.000131	31	0.000131	31	TRUE	TRUE	
1	QKSIVAYTM	671	679	9	HLA-A*02:01	671	31	QKSIVAYTM	QKSIVAYTM	0.000131	31	0.000131	31	TRUE	TRUE	
1	ERDISNVPF	452	460	9	HLA-A*02:01	452	31	ERDISNVPF	ERDISNVPF	0.00013	31	0.00013	31	TRUE	TRUE	
1	GDINARDLI	824	832	9	HLA-A*02:01	824	31	GDINARDLI	GDINARDLI	0.00013	31	0.00013	31	TRUE	TRUE	
1	IAAEQDRNT	752	760	9	HLA-A*02:01	752	31	IAAEQDRNT	IAAEQDRNT	0.000128	31	0.000128	31	TRUE	TRUE	
1	NYNYKYRYL	435	443	9	HLA-A*02:01	435	31	NYNYKYRYL	NYNYKYRYL	0.000126	31	0.000126	31	TRUE	TRUE	
1	YKGYQPIDV	197	205	9	HLA-A*02:01	197	31	YKGYQPIDV	YKGYQPIDV	0.000126	31	0.000126	31	TRUE	TRUE	
1	QQFGRDVSD	549	557	9	HLA-A*02:01	549	31	QQFGRDVSD	QQFGRDVSD	0.000125	31	0.000125	31	TRUE	TRUE	
1	EHVDTSYEC	640	648	9	HLA-A*02:01	640	32	EHVDTSYEC	EHVDTSYEC	0.000123	32	0.000123	32	TRUE	TRUE	
1	TQLNRALSG	743	751	9	HLA-A*02:01	743	32	TQLNRALSG	TQLNRALSG	0.000123	32	0.000123	32	TRUE	TRUE	
1	SQSVIIINN	111	119	9	HLA-A*02:01	111	32	SQSVIIINN	SQSVIIINN	0.000122	32	0.000122	32	TRUE	TRUE	
1	ENGTITDAV	268	276	9	HLA-A*02:01	268	32	ENGTITDAV	ENGTITDAV	0.000121	32	0.000121	32	TRUE	TRUE	
1	CLGDINARD	822	830	9	HLA-A*02:01	822	32	CLGDINARD	CLGDINARD	0.00012	32	0.00012	32	TRUE	TRUE	
1	DLFLPFYSN	57	65	9	HLA-A*02:01	57	32	DLFLPFYSN	DLFLPFYSN	0.000119	32	0.000119	32	TRUE	TRUE	
1	FQQFGRDVS	548	556	9	HLA-A*02:01	548	32	FQQFGRDVS	FQQFGRDVS	0.000119	32	0.000119	32	TRUE	TRUE	
1	NKSQSVIII	109	117	9	HLA-A*02:01	109	32	NKSQSVIII	NKSQSVIII	0.000119	32	0.000119	32	TRUE	TRUE	
1	VFQTQAGCL	628	636	9	HLA-A*02:01	628	32	VFQTQAGCL	VFQTQAGCL	0.000118	32	0.000118	32	TRUE	TRUE	
1	GIINNTVYD	1113	1121	9	HLA-A*02:01	1113	32	GIINNTVYD	GIINNTVYD	0.000117	32	0.000117	32	TRUE	TRUE	
1	FGRDVSDFT	551	559	9	HLA-A*02:01	551	32	FGRDVSDFT	FGRDVSDFT	0.000116	32	0.000116	32	TRUE	TRUE	
1	FPSVYAWER	334	342	9	HLA-A*02:01	334	32	FPSVYAWER	FPSVYAWER	0.000116	32	0.000116	32	TRUE	TRUE	
1	YFGGFNFSQ	778	786	9	HLA-A*02:01	778	32	YFGGFNFSQ	YFGGFNFSQ	0.000116	32	0.000116	32	TRUE	TRUE	
1	ICGDSTECA	724	732	9	HLA-A*02:01	724	32	ICGDSTECA	ICGDSTECA	0.000115	32	0.000115	32	TRUE	TRUE	
1	LNDYGFYTT	478	486	9	HLA-A*02:01	478	32	LNDYGFYTT	LNDYGFYTT	0.000115	32	0.000115	32	TRUE	TRUE	
1	TSTGNYNYK	431	439	9	HLA-A*02:01	431	33	TSTGNYNYK	TSTGNYNYK	0.000113	33	0.000113	33	TRUE	TRUE	
1	AAEQDRNTR	753	761	9	HLA-A*02:01	753	33	AAEQDRNTR	AAEQDRNTR	0.000112	33	0.000112	33	TRUE	TRUE	
1	SRLDKVEAE	964	972	9	HLA-A*02:01	964	33	SRLDKVEAE	SRLDKVEAE	0.000111	33	0.000111	33	TRUE	TRUE	
1	TAIHADQLT	608	616	9	HLA-A*02:01	608	33	TAIHADQLT	TAIHADQLT	0.000111	33	0.000111	33	TRUE	TRUE	
1	VFGSTMNNK	102	110	9	HLA-A*02:01	102	33	VFGSTMNNK	VFGSTMNNK	0.00011	33	0.00011	33	TRUE	TRUE	
1	LPSGFNTLK	209	217	9	HLA-A*02:01	209	33	LPSGFNTLK	LPSGFNTLK	0.000109	33	0.000109	33	TRUE	TRUE	
1	SSNFGAISS	949	957	9	HLA-A*02:01	949	33	SSNFGAISS	SSNFGAISS	0.000108	33	0.000108	33	TRUE	TRUE	
1	IRASANLAA	1000	1008	9	HLA-A*02:01	1000	33	IRASANLAA	IRASANLAA	0.000106	33	0.000106	33	TRUE	TRUE	
1	SATKLNDLC	370	378	9	HLA-A*02:01	370	33	SATKLNDLC	SATKLNDLC	0.000106	33	0.000106	33	TRUE	TRUE	
1	NYKLPDDFM	409	417	9	HLA-A*02:01	409	33	NYKLPDDFM	NYKLPDDFM	0.000105	33	0.000105	33	TRUE	TRUE	
1	APGQTGVIA	398	406	9	HLA-A*02:01	398	34	APGQTGVIA	APGQTGVIA	0.000104	34	0.000104	34	TRUE	TRUE	

seq #	peptide	start	end	peptide length	allele	peptide index	median binding	netmhcpan_el	netmhcpan_el	netmhcpan_el	netmhcpan_el	legacy score	legacy rank	scores equal	percentiles equal
1	LIGAEHVDT	636	644	9	HLA-A*02:01	636	34	LIGAEHVDT	LIGAEHVDT	0.000104	34	0.000104	34	TRUE	TRUE
1	STMNNKSQS	105	113	9	HLA-A*02:01	105	34	STMNNKSQS	STMNNKSQS	0.000103	34	0.000103	34	TRUE	TRUE
1	LYNSTFFST	355	363	9	HLA-A*02:01	355	34	LYNSTFFST	LYNSTFFST	0.0001	34	0.0001	34	TRUE	TRUE
1	VNCTDVSTA	601	609	9	HLA-A*02:01	601	34	VNCTDVSTA	VNCTDVSTA	0.0001	34	0.0001	34	TRUE	TRUE
1	LRSTSQKSI	666	674	9	HLA-A*02:01	666	34	LRSTSQKSI	LRSTSQKSI	0.000099	34	9.90E-05	34	TRUE	TRUE
1	NASVVNIQK	1155	1163	9	HLA-A*02:01	1155	34	NASVVNIQK	NASVVNIQK	0.000099	34	9.90E-05	34	TRUE	TRUE
1	NGLTGTGVL	530	538	9	HLA-A*02:01	530	34	NGLTGTGVL	NGLTGTGVL	0.000099	34	9.90E-05	34	TRUE	TRUE
1	PCSFGGVSV	575	583	9	HLA-A*02:01	575	34	PCSFGGVSV	PCSFGGVSV	0.000098	34	9.80E-05	34	TRUE	TRUE
1	TGVLTPSSK	535	543	9	HLA-A*02:01	535	34	TGVLTPSSK	TGVLTPSSK	0.000097	34	9.70E-05	34	TRUE	TRUE
1	QPYRVVVLS	492	500	9	HLA-A*02:01	492	34	QPYRVVVLS	QPYRVVVLS	0.000096	34	9.60E-05	34	TRUE	TRUE
1	FLTLTSGSD	7	15	9	HLA-A*02:01	7	34	FLTLTSGSD	FLTLTSGSD	0.000095	34	9.50E-05	34	TRUE	TRUE
1	YSTGNNVFQ	622	630	9	HLA-A*02:01	622	34	YSTGNNVFQ	YSTGNNVFQ	0.000095	34	9.50E-05	34	TRUE	TRUE
1	QSLQTYVTQ	984	992	9	HLA-A*02:01	984	35	QSLQTYVTQ	QSLQTYVTQ	0.000094	35	9.40E-05	35	TRUE	TRUE
1	TSNFRVVPS	302	310	9	HLA-A*02:01	302	35	TSNFRVVPS	TSNFRVVPS	0.000093	35	9.30E-05	35	TRUE	TRUE
1	LKYFGGFNF	776	784	9	HLA-A*02:01	776	35	LKYFGGFNF	LKYFGGFNF	0.000092	35	9.20E-05	35	TRUE	TRUE
1	PALNCYWPL	470	478	9	HLA-A*02:01	470	35	PALNCYWPL	PALNCYWPL	0.00009	35	9.00E-05	35	TRUE	TRUE
1	YTTTGIGYQ	484	492	9	HLA-A*02:01	484	35	YTTTGIGYQ	YTTTGIGYQ	0.00009	35	9.00E-05	35	TRUE	TRUE
1	ERKKISNCV	341	349	9	HLA-A*02:01	341	35	ERKKISNCV	ERKKISNCV	0.000089	35	8.90E-05	35	TRUE	TRUE
1	DLQELGKYE	1181	1189	9	HLA-A*02:01	1181	35	DLQELGKYE	DLQELGKYE	0.000088	35	8.80E-05	35	TRUE	TRUE
1	GSDLDRCTT	13	21	9	HLA-A*02:01	13	35	GSDLDRCTT	GSDLDRCTT	0.000088	35	8.80E-05	35	TRUE	TRUE
1	SCCSCLKGA	1221	1229	9	HLA-A*02:01	1221	35	SCCSCLKGA	SCCSCLKGA	0.000088	35	8.80E-05	35	TRUE	TRUE
1	LTSGSDLDR	10	18	9	HLA-A*02:01	10	36	LTSGSDLDR	LTSGSDLDR	0.000086	36	8.60E-05	36	TRUE	TRUE
1	FERDISNVP	451	459	9	HLA-A*02:01	451	36	FERDISNVP	FERDISNVP	0.000085	36	8.50E-05	36	TRUE	TRUE
1	FGEVFNATK	325	333	9	HLA-A*02:01	325	36	FGEVFNATK	FGEVFNATK	0.000085	36	8.50E-05	36	TRUE	TRUE
1	GDSTECANL	726	734	9	HLA-A*02:01	726	36	GDSTECANL	GDSTECANL	0.000085	36	8.50E-05	36	TRUE	TRUE
1	TKRSFIEDL	795	803	9	HLA-A*02:01	795	36	TKRSFIEDL	TKRSFIEDL	0.000085	36	8.50E-05	36	TRUE	TRUE
1	NFSQILPDP	783	791	9	HLA-A*02:01	783	36	NFSQILPDP	NFSQILPDP	0.000084	36	8.40E-05	36	TRUE	TRUE
1	QHTSSMRGV	32	40	9	HLA-A*02:01	32	36	QHTSSMRGV	QHTSSMRGV	0.000084	36	8.40E-05	36	TRUE	TRUE
1	CGPKLSTDL	511	519	9	HLA-A*02:01	511	36	CGPKLSTDL	CGPKLSTDL	0.000083	36	8.30E-05	36	TRUE	TRUE
1	YNYKLPDDF	408	416	9	HLA-A*02:01	408	36	YNYKLPDDF	YNYKLPDDF	0.000082	36	8.20E-05	36	TRUE	TRUE
1	AGICASYHT	654	662	9	HLA-A*02:01	654	36	AGICASYHT	AGICASYHT	0.000081	36	8.10E-05	36	TRUE	TRUE
1	TITDAVDCS	271	279	9	HLA-A*02:01	271	36	TITDAVDCS	TITDAVDCS	0.000081	36	8.10E-05	36	TRUE	TRUE
1	DDMIAAYTA	849	857	9	HLA-A*02:01	849	36	DDMIAAYTA	DDMIAAYTA	0.00008	36	8.00E-05	36	TRUE	TRUE
1	VSEKSGNFK	172	180	9	HLA-A*02:01	172	36	VSEKSGNFK	VSEKSGNFK	0.00008	36	8.00E-05	36	TRUE	TRUE
1	ASVVNIQKE	1156	1164	9	HLA-A*02:01	1156	37	ASVVNIQKE	ASVVNIQKE	0.000079	37	7.90E-05	37	TRUE	TRUE
1	DVQAPNYTQ	24	32	9	HLA-A*02:01	24	37	DVQAPNYTQ	DVQAPNYTQ	0.000079	37	7.90E-05	37	TRUE	TRUE
1	FSPQIITTD	1092	1100	9	HLA-A*02:01	1092	37	FSPQIITTD	FSPQIITTD	0.000078	37	7.80E-05	37	TRUE	TRUE
1	TMIFDNAFN	150	158	9	HLA-A*02:01	150	37	TMIFDNAFN	TMIFDNAFN	0.000078	37	7.80E-05	37	TRUE	TRUE
1	VVLSFELLN	497	505	9	HLA-A*02:01	497	37	VVLSFELLN	VVLSFELLN	0.000078	37	7.80E-05	37	TRUE	TRUE
1	HGKLRPFER	445	453	9	HLA-A*02:01	445	37	HGKLRPFER	HGKLRPFER	0.000077	37	7.70E-05	37	TRUE	TRUE
1	ITDAVDCSQ	272	280	9	HLA-A*02:01	272	37	ITDAVDCSQ	ITDAVDCSQ	0.000075	37	7.50E-05	37	TRUE	TRUE
1	TYVTQQLIR	988	996	9	HLA-A*02:01	988	37	TYVTQQLIR	TYVTQQLIR	0.000075	37	7.50E-05	37	TRUE	TRUE
1	HTSPDVDLG	1141	1149	9	HLA-A*02:01	1141	37	HTSPDVDLG	HTSPDVDLG	0.000074	37	7.40E-05	37	TRUE	TRUE
1	LSTDLIKNQ	515	523	9	HLA-A*02:01	515	37	LSTDLIKNQ	LSTDLIKNQ	0.000074	37	7.40E-05	37	TRUE	TRUE
1	WFITQRNFF	1084	1092	9	HLA-A*02:01	1084	37	WFITQRNFF	WFITQRNFF	0.000074	37	7.40E-05	37	TRUE	TRUE
1	SECVLGQSK	1012	1020	9	HLA-A*02:01	1012	38	SECVLGQSK	SECVLGQSK	0.000073	38	7.30E-05	38	TRUE	TRUE
1	WVFGSTMNN	101	109	9	HLA-A*02:01	101	38	WVFGSTMNN	WVFGSTMNN	0.000073	38	7.30E-05	38	TRUE	TRUE
1	DINARDLIC	825	833	9	HLA-A*02:01	825	38	DINARDLIC	DINARDLIC	0.000072	38	7.20E-05	38	TRUE	TRUE
1	DVVRFPNIT	312	320	9	HLA-A*02:01	312	38	DVVRFPNIT	DVVRFPNIT	0.000072	38	7.20E-05	38	TRUE	TRUE
1	LRHGKLRPF	443	451	9	HLA-A*02:01	443	38	LRHGKLRPF	LRHGKLRPF	0.000072	38	7.20E-05	38	TRUE	TRUE
1	SFIEDLLFN	798	806	9	HLA-A*02:01	798	38	SFIEDLLFN	SFIEDLLFN	0.000072	38	7.20E-05	38	TRUE	TRUE
1	TQRNFFSPQ	1087	1095	9	HLA-A*02:01	1087	38	TQRNFFSPQ	TQRNFFSPQ	0.000072	38	7.20E-05	38	TRUE	TRUE
1	DFMGCVLAW	415	423	9	HLA-A*02:01	415	38	DFMGCVLAW	DFMGCVLAW	0.000071	38	7.10E-05	38	TRUE	TRUE
1	HTSSMRGVY	33	41	9	HLA-A*02:01	33	38	HTSSMRGVY	HTSSMRGVY	0.000071	38	7.10E-05	38	TRUE	TRUE
1	AKNLNESLI	1172	1180	9	HLA-A*02:01	1172	38	AKNLNESLI	AKNLNESLI	0.00007	38	7.00E-05	38	TRUE	TRUE
1	IVAYTMSLG	674	682	9	HLA-A*02:01	674	38	IVAYTMSLG	IVAYTMSLG	0.00007	38	7.00E-05	38	TRUE	TRUE
1	LGINITNFR	224	232	9	HLA-A*02:01	224	38	LGINITNFR	LGINITNFR	0.00007	38	7.00E-05	38	TRUE	TRUE
1	YRFNGIGVT	886	894	9	HLA-A*02:01	886	38	YRFNGIGVT	YRFNGIGVT	0.00007	38	7.00E-05	38	TRUE	TRUE
1	ECLGDINAR	821	829	9	HLA-A*02:01	821	39	ECLGDINAR	ECLGDINAR	0.000067	39	6.70E-05	39	TRUE	TRUE
1	EKSGNFKHL	174	182	9	HLA-A*02:01	174	39	EKSGNFKHL	EKSGNFKHL	0.000067	39	6.70E-05	39	TRUE	TRUE
1	NVPFSPDGK	457	465	9	HLA-A*02:01	457	39	NVPFSPDGK	NVPFSPDGK	0.000067	39	6.70E-05	39	TRUE	TRUE
1	WERKKISNC	340	348	9	HLA-A*02:01	340	39	WERKKISNC	WERKKISNC	0.000067	39	6.70E-05	39	TRUE	TRUE
1	TGTGVLTPS	533	541	9	HLA-A*02:01	533	39	TGTGVLTPS	TGTGVLTPS	0.000066	39	6.60E-05	39	TRUE	TRUE
1	VVKGDDVRQ	388	396	9	HLA-A*02:01	388	39	VVKGDDVRQ	VVKGDDVRQ	0.000066	39	6.60E-05	39	TRUE	TRUE
1	GKGYHLMSF	1026	1034	9	HLA-A*02:01	1026	39	GKGYHLMSF	GKGYHLMSF	0.000065	39	6.50E-05	39	TRUE	TRUE
1	PFERDISNV	450	458	9	HLA-A*02:01	450	39	PFERDISNV	PFERDISNV	0.000065	39	6.50E-05	39	TRUE	TRUE
1	TSEILDISP	567	575	9	HLA-A*02:01	567	39	TSEILDISP	TSEILDISP	0.000065	39	6.50E-05	39	TRUE	TRUE
1	VVIRACNFE	123	131	9	HLA-A*02:01	123	39	VVIRACNFE	VVIRACNFE	0.000065	39	6.50E-05	39	TRUE	TRUE
1	SSVLNDILS	956	964	9	HLA-A*02:01	956	39	SSVLNDILS	SSVLNDILS	0.000064	39	6.40E-05	39	TRUE	TRUE
1	STFKCYGVS	362	370	9	HLA-A*02:01	362	39	STFKCYGVS	STFKCYGVS	0.000064	39	6.40E-05	39	TRUE	TRUE
1	VSGTATAGW	860	868	9	HLA-A*02:01	860	39	VSGTATAGW	VSGTATAGW	0.000064	39	6.40E-05	39	TRUE	TRUE
1	LKPTTFMLK	257	265	9	HLA-A*02:01	257	39	LKPTTFMLK	LKPTTFMLK	0.000063	39	6.30E-05	39	TRUE	TRUE
1	PSQERNFTT	1051	1059	9	HLA-A*02:01	1051	39	PSQERNFTT	PSQERNFTT	0.000063	39	6.30E-05	39	TRUE	TRUE
1	PVLKGVKLH	1245	1253	9	HLA-A*02:01	1245	39	PVLKGVKLH	PVLKGVKLH	0.000063	39	6.30E-05	39	TRUE	TRUE
1	RDLICAQKF	829	837	9	HLA-A*02:01	829	39	RDLICAQKF	RDLICAQKF	0.000063	39	6.30E-05	39	TRUE	TRUE
1	CTDVSTAIH	603	611	9	HLA-A*02:01	603	40	CTDVSTAIH	CTDVSTAIH	0.000062	40	6.20E-05	40	TRUE	TRUE
1	DGKPCTPPA	463	471	9	HLA-A*02:01	463	40	DGKPCTPPA	DGKPCTPPA	0.000062	40	6.20E-05	40	TRUE	TRUE
1	SPDVDLGDI	1143	1151	9	HLA-A*02:01	1143	40	SPDVDLGDI	SPDVDLGDI	0.000062	40	6.20E-05	40	TRUE	TRUE
1	FYSNVTGFH	62	70	9	HLA-A*02:01	62	40	FYSNVTGFH	FYSNVTGFH	0.000061	40	6.10E-05	40	TRUE	TRUE
1	SMRGVYYPD	36	44	9	HLA-A*02:01	36	40	SMRGVYYPD	SMRGVYYPD	0.000061	40	6.10E-05	40	TRUE	TRUE
1	IYFAATEKS	87	95	9	HLA-A*02:01	87	40	IYFAATEKS	IYFAATEKS	0.00006	40	6.00E-05	40	TRUE	TRUE
1	KSIVAYTMS	672	680	9	HLA-A*02:01	672	40	KSIVAYTMS	KSIVAYTMS	0.00006	40	6.00E-05	40	TRUE	TRUE
1	NAPATVCGP	505	513	9	HLA-A*02:01	505	40	NAPATVCGP	NAPATVCGP	0.000059	40	5.90E-05	40	TRUE	TRUE
1	GFMKQYGEC	814	822	9	HLA-A*02:01	814	41	GFMKQYGEC	GFMKQYGEC	0.000057	41	5.70E-05	41	TRUE	TRUE
1	ISQIQESLT	913	921	9	HLA-A*02:01	913	41	ISQIQESLT	ISQIQESLT	0.000057	41	5.70E-05	41	TRUE	TRUE
1	NHTFGNPVI	73	81	9	HLA-A*02:01	73	41	NHTFGNPVI	NHTFGNPVI	0.000057	41	5.70E-05	41	TRUE	TRUE
1	SFPQAAPHG	1033	1041	9	HLA-A*02:01	1033	41	SFPQAAPHG	SFPQAAPHG	0.000057	41	5.70E-05	41	TRUE	TRUE
1	VVRFPNITN	313	321	9	HLA-A*02:01	313	41	VVRFPNITN	VVRFPNITN	0.000057	41	5.70E-05	41	TRUE	TRUE
1	AGFMKQYGE	813	821	9	HLA-A*02:01	813	41	AGFMKQYGE	AGFMKQYGE	0.000056	41	5.60E-05	41	TRUE	TRUE
1	CYGVSATKL	366	374	9	HLA-A*02:01	366	41	CYGVSATKL	CYGVSATKL	0.000056	41	5.60E-05	41	TRUE	TRUE
1	NVLYENQKQ	896	904	9	HLA-A*02:01	896	41	NVLYENQKQ	NVLYENQKQ	0.000056	41	5.60E-05	41	TRUE	TRUE
1	ALNCYWPLN	471	479	9	HLA-A*02:01	471	41	ALNCYWPLN	ALNCYWPLN	0.000055	41	5.50E-05	41	TRUE	TRUE
1	ATVCGPKLS	508	516	9	HLA-A*02:01	508	41	ATVCGPKLS	ATVCGPKLS	0.000055	41	5.50E-05	41	TRUE	TRUE
1	FNTLKPIFK	213	221	9	HLA-A*02:01	213	41	FNTLKPIFK	FNTLKPIFK	0.000055	41	5.50E-05	41	TRUE	TRUE
1	LPPLLTDDM	843	851	9	HLA-A*02:01	843	41	LPPLLTDDM	LPPLLTDDM	0.000055	41	5.50E-05	41	TRUE	TRUE
1	TGNNVFQTQ	624	632	9	HLA-A*02:01	624	41	TGNNVFQTQ	TGNNVFQTQ	0.000055	41	5.50E-05	41	TRUE	TRUE
1	YQPIDVVRD	200	208	9	HLA-A*02:01	200	41	YQPIDVVRD	YQPIDVVRD	0.000054	41	5.40E-05	41	TRUE	TRUE
1	FNKVTLADA	805	813	9	HLA-A*02:01	805	42	FNKVTLADA	FNKVTLADA	0.000053	42	5.30E-05	42	TRUE	TRUE
1	LKPTKRSFI	792	800	9	HLA-A*02:01	792	42	LKPTKRSFI	LKPTKRSFI	0.000053	42	5.30E-05	42	TRUE	TRUE
1	NVVIRACNF	122	130	9	HLA-A*02:01	122	42	NVVIRACNF	NVVIRACNF	0.000053	42	5.30E-05	42	TRUE	TRUE
1	SNNTIAIPT	690	698	9	HLA-A*02:01	690	42	SNNTIAIPT	SNNTIAIPT	0.000053	42	5.30E-05	42	TRUE	TRUE
1	GAGAALQIP	871	879	9	HLA-A*02:01	871	42	GAGAALQIP	GAGAALQIP	0.000051	42	5.10E-05	42	TRUE	TRUE
1	QSVIIINNS	112	120	9	HLA-A*02:01	112	42	QSVIIINNS	QSVIIINNS	0.000051	42	5.10E-05	42	TRUE	TRUE
1	TPPALNCYW	468	476	9	HLA-A*02:01	468	42	TPPALNCYW	TPPALNCYW	0.000051	42	5.10E-05	42	TRUE	TRUE
1	DDFMGCVLA	414	422	9	HLA-A*02:01	414	42	DDFMGCVLA	DDFMGCVLA	0.00005	42	5.00E-05	42	TRUE	TRUE
1	DAFSLDVSE	166	174	9	HLA-A*02:01	166	43	DAFSLDVSE	DAFSLDVSE	0.000049	43	4.90E-05	43	TRUE	TRUE
1	SNVVRGWVF	95	103	9	HLA-A*02:01	95	43	SNVVRGWVF	SNVVRGWVF	0.000049	43	4.90E-05	43	TRUE	TRUE
1	NNKSQSVII	108	116	9	HLA-A*02:01	108	43	NNKSQSVII	NNKSQSVII	0.000048	43	4.80E-05	43	TRUE	TRUE
1	NSTFFSTFK	357	365	9	HLA-A*02:01	357	43	NSTFFSTFK	NSTFFSTFK	0.000048	43	4.80E-05	43	TRUE	TRUE
1	RYLRHGKLR	441	449	9	HLA-A*02:01	441	43	RYLRHGKLR	RYLRHGKLR	0.000048	43	4.80E-05	43	TRUE	TRUE
1	VYAWERKKI	337	345	9	HLA-A*02:01	337	43	VYAWERKKI	VYAWERKKI	0.000048	43	4.80E-05	43	TRUE	TRUE
1	TGRLQSLQT	980	988	9	HLA-A*02:01	980	43	TGRLQSLQT	TGRLQSLQT	0.000047	43	4.70E-05	43	TRUE	TRUE
1	VSLLRSTSQ	663	671	9	HLA-A*02:01	663	43	VSLLRSTSQ	VSLLRSTSQ	0.000047	43	4.70E-05	43	TRUE	TRUE
1	APNYTQHTS	27	35	9	HLA-A*02:01	27	44	APNYTQHTS	APNYTQHTS	0.000046	44	4.60E-05	44	TRUE	TRUE
1	FNGLTVLPP	837	845	9	HLA-A*02:01	837	44	FNGLTVLPP	FNGLTVLPP	0.000046	44	4.60E-05	44	TRUE	TRUE

seq #	peptide	start	end	peptide length	allele	peptide index	median binding	netmhcpan_el	netmhcpan_el	netmhcpan_el	netmhcpan_el	legacy score	legacy rank	scores equal	percentiles equal
1	NPFFAVSKP	135	143	9	HLA-A*02:01	135	44	NPFFAVSKP	NPFFAVSKP	0.000046	44	4.60E-05	44	TRUE	TRUE
1	PATVCGPKL	507	515	9	HLA-A*02:01	507	44	PATVCGPKL	PATVCGPKL	0.000046	44	4.60E-05	44	TRUE	TRUE
1	ICASYHTVS	656	664	9	HLA-A*02:01	656	44	ICASYHTVS	ICASYHTVS	0.000045	44	4.50E-05	44	TRUE	TRUE
1	KWPWYVWLG	1193	1201	9	HLA-A*02:01	1193	44	KWPWYVWLG	KWPWYVWLG	0.000045	44	4.50E-05	44	TRUE	TRUE
1	TLTSGSDLD	9	17	9	HLA-A*02:01	9	44	TLTSGSDLD	TLTSGSDLD	0.000045	44	4.50E-05	44	TRUE	TRUE
1	TQNVLYENQ	894	902	9	HLA-A*02:01	894	44	TQNVLYENQ	TQNVLYENQ	0.000045	44	4.50E-05	44	TRUE	TRUE
1	FMGCVLAWN	416	424	9	HLA-A*02:01	416	44	FMGCVLAWN	FMGCVLAWN	0.000044	44	4.40E-05	44	TRUE	TRUE
1	LAELKCSVK	283	291	9	HLA-A*02:01	283	44	LAELKCSVK	LAELKCSVK	0.000044	44	4.40E-05	44	TRUE	TRUE
1	LVSGTATAG	859	867	9	HLA-A*02:01	859	44	LVSGTATAG	LVSGTATAG	0.000044	44	4.40E-05	44	TRUE	TRUE
1	SEKSGNFKH	173	181	9	HLA-A*02:01	173	44	SEKSGNFKH	SEKSGNFKH	0.000044	44	4.40E-05	44	TRUE	TRUE
1	CCMTSCCSC	1217	1225	9	HLA-A*02:01	1217	44	CCMTSCCSC	CCMTSCCSC	0.000043	44	4.30E-05	44	TRUE	TRUE
1	GDISGINAS	1149	1157	9	HLA-A*02:01	1149	44	GDISGINAS	GDISGINAS	0.000043	44	4.30E-05	44	TRUE	TRUE
1	SKPMGTQTH	141	149	9	HLA-A*02:01	141	44	SKPMGTQTH	SKPMGTQTH	0.000043	44	4.30E-05	44	TRUE	TRUE
1	TAPAICHEG	1059	1067	9	HLA-A*02:01	1059	44	TAPAICHEG	TAPAICHEG	0.000043	44	4.30E-05	44	TRUE	TRUE
1	EVQIDRLIT	972	980	9	HLA-A*02:01	972	45	EVQIDRLIT	EVQIDRLIT	0.000042	45	4.20E-05	45	TRUE	TRUE
1	HTINHTFGN	70	78	9	HLA-A*02:01	70	45	HTINHTFGN	HTINHTFGN	0.000042	45	4.20E-05	45	TRUE	TRUE
1	TSGSDLDRC	11	19	9	HLA-A*02:01	11	45	TSGSDLDRC	TSGSDLDRC	0.000042	45	4.20E-05	45	TRUE	TRUE
1	WPWYVWLGF	1194	1202	9	HLA-A*02:01	1194	45	WPWYVWLGF	WPWYVWLGF	0.000042	45	4.20E-05	45	TRUE	TRUE
1	YPDEIFRSD	42	50	9	HLA-A*02:01	42	45	YPDEIFRSD	YPDEIFRSD	0.000042	45	4.20E-05	45	TRUE	TRUE
1	DVNCTDVST	600	608	9	HLA-A*02:01	600	45	DVNCTDVST	DVNCTDVST	0.000041	45	4.10E-05	45	TRUE	TRUE
1	FEYISDAFS	161	169	9	HLA-A*02:01	161	45	FEYISDAFS	FEYISDAFS	0.000041	45	4.10E-05	45	TRUE	TRUE
1	KNQCVNFNF	521	529	9	HLA-A*02:01	521	45	KNQCVNFNF	KNQCVNFNF	0.000041	45	4.10E-05	45	TRUE	TRUE
1	QNPLAELKC	280	288	9	HLA-A*02:01	280	45	QNPLAELKC	QNPLAELKC	0.000041	45	4.10E-05	45	TRUE	TRUE
1	AWNTRNIDA	422	430	9	HLA-A*02:01	422	45	AWNTRNIDA	AWNTRNIDA	0.00004	45	4.00E-05	45	TRUE	TRUE
1	PFFAVSKPM	136	144	9	HLA-A*02:01	136	45	PFFAVSKPM	PFFAVSKPM	0.00004	45	4.00E-05	45	TRUE	TRUE
1	VADYSVLYN	349	357	9	HLA-A*02:01	349	45	VADYSVLYN	VADYSVLYN	0.00004	45	4.00E-05	45	TRUE	TRUE
1	AGCLIGAEH	633	641	9	HLA-A*02:01	633	46	AGCLIGAEH	AGCLIGAEH	0.000039	46	3.90E-05	46	TRUE	TRUE
1	IDLQELGKY	1180	1188	9	HLA-A*02:01	1180	46	IDLQELGKY	IDLQELGKY	0.000039	46	3.90E-05	46	TRUE	TRUE
1	ITNLCPFGE	319	327	9	HLA-A*02:01	319	46	ITNLCPFGE	ITNLCPFGE	0.000039	46	3.90E-05	46	TRUE	TRUE
1	LYVYKGYQP	194	202	9	HLA-A*02:01	194	46	LYVYKGYQP	LYVYKGYQP	0.000039	46	3.90E-05	46	TRUE	TRUE
1	NTRNIDATS	424	432	9	HLA-A*02:01	424	46	NTRNIDATS	NTRNIDATS	0.000039	46	3.90E-05	46	TRUE	TRUE
1	TQHTSSMRG	31	39	9	HLA-A*02:01	31	46	TQHTSSMRG	TQHTSSMRG	0.000039	46	3.90E-05	46	TRUE	TRUE
1	IRACNFELC	125	133	9	HLA-A*02:01	125	46	IRACNFELC	IRACNFELC	0.000038	46	3.80E-05	46	TRUE	TRUE
1	PCTPPALNC	466	474	9	HLA-A*02:01	466	46	PCTPPALNC	PCTPPALNC	0.000038	46	3.80E-05	46	TRUE	TRUE
1	TTGIGYQPY	486	494	9	HLA-A*02:01	486	46	TTGIGYQPY	TTGIGYQPY	0.000038	46	3.80E-05	46	TRUE	TRUE
1	GFLYVYKGY	192	200	9	HLA-A*02:01	192	47	GFLYVYKGY	GFLYVYKGY	0.000037	47	3.70E-05	47	TRUE	TRUE
1	IWGTSAAAY	244	252	9	HLA-A*02:01	244	47	IWGTSAAAY	IWGTSAAAY	0.000037	47	3.70E-05	47	TRUE	TRUE
1	KGACSCGSC	1227	1235	9	HLA-A*02:01	1227	47	KGACSCGSC	KGACSCGSC	0.000037	47	3.70E-05	47	TRUE	TRUE
1	NNSTNVVIR	118	126	9	HLA-A*02:01	118	47	NNSTNVVIR	NNSTNVVIR	0.000037	47	3.70E-05	47	TRUE	TRUE
1	TPSSKRFQP	539	547	9	HLA-A*02:01	539	47	TPSSKRFQP	TPSSKRFQP	0.000037	47	3.70E-05	47	TRUE	TRUE
1	DAVDCSQNP	274	282	9	HLA-A*02:01	274	47	DAVDCSQNP	DAVDCSQNP	0.000036	47	3.60E-05	47	TRUE	TRUE
1	GCVLAWNTR	418	426	9	HLA-A*02:01	418	47	GCVLAWNTR	GCVLAWNTR	0.000036	47	3.60E-05	47	TRUE	TRUE
1	NRALSGIAA	746	754	9	HLA-A*02:01	746	47	NRALSGIAA	NRALSGIAA	0.000036	47	3.60E-05	47	TRUE	TRUE
1	DVVRDLPSG	204	212	9	HLA-A*02:01	204	47	DVVRDLPSG	DVVRDLPSG	0.000035	47	3.50E-05	47	TRUE	TRUE
1	TFDDVQAPN	21	29	9	HLA-A*02:01	21	47	TFDDVQAPN	TFDDVQAPN	0.000035	47	3.50E-05	47	TRUE	TRUE
1	FRAILTAFS	231	239	9	HLA-A*02:01	231	48	FRAILTAFS	FRAILTAFS	0.000034	48	3.40E-05	48	TRUE	TRUE
1	WGTSAAAYF	245	253	9	HLA-A*02:01	245	48	WGTSAAAYF	WGTSAAAYF	0.000034	48	3.40E-05	48	TRUE	TRUE
1	DRNTREVFA	757	765	9	HLA-A*02:01	757	48	DRNTREVFA	DRNTREVFA	0.000033	48	3.30E-05	48	TRUE	TRUE
1	LFLTLTSGS	6	14	9	HLA-A*02:01	6	48	LFLTLTSGS	LFLTLTSGS	0.000033	48	3.30E-05	48	TRUE	TRUE
1	VTQNVLYEN	893	901	9	HLA-A*02:01	893	48	VTQNVLYEN	VTQNVLYEN	0.000033	48	3.30E-05	48	TRUE	TRUE
1	CTQLNRALS	742	750	9	HLA-A*02:01	742	49	CTQLNRALS	CTQLNRALS	0.000032	49	3.20E-05	49	TRUE	TRUE
1	KTPTLKYFG	772	780	9	HLA-A*02:01	772	49	KTPTLKYFG	KTPTLKYFG	0.000032	49	3.20E-05	49	TRUE	TRUE
1	VPFSPDGKP	458	466	9	HLA-A*02:01	458	49	VPFSPDGKP	VPFSPDGKP	0.000032	49	3.20E-05	49	TRUE	TRUE
1	VPSQERNFT	1050	1058	9	HLA-A*02:01	1050	49	VPSQERNFT	VPSQERNFT	0.000032	49	3.20E-05	49	TRUE	TRUE
1	YHTVSLLRS	660	668	9	HLA-A*02:01	660	49	YHTVSLLRS	YHTVSLLRS	0.000032	49	3.20E-05	49	TRUE	TRUE
1	AKTSVDCNM	714	722	9	HLA-A*02:01	714	49	AKTSVDCNM	AKTSVDCNM	0.000031	49	3.10E-05	49	TRUE	TRUE
1	DSFKEELDK	1128	1136	9	HLA-A*02:01	1128	49	DSFKEELDK	DSFKEELDK	0.000031	49	3.10E-05	49	TRUE	TRUE
1	LQELGKYEQ	1182	1190	9	HLA-A*02:01	1182	49	LQELGKYEQ	LQELGKYEQ	0.000031	49	3.10E-05	49	TRUE	TRUE
1	NFTTAPAIC	1056	1064	9	HLA-A*02:01	1056	49	NFTTAPAIC	NFTTAPAIC	0.000031	49	3.10E-05	49	TRUE	TRUE
1	PTLKYFGGF	774	782	9	HLA-A*02:01	774	49	PTLKYFGGF	PTLKYFGGF	0.000031	49	3.10E-05	49	TRUE	TRUE
1	QESLTTTST	917	925	9	HLA-A*02:01	917	49	QESLTTTST	QESLTTTST	0.000031	49	3.10E-05	49	TRUE	TRUE
1	DVDLGDISG	1145	1153	9	HLA-A*02:01	1145	49	DVDLGDISG	DVDLGDISG	0.00003	49	3.00E-05	49	TRUE	TRUE
1	NFSISITTE	699	707	9	HLA-A*02:01	699	49	NFSISITTE	NFSISITTE	0.00003	49	3.00E-05	49	TRUE	TRUE
1	QERNFTTAP	1053	1061	9	HLA-A*02:01	1053	49	QERNFTTAP	QERNFTTAP	0.00003	49	3.00E-05	49	TRUE	TRUE
1	SGSDLDRCT	12	20	9	HLA-A*02:01	12	49	SGSDLDRCT	SGSDLDRCT	0.00003	49	3.00E-05	49	TRUE	TRUE
1	THTMIFDNA	148	156	9	HLA-A*02:01	148	49	THTMIFDNA	THTMIFDNA	0.00003	49	3.00E-05	49	TRUE	TRUE
1	NGTSWFITQ	1080	1088	9	HLA-A*02:01	1080	50	NGTSWFITQ	NGTSWFITQ	0.000029	50	2.90E-05	50	TRUE	TRUE
1	PSGDVVRFP	309	317	9	HLA-A*02:01	309	50	PSGDVVRFP	PSGDVVRFP	0.000029	50	2.90E-05	50	TRUE	TRUE
1	TDDMIAAYT	848	856	9	HLA-A*02:01	848	50	TDDMIAAYT	TDDMIAAYT	0.000029	50	2.90E-05	50	TRUE	TRUE
1	EDDSEPVLK	1240	1248	9	HLA-A*02:01	1240	50	EDDSEPVLK	EDDSEPVLK	0.000028	50	2.80E-05	50	TRUE	TRUE
1	FFAVSKPMG	137	145	9	HLA-A*02:01	137	50	FFAVSKPMG	FFAVSKPMG	0.000028	50	2.80E-05	50	TRUE	TRUE
1	LGKYEQYIK	1185	1193	9	HLA-A*02:01	1185	50	LGKYEQYIK	LGKYEQYIK	0.000028	50	2.80E-05	50	TRUE	TRUE
1	QFGRDVSDF	550	558	9	HLA-A*02:01	550	50	QFGRDVSDF	QFGRDVSDF	0.000028	50	2.80E-05	50	TRUE	TRUE
1	REVFAQVKQ	761	769	9	HLA-A*02:01	761	50	REVFAQVKQ	REVFAQVKQ	0.000028	50	2.80E-05	50	TRUE	TRUE
1	VDCSQNPLA	276	284	9	HLA-A*02:01	276	50	VDCSQNPLA	VDCSQNPLA	0.000028	50	2.80E-05	50	TRUE	TRUE
1	CAQKFNGLT	833	841	9	HLA-A*02:01	833	51	CAQKFNGLT	CAQKFNGLT	0.000027	51	2.70E-05	51	TRUE	TRUE
1	CSCGSCCKF	1230	1238	9	HLA-A*02:01	1230	51	CSCGSCCKF	CSCGSCCKF	0.000027	51	2.70E-05	51	TRUE	TRUE
1	DNPFFAVSK	134	142	9	HLA-A*02:01	134	51	DNPFFAVSK	DNPFFAVSK	0.000027	51	2.70E-05	51	TRUE	TRUE
1	EKSNVVRGW	93	101	9	HLA-A*02:01	93	51	EKSNVVRGW	EKSNVVRGW	0.000027	51	2.70E-05	51	TRUE	TRUE
1	KSQSVIIIN	110	118	9	HLA-A*02:01	110	51	KSQSVIIIN	KSQSVIIIN	0.000027	51	2.70E-05	51	TRUE	TRUE
1	LGQSKRVDF	1016	1024	9	HLA-A*02:01	1016	51	LGQSKRVDF	LGQSKRVDF	0.000027	51	2.70E-05	51	TRUE	TRUE
1	PIDVVRDLP	202	210	9	HLA-A*02:01	202	51	PIDVVRDLP	PIDVVRDLP	0.000027	51	2.70E-05	51	TRUE	TRUE
1	SSKRFQPFQ	541	549	9	HLA-A*02:01	541	51	SSKRFQPFQ	SSKRFQPFQ	0.000027	51	2.70E-05	51	TRUE	TRUE
1	STALGKLQD	924	932	9	HLA-A*02:01	924	51	STALGKLQD	STALGKLQD	0.000027	51	2.70E-05	51	TRUE	TRUE
1	TQTHTMIFD	146	154	9	HLA-A*02:01	146	51	TQTHTMIFD	TQTHTMIFD	0.000027	51	2.70E-05	51	TRUE	TRUE
1	KPIFKLPLG	217	225	9	HLA-A*02:01	217	51	KPIFKLPLG	KPIFKLPLG	0.000026	51	2.60E-05	51	TRUE	TRUE
1	LCFSNVYAD	377	385	9	HLA-A*02:01	377	51	LCFSNVYAD	LCFSNVYAD	0.000026	51	2.60E-05	51	TRUE	TRUE
1	LSSNFGAIS	948	956	9	HLA-A*02:01	948	51	LSSNFGAIS	LSSNFGAIS	0.000026	51	2.60E-05	51	TRUE	TRUE
1	SGNFKHLRE	176	184	9	HLA-A*02:01	176	51	SGNFKHLRE	SGNFKHLRE	0.000026	51	2.60E-05	51	TRUE	TRUE
1	ECVLGQSKR	1013	1021	9	HLA-A*02:01	1013	52	ECVLGQSKR	ECVLGQSKR	0.000025	52	2.50E-05	52	TRUE	TRUE
1	FRVVPSGDV	305	313	9	HLA-A*02:01	305	52	FRVVPSGDV	FRVVPSGDV	0.000025	52	2.50E-05	52	TRUE	TRUE
1	VNFNFNGLT	525	533	9	HLA-A*02:01	525	52	VNFNFNGLT	VNFNFNGLT	0.000025	52	2.50E-05	52	TRUE	TRUE
1	EGVFVFNGT	1074	1082	9	HLA-A*02:01	1074	53	EGVFVFNGT	EGVFVFNGT	0.000024	53	2.40E-05	53	TRUE	TRUE
1	FKCYGVSAT	364	372	9	HLA-A*02:01	364	53	FKCYGVSAT	FKCYGVSAT	0.000024	53	2.40E-05	53	TRUE	TRUE
1	MGCVLAWNT	417	425	9	HLA-A*02:01	417	53	MGCVLAWNT	MGCVLAWNT	0.000024	53	2.40E-05	53	TRUE	TRUE
1	PFGEVFNAT	324	332	9	HLA-A*02:01	324	53	PFGEVFNAT	PFGEVFNAT	0.000024	53	2.40E-05	53	TRUE	TRUE
1	SFEIDKGIY	292	300	9	HLA-A*02:01	292	53	SFEIDKGIY	SFEIDKGIY	0.000024	53	2.40E-05	53	TRUE	TRUE
1	SNFRVVPSG	303	311	9	HLA-A*02:01	303	53	SNFRVVPSG	SNFRVVPSG	0.000024	53	2.40E-05	53	TRUE	TRUE
1	WYVWLGFIA	1196	1204	9	HLA-A*02:01	1196	53	WYVWLGFIA	WYVWLGFIA	0.000024	53	2.40E-05	53	TRUE	TRUE
1	FGSTMNNKS	103	111	9	HLA-A*02:01	103	53	FGSTMNNKS	FGSTMNNKS	0.000023	53	2.30E-05	53	TRUE	TRUE
1	GECLGDINA	820	828	9	HLA-A*02:01	820	53	GECLGDINA	GECLGDINA	0.000023	53	2.30E-05	53	TRUE	TRUE
1	GWTFGAGAA	867	875	9	HLA-A*02:01	867	53	GWTFGAGAA	GWTFGAGAA	0.000023	53	2.30E-05	53	TRUE	TRUE
1	IDRLNEVAK	1165	1173	9	HLA-A*02:01	1165	53	IDRLNEVAK	IDRLNEVAK	0.000023	53	2.30E-05	53	TRUE	TRUE
1	PHGVVFLHV	1039	1047	9	HLA-A*02:01	1039	53	PHGVVFLHV	PHGVVFLHV	0.000023	53	2.30E-05	53	TRUE	TRUE
1	RPFERDISN	449	457	9	HLA-A*02:01	449	53	RPFERDISN	RPFERDISN	0.000023	53	2.30E-05	53	TRUE	TRUE
1	SGIAAEQDR	750	758	9	HLA-A*02:01	750	53	SGIAAEQDR	SGIAAEQDR	0.000023	53	2.30E-05	53	TRUE	TRUE
1	VNIQKEIDR	1159	1167	9	HLA-A*02:01	1159	53	VNIQKEIDR	VNIQKEIDR	0.000023	53	2.30E-05	53	TRUE	TRUE
1	ITGRLQSLQ	979	987	9	HLA-A*02:01	979	54	ITGRLQSLQ	ITGRLQSLQ	0.000022	54	2.20E-05	54	TRUE	TRUE
1	LKGVKLHYT	1247	1255	9	HLA-A*02:01	1247	54	LKGVKLHYT	LKGVKLHYT	0.000022	54	2.20E-05	54	TRUE	TRUE
1	LTTTSTALG	920	928	9	HLA-A*02:01	920	54	LTTTSTALG	LTTTSTALG	0.000022	54	2.20E-05	54	TRUE	TRUE
1	YRVVVLSFE	494	502	9	HLA-A*02:01	494	54	YRVVVLSFE	YRVVVLSFE	0.000022	54	2.20E-05	54	TRUE	TRUE
1	DVRQIAPGQ	393	401	9	HLA-A*02:01	393	55	DVRQIAPGQ	DVRQIAPGQ	0.000021	55	2.10E-05	55	TRUE	TRUE
1	GVSATKLND	368	376	9	HLA-A*02:01	368	55	GVSATKLND	GVSATKLND	0.000021	55	2.10E-05	55	TRUE	TRUE
1	IDATSTGNY	428	436	9	HLA-A*02:01	428	55	IDATSTGNY	IDATSTGNY	0.000021	55	2.10E-05	55	TRUE	TRUE
1	NVVRGWVFG	96	104	9	HLA-A*02:01	96	55	NVVRGWVFG	NVVRGWVFG	0.000021	55	2.10E-05	55	TRUE	TRUE

seq #	peptide	start	end	peptide length	allele	peptide index	median binding	netmhcpan_el	netmhcpan_el	netmhcpan_el	netmhcpan_el	legacy score	legacy rank	scores equal	percentiles equal
1	SSEVAVLYQ	591	599	9	HLA-A*02:01	591	55	SSEVAVLYQ	SSEVAVLYQ	0.000021	55	2.10E-05	55	TRUE	TRUE
1	TGNYNYKYR	433	441	9	HLA-A*02:01	433	55	TGNYNYKYR	TGNYNYKYR	0.000021	55	2.10E-05	55	TRUE	TRUE
1	VFNATKFPS	328	336	9	HLA-A*02:01	328	55	VFNATKFPS	VFNATKFPS	0.000021	55	2.10E-05	55	TRUE	TRUE
1	DIPIGAGIC	649	657	9	HLA-A*02:01	649	55	DIPIGAGIC	DIPIGAGIC	0.00002	55	2.00E-05	55	TRUE	TRUE
1	RNIDATSTG	426	434	9	HLA-A*02:01	426	55	RNIDATSTG	RNIDATSTG	0.00002	55	2.00E-05	55	TRUE	TRUE
1	TSPDVDLGD	1142	1150	9	HLA-A*02:01	1142	55	TSPDVDLGD	TSPDVDLGD	0.00002	55	2.00E-05	55	TRUE	TRUE
1	AELKCSVKS	284	292	9	HLA-A*02:01	284	56	AELKCSVKS	AELKCSVKS	0.000019	56	1.90E-05	56	TRUE	TRUE
1	DVSTAIHAD	605	613	9	HLA-A*02:01	605	56	DVSTAIHAD	DVSTAIHAD	0.000019	56	1.90E-05	56	TRUE	TRUE
1	ERNFTTAPA	1054	1062	9	HLA-A*02:01	1054	56	ERNFTTAPA	ERNFTTAPA	0.000019	56	1.90E-05	56	TRUE	TRUE
1	FPNITNLCP	316	324	9	HLA-A*02:01	316	56	FPNITNLCP	FPNITNLCP	0.000019	56	1.90E-05	56	TRUE	TRUE
1	FSPAQDIWG	238	246	9	HLA-A*02:01	238	56	FSPAQDIWG	FSPAQDIWG	0.000019	56	1.90E-05	56	TRUE	TRUE
1	GAISSVLND	953	961	9	HLA-A*02:01	953	56	GAISSVLND	GAISSVLND	0.000019	56	1.90E-05	56	TRUE	TRUE
1	GDDVRQIAP	391	399	9	HLA-A*02:01	391	56	GDDVRQIAP	GDDVRQIAP	0.000019	56	1.90E-05	56	TRUE	TRUE
1	IDKGIYQTS	295	303	9	HLA-A*02:01	295	56	IDKGIYQTS	IDKGIYQTS	0.000019	56	1.90E-05	56	TRUE	TRUE
1	KDGFLYVYK	190	198	9	HLA-A*02:01	190	56	KDGFLYVYK	KDGFLYVYK	0.000019	56	1.90E-05	56	TRUE	TRUE
1	KFPSVYAWE	333	341	9	HLA-A*02:01	333	56	KFPSVYAWE	KFPSVYAWE	0.000019	56	1.90E-05	56	TRUE	TRUE
1	LTAFSPAQD	235	243	9	HLA-A*02:01	235	56	LTAFSPAQD	LTAFSPAQD	0.000019	56	1.90E-05	56	TRUE	TRUE
1	LYENQKQIA	898	906	9	HLA-A*02:01	898	56	LYENQKQIA	LYENQKQIA	0.000019	56	1.90E-05	56	TRUE	TRUE
1	NIDATSTGN	427	435	9	HLA-A*02:01	427	56	NIDATSTGN	NIDATSTGN	0.000019	56	1.90E-05	56	TRUE	TRUE
1	PWYVWLGFI	1195	1203	9	HLA-A*02:01	1195	56	PWYVWLGFI	PWYVWLGFI	0.000019	56	1.90E-05	56	TRUE	TRUE
1	PYRVVVLSF	493	501	9	HLA-A*02:01	493	56	PYRVVVLSF	PYRVVVLSF	0.000019	56	1.90E-05	56	TRUE	TRUE
1	TGVIADYNY	402	410	9	HLA-A*02:01	402	56	TGVIADYNY	TGVIADYNY	0.000019	56	1.90E-05	56	TRUE	TRUE
1	TREVFAQVK	760	768	9	HLA-A*02:01	760	56	TREVFAQVK	TREVFAQVK	0.000019	56	1.90E-05	56	TRUE	TRUE
1	TTFMLKYDE	260	268	9	HLA-A*02:01	260	56	TTFMLKYDE	TTFMLKYDE	0.000019	56	1.90E-05	56	TRUE	TRUE
1	ANLLLQYGS	732	740	9	HLA-A*02:01	732	57	ANLLLQYGS	ANLLLQYGS	0.000018	57	1.80E-05	57	TRUE	TRUE
1	HEGKAYFPR	1065	1073	9	HLA-A*02:01	1065	57	HEGKAYFPR	HEGKAYFPR	0.000018	57	1.80E-05	57	TRUE	TRUE
1	LTPSSKRFQ	538	546	9	HLA-A*02:01	538	57	LTPSSKRFQ	LTPSSKRFQ	0.000018	57	1.80E-05	57	TRUE	TRUE
1	TSWFITQRN	1082	1090	9	HLA-A*02:01	1082	57	TSWFITQRN	TSWFITQRN	0.000018	57	1.80E-05	57	TRUE	TRUE
1	ADQLTPAWR	612	620	9	HLA-A*02:01	612	58	ADQLTPAWR	ADQLTPAWR	0.000017	58	1.70E-05	58	TRUE	TRUE
1	DGFLYVYKG	191	199	9	HLA-A*02:01	191	58	DGFLYVYKG	DGFLYVYKG	0.000017	58	1.70E-05	58	TRUE	TRUE
1	FNFSQILPD	782	790	9	HLA-A*02:01	782	58	FNFSQILPD	FNFSQILPD	0.000017	58	1.70E-05	58	TRUE	TRUE
1	LHVTYVPSQ	1045	1053	9	HLA-A*02:01	1045	58	LHVTYVPSQ	LHVTYVPSQ	0.000017	58	1.70E-05	58	TRUE	TRUE
1	MSECVLGQS	1011	1019	9	HLA-A*02:01	1011	58	MSECVLGQS	MSECVLGQS	0.000017	58	1.70E-05	58	TRUE	TRUE
1	NITNLCPFG	318	326	9	HLA-A*02:01	318	58	NITNLCPFG	NITNLCPFG	0.000017	58	1.70E-05	58	TRUE	TRUE
1	QDRNTREVF	756	764	9	HLA-A*02:01	756	58	QDRNTREVF	QDRNTREVF	0.000017	58	1.70E-05	58	TRUE	TRUE
1	TLKYFGGFN	775	783	9	HLA-A*02:01	775	58	TLKYFGGFN	TLKYFGGFN	0.000017	58	1.70E-05	58	TRUE	TRUE
1	AMQMAYRFN	881	889	9	HLA-A*02:01	881	59	AMQMAYRFN	AMQMAYRFN	0.000016	59	1.60E-05	59	TRUE	TRUE
1	DENGTITDA	267	275	9	HLA-A*02:01	267	59	DENGTITDA	DENGTITDA	0.000016	59	1.60E-05	59	TRUE	TRUE
1	DKYFKNHTS	1135	1143	9	HLA-A*02:01	1135	59	DKYFKNHTS	DKYFKNHTS	0.000016	59	1.60E-05	59	TRUE	TRUE
1	LREFVFKNK	182	190	9	HLA-A*02:01	182	59	LREFVFKNK	LREFVFKNK	0.000016	59	1.60E-05	59	TRUE	TRUE
1	NARDLICAQ	827	835	9	HLA-A*02:01	827	59	NARDLICAQ	NARDLICAQ	0.000016	59	1.60E-05	59	TRUE	TRUE
1	PQIITTDNT	1094	1102	9	HLA-A*02:01	1094	59	PQIITTDNT	PQIITTDNT	0.000016	59	1.60E-05	59	TRUE	TRUE
1	PSSKRFQPF	540	548	9	HLA-A*02:01	540	59	PSSKRFQPF	PSSKRFQPF	0.000016	59	1.60E-05	59	TRUE	TRUE
1	PTNFSISIT	697	705	9	HLA-A*02:01	697	59	PTNFSISIT	PTNFSISIT	0.000016	59	1.60E-05	59	TRUE	TRUE
1	QDLFLPFYS	56	64	9	HLA-A*02:01	56	59	QDLFLPFYS	QDLFLPFYS	0.000016	59	1.60E-05	59	TRUE	TRUE
1	VIIINNSTN	114	122	9	HLA-A*02:01	114	59	VIIINNSTN	VIIINNSTN	0.000016	59	1.60E-05	59	TRUE	TRUE
1	DTSYECDIP	643	651	9	HLA-A*02:01	643	60	DTSYECDIP	DTSYECDIP	0.000015	60	1.50E-05	60	TRUE	TRUE
1	INASVVNIQ	1154	1162	9	HLA-A*02:01	1154	60	INASVVNIQ	INASVVNIQ	0.000015	60	1.50E-05	60	TRUE	TRUE
1	LPFYSNVTG	60	68	9	HLA-A*02:01	60	60	LPFYSNVTG	LPFYSNVTG	0.000015	60	1.50E-05	60	TRUE	TRUE
1	NFELCDNPF	129	137	9	HLA-A*02:01	129	60	NFELCDNPF	NFELCDNPF	0.000015	60	1.50E-05	60	TRUE	TRUE
1	TRNIDATST	425	433	9	HLA-A*02:01	425	60	TRNIDATST	TRNIDATST	0.000015	60	1.50E-05	60	TRUE	TRUE
1	VDTSYECDI	642	650	9	HLA-A*02:01	642	60	VDTSYECDI	VDTSYECDI	0.000015	60	1.50E-05	60	TRUE	TRUE
1	CYWPLNDYG	474	482	9	HLA-A*02:01	474	61	CYWPLNDYG	CYWPLNDYG	0.000014	61	1.40E-05	61	TRUE	TRUE
1	DLIKNQCVN	518	526	9	HLA-A*02:01	518	61	DLIKNQCVN	DLIKNQCVN	0.000014	61	1.40E-05	61	TRUE	TRUE
1	DYSVLYNST	351	359	9	HLA-A*02:01	351	61	DYSVLYNST	DYSVLYNST	0.000014	61	1.40E-05	61	TRUE	TRUE
1	GKLQDVVNQ	928	936	9	HLA-A*02:01	928	61	GKLQDVVNQ	GKLQDVVNQ	0.000014	61	1.40E-05	61	TRUE	TRUE
1	ISNCVADYS	345	353	9	HLA-A*02:01	345	61	ISNCVADYS	ISNCVADYS	0.000014	61	1.40E-05	61	TRUE	TRUE
1	KEELDKYFK	1131	1139	9	HLA-A*02:01	1131	61	KEELDKYFK	KEELDKYFK	0.000014	61	1.40E-05	61	TRUE	TRUE
1	MTSCCSCLK	1219	1227	9	HLA-A*02:01	1219	61	MTSCCSCLK	MTSCCSCLK	0.000014	61	1.40E-05	61	TRUE	TRUE
1	PFQQFGRDV	547	555	9	HLA-A*02:01	547	61	PFQQFGRDV	PFQQFGRDV	0.000014	61	1.40E-05	61	TRUE	TRUE
1	PGTNASSEV	586	594	9	HLA-A*02:01	586	61	PGTNASSEV	PGTNASSEV	0.000014	61	1.40E-05	61	TRUE	TRUE
1	VLGQSKRVD	1015	1023	9	HLA-A*02:01	1015	61	VLGQSKRVD	VLGQSKRVD	0.000014	61	1.40E-05	61	TRUE	TRUE
1	APATVCGPK	506	514	9	HLA-A*02:01	506	62	APATVCGPK	APATVCGPK	0.000013	62	1.30E-05	62	TRUE	TRUE
1	DGIYFAATE	85	93	9	HLA-A*02:01	85	62	DGIYFAATE	DGIYFAATE	0.000013	62	1.30E-05	62	TRUE	TRUE
1	DPLKPTKRS	790	798	9	HLA-A*02:01	790	62	DPLKPTKRS	DPLKPTKRS	0.000013	62	1.30E-05	62	TRUE	TRUE
1	IQKEIDRLN	1161	1169	9	HLA-A*02:01	1161	62	IQKEIDRLN	IQKEIDRLN	0.000013	62	1.30E-05	62	TRUE	TRUE
1	IRAAEIRAS	995	1003	9	HLA-A*02:01	995	62	IRAAEIRAS	IRAAEIRAS	0.000013	62	1.30E-05	62	TRUE	TRUE
1	KYRYLRHGK	439	447	9	HLA-A*02:01	439	62	KYRYLRHGK	KYRYLRHGK	0.000013	62	1.30E-05	62	TRUE	TRUE
1	LFNKVTLAD	804	812	9	HLA-A*02:01	804	62	LFNKVTLAD	LFNKVTLAD	0.000013	62	1.30E-05	62	TRUE	TRUE
1	PDDFMGCVL	413	421	9	HLA-A*02:01	413	62	PDDFMGCVL	PDDFMGCVL	0.000013	62	1.30E-05	62	TRUE	TRUE
1	PPLLTDDMI	844	852	9	HLA-A*02:01	844	62	PPLLTDDMI	PPLLTDDMI	0.000013	62	1.30E-05	62	TRUE	TRUE
1	PSGFNTLKP	210	218	9	HLA-A*02:01	210	62	PSGFNTLKP	PSGFNTLKP	0.000013	62	1.30E-05	62	TRUE	TRUE
1	QNVLYENQK	895	903	9	HLA-A*02:01	895	62	QNVLYENQK	QNVLYENQK	0.000013	62	1.30E-05	62	TRUE	TRUE
1	APAICHEGK	1060	1068	9	HLA-A*02:01	1060	63	APAICHEGK	APAICHEGK	0.000012	63	1.20E-05	63	TRUE	TRUE
1	DDVQAPNYT	23	31	9	HLA-A*02:01	23	63	DDVQAPNYT	DDVQAPNYT	0.000012	63	1.20E-05	63	TRUE	TRUE
1	MYICGDSTE	722	730	9	HLA-A*02:01	722	63	MYICGDSTE	MYICGDSTE	0.000012	63	1.20E-05	63	TRUE	TRUE
1	QTQAGCLIG	630	638	9	HLA-A*02:01	630	63	QTQAGCLIG	QTQAGCLIG	0.000012	63	1.20E-05	63	TRUE	TRUE
1	QYGECLGDI	818	826	9	HLA-A*02:01	818	63	QYGECLGDI	QYGECLGDI	0.000012	63	1.20E-05	63	TRUE	TRUE
1	SEPVLKGVK	1243	1251	9	HLA-A*02:01	1243	63	SEPVLKGVK	SEPVLKGVK	0.000012	63	1.20E-05	63	TRUE	TRUE
1	CDNPFFAVS	133	141	9	HLA-A*02:01	133	64	CDNPFFAVS	CDNPFFAVS	0.000011	64	1.10E-05	64	TRUE	TRUE
1	CVLAWNTRN	419	427	9	HLA-A*02:01	419	64	CVLAWNTRN	CVLAWNTRN	0.000011	64	1.10E-05	64	TRUE	TRUE
1	DISPCSFGG	572	580	9	HLA-A*02:01	572	64	DISPCSFGG	DISPCSFGG	0.000011	64	1.10E-05	64	TRUE	TRUE
1	GACSCGSCC	1228	1236	9	HLA-A*02:01	1228	64	GACSCGSCC	GACSCGSCC	0.000011	64	1.10E-05	64	TRUE	TRUE
1	GGVSVITPG	579	587	9	HLA-A*02:01	579	64	GGVSVITPG	GGVSVITPG	0.000011	64	1.10E-05	64	TRUE	TRUE
1	NDILSRLDK	960	968	9	HLA-A*02:01	960	64	NDILSRLDK	NDILSRLDK	0.000011	64	1.10E-05	64	TRUE	TRUE
1	NESLIDLQE	1176	1184	9	HLA-A*02:01	1176	64	NESLIDLQE	NESLIDLQE	0.000011	64	1.10E-05	64	TRUE	TRUE
1	RDVSDFTDS	553	561	9	HLA-A*02:01	553	64	RDVSDFTDS	RDVSDFTDS	0.000011	64	1.10E-05	64	TRUE	TRUE
1	SKRVDFCGK	1019	1027	9	HLA-A*02:01	1019	64	SKRVDFCGK	SKRVDFCGK	0.000011	64	1.10E-05	64	TRUE	TRUE
1	TSTALGKLQ	923	931	9	HLA-A*02:01	923	64	TSTALGKLQ	TSTALGKLQ	0.000011	64	1.10E-05	64	TRUE	TRUE
1	AYTAALVSG	854	862	9	HLA-A*02:01	854	66	AYTAALVSG	AYTAALVSG	0.00001	66	1.00E-05	66	TRUE	TRUE
1	CLKGACSCG	1225	1233	9	HLA-A*02:01	1225	66	CLKGACSCG	CLKGACSCG	0.00001	66	1.00E-05	66	TRUE	TRUE
1	DLDRCTTFD	15	23	9	HLA-A*02:01	15	66	DLDRCTTFD	DLDRCTTFD	0.00001	66	1.00E-05	66	TRUE	TRUE
1	FGAISSVLN	952	960	9	HLA-A*02:01	952	66	FGAISSVLN	FGAISSVLN	0.00001	66	1.00E-05	66	TRUE	TRUE
1	FNGTSWFIT	1079	1087	9	HLA-A*02:01	1079	66	FNGTSWFIT	FNGTSWFIT	0.00001	66	1.00E-05	66	TRUE	TRUE
1	KPCTPPALN	465	473	9	HLA-A*02:01	465	66	KPCTPPALN	KPCTPPALN	0.00001	66	1.00E-05	66	TRUE	TRUE
1	LICAQKFNG	831	839	9	HLA-A*02:01	831	66	LICAQKFNG	LICAQKFNG	0.00001	66	1.00E-05	66	TRUE	TRUE
1	MPVSMAKTS	709	717	9	HLA-A*02:01	709	66	MPVSMAKTS	MPVSMAKTS	0.00001	66	1.00E-05	66	TRUE	TRUE
1	PIGAGICAS	651	659	9	HLA-A*02:01	651	66	PIGAGICAS	PIGAGICAS	0.00001	66	1.00E-05	66	TRUE	TRUE
1	TECANLLLQ	729	737	9	HLA-A*02:01	729	66	TECANLLLQ	TECANLLLQ	0.00001	66	1.00E-05	66	TRUE	TRUE
1	TFGAGAALQ	869	877	9	HLA-A*02:01	869	66	TFGAGAALQ	TFGAGAALQ	0.00001	66	1.00E-05	66	TRUE	TRUE
1	CGKGYHLMS	1025	1033	9	HLA-A*02:01	1025	67	CGKGYHLMS	CGKGYHLMS	0.000009	67	9.00E-06	67	TRUE	TRUE
1	CKFDEDDSE	1236	1244	9	HLA-A*02:01	1236	67	CKFDEDDSE	CKFDEDDSE	0.000009	67	9.00E-06	67	TRUE	TRUE
1	DFTDSVRDP	557	565	9	HLA-A*02:01	557	67	DFTDSVRDP	DFTDSVRDP	0.000009	67	9.00E-06	67	TRUE	TRUE
1	DSVRDPKTS	560	568	9	HLA-A*02:01	560	67	DSVRDPKTS	DSVRDPKTS	0.000009	67	9.00E-06	67	TRUE	TRUE
1	ENQKQIANQ	900	908	9	HLA-A*02:01	900	67	ENQKQIANQ	ENQKQIANQ	0.000009	67	9.00E-06	67	TRUE	TRUE
1	ISNVPFSPD	455	463	9	HLA-A*02:01	455	67	ISNVPFSPD	ISNVPFSPD	0.000009	67	9.00E-06	67	TRUE	TRUE
1	KDGIYFAAT	84	92	9	HLA-A*02:01	84	67	KDGIYFAAT	KDGIYFAAT	0.000009	67	9.00E-06	67	TRUE	TRUE
1	KVEAEVQID	968	976	9	HLA-A*02:01	968	67	KVEAEVQID	KVEAEVQID	0.000009	67	9.00E-06	67	TRUE	TRUE
1	KYEQYIKWP	1187	1195	9	HLA-A*02:01	1187	67	KYEQYIKWP	KYEQYIKWP	0.000009	67	9.00E-06	67	TRUE	TRUE
1	LPLGINITN	222	230	9	HLA-A*02:01	222	67	LPLGINITN	LPLGINITN	0.000009	67	9.00E-06	67	TRUE	TRUE
1	NPLAELKCS	281	289	9	HLA-A*02:01	281	67	NPLAELKCS	NPLAELKCS	0.000009	67	9.00E-06	67	TRUE	TRUE
1	NVTGFHTIN	65	73	9	HLA-A*02:01	65	67	NVTGFHTIN	NVTGFHTIN	0.000009	67	9.00E-06	67	TRUE	TRUE
1	PSVYAWERK	335	343	9	HLA-A*02:01	335	67	PSVYAWERK	PSVYAWERK	0.000009	67	9.00E-06	67	TRUE	TRUE
1	RNTREVFAQ	758	766	9	HLA-A*02:01	758	67	RNTREVFAQ	RNTREVFAQ	0.000009	67	9.00E-06	67	TRUE	TRUE
1	TDSVRDPKT	559	567	9	HLA-A*02:01	559	67	TDSVRDPKT	TDSVRDPKT	0.000009	67	9.00E-06	67	TRUE	TRUE

seq #	peptide	start	end	peptide length	allele	peptide index	median binding	netmhcpan_el	netmhcpan_el	netmhcpan_el	netmhcpan_el	legacy score	legacy rank	scores equal	percentiles equal
1	VSTAIHADQ	606	614	9	HLA-A*02:01	606	67	VSTAIHADQ	VSTAIHADQ	0.000009	67	9.00E-06	67	TRUE	TRUE
1	AEHVDTSYE	639	647	9	HLA-A*02:01	639	69	AEHVDTSYE	AEHVDTSYE	0.000008	69	8.00E-06	69	TRUE	TRUE
1	AEQDRNTRE	754	762	9	HLA-A*02:01	754	69	AEQDRNTRE	AEQDRNTRE	0.000008	69	8.00E-06	69	TRUE	TRUE
1	CHEGKAYFP	1064	1072	9	HLA-A*02:01	1064	69	CHEGKAYFP	CHEGKAYFP	0.000008	69	8.00E-06	69	TRUE	TRUE
1	DVVIGIINN	1109	1117	9	HLA-A*02:01	1109	69	DVVIGIINN	DVVIGIINN	0.000008	69	8.00E-06	69	TRUE	TRUE
1	FNKAISQIQ	909	917	9	HLA-A*02:01	909	69	FNKAISQIQ	FNKAISQIQ	0.000008	69	8.00E-06	69	TRUE	TRUE
1	GAGICASYH	653	661	9	HLA-A*02:01	653	69	GAGICASYH	GAGICASYH	0.000008	69	8.00E-06	69	TRUE	TRUE
1	GFYTTTGIG	482	490	9	HLA-A*02:01	482	69	GFYTTTGIG	GFYTTTGIG	0.000008	69	8.00E-06	69	TRUE	TRUE
1	GSTMNNKSQ	104	112	9	HLA-A*02:01	104	69	GSTMNNKSQ	GSTMNNKSQ	0.000008	69	8.00E-06	69	TRUE	TRUE
1	HVDTSYECD	641	649	9	HLA-A*02:01	641	69	HVDTSYECD	HVDTSYECD	0.000008	69	8.00E-06	69	TRUE	TRUE
1	NFNFNGLTG	526	534	9	HLA-A*02:01	526	69	NFNFNGLTG	NFNFNGLTG	0.000008	69	8.00E-06	69	TRUE	TRUE
1	RHGKLRPFE	444	452	9	HLA-A*02:01	444	69	RHGKLRPFE	RHGKLRPFE	0.000008	69	8.00E-06	69	TRUE	TRUE
1	SGTATAGWT	861	869	9	HLA-A*02:01	861	69	SGTATAGWT	SGTATAGWT	0.000008	69	8.00E-06	69	TRUE	TRUE
1	TKLNDLCFS	372	380	9	HLA-A*02:01	372	69	TKLNDLCFS	TKLNDLCFS	0.000008	69	8.00E-06	69	TRUE	TRUE
1	TSAAAYFVG	247	255	9	HLA-A*02:01	247	69	TSAAAYFVG	TSAAAYFVG	0.000008	69	8.00E-06	69	TRUE	TRUE
1	VSMAKTSVD	711	719	9	HLA-A*02:01	711	69	VSMAKTSVD	VSMAKTSVD	0.000008	69	8.00E-06	69	TRUE	TRUE
1	ACSCGSCCK	1229	1237	9	HLA-A*02:01	1229	71	ACSCGSCCK	ACSCGSCCK	0.000007	71	7.00E-06	71	TRUE	TRUE
1	ANLAATKMS	1004	1012	9	HLA-A*02:01	1004	71	ANLAATKMS	ANLAATKMS	0.000007	71	7.00E-06	71	TRUE	TRUE
1	CNFELCDNP	128	136	9	HLA-A*02:01	128	71	CNFELCDNP	CNFELCDNP	0.000007	71	7.00E-06	71	TRUE	TRUE
1	CSCLKGACS	1223	1231	9	HLA-A*02:01	1223	71	CSCLKGACS	CSCLKGACS	0.000007	71	7.00E-06	71	TRUE	TRUE
1	DCSQNPLAE	277	285	9	HLA-A*02:01	277	71	DCSQNPLAE	DCSQNPLAE	0.000007	71	7.00E-06	71	TRUE	TRUE
1	DLGDISGIN	1147	1155	9	HLA-A*02:01	1147	71	DLGDISGIN	DLGDISGIN	0.000007	71	7.00E-06	71	TRUE	TRUE
1	DRCTTFDDV	17	25	9	HLA-A*02:01	17	71	DRCTTFDDV	DRCTTFDDV	0.000007	71	7.00E-06	71	TRUE	TRUE
1	EDLLFNKVT	801	809	9	HLA-A*02:01	801	71	EDLLFNKVT	EDLLFNKVT	0.000007	71	7.00E-06	71	TRUE	TRUE
1	EGKAYFPRE	1066	1074	9	HLA-A*02:01	1066	71	EGKAYFPRE	EGKAYFPRE	0.000007	71	7.00E-06	71	TRUE	TRUE
1	FFSTFKCYG	360	368	9	HLA-A*02:01	360	71	FFSTFKCYG	FFSTFKCYG	0.000007	71	7.00E-06	71	TRUE	TRUE
1	FNCTFEYIS	157	165	9	HLA-A*02:01	157	71	FNCTFEYIS	FNCTFEYIS	0.000007	71	7.00E-06	71	TRUE	TRUE
1	NEVAKNLNE	1169	1177	9	HLA-A*02:01	1169	71	NEVAKNLNE	NEVAKNLNE	0.000007	71	7.00E-06	71	TRUE	TRUE
1	SEVAVLYQD	592	600	9	HLA-A*02:01	592	71	SEVAVLYQD	SEVAVLYQD	0.000007	71	7.00E-06	71	TRUE	TRUE
1	SGINASVVN	1152	1160	9	HLA-A*02:01	1152	71	SGINASVVN	SGINASVVN	0.000007	71	7.00E-06	71	TRUE	TRUE
1	TEKSNVVRG	92	100	9	HLA-A*02:01	92	71	TEKSNVVRG	TEKSNVVRG	0.000007	71	7.00E-06	71	TRUE	TRUE
1	VDFCGKGYH	1022	1030	9	HLA-A*02:01	1022	71	VDFCGKGYH	VDFCGKGYH	0.000007	71	7.00E-06	71	TRUE	TRUE
1	WPLNDYGFY	476	484	9	HLA-A*02:01	476	71	WPLNDYGFY	WPLNDYGFY	0.000007	71	7.00E-06	71	TRUE	TRUE
1	YENQKQIAN	899	907	9	HLA-A*02:01	899	71	YENQKQIAN	YENQKQIAN	0.000007	71	7.00E-06	71	TRUE	TRUE
1	YKLPDDFMG	410	418	9	HLA-A*02:01	410	71	YKLPDDFMG	YKLPDDFMG	0.000007	71	7.00E-06	71	TRUE	TRUE
1	DDSEPVLKG	1241	1249	9	HLA-A*02:01	1241	73	DDSEPVLKG	DDSEPVLKG	0.000006	73	6.00E-06	73	TRUE	TRUE
1	DPLQPELDS	1121	1129	9	HLA-A*02:01	1121	73	DPLQPELDS	DPLQPELDS	0.000006	73	6.00E-06	73	TRUE	TRUE
1	EELDKYFKN	1132	1140	9	HLA-A*02:01	1132	73	EELDKYFKN	EELDKYFKN	0.000006	73	6.00E-06	73	TRUE	TRUE
1	GIAAEQDRN	751	759	9	HLA-A*02:01	751	73	GIAAEQDRN	GIAAEQDRN	0.000006	73	6.00E-06	73	TRUE	TRUE
1	NTFVSGNCD	1101	1109	9	HLA-A*02:01	1101	73	NTFVSGNCD	NTFVSGNCD	0.000006	73	6.00E-06	73	TRUE	TRUE
1	PAQDIWGTS	240	248	9	HLA-A*02:01	240	73	PAQDIWGTS	PAQDIWGTS	0.000006	73	6.00E-06	73	TRUE	TRUE
1	PFAMQMAYR	879	887	9	HLA-A*02:01	879	73	PFAMQMAYR	PFAMQMAYR	0.000006	73	6.00E-06	73	TRUE	TRUE
1	SDAFSLDVS	165	173	9	HLA-A*02:01	165	73	SDAFSLDVS	SDAFSLDVS	0.000006	73	6.00E-06	73	TRUE	TRUE
1	VCGPKLSTD	510	518	9	HLA-A*02:01	510	73	VCGPKLSTD	VCGPKLSTD	0.000006	73	6.00E-06	73	TRUE	TRUE
1	ADSSIAYSN	683	691	9	HLA-A*02:01	683	75	ADSSIAYSN	ADSSIAYSN	0.000005	75	5.00E-06	75	TRUE	TRUE
1	CANLLLQYG	731	739	9	HLA-A*02:01	731	75	CANLLLQYG	CANLLLQYG	0.000005	75	5.00E-06	75	TRUE	TRUE
1	KPTKRSFIE	793	801	9	HLA-A*02:01	793	75	KPTKRSFIE	KPTKRSFIE	0.000005	75	5.00E-06	75	TRUE	TRUE
1	LAWNTRNID	421	429	9	HLA-A*02:01	421	75	LAWNTRNID	LAWNTRNID	0.000005	75	5.00E-06	75	TRUE	TRUE
1	LNRALSGIA	745	753	9	HLA-A*02:01	745	75	LNRALSGIA	LNRALSGIA	0.000005	75	5.00E-06	75	TRUE	TRUE
1	LNTLVKQLS	941	949	9	HLA-A*02:01	941	75	LNTLVKQLS	LNTLVKQLS	0.000005	75	5.00E-06	75	TRUE	TRUE
1	MAYRFNGIG	884	892	9	HLA-A*02:01	884	75	MAYRFNGIG	MAYRFNGIG	0.000005	75	5.00E-06	75	TRUE	TRUE
1	NKAISQIQE	910	918	9	HLA-A*02:01	910	75	NKAISQIQE	NKAISQIQE	0.000005	75	5.00E-06	75	TRUE	TRUE
1	NYKYRYLRH	437	445	9	HLA-A*02:01	437	75	NYKYRYLRH	NYKYRYLRH	0.000005	75	5.00E-06	75	TRUE	TRUE
1	PDPLKPTKR	789	797	9	HLA-A*02:01	789	75	PDPLKPTKR	PDPLKPTKR	0.000005	75	5.00E-06	75	TRUE	TRUE
1	PFSPDGKPC	459	467	9	HLA-A*02:01	459	75	PFSPDGKPC	PFSPDGKPC	0.000005	75	5.00E-06	75	TRUE	TRUE
1	QCVNFNFNG	523	531	9	HLA-A*02:01	523	75	QCVNFNFNG	QCVNFNFNG	0.000005	75	5.00E-06	75	TRUE	TRUE
1	REGVFVFNG	1073	1081	9	HLA-A*02:01	1073	75	REGVFVFNG	REGVFVFNG	0.000005	75	5.00E-06	75	TRUE	TRUE
1	RKKISNCVA	342	350	9	HLA-A*02:01	342	75	RKKISNCVA	RKKISNCVA	0.000005	75	5.00E-06	75	TRUE	TRUE
1	SDFTDSVRD	556	564	9	HLA-A*02:01	556	75	SDFTDSVRD	SDFTDSVRD	0.000005	75	5.00E-06	75	TRUE	TRUE
1	SGDVVRFPN	310	318	9	HLA-A*02:01	310	75	SGDVVRFPN	SGDVVRFPN	0.000005	75	5.00E-06	75	TRUE	TRUE
1	SKRFQPFQQ	542	550	9	HLA-A*02:01	542	75	SKRFQPFQQ	SKRFQPFQQ	0.000005	75	5.00E-06	75	TRUE	TRUE
1	SPCSFGGVS	574	582	9	HLA-A*02:01	574	75	SPCSFGGVS	SPCSFGGVS	0.000005	75	5.00E-06	75	TRUE	TRUE
1	VRQIAPGQT	394	402	9	HLA-A*02:01	394	75	VRQIAPGQT	VRQIAPGQT	0.000005	75	5.00E-06	75	TRUE	TRUE
1	VVNIQKEID	1158	1166	9	HLA-A*02:01	1158	75	VVNIQKEID	VVNIQKEID	0.000005	75	5.00E-06	75	TRUE	TRUE
1	YFAATEKSN	88	96	9	HLA-A*02:01	88	75	YFAATEKSN	YFAATEKSN	0.000005	75	5.00E-06	75	TRUE	TRUE
1	CFSNVYADS	378	386	9	HLA-A*02:01	378	78	CFSNVYADS	CFSNVYADS	0.000004	78	4.00E-06	78	TRUE	TRUE
1	DNTFVSGNC	1100	1108	9	HLA-A*02:01	1100	78	DNTFVSGNC	DNTFVSGNC	0.000004	78	4.00E-06	78	TRUE	TRUE
1	ECDIPIGAG	647	655	9	HLA-A*02:01	647	78	ECDIPIGAG	ECDIPIGAG	0.000004	78	4.00E-06	78	TRUE	TRUE
1	GRDVSDFTD	552	560	9	HLA-A*02:01	552	78	GRDVSDFTD	GRDVSDFTD	0.000004	78	4.00E-06	78	TRUE	TRUE
1	LDKYFKNHT	1134	1142	9	HLA-A*02:01	1134	78	LDKYFKNHT	LDKYFKNHT	0.000004	78	4.00E-06	78	TRUE	TRUE
1	LNAPATVCG	504	512	9	HLA-A*02:01	504	78	LNAPATVCG	LNAPATVCG	0.000004	78	4.00E-06	78	TRUE	TRUE
1	LYQDVNCTD	597	605	9	HLA-A*02:01	597	78	LYQDVNCTD	LYQDVNCTD	0.000004	78	4.00E-06	78	TRUE	TRUE
1	NDLCFSNVY	375	383	9	HLA-A*02:01	375	78	NDLCFSNVY	NDLCFSNVY	0.000004	78	4.00E-06	78	TRUE	TRUE
1	PKLSTDLIK	513	521	9	HLA-A*02:01	513	78	PKLSTDLIK	PKLSTDLIK	0.000004	78	4.00E-06	78	TRUE	TRUE
1	PNITNLCPF	317	325	9	HLA-A*02:01	317	78	PNITNLCPF	PNITNLCPF	0.000004	78	4.00E-06	78	TRUE	TRUE
1	QTHTMIFDN	147	155	9	HLA-A*02:01	147	78	QTHTMIFDN	QTHTMIFDN	0.000004	78	4.00E-06	78	TRUE	TRUE
1	SNVPFSPDG	456	464	9	HLA-A*02:01	456	78	SNVPFSPDG	SNVPFSPDG	0.000004	78	4.00E-06	78	TRUE	TRUE
1	TATAGWTFG	863	871	9	HLA-A*02:01	863	78	TATAGWTFG	TATAGWTFG	0.000004	78	4.00E-06	78	TRUE	TRUE
1	VKQMYKTPT	767	775	9	HLA-A*02:01	767	78	VKQMYKTPT	VKQMYKTPT	0.000004	78	4.00E-06	78	TRUE	TRUE
1	VRGWVFGST	98	106	9	HLA-A*02:01	98	78	VRGWVFGST	VRGWVFGST	0.000004	78	4.00E-06	78	TRUE	TRUE
1	VSVITPGTN	581	589	9	HLA-A*02:01	581	78	VSVITPGTN	VSVITPGTN	0.000004	78	4.00E-06	78	TRUE	TRUE
1	DLICAQKFN	830	838	9	HLA-A*02:01	830	82	DLICAQKFN	DLICAQKFN	0.000003	82	3.00E-06	82	TRUE	TRUE
1	GYHLMSFPQ	1028	1036	9	HLA-A*02:01	1028	82	GYHLMSFPQ	GYHLMSFPQ	0.000003	82	3.00E-06	82	TRUE	TRUE
1	IDVVRDLPS	203	211	9	HLA-A*02:01	203	82	IDVVRDLPS	IDVVRDLPS	0.000003	82	3.00E-06	82	TRUE	TRUE
1	IFKLPLGIN	219	227	9	HLA-A*02:01	219	82	IFKLPLGIN	IFKLPLGIN	0.000003	82	3.00E-06	82	TRUE	TRUE
1	KNLNESLID	1173	1181	9	HLA-A*02:01	1173	82	KNLNESLID	KNLNESLID	0.000003	82	3.00E-06	82	TRUE	TRUE
1	LCPFGEVFN	322	330	9	HLA-A*02:01	322	82	LCPFGEVFN	LCPFGEVFN	0.000003	82	3.00E-06	82	TRUE	TRUE
1	LDISPCSFG	571	579	9	HLA-A*02:01	571	82	LDISPCSFG	LDISPCSFG	0.000003	82	3.00E-06	82	TRUE	TRUE
1	LNESLIDLQ	1175	1183	9	HLA-A*02:01	1175	82	LNESLIDLQ	LNESLIDLQ	0.000003	82	3.00E-06	82	TRUE	TRUE
1	NFNGLTGTG	528	536	9	HLA-A*02:01	528	82	NFNGLTGTG	NFNGLTGTG	0.000003	82	3.00E-06	82	TRUE	TRUE
1	PAICHEGKA	1061	1069	9	HLA-A*02:01	1061	82	PAICHEGKA	PAICHEGKA	0.000003	82	3.00E-06	82	TRUE	TRUE
1	PAWRIYSTG	617	625	9	HLA-A*02:01	617	82	PAWRIYSTG	PAWRIYSTG	0.000003	82	3.00E-06	82	TRUE	TRUE
1	PDGKPCTPP	462	470	9	HLA-A*02:01	462	82	PDGKPCTPP	PDGKPCTPP	0.000003	82	3.00E-06	82	TRUE	TRUE
1	PELDSFKEE	1125	1133	9	HLA-A*02:01	1125	82	PELDSFKEE	PELDSFKEE	0.000003	82	3.00E-06	82	TRUE	TRUE
1	RCTTFDDVQ	18	26	9	HLA-A*02:01	18	82	RCTTFDDVQ	RCTTFDDVQ	0.000003	82	3.00E-06	82	TRUE	TRUE
1	REFVFKNKD	183	191	9	HLA-A*02:01	183	82	REFVFKNKD	REFVFKNKD	0.000003	82	3.00E-06	82	TRUE	TRUE
1	TDNTFVSGN	1099	1107	9	HLA-A*02:01	1099	82	TDNTFVSGN	TDNTFVSGN	0.000003	82	3.00E-06	82	TRUE	TRUE
1	TKMSECVLG	1009	1017	9	HLA-A*02:01	1009	82	TKMSECVLG	TKMSECVLG	0.000003	82	3.00E-06	82	TRUE	TRUE
1	VAVLYQDVN	594	602	9	HLA-A*02:01	594	82	VAVLYQDVN	VAVLYQDVN	0.000003	82	3.00E-06	82	TRUE	TRUE
1	WNTRNIDAT	423	431	9	HLA-A*02:01	423	82	WNTRNIDAT	WNTRNIDAT	0.000003	82	3.00E-06	82	TRUE	TRUE
1	YKYRYLRHG	438	446	9	HLA-A*02:01	438	82	YKYRYLRHG	YKYRYLRHG	0.000003	82	3.00E-06	82	TRUE	TRUE
1	AAEIRASAN	997	1005	9	HLA-A*02:01	997	87	AAEIRASAN	AAEIRASAN	0.000002	87	2.00E-06	87	TRUE	TRUE
1	ADYNYKLPD	406	414	9	HLA-A*02:01	406	87	ADYNYKLPD	ADYNYKLPD	0.000002	87	2.00E-06	87	TRUE	TRUE
1	ANQFNKAIS	906	914	9	HLA-A*02:01	906	87	ANQFNKAIS	ANQFNKAIS	0.000002	87	2.00E-06	87	TRUE	TRUE
1	AWERKKISN	339	347	9	HLA-A*02:01	339	87	AWERKKISN	AWERKKISN	0.000002	87	2.00E-06	87	TRUE	TRUE
1	CCSCLKGAC	1222	1230	9	HLA-A*02:01	1222	87	CCSCLKGAC	CCSCLKGAC	0.000002	87	2.00E-06	87	TRUE	TRUE
1	CSVKSFEID	288	296	9	HLA-A*02:01	288	87	CSVKSFEID	CSVKSFEID	0.000002	87	2.00E-06	87	TRUE	TRUE
1	DNAFNCTFE	154	162	9	HLA-A*02:01	154	87	DNAFNCTFE	DNAFNCTFE	0.000002	87	2.00E-06	87	TRUE	TRUE
1	FNGIGVTQN	888	896	9	HLA-A*02:01	888	87	FNGIGVTQN	FNGIGVTQN	0.000002	87	2.00E-06	87	TRUE	TRUE
1	LDKVEAEVQ	966	974	9	HLA-A*02:01	966	87	LDKVEAEVQ	LDKVEAEVQ	0.000002	87	2.00E-06	87	TRUE	TRUE
1	LKCSVKSFE	286	294	9	HLA-A*02:01	286	87	LKCSVKSFE	LKCSVKSFE	0.000002	87	2.00E-06	87	TRUE	TRUE
1	MAKTSVDCN	713	721	9	HLA-A*02:01	713	87	MAKTSVDCN	MAKTSVDCN	0.000002	87	2.00E-06	87	TRUE	TRUE
1	NNTIAIPTN	691	699	9	HLA-A*02:01	691	87	NNTIAIPTN	NNTIAIPTN	0.000002	87	2.00E-06	87	TRUE	TRUE
1	NNTVYDPLQ	1116	1124	9	HLA-A*02:01	1116	87	NNTVYDPLQ	NNTVYDPLQ	0.000002	87	2.00E-06	87	TRUE	TRUE
1	NQCVNFNFN	522	530	9	HLA-A*02:01	522	87	NQCVNFNFN	NQCVNFNFN	0.000002	87	2.00E-06	87	TRUE	TRUE

seq #	peptide	start	end	peptide length	allele	peptide index	median binding	netmhcpan_el	netmhcpan_el	netmhcpan_el	netmhcpan_el	legacy score	legacy rank	scores equal	percentiles equal	
1	PKTSEILDI	565	573	9	HLA-A*02:01	565	87	PKTSEILDI	PKTSEILDI	0.000002	87	2.00E-06	87	TRUE	TRUE	
1	PNYTQHTSS	28	36	9	HLA-A*02:01	28	87	PNYTQHTSS	PNYTQHTSS	0.000002	87	2.00E-06	87	TRUE	TRUE	
1	PPALNCYWP	469	477	9	HLA-A*02:01	469	87	PPALNCYWP	PPALNCYWP	0.000002	87	2.00E-06	87	TRUE	TRUE	
1	QAGCLIGAE	632	640	9	HLA-A*02:01	632	87	QAGCLIGAE	QAGCLIGAE	0.000002	87	2.00E-06	87	TRUE	TRUE	
1	QDVVNQNAQ	931	939	9	HLA-A*02:01	931	87	QDVVNQNAQ	QDVVNQNAQ	0.000002	87	2.00E-06	87	TRUE	TRUE	
1	QPELDSFKE	1124	1132	9	HLA-A*02:01	1124	87	QPELDSFKE	QPELDSFKE	0.000002	87	2.00E-06	87	TRUE	TRUE	
1	QPFQQFGRD	546	554	9	HLA-A*02:01	546	87	QPFQQFGRD	QPFQQFGRD	0.000002	87	2.00E-06	87	TRUE	TRUE	
1	QSKRVDFCG	1018	1026	9	HLA-A*02:01	1018	87	QSKRVDFCG	QSKRVDFCG	0.000002	87	2.00E-06	87	TRUE	TRUE	
1	QTGVIADYN	401	409	9	HLA-A*02:01	401	87	QTGVIADYN	QTGVIADYN	0.000002	87	2.00E-06	87	TRUE	TRUE	
1	RACNFELCD	126	134	9	HLA-A*02:01	126	87	RACNFELCD	RACNFELCD	0.000002	87	2.00E-06	87	TRUE	TRUE	
1	SDTLYLTQD	49	57	9	HLA-A*02:01	49	87	SDTLYLTQD	SDTLYLTQD	0.000002	87	2.00E-06	87	TRUE	TRUE	
1	SGNCDVVIG	1105	1113	9	HLA-A*02:01	1105	87	SGNCDVVIG	SGNCDVVIG	0.000002	87	2.00E-06	87	TRUE	TRUE	
1	SYECDIPIG	645	653	9	HLA-A*02:01	645	87	SYECDIPIG	SYECDIPIG	0.000002	87	2.00E-06	87	TRUE	TRUE	
1	TAGWTFGAG	865	873	9	HLA-A*02:01	865	87	TAGWTFGAG	TAGWTFGAG	0.000002	87	2.00E-06	87	TRUE	TRUE	
1	TFMLKYDEN	261	269	9	HLA-A*02:01	261	87	TFMLKYDEN	TFMLKYDEN	0.000002	87	2.00E-06	87	TRUE	TRUE	
1	TPGTNASSE	585	593	9	HLA-A*02:01	585	87	TPGTNASSE	TPGTNASSE	0.000002	87	2.00E-06	87	TRUE	TRUE	
1	TSCCSCLKG	1220	1228	9	HLA-A*02:01	1220	87	TSCCSCLKG	TSCCSCLKG	0.000002	87	2.00E-06	87	TRUE	TRUE	
1	TYVPSQERN	1048	1056	9	HLA-A*02:01	1048	87	TYVPSQERN	TYVPSQERN	0.000002	87	2.00E-06	87	TRUE	TRUE	
1	VRDLPSGFN	206	214	9	HLA-A*02:01	206	87	VRDLPSGFN	VRDLPSGFN	0.000002	87	2.00E-06	87	TRUE	TRUE	
1	VYKGYQPID	196	204	9	HLA-A*02:01	196	87	VYKGYQPID	VYKGYQPID	0.000002	87	2.00E-06	87	TRUE	TRUE	
1	YDENGTITD	266	274	9	HLA-A*02:01	266	87	YDENGTITD	YDENGTITD	0.000002	87	2.00E-06	87	TRUE	TRUE	
1	YFKNHTSPD	1137	1145	9	HLA-A*02:01	1137	87	YFKNHTSPD	YFKNHTSPD	0.000002	87	2.00E-06	87	TRUE	TRUE	
1	YGSFCTQLN	738	746	9	HLA-A*02:01	738	87	YGSFCTQLN	YGSFCTQLN	0.000002	87	2.00E-06	87	TRUE	TRUE	
1	YGVSATKLN	367	375	9	HLA-A*02:01	367	87	YGVSATKLN	YGVSATKLN	0.000002	87	2.00E-06	87	TRUE	TRUE	
1	ADSFVVKGD	384	392	9	HLA-A*02:01	384	95	ADSFVVKGD	ADSFVVKGD	0.000001	95	1.00E-06	95	TRUE	TRUE	
1	AWRIYSTGN	618	626	9	HLA-A*02:01	618	95	AWRIYSTGN	AWRIYSTGN	0.000001	95	1.00E-06	95	TRUE	TRUE	
1	CDVVIGIIN	1108	1116	9	HLA-A*02:01	1108	95	CDVVIGIIN	CDVVIGIIN	0.000001	95	1.00E-06	95	TRUE	TRUE	
1	DAGFMKQYG	812	820	9	HLA-A*02:01	812	95	DAGFMKQYG	DAGFMKQYG	0.000001	95	1.00E-06	95	TRUE	TRUE	
1	DATSTGNYN	429	437	9	HLA-A*02:01	429	95	DATSTGNYN	DATSTGNYN	0.000001	95	1.00E-06	95	TRUE	TRUE	
1	DDVRQIAPG	392	400	9	HLA-A*02:01	392	95	DDVRQIAPG	DDVRQIAPG	0.000001	95	1.00E-06	95	TRUE	TRUE	
1	DPKTSEILD	564	572	9	HLA-A*02:01	564	95	DPKTSEILD	DPKTSEILD	0.000001	95	1.00E-06	95	TRUE	TRUE	
1	DRLITGRLQ	976	984	9	HLA-A*02:01	976	95	DRLITGRLQ	DRLITGRLQ	0.000001	95	1.00E-06	95	TRUE	TRUE	
1	DRLNEVAKN	1166	1174	9	HLA-A*02:01	1166	95	DRLNEVAKN	DRLNEVAKN	0.000001	95	1.00E-06	95	TRUE	TRUE	
1	DSSIAYSNN	684	692	9	HLA-A*02:01	684	95	DSSIAYSNN	DSSIAYSNN	0.000001	95	1.00E-06	95	TRUE	TRUE	
1	DYGFYTTTG	480	488	9	HLA-A*02:01	480	95	DYGFYTTTG	DYGFYTTTG	0.000001	95	1.00E-06	95	TRUE	TRUE	
1	EFVFKNKDG	184	192	9	HLA-A*02:01	184	95	EFVFKNKDG	EFVFKNKDG	0.000001	95	1.00E-06	95	TRUE	TRUE	
1	FHTINHTFG	69	77	9	HLA-A*02:01	69	95	FHTINHTFG	FHTINHTFG	0.000001	95	1.00E-06	95	TRUE	TRUE	
1	GKAYFPREG	1067	1075	9	HLA-A*02:01	1067	95	GKAYFPREG	GKAYFPREG	0.000001	95	1.00E-06	95	TRUE	TRUE	
1	GNPVIPFKD	77	85	9	HLA-A*02:01	77	95	GNPVIPFKD	GNPVIPFKD	0.000001	95	1.00E-06	95	TRUE	TRUE	
1	GSCCKFDED	1233	1241	9	HLA-A*02:01	1233	95	GSCCKFDED	GSCCKFDED	0.000001	95	1.00E-06	95	TRUE	TRUE	
1	GWVFGSTMN	100	108	9	HLA-A*02:01	100	95	GWVFGSTMN	GWVFGSTMN	0.000001	95	1.00E-06	95	TRUE	TRUE	
1	KKISNCVAD	343	351	9	HLA-A*02:01	343	95	KKISNCVAD	KKISNCVAD	0.000001	95	1.00E-06	95	TRUE	TRUE	
1	KNHTSPDVD	1139	1147	9	HLA-A*02:01	1139	95	KNHTSPDVD	KNHTSPDVD	0.000001	95	1.00E-06	95	TRUE	TRUE	
1	KRVDFCGKG	1020	1028	9	HLA-A*02:01	1020	95	KRVDFCGKG	KRVDFCGKG	0.000001	95	1.00E-06	95	TRUE	TRUE	
1	LCCMTSCCS	1216	1224	9	HLA-A*02:01	1216	95	LCCMTSCCS	LCCMTSCCS	0.000001	95	1.00E-06	95	TRUE	TRUE	
1	LDSFKEELD	1127	1135	9	HLA-A*02:01	1127	95	LDSFKEELD	LDSFKEELD	0.000001	95	1.00E-06	95	TRUE	TRUE	
1	LGKLQDVVN	927	935	9	HLA-A*02:01	927	95	LGKLQDVVN	LGKLQDVVN	0.000001	95	1.00E-06	95	TRUE	TRUE	
1	LNCYWPLND	472	480	9	HLA-A*02:01	472	95	LNCYWPLND	LNCYWPLND	0.000001	95	1.00E-06	95	TRUE	TRUE	
1	LNDILSRLD	959	967	9	HLA-A*02:01	959	95	LNDILSRLD	LNDILSRLD	0.000001	95	1.00E-06	95	TRUE	TRUE	
1	LRPFERDIS	448	456	9	HLA-A*02:01	448	95	LRPFERDIS	LRPFERDIS	0.000001	95	1.00E-06	95	TRUE	TRUE	
1	LSGIAAEQD	749	757	9	HLA-A*02:01	749	95	LSGIAAEQD	LSGIAAEQD	0.000001	95	1.00E-06	95	TRUE	TRUE	
1	MKQYGECLG	816	824	9	HLA-A*02:01	816	95	MKQYGECLG	MKQYGECLG	0.000001	95	1.00E-06	95	TRUE	TRUE	
1	MRGVYYPDE	37	45	9	HLA-A*02:01	37	95	MRGVYYPDE	MRGVYYPDE	0.000001	95	1.00E-06	95	TRUE	TRUE	
1	NCTFEYISD	158	166	9	HLA-A*02:01	158	95	NCTFEYISD	NCTFEYISD	0.000001	95	1.00E-06	95	TRUE	TRUE	
1	NFRVVPSGD	304	312	9	HLA-A*02:01	304	95	NFRVVPSGD	NFRVVPSGD	0.000001	95	1.00E-06	95	TRUE	TRUE	
1	NNVFQTQAG	626	634	9	HLA-A*02:01	626	95	NNVFQTQAG	NNVFQTQAG	0.000001	95	1.00E-06	95	TRUE	TRUE	
1	NPVIPFKDG	78	86	9	HLA-A*02:01	78	95	NPVIPFKDG	NPVIPFKDG	0.000001	95	1.00E-06	95	TRUE	TRUE	
1	PDVDLGDIS	1144	1152	9	HLA-A*02:01	1144	95	PDVDLGDIS	PDVDLGDIS	0.000001	95	1.00E-06	95	TRUE	TRUE	
1	PGQTGVIAD	399	407	9	HLA-A*02:01	399	95	PGQTGVIAD	PGQTGVIAD	0.000001	95	1.00E-06	95	TRUE	TRUE	
1	PTKRSFIED	794	802	9	HLA-A*02:01	794	95	PTKRSFIED	PTKRSFIED	0.000001	95	1.00E-06	95	TRUE	TRUE	
1	PTTFMLKYD	259	267	9	HLA-A*02:01	259	95	PTTFMLKYD	PTTFMLKYD	0.000001	95	1.00E-06	95	TRUE	TRUE	
1	QDVNCTDVS	599	607	9	HLA-A*02:01	599	95	QDVNCTDVS	QDVNCTDVS	0.000001	95	1.00E-06	95	TRUE	TRUE	
1	QKEIDRLNE	1162	1170	9	HLA-A*02:01	1162	95	QKEIDRLNE	QKEIDRLNE	0.000001	95	1.00E-06	95	TRUE	TRUE	
1	SPQIITTDN	1093	1101	9	HLA-A*02:01	1093	95	SPQIITTDN	SPQIITTDN	0.000001	95	1.00E-06	95	TRUE	TRUE	
1	TNVVIRACN	121	129	9	HLA-A*02:01	121	95	TNVVIRACN	TNVVIRACN	0.000001	95	1.00E-06	95	TRUE	TRUE	
1	TPTLKYFGG	773	781	9	HLA-A*02:01	773	95	TPTLKYFGG	TPTLKYFGG	0.000001	95	1.00E-06	95	TRUE	TRUE	
1	VDCNMYICG	718	726	9	HLA-A*02:01	718	95	VDCNMYICG	VDCNMYICG	0.000001	95	1.00E-06	95	TRUE	TRUE	
1	VKQLSSNFG	945	953	9	HLA-A*02:01	945	95	VKQLSSNFG	VKQLSSNFG	0.000001	95	1.00E-06	95	TRUE	TRUE	
1	VKSFEIDKG	290	298	9	HLA-A*02:01	290	95	VKSFEIDKG	VKSFEIDKG	0.000001	95	1.00E-06	95	TRUE	TRUE	
1	VNQNAQALN	934	942	9	HLA-A*02:01	934	95	VNQNAQALN	VNQNAQALN	0.000001	95	1.00E-06	95	TRUE	TRUE	
1	YDPLQPELD	1120	1128	9	HLA-A*02:01	1120	95	YDPLQPELD	YDPLQPELD	0.000001	95	1.00E-06	95	TRUE	TRUE	
1	ACNFELCDN	127	135	9	HLA-A*02:01	127	100	ACNFELCDN	ACNFELCDN	0	100	0	100	TRUE	TRUE	
1	AYTMSLGAD	676	684	9	HLA-A*02:01	676	100	AYTMSLGAD	AYTMSLGAD	0	100	0	100	TRUE	TRUE	
1	CCKFDEDDS	1235	1243	9	HLA-A*02:01	1235	100	CCKFDEDDS	CCKFDEDDS	0	100	0	100	TRUE	TRUE	
1	CGDSTECAN	725	733	9	HLA-A*02:01	725	100	CGDSTECAN	CGDSTECAN	0	100	0	100	TRUE	TRUE	
1	CGSCCKFDE	1232	1240	9	HLA-A*02:01	1232	100	CGSCCKFDE	CGSCCKFDE	0	100	0	100	TRUE	TRUE	
1	CNMYICGDS	720	728	9	HLA-A*02:01	720	100	CNMYICGDS	CNMYICGDS	0	100	0	100	TRUE	TRUE	
1	DCNMYICGD	719	727	9	HLA-A*02:01	719	100	DCNMYICGD	DCNMYICGD	0	100	0	100	TRUE	TRUE	
1	DKGIYQTSN	296	304	9	HLA-A*02:01	296	100	DKGIYQTSN	DKGIYQTSN	0	100	0	100	TRUE	TRUE	
1	DSFVVKGDD	385	393	9	HLA-A*02:01	385	100	DSFVVKGDD	DSFVVKGDD	0	100	0	100	TRUE	TRUE	
1	DYNYKLPDD	407	415	9	HLA-A*02:01	407	100	DYNYKLPDD	DYNYKLPDD	0	100	0	100	TRUE	TRUE	
1	GKLRPFERD	446	454	9	HLA-A*02:01	446	100	GKLRPFERD	GKLRPFERD	0	100	0	100	TRUE	TRUE	
1	IKNQCVNFN	520	528	9	HLA-A*02:01	520	100	IKNQCVNFN	IKNQCVNFN	0	100	0	100	TRUE	TRUE	
1	LDRCTTFDD	16	24	9	HLA-A*02:01	16	100	LDRCTTFDD	LDRCTTFDD	0	100	0	100	TRUE	TRUE	
1	LDVSEKSGN	170	178	9	HLA-A*02:01	170	100	LDVSEKSGN	LDVSEKSGN	0	100	0	100	TRUE	TRUE	
1	LKGACSCGS	1226	1234	9	HLA-A*02:01	1226	100	LKGACSCGS	LKGACSCGS	0	100	0	100	TRUE	TRUE	
1	LNEVAKNLN	1168	1176	9	HLA-A*02:01	1168	100	LNEVAKNLN	LNEVAKNLN	0	100	0	100	TRUE	TRUE	
1	NGTITDAVD	269	277	9	HLA-A*02:01	269	100	NGTITDAVD	NGTITDAVD	0	100	0	100	TRUE	TRUE	
1	NKVTLADAG	806	814	9	HLA-A*02:01	806	100	NKVTLADAG	NKVTLADAG	0	100	0	100	TRUE	TRUE	
1	PDEIFRSDT	43	51	9	HLA-A*02:01	43	100	PDEIFRSDT	PDEIFRSDT	0	100	0	100	TRUE	TRUE	
1	PREGVFVFN	1072	1080	9	HLA-A*02:01	1072	100	PREGVFVFN	PREGVFVFN	0	100	0	100	TRUE	TRUE	
1	QKQIANQFN	902	910	9	HLA-A*02:01	902	100	QKQIANQFN	QKQIANQFN	0	100	0	100	TRUE	TRUE	
1	SCCKFDEDD	1234	1242	9	HLA-A*02:01	1234	100	SCCKFDEDD	SCCKFDEDD	0	100	0	100	TRUE	TRUE	
1	SCGSCCKFD	1231	1239	9	HLA-A*02:01	1231	100	SCGSCCKFD	SCGSCCKFD	0	100	0	100	TRUE	TRUE	
1	TDAVDCSQN	273	281	9	HLA-A*02:01	273	100	TDAVDCSQN	TDAVDCSQN	0	100	0	100	TRUE	TRUE	
1	WRIYSTGNN	619	627	9	HLA-A*02:01	619	100	WRIYSTGNN	WRIYSTGNN	0	100	0	100	TRUE	TRUE	
1	YGECLGDIN	819	827	9	HLA-A*02:01	819	100	YGECLGDIN	YGECLGDIN	0	100	0	100	TRUE	TRUE	

seq #	peptide	start	end	peptide length	allele	peptide index	median binding	netmhcpan_ba	netmhcpan_ba	netmhcpan_ba	netmhcpan_ba	legacy score	legacy rank	scores equal	percentiles equal	
1	FIAGLIAIV	1202	1210	9	HLA-A*02:01	1202	0.05	FIAGLIAIV	FIAGLIAIV	6.61	0.05	6.61	0.05	TRUE	TRUE	
1	RLQSLQTYV	982	990	9	HLA-A*02:01	982	0.11	RLQSLQTYV	RLQSLQTYV	11.92	0.11	11.92	0.11	TRUE	TRUE	
1	YLKPTTFML	256	264	9	HLA-A*02:01	256	0.11	YLKPTTFML	YLKPTTFML	12.25	0.11	12.25	0.11	TRUE	TRUE	
1	LLFNKVTLA	803	811	9	HLA-A*02:01	803	0.12	LLFNKVTLA	LLFNKVTLA	12.82	0.12	12.82	0.12	TRUE	TRUE	
1	MIAAYTAAL	851	859	9	HLA-A*02:01	851	0.12	MIAAYTAAL	MIAAYTAAL	13.04	0.12	13.04	0.12	TRUE	TRUE	
1	VVFLHVTYV	1042	1050	9	HLA-A*02:01	1042	0.16	VVFLHVTYV	VVFLHVTYV	17.07	0.16	17.07	0.16	TRUE	TRUE	
1	VLNDILSRL	958	966	9	HLA-A*02:01	958	0.2	VLNDILSRL	VLNDILSRL	22.77	0.2	22.77	0.2	TRUE	TRUE	
1	VIADYNYKL	404	412	9	HLA-A*02:01	404	0.34	VIADYNYKL	VIADYNYKL	38.03	0.34	38.03	0.34	TRUE	TRUE	
1	FSISITTEV	700	708	9	HLA-A*02:01	700	0.36	FSISITTEV	FSISITTEV	39.59	0.36	39.59	0.36	TRUE	TRUE	
1	HLMSFPQAA	1030	1038	9	HLA-A*02:01	1030	0.37	HLMSFPQAA	HLMSFPQAA	41.54	0.37	41.54	0.37	TRUE	TRUE	
1	LLTDDMIAA	846	854	9	HLA-A*02:01	846	0.39	LLTDDMIAA	LLTDDMIAA	44.23	0.39	44.23	0.39	TRUE	TRUE	
1	RLDKVEAEV	965	973	9	HLA-A*02:01	965	0.41	RLDKVEAEV	RLDKVEAEV	46.73	0.41	46.73	0.41	TRUE	TRUE	
1	YVYKGYQPI	195	203	9	HLA-A*02:01	195	0.45	YVYKGYQPI	YVYKGYQPI	54.47	0.45	54.47	0.45	TRUE	TRUE	
1	NVYADSFVV	381	389	9	HLA-A*02:01	381	0.45	NVYADSFVV	NVYADSFVV	55.34	0.45	55.34	0.45	TRUE	TRUE	
1	SIVAYTMSL	673	681	9	HLA-A*02:01	673	0.49	SIVAYTMSL	SIVAYTMSL	61.63	0.49	61.63	0.49	TRUE	TRUE	
1	VLYNSTFFS	354	362	9	HLA-A*02:01	354	0.52	VLYNSTFFS	VLYNSTFFS	64.98	0.52	64.98	0.52	TRUE	TRUE	
1	SITTEVMPV	703	711	9	HLA-A*02:01	703	0.55	SITTEVMPV	SITTEVMPV	70.36	0.55	70.36	0.55	TRUE	TRUE	
1	KQMYKTPTL	768	776	9	HLA-A*02:01	768	0.6	KQMYKTPTL	KQMYKTPTL	78.16	0.6	78.16	0.6	TRUE	TRUE	
1	FLLFLTLTS	4	12	9	HLA-A*02:01	4	0.65	FLLFLTLTS	FLLFLTLTS	85.93	0.65	85.93	0.65	TRUE	TRUE	
1	NLNESLIDL	1174	1182	9	HLA-A*02:01	1174	0.67	NLNESLIDL	NLNESLIDL	89.53	0.67	89.53	0.67	TRUE	TRUE	
1	GLTVLPPLL	839	847	9	HLA-A*02:01	839	0.75	GLTVLPPLL	GLTVLPPLL	100.5	0.75	100.5	0.75	TRUE	TRUE	
1	ALVSGTATA	858	866	9	HLA-A*02:01	858	0.78	ALVSGTATA	ALVSGTATA	104.99	0.78	104.99	0.78	TRUE	TRUE	
1	VLSFELLNA	498	506	9	HLA-A*02:01	498	0.93	VLSFELLNA	VLSFELLNA	133.32	0.93	133.32	0.93	TRUE	TRUE	
1	ATAGWTFGA	864	872	9	HLA-A*02:01	864	1.3	ATAGWTFGA	ATAGWTFGA	184.5	1.3	184.5	1.3	TRUE	TRUE	
1	IIINNSTNV	115	123	9	HLA-A*02:01	115	1.3	IIINNSTNV	IIINNSTNV	187.46	1.3	187.46	1.3	TRUE	TRUE	
1	KLPDDFMGC	411	419	9	HLA-A*02:01	411	1.3	KLPDDFMGC	KLPDDFMGC	196.44	1.3	196.44	1.3	TRUE	TRUE	
1	AILTAFSPA	233	241	9	HLA-A*02:01	233	1.4	AILTAFSPA	AILTAFSPA	204.09	1.4	204.09	1.4	TRUE	TRUE	
1	GTSAAAYFV	246	254	9	HLA-A*02:01	246	1.4	GTSAAAYFV	GTSAAAYFV	211.55	1.4	211.55	1.4	TRUE	TRUE	
1	RLNEVAKNL	1167	1175	9	HLA-A*02:01	1167	1.5	RLNEVAKNL	RLNEVAKNL	246.13	1.5	246.13	1.5	TRUE	TRUE	
1	MIFDNAFNC	151	159	9	HLA-A*02:01	151	1.5	MIFDNAFNC	MIFDNAFNC	250.37	1.5	250.37	1.5	TRUE	TRUE	
1	GLIAIVMVT	1205	1213	9	HLA-A*02:01	1205	1.7	GLIAIVMVT	GLIAIVMVT	297.22	1.7	297.22	1.7	TRUE	TRUE	
1	FVSGNCDVV	1103	1111	9	HLA-A*02:01	1103	1.8	FVSGNCDVV	FVSGNCDVV	318.47	1.8	318.47	1.8	TRUE	TRUE	
1	FIFLLFLTL	2	10	9	HLA-A*02:01	2	1.8	FIFLLFLTL	FIFLLFLTL	329.3	1.8	329.3	1.8	TRUE	TRUE	
1	RVVVLSFEL	495	503	9	HLA-A*02:01	495	1.9	RVVVLSFEL	RVVVLSFEL	343.47	1.9	343.47	1.9	TRUE	TRUE	
1	ELLNAPATV	502	510	9	HLA-A*02:01	502	1.9	ELLNAPATV	ELLNAPATV	351.36	1.9	351.36	1.9	TRUE	TRUE	
1	VLYENQKQI	897	905	9	HLA-A*02:01	897	1.9	VLYENQKQI	VLYENQKQI	364.81	1.9	364.81	1.9	TRUE	TRUE	
1	TQAGCLIGA	631	639	9	HLA-A*02:01	631	2	TQAGCLIGA	TQAGCLIGA	406.44	2	406.44	2	TRUE	TRUE	
1	FLHVTYVPS	1044	1052	9	HLA-A*02:01	1044	2.1	FLHVTYVPS	FLHVTYVPS	433.31	2.1	433.31	2.1	TRUE	TRUE	
1	FMKQYGECL	815	823	9	HLA-A*02:01	815	2.2	FMKQYGECL	FMKQYGECL	455.97	2.2	455.97	2.2	TRUE	TRUE	
1	FSTFKCYGV	361	369	9	HLA-A*02:01	361	2.2	FSTFKCYGV	FSTFKCYGV	457.64	2.2	457.64	2.2	TRUE	TRUE	
1	TMNNKSQSV	106	114	9	HLA-A*02:01	106	2.2	TMNNKSQSV	TMNNKSQSV	466.77	2.2	466.77	2.2	TRUE	TRUE	
1	IITTDNTFV	1096	1104	9	HLA-A*02:01	1096	2.2	IITTDNTFV	IITTDNTFV	476.54	2.2	476.54	2.2	TRUE	TRUE	
1	ELCDNPFFA	131	139	9	HLA-A*02:01	131	2.4	ELCDNPFFA	ELCDNPFFA	548.01	2.4	548.01	2.4	TRUE	TRUE	
1	GICASYHTV	655	663	9	HLA-A*02:01	655	2.4	GICASYHTV	GICASYHTV	549.73	2.4	549.73	2.4	TRUE	TRUE	
1	SLTTTSTAL	919	927	9	HLA-A*02:01	919	2.4	SLTTTSTAL	SLTTTSTAL	551.21	2.4	551.21	2.4	TRUE	TRUE	
1	ALNTLVKQL	940	948	9	HLA-A*02:01	940	2.4	ALNTLVKQL	ALNTLVKQL	563.85	2.4	563.85	2.4	TRUE	TRUE	
1	RIYSTGNNV	620	628	9	HLA-A*02:01	620	2.4	RIYSTGNNV	RIYSTGNNV	576.1	2.4	576.1	2.4	TRUE	TRUE	
1	FLPFYSNVT	59	67	9	HLA-A*02:01	59	2.5	FLPFYSNVT	FLPFYSNVT	612.34	2.5	612.34	2.5	TRUE	TRUE	
1	YLTQDLFLP	53	61	9	HLA-A*02:01	53	2.6	YLTQDLFLP	YLTQDLFLP	661.13	2.6	661.13	2.6	TRUE	TRUE	
1	YQTSNFRVV	300	308	9	HLA-A*02:01	300	2.7	YQTSNFRVV	YQTSNFRVV	677.82	2.7	677.82	2.7	TRUE	TRUE	
1	WTFGAGAAL	868	876	9	HLA-A*02:01	868	2.7	WTFGAGAAL	WTFGAGAAL	708.79	2.7	708.79	2.7	TRUE	TRUE	
1	YQDVNCTDV	598	606	9	HLA-A*02:01	598	2.7	YQDVNCTDV	YQDVNCTDV	710.25	2.7	710.25	2.7	TRUE	TRUE	
1	KQLSSNFGA	946	954	9	HLA-A*02:01	946	2.8	KQLSSNFGA	KQLSSNFGA	748.39	2.8	748.39	2.8	TRUE	TRUE	
1	LIAIVMVTI	1206	1214	9	HLA-A*02:01	1206	2.8	LIAIVMVTI	LIAIVMVTI	769.87	2.8	769.87	2.8	TRUE	TRUE	
1	WLGFIAGLI	1199	1207	9	HLA-A*02:01	1199	2.9	WLGFIAGLI	WLGFIAGLI	807.56	2.9	807.56	2.9	TRUE	TRUE	
1	QLNRALSGI	744	752	9	HLA-A*02:01	744	2.9	QLNRALSGI	QLNRALSGI	814.15	2.9	814.15	2.9	TRUE	TRUE	
1	QYIKWPWYV	1190	1198	9	HLA-A*02:01	1190	2.9	QYIKWPWYV	QYIKWPWYV	822.82	2.9	822.82	2.9	TRUE	TRUE	
1	ALGKLQDVV	926	934	9	HLA-A*02:01	926	3	ALGKLQDVV	ALGKLQDVV	852.18	3	852.18	3	TRUE	TRUE	
1	LQIPFAMQM	876	884	9	HLA-A*02:01	876	3	LQIPFAMQM	LQIPFAMQM	863.81	3	863.81	3	TRUE	TRUE	
1	CTFEYISDA	159	167	9	HLA-A*02:01	159	3	CTFEYISDA	CTFEYISDA	872.69	3	872.69	3	TRUE	TRUE	
1	VIGIINNTV	1111	1119	9	HLA-A*02:01	1111	3.1	VIGIINNTV	VIGIINNTV	898.53	3.1	898.53	3.1	TRUE	TRUE	
1	LLQYGSFCT	735	743	9	HLA-A*02:01	735	3.1	LLQYGSFCT	LLQYGSFCT	923.23	3.1	923.23	3.1	TRUE	TRUE	
1	QMAYRFNGI	883	891	9	HLA-A*02:01	883	3.1	QMAYRFNGI	QMAYRFNGI	940.57	3.1	940.57	3.1	TRUE	TRUE	
1	ILLCCMTSC	1214	1222	9	HLA-A*02:01	1214	3.2	ILLCCMTSC	ILLCCMTSC	987.81	3.2	987.81	3.2	TRUE	TRUE	
1	VIRACNFEL	124	132	9	HLA-A*02:01	124	3.3	VIRACNFEL	VIRACNFEL	1032.71	3.3	1032.71	3.3	TRUE	TRUE	
1	PLAELKCSV	282	290	9	HLA-A*02:01	282	3.4	PLAELKCSV	PLAELKCSV	1068.17	3.4	1068.17	3.4	TRUE	TRUE	
1	IAIPTNFSI	694	702	9	HLA-A*02:01	694	3.4	IAIPTNFSI	IAIPTNFSI	1094.71	3.4	1094.71	3.4	TRUE	TRUE	
1	QIAPGQTGV	396	404	9	HLA-A*02:01	396	3.6	QIAPGQTGV	QIAPGQTGV	1188.85	3.6	1188.85	3.6	TRUE	TRUE	
1	VLAWNTRNI	420	428	9	HLA-A*02:01	420	3.6	VLAWNTRNI	VLAWNTRNI	1216.08	3.6	1216.08	3.6	TRUE	TRUE	
1	IAAYTAALV	852	860	9	HLA-A*02:01	852	3.6	IAAYTAALV	IAAYTAALV	1224.93	3.6	1224.93	3.6	TRUE	TRUE	
1	FQTQAGCLI	629	637	9	HLA-A*02:01	629	3.7	FQTQAGCLI	FQTQAGCLI	1308.97	3.7	1308.97	3.7	TRUE	TRUE	
1	VLYQDVNCT	596	604	9	HLA-A*02:01	596	3.8	VLYQDVNCT	VLYQDVNCT	1342.97	3.8	1342.97	3.8	TRUE	TRUE	
1	CMTSCCSCL	1218	1226	9	HLA-A*02:01	1218	3.8	CMTSCCSCL	CMTSCCSCL	1350.56	3.8	1350.56	3.8	TRUE	TRUE	
1	FVFNGTSWF	1077	1085	9	HLA-A*02:01	1077	3.8	FVFNGTSWF	FVFNGTSWF	1352.2	3.8	1352.2	3.8	TRUE	TRUE	
1	YQPYRVVVL	491	499	9	HLA-A*02:01	491	3.8	YQPYRVVVL	YQPYRVVVL	1377.75	3.8	1377.75	3.8	TRUE	TRUE	
1	GINASVVNI	1153	1161	9	HLA-A*02:01	1153	3.8	GINASVVNI	GINASVVNI	1383.16	3.8	1383.16	3.8	TRUE	TRUE	
1	AISSVLNDI	954	962	9	HLA-A*02:01	954	3.9	AISSVLNDI	AISSVLNDI	1427.57	3.9	1427.57	3.9	TRUE	TRUE	
1	AIVMVTILL	1208	1216	9	HLA-A*02:01	1208	3.9	AIVMVTILL	AIVMVTILL	1428.93	3.9	1428.93	3.9	TRUE	TRUE	
1	LFLPFYSNV	58	66	9	HLA-A*02:01	58	3.9	LFLPFYSNV	LFLPFYSNV	1450.41	3.9	1450.41	3.9	TRUE	TRUE	
1	LLFLTLTSG	5	13	9	HLA-A*02:01	5	4	LLFLTLTSG	LLFLTLTSG	1492.18	4	1492.18	4	TRUE	TRUE	
1	ILPDPLKPT	787	795	9	HLA-A*02:01	787	4	ILPDPLKPT	ILPDPLKPT	1503.81	4	1503.81	4	TRUE	TRUE	
1	QLSSNFGAI	947	955	9	HLA-A*02:01	947	4.1	QLSSNFGAI	QLSSNFGAI	1550.44	4.1	1550.44	4.1	TRUE	TRUE	
1	GIGYQPYRV	488	496	9	HLA-A*02:01	488	4.4	GIGYQPYRV	GIGYQPYRV	1759.77	4.4	1759.77	4.4	TRUE	TRUE	
1	FNATKFPSV	329	337	9	HLA-A*02:01	329	4.5	FNATKFPSV	FNATKFPSV	1852.82	4.5	1852.82	4.5	TRUE	TRUE	
1	TSYECDIPI	644	652	9	HLA-A*02:01	644	4.6	TSYECDIPI	TSYECDIPI	1978.76	4.6	1978.76	4.6	TRUE	TRUE	
1	VWLGFIAGL	1198	1206	9	HLA-A*02:01	1198	4.8	VWLGFIAGL	VWLGFIAGL	2098.93	4.8	2098.93	4.8	TRUE	TRUE	
1	NTLKPIFKL	214	222	9	HLA-A*02:01	214	4.9	NTLKPIFKL	NTLKPIFKL	2170.48	4.9	2170.48	4.9	TRUE	TRUE	
1	IINNSTNVV	116	124	9	HLA-A*02:01	116	4.9	IINNSTNVV	IINNSTNVV	2188.19	4.9	2188.19	4.9	TRUE	TRUE	
1	ISDAFSLDV	164	172	9	HLA-A*02:01	164	4.9	ISDAFSLDV	ISDAFSLDV	2242.89	4.9	2242.89	4.9	TRUE	TRUE	
1	LCDNPFFAV	132	140	9	HLA-A*02:01	132	5	LCDNPFFAV	LCDNPFFAV	2301.07	5	2301.07	5	TRUE	TRUE	
1	YGFYTTTGI	481	489	9	HLA-A*02:01	481	5.1	YGFYTTTGI	YGFYTTTGI	2335.89	5.1	2335.89	5.1	TRUE	TRUE	
1	LLLQYGSFC	734	742	9	HLA-A*02:01	734	5.2	LLLQYGSFC	LLLQYGSFC	2449.25	5.2	2449.25	5.2	TRUE	TRUE	
1	FMLKYDENG	262	270	9	HLA-A*02:01	262	5.2	FMLKYDENG	FMLKYDENG	2495.1	5.2	2495.1	5.2	TRUE	TRUE	
1	VFNGTSWFI	1078	1086	9	HLA-A*02:01	1078	5.5	VFNGTSWFI	VFNGTSWFI	2676.49	5.5	2676.49	5.5	TRUE	TRUE	
1	AISQIQESL	912	920	9	HLA-A*02:01	912	5.5	AISQIQESL	AISQIQESL	2724.58	5.5	2724.58	5.5	TRUE	TRUE	
1	VVVLSFELL	496	504	9	HLA-A*02:01	496	5.5	VVVLSFELL	VVVLSFELL	2734.33	5.5	2734.33	5.5	TRUE	TRUE	
1	AAAYFVGYL	249	257	9	HLA-A*02:01	249	5.6	AAAYFVGYL	AAAYFVGYL	2777.2	5.6	2777.2	5.6	TRUE	TRUE	
1	MQMAYRFNG	882	890	9	HLA-A*02:01	882	5.8	MQMAYRFNG	MQMAYRFNG	2928.7	5.8	2928.7	5.8	TRUE	TRUE	
1	TSVDCNMYI	716	724	9	HLA-A*02:01	716	6	TSVDCNMYI	TSVDCNMYI	3110.96	6	3110.96	6	TRUE	TRUE	
1	LQTYVTQQL	986	994	9	HLA-A*02:01	986	6	LQTYVTQQL	LQTYVTQQL	3143.75	6	3143.75	6	TRUE	TRUE	
1	PLNDYGFYT	477	485	9	HLA-A*02:01	477	6.1	PLNDYGFYT	PLNDYGFYT	3155.78	6.1	3155.78	6.1	TRUE	TRUE	
1	YVTQQLIRA	989	997	9	HLA-A*02:01	989	6.2	YVTQQLIRA	YVTQQLIRA	3246.41	6.2	3246.41	6.2	TRUE	TRUE	
1	AVDCSQNPL	275	283	9	HLA-A*02:01	275	6.2	AVDCSQNPL	AVDCSQNPL	3262.72	6.2	3262.72	6.2	TRUE	TRUE	
1	ILDISPCSF	570	578	9	HLA-A*02:01	570	6.3	ILDISPCSF	ILDISPCSF	3316.03	6.3	3316.03	6.3	TRUE	TRUE	
1	KEIDRLNEV	1163	1171	9	HLA-A*02:01	1163	6.3	KEIDRLNEV	KEIDRLNEV	3378.69	6.3	3378.69	6.3	TRUE	TRUE	
1	LITGRLQSL	978	986	9	HLA-A*02:01	978	6.4	LITGRLQSL	LITGRLQSL	3501.5	6.4	3501.5	6.4	TRUE	TRUE	
1	SLIDLQELG	1178	1186	9	HLA-A*02:01	1178	6.4	SLIDLQELG	SLIDLQELG	3513	6.4	3513	6.4	TRUE	TRUE	
1	SIAYSNNTI	686	694	9	HLA-A*02:01	686	6.6	SIAYSNNTI	SIAYSNNTI	3618.71	6.6	3618.71	6.6	TRUE	TRUE	
1	GFIAGLIAI	1201	1209	9	HLA-A*02:01	1201	6.6	GFIAGLIAI	GFIAGLIAI	3674.42	6.6	3674.42	6.6	TRUE	TRUE	
1	YTAALVSGT	855	863	9	HLA-A*02:01	855	6.7	YTAALVSGT	YTAALVSGT	3742.95	6.7	3742.95	6.7	TRUE	TRUE	
1	SNFGAISSV	950	958	9	HLA-A*02:01	950	7	SNFGAISSV	SNFGAISSV	4049.61	7	4049.61	7	TRUE	TRUE	
1	CVNFNFNGL	524	532	9	HLA-A*02:01	524	7	CVNFNFNGL	CVNFNFNGL	4051.89	7	4051.89	7	TRUE	TRUE	
1	LMSFPQAAP	1031	1039	9	HLA-A*02:01	1031	7.2	LMSFPQAAP	LMSFPQAAP	4245.04	7.2	4245.04	7.2	TRUE	TRUE	
1	NTREVFAQV	759	767	9	HLA-A*02:01	759	7.2	NTREVFAQV	NTREVFAQV	4251.16	7.2	4251.16	7.2	TRUE	TRUE	
1	CSFGGVSVI	576	584	9	HLA-A*02:01	576	7.3	CSFGGVSVI	CSFGGVSVI	4284.77	7.3	4284.77	7.3	TRUE	TRUE	

seq #	peptide	start	end	peptide length	allele	peptide index	median binding	netmhcpan_ba	netmhcpan_ba	netmhcpan_ba	netmhcpan_ba	legacy score	legacy rank	scores equal	percentiles equal	
1	ISPCSFGGV	573	581	9	HLA-A*02:01	573	7.3	ISPCSFGGV	ISPCSFGGV	4352.57	7.3	4352.57	7.3	TRUE	TRUE	
1	DLCFSNVYA	376	384	9	HLA-A*02:01	376	7.3	DLCFSNVYA	DLCFSNVYA	4367.91	7.3	4367.91	7.3	TRUE	TRUE	
1	KMSECVLGQ	1010	1018	9	HLA-A*02:01	1010	7.4	KMSECVLGQ	KMSECVLGQ	4470.9	7.4	4470.9	7.4	TRUE	TRUE	
1	DMIAAYTAA	850	858	9	HLA-A*02:01	850	7.5	DMIAAYTAA	DMIAAYTAA	4515.92	7.5	4515.92	7.5	TRUE	TRUE	
1	KAYFPREGV	1068	1076	9	HLA-A*02:01	1068	7.6	KAYFPREGV	KAYFPREGV	4638.28	7.6	4638.28	7.6	TRUE	TRUE	
1	IVMVTILLC	1209	1217	9	HLA-A*02:01	1209	7.7	IVMVTILLC	IVMVTILLC	4737.95	7.7	4737.95	7.7	TRUE	TRUE	
1	FLYVYKGYQ	193	201	9	HLA-A*02:01	193	7.7	FLYVYKGYQ	FLYVYKGYQ	4746.52	7.7	4746.52	7.7	TRUE	TRUE	
1	ELDSFKEEL	1126	1134	9	HLA-A*02:01	1126	7.8	ELDSFKEEL	ELDSFKEEL	4841.91	7.8	4841.91	7.8	TRUE	TRUE	
1	VVIGIINNT	1110	1118	9	HLA-A*02:01	1110	7.9	VVIGIINNT	VVIGIINNT	4928.44	7.9	4928.44	7.9	TRUE	TRUE	
1	NMYICGDST	721	729	9	HLA-A*02:01	721	7.9	NMYICGDST	NMYICGDST	4931.32	7.9	4931.32	7.9	TRUE	TRUE	
1	TLYLTQDLF	51	59	9	HLA-A*02:01	51	8	TLYLTQDLF	TLYLTQDLF	5010.7	8	5010.7	8	TRUE	TRUE	
1	FAMQMAYRF	880	888	9	HLA-A*02:01	880	8	FAMQMAYRF	FAMQMAYRF	5026.34	8	5026.34	8	TRUE	TRUE	
1	ELGKYEQYI	1184	1192	9	HLA-A*02:01	1184	8	ELGKYEQYI	ELGKYEQYI	5036.14	8	5036.14	8	TRUE	TRUE	
1	AALQIPFAM	874	882	9	HLA-A*02:01	874	8.1	AALQIPFAM	AALQIPFAM	5112.62	8.1	5112.62	8.1	TRUE	TRUE	
1	AQKFNGLTV	834	842	9	HLA-A*02:01	834	8.3	AQKFNGLTV	AQKFNGLTV	5307.3	8.3	5307.3	8.3	TRUE	TRUE	
1	FSQILPDPL	784	792	9	HLA-A*02:01	784	8.3	FSQILPDPL	FSQILPDPL	5325.82	8.3	5325.82	8.3	TRUE	TRUE	
1	VAYTMSLGA	675	683	9	HLA-A*02:01	675	8.4	VAYTMSLGA	VAYTMSLGA	5365.38	8.4	5365.38	8.4	TRUE	TRUE	
1	MSLGADSSI	679	687	9	HLA-A*02:01	679	8.4	MSLGADSSI	MSLGADSSI	5403.54	8.4	5403.54	8.4	TRUE	TRUE	
1	FITQRNFFS	1085	1093	9	HLA-A*02:01	1085	8.6	FITQRNFFS	FITQRNFFS	5549.72	8.6	5549.72	8.6	TRUE	TRUE	
1	VMVTILLCC	1210	1218	9	HLA-A*02:01	1210	8.6	VMVTILLCC	VMVTILLCC	5594.33	8.6	5594.33	8.6	TRUE	TRUE	
1	ASYHTVSLL	658	666	9	HLA-A*02:01	658	8.6	ASYHTVSLL	ASYHTVSLL	5630.58	8.6	5630.58	8.6	TRUE	TRUE	
1	STFFSTFKC	358	366	9	HLA-A*02:01	358	8.8	STFFSTFKC	STFFSTFKC	5766.41	8.8	5766.41	8.8	TRUE	TRUE	
1	TILLCCMTS	1213	1221	9	HLA-A*02:01	1213	8.8	TILLCCMTS	TILLCCMTS	5820.31	8.8	5820.31	8.8	TRUE	TRUE	
1	SMAKTSVDC	712	720	9	HLA-A*02:01	712	8.8	SMAKTSVDC	SMAKTSVDC	5868.69	8.8	5868.69	8.8	TRUE	TRUE	
1	DISGINASV	1150	1158	9	HLA-A*02:01	1150	8.9	DISGINASV	DISGINASV	5938.95	8.9	5938.95	8.9	TRUE	TRUE	
1	AQDIWGTSA	241	249	9	HLA-A*02:01	241	8.9	AQDIWGTSA	AQDIWGTSA	5959.81	8.9	5959.81	8.9	TRUE	TRUE	
1	KLPLGINIT	221	229	9	HLA-A*02:01	221	9	KLPLGINIT	KLPLGINIT	5997.07	9	5997.07	9	TRUE	TRUE	
1	FGAGAALQI	870	878	9	HLA-A*02:01	870	9.1	FGAGAALQI	FGAGAALQI	6158.5	9.1	6158.5	9.1	TRUE	TRUE	
1	TALGKLQDV	925	933	9	HLA-A*02:01	925	9.1	TALGKLQDV	TALGKLQDV	6173.38	9.1	6173.38	9.1	TRUE	TRUE	
1	SLGADSSIA	680	688	9	HLA-A*02:01	680	9.2	SLGADSSIA	SLGADSSIA	6213.05	9.2	6213.05	9.2	TRUE	TRUE	
1	GAALQIPFA	873	881	9	HLA-A*02:01	873	9.2	GAALQIPFA	GAALQIPFA	6246.55	9.2	6246.55	9.2	TRUE	TRUE	
1	GLTGTGVLT	531	539	9	HLA-A*02:01	531	9.2	GLTGTGVLT	GLTGTGVLT	6267.06	9.2	6267.06	9.2	TRUE	TRUE	
1	DVSDFTDSV	554	562	9	HLA-A*02:01	554	9.3	DVSDFTDSV	DVSDFTDSV	6333.11	9.3	6333.11	9.3	TRUE	TRUE	
1	NGLTVLPPL	838	846	9	HLA-A*02:01	838	9.4	NGLTVLPPL	NGLTVLPPL	6384.78	9.4	6384.78	9.4	TRUE	TRUE	
1	FMGCVLAWN	416	424	9	HLA-A*02:01	416	9.4	FMGCVLAWN	FMGCVLAWN	6390.24	9.4	6390.24	9.4	TRUE	TRUE	
1	IKWPWYVWL	1192	1200	9	HLA-A*02:01	1192	9.4	IKWPWYVWL	IKWPWYVWL	6463.74	9.4	6463.74	9.4	TRUE	TRUE	
1	VMPVSMAKT	708	716	9	HLA-A*02:01	708	9.5	VMPVSMAKT	VMPVSMAKT	6536.25	9.5	6536.25	9.5	TRUE	TRUE	
1	RVVPSGDVV	306	314	9	HLA-A*02:01	306	9.6	RVVPSGDVV	RVVPSGDVV	6611.64	9.6	6611.64	9.6	TRUE	TRUE	
1	PIFKLPLGI	218	226	9	HLA-A*02:01	218	9.6	PIFKLPLGI	PIFKLPLGI	6634.15	9.6	6634.15	9.6	TRUE	TRUE	
1	DLLFNKVTL	802	810	9	HLA-A*02:01	802	9.6	DLLFNKVTL	DLLFNKVTL	6653.19	9.6	6653.19	9.6	TRUE	TRUE	
1	AGLIAIVMV	1204	1212	9	HLA-A*02:01	1204	9.7	AGLIAIVMV	AGLIAIVMV	6691.09	9.7	6691.09	9.7	TRUE	TRUE	
1	PALNCYWPL	470	478	9	HLA-A*02:01	470	9.8	PALNCYWPL	PALNCYWPL	6821.95	9.8	6821.95	9.8	TRUE	TRUE	
1	YFPREGVFV	1070	1078	9	HLA-A*02:01	1070	9.9	YFPREGVFV	YFPREGVFV	6891.24	9.9	6891.24	9.9	TRUE	TRUE	
1	KLNDLCFSN	373	381	9	HLA-A*02:01	373	9.9	KLNDLCFSN	KLNDLCFSN	6928.92	9.9	6928.92	9.9	TRUE	TRUE	
1	FKDGIYFAA	83	91	9	HLA-A*02:01	83	10	FKDGIYFAA	FKDGIYFAA	7024.86	10	7024.86	10	TRUE	TRUE	
1	AAPHGVVFL	1037	1045	9	HLA-A*02:01	1037	10	AAPHGVVFL	AAPHGVVFL	7046.79	10	7046.79	10	TRUE	TRUE	
1	YVWLGFIAG	1197	1205	9	HLA-A*02:01	1197	11	YVWLGFIAG	YVWLGFIAG	7168.44	11	7168.44	11	TRUE	TRUE	
1	LLNAPATVC	503	511	9	HLA-A*02:01	503	11	LLNAPATVC	LLNAPATVC	7191.13	11	7191.13	11	TRUE	TRUE	
1	LLCCMTSCC	1215	1223	9	HLA-A*02:01	1215	12	LLCCMTSCC	LLCCMTSCC	7284.95	12	7284.95	12	TRUE	TRUE	
1	AFNCTFEYI	156	164	9	HLA-A*02:01	156	12	AFNCTFEYI	AFNCTFEYI	7296.23	12	7296.23	12	TRUE	TRUE	
1	MVTILLCCM	1211	1219	9	HLA-A*02:01	1211	12	MVTILLCCM	MVTILLCCM	7318.68	12	7318.68	12	TRUE	TRUE	
1	DLPSGFNTL	208	216	9	HLA-A*02:01	208	12	DLPSGFNTL	DLPSGFNTL	7352.1	12	7352.1	12	TRUE	TRUE	
1	YFVGYLKPT	252	260	9	HLA-A*02:01	252	12	YFVGYLKPT	YFVGYLKPT	7371.85	12	7371.85	12	TRUE	TRUE	
1	QQLIRAAEI	992	1000	9	HLA-A*02:01	992	12	QQLIRAAEI	QQLIRAAEI	7573.49	12	7573.49	12	TRUE	TRUE	
1	NITNFRAIL	227	235	9	HLA-A*02:01	227	12	NITNFRAIL	NITNFRAIL	7631.89	12	7631.89	12	TRUE	TRUE	
1	FGGFNFSQI	779	787	9	HLA-A*02:01	779	12	FGGFNFSQI	FGGFNFSQI	7726.02	12	7726.02	12	TRUE	TRUE	
1	MFIFLLFLT	1	9	9	HLA-A*02:01	1	12	MFIFLLFLT	MFIFLLFLT	7839.28	12	7839.28	12	TRUE	TRUE	
1	DILSRLDKV	961	969	9	HLA-A*02:01	961	12	DILSRLDKV	DILSRLDKV	7976.17	12	7976.17	12	TRUE	TRUE	
1	FFSPQIITT	1091	1099	9	HLA-A*02:01	1091	12	FFSPQIITT	FFSPQIITT	8000.2	12	8000.2	12	TRUE	TRUE	
1	STECANLLL	728	736	9	HLA-A*02:01	728	12	STECANLLL	STECANLLL	8119.32	12	8119.32	12	TRUE	TRUE	
1	SVDCNMYIC	717	725	9	HLA-A*02:01	717	12	SVDCNMYIC	SVDCNMYIC	8134.97	12	8134.97	12	TRUE	TRUE	
1	RLITGRLQS	977	985	9	HLA-A*02:01	977	12	RLITGRLQS	RLITGRLQS	8148.54	12	8148.54	12	TRUE	TRUE	
1	IAIVMVTIL	1207	1215	9	HLA-A*02:01	1207	12	IAIVMVTIL	IAIVMVTIL	8216.62	12	8216.62	12	TRUE	TRUE	
1	SQKSIVAYT	670	678	9	HLA-A*02:01	670	13	SQKSIVAYT	SQKSIVAYT	8384.2	13	8384.2	13	TRUE	TRUE	
1	AGWTFGAGA	866	874	9	HLA-A*02:01	866	13	AGWTFGAGA	AGWTFGAGA	8494.04	13	8494.04	13	TRUE	TRUE	
1	SGFNTLKPI	211	219	9	HLA-A*02:01	211	13	SGFNTLKPI	SGFNTLKPI	8610.63	13	8610.63	13	TRUE	TRUE	
1	VYDPLQPEL	1119	1127	9	HLA-A*02:01	1119	13	VYDPLQPEL	VYDPLQPEL	8641.52	13	8641.52	13	TRUE	TRUE	
1	NAQALNTLV	937	945	9	HLA-A*02:01	937	13	NAQALNTLV	NAQALNTLV	8675.62	13	8675.62	13	TRUE	TRUE	
1	GINITNFRA	225	233	9	HLA-A*02:01	225	13	GINITNFRA	GINITNFRA	8781.02	13	8781.02	13	TRUE	TRUE	
1	FAATEKSNV	89	97	9	HLA-A*02:01	89	13	FAATEKSNV	FAATEKSNV	8805.75	13	8805.75	13	TRUE	TRUE	
1	AQVKQMYKT	765	773	9	HLA-A*02:01	765	13	AQVKQMYKT	AQVKQMYKT	8848.54	13	8848.54	13	TRUE	TRUE	
1	ILTAFSPAQ	234	242	9	HLA-A*02:01	234	13	ILTAFSPAQ	ILTAFSPAQ	8922.57	13	8922.57	13	TRUE	TRUE	
1	KLRPFERDI	447	455	9	HLA-A*02:01	447	13	KLRPFERDI	KLRPFERDI	9199.21	13	9199.21	13	TRUE	TRUE	
1	CASYHTVSL	657	665	9	HLA-A*02:01	657	13	CASYHTVSL	CASYHTVSL	9279.58	13	9279.58	13	TRUE	TRUE	
1	DIWGTSAAA	243	251	9	HLA-A*02:01	243	13	DIWGTSAAA	DIWGTSAAA	9319.12	13	9319.12	13	TRUE	TRUE	
1	LTQDLFLPF	54	62	9	HLA-A*02:01	54	14	LTQDLFLPF	LTQDLFLPF	9745.21	14	9745.21	14	TRUE	TRUE	
1	ALSGIAAEQ	748	756	9	HLA-A*02:01	748	14	ALSGIAAEQ	ALSGIAAEQ	9757.55	14	9757.55	14	TRUE	TRUE	
1	KNKDGFLYV	188	196	9	HLA-A*02:01	188	14	KNKDGFLYV	KNKDGFLYV	9764.1	14	9764.1	14	TRUE	TRUE	
1	FPQAAPHGV	1034	1042	9	HLA-A*02:01	1034	14	FPQAAPHGV	FPQAAPHGV	9786.84	14	9786.84	14	TRUE	TRUE	
1	VTILLCCMT	1212	1220	9	HLA-A*02:01	1212	14	VTILLCCMT	VTILLCCMT	9874.17	14	9874.17	14	TRUE	TRUE	
1	RAAEIRASA	996	1004	9	HLA-A*02:01	996	14	RAAEIRASA	RAAEIRASA	10163.25	14	10163.25	14	TRUE	TRUE	
1	SISITTEVM	701	709	9	HLA-A*02:01	701	14	SISITTEVM	SISITTEVM	10259.37	14	10259.37	14	TRUE	TRUE	
1	KSFEIDKGI	291	299	9	HLA-A*02:01	291	14	KSFEIDKGI	KSFEIDKGI	10269.7	14	10269.7	14	TRUE	TRUE	
1	CVLGQSKRV	1014	1022	9	HLA-A*02:01	1014	14	CVLGQSKRV	CVLGQSKRV	10377.49	14	10377.49	14	TRUE	TRUE	
1	SMRGVYYPD	36	44	9	HLA-A*02:01	36	15	SMRGVYYPD	SMRGVYYPD	10476.88	15	10476.88	15	TRUE	TRUE	
1	CSQNPLAEL	278	286	9	HLA-A*02:01	278	15	CSQNPLAEL	CSQNPLAEL	10479.03	15	10479.03	15	TRUE	TRUE	
1	SQIQESLTT	914	922	9	HLA-A*02:01	914	15	SQIQESLTT	SQIQESLTT	10557.45	15	10557.45	15	TRUE	TRUE	
1	LQDVVNQNA	930	938	9	HLA-A*02:01	930	15	LQDVVNQNA	LQDVVNQNA	10579.06	15	10579.06	15	TRUE	TRUE	
1	VTLADAGFM	808	816	9	HLA-A*02:01	808	15	VTLADAGFM	VTLADAGFM	10636.45	15	10636.45	15	TRUE	TRUE	
1	YISDAFSLD	163	171	9	HLA-A*02:01	163	15	YISDAFSLD	YISDAFSLD	10641.05	15	10641.05	15	TRUE	TRUE	
1	YICGDSTEC	723	731	9	HLA-A*02:01	723	15	YICGDSTEC	YICGDSTEC	10656.61	15	10656.61	15	TRUE	TRUE	
1	LNDLCFSNV	374	382	9	HLA-A*02:01	374	15	LNDLCFSNV	LNDLCFSNV	10726.94	15	10726.94	15	TRUE	TRUE	
1	QTYVTQQLI	987	995	9	HLA-A*02:01	987	15	QTYVTQQLI	QTYVTQQLI	10771.36	15	10771.36	15	TRUE	TRUE	
1	NGIGVTQNV	889	897	9	HLA-A*02:01	889	15	NGIGVTQNV	NGIGVTQNV	10777.43	15	10777.43	15	TRUE	TRUE	
1	QIPFAMQMA	877	885	9	HLA-A*02:01	877	15	QIPFAMQMA	QIPFAMQMA	10804.17	15	10804.17	15	TRUE	TRUE	
1	SVITPGTNA	582	590	9	HLA-A*02:01	582	15	SVITPGTNA	SVITPGTNA	10976.31	15	10976.31	15	TRUE	TRUE	
1	SVLYNSTFF	353	361	9	HLA-A*02:01	353	15	SVLYNSTFF	SVLYNSTFF	11089.96	15	11089.96	15	TRUE	TRUE	
1	KGYQPIDVV	198	206	9	HLA-A*02:01	198	15	KGYQPIDVV	KGYQPIDVV	11112.3	15	11112.3	15	TRUE	TRUE	
1	FNFNGLTGT	527	535	9	HLA-A*02:01	527	15	FNFNGLTGT	FNFNGLTGT	11156.39	15	11156.39	15	TRUE	TRUE	
1	SQERNFTTA	1052	1060	9	HLA-A*02:01	1052	15	SQERNFTTA	SQERNFTTA	11314.54	15	11314.54	15	TRUE	TRUE	
1	DQLTPAWRI	613	621	9	HLA-A*02:01	613	15	DQLTPAWRI	DQLTPAWRI	11393.9	15	11393.9	15	TRUE	TRUE	
1	FAVSKPMGT	138	146	9	HLA-A*02:01	138	16	FAVSKPMGT	FAVSKPMGT	11498.55	16	11498.55	16	TRUE	TRUE	
1	LYLTQDLFL	52	60	9	HLA-A*02:01	52	16	LYLTQDLFL	LYLTQDLFL	11560.55	16	11560.55	16	TRUE	TRUE	
1	NLCPFGEVF	321	329	9	HLA-A*02:01	321	16	NLCPFGEVF	NLCPFGEVF	11777.96	16	11777.96	16	TRUE	TRUE	
1	SVVNIQKEI	1157	1165	9	HLA-A*02:01	1157	16	SVVNIQKEI	SVVNIQKEI	11860.96	16	11860.96	16	TRUE	TRUE	
1	GNCDVVIGI	1106	1114	9	HLA-A*02:01	1106	16	GNCDVVIGI	GNCDVVIGI	11918.98	16	11918.98	16	TRUE	TRUE	
1	NAFNCTFEY	155	163	9	HLA-A*02:01	155	16	NAFNCTFEY	NAFNCTFEY	12150.62	16	12150.62	16	TRUE	TRUE	
1	ISSVLNDIL	955	963	9	HLA-A*02:01	955	16	ISSVLNDIL	ISSVLNDIL	12208.08	16	12208.08	16	TRUE	TRUE	
1	QIANQFNKA	904	912	9	HLA-A*02:01	904	16	QIANQFNKA	QIANQFNKA	12290.51	16	12290.51	16	TRUE	TRUE	
1	GGFNFSQIL	780	788	9	HLA-A*02:01	780	17	GGFNFSQIL	GGFNFSQIL	12506.62	17	12506.62	17	TRUE	TRUE	
1	SSMRGVYYP	35	43	9	HLA-A*02:01	35	17	SSMRGVYYP	SSMRGVYYP	12618.08	17	12618.08	17	TRUE	TRUE	
1	CVADYSVLY	348	356	9	HLA-A*02:01	348	17	CVADYSVLY	CVADYSVLY	12629.28	17	12629.28	17	TRUE	TRUE	
1	PLLTDDMIA	845	853	9	HLA-A*02:01	845	17	PLLTDDMIA	PLLTDDMIA	12645	17	12645	17	TRUE	TRUE	
1	LPDDFMGCV	412	420	9	HLA-A*02:01	412	17	LPDDFMGCV	LPDDFMGCV	12646.92	17	12646.92	17	TRUE	TRUE	
1	TMIFDNAFN	150	158	9	HLA-A*02:01	150	17	TMIFDNAFN	TMIFDNAFN	12647.6	17	12647.6	17	TRUE	TRUE	

seq #	peptide	start	end	peptide length	allele	peptide index	median binding	netmhcpan_ba	netmhcpan_ba	netmhcpan_ba	netmhcpan_ba	legacy score	legacy rank	scores equal	percentiles equal
1	ATKFPSVYA	331	339	9	HLA-A*02:01	331	17	ATKFPSVYA	ATKFPSVYA	12653.9	17	12653.9	17	TRUE	TRUE
1	RNFTTAPAI	1055	1063	9	HLA-A*02:01	1055	17	RNFTTAPAI	RNFTTAPAI	12663.49	17	12663.49	17	TRUE	TRUE
1	IFRSDTLYL	46	54	9	HLA-A*02:01	46	17	IFRSDTLYL	IFRSDTLYL	12720.89	17	12720.89	17	TRUE	TRUE
1	QIQESLTTT	915	923	9	HLA-A*02:01	915	17	QIQESLTTT	QIQESLTTT	12731.49	17	12731.49	17	TRUE	TRUE
1	VVRGWVFGS	97	105	9	HLA-A*02:01	97	17	VVRGWVFGS	VVRGWVFGS	12756.03	17	12756.03	17	TRUE	TRUE
1	RGWVFGSTM	99	107	9	HLA-A*02:01	99	17	RGWVFGSTM	RGWVFGSTM	12857.05	17	12857.05	17	TRUE	TRUE
1	CPFGEVFNA	323	331	9	HLA-A*02:01	323	17	CPFGEVFNA	CPFGEVFNA	12872.5	17	12872.5	17	TRUE	TRUE
1	VVNQNAQAL	933	941	9	HLA-A*02:01	933	17	VVNQNAQAL	VVNQNAQAL	12960.82	17	12960.82	17	TRUE	TRUE
1	LQSLQTYVT	983	991	9	HLA-A*02:01	983	17	LQSLQTYVT	LQSLQTYVT	12961.24	17	12961.24	17	TRUE	TRUE
1	TLADAGFMK	809	817	9	HLA-A*02:01	809	17	TLADAGFMK	TLADAGFMK	13045.23	17	13045.23	17	TRUE	TRUE
1	FVGYLKPTT	253	261	9	HLA-A*02:01	253	17	FVGYLKPTT	FVGYLKPTT	13058.23	17	13058.23	17	TRUE	TRUE
1	FLTLTSGSD	7	15	9	HLA-A*02:01	7	17	FLTLTSGSD	FLTLTSGSD	13069.54	17	13069.54	17	TRUE	TRUE
1	NVFQTQAGC	627	635	9	HLA-A*02:01	627	17	NVFQTQAGC	NVFQTQAGC	13177.73	17	13177.73	17	TRUE	TRUE
1	SFELLNAPA	500	508	9	HLA-A*02:01	500	17	SFELLNAPA	SFELLNAPA	13340.56	17	13340.56	17	TRUE	TRUE
1	SVIIINNST	113	121	9	HLA-A*02:01	113	17	SVIIINNST	SVIIINNST	13397.26	17	13397.26	17	TRUE	TRUE
1	GVSVITPGT	580	588	9	HLA-A*02:01	580	18	GVSVITPGT	GVSVITPGT	13487.87	18	13487.87	18	TRUE	TRUE
1	MLKYDENGT	263	271	9	HLA-A*02:01	263	18	MLKYDENGT	MLKYDENGT	13533.04	18	13533.04	18	TRUE	TRUE
1	TNLCPFGEV	320	328	9	HLA-A*02:01	320	18	TNLCPFGEV	TNLCPFGEV	13588.94	18	13588.94	18	TRUE	TRUE
1	GIGVTQNVL	890	898	9	HLA-A*02:01	890	18	GIGVTQNVL	GIGVTQNVL	13613.52	18	13613.52	18	TRUE	TRUE
1	IPTNFSISI	696	704	9	HLA-A*02:01	696	18	IPTNFSISI	IPTNFSISI	13726.82	18	13726.82	18	TRUE	TRUE
1	SNVTGFHTI	64	72	9	HLA-A*02:01	64	18	SNVTGFHTI	SNVTGFHTI	13922.32	18	13922.32	18	TRUE	TRUE
1	YHLMSFPQA	1029	1037	9	HLA-A*02:01	1029	18	YHLMSFPQA	YHLMSFPQA	14309	18	14309	18	TRUE	TRUE
1	SNVYADSFV	380	388	9	HLA-A*02:01	380	18	SNVYADSFV	SNVYADSFV	14349.94	18	14349.94	18	TRUE	TRUE
1	FIEDLLFNK	799	807	9	HLA-A*02:01	799	18	FIEDLLFNK	FIEDLLFNK	14390.52	18	14390.52	18	TRUE	TRUE
1	LTDDMIAAY	847	855	9	HLA-A*02:01	847	18	LTDDMIAAY	LTDDMIAAY	14401.42	18	14401.42	18	TRUE	TRUE
1	FNGLTGTGV	529	537	9	HLA-A*02:01	529	18	FNGLTGTGV	FNGLTGTGV	14440.89	18	14440.89	18	TRUE	TRUE
1	VQIDRLITG	973	981	9	HLA-A*02:01	973	19	VQIDRLITG	VQIDRLITG	14482.99	19	14482.99	19	TRUE	TRUE
1	FCGKGYHLM	1024	1032	9	HLA-A*02:01	1024	19	FCGKGYHLM	FCGKGYHLM	14521.59	19	14521.59	19	TRUE	TRUE
1	IYQTSNFRV	299	307	9	HLA-A*02:01	299	19	IYQTSNFRV	IYQTSNFRV	14566.91	19	14566.91	19	TRUE	TRUE
1	YTMSLGADS	677	685	9	HLA-A*02:01	677	19	YTMSLGADS	YTMSLGADS	14585.67	19	14585.67	19	TRUE	TRUE
1	STGNNVFQT	623	631	9	HLA-A*02:01	623	19	STGNNVFQT	STGNNVFQT	14764.93	19	14764.93	19	TRUE	TRUE
1	IFLLFLTLT	3	11	9	HLA-A*02:01	3	19	IFLLFLTLT	IFLLFLTLT	14858.05	19	14858.05	19	TRUE	TRUE
1	RAILTAFSP	232	240	9	HLA-A*02:01	232	19	RAILTAFSP	RAILTAFSP	14898.29	19	14898.29	19	TRUE	TRUE
1	AATKMSECV	1007	1015	9	HLA-A*02:01	1007	19	AATKMSECV	AATKMSECV	14977.17	19	14977.17	19	TRUE	TRUE
1	RASANLAAT	1001	1009	9	HLA-A*02:01	1001	19	RASANLAAT	RASANLAAT	14997.27	19	14997.27	19	TRUE	TRUE
1	FVFKNKDGF	185	193	9	HLA-A*02:01	185	19	FVFKNKDGF	FVFKNKDGF	15168.62	19	15168.62	19	TRUE	TRUE
1	CLIGAEHVD	635	643	9	HLA-A*02:01	635	19	CLIGAEHVD	CLIGAEHVD	15200.16	19	15200.16	19	TRUE	TRUE
1	FKLPLGINI	220	228	9	HLA-A*02:01	220	19	FKLPLGINI	FKLPLGINI	15292.38	19	15292.38	19	TRUE	TRUE
1	SLQTYVTQQ	985	993	9	HLA-A*02:01	985	19	SLQTYVTQQ	SLQTYVTQQ	15293.87	19	15293.87	19	TRUE	TRUE
1	EVAVLYQDV	593	601	9	HLA-A*02:01	593	20	EVAVLYQDV	EVAVLYQDV	15593.63	20	15593.63	20	TRUE	TRUE
1	NIQKEIDRL	1160	1168	9	HLA-A*02:01	1160	20	NIQKEIDRL	NIQKEIDRL	15667.71	20	15667.71	20	TRUE	TRUE
1	ITTEVMPVS	704	712	9	HLA-A*02:01	704	20	ITTEVMPVS	ITTEVMPVS	15688.92	20	15688.92	20	TRUE	TRUE
1	RSFIEDLLF	797	805	9	HLA-A*02:01	797	20	RSFIEDLLF	RSFIEDLLF	15705.05	20	15705.05	20	TRUE	TRUE
1	IAYSNNTIA	687	695	9	HLA-A*02:01	687	20	IAYSNNTIA	IAYSNNTIA	15706.06	20	15706.06	20	TRUE	TRUE
1	SNCVADYSV	346	354	9	HLA-A*02:01	346	20	SNCVADYSV	SNCVADYSV	15770.94	20	15770.94	20	TRUE	TRUE
1	ALNCYWPLN	471	479	9	HLA-A*02:01	471	20	ALNCYWPLN	ALNCYWPLN	15779.99	20	15779.99	20	TRUE	TRUE
1	LTVLPPLLT	840	848	9	HLA-A*02:01	840	20	LTVLPPLLT	LTVLPPLLT	15930.1	20	15930.1	20	TRUE	TRUE
1	KSNVVRGWV	94	102	9	HLA-A*02:01	94	20	KSNVVRGWV	KSNVVRGWV	16084.76	20	16084.76	20	TRUE	TRUE
1	MTSCCSCLK	1219	1227	9	HLA-A*02:01	1219	20	MTSCCSCLK	MTSCCSCLK	16147.01	20	16147.01	20	TRUE	TRUE
1	EQDRNTREV	755	763	9	HLA-A*02:01	755	20	EQDRNTREV	EQDRNTREV	16180.58	20	16180.58	20	TRUE	TRUE
1	YSNVTGFHT	63	71	9	HLA-A*02:01	63	20	YSNVTGFHT	YSNVTGFHT	16210.03	20	16210.03	20	TRUE	TRUE
1	LQYGSFCTQ	736	744	9	HLA-A*02:01	736	20	LQYGSFCTQ	LQYGSFCTQ	16229.86	20	16229.86	20	TRUE	TRUE
1	FRSDTLYLT	47	55	9	HLA-A*02:01	47	20	FRSDTLYLT	FRSDTLYLT	16266.42	20	16266.42	20	TRUE	TRUE
1	IAGLIAIVM	1203	1211	9	HLA-A*02:01	1203	20	IAGLIAIVM	IAGLIAIVM	16289.49	20	16289.49	20	TRUE	TRUE
1	STAIHADQL	607	615	9	HLA-A*02:01	607	20	STAIHADQL	STAIHADQL	16314.55	20	16314.55	20	TRUE	TRUE
1	LGFIAGLIA	1200	1208	9	HLA-A*02:01	1200	21	LGFIAGLIA	LGFIAGLIA	16351.12	21	16351.12	21	TRUE	TRUE
1	FELCDNPFF	130	138	9	HLA-A*02:01	130	21	FELCDNPFF	FELCDNPFF	16440.35	21	16440.35	21	TRUE	TRUE
1	TFVSGNCDV	1102	1110	9	HLA-A*02:01	1102	21	TFVSGNCDV	TFVSGNCDV	16546.17	21	16546.17	21	TRUE	TRUE
1	HTMIFDNAF	149	157	9	HLA-A*02:01	149	21	HTMIFDNAF	HTMIFDNAF	16675.21	21	16675.21	21	TRUE	TRUE
1	GCLIGAEHV	634	642	9	HLA-A*02:01	634	21	GCLIGAEHV	GCLIGAEHV	16748.27	21	16748.27	21	TRUE	TRUE
1	KVTLADAGF	807	815	9	HLA-A*02:01	807	21	KVTLADAGF	KVTLADAGF	16816.36	21	16816.36	21	TRUE	TRUE
1	LTLTSGSDL	8	16	9	HLA-A*02:01	8	21	LTLTSGSDL	LTLTSGSDL	16827.28	21	16827.28	21	TRUE	TRUE
1	KLQDVVNQN	929	937	9	HLA-A*02:01	929	21	KLQDVVNQN	KLQDVVNQN	16881.08	21	16881.08	21	TRUE	TRUE
1	YSVLYNSTF	352	360	9	HLA-A*02:01	352	21	YSVLYNSTF	YSVLYNSTF	16891.49	21	16891.49	21	TRUE	TRUE
1	HLREFVFKN	181	189	9	HLA-A*02:01	181	21	HLREFVFKN	HLREFVFKN	16892.78	21	16892.78	21	TRUE	TRUE
1	TNFRAILTA	229	237	9	HLA-A*02:01	229	21	TNFRAILTA	TNFRAILTA	16920.02	21	16920.02	21	TRUE	TRUE
1	VTQQLIRAA	990	998	9	HLA-A*02:01	990	21	VTQQLIRAA	VTQQLIRAA	16959.07	21	16959.07	21	TRUE	TRUE
1	ISGINASVV	1151	1159	9	HLA-A*02:01	1151	21	ISGINASVV	ISGINASVV	17090.77	21	17090.77	21	TRUE	TRUE
1	NLLLQYGSF	733	741	9	HLA-A*02:01	733	21	NLLLQYGSF	NLLLQYGSF	17240.09	21	17240.09	21	TRUE	TRUE
1	KCSVKSFEI	287	295	9	HLA-A*02:01	287	21	KCSVKSFEI	KCSVKSFEI	17253.72	21	17253.72	21	TRUE	TRUE
1	QMYKTPTLK	769	777	9	HLA-A*02:01	769	22	QMYKTPTLK	QMYKTPTLK	17337.18	22	17337.18	22	TRUE	TRUE
1	GVFVFNGTS	1075	1083	9	HLA-A*02:01	1075	22	GVFVFNGTS	GVFVFNGTS	17358.95	22	17358.95	22	TRUE	TRUE
1	FCTQLNRAL	741	749	9	HLA-A*02:01	741	22	FCTQLNRAL	FCTQLNRAL	17436.89	22	17436.89	22	TRUE	TRUE
1	GTATAGWTF	862	870	9	HLA-A*02:01	862	22	GTATAGWTF	GTATAGWTF	17616.47	22	17616.47	22	TRUE	TRUE
1	NFGAISSVL	951	959	9	HLA-A*02:01	951	22	NFGAISSVL	NFGAISSVL	17717.78	22	17717.78	22	TRUE	TRUE
1	INHTFGNPV	72	80	9	HLA-A*02:01	72	22	INHTFGNPV	INHTFGNPV	17825.46	22	17825.46	22	TRUE	TRUE
1	EIDKGIYQT	294	302	9	HLA-A*02:01	294	22	EIDKGIYQT	EIDKGIYQT	17844.17	22	17844.17	22	TRUE	TRUE
1	LSFELLNAP	499	507	9	HLA-A*02:01	499	22	LSFELLNAP	LSFELLNAP	17894.64	22	17894.64	22	TRUE	TRUE
1	IVAYTMSLG	674	682	9	HLA-A*02:01	674	22	IVAYTMSLG	IVAYTMSLG	17901.61	22	17901.61	22	TRUE	TRUE
1	SAAAYFVGY	248	256	9	HLA-A*02:01	248	22	SAAAYFVGY	SAAAYFVGY	17931.66	22	17931.66	22	TRUE	TRUE
1	TAALVSGTA	856	864	9	HLA-A*02:01	856	22	TAALVSGTA	TAALVSGTA	17932.44	22	17932.44	22	TRUE	TRUE
1	LTPAWRIYS	615	623	9	HLA-A*02:01	615	22	LTPAWRIYS	LTPAWRIYS	18055.48	22	18055.48	22	TRUE	TRUE
1	NCTDVSTAI	602	610	9	HLA-A*02:01	602	23	NCTDVSTAI	NCTDVSTAI	18151.85	23	18151.85	23	TRUE	TRUE
1	ICAQKFNGL	832	840	9	HLA-A*02:01	832	23	ICAQKFNGL	ICAQKFNGL	18157.55	23	18157.55	23	TRUE	TRUE
1	CLGDINARD	822	830	9	HLA-A*02:01	822	23	CLGDINARD	CLGDINARD	18166.98	23	18166.98	23	TRUE	TRUE
1	NASSEVAVL	589	597	9	HLA-A*02:01	589	23	NASSEVAVL	NASSEVAVL	18208.3	23	18208.3	23	TRUE	TRUE
1	TVYDPLQPE	1118	1126	9	HLA-A*02:01	1118	23	TVYDPLQPE	TVYDPLQPE	18218.35	23	18218.35	23	TRUE	TRUE
1	CDIPIGAGI	648	656	9	HLA-A*02:01	648	23	CDIPIGAGI	CDIPIGAGI	18229.79	23	18229.79	23	TRUE	TRUE
1	KQIANQFNK	903	911	9	HLA-A*02:01	903	23	KQIANQFNK	KQIANQFNK	18353.88	23	18353.88	23	TRUE	TRUE
1	LIRAAEIRA	994	1002	9	HLA-A*02:01	994	23	LIRAAEIRA	LIRAAEIRA	18407.38	23	18407.38	23	TRUE	TRUE
1	NSTNVVIRA	119	127	9	HLA-A*02:01	119	23	NSTNVVIRA	NSTNVVIRA	18438.07	23	18438.07	23	TRUE	TRUE
1	EYISDAFSL	162	170	9	HLA-A*02:01	162	23	EYISDAFSL	EYISDAFSL	18484.22	23	18484.22	23	TRUE	TRUE
1	MGCVLAWNT	417	425	9	HLA-A*02:01	417	23	MGCVLAWNT	MGCVLAWNT	18500.02	23	18500.02	23	TRUE	TRUE
1	GQSKRVDFC	1017	1025	9	HLA-A*02:01	1017	23	GQSKRVDFC	GQSKRVDFC	18550.94	23	18550.94	23	TRUE	TRUE
1	YECDIPIGA	646	654	9	HLA-A*02:01	646	23	YECDIPIGA	YECDIPIGA	18575.43	23	18575.43	23	TRUE	TRUE
1	VLTPSSKRF	537	545	9	HLA-A*02:01	537	23	VLTPSSKRF	VLTPSSKRF	18576.64	23	18576.64	23	TRUE	TRUE
1	ALQIPFAMQ	875	883	9	HLA-A*02:01	875	23	ALQIPFAMQ	ALQIPFAMQ	18718.08	23	18718.08	23	TRUE	TRUE
1	FSNVYADSF	379	387	9	HLA-A*02:01	379	23	FSNVYADSF	FSNVYADSF	18770	23	18770	23	TRUE	TRUE
1	IGYQPYRVV	489	497	9	HLA-A*02:01	489	23	IGYQPYRVV	IGYQPYRVV	18885.7	23	18885.7	23	TRUE	TRUE
1	AATEKSNVV	90	98	9	HLA-A*02:01	90	23	AATEKSNVV	AATEKSNVV	18919.45	23	18919.45	23	TRUE	TRUE
1	TLKPIFKLP	215	223	9	HLA-A*02:01	215	23	TLKPIFKLP	TLKPIFKLP	18922.73	23	18922.73	23	TRUE	TRUE
1	KWPWYVWLG	1193	1201	9	HLA-A*02:01	1193	23	KWPWYVWLG	KWPWYVWLG	18928.46	23	18928.46	23	TRUE	TRUE
1	KISNCVADY	344	352	9	HLA-A*02:01	344	23	KISNCVADY	KISNCVADY	18932.35	23	18932.35	23	TRUE	TRUE
1	VFLHVTYVP	1043	1051	9	HLA-A*02:01	1043	23	VFLHVTYVP	VFLHVTYVP	18946.09	23	18946.09	23	TRUE	TRUE
1	KYFGGFNFS	777	785	9	HLA-A*02:01	777	24	KYFGGFNFS	KYFGGFNFS	18965.16	24	18965.16	24	TRUE	TRUE
1	ITNFRAILT	228	236	9	HLA-A*02:01	228	24	ITNFRAILT	ITNFRAILT	19028.25	24	19028.25	24	TRUE	TRUE
1	IEDLLFNKV	800	808	9	HLA-A*02:01	800	24	IEDLLFNKV	IEDLLFNKV	19069.69	24	19069.69	24	TRUE	TRUE
1	YIKWPWYVW	1191	1199	9	HLA-A*02:01	1191	24	YIKWPWYVW	YIKWPWYVW	19110.99	24	19110.99	24	TRUE	TRUE
1	TMSLGADSS	678	686	9	HLA-A*02:01	678	24	TMSLGADSS	TMSLGADSS	19121.74	24	19121.74	24	TRUE	TRUE
1	TNASSEVAV	588	596	9	HLA-A*02:01	588	24	TNASSEVAV	TNASSEVAV	19236.5	24	19236.5	24	TRUE	TRUE
1	INNTVYDPL	1115	1123	9	HLA-A*02:01	1115	24	INNTVYDPL	INNTVYDPL	19272.12	24	19272.12	24	TRUE	TRUE
1	FSLDVSEKS	168	176	9	HLA-A*02:01	168	24	FSLDVSEKS	FSLDVSEKS	19385.9	24	19385.9	24	TRUE	TRUE
1	GVYYPDEIF	39	47	9	HLA-A*02:01	39	24	GVYYPDEIF	GVYYPDEIF	19407.3	24	19407.3	24	TRUE	TRUE
1	SSIAYSNNT	685	693	9	HLA-A*02:01	685	24	SSIAYSNNT	SSIAYSNNT	19525.67	24	19525.67	24	TRUE	TRUE
1	ITTDNTFVS	1097	1105	9	HLA-A*02:01	1097	24	ITTDNTFVS	ITTDNTFVS	19574.54	24	19574.54	24	TRUE	TRUE

seq #	peptide	start	end	peptide length	allele	peptide index	median binding	netmhcpan_ba	netmhcpan_ba	netmhcpan_ba	netmhcpan_ba	legacy score	legacy rank	scores equal	percentiles equal
1	VVLSFELLN	497	505	9	HLA-A*02:01	497	24	VVLSFELLN	VVLSFELLN	19653.27	24	19653.27	24	TRUE	TRUE
1	WVFGSTMNN	101	109	9	HLA-A*02:01	101	25	WVFGSTMNN	WVFGSTMNN	19800.54	25	19800.54	25	TRUE	TRUE
1	MGTQTHTMI	144	152	9	HLA-A*02:01	144	25	MGTQTHTMI	MGTQTHTMI	19820.27	25	19820.27	25	TRUE	TRUE
1	LYNSTFFST	355	363	9	HLA-A*02:01	355	25	LYNSTFFST	LYNSTFFST	19823.27	25	19823.27	25	TRUE	TRUE
1	PMGTQTHTM	143	151	9	HLA-A*02:01	143	25	PMGTQTHTM	PMGTQTHTM	19828.41	25	19828.41	25	TRUE	TRUE
1	AAYFVGYLK	250	258	9	HLA-A*02:01	250	25	AAYFVGYLK	AAYFVGYLK	19856.97	25	19856.97	25	TRUE	TRUE
1	CTTFDDVQA	19	27	9	HLA-A*02:01	19	25	CTTFDDVQA	CTTFDDVQA	19867.92	25	19867.92	25	TRUE	TRUE
1	INITNFRAI	226	234	9	HLA-A*02:01	226	25	INITNFRAI	INITNFRAI	19920.01	25	19920.01	25	TRUE	TRUE
1	VRFPNITNL	314	322	9	HLA-A*02:01	314	25	VRFPNITNL	VRFPNITNL	19946.55	25	19946.55	25	TRUE	TRUE
1	STFKCYGVS	362	370	9	HLA-A*02:01	362	25	STFKCYGVS	STFKCYGVS	19956.04	25	19956.04	25	TRUE	TRUE
1	TQDLFLPFY	55	63	9	HLA-A*02:01	55	25	TQDLFLPFY	TQDLFLPFY	20073.41	25	20073.41	25	TRUE	TRUE
1	AVLYQDVNC	595	603	9	HLA-A*02:01	595	25	AVLYQDVNC	AVLYQDVNC	20122.78	25	20122.78	25	TRUE	TRUE
1	AAYTAALVS	853	861	9	HLA-A*02:01	853	25	AAYTAALVS	AAYTAALVS	20132.36	25	20132.36	25	TRUE	TRUE
1	GIYFAATEK	86	94	9	HLA-A*02:01	86	25	GIYFAATEK	GIYFAATEK	20153.5	25	20153.5	25	TRUE	TRUE
1	ITQRNFFSP	1086	1094	9	HLA-A*02:01	1086	25	ITQRNFFSP	ITQRNFFSP	20253.84	25	20253.84	25	TRUE	TRUE
1	VSGNCDVVI	1104	1112	9	HLA-A*02:01	1104	25	VSGNCDVVI	VSGNCDVVI	20277.07	25	20277.07	25	TRUE	TRUE
1	TIAIPTNFS	693	701	9	HLA-A*02:01	693	25	TIAIPTNFS	TIAIPTNFS	20362.61	25	20362.61	25	TRUE	TRUE
1	GTSWFITQR	1081	1089	9	HLA-A*02:01	1081	25	GTSWFITQR	GTSWFITQR	20409.37	25	20409.37	25	TRUE	TRUE
1	AEIRASANL	998	1006	9	HLA-A*02:01	998	25	AEIRASANL	AEIRASANL	20428.15	25	20428.15	25	TRUE	TRUE
1	HTVSLLRST	661	669	9	HLA-A*02:01	661	25	HTVSLLRST	HTVSLLRST	20531.84	25	20531.84	25	TRUE	TRUE
1	FDEDDSEPV	1238	1246	9	HLA-A*02:01	1238	26	FDEDDSEPV	FDEDDSEPV	20568.31	26	20568.31	26	TRUE	TRUE
1	KTSEILDIS	566	574	9	HLA-A*02:01	566	26	KTSEILDIS	KTSEILDIS	20585.01	26	20585.01	26	TRUE	TRUE
1	FQPFQQFGR	545	553	9	HLA-A*02:01	545	26	FQPFQQFGR	FQPFQQFGR	20650.6	26	20650.6	26	TRUE	TRUE
1	KQYGECLGD	817	825	9	HLA-A*02:01	817	26	KQYGECLGD	KQYGECLGD	20677.2	26	20677.2	26	TRUE	TRUE
1	KRSFIEDLL	796	804	9	HLA-A*02:01	796	26	KRSFIEDLL	KRSFIEDLL	20983.71	26	20983.71	26	TRUE	TRUE
1	GQTGVIADY	400	408	9	HLA-A*02:01	400	27	GQTGVIADY	GQTGVIADY	21284.4	27	21284.4	27	TRUE	TRUE
1	IINNTVYDP	1114	1122	9	HLA-A*02:01	1114	27	IINNTVYDP	IINNTVYDP	21323.37	27	21323.37	27	TRUE	TRUE
1	TTSTALGKL	922	930	9	HLA-A*02:01	922	27	TTSTALGKL	TTSTALGKL	21339.52	27	21339.52	27	TRUE	TRUE
1	AGICASYHT	654	662	9	HLA-A*02:01	654	27	AGICASYHT	AGICASYHT	21430.68	27	21430.68	27	TRUE	TRUE
1	NITNLCPFG	318	326	9	HLA-A*02:01	318	27	NITNLCPFG	NITNLCPFG	21638.98	27	21638.98	27	TRUE	TRUE
1	SVLNDILSR	957	965	9	HLA-A*02:01	957	27	SVLNDILSR	SVLNDILSR	21675.29	27	21675.29	27	TRUE	TRUE
1	STSQKSIVA	668	676	9	HLA-A*02:01	668	27	STSQKSIVA	STSQKSIVA	21693.14	27	21693.14	27	TRUE	TRUE
1	RGVYYPDEI	38	46	9	HLA-A*02:01	38	27	RGVYYPDEI	RGVYYPDEI	21727.19	27	21727.19	27	TRUE	TRUE
1	DLFLPFYSN	57	65	9	HLA-A*02:01	57	27	DLFLPFYSN	DLFLPFYSN	21748.84	27	21748.84	27	TRUE	TRUE
1	AYSNNTIAI	688	696	9	HLA-A*02:01	688	27	AYSNNTIAI	AYSNNTIAI	21849.07	27	21849.07	27	TRUE	TRUE
1	NLAATKMSE	1005	1013	9	HLA-A*02:01	1005	27	NLAATKMSE	NLAATKMSE	21870.6	27	21870.6	27	TRUE	TRUE
1	PVIPFKDGI	79	87	9	HLA-A*02:01	79	27	PVIPFKDGI	PVIPFKDGI	21896.88	27	21896.88	27	TRUE	TRUE
1	FELLNAPAT	501	509	9	HLA-A*02:01	501	27	FELLNAPAT	FELLNAPAT	21933.86	27	21933.86	27	TRUE	TRUE
1	IANQFNKAI	905	913	9	HLA-A*02:01	905	28	IANQFNKAI	IANQFNKAI	22004.46	28	22004.46	28	TRUE	TRUE
1	NFFSPQIIT	1090	1098	9	HLA-A*02:01	1090	28	NFFSPQIIT	NFFSPQIIT	22106.61	28	22106.61	28	TRUE	TRUE
1	IHADQLTPA	610	618	9	HLA-A*02:01	610	28	IHADQLTPA	IHADQLTPA	22114.02	28	22114.02	28	TRUE	TRUE
1	LNDYGFYTT	478	486	9	HLA-A*02:01	478	28	LNDYGFYTT	LNDYGFYTT	22174.4	28	22174.4	28	TRUE	TRUE
1	TAFSPAQDI	236	244	9	HLA-A*02:01	236	28	TAFSPAQDI	TAFSPAQDI	22275.15	28	22275.15	28	TRUE	TRUE
1	AGAALQIPF	872	880	9	HLA-A*02:01	872	28	AGAALQIPF	AGAALQIPF	22276.6	28	22276.6	28	TRUE	TRUE
1	VDLGDISGI	1146	1154	9	HLA-A*02:01	1146	28	VDLGDISGI	VDLGDISGI	22306.74	28	22306.74	28	TRUE	TRUE
1	YFGGFNFSQ	778	786	9	HLA-A*02:01	778	28	YFGGFNFSQ	YFGGFNFSQ	22344.42	28	22344.42	28	TRUE	TRUE
1	FFSTFKCYG	360	368	9	HLA-A*02:01	360	28	FFSTFKCYG	FFSTFKCYG	22370.32	28	22370.32	28	TRUE	TRUE
1	QIITTDNTF	1095	1103	9	HLA-A*02:01	1095	28	QIITTDNTF	QIITTDNTF	22429.69	28	22429.69	28	TRUE	TRUE
1	YNSTFFSTF	356	364	9	HLA-A*02:01	356	28	YNSTFFSTF	YNSTFFSTF	22481.93	28	22481.93	28	TRUE	TRUE
1	SEILDISPC	568	576	9	HLA-A*02:01	568	28	SEILDISPC	SEILDISPC	22501.64	28	22501.64	28	TRUE	TRUE
1	YWPLNDYGF	475	483	9	HLA-A*02:01	475	28	YWPLNDYGF	YWPLNDYGF	22533.56	28	22533.56	28	TRUE	TRUE
1	STNVVIRAC	120	128	9	HLA-A*02:01	120	28	STNVVIRAC	STNVVIRAC	22565.28	28	22565.28	28	TRUE	TRUE
1	GIINNTVYD	1113	1121	9	HLA-A*02:01	1113	29	GIINNTVYD	GIINNTVYD	22718.14	29	22718.14	29	TRUE	TRUE
1	GIYQTSNFR	298	306	9	HLA-A*02:01	298	29	GIYQTSNFR	GIYQTSNFR	22837.67	29	22837.67	29	TRUE	TRUE
1	YNYKYRYLR	436	444	9	HLA-A*02:01	436	29	YNYKYRYLR	YNYKYRYLR	22862.15	29	22862.15	29	TRUE	TRUE
1	WYVWLGFIA	1196	1204	9	HLA-A*02:01	1196	29	WYVWLGFIA	WYVWLGFIA	22875.76	29	22875.76	29	TRUE	TRUE
1	YYPDEIFRS	41	49	9	HLA-A*02:01	41	29	YYPDEIFRS	YYPDEIFRS	22878.48	29	22878.48	29	TRUE	TRUE
1	IFDNAFNCT	152	160	9	HLA-A*02:01	152	29	IFDNAFNCT	IFDNAFNCT	23127.86	29	23127.86	29	TRUE	TRUE
1	RALSGIAAE	747	755	9	HLA-A*02:01	747	29	RALSGIAAE	RALSGIAAE	23149.65	29	23149.65	29	TRUE	TRUE
1	TSNFRVVPS	302	310	9	HLA-A*02:01	302	29	TSNFRVVPS	TSNFRVVPS	23159.91	29	23159.91	29	TRUE	TRUE
1	ATKMSECVL	1008	1016	9	HLA-A*02:01	1008	29	ATKMSECVL	ATKMSECVL	23215.87	29	23215.87	29	TRUE	TRUE
1	RQIAPGQTG	395	403	9	HLA-A*02:01	395	30	RQIAPGQTG	RQIAPGQTG	23296.63	30	23296.63	30	TRUE	TRUE
1	GYQPYRVVV	490	498	9	HLA-A*02:01	490	30	GYQPYRVVV	GYQPYRVVV	23318.07	30	23318.07	30	TRUE	TRUE
1	GADSSIAYS	682	690	9	HLA-A*02:01	682	30	GADSSIAYS	GADSSIAYS	23349.88	30	23349.88	30	TRUE	TRUE
1	SFGGVSVIT	577	585	9	HLA-A*02:01	577	30	SFGGVSVIT	SFGGVSVIT	23369.86	30	23369.86	30	TRUE	TRUE
1	LGDISGINA	1148	1156	9	HLA-A*02:01	1148	30	LGDISGINA	LGDISGINA	23422	30	23422	30	TRUE	TRUE
1	KGYHLMSFP	1027	1035	9	HLA-A*02:01	1027	30	KGYHLMSFP	KGYHLMSFP	23458.52	30	23458.52	30	TRUE	TRUE
1	EVMPVSMAK	707	715	9	HLA-A*02:01	707	30	EVMPVSMAK	EVMPVSMAK	23462.06	30	23462.06	30	TRUE	TRUE
1	LAATKMSEC	1006	1014	9	HLA-A*02:01	1006	30	LAATKMSEC	LAATKMSEC	23509.08	30	23509.08	30	TRUE	TRUE
1	HGVVFLHVT	1040	1048	9	HLA-A*02:01	1040	30	HGVVFLHVT	HGVVFLHVT	23529.43	30	23529.43	30	TRUE	TRUE
1	FTDSVRDPK	558	566	9	HLA-A*02:01	558	30	FTDSVRDPK	FTDSVRDPK	23563.32	30	23563.32	30	TRUE	TRUE
1	YSNNTIAIP	689	697	9	HLA-A*02:01	689	30	YSNNTIAIP	YSNNTIAIP	23576.84	30	23576.84	30	TRUE	TRUE
1	YEQYIKWPW	1188	1196	9	HLA-A*02:01	1188	30	YEQYIKWPW	YEQYIKWPW	23592.4	30	23592.4	30	TRUE	TRUE
1	TLVKQLSSN	943	951	9	HLA-A*02:01	943	30	TLVKQLSSN	TLVKQLSSN	23614.36	30	23614.36	30	TRUE	TRUE
1	NCVADYSVL	347	355	9	HLA-A*02:01	347	30	NCVADYSVL	NCVADYSVL	23670.14	30	23670.14	30	TRUE	TRUE
1	INNSTNVVI	117	125	9	HLA-A*02:01	117	30	INNSTNVVI	INNSTNVVI	23770.49	30	23770.49	30	TRUE	TRUE
1	TNFSISITT	698	706	9	HLA-A*02:01	698	30	TNFSISITT	TNFSISITT	23794.68	30	23794.68	30	TRUE	TRUE
1	WGTSAAAYF	245	253	9	HLA-A*02:01	245	30	WGTSAAAYF	WGTSAAAYF	23824.82	30	23824.82	30	TRUE	TRUE
1	RNFFSPQII	1089	1097	9	HLA-A*02:01	1089	30	RNFFSPQII	RNFFSPQII	23851.38	30	23851.38	30	TRUE	TRUE
1	FAQVKQMYK	764	772	9	HLA-A*02:01	764	30	FAQVKQMYK	FAQVKQMYK	23923.74	30	23923.74	30	TRUE	TRUE
1	VVIRACNFE	123	131	9	HLA-A*02:01	123	31	VVIRACNFE	VVIRACNFE	23948.35	31	23948.35	31	TRUE	TRUE
1	GYLKPTTFM	255	263	9	HLA-A*02:01	255	31	GYLKPTTFM	GYLKPTTFM	23971.42	31	23971.42	31	TRUE	TRUE
1	KLSTDLIKN	514	522	9	HLA-A*02:01	514	31	KLSTDLIKN	KLSTDLIKN	24010.87	31	24010.87	31	TRUE	TRUE
1	AIPTNFSIS	695	703	9	HLA-A*02:01	695	31	AIPTNFSIS	AIPTNFSIS	24043.63	31	24043.63	31	TRUE	TRUE
1	FNGLTVLPP	837	845	9	HLA-A*02:01	837	31	FNGLTVLPP	FNGLTVLPP	24048.57	31	24048.57	31	TRUE	TRUE
1	ISNCVADYS	345	353	9	HLA-A*02:01	345	31	ISNCVADYS	ISNCVADYS	24079.81	31	24079.81	31	TRUE	TRUE
1	ASSEVAVLY	590	598	9	HLA-A*02:01	590	31	ASSEVAVLY	ASSEVAVLY	24080.61	31	24080.61	31	TRUE	TRUE
1	FPREGVFVF	1071	1079	9	HLA-A*02:01	1071	31	FPREGVFVF	FPREGVFVF	24101.19	31	24101.19	31	TRUE	TRUE
1	RFPNITNLC	315	323	9	HLA-A*02:01	315	31	RFPNITNLC	RFPNITNLC	24128.07	31	24128.07	31	TRUE	TRUE
1	FKNHTSPDV	1138	1146	9	HLA-A*02:01	1138	31	FKNHTSPDV	FKNHTSPDV	24131.46	31	24131.46	31	TRUE	TRUE
1	GFHTINHTF	68	76	9	HLA-A*02:01	68	31	GFHTINHTF	GFHTINHTF	24294.15	31	24294.15	31	TRUE	TRUE
1	NCDVVIGII	1107	1115	9	HLA-A*02:01	1107	31	NCDVVIGII	NCDVVIGII	24297.57	31	24297.57	31	TRUE	TRUE
1	YADSFVVKG	383	391	9	HLA-A*02:01	383	31	YADSFVVKG	YADSFVVKG	24323.08	31	24323.08	31	TRUE	TRUE
1	TFKCYGVSA	363	371	9	HLA-A*02:01	363	31	TFKCYGVSA	TFKCYGVSA	24342.83	31	24342.83	31	TRUE	TRUE
1	PWYVWLGFI	1195	1203	9	HLA-A*02:01	1195	31	PWYVWLGFI	PWYVWLGFI	24364.96	31	24364.96	31	TRUE	TRUE
1	QFNKAISQI	908	916	9	HLA-A*02:01	908	31	QFNKAISQI	QFNKAISQI	24394.51	31	24394.51	31	TRUE	TRUE
1	WPWYVWLGF	1194	1202	9	HLA-A*02:01	1194	31	WPWYVWLGF	WPWYVWLGF	24410.35	31	24410.35	31	TRUE	TRUE
1	QKFNGLTVL	835	843	9	HLA-A*02:01	835	31	QKFNGLTVL	QKFNGLTVL	24465.34	31	24465.34	31	TRUE	TRUE
1	SQILPDPLK	785	793	9	HLA-A*02:01	785	31	SQILPDPLK	SQILPDPLK	24484.15	31	24484.15	31	TRUE	TRUE
1	GVIADYNYK	403	411	9	HLA-A*02:01	403	31	GVIADYNYK	GVIADYNYK	24499.25	31	24499.25	31	TRUE	TRUE
1	YLRHGKLRP	442	450	9	HLA-A*02:01	442	31	YLRHGKLRP	YLRHGKLRP	24525.24	31	24525.24	31	TRUE	TRUE
1	TTEVMPVSM	705	713	9	HLA-A*02:01	705	31	TTEVMPVSM	TTEVMPVSM	24534.01	31	24534.01	31	TRUE	TRUE
1	GSFCTQLNR	739	747	9	HLA-A*02:01	739	31	GSFCTQLNR	GSFCTQLNR	24582.63	31	24582.63	31	TRUE	TRUE
1	ESLIDLQEL	1177	1185	9	HLA-A*02:01	1177	32	ESLIDLQEL	ESLIDLQEL	24605.79	32	24605.79	32	TRUE	TRUE
1	FGNPVIPFK	76	84	9	HLA-A*02:01	76	32	FGNPVIPFK	FGNPVIPFK	24610.04	32	24610.04	32	TRUE	TRUE
1	SPAQDIWGT	239	247	9	HLA-A*02:01	239	32	SPAQDIWGT	SPAQDIWGT	24624.69	32	24624.69	32	TRUE	TRUE
1	GDVVRFPNI	311	319	9	HLA-A*02:01	311	32	GDVVRFPNI	GDVVRFPNI	24645.47	32	24645.47	32	TRUE	TRUE
1	NTIAIPTNF	692	700	9	HLA-A*02:01	692	32	NTIAIPTNF	NTIAIPTNF	24737.11	32	24737.11	32	TRUE	TRUE
1	FQQFGRDVS	548	556	9	HLA-A*02:01	548	32	FQQFGRDVS	FQQFGRDVS	24801.7	32	24801.7	32	TRUE	TRUE
1	SLLRSTSQK	664	672	9	HLA-A*02:01	664	32	SLLRSTSQK	SLLRSTSQK	24846.83	32	24846.83	32	TRUE	TRUE
1	LSSNFGAIS	948	956	9	HLA-A*02:01	948	32	LSSNFGAIS	LSSNFGAIS	24918.17	32	24918.17	32	TRUE	TRUE
1	QYGSFCTQL	737	745	9	HLA-A*02:01	737	32	QYGSFCTQL	QYGSFCTQL	25042.49	32	25042.49	32	TRUE	TRUE
1	IGAGICASY	652	660	9	HLA-A*02:01	652	32	IGAGICASY	IGAGICASY	25044.12	32	25044.12	32	TRUE	TRUE
1	MSFPQAAPH	1032	1040	9	HLA-A*02:01	1032	32	MSFPQAAPH	MSFPQAAPH	25090.78	32	25090.78	32	TRUE	TRUE
1	GTNASSEVA	587	595	9	HLA-A*02:01	587	32	GTNASSEVA	GTNASSEVA	25091.31	32	25091.31	32	TRUE	TRUE

seq #	peptide	start	end	peptide length	allele	peptide index	median binding	netmhcpan_ba	netmhcpan_ba	netmhcpan_ba	netmhcpan_ba	legacy score	legacy rank	scores equal	percentiles equal
1	TSAAAYFVG	247	255	9	HLA-A*02:01	247	32	TSAAAYFVG	TSAAAYFVG	25115.76	32	25115.76	32	TRUE	TRUE
1	QRNFFSPQI	1088	1096	9	HLA-A*02:01	1088	32	QRNFFSPQI	QRNFFSPQI	25128.27	32	25128.27	32	TRUE	TRUE
1	FNGTSWFIT	1079	1087	9	HLA-A*02:01	1079	32	FNGTSWFIT	FNGTSWFIT	25206.42	32	25206.42	32	TRUE	TRUE
1	LFLTLTSGS	6	14	9	HLA-A*02:01	6	33	LFLTLTSGS	LFLTLTSGS	25252.27	33	25252.27	33	TRUE	TRUE
1	LLRSTSQKS	665	673	9	HLA-A*02:01	665	33	LLRSTSQKS	LLRSTSQKS	25263.75	33	25263.75	33	TRUE	TRUE
1	KSIVAYTMS	672	680	9	HLA-A*02:01	672	33	KSIVAYTMS	KSIVAYTMS	25264.03	33	25264.03	33	TRUE	TRUE
1	IAPGQTGVI	397	405	9	HLA-A*02:01	397	33	IAPGQTGVI	IAPGQTGVI	25315.19	33	25315.19	33	TRUE	TRUE
1	KFNGLTVLP	836	844	9	HLA-A*02:01	836	33	KFNGLTVLP	KFNGLTVLP	25367.57	33	25367.57	33	TRUE	TRUE
1	TVLPPLLTD	841	849	9	HLA-A*02:01	841	33	TVLPPLLTD	TVLPPLLTD	25421.42	33	25421.42	33	TRUE	TRUE
1	EILDISPCS	569	577	9	HLA-A*02:01	569	33	EILDISPCS	EILDISPCS	25453.06	33	25453.06	33	TRUE	TRUE
1	FGRDVSDFT	551	559	9	HLA-A*02:01	551	33	FGRDVSDFT	FGRDVSDFT	25461.34	33	25461.34	33	TRUE	TRUE
1	ITNLCPFGE	319	327	9	HLA-A*02:01	319	33	ITNLCPFGE	ITNLCPFGE	25511.52	33	25511.52	33	TRUE	TRUE
1	FPSVYAWER	334	342	9	HLA-A*02:01	334	33	FPSVYAWER	FPSVYAWER	25557.1	33	25557.1	33	TRUE	TRUE
1	YRYLRHGKL	440	448	9	HLA-A*02:01	440	33	YRYLRHGKL	YRYLRHGKL	25596.95	33	25596.95	33	TRUE	TRUE
1	CCMTSCCSC	1217	1225	9	HLA-A*02:01	1217	33	CCMTSCCSC	CCMTSCCSC	25601.94	33	25601.94	33	TRUE	TRUE
1	TQRNFFSPQ	1087	1095	9	HLA-A*02:01	1087	33	TQRNFFSPQ	TQRNFFSPQ	25678.79	33	25678.79	33	TRUE	TRUE
1	FNFSQILPD	782	790	9	HLA-A*02:01	782	33	FNFSQILPD	FNFSQILPD	25730.8	33	25730.8	33	TRUE	TRUE
1	FEYISDAFS	161	169	9	HLA-A*02:01	161	34	FEYISDAFS	FEYISDAFS	25802.17	34	25802.17	34	TRUE	TRUE
1	MAYRFNGIG	884	892	9	HLA-A*02:01	884	34	MAYRFNGIG	MAYRFNGIG	25833.17	34	25833.17	34	TRUE	TRUE
1	TGTGVLTPS	533	541	9	HLA-A*02:01	533	34	TGTGVLTPS	TGTGVLTPS	25881.3	34	25881.3	34	TRUE	TRUE
1	AMQMAYRFN	881	889	9	HLA-A*02:01	881	34	AMQMAYRFN	AMQMAYRFN	25911.84	34	25911.84	34	TRUE	TRUE
1	NFKHLREFV	178	186	9	HLA-A*02:01	178	34	NFKHLREFV	NFKHLREFV	25991.58	34	25991.58	34	TRUE	TRUE
1	DTLYLTQDL	50	58	9	HLA-A*02:01	50	34	DTLYLTQDL	DTLYLTQDL	26031.55	34	26031.55	34	TRUE	TRUE
1	VSATKLNDL	369	377	9	HLA-A*02:01	369	34	VSATKLNDL	VSATKLNDL	26062.26	34	26062.26	34	TRUE	TRUE
1	TPAWRIYST	616	624	9	HLA-A*02:01	616	34	TPAWRIYST	TPAWRIYST	26064.79	34	26064.79	34	TRUE	TRUE
1	KAISQIQES	911	919	9	HLA-A*02:01	911	34	KAISQIQES	KAISQIQES	26091.6	34	26091.6	34	TRUE	TRUE
1	VFNATKFPS	328	336	9	HLA-A*02:01	328	34	VFNATKFPS	VFNATKFPS	26101.48	34	26101.48	34	TRUE	TRUE
1	GVVFLHVTY	1041	1049	9	HLA-A*02:01	1041	34	GVVFLHVTY	GVVFLHVTY	26185.78	34	26185.78	34	TRUE	TRUE
1	QLIRAAEIR	993	1001	9	HLA-A*02:01	993	34	QLIRAAEIR	QLIRAAEIR	26196.56	34	26196.56	34	TRUE	TRUE
1	AYRFNGIGV	885	893	9	HLA-A*02:01	885	34	AYRFNGIGV	AYRFNGIGV	26218.11	34	26218.11	34	TRUE	TRUE
1	FVVKGDDVR	387	395	9	HLA-A*02:01	387	34	FVVKGDDVR	FVVKGDDVR	26236.83	34	26236.83	34	TRUE	TRUE
1	ILSRLDKVE	962	970	9	HLA-A*02:01	962	34	ILSRLDKVE	ILSRLDKVE	26285.71	34	26285.71	34	TRUE	TRUE
1	PQAAPHGVV	1035	1043	9	HLA-A*02:01	1035	34	PQAAPHGVV	PQAAPHGVV	26327.26	34	26327.26	34	TRUE	TRUE
1	AQALNTLVK	938	946	9	HLA-A*02:01	938	34	AQALNTLVK	AQALNTLVK	26342.09	34	26342.09	34	TRUE	TRUE
1	TFGNPVIPF	75	83	9	HLA-A*02:01	75	34	TFGNPVIPF	TFGNPVIPF	26349.21	34	26349.21	34	TRUE	TRUE
1	FDNAFNCTF	153	161	9	HLA-A*02:01	153	35	FDNAFNCTF	FDNAFNCTF	26449.18	35	26449.18	35	TRUE	TRUE
1	SQSVIIINN	111	119	9	HLA-A*02:01	111	35	SQSVIIINN	SQSVIIINN	26467.79	35	26467.79	35	TRUE	TRUE
1	SNNTIAIPT	690	698	9	HLA-A*02:01	690	35	SNNTIAIPT	SNNTIAIPT	26470.93	35	26470.93	35	TRUE	TRUE
1	LTTTSTALG	920	928	9	HLA-A*02:01	920	35	LTTTSTALG	LTTTSTALG	26493.56	35	26493.56	35	TRUE	TRUE
1	VLKGVKLHY	1246	1254	9	HLA-A*02:01	1246	35	VLKGVKLHY	VLKGVKLHY	26507.34	35	26507.34	35	TRUE	TRUE
1	PLQPELDSF	1122	1130	9	HLA-A*02:01	1122	35	PLQPELDSF	PLQPELDSF	26523.68	35	26523.68	35	TRUE	TRUE
1	NSTFFSTFK	357	365	9	HLA-A*02:01	357	35	NSTFFSTFK	NSTFFSTFK	26546.65	35	26546.65	35	TRUE	TRUE
1	CSCGSCCKF	1230	1238	9	HLA-A*02:01	1230	35	CSCGSCCKF	CSCGSCCKF	26635.27	35	26635.27	35	TRUE	TRUE
1	SCCSCLKGA	1221	1229	9	HLA-A*02:01	1221	35	SCCSCLKGA	SCCSCLKGA	26697.01	35	26697.01	35	TRUE	TRUE
1	TTFDDVQAP	20	28	9	HLA-A*02:01	20	35	TTFDDVQAP	TTFDDVQAP	26796.28	35	26796.28	35	TRUE	TRUE
1	HTFGNPVIP	74	82	9	HLA-A*02:01	74	35	HTFGNPVIP	HTFGNPVIP	26802.66	35	26802.66	35	TRUE	TRUE
1	ESLTTTSTA	918	926	9	HLA-A*02:01	918	35	ESLTTTSTA	ESLTTTSTA	26822.67	35	26822.67	35	TRUE	TRUE
1	SVYAWERKK	336	344	9	HLA-A*02:01	336	35	SVYAWERKK	SVYAWERKK	26836.61	35	26836.61	35	TRUE	TRUE
1	TINHTFGNP	71	79	9	HLA-A*02:01	71	35	TINHTFGNP	TINHTFGNP	26840.96	35	26840.96	35	TRUE	TRUE
1	NVVRGWVFG	96	104	9	HLA-A*02:01	96	35	NVVRGWVFG	NVVRGWVFG	26860.42	35	26860.42	35	TRUE	TRUE
1	KCYGVSATK	365	373	9	HLA-A*02:01	365	35	KCYGVSATK	KCYGVSATK	26877	35	26877	35	TRUE	TRUE
1	INARDLICA	826	834	9	HLA-A*02:01	826	35	INARDLICA	INARDLICA	26900.27	35	26900.27	35	TRUE	TRUE
1	CLKGACSCG	1225	1233	9	HLA-A*02:01	1225	35	CLKGACSCG	CLKGACSCG	26905.79	35	26905.79	35	TRUE	TRUE
1	STDLIKNQC	516	524	9	HLA-A*02:01	516	35	STDLIKNQC	STDLIKNQC	26927.05	35	26927.05	35	TRUE	TRUE
1	IPFKDGIYF	81	89	9	HLA-A*02:01	81	35	IPFKDGIYF	IPFKDGIYF	26954.18	35	26954.18	35	TRUE	TRUE
1	LQPELDSFK	1123	1131	9	HLA-A*02:01	1123	36	LQPELDSFK	LQPELDSFK	27028.05	36	27028.05	36	TRUE	TRUE
1	LKPIFKLPL	216	224	9	HLA-A*02:01	216	36	LKPIFKLPL	LKPIFKLPL	27099.8	36	27099.8	36	TRUE	TRUE
1	AEVQIDRLI	971	979	9	HLA-A*02:01	971	36	AEVQIDRLI	AEVQIDRLI	27144.41	36	27144.41	36	TRUE	TRUE
1	TGFHTINHT	67	75	9	HLA-A*02:01	67	36	TGFHTINHT	TGFHTINHT	27148.8	36	27148.8	36	TRUE	TRUE
1	AALVSGTAT	857	865	9	HLA-A*02:01	857	36	AALVSGTAT	AALVSGTAT	27163.79	36	27163.79	36	TRUE	TRUE
1	TQLNRALSG	743	751	9	HLA-A*02:01	743	36	TQLNRALSG	TQLNRALSG	27294.3	36	27294.3	36	TRUE	TRUE
1	TFEYISDAF	160	168	9	HLA-A*02:01	160	36	TFEYISDAF	TFEYISDAF	27315.29	36	27315.29	36	TRUE	TRUE
1	VAKNLNESL	1171	1179	9	HLA-A*02:01	1171	36	VAKNLNESL	VAKNLNESL	27354.32	36	27354.32	36	TRUE	TRUE
1	SFIEDLLFN	798	806	9	HLA-A*02:01	798	36	SFIEDLLFN	SFIEDLLFN	27392.52	36	27392.52	36	TRUE	TRUE
1	NDYGFYTTT	479	487	9	HLA-A*02:01	479	36	NDYGFYTTT	NDYGFYTTT	27412.98	36	27412.98	36	TRUE	TRUE
1	SQNPLAELK	279	287	9	HLA-A*02:01	279	36	SQNPLAELK	SQNPLAELK	27426.34	36	27426.34	36	TRUE	TRUE
1	KNQCVNFNF	521	529	9	HLA-A*02:01	521	36	KNQCVNFNF	KNQCVNFNF	27445.64	36	27445.64	36	TRUE	TRUE
1	GTQTHTMIF	145	153	9	HLA-A*02:01	145	36	GTQTHTMIF	GTQTHTMIF	27458.99	36	27458.99	36	TRUE	TRUE
1	GTITDAVDC	270	278	9	HLA-A*02:01	270	36	GTITDAVDC	GTITDAVDC	27461.96	36	27461.96	36	TRUE	TRUE
1	EIDRLNEVA	1164	1172	9	HLA-A*02:01	1164	36	EIDRLNEVA	EIDRLNEVA	27488.42	36	27488.42	36	TRUE	TRUE
1	TQTHTMIFD	146	154	9	HLA-A*02:01	146	36	TQTHTMIFD	TQTHTMIFD	27514.92	36	27514.92	36	TRUE	TRUE
1	ITPGTNASS	584	592	9	HLA-A*02:01	584	37	ITPGTNASS	ITPGTNASS	27624.97	37	27624.97	37	TRUE	TRUE
1	DSTECANLL	727	735	9	HLA-A*02:01	727	37	DSTECANLL	DSTECANLL	27632.15	37	27632.15	37	TRUE	TRUE
1	VITPGTNAS	583	591	9	HLA-A*02:01	583	37	VITPGTNAS	VITPGTNAS	27641.42	37	27641.42	37	TRUE	TRUE
1	YTQHTSSMR	30	38	9	HLA-A*02:01	30	37	YTQHTSSMR	YTQHTSSMR	27687.22	37	27687.22	37	TRUE	TRUE
1	QDIWGTSAA	242	250	9	HLA-A*02:01	242	37	QDIWGTSAA	QDIWGTSAA	27709.41	37	27709.41	37	TRUE	TRUE
1	TVCGPKLST	509	517	9	HLA-A*02:01	509	37	TVCGPKLST	TVCGPKLST	27714.79	37	27714.79	37	TRUE	TRUE
1	LIKNQCVNF	519	527	9	HLA-A*02:01	519	37	LIKNQCVNF	LIKNQCVNF	27758	37	27758	37	TRUE	TRUE
1	FRAILTAFS	231	239	9	HLA-A*02:01	231	37	FRAILTAFS	FRAILTAFS	27805.19	37	27805.19	37	TRUE	TRUE
1	PFFAVSKPM	136	144	9	HLA-A*02:01	136	37	PFFAVSKPM	PFFAVSKPM	27852.18	37	27852.18	37	TRUE	TRUE
1	YRFNGIGVT	886	894	9	HLA-A*02:01	886	37	YRFNGIGVT	YRFNGIGVT	27863.03	37	27863.03	37	TRUE	TRUE
1	NQNAQALNT	935	943	9	HLA-A*02:01	935	37	NQNAQALNT	NQNAQALNT	27936.68	37	27936.68	37	TRUE	TRUE
1	IPIGAGICA	650	658	9	HLA-A*02:01	650	37	IPIGAGICA	IPIGAGICA	27993.56	37	27993.56	37	TRUE	TRUE
1	NQFNKAISQ	907	915	9	HLA-A*02:01	907	37	NQFNKAISQ	NQFNKAISQ	28024.79	37	28024.79	37	TRUE	TRUE
1	MSECVLGQS	1011	1019	9	HLA-A*02:01	1011	37	MSECVLGQS	MSECVLGQS	28041.17	37	28041.17	37	TRUE	TRUE
1	PVSMAKTSV	710	718	9	HLA-A*02:01	710	38	PVSMAKTSV	PVSMAKTSV	28135.07	38	28135.07	38	TRUE	TRUE
1	RDISNVPFS	453	461	9	HLA-A*02:01	453	38	RDISNVPFS	RDISNVPFS	28144.51	38	28144.51	38	TRUE	TRUE
1	HADQLTPAW	611	619	9	HLA-A*02:01	611	38	HADQLTPAW	HADQLTPAW	28150.59	38	28150.59	38	TRUE	TRUE
1	CTQLNRALS	742	750	9	HLA-A*02:01	742	38	CTQLNRALS	CTQLNRALS	28189	38	28189	38	TRUE	TRUE
1	QNAQALNTL	936	944	9	HLA-A*02:01	936	38	QNAQALNTL	QNAQALNTL	28231.73	38	28231.73	38	TRUE	TRUE
1	SFCTQLNRA	740	748	9	HLA-A*02:01	740	38	SFCTQLNRA	SFCTQLNRA	28327.81	38	28327.81	38	TRUE	TRUE
1	RVDFCGKGY	1021	1029	9	HLA-A*02:01	1021	38	RVDFCGKGY	RVDFCGKGY	28352.96	38	28352.96	38	TRUE	TRUE
1	EVFAQVKQM	762	770	9	HLA-A*02:01	762	38	EVFAQVKQM	EVFAQVKQM	28353.56	38	28353.56	38	TRUE	TRUE
1	ADYSVLYNS	350	358	9	HLA-A*02:01	350	38	ADYSVLYNS	ADYSVLYNS	28402.99	38	28402.99	38	TRUE	TRUE
1	IPFAMQMAY	878	886	9	HLA-A*02:01	878	38	IPFAMQMAY	IPFAMQMAY	28420.22	38	28420.22	38	TRUE	TRUE
1	TLTSGSDLD	9	17	9	HLA-A*02:01	9	38	TLTSGSDLD	TLTSGSDLD	28456.53	38	28456.53	38	TRUE	TRUE
1	SSVLNDILS	956	964	9	HLA-A*02:01	956	38	SSVLNDILS	SSVLNDILS	28462.37	38	28462.37	38	TRUE	TRUE
1	HTINHTFGN	70	78	9	HLA-A*02:01	70	38	HTINHTFGN	HTINHTFGN	28490.41	38	28490.41	38	TRUE	TRUE
1	FSPDGKPCT	460	468	9	HLA-A*02:01	460	39	FSPDGKPCT	FSPDGKPCT	28615.85	39	28615.85	39	TRUE	TRUE
1	FNCTFEYIS	157	165	9	HLA-A*02:01	157	39	FNCTFEYIS	FNCTFEYIS	28671.32	39	28671.32	39	TRUE	TRUE
1	WFITQRNFF	1084	1092	9	HLA-A*02:01	1084	39	WFITQRNFF	WFITQRNFF	28761.42	39	28761.42	39	TRUE	TRUE
1	QLTPAWRIY	614	622	9	HLA-A*02:01	614	39	QLTPAWRIY	QLTPAWRIY	28778.23	39	28778.23	39	TRUE	TRUE
1	AIHADQLTP	609	617	9	HLA-A*02:01	609	39	AIHADQLTP	AIHADQLTP	28870.86	39	28870.86	39	TRUE	TRUE
1	LCFSNVYAD	377	385	9	HLA-A*02:01	377	39	LCFSNVYAD	LCFSNVYAD	28950.62	39	28950.62	39	TRUE	TRUE
1	GFNFSQILP	781	789	9	HLA-A*02:01	781	39	GFNFSQILP	GFNFSQILP	28951.57	39	28951.57	39	TRUE	TRUE
1	NTLVKQLSS	942	950	9	HLA-A*02:01	942	39	NTLVKQLSS	NTLVKQLSS	28984.15	39	28984.15	39	TRUE	TRUE
1	EVAKNLNES	1170	1178	9	HLA-A*02:01	1170	39	EVAKNLNES	EVAKNLNES	29045.06	39	29045.06	39	TRUE	TRUE
1	LKYFGGFNF	776	784	9	HLA-A*02:01	776	40	LKYFGGFNF	LKYFGGFNF	29108.93	40	29108.93	40	TRUE	TRUE
1	SFVVKGDDV	386	394	9	HLA-A*02:01	386	40	SFVVKGDDV	SFVVKGDDV	29111.76	40	29111.76	40	TRUE	TRUE
1	LGINITNFR	224	232	9	HLA-A*02:01	224	40	LGINITNFR	LGINITNFR	29213.36	40	29213.36	40	TRUE	TRUE
1	CANLLLQYG	731	739	9	HLA-A*02:01	731	40	CANLLLQYG	CANLLLQYG	29231.07	40	29231.07	40	TRUE	TRUE
1	VLPPLLTDD	842	850	9	HLA-A*02:01	842	40	VLPPLLTDD	VLPPLLTDD	29236.13	40	29236.13	40	TRUE	TRUE
1	LGDINARDL	823	831	9	HLA-A*02:01	823	40	LGDINARDL	LGDINARDL	29314.37	40	29314.37	40	TRUE	TRUE
1	VYYPDEIFR	40	48	9	HLA-A*02:01	40	40	VYYPDEIFR	VYYPDEIFR	29330.87	40	29330.87	40	TRUE	TRUE
1	SANLAATKM	1003	1011	9	HLA-A*02:01	1003	40	SANLAATKM	SANLAATKM	29398.86	40	29398.86	40	TRUE	TRUE

seq #	peptide	start	end	peptide length	allele	peptide index	median binding	netmhcpan_ba	netmhcpan_ba	netmhcpan_ba	netmhcpan_ba	legacy score	legacy rank	scores equal	percentiles equal
1	LIGAEHVDT	636	644	9	HLA-A*02:01	636	40	LIGAEHVDT	LIGAEHVDT	29402.67	40	29402.67	40	TRUE	TRUE
1	QKSIVAYTM	671	679	9	HLA-A*02:01	671	40	QKSIVAYTM	QKSIVAYTM	29408.08	40	29408.08	40	TRUE	TRUE
1	VTYVPSQER	1047	1055	9	HLA-A*02:01	1047	40	VTYVPSQER	VTYVPSQER	29420.5	40	29420.5	40	TRUE	TRUE
1	LVSGTATAG	859	867	9	HLA-A*02:01	859	40	LVSGTATAG	LVSGTATAG	29442.78	40	29442.78	40	TRUE	TRUE
1	ASANLAATK	1002	1010	9	HLA-A*02:01	1002	40	ASANLAATK	ASANLAATK	29443.11	40	29443.11	40	TRUE	TRUE
1	FNKVTLADA	805	813	9	HLA-A*02:01	805	40	FNKVTLADA	FNKVTLADA	29451.39	40	29451.39	40	TRUE	TRUE
1	YAWERKKIS	338	346	9	HLA-A*02:01	338	40	YAWERKKIS	YAWERKKIS	29482.63	40	29482.63	40	TRUE	TRUE
1	YTTTGIGYQ	484	492	9	HLA-A*02:01	484	40	YTTTGIGYQ	YTTTGIGYQ	29510.72	40	29510.72	40	TRUE	TRUE
1	TTDNTFVSG	1098	1106	9	HLA-A*02:01	1098	40	TTDNTFVSG	TTDNTFVSG	29532.12	40	29532.12	40	TRUE	TRUE
1	TQQLIRAAE	991	999	9	HLA-A*02:01	991	40	TQQLIRAAE	TQQLIRAAE	29541.38	40	29541.38	40	TRUE	TRUE
1	ISNVPFSPD	455	463	9	HLA-A*02:01	455	41	ISNVPFSPD	ISNVPFSPD	29723.5	41	29723.5	41	TRUE	TRUE
1	GVTQNVLYE	892	900	9	HLA-A*02:01	892	41	GVTQNVLYE	GVTQNVLYE	29795.95	41	29795.95	41	TRUE	TRUE
1	VRDPKTSEI	562	570	9	HLA-A*02:01	562	41	VRDPKTSEI	VRDPKTSEI	29803.68	41	29803.68	41	TRUE	TRUE
1	SSNFGAISS	949	957	9	HLA-A*02:01	949	41	SSNFGAISS	SSNFGAISS	29824	41	29824	41	TRUE	TRUE
1	CTPPALNCY	467	475	9	HLA-A*02:01	467	41	CTPPALNCY	CTPPALNCY	29828.85	41	29828.85	41	TRUE	TRUE
1	CAQKFNGLT	833	841	9	HLA-A*02:01	833	41	CAQKFNGLT	CAQKFNGLT	29865.34	41	29865.34	41	TRUE	TRUE
1	MNNKSQSVI	107	115	9	HLA-A*02:01	107	41	MNNKSQSVI	MNNKSQSVI	29866.31	41	29866.31	41	TRUE	TRUE
1	PLGINITNF	223	231	9	HLA-A*02:01	223	41	PLGINITNF	PLGINITNF	29883.44	41	29883.44	41	TRUE	TRUE
1	PFKDGIYFA	82	90	9	HLA-A*02:01	82	41	PFKDGIYFA	PFKDGIYFA	29891.53	41	29891.53	41	TRUE	TRUE
1	EGVFVFNGT	1074	1082	9	HLA-A*02:01	1074	41	EGVFVFNGT	EGVFVFNGT	29937.16	41	29937.16	41	TRUE	TRUE
1	VNFNFNGLT	525	533	9	HLA-A*02:01	525	41	VNFNFNGLT	VNFNFNGLT	29942.66	41	29942.66	41	TRUE	TRUE
1	FGEVFNATK	325	333	9	HLA-A*02:01	325	41	FGEVFNATK	FGEVFNATK	29958.87	41	29958.87	41	TRUE	TRUE
1	VADYSVLYN	349	357	9	HLA-A*02:01	349	41	VADYSVLYN	VADYSVLYN	29960.16	41	29960.16	41	TRUE	TRUE
1	DFMGCVLAW	415	423	9	HLA-A*02:01	415	41	DFMGCVLAW	DFMGCVLAW	29995.52	41	29995.52	41	TRUE	TRUE
1	GGVSVITPG	579	587	9	HLA-A*02:01	579	42	GGVSVITPG	GGVSVITPG	30084.58	42	30084.58	42	TRUE	TRUE
1	CFSNVYADS	378	386	9	HLA-A*02:01	378	42	CFSNVYADS	CFSNVYADS	30138.33	42	30138.33	42	TRUE	TRUE
1	VGYLKPTTF	254	262	9	HLA-A*02:01	254	42	VGYLKPTTF	VGYLKPTTF	30171.29	42	30171.29	42	TRUE	TRUE
1	EIRASANLA	999	1007	9	HLA-A*02:01	999	42	EIRASANLA	EIRASANLA	30191.87	42	30191.87	42	TRUE	TRUE
1	FGGVSVITP	578	586	9	HLA-A*02:01	578	42	FGGVSVITP	FGGVSVITP	30202.97	42	30202.97	42	TRUE	TRUE
1	DSEPVLKGV	1242	1250	9	HLA-A*02:01	1242	42	DSEPVLKGV	DSEPVLKGV	30287.74	42	30287.74	42	TRUE	TRUE
1	VVRDLPSGF	205	213	9	HLA-A*02:01	205	42	VVRDLPSGF	VVRDLPSGF	30295.26	42	30295.26	42	TRUE	TRUE
1	CYWPLNDYG	474	482	9	HLA-A*02:01	474	42	CYWPLNDYG	CYWPLNDYG	30325.11	42	30325.11	42	TRUE	TRUE
1	TTAPAICHE	1058	1066	9	HLA-A*02:01	1058	42	TTAPAICHE	TTAPAICHE	30327.42	42	30327.42	42	TRUE	TRUE
1	RSTSQKSIV	667	675	9	HLA-A*02:01	667	42	RSTSQKSIV	RSTSQKSIV	30351.05	42	30351.05	42	TRUE	TRUE
1	TVSLLRSTS	662	670	9	HLA-A*02:01	662	42	TVSLLRSTS	TVSLLRSTS	30361.23	42	30361.23	42	TRUE	TRUE
1	FKHLREFVF	179	187	9	HLA-A*02:01	179	42	FKHLREFVF	FKHLREFVF	30386.85	42	30386.85	42	TRUE	TRUE
1	KFPSVYAWE	333	341	9	HLA-A*02:01	333	42	KFPSVYAWE	KFPSVYAWE	30392.46	42	30392.46	42	TRUE	TRUE
1	IQESLTTTS	916	924	9	HLA-A*02:01	916	42	IQESLTTTS	IQESLTTTS	30405.93	42	30405.93	42	TRUE	TRUE
1	ATSTGNYNY	430	438	9	HLA-A*02:01	430	42	ATSTGNYNY	ATSTGNYNY	30409.55	42	30409.55	42	TRUE	TRUE
1	VYADSFVVK	382	390	9	HLA-A*02:01	382	42	VYADSFVVK	VYADSFVVK	30416.79	42	30416.79	42	TRUE	TRUE
1	LTGTGVLTP	532	540	9	HLA-A*02:01	532	42	LTGTGVLTP	LTGTGVLTP	30425.35	42	30425.35	42	TRUE	TRUE
1	IRASANLAA	1000	1008	9	HLA-A*02:01	1000	42	IRASANLAA	IRASANLAA	30462.91	42	30462.91	42	TRUE	TRUE
1	ISITTEVMP	702	710	9	HLA-A*02:01	702	42	ISITTEVMP	ISITTEVMP	30472.79	42	30472.79	42	TRUE	TRUE
1	VTQNVLYEN	893	901	9	HLA-A*02:01	893	43	VTQNVLYEN	VTQNVLYEN	30486.98	43	30486.98	43	TRUE	TRUE
1	VFQTQAGCL	628	636	9	HLA-A*02:01	628	43	VFQTQAGCL	VFQTQAGCL	30522.29	43	30522.29	43	TRUE	TRUE
1	TTTGIGYQP	485	493	9	HLA-A*02:01	485	43	TTTGIGYQP	TTTGIGYQP	30533.52	43	30533.52	43	TRUE	TRUE
1	SCLKGACSC	1224	1232	9	HLA-A*02:01	1224	43	SCLKGACSC	SCLKGACSC	30533.85	43	30533.85	43	TRUE	TRUE
1	LICAQKFNG	831	839	9	HLA-A*02:01	831	43	LICAQKFNG	LICAQKFNG	30576.16	43	30576.16	43	TRUE	TRUE
1	GKPCTPPAL	464	472	9	HLA-A*02:01	464	43	GKPCTPPAL	GKPCTPPAL	30577.5	43	30577.5	43	TRUE	TRUE
1	FSPQIITTD	1092	1100	9	HLA-A*02:01	1092	43	FSPQIITTD	FSPQIITTD	30708.78	43	30708.78	43	TRUE	TRUE
1	NVVIRACNF	122	130	9	HLA-A*02:01	122	43	NVVIRACNF	NVVIRACNF	30723.08	43	30723.08	43	TRUE	TRUE
1	YSTGNNVFQ	622	630	9	HLA-A*02:01	622	43	YSTGNNVFQ	YSTGNNVFQ	30727.74	43	30727.74	43	TRUE	TRUE
1	DISPCSFGG	572	580	9	HLA-A*02:01	572	43	DISPCSFGG	DISPCSFGG	30777.3	43	30777.3	43	TRUE	TRUE
1	NGLTGTGVL	530	538	9	HLA-A*02:01	530	43	NGLTGTGVL	NGLTGTGVL	30815.97	43	30815.97	43	TRUE	TRUE
1	LIDLQELGK	1179	1187	9	HLA-A*02:01	1179	43	LIDLQELGK	LIDLQELGK	30821.97	43	30821.97	43	TRUE	TRUE
1	FKCYGVSAT	364	372	9	HLA-A*02:01	364	43	FKCYGVSAT	FKCYGVSAT	30899.43	43	30899.43	43	TRUE	TRUE
1	STGNYNYKY	432	440	9	HLA-A*02:01	432	44	STGNYNYKY	STGNYNYKY	30949.95	44	30949.95	44	TRUE	TRUE
1	TLKYFGGFN	775	783	9	HLA-A*02:01	775	44	TLKYFGGFN	TLKYFGGFN	31013.98	44	31013.98	44	TRUE	TRUE
1	QTSNFRVVP	301	309	9	HLA-A*02:01	301	44	QTSNFRVVP	QTSNFRVVP	31024.39	44	31024.39	44	TRUE	TRUE
1	CVLAWNTRN	419	427	9	HLA-A*02:01	419	44	CVLAWNTRN	CVLAWNTRN	31039.16	44	31039.16	44	TRUE	TRUE
1	ENGTITDAV	268	276	9	HLA-A*02:01	268	44	ENGTITDAV	ENGTITDAV	31085.19	44	31085.19	44	TRUE	TRUE
1	NFRAILTAF	230	238	9	HLA-A*02:01	230	44	NFRAILTAF	NFRAILTAF	31120.87	44	31120.87	44	TRUE	TRUE
1	ICHEGKAYF	1063	1071	9	HLA-A*02:01	1063	44	ICHEGKAYF	ICHEGKAYF	31147.14	44	31147.14	44	TRUE	TRUE
1	SWFITQRNF	1083	1091	9	HLA-A*02:01	1083	44	SWFITQRNF	SWFITQRNF	31150.51	44	31150.51	44	TRUE	TRUE
1	QPIDVVRDL	201	209	9	HLA-A*02:01	201	44	QPIDVVRDL	QPIDVVRDL	31194.04	44	31194.04	44	TRUE	TRUE
1	SLDVSEKSG	169	177	9	HLA-A*02:01	169	44	SLDVSEKSG	SLDVSEKSG	31233.21	44	31233.21	44	TRUE	TRUE
1	KTSVDCNMY	715	723	9	HLA-A*02:01	715	44	KTSVDCNMY	KTSVDCNMY	31325.6	44	31325.6	44	TRUE	TRUE
1	SYHTVSLLR	659	667	9	HLA-A*02:01	659	45	SYHTVSLLR	SYHTVSLLR	31395.49	45	31395.49	45	TRUE	TRUE
1	QQFGRDVSD	549	557	9	HLA-A*02:01	549	45	QQFGRDVSD	QQFGRDVSD	31460.78	45	31460.78	45	TRUE	TRUE
1	FTTAPAICH	1057	1065	9	HLA-A*02:01	1057	45	FTTAPAICH	FTTAPAICH	31489.05	45	31489.05	45	TRUE	TRUE
1	DDFMGCVLA	414	422	9	HLA-A*02:01	414	45	DDFMGCVLA	DDFMGCVLA	31519.39	45	31519.39	45	TRUE	TRUE
1	FGAISSVLN	952	960	9	HLA-A*02:01	952	45	FGAISSVLN	FGAISSVLN	31522.46	45	31522.46	45	TRUE	TRUE
1	RFQPFQQFG	544	552	9	HLA-A*02:01	544	45	RFQPFQQFG	RFQPFQQFG	31527.91	45	31527.91	45	TRUE	TRUE
1	GDINARDLI	824	832	9	HLA-A*02:01	824	45	GDINARDLI	GDINARDLI	31552.13	45	31552.13	45	TRUE	TRUE
1	ITDAVDCSQ	272	280	9	HLA-A*02:01	272	45	ITDAVDCSQ	ITDAVDCSQ	31561.35	45	31561.35	45	TRUE	TRUE
1	LVKQLSSNF	944	952	9	HLA-A*02:01	944	45	LVKQLSSNF	LVKQLSSNF	31576.05	45	31576.05	45	TRUE	TRUE
1	IRACNFELC	125	133	9	HLA-A*02:01	125	45	IRACNFELC	IRACNFELC	31585.95	45	31585.95	45	TRUE	TRUE
1	KTPTLKYFG	772	780	9	HLA-A*02:01	772	45	KTPTLKYFG	KTPTLKYFG	31621.86	45	31621.86	45	TRUE	TRUE
1	KRFQPFQQF	543	551	9	HLA-A*02:01	543	45	KRFQPFQQF	KRFQPFQQF	31664.31	45	31664.31	45	TRUE	TRUE
1	GFMKQYGEC	814	822	9	HLA-A*02:01	814	45	GFMKQYGEC	GFMKQYGEC	31705.46	45	31705.46	45	TRUE	TRUE
1	TAIHADQLT	608	616	9	HLA-A*02:01	608	45	TAIHADQLT	TAIHADQLT	31719.53	45	31719.53	45	TRUE	TRUE
1	LTAFSPAQD	235	243	9	HLA-A*02:01	235	45	LTAFSPAQD	LTAFSPAQD	31740.81	45	31740.81	45	TRUE	TRUE
1	LADAGFMKQ	810	818	9	HLA-A*02:01	810	46	LADAGFMKQ	LADAGFMKQ	31763.48	46	31763.48	46	TRUE	TRUE
1	QAAPHGVVF	1036	1044	9	HLA-A*02:01	1036	46	QAAPHGVVF	QAAPHGVVF	31787.54	46	31787.54	46	TRUE	TRUE
1	KYDENGTIT	265	273	9	HLA-A*02:01	265	46	KYDENGTIT	KYDENGTIT	31790.98	46	31790.98	46	TRUE	TRUE
1	TEVMPVSMA	706	714	9	HLA-A*02:01	706	46	TEVMPVSMA	TEVMPVSMA	31792.71	46	31792.71	46	TRUE	TRUE
1	KGACSCGSC	1227	1235	9	HLA-A*02:01	1227	46	KGACSCGSC	KGACSCGSC	31861.24	46	31861.24	46	TRUE	TRUE
1	DDMIAAYTA	849	857	9	HLA-A*02:01	849	46	DDMIAAYTA	DDMIAAYTA	31861.92	46	31861.92	46	TRUE	TRUE
1	TGIGYQPYR	487	495	9	HLA-A*02:01	487	46	TGIGYQPYR	TGIGYQPYR	31867.78	46	31867.78	46	TRUE	TRUE
1	QSVIIINNS	112	120	9	HLA-A*02:01	112	46	QSVIIINNS	QSVIIINNS	31890.55	46	31890.55	46	TRUE	TRUE
1	YVPSQERNF	1049	1057	9	HLA-A*02:01	1049	46	YVPSQERNF	YVPSQERNF	31911.25	46	31911.25	46	TRUE	TRUE
1	QILPDPLKP	786	794	9	HLA-A*02:01	786	46	QILPDPLKP	QILPDPLKP	31916.44	46	31916.44	46	TRUE	TRUE
1	THTMIFDNA	148	156	9	HLA-A*02:01	148	46	THTMIFDNA	THTMIFDNA	31937.17	46	31937.17	46	TRUE	TRUE
1	DFCGKGYHL	1023	1031	9	HLA-A*02:01	1023	46	DFCGKGYHL	DFCGKGYHL	32003.58	46	32003.58	46	TRUE	TRUE
1	FSPAQDIWG	238	246	9	HLA-A*02:01	238	46	FSPAQDIWG	FSPAQDIWG	32025.41	46	32025.41	46	TRUE	TRUE
1	FEIDKGIYQ	293	301	9	HLA-A*02:01	293	46	FEIDKGIYQ	FEIDKGIYQ	32079.5	46	32079.5	46	TRUE	TRUE
1	FKNKDGFLY	187	195	9	HLA-A*02:01	187	47	FKNKDGFLY	FKNKDGFLY	32108.33	47	32108.33	47	TRUE	TRUE
1	PCSFGGVSV	575	583	9	HLA-A*02:01	575	47	PCSFGGVSV	PCSFGGVSV	32112.5	47	32112.5	47	TRUE	TRUE
1	CDNPFFAVS	133	141	9	HLA-A*02:01	133	47	CDNPFFAVS	CDNPFFAVS	32163.61	47	32163.61	47	TRUE	TRUE
1	VNCTDVSTA	601	609	9	HLA-A*02:01	601	47	VNCTDVSTA	VNCTDVSTA	32241.32	47	32241.32	47	TRUE	TRUE
1	DISNVPFSP	454	462	9	HLA-A*02:01	454	47	DISNVPFSP	DISNVPFSP	32247.93	47	32247.93	47	TRUE	TRUE
1	QTQAGCLIG	630	638	9	HLA-A*02:01	630	47	QTQAGCLIG	QTQAGCLIG	32311.86	47	32311.86	47	TRUE	TRUE
1	LGADSSIAY	681	689	9	HLA-A*02:01	681	47	LGADSSIAY	LGADSSIAY	32326.18	47	32326.18	47	TRUE	TRUE
1	GTGVLTPSS	534	542	9	HLA-A*02:01	534	47	GTGVLTPSS	GTGVLTPSS	32351.73	47	32351.73	47	TRUE	TRUE
1	LFNKVTLAD	804	812	9	HLA-A*02:01	804	47	LFNKVTLAD	LFNKVTLAD	32374.83	47	32374.83	47	TRUE	TRUE
1	SATKLNDLC	370	378	9	HLA-A*02:01	370	47	SATKLNDLC	SATKLNDLC	32387.11	47	32387.11	47	TRUE	TRUE
1	NAPATVCGP	505	513	9	HLA-A*02:01	505	47	NAPATVCGP	NAPATVCGP	32429.54	47	32429.54	47	TRUE	TRUE
1	VSDFTDSVR	555	563	9	HLA-A*02:01	555	47	VSDFTDSVR	VSDFTDSVR	32441.11	47	32441.11	47	TRUE	TRUE
1	VFVFNGTSW	1076	1084	9	HLA-A*02:01	1076	48	VFVFNGTSW	VFVFNGTSW	32507.52	48	32507.52	48	TRUE	TRUE
1	QYGECLGDI	818	826	9	HLA-A*02:01	818	48	QYGECLGDI	QYGECLGDI	32543.77	48	32543.77	48	TRUE	TRUE
1	QIDRLITGR	974	982	9	HLA-A*02:01	974	48	QIDRLITGR	QIDRLITGR	32549.05	48	32549.05	48	TRUE	TRUE
1	SPDVDLGDI	1143	1151	9	HLA-A*02:01	1143	48	SPDVDLGDI	SPDVDLGDI	32568.08	48	32568.08	48	TRUE	TRUE
1	LPFYSNVTG	60	68	9	HLA-A*02:01	60	48	LPFYSNVTG	LPFYSNVTG	32573.37	48	32573.37	48	TRUE	TRUE
1	TAGWTFGAG	865	873	9	HLA-A*02:01	865	48	TAGWTFGAG	TAGWTFGAG	32582.17	48	32582.17	48	TRUE	TRUE
1	NFELCDNPF	129	137	9	HLA-A*02:01	129	48	NFELCDNPF	NFELCDNPF	32612.87	48	32612.87	48	TRUE	TRUE

seq #	peptide	start	end	peptide length	allele	peptide index	median binding	netmhcpan_ba	netmhcpan_ba	netmhcpan_ba	netmhcpan_ba	legacy score	legacy rank	scores equal	percentiles equal
1	LPPLLTDDM	843	851	9	HLA-A*02:01	843	48	LPPLLTDDM	LPPLLTDDM	32657	48	32657	48	TRUE	TRUE
1	CTDVSTAIH	603	611	9	HLA-A*02:01	603	48	CTDVSTAIH	CTDVSTAIH	32707.57	48	32707.57	48	TRUE	TRUE
1	IGAEHVDTS	637	645	9	HLA-A*02:01	637	48	IGAEHVDTS	IGAEHVDTS	32709.7	48	32709.7	48	TRUE	TRUE
1	QDLFLPFYS	56	64	9	HLA-A*02:01	56	48	QDLFLPFYS	QDLFLPFYS	32715.71	48	32715.71	48	TRUE	TRUE
1	TITDAVDCS	271	279	9	HLA-A*02:01	271	48	TITDAVDCS	TITDAVDCS	32729.15	48	3.27E+04	48	TRUE	TRUE
1	TSCCSCLKG	1220	1228	9	HLA-A*02:01	1220	48	TSCCSCLKG	TSCCSCLKG	32731.64	48	3.27E+04	48	TRUE	TRUE
1	LSRLDKVEA	963	971	9	HLA-A*02:01	963	49	LSRLDKVEA	LSRLDKVEA	32927.72	49	3.29E+04	49	TRUE	TRUE
1	PIGAGICAS	651	659	9	HLA-A*02:01	651	49	PIGAGICAS	PIGAGICAS	32965.13	49	3.30E+04	49	TRUE	TRUE
1	YKGYQPIDV	197	205	9	HLA-A*02:01	197	49	YKGYQPIDV	YKGYQPIDV	32971.55	49	3.30E+04	49	TRUE	TRUE
1	EIFRSDTLY	45	53	9	HLA-A*02:01	45	49	EIFRSDTLY	EIFRSDTLY	33016.2	49	3.30E+04	49	TRUE	TRUE
1	VFKNKDGFL	186	194	9	HLA-A*02:01	186	49	VFKNKDGFL	VFKNKDGFL	33030.12	49	3.30E+04	49	TRUE	TRUE
1	KSQSVIIIN	110	118	9	HLA-A*02:01	110	49	KSQSVIIIN	KSQSVIIIN	33053.35	49	3.31E+04	49	TRUE	TRUE
1	AVSKPMGTQ	139	147	9	HLA-A*02:01	139	49	AVSKPMGTQ	AVSKPMGTQ	33066.59	49	3.31E+04	49	TRUE	TRUE
1	AYFVGYLKP	251	259	9	HLA-A*02:01	251	49	AYFVGYLKP	AYFVGYLKP	33173.39	49	3.32E+04	49	TRUE	TRUE
1	NHTFGNPVI	73	81	9	HLA-A*02:01	73	49	NHTFGNPVI	NHTFGNPVI	33228.7	49	3.32E+04	49	TRUE	TRUE
1	TAPAICHEG	1059	1067	9	HLA-A*02:01	1059	50	TAPAICHEG	TAPAICHEG	33252.79	50	3.33E+04	50	TRUE	TRUE
1	TFFSTFKCY	359	367	9	HLA-A*02:01	359	50	TFFSTFKCY	TFFSTFKCY	33256.39	50	3.33E+04	50	TRUE	TRUE
1	PFYSNVTGF	61	69	9	HLA-A*02:01	61	50	PFYSNVTGF	PFYSNVTGF	33272.59	50	3.33E+04	50	TRUE	TRUE
1	TFDDVQAPN	21	29	9	HLA-A*02:01	21	50	TFDDVQAPN	TFDDVQAPN	33273.32	50	3.33E+04	50	TRUE	TRUE
1	IYFAATEKS	87	95	9	HLA-A*02:01	87	50	IYFAATEKS	IYFAATEKS	33287.36	50	3.33E+04	50	TRUE	TRUE
1	CYGVSATKL	366	374	9	HLA-A*02:01	366	50	CYGVSATKL	CYGVSATKL	33293.84	50	3.33E+04	50	TRUE	TRUE
1	CGPKLSTDL	511	519	9	HLA-A*02:01	511	50	CGPKLSTDL	CGPKLSTDL	33304.3	50	3.33E+04	50	TRUE	TRUE
1	LKYDENGTI	264	272	9	HLA-A*02:01	264	50	LKYDENGTI	LKYDENGTI	33307.18	50	3.33E+04	50	TRUE	TRUE
1	TTFMLKYDE	260	268	9	HLA-A*02:01	260	50	TTFMLKYDE	TTFMLKYDE	33328.79	50	3.33E+04	50	TRUE	TRUE
1	ELKCSVKSF	285	293	9	HLA-A*02:01	285	50	ELKCSVKSF	ELKCSVKSF	33332.4	50	3.33E+04	50	TRUE	TRUE
1	ANLLLQYGS	732	740	9	HLA-A*02:01	732	50	ANLLLQYGS	ANLLLQYGS	33354.42	50	3.34E+04	50	TRUE	TRUE
1	FFAVSKPMG	137	145	9	HLA-A*02:01	137	50	FFAVSKPMG	FFAVSKPMG	33445.84	50	3.34E+04	50	TRUE	TRUE
1	QHTSSMRGV	32	40	9	HLA-A*02:01	32	50	QHTSSMRGV	QHTSSMRGV	33467.57	50	3.35E+04	50	TRUE	TRUE
1	DVVNQNAQA	932	940	9	HLA-A*02:01	932	50	DVVNQNAQA	DVVNQNAQA	33521.94	50	3.35E+04	50	TRUE	TRUE
1	KGDDVRQIA	390	398	9	HLA-A*02:01	390	50	KGDDVRQIA	KGDDVRQIA	33541.16	50	3.35E+04	50	TRUE	TRUE
1	EVFNATKFP	327	335	9	HLA-A*02:01	327	50	EVFNATKFP	EVFNATKFP	33541.53	50	3.35E+04	50	TRUE	TRUE
1	AGFMKQYGE	813	821	9	HLA-A*02:01	813	50	AGFMKQYGE	AGFMKQYGE	33567.65	50	3.36E+04	50	TRUE	TRUE
1	KHLREFVFK	180	188	9	HLA-A*02:01	180	51	KHLREFVFK	KHLREFVFK	33648.02	51	3.36E+04	51	TRUE	TRUE
1	IADYNYKLP	405	413	9	HLA-A*02:01	405	51	IADYNYKLP	IADYNYKLP	33674.98	51	3.37E+04	51	TRUE	TRUE
1	PTLKYFGGF	774	782	9	HLA-A*02:01	774	51	PTLKYFGGF	PTLKYFGGF	33683.36	51	3.37E+04	51	TRUE	TRUE
1	TDDMIAAYT	848	856	9	HLA-A*02:01	848	51	TDDMIAAYT	TDDMIAAYT	33740.62	51	3.37E+04	51	TRUE	TRUE
1	SVKSFEIDK	289	297	9	HLA-A*02:01	289	51	SVKSFEIDK	SVKSFEIDK	33762.52	51	3.38E+04	51	TRUE	TRUE
1	NYNYKYRYL	435	443	9	HLA-A*02:01	435	51	NYNYKYRYL	NYNYKYRYL	33787.01	51	3.38E+04	51	TRUE	TRUE
1	GAISSVLND	953	961	9	HLA-A*02:01	953	51	GAISSVLND	GAISSVLND	33854.34	51	3.39E+04	51	TRUE	TRUE
1	YGSFCTQLN	738	746	9	HLA-A*02:01	738	51	YGSFCTQLN	YGSFCTQLN	33877.79	51	3.39E+04	51	TRUE	TRUE
1	FYTTTGIGY	483	491	9	HLA-A*02:01	483	51	FYTTTGIGY	FYTTTGIGY	33888.44	51	3.39E+04	51	TRUE	TRUE
1	TATAGWTFG	863	871	9	HLA-A*02:01	863	51	TATAGWTFG	TATAGWTFG	33894.29	51	3.39E+04	51	TRUE	TRUE
1	ERNFTTAPA	1054	1062	9	HLA-A*02:01	1054	52	ERNFTTAPA	ERNFTTAPA	33974.7	52	3.40E+04	52	TRUE	TRUE
1	KSGNFKHLR	175	183	9	HLA-A*02:01	175	52	KSGNFKHLR	KSGNFKHLR	34049.04	52	3.40E+04	52	TRUE	TRUE
1	KPIFKLPLG	217	225	9	HLA-A*02:01	217	52	KPIFKLPLG	KPIFKLPLG	34056.41	52	3.41E+04	52	TRUE	TRUE
1	MYICGDSTE	722	730	9	HLA-A*02:01	722	52	MYICGDSTE	MYICGDSTE	34116.89	52	3.41E+04	52	TRUE	TRUE
1	QPYRVVVLS	492	500	9	HLA-A*02:01	492	52	QPYRVVVLS	QPYRVVVLS	34126.13	52	3.41E+04	52	TRUE	TRUE
1	YQPIDVVRD	200	208	9	HLA-A*02:01	200	52	YQPIDVVRD	YQPIDVVRD	34131.66	52	3.41E+04	52	TRUE	TRUE
1	VSMAKTSVD	711	719	9	HLA-A*02:01	711	52	VSMAKTSVD	VSMAKTSVD	34137.2	52	3.41E+04	52	TRUE	TRUE
1	ICASYHTVS	656	664	9	HLA-A*02:01	656	52	ICASYHTVS	ICASYHTVS	34141.63	52	3.41E+04	52	TRUE	TRUE
1	SNVVRGWVF	95	103	9	HLA-A*02:01	95	52	SNVVRGWVF	SNVVRGWVF	34168.61	52	3.42E+04	52	TRUE	TRUE
1	LAWNTRNID	421	429	9	HLA-A*02:01	421	52	LAWNTRNID	LAWNTRNID	34192.29	52	3.42E+04	52	TRUE	TRUE
1	QCVNFNFNG	523	531	9	HLA-A*02:01	523	52	QCVNFNFNG	QCVNFNFNG	34198.95	52	3.42E+04	52	TRUE	TRUE
1	YRVVVLSFE	494	502	9	HLA-A*02:01	494	52	YRVVVLSFE	YRVVVLSFE	34221.14	52	3.42E+04	52	TRUE	TRUE
1	TSTGNYNYK	431	439	9	HLA-A*02:01	431	52	TSTGNYNYK	TSTGNYNYK	34240.04	52	3.42E+04	52	TRUE	TRUE
1	SDLDRCTTF	14	22	9	HLA-A*02:01	14	53	SDLDRCTTF	SDLDRCTTF	34257.82	53	3.43E+04	53	TRUE	TRUE
1	ATVCGPKLS	508	516	9	HLA-A*02:01	508	53	ATVCGPKLS	ATVCGPKLS	34294.54	53	3.43E+04	53	TRUE	TRUE
1	CSCLKGACS	1223	1231	9	HLA-A*02:01	1223	53	CSCLKGACS	CSCLKGACS	34343.56	53	3.43E+04	53	TRUE	TRUE
1	DVNCTDVST	600	608	9	HLA-A*02:01	600	53	DVNCTDVST	DVNCTDVST	34370.7	53	3.44E+04	53	TRUE	TRUE
1	RSDTLYLTQ	48	56	9	HLA-A*02:01	48	53	RSDTLYLTQ	RSDTLYLTQ	34376.27	53	3.44E+04	53	TRUE	TRUE
1	TTTSTALGK	921	929	9	HLA-A*02:01	921	53	TTTSTALGK	TTTSTALGK	34397.49	53	3.44E+04	53	TRUE	TRUE
1	KGIYQTSNF	297	305	9	HLA-A*02:01	297	53	KGIYQTSNF	KGIYQTSNF	34400.46	53	3.44E+04	53	TRUE	TRUE
1	VQAPNYTQH	25	33	9	HLA-A*02:01	25	53	VQAPNYTQH	VQAPNYTQH	34406.78	53	3.44E+04	53	TRUE	TRUE
1	FRVVPSGDV	305	313	9	HLA-A*02:01	305	53	FRVVPSGDV	FRVVPSGDV	34455.21	53	3.45E+04	53	TRUE	TRUE
1	FYSNVTGFH	62	70	9	HLA-A*02:01	62	53	FYSNVTGFH	FYSNVTGFH	34457.46	53	3.45E+04	53	TRUE	TRUE
1	EQYIKWPWY	1189	1197	9	HLA-A*02:01	1189	53	EQYIKWPWY	EQYIKWPWY	34484.31	53	3.45E+04	53	TRUE	TRUE
1	RDLPSGFNT	207	215	9	HLA-A*02:01	207	53	RDLPSGFNT	RDLPSGFNT	34528.35	53	3.45E+04	53	TRUE	TRUE
1	TDLIKNQCV	517	525	9	HLA-A*02:01	517	53	TDLIKNQCV	TDLIKNQCV	34564.62	53	3.46E+04	53	TRUE	TRUE
1	ISQIQESLT	913	921	9	HLA-A*02:01	913	54	ISQIQESLT	ISQIQESLT	34566.11	54	3.46E+04	54	TRUE	TRUE
1	AYFPREGVF	1069	1077	9	HLA-A*02:01	1069	54	AYFPREGVF	AYFPREGVF	34583.33	54	3.46E+04	54	TRUE	TRUE
1	RACNFELCD	126	134	9	HLA-A*02:01	126	54	RACNFELCD	RACNFELCD	34608.41	54	3.46E+04	54	TRUE	TRUE
1	KDGIYFAAT	84	92	9	HLA-A*02:01	84	54	KDGIYFAAT	KDGIYFAAT	34630.13	54	3.46E+04	54	TRUE	TRUE
1	VVPSGDVVR	307	315	9	HLA-A*02:01	307	54	VVPSGDVVR	VVPSGDVVR	34704.76	54	3.47E+04	54	TRUE	TRUE
1	GWTFGAGAA	867	875	9	HLA-A*02:01	867	54	GWTFGAGAA	GWTFGAGAA	34712.28	54	3.47E+04	54	TRUE	TRUE
1	ATKLNDLCF	371	379	9	HLA-A*02:01	371	54	ATKLNDLCF	ATKLNDLCF	34753.63	54	3.48E+04	54	TRUE	TRUE
1	IYSTGNNVF	621	629	9	HLA-A*02:01	621	54	IYSTGNNVF	IYSTGNNVF	34777.3	54	3.48E+04	54	TRUE	TRUE
1	ICGDSTECA	724	732	9	HLA-A*02:01	724	54	ICGDSTECA	ICGDSTECA	34882.83	54	3.49E+04	54	TRUE	TRUE
1	DLDRCTTFD	15	23	9	HLA-A*02:01	15	54	DLDRCTTFD	DLDRCTTFD	34890.39	54	3.49E+04	54	TRUE	TRUE
1	VFAQVKQMY	763	771	9	HLA-A*02:01	763	55	VFAQVKQMY	VFAQVKQMY	34984.12	55	3.50E+04	55	TRUE	TRUE
1	SGTATAGWT	861	869	9	HLA-A*02:01	861	55	SGTATAGWT	SGTATAGWT	34994.73	55	3.50E+04	55	TRUE	TRUE
1	VIPFKDGIY	80	88	9	HLA-A*02:01	80	55	VIPFKDGIY	VIPFKDGIY	34996.62	55	3.50E+04	55	TRUE	TRUE
1	PHGVVFLHV	1039	1047	9	HLA-A*02:01	1039	55	PHGVVFLHV	PHGVVFLHV	35024.67	55	3.50E+04	55	TRUE	TRUE
1	GSDLDRCTT	13	21	9	HLA-A*02:01	13	55	GSDLDRCTT	GSDLDRCTT	35044.75	55	3.50E+04	55	TRUE	TRUE
1	GNFKHLREF	177	185	9	HLA-A*02:01	177	55	GNFKHLREF	GNFKHLREF	35109.65	55	3.51E+04	55	TRUE	TRUE
1	FGSTMNNKS	103	111	9	HLA-A*02:01	103	55	FGSTMNNKS	FGSTMNNKS	35157.91	55	3.52E+04	55	TRUE	TRUE
1	LPSGFNTLK	209	217	9	HLA-A*02:01	209	55	LPSGFNTLK	LPSGFNTLK	35168.57	55	3.52E+04	55	TRUE	TRUE
1	DEIFRSDTL	44	52	9	HLA-A*02:01	44	55	DEIFRSDTL	DEIFRSDTL	35175.04	55	3.52E+04	55	TRUE	TRUE
1	GVLTPSSKR	536	544	9	HLA-A*02:01	536	56	GVLTPSSKR	GVLTPSSKR	35221.12	56	3.52E+04	56	TRUE	TRUE
1	NQKQIANQF	901	909	9	HLA-A*02:01	901	56	NQKQIANQF	NQKQIANQF	35238.66	56	3.52E+04	56	TRUE	TRUE
1	YHTVSLLRS	660	668	9	HLA-A*02:01	660	56	YHTVSLLRS	YHTVSLLRS	35347.5	56	3.53E+04	56	TRUE	TRUE
1	GFNTLKPIF	212	220	9	HLA-A*02:01	212	56	GFNTLKPIF	GFNTLKPIF	35357.83	56	3.54E+04	56	TRUE	TRUE
1	TSWFITQRN	1082	1090	9	HLA-A*02:01	1082	56	TSWFITQRN	TSWFITQRN	35381.55	56	3.54E+04	56	TRUE	TRUE
1	NCYWPLNDY	473	481	9	HLA-A*02:01	473	56	NCYWPLNDY	NCYWPLNDY	35392.28	56	3.54E+04	56	TRUE	TRUE
1	NKSQSVIII	109	117	9	HLA-A*02:01	109	56	NKSQSVIII	NKSQSVIII	35397.62	56	3.54E+04	56	TRUE	TRUE
1	IDRLITGRL	975	983	9	HLA-A*02:01	975	56	IDRLITGRL	IDRLITGRL	35428.66	56	3.54E+04	56	TRUE	TRUE
1	YKTPTLKYF	771	779	9	HLA-A*02:01	771	56	YKTPTLKYF	YKTPTLKYF	35447.83	56	3.54E+04	56	TRUE	TRUE
1	CNMYICGDS	720	728	9	HLA-A*02:01	720	56	CNMYICGDS	CNMYICGDS	35461.64	56	3.55E+04	56	TRUE	TRUE
1	AFSPAQDIW	237	245	9	HLA-A*02:01	237	56	AFSPAQDIW	AFSPAQDIW	35470.47	56	3.55E+04	56	TRUE	TRUE
1	TDVSTAIHA	604	612	9	HLA-A*02:01	604	56	TDVSTAIHA	TDVSTAIHA	35475.85	56	3.55E+04	56	TRUE	TRUE
1	NTRNIDATS	424	432	9	HLA-A*02:01	424	56	NTRNIDATS	NTRNIDATS	35499.26	56	3.55E+04	56	TRUE	TRUE
1	GNYNYKYRY	434	442	9	HLA-A*02:01	434	56	GNYNYKYRY	GNYNYKYRY	35518.47	56	3.55E+04	56	TRUE	TRUE
1	VSGTATAGW	860	868	9	HLA-A*02:01	860	57	VSGTATAGW	VSGTATAGW	35562.71	57	3.56E+04	57	TRUE	TRUE
1	GPKLSTDLI	512	520	9	HLA-A*02:01	512	57	GPKLSTDLI	GPKLSTDLI	35566.17	57	3.56E+04	57	TRUE	TRUE
1	AYTAALVSG	854	862	9	HLA-A*02:01	854	57	AYTAALVSG	AYTAALVSG	35576.56	57	3.56E+04	57	TRUE	TRUE
1	GEVFNATKF	326	334	9	HLA-A*02:01	326	57	GEVFNATKF	GEVFNATKF	35577.32	57	3.56E+04	57	TRUE	TRUE
1	RFNGIGVTQ	887	895	9	HLA-A*02:01	887	57	RFNGIGVTQ	RFNGIGVTQ	35578.86	57	3.56E+04	57	TRUE	TRUE
1	FNTLKPIFK	213	221	9	HLA-A*02:01	213	57	FNTLKPIFK	FNTLKPIFK	35608.91	57	3.56E+04	57	TRUE	TRUE
1	LKPTTFMLK	257	265	9	HLA-A*02:01	257	57	LKPTTFMLK	LKPTTFMLK	35611.23	57	3.56E+04	57	TRUE	TRUE
1	SNFRVVPSG	303	311	9	HLA-A*02:01	303	57	SNFRVVPSG	SNFRVVPSG	35622.78	57	3.56E+04	57	TRUE	TRUE
1	AFSLDVSEK	167	175	9	HLA-A*02:01	167	57	AFSLDVSEK	AFSLDVSEK	35630.11	57	3.56E+04	57	TRUE	TRUE
1	YPDEIFRSD	42	50	9	HLA-A*02:01	42	57	YPDEIFRSD	YPDEIFRSD	35635.88	57	3.56E+04	57	TRUE	TRUE
1	NKDGFLYVY	189	197	9	HLA-A*02:01	189	57	NKDGFLYVY	NKDGFLYVY	35649	57	3.56E+04	57	TRUE	TRUE
1	GDSTECANL	726	734	9	HLA-A*02:01	726	57	GDSTECANL	GDSTECANL	35680.26	57	3.57E+04	57	TRUE	TRUE
1	REGVFVFNG	1073	1081	9	HLA-A*02:01	1073	57	REGVFVFNG	REGVFVFNG	35714.24	57	3.57E+04	57	TRUE	TRUE
1	HTSPDVDLG	1141	1149	9	HLA-A*02:01	1141	57	HTSPDVDLG	HTSPDVDLG	35736.27	57	3.57E+04	57	TRUE	TRUE

seq #	peptide	start	end	peptide length	allele	peptide index	median binding	netmhcpan_ba	netmhcpan_ba	netmhcpan_ba	netmhcpan_ba	legacy score	legacy rank	scores equal	percentiles equal	
1	ARDLICAQK	828	836	9	HLA-A*02:01	828	57	ARDLICAQK	ARDLICAQK	35753.67	57	3.58E+04	57	TRUE	TRUE	
1	HVTYVPSQE	1046	1054	9	HLA-A*02:01	1046	57	HVTYVPSQE	HVTYVPSQE	35754.84	57	3.58E+04	57	TRUE	TRUE	
1	STMNNKSQS	105	113	9	HLA-A*02:01	105	57	STMNNKSQS	STMNNKSQS	35759.48	57	3.58E+04	57	TRUE	TRUE	
1	TSSMRGVYY	34	42	9	HLA-A*02:01	34	57	TSSMRGVYY	TSSMRGVYY	35777.68	57	3.58E+04	57	TRUE	TRUE	
1	PFGEVFNAT	324	332	9	HLA-A*02:01	324	57	PFGEVFNAT	PFGEVFNAT	35833.07	57	3.58E+04	57	TRUE	TRUE	
1	NASVVNIQK	1155	1163	9	HLA-A*02:01	1155	57	NASVVNIQK	NASVVNIQK	35833.47	57	3.58E+04	57	TRUE	TRUE	
1	DEDDSEPVL	1239	1247	9	HLA-A*02:01	1239	58	DEDDSEPVL	DEDDSEPVL	35852.08	58	3.59E+04	58	TRUE	TRUE	
1	CNFELCDNP	128	136	9	HLA-A*02:01	128	58	CNFELCDNP	CNFELCDNP	35885.06	58	3.59E+04	58	TRUE	TRUE	
1	VDTSYECDI	642	650	9	HLA-A*02:01	642	58	VDTSYECDI	VDTSYECDI	35897.1	58	3.59E+04	58	TRUE	TRUE	
1	HTSSMRGVY	33	41	9	HLA-A*02:01	33	58	HTSSMRGVY	HTSSMRGVY	35897.49	58	3.59E+04	58	TRUE	TRUE	
1	TKFPSVYAW	332	340	9	HLA-A*02:01	332	58	TKFPSVYAW	TKFPSVYAW	35925.07	58	3.59E+04	58	TRUE	TRUE	
1	IGVTQNVLY	891	899	9	HLA-A*02:01	891	58	IGVTQNVLY	IGVTQNVLY	35972.53	58	3.60E+04	58	TRUE	TRUE	
1	VVRFPNITN	313	321	9	HLA-A*02:01	313	58	VVRFPNITN	VVRFPNITN	35981.86	58	3.60E+04	58	TRUE	TRUE	
1	QAPNYTQHT	26	34	9	HLA-A*02:01	26	58	QAPNYTQHT	QAPNYTQHT	36010.3	58	3.60E+04	58	TRUE	TRUE	
1	ELDKYFKNH	1133	1141	9	HLA-A*02:01	1133	58	ELDKYFKNH	ELDKYFKNH	36011.85	58	3.60E+04	58	TRUE	TRUE	
1	NQCVNFNFN	522	530	9	HLA-A*02:01	522	58	NQCVNFNFN	NQCVNFNFN	36018.1	58	3.60E+04	58	TRUE	TRUE	
1	FKEELDKYF	1130	1138	9	HLA-A*02:01	1130	58	FKEELDKYF	FKEELDKYF	36030.18	58	3.60E+04	58	TRUE	TRUE	
1	NFSQILPDP	783	791	9	HLA-A*02:01	783	58	NFSQILPDP	NFSQILPDP	36035.25	58	3.60E+04	58	TRUE	TRUE	
1	NRALSGIAA	746	754	9	HLA-A*02:01	746	58	NRALSGIAA	NRALSGIAA	36038.36	58	3.60E+04	58	TRUE	TRUE	
1	GFYTTTGIG	482	490	9	HLA-A*02:01	482	58	GFYTTTGIG	GFYTTTGIG	36077.78	58	3.61E+04	58	TRUE	TRUE	
1	LCCMTSCCS	1216	1224	9	HLA-A*02:01	1216	58	LCCMTSCCS	LCCMTSCCS	36078.15	58	3.61E+04	58	TRUE	TRUE	
1	CSVKSFEID	288	296	9	HLA-A*02:01	288	58	CSVKSFEID	CSVKSFEID	36083.62	58	3.61E+04	58	TRUE	TRUE	
1	PNITNLCPF	317	325	9	HLA-A*02:01	317	58	PNITNLCPF	PNITNLCPF	36126.6	58	3.61E+04	58	TRUE	TRUE	
1	LTSGSDLDR	10	18	9	HLA-A*02:01	10	59	LTSGSDLDR	LTSGSDLDR	36159.45	59	3.62E+04	59	TRUE	TRUE	
1	ERDISNVPF	452	460	9	HLA-A*02:01	452	59	ERDISNVPF	ERDISNVPF	36164.93	59	3.62E+04	59	TRUE	TRUE	
1	SEVAVLYQD	592	600	9	HLA-A*02:01	592	59	SEVAVLYQD	SEVAVLYQD	36175.09	59	3.62E+04	59	TRUE	TRUE	
1	LDISPCSFG	571	579	9	HLA-A*02:01	571	59	LDISPCSFG	LDISPCSFG	36212.3	59	3.62E+04	59	TRUE	TRUE	
1	AKTSVDCNM	714	722	9	HLA-A*02:01	714	59	AKTSVDCNM	AKTSVDCNM	36266.01	59	3.63E+04	59	TRUE	TRUE	
1	AKNLNESLI	1172	1180	9	HLA-A*02:01	1172	59	AKNLNESLI	AKNLNESLI	36267.97	59	3.63E+04	59	TRUE	TRUE	
1	APATVCGPK	506	514	9	HLA-A*02:01	506	59	APATVCGPK	APATVCGPK	36336.34	59	3.63E+04	59	TRUE	TRUE	
1	SGDVVRFPN	310	318	9	HLA-A*02:01	310	59	SGDVVRFPN	SGDVVRFPN	36353.63	59	3.64E+04	59	TRUE	TRUE	
1	TSQKSIVAY	669	677	9	HLA-A*02:01	669	59	TSQKSIVAY	TSQKSIVAY	36366.22	59	3.64E+04	59	TRUE	TRUE	
1	DVVRFPNIT	312	320	9	HLA-A*02:01	312	59	DVVRFPNIT	DVVRFPNIT	36407.15	59	3.64E+04	59	TRUE	TRUE	
1	KYFKNHTSP	1136	1144	9	HLA-A*02:01	1136	59	KYFKNHTSP	KYFKNHTSP	36409.92	59	3.64E+04	59	TRUE	TRUE	
1	TKLNDLCFS	372	380	9	HLA-A*02:01	372	59	TKLNDLCFS	TKLNDLCFS	36419.76	59	3.64E+04	59	TRUE	TRUE	
1	GYQPIDVVR	199	207	9	HLA-A*02:01	199	60	GYQPIDVVR	GYQPIDVVR	36463.54	60	3.65E+04	60	TRUE	TRUE	
1	NFTTAPAIC	1056	1064	9	HLA-A*02:01	1056	60	NFTTAPAIC	NFTTAPAIC	36469.85	60	3.65E+04	60	TRUE	TRUE	
1	GYHLMSFPQ	1028	1036	9	HLA-A*02:01	1028	60	GYHLMSFPQ	GYHLMSFPQ	36495.52	60	3.65E+04	60	TRUE	TRUE	
1	IGIINNTVY	1112	1120	9	HLA-A*02:01	1112	60	IGIINNTVY	IGIINNTVY	36497.47	60	3.65E+04	60	TRUE	TRUE	
1	GNNVFQTQA	625	633	9	HLA-A*02:01	625	60	GNNVFQTQA	GNNVFQTQA	36524.73	60	3.65E+04	60	TRUE	TRUE	
1	NIDATSTGN	427	435	9	HLA-A*02:01	427	60	NIDATSTGN	NIDATSTGN	36525.14	60	3.65E+04	60	TRUE	TRUE	
1	VIIINNSTN	114	122	9	HLA-A*02:01	114	60	VIIINNSTN	VIIINNSTN	36552.4	60	3.66E+04	60	TRUE	TRUE	
1	DGKPCTPPA	463	471	9	HLA-A*02:01	463	60	DGKPCTPPA	DGKPCTPPA	36561.9	60	3.66E+04	60	TRUE	TRUE	
1	LAELKCSVK	283	291	9	HLA-A*02:01	283	60	LAELKCSVK	LAELKCSVK	36641.9	60	3.66E+04	60	TRUE	TRUE	
1	VPSGDVVRF	308	316	9	HLA-A*02:01	308	60	VPSGDVVRF	VPSGDVVRF	36706.99	60	3.67E+04	60	TRUE	TRUE	
1	SFPQAAPHG	1033	1041	9	HLA-A*02:01	1033	60	SFPQAAPHG	SFPQAAPHG	36712.93	60	3.67E+04	60	TRUE	TRUE	
1	AICHEGKAY	1062	1070	9	HLA-A*02:01	1062	60	AICHEGKAY	AICHEGKAY	36715.72	60	3.67E+04	60	TRUE	TRUE	
1	IWGTSAAAY	244	252	9	HLA-A*02:01	244	61	IWGTSAAAY	IWGTSAAAY	36779.73	61	3.68E+04	61	TRUE	TRUE	
1	NGTSWFITQ	1080	1088	9	HLA-A*02:01	1080	61	NGTSWFITQ	NGTSWFITQ	36785.71	61	3.68E+04	61	TRUE	TRUE	
1	GFLYVYKGY	192	200	9	HLA-A*02:01	192	61	GFLYVYKGY	GFLYVYKGY	36833.5	61	3.68E+04	61	TRUE	TRUE	
1	GCVLAWNTR	418	426	9	HLA-A*02:01	418	61	GCVLAWNTR	GCVLAWNTR	36834.69	61	3.68E+04	61	TRUE	TRUE	
1	VFGSTMNNK	102	110	9	HLA-A*02:01	102	61	VFGSTMNNK	VFGSTMNNK	36853.43	61	3.69E+04	61	TRUE	TRUE	
1	DGFLYVYKG	191	199	9	HLA-A*02:01	191	61	DGFLYVYKG	DGFLYVYKG	36879.75	61	3.69E+04	61	TRUE	TRUE	
1	PATVCGPKL	507	515	9	HLA-A*02:01	507	61	PATVCGPKL	PATVCGPKL	36892.12	61	3.69E+04	61	TRUE	TRUE	
1	GDISGINAS	1149	1157	9	HLA-A*02:01	1149	61	GDISGINAS	GDISGINAS	36909.28	61	3.69E+04	61	TRUE	TRUE	
1	NHTSPDVDL	1140	1148	9	HLA-A*02:01	1140	61	NHTSPDVDL	NHTSPDVDL	36948.05	61	3.69E+04	61	TRUE	TRUE	
1	HEGKAYFPR	1065	1073	9	HLA-A*02:01	1065	61	HEGKAYFPR	HEGKAYFPR	37005.26	61	3.70E+04	61	TRUE	TRUE	
1	YNYKLPDDF	408	416	9	HLA-A*02:01	408	62	YNYKLPDDF	YNYKLPDDF	37039.31	62	3.70E+04	62	TRUE	TRUE	
1	TTGIGYQPY	486	494	9	HLA-A*02:01	486	62	TTGIGYQPY	TTGIGYQPY	37040.5	62	3.70E+04	62	TRUE	TRUE	
1	EHVDTSYEC	640	648	9	HLA-A*02:01	640	62	EHVDTSYEC	EHVDTSYEC	37066.57	62	3.71E+04	62	TRUE	TRUE	
1	CGKGYHLMS	1025	1033	9	HLA-A*02:01	1025	62	CGKGYHLMS	CGKGYHLMS	37087.83	62	3.71E+04	62	TRUE	TRUE	
1	NYTQHTSSM	29	37	9	HLA-A*02:01	29	62	NYTQHTSSM	NYTQHTSSM	37116.73	62	3.71E+04	62	TRUE	TRUE	
1	VSLLRSTSQ	663	671	9	HLA-A*02:01	663	62	VSLLRSTSQ	VSLLRSTSQ	37138.84	62	3.71E+04	62	TRUE	TRUE	
1	DINARDLIC	825	833	9	HLA-A*02:01	825	62	DINARDLIC	DINARDLIC	37165.36	62	3.72E+04	62	TRUE	TRUE	
1	PTNFSISIT	697	705	9	HLA-A*02:01	697	62	PTNFSISIT	PTNFSISIT	37173.8	62	3.72E+04	62	TRUE	TRUE	
1	NYKLPDDFM	409	417	9	HLA-A*02:01	409	62	NYKLPDDFM	NYKLPDDFM	37175.02	62	3.72E+04	62	TRUE	TRUE	
1	TKMSECVLG	1009	1017	9	HLA-A*02:01	1009	62	TKMSECVLG	TKMSECVLG	37232.58	62	3.72E+04	62	TRUE	TRUE	
1	VRGWVFGST	98	106	9	HLA-A*02:01	98	62	VRGWVFGST	VRGWVFGST	37249.51	62	3.72E+04	62	TRUE	TRUE	
1	PFERDISNV	450	458	9	HLA-A*02:01	450	62	PFERDISNV	PFERDISNV	37256.77	62	3.73E+04	62	TRUE	TRUE	
1	PSQERNFTT	1051	1059	9	HLA-A*02:01	1051	62	PSQERNFTT	PSQERNFTT	37257.15	62	3.73E+04	62	TRUE	TRUE	
1	QAGCLIGAE	632	640	9	HLA-A*02:01	632	62	QAGCLIGAE	QAGCLIGAE	37267.24	62	3.73E+04	62	TRUE	TRUE	
1	WPLNDYGFY	476	484	9	HLA-A*02:01	476	62	WPLNDYGFY	WPLNDYGFY	37281.36	62	3.73E+04	62	TRUE	TRUE	
1	ECANLLLQY	730	738	9	HLA-A*02:01	730	63	ECANLLLQY	ECANLLLQY	37297.9	63	3.73E+04	63	TRUE	TRUE	
1	FPNITNLCP	316	324	9	HLA-A*02:01	316	63	FPNITNLCP	FPNITNLCP	37312.02	63	3.73E+04	63	TRUE	TRUE	
1	YKLPDDFMG	410	418	9	HLA-A*02:01	410	63	YKLPDDFMG	YKLPDDFMG	37319.7	63	3.73E+04	63	TRUE	TRUE	
1	DLICAQKFN	830	838	9	HLA-A*02:01	830	63	DLICAQKFN	DLICAQKFN	37374.64	63	3.74E+04	63	TRUE	TRUE	
1	VTGFHTINH	66	74	9	HLA-A*02:01	66	63	VTGFHTINH	VTGFHTINH	37452.38	63	3.75E+04	63	TRUE	TRUE	
1	NFNFNGLTG	526	534	9	HLA-A*02:01	526	63	NFNFNGLTG	NFNFNGLTG	37495.35	63	3.75E+04	63	TRUE	TRUE	
1	QSLQTYVTQ	984	992	9	HLA-A*02:01	984	63	QSLQTYVTQ	QSLQTYVTQ	37496.97	63	3.75E+04	63	TRUE	TRUE	
1	DLQELGKYE	1181	1189	9	HLA-A*02:01	1181	63	DLQELGKYE	DLQELGKYE	37519.29	63	3.75E+04	63	TRUE	TRUE	
1	GACSCGSCC	1228	1236	9	HLA-A*02:01	1228	63	GACSCGSCC	GACSCGSCC	37529.85	63	3.75E+04	63	TRUE	TRUE	
1	KPMGTQTHT	142	150	9	HLA-A*02:01	142	63	KPMGTQTHT	KPMGTQTHT	37551.37	63	3.76E+04	63	TRUE	TRUE	
1	NTFVSGNCD	1101	1109	9	HLA-A*02:01	1101	64	NTFVSGNCD	NTFVSGNCD	37624.57	64	3.76E+04	64	TRUE	TRUE	
1	TQHTSSMRG	31	39	9	HLA-A*02:01	31	64	TQHTSSMRG	TQHTSSMRG	37647.39	64	3.76E+04	64	TRUE	TRUE	
1	LYVYKGYQP	194	202	9	HLA-A*02:01	194	64	LYVYKGYQP	LYVYKGYQP	37679.99	64	3.77E+04	64	TRUE	TRUE	
1	DGIYFAATE	85	93	9	HLA-A*02:01	85	64	DGIYFAATE	DGIYFAATE	37782.87	64	3.78E+04	64	TRUE	TRUE	
1	TPPALNCYW	468	476	9	HLA-A*02:01	468	64	TPPALNCYW	TPPALNCYW	37791.45	64	3.78E+04	64	TRUE	TRUE	
1	KPTTFMLKY	258	266	9	HLA-A*02:01	258	64	KPTTFMLKY	KPTTFMLKY	37811.49	64	3.78E+04	64	TRUE	TRUE	
1	KFDEDDSEP	1237	1245	9	HLA-A*02:01	1237	64	KFDEDDSEP	KFDEDDSEP	37824.17	64	3.78E+04	64	TRUE	TRUE	
1	GECLGDINA	820	828	9	HLA-A*02:01	820	65	GECLGDINA	GECLGDINA	37846.69	65	3.78E+04	65	TRUE	TRUE	
1	PDDFMGCVL	413	421	9	HLA-A*02:01	413	65	PDDFMGCVL	PDDFMGCVL	37866.34	65	3.79E+04	65	TRUE	TRUE	
1	NTVYDPLQP	1117	1125	9	HLA-A*02:01	1117	65	NTVYDPLQP	NTVYDPLQP	37893.41	65	3.79E+04	65	TRUE	TRUE	
1	WNTRNIDAT	423	431	9	HLA-A*02:01	423	65	WNTRNIDAT	WNTRNIDAT	37904.48	65	3.79E+04	65	TRUE	TRUE	
1	RDPKTSEIL	563	571	9	HLA-A*02:01	563	65	RDPKTSEIL	RDPKTSEIL	37982.47	65	3.80E+04	65	TRUE	TRUE	
1	GRLQSLQTY	981	989	9	HLA-A*02:01	981	65	GRLQSLQTY	GRLQSLQTY	37986.59	65	3.80E+04	65	TRUE	TRUE	
1	APGQTGVIA	398	406	9	HLA-A*02:01	398	65	APGQTGVIA	APGQTGVIA	38041.28	65	3.80E+04	65	TRUE	TRUE	
1	PAWRIYSTG	617	625	9	HLA-A*02:01	617	65	PAWRIYSTG	PAWRIYSTG	38042.53	65	3.80E+04	65	TRUE	TRUE	
1	QALNTLVKQ	939	947	9	HLA-A*02:01	939	65	QALNTLVKQ	QALNTLVKQ	38046.65	65	3.80E+04	65	TRUE	TRUE	
1	PPLLTDDMI	844	852	9	HLA-A*02:01	844	65	PPLLTDDMI	PPLLTDDMI	38065.57	65	3.81E+04	65	TRUE	TRUE	
1	SSEVAVLYQ	591	599	9	HLA-A*02:01	591	65	SSEVAVLYQ	SSEVAVLYQ	38084.53	65	3.81E+04	65	TRUE	TRUE	
1	SDAFSLDVS	165	173	9	HLA-A*02:01	165	65	SDAFSLDVS	SDAFSLDVS	38087.41	65	3.81E+04	65	TRUE	TRUE	
1	TKRSFIEDL	795	803	9	HLA-A*02:01	795	66	TKRSFIEDL	TKRSFIEDL	38146.8	66	3.81E+04	66	TRUE	TRUE	
1	QTHTMIFDN	147	155	9	HLA-A*02:01	147	66	QTHTMIFDN	QTHTMIFDN	38153.83	66	3.82E+04	66	TRUE	TRUE	
1	ATEKSNVVR	91	99	9	HLA-A*02:01	91	66	ATEKSNVVR	ATEKSNVVR	38194.3	66	3.82E+04	66	TRUE	TRUE	
1	DLIKNQCVN	518	526	9	HLA-A*02:01	518	66	DLIKNQCVN	DLIKNQCVN	38217.44	66	3.82E+04	66	TRUE	TRUE	
1	LKGVKLHYT	1247	1255	9	HLA-A*02:01	1247	66	LKGVKLHYT	LKGVKLHYT	38217.44	66	3.82E+04	66	TRUE	TRUE	
1	EPVLKGVKL	1244	1252	9	HLA-A*02:01	1244	66	EPVLKGVKL	EPVLKGVKL	38245.15	66	3.82E+04	66	TRUE	TRUE	
1	GAGAALQIP	871	879	9	HLA-A*02:01	871	66	GAGAALQIP	GAGAALQIP	38316.82	66	3.83E+04	66	TRUE	TRUE	
1	IAAEQDRNT	752	760	9	HLA-A*02:01	752	66	IAAEQDRNT	IAAEQDRNT	38329.68	66	3.83E+04	66	TRUE	TRUE	
1	KDGFLYVYK	190	198	9	HLA-A*02:01	190	66	KDGFLYVYK	KDGFLYVYK	38360.36	66	3.84E+04	66	TRUE	TRUE	
1	DVVRDLPSG	204	212	9	HLA-A*02:01	204	67	DVVRDLPSG	DVVRDLPSG	38384.86	67	3.84E+04	67	TRUE	TRUE	
1	ITGRLQSLQ	979	987	9	HLA-A*02:01	979	67	ITGRLQSLQ	ITGRLQSLQ	38392.34	67	3.84E+04	67	TRUE	TRUE	
1	NATKFPSVY	330	338	9	HLA-A*02:01	330	67	NATKFPSVY	NATKFPSVY	38405.21	67	3.84E+04	67	TRUE	TRUE	
1	AWNTRNIDA	422	430	9	HLA-A*02:01	422	67	AWNTRNIDA	AWNTRNIDA	38411.04	67	3.84E+04	67	TRUE	TRUE	
1	NFSISITTE	699	707	9	HLA-A*02:01	699	67	NFSISITTE	NFSISITTE	38420.18	67	3.84E+04	67	TRUE	TRUE	

seq #	peptide	start	end	peptide length	allele	peptide index	median binding	netmhcpan_ba	netmhcpan_ba	netmhcpan_ba	netmhcpan_ba	legacy score	legacy rank	scores equal	percentiles equal
1	LRHGKLRPF	443	451	9	HLA-A*02:01	443	67	LRHGKLRPF	LRHGKLRPF	38456.37	67	3.85E+04	67	TRUE	TRUE
1	GVSATKLND	368	376	9	HLA-A*02:01	368	67	GVSATKLND	GVSATKLND	38481.75	67	3.85E+04	67	TRUE	TRUE
1	VEAEVQIDR	969	977	9	HLA-A*02:01	969	67	VEAEVQIDR	VEAEVQIDR	38506.75	67	3.85E+04	67	TRUE	TRUE
1	NVPFSPDGK	457	465	9	HLA-A*02:01	457	67	NVPFSPDGK	NVPFSPDGK	38508.41	67	3.85E+04	67	TRUE	TRUE
1	DAFSLDVSE	166	174	9	HLA-A*02:01	166	67	DAFSLDVSE	DAFSLDVSE	38509.26	67	3.85E+04	67	TRUE	TRUE
1	EAEVQIDRL	970	978	9	HLA-A*02:01	970	67	EAEVQIDRL	EAEVQIDRL	38529.25	67	3.85E+04	67	TRUE	TRUE
1	GKGYHLMSF	1026	1034	9	HLA-A*02:01	1026	67	GKGYHLMSF	GKGYHLMSF	38529.25	67	3.85E+04	67	TRUE	TRUE
1	GAGICASYH	653	661	9	HLA-A*02:01	653	67	GAGICASYH	GAGICASYH	38536.76	67	3.85E+04	67	TRUE	TRUE
1	ADAGFMKQY	811	819	9	HLA-A*02:01	811	67	ADAGFMKQY	ADAGFMKQY	38556.77	67	3.86E+04	67	TRUE	TRUE
1	RDLICAQKF	829	837	9	HLA-A*02:01	829	67	RDLICAQKF	RDLICAQKF	38566.37	67	3.86E+04	67	TRUE	TRUE
1	PQIITTDNT	1094	1102	9	HLA-A*02:01	1094	67	PQIITTDNT	PQIITTDNT	38575.13	67	3.86E+04	67	TRUE	TRUE
1	YKYRYLRHG	438	446	9	HLA-A*02:01	438	67	YKYRYLRHG	YKYRYLRHG	38581.82	67	3.86E+04	67	TRUE	TRUE
1	PFAMQMAYR	879	887	9	HLA-A*02:01	879	67	PFAMQMAYR	PFAMQMAYR	38583.91	67	3.86E+04	67	TRUE	TRUE
1	LKPTKRSFI	792	800	9	HLA-A*02:01	792	67	LKPTKRSFI	LKPTKRSFI	38609.38	67	3.86E+04	67	TRUE	TRUE
1	FDDVQAPNY	22	30	9	HLA-A*02:01	22	68	FDDVQAPNY	FDDVQAPNY	38667.06	68	3.87E+04	68	TRUE	TRUE
1	SGINASVVN	1152	1160	9	HLA-A*02:01	1152	68	SGINASVVN	SGINASVVN	38680.05	68	3.87E+04	68	TRUE	TRUE
1	SPCSFGGVS	574	582	9	HLA-A*02:01	574	68	SPCSFGGVS	SPCSFGGVS	38688.84	68	3.87E+04	68	TRUE	TRUE
1	VSKPMGTQT	140	148	9	HLA-A*02:01	140	68	VSKPMGTQT	VSKPMGTQT	38689.66	68	3.87E+04	68	TRUE	TRUE
1	RNIDATSTG	426	434	9	HLA-A*02:01	426	68	RNIDATSTG	RNIDATSTG	38741.19	68	3.87E+04	68	TRUE	TRUE
1	GAEHVDTSY	638	646	9	HLA-A*02:01	638	68	GAEHVDTSY	GAEHVDTSY	38751.26	68	3.88E+04	68	TRUE	TRUE
1	SFKEELDKY	1129	1137	9	HLA-A*02:01	1129	68	SFKEELDKY	SFKEELDKY	38776.84	68	3.88E+04	68	TRUE	TRUE
1	MPVSMAKTS	709	717	9	HLA-A*02:01	709	68	MPVSMAKTS	MPVSMAKTS	38783.14	68	3.88E+04	68	TRUE	TRUE
1	VSEKSGNFK	172	180	9	HLA-A*02:01	172	68	VSEKSGNFK	VSEKSGNFK	38799.5	68	3.88E+04	68	TRUE	TRUE
1	EVQIDRLIT	972	980	9	HLA-A*02:01	972	69	EVQIDRLIT	EVQIDRLIT	38892.82	69	3.89E+04	69	TRUE	TRUE
1	TQNVLYENQ	894	902	9	HLA-A*02:01	894	69	TQNVLYENQ	TQNVLYENQ	38916.8	69	3.89E+04	69	TRUE	TRUE
1	DIPIGAGIC	649	657	9	HLA-A*02:01	649	69	DIPIGAGIC	DIPIGAGIC	38967.79	69	3.90E+04	69	TRUE	TRUE
1	FERDISNVP	451	459	9	HLA-A*02:01	451	69	FERDISNVP	FERDISNVP	39000.27	69	3.90E+04	69	TRUE	TRUE
1	LRSTSQKSI	666	674	9	HLA-A*02:01	666	69	LRSTSQKSI	LRSTSQKSI	39009.55	69	3.90E+04	69	TRUE	TRUE
1	QTGVIADYN	401	409	9	HLA-A*02:01	401	69	QTGVIADYN	QTGVIADYN	39030.66	69	3.90E+04	69	TRUE	TRUE
1	NVTGFHTIN	65	73	9	HLA-A*02:01	65	69	NVTGFHTIN	NVTGFHTIN	39036.15	69	3.90E+04	69	TRUE	TRUE
1	LQELGKYEQ	1182	1190	9	HLA-A*02:01	1182	69	LQELGKYEQ	LQELGKYEQ	39069.52	69	3.91E+04	69	TRUE	TRUE
1	LNRALSGIA	745	753	9	HLA-A*02:01	745	69	LNRALSGIA	LNRALSGIA	39072.07	69	3.91E+04	69	TRUE	TRUE
1	TGVIADYNY	402	410	9	HLA-A*02:01	402	69	TGVIADYNY	TGVIADYNY	39082.63	69	3.91E+04	69	TRUE	TRUE
1	LYQDVNCTD	597	605	9	HLA-A*02:01	597	69	LYQDVNCTD	LYQDVNCTD	39092.36	69	3.91E+04	69	TRUE	TRUE
1	LPLGINITN	222	230	9	HLA-A*02:01	222	69	LPLGINITN	LPLGINITN	39108.88	69	3.91E+04	69	TRUE	TRUE
1	TSGSDLDRC	11	19	9	HLA-A*02:01	11	69	TSGSDLDRC	TSGSDLDRC	39125.38	69	3.91E+04	69	TRUE	TRUE
1	IDVVRDLPS	203	211	9	HLA-A*02:01	203	70	IDVVRDLPS	IDVVRDLPS	39167.73	70	3.92E+04	70	TRUE	TRUE
1	NPFFAVSKP	135	143	9	HLA-A*02:01	135	70	NPFFAVSKP	NPFFAVSKP	39199.1	70	3.92E+04	70	TRUE	TRUE
1	VDCSQNPLA	276	284	9	HLA-A*02:01	276	70	VDCSQNPLA	VDCSQNPLA	39222.44	70	3.92E+04	70	TRUE	TRUE
1	TGNYNYKYR	433	441	9	HLA-A*02:01	433	70	TGNYNYKYR	TGNYNYKYR	39260.22	70	3.93E+04	70	TRUE	TRUE
1	VSTAIHADQ	606	614	9	HLA-A*02:01	606	70	VSTAIHADQ	VSTAIHADQ	39304.86	70	3.93E+04	70	TRUE	TRUE
1	SSKRFQPFQ	541	549	9	HLA-A*02:01	541	70	SSKRFQPFQ	SSKRFQPFQ	39307.39	70	3.93E+04	70	TRUE	TRUE
1	STALGKLQD	924	932	9	HLA-A*02:01	924	70	STALGKLQD	STALGKLQD	39352.08	70	3.94E+04	70	TRUE	TRUE
1	PGTNASSEV	586	594	9	HLA-A*02:01	586	71	PGTNASSEV	PGTNASSEV	39395.53	71	3.94E+04	71	TRUE	TRUE
1	VAVLYQDVN	594	602	9	HLA-A*02:01	594	71	VAVLYQDVN	VAVLYQDVN	39439.02	71	3.94E+04	71	TRUE	TRUE
1	RYLRHGKLR	441	449	9	HLA-A*02:01	441	71	RYLRHGKLR	RYLRHGKLR	39448.84	71	3.94E+04	71	TRUE	TRUE
1	CCSCLKGAC	1222	1230	9	HLA-A*02:01	1222	71	CCSCLKGAC	CCSCLKGAC	39450.98	71	3.95E+04	71	TRUE	TRUE
1	TFGAGAALQ	869	877	9	HLA-A*02:01	869	71	TFGAGAALQ	TFGAGAALQ	39462.94	71	3.95E+04	71	TRUE	TRUE
1	PIDVVRDLP	202	210	9	HLA-A*02:01	202	71	PIDVVRDLP	PIDVVRDLP	39511.22	71	3.95E+04	71	TRUE	TRUE
1	NDLCFSNVY	375	383	9	HLA-A*02:01	375	71	NDLCFSNVY	NDLCFSNVY	39519.35	71	3.95E+04	71	TRUE	TRUE
1	LHVTYVPSQ	1045	1053	9	HLA-A*02:01	1045	71	LHVTYVPSQ	LHVTYVPSQ	39556.99	71	3.96E+04	71	TRUE	TRUE
1	SGNCDVVIG	1105	1113	9	HLA-A*02:01	1105	71	SGNCDVVIG	SGNCDVVIG	39562.12	71	3.96E+04	71	TRUE	TRUE
1	AELKCSVKS	284	292	9	HLA-A*02:01	284	71	AELKCSVKS	AELKCSVKS	39578.4	71	3.96E+04	71	TRUE	TRUE
1	LNCYWPLND	472	480	9	HLA-A*02:01	472	72	LNCYWPLND	LNCYWPLND	39609.68	72	3.96E+04	72	TRUE	TRUE
1	ASVVNIQKE	1156	1164	9	HLA-A*02:01	1156	72	ASVVNIQKE	ASVVNIQKE	39668.86	72	3.97E+04	72	TRUE	TRUE
1	AGCLIGAEH	633	641	9	HLA-A*02:01	633	72	AGCLIGAEH	AGCLIGAEH	39682.6	72	3.97E+04	72	TRUE	TRUE
1	TGVLTPSSK	535	543	9	HLA-A*02:01	535	72	TGVLTPSSK	TGVLTPSSK	39687.31	72	3.97E+04	72	TRUE	TRUE
1	TECANLLLQ	729	737	9	HLA-A*02:01	729	72	TECANLLLQ	TECANLLLQ	39759.96	72	3.98E+04	72	TRUE	TRUE
1	YFKNHTSPD	1137	1145	9	HLA-A*02:01	1137	72	YFKNHTSPD	YFKNHTSPD	39776.73	72	3.98E+04	72	TRUE	TRUE
1	WERKKISNC	340	348	9	HLA-A*02:01	340	72	WERKKISNC	WERKKISNC	39818.08	72	3.98E+04	72	TRUE	TRUE
1	LNAPATVCG	504	512	9	HLA-A*02:01	504	73	LNAPATVCG	LNAPATVCG	39873.25	73	3.99E+04	73	TRUE	TRUE
1	DVSTAIHAD	605	613	9	HLA-A*02:01	605	73	DVSTAIHAD	DVSTAIHAD	39932.39	73	3.99E+04	73	TRUE	TRUE
1	FHTINHTFG	69	77	9	HLA-A*02:01	69	73	FHTINHTFG	FHTINHTFG	39936.28	73	3.99E+04	73	TRUE	TRUE
1	LGKYEQYIK	1185	1193	9	HLA-A*02:01	1185	73	LGKYEQYIK	LGKYEQYIK	39958.33	73	4.00E+04	73	TRUE	TRUE
1	DRCTTFDDV	17	25	9	HLA-A*02:01	17	73	DRCTTFDDV	DRCTTFDDV	39983.85	73	4.00E+04	73	TRUE	TRUE
1	TSEILDISP	567	575	9	HLA-A*02:01	567	73	TSEILDISP	TSEILDISP	39991.64	73	4.00E+04	73	TRUE	TRUE
1	TGRLQSLQT	980	988	9	HLA-A*02:01	980	73	TGRLQSLQT	TGRLQSLQT	40015.86	73	4.00E+04	73	TRUE	TRUE
1	SECVLGQSK	1012	1020	9	HLA-A*02:01	1012	73	SECVLGQSK	SECVLGQSK	40026.27	73	4.00E+04	73	TRUE	TRUE
1	APHGVVFLH	1038	1046	9	HLA-A*02:01	1038	73	APHGVVFLH	APHGVVFLH	40026.7	73	4.00E+04	73	TRUE	TRUE
1	TSPDVDLGD	1142	1150	9	HLA-A*02:01	1142	73	TSPDVDLGD	TSPDVDLGD	40040.57	73	4.00E+04	73	TRUE	TRUE
1	TFMLKYDEN	261	269	9	HLA-A*02:01	261	73	TFMLKYDEN	TFMLKYDEN	40047.05	73	4.00E+04	73	TRUE	TRUE
1	VYAWERKKI	337	345	9	HLA-A*02:01	337	74	VYAWERKKI	VYAWERKKI	40119.48	74	4.01E+04	74	TRUE	TRUE
1	SVRDPKTSE	561	569	9	HLA-A*02:01	561	74	SVRDPKTSE	SVRDPKTSE	40122.09	74	4.01E+04	74	TRUE	TRUE
1	VKGDDVRQI	389	397	9	HLA-A*02:01	389	74	VKGDDVRQI	VKGDDVRQI	40134.26	74	4.01E+04	74	TRUE	TRUE
1	DKVEAEVQI	967	975	9	HLA-A*02:01	967	74	DKVEAEVQI	DKVEAEVQI	40155.1	74	4.02E+04	74	TRUE	TRUE
1	HVDTSYECD	641	649	9	HLA-A*02:01	641	74	HVDTSYECD	HVDTSYECD	40156.83	74	4.02E+04	74	TRUE	TRUE
1	ACSCGSCCK	1229	1237	9	HLA-A*02:01	1229	74	ACSCGSCCK	ACSCGSCCK	40197.25	74	4.02E+04	74	TRUE	TRUE
1	DNPFFAVSK	134	142	9	HLA-A*02:01	134	74	DNPFFAVSK	DNPFFAVSK	40205.96	74	4.02E+04	74	TRUE	TRUE
1	PPALNCYWP	469	477	9	HLA-A*02:01	469	74	PPALNCYWP	PPALNCYWP	40259.49	74	4.03E+04	74	TRUE	TRUE
1	QESLTTTST	917	925	9	HLA-A*02:01	917	74	QESLTTTST	QESLTTTST	40269.52	74	4.03E+04	74	TRUE	TRUE
1	DLGDISGIN	1147	1155	9	HLA-A*02:01	1147	74	DLGDISGIN	DLGDISGIN	40309.63	74	4.03E+04	74	TRUE	TRUE
1	HGKLRPFER	445	453	9	HLA-A*02:01	445	75	HGKLRPFER	HGKLRPFER	40336.24	75	4.03E+04	75	TRUE	TRUE
1	VLGQSKRVD	1015	1023	9	HLA-A*02:01	1015	75	VLGQSKRVD	VLGQSKRVD	40354.58	75	4.04E+04	75	TRUE	TRUE
1	TGNNVFQTQ	624	632	9	HLA-A*02:01	624	75	TGNNVFQTQ	TGNNVFQTQ	40366.81	75	4.04E+04	75	TRUE	TRUE
1	PSSKRFQPF	540	548	9	HLA-A*02:01	540	75	PSSKRFQPF	PSSKRFQPF	40393.9	75	4.04E+04	75	TRUE	TRUE
1	MYKTPTLKY	770	778	9	HLA-A*02:01	770	75	MYKTPTLKY	MYKTPTLKY	40406.56	75	4.04E+04	75	TRUE	TRUE
1	TPTLKYFGG	773	781	9	HLA-A*02:01	773	75	TPTLKYFGG	TPTLKYFGG	40429.32	75	4.04E+04	75	TRUE	TRUE
1	NFNGLTGTG	528	536	9	HLA-A*02:01	528	75	NFNGLTGTG	NFNGLTGTG	40456	75	4.05E+04	75	TRUE	TRUE
1	LPDPLKPTK	788	796	9	HLA-A*02:01	788	75	LPDPLKPTK	LPDPLKPTK	40463.02	75	4.05E+04	75	TRUE	TRUE
1	GKYEQYIKW	1186	1194	9	HLA-A*02:01	1186	75	GKYEQYIKW	GKYEQYIKW	40505.5	75	4.05E+04	75	TRUE	TRUE
1	LCPFGEVFN	322	330	9	HLA-A*02:01	322	76	LCPFGEVFN	LCPFGEVFN	40579.2	76	4.06E+04	76	TRUE	TRUE
1	DYSVLYNST	351	359	9	HLA-A*02:01	351	76	DYSVLYNST	DYSVLYNST	40596.77	76	4.06E+04	76	TRUE	TRUE
1	TYVTQQLIR	988	996	9	HLA-A*02:01	988	76	TYVTQQLIR	TYVTQQLIR	40605.98	76	4.06E+04	76	TRUE	TRUE
1	LSTDLIKNQ	515	523	9	HLA-A*02:01	515	76	LSTDLIKNQ	LSTDLIKNQ	40627.96	76	4.06E+04	76	TRUE	TRUE
1	LREFVFKNK	182	190	9	HLA-A*02:01	182	76	LREFVFKNK	LREFVFKNK	40685.14	76	4.07E+04	76	TRUE	TRUE
1	ADYNYKLPD	406	414	9	HLA-A*02:01	406	76	ADYNYKLPD	ADYNYKLPD	40699.68	76	4.07E+04	76	TRUE	TRUE
1	NARDLICAQ	827	835	9	HLA-A*02:01	827	76	NARDLICAQ	NARDLICAQ	40707.16	76	4.07E+04	76	TRUE	TRUE
1	CHEGKAYFP	1064	1072	9	HLA-A*02:01	1064	76	CHEGKAYFP	CHEGKAYFP	40712.44	76	4.07E+04	76	TRUE	TRUE
1	DVDLGDISG	1145	1153	9	HLA-A*02:01	1145	76	DVDLGDISG	DVDLGDISG	40720.82	76	4.07E+04	76	TRUE	TRUE
1	PAQDIWGTS	240	248	9	HLA-A*02:01	240	76	PAQDIWGTS	PAQDIWGTS	40752.55	76	4.08E+04	76	TRUE	TRUE
1	QFGRDVSDF	550	558	9	HLA-A*02:01	550	76	QFGRDVSDF	QFGRDVSDF	40755.63	76	4.08E+04	76	TRUE	TRUE
1	YGVSATKLN	367	375	9	HLA-A*02:01	367	76	YGVSATKLN	YGVSATKLN	40784.76	76	4.08E+04	76	TRUE	TRUE
1	KVEAEVQID	968	976	9	HLA-A*02:01	968	77	KVEAEVQID	KVEAEVQID	40853.65	77	4.09E+04	77	TRUE	TRUE
1	PFQQFGRDV	547	555	9	HLA-A*02:01	547	77	PFQQFGRDV	PFQQFGRDV	40890.79	77	4.09E+04	77	TRUE	TRUE
1	INASVVNIQ	1154	1162	9	HLA-A*02:01	1154	77	INASVVNIQ	INASVVNIQ	40911.16	77	4.09E+04	77	TRUE	TRUE
1	GSCCKFDED	1233	1241	9	HLA-A*02:01	1233	77	GSCCKFDED	GSCCKFDED	40951.89	77	4.10E+04	77	TRUE	TRUE
1	REVFAQVKQ	761	769	9	HLA-A*02:01	761	77	REVFAQVKQ	REVFAQVKQ	40955.45	77	4.10E+04	77	TRUE	TRUE
1	DVSEKSGNF	171	179	9	HLA-A*02:01	171	77	DVSEKSGNF	DVSEKSGNF	41020.19	77	4.10E+04	77	TRUE	TRUE
1	VPSQERNFT	1050	1058	9	HLA-A*02:01	1050	77	VPSQERNFT	VPSQERNFT	41041.49	77	4.10E+04	77	TRUE	TRUE
1	PYRVVVLSF	493	501	9	HLA-A*02:01	493	77	PYRVVVLSF	PYRVVVLSF	41048.61	77	4.10E+04	77	TRUE	TRUE
1	IRAAEIRAS	995	1003	9	HLA-A*02:01	995	78	IRAAEIRAS	IRAAEIRAS	41050.83	78	4.11E+04	78	TRUE	TRUE
1	NNKSQSVII	108	116	9	HLA-A*02:01	108	78	NNKSQSVII	NNKSQSVII	41103.71	78	4.11E+04	78	TRUE	TRUE
1	RPFERDISN	449	457	9	HLA-A*02:01	449	78	RPFERDISN	RPFERDISN	41127.73	78	4.11E+04	78	TRUE	TRUE
1	LNTLVKQLS	941	949	9	HLA-A*02:01	941	78	LNTLVKQLS	LNTLVKQLS	41139.3	78	4.11E+04	78	TRUE	TRUE
1	RDVSDFTDS	553	561	9	HLA-A*02:01	553	78	RDVSDFTDS	RDVSDFTDS	41140.65	78	4.11E+04	78	TRUE	TRUE

seq #	peptide	start	end	peptide length	allele	peptide index	median binding	netmhcpan_ba	netmhcpan_ba	netmhcpan_ba	netmhcpan_ba	legacy score	legacy rank	scores equal	percentiles equal
1	DVQAPNYTQ	24	32	9	HLA-A*02:01	24	78	DVQAPNYTQ	DVQAPNYTQ	41220.4	78	4.12E+04	78	TRUE	TRUE
1	DTSYECDIP	643	651	9	HLA-A*02:01	643	78	DTSYECDIP	DTSYECDIP	41239.57	78	4.12E+04	78	TRUE	TRUE
1	SRLDKVEAE	964	972	9	HLA-A*02:01	964	78	SRLDKVEAE	SRLDKVEAE	41240.93	78	4.12E+04	78	TRUE	TRUE
1	DVVIGIINN	1109	1117	9	HLA-A*02:01	1109	78	DVVIGIINN	DVVIGIINN	41249.39	78	4.12E+04	78	TRUE	TRUE
1	PLKPTKRSF	791	799	9	HLA-A*02:01	791	78	PLKPTKRSF	PLKPTKRSF	41258.78	78	4.13E+04	78	TRUE	TRUE
1	TREVFAQVK	760	768	9	HLA-A*02:01	760	78	TREVFAQVK	TREVFAQVK	41282.44	78	4.13E+04	78	TRUE	TRUE
1	PAICHEGKA	1061	1069	9	HLA-A*02:01	1061	79	PAICHEGKA	PAICHEGKA	41345.03	79	4.13E+04	79	TRUE	TRUE
1	PSVYAWERK	335	343	9	HLA-A*02:01	335	79	PSVYAWERK	PSVYAWERK	41347.7	79	4.13E+04	79	TRUE	TRUE
1	KYRYLRHGK	439	447	9	HLA-A*02:01	439	79	KYRYLRHGK	KYRYLRHGK	41364.26	79	4.14E+04	79	TRUE	TRUE
1	QVKQMYKTP	766	774	9	HLA-A*02:01	766	79	QVKQMYKTP	QVKQMYKTP	41364.71	79	4.14E+04	79	TRUE	TRUE
1	SYECDIPIG	645	653	9	HLA-A*02:01	645	79	SYECDIPIG	SYECDIPIG	41400.07	79	4.14E+04	79	TRUE	TRUE
1	APNYTQHTS	27	35	9	HLA-A*02:01	27	79	APNYTQHTS	APNYTQHTS	41431.46	79	4.14E+04	79	TRUE	TRUE
1	IQKEIDRLN	1161	1169	9	HLA-A*02:01	1161	79	IQKEIDRLN	IQKEIDRLN	41432.34	79	4.14E+04	79	TRUE	TRUE
1	LSGIAAEQD	749	757	9	HLA-A*02:01	749	79	LSGIAAEQD	LSGIAAEQD	41467.79	79	4.15E+04	79	TRUE	TRUE
1	SKRVDFCGK	1019	1027	9	HLA-A*02:01	1019	79	SKRVDFCGK	SKRVDFCGK	41501.44	79	4.15E+04	79	TRUE	TRUE
1	MKQYGECLG	816	824	9	HLA-A*02:01	816	79	MKQYGECLG	MKQYGECLG	41505.48	79	4.15E+04	79	TRUE	TRUE
1	ECLGDINAR	821	829	9	HLA-A*02:01	821	79	ECLGDINAR	ECLGDINAR	41520.31	79	4.15E+04	79	TRUE	TRUE
1	SGSDLDRCT	12	20	9	HLA-A*02:01	12	79	SGSDLDRCT	SGSDLDRCT	41523.45	79	4.15E+04	79	TRUE	TRUE
1	YFAATEKSN	88	96	9	HLA-A*02:01	88	80	YFAATEKSN	YFAATEKSN	41576.05	80	4.16E+04	80	TRUE	TRUE
1	VSVITPGTN	581	589	9	HLA-A*02:01	581	80	VSVITPGTN	VSVITPGTN	41585.03	80	4.16E+04	80	TRUE	TRUE
1	NVLYENQKQ	896	904	9	HLA-A*02:01	896	80	NVLYENQKQ	NVLYENQKQ	41664.76	80	4.17E+04	80	TRUE	TRUE
1	VDFCGKGYH	1022	1030	9	HLA-A*02:01	1022	80	VDFCGKGYH	VDFCGKGYH	41686.85	80	4.17E+04	80	TRUE	TRUE
1	QSKRVDFCG	1018	1026	9	HLA-A*02:01	1018	80	QSKRVDFCG	QSKRVDFCG	41722.06	80	4.17E+04	80	TRUE	TRUE
1	RNTREVFAQ	758	766	9	HLA-A*02:01	758	81	RNTREVFAQ	RNTREVFAQ	41754.11	81	4.18E+04	81	TRUE	TRUE
1	MAKTSVDCN	713	721	9	HLA-A*02:01	713	81	MAKTSVDCN	MAKTSVDCN	41764.97	81	4.18E+04	81	TRUE	TRUE
1	KKISNCVAD	343	351	9	HLA-A*02:01	343	81	KKISNCVAD	KKISNCVAD	41786.2	81	4.18E+04	81	TRUE	TRUE
1	ANLAATKMS	1004	1012	9	HLA-A*02:01	1004	81	ANLAATKMS	ANLAATKMS	41791.62	81	4.18E+04	81	TRUE	TRUE
1	IDATSTGNY	428	436	9	HLA-A*02:01	428	81	IDATSTGNY	IDATSTGNY	41810.62	81	4.18E+04	81	TRUE	TRUE
1	SGNFKHLRE	176	184	9	HLA-A*02:01	176	81	SGNFKHLRE	SGNFKHLRE	41859.05	81	4.19E+04	81	TRUE	TRUE
1	LTPSSKRFQ	538	546	9	HLA-A*02:01	538	81	LTPSSKRFQ	LTPSSKRFQ	41873.55	81	4.19E+04	81	TRUE	TRUE
1	KRVDFCGKG	1020	1028	9	HLA-A*02:01	1020	81	KRVDFCGKG	KRVDFCGKG	41940.65	81	4.19E+04	81	TRUE	TRUE
1	AYTMSLGAD	676	684	9	HLA-A*02:01	676	82	AYTMSLGAD	AYTMSLGAD	42014.7	82	4.20E+04	82	TRUE	TRUE
1	IDRLNEVAK	1165	1173	9	HLA-A*02:01	1165	82	IDRLNEVAK	IDRLNEVAK	42070.2	82	4.21E+04	82	TRUE	TRUE
1	VKQMYKTPT	767	775	9	HLA-A*02:01	767	82	VKQMYKTPT	VKQMYKTPT	42080.21	82	4.21E+04	82	TRUE	TRUE
1	IDLQELGKY	1180	1188	9	HLA-A*02:01	1180	82	IDLQELGKY	IDLQELGKY	42131.7	82	4.21E+04	82	TRUE	TRUE
1	PVLKGVKLH	1245	1253	9	HLA-A*02:01	1245	82	PVLKGVKLH	PVLKGVKLH	42167.25	82	4.22E+04	82	TRUE	TRUE
1	LGQSKRVDF	1016	1024	9	HLA-A*02:01	1016	82	LGQSKRVDF	LGQSKRVDF	42173.18	82	4.22E+04	82	TRUE	TRUE
1	VVKGDDVRQ	388	396	9	HLA-A*02:01	388	82	VVKGDDVRQ	VVKGDDVRQ	42178.21	82	4.22E+04	82	TRUE	TRUE
1	CGDSTECAN	725	733	9	HLA-A*02:01	725	83	CGDSTECAN	CGDSTECAN	42227.06	83	4.22E+04	83	TRUE	TRUE
1	APAICHEGK	1060	1068	9	HLA-A*02:01	1060	83	APAICHEGK	APAICHEGK	42249.92	83	4.22E+04	83	TRUE	TRUE
1	NNSTNVVIR	118	126	9	HLA-A*02:01	118	83	NNSTNVVIR	NNSTNVVIR	42272.32	83	4.23E+04	83	TRUE	TRUE
1	QERNFTTAP	1053	1061	9	HLA-A*02:01	1053	83	QERNFTTAP	QERNFTTAP	42280.55	83	4.23E+04	83	TRUE	TRUE
1	SGIAAEQDR	750	758	9	HLA-A*02:01	750	83	SGIAAEQDR	SGIAAEQDR	42286.96	83	4.23E+04	83	TRUE	TRUE
1	ADSSIAYSN	683	691	9	HLA-A*02:01	683	83	ADSSIAYSN	ADSSIAYSN	42317.63	83	4.23E+04	83	TRUE	TRUE
1	DNAFNCTFE	154	162	9	HLA-A*02:01	154	83	DNAFNCTFE	DNAFNCTFE	42318.09	83	4.23E+04	83	TRUE	TRUE
1	GWVFGSTMN	100	108	9	HLA-A*02:01	100	83	GWVFGSTMN	GWVFGSTMN	42321.75	83	4.23E+04	83	TRUE	TRUE
1	VDCNMYICG	718	726	9	HLA-A*02:01	718	83	VDCNMYICG	VDCNMYICG	42336.41	83	4.23E+04	83	TRUE	TRUE
1	DSFKEELDK	1128	1136	9	HLA-A*02:01	1128	83	DSFKEELDK	DSFKEELDK	42343.26	83	4.23E+04	83	TRUE	TRUE
1	KYEQYIKWP	1187	1195	9	HLA-A*02:01	1187	83	KYEQYIKWP	KYEQYIKWP	42373.97	83	4.24E+04	83	TRUE	TRUE
1	IFKLPLGIN	219	227	9	HLA-A*02:01	219	83	IFKLPLGIN	IFKLPLGIN	42395.54	83	4.24E+04	83	TRUE	TRUE
1	QNVLYENQK	895	903	9	HLA-A*02:01	895	83	QNVLYENQK	QNVLYENQK	42428.57	83	4.24E+04	83	TRUE	TRUE
1	YGECLGDIN	819	827	9	HLA-A*02:01	819	83	YGECLGDIN	YGECLGDIN	42433.17	83	4.24E+04	83	TRUE	TRUE
1	RKKISNCVA	342	350	9	HLA-A*02:01	342	83	RKKISNCVA	RKKISNCVA	42437.77	83	4.24E+04	83	TRUE	TRUE
1	SNVPFSPDG	456	464	9	HLA-A*02:01	456	84	SNVPFSPDG	SNVPFSPDG	42453.84	84	4.25E+04	84	TRUE	TRUE
1	PCTPPALNC	466	474	9	HLA-A*02:01	466	84	PCTPPALNC	PCTPPALNC	42461.18	84	4.25E+04	84	TRUE	TRUE
1	CGSCCKFDE	1232	1240	9	HLA-A*02:01	1232	84	CGSCCKFDE	CGSCCKFDE	42500.24	84	4.25E+04	84	TRUE	TRUE
1	ERKKISNCV	341	349	9	HLA-A*02:01	341	84	ERKKISNCV	ERKKISNCV	42527.85	84	4.25E+04	84	TRUE	TRUE
1	QNPLAELKC	280	288	9	HLA-A*02:01	280	84	QNPLAELKC	QNPLAELKC	42536.6	84	4.25E+04	84	TRUE	TRUE
1	EKSGNFKHL	174	182	9	HLA-A*02:01	174	84	EKSGNFKHL	EKSGNFKHL	42552.71	84	4.26E+04	84	TRUE	TRUE
1	REFVFKNKD	183	191	9	HLA-A*02:01	183	84	REFVFKNKD	REFVFKNKD	42561	84	4.26E+04	84	TRUE	TRUE
1	TSTALGKLQ	923	931	9	HLA-A*02:01	923	84	TSTALGKLQ	TSTALGKLQ	42651.81	84	4.27E+04	84	TRUE	TRUE
1	LKGACSCGS	1226	1234	9	HLA-A*02:01	1226	84	LKGACSCGS	LKGACSCGS	42661.02	84	4.27E+04	84	TRUE	TRUE
1	LNDILSRLD	959	967	9	HLA-A*02:01	959	84	LNDILSRLD	LNDILSRLD	42669.34	84	4.27E+04	84	TRUE	TRUE
1	SFEIDKGIY	292	300	9	HLA-A*02:01	292	85	SFEIDKGIY	SFEIDKGIY	42727.55	85	4.27E+04	85	TRUE	TRUE
1	PTTFMLKYD	259	267	9	HLA-A*02:01	259	85	PTTFMLKYD	PTTFMLKYD	42753.46	85	4.28E+04	85	TRUE	TRUE
1	KNLNESLID	1173	1181	9	HLA-A*02:01	1173	85	KNLNESLID	KNLNESLID	42767.8	85	4.28E+04	85	TRUE	TRUE
1	PSGDVVRFP	309	317	9	HLA-A*02:01	309	85	PSGDVVRFP	PSGDVVRFP	42801.6	85	4.28E+04	85	TRUE	TRUE
1	AAEQDRNTR	753	761	9	HLA-A*02:01	753	85	AAEQDRNTR	AAEQDRNTR	42871.57	85	4.29E+04	85	TRUE	TRUE
1	MRGVYYPDE	37	45	9	HLA-A*02:01	37	85	MRGVYYPDE	MRGVYYPDE	42877.13	85	4.29E+04	85	TRUE	TRUE
1	LYENQKQIA	898	906	9	HLA-A*02:01	898	86	LYENQKQIA	LYENQKQIA	42935.18	86	4.29E+04	86	TRUE	TRUE
1	DYGFYTTTG	480	488	9	HLA-A*02:01	480	86	DYGFYTTTG	DYGFYTTTG	42942.13	86	4.29E+04	86	TRUE	TRUE
1	VPFSPDGKP	458	466	9	HLA-A*02:01	458	86	VPFSPDGKP	VPFSPDGKP	42948.64	86	4.29E+04	86	TRUE	TRUE
1	NCTFEYISD	158	166	9	HLA-A*02:01	158	86	NCTFEYISD	NCTFEYISD	42956.54	86	4.30E+04	86	TRUE	TRUE
1	RHGKLRPFE	444	452	9	HLA-A*02:01	444	86	RHGKLRPFE	RHGKLRPFE	42971.42	86	4.30E+04	86	TRUE	TRUE
1	DAVDCSQNP	274	282	9	HLA-A*02:01	274	86	DAVDCSQNP	DAVDCSQNP	42981.2	86	4.30E+04	86	TRUE	TRUE
1	SPDGKPCTP	461	469	9	HLA-A*02:01	461	86	SPDGKPCTP	SPDGKPCTP	42986.3	86	4.30E+04	86	TRUE	TRUE
1	LKCSVKSFE	286	294	9	HLA-A*02:01	286	86	LKCSVKSFE	LKCSVKSFE	43050.53	86	4.31E+04	86	TRUE	TRUE
1	GIAAEQDRN	751	759	9	HLA-A*02:01	751	86	GIAAEQDRN	GIAAEQDRN	43113.93	86	4.31E+04	86	TRUE	TRUE
1	KPTKRSFIE	793	801	9	HLA-A*02:01	793	86	KPTKRSFIE	KPTKRSFIE	43140.52	86	4.31E+04	86	TRUE	TRUE
1	FNKAISQIQ	909	917	9	HLA-A*02:01	909	87	FNKAISQIQ	FNKAISQIQ	43183.5	87	4.32E+04	87	TRUE	TRUE
1	SDTLYLTQD	49	57	9	HLA-A*02:01	49	87	SDTLYLTQD	SDTLYLTQD	43190.49	87	4.32E+04	87	TRUE	TRUE
1	ADQLTPAWR	612	620	9	HLA-A*02:01	612	87	ADQLTPAWR	ADQLTPAWR	43190.96	87	4.32E+04	87	TRUE	TRUE
1	EDLLFNKVT	801	809	9	HLA-A*02:01	801	87	EDLLFNKVT	EDLLFNKVT	43212	87	4.32E+04	87	TRUE	TRUE
1	AEHVDTSYE	639	647	9	HLA-A*02:01	639	87	AEHVDTSYE	AEHVDTSYE	43252.23	87	4.33E+04	87	TRUE	TRUE
1	NESLIDLQE	1176	1184	9	HLA-A*02:01	1176	87	NESLIDLQE	NESLIDLQE	43274.24	87	4.33E+04	87	TRUE	TRUE
1	IDKGIYQTS	295	303	9	HLA-A*02:01	295	87	IDKGIYQTS	IDKGIYQTS	43282.67	87	4.33E+04	87	TRUE	TRUE
1	NDILSRLDK	960	968	9	HLA-A*02:01	960	87	NDILSRLDK	NDILSRLDK	43286.89	87	4.33E+04	87	TRUE	TRUE
1	KEELDKYFK	1131	1139	9	HLA-A*02:01	1131	87	KEELDKYFK	KEELDKYFK	43299.54	87	4.33E+04	87	TRUE	TRUE
1	TDNTFVSGN	1099	1107	9	HLA-A*02:01	1099	87	TDNTFVSGN	TDNTFVSGN	43340.77	87	4.33E+04	87	TRUE	TRUE
1	FNGIGVTQN	888	896	9	HLA-A*02:01	888	87	FNGIGVTQN	FNGIGVTQN	43365.62	87	4.34E+04	87	TRUE	TRUE
1	AWRIYSTGN	618	626	9	HLA-A*02:01	618	88	AWRIYSTGN	AWRIYSTGN	43427.62	88	4.34E+04	88	TRUE	TRUE
1	DRNTREVFA	757	765	9	HLA-A*02:01	757	88	DRNTREVFA	DRNTREVFA	43441.71	88	4.34E+04	88	TRUE	TRUE
1	RCTTFDDVQ	18	26	9	HLA-A*02:01	18	88	RCTTFDDVQ	RCTTFDDVQ	43514.16	88	4.35E+04	88	TRUE	TRUE
1	NGTITDAVD	269	277	9	HLA-A*02:01	269	88	NGTITDAVD	NGTITDAVD	43521.68	88	4.35E+04	88	TRUE	TRUE
1	SEPVLKGVK	1243	1251	9	HLA-A*02:01	1243	88	SEPVLKGVK	SEPVLKGVK	43530.64	88	4.35E+04	88	TRUE	TRUE
1	YENQKQIAN	899	907	9	HLA-A*02:01	899	88	YENQKQIAN	YENQKQIAN	43531.59	88	4.35E+04	88	TRUE	TRUE
1	KPCTPPALN	465	473	9	HLA-A*02:01	465	88	KPCTPPALN	KPCTPPALN	43556.55	88	4.36E+04	88	TRUE	TRUE
1	NPLAELKCS	281	289	9	HLA-A*02:01	281	88	NPLAELKCS	NPLAELKCS	43573.52	88	4.36E+04	88	TRUE	TRUE
1	CDVVIGIIN	1108	1116	9	HLA-A*02:01	1108	88	CDVVIGIIN	CDVVIGIIN	43591.43	88	4.36E+04	88	TRUE	TRUE
1	ECDIPIGAG	647	655	9	HLA-A*02:01	647	88	ECDIPIGAG	ECDIPIGAG	43593.79	88	4.36E+04	88	TRUE	TRUE
1	TPSSKRFQP	539	547	9	HLA-A*02:01	539	89	TPSSKRFQP	TPSSKRFQP	43633.9	89	4.36E+04	89	TRUE	TRUE
1	VVNIQKEID	1158	1166	9	HLA-A*02:01	1158	89	VVNIQKEID	VVNIQKEID	43678.3	89	4.37E+04	89	TRUE	TRUE
1	ANQFNKAIS	906	914	9	HLA-A*02:01	906	89	ANQFNKAIS	ANQFNKAIS	43735.07	89	4.37E+04	89	TRUE	TRUE
1	DNTFVSGNC	1100	1108	9	HLA-A*02:01	1100	89	DNTFVSGNC	DNTFVSGNC	43738.84	89	4.37E+04	89	TRUE	TRUE
1	VNQNAQALN	934	942	9	HLA-A*02:01	934	89	VNQNAQALN	VNQNAQALN	43777.2	89	4.38E+04	89	TRUE	TRUE
1	TNVVIRACN	121	129	9	HLA-A*02:01	121	89	TNVVIRACN	TNVVIRACN	43808.94	89	4.38E+04	89	TRUE	TRUE
1	ECVLGQSKR	1013	1021	9	HLA-A*02:01	1013	89	ECVLGQSKR	ECVLGQSKR	43820.78	89	4.38E+04	89	TRUE	TRUE
1	QPFQQFGRD	546	554	9	HLA-A*02:01	546	89	QPFQQFGRD	QPFQQFGRD	43862.06	89	4.39E+04	89	TRUE	TRUE
1	DKYFKNHTS	1135	1143	9	HLA-A*02:01	1135	89	DKYFKNHTS	DKYFKNHTS	43864.44	89	4.39E+04	89	TRUE	TRUE
1	DSSIAYSNN	684	692	9	HLA-A*02:01	684	90	DSSIAYSNN	DSSIAYSNN	43875.36	90	4.39E+04	90	TRUE	TRUE
1	VNIQKEIDR	1159	1167	9	HLA-A*02:01	1159	90	VNIQKEIDR	VNIQKEIDR	43878.67	90	4.39E+04	90	TRUE	TRUE
1	AAEIRASAN	997	1005	9	HLA-A*02:01	997	90	AAEIRASAN	AAEIRASAN	43900.52	90	4.39E+04	90	TRUE	TRUE
1	GRDVSDFTD	552	560	9	HLA-A*02:01	552	90	GRDVSDFTD	GRDVSDFTD	43952.79	90	4.40E+04	90	TRUE	TRUE
1	QELGKYEQY	1183	1191	9	HLA-A*02:01	1183	90	QELGKYEQY	QELGKYEQY	43990.39	90	4.40E+04	90	TRUE	TRUE
1	EGKAYFPRE	1066	1074	9	HLA-A*02:01	1066	90	EGKAYFPRE	EGKAYFPRE	44021.32	90	4.40E+04	90	TRUE	TRUE

seq #	peptide	start	end	peptide length	allele	peptide index	median binding	netmhcpan_ba	netmhcpan_ba	netmhcpan_ba	netmhcpan_ba	legacy score	legacy rank	scores equal	percentiles equal	
1	PSGFNTLKP	210	218	9	HLA-A*02:01	210	90	PSGFNTLKP	PSGFNTLKP	44056.11	90	4.41E+04	90	TRUE	TRUE	
1	TRNIDATST	425	433	9	HLA-A*02:01	425	90	TRNIDATST	TRNIDATST	44083.29	90	4.41E+04	90	TRUE	TRUE	
1	SDFTDSVRD	556	564	9	HLA-A*02:01	556	90	SDFTDSVRD	SDFTDSVRD	44087.09	90	4.41E+04	90	TRUE	TRUE	
1	CKFDEDDSE	1236	1244	9	HLA-A*02:01	1236	91	CKFDEDDSE	CKFDEDDSE	44172.1	91	4.42E+04	91	TRUE	TRUE	
1	DVRQIAPGQ	393	401	9	HLA-A*02:01	393	91	DVRQIAPGQ	DVRQIAPGQ	44208.44	91	4.42E+04	91	TRUE	TRUE	
1	QDVNCTDVS	599	607	9	HLA-A*02:01	599	91	QDVNCTDVS	QDVNCTDVS	44255.82	91	4.43E+04	91	TRUE	TRUE	
1	GSTMNNKSQ	104	112	9	HLA-A*02:01	104	91	GSTMNNKSQ	GSTMNNKSQ	44293.65	91	4.43E+04	91	TRUE	TRUE	
1	DAGFMKQYG	812	820	9	HLA-A*02:01	812	91	DAGFMKQYG	DAGFMKQYG	44336.81	91	4.43E+04	91	TRUE	TRUE	
1	DDVRQIAPG	392	400	9	HLA-A*02:01	392	91	DDVRQIAPG	DDVRQIAPG	44359.83	91	4.44E+04	91	TRUE	TRUE	
1	WRIYSTGNN	619	627	9	HLA-A*02:01	619	91	WRIYSTGNN	WRIYSTGNN	44367.04	91	4.44E+04	91	TRUE	TRUE	
1	DENGTITDA	267	275	9	HLA-A*02:01	267	92	DENGTITDA	DENGTITDA	44389.13	92	4.44E+04	92	TRUE	TRUE	
1	VRDLPSGFN	206	214	9	HLA-A*02:01	206	92	VRDLPSGFN	VRDLPSGFN	44404.51	92	4.44E+04	92	TRUE	TRUE	
1	YDENGTITD	266	274	9	HLA-A*02:01	266	92	YDENGTITD	YDENGTITD	44405.94	92	4.44E+04	92	TRUE	TRUE	
1	VKQLSSNFG	945	953	9	HLA-A*02:01	945	92	VKQLSSNFG	VKQLSSNFG	44411.24	92	4.44E+04	92	TRUE	TRUE	
1	PTKRSFIED	794	802	9	HLA-A*02:01	794	92	PTKRSFIED	PTKRSFIED	44417	92	4.44E+04	92	TRUE	TRUE	
1	NNVFQTQAG	626	634	9	HLA-A*02:01	626	92	NNVFQTQAG	NNVFQTQAG	44433.33	92	4.44E+04	92	TRUE	TRUE	
1	NNTIAIPTN	691	699	9	HLA-A*02:01	691	92	NNTIAIPTN	NNTIAIPTN	44489.16	92	4.45E+04	92	TRUE	TRUE	
1	PFSPDGKPC	459	467	9	HLA-A*02:01	459	92	PFSPDGKPC	PFSPDGKPC	44515.13	92	4.45E+04	92	TRUE	TRUE	
1	LNESLIDLQ	1175	1183	9	HLA-A*02:01	1175	92	LNESLIDLQ	LNESLIDLQ	44527.68	92	4.45E+04	92	TRUE	TRUE	
1	ACNFELCDN	127	135	9	HLA-A*02:01	127	92	ACNFELCDN	ACNFELCDN	44551.77	92	4.46E+04	92	TRUE	TRUE	
1	VCGPKLSTD	510	518	9	HLA-A*02:01	510	92	VCGPKLSTD	VCGPKLSTD	44560.45	92	4.46E+04	92	TRUE	TRUE	
1	NEVAKNLNE	1169	1177	9	HLA-A*02:01	1169	92	NEVAKNLNE	NEVAKNLNE	44640.07	92	4.46E+04	92	TRUE	TRUE	
1	NFRVVPSGD	304	312	9	HLA-A*02:01	304	92	NFRVVPSGD	NFRVVPSGD	44642	92	4.46E+04	92	TRUE	TRUE	
1	ADSFVVKGD	384	392	9	HLA-A*02:01	384	93	ADSFVVKGD	ADSFVVKGD	44649.25	93	4.46E+04	93	TRUE	TRUE	
1	LDRCTTFDD	16	24	9	HLA-A*02:01	16	93	LDRCTTFDD	LDRCTTFDD	44649.25	93	4.46E+04	93	TRUE	TRUE	
1	DCNMYICGD	719	727	9	HLA-A*02:01	719	93	DCNMYICGD	DCNMYICGD	44780.35	93	4.48E+04	93	TRUE	TRUE	
1	LGKLQDVVN	927	935	9	HLA-A*02:01	927	93	LGKLQDVVN	LGKLQDVVN	44804.58	93	4.48E+04	93	TRUE	TRUE	
1	PKTSEILDI	565	573	9	HLA-A*02:01	565	93	PKTSEILDI	PKTSEILDI	44841.93	93	4.48E+04	93	TRUE	TRUE	
1	DDVQAPNYT	23	31	9	HLA-A*02:01	23	93	DDVQAPNYT	DDVQAPNYT	44894.86	93	4.49E+04	93	TRUE	TRUE	
1	VRQIAPGQT	394	402	9	HLA-A*02:01	394	93	VRQIAPGQT	VRQIAPGQT	44910.87	93	4.49E+04	93	TRUE	TRUE	
1	LDSFKEELD	1127	1135	9	HLA-A*02:01	1127	94	LDSFKEELD	LDSFKEELD	44950.25	94	4.50E+04	94	TRUE	TRUE	
1	SCGSCCKFD	1231	1239	9	HLA-A*02:01	1231	94	SCGSCCKFD	SCGSCCKFD	44950.25	94	4.50E+04	94	TRUE	TRUE	
1	NNTVYDPLQ	1116	1124	9	HLA-A*02:01	1116	94	NNTVYDPLQ	NNTVYDPLQ	44960.95	94	4.50E+04	94	TRUE	TRUE	
1	IKNQCVNFN	520	528	9	HLA-A*02:01	520	94	IKNQCVNFN	IKNQCVNFN	44970.2	94	4.50E+04	94	TRUE	TRUE	
1	SEKSGNFKH	173	181	9	HLA-A*02:01	173	94	SEKSGNFKH	SEKSGNFKH	44990.65	94	4.50E+04	94	TRUE	TRUE	
1	QDRNTREVF	756	764	9	HLA-A*02:01	756	94	QDRNTREVF	QDRNTREVF	45014.5	94	4.50E+04	94	TRUE	TRUE	
1	NKAISQIQE	910	918	9	HLA-A*02:01	910	94	NKAISQIQE	NKAISQIQE	45050.08	94	4.51E+04	94	TRUE	TRUE	
1	DATSTGNYN	429	437	9	HLA-A*02:01	429	94	DATSTGNYN	DATSTGNYN	45108.6	94	4.51E+04	94	TRUE	TRUE	
1	DFTDSVRDP	557	565	9	HLA-A*02:01	557	94	DFTDSVRDP	DFTDSVRDP	45191.15	94	4.52E+04	94	TRUE	TRUE	
1	EELDKYFKN	1132	1140	9	HLA-A*02:01	1132	94	EELDKYFKN	EELDKYFKN	45193.1	94	4.52E+04	94	TRUE	TRUE	
1	PKLSTDLIK	513	521	9	HLA-A*02:01	513	95	PKLSTDLIK	PKLSTDLIK	45261.62	95	4.53E+04	95	TRUE	TRUE	
1	VYKGYQPID	196	204	9	HLA-A*02:01	196	95	VYKGYQPID	VYKGYQPID	45283.66	95	4.53E+04	95	TRUE	TRUE	
1	SPQIITTDN	1093	1101	9	HLA-A*02:01	1093	95	SPQIITTDN	SPQIITTDN	45335.61	95	4.53E+04	95	TRUE	TRUE	
1	GDDVRQIAP	391	399	9	HLA-A*02:01	391	95	GDDVRQIAP	GDDVRQIAP	45340.03	95	4.53E+04	95	TRUE	TRUE	
1	LDKYFKNHT	1134	1142	9	HLA-A*02:01	1134	95	LDKYFKNHT	LDKYFKNHT	45377.33	95	4.54E+04	95	TRUE	TRUE	
1	NPVIPFKDG	78	86	9	HLA-A*02:01	78	95	NPVIPFKDG	NPVIPFKDG	45378.82	95	4.54E+04	95	TRUE	TRUE	
1	EKSNVVRGW	93	101	9	HLA-A*02:01	93	95	EKSNVVRGW	EKSNVVRGW	45421.07	95	4.54E+04	95	TRUE	TRUE	
1	LRPFERDIS	448	456	9	HLA-A*02:01	448	95	LRPFERDIS	LRPFERDIS	45444.16	95	4.54E+04	95	TRUE	TRUE	
1	NKVTLADAG	806	814	9	HLA-A*02:01	806	95	NKVTLADAG	NKVTLADAG	45462.86	95	4.55E+04	95	TRUE	TRUE	
1	GKLQDVVNQ	928	936	9	HLA-A*02:01	928	96	GKLQDVVNQ	GKLQDVVNQ	45532.25	96	4.55E+04	96	TRUE	TRUE	
1	DCSQNPLAE	277	285	9	HLA-A*02:01	277	96	DCSQNPLAE	DCSQNPLAE	45550.49	96	4.56E+04	96	TRUE	TRUE	
1	GNPVIPFKD	77	85	9	HLA-A*02:01	77	96	GNPVIPFKD	GNPVIPFKD	45628.93	96	4.56E+04	96	TRUE	TRUE	
1	DSFVVKGDD	385	393	9	HLA-A*02:01	385	96	DSFVVKGDD	DSFVVKGDD	45648.68	96	4.56E+04	96	TRUE	TRUE	
1	SKRFQPFQQ	542	550	9	HLA-A*02:01	542	96	SKRFQPFQQ	SKRFQPFQQ	45671.89	96	4.57E+04	96	TRUE	TRUE	
1	YDPLQPELD	1120	1128	9	HLA-A*02:01	1120	96	YDPLQPELD	YDPLQPELD	45712.43	96	4.57E+04	96	TRUE	TRUE	
1	SKPMGTQTH	141	149	9	HLA-A*02:01	141	96	SKPMGTQTH	SKPMGTQTH	45712.93	96	4.57E+04	96	TRUE	TRUE	
1	NYKYRYLRH	437	445	9	HLA-A*02:01	437	96	NYKYRYLRH	NYKYRYLRH	45824.36	96	4.58E+04	96	TRUE	TRUE	
1	GKAYFPREG	1067	1075	9	HLA-A*02:01	1067	97	GKAYFPREG	GKAYFPREG	45864.54	97	4.59E+04	97	TRUE	TRUE	
1	CCKFDEDDS	1235	1243	9	HLA-A*02:01	1235	97	CCKFDEDDS	CCKFDEDDS	45925.1	97	4.59E+04	97	TRUE	TRUE	
1	TDSVRDPKT	559	567	9	HLA-A*02:01	559	97	TDSVRDPKT	TDSVRDPKT	45929.57	97	4.59E+04	97	TRUE	TRUE	
1	AEQDRNTRE	754	762	9	HLA-A*02:01	754	97	AEQDRNTRE	AEQDRNTRE	45931.58	97	4.59E+04	97	TRUE	TRUE	
1	PNYTQHTSS	28	36	9	HLA-A*02:01	28	97	PNYTQHTSS	PNYTQHTSS	45939.02	97	4.59E+04	97	TRUE	TRUE	
1	DSVRDPKTS	560	568	9	HLA-A*02:01	560	97	DSVRDPKTS	DSVRDPKTS	45941.51	97	4.59E+04	97	TRUE	TRUE	
1	PGQTGVIAD	399	407	9	HLA-A*02:01	399	97	PGQTGVIAD	PGQTGVIAD	45979.3	97	4.60E+04	97	TRUE	TRUE	
1	TEKSNVVRG	92	100	9	HLA-A*02:01	92	97	TEKSNVVRG	TEKSNVVRG	46075.92	97	4.61E+04	97	TRUE	TRUE	
1	TPGTNASSE	585	593	9	HLA-A*02:01	585	97	TPGTNASSE	TPGTNASSE	46206.22	97	4.62E+04	97	TRUE	TRUE	
1	SCCKFDEDD	1234	1242	9	HLA-A*02:01	1234	98	SCCKFDEDD	SCCKFDEDD	46289.8	98	4.63E+04	98	TRUE	TRUE	
1	DPLQPELDS	1121	1129	9	HLA-A*02:01	1121	98	DPLQPELDS	DPLQPELDS	46306.31	98	4.63E+04	98	TRUE	TRUE	
1	LNEVAKNLN	1168	1176	9	HLA-A*02:01	1168	98	LNEVAKNLN	LNEVAKNLN	46312.34	98	4.63E+04	98	TRUE	TRUE	
1	TYVPSQERN	1048	1056	9	HLA-A*02:01	1048	98	TYVPSQERN	TYVPSQERN	46316.34	98	4.63E+04	98	TRUE	TRUE	
1	QPELDSFKE	1124	1132	9	HLA-A*02:01	1124	98	QPELDSFKE	QPELDSFKE	46429.72	98	4.64E+04	98	TRUE	TRUE	
1	VKSFEIDKG	290	298	9	HLA-A*02:01	290	98	VKSFEIDKG	VKSFEIDKG	46440.29	98	46440.29	98	TRUE	TRUE	
1	PREGVFVFN	1072	1080	9	HLA-A*02:01	1072	98	PREGVFVFN	PREGVFVFN	46551.45	98	46551.45	98	TRUE	TRUE	
1	EDDSEPVLK	1240	1248	9	HLA-A*02:01	1240	99	EDDSEPVLK	EDDSEPVLK	46595.3	99	46595.3	99	TRUE	TRUE	
1	TDAVDCSQN	273	281	9	HLA-A*02:01	273	99	TDAVDCSQN	TDAVDCSQN	46694.21	99	46694.21	99	TRUE	TRUE	
1	DRLITGRLQ	976	984	9	HLA-A*02:01	976	99	DRLITGRLQ	DRLITGRLQ	46703.81	99	46703.81	99	TRUE	TRUE	
1	KNHTSPDVD	1139	1147	9	HLA-A*02:01	1139	99	KNHTSPDVD	KNHTSPDVD	46713.93	99	46713.93	99	TRUE	TRUE	
1	QDVVNQNAQ	931	939	9	HLA-A*02:01	931	99	QDVVNQNAQ	QDVVNQNAQ	46758.93	99	46758.93	99	TRUE	TRUE	
1	GKLRPFERD	446	454	9	HLA-A*02:01	446	99	GKLRPFERD	GKLRPFERD	46764	99	46764	99	TRUE	TRUE	
1	ENQKQIANQ	900	908	9	HLA-A*02:01	900	99	ENQKQIANQ	ENQKQIANQ	46788.8	99	46788.8	99	TRUE	TRUE	
1	EFVFKNKDG	184	192	9	HLA-A*02:01	184	99	EFVFKNKDG	EFVFKNKDG	46806	99	46806	99	TRUE	TRUE	
1	LDVSEKSGN	170	178	9	HLA-A*02:01	170	99	LDVSEKSGN	LDVSEKSGN	46910.44	99	46910.44	99	TRUE	TRUE	
1	AWERKKISN	339	347	9	HLA-A*02:01	339	99	AWERKKISN	AWERKKISN	46924.14	99	46924.14	99	TRUE	TRUE	
1	PELDSFKEE	1125	1133	9	HLA-A*02:01	1125	99	PELDSFKEE	PELDSFKEE	46932.29	99	46932.29	99	TRUE	TRUE	
1	QKQIANQFN	902	910	9	HLA-A*02:01	902	99	QKQIANQFN	QKQIANQFN	47012.59	99	47012.59	99	TRUE	TRUE	
1	DPLKPTKRS	790	798	9	HLA-A*02:01	790	99	DPLKPTKRS	DPLKPTKRS	47022.23	99	47022.23	99	TRUE	TRUE	
1	LDKVEAEVQ	966	974	9	HLA-A*02:01	966	100	LDKVEAEVQ	LDKVEAEVQ	47138.4	100	47138.4	100	TRUE	TRUE	
1	PDVDLGDIS	1144	1152	9	HLA-A*02:01	1144	100	PDVDLGDIS	PDVDLGDIS	47167.47	100	47167.47	100	TRUE	TRUE	
1	DDSEPVLKG	1241	1249	9	HLA-A*02:01	1241	100	DDSEPVLKG	DDSEPVLKG	47209.84	100	47209.84	100	TRUE	TRUE	
1	DPKTSEILD	564	572	9	HLA-A*02:01	564	100	DPKTSEILD	DPKTSEILD	47267.6	100	47267.6	100	TRUE	TRUE	
1	DYNYKLPDD	407	415	9	HLA-A*02:01	407	100	DYNYKLPDD	DYNYKLPDD	47280.4	100	47280.4	100	TRUE	TRUE	
1	QKEIDRLNE	1162	1170	9	HLA-A*02:01	1162	100	QKEIDRLNE	QKEIDRLNE	47429.5	100	47429.5	100	TRUE	TRUE	
1	PDGKPCTPP	462	470	9	HLA-A*02:01	462	100	PDGKPCTPP	PDGKPCTPP	47490.59	100	47490.59	100	TRUE	TRUE	
1	DRLNEVAKN	1166	1174	9	HLA-A*02:01	1166	100	DRLNEVAKN	DRLNEVAKN	47790.61	100	47790.61	100	TRUE	TRUE	
1	DKGIYQTSN	296	304	9	HLA-A*02:01	296	100	DKGIYQTSN	DKGIYQTSN	48200.85	100	48200.85	100	TRUE	TRUE	
1	PDEIFRSDT	43	51	9	HLA-A*02:01	43	100	PDEIFRSDT	PDEIFRSDT	48213.35	100	48213.35	100	TRUE	TRUE	
1	PDPLKPTKR	789	797	9	HLA-A*02:01	789	100	PDPLKPTKR	PDPLKPTKR	48243.11	100	48243.11	100	TRUE	TRUE	

seq #	peptide	start	end	peptide length	allele	peptide index	median binding	consensus perc	smm IC50	smm percentil	ann IC50	ann percentile	comblib_sidne	comblib_sidne	legacy consens	percentiles equal	
1	FSISITTEV	700	708	9	HLA-A*02:01	700	0.27	0.27	111.9721455	1.8	30.13	0.27	2.64E-06	0.2	0.27	TRUE	
1	FIAGLIAIV	1202	1210	9	HLA-A*02:01	1202	0.4	0.4	20.18877649	0.4	10.29	0.09	2.89E-05	1.7	0.4	TRUE	
1	VLYNSTFFS	354	362	9	HLA-A*02:01	354	0.64	0.64	182.0161819	2.6	70.21	0.64	3.72E-06	0.3	0.64	TRUE	
1	RLQSLQTYV	982	990	9	HLA-A*02:01	982	0.7	0.7	36.73753391	0.7	16.66	0.16	1.09E-05	0.8	0.7	TRUE	
1	FIFLLFLTL	2	10	9	HLA-A*02:01	2	0.7	0.7	41.03080144	0.7	71.52	0.66	0.000171	9.1	0.7	TRUE	
1	VIADYNYKL	404	412	9	HLA-A*02:01	404	0.9	0.9	51.6547186	0.9	50.27	0.46	0.00017824	9.4	0.9	TRUE	
1	RLDKVEAEV	965	973	9	HLA-A*02:01	965	0.9	0.9	53.2243051	0.9	38.95	0.35	1.47E-05	0.9	0.9	TRUE	
1	YLKPTTFML	256	264	9	HLA-A*02:01	256	1	1	54.33879457	1	18.56	0.17	5.55E-05	3.2	1	TRUE	
1	VLNDILSRL	958	966	9	HLA-A*02:01	958	1.1	1.1	58.35929005	1.1	33.57	0.3	9.73E-05	5.5	1.1	TRUE	
1	HLMSFPQAA	1030	1038	9	HLA-A*02:01	1030	1.1	1.1	101.6506125	1.6	30.89	0.28	1.88E-05	1.1	1.1	TRUE	
1	LLFNKVTLA	803	811	9	HLA-A*02:01	803	1.2	1.2	63.69568317	1.2	25.41	0.22	7.52E-05	4.3	1.2	TRUE	
1	VVFLHVTYV	1042	1050	9	HLA-A*02:01	1042	1.2	1.2	64.87987433	1.2	36.56	0.33	6.17E-05	3.6	1.2	TRUE	
1	MIAAYTAAL	851	859	9	HLA-A*02:01	851	1.2	1.2	65.32960014	1.2	14.39	0.13	0.00022542	13	1.2	TRUE	
1	FLLFLTLTS	4	12	9	HLA-A*02:01	4	1.2	1.2	158.8949154	2.3	141.86	1.2	1.52E-05	1	1.2	TRUE	
1	LLTDDMIAA	846	854	9	HLA-A*02:01	846	1.4	1.4	78.00276543	1.4	32.91	0.29	0.00011376	6.3	1.4	TRUE	
1	GTSAAAYFV	246	254	9	HLA-A*02:01	246	1.4	1.4	269.8422814	3.4	167.91	1.4	9.62E-06	0.7	1.4	TRUE	
1	ELLNAPATV	502	510	9	HLA-A*02:01	502	2	2	293.8393807	3.6	279.55	2	1.93E-05	1.2	2	TRUE	
1	NVYADSFVV	381	389	9	HLA-A*02:01	381	2.3	2.3	151.7434661	2.3	87.86	0.77	6.53E-05	3.7	2.3	TRUE	
1	KQMYKTPTL	768	776	9	HLA-A*02:01	768	2.3	2.3	154.5645878	2.3	47.7	0.43	7.24E-05	4.1	2.3	TRUE	
1	SIVAYTMSL	673	681	9	HLA-A*02:01	673	2.3	2.3	164.1005362	2.3	37.41	0.33	0.00034914	17	2.3	TRUE	
1	ELCDNPFFA	131	139	9	HLA-A*02:01	131	2.5	2.5	306.2739079	3.7	408.87	2.5	4.03E-05	2.3	2.5	TRUE	
1	ALVSGTATA	858	866	9	HLA-A*02:01	858	2.6	2.6	204.225515	2.8	122.57	0.97	4.57E-05	2.6	2.6	TRUE	
1	GLTVLPPLL	839	847	9	HLA-A*02:01	839	2.8	2.8	207.5439127	2.8	130.4	1.2	0.00025177	14	2.8	TRUE	
1	NLNESLIDL	1174	1182	9	HLA-A*02:01	1174	2.8	2.8	208.9825384	2.8	177.32	1.4	0.00011041	6.2	2.8	TRUE	
1	FSTFKCYGV	361	369	9	HLA-A*02:01	361	2.8	2.8	407.4834739	4.2	504.47	2.8	3.72E-05	2.1	2.8	TRUE	
1	YVYKGYQPI	195	203	9	HLA-A*02:01	195	2.9	2.9	215.332707	2.9	47.04	0.43	0.00012359	6.8	2.9	TRUE	
1	SITTEVMPV	703	711	9	HLA-A*02:01	703	2.9	2.9	217.8261422	2.9	50.81	0.46	0.00017498	9.3	2.9	TRUE	
1	FVSGNCDVV	1103	1111	9	HLA-A*02:01	1103	3	3	230.202481	3	290.37	2	0.00011015	6.2	3	TRUE	
1	KQLSSNFGA	946	954	9	HLA-A*02:01	946	3.2	3.2	242.1642333	3.2	141.16	1.2	0.00012882	7	3.2	TRUE	
1	TSYECDIPI	644	652	9	HLA-A*02:01	644	3.2	3.2	1282.655418	8.4	639.51	3.2	1.17E-05	0.8	3.2	TRUE	
1	ATAGWTFGA	864	872	9	HLA-A*02:01	864	3.3	3.3	258.291432	3.3	207.35	1.6	0.00024099	13	3.3	TRUE	
1	GLIAIVMVT	1205	1213	9	HLA-A*02:01	1205	3.3	3.3	260.681373	3.3	242.04	1.8	6.85E-05	3.9	3.3	TRUE	
1	FLPFYSNVT	59	67	9	HLA-A*02:01	59	3.3	3.3	261.8846237	3.3	394.17	2.4	0.00033037	17	3.3	TRUE	
1	ILLCCMTSC	1214	1222	9	HLA-A*02:01	1214	3.3	3.3	264.3078123	3.3	403.87	2.5	0.0002319	13	3.3	TRUE	
1	IIINNSTNV	115	123	9	HLA-A*02:01	115	3.4	3.4	267.9847002	3.4	228.99	1.7	0.00033343	17	3.4	TRUE	
1	IITTDNTFV	1096	1104	9	HLA-A*02:01	1096	3.4	3.4	267.9847002	3.4	338.82	2.2	0.00043954	21	3.4	TRUE	
1	ALGKLQDVV	926	934	9	HLA-A*02:01	926	3.6	3.6	291.1454451	3.6	717.01	3.4	0.00015922	8.5	3.6	TRUE	
1	AIVMVTILL	1208	1216	9	HLA-A*02:01	1208	3.6	3.6	291.8166046	3.6	711.15	3.4	0.00031117	16	3.6	TRUE	
1	TMNNKSQSV	106	114	9	HLA-A*02:01	106	3.7	3.7	306.2739079	3.7	254.01	1.8	0.0001932	12	3.7	TRUE	
1	VLSFELLNA	498	506	9	HLA-A*02:01	498	3.7	3.7	314.854569	3.7	273.66	2	0.00104232	35	3.7	TRUE	
1	YQDVNCTDV	598	606	9	HLA-A*02:01	598	3.8	3.8	322.1884741	3.8	612.82	3.2	0.00070795	28	3.8	TRUE	
1	CMTSCCSCL	1218	1226	9	HLA-A*02:01	1218	4	4	350.8407252	4	629.65	3.2	0.00036141	18	4	TRUE	
1	RVVVLSFEL	495	503	9	HLA-A*02:01	495	4	4	364.0072218	4	419.83	2.5	0.00293765	59	4	TRUE	
1	SLTTTSTAL	919	927	9	HLA-A*02:01	919	4	4	364.8463451	4	300.04	2	0.00316228	60	4	TRUE	
1	VLAWNTRNI	420	428	9	HLA-A*02:01	420	4.1	4.1	380.2857045	4.1	808.52	3.7	9.04E-05	5.1	4.1	TRUE	
1	VIRACNFEL	124	132	9	HLA-A*02:01	124	4.1	4.1	385.5760057	4.1	786.23	3.6	0.00012942	7.1	4.1	TRUE	
1	LLQYGSFCT	735	743	9	HLA-A*02:01	735	4.1	4.1	391.8411118	4.1	541.11	2.9	0.0012331	39	4.1	TRUE	
1	YLTQDLFLP	53	61	9	HLA-A*02:01	53	4.2	4.2	242.7224795	3.2	975.48	4.2	0.00027359	15	4.2	TRUE	
1	VLYENQKQI	897	905	9	HLA-A*02:01	897	4.2	4.2	399.1259819	4.2	562.95	3	8.85E-05	5	4.2	TRUE	
1	LIAIVMVTI	1206	1214	9	HLA-A*02:01	1206	4.3	4.3	419.8653158	4.3	691.85	3.4	0.00435512	68	4.3	TRUE	
1	FMKQYGECL	815	823	9	HLA-A*02:01	815	4.3	4.3	422.7756832	4.3	698.66	3.4	0.00083946	31	4.3	TRUE	
1	KLPDDFMGC	411	419	9	HLA-A*02:01	411	4.4	4.4	439.6529586	4.4	1013.85	4.3	0.00054866	24	4.4	TRUE	
1	AILTAFSPA	233	241	9	HLA-A*02:01	233	4.6	4.6	457.2039776	4.6	351.2	2.3	0.00049317	22	4.6	TRUE	
1	MIFDNAFNC	151	159	9	HLA-A*02:01	151	4.6	4.6	473.271116	4.6	421.14	2.5	0.00016916	9	4.6	TRUE	
1	FLHVTYVPS	1044	1052	9	HLA-A*02:01	1044	4.7	4.7	979.7381063	7.3	1187.08	4.7	6.49E-05	3.7	4.7	TRUE	
1	YQPYRVVVL	491	499	9	HLA-A*02:01	491	4.8	4.8	502.4698412	4.8	862.1	3.9	0.00554626	73	4.8	TRUE	
1	PLAELKCSV	282	290	9	HLA-A*02:01	282	4.9	4.9	370.7746213	4.1	1451.87	5.3	8.49E-05	4.9	4.9	TRUE	
1	YQTSNFRVV	300	308	9	HLA-A*02:01	300	4.9	4.9	518.9314595	4.9	1149.79	4.6	0.00040551	20	4.9	TRUE	
1	TQAGCLIGA	631	639	9	HLA-A*02:01	631	4.9	4.9	524.9404026	4.9	551.45	3	0.00010139	5.7	4.9	TRUE	
1	QMAYRFNGI	883	891	9	HLA-A*02:01	883	4.9	4.9	526.1505152	4.9	550.3	3	0.00257632	56	4.9	TRUE	
1	GICASYHTV	655	663	9	HLA-A*02:01	655	4.9	4.9	528.5791156	4.9	546.07	3	0.0006223	26	4.9	TRUE	
1	RLNEVAKNL	1167	1175	9	HLA-A*02:01	1167	5.1	5.1	562.4837755	5.1	940.92	4.1	0.00054325	24	5.1	TRUE	
1	GIGYQPYRV	488	496	9	HLA-A*02:01	488	5.2	5.2	472.1826226	4.6	1383.29	5.2	0.00058345	25	5.2	TRUE	
1	TSVDCNMYI	716	724	9	HLA-A*02:01	716	5.2	5.2	1503.522737	9.2	1314.15	5	9.26E-05	5.2	5.2	TRUE	
1	IAIPTNFSI	694	702	9	HLA-A*02:01	694	5.3	5.3	604.101619	5.3	487.32	2.8	0.00016527	8.8	5.3	TRUE	
1	QIAPGQTGV	396	404	9	HLA-A*02:01	396	5.4	5.4	507.1191374	4.9	1487.84	5.4	0.00018365	9.8	5.4	TRUE	
1	QYIKWPWYV	1190	1198	9	HLA-A*02:01	1190	5.6	5.6	191.0337056	2.7	1620.14	5.6	0.00014561	7.9	5.6	TRUE	
1	WLGFIAGLI	1199	1207	9	HLA-A*02:01	1199	5.6	5.6	605.4942175	5.3	1603.03	5.6	0.00039355	19	5.6	TRUE	
1	ALNTLVKQL	940	948	9	HLA-A*02:01	940	5.7	5.7	601.3260247	5.3	1633.23	5.7	0.00137721	41	5.7	TRUE	
1	VLYQDVNCT	596	604	9	HLA-A*02:01	596	6	6	698.4092778	6	1463.83	5.3	0.00011561	6.4	6	TRUE	
1	IINNSTNVV	116	124	9	HLA-A*02:01	116	6.1	6.1	724.6194716	6.1	1073.84	4.4	0.00011041	6.2	6.1	TRUE	
1	FVFNGTSWF	1077	1085	9	HLA-A*02:01	1077	6.1	6.1	1151.091906	7.8	1869.65	6.1	1.65E-05	1	6.1	TRUE	
1	KLNDLCFSN	373	381	9	HLA-A*02:01	373	6.2	6.2	450.9309038	4.5	3317	8.7	0.00011148	6.2	6.2	TRUE	
1	VWLGFIAGL	1198	1206	9	HLA-A*02:01	1198	6.5	6.5	529.7976163	4.9	2036.12	6.5	0.0006246	26	6.5	TRUE	
1	FNATKFPSV	329	337	9	HLA-A*02:01	329	6.5	6.5	783.6280984	6.5	2673.48	7.6	3.12E-05	1.8	6.5	TRUE	
1	RIYSTGNNV	620	628	9	HLA-A*02:01	620	6.5	6.5	891.4767066	6.9	1067.65	4.4	0.00011722	6.5	6.5	TRUE	
1	QLNRALSGI	744	752	9	HLA-A*02:01	744	6.7	6.7	837.7414421	6.7	1406.79	5.2	0.00026977	15	6.7	TRUE	
1	YVTQQLIRA	989	997	9	HLA-A*02:01	989	6.7	6.7	837.7414421	6.7	1577.6	5.6	0.00098175	34	6.7	TRUE	
1	LLLQYGSFC	734	742	9	HLA-A*02:01	734	6.8	6.8	719.6312263	6.1	2742.92	7.7	0.0001234	6.8	6.8	TRUE	
1	VIGIINNTV	1111	1119	9	HLA-A*02:01	1111	6.9	6.9	877.2229799	6.9	1299.39	5	0.00062087	26	6.9	TRUE	
1	VMVTILLCC	1210	1218	9	HLA-A*02:01	1210	6.9	6.9	901.7995204	6.9	1723.82	5.9	0.0081903	81	6.9	TRUE	
1	IAAYTAALV	852	860	9	HLA-A*02:01	852	7	7	914.3448014	7	1800.7	6	0.00035335	18	7	TRUE	
1	MFIFLLFLT	1	9	9	HLA-A*02:01	1	7.1	7.1	865.1870294	6.8	2359.69	7.1	0.0008931	32	7.1	TRUE	
1	CTFEYISDA	159	167	9	HLA-A*02:01	159	7.1	7.1	924.932415	7.1	1478.53	5.4	0.00058884	25	7.1	TRUE	
1	FMLKYDENG	262	270	9	HLA-A*02:01	262	7.3	7.3	711.3936757	6.1	2462.27	7.3	0.00049204	22	7.3	TRUE	
1	LFLPFYSNV	58	66	9	HLA-A*02:01	58	7.3	7.3	929.2017092	7.1	2506	7.3	0.00025229	14	7.3	TRUE	
1	QLSSNFGAI	947	955	9	HLA-A*02:01	947	7.4	7.4	1007.186741	7.4	1808.71	6	0.00021928	13	7.4	TRUE	
1	YVWLGFIAG	1197	1205	9	HLA-A*02:01	1197	7.5	7.5	887.3807432	6.9	2568.76	7.5	0.00045082	21	7.5	TRUE	
1	FMGCVLAWN	416	424	9	HLA-A*02:01	416	7.6	7.6	476.5516746	4.6	2674.95	7.6	0.00102141	35	7.6	TRUE	
1	PLNDYGFYT	477	485	9	HLA-A*02:01	477	7.6	7.6	599.9430132	5.3	2662.8	7.6	0.00016285	8.7	7.6	TRUE	
1	AVDCSQNPL	275	283	9	HLA-A*02:01	275	7.6	7.6	1086.700833	7.6	1719.38	5.8	0.00066374	27	7.6	TRUE	
1	LQIPFAMQM	876	884	9	HLA-A*02:01	876	7.7	7.7	1091.716816	7.7	896.61	4	0.00067764	27	7.7	TRUE	
1	LLFLTLTSG	5	13	9	HLA-A*02:01	5	7.7	7.7	1112.013347	7.7	1676.81	5.7	0.00018535	9.9	7.7	TRUE	
1	SLIDLQELG	1178	1186	9	HLA-A*02:01	1178	8	8	1183.34124	8	2973.3	8.1	5.15E-05	2.9	8	TRUE	
1	KEIDRLNEV	1163	1171	9	HLA-A*02:01	1163	8.1	8.1	1205.341196	8.1	6029.01	14	5.75E-05	3.4	8.1	TRUE	
1	ELDSFKEEL	1126	1134	9	HLA-A*02:01	1126	8.1	8.1	1849.737069	12	3001.36	8.1	8.07E-05	4.7	8.1	TRUE	
1	AAAYFVGYL	249	257	9	HLA-A*02:01	249	8.3	8.3	1276.762152	8.3	1975.49	6.4	0.00134338	41	8.3	TRUE	
1	ILPDPLKPT	787	795	9	HLA-A*02:01	787	8.3	8.3	1496.614677	9.1	2037.13	6.5	0.00015417	8.3	8.3	TRUE	
1	GFIAGLIAI	1201	1209	9	HLA-A*02:01	1201	8.6	8.6	1346.201283	8.6	3496.47	9	0.0001114	6.2	8.6	TRUE	
1	GINASVVNI	1153	1161	9	HLA-A*02:01	1153	8.7	8.7	1374.390042	8.7	1346.83	5.1	0.00124165	39	8.7	TRUE	
1	FITQRNFFS	1085	1093	9	HLA-A*02:01	1085	8.7	8.7	1774.638912	10	3306.75	8.7	6.24E-05	3.6	8.7	TRUE	
1	YTAALVSGT	855	863	9	HLA-A*02:01	855	8.8	8.8	1403.16906	8.8	1924.22	6.3	0.00042364	20	8.8	TRUE	
1	FPQAAPHGV	1034	1042	9	HLA-A*02:01	1034	8.8	8.8	1422.689086	8.8	11255.93	21	3.07E-05	1.8	8.8	TRUE	
1	FQTQAGCLI	629	637	9	HLA-A*02:01	629	8.9	8.9	1449.138851	8.9	2136.71	6.7	0.00045709	21	8.9	TRUE	
1	DVSDFTDSV	554	562	9	HLA-A*02:01	554	9	9	1469.298381	9	7938.29	17	9.21E-05	5.2	9	TRUE	
1	LITGRLQSL	978	986	9	HLA-A*02:01	978	9.2	9.2	1517.434661	9.2	1675.27	5.7	0.00147571	43	9.2	TRUE	
1	LCDNPFFAV	132	140	9	HLA-A*02:01	132	9.2	9.2	1730.25455	9.9	2259.03	6.9	0.00017155	9.2	9.2	TRUE	
1	FKDGIYFAA	83	91	9	HLA-A*02:01	83	9.3	9.3	1023.552209	7.4	3682.77	9.3	0.00029376	15	9.3	TRUE	
1	MQMAYRFNG	882	890	9	HLA-A*02:01	882	9.3	9.3	1137.915466	7.8	3716	9.3	0.00028314	15	9.3	TRUE	
1	AISSVLNDI	954	962	9	HLA-A*02:01	954	9.3	9.3	1545.645878	9.3	1829.77	6.1	0.00093972	33	9.3	TRUE	
1	SIAYSNNTI	686	694	9	HLA-A*02:01	686	9.3	9.3	1567.147954	9.3	2910.15	8	0.00171002	47	9.3	TRUE	
1	NTLKPIFKL	214	222	9	HLA-A*02:01	214	9.5	9.5	1585.294672	9.5	3454.35	8.9	0.0008414	31	9.5	TRUE	
1	SLGADSSIA	680	688	9	HLA-A*02:01	680	9.7	9.7	831.974471	6.6	3949.05	9.7	0.00075858	29	9.7	TRUE	
1	KLPLGINIT	221	229	9	HLA-A*02:01	221	9.7	9.7	1663.83402	9.7	3200.59	8.5	0.00038637	19	9.7	TRUE	
1	KMSECVLGQ	1010	1018	9	HLA-A*02:01	1010	9.8	9.8	1371.229033	8.7	4047.12	9.8	0.0004761	22	9.8	TRUE	
1	AISQIQESL	912	920	9	HLA-A*02:01	912	9.8	9.8	1690.869148	9.8	2333.11	7.1	0.00129718	40	9.8	TRUE	
1	AALQIPFAM	874	882	9	HLA-A*02:01	874	9.9	9.9	1730.25455	9.9	2535.79	7.4	0.00118359	38	9.9	TRUE	
1	AAPHGVVFL	1037	1045	9	HLA-A*02:01	1037	10	10	1750.289954	10	3528.66	9.1	0.00264363	56	10	TRUE	
1	WTFGAGAAL	868	876	9	HLA-A*02:01	868	10	10	1770.557356	10	1468.2	5.3	0.0027227	57	10	TRUE	
1	IVMVTILLC	1209	1217	9	HLA-A*02:01	1209	11	11	1786.940122	11	2591.76	7.5	0.00055886	24	11	TRUE	
1	FAMQMAYRF	880	888	9	HLA-A*02:01	880	11	11	1923.57888	12	4263.5	11	2.31E-06	0.2	11	TRUE	
1	IKWPWYVWL	1192	1200	9	HLA-A*02:01	1192	12	12	726.289892	6.1	4469.74	12	0.00161436	45	12	TRUE	

seq #	peptide	start	end	peptide length	allele	peptide index	median bindin	consensus perc	smm IC50	smm percentil	ann IC50	ann percentile	comblib_sidne	comblib_sidne	legacy consens	percentiles equal
1	VFNGTSWFI	1078	1086	9	HLA-A*02:01	1078	12	12	1412.895364	8.8	4422.26	12	0.00127028	39	12	TRUE
1	ISDAFSLDV	164	172	9	HLA-A*02:01	164	12	12	1884.126249	12	3153.15	8.4	0.0003096	16	12	TRUE
1	GINITNFRA	225	233	9	HLA-A*02:01	225	12	12	1905.943402	12	4954.26	12	0.00215774	52	12	TRUE
1	FDEDDSEPV	1238	1246	9	HLA-A*02:01	1238	12	12	1972.922383	12	11257.51	21	1.63E-05	1	12	TRUE
1	VVVLSFELL	496	504	9	HLA-A*02:01	496	12	12	2000.368468	12	3574.54	9.1	0.00204174	50	12	TRUE
1	ELGKYEQYI	1184	1192	9	HLA-A*02:01	1184	12	12	2065.903351	12	4787.32	12	6.27E-05	3.6	12	TRUE
1	YGFYTTTGI	481	489	9	HLA-A*02:01	481	12	12	2075.439127	12	4807.2	12	0.00066222	27	12	TRUE
1	FLYVYKGYQ	193	201	9	HLA-A*02:01	193	12	12	2114.024421	12	5906.21	14	5.13E-05	2.9	12	TRUE
1	PALNCYWPL	470	478	9	HLA-A*02:01	470	12	12	2168.25322	12	5293.19	13	7.67E-05	4.4	12	TRUE
1	GAALQIPFA	873	881	9	HLA-A*02:01	873	12	12	2312.650474	13	4492.48	12	0.00010695	6	12	TRUE
1	FSQILPDPL	784	792	9	HLA-A*02:01	784	13	13	2046.963035	12	5056.67	13	0.00035547	18	13	TRUE
1	VYDPLQPEL	1119	1127	9	HLA-A*02:01	1119	13	13	2234.138023	13	14708.49	26	0.00016604	8.8	13	TRUE
1	IAIVMVTIL	1207	1215	9	HLA-A*02:01	1207	13	13	2328.681001	13	4092.03	9.9	0.00067019	27	13	TRUE
1	MVTILLCCM	1211	1219	9	HLA-A*02:01	1211	13	13	2483.762124	13	3125.64	8.4	0.00719449	79	13	TRUE
1	FIEDLLFNK	799	807	9	HLA-A*02:01	799	13	13	2524.120012	13	8286.79	17	0.00019692	12	13	TRUE
1	AGLIAIVMV	1204	1212	9	HLA-A*02:01	1204	13	13	2524.12001	13	5595.06	13	0.00017022	9.1	13	TRUE
1	NAQALNTLV	937	945	9	HLA-A*02:01	937	13	13	2559.234019	13	6555.94	15	0.00017832	9.4	13	TRUE
1	SNFGAISSV	950	958	9	HLA-A*02:01	950	13	13	2565.133662	13	6207.81	14	3.92E-05	2.2	13	TRUE
1	LLCCMTSCC	1215	1223	9	HLA-A*02:01	1215	13	13	2938.393807	14	5179.09	13	6.48E-05	3.7	13	TRUE
1	FAATEKSNV	89	97	9	HLA-A*02:01	89	14	14	1738.241042	10	6445.23	15	0.00024558	14	14	TRUE
1	ILDISPCSF	570	578	9	HLA-A*02:01	570	14	14	2168.25322	12	6194.59	14	0.00073451	29	14	TRUE
1	NGLTVLPPL	838	846	9	HLA-A*02:01	838	14	14	2588.868552	14	6240.94	14	0.00015996	8.6	14	TRUE
1	NTREVFAQV	759	767	9	HLA-A*02:01	759	14	14	2643.078123	14	5448.1	13	0.00121899	38	14	TRUE
1	QQLIRAAEI	992	1000	9	HLA-A*02:01	992	14	14	2710.878168	14	5078.32	13	0.00074302	29	14	TRUE
1	FKLPLGINI	220	228	9	HLA-A*02:01	220	14	14	2723.391013	14	6821.73	15	5.11E-05	2.9	14	TRUE
1	LQTYVTQQL	986	994	9	HLA-A*02:01	986	14	14	2825.595563	14	3910.02	9.6	0.00108643	36	14	TRUE
1	KLQDVVNQN	929	937	9	HLA-A*02:01	929	14	14	2931.635689	14	14583.15	26	0.00016489	8.8	14	TRUE
1	FFSPQIITT	1091	1099	9	HLA-A*02:01	1091	14	14	2931.635689	14	8456.81	17	6.47E-05	3.7	14	TRUE
1	DILSRLDKV	961	969	9	HLA-A*02:01	961	14	14	2951.956816	14	13420.04	24	0.00017187	9.2	14	TRUE
1	ASYHTVSLL	658	666	9	HLA-A*02:01	658	14	14	2958.76178	14	4557.1	12	0.00067733	27	14	TRUE
1	VAYTMSLGA	675	683	9	HLA-A*02:01	675	14	14	3055.694975	14	4409.45	12	0.00071812	28	14	TRUE
1	TALGKLQDV	925	933	9	HLA-A*02:01	925	14	14	3838.044448	16	6323.53	14	0.00015639	8.4	14	TRUE
1	TLYLTQDLF	51	59	9	HLA-A*02:01	51	14	14	5650.800826	19	5741.19	14	0.00010046	5.7	14	TRUE
1	DISGINASV	1150	1158	9	HLA-A*02:01	1150	15	15	1663.83402	9.7	6745.18	15	0.00031275	16	15	TRUE
1	DLPSGFNTL	208	216	9	HLA-A*02:01	208	15	15	1824.357725	12	6837.1	15	0.00029936	16	15	TRUE
1	AQDIWGTSA	241	249	9	HLA-A*02:01	241	15	15	1919.154771	12	6897.95	15	0.0057544	74	15	TRUE
1	VMPVSMAKT	708	716	9	HLA-A*02:01	708	15	15	2710.878168	14	7054.87	15	0.02123244	95	15	TRUE
1	NMYICGDST	721	729	9	HLA-A*02:01	721	15	15	3126.871254	15	4715.71	12	0.00122462	38	15	TRUE
1	VVIGIINNT	1110	1118	9	HLA-A*02:01	1110	15	15	3214.474612	15	3845.5	9.5	0.00110917	36	15	TRUE
1	FRSDTLYLT	47	55	9	HLA-A*02:01	47	15	15	3436.449768	15	9602.76	19	6.81E-05	3.9	15	TRUE
1	TLADAGFMK	809	817	9	HLA-A*02:01	809	15	15	3615.014137	15	14227.49	25	0.00011866	6.5	15	TRUE
1	FKNHTSPDV	1138	1146	9	HLA-A*02:01	1138	16	16	3820.41024	16	14692.27	26	0.0001406	7.6	16	TRUE
1	KNKDGFLYV	188	196	9	HLA-A*02:01	188	16	16	3909.399022	16	11074.97	21	0.00023324	13	16	TRUE
1	LQDVVNQNA	930	938	9	HLA-A*02:01	930	16	16	3918.411118	16	7661.76	16	0.00124738	39	16	TRUE
1	VQIDRLITG	973	981	9	HLA-A*02:01	973	16	16	3963.78418	16	8170.43	17	0.00019498	12	16	TRUE
1	DMIAAYTAA	850	858	9	HLA-A*02:01	850	17	17	1652.380267	9.7	8489.72	17	0.00166802	46	17	TRUE
1	DLCFSNVYA	376	384	9	HLA-A*02:01	376	17	17	1963.857628	12	8197.17	17	0.00032524	17	17	TRUE
1	DLLFNKVTL	802	810	9	HLA-A*02:01	802	17	17	2302.02481	13	8350.43	17	0.00082073	31	17	TRUE
1	LPDDFMGCV	412	420	9	HLA-A*02:01	412	17	17	3069.799422	14	15509.17	27	0.00033312	17	17	TRUE
1	DIWGTSAAA	243	251	9	HLA-A*02:01	243	17	17	3069.799422	14	8398.91	17	0.00531129	72	17	TRUE
1	HLREFVFKN	181	189	9	HLA-A*02:01	181	17	17	4218.033261	17	13623.84	24	0.00023627	13	17	TRUE
1	KLRPFERDI	447	455	9	HLA-A*02:01	447	17	17	4247.271271	17	9035.84	18	0.00022856	13	17	TRUE
1	GLTGTGVLT	531	539	9	HLA-A*02:01	531	17	17	4396.529586	17	6273.84	14	0.00168267	46	17	TRUE
1	MSLGADSSI	679	687	9	HLA-A*02:01	679	17	17	4457.691405	17	7625.78	16	0.00063212	26	17	TRUE
1	FGAGAALQI	870	878	9	HLA-A*02:01	870	17	17	4467.967444	17	10636.91	20	0.00011508	6.3	17	TRUE
1	LTQDLFLPF	54	62	9	HLA-A*02:01	54	17	17	4509.309038	17	8106.15	17	0.00055463	24	17	TRUE
1	AFNCTFEYI	156	164	9	HLA-A*02:01	156	17	17	4509.309038	17	8641.06	17	0.00306126	60	17	TRUE
1	ILTAFSPAQ	234	242	9	HLA-A*02:01	234	17	17	5703.087043	19	8494.96	17	0.00012814	7	17	TRUE
1	ALNCYWPLN	471	479	9	HLA-A*02:01	471	18	18	1596.28341	9.5	9365.42	18	0.00058506	25	18	TRUE
1	IYQTSNFRV	299	307	9	HLA-A*02:01	299	18	18	1671.513929	9.7	14767.02	26	0.00037342	18	18	TRUE
1	YFPREGVFV	1070	1078	9	HLA-A*02:01	1070	18	18	2270.439846	13	8723.82	18	0.00158453	45	18	TRUE
1	PIFKLPLGI	218	226	9	HLA-A*02:01	218	18	18	3640.072218	15	9026.57	18	0.0020408	50	18	TRUE
1	CVNFNFNGL	524	532	9	HLA-A*02:01	524	18	18	4074.834739	16	8779.02	18	0.00549541	73	18	TRUE
1	FVGYLKPTT	253	261	9	HLA-A*02:01	253	18	18	4376.329367	17	9139.98	18	0.0008147	31	18	TRUE
1	IFLLFLTLT	3	11	9	HLA-A*02:01	3	18	18	5001.612019	18	5801.83	14	0.00141547	42	18	TRUE
1	IAYSNNTIA	687	695	9	HLA-A*02:01	687	18	18	5141.738673	18	14156.55	25	0.00032825	17	18	TRUE
1	FAVSKPMGT	138	146	9	HLA-A*02:01	138	18	18	5165.47186	18	9917.21	19	0.00032599	17	18	TRUE
1	CSFGGVSVI	576	584	9	HLA-A*02:01	576	18	18	5322.43051	18	7045.19	15	0.00048843	22	18	TRUE
1	YECDIPIGA	646	654	9	HLA-A*02:01	646	19	19	2449.68363	13	9971.65	19	0.00042658	20	19	TRUE
1	CASYHTVSL	657	665	9	HLA-A*02:01	657	19	19	4700.131405	17	9770.02	19	0.00299364	59	19	TRUE
1	QIQESLTTT	915	923	9	HLA-A*02:01	915	19	19	5611.900987	19	8616.79	17	0.00644169	76	19	TRUE
1	SSMRGVYYP	35	43	9	HLA-A*02:01	35	19	19	5676.883738	19	17887.29	30	0.00012529	6.9	19	TRUE
1	IHADQLTPA	610	618	9	HLA-A*02:01	610	19	19	5689.970306	19	20551.63	35	0.0003376	17	19	TRUE
1	STFFSTFKC	358	366	9	HLA-A*02:01	358	19	19	5876.381732	19	7194.17	16	0.00061277	26	19	TRUE
1	VTLADAGFM	808	816	9	HLA-A*02:01	808	19	19	5971.865254	19	11049.95	21	0.00036728	18	19	TRUE
1	KAYFPREGV	1068	1076	9	HLA-A*02:01	1068	19	19	6282.174563	19	9501.68	19	0.00032226	17	19	TRUE
1	YICGDSTEC	723	731	9	HLA-A*02:01	723	20	20	2280.91972	13	10316.7	20	0.01092195	87	20	TRUE
1	YFVGYLKPT	252	260	9	HLA-A*02:01	252	20	20	4018.925911	16	10835.89	20	0.0017374	47	20	TRUE
1	IFRSDTLYL	46	54	9	HLA-A*02:01	46	20	20	5716.23402	19	14269.57	25	0.00040448	20	20	TRUE
1	KCSVKSFEI	287	295	9	HLA-A*02:01	287	20	20	6369.568317	20	10279.26	20	0.00353509	63	20	TRUE
1	ALSGIAAEQ	748	756	9	HLA-A*02:01	748	20	20	6428.505207	20	10705.48	20	0.00022269	13	20	TRUE
1	KSFEIDKGI	291	299	9	HLA-A*02:01	291	20	20	6487.987433	20	7219.35	16	0.00228981	53	20	TRUE
1	TILLCCMTS	1213	1221	9	HLA-A*02:01	1213	20	20	6793.756861	20	4600.05	12	0.00063241	26	20	TRUE
1	SQKSIVAYT	670	678	9	HLA-A*02:01	670	20	20	6825.115418	20	9150.26	18	0.00057412	24	20	TRUE
1	AATKMSECV	1007	1015	9	HLA-A*02:01	1007	20	20	6840.848933	20	13572.35	24	0.0002939	15	20	TRUE
1	LLNAPATVC	503	511	9	HLA-A*02:01	503	20	20	6888.267433	20	13968.64	25	0.00038133	19	20	TRUE
1	LNDLCFSNV	374	382	9	HLA-A*02:01	374	20	20	7016.329886	20	13337.68	24	9.77E-05	5.5	20	TRUE
1	SVIIINNST	113	121	9	HLA-A*02:01	113	20	20	7064.964761	20	10133.05	19	0.00140281	42	20	TRUE
1	FNFNGLTGT	527	535	9	HLA-A*02:01	527	20	20	7064.964761	20	11889.74	22	0.00012555	6.9	20	TRUE
1	QTYVTQQLI	987	995	9	HLA-A*02:01	987	20	20	9618.558706	23	9449.7	19	0.00041976	20	20	TRUE
1	GCLIGAEHV	634	642	9	HLA-A*02:01	634	20	20	11326.87219	25	10325.63	20	0.00021847	13	20	TRUE
1	KLSTDLIKN	514	522	9	HLA-A*02:01	514	21	21	2938.393807	14	16471.51	28	0.00045415	21	21	TRUE
1	RAAEIRASA	996	1004	9	HLA-A*02:01	996	21	21	4921.641774	18	11342.37	21	0.00383884	65	21	TRUE
1	SVVNIQKEI	1157	1165	9	HLA-A*02:01	1157	21	21	5297.976163	18	11039.07	21	0.00100231	35	21	TRUE
1	VDLGDISGI	1146	1154	9	HLA-A*02:01	1146	21	21	6167.511955	19	23234.21	40	0.00045331	21	21	TRUE
1	ISPCSFGGV	573	581	9	HLA-A*02:01	573	21	21	6856.618719	20	11554.05	21	0.00334811	62	21	TRUE
1	STECANLLL	728	736	9	HLA-A*02:01	728	21	21	7262.89892	21	7497.25	16	0.00431519	67	21	TRUE
1	AQKFNGLTV	834	842	9	HLA-A*02:01	834	21	21	7296.422938	21	10368.17	20	0.00187932	49	21	TRUE
1	AYSNNTIAI	688	696	9	HLA-A*02:01	688	21	21	7363.935909	21	20948.33	36	9.38E-05	5.4	21	TRUE
1	GVSVITPGT	580	588	9	HLA-A*02:01	580	21	21	7397.926292	21	11201.74	21	0.00053456	23	21	TRUE
1	KGYQPIDVV	198	206	9	HLA-A*02:01	198	21	21	7728.76318	21	15107.21	26	0.0001205	6.6	21	TRUE
1	NLCPFGEVF	321	329	9	HLA-A*02:01	321	21	21	18668.52475	29	11598.13	21	0.00038815	19	21	TRUE
1	CVLGQSKRV	1014	1022	9	HLA-A*02:01	1014	22	22	2898.077532	14	12186.56	22	0.00131522	40	22	TRUE
1	RVVPSGDVV	306	314	9	HLA-A*02:01	306	22	22	3404.944207	15	11982.21	22	0.00114288	37	22	TRUE
1	AATEKSNVV	90	98	9	HLA-A*02:01	90	22	22	3573.633412	15	11753.9	22	0.00128292	40	22	TRUE
1	LQSLQTYVT	983	991	9	HLA-A*02:01	983	22	22	4978.6317	18	12769.85	23	0.00046881	22	22	TRUE
1	SVDCNMYIC	717	725	9	HLA-A*02:01	717	22	22	5036.281538	18	11729.76	22	0.00260196	56	22	TRUE
1	SMAKTSVDC	712	720	9	HLA-A*02:01	712	22	22	5165.47186	18	12272.58	22	0.011893235	88	22	TRUE
1	WYVWLGFIA	1196	1204	9	HLA-A*02:01	1196	22	22	6793.756861	20	12220.1	22	0.00553605	73	22	TRUE
1	SVLYNSTFF	353	361	9	HLA-A*02:01	353	22	22	7146.7732	20	11760.01	22	0.00203236	50	22	TRUE
1	GNCDVVIGI	1106	1114	9	HLA-A*02:01	1106	22	22	7963.610307	22	12675.55	23	0.00012439	6.8	22	TRUE
1	CLGDINARD	822	830	9	HLA-A*02:01	822	22	22	8111.664876	22	22643.78	39	0.0001982	12	22	TRUE
1	NITNFRAIL	227	235	9	HLA-A*02:01	227	22	22	8111.664876	22	10801.48	20	0.0074817	79	22	TRUE
1	SQIQESLTT	914	922	9	HLA-A*02:01	914	22	22	8955.915762	22	10249.72	20	0.00421697	67	22	TRUE
1	IEDLLFNKV	800	808	9	HLA-A*02:01	800	22	22	8976.56128	22	14990.3	26	0.00033884	17	22	TRUE
1	FELCDNPFF	130	138	9	HLA-A*02:01	130	22	22	9017.995204	22	19881.25	34	3.52E-05	2	22	TRUE
1	PLLTDDMIA	845	853	9	HLA-A*02:01	845	23	23	2606.813732	14	12869.44	23	0.00237028	54	23	TRUE
1	LYLTQDLFL	52	60	9	HLA-A*02:01	52	23	23	3444.371603	15	12607.43	23	0.00361576	63	23	TRUE
1	YHLMSFPQA	1029	1037	9	HLA-A*02:01	1029	23	23	4614.344339	17	12764.45	23	0.001692	46	23	TRUE
1	FQPFQQFGR	545	553	9	HLA-A*02:01	545	23	23	5534.902819	19	21801.37	37	0.0005157	23	23	TRUE
1	SVLNDILSR	957	965	9	HLA-A*02:01	957	23	23	7675.558746	21	19677.1	33	0.00053753	23	23	TRUE
1	MLKYDENGT	263	271	9	HLA-A*02:01	263	23	23	7764.437528	21	14673.52	26	0.0005358	23	23	TRUE

seq #	peptide	start	end	peptide length	allele	peptide index	median binding	consensus perc	smm IC50	smm percentil	ann IC50	ann percentile	comblib_sidne	comblib_sidne	legacy consens	percentiles equal
1	LIRAAEIRA	994	1002	9	HLA-A*02:01	994	23	23	9249.32415	23	15695.36	27	0.0002138	12	23	TRUE
1	VTILLCCMT	1212	1220	9	HLA-A*02:01	1212	23	23	9313.437404	23	6570.5	15	0.00298538	59	23	TRUE
1	GIGVTQNVL	890	898	9	HLA-A*02:01	890	23	23	9356.42628	23	12465.29	23	0.00805378	81	23	TRUE
1	TFVSGNCDV	1102	1110	9	HLA-A*02:01	1102	23	23	9421.281926	23	23803.95	41	0.00017454	9.3	23	TRUE
1	QIPFAMQMA	877	885	9	HLA-A*02:01	877	23	23	9443.000224	23	10423.73	20	0.00498884	71	23	TRUE
1	SISITTEVM	701	709	9	HLA-A*02:01	701	23	23	9596.436635	23	9359.54	18	0.00198609	50	23	TRUE
1	FSLDVSEKS	168	176	9	HLA-A*02:01	168	23	23	9979.52798	23	15627.92	27	0.00020549	12	23	TRUE
1	TQDLFLPFY	55	63	9	HLA-A*02:01	55	23	23	10211.98104	24	12502.84	23	0.00010568	5.9	23	TRUE
1	RLITGRLQS	977	985	9	HLA-A*02:01	977	24	24	6096.91303	19	13798.74	24	0.00172982	47	24	TRUE
1	VVNQNAQAL	933	941	9	HLA-A*02:01	933	24	24	6267.725963	19	13609.4	24	0.00083176	31	24	TRUE
1	YTMSLGADS	677	685	9	HLA-A*02:01	677	24	24	7535.464011	21	13160.35	24	0.00087902	32	24	TRUE
1	AGWTFGAGA	866	874	9	HLA-A*02:01	866	24	24	8552.833153	22	13569.85	24	0.00693426	78	24	TRUE
1	STGNNVFQT	623	631	9	HLA-A*02:01	623	24	24	8752.05423	22	13116.85	24	0.0009528	34	24	TRUE
1	NGIGVTQNV	889	897	9	HLA-A*02:01	889	24	24	10165.0613	24	15660.25	27	5.06E-05	2.9	24	TRUE
1	ITTDNTFVS	1097	1105	9	HLA-A*02:01	1097	24	24	10282.76712	24	20447.83	35	0.00032659	17	24	TRUE
1	VRFPNITNL	314	322	9	HLA-A*02:01	314	24	24	10570.85218	24	16857.71	29	0.0005577	24	24	TRUE
1	EYISDAFSL	162	170	9	HLA-A*02:01	162	24	24	10693.25688	24	24469.58	42	0.00022761	13	24	TRUE
1	SGFNTLKPI	211	219	9	HLA-A*02:01	211	24	24	13216.3034	26	13799.49	24	0.00020417	12	24	TRUE
1	QIANQFNKA	904	912	9	HLA-A*02:01	904	25	25	5637.804345	19	14253.52	25	0.00334195	62	25	TRUE
1	FLTLTSGSD	7	15	9	HLA-A*02:01	7	25	25	11405.38632	25	21544.6	37	0.00017262	9.2	25	TRUE
1	CLIGAEHVD	635	643	9	HLA-A*02:01	635	25	25	11510.91906	25	28318.62	51	0.00012165	6.7	25	TRUE
1	FSPDGKPCT	460	468	9	HLA-A*02:01	460	25	25	11998.03156	25	21197.76	36	0.00032494	17	25	TRUE
1	FCTQLNRAL	741	749	9	HLA-A*02:01	741	25	25	19820.28979	30	13758.94	24	0.0006115	25	25	TRUE
1	SMRGVYYPD	36	44	9	HLA-A*02:01	36	26	26	4743.621186	17	18303.31	31	0.0006139	26	26	TRUE
1	VVRGWVFGS	97	105	9	HLA-A*02:01	97	26	26	5769.125678	19	14834.27	26	0.00142233	42	26	TRUE
1	YADSFVVKG	383	391	9	HLA-A*02:01	383	26	26	7981.968324	22	15190.79	26	0.0007766	30	26	TRUE
1	CPFGEVFNA	323	331	9	HLA-A*02:01	323	26	26	8000.368662	22	14616.32	26	0.00076666	30	26	TRUE
1	NASSEVAVL	589	597	9	HLA-A*02:01	589	26	26	9443.000224	23	15001.98	26	0.00123367	39	26	TRUE
1	FGGFNFSQI	779	787	9	HLA-A*02:01	779	26	26	12621.47176	26	16136.18	28	0.00062087	26	26	TRUE
1	IPTNFSISI	696	704	9	HLA-A*02:01	696	26	26	12679.72983	26	16964.38	29	0.00049728	22	26	TRUE
1	LTDDMIAAY	847	855	9	HLA-A*02:01	847	26	26	12738.2568	26	18571.42	32	0.00012764	7	26	TRUE
1	SNCVADYSV	346	354	9	HLA-A*02:01	346	26	26	12767.62152	26	18476.62	31	0.00029771	16	26	TRUE
1	EQDRNTREV	755	763	9	HLA-A*02:01	755	26	26	12915.46361	26	23909.52	41	0.00018323	9.8	26	TRUE
1	GIINNTVYD	1113	1121	9	HLA-A*02:01	1113	26	26	12945.23683	26	27614.52	49	0.00018668	10	26	TRUE
1	PWYVWLGFI	1195	1203	9	HLA-A*02:01	1195	26	26	13004.98932	26	15025.04	26	0.00044096	21	26	TRUE
1	CSQNPLAEL	278	286	9	HLA-A*02:01	278	26	26	13246.77013	26	13747.62	24	0.00185695	48	26	TRUE
1	KQIANQFNK	903	911	9	HLA-A*02:01	903	26	26	13277.30709	26	27104.49	48	0.00033752	17	26	TRUE
1	FCGKGYHLM	1024	1032	9	HLA-A*02:01	1024	26	26	13586.5753	26	12753.41	23	0.00216471	52	26	TRUE
1	GGFNFSQIL	780	788	9	HLA-A*02:01	780	26	26	13743.90042	26	14001.63	25	0.00095499	34	26	TRUE
1	FNGLTGTGV	529	537	9	HLA-A*02:01	529	26	26	13999.41861	26	16061.8	28	0.00019311	12	26	TRUE
1	SLLRSTSQK	664	672	9	HLA-A*02:01	664	26	26	14161.52424	26	27932.15	50	0.00015787	8.4	26	TRUE
1	NAFNCTFEY	155	163	9	HLA-A*02:01	155	26	26	14194.16992	26	15839.69	27	3.38E-05	2	26	TRUE
1	NLLLQYGSF	733	741	9	HLA-A*02:01	733	26	26	14558.27772	27	12869.44	23	0.00062951	26	26	TRUE
1	IAGLIAIVM	1203	1211	9	HLA-A*02:01	1203	26	26	19503.38527	30	9186.67	18	0.00062402	26	26	TRUE
1	KWPWYVWLG	1193	1201	9	HLA-A*02:01	1193	27	27	4416.823044	17	15755.43	27	0.0016734	46	27	TRUE
1	SLQTYVTQQ	985	993	9	HLA-A*02:01	985	27	27	6167.511955	19	15487.54	27	0.00128145	40	27	TRUE
1	STAIHADQL	607	615	9	HLA-A*02:01	607	27	27	6888.267433	20	15277.66	27	0.00503501	71	27	TRUE
1	EIDKGIYQT	294	302	9	HLA-A*02:01	294	27	27	11671.0519	25	20874.11	35	0.00068077	27	27	TRUE
1	ITTEVMPVS	704	712	9	HLA-A*02:01	704	27	27	13338.59235	26	15266.09	27	0.00167494	46	27	TRUE
1	VTQNVLYEN	893	901	9	HLA-A*02:01	893	27	27	14591.83802	27	23365.3	40	0.00041419	20	27	TRUE
1	DLFLPFYSN	57	65	9	HLA-A*02:01	57	27	27	15000.6473	27	15671.27	27	7.93E-05	4.6	27	TRUE
1	RNFTTAPAI	1055	1063	9	HLA-A*02:01	1055	27	27	15174.34661	27	20541.18	35	0.00042638	20	27	TRUE
1	GADSSIAYS	682	690	9	HLA-A*02:01	682	27	27	15209.32709	27	17677.38	30	0.00030283	16	27	TRUE
1	ISGINASVV	1151	1159	9	HLA-A*02:01	1151	27	27	15350.05727	27	16723.28	29	0.00043934	21	27	TRUE
1	FPSVYAWER	334	342	9	HLA-A*02:01	334	27	27	15635.43614	27	25390.08	44	0.00029854	16	27	TRUE
1	TLVKQLSSN	943	951	9	HLA-A*02:01	943	27	27	15707.60603	27	28337	51	0.00051665	23	27	TRUE
1	IINNTVYDP	1114	1122	9	HLA-A*02:01	1114	27	27	15889.49154	27	21192.48	36	0.0006311	26	27	TRUE
1	AQVKQMYKT	765	773	9	HLA-A*02:01	765	28	28	5835.92901	19	16403.57	28	0.0031989	61	28	TRUE
1	NIQKEIDRL	1160	1168	9	HLA-A*02:01	1160	28	28	6593.408748	20	15911.67	28	0.00432514	67	28	TRUE
1	STSQKSIVA	668	676	9	HLA-A*02:01	668	28	28	8205.593529	22	16136	28	0.00246604	55	28	TRUE
1	VTQQLIRAA	990	998	9	HLA-A*02:01	990	28	28	10141.68226	23	16105.66	28	0.00378443	64	28	TRUE
1	EILDISPCS	569	577	9	HLA-A*02:01	569	28	28	16869.8026	28	24271.04	42	0.00064565	26	28	TRUE
1	INHTFGNPV	72	80	9	HLA-A*02:01	72	28	28	17025.89693	28	16426.31	28	0.00036627	18	28	TRUE
1	VVLSFELLN	497	505	9	HLA-A*02:01	497	28	28	17262.75075	28	19418.86	33	0.00024957	14	28	TRUE
1	SNVYADSFV	380	388	9	HLA-A*02:01	380	28	28	17302.5455	28	24017.38	41	0.00027784	15	28	TRUE
1	YSNVTGFHT	63	71	9	HLA-A*02:01	63	28	28	17302.5455	28	16241.45	28	0.01643615	92	28	TRUE
1	NSTNVVIRA	119	127	9	HLA-A*02:01	119	28	28	21091.623	30	16593.15	28	0.00035793	18	28	TRUE
1	TMIFDNAFN	150	158	9	HLA-A*02:01	150	29	29	3598.40466	15	18736.71	32	0.00074679	29	29	TRUE
1	YYPDEIFRS	41	49	9	HLA-A*02:01	41	29	29	6054.942175	19	24746.75	43	0.00073824	29	29	TRUE
1	DQLTPAWRI	613	621	9	HLA-A*02:01	613	29	29	6654.416808	20	16621.54	29	0.00100508	35	29	TRUE
1	VLPPLLTDD	842	850	9	HLA-A*02:01	842	29	29	10095.08546	23	23812.18	41	0.00073978	29	29	TRUE
1	QKFNGLTVL	835	843	9	HLA-A*02:01	835	29	29	10354.04385	24	16919.84	29	0.00106905	36	29	TRUE
1	YIKWPWYVW	1191	1199	9	HLA-A*02:01	1191	29	29	11942.90561	25	17003.34	29	0.0046957	69	29	TRUE
1	LTPAWRIYS	615	623	9	HLA-A*02:01	615	29	29	15420.90991	27	19751.97	33	0.00074817	29	29	TRUE
1	ITNFRAILT	228	236	9	HLA-A*02:01	228	29	29	18285.63304	29	15172.4	26	0.00103992	35	29	TRUE
1	LSFELLNAP	499	507	9	HLA-A*02:01	499	29	29	18754.69475	29	15054.17	26	0.00156279	44	29	TRUE
1	LKYDENGTI	264	272	9	HLA-A*02:01	264	29	29	18754.69475	29	28355.41	51	0.00027797	15	29	TRUE
1	FELLNAPAT	501	509	9	HLA-A*02:01	501	29	29	19015.59854	29	22796.44	39	2.92E-05	1.7	29	TRUE
1	TTFDDVQAP	20	28	9	HLA-A*02:01	20	29	29	19103.37056	29	25888.57	45	0.00033504	17	29	TRUE
1	SFELLNAPA	500	508	9	HLA-A*02:01	500	29	29	26128.23058	33	17238.6	29	0.00060104	25	29	TRUE
1	YKGYQPIDV	197	205	9	HLA-A*02:01	197	30	30	5689.970306	19	17741.19	30	0.00077625	30	30	TRUE
1	FVFKNKDGF	185	193	9	HLA-A*02:01	185	30	30	11564.05104	25	21125.18	36	0.00077625	30	30	TRUE
1	KTSEILDIS	566	574	9	HLA-A*02:01	566	30	30	14692.98381	27	17276.51	30	0.00855067	82	30	TRUE
1	SFIEDLLFN	798	806	9	HLA-A*02:01	798	30	30	15420.90991	27	20919.11	36	0.00080002	30	30	TRUE
1	RASANLAAT	1001	1009	9	HLA-A*02:01	1001	30	30	16147.675	28	17348.62	30	0.0022346	52	30	TRUE
1	ISSVLNDIL	955	963	9	HLA-A*02:01	955	30	30	19369.12491	30	16683.15	29	0.010685627	86	30	TRUE
1	IANQFNKAI	905	913	9	HLA-A*02:01	905	30	30	19458.52873	30	17632.87	30	0.00293224	59	30	TRUE
1	ICAQKFNGL	832	840	9	HLA-A*02:01	832	30	30	19957.67748	30	15524.28	27	0.00256094	56	30	TRUE
1	EVAVLYQDV	593	601	9	HLA-A*02:01	593	30	30	20096.01749	30	18618.09	32	0.00080724	30	30	TRUE
1	VSGNCDVVI	1104	1112	9	HLA-A*02:01	1104	30	30	20142.34359	30	14932.5	26	0.00155167	44	30	TRUE
1	KAISQIQES	911	919	9	HLA-A*02:01	911	30	30	20235.31643	30	26201.66	46	0.00016226	8.6	30	TRUE
1	NITNLCPFG	318	326	9	HLA-A*02:01	318	30	30	20611.51905	30	20657.74	35	0.00030061	16	30	TRUE
1	PFKDGIYFA	82	90	9	HLA-A*02:01	82	30	30	20994.7158	30	32291.24	62	0.00013083	7.2	30	TRUE
1	RAILTAFSP	232	240	9	HLA-A*02:01	232	30	30	34049.44207	36	17324.99	30	0.00052638	23	30	TRUE
1	FRAILTAFS	231	239	9	HLA-A*02:01	231	30	30	37506.79891	37	17540.01	30	7.42E-05	4.3	30	TRUE
1	CVADYSVLY	348	356	9	HLA-A*02:01	348	31	31	7130.336075	20	18163.24	31	0.00287078	58	31	TRUE
1	SVITPGTNA	582	590	9	HLA-A*02:01	582	31	31	10118.35704	23	18160.3	31	0.01914256	94	31	TRUE
1	KYFGGFNFS	777	785	9	HLA-A*02:01	777	31	31	16869.80257	28	21087.51	36	0.00082073	31	31	TRUE
1	TNASSEVAV	588	596	9	HLA-A*02:01	588	31	31	21782.61422	31	25035.73	43	8.12E-05	4.7	31	TRUE
1	GYLKPTTFM	255	263	9	HLA-A*02:01	255	31	31	21782.61422	31	26974	48	0.00057306	24	31	TRUE
1	VFLHVTYVP	1043	1051	9	HLA-A*02:01	1043	31	31	21832.82833	31	21929.13	37	0.00055081	24	31	TRUE
1	ADYSVLYNS	350	358	9	HLA-A*02:01	350	31	31	22289.99649	31	21505.25	37	0.00011231	6.2	31	TRUE
1	YSVLYNSTF	352	360	9	HLA-A*02:01	352	31	31	22444.50336	31	15759.18	27	0.00133906	41	31	TRUE
1	LKPIFKLPL	216	224	9	HLA-A*02:01	216	31	31	22652.17979	31	15981.2	28	0.00416869	67	31	TRUE
1	GIYQTSNFR	298	306	9	HLA-A*02:01	298	31	31	22756.7375	31	22662.42	39	0.00069566	28	31	TRUE
1	CVLAWNTRN	419	427	9	HLA-A*02:01	419	31	31	23179.81684	31	33208.94	65	0.0006269	26	31	TRUE
1	KISNCVADY	344	352	9	HLA-A*02:01	344	31	31	23502.28021	31	20411.79	35	0.00072611	29	31	TRUE
1	AAYFVGYLK	250	258	9	HLA-A*02:01	250	31	31	47436.21186	39	15222.23	26	0.00084431	31	31	TRUE
1	LTLTSGSDL	8	16	9	HLA-A*02:01	8	32	32	9979.52798	23	18833.46	32	0.00887156	83	32	TRUE
1	SQERNFTTA	1052	1060	9	HLA-A*02:01	1052	32	32	15420.90991	27	18978.7	32	0.015240528	91	32	TRUE
1	SPAQDIWGT	239	247	9	HLA-A*02:01	239	32	32	18034.74475	29	26342.09	46	0.00085428	32	32	TRUE
1	TDLIKNQCV	517	525	9	HLA-A*02:01	517	32	32	23884.16155	32	30660.31	57	0.00017155	9.2	32	TRUE
1	TLTSGSDLD	9	17	9	HLA-A*02:01	9	32	32	24553.30734	32	34918.33	70	0.00062101	26	32	TRUE
1	ATKFPSVYA	331	339	9	HLA-A*02:01	331	32	32	24609.90856	32	17684.07	30	0.014996848	91	32	TRUE
1	FPREGVFVF	1071	1079	9	HLA-A*02:01	1071	32	32	24837.62124	32	19145.96	32	0.00020119	12	32	TRUE
1	DLDRCTTFD	15	23	9	HLA-A*02:01	15	32	32	24837.62124	32	32877.15	64	3.31E-05	1.9	32	TRUE
1	FKCYGVSAT	364	372	9	HLA-A*02:01	364	32	32	25533.47944	32	25388.98	44	0.00046026	21	32	TRUE
1	FGNPVIPFK	76	84	9	HLA-A*02:01	76	32	32	25829.1432	32	23501.19	40	1.58E-05	1	32	TRUE
1	PLGINITNF	223	231	9	HLA-A*02:01	223	32	32	52373.30732	40	18949.15	32	0.00080316	30	32	TRUE
1	FNGLTVLPP	837	845	9	HLA-A*02:01	837	32	32	116174.2828	50	18547.33	32	8.97E-05	5.1	32	TRUE
1	FKHLREFVF	179	187	9	HLA-A*02:01	179	32	32	139350.9712	52	18887.33	32	9.42E-05	5.4	32	TRUE
1	LMSFPQAAP	1031	1039	9	HLA-A*02:01	1031	33	33	9752.366186	23	19502.44	33	0.00897635	83	33	TRUE

seq #	peptide	start	end	peptide length	allele	peptide index	median binding	consensus perc	smm IC50	smm percentile	ann IC50	ann percentile	comblib_sidney	comblib_sidney	legacy consens	percentiles equal	
1	VSATKLNDL	369	377	9	HLA-A*02:01	369	33	33	15279.53016	27	19298.01	33	0.00705992	78	33	TRUE	
1	WVFGSTMNN	101	109	9	HLA-A*02:01	101	33	33	17705.57356	29	22782.39	39	0.00091664	33	33	TRUE	
1	KSNVVRGWV	94	102	9	HLA-A*02:01	94	33	33	18285.63304	29	19318.48	33	0.0032419	61	33	TRUE	
1	NCTDVSTAI	602	610	9	HLA-A*02:01	602	33	33	26369.99212	33	17630.39	30	0.00399393	66	33	TRUE	
1	GTATAGWTF	862	870	9	HLA-A*02:01	862	33	33	27108.78168	33	19075.05	32	0.00548277	73	33	TRUE	
1	FNFSQILPD	782	790	9	HLA-A*02:01	782	33	33	27108.78168	33	23189.76	40	1.18E-05	0.8	33	TRUE	
1	HTINHTFGN	70	78	9	HLA-A*02:01	70	33	33	27233.91013	33	32735.88	63	0.00022615	13	33	TRUE	
1	QDIWGTSAA	242	250	9	HLA-A*02:01	242	33	33	27296.69078	33	33298.16	65	0.00042599	20	33	TRUE	
1	AYRFNGIGV	885	893	9	HLA-A*02:01	885	33	33	27422.68658	33	23472.23	40	0.00046047	21	33	TRUE	
1	LQYGSFCTQ	736	744	9	HLA-A*02:01	736	33	33	27740.22632	33	16475.79	28	0.00205921	51	33	TRUE	
1	FEYISDAFS	161	169	9	HLA-A*02:01	161	33	33	44989.37915	39	19569.46	33	0.00018281	9.7	33	TRUE	
1	SCCSCLKGA	1221	1229	9	HLA-A*02:01	1221	33	33	59171.1486	42	19298.42	33	0.00070372	28	33	TRUE	
1	TTSTALGKL	922	930	9	HLA-A*02:01	922	34	34	13743.90042	26	19868.36	34	0.00995405	85	34	TRUE	
1	KQYGECLGD	817	825	9	HLA-A*02:01	817	34	34	20754.39127	30	24814.85	43	0.00099334	34	34	TRUE	
1	TNLCPFGEV	320	328	9	HLA-A*02:01	320	34	34	21732.5156	31	23102.36	39	0.00095455	34	34	TRUE	
1	YFGGFNFSQ	778	786	9	HLA-A*02:01	778	34	34	23502.28021	31	19948.27	34	0.00133537	41	34	TRUE	
1	SQSVIIINN	111	119	9	HLA-A*02:01	111	34	34	26491.71047	33	19817.48	34	0.00097096	34	34	TRUE	
1	TPAWRIYST	616	624	9	HLA-A*02:01	616	34	34	28255.9556	34	28460.22	51	0.00026767	14	34	TRUE	
1	VDCSQNPLA	276	284	9	HLA-A*02:01	276	34	34	28517.40477	34	36849.44	77	0.00029268	15	34	TRUE	
1	FGRDVSDFT	551	559	9	HLA-A*02:01	551	34	34	28649.03513	34	25822.56	45	0.00046452	21	34	TRUE	
1	NVFQTQAGC	627	635	9	HLA-A*02:01	627	34	34	29114.54451	34	18867.94	32	0.00144644	42	34	TRUE	
1	ESLIDLQEL	1177	1185	9	HLA-A*02:01	1177	34	34	29316.35689	34	18638.46	32	0.00111122	36	34	TRUE	
1	TAALVSGTA	856	864	9	HLA-A*02:01	856	34	34	29451.67504	34	19532.65	33	0.00622587	76	34	TRUE	
1	FSNVYADSF	379	387	9	HLA-A*02:01	379	34	34	29519.56816	34	19687.32	33	0.00097006	34	34	TRUE	
1	DISPCSFGG	572	580	9	HLA-A*02:01	572	34	34	30768.76041	34	29948.49	55	0.00090615	33	34	TRUE	
1	TAFSPAQDI	236	244	9	HLA-A*02:01	236	34	34	36568.74028	36	20332.01	34	0.00018374	9.8	34	TRUE	
1	DFMGCVLAW	415	423	9	HLA-A*02:01	415	34	34	46037.31641	39	20000.57	34	0.00022019	13	34	TRUE	
1	YISDAFSLD	163	171	9	HLA-A*02:01	163	35	35	3119.67965	15	20839.81	35	0.00131856	40	35	TRUE	
1	GYQPYRVVV	490	498	9	HLA-A*02:01	490	35	35	6984.092778	20	24362.86	42	0.00105487	35	35	TRUE	
1	VAKNLNESL	1171	1179	9	HLA-A*02:01	1171	35	35	28451.81656	34	22543.31	38	0.00104761	35	35	TRUE	
1	LTVLPPLLT	840	848	9	HLA-A*02:01	840	35	35	31053.46024	35	15684.84	27	0.00680769	78	35	TRUE	
1	TNFRAILTA	229	237	9	HLA-A*02:01	229	35	35	31340.79437	35	16482.03	28	0.0012297	39	35	TRUE	
1	GIYFAATEK	86	94	9	HLA-A*02:01	86	35	35	31485.4569	35	24518.35	42	0.00061987	26	35	TRUE	
1	HTMIFDNAF	149	157	9	HLA-A*02:01	149	35	35	31630.78717	35	13940.56	25	0.00241657	54	35	TRUE	
1	IPIGAGICA	650	658	9	HLA-A*02:01	650	35	35	31776.78825	35	24485.46	42	0.00094755	34	35	TRUE	
1	LLRSTSQKS	665	673	9	HLA-A*02:01	665	35	35	31850.04131	35	26617.12	47	0.0004256	20	35	TRUE	
1	YNYKYRYLR	436	444	9	HLA-A*02:01	436	35	35	32442.17779	35	32407.8	62	0.00089578	32	35	TRUE	
1	DTLYLTQDL	50	58	9	HLA-A*02:01	50	35	35	32742.36157	35	25831.77	45	0.00060842	25	35	TRUE	
1	VRDPKTSEI	562	570	9	HLA-A*02:01	562	35	35	33274.38214	35	30979.1	58	0.00014335	7.8	35	TRUE	
1	GVFVFNGTS	1075	1083	9	HLA-A*02:01	1075	35	35	33582.26621	35	17587.14	30	0.00248313	55	35	TRUE	
1	TNFSISITT	698	706	9	HLA-A*02:01	698	35	35	53346.9998	41	20403.19	35	0.00031246	16	35	TRUE	
1	CANLLLQYG	731	739	9	HLA-A*02:01	731	35	35	98199.6636	48	20566.76	35	0.00101906	35	35	TRUE	
1	LYNSTFFST	355	363	9	HLA-A*02:01	355	36	36	10141.68226	23	21282.56	36	0.00132801	41	36	TRUE	
1	QMYKTPTLK	769	777	9	HLA-A*02:01	769	36	36	10546.5399	24	21182.63	36	0.00256626	56	36	TRUE	
1	TMSLGADSS	678	686	9	HLA-A*02:01	678	36	36	12053.41196	25	21103.48	36	0.00233346	53	36	TRUE	
1	SNVTGFHTI	64	72	9	HLA-A*02:01	64	36	36	16986.73845	28	21127.24	36	0.00765244	80	36	TRUE	
1	IVAYTMSLG	674	682	9	HLA-A*02:01	674	36	36	19503.38527	30	21038.28	36	0.00392645	65	36	TRUE	
1	SAAAYFVGY	248	256	9	HLA-A*02:01	248	36	36	22861.77783	31	20956.49	36	0.00211934	51	36	TRUE	
1	INNTVYDPL	1115	1123	9	HLA-A*02:01	1115	36	36	24780.49629	32	21442.75	36	0.00607855	75	36	TRUE	
1	VITPGTNAS	583	591	9	HLA-A*02:01	583	36	36	25357.7082	32	25457.2	44	0.00107895	36	36	TRUE	
1	FNGTSWFIT	1079	1087	9	HLA-A*02:01	1079	36	36	32218.84741	35	21236.78	36	0.00337909	62	36	TRUE	
1	QLIRAAEIR	993	1001	9	HLA-A*02:01	993	36	36	33892.99909	36	25251.46	44	0.00031434	16	36	TRUE	
1	IGYQPYRVV	489	497	9	HLA-A*02:01	489	36	36	34364.49768	36	22954.84	39	0.00069024	28	36	TRUE	
1	FDNAFNCTF	153	161	9	HLA-A*02:01	153	36	36	35164.94966	36	21212.68	36	0.00019249	12	36	TRUE	
1	RSTSQKSIV	667	675	9	HLA-A*02:01	667	36	36	36150.14137	36	33182.72	65	0.0009264	33	36	TRUE	
1	TKFPSVYAW	332	340	9	HLA-A*02:01	332	36	36	36400.72218	36	26960.59	47	0.0002232	13	36	TRUE	
1	IRASANLAA	1000	1008	9	HLA-A*02:01	1000	36	36	36568.74028	36	27393.42	48	0.00042501	20	36	TRUE	
1	ALQIPFAMQ	875	883	9	HLA-A*02:01	875	36	36	36992.18633	36	12721.72	23	0.00203095	50	36	TRUE	
1	FGGVSVITP	578	586	9	HLA-A*02:01	578	36	36	102827.6712	49	21023.71	36	0.00026922	14	36	TRUE	
1	MTSCCSCLK	1219	1227	9	HLA-A*02:01	1219	37	37	25357.7082	32	21595.01	37	0.00589251	75	37	TRUE	
1	NTIAIPTNF	692	700	9	HLA-A*02:01	692	37	37	37334.47082	37	21445.29	36	0.00209411	51	37	TRUE	
1	ICGDSTECA	724	732	9	HLA-A*02:01	724	37	37	37766.78362	37	23965.71	41	0.0007251	29	37	TRUE	
1	RGWVFGSTM	99	107	9	HLA-A*02:01	99	37	37	37941.10717	37	14422.78	25	0.00503501	71	37	TRUE	
1	MGCVLAWNT	417	425	9	HLA-A*02:01	417	37	37	38028.57045	37	17484.31	30	0.00279254	57	37	TRUE	
1	CYWPLNDYG	474	482	9	HLA-A*02:01	474	37	37	38557.60057	37	28640	52	0.00069534	28	37	TRUE	
1	LFLTLTSGS	6	14	9	HLA-A*02:01	6	37	37	38557.60057	37	30240.25	56	0.00105657	35	37	TRUE	
1	SLDVSEKSG	169	177	9	HLA-A*02:01	169	37	37	38646.48502	37	29514.23	54	0.00051404	23	37	TRUE	
1	TIAIPTNFS	693	701	9	HLA-A*02:01	693	37	37	38914.36965	37	25042.49	43	0.00066222	27	37	TRUE	
1	YRFNGIGVT	886	894	9	HLA-A*02:01	886	37	37	39546.67729	37	25356.32	44	0.00076454	30	37	TRUE	
1	DDMIAAYTA	849	857	9	HLA-A*02:01	849	37	37	39820.80176	37	27516.69	49	0.00017807	9.4	37	TRUE	
1	FTDSVRDPK	558	566	9	HLA-A*02:01	558	37	37	39912.59819	37	27644.12	49	9.13E-05	5.2	37	TRUE	
1	PLQPELDSF	1122	1130	9	HLA-A*02:01	1122	37	37	39912.59819	37	25026.51	43	0.00043934	21	37	TRUE	
1	STGNYNYKY	432	440	9	HLA-A*02:01	432	37	37	40004.60623	37	32640.03	63	0.0006792	27	37	TRUE	
1	IPFKDGIYF	81	89	9	HLA-A*02:01	81	37	37	76402.9276	45	22014.23	37	0.00083676	31	37	TRUE	
1	QYGSFCTQL	737	745	9	HLA-A*02:01	737	38	38	19059.43402	29	22157.85	38	0.00327491	61	38	TRUE	
1	NFGAISSVL	951	959	9	HLA-A*02:01	951	38	38	23884.16155	32	22196.23	38	0.00309671	60	38	TRUE	
1	CDIPIGAGI	648	656	9	HLA-A*02:01	648	38	38	24666.64026	32	22171.28	38	0.0014669	43	38	TRUE	
1	QDLFLPFYS	56	64	9	HLA-A*02:01	56	38	38	31053.46024	35	22507.74	38	0.00138484	42	38	TRUE	
1	HTVSLLRST	661	669	9	HLA-A*02:01	661	38	38	34049.44207	36	22285.99	38	0.00263148	56	38	TRUE	
1	ATKMSECVL	1008	1016	9	HLA-A*02:01	1008	38	38	41410.45477	38	22175.6	38	0.00701455	78	38	TRUE	
1	WPWYVWLGF	1194	1202	9	HLA-A*02:01	1194	38	38	41986.53158	38	12528.43	23	0.00639146	76	38	TRUE	
1	TQLNRALSG	743	751	9	HLA-A*02:01	743	38	38	43162.83877	38	26252.46	46	0.0005105	23	38	TRUE	
1	RDISNVPFS	453	461	9	HLA-A*02:01	453	38	38	43462.0293	38	34970.14	71	3.68E-05	2.1	38	TRUE	
1	AGAALQIPF	872	880	9	HLA-A*02:01	872	38	38	52979.76163	41	22418.29	38	4.34E-05	2.5	38	TRUE	
1	PMGTQTHTM	143	151	9	HLA-A*02:01	143	39	39	7764.437528	21	22784.86	39	0.00541751	73	39	TRUE	
1	GTSWFITQR	1081	1089	9	HLA-A*02:01	1081	39	39	30416.55332	34	22934.24	39	0.00296074	59	39	TRUE	
1	PQAAPHGVV	1035	1043	9	HLA-A*02:01	1035	39	39	44885.90645	39	34029.52	67	0.00059814	25	39	TRUE	
1	NKSQSVIII	109	117	9	HLA-A*02:01	109	39	39	45615.24376	39	24658.54	42	0.00070632	28	39	TRUE	
1	IQESLTTTS	916	924	9	HLA-A*02:01	916	39	39	46785.36263	39	24817.54	43	0.0008414	31	39	TRUE	
1	LGFIAGLIA	1200	1208	9	HLA-A*02:01	1200	39	39	47001.31405	39	12989.46	23	0.00822243	81	39	TRUE	
1	DSTECANLL	727	735	9	HLA-A*02:01	727	39	39	47001.31405	39	24740.59	43	0.00063533	26	39	TRUE	
1	FAQVKQMYK	764	772	9	HLA-A*02:01	764	39	39	47985.4973	39	27026.59	48	0.00064047	26	39	TRUE	
1	TLKPIFKLP	215	223	9	HLA-A*02:01	215	39	39	57162.34016	42	23087.61	39	0.00108668	36	39	TRUE	
1	YLRHGKLRP	442	450	9	HLA-A*02:01	442	40	40	13338.59235	26	23509.85	40	0.0047874	70	40	TRUE	
1	GTNASSEVA	587	595	9	HLA-A*02:01	587	40	40	18159.75563	29	23454.2	40	0.00357273	63	40	TRUE	
1	VLTPSSKRF	537	545	9	HLA-A*02:01	537	40	40	23994.40583	32	23675.51	40	0.00159588	45	40	TRUE	
1	CTTFDDVQA	19	27	9	HLA-A*02:01	19	40	40	30982.03926	35	23283.28	40	0.033884416	99	40	TRUE	
1	GVIADYNYK	403	411	9	HLA-A*02:01	403	40	40	34523.11699	36	30202.66	56	0.00132221	40	40	TRUE	
1	RNFFSPQII	1089	1097	9	HLA-A*02:01	1089	40	40	47218.26226	39	23369.09	40	0.00198518	50	40	TRUE	
1	INNSTNVVI	117	125	9	HLA-A*02:01	117	40	40	51181.1453	40	26634.4	47	0.00087862	32	40	TRUE	
1	FSPAQDIWG	238	246	9	HLA-A*02:01	238	40	40	51299.13002	40	29147.7	53	0.0002327	13	40	TRUE	
1	VFNATKFPS	328	336	9	HLA-A*02:01	328	40	40	52012.77201	40	31603.74	60	0.00075492	29	40	TRUE	
1	EQYIKWPWY	1189	1197	9	HLA-A*02:01	1189	40	40	217826.1422	58	23404.77	40	0.00089743	32	40	TRUE	
1	SSIAYSNNT	685	693	9	HLA-A*02:01	685	41	41	18370.03569	29	23840.54	41	0.00279126	57	41	TRUE	
1	FFSTFKCYG	360	368	9	HLA-A*02:01	360	41	41	21286.78139	31	24035.57	41	0.00223821	52	41	TRUE	
1	AVLYQDVNC	595	603	9	HLA-A*02:01	595	41	41	25299.38709	32	24028.54	41	0.014037831	90	41	TRUE	
1	QPIDVVRDL	201	209	9	HLA-A*02:01	201	41	41	34364.49768	36	26923.86	47	0.00133537	41	41	TRUE	
1	YSNNTIAIP	689	697	9	HLA-A*02:01	689	41	41	48765.19891	39	23733.48	41	0.0027919	57	41	TRUE	
1	HADQLTPAW	611	619	9	HLA-A*02:01	611	41	41	53469.97733	41	30797.97	58	0.00029383	15	41	TRUE	
1	VYYPDEIFR	40	48	9	HLA-A*02:01	40	41	41	53469.97733	41	31699.61	60	3.38E-05	2	41	TRUE	
1	YNSTFFSTF	356	364	9	HLA-A*02:01	356	41	41	54089.13049	41	20820.89	35	0.00911591	83	41	TRUE	
1	AAYTAALVS	853	861	9	HLA-A*02:01	853	41	41	55094.7225	41	21229.66	36	0.00134958	41	41	TRUE	
1	FSPQIITTD	1092	1100	9	HLA-A*02:01	1092	41	41	55476.62088	41	25962.63	45	0.00050338	22	41	TRUE	
1	NLAATKMSE	1005	1013	9	HLA-A*02:01	1005	41	41	56899.70306	41	28723.47	52	0.00035237	18	41	TRUE	
1	GTGVLTPSS	534	542	9	HLA-A*02:01	534	41	41	56899.70306	41	29349.91	54	0.0007709	30	41	TRUE	
1	QAPNYTQHT	26	34	9	HLA-A*02:01	26	41	41	57030.87043	41	32273.42	62	0.0009732	34	41	TRUE	
1	GQSKRVDFC	1017	1025	9	HLA-A*02:01	1017	42	42	24894.87787	32	24274.18	42	0.00221973	52	42	TRUE	
1	YWPLNDYGF	475	483	9	HLA-A*02:01	475	42	42	25948.3651	32	24304.4	42	0.00172822	47	42	TRUE	
1	YEQYIKWPW	1188	1196	9	HLA-A*02:01	1188	42	42	30207.16724	34	24270.51	42	0.0021712	52	42	TRUE	
1	KPMGTQTHT	142	150	9	HLA-A*02:01	142	42	42	33121.50024	35	34350.25	69	0.00145077	42	42	TRUE	
1	INITNFRAI	226	234	9	HLA-A*02:01	226	42	42	39004.07654	37	24450.26	42	0.00619156	76	42	TRUE	
1	TVLPPLLTD	841	849	9	HLA-A*02:01	841	42	42	47218.26226	39	26932.89	47	0.00142922	42	42	TRUE	

seq #	peptide	start	end	peptide length	allele	peptide index	median binding	consensus perc	smm IC50	smm percentile	ann IC50	ann percentile	comblib_sidney	comblib_sidney	legacy consens	percentiles equal
1	KRSFIEDLL	796	804	9	HLA-A*02:01	796	42	42	51299.13002	40	24463.23	42	0.00337598	62	42	TRUE
1	VDTSYECDI	642	650	9	HLA-A*02:01	642	42	42	57558.57057	42	36567.44	76	0.00092555	33	42	TRUE
1	DDFMGCVLA	414	422	9	HLA-A*02:01	414	42	42	57691.25678	42	19239.01	33	0.00417446	67	42	TRUE
1	TQTHTMIFD	146	154	9	HLA-A*02:01	146	42	42	57691.25678	42	30327.42	56	0.00129748	40	42	TRUE
1	QKSIVAYTM	671	679	9	HLA-A*02:01	671	42	42	58091.15347	42	18678.62	32	0.00163682	45	42	TRUE
1	TDVSTAIHA	604	612	9	HLA-A*02:01	604	42	42	58091.1535	42	29031.24	53	0.00029813	16	42	TRUE
1	SFGGVSVIT	577	585	9	HLA-A*02:01	577	42	42	58493.82211	42	22588.96	38	0.02697118	97	42	TRUE
1	CLKGACSCG	1225	1233	9	HLA-A*02:01	1225	42	42	58628.66429	42	25829.26	45	0.0007656	30	42	TRUE
1	LCFSNVYAD	377	385	9	HLA-A*02:01	377	42	42	58899.28191	42	26128.61	46	0.00011003	6.2	42	TRUE
1	VLKGVKLHY	1246	1254	9	HLA-A*02:01	1246	42	42	60271.2224	42	18978.49	32	0.0033266	62	42	TRUE
1	TGIGYQPYR	487	495	9	HLA-A*02:01	487	42	42	61675.11955	42	40161.18	89	0.00027252	15	42	TRUE
1	MSFPQAAPH	1032	1040	9	HLA-A*02:01	1032	42	42	63111.71763	42	27676.13	49	0.00015101	8.2	42	TRUE
1	NFKHLREFV	178	186	9	HLA-A*02:01	178	42	42	63257.20517	42	27665.96	49	0.0010787	36	42	TRUE
1	SEILDISPC	568	576	9	HLA-A*02:01	568	42	42	79636.10307	45	24300.47	42	0.00015321	8.2	42	TRUE
1	ITNLCPFGE	319	327	9	HLA-A*02:01	319	42	42	83389.23698	46	24643.61	42	0.00068234	27	42	TRUE
1	IPFAMQMAY	878	886	9	HLA-A*02:01	878	42	42	206590.3351	57	24674.56	42	0.00014608	7.9	42	TRUE
1	APHGVVFLH	1038	1046	9	HLA-A*02:01	1038	42	42	220856.4057	58	24249.51	42	0.00105877	35	42	TRUE
1	INARDLICA	826	834	9	HLA-A*02:01	826	43	43	20375.58094	30	24958.64	43	0.00179391	48	43	TRUE
1	LNDYGFYTT	478	486	9	HLA-A*02:01	478	43	43	25299.38709	32	25027.33	43	0.00206443	51	43	TRUE
1	QFNKAISQI	908	916	9	HLA-A*02:01	908	43	43	29451.67504	34	28375.05	51	0.0015028	43	43	TRUE
1	KDGIYFAAT	84	92	9	HLA-A*02:01	84	43	43	44066.64633	38	24766.84	43	0.018424682	94	43	TRUE
1	LTGTGVLTP	532	540	9	HLA-A*02:01	532	43	43	47001.31405	39	25018.39	43	0.00993345	85	43	TRUE
1	VIIINNSTN	114	122	9	HLA-A*02:01	114	43	43	55094.7225	41	33642.57	66	0.00147299	43	43	TRUE
1	QIITTDNTF	1095	1103	9	HLA-A*02:01	1095	43	43	55221.72896	41	24771.39	43	0.01122018	87	43	TRUE
1	KVTLADAGF	807	815	9	HLA-A*02:01	807	43	43	65029.43789	43	23159.67	39	0.0042462	67	43	TRUE
1	STMNNKSQS	105	113	9	HLA-A*02:01	105	43	43	65329.60014	43	36444.59	76	0.00078705	30	43	TRUE
1	EVMPVSMAK	707	715	9	HLA-A*02:01	707	43	43	66851.322	43	26207.61	46	0.00108468	36	43	TRUE
1	STNVVIRAC	120	128	9	HLA-A*02:01	120	43	43	67469.88967	43	17494.33	30	0.00966718	84	43	TRUE
1	FEIDKGIYQ	293	301	9	HLA-A*02:01	293	43	43	67781.3165	43	29461.27	54	3.78E-05	2.1	43	TRUE
1	TFDDVQAPN	21	29	9	HLA-A*02:01	21	43	43	68408.48933	43	37104.7	78	0.00023394	13	43	TRUE
1	TVYDPLQPE	1118	1126	9	HLA-A*02:01	1118	43	43	68566.1872	43	26763.81	47	4.07E-05	2.3	43	TRUE
1	EVFAQVKQM	762	770	9	HLA-A*02:01	762	43	43	68882.67433	43	24977.28	43	0.00166341	46	43	TRUE
1	TFGNPVIPF	75	83	9	HLA-A*02:01	75	43	43	124196.6839	51	24984.58	43	0.00011479	6.3	43	TRUE
1	SFCTQLNRA	740	748	9	HLA-A*02:01	740	44	44	24105.15897	32	25434.9	44	0.010614511	86	44	TRUE
1	NQNAQALNT	935	943	9	HLA-A*02:01	935	44	44	37077.46213	37	25212.69	44	0.00238781	54	44	TRUE
1	TDDMIAAYT	848	856	9	HLA-A*02:01	848	44	44	52494.04026	40	25956.16	45	0.00154668	44	44	TRUE
1	SANLAATKM	1003	1011	9	HLA-A*02:01	1003	44	44	56378.04345	41	25191.42	44	0.00297303	59	44	TRUE
1	QNAQALNTL	936	944	9	HLA-A*02:01	936	44	44	56638.27268	41	25973.03	45	0.00156603	44	44	TRUE
1	STFKCYGVS	362	370	9	HLA-A*02:01	362	44	44	61109.6787	42	25229.61	44	0.00326588	61	44	TRUE
1	GTQTHTMIF	145	153	9	HLA-A*02:01	145	44	44	63257.20517	42	25429.95	44	0.0061235	75	44	TRUE
1	KGYHLMSFP	1027	1035	9	HLA-A*02:01	1027	44	44	69200.62231	44	29221.27	53	0.00079086	30	44	TRUE
1	FFAVSKPMG	137	145	9	HLA-A*02:01	137	44	44	70163.29886	44	36757.07	77	0.00061645	26	44	TRUE
1	FNCTFEYIS	157	165	9	HLA-A*02:01	157	44	44	70975.75157	44	25384.04	44	0.00024199	13	44	TRUE
1	FRVVPSGDV	305	313	9	HLA-A*02:01	305	44	44	73132.4293	44	28510.15	51	0.00065826	27	44	TRUE
1	TTEVMPVSM	705	713	9	HLA-A*02:01	705	44	44	74492.06268	44	20919.11	36	0.00306196	60	44	TRUE
1	AQALNTLVK	938	946	9	HLA-A*02:01	938	44	44	80930.0856	45	25491.37	44	0.00054739	24	44	TRUE
1	NQFNKAISQ	907	915	9	HLA-A*02:01	907	44	44	83197.44705	46	25462.72	44	0.00011768	6.5	44	TRUE
1	KSQSVIIIN	110	118	9	HLA-A*02:01	110	44	44	109423.348	49	25261.57	44	0.00031333	16	44	TRUE
1	PFFAVSKPM	136	144	9	HLA-A*02:01	136	44	44	112230.268	50	25491.37	44	0.00078832	30	44	TRUE
1	SSVLNDILS	956	964	9	HLA-A*02:01	956	44	44	222387.3092	58	25608.59	44	0.0011825	38	44	TRUE
1	YRVVVLSFE	494	502	9	HLA-A*02:01	494	44	44	515359.1605	68	18002.03	31	0.00155024	44	44	TRUE
1	AMQMAYRFN	881	889	9	HLA-A*02:01	881	45	45	21091.623	30	25625.23	45	0.00668652	77	45	TRUE
1	ITQRNFFSP	1086	1094	9	HLA-A*02:01	1086	45	45	28190.96874	34	25732.19	45	0.00435612	68	45	TRUE
1	VADYSVLYN	349	357	9	HLA-A*02:01	349	45	45	36992.1863	36	25729.12	45	0.00201094	50	45	TRUE
1	LIGAEHVDT	636	644	9	HLA-A*02:01	636	45	45	48429.50164	39	25695.74	45	0.010864256	87	45	TRUE
1	CCMTSCCSC	1217	1225	9	HLA-A*02:01	1217	45	45	75181.32925	45	15758.83	27	0.0033243	62	45	TRUE
1	NFFSPQIIT	1090	1098	9	HLA-A*02:01	1090	45	45	75181.32925	45	27581.68	49	0.00033258	17	45	TRUE
1	SNNTIAIPT	690	698	9	HLA-A*02:01	690	45	45	75354.64011	45	27088.95	48	0.00033022	17	45	TRUE
1	TKLNDLCFS	372	380	9	HLA-A*02:01	372	45	45	75876.97352	45	32104.86	61	0.00061094	25	45	TRUE
1	SDLDRCTTF	14	22	9	HLA-A*02:01	14	45	45	76932.52737	45	29070.21	53	0.00149417	43	45	TRUE
1	ISNCVADYS	345	353	9	HLA-A*02:01	345	45	45	77465.79817	45	24266.04	42	0.00592652	75	45	TRUE
1	GDINARDLI	824	832	9	HLA-A*02:01	824	45	45	78905.99489	45	37590.82	80	0.00158271	45	45	TRUE
1	NKDGFLYVY	189	197	9	HLA-A*02:01	189	45	45	153500.5727	53	25813.9	45	0.00070632	28	45	TRUE
1	YKLPDDFMG	410	418	9	HLA-A*02:01	410	46	46	22034.84501	31	28812.51	52	0.00169824	46	46	TRUE
1	QYGECLGDI	818	826	9	HLA-A*02:01	818	46	46	42083.32054	38	26378.87	46	0.00167958	46	46	TRUE
1	KGDDVRQIA	390	398	9	HLA-A*02:01	390	46	46	44066.6463	38	31581.51	60	0.00169434	46	46	TRUE
1	LGDISGINA	1148	1156	9	HLA-A*02:01	1148	46	46	67469.88967	43	26533.44	46	0.0034435	62	46	TRUE
1	NVVRGWVFG	96	104	9	HLA-A*02:01	96	46	46	70001.92775	44	26163.97	46	0.00656145	77	46	TRUE
1	LKYFGGFNF	776	784	9	HLA-A*02:01	776	46	46	85528.33153	46	21449.47	36	0.00204174	50	46	TRUE
1	NCDVVIGII	1107	1115	9	HLA-A*02:01	1107	46	46	85725.4947	46	15178.31	26	0.00308603	60	46	TRUE
1	KPIFKLPLG	217	225	9	HLA-A*02:01	217	46	46	86319.71544	46	30437.87	57	0.00082909	31	46	TRUE
1	DSEPVLKGV	1242	1250	9	HLA-A*02:01	1242	46	46	157801.0905	54	26372.02	46	0.00086099	32	46	TRUE
1	QPYRVVVLS	492	500	9	HLA-A*02:01	492	46	46	198202.8979	56	22831.25	39	0.00169668	46	46	TRUE
1	IDRLITGRL	975	983	9	HLA-A*02:01	975	46	46	217826.1422	58	26304.77	46	0.0016573	46	46	TRUE
1	GYHLMSFPQ	1028	1036	9	HLA-A*02:01	1028	46	46	322188.4741	63	26455.47	46	0.00012048	6.6	46	TRUE
1	FGEVFNATK	325	333	9	HLA-A*02:01	325	46	46	650294.3789	71	26393.42	46	0.00107721	36	46	TRUE
1	NFELCDNPF	129	137	9	HLA-A*02:01	129	46	46	997952.798	76	26153.79	46	0.00062791	26	46	TRUE
1	IFDNAFNCT	152	160	9	HLA-A*02:01	152	47	47	35654.14284	36	26625.76	47	0.00190941	49	47	TRUE
1	EIDRLNEVA	1164	1172	9	HLA-A*02:01	1164	47	47	38735.57436	37	26770.47	47	0.00233346	53	47	TRUE
1	YTTTGIGYQ	484	492	9	HLA-A*02:01	484	47	47	46570.40341	39	26932.3	47	0.00407099	66	47	TRUE
1	NCVADYSVL	347	355	9	HLA-A*02:01	347	47	47	75702.46132	45	26658.34	47	0.00372735	64	47	TRUE
1	SQNPLAELK	279	287	9	HLA-A*02:01	279	47	47	88533.98242	47	27395.49	48	0.00086756	32	47	TRUE
1	LIDLQELGK	1179	1187	9	HLA-A*02:01	1179	47	47	90596.20379	47	27789.56	49	0.00131009	40	47	TRUE
1	SVYAWERKK	336	344	9	HLA-A*02:01	336	47	47	91434.48014	47	35478.91	72	0.0003787	19	47	TRUE
1	LIKNQCVNF	519	527	9	HLA-A*02:01	519	47	47	91645.25838	47	24920.33	43	0.00952796	84	47	TRUE
1	GQTGVIADY	400	408	9	HLA-A*02:01	400	47	47	93564.2628	47	22737.81	39	0.003155	60	47	TRUE
1	ASSEVAVLY	590	598	9	HLA-A*02:01	590	47	47	93779.9507	47	19447.87	33	0.00281061	58	47	TRUE
1	IYFAATEKS	87	95	9	HLA-A*02:01	87	47	47	93779.95068	47	32529.33	63	0.00016376	8.7	47	TRUE
1	TSNFRVVPS	302	310	9	HLA-A*02:01	302	47	47	153147.5311	53	26719.56	47	0.00137658	41	47	TRUE
1	RDLICAQKF	829	837	9	HLA-A*02:01	829	47	47	398208.0176	65	26602.73	47	0.00037188	18	47	TRUE
1	YQPIDVVRD	200	208	9	HLA-A*02:01	200	48	48	19059.43402	29	27125.91	48	0.00873172	82	48	TRUE
1	AGICASYHT	654	662	9	HLA-A*02:01	654	48	48	21682.5322	31	27116.22	48	0.00279254	57	48	TRUE
1	AIHADQLTP	609	617	9	HLA-A*02:01	609	48	48	38468.92055	37	27240.91	48	0.0022914	53	48	TRUE
1	DISNVPFSP	454	462	9	HLA-A*02:01	454	48	48	40096.82638	37	28664.5	52	0.00187197	48	48	TRUE
1	GDVVRFPNI	311	319	9	HLA-A*02:01	311	48	48	45510.33161	39	26991.82	48	0.00213501	52	48	TRUE
1	TTDNTFVSG	1098	1106	9	HLA-A*02:01	1098	48	48	61959.79897	42	26994.74	48	0.00229087	53	48	TRUE
1	HTFGNPVIP	74	82	9	HLA-A*02:01	74	48	48	73809.11534	44	27167.31	48	0.00578496	74	48	TRUE
1	RFPNITNLC	315	323	9	HLA-A*02:01	315	48	48	96185.58706	48	28681.25	52	0.00097994	34	48	TRUE
1	DLICAQKFN	830	838	9	HLA-A*02:01	830	48	48	96407.31774	48	35556.93	73	0.00030088	16	48	TRUE
1	SQILPDPLK	785	793	9	HLA-A*02:01	785	48	48	96629.55956	48	30169.99	56	0.00082851	31	48	TRUE
1	DLIKNQCVN	518	526	9	HLA-A*02:01	518	48	48	97075.5813	48	37729.35	80	0.00047797	22	48	TRUE
1	VNFNFNGLT	525	533	9	HLA-A*02:01	525	48	48	98199.6636	48	29496.36	54	0.00075301	29	48	TRUE
1	AKNLNESLI	1172	1180	9	HLA-A*02:01	1172	48	48	98652.93264	48	32835.58	64	0.00046559	21	48	TRUE
1	ESLTTTSTA	918	926	9	HLA-A*02:01	918	48	48	98880.3511	48	33016.54	64	0.00127585	39	48	TRUE
1	EIRASANLA	999	1007	9	HLA-A*02:01	999	48	48	99565.75699	48	28550.28	51	0.0018578	48	48	TRUE
1	HGVVFLHVT	1040	1048	9	HLA-A*02:01	1040	48	48	100255.9138	48	18214.8	31	0.00957635	84	48	TRUE
1	TSAAAYFVG	247	255	9	HLA-A*02:01	247	48	48	101884.9414	48	30772.65	57	0.00122687	38	48	TRUE
1	GGVSVITPG	579	587	9	HLA-A*02:01	579	48	48	128265.5418	51	27304.35	48	0.00020137	12	48	TRUE
1	WGTSAAAYF	245	253	9	HLA-A*02:01	245	48	48	189282.298	56	26974.89	48	0.00047863	22	48	TRUE
1	RGVYYPDEI	38	46	9	HLA-A*02:01	38	49	49	12708.95963	26	27529.51	49	0.00656145	77	49	TRUE
1	NGLTGTGVL	530	538	9	HLA-A*02:01	530	49	49	56378.04345	41	32558.56	63	0.00192309	49	49	TRUE
1	KFNGLTVLP	836	844	9	HLA-A*02:01	836	49	49	59307.55223	42	27519.37	49	0.00191867	49	49	TRUE
1	TGFHTINHT	67	75	9	HLA-A*02:01	67	49	49	89147.67066	47	27520.56	49	0.00317687	60	49	TRUE
1	GAISSVLND	953	961	9	HLA-A*02:01	953	49	49	102591.1741	49	33184.52	65	0.00084392	31	49	TRUE
1	KNQCVNFNF	521	529	9	HLA-A*02:01	521	49	49	104980.8295	49	25221.43	44	0.00346577	63	49	TRUE
1	QHTSSMRGV	32	40	9	HLA-A*02:01	32	49	49	105465.399	49	34928.15	70	0.00073689	29	49	TRUE
1	FTTAPAICH	1057	1065	9	HLA-A*02:01	1057	49	49	106441.2583	49	29529.57	54	8.32E-05	4.8	49	TRUE
1	YKTPTLKYF	771	779	9	HLA-A*02:01	771	49	49	106932.5688	49	26702.51	47	0.00283792	58	49	TRUE
1	RALSGIAAE	747	755	9	HLA-A*02:01	747	49	49	107922.0037	49	28907.43	52	0.00040383	20	49	TRUE
1	ANLLLQYGS	732	740	9	HLA-A*02:01	732	49	49	108920.5937	49	30491.27	57	0.00032048	16	49	TRUE
1	FKNKDGFLY	187	195	9	HLA-A*02:01	187	49	49	109928.4236	49	29570.81	54	0.00136773	41	49	TRUE
1	VGYLKPTTF	254	262	9	HLA-A*02:01	254	49	49	240497.1865	59	25654.35	45	0.00192752	49	49	TRUE

seq #	peptide	start	end	peptide length	allele	peptide index	median binding	consensus perc	smm IC50	smm percentile	ann IC50	ann percentile	comblib_sidney	comblib_sidney	legacy consens	percentiles equal
1	DGFLYVYKG	191	199	9	HLA-A*02:01	191	49	49	263699.9212	60	27625.28	49	0.00036745	18	49	TRUE
1	NCYWPLNDY	473	481	9	HLA-A*02:01	473	49	49	2270439.846	85	27759.81	49	0.00158271	45	49	TRUE
1	GKPCTPPAL	464	472	9	HLA-A*02:01	464	50	50	24553.30734	32	27833.79	50	0.00516416	72	50	TRUE
1	IAPGQTGVI	397	405	9	HLA-A*02:01	397	50	50	25769.73782	32	28014.16	50	0.00264363	56	50	TRUE
1	SFVVKGDDV	386	394	9	HLA-A*02:01	386	50	50	50478.91366	40	28124.71	50	0.00531986	72	50	TRUE
1	ITPGTNASS	584	592	9	HLA-A*02:01	584	50	50	52494.0403	40	28153.33	50	0.00405509	66	50	TRUE
1	MGTQTHTMI	144	152	9	HLA-A*02:01	144	50	50	63403.02809	42	28143.29	50	0.00603949	75	50	TRUE
1	YRYLRHGKL	440	448	9	HLA-A*02:01	440	50	50	75876.97352	45	28152.73	50	0.00223049	52	50	TRUE
1	PIGAGICAS	651	659	9	HLA-A*02:01	651	50	50	104018.3595	49	32266.78	62	0.0020128	50	50	TRUE
1	FYSNVTGFH	62	70	9	HLA-A*02:01	62	50	50	114844.4468	50	31744.93	60	5.89E-05	3.5	50	TRUE
1	SSNFGAISS	949	957	9	HLA-A*02:01	949	50	50	115109.1906	50	29431.96	54	0.00100647	35	50	TRUE
1	QLTPAWRIY	614	622	9	HLA-A*02:01	614	50	50	115640.5104	50	23738.87	41	0.00206538	51	50	TRUE
1	AEVQIDRLI	971	979	9	HLA-A*02:01	971	50	50	116174.2828	50	26456.61	46	0.00572796	74	50	TRUE
1	DEDDSEPVL	1239	1247	9	HLA-A*02:01	1239	50	50	117249.2302	50	39362.72	86	0.00015567	8.4	50	TRUE
1	GFHTINHTF	68	76	9	HLA-A*02:01	68	50	50	118606.9123	50	27677.33	49	0.0054063	72	50	TRUE
1	VVIRACNFE	123	131	9	HLA-A*02:01	123	50	50	132163.034	52	27893.79	50	0.00115345	37	50	TRUE
1	FYTTTGIGY	483	491	9	HLA-A*02:01	483	50	50	213850.367	57	27836.81	50	0.00089166	32	50	TRUE
1	PVIPFKDGI	79	87	9	HLA-A*02:01	79	51	51	21933.60409	31	29497.31	54	0.00209315	51	51	TRUE
1	AWNTRNIDA	422	430	9	HLA-A*02:01	422	51	51	34523.11699	36	33381.5	65	0.00212618	51	51	TRUE
1	LQPELDSFK	1123	1131	9	HLA-A*02:01	1123	51	51	38557.60057	37	29286.15	53	0.00208113	51	51	TRUE
1	AALVSGTAT	857	865	9	HLA-A*02:01	857	51	51	52857.91156	40	28262.9	51	0.015602709	92	51	TRUE
1	LKGVKLHYT	1247	1255	9	HLA-A*02:01	1247	51	51	54338.79457	41	28305.75	51	0.00709578	78	51	TRUE
1	NDYGFYTTT	479	487	9	HLA-A*02:01	479	51	51	75528.3505	45	28561.71	51	0.00328398	61	51	TRUE
1	QRNFFSPQI	1088	1096	9	HLA-A*02:01	1088	51	51	76755.58746	45	28504.91	51	0.00294713	59	51	TRUE
1	TVCGPKLST	509	517	9	HLA-A*02:01	509	51	51	95523.45013	48	28543.17	51	0.012735031	89	51	TRUE
1	MAYRFNGIG	884	892	9	HLA-A*02:01	884	51	51	103302.3021	49	30357.94	56	0.00208065	51	51	TRUE
1	KSIVAYTMS	672	680	9	HLA-A*02:01	672	51	51	119154.3769	51	27234.13	48	0.010539014	86	51	TRUE
1	FGAISSVLN	952	960	9	HLA-A*02:01	952	51	51	119704.3685	51	29448.2	54	0.00169122	46	51	TRUE
1	LGINITNFR	224	232	9	HLA-A*02:01	224	51	51	119704.3685	51	29924.53	55	0.00013044	7.1	51	TRUE
1	ITDAVDCSQ	272	280	9	HLA-A*02:01	272	51	51	122775.0162	51	26618.27	47	0.00234261	53	51	TRUE
1	GVVFLHVTY	1041	1049	9	HLA-A*02:01	1041	51	51	123058.0418	51	17901.22	31	0.015170504	91	51	TRUE
1	GVYYPDEIF	39	47	9	HLA-A*02:01	39	51	51	123058.0418	51	25003.78	43	0.00404576	66	51	TRUE
1	FNKVTLADA	805	813	9	HLA-A*02:01	805	51	51	123341.7199	51	23598.53	40	0.00217671	52	51	TRUE
1	RSFIEDLLF	797	805	9	HLA-A*02:01	797	51	51	125345.8616	51	15557.23	27	0.00579162	74	51	TRUE
1	SNFRVVPSG	303	311	9	HLA-A*02:01	303	51	51	126505.6726	51	31825.06	61	0.00049636	22	51	TRUE
1	GKYEQYIKW	1186	1194	9	HLA-A*02:01	1186	51	51	126505.6726	51	32395.51	62	0.00081979	31	51	TRUE
1	VTYVPSQER	1047	1055	9	HLA-A*02:01	1047	51	51	129750.7868	51	33633.46	66	0.00177174	47	51	TRUE
1	TKRSFIEDL	795	803	9	HLA-A*02:01	795	51	51	157076.0603	54	28320.15	51	0.00068391	27	51	TRUE
1	GVTQNVLYE	892	900	9	HLA-A*02:01	892	51	51	169086.9148	55	28264.12	51	0.00108393	36	51	TRUE
1	FNTLKPIFK	213	221	9	HLA-A*02:01	213	51	51	333510.8752	63	28399	51	7.98E-05	4.6	51	TRUE
1	KRFQPFQQF	543	551	9	HLA-A*02:01	543	51	51	781825.8038	74	28583.66	51	0.00171554	47	51	TRUE
1	TECANLLLQ	729	737	9	HLA-A*02:01	729	51	51	1671513.929	82	28365.83	51	0.00142462	42	51	TRUE
1	PVSMAKTSV	710	718	9	HLA-A*02:01	710	52	52	15244.38821	27	31052.59	58	0.00224285	52	52	TRUE
1	QILPDPLKP	786	794	9	HLA-A*02:01	786	52	52	27740.22632	33	28770.45	52	0.00372477	64	52	TRUE
1	VVRDLPSGF	205	213	9	HLA-A*02:01	205	52	52	39912.59819	37	28871.49	52	0.00222331	52	52	TRUE
1	RDLPSGFNT	207	215	9	HLA-A*02:01	207	52	52	53840.61351	41	33510.68	66	0.00213501	52	52	TRUE
1	YGSFCTQLN	738	746	9	HLA-A*02:01	738	52	52	131859.0675	52	32006	61	0.00033744	17	52	TRUE
1	VSMAKTSVD	711	719	9	HLA-A*02:01	711	52	52	131859.0675	52	41690.02	93	0.0001191	6.5	52	TRUE
1	LGADSSIAY	681	689	9	HLA-A*02:01	681	52	52	133079.1444	52	32553.99	63	0.00092257	33	52	TRUE
1	PGTNASSEV	586	594	9	HLA-A*02:01	586	52	52	134310.511	52	41899.83	94	0.00027403	15	52	TRUE
1	YSTGNNVFQ	622	630	9	HLA-A*02:01	622	52	52	135865.753	52	31534.74	60	0.00122603	38	52	TRUE
1	SCLKGACSC	1224	1232	9	HLA-A*02:01	1224	52	52	137439.0042	52	28589.23	51	0.00215625	52	52	TRUE
1	RFQPFQQFG	544	552	9	HLA-A*02:01	544	52	52	138710.7117	52	32959.44	64	0.00034986	17	52	TRUE
1	NYTQHTSSM	29	37	9	HLA-A*02:01	29	52	52	138710.7117	52	38610.22	84	0.00109698	36	52	TRUE
1	GDISGINAS	1149	1157	9	HLA-A*02:01	1149	52	52	138710.7117	52	37216.88	79	0.0004087	20	52	TRUE
1	NHTFGNPVI	73	81	9	HLA-A*02:01	73	52	52	139350.9712	52	38744.54	84	0.00081546	31	52	TRUE
1	PHGVVFLHV	1039	1047	9	HLA-A*02:01	1039	52	52	141615.2424	52	21063.09	36	0.00307044	60	52	TRUE
1	DLGDISGIN	1147	1155	9	HLA-A*02:01	1147	52	52	142596.8723	52	35481.22	72	0.0016573	46	52	TRUE
1	TITDAVDCS	271	279	9	HLA-A*02:01	271	53	53	28386.3792	34	29470.52	54	0.00224388	53	53	TRUE
1	AEIRASANL	998	1006	9	HLA-A*02:01	998	53	53	29316.35689	34	29065.5	53	0.00676083	78	53	TRUE
1	LICAQKFNG	831	839	9	HLA-A*02:01	831	53	53	31268.71254	35	29108.29	53	0.00493174	70	53	TRUE
1	ATSTGNYNY	430	438	9	HLA-A*02:01	430	53	53	49557.56965	40	31900.22	61	0.00234423	53	53	TRUE
1	TFKCYGVSA	363	371	9	HLA-A*02:01	363	53	53	63549.18716	42	29307.39	53	0.03356603	99	53	TRUE
1	MNNKSQSVI	107	115	9	HLA-A*02:01	107	53	53	70487.15793	44	36893.31	77	0.002311	53	53	TRUE
1	EVAKNLNES	1170	1178	9	HLA-A*02:01	1170	53	53	143916.305	53	37763.23	81	0.00208449	51	53	TRUE
1	TATAGWTFG	863	871	9	HLA-A*02:01	863	53	53	147608.0354	53	33992.36	67	0.00232381	53	53	TRUE
1	STDLIKNQC	516	524	9	HLA-A*02:01	516	53	53	148973.8357	53	28417.45	51	0.00601589	75	53	TRUE
1	TKMSECVLG	1009	1017	9	HLA-A*02:01	1009	53	53	149317.2559	53	33321.23	65	0.00042267	20	53	TRUE
1	GECLGDINA	820	828	9	HLA-A*02:01	820	53	53	149661.4677	53	29579.45	54	0.00170216	46	53	TRUE
1	RQIAPGQTG	395	403	9	HLA-A*02:01	395	53	53	150352.2737	53	33731.12	67	0.00125314	39	53	TRUE
1	LYQDVNCTD	597	605	9	HLA-A*02:01	597	53	53	150698.8715	53	38718.14	84	0.00206681	51	53	TRUE
1	AYTAALVSG	854	862	9	HLA-A*02:01	854	53	53	151046.2682	53	31815.41	61	0.00171475	47	53	TRUE
1	GIAAEQDRN	751	759	9	HLA-A*02:01	751	53	53	154564.5878	53	42900.81	96	0.00200078	50	53	TRUE
1	PATVCGPKL	507	515	9	HLA-A*02:01	507	53	53	155635.9783	53	35258.12	72	0.00211349	51	53	TRUE
1	PFERDISNV	450	458	9	HLA-A*02:01	450	53	53	155994.7563	53	37047.72	78	0.00116332	37	53	TRUE
1	TLKYFGGFN	775	783	9	HLA-A*02:01	775	53	53	157076.0603	54	29288.68	53	0.00140346	42	53	TRUE
1	SEVAVLYQD	592	600	9	HLA-A*02:01	592	53	53	176648.5187	55	29296.92	53	0.00057161	24	53	TRUE
1	FKEELDKYF	1130	1138	9	HLA-A*02:01	1130	53	53	258886.8552	60	29251.31	53	0.00124738	39	53	TRUE
1	FPNITNLCP	316	324	9	HLA-A*02:01	316	53	53	264917.1047	60	28975.07	53	0.00115798	37	53	TRUE
1	QCVNFNFNG	523	531	9	HLA-A*02:01	523	53	53	270464.3321	60	29163.46	53	0.00095808	34	53	TRUE
1	ELKCSVKSF	285	293	9	HLA-A*02:01	285	53	53	297241.8804	62	26646.22	47	0.00225944	53	53	TRUE
1	AIPTNFSIS	695	703	9	HLA-A*02:01	695	54	54	41986.53158	38	29416.37	54	0.01892344	94	54	TRUE
1	DVVNQNAQA	932	940	9	HLA-A*02:01	932	54	54	47436.21186	39	36030.58	74	0.00236701	54	54	TRUE
1	TAPAICHEG	1059	1067	9	HLA-A*02:01	1059	54	54	71303.36075	44	30642.74	57	0.00239994	54	54	TRUE
1	CYGVSATKL	366	374	9	HLA-A*02:01	366	54	54	7.68E+04	45	29625.88	5.40E+01	0.011808643	88	54	TRUE
1	DFCGKGYHL	1023	1031	9	HLA-A*02:01	1023	54	54	7.80E+04	45	29661.17	5.40E+01	0.00405602	66	54	TRUE
1	PCSFGGVSV	575	583	9	HLA-A*02:01	575	54	54	1.26E+05	51	29463.18	5.40E+01	0.00242773	54	54	TRUE
1	ELDKYFKNH	1133	1141	9	HLA-A*02:01	1133	54	54	1.57E+05	54	32887.84	6.40E+01	0.00214882	52	54	TRUE
1	TEVMPVSMA	706	714	9	HLA-A*02:01	706	54	54	1.62E+05	54	27646.81	4.90E+01	0.00394457	65	54	TRUE
1	KTSVDCNMY	715	723	9	HLA-A*02:01	715	54	54	1.65E+05	54	27118.28	4.80E+01	0.016595869	92	54	TRUE
1	ICHEGKAYF	1063	1071	9	HLA-A*02:01	1063	54	54	1.65E+05	54	25121.74	4.30E+01	0.00377051	64	54	TRUE
1	QTHTMIFDN	147	155	9	HLA-A*02:01	147	54	54	1.66E+05	54	31519.73	6.00E+01	0.00060284	25	54	TRUE
1	FGSTMNNKS	103	111	9	HLA-A*02:01	103	54	54	1.66E+05	54	39781.04	8.80E+01	0.00021038	12	54	TRUE
1	PSQERNFTT	1051	1059	9	HLA-A*02:01	1051	54	54	1.67E+05	54	37494.94	8.00E+01	0.00163155	45	54	TRUE
1	TQRNFFSPQ	1087	1095	9	HLA-A*02:01	1087	54	54	2.33E+05	59	29378.83	5.40E+01	0.00051844	23	54	TRUE
1	TTGIGYQPY	486	494	9	HLA-A*02:01	486	54	54	4.37E+05	66	29348.32	5.40E+01	0.00212814	51	54	TRUE
1	LRHGKLRPF	443	451	9	HLA-A*02:01	443	54	54	8.96E+05	75	29702.92	5.40E+01	0.00173141	47	54	TRUE
1	GRLQSLQTY	981	989	9	HLA-A*02:01	981	54	54	1.57E+06	81	29702.92	5.40E+01	0.00111532	36	54	TRUE
1	ECANLLLQY	730	738	9	HLA-A*02:01	730	54	54	2.58E+06	86	27775.73	4.90E+01	0.0023518	54	54	TRUE
1	QIDRLITGR	974	982	9	HLA-A*02:01	974	55	55	3.22E+04	35	29966.98	5.50E+01	0.00340722	62	55	TRUE
1	LADAGFMKQ	810	818	9	HLA-A*02:01	810	55	55	1.18E+05	50	30307.06	5.60E+01	0.0025229	55	55	TRUE
1	QTQAGCLIG	630	638	9	HLA-A*02:01	630	55	55	1.69E+05	55	29464.46	5.40E+01	0.00504661	71	55	TRUE
1	LPLGINITN	222	230	9	HLA-A*02:01	222	55	55	1.72E+05	55	31351.71	5.90E+01	0.00034658	17	55	TRUE
1	NQCVNFNFN	522	530	9	HLA-A*02:01	522	55	55	1.73E+05	55	28739.02	5.20E+01	0.0027555	57	55	TRUE
1	AYFVGYLKP	251	259	9	HLA-A*02:01	251	55	55	1.75E+05	55	27730.7	4.90E+01	0.00998389	85	55	TRUE
1	GSFCTQLNR	739	747	9	HLA-A*02:01	739	55	55	1.81E+05	55	29050.72	5.30E+01	0.00589115	75	55	TRUE
1	DVVIGIINN	1109	1117	9	HLA-A*02:01	1109	55	55	3.35E+05	63	29811.1	5.50E+01	0.00107498	36	55	TRUE
1	FVVKGDDVR	387	395	9	HLA-A*02:01	387	56	56	3.99E+04	37	35416.78	7.20E+01	0.00257276	56	56	TRUE
1	YAWERKKIS	338	346	9	HLA-A*02:01	338	56	56	4.26E+04	38	30141.6	5.60E+01	0.016565326	92	56	TRUE
1	LVSGTATAG	859	867	9	HLA-A*02:01	859	56	56	4.30E+04	38	30236.33	5.60E+01	0.00524807	72	56	TRUE
1	WFITQRNFF	1084	1092	9	HLA-A*02:01	1084	56	56	1.45E+05	53	30347.76	5.60E+01	0.00543125	73	56	TRUE
1	IGAGICASY	652	660	9	HLA-A*02:01	652	56	56	1.81E+05	56	24799.83	4.30E+01	0.0028774	58	56	TRUE
1	ENGTITDAV	268	276	9	HLA-A*02:01	268	56	56	1.84E+05	56	32618.15	6.30E+01	0.00095675	34	56	TRUE
1	ADYNYKLPD	406	414	9	HLA-A*02:01	406	56	56	1.86E+05	56	39881.46	8.80E+01	0.00032174	17	56	TRUE
1	NVVIRACNF	122	130	9	HLA-A*02:01	122	56	56	1.86E+05	56	20683.01	3.50E+01	0.00647143	77	56	TRUE
1	TGTGVLTPS	533	541	9	HLA-A*02:01	533	56	56	1.87E+05	56	30540.45	5.70E+01	0.00140929	42	56	TRUE
1	DVVRDLPSG	204	212	9	HLA-A*02:01	204	56	56	1.88E+05	56	40510.75	9.00E+01	0.00249	55	56	TRUE
1	ADSSIAYSN	683	691	9	HLA-A*02:01	683	56	56	1.90E+05	56	38134.84	8.20E+01	0.00023747	13	56	TRUE
1	TSWFITQRN	1082	1090	9	HLA-A*02:01	1082	56	56	1.97E+05	56	30457.96	5.70E+01	0.00044055	21	56	TRUE
1	KCYGVSATK	365	373	9	HLA-A*02:01	365	56	56	1.99E+05	57	22514.3	3.80E+01	0.00261036	56	56	TRUE
1	NRALSGIAA	746	754	9	HLA-A*02:01	746	56	56	2.54E+05	60	30377.66	5.60E+01	0.00138484	42	56	TRUE
1	QALNTLVKQ	939	947	9	HLA-A*02:01	939	56	56	3.36E+05	63	30397.04	5.60E+01	0.0022228	52	56	TRUE

seq #	peptide	start	end	peptide length	allele	peptide index	median binding	consensus perc	smm IC50	smm percentile	ann IC50	ann percentile	comblib_sidney	comblib_sidney	legacy consens	percentiles equal
1	NASVVNIQK	1155	1163	9	HLA-A*02:01	1155	56	56	5.19E+05	68	30371.75	5.60E+01	0.00203939	50	56	TRUE
1	FQQFGRDVS	548	556	9	HLA-A*02:01	548	57	57	4.15E+04	38	30674.57	5.70E+01	0.00317687	60	57	TRUE
1	GDSTECANL	726	734	9	HLA-A*02:01	726	57	57	4.28E+04	38	30559.64	5.70E+01	0.017354016	93	57	TRUE
1	KFDEDDSEP	1237	1245	9	HLA-A*02:01	1237	57	57	6.40E+04	43	38805.8	8.40E+01	0.00271019	57	57	TRUE
1	NTLVKQLSS	942	950	9	HLA-A*02:01	942	57	57	9.19E+04	47	30731.04	5.70E+01	0.00360579	63	57	TRUE
1	SPDVDLGDI	1143	1151	9	HLA-A*02:01	1143	57	57	1.15E+05	50	30737.37	5.70E+01	0.00640619	76	57	TRUE
1	LGDINARDL	823	831	9	HLA-A*02:01	823	57	57	1.79E+05	55	30499.51	5.70E+01	0.00506991	71	57	TRUE
1	LVKQLSSNF	944	952	9	HLA-A*02:01	944	57	57	2.00E+05	57	29725.11	5.50E+01	0.010185914	85	57	TRUE
1	VPSGDVVRF	308	316	9	HLA-A*02:01	308	57	57	2.04E+05	57	29967.95	5.50E+01	0.0051357	71	57	TRUE
1	REGVFVFNG	1073	1081	9	HLA-A*02:01	1073	57	57	2.04E+05	57	25500.48	4.40E+01	0.00371535	64	57	TRUE
1	LSSNFGAIS	948	956	9	HLA-A*02:01	948	57	57	2.06E+05	57	29264.61	5.30E+01	0.011397248	87	57	TRUE
1	GKGYHLMSF	1026	1034	9	HLA-A*02:01	1026	57	57	2.07E+05	57	28695.83	5.20E+01	0.00885116	83	57	TRUE
1	THTMIFDNA	148	156	9	HLA-A*02:01	148	57	57	2.09E+05	57	29252.59	5.30E+01	0.023680998	96	57	TRUE
1	ISITTEVMP	702	710	9	HLA-A*02:01	702	57	57	2.10E+05	57	29481.03	5.40E+01	0.00361327	63	57	TRUE
1	QAAPHGVVF	1036	1044	9	HLA-A*02:01	1036	57	57	2.13E+05	57	30725.73	5.70E+01	0.00668652	77	57	TRUE
1	GFNFSQILP	781	789	9	HLA-A*02:01	781	57	57	2.13E+05	57	33147.55	6.50E+01	0.00187932	49	57	TRUE
1	KTPTLKYFG	772	780	9	HLA-A*02:01	772	58	58	2.81E+04	33	30942.92	5.80E+01	0.00331131	61	58	TRUE
1	YVPSQERNF	1049	1057	9	HLA-A*02:01	1049	58	58	6.04E+04	42	32175.1	6.20E+01	0.00288403	58	58	TRUE
1	GTITDAVDC	270	278	9	HLA-A*02:01	270	58	58	6.86E+04	43	30963.34	5.80E+01	0.00755614	80	58	TRUE
1	IGAEHVDTS	637	645	9	HLA-A*02:01	637	58	58	8.07E+04	45	34878.31	7.00E+01	0.00283792	58	58	TRUE
1	CAQKFNGLT	833	841	9	HLA-A*02:01	833	58	58	1.11E+05	50	30773.64	5.80E+01	0.00618301	76	58	TRUE
1	PQIITTDNT	1094	1102	9	HLA-A*02:01	1094	58	58	1.55E+05	53	35048.16	7.10E+01	0.00283009	58	58	TRUE
1	YNYKLPDDF	408	416	9	HLA-A*02:01	408	58	58	1.84E+05	56	30823.62	5.80E+01	0.00446478	68	58	TRUE
1	LFNKVTLAD	804	812	9	HLA-A*02:01	804	58	58	1.96E+05	56	31058.64	5.80E+01	0.00345939	62	58	TRUE
1	YFKNHTSPD	1137	1145	9	HLA-A*02:01	1137	58	58	2.14E+05	58	43067.3	9.70E+01	0.00021727	13	58	TRUE
1	SYHTVSLLR	659	667	9	HLA-A*02:01	659	58	58	2.18E+05	58	26881.06	4.70E+01	0.01864661	94	58	TRUE
1	GACSCGSCC	1228	1236	9	HLA-A*02:01	1228	58	58	2.18E+05	58	33435.36	6.60E+01	0.00116011	37	58	TRUE
1	VTGFHTINH	66	74	9	HLA-A*02:01	66	58	58	2.21E+05	58	33299.62	6.50E+01	0.00120282	38	58	TRUE
1	TTAPAICHE	1058	1066	9	HLA-A*02:01	1058	58	58	2.25E+05	58	33724.55	6.70E+01	0.00093325	33	58	TRUE
1	FDDVQAPNY	22	30	9	HLA-A*02:01	22	58	58	2.30E+05	58	34126.86	6.80E+01	0.00020249	12	58	TRUE
1	NAPATVCGP	505	513	9	HLA-A*02:01	505	58	58	2.30E+05	58	34149.03	6.80E+01	0.00264423	56	58	TRUE
1	KGIYQTSNF	297	305	9	HLA-A*02:01	297	58	58	2.31E+05	58	36804.81	7.70E+01	0.00140281	42	58	TRUE
1	GAGICASYH	653	661	9	HLA-A*02:01	653	58	58	4.04E+05	65	30921.84	5.80E+01	0.00075753	29	58	TRUE
1	PNITNLCPF	317	325	9	HLA-A*02:01	317	58	58	9.08E+05	75	30830.97	5.80E+01	0.00106316	36	58	TRUE
1	LSRLDKVEA	963	971	9	HLA-A*02:01	963	59	59	1.19E+05	51	31247.39	5.90E+01	0.015128644	91	59	TRUE
1	AKTSVDCNM	714	722	9	HLA-A*02:01	714	59	59	1.64E+05	54	31303.24	5.90E+01	0.00566239	74	59	TRUE
1	AELKCSVKS	284	292	9	HLA-A*02:01	284	59	59	2.04E+05	57	36000.57	7.40E+01	0.00303389	59	59	TRUE
1	LKGACSCGS	1226	1234	9	HLA-A*02:01	1226	59	59	2.35E+05	59	36447.37	7.60E+01	0.00228034	53	59	TRUE
1	PFGEVFNAT	324	332	9	HLA-A*02:01	324	59	59	2.36E+05	59	31396.51	5.90E+01	0.0093261	84	59	TRUE
1	YHTVSLLRS	660	668	9	HLA-A*02:01	660	59	59	2.38E+05	59	27108	4.80E+01	0.011492116	87	59	TRUE
1	SWFITQRNF	1083	1091	9	HLA-A*02:01	1083	59	59	2.44E+05	59	25376.08	4.40E+01	0.00694384	78	59	TRUE
1	IRACNFELC	125	133	9	HLA-A*02:01	125	59	59	2.46E+05	59	26460.33	4.60E+01	0.00450505	68	59	TRUE
1	LDISPCSFG	571	579	9	HLA-A*02:01	571	59	59	2.51E+05	59	36413.86	7.60E+01	0.00035188	18	59	TRUE
1	IGIINNTVY	1112	1120	9	HLA-A*02:01	1112	59	59	8.28E+05	74	30252.03	5.60E+01	0.00301301	59	59	TRUE
1	LAATKMSEC	1006	1014	9	HLA-A*02:01	1006	60	60	3.05E+04	34	31588.01	6.00E+01	0.00574778	74	60	TRUE
1	VKGDDVRQI	389	397	9	HLA-A*02:01	389	60	60	3.64E+04	36	33393.06	6.50E+01	0.00306196	60	60	TRUE
1	NYNYKYRYL	435	443	9	HLA-A*02:01	435	60	60	4.87E+04	39	31686.25	6.00E+01	0.018758582	94	60	TRUE
1	YKYRYLRHG	438	446	9	HLA-A*02:01	438	60	60	5.29E+04	40	31705.46	6.00E+01	0.00534564	72	60	TRUE
1	TAIHADQLT	608	616	9	HLA-A*02:01	608	60	60	7.55E+04	45	31619.13	6.00E+01	0.00338221	62	60	TRUE
1	AFSLDVSEK	167	175	9	HLA-A*02:01	167	60	60	2.54E+05	60	34479.82	6.90E+01	0.00156387	44	60	TRUE
1	CDNPFFAVS	133	141	9	HLA-A*02:01	133	60	60	2.54E+05	60	29209.89	5.30E+01	0.00573324	74	60	TRUE
1	DGKPCTPPA	463	471	9	HLA-A*02:01	463	60	60	2.56E+05	60	37845.47	8.10E+01	2.22E-05	1.4	60	TRUE
1	RNIDATSTG	426	434	9	HLA-A*02:01	426	60	60	2.57E+05	60	41642.68	9.30E+01	0.00018698	10	60	TRUE
1	CTPPALNCY	467	475	9	HLA-A*02:01	467	60	60	2.58E+05	60	28741.21	5.20E+01	0.00381944	65	60	TRUE
1	ASANLAATK	1002	1010	9	HLA-A*02:01	1002	60	60	2.61E+05	60	32658.41	6.30E+01	0.00114578	37	60	TRUE
1	TPPALNCYW	468	476	9	HLA-A*02:01	468	60	60	2.61E+05	60	33304.3	6.50E+01	0.00123396	39	60	TRUE
1	VFVFNGTSW	1076	1084	9	HLA-A*02:01	1076	60	60	2.70E+05	60	30048.8	5.50E+01	0.016542456	92	60	TRUE
1	DKVEAEVQI	967	975	9	HLA-A*02:01	967	60	60	2.72E+05	60	36658.16	7.70E+01	0.00114868	37	60	TRUE
1	LQELGKYEQ	1182	1190	9	HLA-A*02:01	1182	60	60	2.74E+05	60	35339.09	7.20E+01	0.00203564	50	60	TRUE
1	TFMLKYDEN	261	269	9	HLA-A*02:01	261	60	60	2.74E+05	60	40041.86	8.80E+01	0.00185823	48	60	TRUE
1	TFFSTFKCY	359	367	9	HLA-A*02:01	359	60	60	6.31E+05	71	27791.96	4.90E+01	0.00308248	60	60	TRUE
1	NGTSWFITQ	1080	1088	9	HLA-A*02:01	1080	60	60	7.76E+05	73	31728.1	6.00E+01	0.00066328	27	60	TRUE
1	SSEVAVLYQ	591	599	9	HLA-A*02:01	591	60	60	9.55E+05	76	25490.27	4.40E+01	0.00317322	60	60	TRUE
1	INASVVNIQ	1154	1162	9	HLA-A*02:01	1154	60	60	1.10E+06	77	31603.04	6.00E+01	0.00097611	34	60	TRUE
1	VNCTDVSTA	601	609	9	HLA-A*02:01	601	61	61	6.08E+04	42	32123.62	6.10E+01	0.00591289	75	61	TRUE
1	ICASYHTVS	656	664	9	HLA-A*02:01	656	61	61	1.51E+05	53	37504.27	8.00E+01	0.00323892	61	61	TRUE
1	ISQIQESLT	913	921	9	HLA-A*02:01	913	61	61	2.06E+05	57	34591.56	6.90E+01	0.00320479	61	61	TRUE
1	GNFKHLREF	177	185	9	HLA-A*02:01	177	61	61	2.80E+05	61	31333.05	5.90E+01	0.00330217	61	61	TRUE
1	NFRAILTAF	230	238	9	HLA-A*02:01	230	61	61	2.80E+05	61	22628.84	3.80E+01	0.00368044	64	61	TRUE
1	KKISNCVAD	343	351	9	HLA-A*02:01	343	61	61	2.86E+05	61	38117.93	8.20E+01	0.00108418	36	61	TRUE
1	YFAATEKSN	88	96	9	HLA-A*02:01	88	61	61	2.92E+05	61	42045.16	9.40E+01	0.00123623	39	61	TRUE
1	PFYSNVTGF	61	69	9	HLA-A*02:01	61	61	61	2.95E+05	61	32973	6.40E+01	0.00023372	13	61	TRUE
1	SGDVVRFPN	310	318	9	HLA-A*02:01	310	61	61	3.07E+05	62	31978.66	6.10E+01	0.0011434	37	61	TRUE
1	GPKLSTDLI	512	520	9	HLA-A*02:01	512	61	61	3.73E+05	64	32025.05	6.10E+01	0.0016278	45	61	TRUE
1	ARDLICAQK	828	836	9	HLA-A*02:01	828	61	61	1.71E+06	82	27992.06	5.00E+01	0.00328625	61	61	TRUE
1	ERDISNVPF	452	460	9	HLA-A*02:01	452	61	61	3.56E+06	89	32045.86	6.10E+01	0.00086976	32	61	TRUE
1	NIDATSTGN	427	435	9	HLA-A*02:01	427	62	62	7.23E+04	44	36508.13	7.60E+01	0.0033358	62	62	TRUE
1	VYADSFVVK	382	390	9	HLA-A*02:01	382	62	62	1.14E+05	50	33936.86	6.70E+01	0.00337676	62	62	TRUE
1	CGPKLSTDL	511	519	9	HLA-A*02:01	511	62	62	1.15E+05	50	32286.7	6.20E+01	0.03184198	98	62	TRUE
1	RPFERDISN	449	457	9	HLA-A*02:01	449	62	62	3.06E+05	62	35468.18	7.20E+01	0.00066039	27	62	TRUE
1	DVDLGDISG	1145	1153	9	HLA-A*02:01	1145	62	62	3.07E+05	62	35003.05	7.10E+01	0.00080575	30	62	TRUE
1	EVFNATKFP	327	335	9	HLA-A*02:01	327	62	62	3.08E+05	62	36410.7	7.60E+01	0.00017104	9.1	62	TRUE
1	NFSQILPDP	783	791	9	HLA-A*02:01	783	62	62	3.08E+05	62	33242.37	6.50E+01	0.00157036	44	62	TRUE
1	VEAEVQIDR	969	977	9	HLA-A*02:01	969	62	62	3.13E+05	62	35588.89	7.30E+01	0.00068612	27	62	TRUE
1	FERDISNVP	451	459	9	HLA-A*02:01	451	62	62	3.14E+05	62	35290.17	7.20E+01	0.00084742	31	62	TRUE
1	TTFMLKYDE	260	268	9	HLA-A*02:01	260	62	62	4.12E+05	65	32422.52	6.20E+01	0.00283139	58	62	TRUE
1	DLQELGKYE	1181	1189	9	HLA-A*02:01	1181	62	62	4.20E+05	66	32334.58	6.20E+01	0.00110459	36	62	TRUE
1	KHLREFVFK	180	188	9	HLA-A*02:01	180	62	62	5.55E+05	69	32426.38	6.20E+01	0.00299709	59	62	TRUE
1	NQKQIANQF	901	909	9	HLA-A*02:01	901	62	62	6.04E+05	70	29420.82	5.40E+01	0.00341193	62	62	TRUE
1	VFQTQAGCL	628	636	9	HLA-A*02:01	628	63	63	4.84E+04	39	32732	6.30E+01	0.01527214	91	63	TRUE
1	VFKNKDGFL	186	194	9	HLA-A*02:01	186	63	63	1.25E+05	51	35311.93	7.20E+01	0.00361327	63	63	TRUE
1	PTLKYFGGF	774	782	9	HLA-A*02:01	774	63	63	1.79E+05	55	32705.44	6.30E+01	0.00609256	75	63	TRUE
1	LPFYSNVTG	60	68	9	HLA-A*02:01	60	63	63	2.15E+05	58	32581.82	6.30E+01	0.00358592	63	63	TRUE
1	SFPQAAPHG	1033	1041	9	HLA-A*02:01	1033	63	63	3.20E+05	63	39736.73	8.70E+01	0.00110129	36	63	TRUE
1	RVDFCGKGY	1021	1029	9	HLA-A*02:01	1021	63	63	3.24E+05	63	29757.6	5.50E+01	0.00484172	70	63	TRUE
1	GAGAALQIP	871	879	9	HLA-A*02:01	871	63	63	3.24E+05	63	32841.25	6.40E+01	0.00153568	44	63	TRUE
1	LHVTYVPSQ	1045	1053	9	HLA-A*02:01	1045	63	63	3.33E+05	63	36891.72	7.70E+01	0.00155632	44	63	TRUE
1	QSVIIINNS	112	120	9	HLA-A*02:01	112	63	63	3.38E+05	63	22566.25	3.80E+01	0.00494083	70	63	TRUE
1	CNFELCDNP	128	136	9	HLA-A*02:01	128	63	63	3.42E+05	63	29648.97	5.40E+01	0.00455932	69	63	TRUE
1	APGQTGVIA	398	406	9	HLA-A*02:01	398	63	63	3.44E+05	63	30789.29	5.80E+01	0.024355665	96	63	TRUE
1	IKNQCVNFN	520	528	9	HLA-A*02:01	520	63	63	3.46E+05	63	34145.33	6.80E+01	0.00029868	16	63	TRUE
1	GNYNYKYRY	434	442	9	HLA-A*02:01	434	63	63	3.49E+05	63	38339.22	8.30E+01	0.00041191	20	63	TRUE
1	RACNFELCD	126	134	9	HLA-A*02:01	126	63	63	3.51E+05	63	32881.07	6.40E+01	0.0015181	44	63	TRUE
1	NARDLICAQ	827	835	9	HLA-A*02:01	827	63	63	5.29E+05	69	32720.67	6.30E+01	0.00239828	54	63	TRUE
1	LTTTSTALG	920	928	9	HLA-A*02:01	920	64	64	6.84E+04	43	32971.21	6.40E+01	0.00501187	71	64	TRUE
1	YPDEIFRSD	42	50	9	HLA-A*02:01	42	64	64	9.55E+04	48	32999.4	6.40E+01	0.00458881	69	64	TRUE
1	QQFGRDVSD	549	557	9	HLA-A*02:01	549	64	64	9.96E+04	48	34129.45	6.80E+01	0.0037853	64	64	TRUE
1	VSGTATAGW	860	868	9	HLA-A*02:01	860	64	64	1.70E+05	55	32971.21	6.40E+01	0.00367705	64	64	TRUE
1	ILSRLDKVE	962	970	9	HLA-A*02:01	962	64	64	2.47E+05	59	33069.45	6.40E+01	0.00490908	70	64	TRUE
1	MSECVLGQS	1011	1019	9	HLA-A*02:01	1011	64	64	3.52E+05	64	25473.74	4.40E+01	0.01976059	94	64	TRUE
1	VRGWVFGST	98	106	9	HLA-A*02:01	98	64	64	3.57E+05	64	29024.65	5.30E+01	0.00984918	85	64	TRUE
1	CFSNVYADS	378	386	9	HLA-A*02:01	378	64	64	3.63E+05	64	31506.42	6.00E+01	0.01163858	88	64	TRUE
1	LPPLLTDDM	843	851	9	HLA-A*02:01	843	64	64	3.65E+05	64	29935.23	5.50E+01	0.00381593	65	64	TRUE
1	IWGTSAAAY	244	252	9	HLA-A*02:01	244	64	64	3.76E+05	64	31084.19	5.80E+01	0.012811501	89	64	TRUE
1	PFQQFGRDV	547	555	9	HLA-A*02:01	547	64	64	4.38E+05	66	33049.07	6.40E+01	0.00324116	61	64	TRUE
1	PDDFMGCVL	413	421	9	HLA-A*02:01	413	64	64	4.66E+05	67	32969.43	6.40E+01	0.00221411	52	64	TRUE
1	IDVVRDLPS	203	211	9	HLA-A*02:01	203	64	64	5.25E+05	69	32888.9	6.40E+01	0.00363413	63	64	TRUE
1	SVKSFEIDK	289	297	9	HLA-A*02:01	289	64	64	6.47E+05	71	32849.79	6.40E+01	0.00029127	15	64	TRUE
1	CNMYICGDS	720	728	9	HLA-A*02:01	720	64	64	8.15E+05	74	32751.83	6.30E+01	0.003748	64	64	TRUE
1	TTTGIGYQP	485	493	9	HLA-A*02:01	485	65	65	1.47E+05	53	33655.68	6.60E+01	0.00393641	65	65	TRUE
1	EIFRSDTLY	45	53	9	HLA-A*02:01	45	65	65	3.79E+05	65	32632.98	6.30E+01	0.01191242	88	65	TRUE
1	LNAPATVCG	504	512	9	HLA-A*02:01	504	65	65	3.82E+05	65	38123.7	8.20E+01	0.00074439	29	65	TRUE

seq #	peptide	start	end	peptide length	allele	peptide index	median binding	consensus perc	smm IC50	smm percentile	ann IC50	ann percentile	comblib_sidney	comblib_sidney	legacy consens	percentiles equal
1	DINARDLIC	825	833	9	HLA-A*02:01	825	65	65	3.92E+05	65	32828.11	6.40E+01	0.010197648	85	65	TRUE
1	EAEVQIDRL	970	978	9	HLA-A*02:01	970	65	65	3.96E+05	65	31976.93	6.10E+01	0.012195511	88	65	TRUE
1	MKQYGECLG	816	824	9	HLA-A*02:01	816	65	65	3.98E+05	65	39183.83	8.60E+01	0.00316228	60	65	TRUE
1	CSCGSCCKF	1230	1238	9	HLA-A*02:01	1230	65	65	3.99E+05	65	26097.54	4.60E+01	0.011086641	87	65	TRUE
1	CTQLNRALS	742	750	9	HLA-A*02:01	742	65	65	4.01E+05	65	30813.62	5.80E+01	0.00816582	81	65	TRUE
1	YDENGTITD	266	274	9	HLA-A*02:01	266	65	65	4.03E+05	65	39298.04	8.60E+01	0.00030796	16	65	TRUE
1	KVEAEVQID	968	976	9	HLA-A*02:01	968	65	65	4.07E+05	65	37782.04	8.10E+01	0.00141286	42	65	TRUE
1	CTDVSTAIH	603	611	9	HLA-A*02:01	603	65	65	4.37E+05	66	31564.1	6.00E+01	0.00394639	65	65	TRUE
1	YTQHTSSMR	30	38	9	HLA-A*02:01	30	66	66	5.13E+04	40	33660.41	6.60E+01	0.019971009	94	66	TRUE
1	IAAEQDRNT	752	760	9	HLA-A*02:01	752	66	66	6.58E+04	43	33599.64	6.60E+01	0.016641789	92	66	TRUE
1	GFMKQYGEC	814	822	9	HLA-A*02:01	814	66	66	8.79E+04	46	39736.73	8.70E+01	0.00400129	66	66	TRUE
1	QTGVIADYN	401	409	9	HLA-A*02:01	401	66	66	4.07E+05	65	33446.57	6.60E+01	0.00619726	76	66	TRUE
1	KFPSVYAWE	333	341	9	HLA-A*02:01	333	66	66	4.25E+05	66	32617.81	6.30E+01	0.00503385	71	66	TRUE
1	LPSGFNTLK	209	217	9	HLA-A*02:01	209	66	66	4.30E+05	66	35029.59	7.10E+01	0.00389852	65	66	TRUE
1	KDGFLYVYK	190	198	9	HLA-A*02:01	190	66	66	4.44E+05	66	29404.59	5.40E+01	0.01344931	90	66	TRUE
1	VVRFPNITN	313	321	9	HLA-A*02:01	313	66	66	4.44E+05	66	33879.26	6.70E+01	0.00228139	53	66	TRUE
1	FNGIGVTQN	888	896	9	HLA-A*02:01	888	66	66	4.45E+05	66	35277.95	7.20E+01	0.00138995	42	66	TRUE
1	GFYTTTGIG	482	490	9	HLA-A*02:01	482	66	66	4.45E+05	66	36178.62	7.50E+01	0.00227457	53	66	TRUE
1	APATVCGPK	506	514	9	HLA-A*02:01	506	66	66	8.36E+05	74	33500.89	6.60E+01	0.00242605	54	66	TRUE
1	VFAQVKQMY	763	771	9	HLA-A*02:01	763	66	66	8.77E+05	75	30172.6	5.60E+01	0.00411055	66	66	TRUE
1	TGVIADYNY	402	410	9	HLA-A*02:01	402	66	66	1.77E+06	82	32654.88	6.30E+01	0.00402717	66	66	TRUE
1	LNESLIDLQ	1175	1183	9	HLA-A*02:01	1175	66	66	3.71E+06	89	33458.87	6.60E+01	0.00195209	49	66	TRUE
1	HTSPDVDLG	1141	1149	9	HLA-A*02:01	1141	67	67	8.51E+04	46	33753.4	6.70E+01	0.00435712	68	67	TRUE
1	RDPKTSEIL	563	571	9	HLA-A*02:01	563	67	67	1.14E+05	50	33810.05	6.70E+01	0.019787913	94	67	TRUE
1	DVNCTDVST	600	608	9	HLA-A*02:01	600	67	67	1.50E+05	53	34025.48	6.70E+01	0.028131961	98	67	TRUE
1	RSDTLYLTQ	48	56	9	HLA-A*02:01	48	67	67	4.46E+05	67	28777.91	5.20E+01	0.016768723	92	67	TRUE
1	QESLTTTST	917	925	9	HLA-A*02:01	917	67	67	4.48E+05	67	37456.03	7.90E+01	0.00193197	49	67	TRUE
1	YENQKQIAN	899	907	9	HLA-A*02:01	899	67	67	4.51E+05	67	39925.05	8.80E+01	0.00127997	40	67	TRUE
1	AYFPREGVF	1069	1077	9	HLA-A*02:01	1069	67	67	4.60E+05	67	33966.61	6.70E+01	0.00077304	30	67	TRUE
1	ERNFTTAPA	1054	1062	9	HLA-A*02:01	1054	67	67	4.68E+05	67	34164.18	6.80E+01	0.00066589	27	67	TRUE
1	ATVCGPKLS	508	516	9	HLA-A*02:01	508	68	68	1.21E+05	51	34335.76	6.80E+01	0.02471724	96	68	TRUE
1	TQHTSSMRG	31	39	9	HLA-A*02:01	31	68	68	1.22E+05	51	34306.04	6.80E+01	0.018535316	94	68	TRUE
1	SYECDIPIG	645	653	9	HLA-A*02:01	645	68	68	3.56E+05	64	37442.64	7.90E+01	0.00452064	68	68	TRUE
1	SGNCDVVIG	1105	1113	9	HLA-A*02:01	1105	68	68	3.79E+05	65	34084.8	6.80E+01	0.020941125	95	68	TRUE
1	TTTSTALGK	921	929	9	HLA-A*02:01	921	68	68	4.97E+05	68	35298.57	7.20E+01	0.00127732	39	68	TRUE
1	NTRNIDATS	424	432	9	HLA-A*02:01	424	68	68	5.14E+05	68	33778.62	6.70E+01	0.00866962	82	68	TRUE
1	FHTINHTFG	69	77	9	HLA-A*02:01	69	68	68	5.18E+05	68	37507.51	8.00E+01	0.00093411	33	68	TRUE
1	WPLNDYGFY	476	484	9	HLA-A*02:01	476	68	68	6.70E+05	72	34113.21	6.80E+01	0.00207778	51	68	TRUE
1	TREVFAQVK	760	768	9	HLA-A*02:01	760	68	68	3.69E+07	99	34335.01	6.80E+01	0.00412762	66	68	TRUE
1	VQAPNYTQH	25	33	9	HLA-A*02:01	25	69	69	1.09E+05	49	37120.35	7.80E+01	0.0046366	69	69	TRUE
1	GNNVFQTQA	625	633	9	HLA-A*02:01	625	69	69	1.40E+05	52	34511.19	6.90E+01	0.010834279	86	69	TRUE
1	GSDLDRCTT	13	21	9	HLA-A*02:01	13	69	69	2.35E+05	59	34475.35	6.90E+01	0.00992659	85	69	TRUE
1	TSGSDLDRC	11	19	9	HLA-A*02:01	11	69	69	3.24E+05	63	37324.12	7.90E+01	0.00461424	69	69	TRUE
1	IQKEIDRLN	1161	1169	9	HLA-A*02:01	1161	69	69	3.30E+05	63	34496.25	6.90E+01	0.013464803	90	69	TRUE
1	DEIFRSDTL	44	52	9	HLA-A*02:01	44	69	69	3.32E+05	63	34545.54	6.90E+01	0.011277167	87	69	TRUE
1	NSTFFSTFK	357	365	9	HLA-A*02:01	357	69	69	5.24E+05	69	30580.14	5.70E+01	0.0078001	80	69	TRUE
1	TAGWTFGAG	865	873	9	HLA-A*02:01	865	69	69	5.26E+05	69	33791.04	6.70E+01	0.014066952	90	69	TRUE
1	SGTATAGWT	861	869	9	HLA-A*02:01	861	69	69	5.30E+05	69	39380.62	8.60E+01	0.0024322	54	69	TRUE
1	HEGKAYFPR	1065	1073	9	HLA-A*02:01	1065	69	69	5.36E+05	69	36465.12	7.60E+01	0.00184332	48	69	TRUE
1	TSQKSIVAY	669	677	9	HLA-A*02:01	669	69	69	5.36E+05	69	29996.51	5.50E+01	0.00581835	74	69	TRUE
1	AGFMKQYGE	813	821	9	HLA-A*02:01	813	69	69	5.46E+05	69	36696.27	7.70E+01	0.00072946	29	69	TRUE
1	EHVDTSYEC	640	648	9	HLA-A*02:01	640	69	69	5.46E+05	69	41210.14	9.20E+01	0.00097432	34	69	TRUE
1	LNRALSGIA	745	753	9	HLA-A*02:01	745	69	69	5.51E+05	69	33798.71	6.70E+01	0.01544543	92	69	TRUE
1	YGVSATKLN	367	375	9	HLA-A*02:01	367	69	69	5.55E+05	69	40452.95	9.00E+01	0.0007034	28	69	TRUE
1	LCCMTSCCS	1216	1224	9	HLA-A*02:01	1216	69	69	5.61E+05	69	26320.99	4.60E+01	0.03948208	99	69	TRUE
1	NVLYENQKQ	896	904	9	HLA-A*02:01	896	69	69	5.64E+05	69	41995.6	9.40E+01	0.00142135	42	69	TRUE
1	VSLLRSTSQ	663	671	9	HLA-A*02:01	663	69	69	5.85E+05	70	34169.36	6.80E+01	0.00463981	69	69	TRUE
1	IDLQELGKY	1180	1188	9	HLA-A*02:01	1180	69	69	1.59E+06	81	34397.11	6.90E+01	0.00385834	65	69	TRUE
1	SATKLNDLC	370	378	9	HLA-A*02:01	370	70	70	1.29E+05	51	34697.64	7.00E+01	0.00782528	80	70	TRUE
1	VLGQSKRVD	1015	1023	9	HLA-A*02:01	1015	70	70	2.09E+05	57	39768.55	8.80E+01	0.00475445	70	70	TRUE
1	TVSLLRSTS	662	670	9	HLA-A*02:01	662	70	70	2.14E+05	57	34758.52	7.00E+01	0.0060256	75	70	TRUE
1	GFNTLKPIF	212	220	9	HLA-A*02:01	212	70	70	3.19E+05	63	34840.97	7.00E+01	0.012103194	88	70	TRUE
1	QTSNFRVVP	301	309	9	HLA-A*02:01	301	70	70	3.52E+05	64	34763.78	7.00E+01	0.00955213	84	70	TRUE
1	PFAMQMAYR	879	887	9	HLA-A*02:01	879	70	70	4.58E+05	67	34814.22	7.00E+01	0.0074319	79	70	TRUE
1	MYICGDSTE	722	730	9	HLA-A*02:01	722	70	70	4.88E+05	68	40210.32	8.90E+01	0.00483281	70	70	TRUE
1	LKPTTFMLK	257	265	9	HLA-A*02:01	257	70	70	5.72E+05	70	31007.26	5.80E+01	0.026079544	97	70	TRUE
1	TSCCSCLKG	1220	1228	9	HLA-A*02:01	1220	70	70	5.84E+05	70	32027.82	6.10E+01	0.020361042	95	70	TRUE
1	GYQPIDVVR	199	207	9	HLA-A*02:01	199	70	70	5.86E+05	70	34494.39	6.90E+01	0.00761377	80	70	TRUE
1	ISNVPFSPD	455	463	9	HLA-A*02:01	455	70	70	5.90E+05	70	35527.32	7.30E+01	0.00267855	57	70	TRUE
1	VIPFKDGIY	80	88	9	HLA-A*02:01	80	70	70	6.08E+05	70	33640.38	6.60E+01	0.06324119	100	70	TRUE
1	PPLLTDDMI	844	852	9	HLA-A*02:01	844	70	70	7.55E+05	73	34833.8	7.00E+01	0.00410582	66	70	TRUE
1	PSSKRFQPF	540	548	9	HLA-A*02:01	540	70	70	1.56E+06	81	34641.36	7.00E+01	0.00121227	38	70	TRUE
1	TSEILDISP	567	575	9	HLA-A*02:01	567	70	70	1.63E+06	81	31543.95	6.00E+01	0.00486295	70	70	TRUE
1	FNKAISQIQ	909	917	9	HLA-A*02:01	909	70	70	3.77E+06	89	34883.6	7.00E+01	0.00063606	26	70	TRUE
1	GWTFGAGAA	867	875	9	HLA-A*02:01	867	71	71	7.38E+04	44	34945.54	7.10E+01	0.00738924	79	71	TRUE
1	TINHTFGNP	71	79	9	HLA-A*02:01	71	71	71	1.95E+05	56	35173.16	7.10E+01	0.038556713	99	71	TRUE
1	DVVRFPNIT	312	320	9	HLA-A*02:01	312	71	71	2.01E+05	57	34972.39	7.10E+01	0.019373139	94	71	TRUE
1	DVSTAIHAD	605	613	9	HLA-A*02:01	605	71	71	2.44E+05	59	40090.41	8.90E+01	0.00514399	71	71	TRUE
1	VDCNMYICG	718	726	9	HLA-A*02:01	718	71	71	6.11E+05	71	35133.21	7.10E+01	0.00036174	18	71	TRUE
1	GFLYVYKGY	192	200	9	HLA-A*02:01	192	71	71	6.24E+05	71	26952.42	4.70E+01	0.016745573	92	71	TRUE
1	SGINASVVN	1152	1160	9	HLA-A*02:01	1152	71	71	6.25E+05	71	39814.61	8.80E+01	0.00274284	57	71	TRUE
1	KPTTFMLKY	258	266	9	HLA-A*02:01	258	71	71	6.44E+05	71	28725.66	5.20E+01	0.02111543	95	71	TRUE
1	PKTSEILDI	565	573	9	HLA-A*02:01	565	71	71	6.50E+05	71	36750.69	7.70E+01	0.00094363	33	71	TRUE
1	DAVDCSQNP	274	282	9	HLA-A*02:01	274	71	71	6.55E+05	71	41421.14	9.30E+01	0.00099426	34	71	TRUE
1	EVQIDRLIT	972	980	9	HLA-A*02:01	972	71	71	6.59E+05	71	34180.82	6.80E+01	0.04786301	100	71	TRUE
1	DRCTTFDDV	17	25	9	HLA-A*02:01	17	71	71	6.79E+05	72	32729.51	6.30E+01	0.0051357	71	71	TRUE
1	GEVFNATKF	326	334	9	HLA-A*02:01	326	71	71	1.36E+06	79	33991.99	6.70E+01	0.00509331	71	71	TRUE
1	TGNNVFQTQ	624	632	9	HLA-A*02:01	624	71	71	2.91E+06	87	35004.95	7.10E+01	0.00211203	51	71	TRUE
1	DGIYFAATE	85	93	9	HLA-A*02:01	85	71	71	7.76E+06	94	35226.09	7.10E+01	0.00298676	59	71	TRUE
1	KYFKNHTSP	1136	1144	9	HLA-A*02:01	1136	72	72	1.63E+05	54	41172.26	9.20E+01	0.00523962	72	72	TRUE
1	NTVYDPLQP	1117	1125	9	HLA-A*02:01	1117	72	72	2.00E+05	57	35261.55	7.20E+01	0.023019718	96	72	TRUE
1	IYSTGNNVF	621	629	9	HLA-A*02:01	621	72	72	2.08E+05	57	35427.51	7.20E+01	0.00657961	77	72	TRUE
1	LTSGSDLDR	10	18	9	HLA-A*02:01	10	72	72	2.67E+05	60	35389.2	7.20E+01	0.019787913	94	72	TRUE
1	SNVPFSPDG	456	464	9	HLA-A*02:01	456	72	72	6.75E+05	72	39779.32	8.80E+01	0.00164361	46	72	TRUE
1	IGVTQNVLY	891	899	9	HLA-A*02:01	891	72	72	6.75E+05	72	31081.5	5.80E+01	0.00931108	84	72	TRUE
1	TQNVLYENQ	894	902	9	HLA-A*02:01	894	72	72	6.81E+05	72	31997.7	6.10E+01	0.01562068	92	72	TRUE
1	DENGTITDA	267	275	9	HLA-A*02:01	267	72	72	6.95E+05	72	37248.7	7.90E+01	0.00515466	71	72	TRUE
1	ATKLNDLCF	371	379	9	HLA-A*02:01	371	72	72	7.08E+05	72	26988.6	4.80E+01	0.01051962	86	72	TRUE
1	NPFFAVSKP	135	143	9	HLA-A*02:01	135	72	72	7.52E+05	73	35250.09	7.20E+01	0.0012494	39	72	TRUE
1	DAFSLDVSE	166	174	9	HLA-A*02:01	166	72	72	1.84E+06	83	35367.77	7.20E+01	0.0015574	44	72	TRUE
1	AGCLIGAEH	633	641	9	HLA-A*02:01	633	72	72	2.57E+06	86	30100.86	5.60E+01	0.00536043	72	72	TRUE
1	IDRLNEVAK	1165	1173	9	HLA-A*02:01	1165	72	72	1.01E+07	95	35252	7.20E+01	0.00307397	60	72	TRUE
1	LKPTKRSFI	792	800	9	HLA-A*02:01	792	73	73	9.55E+04	48	38900.8	8.50E+01	0.00547016	73	73	TRUE
1	AFSPAQDIW	237	245	9	HLA-A*02:01	237	73	73	2.86E+05	61	36447.77	7.60E+01	0.00547772	73	73	TRUE
1	PAICHEGKA	1061	1069	9	HLA-A*02:01	1061	73	73	2.87E+05	61	43186.28	9.70E+01	0.0054325	73	73	TRUE
1	LAWNTRNID	421	429	9	HLA-A*02:01	421	73	73	3.02E+05	62	37362.11	7.90E+01	0.00561436	73	73	TRUE
1	TSPDVDLGD	1142	1150	9	HLA-A*02:01	1142	73	73	4.46E+05	67	37899.55	8.10E+01	0.00559629	73	73	TRUE
1	LNCYWPLND	472	480	9	HLA-A*02:01	472	73	73	7.08E+05	72	35692.62	7.30E+01	0.016861647	92	73	TRUE
1	IRAAEIRAS	995	1003	9	HLA-A*02:01	995	73	73	7.47E+05	73	37828.68	8.10E+01	0.001508	43	73	TRUE
1	NDLCFSNVY	375	383	9	HLA-A*02:01	375	73	73	7.47E+05	73	28828.1	5.20E+01	0.0107004	86	73	TRUE
1	KRVDFCGKG	1020	1028	9	HLA-A*02:01	1020	73	73	7.62E+05	73	38415.62	8.30E+01	0.0038054	64	73	TRUE
1	NFNGLTGTG	528	536	9	HLA-A*02:01	528	73	73	7.78E+05	73	38048.69	8.20E+01	0.00316884	60	73	TRUE
1	LNTLVKQLS	941	949	9	HLA-A*02:01	941	73	73	2.60E+06	86	35561.17	7.30E+01	0.0052942	72	73	TRUE
1	PREGVFVFN	1072	1080	9	HLA-A*02:01	1072	73	73	1.60E+07	97	34676.61	7.00E+01	0.00543751	73	73	TRUE
1	VVPSGDVVR	307	315	9	HLA-A*02:01	307	74	74	1.01E+05	48	35980.7	7.40E+01	0.00605899	75	74	TRUE
1	SDAFSLDVS	165	173	9	HLA-A*02:01	165	74	74	3.31E+05	63	36070.75	7.40E+01	0.00934114	84	74	TRUE
1	ADQLTPAWR	612	620	9	HLA-A*02:01	612	74	74	7.95E+05	74	34504.09	6.90E+01	0.00655843	77	74	TRUE
1	VSKPMGTQT	140	148	9	HLA-A*02:01	140	74	74	7.96E+05	74	37639.65	8.00E+01	0.00337132	62	74	TRUE
1	VPSQERNFT	1050	1058	9	HLA-A*02:01	1050	74	74	8.00E+05	74	41725.66	9.30E+01	0.00489328	70	74	TRUE
1	TSSMRGVYY	34	42	9	HLA-A*02:01	34	74	74	8.30E+05	74	28010.53	5.00E+01	0.014955468	91	74	TRUE
1	PIDVVRDLP	202	210	9	HLA-A*02:01	202	74	74	8.38E+05	74	35611.23	7.30E+01	0.01981071	94	74	TRUE
1	VFGSTMNNK	102	110	9	HLA-A*02:01	102	74	74	8.45E+05	74	35957.34	7.40E+01	0.00517845	72	74	TRUE

seq #	peptide	start	end	peptide length	allele	peptide index	median binding	consensus perc	smm IC50	smm percentile	ann IC50	ann percentile	comblib_sidney	comblib_sidney	legacy consens	percentiles equal	
1	LCPFGEVFN	322	330	9	HLA-A*02:01	322	74	74	8.45E+05	74	36267.2	7.50E+01	0.00226778	53	74	TRUE	
1	LSTDLIKNQ	515	523	9	HLA-A*02:01	515	74	74	8.45E+05	74	36267.2	7.50E+01	0.00366016	64	74	TRUE	
1	GCVLAWNTR	418	426	9	HLA-A*02:01	418	74	74	2.19E+06	85	26795.69	4.70E+01	0.00585868	74	74	TRUE	
1	ASVVNIQKE	1156	1164	9	HLA-A*02:01	1156	74	74	2.96E+06	87	35861.37	7.40E+01	0.00174904	47	74	TRUE	
1	EKSNVVRGW	93	101	9	HLA-A*02:01	93	74	74	3.67E+06	89	35969.43	7.40E+01	0.00359501	63	74	TRUE	
1	PTNFSISIT	697	705	9	HLA-A*02:01	697	75	75	2.90E+05	61	36209.17	7.50E+01	0.00619156	76	75	TRUE	
1	RDVSDFTDS	553	561	9	HLA-A*02:01	553	75	75	5.82E+05	70	40982.93	9.10E+01	0.00611505	75	75	TRUE	
1	NATKFPSVY	330	338	9	HLA-A*02:01	330	75	75	8.53E+05	75	34201.53	6.80E+01	0.0099816	85	75	TRUE	
1	VYKGYQPID	196	204	9	HLA-A*02:01	196	75	75	8.55E+05	75	45094.44	1.00E+02	0.00330598	61	75	TRUE	
1	VKQLSSNFG	945	953	9	HLA-A*02:01	945	75	75	8.67E+05	75	37601.39	8.00E+01	0.00337287	62	75	TRUE	
1	TFEYISDAF	160	168	9	HLA-A*02:01	160	75	75	8.71E+05	75	29355.3	5.40E+01	0.03809781	99	75	TRUE	
1	LPDPLKPTK	788	796	9	HLA-A*02:01	788	75	75	8.81E+05	75	39216.92	8.60E+01	0.00129688	40	75	TRUE	
1	TFGAGAALQ	869	877	9	HLA-A*02:01	869	75	75	8.83E+05	75	36119.96	7.50E+01	0.00170451	46	75	TRUE	
1	MPVSMAKTS	709	717	9	HLA-A*02:01	709	75	75	8.96E+05	75	38075.89	8.20E+01	0.00029349	15	75	TRUE	
1	TDNTFVSGN	1099	1107	9	HLA-A*02:01	1099	75	75	9.02E+05	75	39556.99	8.70E+01	0.00408884	66	75	TRUE	
1	SNVVRGWVF	95	103	9	HLA-A*02:01	95	75	75	9.14E+05	75	23953.54	4.10E+01	0.01095973	87	75	TRUE	
1	GKLQDVVNQ	928	936	9	HLA-A*02:01	928	75	75	9.16E+05	75	36571.8	7.60E+01	0.00279705	58	75	TRUE	
1	SCGSCCKFD	1231	1239	9	HLA-A*02:01	1231	75	75	1.47E+06	80	36094.95	7.50E+01	0.00061165	25	75	TRUE	
1	ECLGDINAR	821	829	9	HLA-A*02:01	821	75	75	1.66E+06	82	36322.96	7.50E+01	0.00191337	49	75	TRUE	
1	QELGKYEQY	1183	1191	9	HLA-A*02:01	1183	75	75	3.43E+06	88	35596.19	7.30E+01	0.00601174	75	75	TRUE	
1	KGACSCGSC	1227	1235	9	HLA-A*02:01	1227	76	76	2.52E+05	59	36522.36	7.60E+01	0.011649305	88	76	TRUE	
1	ITGRLQSLQ	979	987	9	HLA-A*02:01	979	76	76	5.12E+05	68	37757.54	8.00E+01	0.00619013	76	76	TRUE	
1	KYEQYIKWP	1187	1195	9	HLA-A*02:01	1187	76	76	5.31E+05	69	36506.96	7.60E+01	0.00618443	76	76	TRUE	
1	QDVNCTDVS	599	607	9	HLA-A*02:01	599	76	76	9.31E+05	76	41627.37	9.30E+01	0.00056416	24	76	TRUE	
1	ANLAATKMS	1004	1012	9	HLA-A*02:01	1004	76	76	9.31E+05	76	40136.84	8.90E+01	0.00053064	23	76	TRUE	
1	GDDVRQIAP	391	399	9	HLA-A*02:01	391	76	76	9.33E+05	76	39282.3	8.60E+01	0.00130768	40	76	TRUE	
1	RFNGIGVTQ	887	895	9	HLA-A*02:01	887	76	76	9.38E+05	76	30166.72	5.60E+01	0.01620317	92	76	TRUE	
1	DVQAPNYTQ	24	32	9	HLA-A*02:01	24	76	76	9.38E+05	76	40000.72	8.80E+01	0.00055195	24	76	TRUE	
1	AICHEGKAY	1062	1070	9	HLA-A*02:01	1062	76	76	9.42E+05	76	34617.76	6.90E+01	0.06309573	100	76	TRUE	
1	KNLNESLID	1173	1181	9	HLA-A*02:01	1173	76	76	9.60E+05	76	40606.43	9.00E+01	0.00477419	70	76	TRUE	
1	PSGDVVRFP	309	317	9	HLA-A*02:01	309	76	76	1.00E+06	76	37075.39	7.80E+01	0.00136364	41	76	TRUE	
1	EGVFVFNGT	1074	1082	9	HLA-A*02:01	1074	76	76	1.02E+06	76	27694.7	4.90E+01	0.0097949	85	76	TRUE	
1	DIPIGAGIC	649	657	9	HLA-A*02:01	649	76	76	1.02E+06	76	34698	7.00E+01	0.02006781	94	76	TRUE	
1	APAICHEGK	1060	1068	9	HLA-A*02:01	1060	76	76	2.09E+06	84	36488.79	7.60E+01	0.00184035	48	76	TRUE	
1	ECDIPIGAG	647	655	9	HLA-A*02:01	647	76	76	1.56E+07	97	36205.64	7.50E+01	0.0062431	76	76	TRUE	
1	VAVLYQDVN	594	602	9	HLA-A*02:01	594	77	77	3.10E+05	62	38756.29	8.40E+01	0.00665886	77	77	TRUE	
1	IADYNYKLP	405	413	9	HLA-A*02:01	405	77	77	3.55E+05	64	36641.5	7.70E+01	0.017151417	93	77	TRUE	
1	EKSGNFKHL	174	182	9	HLA-A*02:01	174	77	77	6.34E+05	71	36853.81	7.70E+01	0.01555966	92	77	TRUE	
1	PLKPTKRSF	791	799	9	HLA-A*02:01	791	77	77	1.04E+06	77	36857.81	7.70E+01	0.00232702	53	77	TRUE	
1	PVLKGVKLH	1245	1253	9	HLA-A*02:01	1245	77	77	1.07E+06	77	39673.57	8.70E+01	0.00359749	63	77	TRUE	
1	TPTLKYFGG	773	781	9	HLA-A*02:01	773	77	77	1.10E+06	77	36921.68	7.80E+01	0.00506524	71	77	TRUE	
1	ERKKISNCV	341	349	9	HLA-A*02:01	341	77	77	1.31E+06	79	36918.09	7.70E+01	0.00352696	63	77	TRUE	
1	PPALNCYWP	469	477	9	HLA-A*02:01	469	77	77	1.42E+06	80	36731.61	7.70E+01	0.00069263	28	77	TRUE	
1	LSGIAAEQD	749	757	9	HLA-A*02:01	749	77	77	2.25E+06	85	36737.96	7.70E+01	0.00496478	71	77	TRUE	
1	NFTTAPAIC	1056	1064	9	HLA-A*02:01	1056	78	78	4.41E+05	66	37194.32	7.80E+01	0.029661971	98	78	TRUE	
1	PAWRIYSTG	617	625	9	HLA-A*02:01	617	78	78	4.96E+05	68	37058.54	7.80E+01	0.00845279	82	78	TRUE	
1	YDPLQPELD	1120	1128	9	HLA-A*02:01	1120	78	78	6.50E+05	71	38947.56	8.50E+01	0.00691035	78	78	TRUE	
1	DYSVLYNST	351	359	9	HLA-A*02:01	351	78	78	9.06E+05	75	36987.65	7.80E+01	0.022356304	96	78	TRUE	
1	TDSVRDPKT	559	567	9	HLA-A*02:01	559	78	78	9.10E+05	75	43854	9.80E+01	0.00706968	78	78	TRUE	
1	NHTSPDVDL	1140	1148	9	HLA-A*02:01	1140	78	78	9.75E+05	76	37318.09	7.90E+01	0.00710232	78	78	TRUE	
1	LTPSSKRFQ	538	546	9	HLA-A*02:01	538	78	78	1.13E+06	78	42578.95	9.60E+01	0.00536661	72	78	TRUE	
1	TSTGNYNYK	431	439	9	HLA-A*02:01	431	78	78	1.14E+06	78	40422.3	9.00E+01	0.00556032	73	78	TRUE	
1	ADAGFMKQY	811	819	9	HLA-A*02:01	811	78	78	1.15E+06	78	33196.35	6.50E+01	0.010125116	85	78	TRUE	
1	DYGFYTTTG	480	488	9	HLA-A*02:01	480	78	78	1.16E+06	78	40790.5	9.10E+01	0.00586678	75	78	TRUE	
1	DRNTREVFA	757	765	9	HLA-A*02:01	757	78	78	1.16E+06	78	39509.08	8.70E+01	0.00257395	56	78	TRUE	
1	TQQLIRAAE	991	999	9	HLA-A*02:01	991	78	78	1.18E+06	78	31400.25	5.90E+01	0.00704693	78	78	TRUE	
1	QFGRDVSDF	550	558	9	HLA-A*02:01	550	78	78	1.18E+06	78	36760.24	7.70E+01	0.010836774	86	78	TRUE	
1	DDVQAPNYT	23	31	9	HLA-A*02:01	23	78	78	1.19E+06	78	41454.76	9.30E+01	0.00278997	57	78	TRUE	
1	NFNFNGLTG	526	534	9	HLA-A*02:01	526	78	78	1.20E+06	78	34432.86	6.90E+01	0.01999401	94	78	TRUE	
1	QSLQTYVTQ	984	992	9	HLA-A*02:01	984	78	78	1.20E+06	78	30072.86	5.60E+01	0.00830807	81	78	TRUE	
1	VCGPKLSTD	510	518	9	HLA-A*02:01	510	78	78	1.22E+06	78	38796.97	8.40E+01	0.00320258	61	78	TRUE	
1	CHEGKAYFP	1064	1072	9	HLA-A*02:01	1064	78	78	1.71E+06	82	36956.85	7.80E+01	0.00058412	25	78	TRUE	
1	DCNMYICGD	719	727	9	HLA-A*02:01	719	78	78	1.85E+06	83	36068.01	7.40E+01	0.00688177	78	78	TRUE	
1	SPCSFGGVS	574	582	9	HLA-A*02:01	574	78	78	2.50E+06	86	37109.91	7.80E+01	0.00586949	75	78	TRUE	
1	KYDENGTIT	265	273	9	HLA-A*02:01	265	79	79	6.05E+04	42	37386.38	7.90E+01	0.00901986	83	79	TRUE	
1	VKQMYKTPT	767	775	9	HLA-A*02:01	767	79	79	9.21E+04	47	37668.16	8.00E+01	0.00712853	79	79	TRUE	
1	KSGNFKHLR	175	183	9	HLA-A*02:01	175	79	79	2.37E+05	59	37600.97	8.00E+01	0.00713181	79	79	TRUE	
1	STALGKLQD	924	932	9	HLA-A*02:01	924	79	79	3.40E+05	63	38810.84	8.40E+01	0.0074319	79	79	TRUE	
1	SPDGKPCTP	461	469	9	HLA-A*02:01	461	79	79	5.72E+05	70	38724.01	8.40E+01	0.00714003	79	79	TRUE	
1	SDTLYLTQD	49	57	9	HLA-A*02:01	49	79	79	5.84E+05	70	37884.79	8.10E+01	0.00733669	79	79	TRUE	
1	AVSKPMGTQ	139	147	9	HLA-A*02:01	139	79	79	6.84E+05	72	37425.24	7.90E+01	0.020878534	95	79	TRUE	
1	VSDFTDSVR	555	563	9	HLA-A*02:01	555	79	79	7.10E+05	72	37451.97	7.90E+01	0.00845668	82	79	TRUE	
1	GAEHVDTSY	638	646	9	HLA-A*02:01	638	79	79	1.29E+06	79	34254.86	6.80E+01	0.04075679	100	79	TRUE	
1	NFSISITTE	699	707	9	HLA-A*02:01	699	79	79	1.54E+06	81	32784.1	6.40E+01	0.00742848	79	79	TRUE	
1	HGKLRPFER	445	453	9	HLA-A*02:01	445	79	79	2.41E+06	85	37287	7.90E+01	0.00370339	64	79	TRUE	
1	LAELKCSVK	283	291	9	HLA-A*02:01	283	79	79	2.47E+06	86	31732.22	6.00E+01	0.00733669	79	79	TRUE	
1	DVRQIAPGQ	393	401	9	HLA-A*02:01	393	79	79	3.53E+06	88	37288.62	7.90E+01	0.00091812	33	79	TRUE	
1	RKKISNCVA	342	350	9	HLA-A*02:01	342	80	80	5.04E+05	68	37609.51	8.00E+01	0.011428783	87	80	TRUE	
1	NNKSQSVII	108	116	9	HLA-A*02:01	108	80	80	6.84E+05	72	37599.35	8.00E+01	0.0084879	82	80	TRUE	
1	EELDKYFKN	1132	1140	9	HLA-A*02:01	1132	80	80	1.38E+06	80	43467.57	9.80E+01	0.00174663	47	80	TRUE	
1	SRLDKVEAE	964	972	9	HLA-A*02:01	964	80	80	1.41E+06	80	38777.26	8.40E+01	0.00046817	21	80	TRUE	
1	NPLAELKCS	281	289	9	HLA-A*02:01	281	80	80	1.45E+06	80	39244.93	8.60E+01	0.00302413	59	80	TRUE	
1	EPVLKGVKL	1244	1252	9	HLA-A*02:01	1244	80	80	1.46E+06	80	31296.46	5.90E+01	0.012067019	88	80	TRUE	
1	DKYFKNHTS	1135	1143	9	HLA-A*02:01	1135	80	80	1.49E+06	80	42652.28	9.60E+01	0.00166802	46	80	TRUE	
1	IDKGIYQTS	295	303	9	HLA-A*02:01	295	80	80	1.94E+06	83	36593.56	7.60E+01	0.00784152	80	80	TRUE	
1	RNTREVFAQ	758	766	9	HLA-A*02:01	758	80	80	2.02E+06	84	37617.25	8.00E+01	0.00454465	69	80	TRUE	
1	MYKTPTLKY	770	778	9	HLA-A*02:01	770	80	80	2.28E+06	85	34974.67	7.10E+01	0.00748514	80	80	TRUE	
1	VSTAIHADQ	606	614	9	HLA-A*02:01	606	80	80	2.61E+06	86	37750.99	8.00E+01	0.00770016	80	80	TRUE	
1	PYRVVVLSF	493	501	9	HLA-A*02:01	493	80	80	2.76E+06	87	24115.02	4.10E+01	0.0076913	80	80	TRUE	
1	SPQIITTDN	1093	1101	9	HLA-A*02:01	1093	80	80	2.99E+06	87	37627.03	8.00E+01	0.00471846	69	80	TRUE	
1	SECVLGQSK	1012	1020	9	HLA-A*02:01	1012	80	80	6.97E+06	93	34546.66	6.90E+01	0.00755614	80	80	TRUE	
1	DNPFFAVSK	134	142	9	HLA-A*02:01	134	80	80	1.26E+07	96	34192.66	6.80E+01	0.00755614	80	80	TRUE	
1	LYVYKGYQP	194	202	9	HLA-A*02:01	194	81	81	3.16E+05	62	38323.45	8.30E+01	0.00822811	81	81	TRUE	
1	ACSCGSCCK	1229	1237	9	HLA-A*02:01	1229	81	81	9.33E+05	76	37886.03	8.10E+01	0.04137138	100	81	TRUE	
1	PAQDIWGTS	240	248	9	HLA-A*02:01	240	81	81	1.04E+06	77	39609.68	8.70E+01	0.00826038	81	81	TRUE	
1	GRDVSDFTD	552	560	9	HLA-A*02:01	552	81	81	1.52E+06	81	40193.78	8.90E+01	0.00640472	76	81	TRUE	
1	SFKEELDKY	1129	1137	9	HLA-A*02:01	1129	81	81	1.53E+06	81	35165.92	7.10E+01	0.013424558	90	81	TRUE	
1	SSKRFQPFQ	541	549	9	HLA-A*02:01	541	81	81	1.53E+06	81	39802.98	8.80E+01	0.0010292	35	81	TRUE	
1	CSVKSFEID	288	296	9	HLA-A*02:01	288	81	81	1.55E+06	81	34770.53	7.00E+01	0.014924507	91	81	TRUE	
1	CSCLKGACS	1223	1231	9	HLA-A*02:01	1223	81	81	1.57E+06	81	31784.79	6.00E+01	0.017013748	93	81	TRUE	
1	TPSSKRFQP	539	547	9	HLA-A*02:01	539	81	81	1.59E+06	81	42904.07	9.60E+01	0.0033861	62	81	TRUE	
1	NYKLPDDFM	409	417	9	HLA-A*02:01	409	81	81	1.60E+06	81	34426.89	6.90E+01	0.00839847	82	81	TRUE	
1	EDLLFNKVT	801	809	9	HLA-A*02:01	801	81	81	2.65E+06	86	37820.48	8.10E+01	0.00261457	56	81	TRUE	
1	LGQSKRVDF	1016	1024	9	HLA-A*02:01	1016	81	81	2.85E+06	87	36146.53	7.50E+01	0.00799834	81	81	TRUE	
1	CDVVIGIIN	1108	1116	9	HLA-A*02:01	1108	81	81	3.26E+06	88	27453.06	4.90E+01	0.0079726	81	81	TRUE	
1	GVSATKLND	368	376	9	HLA-A*02:01	368	82	82	2.92E+05	61	39892.67	8.80E+01	0.00835795	82	82	TRUE	
1	DVSEKSGNF	171	179	9	HLA-A*02:01	171	82	82	5.06E+05	68	38114.63	8.20E+01	0.017790985	93	82	TRUE	
1	IDATSTGNY	428	436	9	HLA-A*02:01	428	82	82	1.72E+06	82	39580.54	8.70E+01	0.00508628	71	82	TRUE	
1	ADSFVVKGD	384	392	9	HLA-A*02:01	384	82	82	1.74E+06	82	37934.41	8.10E+01	0.0092151	83	82	TRUE	
1	CGSCCKFDE	1232	1240	9	HLA-A*02:01	1232	82	82	3.87E+06	89	38289.48	8.20E+01	0.00696627	78	82	TRUE	
1	DSFKEELDK	1128	1136	9	HLA-A*02:01	1128	82	82	1.16E+07	96	38174.06	8.20E+01	0.00276249	57	82	TRUE	
1	NDILSRLDK	960	968	9	HLA-A*02:01	960	82	82	1.28E+07	96	38099.37	8.20E+01	0.00812269	81	82	TRUE	
1	GKAYFPREG	1067	1075	9	HLA-A*02:01	1067	83	83	5.32E+05	69	41086.38	9.20E+01	0.00889201	83	83	TRUE	
1	NTFVSGNCD	1101	1109	9	HLA-A*02:01	1101	83	83	7.26E+05	72	38545.93	8.30E+01	0.012362321	89	83	TRUE	
1	VDFCGKGYH	1022	1030	9	HLA-A*02:01	1022	83	83	1.18E+06	78	40454.69	9.00E+01	0.00884301	83	83	TRUE	
1	VNQNAQALN	934	942	9	HLA-A*02:01	934	83	83	1.79E+06	83	43440.3	9.70E+01	0.0018578	48	83	TRUE	
1	CGKGYHLMS	1025	1033	9	HLA-A*02:01	1025	83	83	1.81E+06	83	35943.73	7.40E+01	0.02162719	95	83	TRUE	
1	LDRCTTFDD	16	24	9	HLA-A*02:01	16	83	83	1.85E+06	83	39054.73	8.50E+01	0.00459727	69	83	TRUE	
1	WERKKISNC	340	348	9	HLA-A*02:01	340	83	83	1.87E+06	83	38679.21	8.40E+01	0.00615602	76	83	TRUE	
1	VSVITPGTN	581	589	9	HLA-A*02:01	581	83	83	1.87E+06	83	36972.44	7.80E+01	0.023227368	96	83	TRUE	
1	QSKRVDFCG	1018	1026	9	HLA-A*02:01	1018	83	83	1.88E+06	83	37251.5	7.90E+01	0.023323837	96	83	TRUE	
1	NNSTNVVIR	118	126	9	HLA-A*02:01	118	83	83	1.95E+06	83	36860.99	7.70E+01	0.013905929	90	83	TRUE	

seq #	peptide	start	end	peptide length	allele	peptide index	median binding	consensus perc	smm IC50	smm percentile	ann IC50	ann percentile	comblib_sidney	comblib_sidney	legacy consens	percentiles equal
1	QERNFTTAP	1053	1061	9	HLA-A*02:01	1053	83	83	3.03E+06	87	38424.34	8.30E+01	0.00194581	49	83	TRUE
1	QNPLAELKC	280	288	9	HLA-A*02:01	280	83	83	4.91E+06	91	38354.16	8.30E+01	0.0030486	60	83	TRUE
1	LTAFSPAQD	235	243	9	HLA-A*02:01	235	84	84	1.08E+05	49	38654.94	8.40E+01	0.012164661	88	84	TRUE
1	VYAWERKKI	337	345	9	HLA-A*02:01	337	84	84	1.57E+05	54	41556.26	9.30E+01	0.00931537	84	84	TRUE
1	WNTRNIDAT	423	431	9	HLA-A*02:01	423	84	84	5.43E+05	69	40249.06	8.90E+01	0.0093713	84	84	TRUE
1	AWRIYSTGN	618	626	9	HLA-A*02:01	618	84	84	5.51E+05	69	38639.46	8.40E+01	0.03279442	98	84	TRUE
1	ATEKSNVVR	91	99	9	HLA-A*02:01	91	84	84	6.30E+05	71	38828.49	8.40E+01	0.010553585	86	84	TRUE
1	TRNIDATST	425	433	9	HLA-A*02:01	425	84	84	8.59E+05	75	39930.66	8.80E+01	0.00931966	84	84	TRUE
1	HVTYVPSQE	1046	1054	9	HLA-A*02:01	1046	84	84	9.10E+05	75	38644.9	8.40E+01	0.012858787	89	84	TRUE
1	VVKGDDVRQ	388	396	9	HLA-A*02:01	388	84	84	1.12E+06	78	38863.79	8.40E+01	0.02353423	96	84	TRUE
1	AYTMSLGAD	676	684	9	HLA-A*02:01	676	84	84	1.19E+06	78	38812.52	8.40E+01	0.018167702	93	84	TRUE
1	GNPVIPFKD	77	85	9	HLA-A*02:01	77	84	84	1.21E+06	78	38710.6	8.40E+01	0.010861755	87	84	TRUE
1	LNDILSRLD	959	967	9	HLA-A*02:01	959	84	84	2.01E+06	84	36107.46	7.50E+01	0.018832154	94	84	TRUE
1	QAGCLIGAE	632	640	9	HLA-A*02:01	632	84	84	2.01E+06	84	33915.94	6.70E+01	0.012393672	89	84	TRUE
1	CCSCLKGAC	1222	1230	9	HLA-A*02:01	1222	84	84	2.03E+06	84	35357.05	7.20E+01	0.06218706	100	84	TRUE
1	CGDSTECAN	725	733	9	HLA-A*02:01	725	84	84	2.03E+06	84	39969.15	8.80E+01	0.00086338	32	84	TRUE
1	TYVPSQERN	1048	1056	9	HLA-A*02:01	1048	84	84	2.08E+06	84	44953.18	1.00E+02	0.00180053	48	84	TRUE
1	TGRLQSLQT	980	988	9	HLA-A*02:01	980	84	84	2.16E+06	84	36143.01	7.50E+01	0.02360478	96	84	TRUE
1	AAEIRASAN	997	1005	9	HLA-A*02:01	997	84	84	3.01E+06	87	38754.61	8.40E+01	0.0064328	76	84	TRUE
1	NESLIDLQE	1176	1184	9	HLA-A*02:01	1176	84	84	1.41E+07	97	34027.3	6.70E+01	0.00966051	84	84	TRUE
1	QDRNTREVF	756	764	9	HLA-A*02:01	756	84	84	2.36E+07	98	38725.25	8.40E+01	0.00883894	83	84	TRUE
1	NVTGFHTIN	65	73	9	HLA-A*02:01	65	85	85	3.41E+05	63	39132.14	8.50E+01	0.01996181	94	85	TRUE
1	LYENQKQIA	898	906	9	HLA-A*02:01	898	85	85	5.05E+05	68	41927.51	9.40E+01	0.010027669	85	85	TRUE
1	VKSFEIDKG	290	298	9	HLA-A*02:01	290	85	85	1.15E+06	78	39790.07	8.80E+01	0.010185914	85	85	TRUE
1	SGIAAEQDR	750	758	9	HLA-A*02:01	750	85	85	2.21E+06	85	42452.91	9.50E+01	0.0002341	13	85	TRUE
1	VRQIAPGQT	394	402	9	HLA-A*02:01	394	85	85	2.23E+06	85	39099.54	8.50E+01	0.0204833	95	85	TRUE
1	PTTFMLKYD	259	267	9	HLA-A*02:01	259	85	85	2.25E+06	85	39744.89	8.70E+01	0.00175752	47	85	TRUE
1	TDAVDCSQN	273	281	9	HLA-A*02:01	273	85	85	2.30E+06	85	43062.66	9.70E+01	0.0085428	82	85	TRUE
1	HTSSMRGVY	33	41	9	HLA-A*02:01	33	85	85	2.32E+06	85	39053.48	8.50E+01	0.00167958	46	85	TRUE
1	KEELDKYFK	1131	1139	9	HLA-A*02:01	1131	85	85	2.38E+06	85	39045.02	8.50E+01	0.0017112	47	85	TRUE
1	LDSFKEELD	1127	1135	9	HLA-A*02:01	1127	85	85	2.43E+06	85	42578.05	9.60E+01	0.00310099	60	85	TRUE
1	REFVFKNKD	183	191	9	HLA-A*02:01	183	85	85	2.44E+06	85	39915.55	8.80E+01	0.00966273	84	85	TRUE
1	TGVLTPSSK	535	543	9	HLA-A*02:01	535	85	85	3.94E+06	89	38994.79	8.50E+01	0.00350994	63	85	TRUE
1	DCSQNPLAE	277	285	9	HLA-A*02:01	277	85	85	3.34E+07	99	38994.79	8.50E+01	0.00856249	82	85	TRUE
1	LDKVEAEVQ	966	974	9	HLA-A*02:01	966	85	85	4.25E+07	100	39019.69	8.50E+01	0.00924911	83	85	TRUE
1	GVLTPSSKR	536	544	9	HLA-A*02:01	536	86	86	1.35E+05	52	40964.3	9.10E+01	0.010505095	86	86	TRUE
1	SGSDLDRCT	12	20	9	HLA-A*02:01	12	86	86	2.42E+05	59	39362.72	8.60E+01	0.011857687	88	86	TRUE
1	SVRDPKTSE	561	569	9	HLA-A*02:01	561	86	86	1.90E+06	83	42281.92	9.50E+01	0.010399202	86	86	TRUE
1	ACNFELCDN	127	135	9	HLA-A*02:01	127	86	86	2.49E+06	86	35823	7.40E+01	0.015689171	92	86	TRUE
1	VNIQKEIDR	1159	1167	9	HLA-A*02:01	1159	86	86	2.53E+06	86	39707.94	8.70E+01	0.00042678	20	86	TRUE
1	NNTIAIPTN	691	699	9	HLA-A*02:01	691	86	86	2.57E+06	86	39615.25	8.70E+01	0.00103276	35	86	TRUE
1	DDVRQIAPG	392	400	9	HLA-A*02:01	392	86	86	2.64E+06	86	42151.29	9.50E+01	0.00170844	46	86	TRUE
1	SKPMGTQTH	141	149	9	HLA-A*02:01	141	86	86	2.66E+06	86	40337.56	8.90E+01	0.00199618	50	86	TRUE
1	NCTFEYISD	158	166	9	HLA-A*02:01	158	86	86	3.67E+06	89	39218.62	8.60E+01	0.00511093	71	86	TRUE
1	VSEKSGNFK	172	180	9	HLA-A*02:01	172	86	86	4.72E+06	90	38843.19	8.40E+01	0.010717661	86	86	TRUE
1	GWVFGSTMN	100	108	9	HLA-A*02:01	100	87	87	3.79E+05	65	39513.36	8.70E+01	0.021667061	95	87	TRUE
1	NVPFSPDGK	457	465	9	HLA-A*02:01	457	87	87	5.43E+05	69	39529.6	8.70E+01	0.024963185	97	87	TRUE
1	APNYTQHTS	27	35	9	HLA-A*02:01	27	87	87	5.56E+05	69	41937.92	9.40E+01	0.010929493	87	87	TRUE
1	CKFDEDDSE	1236	1244	9	HLA-A*02:01	1236	87	87	2.81E+06	87	41626	9.30E+01	0.00280543	58	87	TRUE
1	YGECLGDIN	819	827	9	HLA-A*02:01	819	87	87	2.86E+06	87	37142.86	7.80E+01	0.09981596	100	87	TRUE
1	QVKQMYKTP	766	774	9	HLA-A*02:01	766	87	87	3.39E+06	88	39719.54	8.70E+01	0.00665426	77	87	TRUE
1	DDSEPVLKG	1241	1249	9	HLA-A*02:01	1241	87	87	3.54E+06	88	39693.76	8.70E+01	0.00592379	75	87	TRUE
1	AEHVDTSYE	639	647	9	HLA-A*02:01	639	87	87	3.75E+06	89	39550.99	8.70E+01	0.00105196	35	87	TRUE
1	TSTALGKLQ	923	931	9	HLA-A*02:01	923	87	87	9.31E+06	95	38823.03	8.40E+01	0.0110535	87	87	TRUE
1	PCTPPALNC	466	474	9	HLA-A*02:01	466	87	87	1.11E+07	96	39667.99	8.70E+01	0.00513452	71	87	TRUE
1	DNAFNCTFE	154	162	9	HLA-A*02:01	154	87	87	1.48E+07	97	39602.37	8.70E+01	0.00238781	54	87	TRUE
1	QPELDSFKE	1124	1132	9	HLA-A*02:01	1124	87	87	1.63E+07	97	39732.43	8.70E+01	0.011030622	87	87	TRUE
1	LRSTSQKSI	666	674	9	HLA-A*02:01	666	88	88	2.42E+05	59	40674.57	9.00E+01	0.01211714	88	88	TRUE
1	GSCCKFDED	1233	1241	9	HLA-A*02:01	1233	88	88	6.43E+05	71	39782.32	8.80E+01	0.01990215	94	88	TRUE
1	KPCTPPALN	465	473	9	HLA-A*02:01	465	88	88	1.54E+06	81	40014.13	8.80E+01	0.022069881	95	88	TRUE
1	NKVTLADAG	806	814	9	HLA-A*02:01	806	88	88	1.95E+06	83	39965.68	8.80E+01	0.012387966	89	88	TRUE
1	DTSYECDIP	643	651	9	HLA-A*02:01	643	88	88	2.30E+06	85	39887.91	8.80E+01	0.042589251	100	88	TRUE
1	GKLRPFERD	446	454	9	HLA-A*02:01	446	88	88	3.16E+06	88	42617.22	9.60E+01	0.00597173	75	88	TRUE
1	VPFSPDGKP	458	466	9	HLA-A*02:01	458	88	88	3.33E+06	88	42477.72	9.50E+01	0.00390571	65	88	TRUE
1	SEKSGNFKH	173	181	9	HLA-A*02:01	173	88	88	3.34E+06	88	42420.78	9.50E+01	0.00091664	33	88	TRUE
1	WRIYSTGNN	619	627	9	HLA-A*02:01	619	88	88	3.34E+06	88	43914.28	9.80E+01	0.00353671	63	88	TRUE
1	DAGFMKQYG	812	820	9	HLA-A*02:01	812	88	88	3.52E+06	88	41792.53	9.40E+01	0.00133783	41	88	TRUE
1	MRGVYYPDE	37	45	9	HLA-A*02:01	37	88	88	4.26E+06	90	39952.27	8.80E+01	0.00664355	77	88	TRUE
1	SCCKFDEDD	1234	1242	9	HLA-A*02:01	1234	88	88	6.34E+06	93	37914.31	8.10E+01	0.011898713	88	88	TRUE
1	EDDSEPVLK	1240	1248	9	HLA-A*02:01	1240	88	88	6.97E+06	93	39851.25	8.80E+01	0.00229985	53	88	TRUE
1	HVDTSYECD	641	649	9	HLA-A*02:01	641	89	89	2.72E+05	60	40344.98	8.90E+01	0.01564228	92	89	TRUE
1	MAKTSVDCN	713	721	9	HLA-A*02:01	713	89	89	7.36E+05	73	40194.22	8.90E+01	0.019925077	94	89	TRUE
1	KYRYLRHGK	439	447	9	HLA-A*02:01	439	89	89	2.55E+06	86	40214.24	8.90E+01	0.0296142	98	89	TRUE
1	SKRFQPFQQ	542	550	9	HLA-A*02:01	542	89	89	3.59E+06	89	37749.77	8.00E+01	0.015370925	91	89	TRUE
1	PFSPDGKPC	459	467	9	HLA-A*02:01	459	89	89	3.62E+06	89	44025.61	9.90E+01	0.00533335	72	89	TRUE
1	AWERKKISN	339	347	9	HLA-A*02:01	339	89	89	3.65E+06	89	43798.97	9.80E+01	0.00932825	84	89	TRUE
1	NNVFQTQAG	626	634	9	HLA-A*02:01	626	89	89	3.72E+06	89	42210.62	9.50E+01	0.00177337	47	89	TRUE
1	LKCSVKSFE	286	294	9	HLA-A*02:01	286	89	89	3.77E+06	89	40361.14	8.90E+01	0.00104232	35	89	TRUE
1	QKQIANQFN	902	910	9	HLA-A*02:01	902	89	89	3.94E+06	89	42122.57	9.50E+01	0.00073485	29	89	TRUE
1	NFRVVPSGD	304	312	9	HLA-A*02:01	304	89	89	3.95E+06	89	37424.01	7.90E+01	0.014028137	90	89	TRUE
1	LDVSEKSGN	170	178	9	HLA-A*02:01	170	89	89	4.01E+06	89	45758.45	1.00E+02	0.00333887	62	89	TRUE
1	NNTVYDPLQ	1116	1124	9	HLA-A*02:01	1116	89	89	4.01E+06	89	42034.7	9.40E+01	0.00542625	73	89	TRUE
1	TEKSNVVRG	92	100	9	HLA-A*02:01	92	89	89	4.89E+06	91	40182.92	8.90E+01	0.00159221	45	89	TRUE
1	REVFAQVKQ	761	769	9	HLA-A*02:01	761	89	89	4.93E+06	91	40139.9	8.90E+01	0.0069775	78	89	TRUE
1	VRDLPSGFN	206	214	9	HLA-A*02:01	206	89	89	6.11E+06	92	40238.16	8.90E+01	0.00349623	63	89	TRUE
1	QNVLYENQK	895	903	9	HLA-A*02:01	895	89	89	7.78E+06	94	40252.09	8.90E+01	0.00258881	56	89	TRUE
1	EGKAYFPRE	1066	1074	9	HLA-A*02:01	1066	89	89	2.48E+07	98	40195.52	8.90E+01	0.00141906	42	89	TRUE
1	SDFTDSVRD	556	564	9	HLA-A*02:01	556	90	90	3.95E+05	65	40603.78	9.00E+01	0.015328512	91	90	TRUE
1	TYVTQQLIR	988	996	9	HLA-A*02:01	988	90	90	1.06E+06	77	40441.99	9.00E+01	0.02262559	96	90	TRUE
1	KPTKRSFIE	793	801	9	HLA-A*02:01	793	90	90	2.96E+06	87	41390.22	9.30E+01	0.014015223	90	90	TRUE
1	QPFQQFGRD	546	554	9	HLA-A*02:01	546	90	90	4.20E+06	90	40844.8	9.10E+01	0.00637823	76	90	TRUE
1	PTKRSFIED	794	802	9	HLA-A*02:01	794	90	90	4.24E+06	90	41965.17	9.40E+01	0.0047852	70	90	TRUE
1	PGQTGVIAD	399	407	9	HLA-A*02:01	399	90	90	4.26E+06	90	40853.65	9.10E+01	0.00216222	52	90	TRUE
1	SKRVDFCGK	1019	1027	9	HLA-A*02:01	1019	90	90	4.28E+06	90	35888.16	7.40E+01	0.031426788	98	90	TRUE
1	AEQDRNTRE	754	762	9	HLA-A*02:01	754	90	90	4.32E+06	90	44452.1	9.90E+01	0.00166725	46	90	TRUE
1	PKLSTDLIK	513	521	9	HLA-A*02:01	513	90	90	1.16E+07	96	40408.3	9.00E+01	0.00372477	64	90	TRUE
1	PSVYAWERK	335	343	9	HLA-A*02:01	335	90	90	1.40E+07	97	40462.57	9.00E+01	0.01013678	85	90	TRUE
1	TGNYNYKYR	433	441	9	HLA-A*02:01	433	91	91	3.52E+06	88	40874.42	9.10E+01	0.0201558	94	91	TRUE
1	LGKYEQYIK	1185	1193	9	HLA-A*02:01	1185	91	91	4.75E+06	91	42340.53	9.50E+01	0.00671893	78	91	TRUE
1	DATSTGNYN	429	437	9	HLA-A*02:01	429	91	91	4.82E+06	91	43200.32	9.70E+01	0.0032181	61	91	TRUE
1	DFTDSVRDP	557	565	9	HLA-A*02:01	557	91	91	5.32E+06	91	43054.72	9.70E+01	0.00621155	76	91	TRUE
1	DPLQPELDS	1121	1129	9	HLA-A*02:01	1121	91	91	1.92E+07	98	40914.69	9.10E+01	0.00234855	54	91	TRUE
1	LDKYFKNHT	1134	1142	9	HLA-A*02:01	1134	92	92	1.47E+06	80	41068.6	9.20E+01	0.04628071	100	92	TRUE
1	PNYTQHTSS	28	36	9	HLA-A*02:01	28	92	92	5.57E+06	92	45424.99	1.00E+02	0.00960727	84	92	TRUE
1	PSGFNTLKP	210	218	9	HLA-A*02:01	210	92	92	5.65E+06	92	41085.48	9.20E+01	0.00443302	68	92	TRUE
1	DYNYKLPDD	407	415	9	HLA-A*02:01	407	92	92	5.65E+06	92	43630.61	9.80E+01	0.0045867	69	92	TRUE
1	ENQKQIANQ	900	908	9	HLA-A*02:01	900	92	92	3.20E+07	99	41078.38	9.20E+01	0.0090469	83	92	TRUE
1	VVNIQKEID	1158	1166	9	HLA-A*02:01	1158	93	93	9.55E+05	76	41595.84	9.30E+01	0.0352452	99	93	TRUE
1	AAEQDRNTR	753	761	9	HLA-A*02:01	753	93	93	3.57E+06	89	41711.67	9.30E+01	0.02396625	96	93	TRUE
1	PDVDLGDIS	1144	1152	9	HLA-A*02:01	1144	93	93	6.67E+06	93	44249.11	9.90E+01	0.015317927	91	93	TRUE
1	RCTTFDDVQ	18	26	9	HLA-A*02:01	18	93	93	6.67E+06	93	38852.43	8.40E+01	0.045930376	100	93	TRUE
1	NKAISQIQE	910	918	9	HLA-A*02:01	910	93	93	6.89E+06	93	41485.28	9.30E+01	0.00155955	44	93	TRUE
1	IFKLPLGIN	219	227	9	HLA-A*02:01	219	93	93	8.65E+06	94	33608.72	6.60E+01	0.017992852	93	93	TRUE
1	GSTMNNKSQ	104	112	9	HLA-A*02:01	104	94	94	7.43E+06	94	43840.71	9.80E+01	0.00996552	85	94	TRUE
1	DKGIYQTSN	296	304	9	HLA-A*02:01	296	94	94	7.59E+06	94	45031.55	1.00E+02	0.013471005	90	94	TRUE
1	TNVVIRACN	121	129	9	HLA-A*02:01	121	94	94	8.91E+06	94	40770.19	9.10E+01	0.026730064	97	94	TRUE
1	SGNFKHLRE	176	184	9	HLA-A*02:01	176	94	94	1.02E+07	95	40201.17	8.90E+01	0.01918669	94	94	TRUE
1	NEVAKNLNE	1169	1177	9	HLA-A*02:01	1169	94	94	1.06E+07	95	41858.59	9.40E+01	0.00192752	49	94	TRUE
1	RYLRHGKLR	441	449	9	HLA-A*02:01	441	95	95	1.64E+06	81	42373.53	9.50E+01	0.023746521	96	95	TRUE
1	ANQFNKAIS	906	914	9	HLA-A*02:01	906	95	95	3.36E+06	88	42237.58	9.50E+01	0.030605537	98	95	TRUE
1	RHGKLRPFE	444	452	9	HLA-A*02:01	444	95	95	9.14E+06	95	42239.87	9.50E+01	0.00189845	49	95	TRUE
1	DRLNEVAKN	1166	1174	9	HLA-A*02:01	1166	95	95	1.05E+07	95	44014.65	9.80E+01	0.0014054	42	95	TRUE

seq #	peptide	start	end	peptide length	allele	peptide index	median binding	consensus perc	smm IC50	smm percentile	ann IC50	ann percentile	comblib_sidney	comblib_sidney	legacy consens	percentiles equal	
1	PDGKPCTPP	462	470	9	HLA-A*02:01	462	95	95	1.08E+07	95	42198.75	9.50E+01	0.00555009	73	95	TRUE	
1	NGTITDAVD	269	277	9	HLA-A*02:01	269	95	95	10867008.33	95	42077.01	94	0.030276105	98	95	TRUE	
1	CCKFDEDDS	1235	1243	9	HLA-A*02:01	1235	95	95	24553307.34	98	42377.66	95	0.02100874	95	95	TRUE	
1	DRLITGRLQ	976	984	9	HLA-A*02:01	976	95	95	112230268	100	37593.65	80	0.020715718	95	95	TRUE	
1	DSSIAYSNN	684	692	9	HLA-A*02:01	684	96	96	11223026.8	96	42545.33	96	0.017790985	93	96	TRUE	
1	QDVVNQNAQ	931	939	9	HLA-A*02:01	931	96	96	12419668.39	96	43558.91	98	0.00807421	81	96	TRUE	
1	DSFVVKGDD	385	393	9	HLA-A*02:01	385	96	96	26675342.24	99	40916.46	91	0.023555916	96	96	TRUE	
1	PELDSFKEE	1125	1133	9	HLA-A*02:01	1125	97	97	14292559.21	97	43608.41	98	0.00238671	54	97	TRUE	
1	LGKLQDVVN	927	935	9	HLA-A*02:01	927	97	97	14828936.31	97	40330.12	89	0.026804024	97	97	TRUE	
1	SFEIDKGIY	292	300	9	HLA-A*02:01	292	97	97	16372311.54	97	36254.25	75	0.116654098	100	97	TRUE	
1	NYKYRYLRH	437	445	9	HLA-A*02:01	437	97	97	16753671.67	97	38624.84	84	0.037792011	99	97	TRUE	
1	TPGTNASSE	585	593	9	HLA-A*02:01	585	97	97	17143914.81	97	44042.28	99	0.00423253	67	97	TRUE	
1	DNTFVSGNC	1100	1108	9	HLA-A*02:01	1100	97	97	18243577.25	97	40989.56	91	0.04584585	100	97	TRUE	
1	ECVLGQSKR	1013	1021	9	HLA-A*02:01	1013	97	97	20096017.49	98	43337.02	97	0.00317541	60	97	TRUE	
1	LNEVAKNLN	1168	1176	9	HLA-A*02:01	1168	97	97	23829229.5	98	42959.8	96	0.0258226	97	97	TRUE	
1	PDPLKPTKR	789	797	9	HLA-A*02:01	789	97	97	26736835.25	99	43228.83	97	0.021261799	95	97	TRUE	
1	DPKTSEILD	564	572	9	HLA-A*02:01	564	97	97	54589611.05	100	43461.46	97	0.00251131	55	97	TRUE	
1	LRPFERDIS	448	456	9	HLA-A*02:01	448	98	98	11697956.44	96	43836.44	98	0.09298224	100	98	TRUE	
1	DPLKPTKRS	790	798	9	HLA-A*02:01	790	98	98	21883158.2	98	45117.39	100	0.00380014	64	98	TRUE	
1	SEPVLKGVK	1243	1251	9	HLA-A*02:01	1243	98	98	22289996.49	98	41635.02	93	0.05679368	100	98	TRUE	
1	LREFVFKNK	182	190	9	HLA-A*02:01	182	98	98	22652179.79	98	32338.44	62	0.05066406	100	98	TRUE	
1	PDEIFRSDT	43	51	9	HLA-A*02:01	43	98	98	45825794.16	100	43030.04	97	0.03042286	98	98	TRUE	
1	KNHTSPDVD	1139	1147	9	HLA-A*02:01	1139	99	99	4700131.405	90	44272.57	99	0.033181804	99	99	TRUE	
1	DSVRDPKTS	560	568	9	HLA-A*02:01	560	99	99	9888035.11	95	44596.13	99	0.05546257	100	99	TRUE	
1	EFVFKNKDG	184	192	9	HLA-A*02:01	184	99	99	29181660.46	99	44753.7	100	0.018276793	93	99	TRUE	
1	NPVIPFKDG	78	86	9	HLA-A*02:01	78	99	99	29724188.04	99	38761.31	84	0.04792918	100	99	TRUE	
1	QKEIDRLNE	1162	1170	9	HLA-A*02:01	1162	100	100	115374544.6	100	41357.99	92	0.06367955	100	100	TRUE	

seq #	peptide	start	end	peptide length	allele	peptide index	median binding	ann IC50	ann percentile	legacy IC50	legacy rank	scores equal	percentiles equal	
1	FIAGLIAIV	1202	1210	9	HLA-A*02:01	1202	0.09	10.29	0.09	10.29	0.09	TRUE	TRUE	
1	MIAAYTAAL	851	859	9	HLA-A*02:01	851	0.13	14.39	0.13	14.39	0.13	TRUE	TRUE	
1	RLQSLQTYV	982	990	9	HLA-A*02:01	982	0.16	16.66	0.16	16.66	0.16	TRUE	TRUE	
1	YLKPTTFML	256	264	9	HLA-A*02:01	256	0.17	18.56	0.17	18.56	0.17	TRUE	TRUE	
1	LLFNKVTLA	803	811	9	HLA-A*02:01	803	0.22	25.41	0.22	25.41	0.22	TRUE	TRUE	
1	FSISITTEV	700	708	9	HLA-A*02:01	700	0.27	30.13	0.27	30.13	0.27	TRUE	TRUE	
1	HLMSFPQAA	1030	1038	9	HLA-A*02:01	1030	0.28	30.89	0.28	30.89	0.28	TRUE	TRUE	
1	LLTDDMIAA	846	854	9	HLA-A*02:01	846	0.29	32.91	0.29	32.91	0.29	TRUE	TRUE	
1	VLNDILSRL	958	966	9	HLA-A*02:01	958	0.3	33.57	0.3	33.57	0.3	TRUE	TRUE	
1	VVFLHVTYV	1042	1050	9	HLA-A*02:01	1042	0.33	36.56	0.33	36.56	0.33	TRUE	TRUE	
1	SIVAYTMSL	673	681	9	HLA-A*02:01	673	0.33	37.41	0.33	37.41	0.33	TRUE	TRUE	
1	RLDKVEAEV	965	973	9	HLA-A*02:01	965	0.35	38.95	0.35	38.95	0.35	TRUE	TRUE	
1	YVYKGYQPI	195	203	9	HLA-A*02:01	195	0.43	47.04	0.43	47.04	0.43	TRUE	TRUE	
1	KQMYKTPTL	768	776	9	HLA-A*02:01	768	0.43	47.7	0.43	47.7	0.43	TRUE	TRUE	
1	VIADYNYKL	404	412	9	HLA-A*02:01	404	0.46	50.27	0.46	50.27	0.46	TRUE	TRUE	
1	SITTEVMPV	703	711	9	HLA-A*02:01	703	0.46	50.81	0.46	50.81	0.46	TRUE	TRUE	
1	VLYNSTFFS	354	362	9	HLA-A*02:01	354	0.64	70.21	0.64	70.21	0.64	TRUE	TRUE	
1	FIFLLFLTL	2	10	9	HLA-A*02:01	2	0.66	71.52	0.66	71.52	0.66	TRUE	TRUE	
1	NVYADSFVV	381	389	9	HLA-A*02:01	381	0.77	87.86	0.77	87.86	0.77	TRUE	TRUE	
1	ALVSGTATA	858	866	9	HLA-A*02:01	858	0.97	122.57	0.97	122.57	0.97	TRUE	TRUE	
1	GLTVLPPLL	839	847	9	HLA-A*02:01	839	1.2	130.4	1.2	130.4	1.2	TRUE	TRUE	
1	KQLSSNFGA	946	954	9	HLA-A*02:01	946	1.2	141.16	1.2	141.16	1.2	TRUE	TRUE	
1	FLLFLTLTS	4	12	9	HLA-A*02:01	4	1.2	141.86	1.2	141.86	1.2	TRUE	TRUE	
1	GTSAAAYFV	246	254	9	HLA-A*02:01	246	1.4	167.91	1.4	167.91	1.4	TRUE	TRUE	
1	NLNESLIDL	1174	1182	9	HLA-A*02:01	1174	1.4	177.32	1.4	177.32	1.4	TRUE	TRUE	
1	ATAGWTFGA	864	872	9	HLA-A*02:01	864	1.6	207.35	1.6	207.35	1.6	TRUE	TRUE	
1	IIINNSTNV	115	123	9	HLA-A*02:01	115	1.7	228.99	1.7	228.99	1.7	TRUE	TRUE	
1	GLIAIVMVT	1205	1213	9	HLA-A*02:01	1205	1.8	242.04	1.8	242.04	1.8	TRUE	TRUE	
1	TMNNKSQSV	106	114	9	HLA-A*02:01	106	1.8	254.01	1.8	254.01	1.8	TRUE	TRUE	
1	VLSFELLNA	498	506	9	HLA-A*02:01	498	2	273.66	2	273.66	2	TRUE	TRUE	
1	ELLNAPATV	502	510	9	HLA-A*02:01	502	2	279.55	2	279.55	2	TRUE	TRUE	
1	FVSGNCDVV	1103	1111	9	HLA-A*02:01	1103	2	290.37	2	290.37	2	TRUE	TRUE	
1	SLTTTSTAL	919	927	9	HLA-A*02:01	919	2	300.04	2	300.04	2	TRUE	TRUE	
1	IITTDNTFV	1096	1104	9	HLA-A*02:01	1096	2.2	338.82	2.2	338.82	2.2	TRUE	TRUE	
1	AILTAFSPA	233	241	9	HLA-A*02:01	233	2.3	351.2	2.3	351.2	2.3	TRUE	TRUE	
1	FLPFYSNVT	59	67	9	HLA-A*02:01	59	2.4	394.17	2.4	394.17	2.4	TRUE	TRUE	
1	ILLCCMTSC	1214	1222	9	HLA-A*02:01	1214	2.5	403.87	2.5	403.87	2.5	TRUE	TRUE	
1	ELCDNPFFA	131	139	9	HLA-A*02:01	131	2.5	408.87	2.5	408.87	2.5	TRUE	TRUE	
1	RVVVLSFEL	495	503	9	HLA-A*02:01	495	2.5	419.83	2.5	419.83	2.5	TRUE	TRUE	
1	MIFDNAFNC	151	159	9	HLA-A*02:01	151	2.5	421.14	2.5	421.14	2.5	TRUE	TRUE	
1	IAIPTNFSI	694	702	9	HLA-A*02:01	694	2.8	487.32	2.8	487.32	2.8	TRUE	TRUE	
1	FSTFKCYGV	361	369	9	HLA-A*02:01	361	2.8	504.47	2.8	504.47	2.8	TRUE	TRUE	
1	LLQYGSFCT	735	743	9	HLA-A*02:01	735	2.9	541.11	2.9	541.11	2.9	TRUE	TRUE	
1	GICASYHTV	655	663	9	HLA-A*02:01	655	3	546.07	3	546.07	3	TRUE	TRUE	
1	QMAYRFNGI	883	891	9	HLA-A*02:01	883	3	550.3	3	550.3	3	TRUE	TRUE	
1	TQAGCLIGA	631	639	9	HLA-A*02:01	631	3	551.45	3	551.45	3	TRUE	TRUE	
1	VLYENQKQI	897	905	9	HLA-A*02:01	897	3	562.95	3	562.95	3	TRUE	TRUE	
1	YQDVNCTDV	598	606	9	HLA-A*02:01	598	3.2	612.82	3.2	612.82	3.2	TRUE	TRUE	
1	CMTSCCSCL	1218	1226	9	HLA-A*02:01	1218	3.2	629.65	3.2	629.65	3.2	TRUE	TRUE	
1	TSYECDIPI	644	652	9	HLA-A*02:01	644	3.2	639.51	3.2	639.51	3.2	TRUE	TRUE	
1	LIAIVMVTI	1206	1214	9	HLA-A*02:01	1206	3.4	691.85	3.4	691.85	3.4	TRUE	TRUE	
1	FMKQYGECL	815	823	9	HLA-A*02:01	815	3.4	698.66	3.4	698.66	3.4	TRUE	TRUE	
1	AIVMVTILL	1208	1216	9	HLA-A*02:01	1208	3.4	711.15	3.4	711.15	3.4	TRUE	TRUE	
1	ALGKLQDVV	926	934	9	HLA-A*02:01	926	3.4	717.01	3.4	717.01	3.4	TRUE	TRUE	
1	VIRACNFEL	124	132	9	HLA-A*02:01	124	3.6	786.23	3.6	786.23	3.6	TRUE	TRUE	
1	VLAWNTRNI	420	428	9	HLA-A*02:01	420	3.7	808.52	3.7	808.52	3.7	TRUE	TRUE	
1	YQPYRVVVL	491	499	9	HLA-A*02:01	491	3.9	862.1	3.9	862.1	3.9	TRUE	TRUE	
1	LQIPFAMQM	876	884	9	HLA-A*02:01	876	4	896.61	4	896.61	4	TRUE	TRUE	
1	RLNEVAKNL	1167	1175	9	HLA-A*02:01	1167	4.1	940.92	4.1	940.92	4.1	TRUE	TRUE	
1	YLTQDLFLP	53	61	9	HLA-A*02:01	53	4.2	975.48	4.2	975.48	4.2	TRUE	TRUE	
1	KLPDDFMGC	411	419	9	HLA-A*02:01	411	4.3	1013.85	4.3	1013.85	4.3	TRUE	TRUE	
1	RIYSTGNNV	620	628	9	HLA-A*02:01	620	4.4	1067.65	4.4	1067.65	4.4	TRUE	TRUE	
1	IINNSTNVV	116	124	9	HLA-A*02:01	116	4.4	1073.84	4.4	1073.84	4.4	TRUE	TRUE	
1	YQTSNFRVV	300	308	9	HLA-A*02:01	300	4.6	1149.79	4.6	1149.79	4.6	TRUE	TRUE	
1	FLHVTYVPS	1044	1052	9	HLA-A*02:01	1044	4.7	1187.08	4.7	1187.08	4.7	TRUE	TRUE	
1	VIGIINNTV	1111	1119	9	HLA-A*02:01	1111	5	1299.39	5	1299.39	5	TRUE	TRUE	
1	TSVDCNMYI	716	724	9	HLA-A*02:01	716	5	1314.15	5	1314.15	5	TRUE	TRUE	
1	GINASVVNI	1153	1161	9	HLA-A*02:01	1153	5.1	1346.83	5.1	1346.83	5.1	TRUE	TRUE	
1	GIGYQPYRV	488	496	9	HLA-A*02:01	488	5.2	1383.29	5.2	1383.29	5.2	TRUE	TRUE	
1	QLNRALSGI	744	752	9	HLA-A*02:01	744	5.2	1406.79	5.2	1406.79	5.2	TRUE	TRUE	
1	PLAELKCSV	282	290	9	HLA-A*02:01	282	5.3	1451.87	5.3	1451.87	5.3	TRUE	TRUE	
1	VLYQDVNCT	596	604	9	HLA-A*02:01	596	5.3	1463.83	5.3	1463.83	5.3	TRUE	TRUE	
1	WTFGAGAAL	868	876	9	HLA-A*02:01	868	5.3	1468.2	5.3	1468.2	5.3	TRUE	TRUE	
1	CTFEYISDA	159	167	9	HLA-A*02:01	159	5.4	1478.53	5.4	1478.53	5.4	TRUE	TRUE	
1	QIAPGQTGV	396	404	9	HLA-A*02:01	396	5.4	1487.84	5.4	1487.84	5.4	TRUE	TRUE	
1	YVTQQLIRA	989	997	9	HLA-A*02:01	989	5.6	1577.6	5.6	1577.6	5.6	TRUE	TRUE	
1	WLGFIAGLI	1199	1207	9	HLA-A*02:01	1199	5.6	1603.03	5.6	1603.03	5.6	TRUE	TRUE	
1	QYIKWPWYV	1190	1198	9	HLA-A*02:01	1190	5.6	1620.14	5.6	1620.14	5.6	TRUE	TRUE	
1	ALNTLVKQL	940	948	9	HLA-A*02:01	940	5.7	1633.23	5.7	1633.23	5.7	TRUE	TRUE	
1	LITGRLQSL	978	986	9	HLA-A*02:01	978	5.7	1675.27	5.7	1675.27	5.7	TRUE	TRUE	
1	LLFLTLTSG	5	13	9	HLA-A*02:01	5	5.7	1676.81	5.7	1676.81	5.7	TRUE	TRUE	
1	AVDCSQNPL	275	283	9	HLA-A*02:01	275	5.8	1719.38	5.8	1719.38	5.8	TRUE	TRUE	
1	VMVTILLCC	1210	1218	9	HLA-A*02:01	1210	5.9	1723.82	5.9	1723.82	5.9	TRUE	TRUE	
1	IAAYTAALV	852	860	9	HLA-A*02:01	852	6	1800.7	6	1800.7	6	TRUE	TRUE	
1	QLSSNFGAI	947	955	9	HLA-A*02:01	947	6	1808.71	6	1808.71	6	TRUE	TRUE	
1	AISSVLNDI	954	962	9	HLA-A*02:01	954	6.1	1829.77	6.1	1829.77	6.1	TRUE	TRUE	
1	FVFNGTSWF	1077	1085	9	HLA-A*02:01	1077	6.1	1869.65	6.1	1869.65	6.1	TRUE	TRUE	
1	YTAALVSGT	855	863	9	HLA-A*02:01	855	6.3	1924.22	6.3	1924.22	6.3	TRUE	TRUE	
1	AAAYFVGYL	249	257	9	HLA-A*02:01	249	6.4	1975.49	6.4	1975.49	6.4	TRUE	TRUE	
1	VWLGFIAGL	1198	1206	9	HLA-A*02:01	1198	6.5	2036.12	6.5	2036.12	6.5	TRUE	TRUE	
1	ILPDPLKPT	787	795	9	HLA-A*02:01	787	6.5	2037.13	6.5	2037.13	6.5	TRUE	TRUE	
1	FQTQAGCLI	629	637	9	HLA-A*02:01	629	6.7	2136.71	6.7	2136.71	6.7	TRUE	TRUE	
1	LCDNPFFAV	132	140	9	HLA-A*02:01	132	6.9	2259.03	6.9	2259.03	6.9	TRUE	TRUE	
1	AISQIQESL	912	920	9	HLA-A*02:01	912	7.1	2333.11	7.1	2333.11	7.1	TRUE	TRUE	
1	MFIFLLFLT	1	9	9	HLA-A*02:01	1	7.1	2359.69	7.1	2359.69	7.1	TRUE	TRUE	
1	FMLKYDENG	262	270	9	HLA-A*02:01	262	7.3	2462.27	7.3	2462.27	7.3	TRUE	TRUE	
1	LFLPFYSNV	58	66	9	HLA-A*02:01	58	7.3	2506	7.3	2506	7.3	TRUE	TRUE	
1	AALQIPFAM	874	882	9	HLA-A*02:01	874	7.4	2535.79	7.4	2535.79	7.4	TRUE	TRUE	
1	YVWLGFIAG	1197	1205	9	HLA-A*02:01	1197	7.5	2568.76	7.5	2568.76	7.5	TRUE	TRUE	
1	IVMVTILLC	1209	1217	9	HLA-A*02:01	1209	7.5	2591.76	7.5	2591.76	7.5	TRUE	TRUE	
1	PLNDYGFYT	477	485	9	HLA-A*02:01	477	7.6	2662.8	7.6	2662.8	7.6	TRUE	TRUE	
1	FNATKFPSV	329	337	9	HLA-A*02:01	329	7.6	2673.48	7.6	2673.48	7.6	TRUE	TRUE	

seq #	peptide	start	end	peptide length	allele	peptide index	median binding	ann IC50	ann percentile	legacy IC50	legacy rank	scores equal	percentiles equal	
1	FMGCVLAWN	416	424	9	HLA-A*02:01	416	7.6	2674.95	7.6	2674.95	7.6	TRUE	TRUE	
1	LLLQYGSFC	734	742	9	HLA-A*02:01	734	7.7	2742.92	7.7	2742.92	7.7	TRUE	TRUE	
1	SIAYSNNTI	686	694	9	HLA-A*02:01	686	8	2910.15	8	2910.15	8	TRUE	TRUE	
1	SLIDLQELG	1178	1186	9	HLA-A*02:01	1178	8.1	2973.3	8.1	2973.3	8.1	TRUE	TRUE	
1	ELDSFKEEL	1126	1134	9	HLA-A*02:01	1126	8.1	3001.36	8.1	3001.36	8.1	TRUE	TRUE	
1	MVTILLCCM	1211	1219	9	HLA-A*02:01	1211	8.4	3125.64	8.4	3125.64	8.4	TRUE	TRUE	
1	ISDAFSLDV	164	172	9	HLA-A*02:01	164	8.4	3153.15	8.4	3153.15	8.4	TRUE	TRUE	
1	KLPLGINIT	221	229	9	HLA-A*02:01	221	8.5	3200.59	8.5	3200.59	8.5	TRUE	TRUE	
1	FITQRNFFS	1085	1093	9	HLA-A*02:01	1085	8.7	3306.75	8.7	3306.75	8.7	TRUE	TRUE	
1	KLNDLCFSN	373	381	9	HLA-A*02:01	373	8.7	3317	8.7	3317	8.7	TRUE	TRUE	
1	NTLKPIFKL	214	222	9	HLA-A*02:01	214	8.9	3454.35	8.9	3454.35	8.9	TRUE	TRUE	
1	GFIAGLIAI	1201	1209	9	HLA-A*02:01	1201	9	3496.47	9	3496.47	9	TRUE	TRUE	
1	AAPHGVVFL	1037	1045	9	HLA-A*02:01	1037	9.1	3528.66	9.1	3528.66	9.1	TRUE	TRUE	
1	VVVLSFELL	496	504	9	HLA-A*02:01	496	9.1	3574.54	9.1	3574.54	9.1	TRUE	TRUE	
1	FKDGIYFAA	83	91	9	HLA-A*02:01	83	9.3	3682.77	9.3	3682.77	9.3	TRUE	TRUE	
1	MQMAYRFNG	882	890	9	HLA-A*02:01	882	9.3	3716	9.3	3716	9.3	TRUE	TRUE	
1	VVIGIINNT	1110	1118	9	HLA-A*02:01	1110	9.5	3845.5	9.5	3845.5	9.5	TRUE	TRUE	
1	LQTYVTQQL	986	994	9	HLA-A*02:01	986	9.6	3910.02	9.6	3910.02	9.6	TRUE	TRUE	
1	SLGADSSIA	680	688	9	HLA-A*02:01	680	9.7	3949.05	9.7	3949.05	9.7	TRUE	TRUE	
1	KMSECVLGQ	1010	1018	9	HLA-A*02:01	1010	9.8	4047.12	9.8	4047.12	9.8	TRUE	TRUE	
1	IAIVMVTIL	1207	1215	9	HLA-A*02:01	1207	9.9	4092.03	9.9	4092.03	9.9	TRUE	TRUE	
1	FAMQMAYRF	880	888	9	HLA-A*02:01	880	11	4263.5	11	4263.5	11	TRUE	TRUE	
1	VAYTMSLGA	675	683	9	HLA-A*02:01	675	12	4409.45	12	4409.45	12	TRUE	TRUE	
1	VFNGTSWFI	1078	1086	9	HLA-A*02:01	1078	12	4422.26	12	4422.26	12	TRUE	TRUE	
1	IKWPWYVWL	1192	1200	9	HLA-A*02:01	1192	12	4469.74	12	4469.74	12	TRUE	TRUE	
1	GAALQIPFA	873	881	9	HLA-A*02:01	873	12	4492.48	12	4492.48	12	TRUE	TRUE	
1	ASYHTVSLL	658	666	9	HLA-A*02:01	658	12	4557.1	12	4557.1	12	TRUE	TRUE	
1	TILLCCMTS	1213	1221	9	HLA-A*02:01	1213	12	4600.05	12	4600.05	12	TRUE	TRUE	
1	NMYICGDST	721	729	9	HLA-A*02:01	721	12	4715.71	12	4715.71	12	TRUE	TRUE	
1	ELGKYEQYI	1184	1192	9	HLA-A*02:01	1184	12	4787.32	12	4787.32	12	TRUE	TRUE	
1	YGFYTTTGI	481	489	9	HLA-A*02:01	481	12	4807.2	12	4807.2	12	TRUE	TRUE	
1	GINITNFRA	225	233	9	HLA-A*02:01	225	12	4954.26	12	4954.26	12	TRUE	TRUE	
1	FSQILPDPL	784	792	9	HLA-A*02:01	784	13	5056.67	13	5056.67	13	TRUE	TRUE	
1	QQLIRAAEI	992	1000	9	HLA-A*02:01	992	13	5078.32	13	5078.32	13	TRUE	TRUE	
1	LLCCMTSCC	1215	1223	9	HLA-A*02:01	1215	13	5179.09	13	5179.09	13	TRUE	TRUE	
1	PALNCYWPL	470	478	9	HLA-A*02:01	470	13	5293.19	13	5293.19	13	TRUE	TRUE	
1	NTREVFAQV	759	767	9	HLA-A*02:01	759	13	5448.1	13	5448.1	13	TRUE	TRUE	
1	AGLIAIVMV	1204	1212	9	HLA-A*02:01	1204	13	5595.06	13	5595.06	13	TRUE	TRUE	
1	TLYLTQDLF	51	59	9	HLA-A*02:01	51	14	5741.19	14	5741.19	14	TRUE	TRUE	
1	IFLLFLTLT	3	11	9	HLA-A*02:01	3	14	5801.83	14	5801.83	14	TRUE	TRUE	
1	FLYVYKGYQ	193	201	9	HLA-A*02:01	193	14	5906.21	14	5906.21	14	TRUE	TRUE	
1	KEIDRLNEV	1163	1171	9	HLA-A*02:01	1163	14	6029.01	14	6029.01	14	TRUE	TRUE	
1	ILDISPCSF	570	578	9	HLA-A*02:01	570	14	6194.59	14	6194.59	14	TRUE	TRUE	
1	SNFGAISSV	950	958	9	HLA-A*02:01	950	14	6207.81	14	6207.81	14	TRUE	TRUE	
1	NGLTVLPPL	838	846	9	HLA-A*02:01	838	14	6240.94	14	6240.94	14	TRUE	TRUE	
1	GLTGTGVLT	531	539	9	HLA-A*02:01	531	14	6273.84	14	6273.84	14	TRUE	TRUE	
1	TALGKLQDV	925	933	9	HLA-A*02:01	925	14	6323.53	14	6323.53	14	TRUE	TRUE	
1	FAATEKSNV	89	97	9	HLA-A*02:01	89	15	6445.23	15	6445.23	15	TRUE	TRUE	
1	NAQALNTLV	937	945	9	HLA-A*02:01	937	15	6555.94	15	6555.94	15	TRUE	TRUE	
1	VTILLCCMT	1212	1220	9	HLA-A*02:01	1212	15	6570.5	15	6570.5	15	TRUE	TRUE	
1	DISGINASV	1150	1158	9	HLA-A*02:01	1150	15	6745.18	15	6745.18	15	TRUE	TRUE	
1	FKLPLGINI	220	228	9	HLA-A*02:01	220	15	6821.73	15	6821.73	15	TRUE	TRUE	
1	DLPSGFNTL	208	216	9	HLA-A*02:01	208	15	6837.1	15	6837.1	15	TRUE	TRUE	
1	AQDIWGTSA	241	249	9	HLA-A*02:01	241	15	6897.95	15	6897.95	15	TRUE	TRUE	
1	CSFGGVSVI	576	584	9	HLA-A*02:01	576	15	7045.19	15	7045.19	15	TRUE	TRUE	
1	VMPVSMAKT	708	716	9	HLA-A*02:01	708	15	7054.87	15	7054.87	15	TRUE	TRUE	
1	STFFSTFKC	358	366	9	HLA-A*02:01	358	16	7194.17	16	7194.17	16	TRUE	TRUE	
1	KSFEIDKGI	291	299	9	HLA-A*02:01	291	16	7219.35	16	7219.35	16	TRUE	TRUE	
1	STECANLLL	728	736	9	HLA-A*02:01	728	16	7497.25	16	7497.25	16	TRUE	TRUE	
1	MSLGADSSI	679	687	9	HLA-A*02:01	679	16	7625.78	16	7625.78	16	TRUE	TRUE	
1	LQDVVNQNA	930	938	9	HLA-A*02:01	930	16	7661.76	16	7661.76	16	TRUE	TRUE	
1	DVSDFTDSV	554	562	9	HLA-A*02:01	554	17	7938.29	17	7938.29	17	TRUE	TRUE	
1	LTQDLFLPF	54	62	9	HLA-A*02:01	54	17	8106.15	17	8106.15	17	TRUE	TRUE	
1	VQIDRLITG	973	981	9	HLA-A*02:01	973	17	8170.43	17	8170.43	17	TRUE	TRUE	
1	DLCFSNVYA	376	384	9	HLA-A*02:01	376	17	8197.17	17	8197.17	17	TRUE	TRUE	
1	FIEDLLFNK	799	807	9	HLA-A*02:01	799	17	8286.79	17	8286.79	17	TRUE	TRUE	
1	DLLFNKVTL	802	810	9	HLA-A*02:01	802	17	8350.43	17	8350.43	17	TRUE	TRUE	
1	DIWGTSAAA	243	251	9	HLA-A*02:01	243	17	8398.91	17	8398.91	17	TRUE	TRUE	
1	FFSPQIITT	1091	1099	9	HLA-A*02:01	1091	17	8456.81	17	8456.81	17	TRUE	TRUE	
1	DMIAAYTAA	850	858	9	HLA-A*02:01	850	17	8489.72	17	8489.72	17	TRUE	TRUE	
1	ILTAFSPAQ	234	242	9	HLA-A*02:01	234	17	8494.96	17	8494.96	17	TRUE	TRUE	
1	QIQESLTTT	915	923	9	HLA-A*02:01	915	17	8616.79	17	8616.79	17	TRUE	TRUE	
1	AFNCTFEYI	156	164	9	HLA-A*02:01	156	17	8641.06	17	8641.06	17	TRUE	TRUE	
1	YFPREGVFV	1070	1078	9	HLA-A*02:01	1070	18	8723.82	18	8723.82	18	TRUE	TRUE	
1	CVNFNFNGL	524	532	9	HLA-A*02:01	524	18	8779.02	18	8779.02	18	TRUE	TRUE	
1	PIFKLPLGI	218	226	9	HLA-A*02:01	218	18	9026.57	18	9026.57	18	TRUE	TRUE	
1	KLRPFERDI	447	455	9	HLA-A*02:01	447	18	9035.84	18	9035.84	18	TRUE	TRUE	
1	FVGYLKPTT	253	261	9	HLA-A*02:01	253	18	9139.98	18	9139.98	18	TRUE	TRUE	
1	SQKSIVAYT	670	678	9	HLA-A*02:01	670	18	9150.26	18	9150.26	18	TRUE	TRUE	
1	IAGLIAIVM	1203	1211	9	HLA-A*02:01	1203	18	9186.67	18	9186.67	18	TRUE	TRUE	
1	SISITTEVM	701	709	9	HLA-A*02:01	701	18	9359.54	18	9359.54	18	TRUE	TRUE	
1	ALNCYWPLN	471	479	9	HLA-A*02:01	471	18	9365.42	18	9365.42	18	TRUE	TRUE	
1	QTYVTQQLI	987	995	9	HLA-A*02:01	987	19	9449.7	19	9449.7	19	TRUE	TRUE	
1	KAYFPREGV	1068	1076	9	HLA-A*02:01	1068	19	9501.68	19	9501.68	19	TRUE	TRUE	
1	FRSDTLYLT	47	55	9	HLA-A*02:01	47	19	9602.76	19	9602.76	19	TRUE	TRUE	
1	CASYHTVSL	657	665	9	HLA-A*02:01	657	19	9770.02	19	9770.02	19	TRUE	TRUE	
1	FAVSKPMGT	138	146	9	HLA-A*02:01	138	19	9917.21	19	9917.21	19	TRUE	TRUE	
1	YECDIPIGA	646	654	9	HLA-A*02:01	646	19	9971.65	19	9971.65	19	TRUE	TRUE	
1	SVIIINNST	113	121	9	HLA-A*02:01	113	19	10133.05	19	10133.05	19	TRUE	TRUE	
1	SQIQESLTT	914	922	9	HLA-A*02:01	914	20	10249.72	20	10249.72	20	TRUE	TRUE	
1	KCSVKSFEI	287	295	9	HLA-A*02:01	287	20	10279.26	20	10279.26	20	TRUE	TRUE	
1	YICGDSTEC	723	731	9	HLA-A*02:01	723	20	10316.7	20	10316.7	20	TRUE	TRUE	
1	GCLIGAEHV	634	642	9	HLA-A*02:01	634	20	10325.63	20	10325.63	20	TRUE	TRUE	
1	AQKFNGLTV	834	842	9	HLA-A*02:01	834	20	10368.17	20	10368.17	20	TRUE	TRUE	
1	QIPFAMQMA	877	885	9	HLA-A*02:01	877	20	10423.73	20	10423.73	20	TRUE	TRUE	
1	FGAGAALQI	870	878	9	HLA-A*02:01	870	20	10636.91	20	10636.91	20	TRUE	TRUE	
1	ALSGIAAEQ	748	756	9	HLA-A*02:01	748	20	10705.48	20	10705.48	20	TRUE	TRUE	
1	NITNFRAIL	227	235	9	HLA-A*02:01	227	20	10801.48	20	10801.48	20	TRUE	TRUE	
1	YFVGYLKPT	252	260	9	HLA-A*02:01	252	20	10835.89	20	10835.89	20	TRUE	TRUE	
1	SVVNIQKEI	1157	1165	9	HLA-A*02:01	1157	21	11039.07	21	11039.07	21	TRUE	TRUE	
1	VTLADAGFM	808	816	9	HLA-A*02:01	808	21	11049.95	21	11049.95	21	TRUE	TRUE	
1	KNKDGFLYV	188	196	9	HLA-A*02:01	188	21	11074.97	21	11074.97	21	TRUE	TRUE	

seq #	peptide	start	end	peptide length	allele	peptide index	median binding	ann IC50	ann percentile	legacy IC50	legacy rank	scores equal	percentiles equal
1	GVSVITPGT	580	588	9	HLA-A*02:01	580	21	11201.74	21	11201.74	21	TRUE	TRUE
1	FPQAAPHGV	1034	1042	9	HLA-A*02:01	1034	21	11255.93	21	11255.93	21	TRUE	TRUE
1	FDEDDSEPV	1238	1246	9	HLA-A*02:01	1238	21	11257.51	21	11257.51	21	TRUE	TRUE
1	RAAEIRASA	996	1004	9	HLA-A*02:01	996	21	11342.37	21	11342.37	21	TRUE	TRUE
1	ISPCSFGGV	573	581	9	HLA-A*02:01	573	21	11554.05	21	11554.05	21	TRUE	TRUE
1	NLCPFGEVF	321	329	9	HLA-A*02:01	321	21	11598.13	21	11598.13	21	TRUE	TRUE
1	SVDCNMYIC	717	725	9	HLA-A*02:01	717	22	11729.76	22	11729.76	22	TRUE	TRUE
1	AATEKSNVV	90	98	9	HLA-A*02:01	90	22	11753.9	22	11753.9	22	TRUE	TRUE
1	SVLYNSTFF	353	361	9	HLA-A*02:01	353	22	11760.01	22	11760.01	22	TRUE	TRUE
1	FNFNGLTGT	527	535	9	HLA-A*02:01	527	22	11889.74	22	11889.74	22	TRUE	TRUE
1	RVVPSGDVV	306	314	9	HLA-A*02:01	306	22	11982.21	22	11982.21	22	TRUE	TRUE
1	CVLGQSKRV	1014	1022	9	HLA-A*02:01	1014	22	12186.56	22	12186.56	22	TRUE	TRUE
1	WYVWLGFIA	1196	1204	9	HLA-A*02:01	1196	22	12220.1	22	12220.1	22	TRUE	TRUE
1	SMAKTSVDC	712	720	9	HLA-A*02:01	712	22	12272.58	22	12272.58	22	TRUE	TRUE
1	GIGVTQNVL	890	898	9	HLA-A*02:01	890	23	12465.29	23	12465.29	23	TRUE	TRUE
1	TQDLFLPFY	55	63	9	HLA-A*02:01	55	23	12502.84	23	12502.84	23	TRUE	TRUE
1	WPWYVWLGF	1194	1202	9	HLA-A*02:01	1194	23	12528.43	23	12528.43	23	TRUE	TRUE
1	LYLTQDLFL	52	60	9	HLA-A*02:01	52	23	12607.43	23	12607.43	23	TRUE	TRUE
1	GNCDVVIGI	1106	1114	9	HLA-A*02:01	1106	23	12675.55	23	12675.55	23	TRUE	TRUE
1	ALQIPFAMQ	875	883	9	HLA-A*02:01	875	23	12721.72	23	12721.72	23	TRUE	TRUE
1	FCGKGYHLM	1024	1032	9	HLA-A*02:01	1024	23	12753.41	23	12753.41	23	TRUE	TRUE
1	YHLMSFPQA	1029	1037	9	HLA-A*02:01	1029	23	12764.45	23	12764.45	23	TRUE	TRUE
1	LQSLQTYVT	983	991	9	HLA-A*02:01	983	23	12769.85	23	12769.85	23	TRUE	TRUE
1	NLLLQYGSF	733	741	9	HLA-A*02:01	733	23	12869.44	23	12869.44	23	TRUE	TRUE
1	PLLTDDMIA	845	853	9	HLA-A*02:01	845	23	12869.44	23	12869.44	23	TRUE	TRUE
1	LGFIAGLIA	1200	1208	9	HLA-A*02:01	1200	23	12989.46	23	12989.46	23	TRUE	TRUE
1	STGNNVFQT	623	631	9	HLA-A*02:01	623	24	13116.85	24	13116.85	24	TRUE	TRUE
1	YTMSLGADS	677	685	9	HLA-A*02:01	677	24	13160.35	24	13160.35	24	TRUE	TRUE
1	LNDLCFSNV	374	382	9	HLA-A*02:01	374	24	13337.68	24	13337.68	24	TRUE	TRUE
1	DILSRLDKV	961	969	9	HLA-A*02:01	961	24	13420.04	24	13420.04	24	TRUE	TRUE
1	AGWTFGAGA	866	874	9	HLA-A*02:01	866	24	13569.85	24	13569.85	24	TRUE	TRUE
1	AATKMSECV	1007	1015	9	HLA-A*02:01	1007	24	13572.35	24	13572.35	24	TRUE	TRUE
1	VVNQNAQAL	933	941	9	HLA-A*02:01	933	24	13609.4	24	13609.4	24	TRUE	TRUE
1	HLREFVFKN	181	189	9	HLA-A*02:01	181	24	13623.84	24	13623.84	24	TRUE	TRUE
1	CSQNPLAEL	278	286	9	HLA-A*02:01	278	24	13747.62	24	13747.62	24	TRUE	TRUE
1	FCTQLNRAL	741	749	9	HLA-A*02:01	741	24	13758.94	24	13758.94	24	TRUE	TRUE
1	RLITGRLQS	977	985	9	HLA-A*02:01	977	24	13798.74	24	13798.74	24	TRUE	TRUE
1	SGFNTLKPI	211	219	9	HLA-A*02:01	211	24	13799.49	24	13799.49	24	TRUE	TRUE
1	HTMIFDNAF	149	157	9	HLA-A*02:01	149	25	13940.56	25	13940.56	25	TRUE	TRUE
1	LLNAPATVC	503	511	9	HLA-A*02:01	503	25	13968.64	25	13968.64	25	TRUE	TRUE
1	GGFNFSQIL	780	788	9	HLA-A*02:01	780	25	14001.63	25	14001.63	25	TRUE	TRUE
1	IAYSNNTIA	687	695	9	HLA-A*02:01	687	25	14156.55	25	14156.55	25	TRUE	TRUE
1	TLADAGFMK	809	817	9	HLA-A*02:01	809	25	14227.49	25	14227.49	25	TRUE	TRUE
1	QIANQFNKA	904	912	9	HLA-A*02:01	904	25	14253.52	25	14253.52	25	TRUE	TRUE
1	IFRSDTLYL	46	54	9	HLA-A*02:01	46	25	14269.57	25	14269.57	25	TRUE	TRUE
1	RGWVFGSTM	99	107	9	HLA-A*02:01	99	25	14422.78	25	14422.78	25	TRUE	TRUE
1	KLQDVVNQN	929	937	9	HLA-A*02:01	929	26	14583.15	26	14583.15	26	TRUE	TRUE
1	CPFGEVFNA	323	331	9	HLA-A*02:01	323	26	14616.32	26	14616.32	26	TRUE	TRUE
1	MLKYDENGT	263	271	9	HLA-A*02:01	263	26	14673.52	26	14673.52	26	TRUE	TRUE
1	FKNHTSPDV	1138	1146	9	HLA-A*02:01	1138	26	14692.27	26	14692.27	26	TRUE	TRUE
1	VYDPLQPEL	1119	1127	9	HLA-A*02:01	1119	26	14708.49	26	14708.49	26	TRUE	TRUE
1	IYQTSNFRV	299	307	9	HLA-A*02:01	299	26	14767.02	26	14767.02	26	TRUE	TRUE
1	VVRGWVFGS	97	105	9	HLA-A*02:01	97	26	14834.27	26	14834.27	26	TRUE	TRUE
1	VSGNCDVVI	1104	1112	9	HLA-A*02:01	1104	26	14932.5	26	14932.5	26	TRUE	TRUE
1	IEDLLFNKV	800	808	9	HLA-A*02:01	800	26	14990.3	26	14990.3	26	TRUE	TRUE
1	NASSEVAVL	589	597	9	HLA-A*02:01	589	26	15001.98	26	15001.98	26	TRUE	TRUE
1	PWYVWLGFI	1195	1203	9	HLA-A*02:01	1195	26	15025.04	26	15025.04	26	TRUE	TRUE
1	LSFELLNAP	499	507	9	HLA-A*02:01	499	26	15054.17	26	15054.17	26	TRUE	TRUE
1	KGYQPIDVV	198	206	9	HLA-A*02:01	198	26	15107.21	26	15107.21	26	TRUE	TRUE
1	ITNFRAILT	228	236	9	HLA-A*02:01	228	26	15172.4	26	15172.4	26	TRUE	TRUE
1	NCDVVIGII	1107	1115	9	HLA-A*02:01	1107	26	15178.31	26	15178.31	26	TRUE	TRUE
1	YADSFVVKG	383	391	9	HLA-A*02:01	383	26	15190.79	26	15190.79	26	TRUE	TRUE
1	AAYFVGYLK	250	258	9	HLA-A*02:01	250	26	15222.23	26	15222.23	26	TRUE	TRUE
1	ITTEVMPVS	704	712	9	HLA-A*02:01	704	27	15266.09	27	15266.09	27	TRUE	TRUE
1	STAIHADQL	607	615	9	HLA-A*02:01	607	27	15277.66	27	15277.66	27	TRUE	TRUE
1	SLQTYVTQQ	985	993	9	HLA-A*02:01	985	27	15487.54	27	15487.54	27	TRUE	TRUE
1	LPDDFMGCV	412	420	9	HLA-A*02:01	412	27	15509.17	27	15509.17	27	TRUE	TRUE
1	ICAQKFNGL	832	840	9	HLA-A*02:01	832	27	15524.28	27	15524.28	27	TRUE	TRUE
1	RSFIEDLLF	797	805	9	HLA-A*02:01	797	27	15557.23	27	15557.23	27	TRUE	TRUE
1	FSLDVSEKS	168	176	9	HLA-A*02:01	168	27	15627.92	27	15627.92	27	TRUE	TRUE
1	NGIGVTQNV	889	897	9	HLA-A*02:01	889	27	15660.25	27	15660.25	27	TRUE	TRUE
1	DLFLPFYSN	57	65	9	HLA-A*02:01	57	27	15671.27	27	15671.27	27	TRUE	TRUE
1	LTVLPPLLT	840	848	9	HLA-A*02:01	840	27	15684.84	27	15684.84	27	TRUE	TRUE
1	LIRAAEIRA	994	1002	9	HLA-A*02:01	994	27	15695.36	27	15695.36	27	TRUE	TRUE
1	KWPWYVWLG	1193	1201	9	HLA-A*02:01	1193	27	15755.43	27	15755.43	27	TRUE	TRUE
1	CCMTSCCSC	1217	1225	9	HLA-A*02:01	1217	27	15758.83	27	15758.83	27	TRUE	TRUE
1	YSVLYNSTF	352	360	9	HLA-A*02:01	352	27	15759.18	27	15759.18	27	TRUE	TRUE
1	NAFNCTFEY	155	163	9	HLA-A*02:01	155	27	15839.69	27	15839.69	27	TRUE	TRUE
1	NIQKEIDRL	1160	1168	9	HLA-A*02:01	1160	28	15911.67	28	15911.67	28	TRUE	TRUE
1	LKPIFKLPL	216	224	9	HLA-A*02:01	216	28	15981.2	28	15981.2	28	TRUE	TRUE
1	FNGLTGTGV	529	537	9	HLA-A*02:01	529	28	16061.8	28	16061.8	28	TRUE	TRUE
1	VTQQLIRAA	990	998	9	HLA-A*02:01	990	28	16105.66	28	16105.66	28	TRUE	TRUE
1	STSQKSIVA	668	676	9	HLA-A*02:01	668	28	16136	28	16136	28	TRUE	TRUE
1	FGGFNFSQI	779	787	9	HLA-A*02:01	779	28	16136.18	28	16136.18	28	TRUE	TRUE
1	YSNVTGFHT	63	71	9	HLA-A*02:01	63	28	16241.45	28	16241.45	28	TRUE	TRUE
1	AQVKQMYKT	765	773	9	HLA-A*02:01	765	28	16403.57	28	16403.57	28	TRUE	TRUE
1	INHTFGNPV	72	80	9	HLA-A*02:01	72	28	16426.31	28	16426.31	28	TRUE	TRUE
1	KLSTDLIKN	514	522	9	HLA-A*02:01	514	28	16471.51	28	16471.51	28	TRUE	TRUE
1	LQYGSFCTQ	736	744	9	HLA-A*02:01	736	28	16475.79	28	16475.79	28	TRUE	TRUE
1	TNFRAILTA	229	237	9	HLA-A*02:01	229	28	16482.03	28	16482.03	28	TRUE	TRUE
1	NSTNVVIRA	119	127	9	HLA-A*02:01	119	28	16593.15	28	16593.15	28	TRUE	TRUE
1	DQLTPAWRI	613	621	9	HLA-A*02:01	613	29	16621.54	29	16621.54	29	TRUE	TRUE
1	ISSVLNDIL	955	963	9	HLA-A*02:01	955	29	16683.15	29	16683.15	29	TRUE	TRUE
1	ISGINASVV	1151	1159	9	HLA-A*02:01	1151	29	16723.28	29	16723.28	29	TRUE	TRUE
1	VRFPNITNL	314	322	9	HLA-A*02:01	314	29	16857.71	29	16857.71	29	TRUE	TRUE
1	QKFNGLTVL	835	843	9	HLA-A*02:01	835	29	16919.84	29	16919.84	29	TRUE	TRUE
1	IPTNFSISI	696	704	9	HLA-A*02:01	696	29	16964.38	29	16964.38	29	TRUE	TRUE
1	YIKWPWYVW	1191	1199	9	HLA-A*02:01	1191	29	17003.34	29	17003.34	29	TRUE	TRUE
1	SFELLNAPA	500	508	9	HLA-A*02:01	500	29	17238.6	29	17238.6	29	TRUE	TRUE
1	KTSEILDIS	566	574	9	HLA-A*02:01	566	30	17276.51	30	17276.51	30	TRUE	TRUE
1	RAILTAFSP	232	240	9	HLA-A*02:01	232	30	17324.99	30	17324.99	30	TRUE	TRUE
1	RASANLAAT	1001	1009	9	HLA-A*02:01	1001	30	17348.62	30	17348.62	30	TRUE	TRUE

seq #	peptide	start	end	peptide length	allele	peptide index	median binding	ann IC50	ann percentile	legacy IC50	legacy rank	scores equal	percentiles equal
1	MGCVLAWNT	417	425	9	HLA-A*02:01	417	30	17484.31	30	17484.31	30	TRUE	TRUE
1	STNVVIRAC	120	128	9	HLA-A*02:01	120	30	17494.33	30	17494.33	30	TRUE	TRUE
1	FRAILTAFS	231	239	9	HLA-A*02:01	231	30	17540.01	30	17540.01	30	TRUE	TRUE
1	GVFVFNGTS	1075	1083	9	HLA-A*02:01	1075	30	17587.14	30	17587.14	30	TRUE	TRUE
1	NCTDVSTAI	602	610	9	HLA-A*02:01	602	30	17630.39	30	17630.39	30	TRUE	TRUE
1	IANQFNKAI	905	913	9	HLA-A*02:01	905	30	17632.87	30	17632.87	30	TRUE	TRUE
1	GADSSIAYS	682	690	9	HLA-A*02:01	682	30	17677.38	30	17677.38	30	TRUE	TRUE
1	ATKFPSVYA	331	339	9	HLA-A*02:01	331	30	17684.07	30	17684.07	30	TRUE	TRUE
1	YKGYQPIDV	197	205	9	HLA-A*02:01	197	30	17741.19	30	17741.19	30	TRUE	TRUE
1	SSMRGVYYP	35	43	9	HLA-A*02:01	35	30	17887.29	30	17887.29	30	TRUE	TRUE
1	GVVFLHVTY	1041	1049	9	HLA-A*02:01	1041	31	17901.22	31	17901.22	31	TRUE	TRUE
1	YRVVVLSFE	494	502	9	HLA-A*02:01	494	31	18002.03	31	18002.03	31	TRUE	TRUE
1	SVITPGTNA	582	590	9	HLA-A*02:01	582	31	18160.3	31	18160.3	31	TRUE	TRUE
1	CVADYSVLY	348	356	9	HLA-A*02:01	348	31	18163.24	31	18163.24	31	TRUE	TRUE
1	HGVVFLHVT	1040	1048	9	HLA-A*02:01	1040	31	18214.8	31	18214.8	31	TRUE	TRUE
1	SMRGVYYPD	36	44	9	HLA-A*02:01	36	31	18303.31	31	18303.31	31	TRUE	TRUE
1	SNCVADYSV	346	354	9	HLA-A*02:01	346	31	18476.62	31	18476.62	31	TRUE	TRUE
1	FNGLTVLPP	837	845	9	HLA-A*02:01	837	32	18547.33	32	18547.33	32	TRUE	TRUE
1	LTDDMIAAY	847	855	9	HLA-A*02:01	847	32	18571.42	32	18571.42	32	TRUE	TRUE
1	EVAVLYQDV	593	601	9	HLA-A*02:01	593	32	18618.09	32	18618.09	32	TRUE	TRUE
1	ESLIDLQEL	1177	1185	9	HLA-A*02:01	1177	32	18638.46	32	18638.46	32	TRUE	TRUE
1	QKSIVAYTM	671	679	9	HLA-A*02:01	671	32	18678.62	32	18678.62	32	TRUE	TRUE
1	TMIFDNAFN	150	158	9	HLA-A*02:01	150	32	18736.71	32	18736.71	32	TRUE	TRUE
1	LTLTSGSDL	8	16	9	HLA-A*02:01	8	32	18833.46	32	18833.46	32	TRUE	TRUE
1	NVFQTQAGC	627	635	9	HLA-A*02:01	627	32	18867.94	32	18867.94	32	TRUE	TRUE
1	FKHLREFVF	179	187	9	HLA-A*02:01	179	32	18887.33	32	18887.33	32	TRUE	TRUE
1	PLGINITNF	223	231	9	HLA-A*02:01	223	32	18949.15	32	18949.15	32	TRUE	TRUE
1	VLKGVKLHY	1246	1254	9	HLA-A*02:01	1246	32	18978.49	32	18978.49	32	TRUE	TRUE
1	SQERNFTTA	1052	1060	9	HLA-A*02:01	1052	32	18978.7	32	18978.7	32	TRUE	TRUE
1	GTATAGWTF	862	870	9	HLA-A*02:01	862	32	19075.05	32	19075.05	32	TRUE	TRUE
1	FPREGVFVF	1071	1079	9	HLA-A*02:01	1071	32	19145.96	32	19145.96	32	TRUE	TRUE
1	DDFMGCVLA	414	422	9	HLA-A*02:01	414	33	19239.01	33	19239.01	33	TRUE	TRUE
1	VSATKLNDL	369	377	9	HLA-A*02:01	369	33	19298.01	33	19298.01	33	TRUE	TRUE
1	SCCSCLKGA	1221	1229	9	HLA-A*02:01	1221	33	19298.42	33	19298.42	33	TRUE	TRUE
1	KSNVVRGWV	94	102	9	HLA-A*02:01	94	33	19318.48	33	19318.48	33	TRUE	TRUE
1	VVLSFELLN	497	505	9	HLA-A*02:01	497	33	19418.86	33	19418.86	33	TRUE	TRUE
1	ASSEVAVLY	590	598	9	HLA-A*02:01	590	33	19447.87	33	19447.87	33	TRUE	TRUE
1	LMSFPQAAP	1031	1039	9	HLA-A*02:01	1031	33	19502.44	33	19502.44	33	TRUE	TRUE
1	TAALVSGTA	856	864	9	HLA-A*02:01	856	33	19532.65	33	19532.65	33	TRUE	TRUE
1	FEYISDAFS	161	169	9	HLA-A*02:01	161	33	19569.46	33	19569.46	33	TRUE	TRUE
1	SVLNDILSR	957	965	9	HLA-A*02:01	957	33	19677.1	33	19677.1	33	TRUE	TRUE
1	FSNVYADSF	379	387	9	HLA-A*02:01	379	33	19687.32	33	19687.32	33	TRUE	TRUE
1	LTPAWRIYS	615	623	9	HLA-A*02:01	615	33	19751.97	33	19751.97	33	TRUE	TRUE
1	SQSVIIINN	111	119	9	HLA-A*02:01	111	34	19817.48	34	19817.48	34	TRUE	TRUE
1	TTSTALGKL	922	930	9	HLA-A*02:01	922	34	19868.36	34	19868.36	34	TRUE	TRUE
1	FELCDNPFF	130	138	9	HLA-A*02:01	130	34	19881.25	34	19881.25	34	TRUE	TRUE
1	YFGGFNFSQ	778	786	9	HLA-A*02:01	778	34	19948.27	34	19948.27	34	TRUE	TRUE
1	DFMGCVLAW	415	423	9	HLA-A*02:01	415	34	20000.57	34	20000.57	34	TRUE	TRUE
1	TAFSPAQDI	236	244	9	HLA-A*02:01	236	34	20332.01	34	20332.01	34	TRUE	TRUE
1	TNFSISITT	698	706	9	HLA-A*02:01	698	35	20403.19	35	20403.19	35	TRUE	TRUE
1	KISNCVADY	344	352	9	HLA-A*02:01	344	35	20411.79	35	20411.79	35	TRUE	TRUE
1	ITTDNTFVS	1097	1105	9	HLA-A*02:01	1097	35	20447.83	35	20447.83	35	TRUE	TRUE
1	RNFTTAPAI	1055	1063	9	HLA-A*02:01	1055	35	20541.18	35	20541.18	35	TRUE	TRUE
1	IHADQLTPA	610	618	9	HLA-A*02:01	610	35	20551.63	35	20551.63	35	TRUE	TRUE
1	CANLLLQYG	731	739	9	HLA-A*02:01	731	35	20566.76	35	20566.76	35	TRUE	TRUE
1	NITNLCPFG	318	326	9	HLA-A*02:01	318	35	20657.74	35	20657.74	35	TRUE	TRUE
1	NVVIRACNF	122	130	9	HLA-A*02:01	122	35	20683.01	35	20683.01	35	TRUE	TRUE
1	YNSTFFSTF	356	364	9	HLA-A*02:01	356	35	20820.89	35	20820.89	35	TRUE	TRUE
1	YISDAFSLD	163	171	9	HLA-A*02:01	163	35	20839.81	35	20839.81	35	TRUE	TRUE
1	EIDKGIYQT	294	302	9	HLA-A*02:01	294	35	20874.11	35	20874.11	35	TRUE	TRUE
1	SFIEDLLFN	798	806	9	HLA-A*02:01	798	36	20919.11	36	20919.11	36	TRUE	TRUE
1	TTEVMPVSM	705	713	9	HLA-A*02:01	705	36	20919.11	36	20919.11	36	TRUE	TRUE
1	AYSNNTIAI	688	696	9	HLA-A*02:01	688	36	20948.33	36	20948.33	36	TRUE	TRUE
1	SAAAYFVGY	248	256	9	HLA-A*02:01	248	36	20956.49	36	20956.49	36	TRUE	TRUE
1	FGGVSVITP	578	586	9	HLA-A*02:01	578	36	21023.71	36	21023.71	36	TRUE	TRUE
1	IVAYTMSLG	674	682	9	HLA-A*02:01	674	36	21038.28	36	21038.28	36	TRUE	TRUE
1	PHGVVFLHV	1039	1047	9	HLA-A*02:01	1039	36	21063.09	36	21063.09	36	TRUE	TRUE
1	KYFGGFNFS	777	785	9	HLA-A*02:01	777	36	21087.51	36	21087.51	36	TRUE	TRUE
1	TMSLGADSS	678	686	9	HLA-A*02:01	678	36	21103.48	36	21103.48	36	TRUE	TRUE
1	FVFKNKDGF	185	193	9	HLA-A*02:01	185	36	21125.18	36	21125.18	36	TRUE	TRUE
1	SNVTGFHTI	64	72	9	HLA-A*02:01	64	36	21127.24	36	21127.24	36	TRUE	TRUE
1	QMYKTPTLK	769	777	9	HLA-A*02:01	769	36	21182.63	36	21182.63	36	TRUE	TRUE
1	IINNTVYDP	1114	1122	9	HLA-A*02:01	1114	36	21192.48	36	21192.48	36	TRUE	TRUE
1	FSPDGKPCT	460	468	9	HLA-A*02:01	460	36	21197.76	36	21197.76	36	TRUE	TRUE
1	FDNAFNCTF	153	161	9	HLA-A*02:01	153	36	21212.68	36	21212.68	36	TRUE	TRUE
1	AAYTAALVS	853	861	9	HLA-A*02:01	853	36	21229.66	36	21229.66	36	TRUE	TRUE
1	FNGTSWFIT	1079	1087	9	HLA-A*02:01	1079	36	21236.78	36	21236.78	36	TRUE	TRUE
1	LYNSTFFST	355	363	9	HLA-A*02:01	355	36	21282.56	36	21282.56	36	TRUE	TRUE
1	INNTVYDPL	1115	1123	9	HLA-A*02:01	1115	36	21442.75	36	21442.75	36	TRUE	TRUE
1	NTIAIPTNF	692	700	9	HLA-A*02:01	692	36	21445.29	36	21445.29	36	TRUE	TRUE
1	LKYFGGFNF	776	784	9	HLA-A*02:01	776	36	21449.47	36	21449.47	36	TRUE	TRUE
1	ADYSVLYNS	350	358	9	HLA-A*02:01	350	37	21505.25	37	21505.25	37	TRUE	TRUE
1	FLTLTSGSD	7	15	9	HLA-A*02:01	7	37	21544.6	37	21544.6	37	TRUE	TRUE
1	MTSCCSCLK	1219	1227	9	HLA-A*02:01	1219	37	21595.01	37	21595.01	37	TRUE	TRUE
1	FQPFQQFGR	545	553	9	HLA-A*02:01	545	37	21801.37	37	21801.37	37	TRUE	TRUE
1	VFLHVTYVP	1043	1051	9	HLA-A*02:01	1043	37	21929.13	37	21929.13	37	TRUE	TRUE
1	IPFKDGIYF	81	89	9	HLA-A*02:01	81	37	22014.23	37	22014.23	37	TRUE	TRUE
1	QYGSFCTQL	737	745	9	HLA-A*02:01	737	38	22157.85	38	22157.85	38	TRUE	TRUE
1	CDIPIGAGI	648	656	9	HLA-A*02:01	648	38	22171.28	38	22171.28	38	TRUE	TRUE
1	ATKMSECVL	1008	1016	9	HLA-A*02:01	1008	38	22175.6	38	22175.6	38	TRUE	TRUE
1	NFGAISSVL	951	959	9	HLA-A*02:01	951	38	22196.23	38	22196.23	38	TRUE	TRUE
1	HTVSLLRST	661	669	9	HLA-A*02:01	661	38	22285.99	38	22285.99	38	TRUE	TRUE
1	AGAALQIPF	872	880	9	HLA-A*02:01	872	38	22418.29	38	22418.29	38	TRUE	TRUE
1	QDLFLPFYS	56	64	9	HLA-A*02:01	56	38	22507.74	38	22507.74	38	TRUE	TRUE
1	KCYGVSATK	365	373	9	HLA-A*02:01	365	38	22514.3	38	22514.3	38	TRUE	TRUE
1	VAKNLNESL	1171	1179	9	HLA-A*02:01	1171	38	22543.31	38	22543.31	38	TRUE	TRUE
1	QSVIIINNS	112	120	9	HLA-A*02:01	112	38	22566.25	38	22566.25	38	TRUE	TRUE
1	SFGGVSVIT	577	585	9	HLA-A*02:01	577	38	22588.96	38	22588.96	38	TRUE	TRUE
1	NFRAILTAF	230	238	9	HLA-A*02:01	230	38	22628.84	38	22628.84	38	TRUE	TRUE
1	CLGDINARD	822	830	9	HLA-A*02:01	822	39	22643.78	39	22643.78	39	TRUE	TRUE
1	GIYQTSNFR	298	306	9	HLA-A*02:01	298	39	22662.42	39	22662.42	39	TRUE	TRUE
1	GQTGVIADY	400	408	9	HLA-A*02:01	400	39	22737.81	39	22737.81	39	TRUE	TRUE

seq #	peptide	start	end	peptide length	allele	peptide index	median binding	ann IC50	ann percentile	legacy IC50	legacy rank	scores equal	percentiles equal	
1	WVFGSTMNN	101	109	9	HLA-A*02:01	101	39	22782.39	39	22782.39	39	TRUE	TRUE	
1	PMGTQTHTM	143	151	9	HLA-A*02:01	143	39	22784.86	39	22784.86	39	TRUE	TRUE	
1	FELLNAPAT	501	509	9	HLA-A*02:01	501	39	22796.44	39	22796.44	39	TRUE	TRUE	
1	QPYRVVVLS	492	500	9	HLA-A*02:01	492	39	22831.25	39	22831.25	39	TRUE	TRUE	
1	GTSWFITQR	1081	1089	9	HLA-A*02:01	1081	39	22934.24	39	22934.24	39	TRUE	TRUE	
1	IGYQPYRVV	489	497	9	HLA-A*02:01	489	39	22954.84	39	22954.84	39	TRUE	TRUE	
1	TLKPIFKLP	215	223	9	HLA-A*02:01	215	39	23087.61	39	23087.61	39	TRUE	TRUE	
1	TNLCPFGEV	320	328	9	HLA-A*02:01	320	39	23102.36	39	23102.36	39	TRUE	TRUE	
1	KVTLADAGF	807	815	9	HLA-A*02:01	807	39	23159.67	39	23159.67	39	TRUE	TRUE	
1	FNFSQILPD	782	790	9	HLA-A*02:01	782	40	23189.76	40	23189.76	40	TRUE	TRUE	
1	VDLGDISGI	1146	1154	9	HLA-A*02:01	1146	40	23234.21	40	23234.21	40	TRUE	TRUE	
1	CTTFDDVQA	19	27	9	HLA-A*02:01	19	40	23283.28	40	23283.28	40	TRUE	TRUE	
1	VTQNVLYEN	893	901	9	HLA-A*02:01	893	40	23365.3	40	23365.3	40	TRUE	TRUE	
1	RNFFSPQII	1089	1097	9	HLA-A*02:01	1089	40	23369.09	40	23369.09	40	TRUE	TRUE	
1	EQYIKWPWY	1189	1197	9	HLA-A*02:01	1189	40	23404.77	40	23404.77	40	TRUE	TRUE	
1	GTNASSEVA	587	595	9	HLA-A*02:01	587	40	23454.2	40	23454.2	40	TRUE	TRUE	
1	AYRFNGIGV	885	893	9	HLA-A*02:01	885	40	23472.23	40	23472.23	40	TRUE	TRUE	
1	FGNPVIPFK	76	84	9	HLA-A*02:01	76	40	23501.19	40	23501.19	40	TRUE	TRUE	
1	YLRHGKLRP	442	450	9	HLA-A*02:01	442	40	23509.85	40	23509.85	40	TRUE	TRUE	
1	FNKVTLADA	805	813	9	HLA-A*02:01	805	40	23598.53	40	23598.53	40	TRUE	TRUE	
1	VLTPSSKRF	537	545	9	HLA-A*02:01	537	40	23675.51	40	23675.51	40	TRUE	TRUE	
1	YSNNTIAIP	689	697	9	HLA-A*02:01	689	41	23733.48	41	23733.48	41	TRUE	TRUE	
1	QLTPAWRIY	614	622	9	HLA-A*02:01	614	41	23738.87	41	23738.87	41	TRUE	TRUE	
1	TFVSGNCDV	1102	1110	9	HLA-A*02:01	1102	41	23803.95	41	23803.95	41	TRUE	TRUE	
1	VLPPLLTDD	842	850	9	HLA-A*02:01	842	41	23812.18	41	23812.18	41	TRUE	TRUE	
1	SSIAYSNNT	685	693	9	HLA-A*02:01	685	41	23840.54	41	23840.54	41	TRUE	TRUE	
1	EQDRNTREV	755	763	9	HLA-A*02:01	755	41	23909.52	41	23909.52	41	TRUE	TRUE	
1	SNVVRGWVF	95	103	9	HLA-A*02:01	95	41	23953.54	41	23953.54	41	TRUE	TRUE	
1	ICGDSTECA	724	732	9	HLA-A*02:01	724	41	23965.71	41	23965.71	41	TRUE	TRUE	
1	SNVYADSFV	380	388	9	HLA-A*02:01	380	41	24017.38	41	24017.38	41	TRUE	TRUE	
1	AVLYQDVNC	595	603	9	HLA-A*02:01	595	41	24028.54	41	24028.54	41	TRUE	TRUE	
1	FFSTFKCYG	360	368	9	HLA-A*02:01	360	41	24035.57	41	24035.57	41	TRUE	TRUE	
1	PYRVVVLSF	493	501	9	HLA-A*02:01	493	41	24115.02	41	24115.02	41	TRUE	TRUE	
1	APHGVVFLH	1038	1046	9	HLA-A*02:01	1038	42	24249.51	42	24249.51	42	TRUE	TRUE	
1	ISNCVADYS	345	353	9	HLA-A*02:01	345	42	24266.04	42	24266.04	42	TRUE	TRUE	
1	YEQYIKWPW	1188	1196	9	HLA-A*02:01	1188	42	24270.51	42	24270.51	42	TRUE	TRUE	
1	EILDISPCS	569	577	9	HLA-A*02:01	569	42	24271.04	42	24271.04	42	TRUE	TRUE	
1	GQSKRVDFC	1017	1025	9	HLA-A*02:01	1017	42	24274.18	42	24274.18	42	TRUE	TRUE	
1	SEILDISPC	568	576	9	HLA-A*02:01	568	42	24300.47	42	24300.47	42	TRUE	TRUE	
1	YWPLNDYGF	475	483	9	HLA-A*02:01	475	42	24304.4	42	24304.4	42	TRUE	TRUE	
1	GYQPYRVVV	490	498	9	HLA-A*02:01	490	42	24362.86	42	24362.86	42	TRUE	TRUE	
1	INITNFRAI	226	234	9	HLA-A*02:01	226	42	24450.26	42	24450.26	42	TRUE	TRUE	
1	KRSFIEDLL	796	804	9	HLA-A*02:01	796	42	24463.23	42	24463.23	42	TRUE	TRUE	
1	EYISDAFSL	162	170	9	HLA-A*02:01	162	42	24469.58	42	24469.58	42	TRUE	TRUE	
1	IPIGAGICA	650	658	9	HLA-A*02:01	650	42	24485.46	42	24485.46	42	TRUE	TRUE	
1	GIYFAATEK	86	94	9	HLA-A*02:01	86	42	24518.35	42	24518.35	42	TRUE	TRUE	
1	ITNLCPFGE	319	327	9	HLA-A*02:01	319	42	24643.61	42	24643.61	42	TRUE	TRUE	
1	NKSQSVIII	109	117	9	HLA-A*02:01	109	42	24658.54	42	24658.54	42	TRUE	TRUE	
1	IPFAMQMAY	878	886	9	HLA-A*02:01	878	42	24674.56	42	24674.56	42	TRUE	TRUE	
1	DSTECANLL	727	735	9	HLA-A*02:01	727	43	24740.59	43	24740.59	43	TRUE	TRUE	
1	YYPDEIFRS	41	49	9	HLA-A*02:01	41	43	24746.75	43	24746.75	43	TRUE	TRUE	
1	KDGIYFAAT	84	92	9	HLA-A*02:01	84	43	24766.84	43	24766.84	43	TRUE	TRUE	
1	QIITTDNTF	1095	1103	9	HLA-A*02:01	1095	43	24771.39	43	24771.39	43	TRUE	TRUE	
1	IGAGICASY	652	660	9	HLA-A*02:01	652	43	24799.83	43	24799.83	43	TRUE	TRUE	
1	KQYGECLGD	817	825	9	HLA-A*02:01	817	43	24814.85	43	24814.85	43	TRUE	TRUE	
1	IQESLTTTS	916	924	9	HLA-A*02:01	916	43	24817.54	43	24817.54	43	TRUE	TRUE	
1	LIKNQCVNF	519	527	9	HLA-A*02:01	519	43	24920.33	43	24920.33	43	TRUE	TRUE	
1	INARDLICA	826	834	9	HLA-A*02:01	826	43	24958.64	43	24958.64	43	TRUE	TRUE	
1	EVFAQVKQM	762	770	9	HLA-A*02:01	762	43	24977.28	43	24977.28	43	TRUE	TRUE	
1	TFGNPVIPF	75	83	9	HLA-A*02:01	75	43	24984.58	43	24984.58	43	TRUE	TRUE	
1	GVYYPDEIF	39	47	9	HLA-A*02:01	39	43	25003.78	43	25003.78	43	TRUE	TRUE	
1	LTGTGVLTP	532	540	9	HLA-A*02:01	532	43	25018.39	43	25018.39	43	TRUE	TRUE	
1	PLQPELDSF	1122	1130	9	HLA-A*02:01	1122	43	25026.51	43	25026.51	43	TRUE	TRUE	
1	LNDYGFYTT	478	486	9	HLA-A*02:01	478	43	25027.33	43	25027.33	43	TRUE	TRUE	
1	TNASSEVAV	588	596	9	HLA-A*02:01	588	43	25035.73	43	25035.73	43	TRUE	TRUE	
1	TIAIPTNFS	693	701	9	HLA-A*02:01	693	43	25042.49	43	25042.49	43	TRUE	TRUE	
1	ICHEGKAYF	1063	1071	9	HLA-A*02:01	1063	43	25121.74	43	25121.74	43	TRUE	TRUE	
1	SANLAATKM	1003	1011	9	HLA-A*02:01	1003	44	25191.42	44	25191.42	44	TRUE	TRUE	
1	NQNAQALNT	935	943	9	HLA-A*02:01	935	44	25212.69	44	25212.69	44	TRUE	TRUE	
1	KNQCVNFNF	521	529	9	HLA-A*02:01	521	44	25221.43	44	25221.43	44	TRUE	TRUE	
1	STFKCYGVS	362	370	9	HLA-A*02:01	362	44	25229.61	44	25229.61	44	TRUE	TRUE	
1	QLIRAAEIR	993	1001	9	HLA-A*02:01	993	44	25251.46	44	25251.46	44	TRUE	TRUE	
1	KSQSVIIIN	110	118	9	HLA-A*02:01	110	44	25261.57	44	25261.57	44	TRUE	TRUE	
1	YRFNGIGVT	886	894	9	HLA-A*02:01	886	44	25356.32	44	25356.32	44	TRUE	TRUE	
1	SWFITQRNF	1083	1091	9	HLA-A*02:01	1083	44	25376.08	44	25376.08	44	TRUE	TRUE	
1	FNCTFEYIS	157	165	9	HLA-A*02:01	157	44	25384.04	44	25384.04	44	TRUE	TRUE	
1	FKCYGVSAT	364	372	9	HLA-A*02:01	364	44	25388.98	44	25388.98	44	TRUE	TRUE	
1	FPSVYAWER	334	342	9	HLA-A*02:01	334	44	25390.08	44	25390.08	44	TRUE	TRUE	
1	GTQTHTMIF	145	153	9	HLA-A*02:01	145	44	25429.95	44	25429.95	44	TRUE	TRUE	
1	SFCTQLNRA	740	748	9	HLA-A*02:01	740	44	25434.9	44	25434.9	44	TRUE	TRUE	
1	VITPGTNAS	583	591	9	HLA-A*02:01	583	44	25457.2	44	25457.2	44	TRUE	TRUE	
1	NQFNKAISQ	907	915	9	HLA-A*02:01	907	44	25462.72	44	25462.72	44	TRUE	TRUE	
1	MSECVLGQS	1011	1019	9	HLA-A*02:01	1011	44	25473.74	44	25473.74	44	TRUE	TRUE	
1	SSEVAVLYQ	591	599	9	HLA-A*02:01	591	44	25490.27	44	25490.27	44	TRUE	TRUE	
1	AQALNTLVK	938	946	9	HLA-A*02:01	938	44	25491.37	44	25491.37	44	TRUE	TRUE	
1	PFFAVSKPM	136	144	9	HLA-A*02:01	136	44	25491.37	44	25491.37	44	TRUE	TRUE	
1	REGVFVFNG	1073	1081	9	HLA-A*02:01	1073	44	25500.48	44	25500.48	44	TRUE	TRUE	
1	SSVLNDILS	956	964	9	HLA-A*02:01	956	44	25608.59	44	25608.59	44	TRUE	TRUE	
1	AMQMAYRFN	881	889	9	HLA-A*02:01	881	45	25625.23	45	25625.23	45	TRUE	TRUE	
1	VGYLKPTTF	254	262	9	HLA-A*02:01	254	45	25654.35	45	25654.35	45	TRUE	TRUE	
1	LIGAEHVDT	636	644	9	HLA-A*02:01	636	45	25695.74	45	25695.74	45	TRUE	TRUE	
1	VADYSVLYN	349	357	9	HLA-A*02:01	349	45	25729.12	45	25729.12	45	TRUE	TRUE	
1	ITQRNFFSP	1086	1094	9	HLA-A*02:01	1086	45	25732.19	45	25732.19	45	TRUE	TRUE	
1	NKDGFLYVY	189	197	9	HLA-A*02:01	189	45	25813.9	45	25813.9	45	TRUE	TRUE	
1	FGRDVSDFT	551	559	9	HLA-A*02:01	551	45	25822.56	45	25822.56	45	TRUE	TRUE	
1	CLKGACSCG	1225	1233	9	HLA-A*02:01	1225	45	25829.26	45	25829.26	45	TRUE	TRUE	
1	DTLYLTQDL	50	58	9	HLA-A*02:01	50	45	25831.77	45	25831.77	45	TRUE	TRUE	
1	TTFDDVQAP	20	28	9	HLA-A*02:01	20	45	25888.57	45	25888.57	45	TRUE	TRUE	
1	TDDMIAAYT	848	856	9	HLA-A*02:01	848	45	25956.16	45	25956.16	45	TRUE	TRUE	
1	FSPQIITTD	1092	1100	9	HLA-A*02:01	1092	45	25962.63	45	25962.63	45	TRUE	TRUE	
1	QNAQALNTL	936	944	9	HLA-A*02:01	936	45	25973.03	45	25973.03	45	TRUE	TRUE	
1	CSCGSCCKF	1230	1238	9	HLA-A*02:01	1230	46	26097.54	46	26097.54	46	TRUE	TRUE	

seq #	peptide	start	end	peptide length	allele	peptide index	median binding	ann IC50	ann percentile	legacy IC50	legacy rank	scores equal	percentiles equal	
1	LCFSNVYAD	377	385	9	HLA-A*02:01	377	46	26128.61	46	26128.61	46	TRUE	TRUE	
1	NFELCDNPF	129	137	9	HLA-A*02:01	129	46	26153.79	46	26153.79	46	TRUE	TRUE	
1	NVVRGWVFG	96	104	9	HLA-A*02:01	96	46	26163.97	46	26163.97	46	TRUE	TRUE	
1	KAISQIQES	911	919	9	HLA-A*02:01	911	46	26201.66	46	26201.66	46	TRUE	TRUE	
1	EVMPVSMAK	707	715	9	HLA-A*02:01	707	46	26207.61	46	26207.61	46	TRUE	TRUE	
1	TQLNRALSG	743	751	9	HLA-A*02:01	743	46	26252.46	46	26252.46	46	TRUE	TRUE	
1	IDRLITGRL	975	983	9	HLA-A*02:01	975	46	26304.77	46	26304.77	46	TRUE	TRUE	
1	LCCMTSCCS	1216	1224	9	HLA-A*02:01	1216	46	26320.99	46	26320.99	46	TRUE	TRUE	
1	SPAQDIWGT	239	247	9	HLA-A*02:01	239	46	26342.09	46	26342.09	46	TRUE	TRUE	
1	DSEPVLKGV	1242	1250	9	HLA-A*02:01	1242	46	26372.02	46	26372.02	46	TRUE	TRUE	
1	QYGECLGDI	818	826	9	HLA-A*02:01	818	46	26378.87	46	26378.87	46	TRUE	TRUE	
1	FGEVFNATK	325	333	9	HLA-A*02:01	325	46	26393.42	46	26393.42	46	TRUE	TRUE	
1	GYHLMSFPQ	1028	1036	9	HLA-A*02:01	1028	46	26455.47	46	26455.47	46	TRUE	TRUE	
1	AEVQIDRLI	971	979	9	HLA-A*02:01	971	46	26456.61	46	26456.61	46	TRUE	TRUE	
1	IRACNFELC	125	133	9	HLA-A*02:01	125	46	26460.33	46	26460.33	46	TRUE	TRUE	
1	LGDISGINA	1148	1156	9	HLA-A*02:01	1148	46	26533.44	46	26533.44	46	TRUE	TRUE	
1	RDLICAQKF	829	837	9	HLA-A*02:01	829	47	26602.73	47	26602.73	47	TRUE	TRUE	
1	LLRSTSQKS	665	673	9	HLA-A*02:01	665	47	26617.12	47	26617.12	47	TRUE	TRUE	
1	ITDAVDCSQ	272	280	9	HLA-A*02:01	272	47	26618.27	47	26618.27	47	TRUE	TRUE	
1	IFDNAFNCT	152	160	9	HLA-A*02:01	152	47	26625.76	47	26625.76	47	TRUE	TRUE	
1	INNSTNVVI	117	125	9	HLA-A*02:01	117	47	26634.4	47	26634.4	47	TRUE	TRUE	
1	ELKCSVKSF	285	293	9	HLA-A*02:01	285	47	26646.22	47	26646.22	47	TRUE	TRUE	
1	NCVADYSVL	347	355	9	HLA-A*02:01	347	47	26658.34	47	26658.34	47	TRUE	TRUE	
1	YKTPTLKYF	771	779	9	HLA-A*02:01	771	47	26702.51	47	26702.51	47	TRUE	TRUE	
1	TSNFRVVPS	302	310	9	HLA-A*02:01	302	47	26719.56	47	26719.56	47	TRUE	TRUE	
1	TVYDPLQPE	1118	1126	9	HLA-A*02:01	1118	47	26763.81	47	26763.81	47	TRUE	TRUE	
1	EIDRLNEVA	1164	1172	9	HLA-A*02:01	1164	47	26770.47	47	26770.47	47	TRUE	TRUE	
1	GCVLAWNTR	418	426	9	HLA-A*02:01	418	47	26795.69	47	26795.69	47	TRUE	TRUE	
1	SYHTVSLLR	659	667	9	HLA-A*02:01	659	47	26881.06	47	26881.06	47	TRUE	TRUE	
1	QPIDVVRDL	201	209	9	HLA-A*02:01	201	47	26923.86	47	26923.86	47	TRUE	TRUE	
1	YTTTGIGYQ	484	492	9	HLA-A*02:01	484	47	26932.3	47	26932.3	47	TRUE	TRUE	
1	TVLPPLLTD	841	849	9	HLA-A*02:01	841	47	26932.89	47	26932.89	47	TRUE	TRUE	
1	GFLYVYKGY	192	200	9	HLA-A*02:01	192	47	26952.42	47	26952.42	47	TRUE	TRUE	
1	TKFPSVYAW	332	340	9	HLA-A*02:01	332	47	26960.59	47	26960.59	47	TRUE	TRUE	
1	GYLKPTTFM	255	263	9	HLA-A*02:01	255	48	26974	48	26974	48	TRUE	TRUE	
1	WGTSAAAYF	245	253	9	HLA-A*02:01	245	48	26974.89	48	26974.89	48	TRUE	TRUE	
1	ATKLNDLCF	371	379	9	HLA-A*02:01	371	48	26988.6	48	26988.6	48	TRUE	TRUE	
1	GDVVRFPNI	311	319	9	HLA-A*02:01	311	48	26991.82	48	26991.82	48	TRUE	TRUE	
1	TTDNTFVSG	1098	1106	9	HLA-A*02:01	1098	48	26994.74	48	26994.74	48	TRUE	TRUE	
1	FAQVKQMYK	764	772	9	HLA-A*02:01	764	48	27026.59	48	27026.59	48	TRUE	TRUE	
1	SNNTIAIPT	690	698	9	HLA-A*02:01	690	48	27088.95	48	27088.95	48	TRUE	TRUE	
1	KQIANQFNK	903	911	9	HLA-A*02:01	903	48	27104.49	48	27104.49	48	TRUE	TRUE	
1	YHTVSLLRS	660	668	9	HLA-A*02:01	660	48	27108	48	27108	48	TRUE	TRUE	
1	AGICASYHT	654	662	9	HLA-A*02:01	654	48	27116.22	48	27116.22	48	TRUE	TRUE	
1	KTSVDCNMY	715	723	9	HLA-A*02:01	715	48	27118.28	48	27118.28	48	TRUE	TRUE	
1	YQPIDVVRD	200	208	9	HLA-A*02:01	200	48	27125.91	48	27125.91	48	TRUE	TRUE	
1	HTFGNPVIP	74	82	9	HLA-A*02:01	74	48	27167.31	48	27167.31	48	TRUE	TRUE	
1	KSIVAYTMS	672	680	9	HLA-A*02:01	672	48	27234.13	48	27234.13	48	TRUE	TRUE	
1	AIHADQLTP	609	617	9	HLA-A*02:01	609	48	27240.91	48	27240.91	48	TRUE	TRUE	
1	GGVSVITPG	579	587	9	HLA-A*02:01	579	48	27304.35	48	27304.35	48	TRUE	TRUE	
1	IRASANLAA	1000	1008	9	HLA-A*02:01	1000	48	27393.42	48	27393.42	48	TRUE	TRUE	
1	SQNPLAELK	279	287	9	HLA-A*02:01	279	48	27395.49	48	27395.49	48	TRUE	TRUE	
1	CDVVIGIIN	1108	1116	9	HLA-A*02:01	1108	49	27453.06	49	27453.06	49	TRUE	TRUE	
1	DDMIAAYTA	849	857	9	HLA-A*02:01	849	49	27516.69	49	27516.69	49	TRUE	TRUE	
1	KFNGLTVLP	836	844	9	HLA-A*02:01	836	49	27519.37	49	27519.37	49	TRUE	TRUE	
1	TGFHTINHT	67	75	9	HLA-A*02:01	67	49	27520.56	49	27520.56	49	TRUE	TRUE	
1	RGVYYPDEI	38	46	9	HLA-A*02:01	38	49	27529.51	49	27529.51	49	TRUE	TRUE	
1	NFFSPQIIT	1090	1098	9	HLA-A*02:01	1090	49	27581.68	49	27581.68	49	TRUE	TRUE	
1	GIINNTVYD	1113	1121	9	HLA-A*02:01	1113	49	27614.52	49	27614.52	49	TRUE	TRUE	
1	DGFLYVYKG	191	199	9	HLA-A*02:01	191	49	27625.28	49	27625.28	49	TRUE	TRUE	
1	FTDSVRDPK	558	566	9	HLA-A*02:01	558	49	27644.12	49	27644.12	49	TRUE	TRUE	
1	TEVMPVSMA	706	714	9	HLA-A*02:01	706	49	27646.81	49	27646.81	49	TRUE	TRUE	
1	NFKHLREFV	178	186	9	HLA-A*02:01	178	49	27665.96	49	27665.96	49	TRUE	TRUE	
1	MSFPQAAPH	1032	1040	9	HLA-A*02:01	1032	49	27676.13	49	27676.13	49	TRUE	TRUE	
1	GFHTINHTF	68	76	9	HLA-A*02:01	68	49	27677.33	49	27677.33	49	TRUE	TRUE	
1	EGVFVFNGT	1074	1082	9	HLA-A*02:01	1074	49	27694.7	49	27694.7	49	TRUE	TRUE	
1	AYFVGYLKP	251	259	9	HLA-A*02:01	251	49	27730.7	49	27730.7	49	TRUE	TRUE	
1	NCYWPLNDY	473	481	9	HLA-A*02:01	473	49	27759.81	49	27759.81	49	TRUE	TRUE	
1	ECANLLLQY	730	738	9	HLA-A*02:01	730	49	27775.73	49	27775.73	49	TRUE	TRUE	
1	LIDLQELGK	1179	1187	9	HLA-A*02:01	1179	49	27789.56	49	27789.56	49	TRUE	TRUE	
1	TFFSTFKCY	359	367	9	HLA-A*02:01	359	49	27791.96	49	27791.96	49	TRUE	TRUE	
1	GKPCTPPAL	464	472	9	HLA-A*02:01	464	50	27833.79	50	27833.79	50	TRUE	TRUE	
1	FYTTTGIGY	483	491	9	HLA-A*02:01	483	50	27836.81	50	27836.81	50	TRUE	TRUE	
1	VVIRACNFE	123	131	9	HLA-A*02:01	123	50	27893.79	50	27893.79	50	TRUE	TRUE	
1	SLLRSTSQK	664	672	9	HLA-A*02:01	664	50	27932.15	50	27932.15	50	TRUE	TRUE	
1	ARDLICAQK	828	836	9	HLA-A*02:01	828	50	27992.06	50	27992.06	50	TRUE	TRUE	
1	TSSMRGVYY	34	42	9	HLA-A*02:01	34	50	28010.53	50	28010.53	50	TRUE	TRUE	
1	IAPGQTGVI	397	405	9	HLA-A*02:01	397	50	28014.16	50	28014.16	50	TRUE	TRUE	
1	SFVVKGDDV	386	394	9	HLA-A*02:01	386	50	28124.71	50	28124.71	50	TRUE	TRUE	
1	MGTQTHTMI	144	152	9	HLA-A*02:01	144	50	28143.29	50	28143.29	50	TRUE	TRUE	
1	YRYLRHGKL	440	448	9	HLA-A*02:01	440	50	28152.73	50	28152.73	50	TRUE	TRUE	
1	ITPGTNASS	584	592	9	HLA-A*02:01	584	50	28153.33	50	28153.33	50	TRUE	TRUE	
1	AALVSGTAT	857	865	9	HLA-A*02:01	857	51	28262.9	51	28262.9	51	TRUE	TRUE	
1	GVTQNVLYE	892	900	9	HLA-A*02:01	892	51	28264.12	51	28264.12	51	TRUE	TRUE	
1	LKGVKLHYT	1247	1255	9	HLA-A*02:01	1247	51	28305.75	51	28305.75	51	TRUE	TRUE	
1	CLIGAEHVD	635	643	9	HLA-A*02:01	635	51	28318.62	51	28318.62	51	TRUE	TRUE	
1	TKRSFIEDL	795	803	9	HLA-A*02:01	795	51	28320.15	51	28320.15	51	TRUE	TRUE	
1	TLVKQLSSN	943	951	9	HLA-A*02:01	943	51	28337	51	28337	51	TRUE	TRUE	
1	LKYDENGTI	264	272	9	HLA-A*02:01	264	51	28355.41	51	28355.41	51	TRUE	TRUE	
1	TECANLLLQ	729	737	9	HLA-A*02:01	729	51	28365.83	51	28365.83	51	TRUE	TRUE	
1	QFNKAISQI	908	916	9	HLA-A*02:01	908	51	28375.05	51	28375.05	51	TRUE	TRUE	
1	FNTLKPIFK	213	221	9	HLA-A*02:01	213	51	28399	51	28399	51	TRUE	TRUE	
1	STDLIKNQC	516	524	9	HLA-A*02:01	516	51	28417.45	51	28417.45	51	TRUE	TRUE	
1	TPAWRIYST	616	624	9	HLA-A*02:01	616	51	28460.22	51	28460.22	51	TRUE	TRUE	
1	QRNFFSPQI	1088	1096	9	HLA-A*02:01	1088	51	28504.91	51	28504.91	51	TRUE	TRUE	
1	FRVVPSGDV	305	313	9	HLA-A*02:01	305	51	28510.15	51	28510.15	51	TRUE	TRUE	
1	TVCGPKLST	509	517	9	HLA-A*02:01	509	51	28543.17	51	28543.17	51	TRUE	TRUE	
1	EIRASANLA	999	1007	9	HLA-A*02:01	999	51	28550.28	51	28550.28	51	TRUE	TRUE	
1	NDYGFYTTT	479	487	9	HLA-A*02:01	479	51	28561.71	51	28561.71	51	TRUE	TRUE	
1	KRFQPFQQF	543	551	9	HLA-A*02:01	543	51	28583.66	51	28583.66	51	TRUE	TRUE	
1	SCLKGACSC	1224	1232	9	HLA-A*02:01	1224	51	28589.23	51	28589.23	51	TRUE	TRUE	
1	CYWPLNDYG	474	482	9	HLA-A*02:01	474	52	28640	52	28640	52	TRUE	TRUE	

seq #	peptide	start	end	peptide length	allele	peptide index	median binding	ann IC50	ann percentile	legacy IC50	legacy rank	scores equal	percentiles equal	
1	DISNVPFSP	454	462	9	HLA-A*02:01	454	52	28664.5	52	28664.5	52	TRUE	TRUE	
1	RFPNITNLC	315	323	9	HLA-A*02:01	315	52	28681.25	52	28681.25	52	TRUE	TRUE	
1	GKGYHLMSF	1026	1034	9	HLA-A*02:01	1026	52	28695.83	52	28695.83	52	TRUE	TRUE	
1	NLAATKMSE	1005	1013	9	HLA-A*02:01	1005	52	28723.47	52	28723.47	52	TRUE	TRUE	
1	KPTTFMLKY	258	266	9	HLA-A*02:01	258	52	28725.66	52	28725.66	52	TRUE	TRUE	
1	NQCVNFNFN	522	530	9	HLA-A*02:01	522	52	28739.02	52	28739.02	52	TRUE	TRUE	
1	CTPPALNCY	467	475	9	HLA-A*02:01	467	52	28741.21	52	28741.21	52	TRUE	TRUE	
1	QILPDPLKP	786	794	9	HLA-A*02:01	786	52	28770.45	52	28770.45	52	TRUE	TRUE	
1	RSDTLYLTQ	48	56	9	HLA-A*02:01	48	52	28777.91	52	28777.91	52	TRUE	TRUE	
1	YKLPDDFMG	410	418	9	HLA-A*02:01	410	52	28812.51	52	28812.51	52	TRUE	TRUE	
1	NDLCFSNVY	375	383	9	HLA-A*02:01	375	52	28828.1	52	28828.1	52	TRUE	TRUE	
1	VVRDLPSGF	205	213	9	HLA-A*02:01	205	52	28871.49	52	28871.49	52	TRUE	TRUE	
1	RALSGIAAE	747	755	9	HLA-A*02:01	747	52	28907.43	52	28907.43	52	TRUE	TRUE	
1	FPNITNLCP	316	324	9	HLA-A*02:01	316	53	28975.07	53	28975.07	53	TRUE	TRUE	
1	VRGWVFGST	98	106	9	HLA-A*02:01	98	53	29024.65	53	29024.65	53	TRUE	TRUE	
1	TDVSTAIHA	604	612	9	HLA-A*02:01	604	53	29031.24	53	29031.24	53	TRUE	TRUE	
1	GSFCTQLNR	739	747	9	HLA-A*02:01	739	53	29050.72	53	29050.72	53	TRUE	TRUE	
1	AEIRASANL	998	1006	9	HLA-A*02:01	998	53	29065.5	53	29065.5	53	TRUE	TRUE	
1	SDLDRCTTF	14	22	9	HLA-A*02:01	14	53	29070.21	53	29070.21	53	TRUE	TRUE	
1	LICAQKFNG	831	839	9	HLA-A*02:01	831	53	29108.29	53	29108.29	53	TRUE	TRUE	
1	FSPAQDIWG	238	246	9	HLA-A*02:01	238	53	29147.7	53	29147.7	53	TRUE	TRUE	
1	QCVNFNFNG	523	531	9	HLA-A*02:01	523	53	29163.46	53	29163.46	53	TRUE	TRUE	
1	CDNPFFAVS	133	141	9	HLA-A*02:01	133	53	29209.89	53	29209.89	53	TRUE	TRUE	
1	KGYHLMSFP	1027	1035	9	HLA-A*02:01	1027	53	29221.27	53	29221.27	53	TRUE	TRUE	
1	FKEELDKYF	1130	1138	9	HLA-A*02:01	1130	53	29251.31	53	29251.31	53	TRUE	TRUE	
1	THTMIFDNA	148	156	9	HLA-A*02:01	148	53	29252.59	53	29252.59	53	TRUE	TRUE	
1	LSSNFGAIS	948	956	9	HLA-A*02:01	948	53	29264.61	53	29264.61	53	TRUE	TRUE	
1	LQPELDSFK	1123	1131	9	HLA-A*02:01	1123	53	29286.15	53	29286.15	53	TRUE	TRUE	
1	TLKYFGGFN	775	783	9	HLA-A*02:01	775	53	29288.68	53	29288.68	53	TRUE	TRUE	
1	SEVAVLYQD	592	600	9	HLA-A*02:01	592	53	29296.92	53	29296.92	53	TRUE	TRUE	
1	TFKCYGVSA	363	371	9	HLA-A*02:01	363	53	29307.39	53	29307.39	53	TRUE	TRUE	
1	TTGIGYQPY	486	494	9	HLA-A*02:01	486	54	29348.32	54	29348.32	54	TRUE	TRUE	
1	GTGVLTPSS	534	542	9	HLA-A*02:01	534	54	29349.91	54	29349.91	54	TRUE	TRUE	
1	TFEYISDAF	160	168	9	HLA-A*02:01	160	54	29355.3	54	29355.3	54	TRUE	TRUE	
1	TQRNFFSPQ	1087	1095	9	HLA-A*02:01	1087	54	29378.83	54	29378.83	54	TRUE	TRUE	
1	KDGFLYVYK	190	198	9	HLA-A*02:01	190	54	29404.59	54	29404.59	54	TRUE	TRUE	
1	AIPTNFSIS	695	703	9	HLA-A*02:01	695	54	29416.37	54	29416.37	54	TRUE	TRUE	
1	NQKQIANQF	901	909	9	HLA-A*02:01	901	54	29420.82	54	29420.82	54	TRUE	TRUE	
1	SSNFGAISS	949	957	9	HLA-A*02:01	949	54	29431.96	54	29431.96	54	TRUE	TRUE	
1	FGAISSVLN	952	960	9	HLA-A*02:01	952	54	29448.2	54	29448.2	54	TRUE	TRUE	
1	FEIDKGIYQ	293	301	9	HLA-A*02:01	293	54	29461.27	54	29461.27	54	TRUE	TRUE	
1	PCSFGGVSV	575	583	9	HLA-A*02:01	575	54	29463.18	54	29463.18	54	TRUE	TRUE	
1	QTQAGCLIG	630	638	9	HLA-A*02:01	630	54	29464.46	54	29464.46	54	TRUE	TRUE	
1	TITDAVDCS	271	279	9	HLA-A*02:01	271	54	29470.52	54	29470.52	54	TRUE	TRUE	
1	ISITTEVMP	702	710	9	HLA-A*02:01	702	54	29481.03	54	29481.03	54	TRUE	TRUE	
1	VNFNFNGLT	525	533	9	HLA-A*02:01	525	54	29496.36	54	29496.36	54	TRUE	TRUE	
1	PVIPFKDGI	79	87	9	HLA-A*02:01	79	54	29497.31	54	29497.31	54	TRUE	TRUE	
1	SLDVSEKSG	169	177	9	HLA-A*02:01	169	54	29514.23	54	29514.23	54	TRUE	TRUE	
1	FTTAPAICH	1057	1065	9	HLA-A*02:01	1057	54	29529.57	54	29529.57	54	TRUE	TRUE	
1	FKNKDGFLY	187	195	9	HLA-A*02:01	187	54	29570.81	54	29570.81	54	TRUE	TRUE	
1	GECLGDINA	820	828	9	HLA-A*02:01	820	54	29579.45	54	29579.45	54	TRUE	TRUE	
1	CYGVSATKL	366	374	9	HLA-A*02:01	366	54	29625.88	54	29625.88	54	TRUE	TRUE	
1	CNFELCDNP	128	136	9	HLA-A*02:01	128	54	29648.97	54	29648.97	54	TRUE	TRUE	
1	DFCGKGYHL	1023	1031	9	HLA-A*02:01	1023	54	29661.17	54	29661.17	54	TRUE	TRUE	
1	GRLQSLQTY	981	989	9	HLA-A*02:01	981	54	29702.92	54	29702.92	54	TRUE	TRUE	
1	LRHGKLRPF	443	451	9	HLA-A*02:01	443	54	29702.92	54	29702.92	54	TRUE	TRUE	
1	LVKQLSSNF	944	952	9	HLA-A*02:01	944	55	29725.11	55	29725.11	55	TRUE	TRUE	
1	RVDFCGKGY	1021	1029	9	HLA-A*02:01	1021	55	29757.6	55	29757.6	55	TRUE	TRUE	
1	DVVIGIINN	1109	1117	9	HLA-A*02:01	1109	55	29811.1	55	29811.1	55	TRUE	TRUE	
1	LGINITNFR	224	232	9	HLA-A*02:01	224	55	29924.53	55	29924.53	55	TRUE	TRUE	
1	LPPLLTDDM	843	851	9	HLA-A*02:01	843	55	29935.23	55	29935.23	55	TRUE	TRUE	
1	DISPCSFGG	572	580	9	HLA-A*02:01	572	55	29948.49	55	29948.49	55	TRUE	TRUE	
1	QIDRLITGR	974	982	9	HLA-A*02:01	974	55	29966.98	55	29966.98	55	TRUE	TRUE	
1	VPSGDVVRF	308	316	9	HLA-A*02:01	308	55	29967.95	55	29967.95	55	TRUE	TRUE	
1	TSQKSIVAY	669	677	9	HLA-A*02:01	669	55	29996.51	55	29996.51	55	TRUE	TRUE	
1	VFVFNGTSW	1076	1084	9	HLA-A*02:01	1076	55	30048.8	55	30048.8	55	TRUE	TRUE	
1	QSLQTYVTQ	984	992	9	HLA-A*02:01	984	56	30072.86	56	30072.86	56	TRUE	TRUE	
1	AGCLIGAEH	633	641	9	HLA-A*02:01	633	56	30100.86	56	30100.86	56	TRUE	TRUE	
1	YAWERKKIS	338	346	9	HLA-A*02:01	338	56	30141.6	56	30141.6	56	TRUE	TRUE	
1	RFNGIGVTQ	887	895	9	HLA-A*02:01	887	56	30166.72	56	30166.72	56	TRUE	TRUE	
1	SQILPDPLK	785	793	9	HLA-A*02:01	785	56	30169.99	56	30169.99	56	TRUE	TRUE	
1	VFAQVKQMY	763	771	9	HLA-A*02:01	763	56	30172.6	56	30172.6	56	TRUE	TRUE	
1	GVIADYNYK	403	411	9	HLA-A*02:01	403	56	30202.66	56	30202.66	56	TRUE	TRUE	
1	LVSGTATAG	859	867	9	HLA-A*02:01	859	56	30236.33	56	30236.33	56	TRUE	TRUE	
1	LFLTLTSGS	6	14	9	HLA-A*02:01	6	56	30240.25	56	30240.25	56	TRUE	TRUE	
1	IGIINNTVY	1112	1120	9	HLA-A*02:01	1112	56	30252.03	56	30252.03	56	TRUE	TRUE	
1	LADAGFMKQ	810	818	9	HLA-A*02:01	810	56	30307.06	56	30307.06	56	TRUE	TRUE	
1	TQTHTMIFD	146	154	9	HLA-A*02:01	146	56	30327.42	56	30327.42	56	TRUE	TRUE	
1	WFITQRNFF	1084	1092	9	HLA-A*02:01	1084	56	30347.76	56	30347.76	56	TRUE	TRUE	
1	MAYRFNGIG	884	892	9	HLA-A*02:01	884	56	30357.94	56	30357.94	56	TRUE	TRUE	
1	NASVVNIQK	1155	1163	9	HLA-A*02:01	1155	56	30371.75	56	30371.75	56	TRUE	TRUE	
1	NRALSGIAA	746	754	9	HLA-A*02:01	746	56	30377.66	56	30377.66	56	TRUE	TRUE	
1	QALNTLVKQ	939	947	9	HLA-A*02:01	939	56	30397.04	5.60E+01	3.04E+04	56	TRUE	TRUE	
1	KPIFKLPLG	217	225	9	HLA-A*02:01	217	57	30437.87	5.70E+01	3.04E+04	57	TRUE	TRUE	
1	TSWFITQRN	1082	1090	9	HLA-A*02:01	1082	57	30457.96	5.70E+01	3.05E+04	57	TRUE	TRUE	
1	ANLLLQYGS	732	740	9	HLA-A*02:01	732	57	30491.27	5.70E+01	3.05E+04	57	TRUE	TRUE	
1	LGDINARDL	823	831	9	HLA-A*02:01	823	57	30499.51	5.70E+01	3.05E+04	57	TRUE	TRUE	
1	TGTGVLTPS	533	541	9	HLA-A*02:01	533	57	30540.45	5.70E+01	3.05E+04	57	TRUE	TRUE	
1	GDSTECANL	726	734	9	HLA-A*02:01	726	57	30559.64	5.70E+01	3.06E+04	57	TRUE	TRUE	
1	NSTFFSTFK	357	365	9	HLA-A*02:01	357	57	30580.14	5.70E+01	3.06E+04	57	TRUE	TRUE	
1	TAPAICHEG	1059	1067	9	HLA-A*02:01	1059	57	30642.74	5.70E+01	3.06E+04	57	TRUE	TRUE	
1	TDLIKNQCV	517	525	9	HLA-A*02:01	517	57	30660.31	5.70E+01	3.07E+04	57	TRUE	TRUE	
1	FQQFGRDVS	548	556	9	HLA-A*02:01	548	57	30674.57	5.70E+01	3.07E+04	57	TRUE	TRUE	
1	QAAPHGVVF	1036	1044	9	HLA-A*02:01	1036	57	30725.73	5.70E+01	3.07E+04	57	TRUE	TRUE	
1	NTLVKQLSS	942	950	9	HLA-A*02:01	942	57	30731.04	5.70E+01	3.07E+04	57	TRUE	TRUE	
1	SPDVDLGDI	1143	1151	9	HLA-A*02:01	1143	57	30737.37	5.70E+01	3.07E+04	57	TRUE	TRUE	
1	TSAAAYFVG	247	255	9	HLA-A*02:01	247	57	30772.65	5.70E+01	3.08E+04	57	TRUE	TRUE	
1	CAQKFNGLT	833	841	9	HLA-A*02:01	833	58	30773.64	5.80E+01	3.08E+04	58	TRUE	TRUE	
1	APGQTGVIA	398	406	9	HLA-A*02:01	398	58	30789.29	5.80E+01	3.08E+04	58	TRUE	TRUE	
1	HADQLTPAW	611	619	9	HLA-A*02:01	611	58	30797.97	5.80E+01	3.08E+04	58	TRUE	TRUE	
1	CTQLNRALS	742	750	9	HLA-A*02:01	742	58	30813.62	5.80E+01	3.08E+04	58	TRUE	TRUE	
1	YNYKLPDDF	408	416	9	HLA-A*02:01	408	58	30823.62	5.80E+01	3.08E+04	58	TRUE	TRUE	

seq #	peptide	start	end	peptide length	allele	peptide index	median binding	ann IC50	ann percentile	legacy IC50	legacy rank	scores equal	percentiles equal	
1	PNITNLCPF	317	325	9	HLA-A*02:01	317	58	30830.97	5.80E+01	3.08E+04	58	TRUE	TRUE	
1	GAGICASYH	653	661	9	HLA-A*02:01	653	58	30921.84	5.80E+01	3.09E+04	58	TRUE	TRUE	
1	KTPTLKYFG	772	780	9	HLA-A*02:01	772	58	30942.92	5.80E+01	3.09E+04	58	TRUE	TRUE	
1	GTITDAVDC	270	278	9	HLA-A*02:01	270	58	30963.34	5.80E+01	3.10E+04	58	TRUE	TRUE	
1	VRDPKTSEI	562	570	9	HLA-A*02:01	562	58	30979.1	5.80E+01	3.10E+04	58	TRUE	TRUE	
1	LKPTTFMLK	257	265	9	HLA-A*02:01	257	58	31007.26	5.80E+01	3.10E+04	58	TRUE	TRUE	
1	PVSMAKTSV	710	718	9	HLA-A*02:01	710	58	31052.59	5.80E+01	3.11E+04	58	TRUE	TRUE	
1	LFNKVTLAD	804	812	9	HLA-A*02:01	804	58	31058.64	5.80E+01	3.11E+04	58	TRUE	TRUE	
1	IGVTQNVLY	891	899	9	HLA-A*02:01	891	58	31081.5	5.80E+01	3.11E+04	58	TRUE	TRUE	
1	IWGTSAAAY	244	252	9	HLA-A*02:01	244	58	31084.19	5.80E+01	3.11E+04	58	TRUE	TRUE	
1	LSRLDKVEA	963	971	9	HLA-A*02:01	963	59	31247.39	5.90E+01	3.12E+04	59	TRUE	TRUE	
1	EPVLKGVKL	1244	1252	9	HLA-A*02:01	1244	59	31296.46	5.90E+01	3.13E+04	59	TRUE	TRUE	
1	AKTSVDCNM	714	722	9	HLA-A*02:01	714	59	31303.24	5.90E+01	3.13E+04	59	TRUE	TRUE	
1	GNFKHLREF	177	185	9	HLA-A*02:01	177	59	31333.05	5.90E+01	3.13E+04	59	TRUE	TRUE	
1	LPLGINITN	222	230	9	HLA-A*02:01	222	59	31351.71	5.90E+01	3.14E+04	59	TRUE	TRUE	
1	PFGEVFNAT	324	332	9	HLA-A*02:01	324	59	31396.51	5.90E+01	3.14E+04	59	TRUE	TRUE	
1	TQQLIRAAE	991	999	9	HLA-A*02:01	991	59	31400.25	5.90E+01	3.14E+04	59	TRUE	TRUE	
1	CFSNVYADS	378	386	9	HLA-A*02:01	378	60	31506.42	6.00E+01	3.15E+04	60	TRUE	TRUE	
1	QTHTMIFDN	147	155	9	HLA-A*02:01	147	60	31519.73	6.00E+01	3.15E+04	60	TRUE	TRUE	
1	YSTGNNVFQ	622	630	9	HLA-A*02:01	622	60	31534.74	6.00E+01	3.15E+04	60	TRUE	TRUE	
1	TSEILDISP	567	575	9	HLA-A*02:01	567	60	31543.95	6.00E+01	3.15E+04	60	TRUE	TRUE	
1	CTDVSTAIH	603	611	9	HLA-A*02:01	603	60	31564.1	6.00E+01	3.16E+04	60	TRUE	TRUE	
1	KGDDVRQIA	390	398	9	HLA-A*02:01	390	60	31581.51	6.00E+01	3.16E+04	60	TRUE	TRUE	
1	LAATKMSEC	1006	1014	9	HLA-A*02:01	1006	60	31588.01	6.00E+01	3.16E+04	60	TRUE	TRUE	
1	INASVVNIQ	1154	1162	9	HLA-A*02:01	1154	60	31603.04	6.00E+01	3.16E+04	60	TRUE	TRUE	
1	VFNATKFPS	328	336	9	HLA-A*02:01	328	60	31603.74	6.00E+01	3.16E+04	60	TRUE	TRUE	
1	TAIHADQLT	608	616	9	HLA-A*02:01	608	60	31619.13	6.00E+01	3.16E+04	60	TRUE	TRUE	
1	NYNYKYRYL	435	443	9	HLA-A*02:01	435	60	31686.25	6.00E+01	3.17E+04	60	TRUE	TRUE	
1	VYYPDEIFR	40	48	9	HLA-A*02:01	40	60	31699.61	6.00E+01	3.17E+04	60	TRUE	TRUE	
1	YKYRYLRHG	438	446	9	HLA-A*02:01	438	60	31705.46	6.00E+01	3.17E+04	60	TRUE	TRUE	
1	NGTSWFITQ	1080	1088	9	HLA-A*02:01	1080	60	31728.1	6.00E+01	3.17E+04	60	TRUE	TRUE	
1	LAELKCSVK	283	291	9	HLA-A*02:01	283	60	31732.22	6.00E+01	3.17E+04	60	TRUE	TRUE	
1	FYSNVTGFH	62	70	9	HLA-A*02:01	62	60	31744.93	6.00E+01	3.17E+04	60	TRUE	TRUE	
1	CSCLKGACS	1223	1231	9	HLA-A*02:01	1223	60	31784.79	6.00E+01	3.18E+04	60	TRUE	TRUE	
1	AYTAALVSG	854	862	9	HLA-A*02:01	854	61	31815.41	6.10E+01	3.18E+04	61	TRUE	TRUE	
1	SNFRVVPSG	303	311	9	HLA-A*02:01	303	61	31825.06	6.10E+01	3.18E+04	61	TRUE	TRUE	
1	ATSTGNYNY	430	438	9	HLA-A*02:01	430	61	31900.22	6.10E+01	3.19E+04	61	TRUE	TRUE	
1	EAEVQIDRL	970	978	9	HLA-A*02:01	970	61	31976.93	6.10E+01	3.20E+04	61	TRUE	TRUE	
1	SGDVVRFPN	310	318	9	HLA-A*02:01	310	61	31978.66	6.10E+01	3.20E+04	61	TRUE	TRUE	
1	TQNVLYENQ	894	902	9	HLA-A*02:01	894	61	31997.7	6.10E+01	3.20E+04	61	TRUE	TRUE	
1	YGSFCTQLN	738	746	9	HLA-A*02:01	738	61	32006	6.10E+01	3.20E+04	61	TRUE	TRUE	
1	GPKLSTDLI	512	520	9	HLA-A*02:01	512	61	32025.05	6.10E+01	3.20E+04	61	TRUE	TRUE	
1	TSCCSCLKG	1220	1228	9	HLA-A*02:01	1220	61	32027.82	6.10E+01	3.20E+04	61	TRUE	TRUE	
1	ERDISNVPF	452	460	9	HLA-A*02:01	452	61	32045.86	6.10E+01	3.20E+04	61	TRUE	TRUE	
1	TKLNDLCFS	372	380	9	HLA-A*02:01	372	61	32104.86	6.10E+01	3.21E+04	61	TRUE	TRUE	
1	VNCTDVSTA	601	609	9	HLA-A*02:01	601	61	32123.62	6.10E+01	3.21E+04	61	TRUE	TRUE	
1	YVPSQERNF	1049	1057	9	HLA-A*02:01	1049	62	32175.1	6.20E+01	3.22E+04	62	TRUE	TRUE	
1	PIGAGICAS	651	659	9	HLA-A*02:01	651	62	32266.78	6.20E+01	3.23E+04	62	TRUE	TRUE	
1	QAPNYTQHT	26	34	9	HLA-A*02:01	26	62	32273.42	6.20E+01	3.23E+04	62	TRUE	TRUE	
1	CGPKLSTDL	511	519	9	HLA-A*02:01	511	62	32286.7	6.20E+01	3.23E+04	62	TRUE	TRUE	
1	PFKDGIYFA	82	90	9	HLA-A*02:01	82	62	32291.24	6.20E+01	3.23E+04	62	TRUE	TRUE	
1	DLQELGKYE	1181	1189	9	HLA-A*02:01	1181	62	32334.58	6.20E+01	3.23E+04	62	TRUE	TRUE	
1	LREFVFKNK	182	190	9	HLA-A*02:01	182	62	32338.44	6.20E+01	3.23E+04	62	TRUE	TRUE	
1	GKYEQYIKW	1186	1194	9	HLA-A*02:01	1186	62	32395.51	6.20E+01	3.24E+04	62	TRUE	TRUE	
1	YNYKYRYLR	436	444	9	HLA-A*02:01	436	62	32407.8	6.20E+01	3.24E+04	62	TRUE	TRUE	
1	TTFMLKYDE	260	268	9	HLA-A*02:01	260	62	32422.52	6.20E+01	3.24E+04	62	TRUE	TRUE	
1	KHLREFVFK	180	188	9	HLA-A*02:01	180	62	32426.38	6.20E+01	3.24E+04	62	TRUE	TRUE	
1	IYFAATEKS	87	95	9	HLA-A*02:01	87	63	32529.33	6.30E+01	3.25E+04	63	TRUE	TRUE	
1	LGADSSIAY	681	689	9	HLA-A*02:01	681	63	32553.99	6.30E+01	3.26E+04	63	TRUE	TRUE	
1	NGLTGTGVL	530	538	9	HLA-A*02:01	530	63	32558.56	6.30E+01	3.26E+04	63	TRUE	TRUE	
1	LPFYSNVTG	60	68	9	HLA-A*02:01	60	63	32581.82	6.30E+01	3.26E+04	63	TRUE	TRUE	
1	KFPSVYAWE	333	341	9	HLA-A*02:01	333	63	32617.81	6.30E+01	3.26E+04	63	TRUE	TRUE	
1	ENGTITDAV	268	276	9	HLA-A*02:01	268	63	32618.15	6.30E+01	3.26E+04	63	TRUE	TRUE	
1	EIFRSDTLY	45	53	9	HLA-A*02:01	45	63	32632.98	6.30E+01	3.26E+04	63	TRUE	TRUE	
1	STGNYNYKY	432	440	9	HLA-A*02:01	432	63	32640.03	6.30E+01	3.26E+04	63	TRUE	TRUE	
1	TGVIADYNY	402	410	9	HLA-A*02:01	402	63	32654.88	6.30E+01	3.27E+04	63	TRUE	TRUE	
1	ASANLAATK	1002	1010	9	HLA-A*02:01	1002	63	32658.41	6.30E+01	3.27E+04	63	TRUE	TRUE	
1	PTLKYFGGF	774	782	9	HLA-A*02:01	774	63	32705.44	6.30E+01	3.27E+04	63	TRUE	TRUE	
1	NARDLICAQ	827	835	9	HLA-A*02:01	827	63	32720.67	6.30E+01	3.27E+04	63	TRUE	TRUE	
1	DRCTTFDDV	17	25	9	HLA-A*02:01	17	63	32729.51	6.30E+01	3.27E+04	63	TRUE	TRUE	
1	VFQTQAGCL	628	636	9	HLA-A*02:01	628	63	32732	6.30E+01	3.27E+04	63	TRUE	TRUE	
1	HTINHTFGN	70	78	9	HLA-A*02:01	70	63	32735.88	6.30E+01	3.27E+04	63	TRUE	TRUE	
1	CNMYICGDS	720	728	9	HLA-A*02:01	720	63	32751.83	6.30E+01	3.28E+04	63	TRUE	TRUE	
1	NFSISITTE	699	707	9	HLA-A*02:01	699	64	32784.1	6.40E+01	3.28E+04	64	TRUE	TRUE	
1	DINARDLIC	825	833	9	HLA-A*02:01	825	64	32828.11	6.40E+01	3.28E+04	64	TRUE	TRUE	
1	AKNLNESLI	1172	1180	9	HLA-A*02:01	1172	64	32835.58	6.40E+01	3.28E+04	64	TRUE	TRUE	
1	GAGAALQIP	871	879	9	HLA-A*02:01	871	64	32841.25	6.40E+01	3.28E+04	64	TRUE	TRUE	
1	SVKSFEIDK	289	297	9	HLA-A*02:01	289	64	32849.79	6.40E+01	3.28E+04	64	TRUE	TRUE	
1	DLDRCTTFD	15	23	9	HLA-A*02:01	15	64	32877.15	6.40E+01	3.29E+04	64	TRUE	TRUE	
1	RACNFELCD	126	134	9	HLA-A*02:01	126	64	32881.07	6.40E+01	3.29E+04	64	TRUE	TRUE	
1	ELDKYFKNH	1133	1141	9	HLA-A*02:01	1133	64	32887.84	6.40E+01	3.29E+04	64	TRUE	TRUE	
1	IDVVRDLPS	203	211	9	HLA-A*02:01	203	64	32888.9	6.40E+01	3.29E+04	64	TRUE	TRUE	
1	RFQPFQQFG	544	552	9	HLA-A*02:01	544	64	32959.44	6.40E+01	3.30E+04	64	TRUE	TRUE	
1	PDDFMGCVL	413	421	9	HLA-A*02:01	413	64	32969.43	6.40E+01	3.30E+04	64	TRUE	TRUE	
1	LTTTSTALG	920	928	9	HLA-A*02:01	920	64	32971.21	6.40E+01	3.30E+04	64	TRUE	TRUE	
1	VSGTATAGW	860	868	9	HLA-A*02:01	860	64	32971.21	6.40E+01	3.30E+04	64	TRUE	TRUE	
1	PFYSNVTGF	61	69	9	HLA-A*02:01	61	64	32973	6.40E+01	3.30E+04	64	TRUE	TRUE	
1	YPDEIFRSD	42	50	9	HLA-A*02:01	42	64	32999.4	6.40E+01	3.30E+04	64	TRUE	TRUE	
1	ESLTTTSTA	918	926	9	HLA-A*02:01	918	64	33016.54	6.40E+01	3.30E+04	64	TRUE	TRUE	
1	PFQQFGRDV	547	555	9	HLA-A*02:01	547	64	33049.07	6.40E+01	3.30E+04	64	TRUE	TRUE	
1	ILSRLDKVE	962	970	9	HLA-A*02:01	962	64	33069.45	6.40E+01	3.31E+04	64	TRUE	TRUE	
1	GFNFSQILP	781	789	9	HLA-A*02:01	781	65	33147.55	6.50E+01	3.31E+04	65	TRUE	TRUE	
1	RSTSQKSIV	667	675	9	HLA-A*02:01	667	65	33182.72	6.50E+01	3.32E+04	65	TRUE	TRUE	
1	GAISSVLND	953	961	9	HLA-A*02:01	953	65	33184.52	6.50E+01	3.32E+04	65	TRUE	TRUE	
1	ADAGFMKQY	811	819	9	HLA-A*02:01	811	65	33196.35	6.50E+01	3.32E+04	65	TRUE	TRUE	
1	CVLAWNTRN	419	427	9	HLA-A*02:01	419	65	33208.94	6.50E+01	3.32E+04	65	TRUE	TRUE	
1	NFSQILPDP	783	791	9	HLA-A*02:01	783	65	33242.37	6.50E+01	3.32E+04	65	TRUE	TRUE	
1	QDIWGTSAA	242	250	9	HLA-A*02:01	242	65	33298.16	6.50E+01	3.33E+04	65	TRUE	TRUE	
1	VTGFHTINH	66	74	9	HLA-A*02:01	66	65	33299.62	6.50E+01	3.33E+04	65	TRUE	TRUE	
1	TPPALNCYW	468	476	9	HLA-A*02:01	468	65	33304.3	6.50E+01	3.33E+04	65	TRUE	TRUE	
1	TKMSECVLG	1009	1017	9	HLA-A*02:01	1009	65	33321.23	6.50E+01	3.33E+04	65	TRUE	TRUE	
1	AWNTRNIDA	422	430	9	HLA-A*02:01	422	65	33381.5	6.50E+01	3.34E+04	65	TRUE	TRUE	

seq #	peptide	start	end	peptide length	allele	peptide index	median binding	ann IC50	ann percentile	legacy IC50	legacy rank	scores equal	percentiles equal	
1	VKGDDVRQI	389	397	9	HLA-A*02:01	389	65	33393.06	6.50E+01	3.34E+04	65	TRUE	TRUE	
1	GACSCGSCC	1228	1236	9	HLA-A*02:01	1228	66	33435.36	6.60E+01	3.34E+04	66	TRUE	TRUE	
1	QTGVIADYN	401	409	9	HLA-A*02:01	401	66	33446.57	6.60E+01	3.34E+04	66	TRUE	TRUE	
1	LNESLIDLQ	1175	1183	9	HLA-A*02:01	1175	66	33458.87	6.60E+01	3.35E+04	66	TRUE	TRUE	
1	APATVCGPK	506	514	9	HLA-A*02:01	506	66	33500.89	6.60E+01	3.35E+04	66	TRUE	TRUE	
1	RDLPSGFNT	207	215	9	HLA-A*02:01	207	66	33510.68	6.60E+01	3.35E+04	66	TRUE	TRUE	
1	IAAEQDRNT	752	760	9	HLA-A*02:01	752	66	33599.64	6.60E+01	3.36E+04	66	TRUE	TRUE	
1	IFKLPLGIN	219	227	9	HLA-A*02:01	219	66	33608.72	6.60E+01	3.36E+04	66	TRUE	TRUE	
1	VTYVPSQER	1047	1055	9	HLA-A*02:01	1047	66	33633.46	6.60E+01	3.36E+04	66	TRUE	TRUE	
1	VIPFKDGIY	80	88	9	HLA-A*02:01	80	66	33640.38	6.60E+01	3.36E+04	66	TRUE	TRUE	
1	VIIINNSTN	114	122	9	HLA-A*02:01	114	66	33642.57	6.60E+01	3.36E+04	66	TRUE	TRUE	
1	TTTGIGYQP	485	493	9	HLA-A*02:01	485	66	33655.68	6.60E+01	3.37E+04	66	TRUE	TRUE	
1	YTQHTSSMR	30	38	9	HLA-A*02:01	30	66	33660.41	6.60E+01	3.37E+04	66	TRUE	TRUE	
1	TTAPAICHE	1058	1066	9	HLA-A*02:01	1058	67	33724.55	6.70E+01	3.37E+04	67	TRUE	TRUE	
1	RQIAPGQTG	395	403	9	HLA-A*02:01	395	67	33731.12	6.70E+01	3.37E+04	67	TRUE	TRUE	
1	HTSPDVDLG	1141	1149	9	HLA-A*02:01	1141	67	33753.4	6.70E+01	3.38E+04	67	TRUE	TRUE	
1	NTRNIDATS	424	432	9	HLA-A*02:01	424	67	33778.62	6.70E+01	3.38E+04	67	TRUE	TRUE	
1	TAGWTFGAG	865	873	9	HLA-A*02:01	865	67	33791.04	6.70E+01	3.38E+04	67	TRUE	TRUE	
1	LNRALSGIA	745	753	9	HLA-A*02:01	745	67	33798.71	6.70E+01	3.38E+04	67	TRUE	TRUE	
1	RDPKTSEIL	563	571	9	HLA-A*02:01	563	67	33810.05	6.70E+01	3.38E+04	67	TRUE	TRUE	
1	VVRFPNITN	313	321	9	HLA-A*02:01	313	67	33879.26	6.70E+01	3.39E+04	67	TRUE	TRUE	
1	QAGCLIGAE	632	640	9	HLA-A*02:01	632	67	33915.94	6.70E+01	3.39E+04	67	TRUE	TRUE	
1	VYADSFVVK	382	390	9	HLA-A*02:01	382	67	33936.86	6.70E+01	3.39E+04	67	TRUE	TRUE	
1	AYFPREGVF	1069	1077	9	HLA-A*02:01	1069	67	33966.61	6.70E+01	3.40E+04	67	TRUE	TRUE	
1	GEVFNATKF	326	334	9	HLA-A*02:01	326	67	33991.99	6.70E+01	3.40E+04	67	TRUE	TRUE	
1	TATAGWTFG	863	871	9	HLA-A*02:01	863	67	33992.36	6.70E+01	3.40E+04	67	TRUE	TRUE	
1	DVNCTDVST	600	608	9	HLA-A*02:01	600	67	34025.48	6.70E+01	3.40E+04	67	TRUE	TRUE	
1	NESLIDLQE	1176	1184	9	HLA-A*02:01	1176	67	34027.3	6.70E+01	3.40E+04	67	TRUE	TRUE	
1	PQAAPHGVV	1035	1043	9	HLA-A*02:01	1035	67	34029.52	6.70E+01	3.40E+04	67	TRUE	TRUE	
1	SGNCDVVIG	1105	1113	9	HLA-A*02:01	1105	68	34084.8	6.80E+01	3.41E+04	68	TRUE	TRUE	
1	WPLNDYGFY	476	484	9	HLA-A*02:01	476	68	34113.21	6.80E+01	3.41E+04	68	TRUE	TRUE	
1	FDDVQAPNY	22	30	9	HLA-A*02:01	22	68	34126.86	6.80E+01	3.41E+04	68	TRUE	TRUE	
1	QQFGRDVSD	549	557	9	HLA-A*02:01	549	68	34129.45	6.80E+01	3.41E+04	68	TRUE	TRUE	
1	IKNQCVNFN	520	528	9	HLA-A*02:01	520	68	34145.33	6.80E+01	3.41E+04	68	TRUE	TRUE	
1	NAPATVCGP	505	513	9	HLA-A*02:01	505	68	34149.03	6.80E+01	3.41E+04	68	TRUE	TRUE	
1	ERNFTTAPA	1054	1062	9	HLA-A*02:01	1054	68	34164.18	6.80E+01	3.42E+04	68	TRUE	TRUE	
1	VSLLRSTSQ	663	671	9	HLA-A*02:01	663	68	34169.36	6.80E+01	3.42E+04	68	TRUE	TRUE	
1	EVQIDRLIT	972	980	9	HLA-A*02:01	972	68	34180.82	6.80E+01	3.42E+04	68	TRUE	TRUE	
1	DNPFFAVSK	134	142	9	HLA-A*02:01	134	68	34192.66	6.80E+01	3.42E+04	68	TRUE	TRUE	
1	NATKFPSVY	330	338	9	HLA-A*02:01	330	68	34201.53	6.80E+01	3.42E+04	68	TRUE	TRUE	
1	GAEHVDTSY	638	646	9	HLA-A*02:01	638	68	34254.86	6.80E+01	3.43E+04	68	TRUE	TRUE	
1	TQHTSSMRG	31	39	9	HLA-A*02:01	31	68	34306.04	6.80E+01	3.43E+04	68	TRUE	TRUE	
1	TREVFAQVK	760	768	9	HLA-A*02:01	760	68	34335.01	6.80E+01	3.43E+04	68	TRUE	TRUE	
1	ATVCGPKLS	508	516	9	HLA-A*02:01	508	68	34335.76	6.80E+01	3.43E+04	68	TRUE	TRUE	
1	KPMGTQTHT	142	150	9	HLA-A*02:01	142	69	34350.25	6.90E+01	3.44E+04	69	TRUE	TRUE	
1	IDLQELGKY	1180	1188	9	HLA-A*02:01	1180	69	34397.11	6.90E+01	3.44E+04	69	TRUE	TRUE	
1	NYKLPDDFM	409	417	9	HLA-A*02:01	409	69	34426.89	6.90E+01	3.44E+04	69	TRUE	TRUE	
1	NFNFNGLTG	526	534	9	HLA-A*02:01	526	69	34432.86	6.90E+01	3.44E+04	69	TRUE	TRUE	
1	GSDLDRCTT	13	21	9	HLA-A*02:01	13	69	34475.35	6.90E+01	3.45E+04	69	TRUE	TRUE	
1	AFSLDVSEK	167	175	9	HLA-A*02:01	167	69	34479.82	6.90E+01	3.45E+04	69	TRUE	TRUE	
1	GYQPIDVVR	199	207	9	HLA-A*02:01	199	69	34494.39	6.90E+01	3.45E+04	69	TRUE	TRUE	
1	IQKEIDRLN	1161	1169	9	HLA-A*02:01	1161	69	34496.25	6.90E+01	3.45E+04	69	TRUE	TRUE	
1	ADQLTPAWR	612	620	9	HLA-A*02:01	612	69	34504.09	6.90E+01	3.45E+04	69	TRUE	TRUE	
1	GNNVFQTQA	625	633	9	HLA-A*02:01	625	69	34511.19	6.90E+01	3.45E+04	69	TRUE	TRUE	
1	DEIFRSDTL	44	52	9	HLA-A*02:01	44	69	34545.54	6.90E+01	3.45E+04	69	TRUE	TRUE	
1	SECVLGQSK	1012	1020	9	HLA-A*02:01	1012	69	34546.66	6.90E+01	3.45E+04	69	TRUE	TRUE	
1	ISQIQESLT	913	921	9	HLA-A*02:01	913	69	34591.56	6.90E+01	3.46E+04	69	TRUE	TRUE	
1	AICHEGKAY	1062	1070	9	HLA-A*02:01	1062	69	34617.76	6.90E+01	3.46E+04	69	TRUE	TRUE	
1	PSSKRFQPF	540	548	9	HLA-A*02:01	540	70	34641.36	7.00E+01	3.46E+04	70	TRUE	TRUE	
1	PREGVFVFN	1072	1080	9	HLA-A*02:01	1072	70	34676.61	7.00E+01	3.47E+04	70	TRUE	TRUE	
1	SATKLNDLC	370	378	9	HLA-A*02:01	370	70	34697.64	7.00E+01	3.47E+04	70	TRUE	TRUE	
1	DIPIGAGIC	649	657	9	HLA-A*02:01	649	70	34698	7.00E+01	3.47E+04	70	TRUE	TRUE	
1	TVSLLRSTS	662	670	9	HLA-A*02:01	662	70	34758.52	7.00E+01	3.48E+04	70	TRUE	TRUE	
1	QTSNFRVVP	301	309	9	HLA-A*02:01	301	70	34763.78	7.00E+01	3.48E+04	70	TRUE	TRUE	
1	CSVKSFEID	288	296	9	HLA-A*02:01	288	70	34770.53	7.00E+01	3.48E+04	70	TRUE	TRUE	
1	PFAMQMAYR	879	887	9	HLA-A*02:01	879	70	34814.22	7.00E+01	3.48E+04	70	TRUE	TRUE	
1	PPLLTDDMI	844	852	9	HLA-A*02:01	844	70	34833.8	7.00E+01	3.48E+04	70	TRUE	TRUE	
1	GFNTLKPIF	212	220	9	HLA-A*02:01	212	70	34840.97	7.00E+01	3.48E+04	70	TRUE	TRUE	
1	IGAEHVDTS	637	645	9	HLA-A*02:01	637	70	34878.31	7.00E+01	3.49E+04	70	TRUE	TRUE	
1	FNKAISQIQ	909	917	9	HLA-A*02:01	909	70	34883.6	7.00E+01	3.49E+04	70	TRUE	TRUE	
1	TLTSGSDLD	9	17	9	HLA-A*02:01	9	70	34918.33	7.00E+01	3.49E+04	70	TRUE	TRUE	
1	QHTSSMRGV	32	40	9	HLA-A*02:01	32	70	34928.15	7.00E+01	3.49E+04	70	TRUE	TRUE	
1	GWTFGAGAA	867	875	9	HLA-A*02:01	867	71	34945.54	7.10E+01	3.49E+04	71	TRUE	TRUE	
1	RDISNVPFS	453	461	9	HLA-A*02:01	453	71	34970.14	7.10E+01	3.50E+04	71	TRUE	TRUE	
1	DVVRFPNIT	312	320	9	HLA-A*02:01	312	71	34972.39	7.10E+01	3.50E+04	71	TRUE	TRUE	
1	MYKTPTLKY	770	778	9	HLA-A*02:01	770	71	34974.67	7.10E+01	3.50E+04	71	TRUE	TRUE	
1	DVDLGDISG	1145	1153	9	HLA-A*02:01	1145	71	35003.05	7.10E+01	3.50E+04	71	TRUE	TRUE	
1	TGNNVFQTQ	624	632	9	HLA-A*02:01	624	71	35004.95	7.10E+01	3.50E+04	71	TRUE	TRUE	
1	LPSGFNTLK	209	217	9	HLA-A*02:01	209	71	35029.59	7.10E+01	3.50E+04	71	TRUE	TRUE	
1	PQIITTDNT	1094	1102	9	HLA-A*02:01	1094	71	35048.16	7.10E+01	3.50E+04	71	TRUE	TRUE	
1	VDCNMYICG	718	726	9	HLA-A*02:01	718	71	35133.21	7.10E+01	3.51E+04	71	TRUE	TRUE	
1	SFKEELDKY	1129	1137	9	HLA-A*02:01	1129	71	35165.92	7.10E+01	3.52E+04	71	TRUE	TRUE	
1	TINHTFGNP	71	79	9	HLA-A*02:01	71	71	35173.16	7.10E+01	3.52E+04	71	TRUE	TRUE	
1	DGIYFAATE	85	93	9	HLA-A*02:01	85	71	35226.09	7.10E+01	3.52E+04	71	TRUE	TRUE	
1	NPFFAVSKP	135	143	9	HLA-A*02:01	135	72	35250.09	7.20E+01	3.53E+04	72	TRUE	TRUE	
1	IDRLNEVAK	1165	1173	9	HLA-A*02:01	1165	72	35252	7.20E+01	3.53E+04	72	TRUE	TRUE	
1	PATVCGPKL	507	515	9	HLA-A*02:01	507	72	35258.12	7.20E+01	3.53E+04	72	TRUE	TRUE	
1	NTVYDPLQP	1117	1125	9	HLA-A*02:01	1117	72	35261.55	7.20E+01	3.53E+04	72	TRUE	TRUE	
1	FNGIGVTQN	888	896	9	HLA-A*02:01	888	72	35277.95	7.20E+01	3.53E+04	72	TRUE	TRUE	
1	FERDISNVP	451	459	9	HLA-A*02:01	451	72	35290.17	7.20E+01	3.53E+04	72	TRUE	TRUE	
1	TTTSTALGK	921	929	9	HLA-A*02:01	921	72	35298.57	7.20E+01	3.53E+04	72	TRUE	TRUE	
1	VFKNKDGFL	186	194	9	HLA-A*02:01	186	72	35311.93	7.20E+01	3.53E+04	72	TRUE	TRUE	
1	LQELGKYEQ	1182	1190	9	HLA-A*02:01	1182	72	35339.09	7.20E+01	3.53E+04	72	TRUE	TRUE	
1	CCSCLKGAC	1222	1230	9	HLA-A*02:01	1222	72	35357.05	7.20E+01	3.54E+04	72	TRUE	TRUE	
1	DAFSLDVSE	166	174	9	HLA-A*02:01	166	72	35367.77	7.20E+01	3.54E+04	72	TRUE	TRUE	
1	LTSGSDLDR	10	18	9	HLA-A*02:01	10	72	35389.2	7.20E+01	3.54E+04	72	TRUE	TRUE	
1	FVVKGDDVR	387	395	9	HLA-A*02:01	387	72	35416.78	7.20E+01	3.54E+04	72	TRUE	TRUE	
1	IYSTGNNVF	621	629	9	HLA-A*02:01	621	72	35427.51	7.20E+01	3.54E+04	72	TRUE	TRUE	
1	RPFERDISN	449	457	9	HLA-A*02:01	449	72	35468.18	7.20E+01	3.55E+04	72	TRUE	TRUE	
1	SVYAWERKK	336	344	9	HLA-A*02:01	336	72	35478.91	7.20E+01	3.55E+04	72	TRUE	TRUE	
1	DLGDISGIN	1147	1155	9	HLA-A*02:01	1147	72	35481.22	7.20E+01	3.55E+04	72	TRUE	TRUE	
1	ISNVPFSPD	455	463	9	HLA-A*02:01	455	73	35527.32	7.30E+01	3.55E+04	73	TRUE	TRUE	

seq #	peptide	start	end	peptide length	allele	peptide index	median binding	ann IC50	ann percentile	legacy IC50	legacy rank	scores equal	percentiles equal	
1	DLICAQKFN	830	838	9	HLA-A*02:01	830	73	35556.93	7.30E+01	3.56E+04	73	TRUE	TRUE	
1	LNTLVKQLS	941	949	9	HLA-A*02:01	941	73	35561.17	7.30E+01	3.56E+04	73	TRUE	TRUE	
1	VEAEVQIDR	969	977	9	HLA-A*02:01	969	73	35588.89	7.30E+01	3.56E+04	73	TRUE	TRUE	
1	QELGKYEQY	1183	1191	9	HLA-A*02:01	1183	73	35596.19	7.30E+01	3.56E+04	73	TRUE	TRUE	
1	PIDVVRDLP	202	210	9	HLA-A*02:01	202	73	35611.23	7.30E+01	3.56E+04	73	TRUE	TRUE	
1	LNCYWPLND	472	480	9	HLA-A*02:01	472	73	35692.62	7.30E+01	3.57E+04	73	TRUE	TRUE	
1	ACNFELCDN	127	135	9	HLA-A*02:01	127	74	35823	7.40E+01	3.58E+04	74	TRUE	TRUE	
1	ASVVNIQKE	1156	1164	9	HLA-A*02:01	1156	74	35861.37	7.40E+01	3.59E+04	74	TRUE	TRUE	
1	SKRVDFCGK	1019	1027	9	HLA-A*02:01	1019	74	35888.16	7.40E+01	3.59E+04	74	TRUE	TRUE	
1	CGKGYHLMS	1025	1033	9	HLA-A*02:01	1025	74	35943.73	7.40E+01	3.59E+04	74	TRUE	TRUE	
1	VFGSTMNNK	102	110	9	HLA-A*02:01	102	74	35957.34	7.40E+01	3.60E+04	74	TRUE	TRUE	
1	EKSNVVRGW	93	101	9	HLA-A*02:01	93	74	35969.43	7.40E+01	3.60E+04	74	TRUE	TRUE	
1	VVPSGDVVR	307	315	9	HLA-A*02:01	307	74	35980.7	7.40E+01	3.60E+04	74	TRUE	TRUE	
1	AELKCSVKS	284	292	9	HLA-A*02:01	284	74	36000.57	7.40E+01	3.60E+04	74	TRUE	TRUE	
1	DVVNQNAQA	932	940	9	HLA-A*02:01	932	74	36030.58	7.40E+01	3.60E+04	74	TRUE	TRUE	
1	DCNMYICGD	719	727	9	HLA-A*02:01	719	74	36068.01	7.40E+01	3.61E+04	74	TRUE	TRUE	
1	SDAFSLDVS	165	173	9	HLA-A*02:01	165	74	36070.75	7.40E+01	3.61E+04	74	TRUE	TRUE	
1	SCGSCCKFD	1231	1239	9	HLA-A*02:01	1231	75	36094.95	7.50E+01	3.61E+04	75	TRUE	TRUE	
1	LNDILSRLD	959	967	9	HLA-A*02:01	959	75	36107.46	7.50E+01	3.61E+04	75	TRUE	TRUE	
1	TFGAGAALQ	869	877	9	HLA-A*02:01	869	75	36119.96	7.50E+01	3.61E+04	75	TRUE	TRUE	
1	TGRLQSLQT	980	988	9	HLA-A*02:01	980	75	36143.01	7.50E+01	3.61E+04	75	TRUE	TRUE	
1	LGQSKRVDF	1016	1024	9	HLA-A*02:01	1016	75	36146.53	7.50E+01	3.61E+04	75	TRUE	TRUE	
1	GFYTTTGIG	482	490	9	HLA-A*02:01	482	75	36178.62	7.50E+01	3.62E+04	75	TRUE	TRUE	
1	ECDIPIGAG	647	655	9	HLA-A*02:01	647	75	36205.64	7.50E+01	3.62E+04	75	TRUE	TRUE	
1	PTNFSISIT	697	705	9	HLA-A*02:01	697	75	36209.17	7.50E+01	3.62E+04	75	TRUE	TRUE	
1	SFEIDKGIY	292	300	9	HLA-A*02:01	292	75	36254.25	7.50E+01	3.63E+04	75	TRUE	TRUE	
1	LCPFGEVFN	322	330	9	HLA-A*02:01	322	75	36267.2	7.50E+01	3.63E+04	75	TRUE	TRUE	
1	LSTDLIKNQ	515	523	9	HLA-A*02:01	515	75	36267.2	7.50E+01	3.63E+04	75	TRUE	TRUE	
1	ECLGDINAR	821	829	9	HLA-A*02:01	821	75	36322.96	7.50E+01	3.63E+04	75	TRUE	TRUE	
1	EVFNATKFP	327	335	9	HLA-A*02:01	327	76	36410.7	7.60E+01	3.64E+04	76	TRUE	TRUE	
1	LDISPCSFG	571	579	9	HLA-A*02:01	571	76	36413.86	7.60E+01	3.64E+04	76	TRUE	TRUE	
1	STMNNKSQS	105	113	9	HLA-A*02:01	105	76	36444.59	7.60E+01	3.64E+04	76	TRUE	TRUE	
1	LKGACSCGS	1226	1234	9	HLA-A*02:01	1226	76	36447.37	7.60E+01	3.64E+04	76	TRUE	TRUE	
1	AFSPAQDIW	237	245	9	HLA-A*02:01	237	76	36447.77	7.60E+01	3.64E+04	76	TRUE	TRUE	
1	HEGKAYFPR	1065	1073	9	HLA-A*02:01	1065	76	36465.12	7.60E+01	3.65E+04	76	TRUE	TRUE	
1	APAICHEGK	1060	1068	9	HLA-A*02:01	1060	76	36488.79	7.60E+01	3.65E+04	76	TRUE	TRUE	
1	KYEQYIKWP	1187	1195	9	HLA-A*02:01	1187	76	36506.96	7.60E+01	3.65E+04	76	TRUE	TRUE	
1	NIDATSTGN	427	435	9	HLA-A*02:01	427	76	36508.13	7.60E+01	3.65E+04	76	TRUE	TRUE	
1	KGACSCGSC	1227	1235	9	HLA-A*02:01	1227	76	36522.36	7.60E+01	3.65E+04	76	TRUE	TRUE	
1	VDTSYECDI	642	650	9	HLA-A*02:01	642	76	36567.44	7.60E+01	3.66E+04	76	TRUE	TRUE	
1	GKLQDVVNQ	928	936	9	HLA-A*02:01	928	76	36571.8	7.60E+01	3.66E+04	76	TRUE	TRUE	
1	IDKGIYQTS	295	303	9	HLA-A*02:01	295	76	36593.56	7.60E+01	3.66E+04	76	TRUE	TRUE	
1	IADYNYKLP	405	413	9	HLA-A*02:01	405	77	36641.5	7.70E+01	3.66E+04	77	TRUE	TRUE	
1	DKVEAEVQI	967	975	9	HLA-A*02:01	967	77	36658.16	7.70E+01	3.67E+04	77	TRUE	TRUE	
1	AGFMKQYGE	813	821	9	HLA-A*02:01	813	77	36696.27	7.70E+01	3.67E+04	77	TRUE	TRUE	
1	PPALNCYWP	469	477	9	HLA-A*02:01	469	77	36731.61	7.70E+01	3.67E+04	77	TRUE	TRUE	
1	LSGIAAEQD	749	757	9	HLA-A*02:01	749	77	36737.96	7.70E+01	3.67E+04	77	TRUE	TRUE	
1	PKTSEILDI	565	573	9	HLA-A*02:01	565	77	36750.69	7.70E+01	3.68E+04	77	TRUE	TRUE	
1	FFAVSKPMG	137	145	9	HLA-A*02:01	137	77	36757.07	7.70E+01	3.68E+04	77	TRUE	TRUE	
1	QFGRDVSDF	550	558	9	HLA-A*02:01	550	77	36760.24	7.70E+01	3.68E+04	77	TRUE	TRUE	
1	KGIYQTSNF	297	305	9	HLA-A*02:01	297	77	36804.81	7.70E+01	3.68E+04	77	TRUE	TRUE	
1	VDCSQNPLA	276	284	9	HLA-A*02:01	276	77	36849.44	7.70E+01	3.68E+04	77	TRUE	TRUE	
1	EKSGNFKHL	174	182	9	HLA-A*02:01	174	77	36853.81	7.70E+01	3.69E+04	77	TRUE	TRUE	
1	PLKPTKRSF	791	799	9	HLA-A*02:01	791	77	36857.81	7.70E+01	3.69E+04	77	TRUE	TRUE	
1	NNSTNVVIR	118	126	9	HLA-A*02:01	118	77	36860.99	7.70E+01	3.69E+04	77	TRUE	TRUE	
1	LHVTYVPSQ	1045	1053	9	HLA-A*02:01	1045	77	36891.72	7.70E+01	3.69E+04	77	TRUE	TRUE	
1	MNNKSQSVI	107	115	9	HLA-A*02:01	107	77	36893.31	7.70E+01	3.69E+04	77	TRUE	TRUE	
1	ERKKISNCV	341	349	9	HLA-A*02:01	341	77	36918.09	7.70E+01	3.69E+04	77	TRUE	TRUE	
1	TPTLKYFGG	773	781	9	HLA-A*02:01	773	78	36921.68	7.80E+01	3.69E+04	78	TRUE	TRUE	
1	CHEGKAYFP	1064	1072	9	HLA-A*02:01	1064	78	36956.85	7.80E+01	3.70E+04	78	TRUE	TRUE	
1	VSVITPGTN	581	589	9	HLA-A*02:01	581	78	36972.44	7.80E+01	3.70E+04	78	TRUE	TRUE	
1	DYSVLYNST	351	359	9	HLA-A*02:01	351	78	36987.65	7.80E+01	3.70E+04	78	TRUE	TRUE	
1	PFERDISNV	450	458	9	HLA-A*02:01	450	78	37047.72	7.80E+01	3.70E+04	78	TRUE	TRUE	
1	PAWRIYSTG	617	625	9	HLA-A*02:01	617	78	37058.54	7.80E+01	3.71E+04	78	TRUE	TRUE	
1	PSGDVVRFP	309	317	9	HLA-A*02:01	309	78	37075.39	7.80E+01	3.71E+04	78	TRUE	TRUE	
1	TFDDVQAPN	21	29	9	HLA-A*02:01	21	78	37104.7	7.80E+01	3.71E+04	78	TRUE	TRUE	
1	SPCSFGGVS	574	582	9	HLA-A*02:01	574	78	37109.91	7.80E+01	3.71E+04	78	TRUE	TRUE	
1	VQAPNYTQH	25	33	9	HLA-A*02:01	25	78	37120.35	7.80E+01	3.71E+04	78	TRUE	TRUE	
1	YGECLGDIN	819	827	9	HLA-A*02:01	819	78	37142.86	7.80E+01	3.71E+04	78	TRUE	TRUE	
1	NFTTAPAIC	1056	1064	9	HLA-A*02:01	1056	78	37194.32	7.80E+01	3.72E+04	78	TRUE	TRUE	
1	GDISGINAS	1149	1157	9	HLA-A*02:01	1149	79	37216.88	7.90E+01	3.72E+04	79	TRUE	TRUE	
1	DENGTITDA	267	275	9	HLA-A*02:01	267	79	37248.7	7.90E+01	3.72E+04	79	TRUE	TRUE	
1	QSKRVDFCG	1018	1026	9	HLA-A*02:01	1018	79	37251.5	7.90E+01	3.73E+04	79	TRUE	TRUE	
1	HGKLRPFER	445	453	9	HLA-A*02:01	445	79	37287	7.90E+01	3.73E+04	79	TRUE	TRUE	
1	DVRQIAPGQ	393	401	9	HLA-A*02:01	393	79	37288.62	7.90E+01	3.73E+04	79	TRUE	TRUE	
1	NHTSPDVDL	1140	1148	9	HLA-A*02:01	1140	79	37318.09	7.90E+01	3.73E+04	79	TRUE	TRUE	
1	TSGSDLDRC	11	19	9	HLA-A*02:01	11	79	37324.12	7.90E+01	3.73E+04	79	TRUE	TRUE	
1	LAWNTRNID	421	429	9	HLA-A*02:01	421	79	37362.11	7.90E+01	3.74E+04	79	TRUE	TRUE	
1	KYDENGTIT	265	273	9	HLA-A*02:01	265	79	37386.38	7.90E+01	3.74E+04	79	TRUE	TRUE	
1	NFRVVPSGD	304	312	9	HLA-A*02:01	304	79	37424.01	7.90E+01	3.74E+04	79	TRUE	TRUE	
1	AVSKPMGTQ	139	147	9	HLA-A*02:01	139	79	37425.24	7.90E+01	3.74E+04	79	TRUE	TRUE	
1	SYECDIPIG	645	653	9	HLA-A*02:01	645	79	37442.64	7.90E+01	3.74E+04	79	TRUE	TRUE	
1	VSDFTDSVR	555	563	9	HLA-A*02:01	555	79	37451.97	7.90E+01	3.75E+04	79	TRUE	TRUE	
1	QESLTTTST	917	925	9	HLA-A*02:01	917	79	37456.03	7.90E+01	3.75E+04	79	TRUE	TRUE	
1	PSQERNFTT	1051	1059	9	HLA-A*02:01	1051	80	37494.94	8.00E+01	3.75E+04	80	TRUE	TRUE	
1	ICASYHTVS	656	664	9	HLA-A*02:01	656	80	37504.27	8.00E+01	3.75E+04	80	TRUE	TRUE	
1	FHTINHTFG	69	77	9	HLA-A*02:01	69	80	37507.51	8.00E+01	3.75E+04	80	TRUE	TRUE	
1	GDINARDLI	824	832	9	HLA-A*02:01	824	80	37590.82	8.00E+01	3.76E+04	80	TRUE	TRUE	
1	DRLITGRLQ	976	984	9	HLA-A*02:01	976	80	37593.65	8.00E+01	3.76E+04	80	TRUE	TRUE	
1	NNKSQSVII	108	116	9	HLA-A*02:01	108	80	37599.35	8.00E+01	3.76E+04	80	TRUE	TRUE	
1	KSGNFKHLR	175	183	9	HLA-A*02:01	175	80	37600.97	8.00E+01	3.76E+04	80	TRUE	TRUE	
1	VKQLSSNFG	945	953	9	HLA-A*02:01	945	80	37601.39	8.00E+01	3.76E+04	80	TRUE	TRUE	
1	RKKISNCVA	342	350	9	HLA-A*02:01	342	80	37609.51	8.00E+01	3.76E+04	80	TRUE	TRUE	
1	RNTREVFAQ	758	766	9	HLA-A*02:01	758	80	37617.25	8.00E+01	3.76E+04	80	TRUE	TRUE	
1	SPQIITTDN	1093	1101	9	HLA-A*02:01	1093	80	37627.03	8.00E+01	3.76E+04	80	TRUE	TRUE	
1	VSKPMGTQT	140	148	9	HLA-A*02:01	140	80	37639.65	8.00E+01	3.76E+04	80	TRUE	TRUE	
1	VKQMYKTPT	767	775	9	HLA-A*02:01	767	80	37668.16	8.00E+01	3.77E+04	80	TRUE	TRUE	
1	DLIKNQCVN	518	526	9	HLA-A*02:01	518	80	37729.35	8.00E+01	3.77E+04	80	TRUE	TRUE	
1	SKRFQPFQQ	542	550	9	HLA-A*02:01	542	80	37749.77	8.00E+01	3.77E+04	80	TRUE	TRUE	
1	VSTAIHADQ	606	614	9	HLA-A*02:01	606	80	37750.99	8.00E+01	3.78E+04	80	TRUE	TRUE	
1	ITGRLQSLQ	979	987	9	HLA-A*02:01	979	80	37757.54	8.00E+01	3.78E+04	80	TRUE	TRUE	
1	EVAKNLNES	1170	1178	9	HLA-A*02:01	1170	81	37763.23	8.10E+01	3.78E+04	81	TRUE	TRUE	

seq #	peptide	start	end	peptide length	allele	peptide index	median binding	ann IC50	ann percentile	legacy IC50	legacy rank	scores equal	percentiles equal
1	KVEAEVQID	968	976	9	HLA-A*02:01	968	81	37782.04	8.10E+01	3.78E+04	81	TRUE	TRUE
1	EDLLFNKVT	801	809	9	HLA-A*02:01	801	81	37820.48	8.10E+01	3.78E+04	81	TRUE	TRUE
1	IRAAEIRAS	995	1003	9	HLA-A*02:01	995	81	37828.68	8.10E+01	3.78E+04	81	TRUE	TRUE
1	DGKPCTPPA	463	471	9	HLA-A*02:01	463	81	37845.47	8.10E+01	3.78E+04	81	TRUE	TRUE
1	SDTLYLTQD	49	57	9	HLA-A*02:01	49	81	37884.79	8.10E+01	3.79E+04	81	TRUE	TRUE
1	ACSCGSCCK	1229	1237	9	HLA-A*02:01	1229	81	37886.03	8.10E+01	3.79E+04	81	TRUE	TRUE
1	TSPDVDLGD	1142	1150	9	HLA-A*02:01	1142	81	37899.55	8.10E+01	3.79E+04	81	TRUE	TRUE
1	SCCKFDEDD	1234	1242	9	HLA-A*02:01	1234	81	37914.31	8.10E+01	3.79E+04	81	TRUE	TRUE
1	ADSFVVKGD	384	392	9	HLA-A*02:01	384	81	37934.41	8.10E+01	3.79E+04	81	TRUE	TRUE
1	NFNGLTGTG	528	536	9	HLA-A*02:01	528	82	38048.69	8.20E+01	3.80E+04	82	TRUE	TRUE
1	MPVSMAKTS	709	717	9	HLA-A*02:01	709	82	38075.89	8.20E+01	3.81E+04	82	TRUE	TRUE
1	NDILSRLDK	960	968	9	HLA-A*02:01	960	82	38099.37	8.20E+01	3.81E+04	82	TRUE	TRUE
1	DVSEKSGNF	171	179	9	HLA-A*02:01	171	82	38114.63	8.20E+01	3.81E+04	82	TRUE	TRUE
1	KKISNCVAD	343	351	9	HLA-A*02:01	343	82	38117.93	8.20E+01	3.81E+04	82	TRUE	TRUE
1	LNAPATVCG	504	512	9	HLA-A*02:01	504	82	38123.7	8.20E+01	3.81E+04	82	TRUE	TRUE
1	ADSSIAYSN	683	691	9	HLA-A*02:01	683	82	38134.84	8.20E+01	3.81E+04	82	TRUE	TRUE
1	DSFKEELDK	1128	1136	9	HLA-A*02:01	1128	82	38174.06	8.20E+01	3.82E+04	82	TRUE	TRUE
1	CGSCCKFDE	1232	1240	9	HLA-A*02:01	1232	82	38289.48	8.20E+01	3.83E+04	82	TRUE	TRUE
1	LYVYKGYQP	194	202	9	HLA-A*02:01	194	83	38323.45	8.30E+01	3.83E+04	83	TRUE	TRUE
1	GNYNYKYRY	434	442	9	HLA-A*02:01	434	83	38339.22	8.30E+01	3.83E+04	83	TRUE	TRUE
1	QNPLAELKC	280	288	9	HLA-A*02:01	280	83	38354.16	8.30E+01	3.84E+04	83	TRUE	TRUE
1	KRVDFCGKG	1020	1028	9	HLA-A*02:01	1020	83	38415.62	8.30E+01	3.84E+04	83	TRUE	TRUE
1	QERNFTTAP	1053	1061	9	HLA-A*02:01	1053	83	38424.34	8.30E+01	3.84E+04	83	TRUE	TRUE
1	NTFVSGNCD	1101	1109	9	HLA-A*02:01	1101	83	38545.93	8.30E+01	3.85E+04	83	TRUE	TRUE
1	NYTQHTSSM	29	37	9	HLA-A*02:01	29	84	38610.22	8.40E+01	3.86E+04	84	TRUE	TRUE
1	NYKYRYLRH	437	445	9	HLA-A*02:01	437	84	38624.84	8.40E+01	3.86E+04	84	TRUE	TRUE
1	AWRIYSTGN	618	626	9	HLA-A*02:01	618	84	38639.46	8.40E+01	3.86E+04	84	TRUE	TRUE
1	HVTYVPSQE	1046	1054	9	HLA-A*02:01	1046	84	38644.9	8.40E+01	3.86E+04	84	TRUE	TRUE
1	LTAFSPAQD	235	243	9	HLA-A*02:01	235	84	38654.94	8.40E+01	3.87E+04	84	TRUE	TRUE
1	WERKKISNC	340	348	9	HLA-A*02:01	340	84	38679.21	8.40E+01	3.87E+04	84	TRUE	TRUE
1	GNPVIPFKD	77	85	9	HLA-A*02:01	77	84	38710.6	8.40E+01	3.87E+04	84	TRUE	TRUE
1	LYQDVNCTD	597	605	9	HLA-A*02:01	597	84	38718.14	8.40E+01	3.87E+04	84	TRUE	TRUE
1	SPDGKPCTP	461	469	9	HLA-A*02:01	461	84	38724.01	8.40E+01	3.87E+04	84	TRUE	TRUE
1	QDRNTREVF	756	764	9	HLA-A*02:01	756	84	38725.25	8.40E+01	3.87E+04	84	TRUE	TRUE
1	NHTFGNPVI	73	81	9	HLA-A*02:01	73	84	38744.54	8.40E+01	3.87E+04	84	TRUE	TRUE
1	AAEIRASAN	997	1005	9	HLA-A*02:01	997	84	38754.61	8.40E+01	3.88E+04	84	TRUE	TRUE
1	VAVLYQDVN	594	602	9	HLA-A*02:01	594	84	38756.29	8.40E+01	3.88E+04	84	TRUE	TRUE
1	NPVIPFKDG	78	86	9	HLA-A*02:01	78	84	38761.31	8.40E+01	3.88E+04	84	TRUE	TRUE
1	SRLDKVEAE	964	972	9	HLA-A*02:01	964	84	38777.26	8.40E+01	3.88E+04	84	TRUE	TRUE
1	VCGPKLSTD	510	518	9	HLA-A*02:01	510	84	38796.97	8.40E+01	3.88E+04	84	TRUE	TRUE
1	KFDEDDSEP	1237	1245	9	HLA-A*02:01	1237	84	38805.8	8.40E+01	3.88E+04	84	TRUE	TRUE
1	STALGKLQD	924	932	9	HLA-A*02:01	924	84	38810.84	8.40E+01	3.88E+04	84	TRUE	TRUE
1	AYTMSLGAD	676	684	9	HLA-A*02:01	676	84	38812.52	8.40E+01	3.88E+04	84	TRUE	TRUE
1	TSTALGKLQ	923	931	9	HLA-A*02:01	923	84	38823.03	8.40E+01	3.88E+04	84	TRUE	TRUE
1	ATEKSNVVR	91	99	9	HLA-A*02:01	91	84	38828.49	8.40E+01	3.88E+04	84	TRUE	TRUE
1	VSEKSGNFK	172	180	9	HLA-A*02:01	172	84	38843.19	8.40E+01	3.88E+04	84	TRUE	TRUE
1	RCTTFDDVQ	18	26	9	HLA-A*02:01	18	84	38852.43	8.40E+01	3.89E+04	84	TRUE	TRUE
1	VVKGDDVRQ	388	396	9	HLA-A*02:01	388	84	38863.79	8.40E+01	3.89E+04	84	TRUE	TRUE
1	LKPTKRSFI	792	800	9	HLA-A*02:01	792	85	38900.8	8.50E+01	3.89E+04	85	TRUE	TRUE
1	YDPLQPELD	1120	1128	9	HLA-A*02:01	1120	85	38947.56	8.50E+01	3.89E+04	85	TRUE	TRUE
1	DCSQNPLAE	277	285	9	HLA-A*02:01	277	85	38994.79	8.50E+01	3.90E+04	85	TRUE	TRUE
1	TGVLTPSSK	535	543	9	HLA-A*02:01	535	85	38994.79	8.50E+01	3.90E+04	85	TRUE	TRUE
1	LDKVEAEVQ	966	974	9	HLA-A*02:01	966	85	39019.69	8.50E+01	3.90E+04	85	TRUE	TRUE
1	KEELDKYFK	1131	1139	9	HLA-A*02:01	1131	85	39045.02	8.50E+01	3.90E+04	85	TRUE	TRUE
1	HTSSMRGVY	33	41	9	HLA-A*02:01	33	85	39053.48	8.50E+01	3.91E+04	85	TRUE	TRUE
1	LDRCTTFDD	16	24	9	HLA-A*02:01	16	85	39054.73	8.50E+01	3.91E+04	85	TRUE	TRUE
1	VRQIAPGQT	394	402	9	HLA-A*02:01	394	85	39099.54	8.50E+01	3.91E+04	85	TRUE	TRUE
1	NVTGFHTIN	65	73	9	HLA-A*02:01	65	85	39132.14	8.50E+01	3.91E+04	85	TRUE	TRUE
1	MKQYGECLG	816	824	9	HLA-A*02:01	816	86	39183.83	8.60E+01	3.92E+04	86	TRUE	TRUE
1	LPDPLKPTK	788	796	9	HLA-A*02:01	788	86	39216.92	8.60E+01	3.92E+04	86	TRUE	TRUE
1	NCTFEYISD	158	166	9	HLA-A*02:01	158	86	39218.62	8.60E+01	3.92E+04	86	TRUE	TRUE
1	NPLAELKCS	281	289	9	HLA-A*02:01	281	86	39244.93	8.60E+01	3.92E+04	86	TRUE	TRUE
1	GDDVRQIAP	391	399	9	HLA-A*02:01	391	86	39282.3	8.60E+01	3.93E+04	86	TRUE	TRUE
1	YDENGTITD	266	274	9	HLA-A*02:01	266	86	39298.04	8.60E+01	3.93E+04	86	TRUE	TRUE
1	DEDDSEPVL	1239	1247	9	HLA-A*02:01	1239	86	39362.72	8.60E+01	3.94E+04	86	TRUE	TRUE
1	SGSDLDRCT	12	20	9	HLA-A*02:01	12	86	39362.72	8.60E+01	3.94E+04	86	TRUE	TRUE
1	SGTATAGWT	861	869	9	HLA-A*02:01	861	86	39380.62	8.60E+01	3.94E+04	86	TRUE	TRUE
1	DRNTREVFA	757	765	9	HLA-A*02:01	757	87	39509.08	8.70E+01	3.95E+04	87	TRUE	TRUE
1	GWVFGSTMN	100	108	9	HLA-A*02:01	100	87	39513.36	8.70E+01	3.95E+04	87	TRUE	TRUE
1	NVPFSPDGK	457	465	9	HLA-A*02:01	457	87	39529.6	8.70E+01	3.95E+04	87	TRUE	TRUE
1	AEHVDTSYE	639	647	9	HLA-A*02:01	639	87	39550.99	8.70E+01	3.96E+04	87	TRUE	TRUE
1	TDNTFVSGN	1099	1107	9	HLA-A*02:01	1099	87	39556.99	8.70E+01	3.96E+04	87	TRUE	TRUE
1	IDATSTGNY	428	436	9	HLA-A*02:01	428	87	39580.54	8.70E+01	3.96E+04	87	TRUE	TRUE
1	DNAFNCTFE	154	162	9	HLA-A*02:01	154	87	39602.37	8.70E+01	3.96E+04	87	TRUE	TRUE
1	PAQDIWGTS	240	248	9	HLA-A*02:01	240	87	39609.68	8.70E+01	3.96E+04	87	TRUE	TRUE
1	NNTIAIPTN	691	699	9	HLA-A*02:01	691	87	39615.25	8.70E+01	3.96E+04	87	TRUE	TRUE
1	PCTPPALNC	466	474	9	HLA-A*02:01	466	87	39667.99	8.70E+01	3.97E+04	87	TRUE	TRUE
1	PVLKGVKLH	1245	1253	9	HLA-A*02:01	1245	87	39673.57	8.70E+01	3.97E+04	87	TRUE	TRUE
1	DDSEPVLKG	1241	1249	9	HLA-A*02:01	1241	87	39693.76	8.70E+01	3.97E+04	87	TRUE	TRUE
1	VNIQKEIDR	1159	1167	9	HLA-A*02:01	1159	87	39707.94	8.70E+01	3.97E+04	87	TRUE	TRUE
1	QVKQMYKTP	766	774	9	HLA-A*02:01	766	87	39719.54	8.70E+01	3.97E+04	87	TRUE	TRUE
1	QPELDSFKE	1124	1132	9	HLA-A*02:01	1124	87	39732.43	8.70E+01	3.97E+04	87	TRUE	TRUE
1	GFMKQYGEC	814	822	9	HLA-A*02:01	814	87	39736.73	8.70E+01	3.97E+04	87	TRUE	TRUE
1	SFPQAAPHG	1033	1041	9	HLA-A*02:01	1033	87	39736.73	8.70E+01	3.97E+04	87	TRUE	TRUE
1	PTTFMLKYD	259	267	9	HLA-A*02:01	259	87	39744.89	8.70E+01	3.97E+04	87	TRUE	TRUE
1	VLGQSKRVD	1015	1023	9	HLA-A*02:01	1015	88	39768.55	8.80E+01	3.98E+04	88	TRUE	TRUE
1	SNVPFSPDG	456	464	9	HLA-A*02:01	456	88	39779.32	8.80E+01	3.98E+04	88	TRUE	TRUE
1	FGSTMNNKS	103	111	9	HLA-A*02:01	103	88	39781.04	8.80E+01	3.98E+04	88	TRUE	TRUE
1	GSCCKFDED	1233	1241	9	HLA-A*02:01	1233	88	39782.32	8.80E+01	3.98E+04	88	TRUE	TRUE
1	VKSFEIDKG	290	298	9	HLA-A*02:01	290	88	39790.07	8.80E+01	3.98E+04	88	TRUE	TRUE
1	SSKRFQPFQ	541	549	9	HLA-A*02:01	541	88	39802.98	8.80E+01	3.98E+04	88	TRUE	TRUE
1	SGINASVVN	1152	1160	9	HLA-A*02:01	1152	88	39814.61	8.80E+01	3.98E+04	88	TRUE	TRUE
1	EDDSEPVLK	1240	1248	9	HLA-A*02:01	1240	88	39851.25	8.80E+01	3.99E+04	88	TRUE	TRUE
1	ADYNYKLPD	406	414	9	HLA-A*02:01	406	88	39881.46	8.80E+01	3.99E+04	88	TRUE	TRUE
1	DTSYECDIP	643	651	9	HLA-A*02:01	643	88	39887.91	8.80E+01	3.99E+04	88	TRUE	TRUE
1	GVSATKLND	368	376	9	HLA-A*02:01	368	88	39892.67	8.80E+01	3.99E+04	88	TRUE	TRUE
1	REFVFKNKD	183	191	9	HLA-A*02:01	183	88	39915.55	8.80E+01	3.99E+04	88	TRUE	TRUE
1	YENQKQIAN	899	907	9	HLA-A*02:01	899	88	39925.05	8.80E+01	3.99E+04	88	TRUE	TRUE
1	TRNIDATST	425	433	9	HLA-A*02:01	425	88	39930.66	8.80E+01	3.99E+04	88	TRUE	TRUE
1	MRGVYYPDE	37	45	9	HLA-A*02:01	37	88	39952.27	8.80E+01	4.00E+04	88	TRUE	TRUE
1	NKVTLADAG	806	814	9	HLA-A*02:01	806	88	39965.68	8.80E+01	4.00E+04	88	TRUE	TRUE
1	CGDSTECAN	725	733	9	HLA-A*02:01	725	88	39969.15	8.80E+01	4.00E+04	88	TRUE	TRUE

seq #	peptide	start	end	peptide length	allele	peptide index	median binding	ann IC50	ann percentile	legacy IC50	legacy rank	scores equal	percentiles equal	
1	DVQAPNYTQ	24	32	9	HLA-A*02:01	24	88	40000.72	8.80E+01	4.00E+04	88	TRUE	TRUE	
1	KPCTPPALN	465	473	9	HLA-A*02:01	465	88	40014.13	8.80E+01	4.00E+04	88	TRUE	TRUE	
1	TFMLKYDEN	261	269	9	HLA-A*02:01	261	88	40041.86	8.80E+01	4.00E+04	88	TRUE	TRUE	
1	DVSTAIHAD	605	613	9	HLA-A*02:01	605	89	40090.41	8.90E+01	4.01E+04	89	TRUE	TRUE	
1	ANLAATKMS	1004	1012	9	HLA-A*02:01	1004	89	40136.84	8.90E+01	4.01E+04	89	TRUE	TRUE	
1	REVFAQVKQ	761	769	9	HLA-A*02:01	761	89	40139.9	8.90E+01	4.01E+04	89	TRUE	TRUE	
1	TGIGYQPYR	487	495	9	HLA-A*02:01	487	89	40161.18	8.90E+01	4.02E+04	89	TRUE	TRUE	
1	TEKSNVVRG	92	100	9	HLA-A*02:01	92	89	40182.92	8.90E+01	4.02E+04	89	TRUE	TRUE	
1	GRDVSDFTD	552	560	9	HLA-A*02:01	552	89	40193.78	8.90E+01	4.02E+04	89	TRUE	TRUE	
1	MAKTSVDCN	713	721	9	HLA-A*02:01	713	89	40194.22	8.90E+01	4.02E+04	89	TRUE	TRUE	
1	EGKAYFPRE	1066	1074	9	HLA-A*02:01	1066	89	40195.52	8.90E+01	4.02E+04	89	TRUE	TRUE	
1	SGNFKHLRE	176	184	9	HLA-A*02:01	176	89	40201.17	8.90E+01	4.02E+04	89	TRUE	TRUE	
1	MYICGDSTE	722	730	9	HLA-A*02:01	722	89	40210.32	8.90E+01	4.02E+04	89	TRUE	TRUE	
1	KYRYLRHGK	439	447	9	HLA-A*02:01	439	89	40214.24	8.90E+01	4.02E+04	89	TRUE	TRUE	
1	VRDLPSGFN	206	214	9	HLA-A*02:01	206	89	40238.16	8.90E+01	4.02E+04	89	TRUE	TRUE	
1	WNTRNIDAT	423	431	9	HLA-A*02:01	423	89	40249.06	8.90E+01	4.02E+04	89	TRUE	TRUE	
1	QNVLYENQK	895	903	9	HLA-A*02:01	895	89	40252.09	8.90E+01	4.03E+04	89	TRUE	TRUE	
1	LGKLQDVVN	927	935	9	HLA-A*02:01	927	89	40330.12	8.90E+01	4.03E+04	89	TRUE	TRUE	
1	SKPMGTQTH	141	149	9	HLA-A*02:01	141	89	40337.56	8.90E+01	4.03E+04	89	TRUE	TRUE	
1	HVDTSYECD	641	649	9	HLA-A*02:01	641	89	40344.98	8.90E+01	4.03E+04	89	TRUE	TRUE	
1	LKCSVKSFE	286	294	9	HLA-A*02:01	286	89	40361.14	8.90E+01	4.04E+04	89	TRUE	TRUE	
1	PKLSTDLIK	513	521	9	HLA-A*02:01	513	90	40408.3	9.00E+01	4.04E+04	90	TRUE	TRUE	
1	TSTGNYNYK	431	439	9	HLA-A*02:01	431	90	40422.3	9.00E+01	4.04E+04	90	TRUE	TRUE	
1	TYVTQQLIR	988	996	9	HLA-A*02:01	988	90	40441.99	9.00E+01	4.04E+04	90	TRUE	TRUE	
1	YGVSATKLN	367	375	9	HLA-A*02:01	367	90	40452.95	9.00E+01	4.05E+04	90	TRUE	TRUE	
1	VDFCGKGYH	1022	1030	9	HLA-A*02:01	1022	90	40454.69	9.00E+01	4.05E+04	90	TRUE	TRUE	
1	PSVYAWERK	335	343	9	HLA-A*02:01	335	90	40462.57	9.00E+01	4.05E+04	90	TRUE	TRUE	
1	DVVRDLPSG	204	212	9	HLA-A*02:01	204	90	40510.75	9.00E+01	4.05E+04	90	TRUE	TRUE	
1	SDFTDSVRD	556	564	9	HLA-A*02:01	556	90	40603.78	9.00E+01	4.06E+04	90	TRUE	TRUE	
1	KNLNESLID	1173	1181	9	HLA-A*02:01	1173	90	40606.43	9.00E+01	4.06E+04	90	TRUE	TRUE	
1	LRSTSQKSI	666	674	9	HLA-A*02:01	666	90	40674.57	9.00E+01	4.07E+04	90	TRUE	TRUE	
1	TNVVIRACN	121	129	9	HLA-A*02:01	121	91	40770.19	9.10E+01	4.08E+04	91	TRUE	TRUE	
1	DYGFYTTTG	480	488	9	HLA-A*02:01	480	91	40790.5	9.10E+01	4.08E+04	91	TRUE	TRUE	
1	QPFQQFGRD	546	554	9	HLA-A*02:01	546	91	40844.8	9.10E+01	4.08E+04	91	TRUE	TRUE	
1	PGQTGVIAD	399	407	9	HLA-A*02:01	399	91	40853.65	9.10E+01	4.09E+04	91	TRUE	TRUE	
1	TGNYNYKYR	433	441	9	HLA-A*02:01	433	91	40874.42	9.10E+01	4.09E+04	91	TRUE	TRUE	
1	DPLQPELDS	1121	1129	9	HLA-A*02:01	1121	91	40914.69	9.10E+01	4.09E+04	91	TRUE	TRUE	
1	DSFVVKGDD	385	393	9	HLA-A*02:01	385	91	40916.46	9.10E+01	4.09E+04	91	TRUE	TRUE	
1	GVLTPSSKR	536	544	9	HLA-A*02:01	536	91	40964.3	9.10E+01	4.10E+04	91	TRUE	TRUE	
1	RDVSDFTDS	553	561	9	HLA-A*02:01	553	91	40982.93	9.10E+01	4.10E+04	91	TRUE	TRUE	
1	DNTFVSGNC	1100	1108	9	HLA-A*02:01	1100	91	40989.56	9.10E+01	4.10E+04	91	TRUE	TRUE	
1	LDKYFKNHT	1134	1142	9	HLA-A*02:01	1134	92	41068.6	9.20E+01	4.11E+04	92	TRUE	TRUE	
1	ENQKQIANQ	900	908	9	HLA-A*02:01	900	92	41078.38	9.20E+01	4.11E+04	92	TRUE	TRUE	
1	PSGFNTLKP	210	218	9	HLA-A*02:01	210	92	41085.48	9.20E+01	4.11E+04	92	TRUE	TRUE	
1	GKAYFPREG	1067	1075	9	HLA-A*02:01	1067	92	41086.38	9.20E+01	4.11E+04	92	TRUE	TRUE	
1	KYFKNHTSP	1136	1144	9	HLA-A*02:01	1136	92	41172.26	9.20E+01	4.12E+04	92	TRUE	TRUE	
1	EHVDTSYEC	640	648	9	HLA-A*02:01	640	92	41210.14	9.20E+01	4.12E+04	92	TRUE	TRUE	
1	QKEIDRLNE	1162	1170	9	HLA-A*02:01	1162	92	41357.99	9.20E+01	4.14E+04	92	TRUE	TRUE	
1	KPTKRSFIE	793	801	9	HLA-A*02:01	793	93	41390.22	9.30E+01	4.14E+04	93	TRUE	TRUE	
1	DAVDCSQNP	274	282	9	HLA-A*02:01	274	93	41421.14	9.30E+01	4.14E+04	93	TRUE	TRUE	
1	DDVQAPNYT	23	31	9	HLA-A*02:01	23	93	41454.76	9.30E+01	4.15E+04	93	TRUE	TRUE	
1	NKAISQIQE	910	918	9	HLA-A*02:01	910	93	41485.28	9.30E+01	4.15E+04	93	TRUE	TRUE	
1	VYAWERKKI	337	345	9	HLA-A*02:01	337	93	41556.26	9.30E+01	4.16E+04	93	TRUE	TRUE	
1	VVNIQKEID	1158	1166	9	HLA-A*02:01	1158	93	41595.84	9.30E+01	4.16E+04	93	TRUE	TRUE	
1	CKFDEDDSE	1236	1244	9	HLA-A*02:01	1236	93	41626	9.30E+01	4.16E+04	93	TRUE	TRUE	
1	QDVNCTDVS	599	607	9	HLA-A*02:01	599	93	41627.37	9.30E+01	4.16E+04	93	TRUE	TRUE	
1	SEPVLKGVK	1243	1251	9	HLA-A*02:01	1243	93	41635.02	9.30E+01	4.16E+04	93	TRUE	TRUE	
1	RNIDATSTG	426	434	9	HLA-A*02:01	426	93	41642.68	9.30E+01	4.16E+04	93	TRUE	TRUE	
1	VSMAKTSVD	711	719	9	HLA-A*02:01	711	93	41690.02	9.30E+01	4.17E+04	93	TRUE	TRUE	
1	AAEQDRNTR	753	761	9	HLA-A*02:01	753	93	41711.67	9.30E+01	4.17E+04	93	TRUE	TRUE	
1	VPSQERNFT	1050	1058	9	HLA-A*02:01	1050	93	41725.66	9.30E+01	4.17E+04	93	TRUE	TRUE	
1	DAGFMKQYG	812	820	9	HLA-A*02:01	812	94	41792.53	9.40E+01	4.18E+04	94	TRUE	TRUE	
1	NEVAKNLNE	1169	1177	9	HLA-A*02:01	1169	94	41858.59	9.40E+01	4.19E+04	94	TRUE	TRUE	
1	PGTNASSEV	586	594	9	HLA-A*02:01	586	94	41899.83	9.40E+01	4.19E+04	94	TRUE	TRUE	
1	LYENQKQIA	898	906	9	HLA-A*02:01	898	94	41927.51	9.40E+01	4.19E+04	94	TRUE	TRUE	
1	APNYTQHTS	27	35	9	HLA-A*02:01	27	94	41937.92	9.40E+01	4.19E+04	94	TRUE	TRUE	
1	PTKRSFIED	794	802	9	HLA-A*02:01	794	94	41965.17	9.40E+01	4.20E+04	94	TRUE	TRUE	
1	NVLYENQKQ	896	904	9	HLA-A*02:01	896	94	41995.6	9.40E+01	4.20E+04	94	TRUE	TRUE	
1	NNTVYDPLQ	1116	1124	9	HLA-A*02:01	1116	94	42034.7	9.40E+01	4.20E+04	94	TRUE	TRUE	
1	YFAATEKSN	88	96	9	HLA-A*02:01	88	94	42045.16	9.40E+01	4.20E+04	94	TRUE	TRUE	
1	NGTITDAVD	269	277	9	HLA-A*02:01	269	94	42077.01	9.40E+01	4.21E+04	94	TRUE	TRUE	
1	QKQIANQFN	902	910	9	HLA-A*02:01	902	95	42122.57	9.50E+01	4.21E+04	95	TRUE	TRUE	
1	DDVRQIAPG	392	400	9	HLA-A*02:01	392	95	42151.29	9.50E+01	4.22E+04	95	TRUE	TRUE	
1	PDGKPCTPP	462	470	9	HLA-A*02:01	462	95	42198.75	9.50E+01	4.22E+04	95	TRUE	TRUE	
1	NNVFQTQAG	626	634	9	HLA-A*02:01	626	95	42210.62	9.50E+01	4.22E+04	95	TRUE	TRUE	
1	ANQFNKAIS	906	914	9	HLA-A*02:01	906	95	42237.58	9.50E+01	4.22E+04	95	TRUE	TRUE	
1	RHGKLRPFE	444	452	9	HLA-A*02:01	444	95	42239.87	9.50E+01	4.22E+04	95	TRUE	TRUE	
1	SVRDPKTSE	561	569	9	HLA-A*02:01	561	95	42281.92	9.50E+01	4.23E+04	95	TRUE	TRUE	
1	LGKYEQYIK	1185	1193	9	HLA-A*02:01	1185	95	42340.53	9.50E+01	4.23E+04	95	TRUE	TRUE	
1	RYLRHGKLR	441	449	9	HLA-A*02:01	441	95	42373.53	9.50E+01	4.24E+04	95	TRUE	TRUE	
1	CCKFDEDDS	1235	1243	9	HLA-A*02:01	1235	95	42377.66	9.50E+01	4.24E+04	95	TRUE	TRUE	
1	SEKSGNFKH	173	181	9	HLA-A*02:01	173	95	42420.78	9.50E+01	4.24E+04	95	TRUE	TRUE	
1	SGIAAEQDR	750	758	9	HLA-A*02:01	750	95	42452.91	9.50E+01	4.25E+04	95	TRUE	TRUE	
1	VPFSPDGKP	458	466	9	HLA-A*02:01	458	95	42477.72	9.50E+01	4.25E+04	95	TRUE	TRUE	
1	DSSIAYSNN	684	692	9	HLA-A*02:01	684	96	42545.33	9.60E+01	4.25E+04	96	TRUE	TRUE	
1	LDSFKEELD	1127	1135	9	HLA-A*02:01	1127	96	42578.05	9.60E+01	4.26E+04	96	TRUE	TRUE	
1	LTPSSKRFQ	538	546	9	HLA-A*02:01	538	96	42578.95	9.60E+01	4.26E+04	96	TRUE	TRUE	
1	GKLRPFERD	446	454	9	HLA-A*02:01	446	96	42617.22	9.60E+01	4.26E+04	96	TRUE	TRUE	
1	DKYFKNHTS	1135	1143	9	HLA-A*02:01	1135	96	42652.28	9.60E+01	4.27E+04	96	TRUE	TRUE	
1	GIAAEQDRN	751	759	9	HLA-A*02:01	751	96	42900.81	9.60E+01	4.29E+04	96	TRUE	TRUE	
1	TPSSKRFQP	539	547	9	HLA-A*02:01	539	96	42904.07	9.60E+01	4.29E+04	96	TRUE	TRUE	
1	LNEVAKNLN	1168	1176	9	HLA-A*02:01	1168	96	42959.8	9.60E+01	4.30E+04	96	TRUE	TRUE	
1	PDEIFRSDT	43	51	9	HLA-A*02:01	43	97	43030.04	9.70E+01	4.30E+04	97	TRUE	TRUE	
1	DFTDSVRDP	557	565	9	HLA-A*02:01	557	97	43054.72	9.70E+01	4.31E+04	97	TRUE	TRUE	
1	TDAVDCSQN	273	281	9	HLA-A*02:01	273	97	43062.66	9.70E+01	4.31E+04	97	TRUE	TRUE	
1	YFKNHTSPD	1137	1145	9	HLA-A*02:01	1137	97	43067.3	9.70E+01	4.31E+04	97	TRUE	TRUE	
1	PAICHEGKA	1061	1069	9	HLA-A*02:01	1061	97	43186.28	9.70E+01	4.32E+04	97	TRUE	TRUE	
1	DATSTGNYN	429	437	9	HLA-A*02:01	429	97	43200.32	9.70E+01	4.32E+04	97	TRUE	TRUE	
1	PDPLKPTKR	789	797	9	HLA-A*02:01	789	97	43228.83	9.70E+01	4.32E+04	97	TRUE	TRUE	
1	ECVLGQSKR	1013	1021	9	HLA-A*02:01	1013	97	43337.02	97	43337.02	97	TRUE	TRUE	
1	VNQNAQALN	934	942	9	HLA-A*02:01	934	97	43440.3	97	43440.3	97	TRUE	TRUE	
1	DPKTSEILD	564	572	9	HLA-A*02:01	564	97	43461.46	97	43461.46	97	TRUE	TRUE	

seq #	peptide	start	end	peptide length	allele	peptide index	median binding	ann IC50	ann percentile	legacy IC50	legacy rank	scores equal	percentiles equal	
1	EELDKYFKN	1132	1140	9	HLA-A*02:01	1132	98	43467.57	98	43467.57	98	TRUE	TRUE	
1	QDVVNQNAQ	931	939	9	HLA-A*02:01	931	98	43558.91	98	43558.91	98	TRUE	TRUE	
1	PELDSFKEE	1125	1133	9	HLA-A*02:01	1125	98	43608.41	98	43608.41	98	TRUE	TRUE	
1	DYNYKLPDD	407	415	9	HLA-A*02:01	407	98	43630.61	98	43630.61	98	TRUE	TRUE	
1	AWERKKISN	339	347	9	HLA-A*02:01	339	98	43798.97	98	43798.97	98	TRUE	TRUE	
1	LRPFERDIS	448	456	9	HLA-A*02:01	448	98	43836.44	98	43836.44	98	TRUE	TRUE	
1	GSTMNNKSQ	104	112	9	HLA-A*02:01	104	98	43840.71	98	43840.71	98	TRUE	TRUE	
1	TDSVRDPKT	559	567	9	HLA-A*02:01	559	98	43854	98	43854	98	TRUE	TRUE	
1	WRIYSTGNN	619	627	9	HLA-A*02:01	619	98	43914.28	98	43914.28	98	TRUE	TRUE	
1	DRLNEVAKN	1166	1174	9	HLA-A*02:01	1166	98	44014.65	98	44014.65	98	TRUE	TRUE	
1	PFSPDGKPC	459	467	9	HLA-A*02:01	459	99	44025.61	99	44025.61	99	TRUE	TRUE	
1	TPGTNASSE	585	593	9	HLA-A*02:01	585	99	44042.28	99	44042.28	99	TRUE	TRUE	
1	PDVDLGDIS	1144	1152	9	HLA-A*02:01	1144	99	44249.11	99	44249.11	99	TRUE	TRUE	
1	KNHTSPDVD	1139	1147	9	HLA-A*02:01	1139	99	44272.57	99	44272.57	99	TRUE	TRUE	
1	AEQDRNTRE	754	762	9	HLA-A*02:01	754	99	44452.1	99	44452.1	99	TRUE	TRUE	
1	DSVRDPKTS	560	568	9	HLA-A*02:01	560	99	44596.13	99	44596.13	99	TRUE	TRUE	
1	EFVFKNKDG	184	192	9	HLA-A*02:01	184	100	44753.7	100	44753.7	100	TRUE	TRUE	
1	TYVPSQERN	1048	1056	9	HLA-A*02:01	1048	100	44953.18	100	44953.18	100	TRUE	TRUE	
1	DKGIYQTSN	296	304	9	HLA-A*02:01	296	100	45031.55	100	45031.55	100	TRUE	TRUE	
1	VYKGYQPID	196	204	9	HLA-A*02:01	196	100	45094.44	100	45094.44	100	TRUE	TRUE	
1	DPLKPTKRS	790	798	9	HLA-A*02:01	790	100	45117.39	100	45117.39	100	TRUE	TRUE	
1	PNYTQHTSS	28	36	9	HLA-A*02:01	28	100	45424.99	100	45424.99	100	TRUE	TRUE	
1	LDVSEKSGN	170	178	9	HLA-A*02:01	170	100	45758.45	100	45758.45	100	TRUE	TRUE	

seq #	peptide	start	end	peptide length	allele	peptide index	median binding	smmpmbec IC5	smmpmbec pe	legacy IC50	legacy rank	scores equal	percentiles equal
1	FIAGLIAIV	1202	1210	9	HLA-A*02:01	1202	0.4	20.53194064	0.4	20.53194064	0.4	TRUE	TRUE
1	RLQSLQTYV	982	990	9	HLA-A*02:01	982	0.7	36.5115273	0.7	36.5115273	0.7	TRUE	TRUE
1	FIFLLFLTL	2	10	9	HLA-A*02:01	2	0.7	41.44097837	0.7	41.44097837	0.7	TRUE	TRUE
1	VIADYNYKL	404	412	9	HLA-A*02:01	404	0.8	48.68890416	0.8	48.68890416	0.8	TRUE	TRUE
1	YLKPTTFML	256	264	9	HLA-A*02:01	256	1	53.63274187	1	53.63274187	1	TRUE	TRUE
1	VLNDILSRL	958	966	9	HLA-A*02:01	958	1.1	56.41959964	1.1	56.41959964	1.1	TRUE	TRUE
1	RLDKVEAEV	965	973	9	HLA-A*02:01	965	1.1	58.000268	1.1	58.000268	1.1	TRUE	TRUE
1	VVFLHVTYV	1042	1050	9	HLA-A*02:01	1042	1.2	61.4369999	1.2	61.4369999	1.2	TRUE	TRUE
1	MIAAYTAAL	851	859	9	HLA-A*02:01	851	1.2	62.72345896	1.2	62.72345896	1.2	TRUE	TRUE
1	LLFNKVTLA	803	811	9	HLA-A*02:01	803	1.2	62.86805146	1.2	62.86805146	1.2	TRUE	TRUE
1	LLTDDMIAA	846	854	9	HLA-A*02:01	846	1.3	70.2150161	1.3	70.2150161	1.3	TRUE	TRUE
1	HLMSFPQAA	1030	1038	9	HLA-A*02:01	1030	1.6	96.2564853	1.6	96.2564853	1.6	TRUE	TRUE
1	FSISITTEV	700	708	9	HLA-A*02:01	700	1.8	107.5053308	1.8	107.5053308	1.8	TRUE	TRUE
1	NVYADSFVV	381	389	9	HLA-A*02:01	381	2.3	151.1576042	2.3	151.1576042	2.3	TRUE	TRUE
1	SIVAYTMSL	673	681	9	HLA-A*02:01	673	2.3	152.9079268	2.3	152.9079268	2.3	TRUE	TRUE
1	FLLFLTLTS	4	12	9	HLA-A*02:01	4	2.4	165.7410172	2.4	165.7410172	2.4	TRUE	TRUE
1	KQMYKTPTL	768	776	9	HLA-A*02:01	768	2.4	168.0467045	2.4	168.0467045	2.4	TRUE	TRUE
1	VLYNSTFFS	354	362	9	HLA-A*02:01	354	2.5	178.4144395	2.5	178.4144395	2.5	TRUE	TRUE
1	QYIKWPWYV	1190	1198	9	HLA-A*02:01	1190	2.6	183.8357622	2.6	183.8357622	2.6	TRUE	TRUE
1	SITTEVMPV	703	711	9	HLA-A*02:01	703	2.7	201.1083024	2.7	201.1083024	2.7	TRUE	TRUE
1	ALVSGTATA	858	866	9	HLA-A*02:01	858	2.8	204.8471849	2.8	204.8471849	2.8	TRUE	TRUE
1	NLNESLIDL	1174	1182	9	HLA-A*02:01	1174	2.8	210.1019097	2.8	210.1019097	2.8	TRUE	TRUE
1	YVYKGYQPI	195	203	9	HLA-A*02:01	195	2.9	211.0716961	2.9	211.0716961	2.9	TRUE	TRUE
1	GLTVLPPLL	839	847	9	HLA-A*02:01	839	2.9	212.5347752	2.9	212.5347752	2.9	TRUE	TRUE
1	FVSGNCDVV	1103	1111	9	HLA-A*02:01	1103	3.1	233.576959	3.1	233.576959	3.1	TRUE	TRUE
1	GLIAIVMVT	1205	1213	9	HLA-A*02:01	1205	3.1	240.674457	3.1	240.674457	3.1	TRUE	TRUE
1	ELCDNPFFA	131	139	9	HLA-A*02:01	131	3.2	242.90139	3.2	242.90139	3.2	TRUE	TRUE
1	YLTQDLFLP	53	61	9	HLA-A*02:01	53	3.2	243.4613355	3.2	243.4613355	3.2	TRUE	TRUE
1	ATAGWTFGA	864	872	9	HLA-A*02:01	864	3.2	248.5592902	3.2	248.5592902	3.2	TRUE	TRUE
1	IITTDNTFV	1096	1104	9	HLA-A*02:01	1096	3.2	250.859181	3.2	250.859181	3.2	TRUE	TRUE
1	FLPFYSNVT	59	67	9	HLA-A*02:01	59	3.3	255.5230013	3.3	255.5230013	3.3	TRUE	TRUE
1	KQLSSNFGA	946	954	9	HLA-A*02:01	946	3.3	259.6749161	3.3	259.6749161	3.3	TRUE	TRUE
1	GTSAAAYFV	246	254	9	HLA-A*02:01	246	3.3	261.4748966	3.3	261.4748966	3.3	TRUE	TRUE
1	IIINNSTNV	115	123	9	HLA-A*02:01	115	3.4	269.4201043	3.4	269.4201043	3.4	TRUE	TRUE
1	ILLCCMTSC	1214	1222	9	HLA-A*02:01	1214	3.4	272.5398421	3.4	272.5398421	3.4	TRUE	TRUE
1	AIVMVTILL	1208	1216	9	HLA-A*02:01	1208	3.5	281.4686305	3.5	281.4686305	3.5	TRUE	TRUE
1	ELLNAPATV	502	510	9	HLA-A*02:01	502	3.6	288.688843	3.6	288.688843	3.6	TRUE	TRUE
1	ALGKLQDVV	926	934	9	HLA-A*02:01	926	3.7	305.7947324	3.7	305.7947324	3.7	TRUE	TRUE
1	VLSFELLNA	498	506	9	HLA-A*02:01	498	3.8	322.4259588	3.8	322.4259588	3.8	TRUE	TRUE
1	TMNNKSQSV	106	114	9	HLA-A*02:01	106	3.8	325.4093295	3.8	325.4093295	3.8	TRUE	TRUE
1	CMTSCCSCL	1218	1226	9	HLA-A*02:01	1218	4	355.164868	4	355.164868	4	TRUE	TRUE
1	RVVVLSFEL	495	503	9	HLA-A*02:01	495	4.1	367.646131	4.1	367.646131	4.1	TRUE	TRUE
1	VIRACNFEL	124	132	9	HLA-A*02:01	124	4.1	375.3444512	4.1	375.3444512	4.1	TRUE	TRUE
1	VLAWNTRNI	420	428	9	HLA-A*02:01	420	4.1	376.2097095	4.1	376.2097095	4.1	TRUE	TRUE
1	SLTTTSTAL	919	927	9	HLA-A*02:01	919	4.1	377.9462147	4.1	377.9462147	4.1	TRUE	TRUE
1	YQDVNCTDV	598	606	9	HLA-A*02:01	598	4.1	384.9727594	4.1	384.9727594	4.1	TRUE	TRUE
1	LLQYGSFCT	735	743	9	HLA-A*02:01	735	4.1	386.7497129	4.1	386.7497129	4.1	TRUE	TRUE
1	FSTFKCYGV	361	369	9	HLA-A*02:01	361	4.1	388.5348685	4.1	388.5348685	4.1	TRUE	TRUE
1	VLYENQKQI	897	905	9	HLA-A*02:01	897	4.1	390.328264	4.1	390.328264	4.1	TRUE	TRUE
1	PLAELKCSV	282	290	9	HLA-A*02:01	282	4.2	404.0452449	4.2	404.0452449	4.2	TRUE	TRUE
1	FMKQYGECL	815	823	9	HLA-A*02:01	815	4.3	415.3650969	4.3	415.3650969	4.3	TRUE	TRUE
1	LIAIVMVTI	1206	1214	9	HLA-A*02:01	1206	4.3	415.3650969	4.3	415.3650969	4.3	TRUE	TRUE
1	FMGCVLAWN	416	424	9	HLA-A*02:01	416	4.4	436.9482457	4.4	436.9482457	4.4	TRUE	TRUE
1	KLNDLCFSN	373	381	9	HLA-A*02:01	373	4.5	450.2254077	4.5	450.2254077	4.5	TRUE	TRUE
1	AILTAFSPA	233	241	9	HLA-A*02:01	233	4.5	452.3035537	4.5	452.3035537	4.5	TRUE	TRUE
1	GIGYQPYRV	488	496	9	HLA-A*02:01	488	4.7	484.65199	4.7	484.65199	4.7	TRUE	TRUE
1	KLPDDFMGC	411	419	9	HLA-A*02:01	411	4.8	498.2301443	4.8	498.2301443	4.8	TRUE	TRUE
1	GICASYHTV	655	663	9	HLA-A*02:01	655	4.8	501.6837107	4.8	501.6837107	4.8	TRUE	TRUE
1	MIFDNAFNC	151	159	9	HLA-A*02:01	151	4.9	508.6628263	4.9	508.6628263	4.9	TRUE	TRUE
1	YQPYRVVVL	491	499	9	HLA-A*02:01	491	4.9	526.5383401	4.9	526.5383401	4.9	TRUE	TRUE
1	TQAGCLIGA	631	639	9	HLA-A*02:01	631	4.9	532.6353667	4.9	532.6353667	4.9	TRUE	TRUE
1	PLNDYGFYT	477	485	9	HLA-A*02:01	477	5	538.8029936	5	538.8029936	5	TRUE	TRUE
1	YQTSNFRVV	300	308	9	HLA-A*02:01	300	5	538.8029936	5	538.8029936	5	TRUE	TRUE
1	QIAPGQTGV	396	404	9	HLA-A*02:01	396	5	548.8200891	5	548.8200891	5	TRUE	TRUE
1	RLNEVAKNL	1167	1175	9	HLA-A*02:01	1167	5	550.0852501	5	550.0852501	5	TRUE	TRUE
1	WLGFIAGLI	1199	1207	9	HLA-A*02:01	1199	5.1	557.7376982	5.1	557.7376982	5.1	TRUE	TRUE
1	IAIPTNFSI	694	702	9	HLA-A*02:01	694	5.1	565.4966025	5.1	565.4966025	5.1	TRUE	TRUE
1	QMAYRFNGI	883	891	9	HLA-A*02:01	883	5.3	585.3693783	5.3	585.3693783	5.3	TRUE	TRUE
1	VWLGFIAGL	1198	1206	9	HLA-A*02:01	1198	5.3	589.426965	5.3	589.426965	5.3	TRUE	TRUE
1	ALNTLVKQL	940	948	9	HLA-A*02:01	940	5.4	607.337364	5.4	607.337364	5.4	TRUE	TRUE
1	IKWPWYVWL	1192	1200	9	HLA-A*02:01	1192	5.7	646.2938153	5.7	646.2938153	5.7	TRUE	TRUE
1	VLYQDVNCT	596	604	9	HLA-A*02:01	596	5.8	667.467309	5.8	667.467309	5.8	TRUE	TRUE
1	IINNSTNVV	116	124	9	HLA-A*02:01	116	5.9	681.4437301	5.9	681.4437301	5.9	TRUE	TRUE
1	FNATKFPSV	329	337	9	HLA-A*02:01	329	5.9	687.7490513	5.9	687.7490513	5.9	TRUE	TRUE
1	FMLKYDENG	262	270	9	HLA-A*02:01	262	6.4	762.833927	6.4	762.833927	6.4	TRUE	TRUE
1	MFIFLLFLT	1	9	9	HLA-A*02:01	1	6.5	800.6265723	6.5	800.6265723	6.5	TRUE	TRUE
1	LLLQYGSFC	734	742	9	HLA-A*02:01	734	6.6	826.856224	6.6	826.856224	6.6	TRUE	TRUE
1	YVTQQLIRA	989	997	9	HLA-A*02:01	989	6.8	857.8868286	6.8	857.8868286	6.8	TRUE	TRUE
1	RIYSTGNNV	620	628	9	HLA-A*02:01	620	6.9	875.8505358	6.9	875.8505358	6.9	TRUE	TRUE
1	VIGIINNTV	1111	1119	9	HLA-A*02:01	1111	6.9	883.9546811	6.9	883.9546811	6.9	TRUE	TRUE
1	SLGADSSIA	680	688	9	HLA-A*02:01	680	6.9	902.4642357	6.9	902.4642357	6.9	TRUE	TRUE
1	YVWLGFIAG	1197	1205	9	HLA-A*02:01	1197	7	908.7198189	7	908.7198189	7	TRUE	TRUE
1	LFLPFYSNV	58	66	9	HLA-A*02:01	58	7	910.8146345	7	910.8146345	7	TRUE	TRUE
1	IAAYTAALV	852	860	9	HLA-A*02:01	852	7.1	923.4853282	7.1	923.4853282	7.1	TRUE	TRUE
1	CTFEYISDA	159	167	9	HLA-A*02:01	159	7.1	932.030233	7.1	932.030233	7.1	TRUE	TRUE
1	QLNRALSGI	744	752	9	HLA-A*02:01	744	7.1	938.4907576	7.1	938.4907576	7.1	TRUE	TRUE
1	VMVTILLCC	1210	1218	9	HLA-A*02:01	1210	7.3	984.985869	7.3	984.985869	7.3	TRUE	TRUE
1	KEIDRLNEV	1163	1171	9	HLA-A*02:01	1163	7.3	991.8134649	7.3	991.8134649	7.3	TRUE	TRUE
1	AVDCSQNPL	275	283	9	HLA-A*02:01	275	7.5	1026.667939	7.5	1026.667939	7.5	TRUE	TRUE
1	FKDGIYFAA	83	91	9	HLA-A*02:01	83	7.5	1031.406823	7.5	1031.406823	7.5	TRUE	TRUE
1	LQIPFAMQM	876	884	9	HLA-A*02:01	876	7.5	1031.406823	7.5	1031.406823	7.5	TRUE	TRUE
1	FLHVTYVPS	1044	1052	9	HLA-A*02:01	1044	7.5	1040.950313	7.5	1040.950313	7.5	TRUE	TRUE
1	FVFNGTSWF	1077	1085	9	HLA-A*02:01	1077	7.5	1048.165836	7.5	1048.165836	7.5	TRUE	TRUE
1	QLSSNFGAI	947	955	9	HLA-A*02:01	947	7.5	1050.582107	7.5	1050.582107	7.5	TRUE	TRUE
1	KMSECVLGQ	1010	1018	9	HLA-A*02:01	1010	7.7	1092.521519	7.7	1092.521519	7.7	TRUE	TRUE
1	TSYECDIPI	644	652	9	HLA-A*02:01	644	7.8	1128.31406	7.8	1128.31406	7.8	TRUE	TRUE
1	LLFLTLTSG	5	13	9	HLA-A*02:01	5	7.8	1133.522121	7.8	1133.522121	7.8	TRUE	TRUE
1	AAAYFVGYL	249	257	9	HLA-A*02:01	249	8	1173.356545	8	1173.356545	8	TRUE	TRUE
1	SLIDLQELG	1178	1186	9	HLA-A*02:01	1178	8	1181.489864	8	1181.489864	8	TRUE	TRUE
1	MQMAYRFNG	882	890	9	HLA-A*02:01	882	8.2	1225.829306	8.2	1225.829306	8.2	TRUE	TRUE
1	TSVDCNMYI	716	724	9	HLA-A*02:01	716	8.2	1228.655135	8.2	1228.655135	8.2	TRUE	TRUE
1	GFIAGLIAI	1201	1209	9	HLA-A*02:01	1201	8.2	1231.487477	8.2	1231.487477	8.2	TRUE	TRUE

seq #	peptide	start	end	peptide length	allele	peptide index	median binding	smmpmbec IC5	smmpmbec pe	legacy IC50	legacy rank	scores equal	percentiles equal
1	VFNGTSWFI	1078	1086	9	HLA-A*02:01	1078	8.4	1292.498358	8.4	1292.498358	8.4	TRUE	TRUE
1	GINASVVNI	1153	1161	9	HLA-A*02:01	1153	8.4	1295.477874	8.4	1295.477874	8.4	TRUE	TRUE
1	DVSDFTDSV	554	562	9	HLA-A*02:01	554	8.7	1372.239764	8.7	1372.239764	8.7	TRUE	TRUE
1	ALNCYWPLN	471	479	9	HLA-A*02:01	471	8.7	1375.403104	8.7	1375.403104	8.7	TRUE	TRUE
1	FQTQAGCLI	629	637	9	HLA-A*02:01	629	8.8	1404.203335	8.8	1404.203335	8.8	TRUE	TRUE
1	ILPDPLKPT	787	795	9	HLA-A*02:01	787	8.9	1443.543914	8.9	1443.543914	8.9	TRUE	TRUE
1	YTAALVSGT	855	863	9	HLA-A*02:01	855	9	1470.381399	9	1470.381399	9	TRUE	TRUE
1	FPQAAPHGV	1034	1042	9	HLA-A*02:01	1034	9.1	1494.273175	9.1	1494.273175	9.1	TRUE	TRUE
1	LITGRLQSL	978	986	9	HLA-A*02:01	978	9.2	1504.630981	9.2	1504.630981	9.2	TRUE	TRUE
1	AISSVLNDI	954	962	9	HLA-A*02:01	954	9.3	1553.924807	9.3	1553.924807	9.3	TRUE	TRUE
1	SIAYSNNTI	686	694	9	HLA-A*02:01	686	9.7	1646.000641	9.7	1646.000641	9.7	TRUE	TRUE
1	AALQIPFAM	874	882	9	HLA-A*02:01	874	9.9	1711.70926	9.9	1711.70926	9.9	TRUE	TRUE
1	DMIAAYTAA	850	858	9	HLA-A*02:01	850	9.9	1715.655157	9.9	1715.655157	9.9	TRUE	TRUE
1	AAPHGVVFL	1037	1045	9	HLA-A*02:01	1037	9.9	1719.61015	9.9	1719.61015	9.9	TRUE	TRUE
1	DISGINASV	1150	1158	9	HLA-A*02:01	1150	9.9	1719.61015	9.9	1719.61015	9.9	TRUE	TRUE
1	FLYVYKGYQ	193	201	9	HLA-A*02:01	193	10	1739.522298	10	1739.522298	10	TRUE	TRUE
1	KLPLGINIT	221	229	9	HLA-A*02:01	221	10	1743.532311	10	1743.532311	10	TRUE	TRUE
1	FITQRNFFS	1085	1093	9	HLA-A*02:01	1085	10	1751.58009	10	1751.58009	10	TRUE	TRUE
1	YECDIPIGA	646	654	9	HLA-A*02:01	646	10	1755.617899	10	1755.617899	10	TRUE	TRUE
1	IYQTSNFRV	299	307	9	HLA-A*02:01	299	10	1759.665016	10	1759.665016	10	TRUE	TRUE
1	DLCFSNVYA	376	384	9	HLA-A*02:01	376	10	1763.721463	10	1763.721463	10	TRUE	TRUE
1	AISQIQESL	912	920	9	HLA-A*02:01	912	10	1763.721463	10	1763.721463	10	TRUE	TRUE
1	FAMQMAYRF	880	888	9	HLA-A*02:01	880	11	1796.511493	11	1796.511493	11	TRUE	TRUE
1	WTFGAGAAL	868	876	9	HLA-A*02:01	868	12	1817.314116	12	1817.314116	12	TRUE	TRUE
1	NTLKPIFKL	214	222	9	HLA-A*02:01	214	12	1821.503458	12	1821.503458	12	TRUE	TRUE
1	LCDNPFFAV	132	140	9	HLA-A*02:01	132	12	1825.702457	12	1825.702457	12	TRUE	TRUE
1	ELGKYEQYI	1184	1192	9	HLA-A*02:01	1184	12	1859.6448	12	1859.6448	12	TRUE	TRUE
1	YGFYTTTGI	481	489	9	HLA-A*02:01	481	12	1868.22853	12	1868.22853	12	TRUE	TRUE
1	ISDAFSLDV	164	172	9	HLA-A*02:01	164	12	1894.218178	12	1894.218178	12	TRUE	TRUE
1	GINITNFRA	225	233	9	HLA-A*02:01	225	12	1907.348271	12	1907.348271	12	TRUE	TRUE
1	DLPSGFNTL	208	216	9	HLA-A*02:01	208	12	1916.152191	12	1916.152191	12	TRUE	TRUE
1	ELDSFKEEL	1126	1134	9	HLA-A*02:01	1126	12	1960.78511	12	1960.78511	12	TRUE	TRUE
1	FIEDLLFNK	799	807	9	HLA-A*02:01	799	12	1978.92803	12	1978.92803	12	TRUE	TRUE
1	IVMVTILLC	1209	1217	9	HLA-A*02:01	1209	12	2001.842937	12	2001.842937	12	TRUE	TRUE
1	VVVLSFELL	496	504	9	HLA-A*02:01	496	12	2011.083024	12	2011.083024	12	TRUE	TRUE
1	FAATEKSNV	89	97	9	HLA-A*02:01	89	12	2015.719049	12	2015.719049	12	TRUE	TRUE
1	AQDIWGTSA	241	249	9	HLA-A*02:01	241	12	2029.691346	12	2029.691346	12	TRUE	TRUE
1	FSQILPDPL	784	792	9	HLA-A*02:01	784	12	2048.471849	12	2048.471849	12	TRUE	TRUE
1	FDEDDSEPV	1238	1246	9	HLA-A*02:01	1238	12	2053.194064	12	2053.194064	12	TRUE	TRUE
1	ILDISPCSF	570	578	9	HLA-A*02:01	570	12	2145.013353	12	2145.013353	12	TRUE	TRUE
1	MVTILLCCM	1211	1219	9	HLA-A*02:01	1211	12	2174.853461	12	2174.853461	12	TRUE	TRUE
1	PALNCYWPL	470	478	9	HLA-A*02:01	470	13	2194.977132	13	2194.977132	13	TRUE	TRUE
1	SNFGAISSV	950	958	9	HLA-A*02:01	950	13	2205.108686	13	2205.108686	13	TRUE	TRUE
1	IAIVMVTIL	1207	1215	9	HLA-A*02:01	1207	13	2266.887669	13	2266.887669	13	TRUE	TRUE
1	YFPREGVFV	1070	1078	9	HLA-A*02:01	1070	13	2309.03226	13	2309.03226	13	TRUE	TRUE
1	AGLIAIVMV	1204	1212	9	HLA-A*02:01	1204	13	2379.194789	13	2379.194789	13	TRUE	TRUE
1	VYDPLQPEL	1119	1127	9	HLA-A*02:01	1119	13	2395.68658	13	2395.68658	13	TRUE	TRUE
1	NGLTVLPPL	838	846	9	HLA-A*02:01	838	13	2401.209207	13	2401.209207	13	TRUE	TRUE
1	GAALQIPFA	873	881	9	HLA-A*02:01	873	13	2412.292686	13	2412.292686	13	TRUE	TRUE
1	YICGDSTEC	723	731	9	HLA-A*02:01	723	13	2537.639934	13	2537.639934	13	TRUE	TRUE
1	DLLFNKVTL	802	810	9	HLA-A*02:01	802	13	2567.024419	13	2567.024419	13	TRUE	TRUE
1	NAQALNTLV	937	945	9	HLA-A*02:01	937	14	2688.00455	14	2688.00455	14	TRUE	TRUE
1	FKLPLGINI	220	228	9	HLA-A*02:01	220	14	2694.201043	14	2694.201043	14	TRUE	TRUE
1	LLCCMTSCC	1215	1223	9	HLA-A*02:01	1215	14	2700.411818	14	2700.411818	14	TRUE	TRUE
1	LPDDFMGCV	412	420	9	HLA-A*02:01	412	14	2700.411818	14	2700.411818	14	TRUE	TRUE
1	NTREVFAQV	759	767	9	HLA-A*02:01	759	14	2719.130179	14	2719.130179	14	TRUE	TRUE
1	LQTYVTQQL	986	994	9	HLA-A*02:01	986	14	2860.421278	14	2860.421278	14	TRUE	TRUE
1	FFSPQIITT	1091	1099	9	HLA-A*02:01	1091	14	2880.248771	14	2880.248771	14	TRUE	TRUE
1	KLQDVVNQN	929	937	9	HLA-A*02:01	929	14	2886.88843	14	2886.88843	14	TRUE	TRUE
1	PLLTDDMIA	845	853	9	HLA-A*02:01	845	14	2906.899385	14	2906.899385	14	TRUE	TRUE
1	ASYHTVSLL	658	666	9	HLA-A*02:01	658	14	2913.60048	14	2913.60048	14	TRUE	TRUE
1	VAYTMSLGA	675	683	9	HLA-A*02:01	675	14	2967.768357	14	2967.768357	14	TRUE	TRUE
1	KNKDGFLYV	188	196	9	HLA-A*02:01	188	14	2981.466953	14	2981.466953	14	TRUE	TRUE
1	VMPVSMAKT	708	716	9	HLA-A*02:01	708	14	3002.133495	14	3002.133495	14	TRUE	TRUE
1	VVIGIINNT	1110	1118	9	HLA-A*02:01	1110	14	3072.062167	14	3072.062167	14	TRUE	TRUE
1	FRSDTLYLT	47	55	9	HLA-A*02:01	47	14	3086.242162	14	3086.242162	14	TRUE	TRUE
1	DILSRLDKV	961	969	9	HLA-A*02:01	961	15	3158.129977	15	3158.129977	15	TRUE	TRUE
1	QQLIRAAEI	992	1000	9	HLA-A*02:01	992	15	3158.129977	15	3158.129977	15	TRUE	TRUE
1	DIWGTSAAA	243	251	9	HLA-A*02:01	243	15	3209.445461	15	3209.445461	15	TRUE	TRUE
1	KLSTDLIKN	514	522	9	HLA-A*02:01	514	15	3239.142094	15	3239.142094	15	TRUE	TRUE
1	RVVPSGDVV	306	314	9	HLA-A*02:01	306	15	3246.609088	15	3246.609088	15	TRUE	TRUE
1	NMYICGDST	721	729	9	HLA-A*02:01	721	15	3269.113507	15	3269.113507	15	TRUE	TRUE
1	TLADAGFMK	809	817	9	HLA-A*02:01	809	15	3329.890865	15	3329.890865	15	TRUE	TRUE
1	CVLGQSKRV	1014	1022	9	HLA-A*02:01	1014	15	3345.260943	15	3345.260943	15	TRUE	TRUE
1	AATEKSNVV	90	98	9	HLA-A*02:01	90	15	3438.982771	15	3438.982771	15	TRUE	TRUE
1	YFVGYLKPT	252	260	9	HLA-A*02:01	252	15	3626.01837	15	3626.01837	15	TRUE	TRUE
1	AFNCTFEYI	156	164	9	HLA-A*02:01	156	16	3668.005684	16	3668.005684	16	TRUE	TRUE
1	LYLTQDLFL	52	60	9	HLA-A*02:01	52	16	3676.46131	16	3676.46131	16	TRUE	TRUE
1	YISDAFSLD	163	171	9	HLA-A*02:01	163	16	3693.431085	16	3693.431085	16	TRUE	TRUE
1	TMIFDNAFN	150	158	9	HLA-A*02:01	150	16	3701.945323	16	3701.945323	16	TRUE	TRUE
1	PIFKLPLGI	218	226	9	HLA-A*02:01	218	16	3796.907351	16	3796.907351	16	TRUE	TRUE
1	VQIDRLITG	973	981	9	HLA-A*02:01	973	16	3903.28264	16	3903.28264	16	TRUE	TRUE
1	HLREFVFKN	181	189	9	HLA-A*02:01	181	16	3985.015363	16	3985.015363	16	TRUE	TRUE
1	FKNHTSPDV	1138	1146	9	HLA-A*02:01	1138	16	4049.766653	16	4049.766653	16	TRUE	TRUE
1	CVNFNFNGL	524	532	9	HLA-A*02:01	524	16	4077.838295	16	4077.838295	16	TRUE	TRUE
1	TALGKLQDV	925	933	9	HLA-A*02:01	925	16	4125.057437	16	4125.057437	16	TRUE	TRUE
1	LTQDLFLPF	54	62	9	HLA-A*02:01	54	16	4134.566676	16	4134.566676	16	TRUE	TRUE
1	FGAGAALQI	870	878	9	HLA-A*02:01	870	16	4163.226123	16	4163.226123	16	TRUE	TRUE
1	GLTGTGVLT	531	539	9	HLA-A*02:01	531	17	4211.43401	17	4211.43401	17	TRUE	TRUE
1	LQDVVNQNA	930	938	9	HLA-A*02:01	930	17	4471.260778	17	4471.260778	17	TRUE	TRUE
1	ILTAFSPAQ	234	242	9	HLA-A*02:01	234	17	4491.89918	17	4491.89918	17	TRUE	TRUE
1	KWPWYVWLG	1193	1201	9	HLA-A*02:01	1193	17	4502.25408	17	4502.25408	17	TRUE	TRUE
1	KLRPFERDI	447	455	9	HLA-A*02:01	447	17	4512.632844	17	4512.632844	17	TRUE	TRUE
1	MSLGADSSI	679	687	9	HLA-A*02:01	679	17	4564.88667	17	4564.88667	17	TRUE	TRUE
1	FVGYLKPTT	253	261	9	HLA-A*02:01	253	17	4671.21654	17	4671.21654	17	TRUE	TRUE
1	IFLLFLTLT	3	11	9	HLA-A*02:01	3	17	4714.43877	17	4714.43877	17	TRUE	TRUE
1	YHLMSFPQA	1029	1037	9	HLA-A*02:01	1029	17	4725.306678	17	4725.306678	17	TRUE	TRUE
1	CASYHTVSL	657	665	9	HLA-A*02:01	657	18	5016.837107	18	5016.837107	18	TRUE	TRUE
1	FAVSKPMGT	138	146	9	HLA-A*02:01	138	18	5051.61216	18	5051.61216	18	TRUE	TRUE
1	SLQTYVTQQ	985	993	9	HLA-A*02:01	985	18	5133.694255	18	5133.694255	18	TRUE	TRUE
1	IAYSNNTIA	687	695	9	HLA-A*02:01	687	18	5133.694255	18	5133.694255	18	TRUE	TRUE
1	RAAEIRASA	996	1004	9	HLA-A*02:01	996	18	5205.111056	18	5205.111056	18	TRUE	TRUE

seq #	peptide	start	end	peptide length	allele	peptide index	median binding	smmpmbec IC5	smmpmbec pe	legacy IC50	legacy rank	scores equal	percentiles equal
1	CSFGGVSVI	576	584	9	HLA-A*02:01	576	18	5229.136757	18	5229.136757	18	TRUE	TRUE
1	SVVNIQKEI	1157	1165	9	HLA-A*02:01	1157	18	5277.521363	18	5277.521363	18	TRUE	TRUE
1	LQSLQTYVT	983	991	9	HLA-A*02:01	983	18	5314.103394	18	5314.103394	18	TRUE	TRUE
1	IFRSDTLYL	46	54	9	HLA-A*02:01	46	18	5326.35367	18	5326.35367	18	TRUE	TRUE
1	IHADQLTPA	610	618	9	HLA-A*02:01	610	18	5363.274187	18	5363.274187	18	TRUE	TRUE
1	VVRGWVFGS	97	105	9	HLA-A*02:01	97	18	5388.029936	18	5388.029936	18	TRUE	TRUE
1	SVDCNMYIC	717	725	9	HLA-A*02:01	717	18	5412.899952	18	5412.899952	18	TRUE	TRUE
1	GNCDVVIGI	1106	1114	9	HLA-A*02:01	1106	18	5425.377975	18	5425.377975	18	TRUE	TRUE
1	ALSGIAAEQ	748	756	9	HLA-A*02:01	748	19	5500.852501	19	5500.852501	19	TRUE	TRUE
1	SSMRGVYYP	35	43	9	HLA-A*02:01	35	19	5538.982589	19	5538.982589	19	TRUE	TRUE
1	TLYLTQDLF	51	59	9	HLA-A*02:01	51	19	5628.983816	19	5628.983816	19	TRUE	TRUE
1	FNFNGLTGT	527	535	9	HLA-A*02:01	527	19	5707.290781	19	5707.290781	19	TRUE	TRUE
1	VTLADAGFM	808	816	9	HLA-A*02:01	808	19	5746.85183	19	5746.85183	19	TRUE	TRUE
1	QIQESLTTT	915	923	9	HLA-A*02:01	915	19	5760.099692	19	5760.099692	19	TRUE	TRUE
1	SMRGVYYPD	36	44	9	HLA-A*02:01	36	19	5894.26965	19	5894.26965	19	TRUE	TRUE
1	ISPCSFGGV	573	581	9	HLA-A*02:01	573	19	6031.564828	19	6031.564828	19	TRUE	TRUE
1	YYPDEIFRS	41	49	9	HLA-A*02:01	41	19	6045.46902	19	6045.46902	19	TRUE	TRUE
1	YKGYQPIDV	197	205	9	HLA-A*02:01	197	19	6115.472252	19	6115.472252	19	TRUE	TRUE
1	WYVWLGFIA	1196	1204	9	HLA-A*02:01	1196	19	6186.286084	19	6186.286084	19	TRUE	TRUE
1	KSFEIDKGI	291	299	9	HLA-A*02:01	291	19	6229.16737	19	6229.16737	19	TRUE	TRUE
1	LNDLCFSNV	374	382	9	HLA-A*02:01	374	19	6229.16737	19	6229.16737	19	TRUE	TRUE
1	VVNQNAQAL	933	941	9	HLA-A*02:01	933	19	6257.9199	19	6257.9199	19	TRUE	TRUE
1	AQVKQMYKT	765	773	9	HLA-A*02:01	765	19	6286.805146	19	6286.805146	19	TRUE	TRUE
1	QIANQFNKA	904	912	9	HLA-A*02:01	904	20	6448.073808	20	6448.073808	20	TRUE	TRUE
1	STECANLLL	728	736	9	HLA-A*02:01	728	20	6477.836765	20	6477.836765	20	TRUE	TRUE
1	AATKMSECV	1007	1015	9	HLA-A*02:01	1007	20	6477.836765	20	6477.836765	20	TRUE	TRUE
1	FELCDNPFF	130	138	9	HLA-A*02:01	130	20	6659.321771	20	6659.321771	20	TRUE	TRUE
1	SQKSIVAYT	670	678	9	HLA-A*02:01	670	20	6720.939716	20	6720.939716	20	TRUE	TRUE
1	RLITGRLQS	977	985	9	HLA-A*02:01	977	20	6814.437301	20	6814.437301	20	TRUE	TRUE
1	CVADYSVLY	348	356	9	HLA-A*02:01	348	20	6814.4373	20	6814.4373	20	TRUE	TRUE
1	SMAKTSVDC	712	720	9	HLA-A*02:01	712	20	6830.146202	20	6830.146202	20	TRUE	TRUE
1	SVLYNSTFF	353	361	9	HLA-A*02:01	353	20	6957.1281	20	6957.1281	20	TRUE	TRUE
1	KCSVKSFEI	287	295	9	HLA-A*02:01	287	20	6973.165937	20	6973.165937	20	TRUE	TRUE
1	FQPFQQFGR	545	553	9	HLA-A*02:01	545	20	6989.240744	20	6989.240744	20	TRUE	TRUE
1	NIQKEIDRL	1160	1168	9	HLA-A*02:01	1160	20	7005.352608	20	7005.352608	20	TRUE	TRUE
1	TILLCCMTS	1213	1221	9	HLA-A*02:01	1213	20	7053.911393	20	7053.911393	20	TRUE	TRUE
1	SVIIINNST	113	121	9	HLA-A*02:01	113	21	7201.61666	21	7201.61666	21	TRUE	TRUE
1	GYQPYRVVV	490	498	9	HLA-A*02:01	490	21	7201.616655	21	7201.616655	21	TRUE	TRUE
1	KAYFPREGV	1068	1076	9	HLA-A*02:01	1068	21	7218.218096	21	7218.218096	21	TRUE	TRUE
1	AYSNNTIAI	688	696	9	HLA-A*02:01	688	21	7251.535877	21	7251.535877	21	TRUE	TRUE
1	STFFSTFKC	358	366	9	HLA-A*02:01	358	21	7335.504705	21	7335.504705	21	TRUE	TRUE
1	DQLTPAWRI	613	621	9	HLA-A*02:01	613	21	7420.445846	21	7420.445846	21	TRUE	TRUE
1	GVSVITPGT	580	588	9	HLA-A*02:01	580	21	7420.445846	21	7420.445846	21	TRUE	TRUE
1	VDLGDISGI	1146	1154	9	HLA-A*02:01	1146	21	7437.55174	21	7437.55174	21	TRUE	TRUE
1	KGYQPIDVV	198	206	9	HLA-A*02:01	198	21	7454.697068	21	7454.697068	21	TRUE	TRUE
1	AQKFNGLTV	834	842	9	HLA-A*02:01	834	21	7752.289811	21	7752.289811	21	TRUE	TRUE
1	STAIHADQL	607	615	9	HLA-A*02:01	607	21	7788.072755	21	7788.072755	21	TRUE	TRUE
1	MLKYDENGT	263	271	9	HLA-A*02:01	263	22	7987.851823	22	7987.851823	22	TRUE	TRUE
1	AGWTFGAGA	866	874	9	HLA-A*02:01	866	22	8043.220967	22	8043.220967	22	TRUE	TRUE
1	LLNAPATVC	503	511	9	HLA-A*02:01	503	22	8173.912794	22	8173.912794	22	TRUE	TRUE
1	CPFGEVFNA	323	331	9	HLA-A*02:01	323	22	8192.755609	22	8192.755609	22	TRUE	TRUE
1	YADSFVVKG	383	391	9	HLA-A*02:01	383	22	8192.755609	22	8192.755609	22	TRUE	TRUE
1	IEDLLFNKV	800	808	9	HLA-A*02:01	800	22	8192.755609	22	8192.755609	22	TRUE	TRUE
1	YTMSLGADS	677	685	9	HLA-A*02:01	677	22	8539.451966	22	8539.451966	22	TRUE	TRUE
1	NITNFRAIL	227	235	9	HLA-A*02:01	227	22	8559.137436	22	8559.137436	22	TRUE	TRUE
1	STGNNVFQT	623	631	9	HLA-A*02:01	623	22	8758.505358	22	8758.505358	22	TRUE	TRUE
1	PMGTQTHTM	143	151	9	HLA-A*02:01	143	22	8880.348305	22	8880.348305	22	TRUE	TRUE
1	SVLNDILSR	957	965	9	HLA-A*02:01	957	22	8900.819622	22	8900.819622	22	TRUE	TRUE
1	TFVSGNCDV	1102	1110	9	HLA-A*02:01	1102	22	9045.446306	22	9045.446306	22	TRUE	TRUE
1	STSQKSIVA	668	676	9	HLA-A*02:01	668	22	9066.298213	22	9066.298213	22	TRUE	TRUE
1	LIRAAEIRA	994	1002	9	HLA-A*02:01	994	23	9406.542023	23	9406.542023	23	TRUE	TRUE
1	GIGVTQNVL	890	898	9	HLA-A*02:01	890	23	9471.745057	23	9471.745057	23	TRUE	TRUE
1	VTILLCCMT	1212	1220	9	HLA-A*02:01	1212	23	9493.579684	23	9493.579684	23	TRUE	TRUE
1	ITTDNTFVS	1097	1105	9	HLA-A*02:01	1097	23	9559.386035	23	9559.386035	23	TRUE	TRUE
1	NASSEVAVL	589	597	9	HLA-A*02:01	589	23	9714.713563	23	9714.713563	23	TRUE	TRUE
1	NGIGVTQNV	889	897	9	HLA-A*02:01	889	23	9759.554643	23	9759.554643	23	TRUE	TRUE
1	SQIQESLTT	914	922	9	HLA-A*02:01	914	23	9782.05274	23	9782.05274	23	TRUE	TRUE
1	SISITTEVM	701	709	9	HLA-A*02:01	701	23	9782.05274	23	9782.05274	23	TRUE	TRUE
1	LYNSTFFST	355	363	9	HLA-A*02:01	355	23	9804.602701	23	9804.602701	23	TRUE	TRUE
1	QIPFAMQMA	877	885	9	HLA-A*02:01	877	23	9895.323572	23	9895.323572	23	TRUE	TRUE
1	CLGDINARD	822	830	9	HLA-A*02:01	822	23	10079.29137	23	10079.29137	23	TRUE	TRUE
1	FSLDVSEKS	168	176	9	HLA-A*02:01	168	23	10125.81527	23	10125.81527	23	TRUE	TRUE
1	EYISDAFSL	162	170	9	HLA-A*02:01	162	24	10337.84461	24	10337.84461	24	TRUE	TRUE
1	VRFPNITNL	314	322	9	HLA-A*02:01	314	24	10409.50313	24	10409.50313	24	TRUE	TRUE
1	TQDLFLPFY	55	63	9	HLA-A*02:01	55	24	10505.82107	24	10505.82107	24	TRUE	TRUE
1	SNCVADYSV	346	354	9	HLA-A*02:01	346	24	10530.03949	24	10530.03949	24	TRUE	TRUE
1	QMYKTPTLK	769	777	9	HLA-A*02:01	769	24	10603.03024	24	10603.03024	24	TRUE	TRUE
1	QTYVTQQLI	987	995	9	HLA-A*02:01	987	24	10676.52693	24	10676.52693	24	TRUE	TRUE
1	QKFNGLTVL	835	843	9	HLA-A*02:01	835	24	10701.13887	24	10701.13887	24	TRUE	TRUE
1	RGVYYPDEI	38	46	9	HLA-A*02:01	38	24	10875.01839	24	10875.01839	24	TRUE	TRUE
1	LMSFPQAAP	1031	1039	9	HLA-A*02:01	1031	24	10875.0184	24	10875.0184	24	TRUE	TRUE
1	VTQQLIRAA	990	998	9	HLA-A*02:01	990	24	10900.0879	24	10900.0879	24	TRUE	TRUE
1	SVITPGTNA	582	590	9	HLA-A*02:01	582	24	11026.30496	24	11026.30496	24	TRUE	TRUE
1	EIDKGIYQT	294	302	9	HLA-A*02:01	294	24	11026.30496	24	11026.30496	24	TRUE	TRUE
1	LTLTSGSDL	8	16	9	HLA-A*02:01	8	24	11283.1406	24	11283.1406	24	TRUE	TRUE
1	FGGFNFSQI	779	787	9	HLA-A*02:01	779	25	11545.95871	25	11545.95871	25	TRUE	TRUE
1	FSPDGKPCT	460	468	9	HLA-A*02:01	460	25	11545.95871	25	11545.95871	25	TRUE	TRUE
1	IPTNFSISI	696	704	9	HLA-A*02:01	696	25	11760.61411	25	11760.61411	25	TRUE	TRUE
1	SGFNTLKPI	211	219	9	HLA-A*02:01	211	25	11760.61411	25	11760.61411	25	TRUE	TRUE
1	YIKWPWYVW	1191	1199	9	HLA-A*02:01	1191	25	11787.72512	25	11787.72512	25	TRUE	TRUE
1	GCLIGAEHV	634	642	9	HLA-A*02:01	634	25	11842.13479	25	11842.13479	25	TRUE	TRUE
1	FVFKNKDGF	185	193	9	HLA-A*02:01	185	25	11979.26025	25	11979.26025	25	TRUE	TRUE
1	FNGLTGTGV	529	537	9	HLA-A*02:01	529	25	11979.26025	25	11979.26025	25	TRUE	TRUE
1	RNFTTAPAI	1055	1063	9	HLA-A*02:01	1055	25	12062.29651	25	12062.29651	25	TRUE	TRUE
1	LTDDMIAAY	847	855	9	HLA-A*02:01	847	25	12090.10298	25	12090.10298	25	TRUE	TRUE
1	ITTEVMPVS	704	712	9	HLA-A*02:01	704	25	12572.74189	25	12572.74189	25	TRUE	TRUE
1	GGFNFSQIL	780	788	9	HLA-A*02:01	780	26	12630.77503	26	12630.77503	26	TRUE	TRUE
1	VLPPLLTDD	842	850	9	HLA-A*02:01	842	26	12806.48662	26	12806.48662	26	TRUE	TRUE
1	KQIANQFNK	903	911	9	HLA-A*02:01	903	26	13104.78801	26	13104.78801	26	TRUE	TRUE
1	TMSLGADSS	678	686	9	HLA-A*02:01	678	26	13410.03774	26	13410.03774	26	TRUE	TRUE
1	INHTFGNPV	72	80	9	HLA-A*02:01	72	26	13659.34895	26	13659.34895	26	TRUE	TRUE
1	PWYVWLGFI	1195	1203	9	HLA-A*02:01	1195	26	13690.837	26	13690.837	26	TRUE	TRUE

seq #	peptide	start	end	peptide length	allele	peptide index	median binding	smmpmbec IC5	smmpmbec pe	legacy IC50	legacy rank	scores equal	percentiles equal
1	NAFNCTFEY	155	163	9	HLA-A*02:01	155	26	13722.39764	26	13722.39764	26	TRUE	TRUE
1	FLTLTSGSD	7	15	9	HLA-A*02:01	7	26	13945.36867	26	13945.36867	26	TRUE	TRUE
1	GIINNTVYD	1113	1121	9	HLA-A*02:01	1113	26	14204.63243	26	14204.63243	26	TRUE	TRUE
1	FCGKGYHLM	1024	1032	9	HLA-A*02:01	1024	26	14237.37749	26	14237.37749	26	TRUE	TRUE
1	CSQNPLAEL	278	286	9	HLA-A*02:01	278	26	14303.09424	26	14303.09424	26	TRUE	TRUE
1	SFIEDLLFN	798	806	9	HLA-A*02:01	798	26	14402.23855	26	14402.23855	26	TRUE	TRUE
1	EQDRNTREV	755	763	9	HLA-A*02:01	755	27	14502.0701	27	14502.0701	27	TRUE	TRUE
1	SNVTGFHTI	64	72	9	HLA-A*02:01	64	27	14502.0701	27	14502.0701	27	TRUE	TRUE
1	TTSTALGKL	922	930	9	HLA-A*02:01	922	27	14737.70978	27	14737.70978	27	TRUE	TRUE
1	SNVYADSFV	380	388	9	HLA-A*02:01	380	27	14874.07613	27	14874.07613	27	TRUE	TRUE
1	IINNTVYDP	1114	1122	9	HLA-A*02:01	1114	27	14874.07613	27	14874.07613	27	TRUE	TRUE
1	VTQNVLYEN	893	901	9	HLA-A*02:01	893	27	14942.73175	27	14942.73175	27	TRUE	TRUE
1	SLLRSTSQK	664	672	9	HLA-A*02:01	664	27	14977.1783	27	14977.1783	27	TRUE	TRUE
1	EILDISPCS	569	577	9	HLA-A*02:01	569	27	15080.99514	27	15080.99514	27	TRUE	TRUE
1	KTSEILDIS	566	574	9	HLA-A*02:01	566	27	15185.5316	27	15185.5316	27	TRUE	TRUE
1	CLIGAEHVD	635	643	9	HLA-A*02:01	635	27	15255.62483	27	15255.62483	27	TRUE	TRUE
1	ISGINASVV	1151	1159	9	HLA-A*02:01	1151	27	15255.62483	27	15255.62483	27	TRUE	TRUE
1	LTPAWRIYS	615	623	9	HLA-A*02:01	615	27	15503.5088	27	15503.5088	27	TRUE	TRUE
1	GADSSIAYS	682	690	9	HLA-A*02:01	682	27	15539.24807	27	15539.24807	27	TRUE	TRUE
1	NLLLQYGSF	733	741	9	HLA-A*02:01	733	27	15539.24807	27	15539.24807	27	TRUE	TRUE
1	YLRHGKLRP	442	450	9	HLA-A*02:01	442	27	15937.85972	27	15937.85972	27	TRUE	TRUE
1	RASANLAAT	1001	1009	9	HLA-A*02:01	1001	27	15937.85972	27	15937.85972	27	TRUE	TRUE
1	VSATKLNDL	369	377	9	HLA-A*02:01	369	27	16011.42554	27	16011.42554	27	TRUE	TRUE
1	SQERNFTTA	1052	1060	9	HLA-A*02:01	1052	27	16085.33092	27	16085.33092	27	TRUE	TRUE
1	NLCPFGEVF	321	329	9	HLA-A*02:01	321	28	16309.10016	28	16309.10016	28	TRUE	TRUE
1	DLFLPFYSN	57	65	9	HLA-A*02:01	57	28	16346.69652	28	16346.69652	28	TRUE	TRUE
1	FELLNAPAT	501	509	9	HLA-A*02:01	501	28	16460.00641	28	16460.00641	28	TRUE	TRUE
1	LKYDENGTI	264	272	9	HLA-A*02:01	264	28	16921.15486	28	16921.15486	28	TRUE	TRUE
1	YSNVTGFHT	63	71	9	HLA-A*02:01	63	28	16999.25936	28	16999.25936	28	TRUE	TRUE
1	KYFGGFNFS	777	785	9	HLA-A*02:01	777	28	17077.72437	28	17077.72437	28	TRUE	TRUE
1	VVLSFELLN	497	505	9	HLA-A*02:01	497	28	17117.0926	28	17117.0926	28	TRUE	TRUE
1	INARDLICA	826	834	9	HLA-A*02:01	826	28	17156.55157	28	17156.55157	28	TRUE	TRUE
1	LSFELLNAP	499	507	9	HLA-A*02:01	499	28	17235.74261	28	17235.74261	28	TRUE	TRUE
1	TNLCPFGEV	320	328	9	HLA-A*02:01	320	28	17235.74261	28	17235.74261	28	TRUE	TRUE
1	WVFGSTMNN	101	109	9	HLA-A*02:01	101	28	17435.32311	28	17435.32311	28	TRUE	TRUE
1	TLVKQLSSN	943	951	9	HLA-A*02:01	943	29	17515.8009	29	17515.8009	29	TRUE	TRUE
1	SSIAYSNNT	685	693	9	HLA-A*02:01	685	29	17882.57273	29	17882.57273	29	TRUE	TRUE
1	ITNFRAILT	228	236	9	HLA-A*02:01	228	29	18006.5288	29	18006.5288	29	TRUE	TRUE
1	PVSMAKTSV	710	718	9	HLA-A*02:01	710	29	18048.03814	29	18048.03814	29	TRUE	TRUE
1	AGICASYHT	654	662	9	HLA-A*02:01	654	29	18173.14116	29	18173.14116	29	TRUE	TRUE
1	FPSVYAWER	334	342	9	HLA-A*02:01	334	29	18341.29517	29	18341.29517	29	TRUE	TRUE
1	YFGGFNFSQ	778	786	9	HLA-A*02:01	778	29	18511.00508	29	18511.00508	29	TRUE	TRUE
1	GTNASSEVA	587	595	9	HLA-A*02:01	587	29	18596.448	29	18596.448	29	TRUE	TRUE
1	IAGLIAIVM	1203	1211	9	HLA-A*02:01	1203	29	18639.31724	29	18639.31724	29	TRUE	TRUE
1	TTFDDVQAP	20	28	9	HLA-A*02:01	20	29	18725.35242	29	18725.35242	29	TRUE	TRUE
1	VSGNCDVVI	1104	1112	9	HLA-A*02:01	1104	29	18725.35242	29	18725.35242	29	TRUE	TRUE
1	IANQFNKAI	905	913	9	HLA-A*02:01	905	29	18768.51881	29	18768.51881	29	TRUE	TRUE
1	PFKDGIYFA	82	90	9	HLA-A*02:01	82	29	18942.18178	29	18942.18178	29	TRUE	TRUE
1	KSNVVRGWV	94	102	9	HLA-A*02:01	94	29	19029.61492	29	19029.61492	29	TRUE	TRUE
1	TNASSEVAV	588	596	9	HLA-A*02:01	588	29	19029.61492	29	19029.61492	29	TRUE	TRUE
1	SPAQDIWGT	239	247	9	HLA-A*02:01	239	29	19117.45163	29	19117.45163	29	TRUE	TRUE
1	IVAYTMSLG	674	682	9	HLA-A*02:01	674	29	19205.69378	29	19205.69378	29	TRUE	TRUE
1	INNTVYDPL	1115	1123	9	HLA-A*02:01	1115	30	19698.35682	30	19698.35682	30	TRUE	TRUE
1	ISSVLNDIL	955	963	9	HLA-A*02:01	955	30	20018.42937	30	20018.42937	30	TRUE	TRUE
1	FFSTFKCYG	360	368	9	HLA-A*02:01	360	30	20018.42937	30	20018.42937	30	TRUE	TRUE
1	NITNLCPFG	318	326	9	HLA-A*02:01	318	30	20018.42937	30	20018.42937	30	TRUE	TRUE
1	NSTNVVIRA	119	127	9	HLA-A*02:01	119	30	20018.42937	30	20018.42937	30	TRUE	TRUE
1	FGNPVIPFK	76	84	9	HLA-A*02:01	76	30	20157.19049	30	20157.19049	30	TRUE	TRUE
1	QYGSFCTQL	737	745	9	HLA-A*02:01	737	30	20484.71849	30	20484.71849	30	TRUE	TRUE
1	FCTQLNRAL	741	749	9	HLA-A*02:01	741	30	20626.71177	30	20626.71177	30	TRUE	TRUE
1	ICAQKFNGL	832	840	9	HLA-A*02:01	832	30	20913.65792	30	20913.65792	30	TRUE	TRUE
1	MTSCCSCLK	1219	1227	9	HLA-A*02:01	1219	30	20961.86888	30	20961.86888	30	TRUE	TRUE
1	LNDYGFYTT	478	486	9	HLA-A*02:01	478	30	21107.16961	30	21107.16961	30	TRUE	TRUE
1	EVAVLYQDV	593	601	9	HLA-A*02:01	593	30	21155.82666	30	21155.82666	30	TRUE	TRUE
1	YSVLYNSTF	352	360	9	HLA-A*02:01	352	30	21204.59588	30	21204.59588	30	TRUE	TRUE
1	VFLHVTYVP	1043	1051	9	HLA-A*02:01	1043	31	21253.47752	31	21253.47752	31	TRUE	TRUE
1	KAISQIQES	911	919	9	HLA-A*02:01	911	31	22203.93769	31	22203.93769	31	TRUE	TRUE
1	FPREGVFVF	1071	1079	9	HLA-A*02:01	1071	31	22512.82524	31	22512.82524	31	TRUE	TRUE
1	GYLKPTTFM	255	263	9	HLA-A*02:01	255	31	22512.82524	31	22512.82524	31	TRUE	TRUE
1	SAAAYFVGY	248	256	9	HLA-A*02:01	248	31	22564.72266	31	22564.72266	31	TRUE	TRUE
1	GKPCTPPAL	464	472	9	HLA-A*02:01	464	31	22616.73972	31	22616.73972	31	TRUE	TRUE
1	YKLPDDFMG	410	418	9	HLA-A*02:01	410	31	22773.51147	31	22773.51147	31	TRUE	TRUE
1	LQYGSFCTQ	736	744	9	HLA-A*02:01	736	31	22773.51147	31	22773.51147	31	TRUE	TRUE
1	PVIPFKDGI	79	87	9	HLA-A*02:01	79	31	22878.62922	31	22878.62922	31	TRUE	TRUE
1	AMQMAYRFN	881	889	9	HLA-A*02:01	881	31	22984.23217	31	22984.23217	31	TRUE	TRUE
1	FKCYGVSAT	364	372	9	HLA-A*02:01	364	31	23250.37704	31	23250.37704	31	TRUE	TRUE
1	SFCTQLNRA	740	748	9	HLA-A*02:01	740	31	23411.54095	31	23411.54095	31	TRUE	TRUE
1	CVLAWNTRN	419	427	9	HLA-A*02:01	419	31	23465.51013	31	23465.51013	31	TRUE	TRUE
1	KISNCVADY	344	352	9	HLA-A*02:01	344	32	23682.63382	32	23682.63382	32	TRUE	TRUE
1	LKPIFKLPL	216	224	9	HLA-A*02:01	216	32	23956.8658	32	23956.8658	32	TRUE	TRUE
1	NFGAISSVL	951	959	9	HLA-A*02:01	951	32	24067.44566	32	24067.44566	32	TRUE	TRUE
1	VLTPSSKRF	537	545	9	HLA-A*02:01	537	32	24122.9269	32	24122.9269	32	TRUE	TRUE
1	SFELLNAPA	500	508	9	HLA-A*02:01	500	32	24178.5359	32	24178.5359	32	TRUE	TRUE
1	VITPGTNAS	583	591	9	HLA-A*02:01	583	32	24290.139	32	24290.139	32	TRUE	TRUE
1	FGRDVSDFT	551	559	9	HLA-A*02:01	551	32	24798.76197	32	24798.76197	32	TRUE	TRUE
1	GIYQTSNFR	298	306	9	HLA-A*02:01	298	32	25143.74711	32	25143.74711	32	TRUE	TRUE
1	NCTDVSTAI	602	610	9	HLA-A*02:01	602	32	25143.74711	32	25143.74711	32	TRUE	TRUE
1	ATKFPSVYA	331	339	9	HLA-A*02:01	331	32	25259.80537	32	25259.80537	32	TRUE	TRUE
1	TITDAVDCS	271	279	9	HLA-A*02:01	271	32	25259.80537	32	25259.80537	32	TRUE	TRUE
1	YQPIDVVRD	200	208	9	HLA-A*02:01	200	32	25376.39934	32	25376.39934	32	TRUE	TRUE
1	IAPGQTGVI	397	405	9	HLA-A*02:01	397	32	25434.89798	32	25434.89798	32	TRUE	TRUE
1	FNGTSWFIT	1079	1087	9	HLA-A*02:01	1079	32	25670.24419	32	25670.24419	32	TRUE	TRUE
1	HTINHTFGN	70	78	9	HLA-A*02:01	70	33	26818.22314	33	26818.22314	33	TRUE	TRUE
1	TPAWRIYST	616	624	9	HLA-A*02:01	616	33	26818.22314	33	26818.22314	33	TRUE	TRUE
1	ADYSVLYNS	350	358	9	HLA-A*02:01	350	33	26818.22314	33	26818.22314	33	TRUE	TRUE
1	KQYGECLGD	817	825	9	HLA-A*02:01	817	33	26942.01043	33	26942.01043	33	TRUE	TRUE
1	FSNVYADSF	379	387	9	HLA-A*02:01	379	33	27128.76353	33	27128.76353	33	TRUE	TRUE
1	FNFSQILPD	782	790	9	HLA-A*02:01	782	33	27191.30179	33	27191.30179	33	TRUE	TRUE
1	TNFRAILTA	229	237	9	HLA-A*02:01	229	33	27379.78289	33	27379.78289	33	TRUE	TRUE
1	GTATAGWTF	862	870	9	HLA-A*02:01	862	33	27888.81106	33	27888.81106	33	TRUE	TRUE
1	AEIRASANL	998	1006	9	HLA-A*02:01	998	33	27953.10141	33	27953.10141	33	TRUE	TRUE
1	AYRFNGIGV	885	893	9	HLA-A*02:01	885	34	28276.78306	34	28276.78306	34	TRUE	TRUE

seq #	peptide	start	end	peptide length	allele	peptide index	median binding	smmpmbec IC5	smmpmbec pe	legacy IC50	legacy rank	scores equal	percentiles equal	
1	GIYFAATEK	86	94	9	HLA-A*02:01	86	34	28538.42492	34	28538.42492	34	TRUE	TRUE	
1	ESLIDLQEL	1177	1185	9	HLA-A*02:01	1177	34	28802.48771	34	28802.48771	34	TRUE	TRUE	
1	VAKNLNESL	1171	1179	9	HLA-A*02:01	1171	34	29337.96594	34	29337.96594	34	TRUE	TRUE	
1	SQSVIIINN	111	119	9	HLA-A*02:01	111	34	29541.327	34	29541.327	34	TRUE	TRUE	
1	GVIADYNYK	403	411	9	HLA-A*02:01	403	34	29609.42679	34	29609.42679	34	TRUE	TRUE	
1	CDIPIGAGI	648	656	9	HLA-A*02:01	648	34	29677.68357	34	29677.68357	34	TRUE	TRUE	
1	TDLIKNQCV	517	525	9	HLA-A*02:01	517	34	29746.0977	34	29746.0977	34	TRUE	TRUE	
1	YEQYIKWPW	1188	1196	9	HLA-A*02:01	1188	34	29746.0977	34	29746.0977	34	TRUE	TRUE	
1	YWPLNDYGF	475	483	9	HLA-A*02:01	475	34	29883.39945	34	29883.39945	34	TRUE	TRUE	
1	IPIGAGICA	650	658	9	HLA-A*02:01	650	34	30090.54128	34	30090.54128	34	TRUE	TRUE	
1	GQSKRVDFC	1017	1025	9	HLA-A*02:01	1017	34	30159.90714	34	30159.90714	34	TRUE	TRUE	
1	DISPCSFGG	572	580	9	HLA-A*02:01	572	34	30299.11894	34	30299.11894	34	TRUE	TRUE	
1	TLTSGSDLD	9	17	9	HLA-A*02:01	9	34	30299.11894	34	30299.11894	34	TRUE	TRUE	
1	QFNKAISQI	908	916	9	HLA-A*02:01	908	34	30368.96563	34	30368.96563	34	TRUE	TRUE	
1	QILPDPLKP	786	794	9	HLA-A*02:01	786	34	30438.97332	34	30438.97332	34	TRUE	TRUE	
1	KTPTLKYFG	772	780	9	HLA-A*02:01	772	34	30509.1424	34	30509.1424	34	TRUE	TRUE	
1	HTMIFDNAF	149	157	9	HLA-A*02:01	149	34	30720.62167	34	30720.62167	34	TRUE	TRUE	
1	ITQRNFFSP	1086	1094	9	HLA-A*02:01	1086	35	30933.56685	35	30933.56685	35	TRUE	TRUE	
1	TAALVSGTA	856	864	9	HLA-A*02:01	856	35	31291.76067	35	31291.76067	35	TRUE	TRUE	
1	LICAQKFNG	831	839	9	HLA-A*02:01	831	35	31873.51794	35	31873.51794	35	TRUE	TRUE	
1	AVLYQDVNC	595	603	9	HLA-A*02:01	595	35	31946.994	35	31946.994	35	TRUE	TRUE	
1	INITNFRAI	226	234	9	HLA-A*02:01	226	35	32020.63941	35	32020.63941	35	TRUE	TRUE	
1	YNYKYRYLR	436	444	9	HLA-A*02:01	436	35	32168.43997	35	32168.43997	35	TRUE	TRUE	
1	DLDRCTTFD	15	23	9	HLA-A*02:01	15	35	32242.59588	35	32242.59588	35	TRUE	TRUE	
1	QDIWGTSAA	242	250	9	HLA-A*02:01	242	35	32242.59588	35	32242.59588	35	TRUE	TRUE	
1	GTSWFITQR	1081	1089	9	HLA-A*02:01	1081	35	32466.09088	35	32466.09088	35	TRUE	TRUE	
1	CTTFDDVQA	19	27	9	HLA-A*02:01	19	35	32917.73919	35	32917.73919	35	TRUE	TRUE	
1	LTVLPPLLT	840	848	9	HLA-A*02:01	840	35	32917.73919	35	32917.73919	35	TRUE	TRUE	
1	VDCSQNPLA	276	284	9	HLA-A*02:01	276	35	33069.68057	35	33069.68057	35	TRUE	TRUE	
1	IFDNAFNCT	152	160	9	HLA-A*02:01	152	35	33222.32328	35	33222.32328	35	TRUE	TRUE	
1	MGCVLAWNT	417	425	9	HLA-A*02:01	417	35	33452.60943	35	33452.60943	35	TRUE	TRUE	
1	GVFVFNGTS	1075	1083	9	HLA-A*02:01	1075	35	33452.60943	35	33452.60943	35	TRUE	TRUE	
1	HTVSLLRST	661	669	9	HLA-A*02:01	661	36	33839.97239	36	33839.97239	36	TRUE	TRUE	
1	IGYQPYRVV	489	497	9	HLA-A*02:01	489	36	33839.97239	36	33839.97239	36	TRUE	TRUE	
1	ICGDSTECA	724	732	9	HLA-A*02:01	724	36	33917.98158	36	33917.98158	36	TRUE	TRUE	
1	RAILTAFSP	232	240	9	HLA-A*02:01	232	36	34153.0898	36	34153.0898	36	TRUE	TRUE	
1	VRDPKTSEI	562	570	9	HLA-A*02:01	562	36	34310.7333	36	34310.7333	36	TRUE	TRUE	
1	VKGDDVRQI	389	397	9	HLA-A*02:01	389	36	34310.7333	36	34310.7333	36	TRUE	TRUE	
1	ALQIPFAMQ	875	883	9	HLA-A*02:01	875	36	34389.82771	36	34389.82771	36	TRUE	TRUE	
1	KPMGTQTHT	142	150	9	HLA-A*02:01	142	36	34469.10445	36	34469.10445	36	TRUE	TRUE	
1	NVFQTQAGC	627	635	9	HLA-A*02:01	627	36	35029.18234	36	35029.18234	36	TRUE	TRUE	
1	YTTTGIGYQ	484	492	9	HLA-A*02:01	484	36	35598.36076	36	35598.36076	36	TRUE	TRUE	
1	LLRSTSQKS	665	673	9	HLA-A*02:01	665	36	35845.11681	36	35845.11681	36	TRUE	TRUE	
1	DTLYLTQDL	50	58	9	HLA-A*02:01	50	36	36260.1837	36	36260.1837	36	TRUE	TRUE	
1	QPIDVVRDL	201	209	9	HLA-A*02:01	201	36	36260.1837	36	36260.1837	36	TRUE	TRUE	
1	LQPELDSFK	1123	1131	9	HLA-A*02:01	1123	36	36343.77206	36	36343.77206	36	TRUE	TRUE	
1	RGWVFGSTM	99	107	9	HLA-A*02:01	99	36	36511.52729	36	36511.52729	36	TRUE	TRUE	
1	FDNAFNCTF	153	161	9	HLA-A*02:01	153	36	36849.36429	36	36849.36429	36	TRUE	TRUE	
1	IRASANLAA	1000	1008	9	HLA-A*02:01	1000	36	36849.36429	36	36849.36429	36	TRUE	TRUE	
1	YRFNGIGVT	886	894	9	HLA-A*02:01	886	36	36849.36429	36	36849.36429	36	TRUE	TRUE	
1	VADYSVLYN	349	357	9	HLA-A*02:01	349	37	37190.32726	37	37190.32726	37	TRUE	TRUE	
1	VVRDLPSGF	205	213	9	HLA-A*02:01	205	37	37276.05982	37	37276.05982	37	TRUE	TRUE	
1	LAATKMSEC	1006	1014	9	HLA-A*02:01	1006	37	37448.11829	37	37448.11829	37	TRUE	TRUE	
1	NTIAIPTNF	692	700	9	HLA-A*02:01	692	37	37620.97095	37	37620.97095	37	TRUE	TRUE	
1	TKFPSVYAW	332	340	9	HLA-A*02:01	332	37	37881.74707	37	37881.74707	37	TRUE	TRUE	
1	QDLFLPFYS	56	64	9	HLA-A*02:01	56	37	38320.39702	37	38320.39702	37	TRUE	TRUE	
1	RNFFSPQII	1089	1097	9	HLA-A*02:01	1089	37	38853.48685	37	38853.48685	37	TRUE	TRUE	
1	WPWYVWLGF	1194	1202	9	HLA-A*02:01	1194	37	39032.8264	37	39032.8264	37	TRUE	TRUE	
1	CYWPLNDYG	474	482	9	HLA-A*02:01	474	37	39303.38902	37	39303.38902	37	TRUE	TRUE	
1	TAFSPAQDI	236	244	9	HLA-A*02:01	236	37	39303.38902	37	39303.38902	37	TRUE	TRUE	
1	FRAILTAFS	231	239	9	HLA-A*02:01	231	37	39303.38902	37	39303.38902	37	TRUE	TRUE	
1	RSTSQKSIV	667	675	9	HLA-A*02:01	667	37	39484.80522	37	39484.80522	37	TRUE	TRUE	
1	INNSTNVVI	117	125	9	HLA-A*02:01	117	37	39484.80522	37	39484.80522	37	TRUE	TRUE	
1	LFLTLTSGS	6	14	9	HLA-A*02:01	6	37	39667.0588	37	39667.0588	37	TRUE	TRUE	
1	EIDRLNEVA	1164	1172	9	HLA-A*02:01	1164	37	40126.3817	37	40126.3817	37	TRUE	TRUE	
1	NQNAQALNT	935	943	9	HLA-A*02:01	935	38	40497.66653	38	40497.66653	38	TRUE	TRUE	
1	AIHADQLTP	609	617	9	HLA-A*02:01	609	38	40591.0233	38	40591.0233	38	TRUE	TRUE	
1	PLQPELDSF	1122	1130	9	HLA-A*02:01	1122	38	40591.0233	38	40591.0233	38	TRUE	TRUE	
1	FEYISDAFS	161	169	9	HLA-A*02:01	161	38	40684.59527	38	40684.59527	38	TRUE	TRUE	
1	STGNYNYKY	432	440	9	HLA-A*02:01	432	38	40778.38295	38	40778.38295	38	TRUE	TRUE	
1	DISNVPFSP	454	462	9	HLA-A*02:01	454	38	40778.38295	38	40778.38295	38	TRUE	TRUE	
1	AWNTRNIDA	422	430	9	HLA-A*02:01	422	38	40966.60741	38	40966.60741	38	TRUE	TRUE	
1	QYGECLGDI	818	826	9	HLA-A*02:01	818	38	41155.7007	38	41155.7007	38	TRUE	TRUE	
1	FTDSVRDPK	558	566	9	HLA-A*02:01	558	38	41440.97837	38	41440.97837	38	TRUE	TRUE	
1	TIAIPTNFS	693	701	9	HLA-A*02:01	693	38	41824.42702	38	41824.42702	38	TRUE	TRUE	
1	DSTECANLL	727	735	9	HLA-A*02:01	727	38	42017.4798	38	42017.4798	38	TRUE	TRUE	
1	AAYFVGYLK	250	258	9	HLA-A*02:01	250	38	42406.26276	38	42406.26276	38	TRUE	TRUE	
1	QIDRLITGR	974	982	9	HLA-A*02:01	974	38	42504.01929	38	42504.01929	38	TRUE	TRUE	
1	ATKMSECVL	1008	1016	9	HLA-A*02:01	1008	38	42700.20893	38	42700.20893	38	TRUE	TRUE	
1	LVSGTATAG	859	867	9	HLA-A*02:01	859	38	42700.20893	38	42700.20893	38	TRUE	TRUE	
1	FEIDKGIYQ	293	301	9	HLA-A*02:01	293	38	42897.30414	38	42897.30414	38	TRUE	TRUE	
1	NLAATKMSE	1005	1013	9	HLA-A*02:01	1005	38	43194.65405	38	43194.65405	38	TRUE	TRUE	
1	TNFSISITT	698	706	9	HLA-A*02:01	698	38	43997.70262	38	43997.70262	38	TRUE	TRUE	
1	TVYDPLQPE	1118	1126	9	HLA-A*02:01	1118	38	43997.70262	38	43997.70262	38	TRUE	TRUE	
1	YNSTFFSTF	356	364	9	HLA-A*02:01	356	38	44099.1278	38	44099.1278	38	TRUE	TRUE	
1	AIPTNFSIS	695	703	9	HLA-A*02:01	695	39	44712.60778	39	44712.60778	39	TRUE	TRUE	
1	KGDDVRQIA	390	398	9	HLA-A*02:01	390	39	44815.68098	39	44815.68098	39	TRUE	TRUE	
1	QLIRAAEIR	993	1001	9	HLA-A*02:01	993	39	44918.9918	39	44918.9918	39	TRUE	TRUE	
1	LGFIAGLIA	1200	1208	9	HLA-A*02:01	1200	39	45022.5408	39	45022.5408	39	TRUE	TRUE	
1	FAQVKQMYK	764	772	9	HLA-A*02:01	764	39	45126.32844	39	45126.32844	39	TRUE	TRUE	
1	IQESLTTTS	916	924	9	HLA-A*02:01	916	39	45334.6221	39	45334.6221	39	TRUE	TRUE	
1	SLDVSEKSG	169	177	9	HLA-A*02:01	169	39	45543.87722	39	45543.87722	39	TRUE	TRUE	
1	TQLNRALSG	743	751	9	HLA-A*02:01	743	39	46497.54249	39	46497.54249	39	TRUE	TRUE	
1	FVVKGDDVR	387	395	9	HLA-A*02:01	387	39	46604.7304	39	46604.7304	39	TRUE	TRUE	
1	NKSQSVIII	109	117	9	HLA-A*02:01	109	39	46927.77896	39	46927.77896	39	TRUE	TRUE	
1	YSNNTIAIP	689	697	9	HLA-A*02:01	689	39	46927.77896	39	46927.77896	39	TRUE	TRUE	
1	AGAALQIPF	872	880	9	HLA-A*02:01	872	39	47035.95866	39	47035.95866	39	TRUE	TRUE	
1	VNCTDVSTA	601	609	9	HLA-A*02:01	601	39	47361.99635	39	47361.99635	39	TRUE	TRUE	
1	VFQTQAGCL	628	636	9	HLA-A*02:01	628	39	47471.17703	39	47471.17703	39	TRUE	TRUE	
1	DFMGCVLAW	415	423	9	HLA-A*02:01	415	39	47800.23151	39	47800.23151	39	TRUE	TRUE	
1	LTGTGVLTP	532	540	9	HLA-A*02:01	532	39	48020.86735	39	48020.86735	39	TRUE	TRUE	
1	FQQFGRDVS	548	556	9	HLA-A*02:01	548	39	48465.199	39	48465.199	39	TRUE	TRUE	

seq #	peptide	start	end	peptide length	allele	peptide index	median binding	smmpmbec IC5	smmpmbec pe	legacy IC50	legacy rank	scores equal	percentiles equal
1	DDMIAAYTA	849	857	9	HLA-A*02:01	849	39	48913.6419	39	48913.6419	39	TRUE	TRUE
1	DVVNQNAQA	932	940	9	HLA-A*02:01	932	40	49139.41704	40	49139.41704	40	TRUE	TRUE
1	YAWERKKIS	338	346	9	HLA-A*02:01	338	40	49480.03521	40	49480.03521	40	TRUE	TRUE
1	RDISNVPFS	453	461	9	HLA-A*02:01	453	40	50168.37107	40	50168.37107	40	TRUE	TRUE
1	NYNYKYRYL	435	443	9	HLA-A*02:01	435	40	50399.93775	40	50399.93775	40	TRUE	TRUE
1	LIGAEHVDT	636	644	9	HLA-A*02:01	636	40	50749.29342	40	50749.29342	40	TRUE	TRUE
1	FSPAQDIWG	238	246	9	HLA-A*02:01	238	40	51811.95743	40	51811.95743	40	TRUE	TRUE
1	ATSTGNYNY	430	438	9	HLA-A*02:01	430	40	52171.10076	40	52171.10076	40	TRUE	TRUE
1	QNAQALNTL	936	944	9	HLA-A*02:01	936	40	52171.10076	40	52171.10076	40	TRUE	TRUE
1	NGLTGTGVL	530	538	9	HLA-A*02:01	530	40	52171.10076	40	52171.10076	40	TRUE	TRUE
1	VFNATKFPS	328	336	9	HLA-A*02:01	328	40	52291.36757	40	52291.36757	40	TRUE	TRUE
1	KRSFIEDLL	796	804	9	HLA-A*02:01	796	40	52653.83401	40	52653.83401	40	TRUE	TRUE
1	KDGIYFAAT	84	92	9	HLA-A*02:01	84	40	52775.21363	40	52775.21363	40	TRUE	TRUE
1	FNCTFEYIS	157	165	9	HLA-A*02:01	157	41	54128.99952	41	54128.99952	41	TRUE	TRUE
1	LKGVKLHYT	1247	1255	9	HLA-A*02:01	1247	41	54128.99952	41	54128.99952	41	TRUE	TRUE
1	PQAAPHGVV	1035	1043	9	HLA-A*02:01	1035	41	54253.77975	41	54253.77975	41	TRUE	TRUE
1	SFVVKGDDV	386	394	9	HLA-A*02:01	386	41	54755.7838	41	54755.7838	41	TRUE	TRUE
1	SCCSCLKGA	1221	1229	9	HLA-A*02:01	1221	41	54882.00891	41	54882.00891	41	TRUE	TRUE
1	GDSTECANL	726	734	9	HLA-A*02:01	726	41	55008.52501	41	55008.52501	41	TRUE	TRUE
1	YKYRYLRHG	438	446	9	HLA-A*02:01	438	41	55135.33276	41	55135.33276	41	TRUE	TRUE
1	TFKCYGVSA	363	371	9	HLA-A*02:01	363	41	55517.51262	41	55517.51262	41	TRUE	TRUE
1	ITNLCPFGE	319	327	9	HLA-A*02:01	319	41	55645.49371	41	55645.49371	41	TRUE	TRUE
1	PLGINITNF	223	231	9	HLA-A*02:01	223	41	55773.76982	41	55773.76982	41	TRUE	TRUE
1	ITPGTNASS	584	592	9	HLA-A*02:01	584	41	56160.37512	41	56160.37512	41	TRUE	TRUE
1	AAYTAALVS	853	861	9	HLA-A*02:01	853	41	56289.83816	41	56289.83816	41	TRUE	TRUE
1	EVMPVSMAK	707	715	9	HLA-A*02:01	707	41	56419.59964	41	56419.59964	41	TRUE	TRUE
1	QIITTDNTF	1095	1103	9	HLA-A*02:01	1095	41	56680.02068	41	56680.02068	41	TRUE	TRUE
1	GDVVRFPNI	311	319	9	HLA-A*02:01	311	41	56810.68162	41	56810.68162	41	TRUE	TRUE
1	AALVSGTAT	857	865	9	HLA-A*02:01	857	41	56810.68162	41	56810.68162	41	TRUE	TRUE
1	KFNGLTVLP	836	844	9	HLA-A*02:01	836	42	57468.5183	42	57468.5183	42	TRUE	TRUE
1	TLKPIFKLP	215	223	9	HLA-A*02:01	215	42	58000.26796	42	58000.26796	42	TRUE	TRUE
1	QAPNYTQHT	26	34	9	HLA-A*02:01	26	42	58267.98503	42	58267.98503	42	TRUE	TRUE
1	TVLPPLLTD	841	849	9	HLA-A*02:01	841	42	58536.93783	42	58536.93783	42	TRUE	TRUE
1	SFGGVSVIT	577	585	9	HLA-A*02:01	577	42	58671.87941	42	58671.87941	42	TRUE	TRUE
1	VIIINNSTN	114	122	9	HLA-A*02:01	114	42	58942.6965	42	58942.6965	42	TRUE	TRUE
1	VYYPDEIFR	40	48	9	HLA-A*02:01	40	42	59078.57345	42	59078.57345	42	TRUE	TRUE
1	SNNTIAIPT	690	698	9	HLA-A*02:01	690	42	59488.08656	42	59488.08656	42	TRUE	TRUE
1	HADQLTPAW	611	619	9	HLA-A*02:01	611	42	59900.43827	42	59900.43827	42	TRUE	TRUE
1	MNNKSQSVI	107	115	9	HLA-A*02:01	107	42	60454.6902	42	60454.6902	42	TRUE	TRUE
1	SANLAATKM	1003	1011	9	HLA-A*02:01	1003	42	60733.7364	42	60733.7364	42	TRUE	TRUE
1	MGTQTHTMI	144	152	9	HLA-A*02:01	144	42	61014.07057	42	61014.07057	42	TRUE	TRUE
1	TGIGYQPYR	487	495	9	HLA-A*02:01	487	42	61436.9999	42	61436.9999	42	TRUE	TRUE
1	QKSIVAYTM	671	679	9	HLA-A*02:01	671	42	61862.86084	42	61862.86084	42	TRUE	TRUE
1	GTGVLTPSS	534	542	9	HLA-A*02:01	534	42	62291.6737	42	62291.6737	42	TRUE	TRUE
1	TDDMIAAYT	848	856	9	HLA-A*02:01	848	42	62435.27084	42	62435.27084	42	TRUE	TRUE
1	CLKGACSCG	1225	1233	9	HLA-A*02:01	1225	42	62723.45896	42	62723.45896	42	TRUE	TRUE
1	GTQTHTMIF	145	153	9	HLA-A*02:01	145	42	63012.97729	42	63012.97729	42	TRUE	TRUE
1	KYDENGTIT	265	273	9	HLA-A*02:01	265	42	63303.83198	42	63303.83198	42	TRUE	TRUE
1	VDTSYECDI	642	650	9	HLA-A*02:01	642	42	63449.76238	42	63449.76238	42	TRUE	TRUE
1	MSFPQAAPH	1032	1040	9	HLA-A*02:01	1032	43	64036.8558	43	64036.8558	43	TRUE	TRUE
1	STFKCYGVS	362	370	9	HLA-A*02:01	362	43	64184.476	43	64184.476	43	TRUE	TRUE
1	RDLPSGFNT	207	215	9	HLA-A*02:01	207	43	64629.38153	43	64629.38153	43	TRUE	TRUE
1	TTDNTFVSG	1098	1106	9	HLA-A*02:01	1098	43	64778.36765	43	64778.36765	43	TRUE	TRUE
1	KGYHLMSFP	1027	1035	9	HLA-A*02:01	1027	43	64927.69721	43	64927.69721	43	TRUE	TRUE
1	YVPSQERNF	1049	1057	9	HLA-A*02:01	1049	43	65077.37101	43	65077.37101	43	TRUE	TRUE
1	EVFAQVKQM	762	770	9	HLA-A*02:01	762	43	65227.38984	43	65227.38984	43	TRUE	TRUE
1	ISNCVADYS	345	353	9	HLA-A*02:01	345	43	65227.38984	43	65227.38984	43	TRUE	TRUE
1	KVTLADAGF	807	815	9	HLA-A*02:01	807	43	65377.7545	43	65377.7545	43	TRUE	TRUE
1	VLKGVKLHY	1246	1254	9	HLA-A*02:01	1246	43	66134.79322	43	66134.79322	43	TRUE	TRUE
1	YTQHTSSMR	30	38	9	HLA-A*02:01	30	43	66440.05756	43	66440.05756	43	TRUE	TRUE
1	FSPQIITTD	1092	1100	9	HLA-A*02:01	1092	43	66593.21771	43	66593.21771	43	TRUE	TRUE
1	TFDDVQAPN	21	29	9	HLA-A*02:01	21	43	67054.81984	43	67054.81984	43	TRUE	TRUE
1	TDVSTAIHA	604	612	9	HLA-A*02:01	604	43	67209.39716	43	67209.39716	43	TRUE	TRUE
1	NVVRGWVFG	96	104	9	HLA-A*02:01	96	43	67519.62165	43	67519.62165	43	TRUE	TRUE
1	TQTHTMIFD	146	154	9	HLA-A*02:01	146	43	67831.27806	43	67831.27806	43	TRUE	TRUE
1	LCFSNVYAD	377	385	9	HLA-A*02:01	377	43	68144.37301	43	68144.37301	43	TRUE	TRUE
1	LGDISGINA	1148	1156	9	HLA-A*02:01	1148	43	68301.46202	43	68301.46202	43	TRUE	TRUE
1	KFDEDDSEP	1237	1245	9	HLA-A*02:01	1237	43	68458.91315	43	68458.91315	43	TRUE	TRUE
1	TTEVMPVSM	705	713	9	HLA-A*02:01	705	43	68458.91315	43	68458.91315	43	TRUE	TRUE
1	NFKHLREFV	178	186	9	HLA-A*02:01	178	44	69092.35567	44	69092.35567	44	TRUE	TRUE
1	LTTTSTALG	920	928	9	HLA-A*02:01	920	44	69411.2715	44	69411.2715	44	TRUE	TRUE
1	IPFKDGIYF	81	89	9	HLA-A*02:01	81	44	70864.70768	44	70864.70768	44	TRUE	TRUE
1	DDFMGCVLA	414	422	9	HLA-A*02:01	414	44	71191.80431	44	71191.80431	44	TRUE	TRUE
1	IAAEQDRNT	752	760	9	HLA-A*02:01	752	44	71850.53397	44	71850.53397	44	TRUE	TRUE
1	DFCGKGYHL	1023	1031	9	HLA-A*02:01	1023	44	72016.1666	44	72016.1666	44	TRUE	TRUE
1	TKLNDLCFS	372	380	9	HLA-A*02:01	372	44	72016.16655	44	72016.16655	44	TRUE	TRUE
1	NQFNKAISQ	907	915	9	HLA-A*02:01	907	44	72682.52394	44	72682.52394	44	TRUE	TRUE
1	RALSGIAAE	747	755	9	HLA-A*02:01	747	44	73018.01123	44	73018.01123	44	TRUE	TRUE
1	HTFGNPVIP	74	82	9	HLA-A*02:01	74	44	73186.33513	44	73186.33513	44	TRUE	TRUE
1	IGAEHVDTS	637	645	9	HLA-A*02:01	637	44	74033.79295	44	74033.79295	44	TRUE	TRUE
1	TAPAICHEG	1059	1067	9	HLA-A*02:01	1059	45	75584.0223	45	75584.0223	45	TRUE	TRUE
1	VNFNFNGLT	525	533	9	HLA-A*02:01	525	45	75758.26151	45	75758.26151	45	TRUE	TRUE
1	QRNFFSPQI	1088	1096	9	HLA-A*02:01	1088	45	77522.89811	45	77522.89811	45	TRUE	TRUE
1	STMNNKSQS	105	113	9	HLA-A*02:01	105	45	77701.60685	45	77701.60685	45	TRUE	TRUE
1	NIDATSTGN	427	435	9	HLA-A*02:01	427	45	77880.72755	45	77880.72755	45	TRUE	TRUE
1	NFFSPQIIT	1090	1098	9	HLA-A*02:01	1090	45	78240.20866	45	78240.20866	45	TRUE	TRUE
1	GWTFGAGAA	867	875	9	HLA-A*02:01	867	45	78782.54388	45	78782.54388	45	TRUE	TRUE
1	NCVADYSVL	347	355	9	HLA-A*02:01	347	45	78782.54388	45	78782.54388	45	TRUE	TRUE
1	FRVVPSGDV	305	313	9	HLA-A*02:01	305	45	79694.80274	45	79694.80274	45	TRUE	TRUE
1	AQALNTLVK	938	946	9	HLA-A*02:01	938	45	80247.2207	45	80247.2207	45	TRUE	TRUE
1	FFAVSKPMG	137	145	9	HLA-A*02:01	137	45	80617.6251	45	80617.6251	45	TRUE	TRUE
1	SEILDISPC	568	576	9	HLA-A*02:01	568	45	80617.6251	45	80617.6251	45	TRUE	TRUE
1	TAIHADQLT	608	616	9	HLA-A*02:01	608	45	80617.62506	45	80617.62506	45	TRUE	TRUE
1	KNQCVNFNF	521	529	9	HLA-A*02:01	521	45	80617.62506	45	80617.62506	45	TRUE	TRUE
1	TGFHTINHT	67	75	9	HLA-A*02:01	67	46	81363.57076	46	81363.57076	46	TRUE	TRUE
1	LKYFGGFNF	776	784	9	HLA-A*02:01	776	46	81927.55609	46	81927.55609	46	TRUE	TRUE
1	HTSPDVDLG	1141	1149	9	HLA-A*02:01	1141	46	83067.28193	46	83067.28193	46	TRUE	TRUE
1	GTITDAVDC	270	278	9	HLA-A*02:01	270	46	83835.89402	46	83835.89402	46	TRUE	TRUE
1	SQNPLAELK	279	287	9	HLA-A*02:01	279	46	84029.15572	46	84029.15572	46	TRUE	TRUE
1	SVYAWERKK	336	344	9	HLA-A*02:01	336	46	84611.618	46	84611.618	46	TRUE	TRUE
1	STNVVIRAC	120	128	9	HLA-A*02:01	120	46	84806.66792	46	84806.66792	46	TRUE	TRUE
1	YRYLRHGKL	440	448	9	HLA-A*02:01	440	46	86582.47577	46	86582.47577	46	TRUE	TRUE

seq #	peptide	start	end	peptide length	allele	peptide index	median binding	smmpmbec IC5	smmpmbec pe	legacy IC50	legacy rank	scores equal	percentiles equal
1	ASSEVAVLY	590	598	9	HLA-A*02:01	590	46	86982.12231	46	86982.12231	46	TRUE	TRUE
1	HGVVFLHVT	1040	1048	9	HLA-A*02:01	1040	46	87786.95798	46	87786.95798	46	TRUE	TRUE
1	KPIFKLPLG	217	225	9	HLA-A*02:01	217	47	88803.48305	47	88803.48305	47	TRUE	TRUE
1	CYGVSATKL	366	374	9	HLA-A*02:01	366	47	89008.19622	47	89008.19622	47	TRUE	TRUE
1	NCDVVIGII	1107	1115	9	HLA-A*02:01	1107	47	89831.77892	47	89831.77892	47	TRUE	TRUE
1	NDYGFYTTT	479	487	9	HLA-A*02:01	479	47	90454.46306	47	90454.46306	47	TRUE	TRUE
1	CCMTSCCSC	1217	1225	9	HLA-A*02:01	1217	47	91081.46345	47	91081.46345	47	TRUE	TRUE
1	SDLDRCTTF	14	22	9	HLA-A*02:01	14	47	91924.2299	47	91924.2299	47	TRUE	TRUE
1	LIDLQELGK	1179	1187	9	HLA-A*02:01	1179	47	92348.53282	47	92348.53282	47	TRUE	TRUE
1	VVIRACNFE	123	131	9	HLA-A*02:01	123	47	92561.41817	47	92561.41817	47	TRUE	TRUE
1	DLICAQKFN	830	838	9	HLA-A*02:01	830	47	92988.66227	47	92988.66227	47	TRUE	TRUE
1	LIKNQCVNF	519	527	9	HLA-A*02:01	519	47	92988.66227	47	92988.66227	47	TRUE	TRUE
1	ESLTTTSTA	918	926	9	HLA-A*02:01	918	47	93203.02327	47	93203.02327	47	TRUE	TRUE
1	YKTPTLKYF	771	779	9	HLA-A*02:01	771	47	93203.02327	47	93203.02327	47	TRUE	TRUE
1	FNGLTVLPP	837	845	9	HLA-A*02:01	837	47	93849.07576	47	93849.07576	47	TRUE	TRUE
1	EIRASANLA	999	1007	9	HLA-A*02:01	999	48	94282.26342	48	94282.26342	48	TRUE	TRUE
1	VKQMYKTPT	767	775	9	HLA-A*02:01	767	48	95814.22696	48	95814.22696	48	TRUE	TRUE
1	ANLLLQYGS	732	740	9	HLA-A*02:01	732	48	96256.4853	48	96256.4853	48	TRUE	TRUE
1	GQTGVIADY	400	408	9	HLA-A*02:01	400	48	96256.4853	48	96256.4853	48	TRUE	TRUE
1	FGGVSVITP	578	586	9	HLA-A*02:01	578	48	97820.5274	48	97820.5274	48	TRUE	TRUE
1	TVCGPKLST	509	517	9	HLA-A*02:01	509	48	97820.5274	48	97820.5274	48	TRUE	TRUE
1	IYFAATEKS	87	95	9	HLA-A*02:01	87	48	98046.02701	48	98046.02701	48	TRUE	TRUE
1	CANLLLQYG	731	739	9	HLA-A*02:01	731	48	98725.6496	48	98725.6496	48	TRUE	TRUE
1	GDINARDLI	824	832	9	HLA-A*02:01	824	48	99409.98311	48	99409.98311	48	TRUE	TRUE
1	DLIKNQCVN	518	526	9	HLA-A*02:01	518	48	100099.0602	48	100099.0602	48	TRUE	TRUE
1	AEVQIDRLI	971	979	9	HLA-A*02:01	971	48	101025.2654	48	101025.2654	48	TRUE	TRUE
1	AKNLNESLI	1172	1180	9	HLA-A*02:01	1172	48	101025.2654	48	101025.2654	48	TRUE	TRUE
1	TSAAAYFVG	247	255	9	HLA-A*02:01	247	48	101025.2654	48	101025.2654	48	TRUE	TRUE
1	DEDDSEPVL	1239	1247	9	HLA-A*02:01	1239	48	101960.0407	48	101960.0407	48	TRUE	TRUE
1	LADAGFMKQ	810	818	9	HLA-A*02:01	810	49	102195.0828	49	102195.0828	49	TRUE	TRUE
1	VYADSFVVK	382	390	9	HLA-A*02:01	382	49	102666.7939	49	102666.7939	49	TRUE	TRUE
1	PIGAGICAS	651	659	9	HLA-A*02:01	651	49	102903.4653	49	102903.4653	49	TRUE	TRUE
1	LKPTKRSFI	792	800	9	HLA-A*02:01	792	49	103378.4461	49	103378.4461	49	TRUE	TRUE
1	YSTGNNVFQ	622	630	9	HLA-A*02:01	622	49	103378.4461	49	103378.4461	49	TRUE	TRUE
1	ITDAVDCSQ	272	280	9	HLA-A*02:01	272	49	103855.6193	49	103855.6193	49	TRUE	TRUE
1	GVTQNVLYE	892	900	9	HLA-A*02:01	892	49	104095.0313	49	104095.0313	49	TRUE	TRUE
1	SQILPDPLK	785	793	9	HLA-A*02:01	785	49	104334.9951	49	104334.9951	49	TRUE	TRUE
1	RFPNITNLC	315	323	9	HLA-A*02:01	315	49	104575.5121	49	104575.5121	49	TRUE	TRUE
1	MAYRFNGIG	884	892	9	HLA-A*02:01	884	49	105058.2107	49	105058.2107	49	TRUE	TRUE
1	GFMKQYGEC	814	822	9	HLA-A*02:01	814	49	105543.1374	49	105543.1374	49	TRUE	TRUE
1	NTLVKQLSS	942	950	9	HLA-A*02:01	942	49	108250.5222	49	108250.5222	49	TRUE	TRUE
1	FNKVTLADA	805	813	9	HLA-A*02:01	805	49	108750.1839	49	108750.1839	49	TRUE	TRUE
1	FTTAPAICH	1057	1065	9	HLA-A*02:01	1057	49	109000.879	49	109000.879	49	TRUE	TRUE
1	QHTSSMRGV	32	40	9	HLA-A*02:01	32	49	109504.0042	49	109504.0042	49	TRUE	TRUE
1	KSQSVIIIN	110	118	9	HLA-A*02:01	110	49	110263.0496	49	110263.0496	49	TRUE	TRUE
1	FKNKDGFLY	187	195	9	HLA-A*02:01	187	49	110772.0008	49	110772.0008	49	TRUE	TRUE
1	GGVSVITPG	579	587	9	HLA-A*02:01	579	50	111796.9615	50	111796.9615	50	TRUE	TRUE
1	SNFRVVPSG	303	311	9	HLA-A*02:01	303	50	113091.5093	50	113091.5093	50	TRUE	TRUE
1	PFFAVSKPM	136	144	9	HLA-A*02:01	136	50	113091.5093	50	113091.5093	50	TRUE	TRUE
1	TFGNPVIPF	75	83	9	HLA-A*02:01	75	50	113613.516	50	113613.516	50	TRUE	TRUE
1	CAQKFNGLT	833	841	9	HLA-A*02:01	833	50	114401.0472	50	114401.0472	50	TRUE	TRUE
1	FYSNVTGFH	62	70	9	HLA-A*02:01	62	50	114664.7689	50	114664.7689	50	TRUE	TRUE
1	SSNFGAISS	949	957	9	HLA-A*02:01	949	50	115459.5871	50	115459.5871	50	TRUE	TRUE
1	GNNVFQTQA	625	633	9	HLA-A*02:01	625	50	116796.5461	50	116796.5461	50	TRUE	TRUE
1	GFHTINHTF	68	76	9	HLA-A*02:01	68	50	117335.6545	50	117335.6545	50	TRUE	TRUE
1	LGINITNFR	224	232	9	HLA-A*02:01	224	50	117335.6545	50	117335.6545	50	TRUE	TRUE
1	ATVCGPKLS	508	516	9	HLA-A*02:01	508	50	117335.6545	50	117335.6545	50	TRUE	TRUE
1	VVPSGDVVR	307	315	9	HLA-A*02:01	307	50	117606.1411	50	117606.1411	50	TRUE	TRUE
1	QLTPAWRIY	614	622	9	HLA-A*02:01	614	50	118148.9864	50	118148.9864	50	TRUE	TRUE
1	YPDEIFRSD	42	50	9	HLA-A*02:01	42	51	119792.6025	51	119792.6025	51	TRUE	TRUE
1	RSFIEDLLF	797	805	9	HLA-A*02:01	797	51	120901.0298	51	120901.0298	51	TRUE	TRUE
1	LGADSSIAY	681	689	9	HLA-A*02:01	681	51	121179.7354	51	121179.7354	51	TRUE	TRUE
1	VFKNKDGFL	186	194	9	HLA-A*02:01	186	51	121459.0836	51	121459.0836	51	TRUE	TRUE
1	FGAISSVLN	952	960	9	HLA-A*02:01	952	51	121459.0836	51	121459.0836	51	TRUE	TRUE
1	VQAPNYTQH	25	33	9	HLA-A*02:01	25	51	122300.9977	51	122300.9977	51	TRUE	TRUE
1	YGSFCTQLN	738	746	9	HLA-A*02:01	738	51	122865.5135	51	122865.5135	51	TRUE	TRUE
1	GECLGDINA	820	828	9	HLA-A*02:01	820	51	123148.7477	51	123148.7477	51	TRUE	TRUE
1	CGPKLSTDL	511	519	9	HLA-A*02:01	511	51	123717.1765	51	123717.1765	51	TRUE	TRUE
1	GVYYPDEIF	39	47	9	HLA-A*02:01	39	51	125438.2539	51	125438.2539	51	TRUE	TRUE
1	GVVFLHVTY	1041	1049	9	HLA-A*02:01	1041	51	125727.4189	51	125727.4189	51	TRUE	TRUE
1	GAISSVLND	953	961	9	HLA-A*02:01	953	51	126307.7503	51	126307.7503	51	TRUE	TRUE
1	SPDVDLGDI	1143	1151	9	HLA-A*02:01	1143	51	126598.9198	51	126598.9198	51	TRUE	TRUE
1	RFQPFQQFG	544	552	9	HLA-A*02:01	544	51	128360.0862	51	128360.0862	51	TRUE	TRUE
1	KSIVAYTMS	672	680	9	HLA-A*02:01	672	51	128952.5694	51	128952.5694	51	TRUE	TRUE
1	LSRLDKVEA	963	971	9	HLA-A*02:01	963	52	130746.4784	52	130746.4784	52	TRUE	TRUE
1	NHTFGNPVI	73	81	9	HLA-A*02:01	73	52	133484.2421	52	133484.2421	52	TRUE	TRUE
1	PGTNASSEV	586	594	9	HLA-A*02:01	586	52	133791.9551	52	133791.9551	52	TRUE	TRUE
1	TATAGWTFG	863	871	9	HLA-A*02:01	863	52	134409.5107	52	134409.5107	52	TRUE	TRUE
1	PCSFGGVSV	575	583	9	HLA-A*02:01	575	52	136279.3332	52	136279.3332	52	TRUE	TRUE
1	DVNCTDVST	600	608	9	HLA-A*02:01	600	52	136593.4895	52	136593.4895	52	TRUE	TRUE
1	TQHTSSMRG	31	39	9	HLA-A*02:01	31	52	137540.3104	52	137540.3104	52	TRUE	TRUE
1	QQFGRDVSD	549	557	9	HLA-A*02:01	549	52	139453.6867	52	139453.6867	52	TRUE	TRUE
1	DLGDISGIN	1147	1155	9	HLA-A*02:01	1147	52	140420.3335	5.20E+01	1.40E+05	52	TRUE	TRUE
1	DSEPVLKGV	1242	1250	9	HLA-A*02:01	1242	52	141068.4843	5.20E+01	1.41E+05	52	TRUE	TRUE
1	LTAFSPAQD	235	243	9	HLA-A*02:01	235	52	141393.6807	5.20E+01	1.41E+05	52	TRUE	TRUE
1	ENGTITDAV	268	276	9	HLA-A*02:01	268	52	142046.3243	5.20E+01	1.42E+05	52	TRUE	TRUE
1	GKYEQYIKW	1186	1194	9	HLA-A*02:01	1186	52	142046.3243	5.20E+01	1.42E+05	52	TRUE	TRUE
1	FKHLREFVF	179	187	9	HLA-A*02:01	179	52	142701.9803	5.20E+01	1.43E+05	52	TRUE	TRUE
1	RDPKTSEIL	563	571	9	HLA-A*02:01	563	53	144022.3855	5.30E+01	1.44E+05	53	TRUE	TRUE
1	TEVMPVSMA	706	714	9	HLA-A*02:01	706	53	144354.3914	5.30E+01	1.44E+05	53	TRUE	TRUE
1	WFITQRNFF	1084	1092	9	HLA-A*02:01	1084	53	144687.1626	5.30E+01	1.45E+05	53	TRUE	TRUE
1	NYTQHTSSM	29	37	9	HLA-A*02:01	29	53	145020.701	5.30E+01	1.45E+05	53	TRUE	TRUE
1	EVAKNLNES	1170	1178	9	HLA-A*02:01	1170	53	145020.701	5.30E+01	1.45E+05	53	TRUE	TRUE
1	TTTGIGYQP	485	493	9	HLA-A*02:01	485	53	145690.0861	5.30E+01	1.46E+05	53	TRUE	TRUE
1	TSNFRVVPS	302	310	9	HLA-A*02:01	302	53	146362.561	5.30E+01	1.46E+05	53	TRUE	TRUE
1	AYTAALVSG	854	862	9	HLA-A*02:01	854	53	146362.561	5.30E+01	1.46E+05	53	TRUE	TRUE
1	PHGVVFLHV	1039	1047	9	HLA-A*02:01	1039	53	150117.0426	5.30E+01	1.50E+05	53	TRUE	TRUE
1	NKDGFLYVY	189	197	9	HLA-A*02:01	189	53	150117.0426	5.30E+01	1.50E+05	53	TRUE	TRUE
1	TTAPAICHE	1058	1066	9	HLA-A*02:01	1058	53	150463.0981	5.30E+01	1.50E+05	53	TRUE	TRUE
1	TKMSECVLG	1009	1017	9	HLA-A*02:01	1009	53	150463.0981	5.30E+01	1.50E+05	53	TRUE	TRUE
1	TLKYFGGFN	775	783	9	HLA-A*02:01	775	53	152907.9268	5.30E+01	1.53E+05	53	TRUE	TRUE
1	WGTSAAAYF	245	253	9	HLA-A*02:01	245	53	153613.7177	5.30E+01	1.54E+05	53	TRUE	TRUE

seq #	peptide	start	end	peptide length	allele	peptide index	median binding	smmpmbec IC5	smmpmbec pe	legacy IC50	legacy rank	scores equal	percentiles equal	
1	SATKLNDLC	370	378	9	HLA-A*02:01	370	53	155035.088	5.30E+01	1.55E+05	53	TRUE	TRUE	
1	YNYKLPDDF	408	416	9	HLA-A*02:01	408	53	155750.6974	5.30E+01	1.56E+05	53	TRUE	TRUE	
1	AKTSVDCNM	714	722	9	HLA-A*02:01	714	54	158281.4427	5.40E+01	1.58E+05	54	TRUE	TRUE	
1	VSMAKTSVD	711	719	9	HLA-A*02:01	711	54	158281.4427	5.40E+01	1.58E+05	54	TRUE	TRUE	
1	ICHEGKAYF	1063	1071	9	HLA-A*02:01	1063	54	159746.0028	5.40E+01	1.60E+05	54	TRUE	TRUE	
1	TKRSFIEDL	795	803	9	HLA-A*02:01	795	54	160114.2554	5.40E+01	1.60E+05	54	TRUE	TRUE	
1	FGSTMNNKS	103	111	9	HLA-A*02:01	103	54	161595.7743	5.40E+01	1.62E+05	54	TRUE	TRUE	
1	PFERDISNV	450	458	9	HLA-A*02:01	450	54	161968.2911	5.40E+01	1.62E+05	54	TRUE	TRUE	
1	RQIAPGQTG	395	403	9	HLA-A*02:01	395	54	163091.0016	5.40E+01	1.63E+05	54	TRUE	TRUE	
1	SCLKGACSC	1224	1232	9	HLA-A*02:01	1224	54	163091.0016	5.40E+01	1.63E+05	54	TRUE	TRUE	
1	ICASYHTVS	656	664	9	HLA-A*02:01	656	54	163091.0016	5.40E+01	1.63E+05	54	TRUE	TRUE	
1	GIAAEQDRN	751	759	9	HLA-A*02:01	751	54	164221.4945	5.40E+01	1.64E+05	54	TRUE	TRUE	
1	GDISGINAS	1149	1157	9	HLA-A*02:01	1149	54	166123.0897	5.40E+01	1.66E+05	54	TRUE	TRUE	
1	ELDKYFKNH	1133	1141	9	HLA-A*02:01	1133	54	167274.6	5.40E+01	1.67E+05	54	TRUE	TRUE	
1	REGVFVFNG	1073	1081	9	HLA-A*02:01	1073	55	168434.0921	5.50E+01	1.68E+05	55	TRUE	TRUE	
1	TGTGVLTPS	533	541	9	HLA-A*02:01	533	55	168822.3728	5.50E+01	1.69E+05	55	TRUE	TRUE	
1	PSQERNFTT	1051	1059	9	HLA-A*02:01	1051	55	169992.5936	5.50E+01	1.70E+05	55	TRUE	TRUE	
1	VTYVPSQER	1047	1055	9	HLA-A*02:01	1047	55	170384.467	5.50E+01	1.70E+05	55	TRUE	TRUE	
1	NQCVNFNFN	522	530	9	HLA-A*02:01	522	55	170777.2437	5.50E+01	1.71E+05	55	TRUE	TRUE	
1	LYQDVNCTD	597	605	9	HLA-A*02:01	597	55	171565.5157	5.50E+01	1.72E+05	55	TRUE	TRUE	
1	VYAWERKKI	337	345	9	HLA-A*02:01	337	55	171961.015	5.50E+01	1.72E+05	55	TRUE	TRUE	
1	PATVCGPKL	507	515	9	HLA-A*02:01	507	55	171961.015	5.50E+01	1.72E+05	55	TRUE	TRUE	
1	IGAGICASY	652	660	9	HLA-A*02:01	652	55	172754.751	5.50E+01	1.73E+05	55	TRUE	TRUE	
1	PQIITTDNT	1094	1102	9	HLA-A*02:01	1094	55	173152.9919	5.50E+01	1.73E+05	55	TRUE	TRUE	
1	GVLTPSSKR	536	544	9	HLA-A*02:01	536	55	173952.2298	5.50E+01	1.74E+05	55	TRUE	TRUE	
1	SEVAVLYQD	592	600	9	HLA-A*02:01	592	55	174353.2311	5.50E+01	1.74E+05	55	TRUE	TRUE	
1	LPLGINITN	222	230	9	HLA-A*02:01	222	55	175966.5016	5.50E+01	1.76E+05	55	TRUE	TRUE	
1	PTLKYFGGF	774	782	9	HLA-A*02:01	774	55	180480.3814	5.50E+01	1.80E+05	55	TRUE	TRUE	
1	NVVIRACNF	122	130	9	HLA-A*02:01	122	55	180896.4316	5.50E+01	1.81E+05	55	TRUE	TRUE	
1	QTHTMIFDN	147	155	9	HLA-A*02:01	147	56	183835.7622	5.60E+01	1.84E+05	56	TRUE	TRUE	
1	VSGTATAGW	860	868	9	HLA-A*02:01	860	56	183835.7622	5.60E+01	1.84E+05	56	TRUE	TRUE	
1	AELKCSVKS	284	292	9	HLA-A*02:01	284	56	187253.5242	5.60E+01	1.87E+05	56	TRUE	TRUE	
1	KTSVDCNMY	715	723	9	HLA-A*02:01	715	56	188117.8472	5.60E+01	1.88E+05	56	TRUE	TRUE	
1	LGDINARDL	823	831	9	HLA-A*02:01	823	56	188117.8472	5.60E+01	1.88E+05	56	TRUE	TRUE	
1	GSFCTQLNR	739	747	9	HLA-A*02:01	739	56	188986.1597	5.60E+01	1.89E+05	56	TRUE	TRUE	
1	KYFKNHTSP	1136	1144	9	HLA-A*02:01	1136	56	188986.1597	5.60E+01	1.89E+05	56	TRUE	TRUE	
1	ILSRLDKVE	962	970	9	HLA-A*02:01	962	56	189858.4802	5.60E+01	1.90E+05	56	TRUE	TRUE	
1	QTQAGCLIG	630	638	9	HLA-A*02:01	630	56	192056.9378	5.60E+01	1.92E+05	56	TRUE	TRUE	
1	IPFAMQMAY	878	886	9	HLA-A*02:01	878	56	192499.6747	5.60E+01	1.92E+05	56	TRUE	TRUE	
1	KCYGVSATK	365	373	9	HLA-A*02:01	365	56	192943.4323	5.60E+01	1.93E+05	56	TRUE	TRUE	
1	AYFVGYLKP	251	259	9	HLA-A*02:01	251	56	193388.2129	5.60E+01	1.93E+05	56	TRUE	TRUE	
1	GKGYHLMSF	1026	1034	9	HLA-A*02:01	1026	56	195177.612	5.60E+01	1.95E+05	56	TRUE	TRUE	
1	TINHTFGNP	71	79	9	HLA-A*02:01	71	56	195627.5428	5.60E+01	1.96E+05	56	TRUE	TRUE	
1	TQRNFFSPQ	1087	1095	9	HLA-A*02:01	1087	56	195627.5428	5.60E+01	1.96E+05	56	TRUE	TRUE	
1	DVVRDLPSG	204	212	9	HLA-A*02:01	204	57	200184.2937	5.70E+01	2.00E+05	57	TRUE	TRUE	
1	IYSTGNNVF	621	629	9	HLA-A*02:01	621	57	201571.9049	5.70E+01	2.02E+05	57	TRUE	TRUE	
1	VPSGDVVRF	308	316	9	HLA-A*02:01	308	57	202036.5761	5.70E+01	2.02E+05	57	TRUE	TRUE	
1	ISITTEVMP	702	710	9	HLA-A*02:01	702	57	202969.1346	5.70E+01	2.03E+05	57	TRUE	TRUE	
1	RNIDATSTG	426	434	9	HLA-A*02:01	426	57	203437.0268	5.70E+01	2.03E+05	57	TRUE	TRUE	
1	TSWFITQRN	1082	1090	9	HLA-A*02:01	1082	57	203905.9975	5.70E+01	2.04E+05	57	TRUE	TRUE	
1	DVVRFPNIT	312	320	9	HLA-A*02:01	312	57	203905.9975	5.70E+01	2.04E+05	57	TRUE	TRUE	
1	THTMIFDNA	148	156	9	HLA-A*02:01	148	57	204376.0494	5.70E+01	2.04E+05	57	TRUE	TRUE	
1	STDLIKNQC	516	524	9	HLA-A*02:01	516	57	205319.4064	5.70E+01	2.05E+05	57	TRUE	TRUE	
1	LVKQLSSNF	944	952	9	HLA-A*02:01	944	57	206742.6125	5.70E+01	2.07E+05	57	TRUE	TRUE	
1	EQYIKWPWY	1189	1197	9	HLA-A*02:01	1189	57	207219.2035	5.70E+01	2.07E+05	57	TRUE	TRUE	
1	LSSNFGAIS	948	956	9	HLA-A*02:01	948	57	207696.8931	5.70E+01	2.08E+05	57	TRUE	TRUE	
1	GFNFSQILP	781	789	9	HLA-A*02:01	781	57	208655.5784	5.70E+01	2.09E+05	57	TRUE	TRUE	
1	FYTTTGIGY	483	491	9	HLA-A*02:01	483	57	208655.5784	5.70E+01	2.09E+05	57	TRUE	TRUE	
1	NTVYDPLQP	1117	1125	9	HLA-A*02:01	1117	57	211558.2666	5.70E+01	2.12E+05	57	TRUE	TRUE	
1	FKEELDKYF	1130	1138	9	HLA-A*02:01	1130	58	216486.0917	5.80E+01	2.16E+05	58	TRUE	TRUE	
1	LPFYSNVTG	60	68	9	HLA-A*02:01	60	58	219497.7132	5.80E+01	2.19E+05	58	TRUE	TRUE	
1	QAAPHGVVF	1036	1044	9	HLA-A*02:01	1036	58	219497.7132	5.80E+01	2.19E+05	58	TRUE	TRUE	
1	ISQIQESLT	913	921	9	HLA-A*02:01	913	58	220510.8686	5.80E+01	2.21E+05	58	TRUE	TRUE	
1	NAPATVCGP	505	513	9	HLA-A*02:01	505	58	221019.1987	5.80E+01	2.21E+05	58	TRUE	TRUE	
1	QPYRVVVLS	492	500	9	HLA-A*02:01	492	58	221528.7005	5.80E+01	2.22E+05	58	TRUE	TRUE	
1	DGKPCTPPA	463	471	9	HLA-A*02:01	463	58	223578.4804	5.80E+01	2.24E+05	58	TRUE	TRUE	
1	VGYLKPTTF	254	262	9	HLA-A*02:01	254	58	223578.4804	5.80E+01	2.24E+05	58	TRUE	TRUE	
1	LKGACSCGS	1226	1234	9	HLA-A*02:01	1226	58	224093.882	5.80E+01	2.24E+05	58	TRUE	TRUE	
1	GACSCGSCC	1228	1236	9	HLA-A*02:01	1228	58	224610.4718	5.80E+01	2.25E+05	58	TRUE	TRUE	
1	SGSDLDRCT	12	20	9	HLA-A*02:01	12	58	224610.4718	5.80E+01	2.25E+05	58	TRUE	TRUE	
1	KGIYQTSNF	297	305	9	HLA-A*02:01	297	58	225128.2524	5.80E+01	2.25E+05	58	TRUE	TRUE	
1	PFGEVFNAT	324	332	9	HLA-A*02:01	324	58	226167.3972	5.80E+01	2.26E+05	58	TRUE	TRUE	
1	ADSSIAYSN	683	691	9	HLA-A*02:01	683	58	229313.6991	5.80E+01	2.29E+05	58	TRUE	TRUE	
1	LFNKVTLAD	804	812	9	HLA-A*02:01	804	58	230903.2257	5.80E+01	2.31E+05	58	TRUE	TRUE	
1	LQELGKYEQ	1182	1190	9	HLA-A*02:01	1182	58	231969.0266	5.80E+01	2.32E+05	58	TRUE	TRUE	
1	APHGVVFLH	1038	1046	9	HLA-A*02:01	1038	59	232503.7704	5.90E+01	2.33E+05	59	TRUE	TRUE	
1	GNFKHLREF	177	185	9	HLA-A*02:01	177	59	234655.1013	5.90E+01	2.35E+05	59	TRUE	TRUE	
1	SSVLNDILS	956	964	9	HLA-A*02:01	956	59	234655.1013	5.90E+01	2.35E+05	59	TRUE	TRUE	
1	VTGFHTINH	66	74	9	HLA-A*02:01	66	59	239017.6653	5.90E+01	2.39E+05	59	TRUE	TRUE	
1	DGFLYVYKG	191	199	9	HLA-A*02:01	191	59	241229.2686	5.90E+01	2.41E+05	59	TRUE	TRUE	
1	AFSLDVSEK	167	175	9	HLA-A*02:01	167	59	242901.39	5.90E+01	2.43E+05	59	TRUE	TRUE	
1	ASANLAATK	1002	1010	9	HLA-A*02:01	1002	59	244585.102	5.90E+01	2.45E+05	59	TRUE	TRUE	
1	FNTLKPIFK	213	221	9	HLA-A*02:01	213	59	246848.2201	5.90E+01	2.47E+05	59	TRUE	TRUE	
1	YHTVSLLRS	660	668	9	HLA-A*02:01	660	59	250859.181	5.90E+01	2.51E+05	59	TRUE	TRUE	
1	LHVTYVPSQ	1045	1053	9	HLA-A*02:01	1045	59	252598.0537	5.90E+01	2.53E+05	59	TRUE	TRUE	
1	GSDLDRCTT	13	21	9	HLA-A*02:01	13	60	253180.3524	6.00E+01	2.53E+05	60	TRUE	TRUE	
1	LRSTSQKSI	666	674	9	HLA-A*02:01	666	60	254935.3148	6.00E+01	2.55E+05	60	TRUE	TRUE	
1	YFKNHTSPD	1137	1145	9	HLA-A*02:01	1137	60	255523.0013	6.00E+01	2.56E+05	60	TRUE	TRUE	
1	TVSLLRSTS	662	670	9	HLA-A*02:01	662	60	256112.0427	6.00E+01	2.56E+05	60	TRUE	TRUE	
1	DKVEAEVQI	967	975	9	HLA-A*02:01	967	60	256112.0427	6.00E+01	2.56E+05	60	TRUE	TRUE	
1	FERDISNVP	451	459	9	HLA-A*02:01	451	60	256702.4419	6.00E+01	2.57E+05	60	TRUE	TRUE	
1	ELKCSVKSF	285	293	9	HLA-A*02:01	285	60	259077.6804	6.00E+01	2.59E+05	60	TRUE	TRUE	
1	CTPPALNCY	467	475	9	HLA-A*02:01	467	60	259674.9161	6.00E+01	2.60E+05	60	TRUE	TRUE	
1	FPNITNLCP	316	324	9	HLA-A*02:01	316	60	262077.6585	6.00E+01	2.62E+05	60	TRUE	TRUE	
1	IRACNFELC	125	133	9	HLA-A*02:01	125	60	262077.6585	6.00E+01	2.62E+05	60	TRUE	TRUE	
1	IDRLITGRL	975	983	9	HLA-A*02:01	975	60	263894.2941	6.00E+01	2.64E+05	60	TRUE	TRUE	
1	NFRAILTAF	230	238	9	HLA-A*02:01	230	60	265112.3748	6.00E+01	2.65E+05	60	TRUE	TRUE	
1	KGACSCGSC	1227	1235	9	HLA-A*02:01	1227	60	265723.5219	6.00E+01	2.66E+05	60	TRUE	TRUE	
1	GYHLMSFPQ	1028	1036	9	HLA-A*02:01	1028	60	266950.046	6.00E+01	2.67E+05	60	TRUE	TRUE	
1	TPPALNCYW	468	476	9	HLA-A*02:01	468	60	268182.2314	6.00E+01	2.68E+05	60	TRUE	TRUE	
1	ADYNYKLPD	406	414	9	HLA-A*02:01	406	60	269420.1043	6.00E+01	2.69E+05	60	TRUE	TRUE	
1	SYHTVSLLR	659	667	9	HLA-A*02:01	659	60	272539.8421	6.00E+01	2.73E+05	60	TRUE	TRUE	

seq #	peptide	start	end	peptide length	allele	peptide index	median binding	smmpmbec IC5	smmpmbec pe	legacy IC50	legacy rank	scores equal	percentiles equal
1	NRALSGIAA	746	754	9	HLA-A*02:01	746	60	273797.8289	6.00E+01	2.74E+05	60	TRUE	TRUE
1	QCVNFNFNG	523	531	9	HLA-A*02:01	523	61	277606.7361	6.10E+01	2.78E+05	61	TRUE	TRUE
1	EVFNATKFP	327	335	9	HLA-A*02:01	327	61	280175.3996	6.10E+01	2.80E+05	61	TRUE	TRUE
1	DLQELGKYE	1181	1189	9	HLA-A*02:01	1181	61	282117.4827	6.10E+01	2.82E+05	61	TRUE	TRUE
1	MSECVLGQS	1011	1019	9	HLA-A*02:01	1011	61	283419.6778	6.10E+01	2.83E+05	61	TRUE	TRUE
1	PFYSNVTGF	61	69	9	HLA-A*02:01	61	61	285384.2492	6.10E+01	2.85E+05	61	TRUE	TRUE
1	KFPSVYAWE	333	341	9	HLA-A*02:01	333	61	287362.4383	6.10E+01	2.87E+05	61	TRUE	TRUE
1	KSGNFKHLR	175	183	9	HLA-A*02:01	175	61	287362.4383	6.10E+01	2.87E+05	61	TRUE	TRUE
1	PAICHEGKA	1061	1069	9	HLA-A*02:01	1061	61	288688.843	6.10E+01	2.89E+05	61	TRUE	TRUE
1	TFMLKYDEN	261	269	9	HLA-A*02:01	261	61	290689.9385	6.10E+01	2.91E+05	61	TRUE	TRUE
1	VFVFNGTSW	1076	1084	9	HLA-A*02:01	1076	61	292031.7023	6.10E+01	2.92E+05	61	TRUE	TRUE
1	VLGQSKRVD	1015	1023	9	HLA-A*02:01	1015	61	293379.6594	6.10E+01	2.93E+05	61	TRUE	TRUE
1	GNYNYKYRY	434	442	9	HLA-A*02:01	434	61	293379.6594	6.10E+01	2.93E+05	61	TRUE	TRUE
1	SWFITQRNF	1083	1091	9	HLA-A*02:01	1083	62	296776.8357	6.20E+01	2.97E+05	62	TRUE	TRUE
1	CNFELCDNP	128	136	9	HLA-A*02:01	128	62	297460.977	6.20E+01	2.97E+05	62	TRUE	TRUE
1	QALNTLVKQ	939	947	9	HLA-A*02:01	939	62	297460.977	6.20E+01	2.97E+05	62	TRUE	TRUE
1	DEIFRSDTL	44	52	9	HLA-A*02:01	44	62	299522.878	6.20E+01	3.00E+05	62	TRUE	TRUE
1	LNAPATVCG	504	512	9	HLA-A*02:01	504	62	300905.4128	6.20E+01	3.01E+05	62	TRUE	TRUE
1	CDNPFFAVS	133	141	9	HLA-A*02:01	133	62	305091.424	6.20E+01	3.05E+05	62	TRUE	TRUE
1	YFAATEKSN	88	96	9	HLA-A*02:01	88	62	306499.6621	6.20E+01	3.06E+05	62	TRUE	TRUE
1	DVSTAIHAD	605	613	9	HLA-A*02:01	605	62	308624.216	6.20E+01	3.09E+05	62	TRUE	TRUE
1	VEAEVQIDR	969	977	9	HLA-A*02:01	969	62	310048.761	6.20E+01	3.10E+05	62	TRUE	TRUE
1	VAVLYQDVN	594	602	9	HLA-A*02:01	594	62	310763.4971	6.20E+01	3.11E+05	62	TRUE	TRUE
1	RPFERDISN	449	457	9	HLA-A*02:01	449	62	311479.8809	6.20E+01	3.11E+05	62	TRUE	TRUE
1	SYECDIPIG	645	653	9	HLA-A*02:01	645	62	312917.6067	6.20E+01	3.13E+05	62	TRUE	TRUE
1	LDISPCSFG	571	579	9	HLA-A*02:01	571	62	314361.9688	6.20E+01	3.14E+05	62	TRUE	TRUE
1	FDDVQAPNY	22	30	9	HLA-A*02:01	22	62	315086.648	6.20E+01	3.15E+05	62	TRUE	TRUE
1	PTNFSISIT	697	705	9	HLA-A*02:01	697	62	315086.648	6.20E+01	3.15E+05	62	TRUE	TRUE
1	IKNQCVNFN	520	528	9	HLA-A*02:01	520	62	317270.7243	6.20E+01	3.17E+05	62	TRUE	TRUE
1	AFSPAQDIW	237	245	9	HLA-A*02:01	237	63	318002.1088	6.30E+01	3.18E+05	63	TRUE	TRUE
1	VRGWVFGST	98	106	9	HLA-A*02:01	98	63	318002.1088	6.30E+01	3.18E+05	63	TRUE	TRUE
1	LYVYKGYQP	194	202	9	HLA-A*02:01	194	63	318735.1794	6.30E+01	3.19E+05	63	TRUE	TRUE
1	TTFMLKYDE	260	268	9	HLA-A*02:01	260	63	320944.5461	6.30E+01	3.21E+05	63	TRUE	TRUE
1	SGNCDVVIG	1105	1113	9	HLA-A*02:01	1105	63	320944.5461	6.30E+01	3.21E+05	63	TRUE	TRUE
1	HVDTSYECD	641	649	9	HLA-A*02:01	641	63	321684.3997	6.30E+01	3.22E+05	63	TRUE	TRUE
1	GFNTLKPIF	212	220	9	HLA-A*02:01	212	63	323169.2274	6.30E+01	3.23E+05	63	TRUE	TRUE
1	RVDFCGKGY	1021	1029	9	HLA-A*02:01	1021	63	323914.2094	6.30E+01	3.24E+05	63	TRUE	TRUE
1	MYICGDSTE	722	730	9	HLA-A*02:01	722	63	325409.3295	6.30E+01	3.25E+05	63	TRUE	TRUE
1	NFSQILPDP	783	791	9	HLA-A*02:01	783	63	326159.4755	6.30E+01	3.26E+05	63	TRUE	TRUE
1	GAGAALQIP	871	879	9	HLA-A*02:01	871	63	327664.9592	6.30E+01	3.28E+05	63	TRUE	TRUE
1	SGDVVRFPN	310	318	9	HLA-A*02:01	310	63	332223.2328	6.30E+01	3.32E+05	63	TRUE	TRUE
1	SFPQAAPHG	1033	1041	9	HLA-A*02:01	1033	63	333756.7056	6.30E+01	3.34E+05	63	TRUE	TRUE
1	DVDLGDISG	1145	1153	9	HLA-A*02:01	1145	63	336844.9184	6.30E+01	3.37E+05	63	TRUE	TRUE
1	DVVIGIINN	1109	1117	9	HLA-A*02:01	1109	63	343898.277	6.30E+01	3.44E+05	63	TRUE	TRUE
1	IQKEIDRLN	1161	1169	9	HLA-A*02:01	1161	63	346282.066	6.30E+01	3.46E+05	63	TRUE	TRUE
1	LTSGSDLDR	10	18	9	HLA-A*02:01	10	63	347080.3286	6.30E+01	3.47E+05	63	TRUE	TRUE
1	YRVVVLSFE	494	502	9	HLA-A*02:01	494	63	347880.4314	6.30E+01	3.48E+05	63	TRUE	TRUE
1	APGQTGVIA	398	406	9	HLA-A*02:01	398	63	351099.3294	6.30E+01	3.51E+05	63	TRUE	TRUE
1	AGFMKQYGE	813	821	9	HLA-A*02:01	813	64	351908.697	6.40E+01	3.52E+05	64	TRUE	TRUE
1	KKISNCVAD	343	351	9	HLA-A*02:01	343	64	355164.8681	6.40E+01	3.55E+05	64	TRUE	TRUE
1	QSVIIINNS	112	120	9	HLA-A*02:01	112	64	358451.1681	6.40E+01	3.58E+05	64	TRUE	TRUE
1	NVTGFHTIN	65	73	9	HLA-A*02:01	65	64	361767.8759	6.40E+01	3.62E+05	64	TRUE	TRUE
1	RACNFELCD	126	134	9	HLA-A*02:01	126	64	364275.5311	6.40E+01	3.64E+05	64	TRUE	TRUE
1	CSCGSCCKF	1230	1238	9	HLA-A*02:01	1230	64	366800.5684	6.40E+01	3.67E+05	64	TRUE	TRUE
1	RSDTLYLTQ	48	56	9	HLA-A*02:01	48	64	376209.7095	6.40E+01	3.76E+05	64	TRUE	TRUE
1	IADYNYKLP	405	413	9	HLA-A*02:01	405	65	377946.2147	6.50E+01	3.78E+05	65	TRUE	TRUE
1	EAEVQIDRL	970	978	9	HLA-A*02:01	970	65	378817.4707	6.50E+01	3.79E+05	65	TRUE	TRUE
1	TSGSDLDRC	11	19	9	HLA-A*02:01	11	65	378817.4707	6.50E+01	3.79E+05	65	TRUE	TRUE
1	FNGIGVTQN	888	896	9	HLA-A*02:01	888	65	382322.6256	6.50E+01	3.82E+05	65	TRUE	TRUE
1	GPKLSTDLI	512	520	9	HLA-A*02:01	512	65	382322.6256	6.50E+01	3.82E+05	65	TRUE	TRUE
1	GVSATKLND	368	376	9	HLA-A*02:01	368	65	382322.6256	6.50E+01	3.82E+05	65	TRUE	TRUE
1	LPPLLTDDM	843	851	9	HLA-A*02:01	843	65	387641.2631	6.50E+01	3.88E+05	65	TRUE	TRUE
1	MKQYGECLG	816	824	9	HLA-A*02:01	816	65	388534.8685	6.50E+01	3.89E+05	65	TRUE	TRUE
1	EIFRSDTLY	45	53	9	HLA-A*02:01	45	65	394848.0522	6.50E+01	3.95E+05	65	TRUE	TRUE
1	LPSGFNTLK	209	217	9	HLA-A*02:01	209	65	398501.5363	6.50E+01	3.99E+05	65	TRUE	TRUE
1	YENQKQIAN	899	907	9	HLA-A*02:01	899	65	401263.817	6.50E+01	4.01E+05	65	TRUE	TRUE
1	CFSNVYADS	378	386	9	HLA-A*02:01	378	65	403115.9666	6.50E+01	4.03E+05	65	TRUE	TRUE
1	QTSNFRVVP	301	309	9	HLA-A*02:01	301	65	404976.6653	6.50E+01	4.05E+05	65	TRUE	TRUE
1	LAWNTRNID	421	429	9	HLA-A*02:01	421	65	405910.233	6.50E+01	4.06E+05	65	TRUE	TRUE
1	NARDLICAQ	827	835	9	HLA-A*02:01	827	65	405910.233	6.50E+01	4.06E+05	65	TRUE	TRUE
1	GAGICASYH	653	661	9	HLA-A*02:01	653	65	408723.8683	6.50E+01	4.09E+05	65	TRUE	TRUE
1	QESLTTTST	917	925	9	HLA-A*02:01	917	66	412505.7437	6.60E+01	4.13E+05	66	TRUE	TRUE
1	ERNFTTAPA	1054	1062	9	HLA-A*02:01	1054	66	416322.6123	6.60E+01	4.16E+05	66	TRUE	TRUE
1	GFYTTTGIG	482	490	9	HLA-A*02:01	482	66	416322.6123	6.60E+01	4.16E+05	66	TRUE	TRUE
1	SDAFSLDVS	165	173	9	HLA-A*02:01	165	66	427002.0893	6.60E+01	4.27E+05	66	TRUE	TRUE
1	IWGTSAAAY	244	252	9	HLA-A*02:01	244	66	428973.0414	6.60E+01	4.29E+05	66	TRUE	TRUE
1	QTGVIADYN	401	409	9	HLA-A*02:01	401	66	435943.2926	6.60E+01	4.36E+05	66	TRUE	TRUE
1	AYFPREGVF	1069	1077	9	HLA-A*02:01	1069	66	439977.0262	6.60E+01	4.40E+05	66	TRUE	TRUE
1	GWVFGSTMN	100	108	9	HLA-A*02:01	100	67	445071.72	6.70E+01	4.45E+05	67	TRUE	TRUE
1	WNTRNIDAT	423	431	9	HLA-A*02:01	423	67	446097.7163	6.70E+01	4.46E+05	67	TRUE	TRUE
1	KVEAEVQID	968	976	9	HLA-A*02:01	968	67	447126.0778	6.70E+01	4.47E+05	67	TRUE	TRUE
1	NSTFFSTFK	357	365	9	HLA-A*02:01	357	67	449189.918	6.70E+01	4.49E+05	67	TRUE	TRUE
1	SGTATAGWT	861	869	9	HLA-A*02:01	861	67	449189.918	6.70E+01	4.49E+05	67	TRUE	TRUE
1	TTTSTALGK	921	929	9	HLA-A*02:01	921	67	456488.667	6.70E+01	4.56E+05	67	TRUE	TRUE
1	TTGIGYQPY	486	494	9	HLA-A*02:01	486	67	459652.8953	6.70E+01	4.60E+05	67	TRUE	TRUE
1	LNRALSGIA	745	753	9	HLA-A*02:01	745	67	463906.0111	6.70E+01	4.64E+05	67	TRUE	TRUE
1	KDGFLYVYK	190	198	9	HLA-A*02:01	190	67	470359.5866	6.70E+01	4.70E+05	67	TRUE	TRUE
1	ITGRLQSLQ	979	987	9	HLA-A*02:01	979	67	472530.6678	6.70E+01	4.73E+05	67	TRUE	TRUE
1	VVRFPNITN	313	321	9	HLA-A*02:01	313	67	472530.6678	6.70E+01	4.73E+05	67	TRUE	TRUE
1	TAGWTFGAG	865	873	9	HLA-A*02:01	865	67	474711.7703	6.70E+01	4.75E+05	67	TRUE	TRUE
1	DINARDLIC	825	833	9	HLA-A*02:01	825	67	475806.0939	6.70E+01	4.76E+05	67	TRUE	TRUE
1	RDLICAQKF	829	837	9	HLA-A*02:01	829	67	478002.3151	6.70E+01	4.78E+05	67	TRUE	TRUE
1	KYEQYIKWP	1187	1195	9	HLA-A*02:01	1187	67	480208.6735	6.70E+01	4.80E+05	67	TRUE	TRUE
1	PFQQFGRDV	547	555	9	HLA-A*02:01	547	68	489136.419	6.80E+01	4.89E+05	68	TRUE	TRUE
1	DVSEKSGNF	171	179	9	HLA-A*02:01	171	68	491394.1704	6.80E+01	4.91E+05	68	TRUE	TRUE
1	RKKISNCVA	342	350	9	HLA-A*02:01	342	68	493662.3429	6.80E+01	4.94E+05	68	TRUE	TRUE
1	STALGKLQD	924	932	9	HLA-A*02:01	924	68	497084.2468	6.80E+01	4.97E+05	68	TRUE	TRUE
1	HEGKAYFPR	1065	1073	9	HLA-A*02:01	1065	68	498230.1443	6.80E+01	4.98E+05	68	TRUE	TRUE
1	LCCMTSCCS	1216	1224	9	HLA-A*02:01	1216	68	500529.8701	6.80E+01	5.01E+05	68	TRUE	TRUE
1	NVLYENQKQ	896	904	9	HLA-A*02:01	896	68	501683.7107	6.80E+01	5.02E+05	68	TRUE	TRUE
1	CTQLNRALS	742	750	9	HLA-A*02:01	742	68	505161.216	6.80E+01	5.05E+05	68	TRUE	TRUE
1	NFTTAPAIC	1056	1064	9	HLA-A*02:01	1056	68	511010.7072	6.80E+01	5.11E+05	68	TRUE	TRUE

seq #	peptide	start	end	peptide length	allele	peptide index	median binding	smmpmbec IC	smmpmbec pe	legacy IC50	legacy rank	scores equal	percentiles equal
1	LKPTTFMLK	257	265	9	HLA-A*02:01	257	68	513369.4255	6.80E+01	5.13E+05	68	TRUE	TRUE
1	NASVVNIQK	1155	1163	9	HLA-A*02:01	1155	68	513369.4255	6.80E+01	5.13E+05	68	TRUE	TRUE
1	NVPFSPDGK	457	465	9	HLA-A*02:01	457	68	516927.9323	6.80E+01	5.17E+05	68	TRUE	TRUE
1	KHLREFVFK	180	188	9	HLA-A*02:01	180	68	518119.5743	6.80E+01	5.18E+05	68	TRUE	TRUE
1	VSLLRSTSQ	663	671	9	HLA-A*02:01	663	68	518119.5743	6.80E+01	5.18E+05	68	TRUE	TRUE
1	FGEVFNATK	325	333	9	HLA-A*02:01	325	68	519313.9632	6.80E+01	5.19E+05	68	TRUE	TRUE
1	TSCCSCLKG	1220	1228	9	HLA-A*02:01	1220	69	521711.008	6.90E+01	5.22E+05	69	TRUE	TRUE
1	YGVSATKLN	367	375	9	HLA-A*02:01	367	69	524119.1162	6.90E+01	5.24E+05	69	TRUE	TRUE
1	TSPDVDLGD	1142	1150	9	HLA-A*02:01	1142	69	525327.3355	6.90E+01	5.25E+05	69	TRUE	TRUE
1	LYENQKQIA	898	906	9	HLA-A*02:01	898	69	528968.7307	6.90E+01	5.29E+05	69	TRUE	TRUE
1	FHTINHTFG	69	77	9	HLA-A*02:01	69	69	530188.1295	6.90E+01	5.30E+05	69	TRUE	TRUE
1	YDENGTITD	266	274	9	HLA-A*02:01	266	69	530188.1295	6.90E+01	5.30E+05	69	TRUE	TRUE
1	TSQKSIVAY	669	677	9	HLA-A*02:01	669	69	537563.781	6.90E+01	5.38E+05	69	TRUE	TRUE
1	CTDVSTAIH	603	611	9	HLA-A*02:01	603	69	538802.9936	6.90E+01	5.39E+05	69	TRUE	TRUE
1	EHVDTSYEC	640	648	9	HLA-A*02:01	640	69	543788.4763	6.90E+01	5.44E+05	69	TRUE	TRUE
1	NGTSWFITQ	1080	1088	9	HLA-A*02:01	1080	69	547557.838	6.90E+01	5.48E+05	69	TRUE	TRUE
1	GKAYFPREG	1067	1075	9	HLA-A*02:01	1067	69	550085.2501	6.90E+01	5.50E+05	69	TRUE	TRUE
1	PFAMQMAYR	879	887	9	HLA-A*02:01	879	69	557737.6982	6.90E+01	5.58E+05	69	TRUE	TRUE
1	PDDFMGCVL	413	421	9	HLA-A*02:01	413	69	559023.4164	6.90E+01	5.59E+05	69	TRUE	TRUE
1	SNVPFSPDG	456	464	9	HLA-A*02:01	456	70	573363.4438	7.00E+01	5.73E+05	70	TRUE	TRUE
1	NTRNIDATS	424	432	9	HLA-A*02:01	424	70	584023.0661	7.00E+01	5.84E+05	70	TRUE	TRUE
1	HVTYVPSQE	1046	1054	9	HLA-A*02:01	1046	70	585369.3783	7.00E+01	5.85E+05	70	TRUE	TRUE
1	AVSKPMGTQ	139	147	9	HLA-A*02:01	139	70	586718.7941	7.00E+01	5.87E+05	70	TRUE	TRUE
1	PAWRIYSTG	617	625	9	HLA-A*02:01	617	70	589426.965	7.00E+01	5.89E+05	70	TRUE	TRUE
1	TFFSTFKCY	359	367	9	HLA-A*02:01	359	70	592147.636	7.00E+01	5.92E+05	70	TRUE	TRUE
1	DENGTITDA	267	275	9	HLA-A*02:01	267	70	596252.2076	7.00E+01	5.96E+05	70	TRUE	TRUE
1	SGINASVVN	1152	1160	9	HLA-A*02:01	1152	70	599004.3827	7.00E+01	5.99E+05	70	TRUE	TRUE
1	APNYTQHTS	27	35	9	HLA-A*02:01	27	70	600385.2304	7.00E+01	6.00E+05	70	TRUE	TRUE
1	SPDGKPCTP	461	469	9	HLA-A*02:01	461	70	607337.364	7.00E+01	6.07E+05	70	TRUE	TRUE
1	SVKSFEIDK	289	297	9	HLA-A*02:01	289	71	611547.2252	7.10E+01	6.12E+05	71	TRUE	TRUE
1	VDCNMYICG	718	726	9	HLA-A*02:01	718	71	614369.999	7.10E+01	6.14E+05	71	TRUE	TRUE
1	NNKSQSVII	108	116	9	HLA-A*02:01	108	71	614369.999	7.10E+01	6.14E+05	71	TRUE	TRUE
1	SDFTDSVRD	556	564	9	HLA-A*02:01	556	71	615786.2681	7.10E+01	6.16E+05	71	TRUE	TRUE
1	NQKQIANQF	901	909	9	HLA-A*02:01	901	71	618628.6084	7.10E+01	6.19E+05	71	TRUE	TRUE
1	TQNVLYENQ	894	902	9	HLA-A*02:01	894	71	622916.737	7.10E+01	6.23E+05	71	TRUE	TRUE
1	IGVTQNVLY	891	899	9	HLA-A*02:01	891	71	624352.7084	7.10E+01	6.24E+05	71	TRUE	TRUE
1	VIPFKDGIY	80	88	9	HLA-A*02:01	80	71	625791.99	7.10E+01	6.26E+05	71	TRUE	TRUE
1	LNCYWPLND	472	480	9	HLA-A*02:01	472	71	627234.5896	7.10E+01	6.27E+05	71	TRUE	TRUE
1	EKSGNFKHL	174	182	9	HLA-A*02:01	174	71	627234.5896	7.10E+01	6.27E+05	71	TRUE	TRUE
1	GFLYVYKGY	192	200	9	HLA-A*02:01	192	71	627234.5896	7.10E+01	6.27E+05	71	TRUE	TRUE
1	WPLNDYGFY	476	484	9	HLA-A*02:01	476	71	635960.2919	7.10E+01	6.36E+05	71	TRUE	TRUE
1	KPTTFMLKY	258	266	9	HLA-A*02:01	258	71	638895.7512	7.10E+01	6.39E+05	71	TRUE	TRUE
1	LSTDLIKNQ	515	523	9	HLA-A*02:01	515	71	641844.76	7.10E+01	6.42E+05	71	TRUE	TRUE
1	DAVDCSQNP	274	282	9	HLA-A*02:01	274	71	644807.3808	7.10E+01	6.45E+05	71	TRUE	TRUE
1	DRCTTFDDV	17	25	9	HLA-A*02:01	17	71	649276.9721	7.10E+01	6.49E+05	71	TRUE	TRUE
1	GSCCKFDED	1233	1241	9	HLA-A*02:01	1233	71	655284.6579	7.10E+01	6.55E+05	71	TRUE	TRUE
1	PKTSEILDI	565	573	9	HLA-A*02:01	565	71	664400.5756	7.10E+01	6.64E+05	71	TRUE	TRUE
1	IDVVRDLPS	203	211	9	HLA-A*02:01	203	72	665932.1771	7.20E+01	6.66E+05	72	TRUE	TRUE
1	AWRIYSTGN	618	626	9	HLA-A*02:01	618	72	667467.3093	7.20E+01	6.67E+05	72	TRUE	TRUE
1	GYQPIDVVR	199	207	9	HLA-A*02:01	199	72	676752.7045	7.20E+01	6.77E+05	72	TRUE	TRUE
1	SSEVAVLYQ	591	599	9	HLA-A*02:01	591	72	695712.81	7.20E+01	6.96E+05	72	TRUE	TRUE
1	ATEKSNVVR	91	99	9	HLA-A*02:01	91	72	698924.0744	7.20E+01	6.99E+05	72	TRUE	TRUE
1	PNITNLCPF	317	325	9	HLA-A*02:01	317	72	700535.2608	7.20E+01	7.01E+05	72	TRUE	TRUE
1	EVQIDRLIT	972	980	9	HLA-A*02:01	972	72	710280.6771	7.20E+01	7.10E+05	72	TRUE	TRUE
1	TFGAGAALQ	869	877	9	HLA-A*02:01	869	72	711918.0431	7.20E+01	7.12E+05	72	TRUE	TRUE
1	ATKLNDLCF	371	379	9	HLA-A*02:01	371	72	716852.8232	7.20E+01	7.17E+05	72	TRUE	TRUE
1	MAKTSVDCN	713	721	9	HLA-A*02:01	713	72	725153.5877	7.20E+01	7.25E+05	72	TRUE	TRUE
1	ISNVPFSPD	455	463	9	HLA-A*02:01	455	72	725153.5877	7.20E+01	7.25E+05	72	TRUE	TRUE
1	KRVDFCGKG	1020	1028	9	HLA-A*02:01	1020	72	725153.5877	7.20E+01	7.25E+05	72	TRUE	TRUE
1	RFNGIGVTQ	887	895	9	HLA-A*02:01	887	73	730180.1123	7.30E+01	7.30E+05	73	TRUE	TRUE
1	RDVSDFTDS	553	561	9	HLA-A*02:01	553	73	733550.4705	7.30E+01	7.34E+05	73	TRUE	TRUE
1	CNMYICGDS	720	728	9	HLA-A*02:01	720	73	742044.5846	7.30E+01	7.42E+05	73	TRUE	TRUE
1	VSKPMGTQT	140	148	9	HLA-A*02:01	140	73	743755.174	7.30E+01	7.44E+05	73	TRUE	TRUE
1	VFGSTMNNK	102	110	9	HLA-A*02:01	102	73	748910.6386	7.30E+01	7.49E+05	73	TRUE	TRUE
1	INASVVNIQ	1154	1162	9	HLA-A*02:01	1154	73	752367.4531	7.30E+01	7.52E+05	73	TRUE	TRUE
1	NFNGLTGTG	528	536	9	HLA-A*02:01	528	73	752367.4531	7.30E+01	7.52E+05	73	TRUE	TRUE
1	TFEYISDAF	160	168	9	HLA-A*02:01	160	73	755840.223	7.30E+01	7.56E+05	73	TRUE	TRUE
1	ACSCGSCCK	1229	1237	9	HLA-A*02:01	1229	73	757582.615	7.30E+01	7.58E+05	73	TRUE	TRUE
1	IRAAEIRAS	995	1003	9	HLA-A*02:01	995	73	769892.3424	7.30E+01	7.70E+05	73	TRUE	TRUE
1	GKLQDVVNQ	928	936	9	HLA-A*02:01	928	73	771667.128	7.30E+01	7.72E+05	73	TRUE	TRUE
1	IGIINNTVY	1112	1120	9	HLA-A*02:01	1112	73	773446.004	7.30E+01	7.73E+05	73	TRUE	TRUE
1	SNVVRGWVF	95	103	9	HLA-A*02:01	95	73	777016.0685	7.30E+01	7.77E+05	73	TRUE	TRUE
1	APATVCGPK	506	514	9	HLA-A*02:01	506	74	780602.6117	7.40E+01	7.81E+05	74	TRUE	TRUE
1	KRFQPFQQF	543	551	9	HLA-A*02:01	543	74	806176.2506	7.40E+01	8.06E+05	74	TRUE	TRUE
1	DVQAPNYTQ	24	32	9	HLA-A*02:01	24	74	808034.6788	7.40E+01	8.08E+05	74	TRUE	TRUE
1	NPFFAVSKP	135	143	9	HLA-A*02:01	135	74	811764.397	7.40E+01	8.12E+05	74	TRUE	TRUE
1	ANLAATKMS	1004	1012	9	HLA-A*02:01	1004	74	815511.3316	7.40E+01	8.16E+05	74	TRUE	TRUE
1	TSSMRGVYY	34	42	9	HLA-A*02:01	34	74	817391.2794	7.40E+01	8.17E+05	74	TRUE	TRUE
1	NDLCFSNVY	375	383	9	HLA-A*02:01	375	74	826856.2243	7.40E+01	8.27E+05	74	TRUE	TRUE
1	SDTLYLTQD	49	57	9	HLA-A*02:01	49	74	836430.7682	7.40E+01	8.36E+05	74	TRUE	TRUE
1	LCPFGEVFN	322	330	9	HLA-A*02:01	322	74	842228.6293	7.40E+01	8.42E+05	74	TRUE	TRUE
1	PPLLTDDMI	844	852	9	HLA-A*02:01	844	74	844170.1668	7.40E+01	8.44E+05	74	TRUE	TRUE
1	VSDFTDSVR	555	563	9	HLA-A*02:01	555	75	850021.675	7.50E+01	8.50E+05	75	TRUE	TRUE
1	EGVFVFNGT	1074	1082	9	HLA-A*02:01	1074	75	855913.7436	7.50E+01	8.56E+05	75	TRUE	TRUE
1	NATKFPSVY	330	338	9	HLA-A*02:01	330	75	857886.8286	7.50E+01	8.58E+05	75	TRUE	TRUE
1	VPSQERNFT	1050	1058	9	HLA-A*02:01	1050	75	865824.7577	7.50E+01	8.66E+05	75	TRUE	TRUE
1	NFELCDNPF	129	137	9	HLA-A*02:01	129	75	869821.2231	7.50E+01	8.70E+05	75	TRUE	TRUE
1	LRHGKLRPF	443	451	9	HLA-A*02:01	443	75	885992.407	7.50E+01	8.86E+05	75	TRUE	TRUE
1	TRNIDATST	425	433	9	HLA-A*02:01	425	75	885992.407	7.50E+01	8.86E+05	75	TRUE	TRUE
1	MPVSMAKTS	709	717	9	HLA-A*02:01	709	75	896251.7156	7.50E+01	8.96E+05	75	TRUE	TRUE
1	VKQLSSNFG	945	953	9	HLA-A*02:01	945	75	900388.6256	7.50E+01	9.00E+05	75	TRUE	TRUE
1	AICHEGKAY	1062	1070	9	HLA-A*02:01	1062	75	900388.626	7.50E+01	9.00E+05	75	TRUE	TRUE
1	LPDPLKPTK	788	796	9	HLA-A*02:01	788	75	912914.279	7.50E+01	9.13E+05	75	TRUE	TRUE
1	SRLDKVEAE	964	972	9	HLA-A*02:01	964	76	932030.2327	7.60E+01	9.32E+05	76	TRUE	TRUE
1	NTFVSGNCD	1101	1109	9	HLA-A*02:01	1101	76	938490.7576	7.60E+01	9.38E+05	76	TRUE	TRUE
1	TQQLIRAAE	991	999	9	HLA-A*02:01	991	76	949357.9684	7.60E+01	9.49E+05	76	TRUE	TRUE
1	PSGDVVRFP	309	317	9	HLA-A*02:01	309	76	955938.6035	7.60E+01	9.56E+05	76	TRUE	TRUE
1	TSTGNYNYK	431	439	9	HLA-A*02:01	431	76	964783.7945	7.60E+01	9.65E+05	76	TRUE	TRUE
1	VFAQVKQMY	763	771	9	HLA-A*02:01	763	76	975955.4643	7.60E+01	9.76E+05	76	TRUE	TRUE
1	VVKGDDVRQ	388	396	9	HLA-A*02:01	388	76	978205.274	7.60E+01	9.78E+05	76	TRUE	TRUE
1	DYSVLYNST	351	359	9	HLA-A*02:01	351	76	991813.4649	7.60E+01	9.92E+05	76	TRUE	TRUE
1	PAQDIWGTS	240	248	9	HLA-A*02:01	240	76	1014915.768	7.60E+01	1.01E+06	76	TRUE	TRUE

seq #	peptide	start	end	peptide length	allele	peptide index	median binding	smmpmbec IC	smmpmbec pe	legacy IC50	legacy rank	scores equal	percentiles equal
1	KNLNESLID	1173	1181	9	HLA-A*02:01	1173	76	1017255.391	7.60E+01	1.02E+06	76	TRUE	TRUE
1	PIDVVRDLP	202	210	9	HLA-A*02:01	202	76	1024306.668	7.60E+01	1.02E+06	76	TRUE	TRUE
1	YDPLQPELD	1120	1128	9	HLA-A*02:01	1120	77	1038556.193	7.70E+01	1.04E+06	77	TRUE	TRUE
1	QSLQTYVTQ	984	992	9	HLA-A*02:01	984	77	1045755.121	7.70E+01	1.05E+06	77	TRUE	TRUE
1	NHTSPDVDL	1140	1148	9	HLA-A*02:01	1140	77	1055431.374	7.70E+01	1.06E+06	77	TRUE	TRUE
1	TECANLLLQ	729	737	9	HLA-A*02:01	729	77	1055431.374	7.70E+01	1.06E+06	77	TRUE	TRUE
1	LTPSSKRFQ	538	546	9	HLA-A*02:01	538	77	1057864.395	7.70E+01	1.06E+06	77	TRUE	TRUE
1	VYKGYQPID	196	204	9	HLA-A*02:01	196	77	1060303.024	7.70E+01	1.06E+06	77	TRUE	TRUE
1	TDNTFVSGN	1099	1107	9	HLA-A*02:01	1099	77	1067652.693	7.70E+01	1.07E+06	77	TRUE	TRUE
1	ADQLTPAWR	612	620	9	HLA-A*02:01	612	77	1110273.566	7.70E+01	1.11E+06	77	TRUE	TRUE
1	NFSISITTE	699	707	9	HLA-A*02:01	699	77	1110273.566	7.70E+01	1.11E+06	77	TRUE	TRUE
1	GEVFNATKF	326	334	9	HLA-A*02:01	326	78	1123129.928	7.80E+01	1.12E+06	78	TRUE	TRUE
1	QDVNCTDVS	599	607	9	HLA-A*02:01	599	78	1136135.16	7.80E+01	1.14E+06	78	TRUE	TRUE
1	TPTLKYFGG	773	781	9	HLA-A*02:01	773	78	1138754.22	7.80E+01	1.14E+06	78	TRUE	TRUE
1	EELDKYFKN	1132	1140	9	HLA-A*02:01	1132	78	1141379.321	7.80E+01	1.14E+06	78	TRUE	TRUE
1	DRNTREVFA	757	765	9	HLA-A*02:01	757	78	1149290.985	7.80E+01	1.15E+06	78	TRUE	TRUE
1	DYGFYTTTG	480	488	9	HLA-A*02:01	480	78	1154595.871	7.80E+01	1.15E+06	78	TRUE	TRUE
1	PLKPTKRSF	791	799	9	HLA-A*02:01	791	78	1176061.411	7.80E+01	1.18E+06	78	TRUE	TRUE
1	VKSFEIDKG	290	298	9	HLA-A*02:01	290	78	1181489.864	7.80E+01	1.18E+06	78	TRUE	TRUE
1	NFNFNGLTG	526	534	9	HLA-A*02:01	526	78	1195170.871	7.80E+01	1.20E+06	78	TRUE	TRUE
1	PVLKGVKLH	1245	1253	9	HLA-A*02:01	1245	78	1197926.025	7.80E+01	1.20E+06	78	TRUE	TRUE
1	DIPIGAGIC	649	657	9	HLA-A*02:01	649	78	1228655.135	7.80E+01	1.23E+06	78	TRUE	TRUE
1	ERKKISNCV	341	349	9	HLA-A*02:01	341	78	1228655.135	7.80E+01	1.23E+06	78	TRUE	TRUE
1	GDDVRQIAP	391	399	9	HLA-A*02:01	391	79	1242882.291	7.90E+01	1.24E+06	79	TRUE	TRUE
1	GAEHVDTSY	638	646	9	HLA-A*02:01	638	79	1260172.506	7.90E+01	1.26E+06	79	TRUE	TRUE
1	TDSVRDPKT	559	567	9	HLA-A*02:01	559	79	1268907.604	7.90E+01	1.27E+06	79	TRUE	TRUE
1	QAGCLIGAE	632	640	9	HLA-A*02:01	632	79	1286559.867	7.90E+01	1.29E+06	79	TRUE	TRUE
1	GNPVIPFKD	77	85	9	HLA-A*02:01	77	79	1292498.358	7.90E+01	1.29E+06	79	TRUE	TRUE
1	QFGRDVSDF	550	558	9	HLA-A*02:01	550	79	1304457.698	7.90E+01	1.30E+06	79	TRUE	TRUE
1	SSKRFQPFQ	541	549	9	HLA-A*02:01	541	79	1304457.698	7.90E+01	1.30E+06	79	TRUE	TRUE
1	DAFSLDVSE	166	174	9	HLA-A*02:01	166	79	1313499.767	7.90E+01	1.31E+06	79	TRUE	TRUE
1	TYVTQQLIR	988	996	9	HLA-A*02:01	988	79	1319562.606	7.90E+01	1.32E+06	79	TRUE	TRUE
1	VVNIQKEID	1158	1166	9	HLA-A*02:01	1158	79	1328709.377	7.90E+01	1.33E+06	79	TRUE	TRUE
1	VDFCGKGYH	1022	1030	9	HLA-A*02:01	1022	79	1350299.168	7.90E+01	1.35E+06	79	TRUE	TRUE
1	RNTREVFAQ	758	766	9	HLA-A*02:01	758	79	1369083.7	7.90E+01	1.37E+06	79	TRUE	TRUE
1	DDVQAPNYT	23	31	9	HLA-A*02:01	23	80	1372239.764	8.00E+01	1.37E+06	80	TRUE	TRUE
1	ADAGFMKQY	811	819	9	HLA-A*02:01	811	80	1375403.104	8.00E+01	1.38E+06	80	TRUE	TRUE
1	AYTMSLGAD	676	684	9	HLA-A*02:01	676	80	1436911.432	8.00E+01	1.44E+06	80	TRUE	TRUE
1	SVRDPKTSE	561	569	9	HLA-A*02:01	561	80	1443543.914	8.00E+01	1.44E+06	80	TRUE	TRUE
1	NPLAELKCS	281	289	9	HLA-A*02:01	281	80	1490836.442	8.00E+01	1.49E+06	80	TRUE	TRUE
1	EPVLKGVKL	1244	1252	9	HLA-A*02:01	1244	81	1504630.981	8.10E+01	1.50E+06	81	TRUE	TRUE
1	SFKEELDKY	1129	1137	9	HLA-A*02:01	1129	81	1511576.042	8.10E+01	1.51E+06	81	TRUE	TRUE
1	VCGPKLSTD	510	518	9	HLA-A*02:01	510	81	1511576.042	8.10E+01	1.51E+06	81	TRUE	TRUE
1	CHEGKAYFP	1064	1072	9	HLA-A*02:01	1064	81	1536137.177	8.10E+01	1.54E+06	81	TRUE	TRUE
1	CSCLKGACS	1223	1231	9	HLA-A*02:01	1223	81	1539678.339	8.10E+01	1.54E+06	81	TRUE	TRUE
1	PPALNCYWP	469	477	9	HLA-A*02:01	469	81	1561097.399	8.10E+01	1.56E+06	81	TRUE	TRUE
1	KPCTPPALN	465	473	9	HLA-A*02:01	465	81	1568303.097	8.10E+01	1.57E+06	81	TRUE	TRUE
1	TSEILDISP	567	575	9	HLA-A*02:01	567	81	1571918.409	8.10E+01	1.57E+06	81	TRUE	TRUE
1	VNQNAQALN	934	942	9	HLA-A*02:01	934	81	1579174.054	8.10E+01	1.58E+06	81	TRUE	TRUE
1	PSSKRFQPF	540	548	9	HLA-A*02:01	540	81	1615957.743	8.10E+01	1.62E+06	81	TRUE	TRUE
1	GRLQSLQTY	981	989	9	HLA-A*02:01	981	81	1623416.666	8.10E+01	1.62E+06	81	TRUE	TRUE
1	DKYFKNHTS	1135	1143	9	HLA-A*02:01	1135	81	1627159.027	8.10E+01	1.63E+06	81	TRUE	TRUE
1	ARDLICAQK	828	836	9	HLA-A*02:01	828	82	1699925.936	8.20E+01	1.70E+06	82	TRUE	TRUE
1	TGVIADYNY	402	410	9	HLA-A*02:01	402	82	1719610.15	8.20E+01	1.72E+06	82	TRUE	TRUE
1	NYKLPDDFM	409	417	9	HLA-A*02:01	409	82	1763721.463	8.20E+01	1.76E+06	82	TRUE	TRUE
1	SCGSCCKFD	1231	1239	9	HLA-A*02:01	1231	83	1804803.814	8.30E+01	1.80E+06	83	TRUE	TRUE
1	KEELDKYFK	1131	1139	9	HLA-A*02:01	1131	83	1813134.41	8.30E+01	1.81E+06	83	TRUE	TRUE
1	TPSSKRFQP	539	547	9	HLA-A*02:01	539	83	1821503.458	8.30E+01	1.82E+06	83	TRUE	TRUE
1	ECLGDINAR	821	829	9	HLA-A*02:01	821	83	1821503.458	8.30E+01	1.82E+06	83	TRUE	TRUE
1	LDKYFKNHT	1134	1142	9	HLA-A*02:01	1134	83	1834129.517	8.30E+01	1.83E+06	83	TRUE	TRUE
1	VSVITPGTN	581	589	9	HLA-A*02:01	581	83	1838357.622	8.30E+01	1.84E+06	83	TRUE	TRUE
1	CGKGYHLMS	1025	1033	9	HLA-A*02:01	1025	83	1872535.242	8.30E+01	1.87E+06	83	TRUE	TRUE
1	NNSTNVVIR	118	126	9	HLA-A*02:01	118	83	1911745.163	8.30E+01	1.91E+06	83	TRUE	TRUE
1	IDLQELGKY	1180	1188	9	HLA-A*02:01	1180	83	1924996.747	8.30E+01	1.92E+06	83	TRUE	TRUE
1	CKFDEDDSE	1236	1244	9	HLA-A*02:01	1236	83	1933882.129	8.30E+01	1.93E+06	83	TRUE	TRUE
1	WERKKISNC	340	348	9	HLA-A*02:01	340	83	1956275.428	8.30E+01	1.96E+06	83	TRUE	TRUE
1	NKVTLADAG	806	814	9	HLA-A*02:01	806	83	1965305.185	8.30E+01	1.97E+06	83	TRUE	TRUE
1	CGDSTECAN	725	733	9	HLA-A*02:01	725	84	1974376.622	8.40E+01	1.97E+06	84	TRUE	TRUE
1	CSVKSFEID	288	296	9	HLA-A*02:01	288	84	1978928.03	8.40E+01	1.98E+06	84	TRUE	TRUE
1	QSKRVDFCG	1018	1026	9	HLA-A*02:01	1018	84	2034370.268	8.40E+01	2.03E+06	84	TRUE	TRUE
1	GRDVSDFTD	552	560	9	HLA-A*02:01	552	84	2057927.165	8.40E+01	2.06E+06	84	TRUE	TRUE
1	ASVVNIQKE	1156	1164	9	HLA-A*02:01	1156	84	2072192.035	8.40E+01	2.07E+06	84	TRUE	TRUE
1	DCNMYICGD	719	727	9	HLA-A*02:01	719	84	2086555.784	8.40E+01	2.09E+06	84	TRUE	TRUE
1	IDATSTGNY	428	436	9	HLA-A*02:01	428	84	2091365.792	8.40E+01	2.09E+06	84	TRUE	TRUE
1	RYLRHGKLR	441	449	9	HLA-A*02:01	441	84	2110716.961	8.40E+01	2.11E+06	84	TRUE	TRUE
1	APAICHEGK	1060	1068	9	HLA-A*02:01	1060	84	2159881.875	8.40E+01	2.16E+06	84	TRUE	TRUE
1	KPTKRSFIE	793	801	9	HLA-A*02:01	793	84	2164860.917	8.40E+01	2.16E+06	84	TRUE	TRUE
1	TGNNVFQTQ	624	632	9	HLA-A*02:01	624	85	2215287.005	8.50E+01	2.22E+06	85	TRUE	TRUE
1	TYVPSQERN	1048	1056	9	HLA-A*02:01	1048	85	2220393.769	8.50E+01	2.22E+06	85	TRUE	TRUE
1	TGRLQSLQT	980	988	9	HLA-A*02:01	980	85	2220393.769	8.50E+01	2.22E+06	85	TRUE	TRUE
1	NNTIAIPTN	691	699	9	HLA-A*02:01	691	85	2230642.641	8.50E+01	2.23E+06	85	TRUE	TRUE
1	AEHVDTSYE	639	647	9	HLA-A*02:01	639	85	2240938.82	8.50E+01	2.24E+06	85	TRUE	TRUE
1	LAELKCSVK	283	291	9	HLA-A*02:01	283	85	2246104.718	8.50E+01	2.25E+06	85	TRUE	TRUE
1	VSTAIHADQ	606	614	9	HLA-A*02:01	606	85	2246104.718	8.50E+01	2.25E+06	85	TRUE	TRUE
1	VRQIAPGQT	394	402	9	HLA-A*02:01	394	85	2266887.669	8.50E+01	2.27E+06	85	TRUE	TRUE
1	LNDILSRLD	959	967	9	HLA-A*02:01	959	85	2277351.147	8.50E+01	2.28E+06	85	TRUE	TRUE
1	NCYWPLNDY	473	481	9	HLA-A*02:01	473	85	2282600.983	8.50E+01	2.28E+06	85	TRUE	TRUE
1	LNESLIDLQ	1175	1183	9	HLA-A*02:01	1175	85	2282600.983	8.50E+01	2.28E+06	85	TRUE	TRUE
1	SPCSFGGVS	574	582	9	HLA-A*02:01	574	85	2335769.59	8.50E+01	2.34E+06	85	TRUE	TRUE
1	YGECLGDIN	819	827	9	HLA-A*02:01	819	85	2341154.095	8.50E+01	2.34E+06	85	TRUE	TRUE
1	HTSSMRGVY	33	41	9	HLA-A*02:01	33	85	2362816.527	8.50E+01	2.36E+06	85	TRUE	TRUE
1	AGCLIGAEH	633	641	9	HLA-A*02:01	633	85	2390176.653	8.50E+01	2.39E+06	85	TRUE	TRUE
1	LNTLVKQLS	941	949	9	HLA-A*02:01	941	85	2395686.58	8.50E+01	2.40E+06	85	TRUE	TRUE
1	IDKGIYQTS	295	303	9	HLA-A*02:01	295	85	2401209.207	8.50E+01	2.40E+06	85	TRUE	TRUE
1	ACNFELCDN	127	135	9	HLA-A*02:01	127	85	2440225.718	8.50E+01	2.44E+06	85	TRUE	TRUE
1	CCSCLKGAC	1222	1230	9	HLA-A*02:01	1222	85	2440225.718	8.50E+01	2.44E+06	85	TRUE	TRUE
1	GCVLAWNTR	418	426	9	HLA-A*02:01	418	86	2457140.555	8.60E+01	2.46E+06	86	TRUE	TRUE
1	ECANLLLQY	730	738	9	HLA-A*02:01	730	86	2485592.902	8.60E+01	2.49E+06	86	TRUE	TRUE
1	MYKTPTLKY	770	778	9	HLA-A*02:01	770	86	2491322.785	8.60E+01	2.49E+06	86	TRUE	TRUE
1	LKCSVKSFE	286	294	9	HLA-A*02:01	286	86	2491322.785	8.60E+01	2.49E+06	86	TRUE	TRUE
1	LDRCTTFDD	16	24	9	HLA-A*02:01	16	86	2525980.537	8.60E+01	2.53E+06	86	TRUE	TRUE
1	SGIAAEQDR	750	758	9	HLA-A*02:01	750	86	2531803.524	8.60E+01	2.53E+06	86	TRUE	TRUE

seq #	peptide	start	end	peptide length	allele	peptide index	median binding	smmpmbec IC	smmpmbec pe	legacy IC50	legacy rank	scores equal	percentiles equal	
1	CGSCCKFDE	1232	1240	9	HLA-A*02:01	1232	86	2543489.798	8.60E+01	2.54E+06	86	TRUE	TRUE	
1	AEQDRNTRE	754	762	9	HLA-A*02:01	754	86	2561120.427	8.60E+01	2.56E+06	86	TRUE	TRUE	
1	DTSYECDIP	643	651	9	HLA-A*02:01	643	86	2561120.427	8.60E+01	2.56E+06	86	TRUE	TRUE	
1	ADSFVVKGD	384	392	9	HLA-A*02:01	384	86	2572942.022	8.60E+01	2.57E+06	86	TRUE	TRUE	
1	VNIQKEIDR	1159	1167	9	HLA-A*02:01	1159	86	2584818.183	8.60E+01	2.58E+06	86	TRUE	TRUE	
1	HGKLRPFER	445	453	9	HLA-A*02:01	445	86	2596749.161	8.60E+01	2.60E+06	86	TRUE	TRUE	
1	PTTFMLKYD	259	267	9	HLA-A*02:01	259	86	2645026.332	8.60E+01	2.65E+06	86	TRUE	TRUE	
1	FNKAISQIQ	909	917	9	HLA-A*02:01	909	86	2651123.748	8.60E+01	2.65E+06	86	TRUE	TRUE	
1	QERNFTTAP	1053	1061	9	HLA-A*02:01	1053	86	2688004.55	8.60E+01	2.69E+06	86	TRUE	TRUE	
1	LSGIAAEQD	749	757	9	HLA-A*02:01	749	86	2750616.222	8.60E+01	2.75E+06	86	TRUE	TRUE	
1	REFVFKNKD	183	191	9	HLA-A*02:01	183	87	2756957.048	8.70E+01	2.76E+06	87	TRUE	TRUE	
1	KYRYLRHGK	439	447	9	HLA-A*02:01	439	87	2795310.141	8.70E+01	2.80E+06	87	TRUE	TRUE	
1	NNTVYDPLQ	1116	1124	9	HLA-A*02:01	1116	87	2827678.306	8.70E+01	2.83E+06	87	TRUE	TRUE	
1	LGQSKRVDF	1016	1024	9	HLA-A*02:01	1016	87	2906899.385	8.70E+01	2.91E+06	87	TRUE	TRUE	
1	PYRVVVLSF	493	501	9	HLA-A*02:01	493	87	2940559.693	8.70E+01	2.94E+06	87	TRUE	TRUE	
1	SKPMGTQTH	141	149	9	HLA-A*02:01	141	87	2967768.357	8.70E+01	2.97E+06	87	TRUE	TRUE	
1	MRGVYYPDE	37	45	9	HLA-A*02:01	37	87	2988339.94	8.70E+01	2.99E+06	87	TRUE	TRUE	
1	DVRQIAPGQ	393	401	9	HLA-A*02:01	393	87	3009054.128	8.70E+01	3.01E+06	87	TRUE	TRUE	
1	AAEIRASAN	997	1005	9	HLA-A*02:01	997	87	3036896.563	8.70E+01	3.04E+06	87	TRUE	TRUE	
1	EDLLFNKVT	801	809	9	HLA-A*02:01	801	87	3050914.24	8.70E+01	3.05E+06	87	TRUE	TRUE	
1	SEKSGNFKH	173	181	9	HLA-A*02:01	173	88	3180021.088	8.80E+01	3.18E+06	88	TRUE	TRUE	
1	TDAVDCSQN	273	281	9	HLA-A*02:01	273	88	3180021.088	8.80E+01	3.18E+06	88	TRUE	TRUE	
1	SKRFQPFQQ	542	550	9	HLA-A*02:01	542	88	3187351.794	8.80E+01	3.19E+06	88	TRUE	TRUE	
1	QELGKYEQY	1183	1191	9	HLA-A*02:01	1183	88	3187351.794	8.80E+01	3.19E+06	88	TRUE	TRUE	
1	ANQFNKAIS	906	914	9	HLA-A*02:01	906	88	3216843.997	8.80E+01	3.22E+06	88	TRUE	TRUE	
1	NNVFQTQAG	626	634	9	HLA-A*02:01	626	88	3239142.094	8.80E+01	3.24E+06	88	TRUE	TRUE	
1	WRIYSTGNN	619	627	9	HLA-A*02:01	619	88	3329890.865	8.80E+01	3.33E+06	88	TRUE	TRUE	
1	SPQIITTDN	1093	1101	9	HLA-A*02:01	1093	88	3376214.261	8.80E+01	3.38E+06	88	TRUE	TRUE	
1	ERDISNVPF	452	460	9	HLA-A*02:01	452	88	3383997.239	8.80E+01	3.38E+06	88	TRUE	TRUE	
1	DDVRQIAPG	392	400	9	HLA-A*02:01	392	88	3423182.08	8.80E+01	3.42E+06	88	TRUE	TRUE	
1	TGVLTPSSK	535	543	9	HLA-A*02:01	535	88	3470803.286	8.80E+01	3.47E+06	88	TRUE	TRUE	
1	REVFAQVKQ	761	769	9	HLA-A*02:01	761	88	3494861.745	8.80E+01	3.49E+06	88	TRUE	TRUE	
1	DAGFMKQYG	812	820	9	HLA-A*02:01	812	89	3551648.681	8.90E+01	3.55E+06	89	TRUE	TRUE	
1	QVKQMYKTP	766	774	9	HLA-A*02:01	766	89	3642755.311	8.90E+01	3.64E+06	89	TRUE	TRUE	
1	TGNYNYKYR	433	441	9	HLA-A*02:01	433	89	3642755.311	8.90E+01	3.64E+06	89	TRUE	TRUE	
1	LDSFKEELD	1127	1135	9	HLA-A*02:01	1127	89	3684936.429	8.90E+01	3.68E+06	89	TRUE	TRUE	
1	VPFSPDGKP	458	466	9	HLA-A*02:01	458	89	3710479.188	8.90E+01	3.71E+06	89	TRUE	TRUE	
1	EKSNVVRGW	93	101	9	HLA-A*02:01	93	89	3894305.339	8.90E+01	3.89E+06	89	TRUE	TRUE	
1	VSEKSGNFK	172	180	9	HLA-A*02:01	172	89	3921299.374	8.90E+01	3.92E+06	89	TRUE	TRUE	
1	LGKYEQYIK	1185	1193	9	HLA-A*02:01	1185	89	4077838.295	8.90E+01	4.08E+06	89	TRUE	TRUE	
1	SKRVDFCGK	1019	1027	9	HLA-A*02:01	1019	89	4077838.295	8.90E+01	4.08E+06	89	TRUE	TRUE	
1	TEKSNVVRG	92	100	9	HLA-A*02:01	92	90	4182442.702	9.00E+01	4.18E+06	90	TRUE	TRUE	
1	CDVVIGIIN	1108	1116	9	HLA-A*02:01	1108	90	4192084.228	9.00E+01	4.19E+06	90	TRUE	TRUE	
1	AAEQDRNTR	753	761	9	HLA-A*02:01	753	90	4250401.93	9.00E+01	4.25E+06	90	TRUE	TRUE	
1	QKQIANQFN	902	910	9	HLA-A*02:01	902	90	4379555.154	9.00E+01	4.38E+06	90	TRUE	TRUE	
1	PFSPDGKPC	459	467	9	HLA-A*02:01	459	90	4389651.071	9.00E+01	4.39E+06	90	TRUE	TRUE	
1	GKLRPFERD	446	454	9	HLA-A*02:01	446	90	4389651.071	9.00E+01	4.39E+06	90	TRUE	TRUE	
1	NCTFEYISD	158	166	9	HLA-A*02:01	158	90	4460977.163	9.00E+01	4.46E+06	90	TRUE	TRUE	
1	DDSEPVLKG	1241	1249	9	HLA-A*02:01	1241	90	4481568.098	9.00E+01	4.48E+06	90	TRUE	TRUE	
1	AWERKKISN	339	347	9	HLA-A*02:01	339	90	4523035.537	9.00E+01	4.52E+06	90	TRUE	TRUE	
1	DATSTGNYN	429	437	9	HLA-A*02:01	429	90	4649754.249	9.00E+01	4.65E+06	90	TRUE	TRUE	
1	DGIYFAATE	85	93	9	HLA-A*02:01	85	91	4902639.952	9.10E+01	4.90E+06	91	TRUE	TRUE	
1	PGQTGVIAD	399	407	9	HLA-A*02:01	399	91	5005298.701	9.10E+01	5.01E+06	91	TRUE	TRUE	
1	KNHTSPDVD	1139	1147	9	HLA-A*02:01	1139	91	5051612.16	9.10E+01	5.05E+06	91	TRUE	TRUE	
1	NFRVVPSGD	304	312	9	HLA-A*02:01	304	91	5157390.311	9.10E+01	5.16E+06	91	TRUE	TRUE	
1	NKAISQIQE	910	918	9	HLA-A*02:01	910	91	5181195.743	9.10E+01	5.18E+06	91	TRUE	TRUE	
1	DFTDSVRDP	557	565	9	HLA-A*02:01	557	91	5229136.757	9.10E+01	5.23E+06	91	TRUE	TRUE	
1	LDVSEKSGN	170	178	9	HLA-A*02:01	170	91	5289687.31	9.10E+01	5.29E+06	91	TRUE	TRUE	
1	RCTTFDDVQ	18	26	9	HLA-A*02:01	18	91	5326353.67	9.10E+01	5.33E+06	91	TRUE	TRUE	
1	SECVLGQSK	1012	1020	9	HLA-A*02:01	1012	91	5475578.38	9.10E+01	5.48E+06	91	TRUE	TRUE	
1	QNPLAELKC	280	288	9	HLA-A*02:01	280	92	5513533.276	9.20E+01	5.51E+06	92	TRUE	TRUE	
1	PNYTQHTSS	28	36	9	HLA-A*02:01	28	92	5707290.781	9.20E+01	5.71E+06	92	TRUE	TRUE	
1	QPFQQFGRD	546	554	9	HLA-A*02:01	546	92	5786687.105	9.20E+01	5.79E+06	92	TRUE	TRUE	
1	PSGFNTLKP	210	218	9	HLA-A*02:01	210	92	6017692.613	9.20E+01	6.02E+06	92	TRUE	TRUE	
1	PTKRSFIED	794	802	9	HLA-A*02:01	794	92	6059405.266	9.20E+01	6.06E+06	92	TRUE	TRUE	
1	GSTMNNKSQ	104	112	9	HLA-A*02:01	104	92	6129569.872	9.20E+01	6.13E+06	92	TRUE	TRUE	
1	QNVLYENQK	895	903	9	HLA-A*02:01	895	92	6186286.084	9.20E+01	6.19E+06	92	TRUE	TRUE	
1	NEVAKNLNE	1169	1177	9	HLA-A*02:01	1169	93	6374263.318	9.30E+01	6.37E+06	93	TRUE	TRUE	
1	RHGKLRPFE	444	452	9	HLA-A*02:01	444	93	6522738.984	9.30E+01	6.52E+06	93	TRUE	TRUE	
1	VRDLPSGFN	206	214	9	HLA-A*02:01	206	93	6628724.966	9.30E+01	6.63E+06	93	TRUE	TRUE	
1	SGNFKHLRE	176	184	9	HLA-A*02:01	176	93	7234857.808	9.30E+01	7.23E+06	93	TRUE	TRUE	
1	DYNYKLPDD	407	415	9	HLA-A*02:01	407	93	7369363.857	9.30E+01	7.37E+06	93	TRUE	TRUE	
1	EDDSEPVLK	1240	1248	9	HLA-A*02:01	1240	94	7454697.068	9.40E+01	7.45E+06	94	TRUE	TRUE	
1	TSTALGKLQ	923	931	9	HLA-A*02:01	923	94	7523674.531	9.40E+01	7.52E+06	94	TRUE	TRUE	
1	SCCKFDEDD	1234	1242	9	HLA-A*02:01	1234	94	7824020.866	9.40E+01	7.82E+06	94	TRUE	TRUE	
1	DKGIYQTSN	296	304	9	HLA-A*02:01	296	94	8024722.072	9.40E+01	8.02E+06	94	TRUE	TRUE	
1	TNVVIRACN	121	129	9	HLA-A*02:01	121	94	8061762.506	9.40E+01	8.06E+06	94	TRUE	TRUE	
1	PDVDLGDIS	1144	1152	9	HLA-A*02:01	1144	94	8306728.193	9.40E+01	8.31E+06	94	TRUE	TRUE	
1	PELDSFKEE	1125	1133	9	HLA-A*02:01	1125	94	8618466.542	9.40E+01	8.62E+06	94	TRUE	TRUE	
1	DNAFNCTFE	154	162	9	HLA-A*02:01	154	94	8678206.899	9.40E+01	8.68E+06	94	TRUE	TRUE	
1	NESLIDLQE	1176	1184	9	HLA-A*02:01	1176	94	8819216.42	9.40E+01	8.82E+06	94	TRUE	TRUE	
1	IFKLPLGIN	219	227	9	HLA-A*02:01	219	94	8900819.622	9.40E+01	8.90E+06	94	TRUE	TRUE	
1	DNPFFAVSK	134	142	9	HLA-A*02:01	134	95	9537400.057	9.50E+01	9.54E+06	95	TRUE	TRUE	
1	DSVRDPKTS	560	568	9	HLA-A*02:01	560	96	11000945.17	9.60E+01	1.10E+07	96	TRUE	TRUE	
1	DRLNEVAKN	1166	1174	9	HLA-A*02:01	1166	96	11128330.11	9.60E+01	1.11E+07	96	TRUE	TRUE	
1	DSFKEELDK	1128	1136	9	HLA-A*02:01	1128	96	11283140.6	9.60E+01	1.13E+07	96	TRUE	TRUE	
1	IDRLNEVAK	1165	1173	9	HLA-A*02:01	1165	96	11492909.85	9.60E+01	1.15E+07	96	TRUE	TRUE	
1	PKLSTDLIK	513	521	9	HLA-A*02:01	513	96	11706578.99	9.60E+01	1.17E+07	96	TRUE	TRUE	
1	QPELDSFKE	1124	1132	9	HLA-A*02:01	1124	96	11787725.12	9.60E+01	1.18E+07	96	TRUE	TRUE	
1	TPGTNASSE	585	593	9	HLA-A*02:01	585	96	12258293.06	9.60E+01	1.23E+07	96	TRUE	TRUE	
1	DSSIAYSNN	684	692	9	HLA-A*02:01	684	96	12601725.06	9.60E+01	1.26E+07	96	TRUE	TRUE	
1	NGTITDAVD	269	277	9	HLA-A*02:01	269	96	12865598.67	9.60E+01	1.29E+07	96	TRUE	TRUE	
1	PSVYAWERK	335	343	9	HLA-A*02:01	335	96	12984642.59	9.60E+01	1.30E+07	96	TRUE	TRUE	
1	LRPFERDIS	448	456	9	HLA-A*02:01	448	96	13195626.06	9.60E+01	1.32E+07	96	TRUE	TRUE	
1	QDVVNQNAQ	931	939	9	HLA-A*02:01	931	96	13471935.66	9.60E+01	1.35E+07	96	TRUE	TRUE	
1	PCTPPALNC	466	474	9	HLA-A*02:01	466	97	13945368.67	9.70E+01	1.39E+07	97	TRUE	TRUE	
1	EGKAYFPRE	1066	1074	9	HLA-A*02:01	1066	97	14636256.1	9.70E+01	1.46E+07	97	TRUE	TRUE	
1	PDGKPCTPP	462	470	9	HLA-A*02:01	462	97	14839866.7	9.70E+01	1.48E+07	97	TRUE	TRUE	
1	PREGVFVFN	1072	1080	9	HLA-A*02:01	1072	97	14839866.7	9.70E+01	1.48E+07	97	TRUE	TRUE	
1	LGKLQDVVN	927	935	9	HLA-A*02:01	927	97	15220537.9	97	15220537.9	97	TRUE	TRUE	
1	ECDIPIGAG	647	655	9	HLA-A*02:01	647	97	15974600.28	97	15974600.28	97	TRUE	TRUE	
1	SFEIDKGIY	292	300	9	HLA-A*02:01	292	97	16535982.35	97	16535982.35	97	TRUE	TRUE	

seq #	peptide	start	end	peptide length	allele	peptide index	median binding	smmpmbec IC5	smmpmbec pe	legacy IC50	legacy rank	scores equal	percentiles equal	
1	NDILSRLDK	960	968	9	HLA-A*02:01	960	97	16921154.86	97	16921154.86	97	TRUE	TRUE	
1	DNTFVSGNC	1100	1108	9	HLA-A*02:01	1100	97	17677872.61	97	17677872.61	97	TRUE	TRUE	
1	LREFVFKNK	182	190	9	HLA-A*02:01	182	98	19428085.23	98	19428085.23	98	TRUE	TRUE	
1	NYKYRYLRH	437	445	9	HLA-A*02:01	437	98	19562754.28	98	19562754.28	98	TRUE	TRUE	
1	SEPVLKGVK	1243	1251	9	HLA-A*02:01	1243	98	19607851.09	98	19607851.09	98	TRUE	TRUE	
1	LNEVAKNLN	1168	1176	9	HLA-A*02:01	1168	98	20437604.94	98	20437604.94	98	TRUE	TRUE	
1	DPLQPELDS	1121	1129	9	HLA-A*02:01	1121	98	22931369.91	98	22931369.91	98	TRUE	TRUE	
1	ENQKQIANQ	900	908	9	HLA-A*02:01	900	98	23143551.26	98	23143551.26	98	TRUE	TRUE	
1	ECVLGQSKR	1013	1021	9	HLA-A*02:01	1013	98	24178535.9	98	24178535.9	98	TRUE	TRUE	
1	DCSQNPLAE	277	285	9	HLA-A*02:01	277	98	24178535.9	98	24178535.9	98	TRUE	TRUE	
1	DPLKPTKRS	790	798	9	HLA-A*02:01	790	99	25967491.61	99	25967491.61	99	TRUE	TRUE	
1	CCKFDEDDS	1235	1243	9	HLA-A*02:01	1235	99	28211748.27	99	28211748.27	99	TRUE	TRUE	
1	EFVFKNKDG	184	192	9	HLA-A*02:01	184	99	28670152.3	99	28670152.3	99	TRUE	TRUE	
1	QDRNTREVF	756	764	9	HLA-A*02:01	756	99	31147988.09	99	31147988.09	99	TRUE	TRUE	
1	TREVFAQVK	760	768	9	HLA-A*02:01	760	99	31219791.6	99	31219791.6	99	TRUE	TRUE	
1	NPVIPFKDG	78	86	9	HLA-A*02:01	78	99	34231820.8	99	34231820.8	99	TRUE	TRUE	
1	DSFVVKGDD	385	393	9	HLA-A*02:01	385	99	36093583.29	99	36093583.29	99	TRUE	TRUE	
1	PDPLKPTKR	789	797	9	HLA-A*02:01	789	100	43194654.05	100	43194654.05	100	TRUE	TRUE	
1	LDKVEAEVQ	966	974	9	HLA-A*02:01	966	100	43294228.01	100	43294228.01	100	TRUE	TRUE	
1	PDEIFRSDT	43	51	9	HLA-A*02:01	43	100	60454690.2	100	60454690.2	100	TRUE	TRUE	
1	DPKTSEILD	564	572	9	HLA-A*02:01	564	100	70215016.14	100	70215016.14	100	TRUE	TRUE	
1	QKEIDRLNE	1162	1170	9	HLA-A*02:01	1162	100	87786958	100	87786958	100	TRUE	TRUE	
1	DRLITGRLQ	976	984	9	HLA-A*02:01	976	100	100561096.5	100	100561096.5	100	TRUE	TRUE	

seq #	peptide	start	end	peptide length	allele	peptide index	median binding	smm IC50	smm percentil	legacy IC50	legacy rank	scores equal	percentiles equal
1	FIAGLIAIV	1202	1210	9	HLA-A*02:01	1202	0.4	20.18877649	0.4	20.18877649	0.4	TRUE	TRUE
1	RLQSLQTYV	982	990	9	HLA-A*02:01	982	0.7	36.73753391	0.7	36.73753391	0.7	TRUE	TRUE
1	FIFLLFLTL	2	10	9	HLA-A*02:01	2	0.7	41.03080144	0.7	41.03080144	0.7	TRUE	TRUE
1	VIADYNYKL	404	412	9	HLA-A*02:01	404	0.9	51.6547186	0.9	51.6547186	0.9	TRUE	TRUE
1	RLDKVEAEV	965	973	9	HLA-A*02:01	965	0.9	53.2243051	0.9	53.2243051	0.9	TRUE	TRUE
1	YLKPTTFML	256	264	9	HLA-A*02:01	256	1	54.33879457	1	54.33879457	1	TRUE	TRUE
1	VLNDILSRL	958	966	9	HLA-A*02:01	958	1.1	58.35929005	1.1	58.35929005	1.1	TRUE	TRUE
1	LLFNKVTLA	803	811	9	HLA-A*02:01	803	1.2	63.69568317	1.2	63.69568317	1.2	TRUE	TRUE
1	VVFLHVTYV	1042	1050	9	HLA-A*02:01	1042	1.2	64.87987433	1.2	64.87987433	1.2	TRUE	TRUE
1	MIAAYTAAL	851	859	9	HLA-A*02:01	851	1.2	65.32960014	1.2	65.32960014	1.2	TRUE	TRUE
1	LLTDDMIAA	846	854	9	HLA-A*02:01	846	1.4	78.00276543	1.4	78.00276543	1.4	TRUE	TRUE
1	HLMSFPQAA	1030	1038	9	HLA-A*02:01	1030	1.6	101.6506125	1.6	101.6506125	1.6	TRUE	TRUE
1	FSISITTEV	700	708	9	HLA-A*02:01	700	1.8	111.9721455	1.8	111.9721455	1.8	TRUE	TRUE
1	NVYADSFVV	381	389	9	HLA-A*02:01	381	2.3	151.7434661	2.3	151.7434661	2.3	TRUE	TRUE
1	KQMYKTPTL	768	776	9	HLA-A*02:01	768	2.3	154.5645878	2.3	154.5645878	2.3	TRUE	TRUE
1	FLLFLTLTS	4	12	9	HLA-A*02:01	4	2.3	158.8949154	2.3	158.8949154	2.3	TRUE	TRUE
1	SIVAYTMSL	673	681	9	HLA-A*02:01	673	2.3	164.1005362	2.3	164.1005362	2.3	TRUE	TRUE
1	VLYNSTFFS	354	362	9	HLA-A*02:01	354	2.6	182.0161819	2.6	182.0161819	2.6	TRUE	TRUE
1	QYIKWPWYV	1190	1198	9	HLA-A*02:01	1190	2.7	191.0337056	2.7	191.0337056	2.7	TRUE	TRUE
1	ALVSGTATA	858	866	9	HLA-A*02:01	858	2.8	204.225515	2.8	204.225515	2.8	TRUE	TRUE
1	GLTVLPPLL	839	847	9	HLA-A*02:01	839	2.8	207.5439127	2.8	207.5439127	2.8	TRUE	TRUE
1	NLNESLIDL	1174	1182	9	HLA-A*02:01	1174	2.8	208.9825384	2.8	208.9825384	2.8	TRUE	TRUE
1	YVYKGYQPI	195	203	9	HLA-A*02:01	195	2.9	215.332707	2.9	215.332707	2.9	TRUE	TRUE
1	SITTEVMPV	703	711	9	HLA-A*02:01	703	2.9	217.8261422	2.9	217.8261422	2.9	TRUE	TRUE
1	FVSGNCDVV	1103	1111	9	HLA-A*02:01	1103	3	230.202481	3	230.202481	3	TRUE	TRUE
1	KQLSSNFGA	946	954	9	HLA-A*02:01	946	3.2	242.1642333	3.2	242.1642333	3.2	TRUE	TRUE
1	YLTQDLFLP	53	61	9	HLA-A*02:01	53	3.2	242.7224795	3.2	242.7224795	3.2	TRUE	TRUE
1	ATAGWTFGA	864	872	9	HLA-A*02:01	864	3.3	258.291432	3.3	258.291432	3.3	TRUE	TRUE
1	GLIAIVMVT	1205	1213	9	HLA-A*02:01	1205	3.3	260.681373	3.3	260.681373	3.3	TRUE	TRUE
1	FLPFYSNVT	59	67	9	HLA-A*02:01	59	3.3	261.8846237	3.3	261.8846237	3.3	TRUE	TRUE
1	ILLCCMTSC	1214	1222	9	HLA-A*02:01	1214	3.3	264.3078123	3.3	264.3078123	3.3	TRUE	TRUE
1	IIINNSTNV	115	123	9	HLA-A*02:01	115	3.4	267.9847002	3.4	267.9847002	3.4	TRUE	TRUE
1	IITTDNTFV	1096	1104	9	HLA-A*02:01	1096	3.4	267.9847002	3.4	267.9847002	3.4	TRUE	TRUE
1	GTSAAAYFV	246	254	9	HLA-A*02:01	246	3.4	269.8422814	3.4	269.8422814	3.4	TRUE	TRUE
1	ALGKLQDVV	926	934	9	HLA-A*02:01	926	3.6	291.1454451	3.6	291.1454451	3.6	TRUE	TRUE
1	AIVMVTILL	1208	1216	9	HLA-A*02:01	1208	3.6	291.8166046	3.6	291.8166046	3.6	TRUE	TRUE
1	ELLNAPATV	502	510	9	HLA-A*02:01	502	3.6	293.8393807	3.6	293.8393807	3.6	TRUE	TRUE
1	ELCDNPFFA	131	139	9	HLA-A*02:01	131	3.7	306.2739079	3.7	306.2739079	3.7	TRUE	TRUE
1	TMNNKSQSV	106	114	9	HLA-A*02:01	106	3.7	306.2739079	3.7	306.2739079	3.7	TRUE	TRUE
1	VLSFELLNA	498	506	9	HLA-A*02:01	498	3.7	314.854569	3.7	314.854569	3.7	TRUE	TRUE
1	YQDVNCTDV	598	606	9	HLA-A*02:01	598	3.8	322.1884741	3.8	322.1884741	3.8	TRUE	TRUE
1	CMTSCCSCL	1218	1226	9	HLA-A*02:01	1218	4	350.8407252	4	350.8407252	4	TRUE	TRUE
1	RVVVLSFEL	495	503	9	HLA-A*02:01	495	4	364.0072218	4	364.0072218	4	TRUE	TRUE
1	SLTTTSTAL	919	927	9	HLA-A*02:01	919	4	364.8463451	4	364.8463451	4	TRUE	TRUE
1	PLAELKCSV	282	290	9	HLA-A*02:01	282	4.1	370.7746213	4.1	370.7746213	4.1	TRUE	TRUE
1	VLAWNTRNI	420	428	9	HLA-A*02:01	420	4.1	380.2857045	4.1	380.2857045	4.1	TRUE	TRUE
1	VIRACNFEL	124	132	9	HLA-A*02:01	124	4.1	385.5760057	4.1	385.5760057	4.1	TRUE	TRUE
1	LLQYGSFCT	735	743	9	HLA-A*02:01	735	4.1	391.8411118	4.1	391.8411118	4.1	TRUE	TRUE
1	VLYENQKQI	897	905	9	HLA-A*02:01	897	4.2	399.1259819	4.2	399.1259819	4.2	TRUE	TRUE
1	FSTFKCYGV	361	369	9	HLA-A*02:01	361	4.2	407.4834739	4.2	407.4834739	4.2	TRUE	TRUE
1	LIAIVMVTI	1206	1214	9	HLA-A*02:01	1206	4.3	419.8653158	4.3	419.8653158	4.3	TRUE	TRUE
1	FMKQYGECL	815	823	9	HLA-A*02:01	815	4.3	422.7756832	4.3	422.7756832	4.3	TRUE	TRUE
1	KLPDDFMGC	411	419	9	HLA-A*02:01	411	4.4	439.6529586	4.4	439.6529586	4.4	TRUE	TRUE
1	KLNDLCFSN	373	381	9	HLA-A*02:01	373	4.5	450.9309038	4.5	450.9309038	4.5	TRUE	TRUE
1	AILTAFSPA	233	241	9	HLA-A*02:01	233	4.6	457.2039776	4.6	457.2039776	4.6	TRUE	TRUE
1	GIGYQPYRV	488	496	9	HLA-A*02:01	488	4.6	472.1826226	4.6	472.1826226	4.6	TRUE	TRUE
1	MIFDNAFNC	151	159	9	HLA-A*02:01	151	4.6	473.271116	4.6	473.271116	4.6	TRUE	TRUE
1	FMGCVLAWN	416	424	9	HLA-A*02:01	416	4.6	476.5516746	4.6	476.5516746	4.6	TRUE	TRUE
1	YQPYRVVVL	491	499	9	HLA-A*02:01	491	4.8	502.4698412	4.8	502.4698412	4.8	TRUE	TRUE
1	QIAPGQTGV	396	404	9	HLA-A*02:01	396	4.9	507.1191374	4.9	507.1191374	4.9	TRUE	TRUE
1	YQTSNFRVV	300	308	9	HLA-A*02:01	300	4.9	518.9314595	4.9	518.9314595	4.9	TRUE	TRUE
1	TQAGCLIGA	631	639	9	HLA-A*02:01	631	4.9	524.9404026	4.9	524.9404026	4.9	TRUE	TRUE
1	QMAYRFNGI	883	891	9	HLA-A*02:01	883	4.9	526.1505152	4.9	526.1505152	4.9	TRUE	TRUE
1	GICASYHTV	655	663	9	HLA-A*02:01	655	4.9	528.5791156	4.9	528.5791156	4.9	TRUE	TRUE
1	VWLGFIAGL	1198	1206	9	HLA-A*02:01	1198	4.9	529.7976163	4.9	529.7976163	4.9	TRUE	TRUE
1	RLNEVAKNL	1167	1175	9	HLA-A*02:01	1167	5.1	562.4837755	5.1	562.4837755	5.1	TRUE	TRUE
1	PLNDYGFYT	477	485	9	HLA-A*02:01	477	5.3	599.9430132	5.3	599.9430132	5.3	TRUE	TRUE
1	ALNTLVKQL	940	948	9	HLA-A*02:01	940	5.3	601.3260247	5.3	601.3260247	5.3	TRUE	TRUE
1	IAIPTNFSI	694	702	9	HLA-A*02:01	694	5.3	604.101619	5.3	604.101619	5.3	TRUE	TRUE
1	WLGFIAGLI	1199	1207	9	HLA-A*02:01	1199	5.3	605.4942175	5.3	605.4942175	5.3	TRUE	TRUE
1	VLYQDVNCT	596	604	9	HLA-A*02:01	596	6	698.4092778	6	698.4092778	6	TRUE	TRUE
1	FMLKYDENG	262	270	9	HLA-A*02:01	262	6.1	711.3936757	6.1	711.3936757	6.1	TRUE	TRUE
1	LLLQYGSFC	734	742	9	HLA-A*02:01	734	6.1	719.6312263	6.1	719.6312263	6.1	TRUE	TRUE
1	IINNSTNVV	116	124	9	HLA-A*02:01	116	6.1	724.6194716	6.1	724.6194716	6.1	TRUE	TRUE
1	IKWPWYVWL	1192	1200	9	HLA-A*02:01	1192	6.1	726.289892	6.1	726.289892	6.1	TRUE	TRUE
1	FNATKFPSV	329	337	9	HLA-A*02:01	329	6.5	783.6280984	6.5	783.6280984	6.5	TRUE	TRUE
1	SLGADSSIA	680	688	9	HLA-A*02:01	680	6.6	831.974471	6.6	831.974471	6.6	TRUE	TRUE
1	QLNRALSGI	744	752	9	HLA-A*02:01	744	6.7	837.7414421	6.7	837.7414421	6.7	TRUE	TRUE
1	YVTQQLIRA	989	997	9	HLA-A*02:01	989	6.7	837.7414421	6.7	837.7414421	6.7	TRUE	TRUE
1	MFIFLLFLT	1	9	9	HLA-A*02:01	1	6.8	865.1870294	6.8	865.1870294	6.8	TRUE	TRUE
1	VIGIINNTV	1111	1119	9	HLA-A*02:01	1111	6.9	877.2229799	6.9	877.2229799	6.9	TRUE	TRUE
1	YVWLGFIAG	1197	1205	9	HLA-A*02:01	1197	6.9	887.3807432	6.9	887.3807432	6.9	TRUE	TRUE
1	RIYSTGNNV	620	628	9	HLA-A*02:01	620	6.9	891.4767066	6.9	891.4767066	6.9	TRUE	TRUE
1	VMVTILLCC	1210	1218	9	HLA-A*02:01	1210	6.9	901.7995204	6.9	901.7995204	6.9	TRUE	TRUE
1	IAAYTAALV	852	860	9	HLA-A*02:01	852	7	914.3448014	7	914.3448014	7	TRUE	TRUE
1	CTFEYISDA	159	167	9	HLA-A*02:01	159	7.1	924.932415	7.1	924.932415	7.1	TRUE	TRUE
1	LFLPFYSNV	58	66	9	HLA-A*02:01	58	7.1	929.2017092	7.1	929.2017092	7.1	TRUE	TRUE
1	FLHVTYVPS	1044	1052	9	HLA-A*02:01	1044	7.3	979.7381063	7.3	979.7381063	7.3	TRUE	TRUE
1	QLSSNFGAI	947	955	9	HLA-A*02:01	947	7.4	1007.186741	7.4	1007.186741	7.4	TRUE	TRUE
1	FKDGIYFAA	83	91	9	HLA-A*02:01	83	7.4	1023.552209	7.4	1023.552209	7.4	TRUE	TRUE
1	AVDCSQNPL	275	283	9	HLA-A*02:01	275	7.6	1086.700833	7.6	1086.700833	7.6	TRUE	TRUE
1	LQIPFAMQM	876	884	9	HLA-A*02:01	876	7.7	1091.716816	7.7	1091.716816	7.7	TRUE	TRUE
1	LLFLTLTSG	5	13	9	HLA-A*02:01	5	7.7	1112.013347	7.7	1112.013347	7.7	TRUE	TRUE
1	MQMAYRFNG	882	890	9	HLA-A*02:01	882	7.8	1137.915466	7.8	1137.915466	7.8	TRUE	TRUE
1	FVFNGTSWF	1077	1085	9	HLA-A*02:01	1077	7.8	1151.091906	7.8	1151.091906	7.8	TRUE	TRUE
1	SLIDLQELG	1178	1186	9	HLA-A*02:01	1178	8	1183.34124	8	1183.34124	8	TRUE	TRUE
1	KEIDRLNEV	1163	1171	9	HLA-A*02:01	1163	8.1	1205.341196	8.1	1205.341196	8.1	TRUE	TRUE
1	AAAYFVGYL	249	257	9	HLA-A*02:01	249	8.3	1276.762152	8.3	1276.762152	8.3	TRUE	TRUE
1	TSYECDIPI	644	652	9	HLA-A*02:01	644	8.4	1282.655418	8.4	1282.655418	8.4	TRUE	TRUE
1	GFIAGLIAI	1201	1209	9	HLA-A*02:01	1201	8.6	1346.201283	8.6	1346.201283	8.6	TRUE	TRUE
1	KMSECVLGQ	1010	1018	9	HLA-A*02:01	1010	8.7	1371.229033	8.7	1371.229033	8.7	TRUE	TRUE
1	GINASVVNI	1153	1161	9	HLA-A*02:01	1153	8.7	1374.390042	8.7	1374.390042	8.7	TRUE	TRUE

seq #	peptide	start	end	peptide length	allele	peptide index	median binding	smm IC50	smm percentil	legacy IC50	legacy rank	scores equal	percentiles equal
1	YTAALVSGT	855	863	9	HLA-A*02:01	855	8.8	1403.16906	8.8	1403.16906	8.8	TRUE	TRUE
1	VFNGTSWFI	1078	1086	9	HLA-A*02:01	1078	8.8	1412.895364	8.8	1412.895364	8.8	TRUE	TRUE
1	FPQAAPHGV	1034	1042	9	HLA-A*02:01	1034	8.8	1422.689086	8.8	1422.689086	8.8	TRUE	TRUE
1	FQTQAGCLI	629	637	9	HLA-A*02:01	629	8.9	1449.138851	8.9	1449.138851	8.9	TRUE	TRUE
1	DVSDFTDSV	554	562	9	HLA-A*02:01	554	9	1469.298381	9	1469.298381	9	TRUE	TRUE
1	ILPDPLKPT	787	795	9	HLA-A*02:01	787	9.1	1496.614677	9.1	1496.614677	9.1	TRUE	TRUE
1	TSVDCNMYI	716	724	9	HLA-A*02:01	716	9.2	1503.522737	9.2	1503.522737	9.2	TRUE	TRUE
1	LITGRLQSL	978	986	9	HLA-A*02:01	978	9.2	1517.434661	9.2	1517.434661	9.2	TRUE	TRUE
1	AISSVLNDI	954	962	9	HLA-A*02:01	954	9.3	1545.645878	9.3	1545.645878	9.3	TRUE	TRUE
1	SIAYSNNTI	686	694	9	HLA-A*02:01	686	9.3	1567.147954	9.3	1567.147954	9.3	TRUE	TRUE
1	NTLKPIFKL	214	222	9	HLA-A*02:01	214	9.5	1585.294672	9.5	1585.294672	9.5	TRUE	TRUE
1	ALNCYWPLN	471	479	9	HLA-A*02:01	471	9.5	1596.28341	9.5	1596.28341	9.5	TRUE	TRUE
1	DMIAAYTAA	850	858	9	HLA-A*02:01	850	9.7	1652.380267	9.7	1652.380267	9.7	TRUE	TRUE
1	KLPLGINIT	221	229	9	HLA-A*02:01	221	9.7	1663.83402	9.7	1663.83402	9.7	TRUE	TRUE
1	DISGINASV	1150	1158	9	HLA-A*02:01	1150	9.7	1663.83402	9.7	1663.83402	9.7	TRUE	TRUE
1	IYQTSNFRV	299	307	9	HLA-A*02:01	299	9.7	1671.513929	9.7	1671.513929	9.7	TRUE	TRUE
1	AISQIQESL	912	920	9	HLA-A*02:01	912	9.8	1690.869148	9.8	1690.869148	9.8	TRUE	TRUE
1	AALQIPFAM	874	882	9	HLA-A*02:01	874	9.9	1730.25455	9.9	1730.25455	9.9	TRUE	TRUE
1	LCDNPFFAV	132	140	9	HLA-A*02:01	132	9.9	1730.25455	9.9	1730.25455	9.9	TRUE	TRUE
1	FAATEKSNV	89	97	9	HLA-A*02:01	89	10	1738.241042	10	1738.241042	10	TRUE	TRUE
1	AAPHGVVFL	1037	1045	9	HLA-A*02:01	1037	10	1750.289954	10	1750.289954	10	TRUE	TRUE
1	WTFGAGAAL	868	876	9	HLA-A*02:01	868	10	1770.557356	10	1770.557356	10	TRUE	TRUE
1	FITQRNFFS	1085	1093	9	HLA-A*02:01	1085	10	1774.638912	10	1774.638912	10	TRUE	TRUE
1	IVMVTILLC	1209	1217	9	HLA-A*02:01	1209	11	1786.940122	11	1786.940122	11	TRUE	TRUE
1	DLPSGFNTL	208	216	9	HLA-A*02:01	208	12	1824.357725	12	1824.357725	12	TRUE	TRUE
1	ELDSFKEEL	1126	1134	9	HLA-A*02:01	1126	12	1849.737069	12	1849.737069	12	TRUE	TRUE
1	ISDAFSLDV	164	172	9	HLA-A*02:01	164	12	1884.126249	12	1884.126249	12	TRUE	TRUE
1	GINITNFRA	225	233	9	HLA-A*02:01	225	12	1905.943402	12	1905.943402	12	TRUE	TRUE
1	AQDIWGTSA	241	249	9	HLA-A*02:01	241	12	1919.154771	12	1919.154771	12	TRUE	TRUE
1	FAMQMAYRF	880	888	9	HLA-A*02:01	880	12	1923.57888	12	1923.57888	12	TRUE	TRUE
1	DLCFSNVYA	376	384	9	HLA-A*02:01	376	12	1963.857628	12	1963.857628	12	TRUE	TRUE
1	FDEDDSEPV	1238	1246	9	HLA-A*02:01	1238	12	1972.922383	12	1972.922383	12	TRUE	TRUE
1	VVVLSFELL	496	504	9	HLA-A*02:01	496	12	2000.368468	12	2000.368468	12	TRUE	TRUE
1	FSQILPDPL	784	792	9	HLA-A*02:01	784	12	2046.963035	12	2046.963035	12	TRUE	TRUE
1	ELGKYEQYI	1184	1192	9	HLA-A*02:01	1184	12	2065.903351	12	2065.903351	12	TRUE	TRUE
1	YGFYTTTGI	481	489	9	HLA-A*02:01	481	12	2075.439127	12	2075.439127	12	TRUE	TRUE
1	FLYVYKGYQ	193	201	9	HLA-A*02:01	193	12	2114.024421	12	2114.024421	12	TRUE	TRUE
1	PALNCYWPL	470	478	9	HLA-A*02:01	470	12	2168.25322	12	2168.25322	12	TRUE	TRUE
1	ILDISPCSF	570	578	9	HLA-A*02:01	570	12	2168.25322	12	2168.25322	12	TRUE	TRUE
1	VYDPLQPEL	1119	1127	9	HLA-A*02:01	1119	13	2234.138023	13	2234.138023	13	TRUE	TRUE
1	YFPREGVFV	1070	1078	9	HLA-A*02:01	1070	13	2270.439846	13	2270.439846	13	TRUE	TRUE
1	YICGDSTEC	723	731	9	HLA-A*02:01	723	13	2280.91972	13	2280.91972	13	TRUE	TRUE
1	DLLFNKVTL	802	810	9	HLA-A*02:01	802	13	2302.02481	13	2302.02481	13	TRUE	TRUE
1	GAALQIPFA	873	881	9	HLA-A*02:01	873	13	2312.650474	13	2312.650474	13	TRUE	TRUE
1	IAIVMVTIL	1207	1215	9	HLA-A*02:01	1207	13	2328.681001	13	2328.681001	13	TRUE	TRUE
1	YECDIPIGA	646	654	9	HLA-A*02:01	646	13	2449.68363	13	2449.68363	13	TRUE	TRUE
1	MVTILLCCM	1211	1219	9	HLA-A*02:01	1211	13	2483.762124	13	2483.762124	13	TRUE	TRUE
1	FIEDLLFNK	799	807	9	HLA-A*02:01	799	13	2524.120012	13	2524.120012	13	TRUE	TRUE
1	AGLIAIVMV	1204	1212	9	HLA-A*02:01	1204	13	2524.12001	13	2524.12001	13	TRUE	TRUE
1	NAQALNTLV	937	945	9	HLA-A*02:01	937	13	2559.234019	13	2559.234019	13	TRUE	TRUE
1	SNFGAISSV	950	958	9	HLA-A*02:01	950	13	2565.133662	13	2565.133662	13	TRUE	TRUE
1	NGLTVLPPL	838	846	9	HLA-A*02:01	838	14	2588.868552	14	2588.868552	14	TRUE	TRUE
1	PLLTDDMIA	845	853	9	HLA-A*02:01	845	14	2606.813732	14	2606.813732	14	TRUE	TRUE
1	NTREVFAQV	759	767	9	HLA-A*02:01	759	14	2643.078123	14	2643.078123	14	TRUE	TRUE
1	QQLIRAAEI	992	1000	9	HLA-A*02:01	992	14	2710.878168	14	2710.878168	14	TRUE	TRUE
1	VMPVSMAKT	708	716	9	HLA-A*02:01	708	14	2710.878168	14	2710.878168	14	TRUE	TRUE
1	FKLPLGINI	220	228	9	HLA-A*02:01	220	14	2723.391013	14	2723.391013	14	TRUE	TRUE
1	LQTYVTQQL	986	994	9	HLA-A*02:01	986	14	2825.595563	14	2825.595563	14	TRUE	TRUE
1	CVLGQSKRV	1014	1022	9	HLA-A*02:01	1014	14	2898.077532	14	2898.077532	14	TRUE	TRUE
1	KLQDVVNQN	929	937	9	HLA-A*02:01	929	14	2931.635689	14	2931.635689	14	TRUE	TRUE
1	FFSPQIITT	1091	1099	9	HLA-A*02:01	1091	14	2931.635689	14	2931.635689	14	TRUE	TRUE
1	LLCCMTSCC	1215	1223	9	HLA-A*02:01	1215	14	2938.393807	14	2938.393807	14	TRUE	TRUE
1	KLSTDLIKN	514	522	9	HLA-A*02:01	514	14	2938.393807	14	2938.393807	14	TRUE	TRUE
1	DILSRLDKV	961	969	9	HLA-A*02:01	961	14	2951.956816	14	2951.956816	14	TRUE	TRUE
1	ASYHTVSLL	658	666	9	HLA-A*02:01	658	14	2958.76178	14	2958.76178	14	TRUE	TRUE
1	VAYTMSLGA	675	683	9	HLA-A*02:01	675	14	3055.694975	14	3055.694975	14	TRUE	TRUE
1	LPDDFMGCV	412	420	9	HLA-A*02:01	412	14	3069.799422	14	3069.799422	14	TRUE	TRUE
1	DIWGTSAAA	243	251	9	HLA-A*02:01	243	14	3069.799422	14	3069.799422	14	TRUE	TRUE
1	YISDAFSLD	163	171	9	HLA-A*02:01	163	15	3119.67965	15	3119.67965	15	TRUE	TRUE
1	NMYICGDST	721	729	9	HLA-A*02:01	721	15	3126.871254	15	3126.871254	15	TRUE	TRUE
1	VVIGIINNT	1110	1118	9	HLA-A*02:01	1110	15	3214.474612	15	3214.474612	15	TRUE	TRUE
1	RVVPSGDVV	306	314	9	HLA-A*02:01	306	15	3404.944207	15	3404.944207	15	TRUE	TRUE
1	FRSDTLYLT	47	55	9	HLA-A*02:01	47	15	3436.449768	15	3436.449768	15	TRUE	TRUE
1	LYLTQDLFL	52	60	9	HLA-A*02:01	52	15	3444.371603	15	3444.371603	15	TRUE	TRUE
1	AATEKSNVV	90	98	9	HLA-A*02:01	90	15	3573.633412	15	3573.633412	15	TRUE	TRUE
1	TMIFDNAFN	150	158	9	HLA-A*02:01	150	15	3598.40466	15	3598.40466	15	TRUE	TRUE
1	TLADAGFMK	809	817	9	HLA-A*02:01	809	15	3615.014137	15	3615.014137	15	TRUE	TRUE
1	PIFKLPLGI	218	226	9	HLA-A*02:01	218	15	3640.072218	15	3640.072218	15	TRUE	TRUE
1	FKNHTSPDV	1138	1146	9	HLA-A*02:01	1138	16	3820.41024	16	3820.41024	16	TRUE	TRUE
1	TALGKLQDV	925	933	9	HLA-A*02:01	925	16	3838.044448	16	3838.044448	16	TRUE	TRUE
1	KNKDGFLYV	188	196	9	HLA-A*02:01	188	16	3909.399022	16	3909.399022	16	TRUE	TRUE
1	LQDVVNQNA	930	938	9	HLA-A*02:01	930	16	3918.411118	16	3918.411118	16	TRUE	TRUE
1	VQIDRLITG	973	981	9	HLA-A*02:01	973	16	3963.78418	16	3963.78418	16	TRUE	TRUE
1	YFVGYLKPT	252	260	9	HLA-A*02:01	252	16	4018.925911	16	4018.925911	16	TRUE	TRUE
1	CVNFNFNGL	524	532	9	HLA-A*02:01	524	16	4074.834739	16	4074.834739	16	TRUE	TRUE
1	HLREFVFKN	181	189	9	HLA-A*02:01	181	17	4218.033261	17	4218.033261	17	TRUE	TRUE
1	KLRPFERDI	447	455	9	HLA-A*02:01	447	17	4247.271271	17	4247.271271	17	TRUE	TRUE
1	FVGYLKPTT	253	261	9	HLA-A*02:01	253	17	4376.329367	17	4376.329367	17	TRUE	TRUE
1	GLTGTGVLT	531	539	9	HLA-A*02:01	531	17	4396.529586	17	4396.529586	17	TRUE	TRUE
1	KWPWYVWLG	1193	1201	9	HLA-A*02:01	1193	17	4416.823044	17	4416.823044	17	TRUE	TRUE
1	MSLGADSSI	679	687	9	HLA-A*02:01	679	17	4457.691405	17	4457.691405	17	TRUE	TRUE
1	FGAGAALQI	870	878	9	HLA-A*02:01	870	17	4467.967444	17	4467.967444	17	TRUE	TRUE
1	LTQDLFLPF	54	62	9	HLA-A*02:01	54	17	4509.309038	17	4509.309038	17	TRUE	TRUE
1	AFNCTFEYI	156	164	9	HLA-A*02:01	156	17	4509.309038	17	4509.309038	17	TRUE	TRUE
1	YHLMSFPQA	1029	1037	9	HLA-A*02:01	1029	17	4614.344339	17	4614.344339	17	TRUE	TRUE
1	CASYHTVSL	657	665	9	HLA-A*02:01	657	17	4700.131405	17	4700.131405	17	TRUE	TRUE
1	SMRGVYYPD	36	44	9	HLA-A*02:01	36	17	4743.621186	17	4743.621186	17	TRUE	TRUE
1	RAAEIRASA	996	1004	9	HLA-A*02:01	996	18	4921.641774	18	4921.641774	18	TRUE	TRUE
1	LQSLQTYVT	983	991	9	HLA-A*02:01	983	18	4978.6317	18	4978.6317	18	TRUE	TRUE
1	IFLLFLTLT	3	11	9	HLA-A*02:01	3	18	5001.612019	18	5001.612019	18	TRUE	TRUE
1	SVDCNMYIC	717	725	9	HLA-A*02:01	717	18	5036.281538	18	5036.281538	18	TRUE	TRUE
1	IAYSNNTIA	687	695	9	HLA-A*02:01	687	18	5141.738673	18	5141.738673	18	TRUE	TRUE

seq #	peptide	start	end	peptide length	allele	peptide index	median binding	smm IC50	smm percentil	legacy IC50	legacy rank	scores equal	percentiles equal	
1	FAVSKPMGT	138	146	9	HLA-A*02:01	138	18	5165.47186	18	5165.47186	18	TRUE	TRUE	
1	SMAKTSVDC	712	720	9	HLA-A*02:01	712	18	5165.47186	18	5165.47186	18	TRUE	TRUE	
1	SVVNIQKEI	1157	1165	9	HLA-A*02:01	1157	18	5297.976163	18	5297.976163	18	TRUE	TRUE	
1	CSFGGVSVI	576	584	9	HLA-A*02:01	576	18	5322.43051	18	5322.43051	18	TRUE	TRUE	
1	FQPFQQFGR	545	553	9	HLA-A*02:01	545	19	5534.902819	19	5534.902819	19	TRUE	TRUE	
1	QIQESLTTT	915	923	9	HLA-A*02:01	915	19	5611.900987	19	5611.900987	19	TRUE	TRUE	
1	QIANQFNKA	904	912	9	HLA-A*02:01	904	19	5637.804345	19	5637.804345	19	TRUE	TRUE	
1	TLYLTQDLF	51	59	9	HLA-A*02:01	51	19	5650.800826	19	5650.800826	19	TRUE	TRUE	
1	SSMRGVYYP	35	43	9	HLA-A*02:01	35	19	5676.883738	19	5676.883738	19	TRUE	TRUE	
1	IHADQLTPA	610	618	9	HLA-A*02:01	610	19	5689.970306	19	5689.970306	19	TRUE	TRUE	
1	YKGYQPIDV	197	205	9	HLA-A*02:01	197	19	5689.970306	19	5689.970306	19	TRUE	TRUE	
1	ILTAFSPAQ	234	242	9	HLA-A*02:01	234	19	5703.087043	19	5703.087043	19	TRUE	TRUE	
1	IFRSDTLYL	46	54	9	HLA-A*02:01	46	19	5716.23402	19	5716.23402	19	TRUE	TRUE	
1	VVRGWVFGS	97	105	9	HLA-A*02:01	97	19	5769.125678	19	5769.125678	19	TRUE	TRUE	
1	AQVKQMYKT	765	773	9	HLA-A*02:01	765	19	5835.92901	19	5835.92901	19	TRUE	TRUE	
1	STFFSTFKC	358	366	9	HLA-A*02:01	358	19	5876.381732	19	5876.381732	19	TRUE	TRUE	
1	VTLADAGFM	808	816	9	HLA-A*02:01	808	19	5971.865254	19	5971.865254	19	TRUE	TRUE	
1	YYPDEIFRS	41	49	9	HLA-A*02:01	41	19	6054.942175	19	6054.942175	19	TRUE	TRUE	
1	RLITGRLQS	977	985	9	HLA-A*02:01	977	19	6096.91303	19	6096.91303	19	TRUE	TRUE	
1	SLQTYVTQQ	985	993	9	HLA-A*02:01	985	19	6167.511955	19	6167.511955	19	TRUE	TRUE	
1	VDLGDISGI	1146	1154	9	HLA-A*02:01	1146	19	6167.511955	19	6167.511955	19	TRUE	TRUE	
1	VVNQNAQAL	933	941	9	HLA-A*02:01	933	19	6267.725963	19	6267.725963	19	TRUE	TRUE	
1	KAYFPREGV	1068	1076	9	HLA-A*02:01	1068	19	6282.174563	19	6282.174563	19	TRUE	TRUE	
1	KCSVKSFEI	287	295	9	HLA-A*02:01	287	20	6369.568317	20	6369.568317	20	TRUE	TRUE	
1	ALSGIAAEQ	748	756	9	HLA-A*02:01	748	20	6428.505207	20	6428.505207	20	TRUE	TRUE	
1	KSFEIDKGI	291	299	9	HLA-A*02:01	291	20	6487.987433	20	6487.987433	20	TRUE	TRUE	
1	NIQKEIDRL	1160	1168	9	HLA-A*02:01	1160	20	6593.408748	20	6593.408748	20	TRUE	TRUE	
1	DQLTPAWRI	613	621	9	HLA-A*02:01	613	20	6654.416808	20	6654.416808	20	TRUE	TRUE	
1	WYVWLGFIA	1196	1204	9	HLA-A*02:01	1196	20	6793.756861	20	6793.756861	20	TRUE	TRUE	
1	TILLCCMTS	1213	1221	9	HLA-A*02:01	1213	20	6793.756861	20	6793.756861	20	TRUE	TRUE	
1	SQKSIVAYT	670	678	9	HLA-A*02:01	670	20	6825.115418	20	6825.115418	20	TRUE	TRUE	
1	AATKMSECV	1007	1015	9	HLA-A*02:01	1007	20	6840.848933	20	6840.848933	20	TRUE	TRUE	
1	ISPCSFGGV	573	581	9	HLA-A*02:01	573	20	6856.618719	20	6856.618719	20	TRUE	TRUE	
1	STAIHADQL	607	615	9	HLA-A*02:01	607	20	6888.267433	20	6888.267433	20	TRUE	TRUE	
1	LLNAPATVC	503	511	9	HLA-A*02:01	503	20	6888.267433	20	6888.267433	20	TRUE	TRUE	
1	GYQPYRVVV	490	498	9	HLA-A*02:01	490	20	6984.092778	20	6984.092778	20	TRUE	TRUE	
1	LNDLCFSNV	374	382	9	HLA-A*02:01	374	20	7016.329886	20	7016.329886	20	TRUE	TRUE	
1	FNFNGLTGT	527	535	9	HLA-A*02:01	527	20	7064.964761	20	7064.964761	20	TRUE	TRUE	
1	SVIIINNST	113	121	9	HLA-A*02:01	113	20	7064.964761	20	7064.964761	20	TRUE	TRUE	
1	CVADYSVLY	348	356	9	HLA-A*02:01	348	20	7130.336075	20	7130.336075	20	TRUE	TRUE	
1	SVLYNSTFF	353	361	9	HLA-A*02:01	353	20	7146.7732	20	7146.7732	20	TRUE	TRUE	
1	STECANLLL	728	736	9	HLA-A*02:01	728	21	7262.89892	21	7262.89892	21	TRUE	TRUE	
1	AQKFNGLTV	834	842	9	HLA-A*02:01	834	21	7296.422938	21	7296.422938	21	TRUE	TRUE	
1	AYSNNTIAI	688	696	9	HLA-A*02:01	688	21	7363.935909	21	7363.935909	21	TRUE	TRUE	
1	GVSVITPGT	580	588	9	HLA-A*02:01	580	21	7397.926292	21	7397.926292	21	TRUE	TRUE	
1	YTMSLGADS	677	685	9	HLA-A*02:01	677	21	7535.464011	21	7535.464011	21	TRUE	TRUE	
1	SVLNDILSR	957	965	9	HLA-A*02:01	957	21	7675.558746	21	7675.558746	21	TRUE	TRUE	
1	KGYQPIDVV	198	206	9	HLA-A*02:01	198	21	7728.76318	21	7728.76318	21	TRUE	TRUE	
1	PMGTQTHTM	143	151	9	HLA-A*02:01	143	21	7764.437528	21	7764.437528	21	TRUE	TRUE	
1	MLKYDENGT	263	271	9	HLA-A*02:01	263	21	7764.437528	21	7764.437528	21	TRUE	TRUE	
1	GNCDVVIGI	1106	1114	9	HLA-A*02:01	1106	22	7963.610307	22	7963.610307	22	TRUE	TRUE	
1	YADSFVVKG	383	391	9	HLA-A*02:01	383	22	7981.968324	22	7981.968324	22	TRUE	TRUE	
1	CPFGEVFNA	323	331	9	HLA-A*02:01	323	22	8000.368662	22	8000.368662	22	TRUE	TRUE	
1	CLGDINARD	822	830	9	HLA-A*02:01	822	22	8111.664876	22	8111.664876	22	TRUE	TRUE	
1	NITNFRAIL	227	235	9	HLA-A*02:01	227	22	8111.664876	22	8111.664876	22	TRUE	TRUE	
1	STSQKSIVA	668	676	9	HLA-A*02:01	668	22	8205.593529	22	8205.593529	22	TRUE	TRUE	
1	AGWTFGAGA	866	874	9	HLA-A*02:01	866	22	8552.833153	22	8552.833153	22	TRUE	TRUE	
1	STGNNVFQT	623	631	9	HLA-A*02:01	623	22	8752.05423	22	8752.05423	22	TRUE	TRUE	
1	SQIQESLTT	914	922	9	HLA-A*02:01	914	22	8955.915762	22	8955.915762	22	TRUE	TRUE	
1	IEDLLFNKV	800	808	9	HLA-A*02:01	800	22	8976.56128	22	8976.56128	22	TRUE	TRUE	
1	FELCDNPFF	130	138	9	HLA-A*02:01	130	22	9017.995204	22	9017.995204	22	TRUE	TRUE	
1	LIRAAEIRA	994	1002	9	HLA-A*02:01	994	23	9249.32415	23	9249.32415	23	TRUE	TRUE	
1	VTILLCCMT	1212	1220	9	HLA-A*02:01	1212	23	9313.437404	23	9313.437404	23	TRUE	TRUE	
1	GIGVTQNVL	890	898	9	HLA-A*02:01	890	23	9356.42628	23	9356.42628	23	TRUE	TRUE	
1	TFVSGNCDV	1102	1110	9	HLA-A*02:01	1102	23	9421.281926	23	9421.281926	23	TRUE	TRUE	
1	QIPFAMQMA	877	885	9	HLA-A*02:01	877	23	9443.000224	23	9443.000224	23	TRUE	TRUE	
1	NASSEVAVL	589	597	9	HLA-A*02:01	589	23	9443.000224	23	9443.000224	23	TRUE	TRUE	
1	SISITTEVM	701	709	9	HLA-A*02:01	701	23	9596.436635	23	9596.436635	23	TRUE	TRUE	
1	QTYVTQQLI	987	995	9	HLA-A*02:01	987	23	9618.558706	23	9618.558706	23	TRUE	TRUE	
1	LMSFPQAAP	1031	1039	9	HLA-A*02:01	1031	23	9752.366186	23	9752.366186	23	TRUE	TRUE	
1	LTLTSGSDL	8	16	9	HLA-A*02:01	8	23	9979.52798	23	9979.52798	23	TRUE	TRUE	
1	FSLDVSEKS	168	176	9	HLA-A*02:01	168	23	9979.52798	23	9979.52798	23	TRUE	TRUE	
1	VLPPLLTDD	842	850	9	HLA-A*02:01	842	23	10095.08546	23	10095.08546	23	TRUE	TRUE	
1	SVITPGTNA	582	590	9	HLA-A*02:01	582	23	10118.35704	23	10118.35704	23	TRUE	TRUE	
1	VTQQLIRAA	990	998	9	HLA-A*02:01	990	23	10141.68226	23	10141.68226	23	TRUE	TRUE	
1	LYNSTFFST	355	363	9	HLA-A*02:01	355	23	10141.68226	23	10141.68226	23	TRUE	TRUE	
1	NGIGVTQNV	889	897	9	HLA-A*02:01	889	24	10165.0613	24	10165.0613	24	TRUE	TRUE	
1	TQDLFLPFY	55	63	9	HLA-A*02:01	55	24	10211.98104	24	10211.98104	24	TRUE	TRUE	
1	ITTDNTFVS	1097	1105	9	HLA-A*02:01	1097	24	10282.76712	24	10282.76712	24	TRUE	TRUE	
1	QKFNGLTVL	835	843	9	HLA-A*02:01	835	24	10354.04385	24	10354.04385	24	TRUE	TRUE	
1	QMYKTPTLK	769	777	9	HLA-A*02:01	769	24	10546.5399	24	10546.5399	24	TRUE	TRUE	
1	VRFPNITNL	314	322	9	HLA-A*02:01	314	24	10570.85218	24	10570.85218	24	TRUE	TRUE	
1	EYISDAFSL	162	170	9	HLA-A*02:01	162	24	10693.25688	24	10693.25688	24	TRUE	TRUE	
1	GCLIGAEHV	634	642	9	HLA-A*02:01	634	25	11326.87219	25	11326.87219	25	TRUE	TRUE	
1	FLTLTSGSD	7	15	9	HLA-A*02:01	7	25	11405.38632	25	11405.38632	25	TRUE	TRUE	
1	CLIGAEHVD	635	643	9	HLA-A*02:01	635	25	11510.91906	25	11510.91906	25	TRUE	TRUE	
1	FVFKNKDGF	185	193	9	HLA-A*02:01	185	25	11564.05104	25	11564.05104	25	TRUE	TRUE	
1	EIDKGIYQT	294	302	9	HLA-A*02:01	294	25	11671.0519	25	11671.0519	25	TRUE	TRUE	
1	YIKWPWYVW	1191	1199	9	HLA-A*02:01	1191	25	11942.90561	25	11942.90561	25	TRUE	TRUE	
1	FSPDGKPCT	460	468	9	HLA-A*02:01	460	25	11998.03156	25	11998.03156	25	TRUE	TRUE	
1	TMSLGADSS	678	686	9	HLA-A*02:01	678	25	12053.41196	25	12053.41196	25	TRUE	TRUE	
1	FGGFNFSQI	779	787	9	HLA-A*02:01	779	26	12621.47176	26	12621.47176	26	TRUE	TRUE	
1	IPTNFSISI	696	704	9	HLA-A*02:01	696	26	12679.72983	26	12679.72983	26	TRUE	TRUE	
1	RGVYYPDEI	38	46	9	HLA-A*02:01	38	26	12708.95963	26	12708.95963	26	TRUE	TRUE	
1	LTDDMIAAY	847	855	9	HLA-A*02:01	847	26	12738.2568	26	12738.2568	26	TRUE	TRUE	
1	SNCVADYSV	346	354	9	HLA-A*02:01	346	26	12767.62152	26	12767.62152	26	TRUE	TRUE	
1	EQDRNTREV	755	763	9	HLA-A*02:01	755	26	12915.46361	26	12915.46361	26	TRUE	TRUE	
1	GIINNTVYD	1113	1121	9	HLA-A*02:01	1113	26	12945.23683	26	12945.23683	26	TRUE	TRUE	
1	PWYVWLGFI	1195	1203	9	HLA-A*02:01	1195	26	13004.98932	26	13004.98932	26	TRUE	TRUE	
1	SGFNTLKPI	211	219	9	HLA-A*02:01	211	26	13216.3034	26	13216.3034	26	TRUE	TRUE	
1	CSQNPLAEL	278	286	9	HLA-A*02:01	278	26	13246.77013	26	13246.77013	26	TRUE	TRUE	
1	KQIANQFNK	903	911	9	HLA-A*02:01	903	26	13277.30709	26	13277.30709	26	TRUE	TRUE	

seq #	peptide	start	end	peptide length	allele	peptide index	median binding	smm IC50	smm percentil	legacy IC50	legacy rank	scores equal	percentiles equal	
1	ITTEVMPVS	704	712	9	HLA-A*02:01	704	26	13338.59235	26	13338.59235	26	TRUE	TRUE	
1	YLRHGKLRP	442	450	9	HLA-A*02:01	442	26	13338.59235	26	13338.59235	26	TRUE	TRUE	
1	FCGKGYHLM	1024	1032	9	HLA-A*02:01	1024	26	13586.5753	26	13586.5753	26	TRUE	TRUE	
1	GGFNFSQIL	780	788	9	HLA-A*02:01	780	26	13743.90042	26	13743.90042	26	TRUE	TRUE	
1	TTSTALGKL	922	930	9	HLA-A*02:01	922	26	13743.90042	26	13743.90042	26	TRUE	TRUE	
1	FNGLTGTGV	529	537	9	HLA-A*02:01	529	26	13999.41861	26	13999.41861	26	TRUE	TRUE	
1	SLLRSTSQK	664	672	9	HLA-A*02:01	664	26	14161.52424	26	14161.52424	26	TRUE	TRUE	
1	NAFNCTFEY	155	163	9	HLA-A*02:01	155	26	14194.16992	26	14194.16992	26	TRUE	TRUE	
1	NLLLQYGSF	733	741	9	HLA-A*02:01	733	27	14558.27772	27	14558.27772	27	TRUE	TRUE	
1	VTQNVLYEN	893	901	9	HLA-A*02:01	893	27	14591.83802	27	14591.83802	27	TRUE	TRUE	
1	KTSEILDIS	566	574	9	HLA-A*02:01	566	27	14692.98381	27	14692.98381	27	TRUE	TRUE	
1	DLFLPFYSN	57	65	9	HLA-A*02:01	57	27	15000.6473	27	15000.6473	27	TRUE	TRUE	
1	RNFTTAPAI	1055	1063	9	HLA-A*02:01	1055	27	15174.34661	27	15174.34661	27	TRUE	TRUE	
1	GADSSIAYS	682	690	9	HLA-A*02:01	682	27	15209.32709	27	15209.32709	27	TRUE	TRUE	
1	PVSMAKTSV	710	718	9	HLA-A*02:01	710	27	15244.38821	27	15244.38821	27	TRUE	TRUE	
1	VSATKLNDL	369	377	9	HLA-A*02:01	369	27	15279.53016	27	15279.53016	27	TRUE	TRUE	
1	ISGINASVV	1151	1159	9	HLA-A*02:01	1151	27	15350.05727	27	15350.05727	27	TRUE	TRUE	
1	LTPAWRIYS	615	623	9	HLA-A*02:01	615	27	15420.90991	27	15420.90991	27	TRUE	TRUE	
1	SFIEDLLFN	798	806	9	HLA-A*02:01	798	27	15420.90991	27	15420.90991	27	TRUE	TRUE	
1	SQERNFTTA	1052	1060	9	HLA-A*02:01	1052	27	15420.90991	27	15420.90991	27	TRUE	TRUE	
1	FPSVYAWER	334	342	9	HLA-A*02:01	334	27	15635.43614	27	15635.43614	27	TRUE	TRUE	
1	TLVKQLSSN	943	951	9	HLA-A*02:01	943	27	15707.60603	27	15707.60603	27	TRUE	TRUE	
1	IINNTVYDP	1114	1122	9	HLA-A*02:01	1114	27	15889.49154	27	15889.49154	27	TRUE	TRUE	
1	RASANLAAT	1001	1009	9	HLA-A*02:01	1001	28	16147.675	28	16147.675	28	TRUE	TRUE	
1	KYFGGFNFS	777	785	9	HLA-A*02:01	777	28	16869.80257	28	16869.80257	28	TRUE	TRUE	
1	EILDISPCS	569	577	9	HLA-A*02:01	569	28	16869.8026	28	16869.8026	28	TRUE	TRUE	
1	SNVTGFHTI	64	72	9	HLA-A*02:01	64	28	16986.73845	28	16986.73845	28	TRUE	TRUE	
1	INHTFGNPV	72	80	9	HLA-A*02:01	72	28	17025.89693	28	17025.89693	28	TRUE	TRUE	
1	VVLSFELLN	497	505	9	HLA-A*02:01	497	28	17262.75075	28	17262.75075	28	TRUE	TRUE	
1	SNVYADSFV	380	388	9	HLA-A*02:01	380	28	17302.5455	28	17302.5455	28	TRUE	TRUE	
1	YSNVTGFHT	63	71	9	HLA-A*02:01	63	28	17302.5455	28	17302.5455	28	TRUE	TRUE	
1	WVFGSTMNN	101	109	9	HLA-A*02:01	101	29	17705.57356	29	17705.57356	29	TRUE	TRUE	
1	SPAQDIWGT	239	247	9	HLA-A*02:01	239	29	18034.74475	29	18034.74475	29	TRUE	TRUE	
1	GTNASSEVA	587	595	9	HLA-A*02:01	587	29	18159.75563	29	18159.75563	29	TRUE	TRUE	
1	ITNFRAILT	228	236	9	HLA-A*02:01	228	29	18285.63304	29	18285.63304	29	TRUE	TRUE	
1	KSNVVRGWV	94	102	9	HLA-A*02:01	94	29	18285.63304	29	18285.63304	29	TRUE	TRUE	
1	SSIAYSNNT	685	693	9	HLA-A*02:01	685	29	18370.03569	29	18370.03569	29	TRUE	TRUE	
1	NLCPFGEVF	321	329	9	HLA-A*02:01	321	29	18668.52475	29	18668.52475	29	TRUE	TRUE	
1	LSFELLNAP	499	507	9	HLA-A*02:01	499	29	18754.69475	29	18754.69475	29	TRUE	TRUE	
1	LKYDENGTI	264	272	9	HLA-A*02:01	264	29	18754.69475	29	18754.69475	29	TRUE	TRUE	
1	FELLNAPAT	501	509	9	HLA-A*02:01	501	29	19015.59854	29	19015.59854	29	TRUE	TRUE	
1	QYGSFCTQL	737	745	9	HLA-A*02:01	737	29	19059.43402	29	19059.43402	29	TRUE	TRUE	
1	YQPIDVVRD	200	208	9	HLA-A*02:01	200	29	19059.43402	29	19059.43402	29	TRUE	TRUE	
1	TTFDDVQAP	20	28	9	HLA-A*02:01	20	29	19103.37056	29	19103.37056	29	TRUE	TRUE	
1	ISSVLNDIL	955	963	9	HLA-A*02:01	955	30	19369.12491	30	19369.12491	30	TRUE	TRUE	
1	IANQFNKAI	905	913	9	HLA-A*02:01	905	30	19458.52873	30	19458.52873	30	TRUE	TRUE	
1	IAGLIAIVM	1203	1211	9	HLA-A*02:01	1203	30	19503.38527	30	19503.38527	30	TRUE	TRUE	
1	IVAYTMSLG	674	682	9	HLA-A*02:01	674	30	19503.38527	30	19503.38527	30	TRUE	TRUE	
1	FCTQLNRAL	741	749	9	HLA-A*02:01	741	30	19820.28979	30	19820.28979	30	TRUE	TRUE	
1	ICAQKFNGL	832	840	9	HLA-A*02:01	832	30	19957.67748	30	19957.67748	30	TRUE	TRUE	
1	EVAVLYQDV	593	601	9	HLA-A*02:01	593	30	20096.01749	30	20096.01749	30	TRUE	TRUE	
1	VSGNCDVVI	1104	1112	9	HLA-A*02:01	1104	30	20142.34359	30	20142.34359	30	TRUE	TRUE	
1	KAISQIQES	911	919	9	HLA-A*02:01	911	30	20235.31643	30	20235.31643	30	TRUE	TRUE	
1	INARDLICA	826	834	9	HLA-A*02:01	826	30	20375.58094	30	20375.58094	30	TRUE	TRUE	
1	NITNLCPFG	318	326	9	HLA-A*02:01	318	30	20611.51905	30	20611.51905	30	TRUE	TRUE	
1	KQYGECLGD	817	825	9	HLA-A*02:01	817	30	20754.39127	30	20754.39127	30	TRUE	TRUE	
1	PFKDGIYFA	82	90	9	HLA-A*02:01	82	30	20994.7158	30	20994.7158	30	TRUE	TRUE	
1	AMQMAYRFN	881	889	9	HLA-A*02:01	881	30	21091.623	30	21091.623	30	TRUE	TRUE	
1	NSTNVVIRA	119	127	9	HLA-A*02:01	119	30	21091.623	30	21091.623	30	TRUE	TRUE	
1	FFSTFKCYG	360	368	9	HLA-A*02:01	360	31	21286.78139	31	21286.78139	31	TRUE	TRUE	
1	AGICASYHT	654	662	9	HLA-A*02:01	654	31	21682.5322	31	21682.5322	31	TRUE	TRUE	
1	TNLCPFGEV	320	328	9	HLA-A*02:01	320	31	21732.5156	31	21732.5156	31	TRUE	TRUE	
1	GYLKPTTFM	255	263	9	HLA-A*02:01	255	31	21782.61422	31	21782.61422	31	TRUE	TRUE	
1	TNASSEVAV	588	596	9	HLA-A*02:01	588	31	21782.61422	31	21782.61422	31	TRUE	TRUE	
1	VFLHVTYVP	1043	1051	9	HLA-A*02:01	1043	31	21832.82833	31	21832.82833	31	TRUE	TRUE	
1	PVIPFKDGI	79	87	9	HLA-A*02:01	79	31	21933.60409	31	21933.60409	31	TRUE	TRUE	
1	YKLPDDFMG	410	418	9	HLA-A*02:01	410	31	22034.84501	31	22034.84501	31	TRUE	TRUE	
1	ADYSVLYNS	350	358	9	HLA-A*02:01	350	31	22289.99649	31	22289.99649	31	TRUE	TRUE	
1	YSVLYNSTF	352	360	9	HLA-A*02:01	352	31	22444.50336	31	22444.50336	31	TRUE	TRUE	
1	LKPIFKLPL	216	224	9	HLA-A*02:01	216	31	22652.17979	31	22652.17979	31	TRUE	TRUE	
1	GIYQTSNFR	298	306	9	HLA-A*02:01	298	31	22756.7375	31	22756.7375	31	TRUE	TRUE	
1	SAAAYFVGY	248	256	9	HLA-A*02:01	248	31	22861.77783	31	22861.77783	31	TRUE	TRUE	
1	CVLAWNTRN	419	427	9	HLA-A*02:01	419	31	23179.81684	31	23179.81684	31	TRUE	TRUE	
1	KISNCVADY	344	352	9	HLA-A*02:01	344	31	23502.28021	31	23502.28021	31	TRUE	TRUE	
1	YFGGFNFSQ	778	786	9	HLA-A*02:01	778	31	23502.28021	31	23502.28021	31	TRUE	TRUE	
1	TDLIKNQCV	517	525	9	HLA-A*02:01	517	32	23884.16155	32	23884.16155	32	TRUE	TRUE	
1	NFGAISSVL	951	959	9	HLA-A*02:01	951	32	23884.16155	32	23884.16155	32	TRUE	TRUE	
1	VLTPSSKRF	537	545	9	HLA-A*02:01	537	32	23994.40583	32	23994.40583	32	TRUE	TRUE	
1	SFCTQLNRA	740	748	9	HLA-A*02:01	740	32	24105.15897	32	24105.15897	32	TRUE	TRUE	
1	GKPCTPPAL	464	472	9	HLA-A*02:01	464	32	24553.30734	32	24553.30734	32	TRUE	TRUE	
1	TLTSGSDLD	9	17	9	HLA-A*02:01	9	32	24553.30734	32	24553.30734	32	TRUE	TRUE	
1	ATKFPSVYA	331	339	9	HLA-A*02:01	331	32	24609.90856	32	24609.90856	32	TRUE	TRUE	
1	CDIPIGAGI	648	656	9	HLA-A*02:01	648	32	24666.64026	32	24666.64026	32	TRUE	TRUE	
1	INNTVYDPL	1115	1123	9	HLA-A*02:01	1115	32	24780.49629	32	24780.49629	32	TRUE	TRUE	
1	FPREGVFVF	1071	1079	9	HLA-A*02:01	1071	32	24837.62124	32	24837.62124	32	TRUE	TRUE	
1	DLDRCTTFD	15	23	9	HLA-A*02:01	15	32	24837.62124	32	24837.62124	32	TRUE	TRUE	
1	GQSKRVDFC	1017	1025	9	HLA-A*02:01	1017	32	24894.87787	32	24894.87787	32	TRUE	TRUE	
1	AVLYQDVNC	595	603	9	HLA-A*02:01	595	32	25299.38709	32	25299.38709	32	TRUE	TRUE	
1	LNDYGFYTT	478	486	9	HLA-A*02:01	478	32	25299.38709	32	25299.38709	32	TRUE	TRUE	
1	MTSCCSCLK	1219	1227	9	HLA-A*02:01	1219	32	25357.7082	32	25357.7082	32	TRUE	TRUE	
1	VITPGTNAS	583	591	9	HLA-A*02:01	583	32	25357.7082	32	25357.7082	32	TRUE	TRUE	
1	FKCYGVSAT	364	372	9	HLA-A*02:01	364	32	25533.47944	32	25533.47944	32	TRUE	TRUE	
1	IAPGQTGVI	397	405	9	HLA-A*02:01	397	32	25769.73782	32	25769.73782	32	TRUE	TRUE	
1	FGNPVIPFK	76	84	9	HLA-A*02:01	76	32	25829.1432	32	25829.1432	32	TRUE	TRUE	
1	YWPLNDYGF	475	483	9	HLA-A*02:01	475	32	25948.3651	32	25948.3651	32	TRUE	TRUE	
1	SFELLNAPA	500	508	9	HLA-A*02:01	500	33	26128.23058	33	26128.23058	33	TRUE	TRUE	
1	NCTDVSTAI	602	610	9	HLA-A*02:01	602	33	26369.99212	33	26369.99212	33	TRUE	TRUE	
1	SQSVIIINN	111	119	9	HLA-A*02:01	111	33	26491.71047	33	26491.71047	33	TRUE	TRUE	
1	FNFSQILPD	782	790	9	HLA-A*02:01	782	33	27108.78168	33	27108.78168	33	TRUE	TRUE	
1	GTATAGWTF	862	870	9	HLA-A*02:01	862	33	27108.78168	33	27108.78168	33	TRUE	TRUE	
1	HTINHTFGN	70	78	9	HLA-A*02:01	70	33	27233.91013	33	27233.91013	33	TRUE	TRUE	
1	QDIWGTSAA	242	250	9	HLA-A*02:01	242	33	27296.69078	33	27296.69078	33	TRUE	TRUE	

seq #	peptide	start	end	peptide length	allele	peptide index	median binding	smm IC50	smm percentile	legacy IC50	legacy rank	scores equal	percentiles equal
1	AYRFNGIGV	885	893	9	HLA-A*02:01	885	33	27422.68658	33	27422.68658	33	TRUE	TRUE
1	LQYGSFCTQ	736	744	9	HLA-A*02:01	736	33	27740.22632	33	27740.22632	33	TRUE	TRUE
1	QILPDPLKP	786	794	9	HLA-A*02:01	786	33	27740.22632	33	27740.22632	33	TRUE	TRUE
1	KTPTLKYFG	772	780	9	HLA-A*02:01	772	33	28126.13131	33	28126.13131	33	TRUE	TRUE
1	ITQRNFFSP	1086	1094	9	HLA-A*02:01	1086	34	28190.96874	34	28190.96874	34	TRUE	TRUE
1	TPAWRIYST	616	624	9	HLA-A*02:01	616	34	28255.9556	34	28255.9556	34	TRUE	TRUE
1	TITDAVDCS	271	279	9	HLA-A*02:01	271	34	28386.3792	34	28386.3792	34	TRUE	TRUE
1	VAKNLNESL	1171	1179	9	HLA-A*02:01	1171	34	28451.81656	34	28451.81656	34	TRUE	TRUE
1	VDCSQNPLA	276	284	9	HLA-A*02:01	276	34	28517.40477	34	28517.40477	34	TRUE	TRUE
1	FGRDVSDFT	551	559	9	HLA-A*02:01	551	34	28649.03513	34	28649.03513	34	TRUE	TRUE
1	NVFQTQAGC	627	635	9	HLA-A*02:01	627	34	29114.54451	34	29114.54451	34	TRUE	TRUE
1	AEIRASANL	998	1006	9	HLA-A*02:01	998	34	29316.35689	34	29316.35689	34	TRUE	TRUE
1	ESLIDLQEL	1177	1185	9	HLA-A*02:01	1177	34	29316.35689	34	29316.35689	34	TRUE	TRUE
1	QFNKAISQI	908	916	9	HLA-A*02:01	908	34	29451.67504	34	29451.67504	34	TRUE	TRUE
1	TAALVSGTA	856	864	9	HLA-A*02:01	856	34	29451.67504	34	29451.67504	34	TRUE	TRUE
1	FSNVYADSF	379	387	9	HLA-A*02:01	379	34	29519.56816	34	29519.56816	34	TRUE	TRUE
1	YEQYIKWPW	1188	1196	9	HLA-A*02:01	1188	34	30207.16724	34	30207.16724	34	TRUE	TRUE
1	GTSWFITQR	1081	1089	9	HLA-A*02:01	1081	34	30416.55332	34	30416.55332	34	TRUE	TRUE
1	LAATKMSEC	1006	1014	9	HLA-A*02:01	1006	34	30486.67072	34	30486.67072	34	TRUE	TRUE
1	DISPCSFGG	572	580	9	HLA-A*02:01	572	34	30768.76041	34	30768.76041	34	TRUE	TRUE
1	CTTFDDVQA	19	27	9	HLA-A*02:01	19	35	30982.03926	35	30982.03926	35	TRUE	TRUE
1	LTVLPPLLT	840	848	9	HLA-A*02:01	840	35	31053.46024	35	31053.46024	35	TRUE	TRUE
1	QDLFLPFYS	56	64	9	HLA-A*02:01	56	35	31053.46024	35	31053.46024	35	TRUE	TRUE
1	LICAQKFNG	831	839	9	HLA-A*02:01	831	35	31268.71254	35	31268.71254	35	TRUE	TRUE
1	TNFRAILTA	229	237	9	HLA-A*02:01	229	35	31340.79437	35	31340.79437	35	TRUE	TRUE
1	GIYFAATEK	86	94	9	HLA-A*02:01	86	35	31485.4569	35	31485.4569	35	TRUE	TRUE
1	HTMIFDNAF	149	157	9	HLA-A*02:01	149	35	31630.78717	35	31630.78717	35	TRUE	TRUE
1	IPIGAGICA	650	658	9	HLA-A*02:01	650	35	31776.78825	35	31776.78825	35	TRUE	TRUE
1	LLRSTSQKS	665	673	9	HLA-A*02:01	665	35	31850.04131	35	31850.04131	35	TRUE	TRUE
1	FNGTSWFIT	1079	1087	9	HLA-A*02:01	1079	35	32218.84741	35	32218.84741	35	TRUE	TRUE
1	QIDRLITGR	974	982	9	HLA-A*02:01	974	35	32218.84741	35	32218.84741	35	TRUE	TRUE
1	YNYKYRYLR	436	444	9	HLA-A*02:01	436	35	32442.17779	35	32442.17779	35	TRUE	TRUE
1	DTLYLTQDL	50	58	9	HLA-A*02:01	50	35	32742.36157	35	32742.36157	35	TRUE	TRUE
1	KPMGTQTHT	142	150	9	HLA-A*02:01	142	35	33121.50024	35	33121.50024	35	TRUE	TRUE
1	VRDPKTSEI	562	570	9	HLA-A*02:01	562	35	33274.38214	35	33274.38214	35	TRUE	TRUE
1	GVFVFNGTS	1075	1083	9	HLA-A*02:01	1075	35	33582.26621	35	33582.26621	35	TRUE	TRUE
1	QLIRAAEIR	993	1001	9	HLA-A*02:01	993	36	33892.99909	36	33892.99909	36	TRUE	TRUE
1	HTVSLLRST	661	669	9	HLA-A*02:01	661	36	34049.44207	36	34049.44207	36	TRUE	TRUE
1	RAILTAFSP	232	240	9	HLA-A*02:01	232	36	34049.44207	36	34049.44207	36	TRUE	TRUE
1	QPIDVVRDL	201	209	9	HLA-A*02:01	201	36	34364.49768	36	34364.49768	36	TRUE	TRUE
1	IGYQPYRVV	489	497	9	HLA-A*02:01	489	36	34364.49768	36	34364.49768	36	TRUE	TRUE
1	AWNTRNIDA	422	430	9	HLA-A*02:01	422	36	34523.11699	36	34523.11699	36	TRUE	TRUE
1	GVIADYNYK	403	411	9	HLA-A*02:01	403	36	34523.11699	36	34523.11699	36	TRUE	TRUE
1	FDNAFNCTF	153	161	9	HLA-A*02:01	153	36	35164.94966	36	35164.94966	36	TRUE	TRUE
1	IFDNAFNCT	152	160	9	HLA-A*02:01	152	36	35654.14284	36	35654.14284	36	TRUE	TRUE
1	RSTSQKSIV	667	675	9	HLA-A*02:01	667	36	36150.14137	36	36150.14137	36	TRUE	TRUE
1	VKGDDVRQI	389	397	9	HLA-A*02:01	389	36	36400.72218	36	36400.72218	36	TRUE	TRUE
1	TKFPSVYAW	332	340	9	HLA-A*02:01	332	36	36400.72218	36	36400.72218	36	TRUE	TRUE
1	IRASANLAA	1000	1008	9	HLA-A*02:01	1000	36	36568.74028	36	36568.74028	36	TRUE	TRUE
1	TAFSPAQDI	236	244	9	HLA-A*02:01	236	36	36568.74028	36	36568.74028	36	TRUE	TRUE
1	VADYSVLYN	349	357	9	HLA-A*02:01	349	36	36992.1863	36	36992.1863	36	TRUE	TRUE
1	ALQIPFAMQ	875	883	9	HLA-A*02:01	875	36	36992.18633	36	36992.18633	36	TRUE	TRUE
1	NQNAQALNT	935	943	9	HLA-A*02:01	935	37	37077.46213	37	37077.46213	37	TRUE	TRUE
1	NTIAIPTNF	692	700	9	HLA-A*02:01	692	37	37334.47082	37	37334.47082	37	TRUE	TRUE
1	FRAILTAFS	231	239	9	HLA-A*02:01	231	37	37506.79891	37	37506.79891	37	TRUE	TRUE
1	ICGDSTECA	724	732	9	HLA-A*02:01	724	37	37766.78362	37	37766.78362	37	TRUE	TRUE
1	RGWVFGSTM	99	107	9	HLA-A*02:01	99	37	37941.10717	37	37941.10717	37	TRUE	TRUE
1	MGCVLAWNT	417	425	9	HLA-A*02:01	417	37	38028.57045	37	38028.57045	37	TRUE	TRUE
1	AIHADQLTP	609	617	9	HLA-A*02:01	609	37	38468.92055	37	38468.92055	37	TRUE	TRUE
1	LQPELDSFK	1123	1131	9	HLA-A*02:01	1123	37	38557.60057	37	38557.60057	37	TRUE	TRUE
1	CYWPLNDYG	474	482	9	HLA-A*02:01	474	37	38557.60057	37	38557.60057	37	TRUE	TRUE
1	LFLTLTSGS	6	14	9	HLA-A*02:01	6	37	38557.60057	37	38557.60057	37	TRUE	TRUE
1	SLDVSEKSG	169	177	9	HLA-A*02:01	169	37	38646.48502	37	38646.48502	37	TRUE	TRUE
1	EIDRLNEVA	1164	1172	9	HLA-A*02:01	1164	37	38735.57436	37	38735.57436	37	TRUE	TRUE
1	TIAIPTNFS	693	701	9	HLA-A*02:01	693	37	38914.36965	37	38914.36965	37	TRUE	TRUE
1	INITNFRAI	226	234	9	HLA-A*02:01	226	37	39004.07654	37	39004.07654	37	TRUE	TRUE
1	YRFNGIGVT	886	894	9	HLA-A*02:01	886	37	39546.67729	37	39546.67729	37	TRUE	TRUE
1	DDMIAAYTA	849	857	9	HLA-A*02:01	849	37	39820.80176	37	39820.80176	37	TRUE	TRUE
1	FTDSVRDPK	558	566	9	HLA-A*02:01	558	37	39912.59819	37	39912.59819	37	TRUE	TRUE
1	FVVKGDDVR	387	395	9	HLA-A*02:01	387	37	39912.59819	37	39912.59819	37	TRUE	TRUE
1	PLQPELDSF	1122	1130	9	HLA-A*02:01	1122	37	39912.59819	37	39912.59819	37	TRUE	TRUE
1	VVRDLPSGF	205	213	9	HLA-A*02:01	205	37	39912.59819	37	39912.59819	37	TRUE	TRUE
1	STGNYNYKY	432	440	9	HLA-A*02:01	432	37	40004.60623	37	40004.60623	37	TRUE	TRUE
1	DISNVPFSP	454	462	9	HLA-A*02:01	454	37	40096.82638	37	40096.82638	37	TRUE	TRUE
1	ATKMSECVL	1008	1016	9	HLA-A*02:01	1008	38	41410.45477	38	41410.45477	38	TRUE	TRUE
1	FQQFGRDVS	548	556	9	HLA-A*02:01	548	38	41505.91573	38	41505.91573	38	TRUE	TRUE
1	WPWYVWLGF	1194	1202	9	HLA-A*02:01	1194	38	41986.53158	38	41986.53158	38	TRUE	TRUE
1	AIPTNFSIS	695	703	9	HLA-A*02:01	695	38	41986.53158	38	41986.53158	38	TRUE	TRUE
1	QYGECLGDI	818	826	9	HLA-A*02:01	818	38	42083.32054	38	42083.32054	38	TRUE	TRUE
1	YAWERKKIS	338	346	9	HLA-A*02:01	338	38	42570.62242	38	42570.62242	38	TRUE	TRUE
1	GDSTECANL	726	734	9	HLA-A*02:01	726	38	42767.11949	38	42767.11949	38	TRUE	TRUE
1	LVSGTATAG	859	867	9	HLA-A*02:01	859	38	42964.52354	38	42964.52354	38	TRUE	TRUE
1	TQLNRALSG	743	751	9	HLA-A*02:01	743	38	43162.83877	38	43162.83877	38	TRUE	TRUE
1	RDISNVPFS	453	461	9	HLA-A*02:01	453	38	43462.0293	38	43462.0293	38	TRUE	TRUE
1	KGDDVRQIA	390	398	9	HLA-A*02:01	390	38	44066.6463	38	44066.6463	38	TRUE	TRUE
1	KDGIYFAAT	84	92	9	HLA-A*02:01	84	38	44066.64633	38	44066.64633	38	TRUE	TRUE
1	PQAAPHGVV	1035	1043	9	HLA-A*02:01	1035	39	44885.90645	39	44885.90645	39	TRUE	TRUE
1	FEYISDAFS	161	169	9	HLA-A*02:01	161	39	44989.37915	39	44989.37915	39	TRUE	TRUE
1	GDVVRFPNI	311	319	9	HLA-A*02:01	311	39	45510.33161	39	45510.33161	39	TRUE	TRUE
1	NKSQSVIII	109	117	9	HLA-A*02:01	109	39	45615.24376	39	45615.24376	39	TRUE	TRUE
1	DFMGCVLAW	415	423	9	HLA-A*02:01	415	39	46037.31641	39	46037.31641	39	TRUE	TRUE
1	YTTTGIGYQ	484	492	9	HLA-A*02:01	484	39	46570.40341	39	46570.40341	39	TRUE	TRUE
1	IQESLTTTS	916	924	9	HLA-A*02:01	916	39	46785.36263	39	46785.36263	39	TRUE	TRUE
1	DSTECANLL	727	735	9	HLA-A*02:01	727	39	47001.31405	39	47001.31405	39	TRUE	TRUE
1	LGFIAGLIA	1200	1208	9	HLA-A*02:01	1200	39	47001.31405	39	47001.31405	39	TRUE	TRUE
1	LTGTGVLTP	532	540	9	HLA-A*02:01	532	39	47001.31405	39	47001.31405	39	TRUE	TRUE
1	RNFFSPQII	1089	1097	9	HLA-A*02:01	1089	39	47218.26226	39	47218.26226	39	TRUE	TRUE
1	TVLPPLLTD	841	849	9	HLA-A*02:01	841	39	47218.26226	39	47218.26226	39	TRUE	TRUE
1	AAYFVGYLK	250	258	9	HLA-A*02:01	250	39	47436.21186	39	47436.21186	39	TRUE	TRUE
1	DVVNQNAQA	932	940	9	HLA-A*02:01	932	39	47436.21186	39	47436.21186	39	TRUE	TRUE
1	FAQVKQMYK	764	772	9	HLA-A*02:01	764	39	47985.4973	39	47985.4973	39	TRUE	TRUE
1	LIGAEHVDT	636	644	9	HLA-A*02:01	636	39	48429.50164	39	48429.50164	39	TRUE	TRUE

seq #	peptide	start	end	peptide length	allele	peptide index	median binding	smm IC50	smm percentil	legacy IC50	legacy rank	scores equal	percentiles equal
1	VFQTQAGCL	628	636	9	HLA-A*02:01	628	39	48429.50164	39	48429.50164	39	TRUE	TRUE
1	NYNYKYRYL	435	443	9	HLA-A*02:01	435	39	48653.04206	39	48653.04206	39	TRUE	TRUE
1	YSNNTIAIP	689	697	9	HLA-A*02:01	689	39	48765.19891	39	48765.19891	39	TRUE	TRUE
1	ATSTGNYNY	430	438	9	HLA-A*02:01	430	40	49557.56965	40	49557.56965	40	TRUE	TRUE
1	SFVVKGDDV	386	394	9	HLA-A*02:01	386	40	50478.91366	40	50478.91366	40	TRUE	TRUE
1	INNSTNVVI	117	125	9	HLA-A*02:01	117	40	51181.1453	40	51181.1453	40	TRUE	TRUE
1	KRSFIEDLL	796	804	9	HLA-A*02:01	796	40	51299.13002	40	51299.13002	40	TRUE	TRUE
1	FSPAQDIWG	238	246	9	HLA-A*02:01	238	40	51299.13002	40	51299.13002	40	TRUE	TRUE
1	YTQHTSSMR	30	38	9	HLA-A*02:01	30	40	51299.13002	40	51299.13002	40	TRUE	TRUE
1	VFNATKFPS	328	336	9	HLA-A*02:01	328	40	52012.77201	40	52012.77201	40	TRUE	TRUE
1	PLGINITNF	223	231	9	HLA-A*02:01	223	40	52373.30732	40	52373.30732	40	TRUE	TRUE
1	TDDMIAAYT	848	856	9	HLA-A*02:01	848	40	52494.04026	40	52494.04026	40	TRUE	TRUE
1	ITPGTNASS	584	592	9	HLA-A*02:01	584	40	52494.0403	40	52494.0403	40	TRUE	TRUE
1	AALVSGTAT	857	865	9	HLA-A*02:01	857	40	52857.91156	40	52857.91156	40	TRUE	TRUE
1	YKYRYLRHG	438	446	9	HLA-A*02:01	438	40	52857.91156	40	52857.91156	40	TRUE	TRUE
1	AGAALQIPF	872	880	9	HLA-A*02:01	872	41	52979.76163	41	52979.76163	41	TRUE	TRUE
1	TNFSISITT	698	706	9	HLA-A*02:01	698	41	53346.9998	41	53346.9998	41	TRUE	TRUE
1	HADQLTPAW	611	619	9	HLA-A*02:01	611	41	53469.97733	41	53469.97733	41	TRUE	TRUE
1	VYYPDEIFR	40	48	9	HLA-A*02:01	40	41	53469.97733	41	53469.97733	41	TRUE	TRUE
1	RDLPSGFNT	207	215	9	HLA-A*02:01	207	41	53840.61351	41	53840.61351	41	TRUE	TRUE
1	YNSTFFSTF	356	364	9	HLA-A*02:01	356	41	54089.13049	41	54089.13049	41	TRUE	TRUE
1	LKGVKLHYT	1247	1255	9	HLA-A*02:01	1247	41	54338.79457	41	54338.79457	41	TRUE	TRUE
1	AAYTAALVS	853	861	9	HLA-A*02:01	853	41	55094.7225	41	55094.7225	41	TRUE	TRUE
1	VIIINNSTN	114	122	9	HLA-A*02:01	114	41	55094.7225	41	55094.7225	41	TRUE	TRUE
1	QIITTDNTF	1095	1103	9	HLA-A*02:01	1095	41	55221.72896	41	55221.72896	41	TRUE	TRUE
1	FSPQIITTD	1092	1100	9	HLA-A*02:01	1092	41	55476.62088	41	55476.62088	41	TRUE	TRUE
1	SANLAATKM	1003	1011	9	HLA-A*02:01	1003	41	56378.04345	41	56378.04345	41	TRUE	TRUE
1	NGLTGTGVL	530	538	9	HLA-A*02:01	530	41	56378.04345	41	56378.04345	41	TRUE	TRUE
1	QNAQALNTL	936	944	9	HLA-A*02:01	936	41	56638.27268	41	56638.27268	41	TRUE	TRUE
1	GTGVLTPSS	534	542	9	HLA-A*02:01	534	41	56899.70306	41	56899.70306	41	TRUE	TRUE
1	NLAATKMSE	1005	1013	9	HLA-A*02:01	1005	41	56899.70306	41	56899.70306	41	TRUE	TRUE
1	QAPNYTQHT	26	34	9	HLA-A*02:01	26	41	57030.87043	41	57030.87043	41	TRUE	TRUE
1	TLKPIFKLP	215	223	9	HLA-A*02:01	215	42	57162.34016	42	57162.34016	42	TRUE	TRUE
1	VDTSYECDI	642	650	9	HLA-A*02:01	642	42	57558.57057	42	57558.57057	42	TRUE	TRUE
1	DDFMGCVLA	414	422	9	HLA-A*02:01	414	42	57691.25678	42	57691.25678	42	TRUE	TRUE
1	TQTHTMIFD	146	154	9	HLA-A*02:01	146	42	57691.25678	42	57691.25678	42	TRUE	TRUE
1	QKSIVAYTM	671	679	9	HLA-A*02:01	671	42	58091.15347	42	58091.15347	42	TRUE	TRUE
1	TDVSTAIHA	604	612	9	HLA-A*02:01	604	42	58091.1535	42	58091.1535	42	TRUE	TRUE
1	SFGGVSVIT	577	585	9	HLA-A*02:01	577	42	58493.82211	42	58493.82211	42	TRUE	TRUE
1	CLKGACSCG	1225	1233	9	HLA-A*02:01	1225	42	58628.66429	42	58628.66429	42	TRUE	TRUE
1	LCFSNVYAD	377	385	9	HLA-A*02:01	377	42	58899.28191	42	58899.28191	42	TRUE	TRUE
1	SCCSCLKGA	1221	1229	9	HLA-A*02:01	1221	42	59171.1486	42	59171.1486	42	TRUE	TRUE
1	KFNGLTVLP	836	844	9	HLA-A*02:01	836	42	59307.55223	42	59307.55223	42	TRUE	TRUE
1	VLKGVKLHY	1246	1254	9	HLA-A*02:01	1246	42	60271.2224	42	60271.2224	42	TRUE	TRUE
1	YVPSQERNF	1049	1057	9	HLA-A*02:01	1049	42	60410.16195	42	60410.16195	42	TRUE	TRUE
1	KYDENGTIT	265	273	9	HLA-A*02:01	265	42	60549.42175	42	60549.42175	42	TRUE	TRUE
1	VNCTDVSTA	601	609	9	HLA-A*02:01	601	42	60828.90519	42	60828.90519	42	TRUE	TRUE
1	STFKCYGVS	362	370	9	HLA-A*02:01	362	42	61109.6787	42	61109.6787	42	TRUE	TRUE
1	TGIGYQPYR	487	495	9	HLA-A*02:01	487	42	61675.11955	42	61675.11955	42	TRUE	TRUE
1	TTDNTFVSG	1098	1106	9	HLA-A*02:01	1098	42	61959.79897	42	61959.79897	42	TRUE	TRUE
1	MSFPQAAPH	1032	1040	9	HLA-A*02:01	1032	42	63111.71763	42	63111.71763	42	TRUE	TRUE
1	NFKHLREFV	178	186	9	HLA-A*02:01	178	42	63257.20517	42	63257.20517	42	TRUE	TRUE
1	GTQTHTMIF	145	153	9	HLA-A*02:01	145	42	63257.20517	42	63257.20517	42	TRUE	TRUE
1	MGTQTHTMI	144	152	9	HLA-A*02:01	144	42	63403.02809	42	63403.02809	42	TRUE	TRUE
1	TFKCYGVSA	363	371	9	HLA-A*02:01	363	42	63549.18716	42	63549.18716	42	TRUE	TRUE
1	KFDEDDSEP	1237	1245	9	HLA-A*02:01	1237	43	63989.68908	43	63989.68908	43	TRUE	TRUE
1	KVTLADAGF	807	815	9	HLA-A*02:01	807	43	65029.43789	43	65029.43789	43	TRUE	TRUE
1	STMNNKSQS	105	113	9	HLA-A*02:01	105	43	65329.60014	43	65329.60014	43	TRUE	TRUE
1	IAAEQDRNT	752	760	9	HLA-A*02:01	752	43	65782.44329	43	65782.44329	43	TRUE	TRUE
1	EVMPVSMAK	707	715	9	HLA-A*02:01	707	43	66851.322	43	66851.322	43	TRUE	TRUE
1	LGDISGINA	1148	1156	9	HLA-A*02:01	1148	43	67469.88967	43	67469.88967	43	TRUE	TRUE
1	STNVVIRAC	120	128	9	HLA-A*02:01	120	43	67469.88967	43	67469.88967	43	TRUE	TRUE
1	FEIDKGIYQ	293	301	9	HLA-A*02:01	293	43	67781.3165	43	67781.3165	43	TRUE	TRUE
1	LTTTSTALG	920	928	9	HLA-A*02:01	920	43	68408.48933	43	68408.48933	43	TRUE	TRUE
1	TFDDVQAPN	21	29	9	HLA-A*02:01	21	43	68408.48933	43	68408.48933	43	TRUE	TRUE
1	GTITDAVDC	270	278	9	HLA-A*02:01	270	43	68566.18719	43	68566.18719	43	TRUE	TRUE
1	TVYDPLQPE	1118	1126	9	HLA-A*02:01	1118	43	68566.1872	43	68566.1872	43	TRUE	TRUE
1	EVFAQVKQM	762	770	9	HLA-A*02:01	762	43	68882.67433	43	68882.67433	43	TRUE	TRUE
1	KGYHLMSFP	1027	1035	9	HLA-A*02:01	1027	44	69200.62231	44	69200.62231	44	TRUE	TRUE
1	NVVRGWVFG	96	104	9	HLA-A*02:01	96	44	70001.92775	44	70001.92775	44	TRUE	TRUE
1	FFAVSKPMG	137	145	9	HLA-A*02:01	137	44	70163.29886	44	70163.29886	44	TRUE	TRUE
1	MNNKSQSVI	107	115	9	HLA-A*02:01	107	44	70487.15793	44	70487.15793	44	TRUE	TRUE
1	FNCTFEYIS	157	165	9	HLA-A*02:01	157	44	70975.75157	44	70975.75157	44	TRUE	TRUE
1	TAPAICHEG	1059	1067	9	HLA-A*02:01	1059	44	71303.36075	44	71303.36075	44	TRUE	TRUE
1	NIDATSTGN	427	435	9	HLA-A*02:01	427	44	72295.28931	44	72295.28931	44	TRUE	TRUE
1	FRVVPSGDV	305	313	9	HLA-A*02:01	305	44	73132.4293	44	73132.4293	44	TRUE	TRUE
1	HTFGNPVIP	74	82	9	HLA-A*02:01	74	44	73809.11534	44	73809.11534	44	TRUE	TRUE
1	GWTFGAGAA	867	875	9	HLA-A*02:01	867	44	73809.11534	44	73809.11534	44	TRUE	TRUE
1	TTEVMPVSM	705	713	9	HLA-A*02:01	705	44	74492.06268	44	74492.06268	44	TRUE	TRUE
1	CCMTSCCSC	1217	1225	9	HLA-A*02:01	1217	45	75181.32925	45	75181.32925	45	TRUE	TRUE
1	NFFSPQIIT	1090	1098	9	HLA-A*02:01	1090	45	75181.32925	45	75181.32925	45	TRUE	TRUE
1	SNNTIAIPT	690	698	9	HLA-A*02:01	690	45	75354.64011	45	75354.64011	45	TRUE	TRUE
1	TAIHADQLT	608	616	9	HLA-A*02:01	608	45	75528.35049	45	75528.35049	45	TRUE	TRUE
1	NDYGFYTTT	479	487	9	HLA-A*02:01	479	45	75528.3505	45	75528.3505	45	TRUE	TRUE
1	NCVADYSVL	347	355	9	HLA-A*02:01	347	45	75702.46132	45	75702.46132	45	TRUE	TRUE
1	YRYLRHGKL	440	448	9	HLA-A*02:01	440	45	75876.97352	45	75876.97352	45	TRUE	TRUE
1	TKLNDLCFS	372	380	9	HLA-A*02:01	372	45	75876.97352	45	75876.97352	45	TRUE	TRUE
1	IPFKDGIYF	81	89	9	HLA-A*02:01	81	45	76402.9276	45	76402.9276	45	TRUE	TRUE
1	CYGVSATKL	366	374	9	HLA-A*02:01	366	45	76755.58746	45	76755.58746	45	TRUE	TRUE
1	QRNFFSPQI	1088	1096	9	HLA-A*02:01	1088	45	76755.58746	45	76755.58746	45	TRUE	TRUE
1	SDLDRCTTF	14	22	9	HLA-A*02:01	14	45	76932.52737	45	76932.52737	45	TRUE	TRUE
1	ISNCVADYS	345	353	9	HLA-A*02:01	345	45	77465.79817	45	77465.79817	45	TRUE	TRUE
1	DFCGKGYHL	1023	1031	9	HLA-A*02:01	1023	45	78002.76543	45	78002.76543	45	TRUE	TRUE
1	GDINARDLI	824	832	9	HLA-A*02:01	824	45	78905.99489	45	78905.99489	45	TRUE	TRUE
1	SEILDISPC	568	576	9	HLA-A*02:01	568	45	79636.10307	45	79636.10307	45	TRUE	TRUE
1	IGAEHVDTS	637	645	9	HLA-A*02:01	637	45	80743.95162	45	80743.95162	45	TRUE	TRUE
1	AQALNTLVK	938	946	9	HLA-A*02:01	938	45	80930.0856	45	80930.0856	45	TRUE	TRUE
1	NQFNKAISQ	907	915	9	HLA-A*02:01	907	46	83197.44705	46	83197.44705	46	TRUE	TRUE
1	ITNLCPFGE	319	327	9	HLA-A*02:01	319	46	83389.23698	46	83389.23698	46	TRUE	TRUE
1	HTSPDVDLG	1141	1149	9	HLA-A*02:01	1141	46	85135.36455	46	85135.36455	46	TRUE	TRUE
1	LKYFGGFNF	776	784	9	HLA-A*02:01	776	46	85528.33153	46	85528.33153	46	TRUE	TRUE
1	NCDVVIGII	1107	1115	9	HLA-A*02:01	1107	46	85725.4947	46	85725.4947	46	TRUE	TRUE

seq #	peptide	start	end	peptide length	allele	peptide index	median binding	smm IC50	smm percentile	legacy IC50	legacy rank	scores equal	percentiles equal	
1	KPIFKLPLG	217	225	9	HLA-A*02:01	217	46	86319.71544	46	86319.71544	46	TRUE	TRUE	
1	GFMKQYGEC	814	822	9	HLA-A*02:01	814	46	87924.51877	46	87924.51877	46	TRUE	TRUE	
1	SQNPLAELK	279	287	9	HLA-A*02:01	279	47	88533.98242	47	88533.98242	47	TRUE	TRUE	
1	TGFHTINHT	67	75	9	HLA-A*02:01	67	47	89147.67066	47	89147.67066	47	TRUE	TRUE	
1	LIDLQELGK	1179	1187	9	HLA-A*02:01	1179	47	90596.20379	47	90596.20379	47	TRUE	TRUE	
1	SVYAWERKK	336	344	9	HLA-A*02:01	336	47	91434.48014	47	91434.48014	47	TRUE	TRUE	
1	LIKNQCVNF	519	527	9	HLA-A*02:01	519	47	91645.25838	47	91645.25838	47	TRUE	TRUE	
1	NTLVKQLSS	942	950	9	HLA-A*02:01	942	47	91856.52252	47	91856.52252	47	TRUE	TRUE	
1	VKQMYKTPT	767	775	9	HLA-A*02:01	767	47	92068.27368	47	92068.27368	47	TRUE	TRUE	
1	GQTGVIADY	400	408	9	HLA-A*02:01	400	47	93564.2628	47	93564.2628	47	TRUE	TRUE	
1	ASSEVAVLY	590	598	9	HLA-A*02:01	590	47	93779.9507	47	93779.9507	47	TRUE	TRUE	
1	IYFAATEKS	87	95	9	HLA-A*02:01	87	47	93779.95068	47	93779.95068	47	TRUE	TRUE	
1	LKPTKRSFI	792	800	9	HLA-A*02:01	792	48	95523.45013	48	95523.45013	48	TRUE	TRUE	
1	YPDEIFRSD	42	50	9	HLA-A*02:01	42	48	95523.45013	48	95523.45013	48	TRUE	TRUE	
1	TVCGPKLST	509	517	9	HLA-A*02:01	509	48	95523.45013	48	95523.45013	48	TRUE	TRUE	
1	RFPNITNLC	315	323	9	HLA-A*02:01	315	48	96185.58706	48	96185.58706	48	TRUE	TRUE	
1	DLICAQKFN	830	838	9	HLA-A*02:01	830	48	96407.31774	48	96407.31774	48	TRUE	TRUE	
1	SQILPDPLK	785	793	9	HLA-A*02:01	785	48	96629.55956	48	96629.55956	48	TRUE	TRUE	
1	DLIKNQCVN	518	526	9	HLA-A*02:01	518	48	97075.5813	48	97075.5813	48	TRUE	TRUE	
1	CANLLLQYG	731	739	9	HLA-A*02:01	731	48	98199.6636	48	98199.6636	48	TRUE	TRUE	
1	VNFNFNGLT	525	533	9	HLA-A*02:01	525	48	98199.6636	48	98199.6636	48	TRUE	TRUE	
1	AKNLNESLI	1172	1180	9	HLA-A*02:01	1172	48	98652.93264	48	98652.93264	48	TRUE	TRUE	
1	ESLTTTSTA	918	926	9	HLA-A*02:01	918	48	98880.3511	48	98880.3511	48	TRUE	TRUE	
1	EIRASANLA	999	1007	9	HLA-A*02:01	999	48	99565.75699	48	99565.75699	48	TRUE	TRUE	
1	QQFGRDVSD	549	557	9	HLA-A*02:01	549	48	99565.75699	48	99565.75699	48	TRUE	TRUE	
1	HGVVFLHVT	1040	1048	9	HLA-A*02:01	1040	48	100255.9138	48	100255.9138	48	TRUE	TRUE	
1	VVPSGDVVR	307	315	9	HLA-A*02:01	307	48	101183.5704	48	101183.5704	48	TRUE	TRUE	
1	TSAAAYFVG	247	255	9	HLA-A*02:01	247	48	101884.9414	48	101884.9414	48	TRUE	TRUE	
1	GAISSVLND	953	961	9	HLA-A*02:01	953	49	102591.1741	49	102591.1741	49	TRUE	TRUE	
1	FGGVSVITP	578	586	9	HLA-A*02:01	578	49	102827.6712	49	102827.6712	49	TRUE	TRUE	
1	MAYRFNGIG	884	892	9	HLA-A*02:01	884	49	103302.3021	49	103302.3021	49	TRUE	TRUE	
1	PIGAGICAS	651	659	9	HLA-A*02:01	651	49	104018.3595	49	104018.3595	49	TRUE	TRUE	
1	KNQCVNFNF	521	529	9	HLA-A*02:01	521	49	104980.8295	49	104980.8295	49	TRUE	TRUE	
1	QHTSSMRGV	32	40	9	HLA-A*02:01	32	49	105465.399	49	105465.399	49	TRUE	TRUE	
1	FTTAPAICH	1057	1065	9	HLA-A*02:01	1057	49	106441.2583	49	106441.2583	49	TRUE	TRUE	
1	YKTPTLKYF	771	779	9	HLA-A*02:01	771	49	106932.5688	49	106932.5688	49	TRUE	TRUE	
1	RALSGIAAE	747	755	9	HLA-A*02:01	747	49	107922.0037	49	107922.0037	49	TRUE	TRUE	
1	LTAFSPAQD	235	243	9	HLA-A*02:01	235	49	108170.7896	49	108170.7896	49	TRUE	TRUE	
1	ANLLLQYGS	732	740	9	HLA-A*02:01	732	49	108920.5937	49	108920.5937	49	TRUE	TRUE	
1	KSQSVIIIN	110	118	9	HLA-A*02:01	110	49	109423.348	49	109423.348	49	TRUE	TRUE	
1	VQAPNYTQH	25	33	9	HLA-A*02:01	25	49	109423.348	49	109423.348	49	TRUE	TRUE	
1	FKNKDGFLY	187	195	9	HLA-A*02:01	187	49	109928.4236	49	109928.4236	49	TRUE	TRUE	
1	CAQKFNGLT	833	841	9	HLA-A*02:01	833	50	111457.6803	50	111457.6803	50	TRUE	TRUE	
1	PFFAVSKPM	136	144	9	HLA-A*02:01	136	50	112230.268	50	112230.268	50	TRUE	TRUE	
1	RDPKTSEIL	563	571	9	HLA-A*02:01	563	50	114053.8632	50	114053.8632	50	TRUE	TRUE	
1	VYADSFVVK	382	390	9	HLA-A*02:01	382	50	114316.7845	50	114316.7845	50	TRUE	TRUE	
1	SPDVDLGDI	1143	1151	9	HLA-A*02:01	1143	50	114580.3119	50	114580.3119	50	TRUE	TRUE	
1	FYSNVTGFH	62	70	9	HLA-A*02:01	62	50	114844.4468	50	114844.4468	50	TRUE	TRUE	
1	SSNFGAISS	949	957	9	HLA-A*02:01	949	50	115109.1906	50	115109.1906	50	TRUE	TRUE	
1	CGPKLSTDL	511	519	9	HLA-A*02:01	511	50	115374.5446	50	115374.5446	50	TRUE	TRUE	
1	QLTPAWRIY	614	622	9	HLA-A*02:01	614	50	115640.5104	50	115640.5104	50	TRUE	TRUE	
1	AEVQIDRLI	971	979	9	HLA-A*02:01	971	50	116174.2828	50	116174.2828	50	TRUE	TRUE	
1	FNGLTVLPP	837	845	9	HLA-A*02:01	837	50	116174.2828	50	116174.2828	50	TRUE	TRUE	
1	DEDDSEPVL	1239	1247	9	HLA-A*02:01	1239	50	117249.2302	50	117249.2302	50	TRUE	TRUE	
1	LADAGFMKQ	810	818	9	HLA-A*02:01	810	50	118061.963	50	118061.963	50	TRUE	TRUE	
1	GFHTINHTF	68	76	9	HLA-A*02:01	68	50	118606.9123	50	118606.9123	50	TRUE	TRUE	
1	KSIVAYTMS	672	680	9	HLA-A*02:01	672	51	119154.3769	51	119154.3769	51	TRUE	TRUE	
1	LSRLDKVEA	963	971	9	HLA-A*02:01	963	51	119429.0561	51	119429.0561	51	TRUE	TRUE	
1	FGAISSVLN	952	960	9	HLA-A*02:01	952	51	119704.3685	51	119704.3685	51	TRUE	TRUE	
1	LGINITNFR	224	232	9	HLA-A*02:01	224	51	119704.3685	51	119704.3685	51	TRUE	TRUE	
1	ATVCGPKLS	508	516	9	HLA-A*02:01	508	51	120534.1196	51	120534.1196	51	TRUE	TRUE	
1	TQHTSSMRG	31	39	9	HLA-A*02:01	31	51	121649.4081	51	121649.4081	51	TRUE	TRUE	
1	ITDAVDCSQ	272	280	9	HLA-A*02:01	272	51	122775.0162	51	122775.0162	51	TRUE	TRUE	
1	GVVFLHVTY	1041	1049	9	HLA-A*02:01	1041	51	123058.0418	51	123058.0418	51	TRUE	TRUE	
1	GVYYPDEIF	39	47	9	HLA-A*02:01	39	51	123058.0418	51	123058.0418	51	TRUE	TRUE	
1	FNKVTLADA	805	813	9	HLA-A*02:01	805	51	123341.7199	51	123341.7199	51	TRUE	TRUE	
1	TFGNPVIPF	75	83	9	HLA-A*02:01	75	51	124196.6839	51	124196.6839	51	TRUE	TRUE	
1	VFKNKDGFL	186	194	9	HLA-A*02:01	186	51	125057.5741	51	125057.5741	51	TRUE	TRUE	
1	RSFIEDLLF	797	805	9	HLA-A*02:01	797	51	125345.8616	51	125345.8616	51	TRUE	TRUE	
1	PCSFGGVSV	575	583	9	HLA-A*02:01	575	51	125634.8137	51	125634.8137	51	TRUE	TRUE	
1	SNFRVVPSG	303	311	9	HLA-A*02:01	303	51	126505.6726	51	126505.6726	51	TRUE	TRUE	
1	GKYEQYIKW	1186	1194	9	HLA-A*02:01	1186	51	126505.6726	51	126505.6726	51	TRUE	TRUE	
1	GGVSVITPG	579	587	9	HLA-A*02:01	579	51	128265.5418	51	128265.5418	51	TRUE	TRUE	
1	SATKLNDLC	370	378	9	HLA-A*02:01	370	51	128857.5886	51	128857.5886	51	TRUE	TRUE	
1	VTYVPSQER	1047	1055	9	HLA-A*02:01	1047	51	129750.7868	51	129750.7868	51	TRUE	TRUE	
1	YGSFCTQLN	738	746	9	HLA-A*02:01	738	52	131859.0675	52	131859.0675	52	TRUE	TRUE	
1	VSMAKTSVD	711	719	9	HLA-A*02:01	711	52	131859.0675	52	131859.0675	52	TRUE	TRUE	
1	VVIRACNFE	123	131	9	HLA-A*02:01	123	52	132163.034	52	132163.034	52	TRUE	TRUE	
1	LGADSSIAY	681	689	9	HLA-A*02:01	681	52	133079.1444	52	133079.1444	52	TRUE	TRUE	
1	PGTNASSEV	586	594	9	HLA-A*02:01	586	52	134310.511	52	134310.511	52	TRUE	TRUE	
1	GVLTPSSKR	536	544	9	HLA-A*02:01	536	52	134620.1283	52	134620.1283	52	TRUE	TRUE	
1	YSTGNNVFQ	622	630	9	HLA-A*02:01	622	52	135865.753	52	135865.753	52	TRUE	TRUE	
1	SCLKGACSC	1224	1232	9	HLA-A*02:01	1224	52	137439.0042	5.20E+01	1.37E+05	52	TRUE	TRUE	
1	NYTQHTSSM	29	37	9	HLA-A*02:01	29	52	138710.7117	5.20E+01	1.39E+05	52	TRUE	TRUE	
1	RFQPFQQFG	544	552	9	HLA-A*02:01	544	52	138710.7117	5.20E+01	1.39E+05	52	TRUE	TRUE	
1	GDISGINAS	1149	1157	9	HLA-A*02:01	1149	52	138710.7117	5.20E+01	1.39E+05	52	TRUE	TRUE	
1	FKHLREFVF	179	187	9	HLA-A*02:01	179	52	139350.9712	5.20E+01	1.39E+05	52	TRUE	TRUE	
1	NHTFGNPVI	73	81	9	HLA-A*02:01	73	52	139350.9712	5.20E+01	1.39E+05	52	TRUE	TRUE	
1	GNNVFQTQA	625	633	9	HLA-A*02:01	625	52	139672.2084	5.20E+01	1.40E+05	52	TRUE	TRUE	
1	PHGVVFLHV	1039	1047	9	HLA-A*02:01	1039	52	141615.2424	5.20E+01	1.42E+05	52	TRUE	TRUE	
1	DLGDISGIN	1147	1155	9	HLA-A*02:01	1147	52	142596.8723	5.20E+01	1.43E+05	52	TRUE	TRUE	
1	EVAKNLNES	1170	1178	9	HLA-A*02:01	1170	53	143916.305	5.30E+01	1.44E+05	53	TRUE	TRUE	
1	WFITQRNFF	1084	1092	9	HLA-A*02:01	1084	53	144913.8851	5.30E+01	1.45E+05	53	TRUE	TRUE	
1	TTTGIGYQP	485	493	9	HLA-A*02:01	485	53	146929.8381	5.30E+01	1.47E+05	53	TRUE	TRUE	
1	TATAGWTFG	863	871	9	HLA-A*02:01	863	53	147608.0354	5.30E+01	1.48E+05	53	TRUE	TRUE	
1	STDLIKNQC	516	524	9	HLA-A*02:01	516	53	148973.8357	5.30E+01	1.49E+05	53	TRUE	TRUE	
1	TKMSECVLG	1009	1017	9	HLA-A*02:01	1009	53	149317.2559	5.30E+01	1.49E+05	53	TRUE	TRUE	
1	GECLGDINA	820	828	9	HLA-A*02:01	820	53	149661.4677	5.30E+01	1.50E+05	53	TRUE	TRUE	
1	DVNCTDVST	600	608	9	HLA-A*02:01	600	53	149661.4677	5.30E+01	1.50E+05	53	TRUE	TRUE	
1	RQIAPGQTG	395	403	9	HLA-A*02:01	395	53	150352.2737	5.30E+01	1.50E+05	53	TRUE	TRUE	
1	LYQDVNCTD	597	605	9	HLA-A*02:01	597	53	150698.8715	5.30E+01	1.51E+05	53	TRUE	TRUE	
1	AYTAALVSG	854	862	9	HLA-A*02:01	854	53	151046.2682	5.30E+01	1.51E+05	53	TRUE	TRUE	

seq #	peptide	start	end	peptide length	allele	peptide index	median binding	smm IC50	smm percentil	legacy IC50	legacy rank	scores equal	percentiles equal
1	ICASYHTVS	656	664	9	HLA-A*02:01	656	53	151394.4658	5.30E+01	1.51E+05	53	TRUE	TRUE
1	TSNFRVVPS	302	310	9	HLA-A*02:01	302	53	153147.5311	5.30E+01	1.53E+05	53	TRUE	TRUE
1	NKDGFLYVY	189	197	9	HLA-A*02:01	189	53	153500.5727	5.30E+01	1.54E+05	53	TRUE	TRUE
1	GIAAEQDRN	751	759	9	HLA-A*02:01	751	53	154564.5878	5.30E+01	1.55E+05	53	TRUE	TRUE
1	PQIITTDNT	1094	1102	9	HLA-A*02:01	1094	53	155278.0255	5.30E+01	1.55E+05	53	TRUE	TRUE
1	PATVCGPKL	507	515	9	HLA-A*02:01	507	53	155635.9783	5.30E+01	1.56E+05	53	TRUE	TRUE
1	PFERDISNV	450	458	9	HLA-A*02:01	450	53	155994.7563	5.30E+01	1.56E+05	53	TRUE	TRUE
1	ELDKYFKNH	1133	1141	9	HLA-A*02:01	1133	54	156714.7954	5.40E+01	1.57E+05	54	TRUE	TRUE
1	TKRSFIEDL	795	803	9	HLA-A*02:01	795	54	157076.0603	5.40E+01	1.57E+05	54	TRUE	TRUE
1	TLKYFGGFN	775	783	9	HLA-A*02:01	775	54	157076.0603	5.40E+01	1.57E+05	54	TRUE	TRUE
1	VYAWERKKI	337	345	9	HLA-A*02:01	337	54	157438.158	5.40E+01	1.57E+05	54	TRUE	TRUE
1	DSEPVLKGV	1242	1250	9	HLA-A*02:01	1242	54	157801.0905	5.40E+01	1.58E+05	54	TRUE	TRUE
1	TEVMPVSMA	706	714	9	HLA-A*02:01	706	54	161848.9924	5.40E+01	1.62E+05	54	TRUE	TRUE
1	KYFKNHTSP	1136	1144	9	HLA-A*02:01	1136	54	163346.5627	5.40E+01	1.63E+05	54	TRUE	TRUE
1	AKTSVDCNM	714	722	9	HLA-A*02:01	714	54	164478.827	5.40E+01	1.64E+05	54	TRUE	TRUE
1	KTSVDCNMY	715	723	9	HLA-A*02:01	715	54	165238.027	5.40E+01	1.65E+05	54	TRUE	TRUE
1	ICHEGKAYF	1063	1071	9	HLA-A*02:01	1063	54	165238.0267	5.40E+01	1.65E+05	54	TRUE	TRUE
1	QTHTMIFDN	147	155	9	HLA-A*02:01	147	54	166383.402	5.40E+01	1.66E+05	54	TRUE	TRUE
1	FGSTMNNKS	103	111	9	HLA-A*02:01	103	54	166383.402	5.40E+01	1.66E+05	54	TRUE	TRUE
1	PSQERNFTT	1051	1059	9	HLA-A*02:01	1051	54	166766.9554	5.40E+01	1.67E+05	54	TRUE	TRUE
1	GVTQNVLYE	892	900	9	HLA-A*02:01	892	55	169086.9148	5.50E+01	1.69E+05	55	TRUE	TRUE
1	QTQAGCLIG	630	638	9	HLA-A*02:01	630	55	169476.7004	5.50E+01	1.69E+05	55	TRUE	TRUE
1	VSGTATAGW	860	868	9	HLA-A*02:01	860	55	170258.9693	5.50E+01	1.70E+05	55	TRUE	TRUE
1	LPLGINITN	222	230	9	HLA-A*02:01	222	55	171834.3562	5.50E+01	1.72E+05	55	TRUE	TRUE
1	NQCVNFNFN	522	530	9	HLA-A*02:01	522	55	173025.455	5.50E+01	1.73E+05	55	TRUE	TRUE
1	AYFVGYLKP	251	259	9	HLA-A*02:01	251	55	174626.4398	5.50E+01	1.75E+05	55	TRUE	TRUE
1	SEVAVLYQD	592	600	9	HLA-A*02:01	592	55	176648.5187	5.50E+01	1.77E+05	55	TRUE	TRUE
1	LGDINARDL	823	831	9	HLA-A*02:01	823	55	178694.0122	5.50E+01	1.79E+05	55	TRUE	TRUE
1	PTLKYFGGF	774	782	9	HLA-A*02:01	774	55	179105.9444	5.50E+01	1.79E+05	55	TRUE	TRUE
1	GSFCTQLNR	739	747	9	HLA-A*02:01	739	55	180763.1913	5.50E+01	1.81E+05	55	TRUE	TRUE
1	IGAGICASY	652	660	9	HLA-A*02:01	652	56	181179.8935	5.60E+01	1.81E+05	56	TRUE	TRUE
1	YNYKLPDDF	408	416	9	HLA-A*02:01	408	56	183700.3569	5.60E+01	1.84E+05	56	TRUE	TRUE
1	ENGTITDAV	268	276	9	HLA-A*02:01	268	56	184123.8299	5.60E+01	1.84E+05	56	TRUE	TRUE
1	ADYNYKLPD	406	414	9	HLA-A*02:01	406	56	185827.5068	5.60E+01	1.86E+05	56	TRUE	TRUE
1	NVVIRACNF	122	130	9	HLA-A*02:01	122	56	186255.8834	5.60E+01	1.86E+05	56	TRUE	TRUE
1	TGTGVLTPS	533	541	9	HLA-A*02:01	533	56	186685.2475	5.60E+01	1.87E+05	56	TRUE	TRUE
1	DVVRDLPSG	204	212	9	HLA-A*02:01	204	56	187979.2879	5.60E+01	1.88E+05	56	TRUE	TRUE
1	WGTSAAAYF	245	253	9	HLA-A*02:01	245	56	189282.298	5.60E+01	1.89E+05	56	TRUE	TRUE
1	ADSSIAYSN	683	691	9	HLA-A*02:01	683	56	190155.9854	5.60E+01	1.90E+05	56	TRUE	TRUE
1	TINHTFGNP	71	79	9	HLA-A*02:01	71	56	195483.4522	5.60E+01	1.95E+05	56	TRUE	TRUE
1	LFNKVTLAD	804	812	9	HLA-A*02:01	804	56	196385.7628	5.60E+01	1.96E+05	56	TRUE	TRUE
1	TSWFITQRN	1082	1090	9	HLA-A*02:01	1082	56	197292.2383	5.60E+01	1.97E+05	56	TRUE	TRUE
1	QPYRVVVLS	492	500	9	HLA-A*02:01	492	56	198202.8979	5.60E+01	1.98E+05	56	TRUE	TRUE
1	KCYGVSATK	365	373	9	HLA-A*02:01	365	57	198659.8028	5.70E+01	1.99E+05	57	TRUE	TRUE
1	NTVYDPLQP	1117	1125	9	HLA-A*02:01	1117	57	199576.7748	5.70E+01	2.00E+05	57	TRUE	TRUE
1	LVKQLSSNF	944	952	9	HLA-A*02:01	944	57	200497.9793	5.70E+01	2.00E+05	57	TRUE	TRUE
1	DVVRFPNIT	312	320	9	HLA-A*02:01	312	57	200960.175	5.70E+01	2.01E+05	57	TRUE	TRUE
1	AELKCSVKS	284	292	9	HLA-A*02:01	284	57	203755.8094	5.70E+01	2.04E+05	57	TRUE	TRUE
1	VPSGDVVRF	308	316	9	HLA-A*02:01	308	57	203755.8094	5.70E+01	2.04E+05	57	TRUE	TRUE
1	REGVFVFNG	1073	1081	9	HLA-A*02:01	1073	57	204225.515	5.70E+01	2.04E+05	57	TRUE	TRUE
1	ISQIQESLT	913	921	9	HLA-A*02:01	913	57	205641.1387	5.70E+01	2.06E+05	57	TRUE	TRUE
1	LSSNFGAIS	948	956	9	HLA-A*02:01	948	57	205641.1387	5.70E+01	2.06E+05	57	TRUE	TRUE
1	IPFAMQMAY	878	886	9	HLA-A*02:01	878	57	206590.3351	5.70E+01	2.07E+05	57	TRUE	TRUE
1	GKGYHLMSF	1026	1034	9	HLA-A*02:01	1026	57	207066.575	5.70E+01	2.07E+05	57	TRUE	TRUE
1	IYSTGNNVF	621	629	9	HLA-A*02:01	621	57	207543.9127	5.70E+01	2.08E+05	57	TRUE	TRUE
1	THTMIFDNA	148	156	9	HLA-A*02:01	148	57	209464.2929	5.70E+01	2.09E+05	57	TRUE	TRUE
1	VLGQSKRVD	1015	1023	9	HLA-A*02:01	1015	57	209464.2929	5.70E+01	2.09E+05	57	TRUE	TRUE
1	ISITTEVMP	702	710	9	HLA-A*02:01	702	57	210431.1361	5.70E+01	2.10E+05	57	TRUE	TRUE
1	QAAPHGVVF	1036	1044	9	HLA-A*02:01	1036	57	213358.5248	5.70E+01	2.13E+05	57	TRUE	TRUE
1	GFNFSQILP	781	789	9	HLA-A*02:01	781	57	213358.5248	5.70E+01	2.13E+05	57	TRUE	TRUE
1	TVSLLRSTS	662	670	9	HLA-A*02:01	662	57	213850.367	5.70E+01	2.14E+05	57	TRUE	TRUE
1	FYTTTGIGY	483	491	9	HLA-A*02:01	483	57	213850.367	5.70E+01	2.14E+05	57	TRUE	TRUE
1	YFKNHTSPD	1137	1145	9	HLA-A*02:01	1137	58	214343.3431	5.80E+01	2.14E+05	58	TRUE	TRUE
1	LPFYSNVTG	60	68	9	HLA-A*02:01	60	58	214837.4555	5.80E+01	2.15E+05	58	TRUE	TRUE
1	SYHTVSLLR	659	667	9	HLA-A*02:01	659	58	217826.1422	5.80E+01	2.18E+05	58	TRUE	TRUE
1	EQYIKWPWY	1189	1197	9	HLA-A*02:01	1189	58	217826.1422	5.80E+01	2.18E+05	58	TRUE	TRUE
1	GACSCGSCC	1228	1236	9	HLA-A*02:01	1228	58	217826.1422	5.80E+01	2.18E+05	58	TRUE	TRUE
1	IDRLITGRL	975	983	9	HLA-A*02:01	975	58	217826.1422	5.80E+01	2.18E+05	58	TRUE	TRUE
1	APHGVVFLH	1038	1046	9	HLA-A*02:01	1038	58	220856.4057	5.80E+01	2.21E+05	58	TRUE	TRUE
1	VTGFHTINH	66	74	9	HLA-A*02:01	66	58	221365.5323	5.80E+01	2.21E+05	58	TRUE	TRUE
1	SSVLNDILS	956	964	9	HLA-A*02:01	956	58	222387.3092	5.80E+01	2.22E+05	58	TRUE	TRUE
1	TTAPAICHE	1058	1066	9	HLA-A*02:01	1058	58	224962.4328	5.80E+01	2.25E+05	58	TRUE	TRUE
1	FDDVQAPNY	22	30	9	HLA-A*02:01	22	58	229673.03	5.80E+01	2.30E+05	58	TRUE	TRUE
1	NAPATVCGP	505	513	9	HLA-A*02:01	505	58	229673.03	5.80E+01	2.30E+05	58	TRUE	TRUE
1	KGIYQTSNF	297	305	9	HLA-A*02:01	297	58	230733.1526	5.80E+01	2.31E+05	58	TRUE	TRUE
1	TQRNFFSPQ	1087	1095	9	HLA-A*02:01	1087	59	232868.1001	5.90E+01	2.33E+05	59	TRUE	TRUE
1	GSDLDRCTT	13	21	9	HLA-A*02:01	13	59	235022.8021	5.90E+01	2.35E+05	59	TRUE	TRUE
1	LKGACSCGS	1226	1234	9	HLA-A*02:01	1226	59	235022.8021	5.90E+01	2.35E+05	59	TRUE	TRUE
1	PFGEVFNAT	324	332	9	HLA-A*02:01	324	59	235564.5857	5.90E+01	2.36E+05	59	TRUE	TRUE
1	KSGNFKHLR	175	183	9	HLA-A*02:01	175	59	237197.4414	5.90E+01	2.37E+05	59	TRUE	TRUE
1	YHTVSLLRS	660	668	9	HLA-A*02:01	660	59	238292.295	5.90E+01	2.38E+05	59	TRUE	TRUE
1	VGYLKPTTF	254	262	9	HLA-A*02:01	254	59	240497.1865	5.90E+01	2.40E+05	59	TRUE	TRUE
1	LRSTSQKSI	666	674	9	HLA-A*02:01	666	59	241607.271	5.90E+01	2.42E+05	59	TRUE	TRUE
1	SGSDLDRCT	12	20	9	HLA-A*02:01	12	59	242164.2333	5.90E+01	2.42E+05	59	TRUE	TRUE
1	SWFITQRNF	1083	1091	9	HLA-A*02:01	1083	59	243842.8356	5.90E+01	2.44E+05	59	TRUE	TRUE
1	DVSTAIHAD	605	613	9	HLA-A*02:01	605	59	244404.9514	5.90E+01	2.44E+05	59	TRUE	TRUE
1	IRACNFELC	125	133	9	HLA-A*02:01	125	59	246099.0856	5.90E+01	2.46E+05	59	TRUE	TRUE
1	ILSRLDKVE	962	970	9	HLA-A*02:01	962	59	246666.4026	5.90E+01	2.47E+05	59	TRUE	TRUE
1	LDISPCSFG	571	579	9	HLA-A*02:01	571	59	251252.2734	5.90E+01	2.51E+05	59	TRUE	TRUE
1	KGACSCGSC	1227	1235	9	HLA-A*02:01	1227	59	252412.0012	5.90E+01	2.52E+05	59	TRUE	TRUE
1	AFSLDVSEK	167	175	9	HLA-A*02:01	167	60	253577.082	6.00E+01	2.54E+05	60	TRUE	TRUE
1	NRALSGIAA	746	754	9	HLA-A*02:01	746	60	253577.082	6.00E+01	2.54E+05	60	TRUE	TRUE
1	CDNPFFAVS	133	141	9	HLA-A*02:01	133	60	254161.6376	6.00E+01	2.54E+05	60	TRUE	TRUE
1	DGKPCTPPA	463	471	9	HLA-A*02:01	463	60	255923.4019	6.00E+01	2.56E+05	60	TRUE	TRUE
1	RNIDATSTG	426	434	9	HLA-A*02:01	426	60	257104.691	6.00E+01	2.57E+05	60	TRUE	TRUE
1	CTPPALNCY	467	475	9	HLA-A*02:01	467	60	258291.432	6.00E+01	2.58E+05	60	TRUE	TRUE
1	FKEELDKYF	1130	1138	9	HLA-A*02:01	1130	60	258886.8552	6.00E+01	2.59E+05	60	TRUE	TRUE
1	ASANLAATK	1002	1010	9	HLA-A*02:01	1002	60	261282.3058	6.00E+01	2.61E+05	60	TRUE	TRUE
1	TPPALNCYW	468	476	9	HLA-A*02:01	468	60	261282.3058	6.00E+01	2.61E+05	60	TRUE	TRUE
1	DGFLYVYKG	191	199	9	HLA-A*02:01	191	60	263699.9212	6.00E+01	2.64E+05	60	TRUE	TRUE
1	FPNITNLCP	316	324	9	HLA-A*02:01	316	60	264917.1047	6.00E+01	2.65E+05	60	TRUE	TRUE
1	LTSGSDLDR	10	18	9	HLA-A*02:01	10	60	266753.4224	6.00E+01	2.67E+05	60	TRUE	TRUE

seq #	peptide	start	end	peptide length	allele	peptide index	median binding	smm IC50	smm percentil	legacy IC50	legacy rank	scores equal	percentiles equal	
1	QCVNFNFNG	523	531	9	HLA-A*02:01	523	60	270464.3321	6.00E+01	2.70E+05	60	TRUE	TRUE	
1	VFVFNGTSW	1076	1084	9	HLA-A*02:01	1076	60	270464.3321	6.00E+01	2.70E+05	60	TRUE	TRUE	
1	DKVEAEVQI	967	975	9	HLA-A*02:01	967	60	271712.7388	6.00E+01	2.72E+05	60	TRUE	TRUE	
1	HVDTSYECD	641	649	9	HLA-A*02:01	641	60	271712.7388	6.00E+01	2.72E+05	60	TRUE	TRUE	
1	LQELGKYEQ	1182	1190	9	HLA-A*02:01	1182	60	273596.1615	6.00E+01	2.74E+05	60	TRUE	TRUE	
1	TFMLKYDEN	261	269	9	HLA-A*02:01	261	60	273596.1615	6.00E+01	2.74E+05	60	TRUE	TRUE	
1	GNFKHLREF	177	185	9	HLA-A*02:01	177	61	279969.0348	6.10E+01	2.80E+05	61	TRUE	TRUE	
1	NFRAILTAF	230	238	9	HLA-A*02:01	230	61	279969.0348	6.10E+01	2.80E+05	61	TRUE	TRUE	
1	KKISNCVAD	343	351	9	HLA-A*02:01	343	61	285831.4418	6.10E+01	2.86E+05	61	TRUE	TRUE	
1	AFSPAQDIW	237	245	9	HLA-A*02:01	237	61	286490.3513	6.10E+01	2.86E+05	61	TRUE	TRUE	
1	PAICHEGKA	1061	1069	9	HLA-A*02:01	1061	61	287150.7798	6.10E+01	2.87E+05	61	TRUE	TRUE	
1	PTNFSISIT	697	705	9	HLA-A*02:01	697	61	290475.8291	6.10E+01	2.90E+05	61	TRUE	TRUE	
1	GVSATKLND	368	376	9	HLA-A*02:01	368	61	291816.6046	6.10E+01	2.92E+05	61	TRUE	TRUE	
1	YFAATEKSN	88	96	9	HLA-A*02:01	88	61	291816.6046	6.10E+01	2.92E+05	61	TRUE	TRUE	
1	PFYSNVTGF	61	69	9	HLA-A*02:01	61	61	294516.7504	6.10E+01	2.95E+05	61	TRUE	TRUE	
1	ELKCSVKSF	285	293	9	HLA-A*02:01	285	62	297241.8804	6.20E+01	2.97E+05	62	TRUE	TRUE	
1	LAWNTRNID	421	429	9	HLA-A*02:01	421	62	302071.6724	6.20E+01	3.02E+05	62	TRUE	TRUE	
1	RPFERDISN	449	457	9	HLA-A*02:01	449	62	306273.9079	6.20E+01	3.06E+05	62	TRUE	TRUE	
1	SGDVVRFPN	310	318	9	HLA-A*02:01	310	62	306979.9422	6.20E+01	3.07E+05	62	TRUE	TRUE	
1	DVDLGDISG	1145	1153	9	HLA-A*02:01	1145	62	306979.9422	6.20E+01	3.07E+05	62	TRUE	TRUE	
1	EVFNATKFP	327	335	9	HLA-A*02:01	327	62	307687.6041	6.20E+01	3.08E+05	62	TRUE	TRUE	
1	NFSQILPDP	783	791	9	HLA-A*02:01	783	62	307687.6041	6.20E+01	3.08E+05	62	TRUE	TRUE	
1	VAVLYQDVN	594	602	9	HLA-A*02:01	594	62	309820.3926	6.20E+01	3.10E+05	62	TRUE	TRUE	
1	VEAEVQIDR	969	977	9	HLA-A*02:01	969	62	312687.1254	6.20E+01	3.13E+05	62	TRUE	TRUE	
1	FERDISNVP	451	459	9	HLA-A*02:01	451	62	314130.4236	6.20E+01	3.14E+05	62	TRUE	TRUE	
1	LYVYKGYQP	194	202	9	HLA-A*02:01	194	62	315580.3838	6.20E+01	3.16E+05	62	TRUE	TRUE	
1	GFNTLKPIF	212	220	9	HLA-A*02:01	212	63	319234.6324	6.30E+01	3.19E+05	63	TRUE	TRUE	
1	SFPQAAPHG	1033	1041	9	HLA-A*02:01	1033	63	319970.5442	6.30E+01	3.20E+05	63	TRUE	TRUE	
1	GYHLMSFPQ	1028	1036	9	HLA-A*02:01	1028	63	322188.4741	6.30E+01	3.22E+05	63	TRUE	TRUE	
1	RVDFCGKGY	1021	1029	9	HLA-A*02:01	1021	63	323675.629	6.30E+01	3.24E+05	63	TRUE	TRUE	
1	GAGAALQIP	871	879	9	HLA-A*02:01	871	63	324421.7779	6.30E+01	3.24E+05	63	TRUE	TRUE	
1	TSGSDLDRC	11	19	9	HLA-A*02:01	11	63	324421.7779	6.30E+01	3.24E+05	63	TRUE	TRUE	
1	IQKEIDRLN	1161	1169	9	HLA-A*02:01	1161	63	330453.2291	6.30E+01	3.30E+05	63	TRUE	TRUE	
1	SDAFSLDVS	165	173	9	HLA-A*02:01	165	63	331215.0024	6.30E+01	3.31E+05	63	TRUE	TRUE	
1	DEIFRSDTL	44	52	9	HLA-A*02:01	44	63	331978.5319	6.30E+01	3.32E+05	63	TRUE	TRUE	
1	LHVTYVPSQ	1045	1053	9	HLA-A*02:01	1045	63	332743.8214	6.30E+01	3.33E+05	63	TRUE	TRUE	
1	FNTLKPIFK	213	221	9	HLA-A*02:01	213	63	333510.8752	6.30E+01	3.34E+05	63	TRUE	TRUE	
1	DVVIGIINN	1109	1117	9	HLA-A*02:01	1109	63	335050.2914	6.30E+01	3.35E+05	63	TRUE	TRUE	
1	QALNTLVKQ	939	947	9	HLA-A*02:01	939	63	335822.6621	6.30E+01	3.36E+05	63	TRUE	TRUE	
1	QSVIIINNS	112	120	9	HLA-A*02:01	112	63	338150.4736	6.30E+01	3.38E+05	63	TRUE	TRUE	
1	STALGKLQD	924	932	9	HLA-A*02:01	924	63	340494.4207	6.30E+01	3.40E+05	63	TRUE	TRUE	
1	NVTGFHTIN	65	73	9	HLA-A*02:01	65	63	341279.3414	6.30E+01	3.41E+05	63	TRUE	TRUE	
1	CNFELCDNP	128	136	9	HLA-A*02:01	128	63	342066.0715	6.30E+01	3.42E+05	63	TRUE	TRUE	
1	APGQTGVIA	398	406	9	HLA-A*02:01	398	63	343644.9768	6.30E+01	3.44E+05	63	TRUE	TRUE	
1	IKNQCVNFN	520	528	9	HLA-A*02:01	520	63	346027.01	6.30E+01	3.46E+05	63	TRUE	TRUE	
1	GNYNYKYRY	434	442	9	HLA-A*02:01	434	63	349228.7585	6.30E+01	3.49E+05	63	TRUE	TRUE	
1	RACNFELCD	126	134	9	HLA-A*02:01	126	63	350840.7252	6.30E+01	3.51E+05	63	TRUE	TRUE	
1	MSECVLGQS	1011	1019	9	HLA-A*02:01	1011	64	351649.4966	6.40E+01	3.52E+05	64	TRUE	TRUE	
1	QTSNFRVVP	301	309	9	HLA-A*02:01	301	64	352460.1324	6.40E+01	3.52E+05	64	TRUE	TRUE	
1	IADYNYKLP	405	413	9	HLA-A*02:01	405	64	354903.2693	6.40E+01	3.55E+05	64	TRUE	TRUE	
1	SYECDIPIG	645	653	9	HLA-A*02:01	645	64	355721.4058	6.40E+01	3.56E+05	64	TRUE	TRUE	
1	VRGWVFGST	98	106	9	HLA-A*02:01	98	64	356541.4284	6.40E+01	3.57E+05	64	TRUE	TRUE	
1	CFSNVYADS	378	386	9	HLA-A*02:01	378	64	363170.0284	6.40E+01	3.63E+05	64	TRUE	TRUE	
1	LPPLLTDDM	843	851	9	HLA-A*02:01	843	64	364846.3451	6.40E+01	3.65E+05	64	TRUE	TRUE	
1	GPKLSTDLI	512	520	9	HLA-A*02:01	512	64	373344.7082	6.40E+01	3.73E+05	64	TRUE	TRUE	
1	IWGTSAAAY	244	252	9	HLA-A*02:01	244	64	375932.6101	6.40E+01	3.76E+05	64	TRUE	TRUE	
1	SGNCDVVIG	1105	1113	9	HLA-A*02:01	1105	65	378538.4504	6.50E+01	3.79E+05	65	TRUE	TRUE	
1	GWVFGSTMN	100	108	9	HLA-A*02:01	100	65	378538.4504	6.50E+01	3.79E+05	65	TRUE	TRUE	
1	EIFRSDTLY	45	53	9	HLA-A*02:01	45	65	379411.0717	6.50E+01	3.79E+05	65	TRUE	TRUE	
1	LNAPATVCG	504	512	9	HLA-A*02:01	504	65	382041.0236	6.50E+01	3.82E+05	65	TRUE	TRUE	
1	DINARDLIC	825	833	9	HLA-A*02:01	825	65	391841.1118	6.50E+01	3.92E+05	65	TRUE	TRUE	
1	SDFTDSVRD	556	564	9	HLA-A*02:01	556	65	394557.2246	6.50E+01	3.95E+05	65	TRUE	TRUE	
1	EAEVQIDRL	970	978	9	HLA-A*02:01	970	65	396378.418	6.50E+01	3.96E+05	65	TRUE	TRUE	
1	MKQYGECLG	816	824	9	HLA-A*02:01	816	65	398208.018	6.50E+01	3.98E+05	65	TRUE	TRUE	
1	RDLICAQKF	829	837	9	HLA-A*02:01	829	65	398208.0176	6.50E+01	3.98E+05	65	TRUE	TRUE	
1	CSCGSCCKF	1230	1238	9	HLA-A*02:01	1230	65	399125.9819	6.50E+01	3.99E+05	65	TRUE	TRUE	
1	CTQLNRALS	742	750	9	HLA-A*02:01	742	65	400968.2638	6.50E+01	4.01E+05	65	TRUE	TRUE	
1	YDENGTITD	266	274	9	HLA-A*02:01	266	65	402819.0492	6.50E+01	4.03E+05	65	TRUE	TRUE	
1	GAGICASYH	653	661	9	HLA-A*02:01	653	65	403747.643	6.50E+01	4.04E+05	65	TRUE	TRUE	
1	KVEAEVQID	968	976	9	HLA-A*02:01	968	65	406546.2879	6.50E+01	4.07E+05	65	TRUE	TRUE	
1	QTGVIADYN	401	409	9	HLA-A*02:01	401	65	407483.4739	6.50E+01	4.07E+05	65	TRUE	TRUE	
1	TTFMLKYDE	260	268	9	HLA-A*02:01	260	65	412201.9102	6.50E+01	4.12E+05	65	TRUE	TRUE	
1	DLQELGKYE	1181	1189	9	HLA-A*02:01	1181	66	419865.3158	6.60E+01	4.20E+05	66	TRUE	TRUE	
1	KFPSVYAWE	333	341	9	HLA-A*02:01	333	66	424727.1271	6.60E+01	4.25E+05	66	TRUE	TRUE	
1	LPSGFNTLK	209	217	9	HLA-A*02:01	209	66	429645.2354	6.60E+01	4.30E+05	66	TRUE	TRUE	
1	CTDVSTAIH	603	611	9	HLA-A*02:01	603	66	436626.4089	6.60E+01	4.37E+05	66	TRUE	TRUE	
1	TTGIGYQPY	486	494	9	HLA-A*02:01	486	66	436626.409	6.60E+01	4.37E+05	66	TRUE	TRUE	
1	PFQQFGRDV	547	555	9	HLA-A*02:01	547	66	437632.9367	6.60E+01	4.38E+05	66	TRUE	TRUE	
1	NFTTAPAIC	1056	1064	9	HLA-A*02:01	1056	66	440666.4633	6.60E+01	4.41E+05	66	TRUE	TRUE	
1	KDGFLYVYK	190	198	9	HLA-A*02:01	190	66	443721.0173	6.60E+01	4.44E+05	66	TRUE	TRUE	
1	VVRFPNITN	313	321	9	HLA-A*02:01	313	66	443721.0173	6.60E+01	4.44E+05	66	TRUE	TRUE	
1	FNGIGVTQN	888	896	9	HLA-A*02:01	888	66	444743.8999	6.60E+01	4.45E+05	66	TRUE	TRUE	
1	GFYTTTGIG	482	490	9	HLA-A*02:01	482	66	444743.8999	6.60E+01	4.45E+05	66	TRUE	TRUE	
1	RSDTLYLTQ	48	56	9	HLA-A*02:01	48	67	445769.1405	6.70E+01	4.46E+05	67	TRUE	TRUE	
1	TSPDVDLGD	1142	1150	9	HLA-A*02:01	1142	67	445769.1405	6.70E+01	4.46E+05	67	TRUE	TRUE	
1	QESLTTTST	917	925	9	HLA-A*02:01	917	67	447826.7173	6.70E+01	4.48E+05	67	TRUE	TRUE	
1	YENQKQIAN	899	907	9	HLA-A*02:01	899	67	450930.9038	6.70E+01	4.51E+05	67	TRUE	TRUE	
1	PFAMQMAYR	879	887	9	HLA-A*02:01	879	67	458257.9416	6.70E+01	4.58E+05	67	TRUE	TRUE	
1	AYFPREGVF	1069	1077	9	HLA-A*02:01	1069	67	460373.1641	6.70E+01	4.60E+05	67	TRUE	TRUE	
1	PDDFMGCVL	413	421	9	HLA-A*02:01	413	67	465704.0341	6.70E+01	4.66E+05	67	TRUE	TRUE	
1	ERNFTTAPA	1054	1062	9	HLA-A*02:01	1054	67	467853.6263	6.70E+01	4.68E+05	67	TRUE	TRUE	
1	MYICGDSTE	722	730	9	HLA-A*02:01	722	68	487651.9891	6.80E+01	4.88E+05	68	TRUE	TRUE	
1	PAWRIYSTG	617	625	9	HLA-A*02:01	617	68	495575.6965	6.80E+01	4.96E+05	68	TRUE	TRUE	
1	TTTSTALGK	921	929	9	HLA-A*02:01	921	68	496718.1164	6.80E+01	4.97E+05	68	TRUE	TRUE	
1	RKKISNCVA	342	350	9	HLA-A*02:01	342	68	503628.1538	6.80E+01	5.04E+05	68	TRUE	TRUE	
1	LYENQKQIA	898	906	9	HLA-A*02:01	898	68	504789.1366	6.80E+01	5.05E+05	68	TRUE	TRUE	
1	DVSEKSGNF	171	179	9	HLA-A*02:01	171	68	505952.7957	6.80E+01	5.06E+05	68	TRUE	TRUE	
1	ITGRLQSLQ	979	987	9	HLA-A*02:01	979	68	511811.453	6.80E+01	5.12E+05	68	TRUE	TRUE	
1	NTRNIDATS	424	432	9	HLA-A*02:01	424	68	514173.8673	6.80E+01	5.14E+05	68	TRUE	TRUE	
1	YRVVVLSFE	494	502	9	HLA-A*02:01	494	68	515359.1605	6.80E+01	5.15E+05	68	TRUE	TRUE	
1	FHTINHTFG	69	77	9	HLA-A*02:01	69	68	517737.9503	6.80E+01	5.18E+05	68	TRUE	TRUE	
1	NASVVNIQK	1155	1163	9	HLA-A*02:01	1155	68	518931.4595	6.80E+01	5.19E+05	68	TRUE	TRUE	

seq #	peptide	start	end	peptide length	allele	peptide index	median binding	smm IC50	smm percentil	legacy IC50	legacy rank	scores equal	percentiles equal
1	NSTFFSTFK	357	365	9	HLA-A*02:01	357	69	523733.0732	6.90E+01	5.24E+05	69	TRUE	TRUE
1	IDVVRDLPS	203	211	9	HLA-A*02:01	203	69	524940.4026	6.90E+01	5.25E+05	69	TRUE	TRUE
1	TAGWTFGAG	865	873	9	HLA-A*02:01	865	69	526150.5152	6.90E+01	5.26E+05	69	TRUE	TRUE
1	NARDLICAQ	827	835	9	HLA-A*02:01	827	69	528579.1156	6.90E+01	5.29E+05	69	TRUE	TRUE
1	SGTATAGWT	861	869	9	HLA-A*02:01	861	69	529797.6163	6.90E+01	5.30E+05	69	TRUE	TRUE
1	KYEQYIKWP	1187	1195	9	HLA-A*02:01	1187	69	531018.926	6.90E+01	5.31E+05	69	TRUE	TRUE
1	GKAYFPREG	1067	1075	9	HLA-A*02:01	1067	69	532243.051	6.90E+01	5.32E+05	69	TRUE	TRUE
1	HEGKAYFPR	1065	1073	9	HLA-A*02:01	1065	69	535932.3836	6.90E+01	5.36E+05	69	TRUE	TRUE
1	TSQKSIVAY	669	677	9	HLA-A*02:01	669	69	535932.3836	6.90E+01	5.36E+05	69	TRUE	TRUE
1	NVPFSPDGK	457	465	9	HLA-A*02:01	457	69	543387.946	6.90E+01	5.43E+05	69	TRUE	TRUE
1	WNTRNIDAT	423	431	9	HLA-A*02:01	423	69	543387.9457	6.90E+01	5.43E+05	69	TRUE	TRUE
1	AGFMKQYGE	813	821	9	HLA-A*02:01	813	69	545896.1105	6.90E+01	5.46E+05	69	TRUE	TRUE
1	EHVDTSYEC	640	648	9	HLA-A*02:01	640	69	545896.1105	6.90E+01	5.46E+05	69	TRUE	TRUE
1	AWRIYSTGN	618	626	9	HLA-A*02:01	618	69	550947.225	6.90E+01	5.51E+05	69	TRUE	TRUE
1	LNRALSGIA	745	753	9	HLA-A*02:01	745	69	550947.225	6.90E+01	5.51E+05	69	TRUE	TRUE
1	KHLREFVFK	180	188	9	HLA-A*02:01	180	69	554766.2088	6.90E+01	5.55E+05	69	TRUE	TRUE
1	YGVSATKLN	367	375	9	HLA-A*02:01	367	69	554766.2088	6.90E+01	5.55E+05	69	TRUE	TRUE
1	APNYTQHTS	27	35	9	HLA-A*02:01	27	69	556045.077	6.90E+01	5.56E+05	69	TRUE	TRUE
1	LCCMTSCCS	1216	1224	9	HLA-A*02:01	1216	69	561190.0987	6.90E+01	5.61E+05	69	TRUE	TRUE
1	NVLYENQKQ	896	904	9	HLA-A*02:01	896	69	563780.435	6.90E+01	5.64E+05	69	TRUE	TRUE
1	LKPTTFMLK	257	265	9	HLA-A*02:01	257	70	571623.402	7.00E+01	5.72E+05	70	TRUE	TRUE
1	SPDGKPCTP	461	469	9	HLA-A*02:01	461	70	571623.402	7.00E+01	5.72E+05	70	TRUE	TRUE
1	RDVSDFTDS	553	561	9	HLA-A*02:01	553	70	582250.6741	7.00E+01	5.82E+05	70	TRUE	TRUE
1	SDTLYLTQD	49	57	9	HLA-A*02:01	49	70	583592.9005	7.00E+01	5.84E+05	70	TRUE	TRUE
1	TSCCSCLKG	1220	1228	9	HLA-A*02:01	1220	70	583592.9005	7.00E+01	5.84E+05	70	TRUE	TRUE
1	VSLLRSTSQ	663	671	9	HLA-A*02:01	663	70	584938.2211	7.00E+01	5.85E+05	70	TRUE	TRUE
1	GYQPIDVVR	199	207	9	HLA-A*02:01	199	70	586286.6429	7.00E+01	5.86E+05	70	TRUE	TRUE
1	ISNVPFSPD	455	463	9	HLA-A*02:01	455	70	590350.5878	7.00E+01	5.90E+05	70	TRUE	TRUE
1	NQKQIANQF	901	909	9	HLA-A*02:01	901	70	604101.619	7.00E+01	6.04E+05	70	TRUE	TRUE
1	VIPFKDGIY	80	88	9	HLA-A*02:01	80	70	608289.0519	7.00E+01	6.08E+05	70	TRUE	TRUE
1	VDCNMYICG	718	726	9	HLA-A*02:01	718	71	611096.787	7.10E+01	6.11E+05	71	TRUE	TRUE
1	GFLYVYKGY	192	200	9	HLA-A*02:01	192	71	623892.8378	7.10E+01	6.24E+05	71	TRUE	TRUE
1	SGINASVVN	1152	1160	9	HLA-A*02:01	1152	71	625331.0593	7.10E+01	6.25E+05	71	TRUE	TRUE
1	ATEKSNVVR	91	99	9	HLA-A*02:01	91	71	629665.647	7.10E+01	6.30E+05	71	TRUE	TRUE
1	TFFSTFKCY	359	367	9	HLA-A*02:01	359	71	631117.1763	7.10E+01	6.31E+05	71	TRUE	TRUE
1	EKSGNFKHL	174	182	9	HLA-A*02:01	174	71	634030.2809	7.10E+01	6.34E+05	71	TRUE	TRUE
1	GSCCKFDED	1233	1241	9	HLA-A*02:01	1233	71	642850.5207	7.10E+01	6.43E+05	71	TRUE	TRUE
1	KPTTFMLKY	258	266	9	HLA-A*02:01	258	71	644332.4442	7.10E+01	6.44E+05	71	TRUE	TRUE
1	SVKSFEIDK	289	297	9	HLA-A*02:01	289	71	647306.5476	7.10E+01	6.47E+05	71	TRUE	TRUE
1	FGEVFNATK	325	333	9	HLA-A*02:01	325	71	650294.3789	7.10E+01	6.50E+05	71	TRUE	TRUE
1	PKTSEILDI	565	573	9	HLA-A*02:01	565	71	650294.3789	7.10E+01	6.50E+05	71	TRUE	TRUE
1	YDPLQPELD	1120	1128	9	HLA-A*02:01	1120	71	650294.3789	7.10E+01	6.50E+05	71	TRUE	TRUE
1	DAVDCSQNP	274	282	9	HLA-A*02:01	274	71	654802.0042	7.10E+01	6.55E+05	71	TRUE	TRUE
1	EVQIDRLIT	972	980	9	HLA-A*02:01	972	71	659340.875	7.10E+01	6.59E+05	71	TRUE	TRUE
1	WPLNDYGFY	476	484	9	HLA-A*02:01	476	72	670054.3022	7.20E+01	6.70E+05	72	TRUE	TRUE
1	SNVPFSPDG	456	464	9	HLA-A*02:01	456	72	674698.8967	7.20E+01	6.75E+05	72	TRUE	TRUE
1	IGVTQNVLY	891	899	9	HLA-A*02:01	891	72	674698.8967	7.20E+01	6.75E+05	72	TRUE	TRUE
1	DRCTTFDDV	17	25	9	HLA-A*02:01	17	72	679375.6861	7.20E+01	6.79E+05	72	TRUE	TRUE
1	TQNVLYENQ	894	902	9	HLA-A*02:01	894	72	680941.8088	7.20E+01	6.81E+05	72	TRUE	TRUE
1	AVSKPMGTQ	139	147	9	HLA-A*02:01	139	72	684084.8933	7.20E+01	6.84E+05	72	TRUE	TRUE
1	NNKSQSVII	108	116	9	HLA-A*02:01	108	72	684084.8933	7.20E+01	6.84E+05	72	TRUE	TRUE
1	DENGTITDA	267	275	9	HLA-A*02:01	267	72	695200.3787	7.20E+01	6.95E+05	72	TRUE	TRUE
1	LNCYWPLND	472	480	9	HLA-A*02:01	472	72	708125.1187	7.20E+01	7.08E+05	72	TRUE	TRUE
1	ATKLNDLCF	371	379	9	HLA-A*02:01	371	72	708125.1187	7.20E+01	7.08E+05	72	TRUE	TRUE
1	VSDFTDSVR	555	563	9	HLA-A*02:01	555	72	709757.5157	7.20E+01	7.10E+05	72	TRUE	TRUE
1	NTFVSGNCD	1101	1109	9	HLA-A*02:01	1101	72	726289.892	7.20E+01	7.26E+05	72	TRUE	TRUE
1	MAKTSVDCN	713	721	9	HLA-A*02:01	713	73	736393.5909	7.30E+01	7.36E+05	73	TRUE	TRUE
1	IRAAEIRAS	995	1003	9	HLA-A*02:01	995	73	746637.8462	7.30E+01	7.47E+05	73	TRUE	TRUE
1	NDLCFSNVY	375	383	9	HLA-A*02:01	375	73	746637.8462	7.30E+01	7.47E+05	73	TRUE	TRUE
1	NPFFAVSKP	135	143	9	HLA-A*02:01	135	73	751813.2925	7.30E+01	7.52E+05	73	TRUE	TRUE
1	PPLLTDDMI	844	852	9	HLA-A*02:01	844	73	755283.5049	7.30E+01	7.55E+05	73	TRUE	TRUE
1	KRVDFCGKG	1020	1028	9	HLA-A*02:01	1020	73	762272.0572	7.30E+01	7.62E+05	73	TRUE	TRUE
1	NGTSWFITQ	1080	1088	9	HLA-A*02:01	1080	73	776443.7528	7.30E+01	7.76E+05	73	TRUE	TRUE
1	NFNGLTGTG	528	536	9	HLA-A*02:01	528	73	778233.6405	7.30E+01	7.78E+05	73	TRUE	TRUE
1	KRFQPFQQF	543	551	9	HLA-A*02:01	543	74	781825.8038	7.40E+01	7.82E+05	74	TRUE	TRUE
1	ADQLTPAWR	612	620	9	HLA-A*02:01	612	74	794529.4511	7.40E+01	7.95E+05	74	TRUE	TRUE
1	VSKPMGTQT	140	148	9	HLA-A*02:01	140	74	796361.0307	7.40E+01	7.96E+05	74	TRUE	TRUE
1	VPSQERNFT	1050	1058	9	HLA-A*02:01	1050	74	800036.8662	7.40E+01	8.00E+05	74	TRUE	TRUE
1	CNMYICGDS	720	728	9	HLA-A*02:01	720	74	814910.662	7.40E+01	8.15E+05	74	TRUE	TRUE
1	IGIINNTVY	1112	1120	9	HLA-A*02:01	1112	74	828151.895	7.40E+01	8.28E+05	74	TRUE	TRUE
1	TSSMRGVYY	34	42	9	HLA-A*02:01	34	74	830060.9824	7.40E+01	8.30E+05	74	TRUE	TRUE
1	APATVCGPK	506	514	9	HLA-A*02:01	506	74	835814.6902	7.40E+01	8.36E+05	74	TRUE	TRUE
1	PIDVVRDLP	202	210	9	HLA-A*02:01	202	74	837741.4421	7.40E+01	8.38E+05	74	TRUE	TRUE
1	LCPFGEVFN	322	330	9	HLA-A*02:01	322	74	845492.9682	7.40E+01	8.45E+05	74	TRUE	TRUE
1	VFGSTMNNK	102	110	9	HLA-A*02:01	102	74	845492.9682	7.40E+01	8.45E+05	74	TRUE	TRUE
1	LSTDLIKNQ	515	523	9	HLA-A*02:01	515	74	845492.9682	7.40E+01	8.45E+05	74	TRUE	TRUE
1	NATKFPSVY	330	338	9	HLA-A*02:01	330	75	853316.218	7.50E+01	8.53E+05	75	TRUE	TRUE
1	VYKGYQPID	196	204	9	HLA-A*02:01	196	75	855283.3153	7.50E+01	8.55E+05	75	TRUE	TRUE
1	TRNIDATST	425	433	9	HLA-A*02:01	425	75	859231.1238	7.50E+01	8.59E+05	75	TRUE	TRUE
1	VKQLSSNFG	945	953	9	HLA-A*02:01	945	75	867181.4915	7.50E+01	8.67E+05	75	TRUE	TRUE
1	TFEYISDAF	160	168	9	HLA-A*02:01	160	75	871184.2194	7.50E+01	8.71E+05	75	TRUE	TRUE
1	VFAQVKQMY	763	771	9	HLA-A*02:01	763	75	877222.98	7.50E+01	8.77E+05	75	TRUE	TRUE
1	LPDPLKPTK	788	796	9	HLA-A*02:01	788	75	881272.0572	7.50E+01	8.81E+05	75	TRUE	TRUE
1	TFGAGAALQ	869	877	9	HLA-A*02:01	869	75	883303.5991	7.50E+01	8.83E+05	75	TRUE	TRUE
1	LRHGKLRPF	443	451	9	HLA-A*02:01	443	75	895591.5762	7.50E+01	8.96E+05	75	TRUE	TRUE
1	MPVSMAKTS	709	717	9	HLA-A*02:01	709	75	895591.5762	7.50E+01	8.96E+05	75	TRUE	TRUE
1	TDNTFVSGN	1099	1107	9	HLA-A*02:01	1099	75	901799.52	7.50E+01	9.02E+05	75	TRUE	TRUE
1	DYSVLYNST	351	359	9	HLA-A*02:01	351	75	905962.0379	7.50E+01	9.06E+05	75	TRUE	TRUE
1	PNITNLCPF	317	325	9	HLA-A*02:01	317	75	908050.496	7.50E+01	9.08E+05	75	TRUE	TRUE
1	HVTYVPSQE	1046	1054	9	HLA-A*02:01	1046	75	910143.7686	7.50E+01	9.10E+05	75	TRUE	TRUE
1	TDSVRDPKT	559	567	9	HLA-A*02:01	559	75	910143.7686	7.50E+01	9.10E+05	75	TRUE	TRUE
1	SNVVRGWVF	95	103	9	HLA-A*02:01	95	75	914344.8014	7.50E+01	9.14E+05	75	TRUE	TRUE
1	GKLQDVVNQ	928	936	9	HLA-A*02:01	928	75	916452.5838	7.50E+01	9.16E+05	75	TRUE	TRUE
1	QDVNCTDVS	599	607	9	HLA-A*02:01	599	76	931343.7404	7.60E+01	9.31E+05	76	TRUE	TRUE
1	ANLAATKMS	1004	1012	9	HLA-A*02:01	1004	76	931343.74	7.60E+01	9.31E+05	76	TRUE	TRUE
1	ACSCGSCCK	1229	1237	9	HLA-A*02:01	1229	76	933490.7095	7.60E+01	9.33E+05	76	TRUE	TRUE
1	GDDVRQIAP	391	399	9	HLA-A*02:01	391	76	933490.7095	7.60E+01	9.33E+05	76	TRUE	TRUE
1	DVQAPNYTQ	24	32	9	HLA-A*02:01	24	76	937799.5068	7.60E+01	9.38E+05	76	TRUE	TRUE
1	RFNGIGVTQ	887	895	9	HLA-A*02:01	887	76	937799.5068	7.60E+01	9.38E+05	76	TRUE	TRUE
1	AICHEGKAY	1062	1070	9	HLA-A*02:01	1062	76	942128.1926	7.60E+01	9.42E+05	76	TRUE	TRUE
1	SSEVAVLYQ	591	599	9	HLA-A*02:01	591	76	955234.5013	7.60E+01	9.55E+05	76	TRUE	TRUE
1	VVNIQKEID	1158	1166	9	HLA-A*02:01	1158	76	955234.5013	7.60E+01	9.55E+05	76	TRUE	TRUE

seq #	peptide	start	end	peptide length	allele	peptide index	median binding	smm IC50	smm percentil	legacy IC50	legacy rank	scores equal	percentiles equal	
1	KNLNESLID	1173	1181	9	HLA-A*02:01	1173	76	959643.6635	7.60E+01	9.60E+05	76	TRUE	TRUE	
1	NHTSPDVDL	1140	1148	9	HLA-A*02:01	1140	76	975236.6186	7.60E+01	9.75E+05	76	TRUE	TRUE	
1	NFELCDNPF	129	137	9	HLA-A*02:01	129	76	997952.798	7.60E+01	9.98E+05	76	TRUE	TRUE	
1	PSGDVVRFP	309	317	9	HLA-A*02:01	309	76	1002559.138	7.60E+01	1.00E+06	76	TRUE	TRUE	
1	EGVFVFNGT	1074	1082	9	HLA-A*02:01	1074	76	1016506.125	7.60E+01	1.02E+06	76	TRUE	TRUE	
1	DIPIGAGIC	649	657	9	HLA-A*02:01	649	76	1023552.209	7.60E+01	1.02E+06	76	TRUE	TRUE	
1	PAQDIWGTS	240	248	9	HLA-A*02:01	240	77	1037791.239	7.70E+01	1.04E+06	77	TRUE	TRUE	
1	PLKPTKRSF	791	799	9	HLA-A*02:01	791	77	1044984.864	7.70E+01	1.04E+06	77	TRUE	TRUE	
1	TYVTQQLIR	988	996	9	HLA-A*02:01	988	77	1059522.052	7.70E+01	1.06E+06	77	TRUE	TRUE	
1	PVLKGVKLH	1245	1253	9	HLA-A*02:01	1245	77	1066866.308	7.70E+01	1.07E+06	77	TRUE	TRUE	
1	TPTLKYFGG	773	781	9	HLA-A*02:01	773	77	1101818.348	7.70E+01	1.10E+06	77	TRUE	TRUE	
1	INASVVNIQ	1154	1162	9	HLA-A*02:01	1154	77	1101818.348	7.70E+01	1.10E+06	77	TRUE	TRUE	
1	VVKGDDVRQ	388	396	9	HLA-A*02:01	388	78	1124889.855	7.80E+01	1.12E+06	78	TRUE	TRUE	
1	LTPSSKRFQ	538	546	9	HLA-A*02:01	538	78	1130082.111	7.80E+01	1.13E+06	78	TRUE	TRUE	
1	TSTGNYNYK	431	439	9	HLA-A*02:01	431	78	1143167.845	7.80E+01	1.14E+06	78	TRUE	TRUE	
1	VKSFEIDKG	290	298	9	HLA-A*02:01	290	78	1145803.119	7.80E+01	1.15E+06	78	TRUE	TRUE	
1	ADAGFMKQY	811	819	9	HLA-A*02:01	811	78	1151091.906	7.80E+01	1.15E+06	78	TRUE	TRUE	
1	DYGFYTTTG	480	488	9	HLA-A*02:01	480	78	1164420.922	7.80E+01	1.16E+06	78	TRUE	TRUE	
1	DRNTREVFA	757	765	9	HLA-A*02:01	757	78	1164420.922	7.80E+01	1.16E+06	78	TRUE	TRUE	
1	VDFCGKGYH	1022	1030	9	HLA-A*02:01	1022	78	1175195.176	7.80E+01	1.18E+06	78	TRUE	TRUE	
1	TQQLIRAAE	991	999	9	HLA-A*02:01	991	78	1180619.63	7.80E+01	1.18E+06	78	TRUE	TRUE	
1	QFGRDVSDF	550	558	9	HLA-A*02:01	550	78	1183341.24	7.80E+01	1.18E+06	78	TRUE	TRUE	
1	AYTMSLGAD	676	684	9	HLA-A*02:01	676	78	1194290.561	7.80E+01	1.19E+06	78	TRUE	TRUE	
1	DDVQAPNYT	23	31	9	HLA-A*02:01	23	78	1194290.561	7.80E+01	1.19E+06	78	TRUE	TRUE	
1	NFNFNGLTG	526	534	9	HLA-A*02:01	526	78	1197043.685	7.80E+01	1.20E+06	78	TRUE	TRUE	
1	QSLQTYVTQ	984	992	9	HLA-A*02:01	984	78	1199803.156	7.80E+01	1.20E+06	78	TRUE	TRUE	
1	GNPVIPFKD	77	85	9	HLA-A*02:01	77	78	1208119.794	7.80E+01	1.21E+06	78	TRUE	TRUE	
1	VCGPKLSTD	510	518	9	HLA-A*02:01	510	78	1216494.081	7.80E+01	1.22E+06	78	TRUE	TRUE	
1	GAEHVDTSY	638	646	9	HLA-A*02:01	638	79	1288575.886	7.90E+01	1.29E+06	79	TRUE	TRUE	
1	ERKKISNCV	341	349	9	HLA-A*02:01	341	79	1306501.763	7.90E+01	1.31E+06	79	TRUE	TRUE	
1	GEVFNATKF	326	334	9	HLA-A*02:01	326	79	1358657.53	7.90E+01	1.36E+06	79	TRUE	TRUE	
1	EELDKYFKN	1132	1140	9	HLA-A*02:01	1132	80	1383916.859	8.00E+01	1.38E+06	80	TRUE	TRUE	
1	SRLDKVEAE	964	972	9	HLA-A*02:01	964	80	1406403.699	8.00E+01	1.41E+06	80	TRUE	TRUE	
1	PPALNCYWP	469	477	9	HLA-A*02:01	469	80	1422689.086	8.00E+01	1.42E+06	80	TRUE	TRUE	
1	NPLAELKCS	281	289	9	HLA-A*02:01	281	80	1452479.461	8.00E+01	1.45E+06	80	TRUE	TRUE	
1	EPVLKGVKL	1244	1252	9	HLA-A*02:01	1244	80	1462547.568	8.00E+01	1.46E+06	80	TRUE	TRUE	
1	LDKYFKNHT	1134	1142	9	HLA-A*02:01	1134	80	1465919.088	8.00E+01	1.47E+06	80	TRUE	TRUE	
1	SCGSCCKFD	1231	1239	9	HLA-A*02:01	1231	80	1472685.463	8.00E+01	1.47E+06	80	TRUE	TRUE	
1	DKYFKNHTS	1135	1143	9	HLA-A*02:01	1135	80	1486312.054	8.00E+01	1.49E+06	80	TRUE	TRUE	
1	GRDVSDFTD	552	560	9	HLA-A*02:01	552	81	1524438.821	8.10E+01	1.52E+06	81	TRUE	TRUE	
1	SFKEELDKY	1129	1137	9	HLA-A*02:01	1129	81	1531475.311	8.10E+01	1.53E+06	81	TRUE	TRUE	
1	SSKRFQPFQ	541	549	9	HLA-A*02:01	541	81	1531475.311	8.10E+01	1.53E+06	81	TRUE	TRUE	
1	KPCTPPALN	465	473	9	HLA-A*02:01	465	81	1538544.28	8.10E+01	1.54E+06	81	TRUE	TRUE	
1	NFSISITTE	699	707	9	HLA-A*02:01	699	81	1538544.28	8.10E+01	1.54E+06	81	TRUE	TRUE	
1	CSVKSFEID	288	296	9	HLA-A*02:01	288	81	1552780.255	8.10E+01	1.55E+06	81	TRUE	TRUE	
1	PSSKRFQPF	540	548	9	HLA-A*02:01	540	81	1563543.614	8.10E+01	1.56E+06	81	TRUE	TRUE	
1	CSCLKGACS	1223	1231	9	HLA-A*02:01	1223	81	1567147.954	8.10E+01	1.57E+06	81	TRUE	TRUE	
1	GRLQSLQTY	981	989	9	HLA-A*02:01	981	81	1567147.954	8.10E+01	1.57E+06	81	TRUE	TRUE	
1	TPSSKRFQP	539	547	9	HLA-A*02:01	539	81	1585294.672	8.10E+01	1.59E+06	81	TRUE	TRUE	
1	IDLQELGKY	1180	1188	9	HLA-A*02:01	1180	81	1592612.06	8.10E+01	1.59E+06	81	TRUE	TRUE	
1	NYKLPDDFM	409	417	9	HLA-A*02:01	409	81	1603651.519	8.10E+01	1.60E+06	81	TRUE	TRUE	
1	TSEILDISP	567	575	9	HLA-A*02:01	567	81	1633465.627	8.10E+01	1.63E+06	81	TRUE	TRUE	
1	RYLRHGKLR	441	449	9	HLA-A*02:01	441	81	1644788.27	8.10E+01	1.64E+06	81	TRUE	TRUE	
1	ECLGDINAR	821	829	9	HLA-A*02:01	821	82	1660007.308	8.20E+01	1.66E+06	82	TRUE	TRUE	
1	TECANLLLQ	729	737	9	HLA-A*02:01	729	82	1671513.929	8.20E+01	1.67E+06	82	TRUE	TRUE	
1	ARDLICAQK	828	836	9	HLA-A*02:01	828	82	1710448.49	8.20E+01	1.71E+06	82	TRUE	TRUE	
1	CHEGKAYFP	1064	1072	9	HLA-A*02:01	1064	82	1710448.49	8.20E+01	1.71E+06	82	TRUE	TRUE	
1	IDATSTGNY	428	436	9	HLA-A*02:01	428	82	1718343.562	8.20E+01	1.72E+06	82	TRUE	TRUE	
1	ADSFVVKGD	384	392	9	HLA-A*02:01	384	82	1742248.102	8.20E+01	1.74E+06	82	TRUE	TRUE	
1	TGVIADYNY	402	410	9	HLA-A*02:01	402	82	1774638.912	8.20E+01	1.77E+06	82	TRUE	TRUE	
1	VNQNAQALN	934	942	9	HLA-A*02:01	934	83	1791059.444	8.30E+01	1.79E+06	83	TRUE	TRUE	
1	CGKGYHLMS	1025	1033	9	HLA-A*02:01	1025	83	1807631.913	8.30E+01	1.81E+06	83	TRUE	TRUE	
1	DAFSLDVSE	166	174	9	HLA-A*02:01	166	83	1837003.569	8.30E+01	1.84E+06	83	TRUE	TRUE	
1	LDRCTTFDD	16	24	9	HLA-A*02:01	16	83	1849737.069	8.30E+01	1.85E+06	83	TRUE	TRUE	
1	DCNMYICGD	719	727	9	HLA-A*02:01	719	83	1854001.153	8.30E+01	1.85E+06	83	TRUE	TRUE	
1	WERKKISNC	340	348	9	HLA-A*02:01	340	83	1871156.015	8.30E+01	1.87E+06	83	TRUE	TRUE	
1	VSVITPGTN	581	589	9	HLA-A*02:01	581	83	1871156.015	8.30E+01	1.87E+06	83	TRUE	TRUE	
1	QSKRVDFCG	1018	1026	9	HLA-A*02:01	1018	83	1875469.475	8.30E+01	1.88E+06	83	TRUE	TRUE	
1	SVRDPKTSE	561	569	9	HLA-A*02:01	561	83	1901559.854	8.30E+01	1.90E+06	83	TRUE	TRUE	
1	IDKGIYQTS	295	303	9	HLA-A*02:01	295	83	1936912.491	8.30E+01	1.94E+06	83	TRUE	TRUE	
1	NKVTLADAG	806	814	9	HLA-A*02:01	806	83	1945852.873	8.30E+01	1.95E+06	83	TRUE	TRUE	
1	NNSTNVVIR	118	126	9	HLA-A*02:01	118	83	1954834.522	8.30E+01	1.95E+06	83	TRUE	TRUE	
1	LNDILSRLD	959	967	9	HLA-A*02:01	959	84	2009601.749	8.40E+01	2.01E+06	84	TRUE	TRUE	
1	QAGCLIGAE	632	640	9	HLA-A*02:01	632	84	2014234.359	8.40E+01	2.01E+06	84	TRUE	TRUE	
1	RNTREVFAQ	758	766	9	HLA-A*02:01	758	84	2018877.649	8.40E+01	2.02E+06	84	TRUE	TRUE	
1	CCSCLKGAC	1222	1230	9	HLA-A*02:01	1222	84	2032871.84	8.40E+01	2.03E+06	84	TRUE	TRUE	
1	CGDSTECAN	725	733	9	HLA-A*02:01	725	84	2032871.84	8.40E+01	2.03E+06	84	TRUE	TRUE	
1	TYVPSQERN	1048	1056	9	HLA-A*02:01	1048	84	2075439.127	8.40E+01	2.08E+06	84	TRUE	TRUE	
1	APAICHEGK	1060	1068	9	HLA-A*02:01	1060	84	2089825.384	8.40E+01	2.09E+06	84	TRUE	TRUE	
1	TGRLQSLQT	980	988	9	HLA-A*02:01	980	84	2158291.001	8.40E+01	2.16E+06	84	TRUE	TRUE	
1	GCVLAWNTR	418	426	9	HLA-A*02:01	418	85	2193360.409	8.50E+01	2.19E+06	85	TRUE	TRUE	
1	SGIAAEQDR	750	758	9	HLA-A*02:01	750	85	2213655.323	8.50E+01	2.21E+06	85	TRUE	TRUE	
1	VRQIAPGQT	394	402	9	HLA-A*02:01	394	85	2234138.023	8.50E+01	2.23E+06	85	TRUE	TRUE	
1	PTTFMLKYD	259	267	9	HLA-A*02:01	259	85	2249624.328	8.50E+01	2.25E+06	85	TRUE	TRUE	
1	LSGIAAEQD	749	757	9	HLA-A*02:01	749	85	2254810.248	8.50E+01	2.25E+06	85	TRUE	TRUE	
1	NCYWPLNDY	473	481	9	HLA-A*02:01	473	85	2270439.846	8.50E+01	2.27E+06	85	TRUE	TRUE	
1	MYKTPTLKY	770	778	9	HLA-A*02:01	770	85	2280919.72	8.50E+01	2.28E+06	85	TRUE	TRUE	
1	TDAVDCSQN	273	281	9	HLA-A*02:01	273	85	2302024.81	8.50E+01	2.30E+06	85	TRUE	TRUE	
1	DTSYECDIP	643	651	9	HLA-A*02:01	643	85	2302024.81	8.50E+01	2.30E+06	85	TRUE	TRUE	
1	HTSSMRGVY	33	41	9	HLA-A*02:01	33	85	2323325.184	8.50E+01	2.32E+06	85	TRUE	TRUE	
1	KEELDKYFK	1131	1139	9	HLA-A*02:01	1131	85	2382922.95	8.50E+01	2.38E+06	85	TRUE	TRUE	
1	HGKLRPFER	445	453	9	HLA-A*02:01	445	85	2410515.897	8.50E+01	2.41E+06	85	TRUE	TRUE	
1	LDSFKEELD	1127	1135	9	HLA-A*02:01	1127	85	2427224.795	8.50E+01	2.43E+06	85	TRUE	TRUE	
1	REFVFKNKD	183	191	9	HLA-A*02:01	183	85	2438428.356	8.50E+01	2.44E+06	85	TRUE	TRUE	
1	LAELKCSVK	283	291	9	HLA-A*02:01	283	86	2472350.273	8.60E+01	2.47E+06	86	TRUE	TRUE	
1	ACNFELCDN	127	135	9	HLA-A*02:01	127	86	2489487.787	8.60E+01	2.49E+06	86	TRUE	TRUE	
1	SPCSFGGVS	574	582	9	HLA-A*02:01	574	86	2495226.649	8.60E+01	2.50E+06	86	TRUE	TRUE	
1	VNIQKEIDR	1159	1167	9	HLA-A*02:01	1159	86	2529938.709	8.60E+01	2.53E+06	86	TRUE	TRUE	
1	KYRYLRHGK	439	447	9	HLA-A*02:01	439	86	2547475.407	8.60E+01	2.55E+06	86	TRUE	TRUE	
1	NNTIAIPTN	691	699	9	HLA-A*02:01	691	86	2565133.662	8.60E+01	2.57E+06	86	TRUE	TRUE	
1	AGCLIGAEH	633	641	9	HLA-A*02:01	633	86	2571046.906	8.60E+01	2.57E+06	86	TRUE	TRUE	
1	ECANLLLQY	730	738	9	HLA-A*02:01	730	86	2576973.782	8.60E+01	2.58E+06	86	TRUE	TRUE	

seq #	peptide	start	end	peptide length	allele	peptide index	median binding	smm IC50	smm percentil	legacy IC50	legacy rank	scores equal	percentiles equal
1	LNTLVKQLS	941	949	9	HLA-A*02:01	941	86	2600818.226	8.60E+01	2.60E+06	86	TRUE	TRUE
1	VSTAIHADQ	606	614	9	HLA-A*02:01	606	86	2612823.058	8.60E+01	2.61E+06	86	TRUE	TRUE
1	DDVRQIAPG	392	400	9	HLA-A*02:01	392	86	2643078.123	8.60E+01	2.64E+06	86	TRUE	TRUE
1	EDLLFNKVT	801	809	9	HLA-A*02:01	801	86	2649171.047	8.60E+01	2.65E+06	86	TRUE	TRUE
1	SKPMGTQTH	141	149	9	HLA-A*02:01	141	86	2655278.02	8.60E+01	2.66E+06	86	TRUE	TRUE
1	PYRVVVLSF	493	501	9	HLA-A*02:01	493	87	2761277.156	8.70E+01	2.76E+06	87	TRUE	TRUE
1	CKFDEDDSE	1236	1244	9	HLA-A*02:01	1236	87	2806144.3	8.70E+01	2.81E+06	87	TRUE	TRUE
1	LGQSKRVDF	1016	1024	9	HLA-A*02:01	1016	87	2851740.477	8.70E+01	2.85E+06	87	TRUE	TRUE
1	YGECLGDIN	819	827	9	HLA-A*02:01	819	87	2864903.513	8.70E+01	2.86E+06	87	TRUE	TRUE
1	TGNNVFQTQ	624	632	9	HLA-A*02:01	624	87	2911454.451	8.70E+01	2.91E+06	87	TRUE	TRUE
1	ASVVNIQKE	1156	1164	9	HLA-A*02:01	1156	87	2958761.78	8.70E+01	2.96E+06	87	TRUE	TRUE
1	KPTKRSFIE	793	801	9	HLA-A*02:01	793	87	2958761.78	8.70E+01	2.96E+06	87	TRUE	TRUE
1	SPQIITTDN	1093	1101	9	HLA-A*02:01	1093	87	2986138.865	8.70E+01	2.99E+06	87	TRUE	TRUE
1	AAEIRASAN	997	1005	9	HLA-A*02:01	997	87	3013769.268	8.70E+01	3.01E+06	87	TRUE	TRUE
1	QERNFTTAP	1053	1061	9	HLA-A*02:01	1053	87	3034659.719	8.70E+01	3.03E+06	87	TRUE	TRUE
1	GKLRPFERD	446	454	9	HLA-A*02:01	446	88	3155803.84	8.80E+01	3.16E+06	88	TRUE	TRUE
1	CDVVIGIIN	1108	1116	9	HLA-A*02:01	1108	88	3259192.408	8.80E+01	3.26E+06	88	TRUE	TRUE
1	VPFSPDGKP	458	466	9	HLA-A*02:01	458	88	3327438.214	8.80E+01	3.33E+06	88	TRUE	TRUE
1	SEKSGNFKH	173	181	9	HLA-A*02:01	173	88	3342796.971	8.80E+01	3.34E+06	88	TRUE	TRUE
1	WRIYSTGNN	619	627	9	HLA-A*02:01	619	88	3342796.971	8.80E+01	3.34E+06	88	TRUE	TRUE
1	ANQFNKAIS	906	914	9	HLA-A*02:01	906	88	3358226.621	8.80E+01	3.36E+06	88	TRUE	TRUE
1	QVKQMYKTP	766	774	9	HLA-A*02:01	766	88	3389299.909	8.80E+01	3.39E+06	88	TRUE	TRUE
1	QELGKYEQY	1183	1191	9	HLA-A*02:01	1183	88	3428546.153	8.80E+01	3.43E+06	88	TRUE	TRUE
1	TGNYNYKYR	433	441	9	HLA-A*02:01	433	88	3516494.966	8.80E+01	3.52E+06	88	TRUE	TRUE
1	DAGFMKQYG	812	820	9	HLA-A*02:01	812	88	3524601.324	8.80E+01	3.52E+06	88	TRUE	TRUE
1	DVRQIAPGQ	393	401	9	HLA-A*02:01	393	88	3532726.369	8.80E+01	3.53E+06	88	TRUE	TRUE
1	DDSEPVLKG	1241	1249	9	HLA-A*02:01	1241	88	3540870.144	8.80E+01	3.54E+06	88	TRUE	TRUE
1	ERDISNVPF	452	460	9	HLA-A*02:01	452	89	3557214.058	8.90E+01	3.56E+06	89	TRUE	TRUE
1	AAEQDRNTR	753	761	9	HLA-A*02:01	753	89	3573633.412	8.90E+01	3.57E+06	89	TRUE	TRUE
1	SKRFQPFQQ	542	550	9	HLA-A*02:01	542	89	3590128.555	8.90E+01	3.59E+06	89	TRUE	TRUE
1	PFSPDGKPC	459	467	9	HLA-A*02:01	459	89	3623347.605	8.90E+01	3.62E+06	89	TRUE	TRUE
1	AWERKKISN	339	347	9	HLA-A*02:01	339	89	3648463.451	8.90E+01	3.65E+06	89	TRUE	TRUE
1	EKSNVVRGW	93	101	9	HLA-A*02:01	93	89	3665303.993	8.90E+01	3.67E+06	89	TRUE	TRUE
1	NCTFEYISD	158	166	9	HLA-A*02:01	158	89	3665303.993	8.90E+01	3.67E+06	89	TRUE	TRUE
1	LNESLIDLQ	1175	1183	9	HLA-A*02:01	1175	89	3707746.213	8.90E+01	3.71E+06	89	TRUE	TRUE
1	NNVFQTQAG	626	634	9	HLA-A*02:01	626	89	3716293.451	8.90E+01	3.72E+06	89	TRUE	TRUE
1	AEHVDTSYE	639	647	9	HLA-A*02:01	639	89	3750679.891	8.90E+01	3.75E+06	89	TRUE	TRUE
1	LKCSVKSFE	286	294	9	HLA-A*02:01	286	89	3767992.242	8.90E+01	3.77E+06	89	TRUE	TRUE
1	FNKAISQIQ	909	917	9	HLA-A*02:01	909	89	3767992.242	8.90E+01	3.77E+06	89	TRUE	TRUE
1	CGSCCKFDE	1232	1240	9	HLA-A*02:01	1232	89	3873557.436	8.90E+01	3.87E+06	89	TRUE	TRUE
1	QKQIANQFN	902	910	9	HLA-A*02:01	902	89	3936497.682	8.90E+01	3.94E+06	89	TRUE	TRUE
1	TGVLTPSSK	535	543	9	HLA-A*02:01	535	89	3936497.682	8.90E+01	3.94E+06	89	TRUE	TRUE
1	NFRVVPSGD	304	312	9	HLA-A*02:01	304	89	3945572.246	8.90E+01	3.95E+06	89	TRUE	TRUE
1	LDVSEKSGN	170	178	9	HLA-A*02:01	170	89	4009682.638	8.90E+01	4.01E+06	89	TRUE	TRUE
1	NNTVYDPLQ	1116	1124	9	HLA-A*02:01	1116	89	4009682.638	8.90E+01	4.01E+06	89	TRUE	TRUE
1	QPFQQFGRD	546	554	9	HLA-A*02:01	546	90	4198653.158	9.00E+01	4.20E+06	90	TRUE	TRUE
1	PTKRSFIED	794	802	9	HLA-A*02:01	794	90	4237502.818	9.00E+01	4.24E+06	90	TRUE	TRUE
1	MRGVYYPDE	37	45	9	HLA-A*02:01	37	90	4257062.242	9.00E+01	4.26E+06	90	TRUE	TRUE
1	PGQTGVIAD	399	407	9	HLA-A*02:01	399	90	4257062.24	9.00E+01	4.26E+06	90	TRUE	TRUE
1	SKRVDFCGK	1019	1027	9	HLA-A*02:01	1019	90	4276711.949	9.00E+01	4.28E+06	90	TRUE	TRUE
1	AEQDRNTRE	754	762	9	HLA-A*02:01	754	90	4316283.877	9.00E+01	4.32E+06	90	TRUE	TRUE
1	KNHTSPDVD	1139	1147	9	HLA-A*02:01	1139	90	4700131.405	9.00E+01	4.70E+06	90	TRUE	TRUE
1	VSEKSGNFK	172	180	9	HLA-A*02:01	172	90	4721826.226	9.00E+01	4.72E+06	90	TRUE	TRUE
1	LGKYEQYIK	1185	1193	9	HLA-A*02:01	1185	91	4754556.362	9.10E+01	4.75E+06	91	TRUE	TRUE
1	DATSTGNYN	429	437	9	HLA-A*02:01	429	91	4820698.829	9.10E+01	4.82E+06	91	TRUE	TRUE
1	TEKSNVVRG	92	100	9	HLA-A*02:01	92	91	4887761.43	9.10E+01	4.89E+06	91	TRUE	TRUE
1	QNPLAELKC	280	288	9	HLA-A*02:01	280	91	4910322.312	9.10E+01	4.91E+06	91	TRUE	TRUE
1	REVFAQVKQ	761	769	9	HLA-A*02:01	761	91	4932987.33	9.10E+01	4.93E+06	91	TRUE	TRUE
1	DFTDSVRDP	557	565	9	HLA-A*02:01	557	91	5322430.51	9.10E+01	5.32E+06	91	TRUE	TRUE
1	PNYTQHTSS	28	36	9	HLA-A*02:01	28	92	5573268.932	9.20E+01	5.57E+06	92	TRUE	TRUE
1	DYNYKLPDD	407	415	9	HLA-A*02:01	407	92	5650800.826	9.20E+01	5.65E+06	92	TRUE	TRUE
1	PSGFNTLKP	210	218	9	HLA-A*02:01	210	92	5650800.826	9.20E+01	5.65E+06	92	TRUE	TRUE
1	VRDLPSGFN	206	214	9	HLA-A*02:01	206	92	6110967.866	9.20E+01	6.11E+06	92	TRUE	TRUE
1	SCCKFDEDD	1234	1242	9	HLA-A*02:01	1234	93	6340302.81	9.30E+01	6.34E+06	93	TRUE	TRUE
1	PDVDLGDIS	1144	1152	9	HLA-A*02:01	1144	93	6669756.823	9.30E+01	6.67E+06	93	TRUE	TRUE
1	RCTTFDDVQ	18	26	9	HLA-A*02:01	18	93	6669756.823	9.30E+01	6.67E+06	93	TRUE	TRUE
1	NKAISQIQE	910	918	9	HLA-A*02:01	910	93	6888267.433	9.30E+01	6.89E+06	93	TRUE	TRUE
1	SECVLGQSK	1012	1020	9	HLA-A*02:01	1012	93	6968029.811	9.30E+01	6.97E+06	93	TRUE	TRUE
1	EDDSEPVLK	1240	1248	9	HLA-A*02:01	1240	93	6968029.811	9.30E+01	6.97E+06	93	TRUE	TRUE
1	GSTMNNKSQ	104	112	9	HLA-A*02:01	104	94	7432073.57	9.40E+01	7.43E+06	94	TRUE	TRUE
1	DKGIYQTSN	296	304	9	HLA-A*02:01	296	94	7587697.352	9.40E+01	7.59E+06	94	TRUE	TRUE
1	DGIYFAATE	85	93	9	HLA-A*02:01	85	94	7764437.53	9.40E+01	7.76E+06	94	TRUE	TRUE
1	QNVLYENQK	895	903	9	HLA-A*02:01	895	94	7782336.4	9.40E+01	7.78E+06	94	TRUE	TRUE
1	IFKLPLGIN	219	227	9	HLA-A*02:01	219	94	8651870.294	9.40E+01	8.65E+06	94	TRUE	TRUE
1	TNVVIRACN	121	129	9	HLA-A*02:01	121	94	8914767.066	9.40E+01	8.91E+06	94	TRUE	TRUE
1	RHGKLRPFE	444	452	9	HLA-A*02:01	444	95	9143448.01	9.50E+01	9.14E+06	95	TRUE	TRUE
1	TSTALGKLQ	923	931	9	HLA-A*02:01	923	95	9313437.4	9.50E+01	9.31E+06	95	TRUE	TRUE
1	DSVRDPKTS	560	568	9	HLA-A*02:01	560	95	9888035.11	9.50E+01	9.89E+06	95	TRUE	TRUE
1	IDRLNEVAK	1165	1173	9	HLA-A*02:01	1165	95	10118357.04	9.50E+01	1.01E+07	95	TRUE	TRUE
1	SGNFKHLRE	176	184	9	HLA-A*02:01	176	95	10211981.04	9.50E+01	1.02E+07	95	TRUE	TRUE
1	DRLNEVAKN	1166	1174	9	HLA-A*02:01	1166	95	10473938	9.50E+01	1.05E+07	95	TRUE	TRUE
1	NEVAKNLNE	1169	1177	9	HLA-A*02:01	1169	95	10619645.02	9.50E+01	1.06E+07	95	TRUE	TRUE
1	PDGKPCTPP	462	470	9	HLA-A*02:01	462	95	10842014.91	9.50E+01	1.08E+07	95	TRUE	TRUE
1	NGTITDAVD	269	277	9	HLA-A*02:01	269	95	10867008.33	9.50E+01	1.09E+07	95	TRUE	TRUE
1	PCTPPALNC	466	474	9	HLA-A*02:01	466	96	11120133.47	9.60E+01	1.11E+07	96	TRUE	TRUE
1	DSSIAYSNN	684	692	9	HLA-A*02:01	684	96	11223026.8	9.60E+01	1.12E+07	96	TRUE	TRUE
1	PKLSTDLIK	513	521	9	HLA-A*02:01	513	96	11564051.04	9.60E+01	1.16E+07	96	TRUE	TRUE
1	DSFKEELDK	1128	1136	9	HLA-A*02:01	1128	96	11590708.94	9.60E+01	1.16E+07	96	TRUE	TRUE
1	LRPFERDIS	448	456	9	HLA-A*02:01	448	96	11697956.44	9.60E+01	1.17E+07	96	TRUE	TRUE
1	QDVVNQNAQ	931	939	9	HLA-A*02:01	931	96	12419668.39	9.60E+01	1.24E+07	96	TRUE	TRUE
1	DNPFFAVSK	134	142	9	HLA-A*02:01	134	96	12592443.18	9.60E+01	1.26E+07	96	TRUE	TRUE
1	NDILSRLDK	960	968	9	HLA-A*02:01	960	96	12826554.18	9.60E+01	1.28E+07	96	TRUE	TRUE
1	PSVYAWERK	335	343	9	HLA-A*02:01	335	97	13999418.61	9.70E+01	1.40E+07	97	TRUE	TRUE
1	NESLIDLQE	1176	1184	9	HLA-A*02:01	1176	97	14096457.94	9.70E+01	1.41E+07	97	TRUE	TRUE
1	PELDSFKEE	1125	1133	9	HLA-A*02:01	1125	97	14292559.21	9.70E+01	1.43E+07	97	TRUE	TRUE
1	DNAFNCTFE	154	162	9	HLA-A*02:01	154	97	14794830.71	9.70E+01	1.48E+07	97	TRUE	TRUE
1	LGKLQDVVN	927	935	9	HLA-A*02:01	927	97	14828936.31	9.70E+01	1.48E+07	97	TRUE	TRUE
1	ECDIPIGAG	647	655	9	HLA-A*02:01	647	97	15599475.63	9.70E+01	1.56E+07	97	TRUE	TRUE
1	PREGVFVFN	1072	1080	9	HLA-A*02:01	1072	97	15999632.23	9.70E+01	1.60E+07	97	TRUE	TRUE
1	QPELDSFKE	1124	1132	9	HLA-A*02:01	1124	97	16259605.34	97	16259605.34	97	TRUE	TRUE
1	SFEIDKGIY	292	300	9	HLA-A*02:01	292	97	16372311.54	97	16372311.54	97	TRUE	TRUE
1	NYKYRYLRH	437	445	9	HLA-A*02:01	437	97	16753671.67	97	16753671.67	97	TRUE	TRUE

seq #	peptide	start	end	peptide length	allele	peptide index	median binding	smm IC50	smm percentil	legacy IC50	legacy rank	scores equal	percentiles equal	
1	TPGTNASSE	585	593	9	HLA-A*02:01	585	97	17143914.81	97	17143914.81	97	TRUE	TRUE	
1	DNTFVSGNC	1100	1108	9	HLA-A*02:01	1100	97	18243577.25	97	18243577.25	97	TRUE	TRUE	
1	DPLQPELDS	1121	1129	9	HLA-A*02:01	1121	98	19235788.8	98	19235788.8	98	TRUE	TRUE	
1	ECVLGQSKR	1013	1021	9	HLA-A*02:01	1013	98	20096017.49	98	20096017.49	98	TRUE	TRUE	
1	DPLKPTKRS	790	798	9	HLA-A*02:01	790	98	21883158.2	98	21883158.2	98	TRUE	TRUE	
1	SEPVLKGVK	1243	1251	9	HLA-A*02:01	1243	98	22289996.49	98	22289996.49	98	TRUE	TRUE	
1	LREFVFKNK	182	190	9	HLA-A*02:01	182	98	22652179.79	98	22652179.79	98	TRUE	TRUE	
1	QDRNTREVF	756	764	9	HLA-A*02:01	756	98	23556458.57	98	23556458.57	98	TRUE	TRUE	
1	LNEVAKNLN	1168	1176	9	HLA-A*02:01	1168	98	23829229.5	98	23829229.5	98	TRUE	TRUE	
1	CCKFDEDDS	1235	1243	9	HLA-A*02:01	1235	98	24553307.34	98	24553307.34	98	TRUE	TRUE	
1	EGKAYFPRE	1066	1074	9	HLA-A*02:01	1066	98	24780496.29	98	24780496.29	98	TRUE	TRUE	
1	DSFVVKGDD	385	393	9	HLA-A*02:01	385	99	26675342.24	99	26675342.24	99	TRUE	TRUE	
1	PDPLKPTKR	789	797	9	HLA-A*02:01	789	99	26736835.25	99	26736835.25	99	TRUE	TRUE	
1	EFVFKNKDG	184	192	9	HLA-A*02:01	184	99	29181660.46	99	29181660.46	99	TRUE	TRUE	
1	NPVIPFKDG	78	86	9	HLA-A*02:01	78	99	29724188.04	99	29724188.04	99	TRUE	TRUE	
1	ENQKQIANQ	900	908	9	HLA-A*02:01	900	99	31997054.42	99	31997054.42	99	TRUE	TRUE	
1	DCSQNPLAE	277	285	9	HLA-A*02:01	277	99	33427969.71	99	33427969.71	99	TRUE	TRUE	
1	TREVFAQVK	760	768	9	HLA-A*02:01	760	99	36907106.67	99	36907106.67	99	TRUE	TRUE	
1	LDKVEAEVQ	966	974	9	HLA-A*02:01	966	100	42472712.71	100	42472712.71	100	TRUE	TRUE	
1	PDEIFRSDT	43	51	9	HLA-A*02:01	43	100	45825794.16	100	45825794.16	100	TRUE	TRUE	
1	DPKTSEILD	564	572	9	HLA-A*02:01	564	100	54589611.05	100	54589611.05	100	TRUE	TRUE	
1	DRLITGRLQ	976	984	9	HLA-A*02:01	976	100	112230268	100	112230268	100	TRUE	TRUE	
1	QKEIDRLNE	1162	1170	9	HLA-A*02:01	1162	100	115374544.6	100	115374544.6	100	TRUE	TRUE	

seq #	peptide	start	end	peptide length	allele	peptide index	median binding	comblib_sidney	comblib_sidney	legacy IC50	legacy rank	scores equal	percentiles equal	
1	FAMQMAYRF	880	888	9	HLA-A*02:01	880	0.2	2.31E-06	0.2	2.31E-06	0.2	TRUE	TRUE	
1	FSISITTEV	700	708	9	HLA-A*02:01	700	0.2	2.64E-06	0.2	2.64E-06	0.2	TRUE	TRUE	
1	VLYNSTFFS	354	362	9	HLA-A*02:01	354	0.3	3.72E-06	0.3	3.72E-06	0.3	TRUE	TRUE	
1	GTSAAAYFV	246	254	9	HLA-A*02:01	246	0.7	9.62E-06	0.7	9.62E-06	0.7	TRUE	TRUE	
1	RLQSLQTYV	982	990	9	HLA-A*02:01	982	0.8	1.09E-05	0.8	1.09E-05	0.8	TRUE	TRUE	
1	TSYECDIPI	644	652	9	HLA-A*02:01	644	0.8	1.17E-05	0.8	1.17E-05	0.8	TRUE	TRUE	
1	FNFSQILPD	782	790	9	HLA-A*02:01	782	0.8	1.18E-05	0.8	1.18E-05	0.8	TRUE	TRUE	
1	RLDKVEAEV	965	973	9	HLA-A*02:01	965	0.9	1.47E-05	0.9	1.47E-05	0.9	TRUE	TRUE	
1	FLLFLTLTS	4	12	9	HLA-A*02:01	4	1	1.52E-05	1	1.52E-05	1	TRUE	TRUE	
1	FGNPVIPFK	76	84	9	HLA-A*02:01	76	1	1.58E-05	1	1.58E-05	1	TRUE	TRUE	
1	FDEDDSEPV	1238	1246	9	HLA-A*02:01	1238	1	1.63E-05	1	1.63E-05	1	TRUE	TRUE	
1	FVFNGTSWF	1077	1085	9	HLA-A*02:01	1077	1	1.65E-05	1	1.65E-05	1	TRUE	TRUE	
1	HLMSFPQAA	1030	1038	9	HLA-A*02:01	1030	1.1	1.88E-05	1.1	1.88E-05	1.1	TRUE	TRUE	
1	ELLNAPATV	502	510	9	HLA-A*02:01	502	1.2	1.93E-05	1.2	1.93E-05	1.2	TRUE	TRUE	
1	DGKPCTPPA	463	471	9	HLA-A*02:01	463	1.4	2.22E-05	1.4	2.22E-05	1.4	TRUE	TRUE	
1	FIAGLIAIV	1202	1210	9	HLA-A*02:01	1202	1.7	2.89E-05	1.7	2.89E-05	1.7	TRUE	TRUE	
1	FELLNAPAT	501	509	9	HLA-A*02:01	501	1.7	2.92E-05	1.7	2.92E-05	1.7	TRUE	TRUE	
1	FPQAAPHGV	1034	1042	9	HLA-A*02:01	1034	1.8	3.07E-05	1.8	3.07E-05	1.8	TRUE	TRUE	
1	FNATKFPSV	329	337	9	HLA-A*02:01	329	1.8	3.12E-05	1.8	3.12E-05	1.8	TRUE	TRUE	
1	DLDRCTTFD	15	23	9	HLA-A*02:01	15	1.9	3.31E-05	1.9	3.31E-05	1.9	TRUE	TRUE	
1	VYYPDEIFR	40	48	9	HLA-A*02:01	40	2	3.38E-05	2	3.38E-05	2	TRUE	TRUE	
1	NAFNCTFEY	155	163	9	HLA-A*02:01	155	2	3.38E-05	2	3.38E-05	2	TRUE	TRUE	
1	FELCDNPFF	130	138	9	HLA-A*02:01	130	2	3.52E-05	2	3.52E-05	2	TRUE	TRUE	
1	RDISNVPFS	453	461	9	HLA-A*02:01	453	2.1	3.68E-05	2.1	3.68E-05	2.1	TRUE	TRUE	
1	FSTFKCYGV	361	369	9	HLA-A*02:01	361	2.1	3.72E-05	2.1	3.72E-05	2.1	TRUE	TRUE	
1	FEIDKGIYQ	293	301	9	HLA-A*02:01	293	2.1	3.78E-05	2.1	3.78E-05	2.1	TRUE	TRUE	
1	SNFGAISSV	950	958	9	HLA-A*02:01	950	2.2	3.92E-05	2.2	3.92E-05	2.2	TRUE	TRUE	
1	ELCDNPFFA	131	139	9	HLA-A*02:01	131	2.3	4.03E-05	2.3	4.03E-05	2.3	TRUE	TRUE	
1	TVYDPLQPE	1118	1126	9	HLA-A*02:01	1118	2.3	4.07E-05	2.3	4.07E-05	2.3	TRUE	TRUE	
1	AGAALQIPF	872	880	9	HLA-A*02:01	872	2.5	4.34E-05	2.5	4.34E-05	2.5	TRUE	TRUE	
1	ALVSGTATA	858	866	9	HLA-A*02:01	858	2.6	4.57E-05	2.6	4.57E-05	2.6	TRUE	TRUE	
1	NGIGVTQNV	889	897	9	HLA-A*02:01	889	2.9	5.06E-05	2.9	5.06E-05	2.9	TRUE	TRUE	
1	FKLPLGINI	220	228	9	HLA-A*02:01	220	2.9	5.11E-05	2.9	5.11E-05	2.9	TRUE	TRUE	
1	FLYVYKGYQ	193	201	9	HLA-A*02:01	193	2.9	5.13E-05	2.9	5.13E-05	2.9	TRUE	TRUE	
1	SLIDLQELG	1178	1186	9	HLA-A*02:01	1178	2.9	5.15E-05	2.9	5.15E-05	2.9	TRUE	TRUE	
1	YLKPTTFML	256	264	9	HLA-A*02:01	256	3.2	5.55E-05	3.2	5.55E-05	3.2	TRUE	TRUE	
1	KEIDRLNEV	1163	1171	9	HLA-A*02:01	1163	3.4	5.75E-05	3.4	5.75E-05	3.4	TRUE	TRUE	
1	FYSNVTGFH	62	70	9	HLA-A*02:01	62	3.5	5.89E-05	3.5	5.89E-05	3.5	TRUE	TRUE	
1	VVFLHVTYV	1042	1050	9	HLA-A*02:01	1042	3.6	6.17E-05	3.6	6.17E-05	3.6	TRUE	TRUE	
1	FITQRNFFS	1085	1093	9	HLA-A*02:01	1085	3.6	6.24E-05	3.6	6.24E-05	3.6	TRUE	TRUE	
1	ELGKYEQYI	1184	1192	9	HLA-A*02:01	1184	3.6	6.27E-05	3.6	6.27E-05	3.6	TRUE	TRUE	
1	FFSPQIITT	1091	1099	9	HLA-A*02:01	1091	3.7	6.47E-05	3.7	6.47E-05	3.7	TRUE	TRUE	
1	LLCCMTSCC	1215	1223	9	HLA-A*02:01	1215	3.7	6.48E-05	3.7	6.48E-05	3.7	TRUE	TRUE	
1	FLHVTYVPS	1044	1052	9	HLA-A*02:01	1044	3.7	6.49E-05	3.7	6.49E-05	3.7	TRUE	TRUE	
1	NVYADSFVV	381	389	9	HLA-A*02:01	381	3.7	6.53E-05	3.7	6.53E-05	3.7	TRUE	TRUE	
1	FRSDTLYLT	47	55	9	HLA-A*02:01	47	3.9	6.81E-05	3.9	6.81E-05	3.9	TRUE	TRUE	
1	GLIAIVMVT	1205	1213	9	HLA-A*02:01	1205	3.9	6.85E-05	3.9	6.85E-05	3.9	TRUE	TRUE	
1	KQMYKTPTL	768	776	9	HLA-A*02:01	768	4.1	7.24E-05	4.1	7.24E-05	4.1	TRUE	TRUE	
1	FRAILTAFS	231	239	9	HLA-A*02:01	231	4.3	7.42E-05	4.3	7.42E-05	4.3	TRUE	TRUE	
1	LLFNKVTLA	803	811	9	HLA-A*02:01	803	4.3	7.52E-05	4.3	7.52E-05	4.3	TRUE	TRUE	
1	PALNCYWPL	470	478	9	HLA-A*02:01	470	4.4	7.67E-05	4.4	7.67E-05	4.4	TRUE	TRUE	
1	DLFLPFYSN	57	65	9	HLA-A*02:01	57	4.6	7.93E-05	4.6	7.93E-05	4.6	TRUE	TRUE	
1	FNTLKPIFK	213	221	9	HLA-A*02:01	213	4.6	7.98E-05	4.6	7.98E-05	4.6	TRUE	TRUE	
1	ELDSFKEEL	1126	1134	9	HLA-A*02:01	1126	4.7	8.07E-05	4.7	8.07E-05	4.7	TRUE	TRUE	
1	TNASSEVAV	588	596	9	HLA-A*02:01	588	4.7	8.12E-05	4.7	8.12E-05	4.7	TRUE	TRUE	
1	FTTAPAICH	1057	1065	9	HLA-A*02:01	1057	4.8	8.32E-05	4.8	8.32E-05	4.8	TRUE	TRUE	
1	PLAELKCSV	282	290	9	HLA-A*02:01	282	4.9	8.49E-05	4.9	8.49E-05	4.9	TRUE	TRUE	
1	VLYENQKQI	897	905	9	HLA-A*02:01	897	5	8.85E-05	5	8.85E-05	5	TRUE	TRUE	
1	FNGLTVLPP	837	845	9	HLA-A*02:01	837	5.1	8.97E-05	5.1	8.97E-05	5.1	TRUE	TRUE	
1	VLAWNTRNI	420	428	9	HLA-A*02:01	420	5.1	9.04E-05	5.1	9.04E-05	5.1	TRUE	TRUE	
1	FTDSVRDPK	558	566	9	HLA-A*02:01	558	5.2	9.13E-05	5.2	9.13E-05	5.2	TRUE	TRUE	
1	DVSDFTDSV	554	562	9	HLA-A*02:01	554	5.2	9.21E-05	5.2	9.21E-05	5.2	TRUE	TRUE	
1	TSVDCNMYI	716	724	9	HLA-A*02:01	716	5.2	9.26E-05	5.2	9.26E-05	5.2	TRUE	TRUE	
1	AYSNNTIAI	688	696	9	HLA-A*02:01	688	5.4	9.38E-05	5.4	9.38E-05	5.4	TRUE	TRUE	
1	FKHLREFVF	179	187	9	HLA-A*02:01	179	5.4	9.42E-05	5.4	9.42E-05	5.4	TRUE	TRUE	
1	VLNDILSRL	958	966	9	HLA-A*02:01	958	5.5	9.73E-05	5.5	9.73E-05	5.5	TRUE	TRUE	
1	LNDLCFSNV	374	382	9	HLA-A*02:01	374	5.5	9.77E-05	5.5	9.77E-05	5.5	TRUE	TRUE	
1	TLYLTQDLF	51	59	9	HLA-A*02:01	51	5.7	0.00010046	5.7	0.00010046	5.7	TRUE	TRUE	
1	TQAGCLIGA	631	639	9	HLA-A*02:01	631	5.7	0.00010139	5.7	0.00010139	5.7	TRUE	TRUE	
1	TQDLFLPFY	55	63	9	HLA-A*02:01	55	5.9	0.00010568	5.9	0.00010568	5.9	TRUE	TRUE	
1	GAALQIPFA	873	881	9	HLA-A*02:01	873	6	0.00010695	6	0.00010695	6	TRUE	TRUE	
1	LCFSNVYAD	377	385	9	HLA-A*02:01	377	6.2	0.00011003	6.2	0.00011003	6.2	TRUE	TRUE	
1	FVSGNCDVV	1103	1111	9	HLA-A*02:01	1103	6.2	0.00011015	6.2	0.00011015	6.2	TRUE	TRUE	
1	NLNESLIDL	1174	1182	9	HLA-A*02:01	1174	6.2	0.00011041	6.2	0.00011041	6.2	TRUE	TRUE	
1	IINNSTNVV	116	124	9	HLA-A*02:01	116	6.2	0.00011041	6.2	0.00011041	6.2	TRUE	TRUE	
1	GFIAGLIAI	1201	1209	9	HLA-A*02:01	1201	6.2	0.0001114	6.2	0.0001114	6.2	TRUE	TRUE	
1	KLNDLCFSN	373	381	9	HLA-A*02:01	373	6.2	0.00011148	6.2	0.00011148	6.2	TRUE	TRUE	
1	ADYSVLYNS	350	358	9	HLA-A*02:01	350	6.2	0.00011231	6.2	0.00011231	6.2	TRUE	TRUE	
1	LLTDDMIAA	846	854	9	HLA-A*02:01	846	6.3	0.00011376	6.3	0.00011376	6.3	TRUE	TRUE	
1	TFGNPVIPF	75	83	9	HLA-A*02:01	75	6.3	0.00011479	6.3	0.00011479	6.3	TRUE	TRUE	
1	FGAGAALQI	870	878	9	HLA-A*02:01	870	6.3	0.00011508	6.3	0.00011508	6.3	TRUE	TRUE	
1	VLYQDVNCT	596	604	9	HLA-A*02:01	596	6.4	0.00011561	6.4	0.00011561	6.4	TRUE	TRUE	
1	RIYSTGNNV	620	628	9	HLA-A*02:01	620	6.5	0.00011722	6.5	0.00011722	6.5	TRUE	TRUE	
1	NQFNKAISQ	907	915	9	HLA-A*02:01	907	6.5	0.00011768	6.5	0.00011768	6.5	TRUE	TRUE	
1	TLADAGFMK	809	817	9	HLA-A*02:01	809	6.5	0.00011866	6.5	0.00011866	6.5	TRUE	TRUE	
1	VSMAKTSVD	711	719	9	HLA-A*02:01	711	6.5	0.0001191	6.5	0.0001191	6.5	TRUE	TRUE	
1	GYHLMSFPQ	1028	1036	9	HLA-A*02:01	1028	6.6	0.00012048	6.6	0.00012048	6.6	TRUE	TRUE	
1	KGYQPIDVV	198	206	9	HLA-A*02:01	198	6.6	0.0001205	6.6	0.0001205	6.6	TRUE	TRUE	
1	CLIGAEHVD	635	643	9	HLA-A*02:01	635	6.7	0.00012165	6.7	0.00012165	6.7	TRUE	TRUE	
1	LLLQYGSFC	734	742	9	HLA-A*02:01	734	6.8	0.0001234	6.8	0.0001234	6.8	TRUE	TRUE	
1	YVYKGYQPI	195	203	9	HLA-A*02:01	195	6.8	0.00012359	6.8	0.00012359	6.8	TRUE	TRUE	
1	GNCDVVIGI	1106	1114	9	HLA-A*02:01	1106	6.8	0.00012439	6.8	0.00012439	6.8	TRUE	TRUE	
1	SSMRGVYYP	35	43	9	HLA-A*02:01	35	6.9	0.00012529	6.9	0.00012529	6.9	TRUE	TRUE	
1	FNFNGLTGT	527	535	9	HLA-A*02:01	527	6.9	0.00012555	6.9	0.00012555	6.9	TRUE	TRUE	
1	LTDDMIAAY	847	855	9	HLA-A*02:01	847	7	0.00012764	7	0.00012764	7	TRUE	TRUE	
1	ILTAFSPAQ	234	242	9	HLA-A*02:01	234	7	0.00012814	7	0.00012814	7	TRUE	TRUE	
1	KQLSSNFGA	946	954	9	HLA-A*02:01	946	7	0.00012882	7	0.00012882	7	TRUE	TRUE	
1	VIRACNFEL	124	132	9	HLA-A*02:01	124	7.1	0.00012942	7.1	0.00012942	7.1	TRUE	TRUE	
1	LGINITNFR	224	232	9	HLA-A*02:01	224	7.1	0.00013044	7.1	0.00013044	7.1	TRUE	TRUE	
1	PFKDGIYFA	82	90	9	HLA-A*02:01	82	7.2	0.00013083	7.2	0.00013083	7.2	TRUE	TRUE	
1	FKNHTSPDV	1138	1146	9	HLA-A*02:01	1138	7.6	0.0001406	7.6	0.0001406	7.6	TRUE	TRUE	
1	VRDPKTSEI	562	570	9	HLA-A*02:01	562	7.8	0.00014335	7.8	0.00014335	7.8	TRUE	TRUE	

seq #	peptide	start	end	peptide length	allele	peptide index	median binding	comblib_sidney	comblib_sidney	legacy IC50	legacy rank	scores equal	percentiles equal
1	QYIKWPWYV	1190	1198	9	HLA-A*02:01	1190	7.9	0.00014561	7.9	0.00014561	7.9	TRUE	TRUE
1	IPFAMQMAY	878	886	9	HLA-A*02:01	878	7.9	0.00014608	7.9	0.00014608	7.9	TRUE	TRUE
1	MSFPQAAPH	1032	1040	9	HLA-A*02:01	1032	8.2	0.00015101	8.2	0.00015101	8.2	TRUE	TRUE
1	SEILDISPC	568	576	9	HLA-A*02:01	568	8.2	0.00015321	8.2	0.00015321	8.2	TRUE	TRUE
1	ILPDPLKPT	787	795	9	HLA-A*02:01	787	8.3	0.00015417	8.3	0.00015417	8.3	TRUE	TRUE
1	DEDDSEPVL	1239	1247	9	HLA-A*02:01	1239	8.4	0.00015567	8.4	0.00015567	8.4	TRUE	TRUE
1	TALGKLQDV	925	933	9	HLA-A*02:01	925	8.4	0.00015639	8.4	0.00015639	8.4	TRUE	TRUE
1	SLLRSTSQK	664	672	9	HLA-A*02:01	664	8.4	0.00015787	8.4	0.00015787	8.4	TRUE	TRUE
1	ALGKLQDVV	926	934	9	HLA-A*02:01	926	8.5	0.00015922	8.5	0.00015922	8.5	TRUE	TRUE
1	NGLTVLPPL	838	846	9	HLA-A*02:01	838	8.6	0.00015996	8.6	0.00015996	8.6	TRUE	TRUE
1	KAISQIQES	911	919	9	HLA-A*02:01	911	8.6	0.00016226	8.6	0.00016226	8.6	TRUE	TRUE
1	PLNDYGFYT	477	485	9	HLA-A*02:01	477	8.7	0.00016285	8.7	0.00016285	8.7	TRUE	TRUE
1	IYFAATEKS	87	95	9	HLA-A*02:01	87	8.7	0.00016376	8.7	0.00016376	8.7	TRUE	TRUE
1	KLQDVVNQN	929	937	9	HLA-A*02:01	929	8.8	0.00016489	8.8	0.00016489	8.8	TRUE	TRUE
1	IAIPTNFSI	694	702	9	HLA-A*02:01	694	8.8	0.00016527	8.8	0.00016527	8.8	TRUE	TRUE
1	VYDPLQPEL	1119	1127	9	HLA-A*02:01	1119	8.8	0.00016604	8.8	0.00016604	8.8	TRUE	TRUE
1	MIFDNAFNC	151	159	9	HLA-A*02:01	151	9	0.00016916	9	0.00016916	9	TRUE	TRUE
1	AGLIAIVMV	1204	1212	9	HLA-A*02:01	1204	9.1	0.00017022	9.1	0.00017022	9.1	TRUE	TRUE
1	FIFLLFLTL	2	10	9	HLA-A*02:01	2	9.1	0.000171	9.1	0.000171	9.1	TRUE	TRUE
1	EVFNATKFP	327	335	9	HLA-A*02:01	327	9.1	0.00017104	9.1	0.00017104	9.1	TRUE	TRUE
1	LCDNPFFAV	132	140	9	HLA-A*02:01	132	9.2	0.00017155	9.2	0.00017155	9.2	TRUE	TRUE
1	TDLIKNQCV	517	525	9	HLA-A*02:01	517	9.2	0.00017155	9.2	0.00017155	9.2	TRUE	TRUE
1	DILSRLDKV	961	969	9	HLA-A*02:01	961	9.2	0.00017187	9.2	0.00017187	9.2	TRUE	TRUE
1	FLTLTSGSD	7	15	9	HLA-A*02:01	7	9.2	0.00017262	9.2	0.00017262	9.2	TRUE	TRUE
1	TFVSGNCDV	1102	1110	9	HLA-A*02:01	1102	9.3	0.00017454	9.3	0.00017454	9.3	TRUE	TRUE
1	SITTEVMPV	703	711	9	HLA-A*02:01	703	9.3	0.00017498	9.3	0.00017498	9.3	TRUE	TRUE
1	DDMIAAYTA	849	857	9	HLA-A*02:01	849	9.4	0.00017807	9.4	0.00017807	9.4	TRUE	TRUE
1	VIADYNYKL	404	412	9	HLA-A*02:01	404	9.4	0.00017824	9.4	0.00017824	9.4	TRUE	TRUE
1	NAQALNTLV	937	945	9	HLA-A*02:01	937	9.4	0.00017832	9.4	0.00017832	9.4	TRUE	TRUE
1	FEYISDAFS	161	169	9	HLA-A*02:01	161	9.7	0.00018281	9.7	0.00018281	9.7	TRUE	TRUE
1	EQDRNTREV	755	763	9	HLA-A*02:01	755	9.8	0.00018323	9.8	0.00018323	9.8	TRUE	TRUE
1	QIAPGQTGV	396	404	9	HLA-A*02:01	396	9.8	0.00018365	9.8	0.00018365	9.8	TRUE	TRUE
1	TAFSPAQDI	236	244	9	HLA-A*02:01	236	9.8	0.00018374	9.8	0.00018374	9.8	TRUE	TRUE
1	LLFLTLTSG	5	13	9	HLA-A*02:01	5	9.9	0.00018535	9.9	0.00018535	9.9	TRUE	TRUE
1	GIINNTVYD	1113	1121	9	HLA-A*02:01	1113	10	0.00018668	10	0.00018668	10	TRUE	TRUE
1	RNIDATSTG	426	434	9	HLA-A*02:01	426	10	0.00018698	10	0.00018698	10	TRUE	TRUE
1	FDNAFNCTF	153	161	9	HLA-A*02:01	153	12	0.00019249	12	0.00019249	12	TRUE	TRUE
1	FNGLTGTGV	529	537	9	HLA-A*02:01	529	12	0.00019311	12	0.00019311	12	TRUE	TRUE
1	TMNNKSQSV	106	114	9	HLA-A*02:01	106	12	0.0001932	12	0.0001932	12	TRUE	TRUE
1	VQIDRLITG	973	981	9	HLA-A*02:01	973	12	0.00019498	12	0.00019498	12	TRUE	TRUE
1	FIEDLLFNK	799	807	9	HLA-A*02:01	799	12	0.00019692	12	0.00019692	12	TRUE	TRUE
1	CLGDINARD	822	830	9	HLA-A*02:01	822	12	0.0001982	12	0.0001982	12	TRUE	TRUE
1	FPREGVFVF	1071	1079	9	HLA-A*02:01	1071	12	0.00020119	12	0.00020119	12	TRUE	TRUE
1	GGVSVITPG	579	587	9	HLA-A*02:01	579	12	0.00020137	12	0.00020137	12	TRUE	TRUE
1	FDDVQAPNY	22	30	9	HLA-A*02:01	22	12	0.00020249	12	0.00020249	12	TRUE	TRUE
1	SGFNTLKPI	211	219	9	HLA-A*02:01	211	12	0.00020417	12	0.00020417	12	TRUE	TRUE
1	FSLDVSEKS	168	176	9	HLA-A*02:01	168	12	0.00020549	12	0.00020549	12	TRUE	TRUE
1	FGSTMNNKS	103	111	9	HLA-A*02:01	103	12	0.00021038	12	0.00021038	12	TRUE	TRUE
1	LIRAAEIRA	994	1002	9	HLA-A*02:01	994	12	0.0002138	12	0.0002138	12	TRUE	TRUE
1	YFKNHTSPD	1137	1145	9	HLA-A*02:01	1137	13	0.00021727	13	0.00021727	13	TRUE	TRUE
1	GCLIGAEHV	634	642	9	HLA-A*02:01	634	13	0.00021847	13	0.00021847	13	TRUE	TRUE
1	QLSSNFGAI	947	955	9	HLA-A*02:01	947	13	0.00021928	13	0.00021928	13	TRUE	TRUE
1	DFMGCVLAW	415	423	9	HLA-A*02:01	415	13	0.00022019	13	0.00022019	13	TRUE	TRUE
1	ALSGIAAEQ	748	756	9	HLA-A*02:01	748	13	0.00022269	13	0.00022269	13	TRUE	TRUE
1	TKFPSVYAW	332	340	9	HLA-A*02:01	332	13	0.0002232	13	0.0002232	13	TRUE	TRUE
1	MIAAYTAAL	851	859	9	HLA-A*02:01	851	13	0.00022542	13	0.00022542	13	TRUE	TRUE
1	HTINHTFGN	70	78	9	HLA-A*02:01	70	13	0.00022615	13	0.00022615	13	TRUE	TRUE
1	EYISDAFSL	162	170	9	HLA-A*02:01	162	13	0.00022761	13	0.00022761	13	TRUE	TRUE
1	KLRPFERDI	447	455	9	HLA-A*02:01	447	13	0.00022856	13	0.00022856	13	TRUE	TRUE
1	ILLCCMTSC	1214	1222	9	HLA-A*02:01	1214	13	0.0002319	13	0.0002319	13	TRUE	TRUE
1	FSPAQDIWG	238	246	9	HLA-A*02:01	238	13	0.0002327	13	0.0002327	13	TRUE	TRUE
1	KNKDGFLYV	188	196	9	HLA-A*02:01	188	13	0.00023324	13	0.00023324	13	TRUE	TRUE
1	PFYSNVTGF	61	69	9	HLA-A*02:01	61	13	0.00023372	13	0.00023372	13	TRUE	TRUE
1	TFDDVQAPN	21	29	9	HLA-A*02:01	21	13	0.00023394	13	0.00023394	13	TRUE	TRUE
1	SGIAAEQDR	750	758	9	HLA-A*02:01	750	13	0.0002341	13	0.0002341	13	TRUE	TRUE
1	HLREFVFKN	181	189	9	HLA-A*02:01	181	13	0.00023627	13	0.00023627	13	TRUE	TRUE
1	ADSSIAYSN	683	691	9	HLA-A*02:01	683	13	0.00023747	13	0.00023747	13	TRUE	TRUE
1	ATAGWTFGA	864	872	9	HLA-A*02:01	864	13	0.00024099	13	0.00024099	13	TRUE	TRUE
1	FNCTFEYIS	157	165	9	HLA-A*02:01	157	13	0.00024199	13	0.00024199	13	TRUE	TRUE
1	FAATEKSNV	89	97	9	HLA-A*02:01	89	14	0.00024558	14	0.00024558	14	TRUE	TRUE
1	VVLSFELLN	497	505	9	HLA-A*02:01	497	14	0.00024957	14	0.00024957	14	TRUE	TRUE
1	GLTVLPPLL	839	847	9	HLA-A*02:01	839	14	0.00025177	14	0.00025177	14	TRUE	TRUE
1	LFLPFYSNV	58	66	9	HLA-A*02:01	58	14	0.00025229	14	0.00025229	14	TRUE	TRUE
1	TPAWRIYST	616	624	9	HLA-A*02:01	616	14	0.00026767	14	0.00026767	14	TRUE	TRUE
1	FGGVSVITP	578	586	9	HLA-A*02:01	578	14	0.00026922	14	0.00026922	14	TRUE	TRUE
1	QLNRALSGI	744	752	9	HLA-A*02:01	744	15	0.00026977	15	0.00026977	15	TRUE	TRUE
1	TGIGYQPYR	487	495	9	HLA-A*02:01	487	15	0.00027252	15	0.00027252	15	TRUE	TRUE
1	YLTQDLFLP	53	61	9	HLA-A*02:01	53	15	0.00027359	15	0.00027359	15	TRUE	TRUE
1	PGTNASSEV	586	594	9	HLA-A*02:01	586	15	0.00027403	15	0.00027403	15	TRUE	TRUE
1	SNVYADSFV	380	388	9	HLA-A*02:01	380	15	0.00027784	15	0.00027784	15	TRUE	TRUE
1	LKYDENGTI	264	272	9	HLA-A*02:01	264	15	0.00027797	15	0.00027797	15	TRUE	TRUE
1	MQMAYRFNG	882	890	9	HLA-A*02:01	882	15	0.00028314	15	0.00028314	15	TRUE	TRUE
1	SVKSFEIDK	289	297	9	HLA-A*02:01	289	15	0.00029127	15	0.00029127	15	TRUE	TRUE
1	VDCSQNPLA	276	284	9	HLA-A*02:01	276	15	0.00029268	15	0.00029268	15	TRUE	TRUE
1	MPVSMAKTS	709	717	9	HLA-A*02:01	709	15	0.00029349	15	0.00029349	15	TRUE	TRUE
1	FKDGIYFAA	83	91	9	HLA-A*02:01	83	15	0.00029376	15	0.00029376	15	TRUE	TRUE
1	HADQLTPAW	611	619	9	HLA-A*02:01	611	15	0.00029383	15	0.00029383	15	TRUE	TRUE
1	AATKMSECV	1007	1015	9	HLA-A*02:01	1007	15	0.0002939	15	0.0002939	15	TRUE	TRUE
1	SNCVADYSV	346	354	9	HLA-A*02:01	346	16	0.00029771	16	0.00029771	16	TRUE	TRUE
1	TDVSTAIHA	604	612	9	HLA-A*02:01	604	16	0.00029813	16	0.00029813	16	TRUE	TRUE
1	FPSVYAWER	334	342	9	HLA-A*02:01	334	16	0.00029854	16	0.00029854	16	TRUE	TRUE
1	IKNQCVNFN	520	528	9	HLA-A*02:01	520	16	0.00029868	16	0.00029868	16	TRUE	TRUE
1	DLPSGFNTL	208	216	9	HLA-A*02:01	208	16	0.00029936	16	0.00029936	16	TRUE	TRUE
1	NITNLCPFG	318	326	9	HLA-A*02:01	318	16	0.00030061	16	0.00030061	16	TRUE	TRUE
1	DLICAQKFN	830	838	9	HLA-A*02:01	830	16	0.00030088	16	0.00030088	16	TRUE	TRUE
1	GADSSIAYS	682	690	9	HLA-A*02:01	682	16	0.00030283	16	0.00030283	16	TRUE	TRUE
1	YDENGTITD	266	274	9	HLA-A*02:01	266	16	0.00030796	16	0.00030796	16	TRUE	TRUE
1	ISDAFSLDV	164	172	9	HLA-A*02:01	164	16	0.0003096	16	0.0003096	16	TRUE	TRUE
1	AIVMVTILL	1208	1216	9	HLA-A*02:01	1208	16	0.00031117	16	0.00031117	16	TRUE	TRUE
1	TNFSISITT	698	706	9	HLA-A*02:01	698	16	0.00031246	16	0.00031246	16	TRUE	TRUE
1	DISGINASV	1150	1158	9	HLA-A*02:01	1150	16	0.00031275	16	0.00031275	16	TRUE	TRUE
1	KSQSVIIIN	110	118	9	HLA-A*02:01	110	16	0.00031333	16	0.00031333	16	TRUE	TRUE

seq #	peptide	start	end	peptide length	allele	peptide index	median binding	comblib_sidney	comblib_sidney	legacy IC50	legacy rank	scores equal	percentiles equal	
1	QLIRAAEIR	993	1001	9	HLA-A*02:01	993	16	0.00031434	16	0.00031434	16	TRUE	TRUE	
1	ANLLLQYGS	732	740	9	HLA-A*02:01	732	16	0.00032048	16	0.00032048	16	TRUE	TRUE	
1	ADYNYKLPD	406	414	9	HLA-A*02:01	406	17	0.00032174	17	0.00032174	17	TRUE	TRUE	
1	KAYFPREGV	1068	1076	9	HLA-A*02:01	1068	17	0.00032226	17	0.00032226	17	TRUE	TRUE	
1	FSPDGKPCT	460	468	9	HLA-A*02:01	460	17	0.00032494	17	0.00032494	17	TRUE	TRUE	
1	DLCFSNVYA	376	384	9	HLA-A*02:01	376	17	0.00032524	17	0.00032524	17	TRUE	TRUE	
1	FAVSKPMGT	138	146	9	HLA-A*02:01	138	17	0.00032599	17	0.00032599	17	TRUE	TRUE	
1	ITTDNTFVS	1097	1105	9	HLA-A*02:01	1097	17	0.00032659	17	0.00032659	17	TRUE	TRUE	
1	IAYSNNTIA	687	695	9	HLA-A*02:01	687	17	0.00032825	17	0.00032825	17	TRUE	TRUE	
1	SNNTIAIPT	690	698	9	HLA-A*02:01	690	17	0.00033022	17	0.00033022	17	TRUE	TRUE	
1	FLPFYSNVT	59	67	9	HLA-A*02:01	59	17	0.00033037	17	0.00033037	17	TRUE	TRUE	
1	NFFSPQIIT	1090	1098	9	HLA-A*02:01	1090	17	0.00033258	17	0.00033258	17	TRUE	TRUE	
1	LPDDFMGCV	412	420	9	HLA-A*02:01	412	17	0.00033312	17	0.00033312	17	TRUE	TRUE	
1	IIINNSTNV	115	123	9	HLA-A*02:01	115	17	0.00033343	17	0.00033343	17	TRUE	TRUE	
1	TTFDDVQAP	20	28	9	HLA-A*02:01	20	17	0.00033504	17	0.00033504	17	TRUE	TRUE	
1	YGSFCTQLN	738	746	9	HLA-A*02:01	738	17	0.00033744	17	0.00033744	17	TRUE	TRUE	
1	KQIANQFNK	903	911	9	HLA-A*02:01	903	17	0.00033752	17	0.00033752	17	TRUE	TRUE	
1	IHADQLTPA	610	618	9	HLA-A*02:01	610	17	0.0003376	17	0.0003376	17	TRUE	TRUE	
1	IEDLLFNKV	800	808	9	HLA-A*02:01	800	17	0.00033884	17	0.00033884	17	TRUE	TRUE	
1	LPLGINITN	222	230	9	HLA-A*02:01	222	17	0.00034658	17	0.00034658	17	TRUE	TRUE	
1	SIVAYTMSL	673	681	9	HLA-A*02:01	673	17	0.00034914	17	0.00034914	17	TRUE	TRUE	
1	RFQPFQQFG	544	552	9	HLA-A*02:01	544	17	0.00034986	17	0.00034986	17	TRUE	TRUE	
1	LDISPCSFG	571	579	9	HLA-A*02:01	571	18	0.00035188	18	0.00035188	18	TRUE	TRUE	
1	NLAATKMSE	1005	1013	9	HLA-A*02:01	1005	18	0.00035237	18	0.00035237	18	TRUE	TRUE	
1	IAAYTAALV	852	860	9	HLA-A*02:01	852	18	0.00035335	18	0.00035335	18	TRUE	TRUE	
1	FSQILPDPL	784	792	9	HLA-A*02:01	784	18	0.00035547	18	0.00035547	18	TRUE	TRUE	
1	NSTNVVIRA	119	127	9	HLA-A*02:01	119	18	0.00035793	18	0.00035793	18	TRUE	TRUE	
1	CMTSCCSCL	1218	1226	9	HLA-A*02:01	1218	18	0.00036141	18	0.00036141	18	TRUE	TRUE	
1	VDCNMYICG	718	726	9	HLA-A*02:01	718	18	0.00036174	18	0.00036174	18	TRUE	TRUE	
1	INHTFGNPV	72	80	9	HLA-A*02:01	72	18	0.00036627	18	0.00036627	18	TRUE	TRUE	
1	VTLADAGFM	808	816	9	HLA-A*02:01	808	18	0.00036728	18	0.00036728	18	TRUE	TRUE	
1	DGFLYVYKG	191	199	9	HLA-A*02:01	191	18	0.00036745	18	0.00036745	18	TRUE	TRUE	
1	RDLICAQKF	829	837	9	HLA-A*02:01	829	18	0.00037188	18	0.00037188	18	TRUE	TRUE	
1	IYQTSNFRV	299	307	9	HLA-A*02:01	299	18	0.00037342	18	0.00037342	18	TRUE	TRUE	
1	SVYAWERKK	336	344	9	HLA-A*02:01	336	19	0.0003787	19	0.0003787	19	TRUE	TRUE	
1	LLNAPATVC	503	511	9	HLA-A*02:01	503	19	0.00038133	19	0.00038133	19	TRUE	TRUE	
1	KLPLGINIT	221	229	9	HLA-A*02:01	221	19	0.00038637	19	0.00038637	19	TRUE	TRUE	
1	NLCPFGEVF	321	329	9	HLA-A*02:01	321	19	0.00038815	19	0.00038815	19	TRUE	TRUE	
1	WLGFIAGLI	1199	1207	9	HLA-A*02:01	1199	19	0.00039355	19	0.00039355	19	TRUE	TRUE	
1	RALSGIAAE	747	755	9	HLA-A*02:01	747	20	0.00040383	20	0.00040383	20	TRUE	TRUE	
1	IFRSDTLYL	46	54	9	HLA-A*02:01	46	20	0.00040448	20	0.00040448	20	TRUE	TRUE	
1	YQTSNFRVV	300	308	9	HLA-A*02:01	300	20	0.00040551	20	0.00040551	20	TRUE	TRUE	
1	GDISGINAS	1149	1157	9	HLA-A*02:01	1149	20	0.0004087	20	0.0004087	20	TRUE	TRUE	
1	GNYNYKYRY	434	442	9	HLA-A*02:01	434	20	0.00041191	20	0.00041191	20	TRUE	TRUE	
1	VTQNVLYEN	893	901	9	HLA-A*02:01	893	20	0.00041419	20	0.00041419	20	TRUE	TRUE	
1	QTYVTQQLI	987	995	9	HLA-A*02:01	987	20	0.00041976	20	0.00041976	20	TRUE	TRUE	
1	TKMSECVLG	1009	1017	9	HLA-A*02:01	1009	20	0.00042267	20	0.00042267	20	TRUE	TRUE	
1	YTAALVSGT	855	863	9	HLA-A*02:01	855	20	0.00042364	20	0.00042364	20	TRUE	TRUE	
1	IRASANLAA	1000	1008	9	HLA-A*02:01	1000	20	0.00042501	20	0.00042501	20	TRUE	TRUE	
1	LLRSTSQKS	665	673	9	HLA-A*02:01	665	20	0.0004256	20	0.0004256	20	TRUE	TRUE	
1	QDIWGTSAA	242	250	9	HLA-A*02:01	242	20	0.00042599	20	0.00042599	20	TRUE	TRUE	
1	RNFTTAPAI	1055	1063	9	HLA-A*02:01	1055	20	0.00042638	20	0.00042638	20	TRUE	TRUE	
1	YECDIPIGA	646	654	9	HLA-A*02:01	646	20	0.00042658	20	0.00042658	20	TRUE	TRUE	
1	VNIQKEIDR	1159	1167	9	HLA-A*02:01	1159	20	0.00042678	20	0.00042678	20	TRUE	TRUE	
1	PLQPELDSF	1122	1130	9	HLA-A*02:01	1122	21	0.00043934	21	0.00043934	21	TRUE	TRUE	
1	ISGINASVV	1151	1159	9	HLA-A*02:01	1151	21	0.00043934	21	0.00043934	21	TRUE	TRUE	
1	IITTDNTFV	1096	1104	9	HLA-A*02:01	1096	21	0.00043954	21	0.00043954	21	TRUE	TRUE	
1	TSWFITQRN	1082	1090	9	HLA-A*02:01	1082	21	0.00044055	21	0.00044055	21	TRUE	TRUE	
1	PWYVWLGFI	1195	1203	9	HLA-A*02:01	1195	21	0.00044096	21	0.00044096	21	TRUE	TRUE	
1	YVWLGFIAG	1197	1205	9	HLA-A*02:01	1197	21	0.00045082	21	0.00045082	21	TRUE	TRUE	
1	VDLGDISGI	1146	1154	9	HLA-A*02:01	1146	21	0.00045331	21	0.00045331	21	TRUE	TRUE	
1	KLSTDLIKN	514	522	9	HLA-A*02:01	514	21	0.00045415	21	0.00045415	21	TRUE	TRUE	
1	FQTQAGCLI	629	637	9	HLA-A*02:01	629	21	0.00045709	21	0.00045709	21	TRUE	TRUE	
1	FKCYGVSAT	364	372	9	HLA-A*02:01	364	21	0.00046026	21	0.00046026	21	TRUE	TRUE	
1	AYRFNGIGV	885	893	9	HLA-A*02:01	885	21	0.00046047	21	0.00046047	21	TRUE	TRUE	
1	FGRDVSDFT	551	559	9	HLA-A*02:01	551	21	0.00046452	21	0.00046452	21	TRUE	TRUE	
1	AKNLNESLI	1172	1180	9	HLA-A*02:01	1172	21	0.00046559	21	0.00046559	21	TRUE	TRUE	
1	SRLDKVEAE	964	972	9	HLA-A*02:01	964	21	0.00046817	21	0.00046817	21	TRUE	TRUE	
1	LQSLQTYVT	983	991	9	HLA-A*02:01	983	22	0.00046881	22	0.00046881	22	TRUE	TRUE	
1	KMSECVLGQ	1010	1018	9	HLA-A*02:01	1010	22	0.0004761	22	0.0004761	22	TRUE	TRUE	
1	DLIKNQCVN	518	526	9	HLA-A*02:01	518	22	0.00047797	22	0.00047797	22	TRUE	TRUE	
1	WGTSAAAYF	245	253	9	HLA-A*02:01	245	22	0.00047863	22	0.00047863	22	TRUE	TRUE	
1	CSFGGVSVI	576	584	9	HLA-A*02:01	576	22	0.00048843	22	0.00048843	22	TRUE	TRUE	
1	FMLKYDENG	262	270	9	HLA-A*02:01	262	22	0.00049204	22	0.00049204	22	TRUE	TRUE	
1	AILTAFSPA	233	241	9	HLA-A*02:01	233	22	0.00049317	22	0.00049317	22	TRUE	TRUE	
1	SNFRVVPSG	303	311	9	HLA-A*02:01	303	22	0.00049636	22	0.00049636	22	TRUE	TRUE	
1	IPTNFSISI	696	704	9	HLA-A*02:01	696	22	0.00049728	22	0.00049728	22	TRUE	TRUE	
1	FSPQIITTD	1092	1100	9	HLA-A*02:01	1092	22	0.00050338	22	0.00050338	22	TRUE	TRUE	
1	TQLNRALSG	743	751	9	HLA-A*02:01	743	23	0.0005105	23	0.0005105	23	TRUE	TRUE	
1	SLDVSEKSG	169	177	9	HLA-A*02:01	169	23	0.00051404	23	0.00051404	23	TRUE	TRUE	
1	FQPFQQFGR	545	553	9	HLA-A*02:01	545	23	0.0005157	23	0.0005157	23	TRUE	TRUE	
1	TLVKQLSSN	943	951	9	HLA-A*02:01	943	23	0.00051665	23	0.00051665	23	TRUE	TRUE	
1	TQRNFFSPQ	1087	1095	9	HLA-A*02:01	1087	23	0.00051844	23	0.00051844	23	TRUE	TRUE	
1	RAILTAFSP	232	240	9	HLA-A*02:01	232	23	0.00052638	23	0.00052638	23	TRUE	TRUE	
1	ANLAATKMS	1004	1012	9	HLA-A*02:01	1004	23	0.00053064	23	0.00053064	23	TRUE	TRUE	
1	GVSVITPGT	580	588	9	HLA-A*02:01	580	23	0.00053456	23	0.00053456	23	TRUE	TRUE	
1	MLKYDENGT	263	271	9	HLA-A*02:01	263	23	0.0005358	23	0.0005358	23	TRUE	TRUE	
1	SVLNDILSR	957	965	9	HLA-A*02:01	957	23	0.00053753	23	0.00053753	23	TRUE	TRUE	
1	RLNEVAKNL	1167	1175	9	HLA-A*02:01	1167	24	0.00054325	24	0.00054325	24	TRUE	TRUE	
1	AQALNTLVK	938	946	9	HLA-A*02:01	938	24	0.00054739	24	0.00054739	24	TRUE	TRUE	
1	KLPDDFMGC	411	419	9	HLA-A*02:01	411	24	0.00054866	24	0.00054866	24	TRUE	TRUE	
1	VFLHVTYVP	1043	1051	9	HLA-A*02:01	1043	24	0.00055081	24	0.00055081	24	TRUE	TRUE	
1	DVQAPNYTQ	24	32	9	HLA-A*02:01	24	24	0.00055195	24	0.00055195	24	TRUE	TRUE	
1	LTQDLFLPF	54	62	9	HLA-A*02:01	54	24	0.00055463	24	0.00055463	24	TRUE	TRUE	
1	VRFPNITNL	314	322	9	HLA-A*02:01	314	24	0.0005577	24	0.0005577	24	TRUE	TRUE	
1	IVMVTILLC	1209	1217	9	HLA-A*02:01	1209	24	0.00055886	24	0.00055886	24	TRUE	TRUE	
1	QDVNCTDVS	599	607	9	HLA-A*02:01	599	24	0.00056416	24	0.00056416	24	TRUE	TRUE	
1	SEVAVLYQD	592	600	9	HLA-A*02:01	592	24	0.00057161	24	0.00057161	24	TRUE	TRUE	
1	GYLKPTTFM	255	263	9	HLA-A*02:01	255	24	0.00057306	24	0.00057306	24	TRUE	TRUE	
1	SQKSIVAYT	670	678	9	HLA-A*02:01	670	24	0.00057412	24	0.00057412	24	TRUE	TRUE	
1	GIGYQPYRV	488	496	9	HLA-A*02:01	488	25	0.00058345	25	0.00058345	25	TRUE	TRUE	
1	CHEGKAYFP	1064	1072	9	HLA-A*02:01	1064	25	0.00058412	25	0.00058412	25	TRUE	TRUE	

seq #	peptide	start	end	peptide length	allele	peptide index	median binding	comblib_sidney	comblib_sidney	legacy IC50	legacy rank	scores equal	percentiles equal
1	ALNCYWPLN	471	479	9	HLA-A*02:01	471	25	0.00058506	25	0.00058506	25	TRUE	TRUE
1	CTFEYISDA	159	167	9	HLA-A*02:01	159	25	0.00058884	25	0.00058884	25	TRUE	TRUE
1	PQAAPHGVV	1035	1043	9	HLA-A*02:01	1035	25	0.00059814	25	0.00059814	25	TRUE	TRUE
1	SFELLNAPA	500	508	9	HLA-A*02:01	500	25	0.00060104	25	0.00060104	25	TRUE	TRUE
1	QTHTMIFDN	147	155	9	HLA-A*02:01	147	25	0.00060284	25	0.00060284	25	TRUE	TRUE
1	DTLYLTQDL	50	58	9	HLA-A*02:01	50	25	0.00060842	25	0.00060842	25	TRUE	TRUE
1	TKLNDLCFS	372	380	9	HLA-A*02:01	372	25	0.00061094	25	0.00061094	25	TRUE	TRUE
1	FCTQLNRAL	741	749	9	HLA-A*02:01	741	25	0.0006115	25	0.0006115	25	TRUE	TRUE
1	SCGSCCKFD	1231	1239	9	HLA-A*02:01	1231	25	0.00061165	25	0.00061165	25	TRUE	TRUE
1	STFFSTFKC	358	366	9	HLA-A*02:01	358	26	0.00061277	26	0.00061277	26	TRUE	TRUE
1	SMRGVYYPD	36	44	9	HLA-A*02:01	36	26	0.0006139	26	0.0006139	26	TRUE	TRUE
1	FFAVSKPMG	137	145	9	HLA-A*02:01	137	26	0.00061645	26	0.00061645	26	TRUE	TRUE
1	GIYFAATEK	86	94	9	HLA-A*02:01	86	26	0.00061987	26	0.00061987	26	TRUE	TRUE
1	FGGFNFSQI	779	787	9	HLA-A*02:01	779	26	0.00062087	26	0.00062087	26	TRUE	TRUE
1	VIGIINNTV	1111	1119	9	HLA-A*02:01	1111	26	0.00062087	26	0.00062087	26	TRUE	TRUE
1	TLTSGSDLD	9	17	9	HLA-A*02:01	9	26	0.00062101	26	0.00062101	26	TRUE	TRUE
1	GICASYHTV	655	663	9	HLA-A*02:01	655	26	0.0006223	26	0.0006223	26	TRUE	TRUE
1	IAGLIAIVM	1203	1211	9	HLA-A*02:01	1203	26	0.00062402	26	0.00062402	26	TRUE	TRUE
1	VWLGFIAGL	1198	1206	9	HLA-A*02:01	1198	26	0.0006246	26	0.0006246	26	TRUE	TRUE
1	CVLAWNTRN	419	427	9	HLA-A*02:01	419	26	0.0006269	26	0.0006269	26	TRUE	TRUE
1	NFELCDNPF	129	137	9	HLA-A*02:01	129	26	0.00062791	26	0.00062791	26	TRUE	TRUE
1	NLLLQYGSF	733	741	9	HLA-A*02:01	733	26	0.00062951	26	0.00062951	26	TRUE	TRUE
1	IINNTVYDP	1114	1122	9	HLA-A*02:01	1114	26	0.0006311	26	0.0006311	26	TRUE	TRUE
1	MSLGADSSI	679	687	9	HLA-A*02:01	679	26	0.00063212	26	0.00063212	26	TRUE	TRUE
1	TILLCCMTS	1213	1221	9	HLA-A*02:01	1213	26	0.00063241	26	0.00063241	26	TRUE	TRUE
1	DSTECANLL	727	735	9	HLA-A*02:01	727	26	0.00063533	26	0.00063533	26	TRUE	TRUE
1	FNKAISQIQ	909	917	9	HLA-A*02:01	909	26	0.00063606	26	0.00063606	26	TRUE	TRUE
1	FAQVKQMYK	764	772	9	HLA-A*02:01	764	26	0.00064047	26	0.00064047	26	TRUE	TRUE
1	EILDISPCS	569	577	9	HLA-A*02:01	569	26	0.00064565	26	0.00064565	26	TRUE	TRUE
1	FRVVPSGDV	305	313	9	HLA-A*02:01	305	27	0.00065826	27	0.00065826	27	TRUE	TRUE
1	RPFERDISN	449	457	9	HLA-A*02:01	449	27	0.00066039	27	0.00066039	27	TRUE	TRUE
1	TIAIPTNFS	693	701	9	HLA-A*02:01	693	27	0.00066222	27	0.00066222	27	TRUE	TRUE
1	YGFYTTTGI	481	489	9	HLA-A*02:01	481	27	0.00066222	27	0.00066222	27	TRUE	TRUE
1	NGTSWFITQ	1080	1088	9	HLA-A*02:01	1080	27	0.00066328	27	0.00066328	27	TRUE	TRUE
1	AVDCSQNPL	275	283	9	HLA-A*02:01	275	27	0.00066374	27	0.00066374	27	TRUE	TRUE
1	ERNFTTAPA	1054	1062	9	HLA-A*02:01	1054	27	0.00066589	27	0.00066589	27	TRUE	TRUE
1	IAIVMVTIL	1207	1215	9	HLA-A*02:01	1207	27	0.00067019	27	0.00067019	27	TRUE	TRUE
1	ASYHTVSLL	658	666	9	HLA-A*02:01	658	27	0.00067733	27	0.00067733	27	TRUE	TRUE
1	LQIPFAMQM	876	884	9	HLA-A*02:01	876	27	0.00067764	27	0.00067764	27	TRUE	TRUE
1	STGNYNYKY	432	440	9	HLA-A*02:01	432	27	0.0006792	27	0.0006792	27	TRUE	TRUE
1	EIDKGIYQT	294	302	9	HLA-A*02:01	294	27	0.00068077	27	0.00068077	27	TRUE	TRUE
1	ITNLCPFGE	319	327	9	HLA-A*02:01	319	27	0.00068234	27	0.00068234	27	TRUE	TRUE
1	TKRSFIEDL	795	803	9	HLA-A*02:01	795	27	0.00068391	27	0.00068391	27	TRUE	TRUE
1	VEAEVQIDR	969	977	9	HLA-A*02:01	969	27	0.00068612	27	0.00068612	27	TRUE	TRUE
1	IGYQPYRVV	489	497	9	HLA-A*02:01	489	28	0.00069024	28	0.00069024	28	TRUE	TRUE
1	PPALNCYWP	469	477	9	HLA-A*02:01	469	28	0.00069263	28	0.00069263	28	TRUE	TRUE
1	CYWPLNDYG	474	482	9	HLA-A*02:01	474	28	0.00069534	28	0.00069534	28	TRUE	TRUE
1	GIYQTSNFR	298	306	9	HLA-A*02:01	298	28	0.00069566	28	0.00069566	28	TRUE	TRUE
1	YGVSATKLN	367	375	9	HLA-A*02:01	367	28	0.0007034	28	0.0007034	28	TRUE	TRUE
1	SCCSCLKGA	1221	1229	9	HLA-A*02:01	1221	28	0.00070372	28	0.00070372	28	TRUE	TRUE
1	NKDGFLYVY	189	197	9	HLA-A*02:01	189	28	0.00070632	28	0.00070632	28	TRUE	TRUE
1	NKSQSVIII	109	117	9	HLA-A*02:01	109	28	0.00070632	28	0.00070632	28	TRUE	TRUE
1	YQDVNCTDV	598	606	9	HLA-A*02:01	598	28	0.00070795	28	0.00070795	28	TRUE	TRUE
1	VAYTMSLGA	675	683	9	HLA-A*02:01	675	28	0.00071812	28	0.00071812	28	TRUE	TRUE
1	ICGDSTECA	724	732	9	HLA-A*02:01	724	29	0.0007251	29	0.0007251	29	TRUE	TRUE
1	KISNCVADY	344	352	9	HLA-A*02:01	344	29	0.00072611	29	0.00072611	29	TRUE	TRUE
1	AGFMKQYGE	813	821	9	HLA-A*02:01	813	29	0.00072946	29	0.00072946	29	TRUE	TRUE
1	ILDISPCSF	570	578	9	HLA-A*02:01	570	29	0.00073451	29	0.00073451	29	TRUE	TRUE
1	QKQIANQFN	902	910	9	HLA-A*02:01	902	29	0.00073485	29	0.00073485	29	TRUE	TRUE
1	QHTSSMRGV	32	40	9	HLA-A*02:01	32	29	0.00073689	29	0.00073689	29	TRUE	TRUE
1	YYPDEIFRS	41	49	9	HLA-A*02:01	41	29	0.00073824	29	0.00073824	29	TRUE	TRUE
1	VLPPLLTDD	842	850	9	HLA-A*02:01	842	29	0.00073978	29	0.00073978	29	TRUE	TRUE
1	QQLIRAAEI	992	1000	9	HLA-A*02:01	992	29	0.00074302	29	0.00074302	29	TRUE	TRUE
1	LNAPATVCG	504	512	9	HLA-A*02:01	504	29	0.00074439	29	0.00074439	29	TRUE	TRUE
1	TMIFDNAFN	150	158	9	HLA-A*02:01	150	29	0.00074679	29	0.00074679	29	TRUE	TRUE
1	LTPAWRIYS	615	623	9	HLA-A*02:01	615	29	0.00074817	29	0.00074817	29	TRUE	TRUE
1	VNFNFNGLT	525	533	9	HLA-A*02:01	525	29	0.00075301	29	0.00075301	29	TRUE	TRUE
1	VFNATKFPS	328	336	9	HLA-A*02:01	328	29	0.00075492	29	0.00075492	29	TRUE	TRUE
1	GAGICASYH	653	661	9	HLA-A*02:01	653	29	0.00075753	29	0.00075753	29	TRUE	TRUE
1	SLGADSSIA	680	688	9	HLA-A*02:01	680	29	0.00075858	29	0.00075858	29	TRUE	TRUE
1	YRFNGIGVT	886	894	9	HLA-A*02:01	886	30	0.00076454	30	0.00076454	30	TRUE	TRUE
1	CLKGACSCG	1225	1233	9	HLA-A*02:01	1225	30	0.0007656	30	0.0007656	30	TRUE	TRUE
1	CPFGEVFNA	323	331	9	HLA-A*02:01	323	30	0.00076666	30	0.00076666	30	TRUE	TRUE
1	GTGVLTPSS	534	542	9	HLA-A*02:01	534	30	0.0007709	30	0.0007709	30	TRUE	TRUE
1	AYFPREGVF	1069	1077	9	HLA-A*02:01	1069	30	0.00077304	30	0.00077304	30	TRUE	TRUE
1	FVFKNKDGF	185	193	9	HLA-A*02:01	185	30	0.00077625	30	0.00077625	30	TRUE	TRUE
1	YKGYQPIDV	197	205	9	HLA-A*02:01	197	30	0.00077625	30	0.00077625	30	TRUE	TRUE
1	YADSFVVKG	383	391	9	HLA-A*02:01	383	30	0.0007766	30	0.0007766	30	TRUE	TRUE
1	STMNNKSQS	105	113	9	HLA-A*02:01	105	30	0.00078705	30	0.00078705	30	TRUE	TRUE
1	PFFAVSKPM	136	144	9	HLA-A*02:01	136	30	0.00078832	30	0.00078832	30	TRUE	TRUE
1	KGYHLMSFP	1027	1035	9	HLA-A*02:01	1027	30	0.00079086	30	0.00079086	30	TRUE	TRUE
1	SFIEDLLFN	798	806	9	HLA-A*02:01	798	30	0.00080002	30	0.00080002	30	TRUE	TRUE
1	PLGINITNF	223	231	9	HLA-A*02:01	223	30	0.00080316	30	0.00080316	30	TRUE	TRUE
1	DVDLGDISG	1145	1153	9	HLA-A*02:01	1145	30	0.00080575	30	0.00080575	30	TRUE	TRUE
1	EVAVLYQDV	593	601	9	HLA-A*02:01	593	30	0.00080724	30	0.00080724	30	TRUE	TRUE
1	FVGYLKPTT	253	261	9	HLA-A*02:01	253	31	0.0008147	31	0.0008147	31	TRUE	TRUE
1	NHTFGNPVI	73	81	9	HLA-A*02:01	73	31	0.00081546	31	0.00081546	31	TRUE	TRUE
1	GKYEQYIKW	1186	1194	9	HLA-A*02:01	1186	31	0.00081979	31	0.00081979	31	TRUE	TRUE
1	DLLFNKVTL	802	810	9	HLA-A*02:01	802	31	0.00082073	31	0.00082073	31	TRUE	TRUE
1	KYFGGFNFS	777	785	9	HLA-A*02:01	777	31	0.00082073	31	0.00082073	31	TRUE	TRUE
1	SQILPDPLK	785	793	9	HLA-A*02:01	785	31	0.00082851	31	0.00082851	31	TRUE	TRUE
1	KPIFKLPLG	217	225	9	HLA-A*02:01	217	31	0.00082909	31	0.00082909	31	TRUE	TRUE
1	VVNQNAQAL	933	941	9	HLA-A*02:01	933	31	0.00083176	31	0.00083176	31	TRUE	TRUE
1	IPFKDGIYF	81	89	9	HLA-A*02:01	81	31	0.00083676	31	0.00083676	31	TRUE	TRUE
1	FMKQYGECL	815	823	9	HLA-A*02:01	815	31	0.00083946	31	0.00083946	31	TRUE	TRUE
1	NTLKPIFKL	214	222	9	HLA-A*02:01	214	31	0.0008414	31	0.0008414	31	TRUE	TRUE
1	IQESLTTTS	916	924	9	HLA-A*02:01	916	31	0.0008414	31	0.0008414	31	TRUE	TRUE
1	GAISSVLND	953	961	9	HLA-A*02:01	953	31	0.00084392	31	0.00084392	31	TRUE	TRUE
1	AAYFVGYLK	250	258	9	HLA-A*02:01	250	31	0.00084431	31	0.00084431	31	TRUE	TRUE
1	FERDISNVP	451	459	9	HLA-A*02:01	451	31	0.00084742	31	0.00084742	31	TRUE	TRUE
1	SPAQDIWGT	239	247	9	HLA-A*02:01	239	32	0.00085428	32	0.00085428	32	TRUE	TRUE
1	DSEPVLKGV	1242	1250	9	HLA-A*02:01	1242	32	0.00086099	32	0.00086099	32	TRUE	TRUE

seq #	peptide	start	end	peptide length	allele	peptide index	median binding	comblib_sidney	comblib_sidney	legacy IC50	legacy rank	scores equal	percentiles equal	
1	CGDSTECAN	725	733	9	HLA-A*02:01	725	32	0.00086338	32	0.00086338	32	TRUE	TRUE	
1	SQNPLAELK	279	287	9	HLA-A*02:01	279	32	0.00086756	32	0.00086756	32	TRUE	TRUE	
1	ERDISNVPF	452	460	9	HLA-A*02:01	452	32	0.00086976	32	0.00086976	32	TRUE	TRUE	
1	INNSTNVVI	117	125	9	HLA-A*02:01	117	32	0.00087862	32	0.00087862	32	TRUE	TRUE	
1	YTMSLGADS	677	685	9	HLA-A*02:01	677	32	0.00087902	32	0.00087902	32	TRUE	TRUE	
1	FYTTTGIGY	483	491	9	HLA-A*02:01	483	32	0.00089166	32	0.00089166	32	TRUE	TRUE	
1	MFIFLLFLT	1	9	9	HLA-A*02:01	1	32	0.0008931	32	0.0008931	32	TRUE	TRUE	
1	YNYKYRYLR	436	444	9	HLA-A*02:01	436	32	0.00089578	32	0.00089578	32	TRUE	TRUE	
1	EQYIKWPWY	1189	1197	9	HLA-A*02:01	1189	32	0.00089743	32	0.00089743	32	TRUE	TRUE	
1	DISPCSFGG	572	580	9	HLA-A*02:01	572	33	0.00090615	33	0.00090615	33	TRUE	TRUE	
1	SEKSGNFKH	173	181	9	HLA-A*02:01	173	33	0.00091664	33	0.00091664	33	TRUE	TRUE	
1	WVFGSTMNN	101	109	9	HLA-A*02:01	101	33	0.00091664	33	0.00091664	33	TRUE	TRUE	
1	DVRQIAPGQ	393	401	9	HLA-A*02:01	393	33	0.00091812	33	0.00091812	33	TRUE	TRUE	
1	LGADSSIAY	681	689	9	HLA-A*02:01	681	33	0.00092257	33	0.00092257	33	TRUE	TRUE	
1	VDTSYECDI	642	650	9	HLA-A*02:01	642	33	0.00092555	33	0.00092555	33	TRUE	TRUE	
1	RSTSQKSIV	667	675	9	HLA-A*02:01	667	33	0.0009264	33	0.0009264	33	TRUE	TRUE	
1	TTAPAICHE	1058	1066	9	HLA-A*02:01	1058	33	0.00093325	33	0.00093325	33	TRUE	TRUE	
1	FHTINHTFG	69	77	9	HLA-A*02:01	69	33	0.00093411	33	0.00093411	33	TRUE	TRUE	
1	AISSVLNDI	954	962	9	HLA-A*02:01	954	33	0.00093972	33	0.00093972	33	TRUE	TRUE	
1	PKTSEILDI	565	573	9	HLA-A*02:01	565	33	0.00094363	33	0.00094363	33	TRUE	TRUE	
1	IPIGAGICA	650	658	9	HLA-A*02:01	650	34	0.00094755	34	0.00094755	34	TRUE	TRUE	
1	STGNNVFQT	623	631	9	HLA-A*02:01	623	34	0.0009528	34	0.0009528	34	TRUE	TRUE	
1	TNLCPFGEV	320	328	9	HLA-A*02:01	320	34	0.00095455	34	0.00095455	34	TRUE	TRUE	
1	GGFNFSQIL	780	788	9	HLA-A*02:01	780	34	0.00095499	34	0.00095499	34	TRUE	TRUE	
1	ENGTITDAV	268	276	9	HLA-A*02:01	268	34	0.00095675	34	0.00095675	34	TRUE	TRUE	
1	QCVNFNFNG	523	531	9	HLA-A*02:01	523	34	0.00095808	34	0.00095808	34	TRUE	TRUE	
1	FSNVYADSF	379	387	9	HLA-A*02:01	379	34	0.00097006	34	0.00097006	34	TRUE	TRUE	
1	SQSVIIINN	111	119	9	HLA-A*02:01	111	34	0.00097096	34	0.00097096	34	TRUE	TRUE	
1	QAPNYTQHT	26	34	9	HLA-A*02:01	26	34	0.0009732	34	0.0009732	34	TRUE	TRUE	
1	EHVDTSYEC	640	648	9	HLA-A*02:01	640	34	0.00097432	34	0.00097432	34	TRUE	TRUE	
1	INASVVNIQ	1154	1162	9	HLA-A*02:01	1154	34	0.00097611	34	0.00097611	34	TRUE	TRUE	
1	RFPNITNLC	315	323	9	HLA-A*02:01	315	34	0.00097994	34	0.00097994	34	TRUE	TRUE	
1	YVTQQLIRA	989	997	9	HLA-A*02:01	989	34	0.00098175	34	0.00098175	34	TRUE	TRUE	
1	KQYGECLGD	817	825	9	HLA-A*02:01	817	34	0.00099334	34	0.00099334	34	TRUE	TRUE	
1	DAVDCSQNP	274	282	9	HLA-A*02:01	274	34	0.00099426	34	0.00099426	34	TRUE	TRUE	
1	SVVNIQKEI	1157	1165	9	HLA-A*02:01	1157	35	0.00100231	35	0.00100231	35	TRUE	TRUE	
1	DQLTPAWRI	613	621	9	HLA-A*02:01	613	35	0.00100508	35	0.00100508	35	TRUE	TRUE	
1	SSNFGAISS	949	957	9	HLA-A*02:01	949	35	0.00100647	35	0.00100647	35	TRUE	TRUE	
1	CANLLLQYG	731	739	9	HLA-A*02:01	731	35	0.00101906	35	0.00101906	35	TRUE	TRUE	
1	FMGCVLAWN	416	424	9	HLA-A*02:01	416	35	0.00102141	35	0.00102141	35	TRUE	TRUE	
1	SSKRFQPFQ	541	549	9	HLA-A*02:01	541	35	0.0010292	35	0.0010292	35	TRUE	TRUE	
1	NNTIAIPTN	691	699	9	HLA-A*02:01	691	35	0.00103276	35	0.00103276	35	TRUE	TRUE	
1	ITNFRAILT	228	236	9	HLA-A*02:01	228	35	0.00103992	35	0.00103992	35	TRUE	TRUE	
1	VLSFELLNA	498	506	9	HLA-A*02:01	498	35	0.00104232	35	0.00104232	35	TRUE	TRUE	
1	LKCSVKSFE	286	294	9	HLA-A*02:01	286	35	0.00104232	35	0.00104232	35	TRUE	TRUE	
1	VAKNLNESL	1171	1179	9	HLA-A*02:01	1171	35	0.00104761	35	0.00104761	35	TRUE	TRUE	
1	AEHVDTSYE	639	647	9	HLA-A*02:01	639	35	0.00105196	35	0.00105196	35	TRUE	TRUE	
1	GYQPYRVVV	490	498	9	HLA-A*02:01	490	35	0.00105487	35	0.00105487	35	TRUE	TRUE	
1	LFLTLTSGS	6	14	9	HLA-A*02:01	6	35	0.00105657	35	0.00105657	35	TRUE	TRUE	
1	APHGVVFLH	1038	1046	9	HLA-A*02:01	1038	35	0.00105877	35	0.00105877	35	TRUE	TRUE	
1	PNITNLCPF	317	325	9	HLA-A*02:01	317	36	0.00106316	36	0.00106316	36	TRUE	TRUE	
1	QKFNGLTVL	835	843	9	HLA-A*02:01	835	36	0.00106905	36	0.00106905	36	TRUE	TRUE	
1	DVVIGIINN	1109	1117	9	HLA-A*02:01	1109	36	0.00107498	36	0.00107498	36	TRUE	TRUE	
1	FGEVFNATK	325	333	9	HLA-A*02:01	325	36	0.00107721	36	0.00107721	36	TRUE	TRUE	
1	NFKHLREFV	178	186	9	HLA-A*02:01	178	36	0.0010787	36	0.0010787	36	TRUE	TRUE	
1	VITPGTNAS	583	591	9	HLA-A*02:01	583	36	0.00107895	36	0.00107895	36	TRUE	TRUE	
1	GVTQNVLYE	892	900	9	HLA-A*02:01	892	36	0.00108393	36	0.00108393	36	TRUE	TRUE	
1	KKISNCVAD	343	351	9	HLA-A*02:01	343	36	0.00108418	36	0.00108418	36	TRUE	TRUE	
1	EVMPVSMAK	707	715	9	HLA-A*02:01	707	36	0.00108468	36	0.00108468	36	TRUE	TRUE	
1	LQTYVTQQL	986	994	9	HLA-A*02:01	986	36	0.00108643	36	0.00108643	36	TRUE	TRUE	
1	TLKPIFKLP	215	223	9	HLA-A*02:01	215	36	0.00108668	36	0.00108668	36	TRUE	TRUE	
1	NYTQHTSSM	29	37	9	HLA-A*02:01	29	36	0.00109698	36	0.00109698	36	TRUE	TRUE	
1	SFPQAAPHG	1033	1041	9	HLA-A*02:01	1033	36	0.00110129	36	0.00110129	36	TRUE	TRUE	
1	DLQELGKYE	1181	1189	9	HLA-A*02:01	1181	36	0.00110459	36	0.00110459	36	TRUE	TRUE	
1	VVIGIINNT	1110	1118	9	HLA-A*02:01	1110	36	0.00110917	36	0.00110917	36	TRUE	TRUE	
1	ESLIDLQEL	1177	1185	9	HLA-A*02:01	1177	36	0.00111122	36	0.00111122	36	TRUE	TRUE	
1	GRLQSLQTY	981	989	9	HLA-A*02:01	981	36	0.00111532	36	0.00111532	36	TRUE	TRUE	
1	RVVPSGDVV	306	314	9	HLA-A*02:01	306	37	0.00114288	37	0.00114288	37	TRUE	TRUE	
1	SGDVVRFPN	310	318	9	HLA-A*02:01	310	37	0.0011434	37	0.0011434	37	TRUE	TRUE	
1	ASANLAATK	1002	1010	9	HLA-A*02:01	1002	37	0.00114578	37	0.00114578	37	TRUE	TRUE	
1	DKVEAEVQI	967	975	9	HLA-A*02:01	967	37	0.00114868	37	0.00114868	37	TRUE	TRUE	
1	VVIRACNFE	123	131	9	HLA-A*02:01	123	37	0.00115345	37	0.00115345	37	TRUE	TRUE	
1	FPNITNLCP	316	324	9	HLA-A*02:01	316	37	0.00115798	37	0.00115798	37	TRUE	TRUE	
1	GACSCGSCC	1228	1236	9	HLA-A*02:01	1228	37	0.00116011	37	0.00116011	37	TRUE	TRUE	
1	PFERDISNV	450	458	9	HLA-A*02:01	450	37	0.00116332	37	0.00116332	37	TRUE	TRUE	
1	SSVLNDILS	956	964	9	HLA-A*02:01	956	38	0.0011825	38	0.0011825	38	TRUE	TRUE	
1	AALQIPFAM	874	882	9	HLA-A*02:01	874	38	0.00118359	38	0.00118359	38	TRUE	TRUE	
1	VTGFHTINH	66	74	9	HLA-A*02:01	66	38	0.00120282	38	0.00120282	38	TRUE	TRUE	
1	PSSKRFQPF	540	548	9	HLA-A*02:01	540	38	0.00121227	38	0.00121227	38	TRUE	TRUE	
1	NTREVFAQV	759	767	9	HLA-A*02:01	759	38	0.00121899	38	0.00121899	38	TRUE	TRUE	
1	NMYICGDST	721	729	9	HLA-A*02:01	721	38	0.00122462	38	0.00122462	38	TRUE	TRUE	
1	YSTGNNVFQ	622	630	9	HLA-A*02:01	622	38	0.00122603	38	0.00122603	38	TRUE	TRUE	
1	TSAAAYFVG	247	255	9	HLA-A*02:01	247	38	0.00122687	38	0.00122687	38	TRUE	TRUE	
1	TNFRAILTA	229	237	9	HLA-A*02:01	229	39	0.0012297	39	0.0012297	39	TRUE	TRUE	
1	LLQYGSFCT	735	743	9	HLA-A*02:01	735	39	0.0012331	39	0.0012331	39	TRUE	TRUE	
1	NASSEVAVL	589	597	9	HLA-A*02:01	589	39	0.00123367	39	0.00123367	39	TRUE	TRUE	
1	TPPALNCYW	468	476	9	HLA-A*02:01	468	39	0.00123396	39	0.00123396	39	TRUE	TRUE	
1	YFAATEKSN	88	96	9	HLA-A*02:01	88	39	0.00123623	39	0.00123623	39	TRUE	TRUE	
1	GINASVVNI	1153	1161	9	HLA-A*02:01	1153	39	0.00124165	39	0.00124165	39	TRUE	TRUE	
1	FKEELDKYF	1130	1138	9	HLA-A*02:01	1130	39	0.00124738	39	0.00124738	39	TRUE	TRUE	
1	LQDVVNQNA	930	938	9	HLA-A*02:01	930	39	0.00124738	39	0.00124738	39	TRUE	TRUE	
1	NPFFAVSKP	135	143	9	HLA-A*02:01	135	39	0.0012494	39	0.0012494	39	TRUE	TRUE	
1	RQIAPGQTG	395	403	9	HLA-A*02:01	395	39	0.00125314	39	0.00125314	39	TRUE	TRUE	
1	VFNGTSWFI	1078	1086	9	HLA-A*02:01	1078	39	0.00127028	39	0.00127028	39	TRUE	TRUE	
1	ESLTTTSTA	918	926	9	HLA-A*02:01	918	39	0.00127585	39	0.00127585	39	TRUE	TRUE	
1	TTTSTALGK	921	929	9	HLA-A*02:01	921	39	0.00127732	39	0.00127732	39	TRUE	TRUE	
1	YENQKQIAN	899	907	9	HLA-A*02:01	899	40	0.00127997	40	0.00127997	40	TRUE	TRUE	
1	SLQTYVTQQ	985	993	9	HLA-A*02:01	985	40	0.00128145	40	0.00128145	40	TRUE	TRUE	
1	AATEKSNVV	90	98	9	HLA-A*02:01	90	40	0.00128292	40	0.00128292	40	TRUE	TRUE	
1	LPDPLKPTK	788	796	9	HLA-A*02:01	788	40	0.00129688	40	0.00129688	40	TRUE	TRUE	
1	AISQIQESL	912	920	9	HLA-A*02:01	912	40	0.00129718	40	0.00129718	40	TRUE	TRUE	
1	TQTHTMIFD	146	154	9	HLA-A*02:01	146	40	0.00129748	40	0.00129748	40	TRUE	TRUE	

seq #	peptide	start	end	peptide length	allele	peptide index	median binding	comblib_sidne	comblib_sidne	legacy IC50	legacy rank	scores equal	percentiles equal
1	GDDVRQIAP	391	399	9	HLA-A*02:01	391	40	0.00130768	40	0.00130768	40	TRUE	TRUE
1	LIDLQELGK	1179	1187	9	HLA-A*02:01	1179	40	0.00131009	40	0.00131009	40	TRUE	TRUE
1	CVLGQSKRV	1014	1022	9	HLA-A*02:01	1014	40	0.00131522	40	0.00131522	40	TRUE	TRUE
1	YISDAFSLD	163	171	9	HLA-A*02:01	163	40	0.00131856	40	0.00131856	40	TRUE	TRUE
1	GVIADYNYK	403	411	9	HLA-A*02:01	403	40	0.00132221	40	0.00132221	40	TRUE	TRUE
1	LYNSTFFST	355	363	9	HLA-A*02:01	355	41	0.00132801	41	0.00132801	41	TRUE	TRUE
1	QPIDVVRDL	201	209	9	HLA-A*02:01	201	41	0.00133537	41	0.00133537	41	TRUE	TRUE
1	YFGGFNFSQ	778	786	9	HLA-A*02:01	778	41	0.00133537	41	0.00133537	41	TRUE	TRUE
1	DAGFMKQYG	812	820	9	HLA-A*02:01	812	41	0.00133783	41	0.00133783	41	TRUE	TRUE
1	YSVLYNSTF	352	360	9	HLA-A*02:01	352	41	0.00133906	41	0.00133906	41	TRUE	TRUE
1	AAAYFVGYL	249	257	9	HLA-A*02:01	249	41	0.00134338	41	0.00134338	41	TRUE	TRUE
1	AAYTAALVS	853	861	9	HLA-A*02:01	853	41	0.00134958	41	0.00134958	41	TRUE	TRUE
1	PSGDVVRFP	309	317	9	HLA-A*02:01	309	41	0.00136364	41	0.00136364	41	TRUE	TRUE
1	FKNKDGFLY	187	195	9	HLA-A*02:01	187	41	0.00136773	41	0.00136773	41	TRUE	TRUE
1	TSNFRVVPS	302	310	9	HLA-A*02:01	302	41	0.00137658	41	0.00137658	41	TRUE	TRUE
1	ALNTLVKQL	940	948	9	HLA-A*02:01	940	41	0.00137721	41	0.00137721	41	TRUE	TRUE
1	NRALSGIAA	746	754	9	HLA-A*02:01	746	42	0.00138484	42	0.00138484	42	TRUE	TRUE
1	QDLFLPFYS	56	64	9	HLA-A*02:01	56	42	0.00138484	42	0.00138484	42	TRUE	TRUE
1	FNGIGVTQN	888	896	9	HLA-A*02:01	888	42	0.00138995	42	0.00138995	42	TRUE	TRUE
1	KGIYQTSNF	297	305	9	HLA-A*02:01	297	42	0.00140281	42	0.00140281	42	TRUE	TRUE
1	SVIIINNST	113	121	9	HLA-A*02:01	113	42	0.00140281	42	0.00140281	42	TRUE	TRUE
1	TLKYFGGFN	775	783	9	HLA-A*02:01	775	42	0.00140346	42	0.00140346	42	TRUE	TRUE
1	DRLNEVAKN	1166	1174	9	HLA-A*02:01	1166	42	0.0014054	42	0.0014054	42	TRUE	TRUE
1	TGTGVLTPS	533	541	9	HLA-A*02:01	533	42	0.00140929	42	0.00140929	42	TRUE	TRUE
1	KVEAEVQID	968	976	9	HLA-A*02:01	968	42	0.00141286	42	0.00141286	42	TRUE	TRUE
1	IFLLFLTLT	3	11	9	HLA-A*02:01	3	42	0.00141547	42	0.00141547	42	TRUE	TRUE
1	EGKAYFPRE	1066	1074	9	HLA-A*02:01	1066	42	0.00141906	42	0.00141906	42	TRUE	TRUE
1	NVLYENQKQ	896	904	9	HLA-A*02:01	896	42	0.00142135	42	0.00142135	42	TRUE	TRUE
1	VVRGWVFGS	97	105	9	HLA-A*02:01	97	42	0.00142233	42	0.00142233	42	TRUE	TRUE
1	TECANLLLQ	729	737	9	HLA-A*02:01	729	42	0.00142462	42	0.00142462	42	TRUE	TRUE
1	TVLPPLLTD	841	849	9	HLA-A*02:01	841	42	0.00142922	42	0.00142922	42	TRUE	TRUE
1	NVFQTQAGC	627	635	9	HLA-A*02:01	627	42	0.00144644	42	0.00144644	42	TRUE	TRUE
1	KPMGTQTHT	142	150	9	HLA-A*02:01	142	42	0.00145077	42	0.00145077	42	TRUE	TRUE
1	CDIPIGAGI	648	656	9	HLA-A*02:01	648	43	0.0014669	43	0.0014669	43	TRUE	TRUE
1	VIIINNSTN	114	122	9	HLA-A*02:01	114	43	0.00147299	43	0.00147299	43	TRUE	TRUE
1	LITGRLQSL	978	986	9	HLA-A*02:01	978	43	0.00147571	43	0.00147571	43	TRUE	TRUE
1	SDLDRCTTF	14	22	9	HLA-A*02:01	14	43	0.00149417	43	0.00149417	43	TRUE	TRUE
1	QFNKAISQI	908	916	9	HLA-A*02:01	908	43	0.0015028	43	0.0015028	43	TRUE	TRUE
1	IRAAEIRAS	995	1003	9	HLA-A*02:01	995	43	0.001508	43	0.001508	43	TRUE	TRUE
1	RACNFELCD	126	134	9	HLA-A*02:01	126	44	0.0015181	44	0.0015181	44	TRUE	TRUE
1	GAGAALQIP	871	879	9	HLA-A*02:01	871	44	0.00153568	44	0.00153568	44	TRUE	TRUE
1	TDDMIAAYT	848	856	9	HLA-A*02:01	848	44	0.00154668	44	0.00154668	44	TRUE	TRUE
1	YRVVVLSFE	494	502	9	HLA-A*02:01	494	44	0.00155024	44	0.00155024	44	TRUE	TRUE
1	VSGNCDVVI	1104	1112	9	HLA-A*02:01	1104	44	0.00155167	44	0.00155167	44	TRUE	TRUE
1	LHVTYVPSQ	1045	1053	9	HLA-A*02:01	1045	44	0.00155632	44	0.00155632	44	TRUE	TRUE
1	DAFSLDVSE	166	174	9	HLA-A*02:01	166	44	0.0015574	44	0.0015574	44	TRUE	TRUE
1	NKAISQIQE	910	918	9	HLA-A*02:01	910	44	0.00155955	44	0.00155955	44	TRUE	TRUE
1	LSFELLNAP	499	507	9	HLA-A*02:01	499	44	0.00156279	44	0.00156279	44	TRUE	TRUE
1	AFSLDVSEK	167	175	9	HLA-A*02:01	167	44	0.00156387	44	0.00156387	44	TRUE	TRUE
1	QNAQALNTL	936	944	9	HLA-A*02:01	936	44	0.00156603	44	0.00156603	44	TRUE	TRUE
1	NFSQILPDP	783	791	9	HLA-A*02:01	783	44	0.00157036	44	0.00157036	44	TRUE	TRUE
1	NCYWPLNDY	473	481	9	HLA-A*02:01	473	45	0.00158271	45	0.00158271	45	TRUE	TRUE
1	GDINARDLI	824	832	9	HLA-A*02:01	824	45	0.00158271	45	0.00158271	45	TRUE	TRUE
1	YFPREGVFV	1070	1078	9	HLA-A*02:01	1070	45	0.00158453	45	0.00158453	45	TRUE	TRUE
1	TEKSNVVRG	92	100	9	HLA-A*02:01	92	45	0.00159221	45	0.00159221	45	TRUE	TRUE
1	VLTPSSKRF	537	545	9	HLA-A*02:01	537	45	0.00159588	45	0.00159588	45	TRUE	TRUE
1	IKWPWYVWL	1192	1200	9	HLA-A*02:01	1192	45	0.00161436	45	0.00161436	45	TRUE	TRUE
1	GPKLSTDLI	512	520	9	HLA-A*02:01	512	45	0.0016278	45	0.0016278	45	TRUE	TRUE
1	PSQERNFTT	1051	1059	9	HLA-A*02:01	1051	45	0.00163155	45	0.00163155	45	TRUE	TRUE
1	QKSIVAYTM	671	679	9	HLA-A*02:01	671	45	0.00163682	45	0.00163682	45	TRUE	TRUE
1	SNVPFSPDG	456	464	9	HLA-A*02:01	456	46	0.00164361	46	0.00164361	46	TRUE	TRUE
1	IDRLITGRL	975	983	9	HLA-A*02:01	975	46	0.0016573	46	0.0016573	46	TRUE	TRUE
1	DLGDISGIN	1147	1155	9	HLA-A*02:01	1147	46	0.0016573	46	0.0016573	46	TRUE	TRUE
1	EVFAQVKQM	762	770	9	HLA-A*02:01	762	46	0.00166341	46	0.00166341	46	TRUE	TRUE
1	AEQDRNTRE	754	762	9	HLA-A*02:01	754	46	0.00166725	46	0.00166725	46	TRUE	TRUE
1	DKYFKNHTS	1135	1143	9	HLA-A*02:01	1135	46	0.00166802	46	0.00166802	46	TRUE	TRUE
1	DMIAAYTAA	850	858	9	HLA-A*02:01	850	46	0.00166802	46	0.00166802	46	TRUE	TRUE
1	KWPWYVWLG	1193	1201	9	HLA-A*02:01	1193	46	0.0016734	46	0.0016734	46	TRUE	TRUE
1	ITTEVMPVS	704	712	9	HLA-A*02:01	704	46	0.00167494	46	0.00167494	46	TRUE	TRUE
1	QYGECLGDI	818	826	9	HLA-A*02:01	818	46	0.00167958	46	0.00167958	46	TRUE	TRUE
1	HTSSMRGVY	33	41	9	HLA-A*02:01	33	46	0.00167958	46	0.00167958	46	TRUE	TRUE
1	GLTGTGVLT	531	539	9	HLA-A*02:01	531	46	0.00168267	46	0.00168267	46	TRUE	TRUE
1	FGAISSVLN	952	960	9	HLA-A*02:01	952	46	0.00169122	46	0.00169122	46	TRUE	TRUE
1	YHLMSFPQA	1029	1037	9	HLA-A*02:01	1029	46	0.001692	46	0.001692	46	TRUE	TRUE
1	KGDDVRQIA	390	398	9	HLA-A*02:01	390	46	0.00169434	46	0.00169434	46	TRUE	TRUE
1	QPYRVVVLS	492	500	9	HLA-A*02:01	492	46	0.00169668	46	0.00169668	46	TRUE	TRUE
1	YKLPDDFMG	410	418	9	HLA-A*02:01	410	46	0.00169824	46	0.00169824	46	TRUE	TRUE
1	GECLGDINA	820	828	9	HLA-A*02:01	820	46	0.00170216	46	0.00170216	46	TRUE	TRUE
1	TFGAGAALQ	869	877	9	HLA-A*02:01	869	46	0.00170451	46	0.00170451	46	TRUE	TRUE
1	DDVRQIAPG	392	400	9	HLA-A*02:01	392	46	0.00170844	46	0.00170844	46	TRUE	TRUE
1	SIAYSNNTI	686	694	9	HLA-A*02:01	686	47	0.00171002	47	0.00171002	47	TRUE	TRUE
1	KEELDKYFK	1131	1139	9	HLA-A*02:01	1131	47	0.0017112	47	0.0017112	47	TRUE	TRUE
1	AYTAALVSG	854	862	9	HLA-A*02:01	854	47	0.00171475	47	0.00171475	47	TRUE	TRUE
1	KRFQPFQQF	543	551	9	HLA-A*02:01	543	47	0.00171554	47	0.00171554	47	TRUE	TRUE
1	YWPLNDYGF	475	483	9	HLA-A*02:01	475	47	0.00172822	47	0.00172822	47	TRUE	TRUE
1	RLITGRLQS	977	985	9	HLA-A*02:01	977	47	0.00172982	47	0.00172982	47	TRUE	TRUE
1	LRHGKLRPF	443	451	9	HLA-A*02:01	443	47	0.00173141	47	0.00173141	47	TRUE	TRUE
1	YFVGYLKPT	252	260	9	HLA-A*02:01	252	47	0.0017374	47	0.0017374	47	TRUE	TRUE
1	EELDKYFKN	1132	1140	9	HLA-A*02:01	1132	47	0.00174663	47	0.00174663	47	TRUE	TRUE
1	ASVVNIQKE	1156	1164	9	HLA-A*02:01	1156	47	0.00174904	47	0.00174904	47	TRUE	TRUE
1	PTTFMLKYD	259	267	9	HLA-A*02:01	259	47	0.00175752	47	0.00175752	47	TRUE	TRUE
1	VTYVPSQER	1047	1055	9	HLA-A*02:01	1047	47	0.00177174	47	0.00177174	47	TRUE	TRUE
1	NNVFQTQAG	626	634	9	HLA-A*02:01	626	47	0.00177337	47	0.00177337	47	TRUE	TRUE
1	INARDLICA	826	834	9	HLA-A*02:01	826	48	0.00179391	48	0.00179391	48	TRUE	TRUE
1	TYVPSQERN	1048	1056	9	HLA-A*02:01	1048	48	0.00180053	48	0.00180053	48	TRUE	TRUE
1	APAICHEGK	1060	1068	9	HLA-A*02:01	1060	48	0.00184035	48	0.00184035	48	TRUE	TRUE
1	HEGKAYFPR	1065	1073	9	HLA-A*02:01	1065	48	0.00184332	48	0.00184332	48	TRUE	TRUE
1	CSQNPLAEL	278	286	9	HLA-A*02:01	278	48	0.00185695	48	0.00185695	48	TRUE	TRUE
1	EIRASANLA	999	1007	9	HLA-A*02:01	999	48	0.0018578	48	0.0018578	48	TRUE	TRUE
1	VNQNAQALN	934	942	9	HLA-A*02:01	934	48	0.0018578	48	0.0018578	48	TRUE	TRUE
1	TFMLKYDEN	261	269	9	HLA-A*02:01	261	48	0.00185823	48	0.00185823	48	TRUE	TRUE
1	DISNVPFSP	454	462	9	HLA-A*02:01	454	48	0.00187197	48	0.00187197	48	TRUE	TRUE

seq #	peptide	start	end	peptide length	allele	peptide index	median binding	comblib_sidney	comblib_sidney	legacy IC50	legacy rank	scores equal	percentiles equal
1	AQKFNGLTV	834	842	9	HLA-A*02:01	834	49	0.00187932	49	0.00187932	49	TRUE	TRUE
1	GFNFSQILP	781	789	9	HLA-A*02:01	781	49	0.00187932	49	0.00187932	49	TRUE	TRUE
1	RHGKLRPFE	444	452	9	HLA-A*02:01	444	49	0.00189845	49	0.00189845	49	TRUE	TRUE
1	IFDNAFNCT	152	160	9	HLA-A*02:01	152	49	0.00190941	49	0.00190941	49	TRUE	TRUE
1	ECLGDINAR	821	829	9	HLA-A*02:01	821	49	0.00191337	49	0.00191337	49	TRUE	TRUE
1	KFNGLTVLP	836	844	9	HLA-A*02:01	836	49	0.00191867	49	0.00191867	49	TRUE	TRUE
1	NGLTGTGVL	530	538	9	HLA-A*02:01	530	49	0.00192309	49	0.00192309	49	TRUE	TRUE
1	NEVAKNLNE	1169	1177	9	HLA-A*02:01	1169	49	0.00192752	49	0.00192752	49	TRUE	TRUE
1	VGYLKPTTF	254	262	9	HLA-A*02:01	254	49	0.00192752	49	0.00192752	49	TRUE	TRUE
1	QESLTTTST	917	925	9	HLA-A*02:01	917	49	0.00193197	49	0.00193197	49	TRUE	TRUE
1	QERNFTTAP	1053	1061	9	HLA-A*02:01	1053	49	0.00194581	49	0.00194581	49	TRUE	TRUE
1	LNESLIDLQ	1175	1183	9	HLA-A*02:01	1175	49	0.00195209	49	0.00195209	49	TRUE	TRUE
1	RNFFSPQII	1089	1097	9	HLA-A*02:01	1089	50	0.00198518	50	0.00198518	50	TRUE	TRUE
1	SISITTEVM	701	709	9	HLA-A*02:01	701	50	0.00198609	50	0.00198609	50	TRUE	TRUE
1	SKPMGTQTH	141	149	9	HLA-A*02:01	141	50	0.00199618	50	0.00199618	50	TRUE	TRUE
1	GIAAEQDRN	751	759	9	HLA-A*02:01	751	50	0.00200078	50	0.00200078	50	TRUE	TRUE
1	VADYSVLYN	349	357	9	HLA-A*02:01	349	50	0.00201094	50	0.00201094	50	TRUE	TRUE
1	PIGAGICAS	651	659	9	HLA-A*02:01	651	50	0.0020128	50	0.0020128	50	TRUE	TRUE
1	ALQIPFAMQ	875	883	9	HLA-A*02:01	875	50	0.00203095	50	0.00203095	50	TRUE	TRUE
1	SVLYNSTFF	353	361	9	HLA-A*02:01	353	50	0.00203236	50	0.00203236	50	TRUE	TRUE
1	LQELGKYEQ	1182	1190	9	HLA-A*02:01	1182	50	0.00203564	50	0.00203564	50	TRUE	TRUE
1	NASVVNIQK	1155	1163	9	HLA-A*02:01	1155	50	0.00203939	50	0.00203939	50	TRUE	TRUE
1	PIFKLPLGI	218	226	9	HLA-A*02:01	218	50	0.0020408	50	0.0020408	50	TRUE	TRUE
1	LKYFGGFNF	776	784	9	HLA-A*02:01	776	50	0.00204174	50	0.00204174	50	TRUE	TRUE
1	VVVLSFELL	496	504	9	HLA-A*02:01	496	50	0.00204174	50	0.00204174	50	TRUE	TRUE
1	LQYGSFCTQ	736	744	9	HLA-A*02:01	736	51	0.00205921	51	0.00205921	51	TRUE	TRUE
1	LNDYGFYTT	478	486	9	HLA-A*02:01	478	51	0.00206443	51	0.00206443	51	TRUE	TRUE
1	QLTPAWRIY	614	622	9	HLA-A*02:01	614	51	0.00206538	51	0.00206538	51	TRUE	TRUE
1	LYQDVNCTD	597	605	9	HLA-A*02:01	597	51	0.00206681	51	0.00206681	51	TRUE	TRUE
1	WPLNDYGFY	476	484	9	HLA-A*02:01	476	51	0.00207778	51	0.00207778	51	TRUE	TRUE
1	MAYRFNGIG	884	892	9	HLA-A*02:01	884	51	0.00208065	51	0.00208065	51	TRUE	TRUE
1	LQPELDSFK	1123	1131	9	HLA-A*02:01	1123	51	0.00208113	51	0.00208113	51	TRUE	TRUE
1	EVAKNLNES	1170	1178	9	HLA-A*02:01	1170	51	0.00208449	51	0.00208449	51	TRUE	TRUE
1	PVIPFKDGI	79	87	9	HLA-A*02:01	79	51	0.00209315	51	0.00209315	51	TRUE	TRUE
1	NTIAIPTNF	692	700	9	HLA-A*02:01	692	51	0.00209411	51	0.00209411	51	TRUE	TRUE
1	TGNNVFQTQ	624	632	9	HLA-A*02:01	624	51	0.00211203	51	0.00211203	51	TRUE	TRUE
1	PATVCGPKL	507	515	9	HLA-A*02:01	507	51	0.00211349	51	0.00211349	51	TRUE	TRUE
1	SAAAYFVGY	248	256	9	HLA-A*02:01	248	51	0.00211934	51	0.00211934	51	TRUE	TRUE
1	AWNTRNIDA	422	430	9	HLA-A*02:01	422	51	0.00212618	51	0.00212618	51	TRUE	TRUE
1	TTGIGYQPY	486	494	9	HLA-A*02:01	486	51	0.00212814	51	0.00212814	51	TRUE	TRUE
1	GDVVRFPNI	311	319	9	HLA-A*02:01	311	52	0.00213501	52	0.00213501	52	TRUE	TRUE
1	RDLPSGFNT	207	215	9	HLA-A*02:01	207	52	0.00213501	52	0.00213501	52	TRUE	TRUE
1	ELDKYFKNH	1133	1141	9	HLA-A*02:01	1133	52	0.00214882	52	0.00214882	52	TRUE	TRUE
1	SCLKGACSC	1224	1232	9	HLA-A*02:01	1224	52	0.00215625	52	0.00215625	52	TRUE	TRUE
1	GINITNFRA	225	233	9	HLA-A*02:01	225	52	0.00215774	52	0.00215774	52	TRUE	TRUE
1	PGQTGVIAD	399	407	9	HLA-A*02:01	399	52	0.00216222	52	0.00216222	52	TRUE	TRUE
1	FCGKGYHLM	1024	1032	9	HLA-A*02:01	1024	52	0.00216471	52	0.00216471	52	TRUE	TRUE
1	YEQYIKWPW	1188	1196	9	HLA-A*02:01	1188	52	0.0021712	52	0.0021712	52	TRUE	TRUE
1	FNKVTLADA	805	813	9	HLA-A*02:01	805	52	0.00217671	52	0.00217671	52	TRUE	TRUE
1	PDDFMGCVL	413	421	9	HLA-A*02:01	413	52	0.00221411	52	0.00221411	52	TRUE	TRUE
1	GQSKRVDFC	1017	1025	9	HLA-A*02:01	1017	52	0.00221973	52	0.00221973	52	TRUE	TRUE
1	QALNTLVKQ	939	947	9	HLA-A*02:01	939	52	0.0022228	52	0.0022228	52	TRUE	TRUE
1	VVRDLPSGF	205	213	9	HLA-A*02:01	205	52	0.00222331	52	0.00222331	52	TRUE	TRUE
1	YRYLRHGKL	440	448	9	HLA-A*02:01	440	52	0.00223049	52	0.00223049	52	TRUE	TRUE
1	RASANLAAT	1001	1009	9	HLA-A*02:01	1001	52	0.0022346	52	0.0022346	52	TRUE	TRUE
1	FFSTFKCYG	360	368	9	HLA-A*02:01	360	52	0.00223821	52	0.00223821	52	TRUE	TRUE
1	PVSMAKTSV	710	718	9	HLA-A*02:01	710	52	0.00224285	52	0.00224285	52	TRUE	TRUE
1	TITDAVDCS	271	279	9	HLA-A*02:01	271	53	0.00224388	53	0.00224388	53	TRUE	TRUE
1	ELKCSVKSF	285	293	9	HLA-A*02:01	285	53	0.00225944	53	0.00225944	53	TRUE	TRUE
1	LCPFGEVFN	322	330	9	HLA-A*02:01	322	53	0.00226778	53	0.00226778	53	TRUE	TRUE
1	GFYTTTGIG	482	490	9	HLA-A*02:01	482	53	0.00227457	53	0.00227457	53	TRUE	TRUE
1	LKGACSCGS	1226	1234	9	HLA-A*02:01	1226	53	0.00228034	53	0.00228034	53	TRUE	TRUE
1	VVRFPNITN	313	321	9	HLA-A*02:01	313	53	0.00228139	53	0.00228139	53	TRUE	TRUE
1	KSFEIDKGI	291	299	9	HLA-A*02:01	291	53	0.00228981	53	0.00228981	53	TRUE	TRUE
1	TTDNTFVSG	1098	1106	9	HLA-A*02:01	1098	53	0.00229087	53	0.00229087	53	TRUE	TRUE
1	AIHADQLTP	609	617	9	HLA-A*02:01	609	53	0.0022914	53	0.0022914	53	TRUE	TRUE
1	EDDSEPVLK	1240	1248	9	HLA-A*02:01	1240	53	0.00229985	53	0.00229985	53	TRUE	TRUE
1	MNNKSQSVI	107	115	9	HLA-A*02:01	107	53	0.002311	53	0.002311	53	TRUE	TRUE
1	TATAGWTFG	863	871	9	HLA-A*02:01	863	53	0.00232381	53	0.00232381	53	TRUE	TRUE
1	PLKPTKRSF	791	799	9	HLA-A*02:01	791	53	0.00232702	53	0.00232702	53	TRUE	TRUE
1	EIDRLNEVA	1164	1172	9	HLA-A*02:01	1164	53	0.00233346	53	0.00233346	53	TRUE	TRUE
1	TMSLGADSS	678	686	9	HLA-A*02:01	678	53	0.00233346	53	0.00233346	53	TRUE	TRUE
1	ITDAVDCSQ	272	280	9	HLA-A*02:01	272	53	0.00234261	53	0.00234261	53	TRUE	TRUE
1	ATSTGNYNY	430	438	9	HLA-A*02:01	430	53	0.00234423	53	0.00234423	53	TRUE	TRUE
1	DPLQPELDS	1121	1129	9	HLA-A*02:01	1121	54	0.00234855	54	0.00234855	54	TRUE	TRUE
1	ECANLLLQY	730	738	9	HLA-A*02:01	730	54	0.0023518	54	0.0023518	54	TRUE	TRUE
1	DVVNQNAQA	932	940	9	HLA-A*02:01	932	54	0.00236701	54	0.00236701	54	TRUE	TRUE
1	PLLTDDMIA	845	853	9	HLA-A*02:01	845	54	0.00237028	54	0.00237028	54	TRUE	TRUE
1	PELDSFKEE	1125	1133	9	HLA-A*02:01	1125	54	0.00238671	54	0.00238671	54	TRUE	TRUE
1	NQNAQALNT	935	943	9	HLA-A*02:01	935	54	0.00238781	54	0.00238781	54	TRUE	TRUE
1	DNAFNCTFE	154	162	9	HLA-A*02:01	154	54	0.00238781	54	0.00238781	54	TRUE	TRUE
1	NARDLICAQ	827	835	9	HLA-A*02:01	827	54	0.00239828	54	0.00239828	54	TRUE	TRUE
1	TAPAICHEG	1059	1067	9	HLA-A*02:01	1059	54	0.00239994	5.40E+01	2.40E-03	54	TRUE	TRUE
1	HTMIFDNAF	149	157	9	HLA-A*02:01	149	54	0.00241657	5.40E+01	2.42E-03	54	TRUE	TRUE
1	APATVCGPK	506	514	9	HLA-A*02:01	506	54	0.00242605	5.40E+01	2.43E-03	54	TRUE	TRUE
1	PCSFGGVSV	575	583	9	HLA-A*02:01	575	54	0.00242773	5.40E+01	2.43E-03	54	TRUE	TRUE
1	SGTATAGWT	861	869	9	HLA-A*02:01	861	54	0.0024322	5.40E+01	2.43E-03	54	TRUE	TRUE
1	STSQKSIVA	668	676	9	HLA-A*02:01	668	55	0.00246604	5.50E+01	2.47E-03	55	TRUE	TRUE
1	GVFVFNGTS	1075	1083	9	HLA-A*02:01	1075	55	0.00248313	5.50E+01	2.48E-03	55	TRUE	TRUE
1	DVVRDLPSG	204	212	9	HLA-A*02:01	204	55	0.00249	5.50E+01	2.49E-03	55	TRUE	TRUE
1	DPKTSEILD	564	572	9	HLA-A*02:01	564	55	0.00251131	5.50E+01	2.51E-03	55	TRUE	TRUE
1	LADAGFMKQ	810	818	9	HLA-A*02:01	810	55	0.0025229	5.50E+01	2.52E-03	55	TRUE	TRUE
1	ICAQKFNGL	832	840	9	HLA-A*02:01	832	56	0.00256094	5.60E+01	2.56E-03	56	TRUE	TRUE
1	QMYKTPTLK	769	777	9	HLA-A*02:01	769	56	0.00256626	5.60E+01	2.57E-03	56	TRUE	TRUE
1	FVVKGDDVR	387	395	9	HLA-A*02:01	387	56	0.00257276	5.60E+01	2.57E-03	56	TRUE	TRUE
1	DRNTREVFA	757	765	9	HLA-A*02:01	757	56	0.00257395	5.60E+01	2.57E-03	56	TRUE	TRUE
1	QMAYRFNGI	883	891	9	HLA-A*02:01	883	56	0.00257632	5.60E+01	2.58E-03	56	TRUE	TRUE
1	QNVLYENQK	895	903	9	HLA-A*02:01	895	56	0.00258881	5.60E+01	2.59E-03	56	TRUE	TRUE
1	SVDCNMYIC	717	725	9	HLA-A*02:01	717	56	0.00260196	5.60E+01	2.60E-03	56	TRUE	TRUE
1	KCYGVSATK	365	373	9	HLA-A*02:01	365	56	0.00261036	5.60E+01	2.61E-03	56	TRUE	TRUE
1	EDLLFNKVT	801	809	9	HLA-A*02:01	801	56	0.00261457	5.60E+01	2.61E-03	56	TRUE	TRUE
1	HTVSLLRST	661	669	9	HLA-A*02:01	661	56	0.00263148	5.60E+01	2.63E-03	56	TRUE	TRUE

seq #	peptide	start	end	peptide length	allele	peptide index	median binding	comblib_sidne	comblib_sidne	legacy IC50	legacy rank	scores equal	percentiles equal
1	AAPHGVVFL	1037	1045	9	HLA-A*02:01	1037	56	0.00264363	5.60E+01	2.64E-03	56	TRUE	TRUE
1	IAPGQTGVI	397	405	9	HLA-A*02:01	397	56	0.00264363	5.60E+01	2.64E-03	56	TRUE	TRUE
1	NAPATVCGP	505	513	9	HLA-A*02:01	505	56	0.00264423	5.60E+01	2.64E-03	56	TRUE	TRUE
1	ISNVPFSPD	455	463	9	HLA-A*02:01	455	57	0.00267855	5.70E+01	2.68E-03	57	TRUE	TRUE
1	KFDEDDSEP	1237	1245	9	HLA-A*02:01	1237	57	0.00271019	5.70E+01	2.71E-03	57	TRUE	TRUE
1	WTFGAGAAL	868	876	9	HLA-A*02:01	868	57	0.0027227	5.70E+01	2.72E-03	57	TRUE	TRUE
1	SGINASVVN	1152	1160	9	HLA-A*02:01	1152	57	0.00274284	5.70E+01	2.74E-03	57	TRUE	TRUE
1	NQCVNFNFN	522	530	9	HLA-A*02:01	522	57	0.0027555	5.70E+01	2.76E-03	57	TRUE	TRUE
1	DSFKEELDK	1128	1136	9	HLA-A*02:01	1128	57	0.00276249	5.70E+01	2.76E-03	57	TRUE	TRUE
1	DDVQAPNYT	23	31	9	HLA-A*02:01	23	57	0.00278997	5.70E+01	2.79E-03	57	TRUE	TRUE
1	SSIAYSNNT	685	693	9	HLA-A*02:01	685	57	0.00279126	5.70E+01	2.79E-03	57	TRUE	TRUE
1	YSNNTIAIP	689	697	9	HLA-A*02:01	689	57	0.0027919	5.70E+01	2.79E-03	57	TRUE	TRUE
1	MGCVLAWNT	417	425	9	HLA-A*02:01	417	57	0.00279254	5.70E+01	2.79E-03	57	TRUE	TRUE
1	AGICASYHT	654	662	9	HLA-A*02:01	654	57	0.00279254	5.70E+01	2.79E-03	57	TRUE	TRUE
1	GKLQDVVNQ	928	936	9	HLA-A*02:01	928	58	0.00279705	5.80E+01	2.80E-03	58	TRUE	TRUE
1	CKFDEDDSE	1236	1244	9	HLA-A*02:01	1236	58	0.00280543	5.80E+01	2.81E-03	58	TRUE	TRUE
1	ASSEVAVLY	590	598	9	HLA-A*02:01	590	58	0.00281061	5.80E+01	2.81E-03	58	TRUE	TRUE
1	PQIITTDNT	1094	1102	9	HLA-A*02:01	1094	58	0.00283009	5.80E+01	2.83E-03	58	TRUE	TRUE
1	TTFMLKYDE	260	268	9	HLA-A*02:01	260	58	0.00283139	5.80E+01	2.83E-03	58	TRUE	TRUE
1	YKTPTLKYF	771	779	9	HLA-A*02:01	771	58	0.00283792	5.80E+01	2.84E-03	58	TRUE	TRUE
1	IGAEHVDTS	637	645	9	HLA-A*02:01	637	58	0.00283792	5.80E+01	2.84E-03	58	TRUE	TRUE
1	CVADYSVLY	348	356	9	HLA-A*02:01	348	58	0.00287078	5.80E+01	2.87E-03	58	TRUE	TRUE
1	IGAGICASY	652	660	9	HLA-A*02:01	652	58	0.0028774	5.80E+01	2.88E-03	58	TRUE	TRUE
1	YVPSQERNF	1049	1057	9	HLA-A*02:01	1049	58	0.00288403	5.80E+01	2.88E-03	58	TRUE	TRUE
1	IANQFNKAI	905	913	9	HLA-A*02:01	905	59	0.00293224	5.90E+01	2.93E-03	59	TRUE	TRUE
1	RVVVLSFEL	495	503	9	HLA-A*02:01	495	59	0.00293765	5.90E+01	2.94E-03	59	TRUE	TRUE
1	QRNFFSPQI	1088	1096	9	HLA-A*02:01	1088	59	0.00294713	5.90E+01	2.95E-03	59	TRUE	TRUE
1	GTSWFITQR	1081	1089	9	HLA-A*02:01	1081	59	0.00296074	5.90E+01	2.96E-03	59	TRUE	TRUE
1	SANLAATKM	1003	1011	9	HLA-A*02:01	1003	59	0.00297303	5.90E+01	2.97E-03	59	TRUE	TRUE
1	VTILLCCMT	1212	1220	9	HLA-A*02:01	1212	59	0.00298538	5.90E+01	2.99E-03	59	TRUE	TRUE
1	DGIYFAATE	85	93	9	HLA-A*02:01	85	59	0.00298676	5.90E+01	2.99E-03	59	TRUE	TRUE
1	CASYHTVSL	657	665	9	HLA-A*02:01	657	59	0.00299364	5.90E+01	2.99E-03	59	TRUE	TRUE
1	KHLREFVFK	180	188	9	HLA-A*02:01	180	59	0.00299709	5.90E+01	3.00E-03	59	TRUE	TRUE
1	IGIINNTVY	1112	1120	9	HLA-A*02:01	1112	59	0.00301301	5.90E+01	3.01E-03	59	TRUE	TRUE
1	NPLAELKCS	281	289	9	HLA-A*02:01	281	59	0.00302413	5.90E+01	3.02E-03	59	TRUE	TRUE
1	AELKCSVKS	284	292	9	HLA-A*02:01	284	59	0.00303389	5.90E+01	3.03E-03	59	TRUE	TRUE
1	QNPLAELKC	280	288	9	HLA-A*02:01	280	60	0.0030486	6.00E+01	3.05E-03	60	TRUE	TRUE
1	AFNCTFEYI	156	164	9	HLA-A*02:01	156	60	0.00306126	6.00E+01	3.06E-03	60	TRUE	TRUE
1	TTEVMPVSM	705	713	9	HLA-A*02:01	705	60	0.00306196	6.00E+01	3.06E-03	60	TRUE	TRUE
1	VKGDDVRQI	389	397	9	HLA-A*02:01	389	60	0.00306196	6.00E+01	3.06E-03	60	TRUE	TRUE
1	PHGVVFLHV	1039	1047	9	HLA-A*02:01	1039	60	0.00307044	6.00E+01	3.07E-03	60	TRUE	TRUE
1	IDRLNEVAK	1165	1173	9	HLA-A*02:01	1165	60	0.00307397	6.00E+01	3.07E-03	60	TRUE	TRUE
1	TFFSTFKCY	359	367	9	HLA-A*02:01	359	60	0.00308248	6.00E+01	3.08E-03	60	TRUE	TRUE
1	NCDVVIGII	1107	1115	9	HLA-A*02:01	1107	60	0.00308603	6.00E+01	3.09E-03	60	TRUE	TRUE
1	NFGAISSVL	951	959	9	HLA-A*02:01	951	60	0.00309671	6.00E+01	3.10E-03	60	TRUE	TRUE
1	LDSFKEELD	1127	1135	9	HLA-A*02:01	1127	60	0.00310099	6.00E+01	3.10E-03	60	TRUE	TRUE
1	GQTGVIADY	400	408	9	HLA-A*02:01	400	60	0.003155	6.00E+01	3.16E-03	60	TRUE	TRUE
1	SLTTTSTAL	919	927	9	HLA-A*02:01	919	60	0.00316228	6.00E+01	3.16E-03	60	TRUE	TRUE
1	MKQYGECLG	816	824	9	HLA-A*02:01	816	60	0.00316228	6.00E+01	3.16E-03	60	TRUE	TRUE
1	NFNGLTGTG	528	536	9	HLA-A*02:01	528	60	0.00316884	6.00E+01	3.17E-03	60	TRUE	TRUE
1	SSEVAVLYQ	591	599	9	HLA-A*02:01	591	60	0.00317322	6.00E+01	3.17E-03	60	TRUE	TRUE
1	ECVLGQSKR	1013	1021	9	HLA-A*02:01	1013	60	0.00317541	6.00E+01	3.18E-03	60	TRUE	TRUE
1	FQQFGRDVS	548	556	9	HLA-A*02:01	548	60	0.00317687	6.00E+01	3.18E-03	60	TRUE	TRUE
1	TGFHTINHT	67	75	9	HLA-A*02:01	67	60	0.00317687	6.00E+01	3.18E-03	60	TRUE	TRUE
1	AQVKQMYKT	765	773	9	HLA-A*02:01	765	61	0.0031989	6.10E+01	3.20E-03	61	TRUE	TRUE
1	VCGPKLSTD	510	518	9	HLA-A*02:01	510	61	0.00320258	6.10E+01	3.20E-03	61	TRUE	TRUE
1	ISQIQESLT	913	921	9	HLA-A*02:01	913	61	0.00320479	6.10E+01	3.20E-03	61	TRUE	TRUE
1	DATSTGNYN	429	437	9	HLA-A*02:01	429	61	0.0032181	6.10E+01	3.22E-03	61	TRUE	TRUE
1	ICASYHTVS	656	664	9	HLA-A*02:01	656	61	0.00323892	6.10E+01	3.24E-03	61	TRUE	TRUE
1	PFQQFGRDV	547	555	9	HLA-A*02:01	547	61	0.00324116	6.10E+01	3.24E-03	61	TRUE	TRUE
1	KSNVVRGWV	94	102	9	HLA-A*02:01	94	61	0.0032419	6.10E+01	3.24E-03	61	TRUE	TRUE
1	STFKCYGVS	362	370	9	HLA-A*02:01	362	61	0.00326588	6.10E+01	3.27E-03	61	TRUE	TRUE
1	QYGSFCTQL	737	745	9	HLA-A*02:01	737	61	0.00327491	6.10E+01	3.27E-03	61	TRUE	TRUE
1	NDYGFYTTT	479	487	9	HLA-A*02:01	479	61	0.00328398	6.10E+01	3.28E-03	61	TRUE	TRUE
1	ARDLICAQK	828	836	9	HLA-A*02:01	828	61	0.00328625	6.10E+01	3.29E-03	61	TRUE	TRUE
1	GNFKHLREF	177	185	9	HLA-A*02:01	177	61	0.00330217	6.10E+01	3.30E-03	61	TRUE	TRUE
1	VYKGYQPID	196	204	9	HLA-A*02:01	196	61	0.00330598	6.10E+01	3.31E-03	61	TRUE	TRUE
1	KTPTLKYFG	772	780	9	HLA-A*02:01	772	61	0.00331131	6.10E+01	3.31E-03	61	TRUE	TRUE
1	CCMTSCCSC	1217	1225	9	HLA-A*02:01	1217	62	0.0033243	6.20E+01	3.32E-03	62	TRUE	TRUE
1	VLKGVKLHY	1246	1254	9	HLA-A*02:01	1246	62	0.0033266	6.20E+01	3.33E-03	62	TRUE	TRUE
1	NIDATSTGN	427	435	9	HLA-A*02:01	427	62	0.0033358	6.20E+01	3.34E-03	62	TRUE	TRUE
1	LDVSEKSGN	170	178	9	HLA-A*02:01	170	62	0.00333887	6.20E+01	3.34E-03	62	TRUE	TRUE
1	QIANQFNKA	904	912	9	HLA-A*02:01	904	62	0.00334195	6.20E+01	3.34E-03	62	TRUE	TRUE
1	ISPCSFGGV	573	581	9	HLA-A*02:01	573	62	0.00334811	6.20E+01	3.35E-03	62	TRUE	TRUE
1	VSKPMGTQT	140	148	9	HLA-A*02:01	140	62	0.00337132	6.20E+01	3.37E-03	62	TRUE	TRUE
1	VKQLSSNFG	945	953	9	HLA-A*02:01	945	62	0.00337287	6.20E+01	3.37E-03	62	TRUE	TRUE
1	KRSFIEDLL	796	804	9	HLA-A*02:01	796	62	0.00337598	6.20E+01	3.38E-03	62	TRUE	TRUE
1	VYADSFVVK	382	390	9	HLA-A*02:01	382	62	0.00337676	6.20E+01	3.38E-03	62	TRUE	TRUE
1	FNGTSWFIT	1079	1087	9	HLA-A*02:01	1079	62	0.00337909	6.20E+01	3.38E-03	62	TRUE	TRUE
1	TAIHADQLT	608	616	9	HLA-A*02:01	608	62	0.00338221	6.20E+01	3.38E-03	62	TRUE	TRUE
1	TPSSKRFQP	539	547	9	HLA-A*02:01	539	62	0.0033861	6.20E+01	3.39E-03	62	TRUE	TRUE
1	QIDRLITGR	974	982	9	HLA-A*02:01	974	62	0.00340722	6.20E+01	3.41E-03	62	TRUE	TRUE
1	NQKQIANQF	901	909	9	HLA-A*02:01	901	62	0.00341193	6.20E+01	3.41E-03	62	TRUE	TRUE
1	LGDISGINA	1148	1156	9	HLA-A*02:01	1148	62	0.0034435	6.20E+01	3.44E-03	62	TRUE	TRUE
1	LFNKVTLAD	804	812	9	HLA-A*02:01	804	62	0.00345939	6.20E+01	3.46E-03	62	TRUE	TRUE
1	KNQCVNFNF	521	529	9	HLA-A*02:01	521	63	0.00346577	6.30E+01	3.47E-03	63	TRUE	TRUE
1	VRDLPSGFN	206	214	9	HLA-A*02:01	206	63	0.00349623	6.30E+01	3.50E-03	63	TRUE	TRUE
1	TGVLTPSSK	535	543	9	HLA-A*02:01	535	63	0.00350994	6.30E+01	3.51E-03	63	TRUE	TRUE
1	ERKKISNCV	341	349	9	HLA-A*02:01	341	63	0.00352696	6.30E+01	3.53E-03	63	TRUE	TRUE
1	KCSVKSFEI	287	295	9	HLA-A*02:01	287	63	0.00353509	6.30E+01	3.54E-03	63	TRUE	TRUE
1	WRIYSTGNN	619	627	9	HLA-A*02:01	619	63	0.00353671	6.30E+01	3.54E-03	63	TRUE	TRUE
1	GTNASSEVA	587	595	9	HLA-A*02:01	587	63	0.00357273	6.30E+01	3.57E-03	63	TRUE	TRUE
1	LPFYSNVTG	60	68	9	HLA-A*02:01	60	63	0.00358592	6.30E+01	3.59E-03	63	TRUE	TRUE
1	EKSNVVRGW	93	101	9	HLA-A*02:01	93	63	0.00359501	6.30E+01	3.60E-03	63	TRUE	TRUE
1	PVLKGVKLH	1245	1253	9	HLA-A*02:01	1245	63	0.00359749	6.30E+01	3.60E-03	63	TRUE	TRUE
1	NTLVKQLSS	942	950	9	HLA-A*02:01	942	63	0.00360579	6.30E+01	3.61E-03	63	TRUE	TRUE
1	VFKNKDGFL	186	194	9	HLA-A*02:01	186	63	0.00361327	6.30E+01	3.61E-03	63	TRUE	TRUE
1	ISITTEVMP	702	710	9	HLA-A*02:01	702	63	0.00361327	6.30E+01	3.61E-03	63	TRUE	TRUE
1	LYLTQDLFL	52	60	9	HLA-A*02:01	52	63	0.00361576	6.30E+01	3.62E-03	63	TRUE	TRUE
1	IDVVRDLPS	203	211	9	HLA-A*02:01	203	63	0.00363413	6.30E+01	3.63E-03	63	TRUE	TRUE
1	LSTDLIKNQ	515	523	9	HLA-A*02:01	515	64	0.00366016	6.40E+01	3.66E-03	64	TRUE	TRUE
1	VSGTATAGW	860	868	9	HLA-A*02:01	860	64	0.00367705	6.40E+01	3.68E-03	64	TRUE	TRUE

seq #	peptide	start	end	peptide length	allele	peptide index	median binding	comblib_sidney	comblib_sidney	legacy IC50	legacy rank	scores equal	percentiles equal	
1	NFRAILTAF	230	238	9	HLA-A*02:01	230	64	0.00368044	6.40E+01	3.68E-03	64	TRUE	TRUE	
1	HGKLRPFER	445	453	9	HLA-A*02:01	445	64	0.00370339	6.40E+01	3.70E-03	64	TRUE	TRUE	
1	REGVFVFNG	1073	1081	9	HLA-A*02:01	1073	64	0.00371535	6.40E+01	3.72E-03	64	TRUE	TRUE	
1	QILPDPLKP	786	794	9	HLA-A*02:01	786	64	0.00372477	6.40E+01	3.72E-03	64	TRUE	TRUE	
1	PKLSTDLIK	513	521	9	HLA-A*02:01	513	64	0.00372477	6.40E+01	3.72E-03	64	TRUE	TRUE	
1	NCVADYSVL	347	355	9	HLA-A*02:01	347	64	0.00372735	6.40E+01	3.73E-03	64	TRUE	TRUE	
1	CNMYICGDS	720	728	9	HLA-A*02:01	720	64	0.003748	6.40E+01	3.75E-03	64	TRUE	TRUE	
1	ICHEGKAYF	1063	1071	9	HLA-A*02:01	1063	64	0.00377051	6.40E+01	3.77E-03	64	TRUE	TRUE	
1	VTQQLIRAA	990	998	9	HLA-A*02:01	990	64	0.00378443	6.40E+01	3.78E-03	64	TRUE	TRUE	
1	QQFGRDVSD	549	557	9	HLA-A*02:01	549	64	0.0037853	6.40E+01	3.79E-03	64	TRUE	TRUE	
1	DPLKPTKRS	790	798	9	HLA-A*02:01	790	64	0.00380014	6.40E+01	3.80E-03	64	TRUE	TRUE	
1	KRVDFCGKG	1020	1028	9	HLA-A*02:01	1020	64	0.0038054	6.40E+01	3.81E-03	64	TRUE	TRUE	
1	LPPLLTDDM	843	851	9	HLA-A*02:01	843	65	0.00381593	6.50E+01	3.82E-03	65	TRUE	TRUE	
1	CTPPALNCY	467	475	9	HLA-A*02:01	467	65	0.00381944	6.50E+01	3.82E-03	65	TRUE	TRUE	
1	RAAEIRASA	996	1004	9	HLA-A*02:01	996	65	0.00383884	6.50E+01	3.84E-03	65	TRUE	TRUE	
1	IDLQELGKY	1180	1188	9	HLA-A*02:01	1180	65	0.00385834	6.50E+01	3.86E-03	65	TRUE	TRUE	
1	LPSGFNTLK	209	217	9	HLA-A*02:01	209	65	0.00389852	6.50E+01	3.90E-03	65	TRUE	TRUE	
1	VPFSPDGKP	458	466	9	HLA-A*02:01	458	65	0.00390571	6.50E+01	3.91E-03	65	TRUE	TRUE	
1	IVAYTMSLG	674	682	9	HLA-A*02:01	674	65	0.00392645	6.50E+01	3.93E-03	65	TRUE	TRUE	
1	TTTGIGYQP	485	493	9	HLA-A*02:01	485	65	0.00393641	6.50E+01	3.94E-03	65	TRUE	TRUE	
1	TEVMPVSMA	706	714	9	HLA-A*02:01	706	65	0.00394457	6.50E+01	3.94E-03	65	TRUE	TRUE	
1	CTDVSTAIH	603	611	9	HLA-A*02:01	603	65	0.00394639	6.50E+01	3.95E-03	65	TRUE	TRUE	
1	NCTDVSTAI	602	610	9	HLA-A*02:01	602	66	0.00399393	6.60E+01	3.99E-03	66	TRUE	TRUE	
1	GFMKQYGEC	814	822	9	HLA-A*02:01	814	66	0.00400129	6.60E+01	4.00E-03	66	TRUE	TRUE	
1	TGVIADYNY	402	410	9	HLA-A*02:01	402	66	0.00402717	6.60E+01	4.03E-03	66	TRUE	TRUE	
1	GVYYPDEIF	39	47	9	HLA-A*02:01	39	66	0.00404576	6.60E+01	4.05E-03	66	TRUE	TRUE	
1	ITPGTNASS	584	592	9	HLA-A*02:01	584	66	0.00405509	6.60E+01	4.06E-03	66	TRUE	TRUE	
1	DFCGKGYHL	1023	1031	9	HLA-A*02:01	1023	66	0.00405602	6.60E+01	4.06E-03	66	TRUE	TRUE	
1	YTTTGIGYQ	484	492	9	HLA-A*02:01	484	66	0.00407099	6.60E+01	4.07E-03	66	TRUE	TRUE	
1	TDNTFVSGN	1099	1107	9	HLA-A*02:01	1099	66	0.00408884	6.60E+01	4.09E-03	66	TRUE	TRUE	
1	PPLLTDDMI	844	852	9	HLA-A*02:01	844	66	0.00410582	6.60E+01	4.11E-03	66	TRUE	TRUE	
1	VFAQVKQMY	763	771	9	HLA-A*02:01	763	66	0.00411055	6.60E+01	4.11E-03	66	TRUE	TRUE	
1	TREVFAQVK	760	768	9	HLA-A*02:01	760	66	0.00412762	6.60E+01	4.13E-03	66	TRUE	TRUE	
1	LKPIFKLPL	216	224	9	HLA-A*02:01	216	67	0.00416869	6.70E+01	4.17E-03	67	TRUE	TRUE	
1	DDFMGCVLA	414	422	9	HLA-A*02:01	414	67	0.00417446	6.70E+01	4.17E-03	67	TRUE	TRUE	
1	SQIQESLTT	914	922	9	HLA-A*02:01	914	67	0.00421697	6.70E+01	4.22E-03	67	TRUE	TRUE	
1	TPGTNASSE	585	593	9	HLA-A*02:01	585	67	0.00423253	6.70E+01	4.23E-03	67	TRUE	TRUE	
1	KVTLADAGF	807	815	9	HLA-A*02:01	807	67	0.0042462	6.70E+01	4.25E-03	67	TRUE	TRUE	
1	STECANLLL	728	736	9	HLA-A*02:01	728	67	0.00431519	6.70E+01	4.32E-03	67	TRUE	TRUE	
1	NIQKEIDRL	1160	1168	9	HLA-A*02:01	1160	67	0.00432514	6.70E+01	4.33E-03	67	TRUE	TRUE	
1	LIAIVMVTI	1206	1214	9	HLA-A*02:01	1206	68	0.00435512	6.80E+01	4.36E-03	68	TRUE	TRUE	
1	ITQRNFFSP	1086	1094	9	HLA-A*02:01	1086	68	0.00435612	6.80E+01	4.36E-03	68	TRUE	TRUE	
1	HTSPDVDLG	1141	1149	9	HLA-A*02:01	1141	68	0.00435712	6.80E+01	4.36E-03	68	TRUE	TRUE	
1	PSGFNTLKP	210	218	9	HLA-A*02:01	210	68	0.00443302	6.80E+01	4.43E-03	68	TRUE	TRUE	
1	YNYKLPDDF	408	416	9	HLA-A*02:01	408	68	0.00446478	6.80E+01	4.46E-03	68	TRUE	TRUE	
1	IRACNFELC	125	133	9	HLA-A*02:01	125	68	0.00450505	6.80E+01	4.51E-03	68	TRUE	TRUE	
1	SYECDIPIG	645	653	9	HLA-A*02:01	645	68	0.00452064	6.80E+01	4.52E-03	68	TRUE	TRUE	
1	RNTREVFAQ	758	766	9	HLA-A*02:01	758	69	0.00454465	6.90E+01	4.54E-03	69	TRUE	TRUE	
1	CNFELCDNP	128	136	9	HLA-A*02:01	128	69	0.00455932	6.90E+01	4.56E-03	69	TRUE	TRUE	
1	DYNYKLPDD	407	415	9	HLA-A*02:01	407	69	0.0045867	6.90E+01	4.59E-03	69	TRUE	TRUE	
1	YPDEIFRSD	42	50	9	HLA-A*02:01	42	69	0.00458881	6.90E+01	4.59E-03	69	TRUE	TRUE	
1	LDRCTTFDD	16	24	9	HLA-A*02:01	16	69	0.00459727	6.90E+01	4.60E-03	69	TRUE	TRUE	
1	TSGSDLDRC	11	19	9	HLA-A*02:01	11	69	0.00461424	6.90E+01	4.61E-03	69	TRUE	TRUE	
1	VQAPNYTQH	25	33	9	HLA-A*02:01	25	69	0.0046366	6.90E+01	4.64E-03	69	TRUE	TRUE	
1	VSLLRSTSQ	663	671	9	HLA-A*02:01	663	69	0.00463981	6.90E+01	4.64E-03	69	TRUE	TRUE	
1	YIKWPWYVW	1191	1199	9	HLA-A*02:01	1191	69	0.0046957	6.90E+01	4.70E-03	69	TRUE	TRUE	
1	SPQIITTDN	1093	1101	9	HLA-A*02:01	1093	69	0.00471846	6.90E+01	4.72E-03	69	TRUE	TRUE	
1	VLGQSKRVD	1015	1023	9	HLA-A*02:01	1015	70	0.00475445	7.00E+01	4.75E-03	70	TRUE	TRUE	
1	KNLNESLID	1173	1181	9	HLA-A*02:01	1173	70	0.00477419	7.00E+01	4.77E-03	70	TRUE	TRUE	
1	PTKRSFIED	794	802	9	HLA-A*02:01	794	70	0.0047852	7.00E+01	4.79E-03	70	TRUE	TRUE	
1	YLRHGKLRP	442	450	9	HLA-A*02:01	442	70	0.0047874	7.00E+01	4.79E-03	70	TRUE	TRUE	
1	MYICGDSTE	722	730	9	HLA-A*02:01	722	70	0.00483281	7.00E+01	4.83E-03	70	TRUE	TRUE	
1	RVDFCGKGY	1021	1029	9	HLA-A*02:01	1021	70	0.00484172	7.00E+01	4.84E-03	70	TRUE	TRUE	
1	TSEILDISP	567	575	9	HLA-A*02:01	567	70	0.00486295	7.00E+01	4.86E-03	70	TRUE	TRUE	
1	VPSQERNFT	1050	1058	9	HLA-A*02:01	1050	70	0.00489328	7.00E+01	4.89E-03	70	TRUE	TRUE	
1	ILSRLDKVE	962	970	9	HLA-A*02:01	962	70	0.00490908	7.00E+01	4.91E-03	70	TRUE	TRUE	
1	LICAQKFNG	831	839	9	HLA-A*02:01	831	70	0.00493174	7.00E+01	4.93E-03	70	TRUE	TRUE	
1	QSVIIINNS	112	120	9	HLA-A*02:01	112	70	0.00494083	7.00E+01	4.94E-03	70	TRUE	TRUE	
1	LSGIAAEQD	749	757	9	HLA-A*02:01	749	71	0.00496478	7.10E+01	4.96E-03	71	TRUE	TRUE	
1	QIPFAMQMA	877	885	9	HLA-A*02:01	877	71	0.00498884	7.10E+01	4.99E-03	71	TRUE	TRUE	
1	LTTTSTALG	920	928	9	HLA-A*02:01	920	71	0.00501187	7.10E+01	5.01E-03	71	TRUE	TRUE	
1	KFPSVYAWE	333	341	9	HLA-A*02:01	333	71	0.00503385	7.10E+01	5.03E-03	71	TRUE	TRUE	
1	RGWVFGSTM	99	107	9	HLA-A*02:01	99	71	0.00503501	7.10E+01	5.04E-03	71	TRUE	TRUE	
1	STAIHADQL	607	615	9	HLA-A*02:01	607	71	0.00503501	7.10E+01	5.04E-03	71	TRUE	TRUE	
1	QTQAGCLIG	630	638	9	HLA-A*02:01	630	71	0.00504661	7.10E+01	5.05E-03	71	TRUE	TRUE	
1	TPTLKYFGG	773	781	9	HLA-A*02:01	773	71	0.00506524	7.10E+01	5.07E-03	71	TRUE	TRUE	
1	LGDINARDL	823	831	9	HLA-A*02:01	823	71	0.00506991	7.10E+01	5.07E-03	71	TRUE	TRUE	
1	IDATSTGNY	428	436	9	HLA-A*02:01	428	71	0.00508628	7.10E+01	5.09E-03	71	TRUE	TRUE	
1	GEVFNATKF	326	334	9	HLA-A*02:01	326	71	0.00509331	7.10E+01	5.09E-03	71	TRUE	TRUE	
1	NCTFEYISD	158	166	9	HLA-A*02:01	158	71	0.00511093	7.10E+01	5.11E-03	71	TRUE	TRUE	
1	PCTPPALNC	466	474	9	HLA-A*02:01	466	71	0.00513452	7.10E+01	5.13E-03	71	TRUE	TRUE	
1	VPSGDVVRF	308	316	9	HLA-A*02:01	308	71	0.0051357	7.10E+01	5.14E-03	71	TRUE	TRUE	
1	DRCTTFDDV	17	25	9	HLA-A*02:01	17	71	0.0051357	7.10E+01	5.14E-03	71	TRUE	TRUE	
1	DVSTAIHAD	605	613	9	HLA-A*02:01	605	71	0.00514399	7.10E+01	5.14E-03	71	TRUE	TRUE	
1	DENGTITDA	267	275	9	HLA-A*02:01	267	71	0.00515466	7.10E+01	5.15E-03	71	TRUE	TRUE	
1	GKPCTPPAL	464	472	9	HLA-A*02:01	464	72	0.00516416	7.20E+01	5.16E-03	72	TRUE	TRUE	
1	VFGSTMNNK	102	110	9	HLA-A*02:01	102	72	0.00517845	7.20E+01	5.18E-03	72	TRUE	TRUE	
1	KYFKNHTSP	1136	1144	9	HLA-A*02:01	1136	72	0.00523962	7.20E+01	5.24E-03	72	TRUE	TRUE	
1	LVSGTATAG	859	867	9	HLA-A*02:01	859	72	0.00524807	7.20E+01	5.25E-03	72	TRUE	TRUE	
1	LNTLVKQLS	941	949	9	HLA-A*02:01	941	72	0.0052942	7.20E+01	5.29E-03	72	TRUE	TRUE	
1	DIWGTSAAA	243	251	9	HLA-A*02:01	243	72	0.00531129	7.20E+01	5.31E-03	72	TRUE	TRUE	
1	SFVVKGDDV	386	394	9	HLA-A*02:01	386	72	0.00531986	7.20E+01	5.32E-03	72	TRUE	TRUE	
1	PFSPDGKPC	459	467	9	HLA-A*02:01	459	72	0.00533335	7.20E+01	5.33E-03	72	TRUE	TRUE	
1	YKYRYLRHG	438	446	9	HLA-A*02:01	438	72	0.00534564	7.20E+01	5.35E-03	72	TRUE	TRUE	
1	AGCLIGAEH	633	641	9	HLA-A*02:01	633	72	0.00536043	7.20E+01	5.36E-03	72	TRUE	TRUE	
1	LTPSSKRFQ	538	546	9	HLA-A*02:01	538	72	0.00536661	7.20E+01	5.37E-03	72	TRUE	TRUE	
1	GFHTINHTF	68	76	9	HLA-A*02:01	68	72	0.0054063	7.20E+01	5.41E-03	72	TRUE	TRUE	
1	PMGTQTHTM	143	151	9	HLA-A*02:01	143	73	0.00541751	7.30E+01	5.42E-03	73	TRUE	TRUE	
1	NNTVYDPLQ	1116	1124	9	HLA-A*02:01	1116	73	0.00542625	7.30E+01	5.43E-03	73	TRUE	TRUE	
1	WFITQRNFF	1084	1092	9	HLA-A*02:01	1084	73	0.00543125	7.30E+01	5.43E-03	73	TRUE	TRUE	
1	PAICHEGKA	1061	1069	9	HLA-A*02:01	1061	73	0.0054325	7.30E+01	5.43E-03	73	TRUE	TRUE	
1	PREGVFVFN	1072	1080	9	HLA-A*02:01	1072	73	0.00543751	7.30E+01	5.44E-03	73	TRUE	TRUE	

seq #	peptide	start	end	peptide length	allele	peptide index	median binding	comblib_sidney	comblib_sidney	legacy IC50	legacy rank	scores equal	percentiles equal	
1	LKPTKRSFI	792	800	9	HLA-A*02:01	792	73	0.00547016	7.30E+01	5.47E-03	73	TRUE	TRUE	
1	AFSPAQDIW	237	245	9	HLA-A*02:01	237	73	0.00547772	7.30E+01	5.48E-03	73	TRUE	TRUE	
1	GTATAGWTF	862	870	9	HLA-A*02:01	862	73	0.00548277	7.30E+01	5.48E-03	73	TRUE	TRUE	
1	CVNFNFNGL	524	532	9	HLA-A*02:01	524	73	0.00549541	7.30E+01	5.50E-03	73	TRUE	TRUE	
1	WYVWLGFIA	1196	1204	9	HLA-A*02:01	1196	73	0.00553605	7.30E+01	5.54E-03	73	TRUE	TRUE	
1	YQPYRVVVL	491	499	9	HLA-A*02:01	491	73	0.00554626	7.30E+01	5.55E-03	73	TRUE	TRUE	
1	PDGKPCTPP	462	470	9	HLA-A*02:01	462	73	0.00555009	7.30E+01	5.55E-03	73	TRUE	TRUE	
1	TSTGNYNYK	431	439	9	HLA-A*02:01	431	73	0.00556032	7.30E+01	5.56E-03	73	TRUE	TRUE	
1	TSPDVDLGD	1142	1150	9	HLA-A*02:01	1142	73	0.00559629	7.30E+01	5.60E-03	73	TRUE	TRUE	
1	LAWNTRNID	421	429	9	HLA-A*02:01	421	73	0.00561436	7.30E+01	5.61E-03	73	TRUE	TRUE	
1	AKTSVDCNM	714	722	9	HLA-A*02:01	714	74	0.00566239	7.40E+01	5.66E-03	74	TRUE	TRUE	
1	AEVQIDRLI	971	979	9	HLA-A*02:01	971	74	0.00572796	7.40E+01	5.73E-03	74	TRUE	TRUE	
1	CDNPFFAVS	133	141	9	HLA-A*02:01	133	74	0.00573324	7.40E+01	5.73E-03	74	TRUE	TRUE	
1	LAATKMSEC	1006	1014	9	HLA-A*02:01	1006	74	0.00574778	7.40E+01	5.75E-03	74	TRUE	TRUE	
1	AQDIWGTSA	241	249	9	HLA-A*02:01	241	74	0.0057544	7.40E+01	5.75E-03	74	TRUE	TRUE	
1	HTFGNPVIP	74	82	9	HLA-A*02:01	74	74	0.00578496	7.40E+01	5.78E-03	74	TRUE	TRUE	
1	RSFIEDLLF	797	805	9	HLA-A*02:01	797	74	0.00579162	7.40E+01	5.79E-03	74	TRUE	TRUE	
1	TSQKSIVAY	669	677	9	HLA-A*02:01	669	74	0.00581835	7.40E+01	5.82E-03	74	TRUE	TRUE	
1	GCVLAWNTR	418	426	9	HLA-A*02:01	418	74	0.00585868	7.40E+01	5.86E-03	74	TRUE	TRUE	
1	DYGFYTTTG	480	488	9	HLA-A*02:01	480	75	0.00586678	7.50E+01	5.87E-03	75	TRUE	TRUE	
1	SPCSFGGVS	574	582	9	HLA-A*02:01	574	75	0.00586949	7.50E+01	5.87E-03	75	TRUE	TRUE	
1	GSFCTQLNR	739	747	9	HLA-A*02:01	739	75	0.00589115	7.50E+01	5.89E-03	75	TRUE	TRUE	
1	MTSCCSCLK	1219	1227	9	HLA-A*02:01	1219	75	0.00589251	7.50E+01	5.89E-03	75	TRUE	TRUE	
1	VNCTDVSTA	601	609	9	HLA-A*02:01	601	75	0.00591289	7.50E+01	5.91E-03	75	TRUE	TRUE	
1	DDSEPVLKG	1241	1249	9	HLA-A*02:01	1241	75	0.00592379	7.50E+01	5.92E-03	75	TRUE	TRUE	
1	ISNCVADYS	345	353	9	HLA-A*02:01	345	75	0.00592652	7.50E+01	5.93E-03	75	TRUE	TRUE	
1	GKLRPFERD	446	454	9	HLA-A*02:01	446	75	0.00597173	7.50E+01	5.97E-03	75	TRUE	TRUE	
1	QELGKYEQY	1183	1191	9	HLA-A*02:01	1183	75	0.00601174	7.50E+01	6.01E-03	75	TRUE	TRUE	
1	STDLIKNQC	516	524	9	HLA-A*02:01	516	75	0.00601589	7.50E+01	6.02E-03	75	TRUE	TRUE	
1	TVSLLRSTS	662	670	9	HLA-A*02:01	662	75	0.0060256	7.50E+01	6.03E-03	75	TRUE	TRUE	
1	MGTQTHTMI	144	152	9	HLA-A*02:01	144	75	0.00603949	7.50E+01	6.04E-03	75	TRUE	TRUE	
1	VVPSGDVVR	307	315	9	HLA-A*02:01	307	75	0.00605899	7.50E+01	6.06E-03	75	TRUE	TRUE	
1	INNTVYDPL	1115	1123	9	HLA-A*02:01	1115	75	0.00607855	7.50E+01	6.08E-03	75	TRUE	TRUE	
1	PTLKYFGGF	774	782	9	HLA-A*02:01	774	75	0.00609256	7.50E+01	6.09E-03	75	TRUE	TRUE	
1	RDVSDFTDS	553	561	9	HLA-A*02:01	553	75	0.00611505	7.50E+01	6.12E-03	75	TRUE	TRUE	
1	GTQTHTMIF	145	153	9	HLA-A*02:01	145	75	0.0061235	7.50E+01	6.12E-03	75	TRUE	TRUE	
1	WERKKISNC	340	348	9	HLA-A*02:01	340	76	0.00615602	7.60E+01	6.16E-03	76	TRUE	TRUE	
1	CAQKFNGLT	833	841	9	HLA-A*02:01	833	76	0.00618301	7.60E+01	6.18E-03	76	TRUE	TRUE	
1	KYEQYIKWP	1187	1195	9	HLA-A*02:01	1187	76	0.00618443	7.60E+01	6.18E-03	76	TRUE	TRUE	
1	ITGRLQSLQ	979	987	9	HLA-A*02:01	979	76	0.00619013	7.60E+01	6.19E-03	76	TRUE	TRUE	
1	INITNFRAI	226	234	9	HLA-A*02:01	226	76	0.00619156	7.60E+01	6.19E-03	76	TRUE	TRUE	
1	PTNFSISIT	697	705	9	HLA-A*02:01	697	76	0.00619156	7.60E+01	6.19E-03	76	TRUE	TRUE	
1	QTGVIADYN	401	409	9	HLA-A*02:01	401	76	0.00619726	7.60E+01	6.20E-03	76	TRUE	TRUE	
1	DFTDSVRDP	557	565	9	HLA-A*02:01	557	76	0.00621155	7.60E+01	6.21E-03	76	TRUE	TRUE	
1	TAALVSGTA	856	864	9	HLA-A*02:01	856	76	0.00622587	7.60E+01	6.23E-03	76	TRUE	TRUE	
1	ECDIPIGAG	647	655	9	HLA-A*02:01	647	76	0.0062431	7.60E+01	6.24E-03	76	TRUE	TRUE	
1	QPFQQFGRD	546	554	9	HLA-A*02:01	546	76	0.00637823	7.60E+01	6.38E-03	76	TRUE	TRUE	
1	WPWYVWLGF	1194	1202	9	HLA-A*02:01	1194	76	0.00639146	7.60E+01	6.39E-03	76	TRUE	TRUE	
1	GRDVSDFTD	552	560	9	HLA-A*02:01	552	76	0.00640472	7.60E+01	6.40E-03	76	TRUE	TRUE	
1	SPDVDLGDI	1143	1151	9	HLA-A*02:01	1143	76	0.00640619	7.60E+01	6.41E-03	76	TRUE	TRUE	
1	AAEIRASAN	997	1005	9	HLA-A*02:01	997	76	0.0064328	7.60E+01	6.43E-03	76	TRUE	TRUE	
1	QIQESLTTT	915	923	9	HLA-A*02:01	915	76	0.00644169	7.60E+01	6.44E-03	76	TRUE	TRUE	
1	NVVIRACNF	122	130	9	HLA-A*02:01	122	77	0.00647143	7.70E+01	6.47E-03	77	TRUE	TRUE	
1	ADQLTPAWR	612	620	9	HLA-A*02:01	612	77	0.00655843	7.70E+01	6.56E-03	77	TRUE	TRUE	
1	NVVRGWVFG	96	104	9	HLA-A*02:01	96	77	0.00656145	7.70E+01	6.56E-03	77	TRUE	TRUE	
1	RGVYYPDEI	38	46	9	HLA-A*02:01	38	77	0.00656145	7.70E+01	6.56E-03	77	TRUE	TRUE	
1	IYSTGNNVF	621	629	9	HLA-A*02:01	621	77	0.00657961	7.70E+01	6.58E-03	77	TRUE	TRUE	
1	MRGVYYPDE	37	45	9	HLA-A*02:01	37	77	0.00664355	7.70E+01	6.64E-03	77	TRUE	TRUE	
1	QVKQMYKTP	766	774	9	HLA-A*02:01	766	77	0.00665426	7.70E+01	6.65E-03	77	TRUE	TRUE	
1	VAVLYQDVN	594	602	9	HLA-A*02:01	594	77	0.00665886	7.70E+01	6.66E-03	77	TRUE	TRUE	
1	AMQMAYRFN	881	889	9	HLA-A*02:01	881	77	0.00668652	7.70E+01	6.69E-03	77	TRUE	TRUE	
1	QAAPHGVVF	1036	1044	9	HLA-A*02:01	1036	77	0.00668652	7.70E+01	6.69E-03	77	TRUE	TRUE	
1	LGKYEQYIK	1185	1193	9	HLA-A*02:01	1185	78	0.00671893	7.80E+01	6.72E-03	78	TRUE	TRUE	
1	AEIRASANL	998	1006	9	HLA-A*02:01	998	78	0.00676083	7.80E+01	6.76E-03	78	TRUE	TRUE	
1	LTVLPPLLT	840	848	9	HLA-A*02:01	840	78	0.00680769	7.80E+01	6.81E-03	78	TRUE	TRUE	
1	DCNMYICGD	719	727	9	HLA-A*02:01	719	78	0.00688177	7.80E+01	6.88E-03	78	TRUE	TRUE	
1	YDPLQPELD	1120	1128	9	HLA-A*02:01	1120	78	0.00691035	7.80E+01	6.91E-03	78	TRUE	TRUE	
1	AGWTFGAGA	866	874	9	HLA-A*02:01	866	78	0.00693426	7.80E+01	6.93E-03	78	TRUE	TRUE	
1	SWFITQRNF	1083	1091	9	HLA-A*02:01	1083	78	0.00694384	7.80E+01	6.94E-03	78	TRUE	TRUE	
1	CGSCCKFDE	1232	1240	9	HLA-A*02:01	1232	78	0.00696627	7.80E+01	6.97E-03	78	TRUE	TRUE	
1	REVFAQVKQ	761	769	9	HLA-A*02:01	761	78	0.0069775	7.80E+01	6.98E-03	78	TRUE	TRUE	
1	ATKMSECVL	1008	1016	9	HLA-A*02:01	1008	78	0.00701455	7.80E+01	7.01E-03	78	TRUE	TRUE	
1	TQQLIRAAE	991	999	9	HLA-A*02:01	991	78	0.00704693	7.80E+01	7.05E-03	78	TRUE	TRUE	
1	VSATKLNDL	369	377	9	HLA-A*02:01	369	78	0.00705992	7.80E+01	7.06E-03	78	TRUE	TRUE	
1	TDSVRDPKT	559	567	9	HLA-A*02:01	559	78	0.00706968	7.80E+01	7.07E-03	78	TRUE	TRUE	
1	LKGVKLHYT	1247	1255	9	HLA-A*02:01	1247	78	0.00709578	7.80E+01	7.10E-03	78	TRUE	TRUE	
1	NHTSPDVDL	1140	1148	9	HLA-A*02:01	1140	78	0.00710232	7.80E+01	7.10E-03	78	TRUE	TRUE	
1	VKQMYKTPT	767	775	9	HLA-A*02:01	767	79	0.00712853	7.90E+01	7.13E-03	79	TRUE	TRUE	
1	KSGNFKHLR	175	183	9	HLA-A*02:01	175	79	0.00713181	7.90E+01	7.13E-03	79	TRUE	TRUE	
1	SPDGKPCTP	461	469	9	HLA-A*02:01	461	79	0.00714003	7.90E+01	7.14E-03	79	TRUE	TRUE	
1	MVTILLCCM	1211	1219	9	HLA-A*02:01	1211	79	0.00719449	7.90E+01	7.19E-03	79	TRUE	TRUE	
1	LAELKCSVK	283	291	9	HLA-A*02:01	283	79	0.00733669	7.90E+01	7.34E-03	79	TRUE	TRUE	
1	SDTLYLTQD	49	57	9	HLA-A*02:01	49	79	0.00733669	7.90E+01	7.34E-03	79	TRUE	TRUE	
1	GWTFGAGAA	867	875	9	HLA-A*02:01	867	79	0.00738924	7.90E+01	7.39E-03	79	TRUE	TRUE	
1	NFSISITTE	699	707	9	HLA-A*02:01	699	79	0.00742848	7.90E+01	7.43E-03	79	TRUE	TRUE	
1	STALGKLQD	924	932	9	HLA-A*02:01	924	79	0.0074319	7.90E+01	7.43E-03	79	TRUE	TRUE	
1	PFAMQMAYR	879	887	9	HLA-A*02:01	879	79	0.0074319	7.90E+01	7.43E-03	79	TRUE	TRUE	
1	NITNFRAIL	227	235	9	HLA-A*02:01	227	79	0.0074817	7.90E+01	7.48E-03	79	TRUE	TRUE	
1	MYKTPTLKY	770	778	9	HLA-A*02:01	770	80	0.00748514	8.00E+01	7.49E-03	80	TRUE	TRUE	
1	DNPFFAVSK	134	142	9	HLA-A*02:01	134	80	0.00755614	8.00E+01	7.56E-03	80	TRUE	TRUE	
1	GTITDAVDC	270	278	9	HLA-A*02:01	270	80	0.00755614	8.00E+01	7.56E-03	80	TRUE	TRUE	
1	SECVLGQSK	1012	1020	9	HLA-A*02:01	1012	80	0.00755614	8.00E+01	7.56E-03	80	TRUE	TRUE	
1	GYQPIDVVR	199	207	9	HLA-A*02:01	199	80	0.00761377	8.00E+01	7.61E-03	80	TRUE	TRUE	
1	SNVTGFHTI	64	72	9	HLA-A*02:01	64	80	0.00765244	8.00E+01	7.65E-03	80	TRUE	TRUE	
1	PYRVVVLSF	493	501	9	HLA-A*02:01	493	80	0.0076913	8.00E+01	7.69E-03	80	TRUE	TRUE	
1	VSTAIHADQ	606	614	9	HLA-A*02:01	606	80	0.00770016	8.00E+01	7.70E-03	80	TRUE	TRUE	
1	NSTFFSTFK	357	365	9	HLA-A*02:01	357	80	0.0078001	8.00E+01	7.80E-03	80	TRUE	TRUE	
1	SATKLNDLC	370	378	9	HLA-A*02:01	370	80	0.00782528	8.00E+01	7.83E-03	80	TRUE	TRUE	
1	IDKGIYQTS	295	303	9	HLA-A*02:01	295	80	0.00784152	8.00E+01	7.84E-03	80	TRUE	TRUE	
1	CDVVIGIIN	1108	1116	9	HLA-A*02:01	1108	81	0.0079726	8.10E+01	7.97E-03	81	TRUE	TRUE	
1	LGQSKRVDF	1016	1024	9	HLA-A*02:01	1016	81	0.00799834	8.10E+01	8.00E-03	81	TRUE	TRUE	
1	GIGVTQNVL	890	898	9	HLA-A*02:01	890	81	0.00805378	8.10E+01	8.05E-03	81	TRUE	TRUE	

seq #	peptide	start	end	peptide length	allele	peptide index	median binding	comblib_sidney	comblib_sidney	legacy IC50	legacy rank	scores equal	percentiles equal
1	QDVVNQNAQ	931	939	9	HLA-A*02:01	931	81	0.00807421	8.10E+01	8.07E-03	81	TRUE	TRUE
1	NDILSRLDK	960	968	9	HLA-A*02:01	960	81	0.00812269	8.10E+01	8.12E-03	81	TRUE	TRUE
1	CTQLNRALS	742	750	9	HLA-A*02:01	742	81	0.00816582	8.10E+01	8.17E-03	81	TRUE	TRUE
1	VMVTILLCC	1210	1218	9	HLA-A*02:01	1210	81	0.0081903	8.10E+01	8.19E-03	81	TRUE	TRUE
1	LGFIAGLIA	1200	1208	9	HLA-A*02:01	1200	81	0.00822243	8.10E+01	8.22E-03	81	TRUE	TRUE
1	LYVYKGYQP	194	202	9	HLA-A*02:01	194	81	0.00822811	8.10E+01	8.23E-03	81	TRUE	TRUE
1	PAQDIWGTS	240	248	9	HLA-A*02:01	240	81	0.00826038	8.10E+01	8.26E-03	81	TRUE	TRUE
1	QSLQTYVTQ	984	992	9	HLA-A*02:01	984	81	0.00830807	8.10E+01	8.31E-03	81	TRUE	TRUE
1	GVSATKLND	368	376	9	HLA-A*02:01	368	82	0.00835795	8.20E+01	8.36E-03	82	TRUE	TRUE
1	NYKLPDDFM	409	417	9	HLA-A*02:01	409	82	0.00839847	8.20E+01	8.40E-03	82	TRUE	TRUE
1	PAWRIYSTG	617	625	9	HLA-A*02:01	617	82	0.00845279	8.20E+01	8.45E-03	82	TRUE	TRUE
1	VSDFTDSVR	555	563	9	HLA-A*02:01	555	82	0.00845668	8.20E+01	8.46E-03	82	TRUE	TRUE
1	NNKSQSVII	108	116	9	HLA-A*02:01	108	82	0.0084879	8.20E+01	8.49E-03	82	TRUE	TRUE
1	TDAVDCSQN	273	281	9	HLA-A*02:01	273	82	0.0085428	8.20E+01	8.54E-03	82	TRUE	TRUE
1	KTSEILDIS	566	574	9	HLA-A*02:01	566	82	0.00855067	8.20E+01	8.55E-03	82	TRUE	TRUE
1	DCSQNPLAE	277	285	9	HLA-A*02:01	277	82	0.00856249	8.20E+01	8.56E-03	82	TRUE	TRUE
1	NTRNIDATS	424	432	9	HLA-A*02:01	424	82	0.00866962	8.20E+01	8.67E-03	82	TRUE	TRUE
1	YQPIDVVRD	200	208	9	HLA-A*02:01	200	82	0.00873172	8.20E+01	8.73E-03	82	TRUE	TRUE
1	QDRNTREVF	756	764	9	HLA-A*02:01	756	83	0.00883894	8.30E+01	8.84E-03	83	TRUE	TRUE
1	VDFCGKGYH	1022	1030	9	HLA-A*02:01	1022	83	0.00884301	8.30E+01	8.84E-03	83	TRUE	TRUE
1	GKGYHLMSF	1026	1034	9	HLA-A*02:01	1026	83	0.00885116	8.30E+01	8.85E-03	83	TRUE	TRUE
1	LTLTSGSDL	8	16	9	HLA-A*02:01	8	83	0.00887156	8.30E+01	8.87E-03	83	TRUE	TRUE
1	GKAYFPREG	1067	1075	9	HLA-A*02:01	1067	83	0.00889201	8.30E+01	8.89E-03	83	TRUE	TRUE
1	LMSFPQAAP	1031	1039	9	HLA-A*02:01	1031	83	0.00897635	8.30E+01	8.98E-03	83	TRUE	TRUE
1	KYDENGTIT	265	273	9	HLA-A*02:01	265	83	0.00901986	8.30E+01	9.02E-03	83	TRUE	TRUE
1	ENQKQIANQ	900	908	9	HLA-A*02:01	900	83	0.0090469	8.30E+01	9.05E-03	83	TRUE	TRUE
1	YNSTFFSTF	356	364	9	HLA-A*02:01	356	83	0.00911591	8.30E+01	9.12E-03	83	TRUE	TRUE
1	ADSFVVKGD	384	392	9	HLA-A*02:01	384	83	0.0092151	8.30E+01	9.22E-03	83	TRUE	TRUE
1	LDKVEAEVQ	966	974	9	HLA-A*02:01	966	83	0.00924911	8.30E+01	9.25E-03	83	TRUE	TRUE
1	IGVTQNVLY	891	899	9	HLA-A*02:01	891	84	0.00931108	8.40E+01	9.31E-03	84	TRUE	TRUE
1	VYAWERKKI	337	345	9	HLA-A*02:01	337	84	0.00931537	8.40E+01	9.32E-03	84	TRUE	TRUE
1	TRNIDATST	425	433	9	HLA-A*02:01	425	84	0.00931966	8.40E+01	9.32E-03	84	TRUE	TRUE
1	PFGEVFNAT	324	332	9	HLA-A*02:01	324	84	0.0093261	8.40E+01	9.33E-03	84	TRUE	TRUE
1	AWERKKISN	339	347	9	HLA-A*02:01	339	84	0.00932825	8.40E+01	9.33E-03	84	TRUE	TRUE
1	SDAFSLDVS	165	173	9	HLA-A*02:01	165	84	0.00934114	8.40E+01	9.34E-03	84	TRUE	TRUE
1	WNTRNIDAT	423	431	9	HLA-A*02:01	423	84	0.0093713	8.40E+01	9.37E-03	84	TRUE	TRUE
1	LIKNQCVNF	519	527	9	HLA-A*02:01	519	84	0.00952796	8.40E+01	9.53E-03	84	TRUE	TRUE
1	QTSNFRVVP	301	309	9	HLA-A*02:01	301	84	0.00955213	8.40E+01	9.55E-03	84	TRUE	TRUE
1	HGVVFLHVT	1040	1048	9	HLA-A*02:01	1040	84	0.00957635	8.40E+01	9.58E-03	84	TRUE	TRUE
1	PNYTQHTSS	28	36	9	HLA-A*02:01	28	84	0.00960727	8.40E+01	9.61E-03	84	TRUE	TRUE
1	NESLIDLQE	1176	1184	9	HLA-A*02:01	1176	84	0.00966051	8.40E+01	9.66E-03	84	TRUE	TRUE
1	REFVFKNKD	183	191	9	HLA-A*02:01	183	84	0.00966273	8.40E+01	9.66E-03	84	TRUE	TRUE
1	STNVVIRAC	120	128	9	HLA-A*02:01	120	84	0.00966718	8.40E+01	9.67E-03	84	TRUE	TRUE
1	EGVFVFNGT	1074	1082	9	HLA-A*02:01	1074	85	0.0097949	8.50E+01	9.79E-03	85	TRUE	TRUE
1	VRGWVFGST	98	106	9	HLA-A*02:01	98	85	0.00984918	8.50E+01	9.85E-03	85	TRUE	TRUE
1	GSDLDRCTT	13	21	9	HLA-A*02:01	13	85	0.00992659	8.50E+01	9.93E-03	85	TRUE	TRUE
1	LTGTGVLTP	532	540	9	HLA-A*02:01	532	85	0.00993345	8.50E+01	9.93E-03	85	TRUE	TRUE
1	TTSTALGKL	922	930	9	HLA-A*02:01	922	85	0.00995405	8.50E+01	9.95E-03	85	TRUE	TRUE
1	GSTMNNKSQ	104	112	9	HLA-A*02:01	104	85	0.00996552	8.50E+01	9.97E-03	85	TRUE	TRUE
1	NATKFPSVY	330	338	9	HLA-A*02:01	330	85	0.0099816	8.50E+01	9.98E-03	85	TRUE	TRUE
1	AYFVGYLKP	251	259	9	HLA-A*02:01	251	85	0.00998389	8.50E+01	9.98E-03	85	TRUE	TRUE
1	LYENQKQIA	898	906	9	HLA-A*02:01	898	85	0.010027669	8.50E+01	1.00E-02	85	TRUE	TRUE
1	ADAGFMKQY	811	819	9	HLA-A*02:01	811	85	0.010125116	8.50E+01	1.01E-02	85	TRUE	TRUE
1	PSVYAWERK	335	343	9	HLA-A*02:01	335	85	0.01013678	8.50E+01	1.01E-02	85	TRUE	TRUE
1	LVKQLSSNF	944	952	9	HLA-A*02:01	944	85	0.010185914	8.50E+01	1.02E-02	85	TRUE	TRUE
1	VKSFEIDKG	290	298	9	HLA-A*02:01	290	85	0.010185914	8.50E+01	1.02E-02	85	TRUE	TRUE
1	DINARDLIC	825	833	9	HLA-A*02:01	825	85	0.010197648	8.50E+01	1.02E-02	85	TRUE	TRUE
1	SVRDPKTSE	561	569	9	HLA-A*02:01	561	86	0.010399202	8.60E+01	1.04E-02	86	TRUE	TRUE
1	GVLTPSSKR	536	544	9	HLA-A*02:01	536	86	0.010505095	8.60E+01	1.05E-02	86	TRUE	TRUE
1	ATKLNDLCF	371	379	9	HLA-A*02:01	371	86	0.01051962	8.60E+01	1.05E-02	86	TRUE	TRUE
1	KSIVAYTMS	672	680	9	HLA-A*02:01	672	86	0.010539014	8.60E+01	1.05E-02	86	TRUE	TRUE
1	ATEKSNVVR	91	99	9	HLA-A*02:01	91	86	0.010553585	8.60E+01	1.06E-02	86	TRUE	TRUE
1	SFCTQLNRA	740	748	9	HLA-A*02:01	740	86	0.010614511	8.60E+01	1.06E-02	86	TRUE	TRUE
1	ISSVLNDIL	955	963	9	HLA-A*02:01	955	86	0.010685627	8.60E+01	1.07E-02	86	TRUE	TRUE
1	NDLCFSNVY	375	383	9	HLA-A*02:01	375	86	0.0107004	8.60E+01	1.07E-02	86	TRUE	TRUE
1	VSEKSGNFK	172	180	9	HLA-A*02:01	172	86	0.010717661	8.60E+01	1.07E-02	86	TRUE	TRUE
1	GNNVFQTQA	625	633	9	HLA-A*02:01	625	86	0.010834279	8.60E+01	1.08E-02	86	TRUE	TRUE
1	QFGRDVSDF	550	558	9	HLA-A*02:01	550	86	0.010836774	8.60E+01	1.08E-02	86	TRUE	TRUE
1	GNPVIPFKD	77	85	9	HLA-A*02:01	77	87	0.010861755	8.70E+01	1.09E-02	87	TRUE	TRUE
1	LIGAEHVDT	636	644	9	HLA-A*02:01	636	87	0.010864256	8.70E+01	1.09E-02	87	TRUE	TRUE
1	YICGDSTEC	723	731	9	HLA-A*02:01	723	87	0.01092195	8.70E+01	1.09E-02	87	TRUE	TRUE
1	APNYTQHTS	27	35	9	HLA-A*02:01	27	87	0.010929493	8.70E+01	1.09E-02	87	TRUE	TRUE
1	SNVVRGWVF	95	103	9	HLA-A*02:01	95	87	0.01095973	8.70E+01	1.10E-02	87	TRUE	TRUE
1	QPELDSFKE	1124	1132	9	HLA-A*02:01	1124	87	0.011030622	8.70E+01	1.10E-02	87	TRUE	TRUE
1	TSTALGKLQ	923	931	9	HLA-A*02:01	923	87	0.0110535	8.70E+01	1.11E-02	87	TRUE	TRUE
1	CSCGSCCKF	1230	1238	9	HLA-A*02:01	1230	87	0.011086641	8.70E+01	1.11E-02	87	TRUE	TRUE
1	QIITTDNTF	1095	1103	9	HLA-A*02:01	1095	87	0.01122018	8.70E+01	1.12E-02	87	TRUE	TRUE
1	DEIFRSDTL	44	52	9	HLA-A*02:01	44	87	0.011277167	8.70E+01	1.13E-02	87	TRUE	TRUE
1	LSSNFGAIS	948	956	9	HLA-A*02:01	948	87	0.011397248	8.70E+01	1.14E-02	87	TRUE	TRUE
1	RKKISNCVA	342	350	9	HLA-A*02:01	342	87	0.011428783	8.70E+01	1.14E-02	87	TRUE	TRUE
1	YHTVSLLRS	660	668	9	HLA-A*02:01	660	87	0.011492116	8.70E+01	1.15E-02	87	TRUE	TRUE
1	CFSNVYADS	378	386	9	HLA-A*02:01	378	88	0.01163858	8.80E+01	1.16E-02	88	TRUE	TRUE
1	KGACSCGSC	1227	1235	9	HLA-A*02:01	1227	88	0.011649305	8.80E+01	1.16E-02	88	TRUE	TRUE
1	CYGVSATKL	366	374	9	HLA-A*02:01	366	88	0.011808643	8.80E+01	1.18E-02	88	TRUE	TRUE
1	SGSDLDRCT	12	20	9	HLA-A*02:01	12	88	0.011857687	8.80E+01	1.19E-02	88	TRUE	TRUE
1	SMAKTSVDC	712	720	9	HLA-A*02:01	712	88	0.011893235	8.80E+01	1.19E-02	88	TRUE	TRUE
1	SCCKFDEDD	1234	1242	9	HLA-A*02:01	1234	88	0.011898713	8.80E+01	1.19E-02	88	TRUE	TRUE
1	EIFRSDTLY	45	53	9	HLA-A*02:01	45	88	0.01191242	8.80E+01	1.19E-02	88	TRUE	TRUE
1	EPVLKGVKL	1244	1252	9	HLA-A*02:01	1244	88	0.012067019	8.80E+01	1.21E-02	88	TRUE	TRUE
1	GFNTLKPIF	212	220	9	HLA-A*02:01	212	88	0.012103194	8.80E+01	1.21E-02	88	TRUE	TRUE
1	LRSTSQKSI	666	674	9	HLA-A*02:01	666	88	0.01211714	8.80E+01	1.21E-02	88	TRUE	TRUE
1	LTAFSPAQD	235	243	9	HLA-A*02:01	235	88	0.012164661	8.80E+01	1.22E-02	88	TRUE	TRUE
1	EAEVQIDRL	970	978	9	HLA-A*02:01	970	88	0.012195511	8.80E+01	1.22E-02	88	TRUE	TRUE
1	NTFVSGNCD	1101	1109	9	HLA-A*02:01	1101	89	0.012362321	8.90E+01	1.24E-02	89	TRUE	TRUE
1	NKVTLADAG	806	814	9	HLA-A*02:01	806	89	0.012387966	8.90E+01	1.24E-02	89	TRUE	TRUE
1	QAGCLIGAE	632	640	9	HLA-A*02:01	632	89	0.012393672	8.90E+01	1.24E-02	89	TRUE	TRUE
1	TVCGPKLST	509	517	9	HLA-A*02:01	509	89	0.012735031	8.90E+01	1.27E-02	89	TRUE	TRUE
1	IWGTSAAAY	244	252	9	HLA-A*02:01	244	89	0.012811501	8.90E+01	1.28E-02	89	TRUE	TRUE
1	HVTYVPSQE	1046	1054	9	HLA-A*02:01	1046	89	0.012858787	8.90E+01	1.29E-02	89	TRUE	TRUE
1	SFKEELDKY	1129	1137	9	HLA-A*02:01	1129	90	0.013424558	9.00E+01	1.34E-02	90	TRUE	TRUE
1	KDGFLYVYK	190	198	9	HLA-A*02:01	190	90	0.01344931	9.00E+01	1.34E-02	90	TRUE	TRUE
1	IQKEIDRLN	1161	1169	9	HLA-A*02:01	1161	90	0.013464803	9.00E+01	1.35E-02	90	TRUE	TRUE

seq #	peptide	start	end	peptide length	allele	peptide index	median binding	comblib_sidney	comblib_sidney	legacy IC50	legacy rank	scores equal	percentiles equal
1	DKGIYQTSN	296	304	9	HLA-A*02:01	296	90	0.013471005	9.00E+01	1.35E-02	90	TRUE	TRUE
1	NNSTNVVIR	118	126	9	HLA-A*02:01	118	90	0.013905929	9.00E+01	1.39E-02	90	TRUE	TRUE
1	KPTKRSFIE	793	801	9	HLA-A*02:01	793	90	0.014015223	9.00E+01	1.40E-02	90	TRUE	TRUE
1	NFRVVPSGD	304	312	9	HLA-A*02:01	304	90	0.014028137	9.00E+01	1.40E-02	90	TRUE	TRUE
1	AVLYQDVNC	595	603	9	HLA-A*02:01	595	90	0.014037831	9.00E+01	1.40E-02	90	TRUE	TRUE
1	TAGWTFGAG	865	873	9	HLA-A*02:01	865	90	0.014066952	9.00E+01	1.41E-02	90	TRUE	TRUE
1	CSVKSFEID	288	296	9	HLA-A*02:01	288	91	0.014924507	9.10E+01	1.49E-02	91	TRUE	TRUE
1	TSSMRGVYY	34	42	9	HLA-A*02:01	34	91	0.014955468	9.10E+01	1.50E-02	91	TRUE	TRUE
1	ATKFPSVYA	331	339	9	HLA-A*02:01	331	91	0.014996848	9.10E+01	1.50E-02	91	TRUE	TRUE
1	LSRLDKVEA	963	971	9	HLA-A*02:01	963	91	0.015128644	9.10E+01	1.51E-02	91	TRUE	TRUE
1	GVVFLHVTY	1041	1049	9	HLA-A*02:01	1041	91	0.015170504	9.10E+01	1.52E-02	91	TRUE	TRUE
1	SQERNFTTA	1052	1060	9	HLA-A*02:01	1052	91	0.015240528	9.10E+01	1.52E-02	91	TRUE	TRUE
1	VFQTQAGCL	628	636	9	HLA-A*02:01	628	91	0.01527214	9.10E+01	1.53E-02	91	TRUE	TRUE
1	PDVDLGDIS	1144	1152	9	HLA-A*02:01	1144	91	0.015317927	9.10E+01	1.53E-02	91	TRUE	TRUE
1	SDFTDSVRD	556	564	9	HLA-A*02:01	556	91	0.015328512	9.10E+01	1.53E-02	91	TRUE	TRUE
1	SKRFQPFQQ	542	550	9	HLA-A*02:01	542	91	0.015370925	9.10E+01	1.54E-02	91	TRUE	TRUE
1	LNRALSGIA	745	753	9	HLA-A*02:01	745	92	0.01544543	9.20E+01	1.54E-02	92	TRUE	TRUE
1	EKSGNFKHL	174	182	9	HLA-A*02:01	174	92	0.01555966	9.20E+01	1.56E-02	92	TRUE	TRUE
1	AALVSGTAT	857	865	9	HLA-A*02:01	857	92	0.015602709	9.20E+01	1.56E-02	92	TRUE	TRUE
1	TQNVLYENQ	894	902	9	HLA-A*02:01	894	92	0.01562068	9.20E+01	1.56E-02	92	TRUE	TRUE
1	HVDTSYECD	641	649	9	HLA-A*02:01	641	92	0.01564228	9.20E+01	1.56E-02	92	TRUE	TRUE
1	ACNFELCDN	127	135	9	HLA-A*02:01	127	92	0.015689171	9.20E+01	1.57E-02	92	TRUE	TRUE
1	RFNGIGVTQ	887	895	9	HLA-A*02:01	887	92	0.01620317	9.20E+01	1.62E-02	92	TRUE	TRUE
1	YSNVTGFHT	63	71	9	HLA-A*02:01	63	92	0.01643615	9.20E+01	1.64E-02	92	TRUE	TRUE
1	VFVFNGTSW	1076	1084	9	HLA-A*02:01	1076	92	0.016542456	9.20E+01	1.65E-02	92	TRUE	TRUE
1	YAWERKKIS	338	346	9	HLA-A*02:01	338	92	0.016565326	9.20E+01	1.66E-02	92	TRUE	TRUE
1	KTSVDCNMY	715	723	9	HLA-A*02:01	715	92	0.016595869	9.20E+01	1.66E-02	92	TRUE	TRUE
1	IAAEQDRNT	752	760	9	HLA-A*02:01	752	92	0.016641789	9.20E+01	1.66E-02	92	TRUE	TRUE
1	GFLYVYKGY	192	200	9	HLA-A*02:01	192	92	0.016745573	9.20E+01	1.67E-02	92	TRUE	TRUE
1	RSDTLYLTQ	48	56	9	HLA-A*02:01	48	92	0.016768723	9.20E+01	1.68E-02	92	TRUE	TRUE
1	LNCYWPLND	472	480	9	HLA-A*02:01	472	92	0.016861647	9.20E+01	1.69E-02	92	TRUE	TRUE
1	CSCLKGACS	1223	1231	9	HLA-A*02:01	1223	93	0.017013748	9.30E+01	1.70E-02	93	TRUE	TRUE
1	IADYNYKLP	405	413	9	HLA-A*02:01	405	93	0.017151417	9.30E+01	1.72E-02	93	TRUE	TRUE
1	GDSTECANL	726	734	9	HLA-A*02:01	726	93	0.017354016	9.30E+01	1.74E-02	93	TRUE	TRUE
1	DVSEKSGNF	171	179	9	HLA-A*02:01	171	93	0.017790985	9.30E+01	1.78E-02	93	TRUE	TRUE
1	DSSIAYSNN	684	692	9	HLA-A*02:01	684	93	0.017790985	9.30E+01	1.78E-02	93	TRUE	TRUE
1	IFKLPLGIN	219	227	9	HLA-A*02:01	219	93	0.017992852	9.30E+01	1.80E-02	93	TRUE	TRUE
1	AYTMSLGAD	676	684	9	HLA-A*02:01	676	93	0.018167702	9.30E+01	1.82E-02	93	TRUE	TRUE
1	EFVFKNKDG	184	192	9	HLA-A*02:01	184	93	0.018276793	9.30E+01	1.83E-02	93	TRUE	TRUE
1	KDGIYFAAT	84	92	9	HLA-A*02:01	84	94	0.018424682	9.40E+01	1.84E-02	94	TRUE	TRUE
1	TQHTSSMRG	31	39	9	HLA-A*02:01	31	94	0.018535316	9.40E+01	1.85E-02	94	TRUE	TRUE
1	SYHTVSLLR	659	667	9	HLA-A*02:01	659	94	0.01864661	9.40E+01	1.86E-02	94	TRUE	TRUE
1	NYNYKYRYL	435	443	9	HLA-A*02:01	435	94	0.018758582	9.40E+01	1.88E-02	94	TRUE	TRUE
1	LNDILSRLD	959	967	9	HLA-A*02:01	959	94	0.018832154	9.40E+01	1.88E-02	94	TRUE	TRUE
1	AIPTNFSIS	695	703	9	HLA-A*02:01	695	94	0.01892344	9.40E+01	1.89E-02	94	TRUE	TRUE
1	SVITPGTNA	582	590	9	HLA-A*02:01	582	94	0.01914256	9.40E+01	1.91E-02	94	TRUE	TRUE
1	SGNFKHLRE	176	184	9	HLA-A*02:01	176	94	0.01918669	9.40E+01	1.92E-02	94	TRUE	TRUE
1	DVVRFPNIT	312	320	9	HLA-A*02:01	312	94	0.019373139	9.40E+01	1.94E-02	94	TRUE	TRUE
1	MSECVLGQS	1011	1019	9	HLA-A*02:01	1011	94	0.01976059	9.40E+01	1.98E-02	94	TRUE	TRUE
1	RDPKTSEIL	563	571	9	HLA-A*02:01	563	94	0.019787913	9.40E+01	1.98E-02	94	TRUE	TRUE
1	LTSGSDLDR	10	18	9	HLA-A*02:01	10	94	0.019787913	9.40E+01	1.98E-02	94	TRUE	TRUE
1	PIDVVRDLP	202	210	9	HLA-A*02:01	202	94	0.01981071	9.40E+01	1.98E-02	94	TRUE	TRUE
1	GSCCKFDED	1233	1241	9	HLA-A*02:01	1233	94	0.01990215	9.40E+01	1.99E-02	94	TRUE	TRUE
1	MAKTSVDCN	713	721	9	HLA-A*02:01	713	94	0.019925077	9.40E+01	1.99E-02	94	TRUE	TRUE
1	NVTGFHTIN	65	73	9	HLA-A*02:01	65	94	0.01996181	9.40E+01	2.00E-02	94	TRUE	TRUE
1	YTQHTSSMR	30	38	9	HLA-A*02:01	30	94	0.019971009	9.40E+01	2.00E-02	94	TRUE	TRUE
1	NFNFNGLTG	526	534	9	HLA-A*02:01	526	94	0.01999401	9.40E+01	2.00E-02	94	TRUE	TRUE
1	DIPIGAGIC	649	657	9	HLA-A*02:01	649	94	0.02006781	9.40E+01	2.01E-02	94	TRUE	TRUE
1	TGNYNYKYR	433	441	9	HLA-A*02:01	433	94	0.0201558	9.40E+01	2.02E-02	94	TRUE	TRUE
1	TSCCSCLKG	1220	1228	9	HLA-A*02:01	1220	95	0.020361042	9.50E+01	2.04E-02	95	TRUE	TRUE
1	VRQIAPGQT	394	402	9	HLA-A*02:01	394	95	0.0204833	9.50E+01	2.05E-02	95	TRUE	TRUE
1	DRLITGRLQ	976	984	9	HLA-A*02:01	976	95	0.020715718	9.50E+01	2.07E-02	95	TRUE	TRUE
1	AVSKPMGTQ	139	147	9	HLA-A*02:01	139	95	0.020878534	9.50E+01	2.09E-02	95	TRUE	TRUE
1	SGNCDVVIG	1105	1113	9	HLA-A*02:01	1105	95	0.020941125	9.50E+01	2.09E-02	95	TRUE	TRUE
1	CCKFDEDDS	1235	1243	9	HLA-A*02:01	1235	95	0.02100874	9.50E+01	2.10E-02	95	TRUE	TRUE
1	KPTTFMLKY	258	266	9	HLA-A*02:01	258	95	0.02111543	9.50E+01	2.11E-02	95	TRUE	TRUE
1	VMPVSMAKT	708	716	9	HLA-A*02:01	708	95	0.02123244	9.50E+01	2.12E-02	95	TRUE	TRUE
1	PDPLKPTKR	789	797	9	HLA-A*02:01	789	95	0.021261799	9.50E+01	2.13E-02	95	TRUE	TRUE
1	CGKGYHLMS	1025	1033	9	HLA-A*02:01	1025	95	0.02162719	9.50E+01	2.16E-02	95	TRUE	TRUE
1	GWVFGSTMN	100	108	9	HLA-A*02:01	100	95	0.021667061	9.50E+01	2.17E-02	95	TRUE	TRUE
1	KPCTPPALN	465	473	9	HLA-A*02:01	465	95	0.022069881	9.50E+01	2.21E-02	95	TRUE	TRUE
1	DYSVLYNST	351	359	9	HLA-A*02:01	351	96	0.022356304	9.60E+01	2.24E-02	96	TRUE	TRUE
1	TYVTQQLIR	988	996	9	HLA-A*02:01	988	96	0.02262559	9.60E+01	2.26E-02	96	TRUE	TRUE
1	NTVYDPLQP	1117	1125	9	HLA-A*02:01	1117	96	0.023019718	9.60E+01	2.30E-02	96	TRUE	TRUE
1	VSVITPGTN	581	589	9	HLA-A*02:01	581	96	0.023227368	9.60E+01	2.32E-02	96	TRUE	TRUE
1	QSKRVDFCG	1018	1026	9	HLA-A*02:01	1018	96	0.023323837	9.60E+01	2.33E-02	96	TRUE	TRUE
1	VVKGDDVRQ	388	396	9	HLA-A*02:01	388	96	0.02353423	9.60E+01	2.35E-02	96	TRUE	TRUE
1	DSFVVKGDD	385	393	9	HLA-A*02:01	385	96	0.023555916	9.60E+01	2.36E-02	96	TRUE	TRUE
1	TGRLQSLQT	980	988	9	HLA-A*02:01	980	96	0.02360478	9.60E+01	2.36E-02	96	TRUE	TRUE
1	THTMIFDNA	148	156	9	HLA-A*02:01	148	96	0.023680998	9.60E+01	2.37E-02	96	TRUE	TRUE
1	RYLRHGKLR	441	449	9	HLA-A*02:01	441	96	0.023746521	9.60E+01	2.37E-02	96	TRUE	TRUE
1	AAEQDRNTR	753	761	9	HLA-A*02:01	753	96	0.02396625	9.60E+01	2.40E-02	96	TRUE	TRUE
1	APGQTGVIA	398	406	9	HLA-A*02:01	398	96	0.024355665	9.60E+01	2.44E-02	96	TRUE	TRUE
1	ATVCGPKLS	508	516	9	HLA-A*02:01	508	96	0.02471724	9.60E+01	2.47E-02	96	TRUE	TRUE
1	NVPFSPDGK	457	465	9	HLA-A*02:01	457	97	0.024963185	9.70E+01	2.50E-02	97	TRUE	TRUE
1	LNEVAKNLN	1168	1176	9	HLA-A*02:01	1168	97	0.0258226	9.70E+01	2.58E-02	97	TRUE	TRUE
1	LKPTTFMLK	257	265	9	HLA-A*02:01	257	97	0.026079544	9.70E+01	2.61E-02	97	TRUE	TRUE
1	TNVVIRACN	121	129	9	HLA-A*02:01	121	97	0.026730064	9.70E+01	2.67E-02	97	TRUE	TRUE
1	LGKLQDVVN	927	935	9	HLA-A*02:01	927	97	0.026804024	9.70E+01	2.68E-02	97	TRUE	TRUE
1	SFGGVSVIT	577	585	9	HLA-A*02:01	577	97	0.02697118	9.70E+01	2.70E-02	97	TRUE	TRUE
1	DVNCTDVST	600	608	9	HLA-A*02:01	600	98	0.028131961	9.80E+01	2.81E-02	98	TRUE	TRUE
1	KYRYLRHGK	439	447	9	HLA-A*02:01	439	98	0.0296142	9.80E+01	2.96E-02	98	TRUE	TRUE
1	NFTTAPAIC	1056	1064	9	HLA-A*02:01	1056	98	0.029661971	9.80E+01	2.97E-02	98	TRUE	TRUE
1	NGTITDAVD	269	277	9	HLA-A*02:01	269	98	0.030276105	9.80E+01	3.03E-02	98	TRUE	TRUE
1	PDEIFRSDT	43	51	9	HLA-A*02:01	43	98	0.03042286	9.80E+01	3.04E-02	98	TRUE	TRUE
1	ANQFNKAIS	906	914	9	HLA-A*02:01	906	98	0.030605537	9.80E+01	3.06E-02	98	TRUE	TRUE
1	SKRVDFCGK	1019	1027	9	HLA-A*02:01	1019	98	0.031426788	9.80E+01	3.14E-02	98	TRUE	TRUE
1	CGPKLSTDL	511	519	9	HLA-A*02:01	511	98	0.03184198	9.80E+01	3.18E-02	98	TRUE	TRUE
1	AWRIYSTGN	618	626	9	HLA-A*02:01	618	98	0.03279442	9.80E+01	3.28E-02	98	TRUE	TRUE
1	KNHTSPDVD	1139	1147	9	HLA-A*02:01	1139	99	0.033181804	99	0.033181804	99	TRUE	TRUE
1	TFKCYGVSA	363	371	9	HLA-A*02:01	363	99	0.03356603	99	0.03356603	99	TRUE	TRUE
1	CTTFDDVQA	19	27	9	HLA-A*02:01	19	99	0.033884416	99	0.033884416	99	TRUE	TRUE

seq #	peptide	start	end	peptide length	allele	peptide index	median binding	comblib_sidney	comblib_sidney	legacy IC50	legacy rank	scores equal	percentiles equal	
1	VVNIQKEID	1158	1166	9	HLA-A*02:01	1158	99	0.0352452	99	0.0352452	99	TRUE	TRUE	
1	NYKYRYLRH	437	445	9	HLA-A*02:01	437	99	0.037792011	99	0.037792011	99	TRUE	TRUE	
1	TFEYISDAF	160	168	9	HLA-A*02:01	160	99	0.03809781	99	0.03809781	99	TRUE	TRUE	
1	TINHTFGNP	71	79	9	HLA-A*02:01	71	99	0.038556713	99	0.038556713	99	TRUE	TRUE	
1	LCCMTSCCS	1216	1224	9	HLA-A*02:01	1216	99	0.03948208	99	0.03948208	99	TRUE	TRUE	
1	GAEHVDTSY	638	646	9	HLA-A*02:01	638	100	0.04075679	100	0.04075679	100	TRUE	TRUE	
1	ACSCGSCCK	1229	1237	9	HLA-A*02:01	1229	100	0.04137138	100	0.04137138	100	TRUE	TRUE	
1	DTSYECDIP	643	651	9	HLA-A*02:01	643	100	0.042589251	100	0.042589251	100	TRUE	TRUE	
1	DNTFVSGNC	1100	1108	9	HLA-A*02:01	1100	100	0.04584585	100	0.04584585	100	TRUE	TRUE	
1	RCTTFDDVQ	18	26	9	HLA-A*02:01	18	100	0.045930376	100	0.045930376	100	TRUE	TRUE	
1	LDKYFKNHT	1134	1142	9	HLA-A*02:01	1134	100	0.04628071	100	0.04628071	100	TRUE	TRUE	
1	EVQIDRLIT	972	980	9	HLA-A*02:01	972	100	0.04786301	100	0.04786301	100	TRUE	TRUE	
1	NPVIPFKDG	78	86	9	HLA-A*02:01	78	100	0.04792918	100	0.04792918	100	TRUE	TRUE	
1	LREFVFKNK	182	190	9	HLA-A*02:01	182	100	0.05066406	100	0.05066406	100	TRUE	TRUE	
1	DSVRDPKTS	560	568	9	HLA-A*02:01	560	100	0.05546257	100	0.05546257	100	TRUE	TRUE	
1	SEPVLKGVK	1243	1251	9	HLA-A*02:01	1243	100	0.05679368	100	0.05679368	100	TRUE	TRUE	
1	CCSCLKGAC	1222	1230	9	HLA-A*02:01	1222	100	0.06218706	100	0.06218706	100	TRUE	TRUE	
1	AICHEGKAY	1062	1070	9	HLA-A*02:01	1062	100	0.06309573	100	0.06309573	100	TRUE	TRUE	
1	VIPFKDGIY	80	88	9	HLA-A*02:01	80	100	0.06324119	100	0.06324119	100	TRUE	TRUE	
1	QKEIDRLNE	1162	1170	9	HLA-A*02:01	1162	100	0.06367955	100	0.06367955	100	TRUE	TRUE	
1	LRPFERDIS	448	456	9	HLA-A*02:01	448	100	0.09298224	100	0.09298224	100	TRUE	TRUE	
1	YGECLGDIN	819	827	9	HLA-A*02:01	819	100	0.09981596	100	0.09981596	100	TRUE	TRUE	
1	SFEIDKGIY	292	300	9	HLA-A*02:01	292	100	0.116654098	100	0.116654098	100	TRUE	TRUE	

seq #	peptide	start	end	peptide length	allele	peptide index	median binding	mhcnp score	mhcnp percent	legacy score	scores equal
1	VLNDILSRL	958	966	9	HLA-A*02:01	958	0.33	0.51407719	0.33	0.5141	TRUE
1	PLAELKCSV	282	290	9	HLA-A*02:01	282	0.38	0.50286753	0.38	0.5029	TRUE
1	TALGKLQDV	925	933	9	HLA-A*02:01	925	0.47	0.46350682	0.47	0.4635	TRUE
1	ALGKLQDVV	926	934	9	HLA-A*02:01	926	0.78	0.38611834	0.78	0.3861	TRUE
1	FNATKFPSV	329	337	9	HLA-A*02:01	329	1.1	0.33525533	1.1	0.3353	TRUE
1	AAPHGVVFL	1037	1045	9	HLA-A*02:01	1037	1.5	0.29246455	1.5	0.2925	TRUE
1	ALNTLVKQL	940	948	9	HLA-A*02:01	940	1.5	0.29198482	1.5	0.292	TRUE
1	QIAPGQTGV	396	404	9	HLA-A*02:01	396	1.6	0.29007222	1.6	0.2901	TRUE
1	GLTVLPPLL	839	847	9	HLA-A*02:01	839	1.7	0.27685497	1.7	0.2769	TRUE
1	KEIDRLNEV	1163	1171	9	HLA-A*02:01	1163	1.8	0.27137281	1.8	0.2714	TRUE
1	NTLKPIFKL	214	222	9	HLA-A*02:01	214	2.5	0.21385032	2.5	0.2139	TRUE
1	GINASVVNI	1153	1161	9	HLA-A*02:01	1153	2.5	0.20680475	2.5	0.2068	TRUE
1	NLNESLIDL	1174	1182	9	HLA-A*02:01	1174	2.6	0.20476932	2.6	0.2048	TRUE
1	RLNEVAKNL	1167	1175	9	HLA-A*02:01	1167	2.6	0.20191155	2.6	0.2019	TRUE
1	LITGRLQSL	978	986	9	HLA-A*02:01	978	2.6	0.20122544	2.6	0.2012	TRUE
1	SLIDLQELG	1178	1186	9	HLA-A*02:01	1178	2.7	0.19946169	2.7	0.1995	TRUE
1	KLQDVVNQN	929	937	9	HLA-A*02:01	929	2.8	0.19512198	2.8	0.1951	TRUE
1	RLDKVEAEV	965	973	9	HLA-A*02:01	965	2.9	0.19119711	2.9	0.1912	TRUE
1	VDLGDISGI	1146	1154	9	HLA-A*02:01	1146	3.1	0.18112875	3.1	0.1811	TRUE
1	VIADYNYKL	404	412	9	HLA-A*02:01	404	3.4	0.17094901	3.4	0.1709	TRUE
1	FKLPLGINI	220	228	9	HLA-A*02:01	220	3.6	0.16503852	3.6	0.165	TRUE
1	NIQKEIDRL	1160	1168	9	HLA-A*02:01	1160	3.7	0.16283848	3.7	0.1628	TRUE
1	DILSRLDKV	961	969	9	HLA-A*02:01	961	3.8	0.1612244	3.8	0.1612	TRUE
1	ILPDPLKPT	787	795	9	HLA-A*02:01	787	3.9	0.15693814	3.9	0.1569	TRUE
1	VLYENQKQI	897	905	9	HLA-A*02:01	897	3.9	0.15581894	3.9	0.1558	TRUE
1	GIGYQPYRV	488	496	9	HLA-A*02:01	488	4.1	0.15196815	4.1	0.152	TRUE
1	GAALQIPFA	873	881	9	HLA-A*02:01	873	4.1	0.15024547	4.1	0.1502	TRUE
1	AISSVLNDI	954	962	9	HLA-A*02:01	954	4.5	0.14192251	4.5	0.1419	TRUE
1	NGLTVLPPL	838	846	9	HLA-A*02:01	838	4.6	0.13818821	4.6	0.1382	TRUE
1	QILPDPLKP	786	794	9	HLA-A*02:01	786	4.6	0.1373474	4.6	0.1373	TRUE
1	RVVPSGDVV	306	314	9	HLA-A*02:01	306	4.8	0.13038783	4.8	0.1304	TRUE
1	CLGDINARD	822	830	9	HLA-A*02:01	822	5	0.12752418	5	0.1275	TRUE
1	LGDISGINA	1148	1156	9	HLA-A*02:01	1148	5.1	0.12625269	5.1	0.1263	TRUE
1	YVTQQLIRA	989	997	9	HLA-A*02:01	989	5.1	0.12547042	5.1	0.1255	TRUE
1	ALSGIAAEQ	748	756	9	HLA-A*02:01	748	5.4	0.12040694	5.4	0.1204	TRUE
1	FAATEKSNV	89	97	9	HLA-A*02:01	89	5.4	0.11990187	5.4	0.1199	TRUE
1	NGIGVTQNV	889	897	9	HLA-A*02:01	889	5.6	0.11488156	5.6	0.1149	TRUE
1	FKNHTSPDV	1138	1146	9	HLA-A*02:01	1138	5.7	0.11402503	5.7	0.114	TRUE
1	QIQESLTTT	915	923	9	HLA-A*02:01	915	5.7	0.11271685	5.7	0.1127	TRUE
1	KQMYKTPTL	768	776	9	HLA-A*02:01	768	5.7	0.11180853	5.7	0.1118	TRUE
1	RAAEIRASA	996	1004	9	HLA-A*02:01	996	5.8	0.11008365	5.8	0.1101	TRUE
1	AISQIQESL	912	920	9	HLA-A*02:01	912	5.9	0.10819686	5.9	0.1082	TRUE
1	AATEKSNVV	90	98	9	HLA-A*02:01	90	6.1	0.10555933	6.1	0.1056	TRUE
1	KGYQPIDVV	198	206	9	HLA-A*02:01	198	6.1	0.10498001	6.1	0.105	TRUE
1	DISGINASV	1150	1158	9	HLA-A*02:01	1150	6.2	0.10321196	6.2	0.1032	TRUE
1	KAISQIQES	911	919	9	HLA-A*02:01	911	6.2	0.10217242	6.2	0.1022	TRUE
1	TDVSTAIHA	604	612	9	HLA-A*02:01	604	6.3	0.10088147	6.3	0.1009	TRUE
1	LLNAPATVC	503	511	9	HLA-A*02:01	503	6.3	0.10081947	6.3	0.1008	TRUE
1	YQPYRVVVL	491	499	9	HLA-A*02:01	491	6.4	0.09923114	6.4	0.0992	TRUE
1	LQIPFAMQM	876	884	9	HLA-A*02:01	876	6.7	0.09569686	6.7	0.0957	TRUE
1	STAIHADQL	607	615	9	HLA-A*02:01	607	6.8	0.09487213	6.8	0.0949	TRUE
1	ELLNAPATV	502	510	9	HLA-A*02:01	502	6.8	0.09470173	6.8	0.0947	TRUE
1	PIFKLPLGI	218	226	9	HLA-A*02:01	218	7.2	0.09102859	7.2	0.091	TRUE
1	MIFDNAFNC	151	159	9	HLA-A*02:01	151	7.2	0.09080319	7.2	0.0908	TRUE
1	FSLDVSEKS	168	176	9	HLA-A*02:01	168	7.2	0.0904238	7.2	0.0904	TRUE
1	SMAKTSVDC	712	720	9	HLA-A*02:01	712	7.4	0.08911998	7.4	0.0891	TRUE
1	VQIDRLITG	973	981	9	HLA-A*02:01	973	7.7	0.08457596	7.7	0.0846	TRUE
1	IINNSTNVV	116	124	9	HLA-A*02:01	116	7.8	0.0833547	7.8	0.0834	TRUE
1	AALQIPFAM	874	882	9	HLA-A*02:01	874	7.9	0.08136104	7.9	0.0814	TRUE
1	SNFGAISSV	950	958	9	HLA-A*02:01	950	8	0.07974172	8	0.0797	TRUE
1	SVVNIQKEI	1157	1165	9	HLA-A*02:01	1157	8	0.07934265	8	0.0793	TRUE
1	RALSGIAAE	747	755	9	HLA-A*02:01	747	8.1	0.07921456	8.1	0.0792	TRUE
1	IAPGQTGVI	397	405	9	HLA-A*02:01	397	8.1	0.07901827	8.1	0.079	TRUE
1	FDEDDSEPV	1238	1246	9	HLA-A*02:01	1238	8.1	0.07882871	8.1	0.0788	TRUE
1	SLQTYVTQQ	985	993	9	HLA-A*02:01	985	8.3	0.07729409	8.3	0.0773	TRUE
1	VRFPNITNL	314	322	9	HLA-A*02:01	314	8.5	0.07440653	8.5	0.0744	TRUE
1	IAIPTNFSI	694	702	9	HLA-A*02:01	694	8.7	0.07231771	8.7	0.0723	TRUE
1	SITTEVMPV	703	711	9	HLA-A*02:01	703	8.7	0.07197921	8.7	0.072	TRUE
1	ILDISPCSF	570	578	9	HLA-A*02:01	570	8.9	0.07010126	8.9	0.0701	TRUE
1	TTAPAICHE	1058	1066	9	HLA-A*02:01	1058	8.9	0.06998456	8.9	0.07	TRUE
1	YLTQDLFLP	53	61	9	HLA-A*02:01	53	9	0.06957757	9	0.0696	TRUE
1	LLFNKVTLA	803	811	9	HLA-A*02:01	803	9.3	0.06622592	9.3	0.0662	TRUE
1	RLQSLQTYV	982	990	9	HLA-A*02:01	982	9.5	0.06518358	9.5	0.0652	TRUE
1	ALVSGTATA	858	866	9	HLA-A*02:01	858	9.6	0.06384429	9.6	0.0638	TRUE
1	ICGDSTECA	724	732	9	HLA-A*02:01	724	9.6	0.06376547	9.6	0.0638	TRUE
1	YLKPTTFML	256	264	9	HLA-A*02:01	256	9.6	0.06342291	9.6	0.0634	TRUE
1	IIINNSTNV	115	123	9	HLA-A*02:01	115	9.8	0.06232173	9.8	0.0623	TRUE
1	CSFGGVSVI	576	584	9	HLA-A*02:01	576	9.8	0.06193857	9.8	0.0619	TRUE
1	GKPCTPPAL	464	472	9	HLA-A*02:01	464	9.9	0.06161229	9.9	0.0616	TRUE
1	VVFLHVTYV	1042	1050	9	HLA-A*02:01	1042	9.9	0.06159169	9.9	0.0616	TRUE

seq #	peptide	start	end	peptide length	allele	peptide index	median binding	mhcnp score	mhcnp percent	legacy score	scores equal
1	GCLIGAEHV	634	642	9	HLA-A*02:01	634	10	0.06115253	10	0.0612	TRUE
1	HLMSFPQAA	1030	1038	9	HLA-A*02:01	1030	10	0.06101688	10	0.061	TRUE
1	NVFQTQAGC	627	635	9	HLA-A*02:01	627	10	0.06088079	10	0.0609	TRUE
1	KLPDDFMGC	411	419	9	HLA-A*02:01	411	11	0.05778696	11	0.0578	TRUE
1	SLTTTSTAL	919	927	9	HLA-A*02:01	919	11	0.05636094	11	0.0564	TRUE
1	RNFTTAPAI	1055	1063	9	HLA-A*02:01	1055	11	0.05553359	11	0.0555	TRUE
1	QLNRALSGI	744	752	9	HLA-A*02:01	744	11	0.05541972	11	0.0554	TRUE
1	YICGDSTEC	723	731	9	HLA-A*02:01	723	11	0.05458271	11	0.0546	TRUE
1	ESLIDLQEL	1177	1185	9	HLA-A*02:01	1177	11	0.05365192	11	0.0537	TRUE
1	RIYSTGNNV	620	628	9	HLA-A*02:01	620	12	0.05246679	12	0.0525	TRUE
1	TNLCPFGEV	320	328	9	HLA-A*02:01	320	12	0.05222114	12	0.0522	TRUE
1	DLLFNKVTL	802	810	9	HLA-A*02:01	802	12	0.05165107	12	0.0517	TRUE
1	NLCPFGEVF	321	329	9	HLA-A*02:01	321	12	0.05052496	12	0.0505	TRUE
1	VRDPKTSEI	562	570	9	HLA-A*02:01	562	12	0.04997827	12	0.05	TRUE
1	FVGYLKPTT	253	261	9	HLA-A*02:01	253	12	0.04936128	12	0.0494	TRUE
1	FGAGAALQI	870	878	9	HLA-A*02:01	870	12	0.04836751	12	0.0484	TRUE
1	FAVSKPMGT	138	146	9	HLA-A*02:01	138	12	0.04764157	12	0.0476	TRUE
1	FPQAAPHGV	1034	1042	9	HLA-A*02:01	1034	12	0.04748009	12	0.0475	TRUE
1	LQTYVTQQL	986	994	9	HLA-A*02:01	986	12	0.04743262	12	0.0474	TRUE
1	VSATKLNDL	369	377	9	HLA-A*02:01	369	13	0.04689567	13	0.0469	TRUE
1	NLAATKMSE	1005	1013	9	HLA-A*02:01	1005	13	0.04609722	13	0.0461	TRUE
1	KLRPFERDI	447	455	9	HLA-A*02:01	447	13	0.04541741	13	0.0454	TRUE
1	FSQILPDPL	784	792	9	HLA-A*02:01	784	13	0.04492102	13	0.0449	TRUE
1	AGLIAIVMV	1204	1212	9	HLA-A*02:01	1204	13	0.04373221	13	0.0437	TRUE
1	TQAGCLIGA	631	639	9	HLA-A*02:01	631	13	0.04335869	13	0.0434	TRUE
1	FIAGLIAIV	1202	1210	9	HLA-A*02:01	1202	13	0.04313334	13	0.0431	TRUE
1	FSISITTEV	700	708	9	HLA-A*02:01	700	14	0.04214322	14	0.0421	TRUE
1	TVLPPLLTD	841	849	9	HLA-A*02:01	841	14	0.04209912	14	0.0421	TRUE
1	RQIAPGQTG	395	403	9	HLA-A*02:01	395	14	0.04189831	14	0.0419	TRUE
1	FMLKYDENG	262	270	9	HLA-A*02:01	262	14	0.04174757	14	0.0417	TRUE
1	DVSDFTDSV	554	562	9	HLA-A*02:01	554	14	0.04121724	14	0.0412	TRUE
1	LLTDDMIAA	846	854	9	HLA-A*02:01	846	14	0.04097741	14	0.041	TRUE
1	QQLIRAAEI	992	1000	9	HLA-A*02:01	992	14	0.03978175	14	0.0398	TRUE
1	ICAQKFNGL	832	840	9	HLA-A*02:01	832	14	0.03974132	14	0.0397	TRUE
1	LQDVVNQNA	930	938	9	HLA-A*02:01	930	14	0.03934413	14	0.0393	TRUE
1	FSPDGKPCT	460	468	9	HLA-A*02:01	460	14	0.03915685	14	0.0392	TRUE
1	ILTAFSPAQ	234	242	9	HLA-A*02:01	234	14	0.03879547	14	0.0388	TRUE
1	VYDPLQPEL	1119	1127	9	HLA-A*02:01	1119	14	0.0387888	14	0.0388	TRUE
1	LSRLDKVEA	963	971	9	HLA-A*02:01	963	14	0.03820193	14	0.0382	TRUE
1	YECDIPIGA	646	654	9	HLA-A*02:01	646	14	0.0376896	14	0.0377	TRUE
1	SLDVSEKSG	169	177	9	HLA-A*02:01	169	14	0.03729119	14	0.0373	TRUE
1	GIGVTQNVL	890	898	9	HLA-A*02:01	890	15	0.03691382	15	0.0369	TRUE
1	SLLRSTSQK	664	672	9	HLA-A*02:01	664	15	0.03684491	15	0.0368	TRUE
1	GFIAGLIAI	1201	1209	9	HLA-A*02:01	1201	15	0.03670214	15	0.0367	TRUE
1	CVLGQSKRV	1014	1022	9	HLA-A*02:01	1014	15	0.03669704	15	0.0367	TRUE
1	PMGTQTHTM	143	151	9	HLA-A*02:01	143	15	0.0363262	15	0.0363	TRUE
1	TTSTALGKL	922	930	9	HLA-A*02:01	922	15	0.03590287	15	0.0359	TRUE
1	SIAYSNNTI	686	694	9	HLA-A*02:01	686	15	0.03576201	15	0.0358	TRUE
1	SISITTEVM	701	709	9	HLA-A*02:01	701	15	0.03553456	15	0.0355	TRUE
1	ILSRLDKVE	962	970	9	HLA-A*02:01	962	15	0.03528217	15	0.0353	TRUE
1	SQIQESLTT	914	922	9	HLA-A*02:01	914	15	0.03522789	15	0.0352	TRUE
1	QIANQFNKA	904	912	9	HLA-A*02:01	904	15	0.03506015	15	0.0351	TRUE
1	CDIPIGAGI	648	656	9	HLA-A*02:01	648	15	0.03500512	15	0.035	TRUE
1	CSQNPLAEL	278	286	9	HLA-A*02:01	278	15	0.03499085	15	0.035	TRUE
1	GDVVRFPNI	311	319	9	HLA-A*02:01	311	15	0.03459702	15	0.0346	TRUE
1	GVSVITPGT	580	588	9	HLA-A*02:01	580	15	0.03415779	15	0.0342	TRUE
1	RDLPSGFNT	207	215	9	HLA-A*02:01	207	15	0.03392293	15	0.0339	TRUE
1	GDISGINAS	1149	1157	9	HLA-A*02:01	1149	15	0.03383185	15	0.0338	TRUE
1	TNASSEVAV	588	596	9	HLA-A*02:01	588	16	0.03348319	16	0.0335	TRUE
1	TMNNKSQSV	106	114	9	HLA-A*02:01	106	16	0.03308691	16	0.0331	TRUE
1	SRLDKVEAE	964	972	9	HLA-A*02:01	964	16	0.03281125	16	0.0328	TRUE
1	AVLYQDVNC	595	603	9	HLA-A*02:01	595	16	0.03267766	16	0.0327	TRUE
1	YKLPDDFMG	410	418	9	HLA-A*02:01	410	16	0.03266822	16	0.0327	TRUE
1	GLTGTGVLT	531	539	9	HLA-A*02:01	531	16	0.03251108	16	0.0325	TRUE
1	KPIFKLPLG	217	225	9	HLA-A*02:01	217	16	0.03223713	16	0.0322	TRUE
1	KNKDGFLYV	188	196	9	HLA-A*02:01	188	16	0.03220487	16	0.0322	TRUE
1	LAATKMSEC	1006	1014	9	HLA-A*02:01	1006	16	0.03201939	16	0.032	TRUE
1	GYQPYRVVV	490	498	9	HLA-A*02:01	490	16	0.03201046	16	0.032	TRUE
1	SVLNDILSR	957	965	9	HLA-A*02:01	957	16	0.03179891	16	0.0318	TRUE
1	FNGLTGTGV	529	537	9	HLA-A*02:01	529	16	0.03174122	16	0.0317	TRUE
1	RLITGRLQS	977	985	9	HLA-A*02:01	977	16	0.03141117	16	0.0314	TRUE
1	FRVVPSGDV	305	313	9	HLA-A*02:01	305	16	0.03103819	16	0.031	TRUE
1	GDSTECANL	726	734	9	HLA-A*02:01	726	16	0.03083152	16	0.0308	TRUE
1	YFPREGVFV	1070	1078	9	HLA-A*02:01	1070	16	0.03074659	16	0.0307	TRUE
1	RVVVLSFEL	495	503	9	HLA-A*02:01	495	17	0.03011642	17	0.0301	TRUE
1	SNVTGFHTI	64	72	9	HLA-A*02:01	64	17	0.02894373	17	0.0289	TRUE
1	AGWTFGAGA	866	874	9	HLA-A*02:01	866	17	0.02848988	17	0.0285	TRUE
1	LYLTQDLFL	52	60	9	HLA-A*02:01	52	17	0.02817001	17	0.0282	TRUE
1	FSPAQDIWG	238	246	9	HLA-A*02:01	238	17	0.0281237	17	0.0281	TRUE
1	IAAYTAALV	852	860	9	HLA-A*02:01	852	17	0.02808748	17	0.0281	TRUE

seq #	peptide	start	end	peptide length	allele	peptide index	median binding	mhcnp score	mhcnp percent	legacy score	scores equal
1	FIEDLLFNK	799	807	9	HLA-A*02:01	799	17	0.02800397	17	0.028	TRUE
1	TAPAICHEG	1059	1067	9	HLA-A*02:01	1059	17	0.02792467	17	0.0279	TRUE
1	ISSVLNDIL	955	963	9	HLA-A*02:01	955	17	0.02791205	17	0.0279	TRUE
1	YADSFVVKG	383	391	9	HLA-A*02:01	383	17	0.02790394	17	0.0279	TRUE
1	NASSEVAVL	589	597	9	HLA-A*02:01	589	17	0.02781321	17	0.0278	TRUE
1	FAMQMAYRF	880	888	9	HLA-A*02:01	880	18	0.02690272	18	0.0269	TRUE
1	VVRDLPSGF	205	213	9	HLA-A*02:01	205	18	0.02679673	18	0.0268	TRUE
1	LCDNPFFAV	132	140	9	HLA-A*02:01	132	18	0.02647	18	0.0265	TRUE
1	NAQALNTLV	937	945	9	HLA-A*02:01	937	18	0.02645217	18	0.0265	TRUE
1	ATKFPSVYA	331	339	9	HLA-A*02:01	331	18	0.02636774	18	0.0264	TRUE
1	QAAPHGVVF	1036	1044	9	HLA-A*02:01	1036	18	0.02591958	18	0.0259	TRUE
1	FFSPQIITT	1091	1099	9	HLA-A*02:01	1091	18	0.02567719	18	0.0257	TRUE
1	AQKFNGLTV	834	842	9	HLA-A*02:01	834	18	0.02553317	18	0.0255	TRUE
1	LICAQKFNG	831	839	9	HLA-A*02:01	831	18	0.02497461	18	0.025	TRUE
1	FCGKGYHLM	1024	1032	9	HLA-A*02:01	1024	18	0.0248573	18	0.0249	TRUE
1	KSFEIDKGI	291	299	9	HLA-A*02:01	291	19	0.02455531	19	0.0246	TRUE
1	IGAEHVDTS	637	645	9	HLA-A*02:01	637	19	0.02442338	19	0.0244	TRUE
1	SVITPGTNA	582	590	9	HLA-A*02:01	582	19	0.02422274	19	0.0242	TRUE
1	CPFGEVFNA	323	331	9	HLA-A*02:01	323	19	0.02415787	19	0.0242	TRUE
1	QNAQALNTL	936	944	9	HLA-A*02:01	936	19	0.0237965	19	0.0238	TRUE
1	GTNASSEVA	587	595	9	HLA-A*02:01	587	19	0.02368688	19	0.0237	TRUE
1	NTREVFAQV	759	767	9	HLA-A*02:01	759	19	0.02300744	19	0.023	TRUE
1	LKPIFKLPL	216	224	9	HLA-A*02:01	216	19	0.02294121	19	0.0229	TRUE
1	GNCDVVIGI	1106	1114	9	HLA-A*02:01	1106	19	0.02292518	19	0.0229	TRUE
1	QFNKAISQI	908	916	9	HLA-A*02:01	908	19	0.0229197	19	0.0229	TRUE
1	FCTQLNRAL	741	749	9	HLA-A*02:01	741	19	0.02285279	19	0.0229	TRUE
1	GICASYHTV	655	663	9	HLA-A*02:01	655	19	0.02277131	19	0.0228	TRUE
1	IFRSDTLYL	46	54	9	HLA-A*02:01	46	20	0.02223818	20	0.0222	TRUE
1	EILDISPCS	569	577	9	HLA-A*02:01	569	20	0.02210034	20	0.0221	TRUE
1	DLPSGFNTL	208	216	9	HLA-A*02:01	208	20	0.02152472	20	0.0215	TRUE
1	MIAAYTAAL	851	859	9	HLA-A*02:01	851	20	0.02118779	20	0.0212	TRUE
1	TLADAGFMK	809	817	9	HLA-A*02:01	809	20	0.02102401	20	0.021	TRUE
1	NGLTGTGVL	530	538	9	HLA-A*02:01	530	20	0.02080947	20	0.0208	TRUE
1	HLREFVFKN	181	189	9	HLA-A*02:01	181	20	0.02052212	20	0.0205	TRUE
1	AGAALQIPF	872	880	9	HLA-A*02:01	872	20	0.02043646	20	0.0204	TRUE
1	AVDCSQNPL	275	283	9	HLA-A*02:01	275	20	0.0202172	20	0.0202	TRUE
1	LGDINARDL	823	831	9	HLA-A*02:01	823	20	0.02005371	20	0.0201	TRUE
1	LIRAAEIRA	994	1002	9	HLA-A*02:01	994	20	0.01994601	20	0.0199	TRUE
1	DIWGTSAAA	243	251	9	HLA-A*02:01	243	21	0.01987477	21	0.0199	TRUE
1	ELDSFKEEL	1126	1134	9	HLA-A*02:01	1126	21	0.01985101	21	0.0199	TRUE
1	ASYHTVSLL	658	666	9	HLA-A*02:01	658	21	0.01979013	21	0.0198	TRUE
1	QKFNGLTVL	835	843	9	HLA-A*02:01	835	21	0.01972665	21	0.0197	TRUE
1	HTSPDVDLG	1141	1149	9	HLA-A*02:01	1141	21	0.01957176	21	0.0196	TRUE
1	KCSVKSFEI	287	295	9	HLA-A*02:01	287	21	0.01949463	21	0.0195	TRUE
1	VTQQLIRAA	990	998	9	HLA-A*02:01	990	21	0.01929848	21	0.0193	TRUE
1	ISGINASVV	1151	1159	9	HLA-A*02:01	1151	21	0.01915774	21	0.0192	TRUE
1	VITPGTNAS	583	591	9	HLA-A*02:01	583	21	0.01912957	21	0.0191	TRUE
1	ASVVNIQKE	1156	1164	9	HLA-A*02:01	1156	21	0.01911961	21	0.0191	TRUE
1	FLHVTYVPS	1044	1052	9	HLA-A*02:01	1044	21	0.01894548	21	0.0189	TRUE
1	INARDLICA	826	834	9	HLA-A*02:01	826	21	0.01876138	21	0.0188	TRUE
1	LNAPATVCG	504	512	9	HLA-A*02:01	504	21	0.01840377	21	0.0184	TRUE
1	SSIAYSNNT	685	693	9	HLA-A*02:01	685	22	0.01788019	22	0.0179	TRUE
1	DQLTPAWRI	613	621	9	HLA-A*02:01	613	22	0.01766551	22	0.0177	TRUE
1	STSQKSIVA	668	676	9	HLA-A*02:01	668	22	0.01762471	22	0.0176	TRUE
1	VTLADAGFM	808	816	9	HLA-A*02:01	808	22	0.01748368	22	0.0175	TRUE
1	VIRACNFEL	124	132	9	HLA-A*02:01	124	22	0.01744871	22	0.0174	TRUE
1	KLNDLCFSN	373	381	9	HLA-A*02:01	373	22	0.01738825	22	0.0174	TRUE
1	VKGDDVRQI	389	397	9	HLA-A*02:01	389	22	0.01722438	22	0.0172	TRUE
1	GGVSVITPG	579	587	9	HLA-A*02:01	579	22	0.01718567	22	0.0172	TRUE
1	GAISSVLND	953	961	9	HLA-A*02:01	953	22	0.01709276	22	0.0171	TRUE
1	VVNQNAQAL	933	941	9	HLA-A*02:01	933	22	0.01692469	22	0.0169	TRUE
1	SLGADSSIA	680	688	9	HLA-A*02:01	680	22	0.0168714	22	0.0169	TRUE
1	KMSECVLGQ	1010	1018	9	HLA-A*02:01	1010	22	0.0167525	22	0.0168	TRUE
1	ISPCSFGGV	573	581	9	HLA-A*02:01	573	23	0.01661168	23	0.0166	TRUE
1	VIGIINNTV	1111	1119	9	HLA-A*02:01	1111	23	0.01647296	23	0.0165	TRUE
1	QLSSNFGAI	947	955	9	HLA-A*02:01	947	23	0.01622457	23	0.0162	TRUE
1	QIDRLITGR	974	982	9	HLA-A*02:01	974	23	0.01610823	23	0.0161	TRUE
1	VFQTQAGCL	628	636	9	HLA-A*02:01	628	23	0.01604454	23	0.016	TRUE
1	ISDAFSLDV	164	172	9	HLA-A*02:01	164	23	0.01599775	23	0.016	TRUE
1	RDISNVPFS	453	461	9	HLA-A*02:01	453	23	0.01596777	23	0.016	TRUE
1	AAAYFVGYL	249	257	9	HLA-A*02:01	249	23	0.01596434	23	0.016	TRUE
1	RDPKTSEIL	563	571	9	HLA-A*02:01	563	23	0.01593452	23	0.0159	TRUE
1	SFGGVSVIT	577	585	9	HLA-A*02:01	577	23	0.01582254	23	0.0158	TRUE
1	TVYDPLQPE	1118	1126	9	HLA-A*02:01	1118	23	0.01569242	23	0.0157	TRUE
1	FELLNAPAT	501	509	9	HLA-A*02:01	501	23	0.01562295	23	0.0156	TRUE
1	VWLGFIAGL	1198	1206	9	HLA-A*02:01	1198	23	0.01560447	23	0.0156	TRUE
1	FKDGIYFAA	83	91	9	HLA-A*02:01	83	23	0.01557671	23	0.0156	TRUE
1	QPIDVVRDL	201	209	9	HLA-A*02:01	201	23	0.01544571	23	0.0154	TRUE
1	INNSTNVVI	117	125	9	HLA-A*02:01	117	24	0.01534057	24	0.0153	TRUE
1	PQAAPHGVV	1035	1043	9	HLA-A*02:01	1035	24	0.01531927	24	0.0153	TRUE

seq #	peptide	start	end	peptide length	allele	peptide index	median binding	mhcnp score	mhcnp percent	legacy score	scores equal
1	LLLQYGSFC	734	742	9	HLA-A*02:01	734	24	0.01496313	24	0.015	TRUE
1	GTSAAAYFV	246	254	9	HLA-A*02:01	246	24	0.01487287	24	0.0149	TRUE
1	ATAGWTFGA	864	872	9	HLA-A*02:01	864	24	0.01482541	24	0.0148	TRUE
1	AATKMSECV	1007	1015	9	HLA-A*02:01	1007	24	0.01473471	24	0.0147	TRUE
1	NCTDVSTAI	602	610	9	HLA-A*02:01	602	24	0.01455798	24	0.0146	TRUE
1	VDCSQNPLA	276	284	9	HLA-A*02:01	276	24	0.01439107	24	0.0144	TRUE
1	LNDLCFSNV	374	382	9	HLA-A*02:01	374	24	0.0143087	24	0.0143	TRUE
1	NITNFRAIL	227	235	9	HLA-A*02:01	227	25	0.01397946	25	0.014	TRUE
1	SIVAYTMSL	673	681	9	HLA-A*02:01	673	25	0.01388064	25	0.0139	TRUE
1	QMYKTPTLK	769	777	9	HLA-A*02:01	769	25	0.01381722	25	0.0138	TRUE
1	VLAWNTRNI	420	428	9	HLA-A*02:01	420	25	0.013729	25	0.0137	TRUE
1	ALQIPFAMQ	875	883	9	HLA-A*02:01	875	25	0.01370487	25	0.0137	TRUE
1	VLPPLLTDD	842	850	9	HLA-A*02:01	842	25	0.01355058	25	0.0136	TRUE
1	INHTFGNPV	72	80	9	HLA-A*02:01	72	25	0.01348599	25	0.0135	TRUE
1	TLVKQLSSN	943	951	9	HLA-A*02:01	943	25	0.01338311	25	0.0134	TRUE
1	KLPLGINIT	221	229	9	HLA-A*02:01	221	25	0.01330046	25	0.0133	TRUE
1	FITQRNFFS	1085	1093	9	HLA-A*02:01	1085	25	0.0131508	25	0.0132	TRUE
1	ATVCGPKLS	508	516	9	HLA-A*02:01	508	25	0.01282446	25	0.0128	TRUE
1	TAFSPAQDI	236	244	9	HLA-A*02:01	236	25	0.0128045	25	0.0128	TRUE
1	FVSGNCDVV	1103	1111	9	HLA-A*02:01	1103	25	0.01277531	25	0.0128	TRUE
1	QMAYRFNGI	883	891	9	HLA-A*02:01	883	26	0.01271456	26	0.0127	TRUE
1	SGFNTLKPI	211	219	9	HLA-A*02:01	211	26	0.01271147	26	0.0127	TRUE
1	FGGVSVITP	578	586	9	HLA-A*02:01	578	26	0.01259601	26	0.0126	TRUE
1	YQPIDVVRD	200	208	9	HLA-A*02:01	200	26	0.01254809	26	0.0125	TRUE
1	DFCGKGYHL	1023	1031	9	HLA-A*02:01	1023	26	0.01252217	26	0.0125	TRUE
1	SQILPDPLK	785	793	9	HLA-A*02:01	785	26	0.01225731	26	0.0123	TRUE
1	PVIPFKDGI	79	87	9	HLA-A*02:01	79	26	0.01219421	26	0.0122	TRUE
1	IDVVRDLPS	203	211	9	HLA-A*02:01	203	26	0.01215049	26	0.0122	TRUE
1	GKLQDVVNQ	928	936	9	HLA-A*02:01	928	26	0.01213285	26	0.0121	TRUE
1	IEDLLFNKV	800	808	9	HLA-A*02:01	800	26	0.01208866	26	0.0121	TRUE
1	TVCGPKLST	509	517	9	HLA-A*02:01	509	26	0.01200127	26	0.012	TRUE
1	ICHEGKAYF	1063	1071	9	HLA-A*02:01	1063	26	0.01197389	26	0.012	TRUE
1	VVIGIINNT	1110	1118	9	HLA-A*02:01	1110	26	0.01192658	26	0.0119	TRUE
1	SNVPFSPDG	456	464	9	HLA-A*02:01	456	26	0.01190066	26	0.0119	TRUE
1	SFCTQLNRA	740	748	9	HLA-A*02:01	740	27	0.01177413	27	0.0118	TRUE
1	KQLSSNFGA	946	954	9	HLA-A*02:01	946	27	0.01166619	27	0.0117	TRUE
1	STALGKLQD	924	932	9	HLA-A*02:01	924	27	0.01162204	27	0.0116	TRUE
1	IGYQPYRVV	489	497	9	HLA-A*02:01	489	27	0.01151306	27	0.0115	TRUE
1	LQSLQTYVT	983	991	9	HLA-A*02:01	983	27	0.01149182	27	0.0115	TRUE
1	FGNPVIPFK	76	84	9	HLA-A*02:01	76	27	0.01148888	27	0.0115	TRUE
1	FDDVQAPNY	22	30	9	HLA-A*02:01	22	27	0.01145466	27	0.0115	TRUE
1	RFPNITNLC	315	323	9	HLA-A*02:01	315	27	0.0113801	27	0.0114	TRUE
1	AQDIWGTSA	241	249	9	HLA-A*02:01	241	27	0.01123569	27	0.0112	TRUE
1	LIGAEHVDT	636	644	9	HLA-A*02:01	636	27	0.01100667	27	0.011	TRUE
1	VLSFELLNA	498	506	9	HLA-A*02:01	498	27	0.01090517	27	0.0109	TRUE
1	GADSSIAYS	682	690	9	HLA-A*02:01	682	28	0.01081139	28	0.0108	TRUE
1	TQLNRALSG	743	751	9	HLA-A*02:01	743	28	0.01059551	28	0.0106	TRUE
1	PATVCGPKL	507	515	9	HLA-A*02:01	507	28	0.01044298	28	0.0104	TRUE
1	VVVLSFELL	496	504	9	HLA-A*02:01	496	28	0.01041929	28	0.0104	TRUE
1	KGACSCGSC	1227	1235	9	HLA-A*02:01	1227	28	0.01035352	28	0.0104	TRUE
1	FTTAPAICH	1057	1065	9	HLA-A*02:01	1057	28	0.01032523	28	0.0103	TRUE
1	DVVRDLPSG	204	212	9	HLA-A*02:01	204	28	0.01029319	28	0.0103	TRUE
1	GTGVLTPSS	534	542	9	HLA-A*02:01	534	28	0.01020623	28	0.0102	TRUE
1	SCLKGACSC	1224	1232	9	HLA-A*02:01	1224	28	0.01015442	28	0.0102	TRUE
1	IDRLITGRL	975	983	9	HLA-A*02:01	975	28	0.01015266	28	0.0102	TRUE
1	KLSTDLIKN	514	522	9	HLA-A*02:01	514	28	0.0101159	28	0.0101	TRUE
1	AILTAFSPA	233	241	9	HLA-A*02:01	233	28	0.0101116	28	0.0101	TRUE
1	QRNFFSPQI	1088	1096	9	HLA-A*02:01	1088	28	0.01010091	28	0.0101	TRUE
1	GINITNFRA	225	233	9	HLA-A*02:01	225	29	0.00981933	29	0.0098	TRUE
1	LLRSTSQKS	665	673	9	HLA-A*02:01	665	29	0.00976841	29	0.0098	TRUE
1	FFAVSKPMG	137	145	9	HLA-A*02:01	137	29	0.0097319	29	0.0097	TRUE
1	KAYFPREGV	1068	1076	9	HLA-A*02:01	1068	29	0.00970626	29	0.0097	TRUE
1	SNFRVVPSG	303	311	9	HLA-A*02:01	303	29	0.00966384	29	0.0097	TRUE
1	GQSKRVDFC	1017	1025	9	HLA-A*02:01	1017	29	0.00950639	29	0.0095	TRUE
1	YKGYQPIDV	197	205	9	HLA-A*02:01	197	29	0.00949912	29	0.0095	TRUE
1	GDINARDLI	824	832	9	HLA-A*02:01	824	29	0.00940809	29	0.0094	TRUE
1	AIVMVTILL	1208	1216	9	HLA-A*02:01	1208	29	0.00924518	29	0.0092	TRUE
1	PCSFGGVSV	575	583	9	HLA-A*02:01	575	29	0.00923068	29	0.0092	TRUE
1	CLIGAEHVD	635	643	9	HLA-A*02:01	635	30	0.00917423	30	0.0092	TRUE
1	YHLMSFPQA	1029	1037	9	HLA-A*02:01	1029	30	0.00902178	30	0.009	TRUE
1	FLLFLTLTS	4	12	9	HLA-A*02:01	4	30	0.00896111	30	0.009	TRUE
1	QDIWGTSAA	242	250	9	HLA-A*02:01	242	30	0.00891994	30	0.0089	TRUE
1	GLIAIVMVT	1205	1213	9	HLA-A*02:01	1205	30	0.00890949	30	0.0089	TRUE
1	IHADQLTPA	610	618	9	HLA-A*02:01	610	30	0.00888038	30	0.0089	TRUE
1	GGFNFSQIL	780	788	9	HLA-A*02:01	780	30	0.00883929	30	0.0088	TRUE
1	SFVVKGDDV	386	394	9	HLA-A*02:01	386	30	0.00883671	30	0.0088	TRUE
1	IANQFNKAI	905	913	9	HLA-A*02:01	905	30	0.00880562	30	0.0088	TRUE
1	QALNTLVKQ	939	947	9	HLA-A*02:01	939	30	0.00879749	30	0.0088	TRUE
1	FSTFKCYGV	361	369	9	HLA-A*02:01	361	30	0.00877171	30	0.0088	TRUE
1	PLNDYGFYT	477	485	9	HLA-A*02:01	477	30	0.00875248	30	0.0088	TRUE

seq #	peptide	start	end	peptide length	allele	peptide index	median binding	mhcnp score	mhcnp percent	legacy score	scores equal
1	LTVLPPLLT	840	848	9	HLA-A*02:01	840	30	0.00872291	30	0.0087	TRUE
1	LIAIVMVTI	1206	1214	9	HLA-A*02:01	1206	30	0.0086815	30	0.0087	TRUE
1	QQFGRDVSD	549	557	9	HLA-A*02:01	549	30	0.00865262	30	0.0087	TRUE
1	NIDATSTGN	427	435	9	HLA-A*02:01	427	30	0.00865043	30	0.0087	TRUE
1	LVSGTATAG	859	867	9	HLA-A*02:01	859	30	0.00862314	30	0.0086	TRUE
1	SATKLNDLC	370	378	9	HLA-A*02:01	370	30	0.00860075	30	0.0086	TRUE
1	TDLIKNQCV	517	525	9	HLA-A*02:01	517	30	0.00860023	30	0.0086	TRUE
1	VLYQDVNCT	596	604	9	HLA-A*02:01	596	30	0.00856329	30	0.0086	TRUE
1	FKHLREFVF	179	187	9	HLA-A*02:01	179	31	0.0083979	31	0.0084	TRUE
1	FGGFNFSQI	779	787	9	HLA-A*02:01	779	31	0.00837235	31	0.0084	TRUE
1	RNIDATSTG	426	434	9	HLA-A*02:01	426	31	0.00835402	31	0.0084	TRUE
1	VLTPSSKRF	537	545	9	HLA-A*02:01	537	31	0.00834298	31	0.0083	TRUE
1	FIFLLFLTL	2	10	9	HLA-A*02:01	2	31	0.00833317	31	0.0083	TRUE
1	YFVGYLKPT	252	260	9	HLA-A*02:01	252	31	0.00832012	31	0.0083	TRUE
1	KPMGTQTHT	142	150	9	HLA-A*02:01	142	31	0.00812409	31	0.0081	TRUE
1	TGFHTINHT	67	75	9	HLA-A*02:01	67	31	0.00809189	31	0.0081	TRUE
1	TAALVSGTA	856	864	9	HLA-A*02:01	856	31	0.00805026	31	0.0081	TRUE
1	CVADYSVLY	348	356	9	HLA-A*02:01	348	31	0.00804591	31	0.008	TRUE
1	NKAISQIQE	910	918	9	HLA-A*02:01	910	31	0.00795087	31	0.008	TRUE
1	IAAEQDRNT	752	760	9	HLA-A*02:01	752	31	0.0079451	31	0.0079	TRUE
1	RGVYYPDEI	38	46	9	HLA-A*02:01	38	31	0.00794499	31	0.0079	TRUE
1	VSMAKTSVD	711	719	9	HLA-A*02:01	711	31	0.00794141	31	0.0079	TRUE
1	EVFAQVKQM	762	770	9	HLA-A*02:01	762	31	0.0079304	31	0.0079	TRUE
1	GTITDAVDC	270	278	9	HLA-A*02:01	270	31	0.00792372	31	0.0079	TRUE
1	FNGLTVLPP	837	845	9	HLA-A*02:01	837	32	0.00788599	32	0.0079	TRUE
1	FQTQAGCLI	629	637	9	HLA-A*02:01	629	32	0.00788103	32	0.0079	TRUE
1	FNKVTLADA	805	813	9	HLA-A*02:01	805	32	0.00785367	32	0.0079	TRUE
1	PLQPELDSF	1122	1130	9	HLA-A*02:01	1122	32	0.00776788	32	0.0078	TRUE
1	LLFLTLTSG	5	13	9	HLA-A*02:01	5	32	0.00775336	32	0.0078	TRUE
1	FLTLTSGSD	7	15	9	HLA-A*02:01	7	32	0.007708	32	0.0077	TRUE
1	LTAFSPAQD	235	243	9	HLA-A*02:01	235	32	0.00768856	32	0.0077	TRUE
1	CASYHTVSL	657	665	9	HLA-A*02:01	657	32	0.0075833	32	0.0076	TRUE
1	MQMAYRFNG	882	890	9	HLA-A*02:01	882	32	0.00755945	32	0.0076	TRUE
1	KGDDVRQIA	390	398	9	HLA-A*02:01	390	32	0.00752129	32	0.0075	TRUE
1	YTAALVSGT	855	863	9	HLA-A*02:01	855	32	0.00749244	32	0.0075	TRUE
1	MGTQTHTMI	144	152	9	HLA-A*02:01	144	32	0.00747123	32	0.0075	TRUE
1	QIPFAMQMA	877	885	9	HLA-A*02:01	877	32	0.00747099	32	0.0075	TRUE
1	ISQIQESLT	913	921	9	HLA-A*02:01	913	32	0.00747059	32	0.0075	TRUE
1	TLYLTQDLF	51	59	9	HLA-A*02:01	51	33	0.0074508	33	0.0075	TRUE
1	IPIGAGICA	650	658	9	HLA-A*02:01	650	33	0.00736303	33	0.0074	TRUE
1	VSLLRSTSQ	663	671	9	HLA-A*02:01	663	33	0.00725005	33	0.0073	TRUE
1	ITNLCPFGE	319	327	9	HLA-A*02:01	319	33	0.00710245	33	0.0071	TRUE
1	KFNGLTVLP	836	844	9	HLA-A*02:01	836	34	0.00690679	34	0.0069	TRUE
1	TNFRAILTA	229	237	9	HLA-A*02:01	229	34	0.00690191	34	0.0069	TRUE
1	VAKNLNESL	1171	1179	9	HLA-A*02:01	1171	34	0.00686623	34	0.0069	TRUE
1	LFLPFYSNV	58	66	9	HLA-A*02:01	58	34	0.0067611	34	0.0068	TRUE
1	RSFIEDLLF	797	805	9	HLA-A*02:01	797	34	0.00664436	34	0.0066	TRUE
1	TITDAVDCS	271	279	9	HLA-A*02:01	271	34	0.0065892	34	0.0066	TRUE
1	AEIRASANL	998	1006	9	HLA-A*02:01	998	34	0.00657345	34	0.0066	TRUE
1	LADAGFMKQ	810	818	9	HLA-A*02:01	810	34	0.00651781	34	0.0065	TRUE
1	LIDLQELGK	1179	1187	9	HLA-A*02:01	1179	34	0.00650562	34	0.0065	TRUE
1	AALVSGTAT	857	865	9	HLA-A*02:01	857	34	0.00649626	34	0.0065	TRUE
1	YSNVTGFHT	63	71	9	HLA-A*02:01	63	34	0.00641215	34	0.0064	TRUE
1	FVVKGDDVR	387	395	9	HLA-A*02:01	387	35	0.00630775	35	0.0063	TRUE
1	IVMVTILLC	1209	1217	9	HLA-A*02:01	1209	35	0.00628627	35	0.0063	TRUE
1	TGIGYQPYR	487	495	9	HLA-A*02:01	487	35	0.00625896	35	0.0063	TRUE
1	FNFNGLTGT	527	535	9	HLA-A*02:01	527	35	0.00624886	35	0.0062	TRUE
1	YVYKGYQPI	195	203	9	HLA-A*02:01	195	35	0.00621912	35	0.0062	TRUE
1	VNCTDVSTA	601	609	9	HLA-A*02:01	601	35	0.00616646	35	0.0062	TRUE
1	PIGAGICAS	651	659	9	HLA-A*02:01	651	35	0.00616509	35	0.0062	TRUE
1	RNFFSPQII	1089	1097	9	HLA-A*02:01	1089	35	0.00610026	35	0.0061	TRUE
1	RSTSQKSIV	667	675	9	HLA-A*02:01	667	35	0.00608648	35	0.0061	TRUE
1	WLGFIAGLI	1199	1207	9	HLA-A*02:01	1199	35	0.0060791	35	0.0061	TRUE
1	QAPNYTQHT	26	34	9	HLA-A*02:01	26	36	0.00588469	36	0.0059	TRUE
1	YYPDEIFRS	41	49	9	HLA-A*02:01	41	36	0.00586357	36	0.0059	TRUE
1	DSEPVLKGV	1242	1250	9	HLA-A*02:01	1242	36	0.00578695	36	0.0058	TRUE
1	TSGSDLDRC	11	19	9	HLA-A*02:01	11	36	0.00576403	36	0.0058	TRUE
1	DKVEAEVQI	967	975	9	HLA-A*02:01	967	36	0.0057144	36	0.0057	TRUE
1	NVYADSFVV	381	389	9	HLA-A*02:01	381	36	0.00567733	36	0.0057	TRUE
1	FNGIGVTQN	888	896	9	HLA-A*02:01	888	36	0.0056413	36	0.0056	TRUE
1	DLGDISGIN	1147	1155	9	HLA-A*02:01	1147	36	0.00563928	36	0.0056	TRUE
1	NSTNVVIRA	119	127	9	HLA-A*02:01	119	36	0.00561856	36	0.0056	TRUE
1	MSLGADSSI	679	687	9	HLA-A*02:01	679	36	0.00558695	36	0.0056	TRUE
1	VLKGVKLHY	1246	1254	9	HLA-A*02:01	1246	36	0.00558317	36	0.0056	TRUE
1	ITNFRAILT	228	236	9	HLA-A*02:01	228	37	0.0055312	37	0.0055	TRUE
1	EIDRLNEVA	1164	1172	9	HLA-A*02:01	1164	37	0.00550787	37	0.0055	TRUE
1	TLKPIFKLP	215	223	9	HLA-A*02:01	215	37	0.00549087	37	0.0055	TRUE
1	IRAAEIRAS	995	1003	9	HLA-A*02:01	995	37	0.00533312	37	0.0053	TRUE
1	NFGAISSVL	951	959	9	HLA-A*02:01	951	37	0.00526775	37	0.0053	TRUE
1	NQNAQALNT	935	943	9	HLA-A*02:01	935	38	0.00523108	38	0.0052	TRUE

seq #	peptide	start	end	peptide length	allele	peptide index	median binding	mhcnp score	mhcnp percent	legacy score	scores equal
1	TDSVRDPKT	559	567	9	HLA-A*02:01	559	38	0.00521063	38	0.0052	TRUE
1	NAPATVCGP	505	513	9	HLA-A*02:01	505	38	0.00517233	38	0.0052	TRUE
1	DINARDLIC	825	833	9	HLA-A*02:01	825	38	0.00517118	38	0.0052	TRUE
1	GVSATKLND	368	376	9	HLA-A*02:01	368	38	0.00514523	38	0.0051	TRUE
1	SNCVADYSV	346	354	9	HLA-A*02:01	346	38	0.00512879	38	0.0051	TRUE
1	GECLGDINA	820	828	9	HLA-A*02:01	820	38	0.00509687	38	0.0051	TRUE
1	YQTSNFRVV	300	308	9	HLA-A*02:01	300	38	0.00509146	38	0.0051	TRUE
1	YQDVNCTDV	598	606	9	HLA-A*02:01	598	38	0.00491401	38	0.0049	TRUE
1	NRALSGIAA	746	754	9	HLA-A*02:01	746	38	0.0048998	38	0.0049	TRUE
1	GVTQNVLYE	892	900	9	HLA-A*02:01	892	38	0.00489232	38	0.0049	TRUE
1	NLLLQYGSF	733	741	9	HLA-A*02:01	733	39	0.00478516	39	0.0048	TRUE
1	LKYDENGTI	264	272	9	HLA-A*02:01	264	39	0.00478077	39	0.0048	TRUE
1	VADYSVLYN	349	357	9	HLA-A*02:01	349	39	0.00477431	39	0.0048	TRUE
1	AIHADQLTP	609	617	9	HLA-A*02:01	609	39	0.00477132	39	0.0048	TRUE
1	PCTPPALNC	466	474	9	HLA-A*02:01	466	39	0.00474026	39	0.0047	TRUE
1	SFELLNAPA	500	508	9	HLA-A*02:01	500	39	0.00473721	39	0.0047	TRUE
1	GIAAEQDRN	751	759	9	HLA-A*02:01	751	39	0.00473217	39	0.0047	TRUE
1	SVRDPKTSE	561	569	9	HLA-A*02:01	561	39	0.00471888	39	0.0047	TRUE
1	QNPLAELKC	280	288	9	HLA-A*02:01	280	39	0.00471231	39	0.0047	TRUE
1	IKWPWYVWL	1192	1200	9	HLA-A*02:01	1192	39	0.0046977	39	0.0047	TRUE
1	YVWLGFIAG	1197	1205	9	HLA-A*02:01	1197	39	0.0046895	39	0.0047	TRUE
1	LTTTSTALG	920	928	9	HLA-A*02:01	920	39	0.00468272	39	0.0047	TRUE
1	TIAIPTNFS	693	701	9	HLA-A*02:01	693	39	0.00467624	39	0.0047	TRUE
1	YGFYTTTGI	481	489	9	HLA-A*02:01	481	39	0.00466102	39	0.0047	TRUE
1	TKFPSVYAW	332	340	9	HLA-A*02:01	332	39	0.00459948	39	0.0046	TRUE
1	ITPGTNASS	584	592	9	HLA-A*02:01	584	39	0.00455426	39	0.0046	TRUE
1	VRQIAPGQT	394	402	9	HLA-A*02:01	394	39	0.00455276	39	0.0046	TRUE
1	LTLTSGSDL	8	16	9	HLA-A*02:01	8	40	0.00449136	40	0.0045	TRUE
1	HVTYVPSQE	1046	1054	9	HLA-A*02:01	1046	40	0.00440656	40	0.0044	TRUE
1	QTYVTQQLI	987	995	9	HLA-A*02:01	987	40	0.00440481	40	0.0044	TRUE
1	IQESLTTTS	916	924	9	HLA-A*02:01	916	40	0.00438256	40	0.0044	TRUE
1	TAIHADQLT	608	616	9	HLA-A*02:01	608	40	0.00437478	40	0.0044	TRUE
1	ELCDNPFFA	131	139	9	HLA-A*02:01	131	40	0.00435446	40	0.0044	TRUE
1	PAICHEGKA	1061	1069	9	HLA-A*02:01	1061	40	0.00433707	40	0.0043	TRUE
1	AQVKQMYKT	765	773	9	HLA-A*02:01	765	40	0.0043004	40	0.0043	TRUE
1	IAIVMVTIL	1207	1215	9	HLA-A*02:01	1207	40	0.00427986	40	0.0043	TRUE
1	VGYLKPTTF	254	262	9	HLA-A*02:01	254	40	0.00427878	40	0.0043	TRUE
1	SFIEDLLFN	798	806	9	HLA-A*02:01	798	40	0.00420715	40	0.0042	TRUE
1	ISITTEVMP	702	710	9	HLA-A*02:01	702	41	0.00418916	41	0.0042	TRUE
1	IAGLIAIVM	1203	1211	9	HLA-A*02:01	1203	41	0.0041874	41	0.0042	TRUE
1	YISDAFSLD	163	171	9	HLA-A*02:01	163	41	0.0041684	41	0.0042	TRUE
1	NTIAIPTNF	692	700	9	HLA-A*02:01	692	41	0.00414021	41	0.0041	TRUE
1	TFDDVQAPN	21	29	9	HLA-A*02:01	21	41	0.00413693	41	0.0041	TRUE
1	FEIDKGIYQ	293	301	9	HLA-A*02:01	293	41	0.00411104	41	0.0041	TRUE
1	FGAISSVLN	952	960	9	HLA-A*02:01	952	41	0.0041085	41	0.0041	TRUE
1	TGTGVLTPS	533	541	9	HLA-A*02:01	533	41	0.00407115	41	0.0041	TRUE
1	NNVFQTQAG	626	634	9	HLA-A*02:01	626	41	0.00405755	41	0.0041	TRUE
1	VFLHVTYVP	1043	1051	9	HLA-A*02:01	1043	41	0.00402224	41	0.004	TRUE
1	ELGKYEQYI	1184	1192	9	HLA-A*02:01	1184	42	0.00389076	42	0.0039	TRUE
1	NPLAELKCS	281	289	9	HLA-A*02:01	281	42	0.00388046	42	0.0039	TRUE
1	ISNVPFSPD	455	463	9	HLA-A*02:01	455	42	0.00384997	42	0.0038	TRUE
1	SDLDRCTTF	14	22	9	HLA-A*02:01	14	42	0.00380245	42	0.0038	TRUE
1	APGQTGVIA	398	406	9	HLA-A*02:01	398	42	0.00380105	42	0.0038	TRUE
1	GNNVFQTQA	625	633	9	HLA-A*02:01	625	42	0.00379712	42	0.0038	TRUE
1	YLRHGKLRP	442	450	9	HLA-A*02:01	442	42	0.00374785	42	0.0037	TRUE
1	STDLIKNQC	516	524	9	HLA-A*02:01	516	42	0.00371683	42	0.0037	TRUE
1	VVPSGDVVR	307	315	9	HLA-A*02:01	307	42	0.00370095	42	0.0037	TRUE
1	SCCSCLKGA	1221	1229	9	HLA-A*02:01	1221	42	0.0036692	42	0.0037	TRUE
1	FRSDTLYLT	47	55	9	HLA-A*02:01	47	42	0.00366902	42	0.0037	TRUE
1	LTPAWRIYS	615	623	9	HLA-A*02:01	615	42	0.00366723	42	0.0037	TRUE
1	KFDEDDSEP	1237	1245	9	HLA-A*02:01	1237	43	0.00364081	43	0.0036	TRUE
1	TTFDDVQAP	20	28	9	HLA-A*02:01	20	43	0.00362043	43	0.0036	TRUE
1	SSVLNDILS	956	964	9	HLA-A*02:01	956	43	0.00359769	43	0.0036	TRUE
1	SEILDISPC	568	576	9	HLA-A*02:01	568	43	0.00357024	43	0.0036	TRUE
1	FMKQYGECL	815	823	9	HLA-A*02:01	815	43	0.00354907	43	0.0035	TRUE
1	FVFKNKDGF	185	193	9	HLA-A*02:01	185	43	0.00352969	43	0.0035	TRUE
1	IAYSNNTIA	687	695	9	HLA-A*02:01	687	43	0.00349582	43	0.0035	TRUE
1	HADQLTPAW	611	619	9	HLA-A*02:01	611	43	0.00347613	43	0.0035	TRUE
1	LTQDLFLPF	54	62	9	HLA-A*02:01	54	43	0.00344968	43	0.0034	TRUE
1	NTLVKQLSS	942	950	9	HLA-A*02:01	942	43	0.00344644	43	0.0034	TRUE
1	FLPFYSNVT	59	67	9	HLA-A*02:01	59	43	0.0034415	43	0.0034	TRUE
1	YDPLQPELD	1120	1128	9	HLA-A*02:01	1120	43	0.00343325	43	0.0034	TRUE
1	STECANLLL	728	736	9	HLA-A*02:01	728	43	0.00343106	43	0.0034	TRUE
1	NFTTAPAIC	1056	1064	9	HLA-A*02:01	1056	43	0.00340619	43	0.0034	TRUE
1	ITTEVMPVS	704	712	9	HLA-A*02:01	704	43	0.00339802	43	0.0034	TRUE
1	FLYVYKGYQ	193	201	9	HLA-A*02:01	193	44	0.00337717	44	0.0034	TRUE
1	VMVTILLCC	1210	1218	9	HLA-A*02:01	1210	44	0.00335707	44	0.0034	TRUE
1	FGRDVSDFT	551	559	9	HLA-A*02:01	551	44	0.00335574	44	0.0034	TRUE
1	DGKPCTPPA	463	471	9	HLA-A*02:01	463	44	0.00335258	44	0.0034	TRUE
1	VMPVSMAKT	708	716	9	HLA-A*02:01	708	44	0.00332991	44	0.0033	TRUE

seq #	peptide	start	end	peptide length	allele	peptide index	median binding	mhcnp score	mhcnp percent	legacy score	scores equal
1	HTVSLLRST	661	669	9	HLA-A*02:01	661	44	0.00332565	44	0.0033	TRUE
1	LPDDFMGCV	412	420	9	HLA-A*02:01	412	44	0.00331847	44	0.0033	TRUE
1	VFNATKFPS	328	336	9	HLA-A*02:01	328	44	0.00331403	44	0.0033	TRUE
1	AGICASYHT	654	662	9	HLA-A*02:01	654	44	0.00330829	44	0.0033	TRUE
1	TMIFDNAFN	150	158	9	HLA-A*02:01	150	44	0.0033064	44	0.0033	TRUE
1	QAGCLIGAE	632	640	9	HLA-A*02:01	632	44	0.00325578	44	0.0033	TRUE
1	STGNNVFQT	623	631	9	HLA-A*02:01	623	44	0.00321788	44	0.0032	TRUE
1	QSLQTYVTQ	984	992	9	HLA-A*02:01	984	44	0.00317964	44	0.0032	TRUE
1	IITTDNTFV	1096	1104	9	HLA-A*02:01	1096	44	0.00317258	44	0.0032	TRUE
1	ILLCCMTSC	1214	1222	9	HLA-A*02:01	1214	44	0.00314588	44	0.0031	TRUE
1	QLIRAAEIR	993	1001	9	HLA-A*02:01	993	44	0.00314513	44	0.0031	TRUE
1	ANLLLQYGS	732	740	9	HLA-A*02:01	732	44	0.00312891	44	0.0031	TRUE
1	RASANLAAT	1001	1009	9	HLA-A*02:01	1001	45	0.00312373	45	0.0031	TRUE
1	ASSEVAVLY	590	598	9	HLA-A*02:01	590	45	0.00311722	45	0.0031	TRUE
1	SANLAATKM	1003	1011	9	HLA-A*02:01	1003	45	0.00311586	45	0.0031	TRUE
1	STNVVIRAC	120	128	9	HLA-A*02:01	120	45	0.00309112	45	0.0031	TRUE
1	TSYECDIPI	644	652	9	HLA-A*02:01	644	45	0.0030464	45	0.003	TRUE
1	SSNFGAISS	949	957	9	HLA-A*02:01	949	45	0.00303834	45	0.003	TRUE
1	DVDLGDISG	1145	1153	9	HLA-A*02:01	1145	45	0.00301977	45	0.003	TRUE
1	PGTNASSEV	586	594	9	HLA-A*02:01	586	45	0.00300826	45	0.003	TRUE
1	TSNFRVVPS	302	310	9	HLA-A*02:01	302	45	0.00297721	45	0.003	TRUE
1	NKSQSVIII	109	117	9	HLA-A*02:01	109	45	0.00297165	45	0.003	TRUE
1	LPDPLKPTK	788	796	9	HLA-A*02:01	788	45	0.00294875	45	0.0029	TRUE
1	LSGIAAEQD	749	757	9	HLA-A*02:01	749	46	0.00283884	46	0.0028	TRUE
1	DVVNQNAQA	932	940	9	HLA-A*02:01	932	46	0.00282726	46	0.0028	TRUE
1	SVIIINNST	113	121	9	HLA-A*02:01	113	46	0.00282368	46	0.0028	TRUE
1	VTYVPSQER	1047	1055	9	HLA-A*02:01	1047	46	0.00281696	46	0.0028	TRUE
1	QYIKWPWYV	1190	1198	9	HLA-A*02:01	1190	46	0.00281449	46	0.0028	TRUE
1	FDNAFNCTF	153	161	9	HLA-A*02:01	153	46	0.00281441	46	0.0028	TRUE
1	PFERDISNV	450	458	9	HLA-A*02:01	450	46	0.00278036	46	0.0028	TRUE
1	VSTAIHADQ	606	614	9	HLA-A*02:01	606	46	0.00277875	46	0.0028	TRUE
1	FNTLKPIFK	213	221	9	HLA-A*02:01	213	46	0.00276148	46	0.0028	TRUE
1	INASVVNIQ	1154	1162	9	HLA-A*02:01	1154	46	0.00274161	46	0.0027	TRUE
1	GVYYPDEIF	39	47	9	HLA-A*02:01	39	46	0.00272388	46	0.0027	TRUE
1	YRYLRHGKL	440	448	9	HLA-A*02:01	440	47	0.00271683	47	0.0027	TRUE
1	DLCFSNVYA	376	384	9	HLA-A*02:01	376	47	0.00271484	47	0.0027	TRUE
1	NASVVNIQK	1155	1163	9	HLA-A*02:01	1155	47	0.00268399	47	0.0027	TRUE
1	NFKHLREFV	178	186	9	HLA-A*02:01	178	47	0.00267926	47	0.0027	TRUE
1	VVNIQKEID	1158	1166	9	HLA-A*02:01	1158	47	0.00267431	47	0.0027	TRUE
1	QYGSFCTQL	737	745	9	HLA-A*02:01	737	47	0.00266608	47	0.0027	TRUE
1	LNDYGFYTT	478	486	9	HLA-A*02:01	478	47	0.00265684	47	0.0027	TRUE
1	SSMRGVYYP	35	43	9	HLA-A*02:01	35	47	0.00264284	47	0.0026	TRUE
1	KQIANQFNK	903	911	9	HLA-A*02:01	903	47	0.00262796	47	0.0026	TRUE
1	NQFNKAISQ	907	915	9	HLA-A*02:01	907	47	0.0026015	47	0.0026	TRUE
1	GAGAALQIP	871	879	9	HLA-A*02:01	871	47	0.00259749	47	0.0026	TRUE
1	VCGPKLSTD	510	518	9	HLA-A*02:01	510	47	0.00259727	47	0.0026	TRUE
1	TSPDVDLGD	1142	1150	9	HLA-A*02:01	1142	47	0.00255045	47	0.0026	TRUE
1	LMSFPQAAP	1031	1039	9	HLA-A*02:01	1031	47	0.00254798	47	0.0025	TRUE
1	VAYTMSLGA	675	683	9	HLA-A*02:01	675	47	0.00254336	47	0.0025	TRUE
1	LKPTKRSFI	792	800	9	HLA-A*02:01	792	47	0.00254327	47	0.0025	TRUE
1	GTQTHTMIF	145	153	9	HLA-A*02:01	145	47	0.00253576	47	0.0025	TRUE
1	IYQTSNFRV	299	307	9	HLA-A*02:01	299	47	0.00253427	47	0.0025	TRUE
1	SQERNFTTA	1052	1060	9	HLA-A*02:01	1052	47	0.0025341	47	0.0025	TRUE
1	TQQLIRAAE	991	999	9	HLA-A*02:01	991	47	0.00251535	47	0.0025	TRUE
1	NITNLCPFG	318	326	9	HLA-A*02:01	318	47	0.00250691	47	0.0025	TRUE
1	VKSFEIDKG	290	298	9	HLA-A*02:01	290	47	0.00250237	47	0.0025	TRUE
1	FTDSVRDPK	558	566	9	HLA-A*02:01	558	48	0.00247882	48	0.0025	TRUE
1	ECLGDINAR	821	829	9	HLA-A*02:01	821	48	0.00243967	48	0.0024	TRUE
1	VFKNKDGFL	186	194	9	HLA-A*02:01	186	48	0.00242341	48	0.0024	TRUE
1	LKGVKLHYT	1247	1255	9	HLA-A*02:01	1247	48	0.00241348	48	0.0024	TRUE
1	SQNPLAELK	279	287	9	HLA-A*02:01	279	48	0.00241216	48	0.0024	TRUE
1	LLQYGSFCT	735	743	9	HLA-A*02:01	735	48	0.00240322	48	0.0024	TRUE
1	VSGNCDVVI	1104	1112	9	HLA-A*02:01	1104	48	0.00238741	48	0.0024	TRUE
1	QSVIIINNS	112	120	9	HLA-A*02:01	112	48	0.00238331	48	0.0024	TRUE
1	EIDKGIYQT	294	302	9	HLA-A*02:01	294	48	0.00235594	48	0.0024	TRUE
1	NFNGLTGTG	528	536	9	HLA-A*02:01	528	48	0.00235373	48	0.0024	TRUE
1	KVTLADAGF	807	815	9	HLA-A*02:01	807	48	0.00235362	48	0.0024	TRUE
1	NHTSPDVDL	1140	1148	9	HLA-A*02:01	1140	48	0.00235206	48	0.0024	TRUE
1	ANLAATKMS	1004	1012	9	HLA-A*02:01	1004	48	0.00235042	48	0.0024	TRUE
1	LNRALSGIA	745	753	9	HLA-A*02:01	745	48	0.00234725	48	0.0023	TRUE
1	ADQLTPAWR	612	620	9	HLA-A*02:01	612	48	0.00232581	48	0.0023	TRUE
1	TLTSGSDLD	9	17	9	HLA-A*02:01	9	49	0.0023168	49	0.0023	TRUE
1	TFGNPVIPF	75	83	9	HLA-A*02:01	75	49	0.0023106	49	0.0023	TRUE
1	NEVAKNLNE	1169	1177	9	HLA-A*02:01	1169	49	0.00230898	49	0.0023	TRUE
1	SPDVDLGDI	1143	1151	9	HLA-A*02:01	1143	49	0.00228589	49	0.0023	TRUE
1	GVLTPSSKR	536	544	9	HLA-A*02:01	536	49	0.00225867	49	0.0023	TRUE
1	FQQFGRDVS	548	556	9	HLA-A*02:01	548	49	0.00225343	49	0.0023	TRUE
1	TKMSECVLG	1009	1017	9	HLA-A*02:01	1009	49	0.00221504	49	0.0022	TRUE
1	GIYQTSNFR	298	306	9	HLA-A*02:01	298	49	0.00220197	49	0.0022	TRUE
1	PFKDGIYFA	82	90	9	HLA-A*02:01	82	49	0.00219181	49	0.0022	TRUE

seq #	peptide	start	end	peptide length	allele	peptide index	median binding	mhcnp score	mhcnp percent	legacy score	scores equal
1	DVSTAIHAD	605	613	9	HLA-A*02:01	605	49	0.00218974	49	0.0022	TRUE
1	KSNVVRGWV	94	102	9	HLA-A*02:01	94	49	0.00217569	49	0.0022	TRUE
1	KTSEILDIS	566	574	9	HLA-A*02:01	566	50	0.00216906	50	0.0022	TRUE
1	GIYFAATEK	86	94	9	HLA-A*02:01	86	50	0.00216658	50	0.0022	TRUE
1	KRVDFCGKG	1020	1028	9	HLA-A*02:01	1020	50	0.00215578	50	0.0022	TRUE
1	IRACNFELC	125	133	9	HLA-A*02:01	125	50	0.00215524	50	0.0022	TRUE
1	PVSMAKTSV	710	718	9	HLA-A*02:01	710	50	0.0021398	50	0.0021	TRUE
1	AICHEGKAY	1062	1070	9	HLA-A*02:01	1062	50	0.00213682	50	0.0021	TRUE
1	LTGTGVLTP	532	540	9	HLA-A*02:01	532	50	0.00212759	50	0.0021	TRUE
1	VLYNSTFFS	354	362	9	HLA-A*02:01	354	50	0.00212383	50	0.0021	TRUE
1	IFDNAFNCT	152	160	9	HLA-A*02:01	152	50	0.00212205	50	0.0021	TRUE
1	VLGQSKRVD	1015	1023	9	HLA-A*02:01	1015	50	0.00212151	50	0.0021	TRUE
1	VSVITPGTN	581	589	9	HLA-A*02:01	581	50	0.00211877	50	0.0021	TRUE
1	IGVTQNVLY	891	899	9	HLA-A*02:01	891	50	0.00211671	50	0.0021	TRUE
1	VPSGDVVRF	308	316	9	HLA-A*02:01	308	50	0.0021144	50	0.0021	TRUE
1	GFMKQYGEC	814	822	9	HLA-A*02:01	814	50	0.00211413	50	0.0021	TRUE
1	CTTFDDVQA	19	27	9	HLA-A*02:01	19	50	0.0020831	50	0.0021	TRUE
1	QLTPAWRIY	614	622	9	HLA-A*02:01	614	50	0.00208267	50	0.0021	TRUE
1	DISPCSFGG	572	580	9	HLA-A*02:01	572	50	0.00206966	50	0.0021	TRUE
1	SPAQDIWGT	239	247	9	HLA-A*02:01	239	50	0.00206656	50	0.0021	TRUE
1	WTFGAGAAL	868	876	9	HLA-A*02:01	868	50	0.00206396	50	0.0021	TRUE
1	DMIAAYTAA	850	858	9	HLA-A*02:01	850	50	0.00205749	50	0.0021	TRUE
1	YSTGNNVFQ	622	630	9	HLA-A*02:01	622	50	0.00205541	50	0.0021	TRUE
1	LKYFGGFNF	776	784	9	HLA-A*02:01	776	50	0.00204146	50	0.002	TRUE
1	FPREGVFVF	1071	1079	9	HLA-A*02:01	1071	50	0.00203763	50	0.002	TRUE
1	SQSVIIINN	111	119	9	HLA-A*02:01	111	50	0.00203512	50	0.002	TRUE
1	ADYSVLYNS	350	358	9	HLA-A*02:01	350	51	0.00202202	51	0.002	TRUE
1	FNFSQILPD	782	790	9	HLA-A*02:01	782	51	0.00202081	51	0.002	TRUE
1	RNTREVFAQ	758	766	9	HLA-A*02:01	758	51	0.00201941	51	0.002	TRUE
1	GTATAGWTF	862	870	9	HLA-A*02:01	862	51	0.00199814	51	0.002	TRUE
1	SQKSIVAYT	670	678	9	HLA-A*02:01	670	51	0.00198952	51	0.002	TRUE
1	DISNVPFSP	454	462	9	HLA-A*02:01	454	51	0.00197085	51	0.002	TRUE
1	DDSEPVLKG	1241	1249	9	HLA-A*02:01	1241	51	0.0019706	51	0.002	TRUE
1	AYRFNGIGV	885	893	9	HLA-A*02:01	885	51	0.0019646	51	0.002	TRUE
1	INITNFRAI	226	234	9	HLA-A*02:01	226	51	0.00196328	51	0.002	TRUE
1	NHTFGNPVI	73	81	9	HLA-A*02:01	73	51	0.00192108	51	0.0019	TRUE
1	TGRLQSLQT	980	988	9	HLA-A*02:01	980	51	0.00191471	51	0.0019	TRUE
1	LGFIAGLIA	1200	1208	9	HLA-A*02:01	1200	51	0.00190389	51	0.0019	TRUE
1	GFNTLKPIF	212	220	9	HLA-A*02:01	212	52	0.00188613	52	0.0019	TRUE
1	AQALNTLVK	938	946	9	HLA-A*02:01	938	52	0.00188357	52	0.0019	TRUE
1	YVPSQERNF	1049	1057	9	HLA-A*02:01	1049	52	0.00187365	52	0.0019	TRUE
1	AKTSVDCNM	714	722	9	HLA-A*02:01	714	52	0.00186815	52	0.0019	TRUE
1	AGFMKQYGE	813	821	9	HLA-A*02:01	813	52	0.00186425	52	0.0019	TRUE
1	DVSEKSGNF	171	179	9	HLA-A*02:01	171	52	0.00185003	52	0.0019	TRUE
1	VFNGTSWFI	1078	1086	9	HLA-A*02:01	1078	52	0.00184709	52	0.0018	TRUE
1	SFPQAAPHG	1033	1041	9	HLA-A*02:01	1033	52	0.00184172	52	0.0018	TRUE
1	YFGGFNFSQ	778	786	9	HLA-A*02:01	778	52	0.00183636	52	0.0018	TRUE
1	LNDILSRLD	959	967	9	HLA-A*02:01	959	52	0.00182911	52	0.0018	TRUE
1	STFFSTFKC	358	366	9	HLA-A*02:01	358	52	0.00181893	52	0.0018	TRUE
1	AVSKPMGTQ	139	147	9	HLA-A*02:01	139	52	0.00181387	52	0.0018	TRUE
1	AAYTAALVS	853	861	9	HLA-A*02:01	853	52	0.00179644	52	0.0018	TRUE
1	FELCDNPFF	130	138	9	HLA-A*02:01	130	52	0.00179629	52	0.0018	TRUE
1	KVEAEVQID	968	976	9	HLA-A*02:01	968	52	0.00177678	52	0.0018	TRUE
1	RFNGIGVTQ	887	895	9	HLA-A*02:01	887	52	0.0017747	52	0.0018	TRUE
1	AFSLDVSEK	167	175	9	HLA-A*02:01	167	52	0.00177148	52	0.0018	TRUE
1	YKTPTLKYF	771	779	9	HLA-A*02:01	771	52	0.00176276	52	0.0018	TRUE
1	TVSLLRSTS	662	670	9	HLA-A*02:01	662	52	0.00174725	52	0.0017	TRUE
1	GSDLDRCTT	13	21	9	HLA-A*02:01	13	53	0.00172176	53	0.0017	TRUE
1	IPTNFSISI	696	704	9	HLA-A*02:01	696	53	0.0017191	53	0.0017	TRUE
1	KQYGECLGD	817	825	9	HLA-A*02:01	817	53	0.00168792	53	0.0017	TRUE
1	FAQVKQMYK	764	772	9	HLA-A*02:01	764	53	0.0016861	53	0.0017	TRUE
1	RPFERDISN	449	457	9	HLA-A*02:01	449	53	0.00167662	53	0.0017	TRUE
1	DEDDSEPVL	1239	1247	9	HLA-A*02:01	1239	53	0.00166706	53	0.0017	TRUE
1	HTFGNPVIP	74	82	9	HLA-A*02:01	74	53	0.00166096	53	0.0017	TRUE
1	ENGTITDAV	268	276	9	HLA-A*02:01	268	53	0.00164642	53	0.0016	TRUE
1	TSVDCNMYI	716	724	9	HLA-A*02:01	716	54	0.00162784	54	0.0016	TRUE
1	ERNFTTAPA	1054	1062	9	HLA-A*02:01	1054	54	0.00161634	54	0.0016	TRUE
1	FGSTMNNKS	103	111	9	HLA-A*02:01	103	54	0.001608	54	0.0016	TRUE
1	EPVLKGVKL	1244	1252	9	HLA-A*02:01	1244	54	0.0015917	54	0.0016	TRUE
1	DLQELGKYE	1181	1189	9	HLA-A*02:01	1181	54	0.00158428	54	0.0016	TRUE
1	FKNKDGFLY	187	195	9	HLA-A*02:01	187	54	0.00157906	54	0.0016	TRUE
1	IPFKDGIYF	81	89	9	HLA-A*02:01	81	54	0.00156862	54	0.0016	TRUE
1	QHTSSMRGV	32	40	9	HLA-A*02:01	32	54	0.00156131	54	0.0016	TRUE
1	YAWERKKIS	338	346	9	HLA-A*02:01	338	54	0.00154379	54	0.0015	TRUE
1	ECDIPIGAG	647	655	9	HLA-A*02:01	647	54	0.00154201	54	0.0015	TRUE
1	PLLTDDMIA	845	853	9	HLA-A*02:01	845	54	0.00153764	54	0.0015	TRUE
1	FQPFQQFGR	545	553	9	HLA-A*02:01	545	54	0.00153546	54	0.0015	TRUE
1	SGNCDVVIG	1105	1113	9	HLA-A*02:01	1105	54	0.00153028	54	0.0015	TRUE
1	GYLKPTTFM	255	263	9	HLA-A*02:01	255	54	0.00152726	54	0.0015	TRUE
1	GQTGVIADY	400	408	9	HLA-A*02:01	400	54	0.00152699	54	0.0015	TRUE

seq #	peptide	start	end	peptide length	allele	peptide index	median binding	mhcnp score	mhcnp percent	legacy score	scores equal
1	HGVVFLHVT	1040	1048	9	HLA-A*02:01	1040	54	0.00152612	54	0.0015	TRUE
1	SGSDLDRCT	12	20	9	HLA-A*02:01	12	55	0.00150829	55	0.0015	TRUE
1	FVFNGTSWF	1077	1085	9	HLA-A*02:01	1077	55	0.0014852	55	0.0015	TRUE
1	LFNKVTLAD	804	812	9	HLA-A*02:01	804	55	0.00148214	55	0.0015	TRUE
1	TPAWRIYST	616	624	9	HLA-A*02:01	616	55	0.00147998	55	0.0015	TRUE
1	AKNLNESLI	1172	1180	9	HLA-A*02:01	1172	55	0.00147753	55	0.0015	TRUE
1	VYYPDEIFR	40	48	9	HLA-A*02:01	40	55	0.00147256	55	0.0015	TRUE
1	TSAAAYFVG	247	255	9	HLA-A*02:01	247	55	0.00145875	55	0.0015	TRUE
1	ATKMSECVL	1008	1016	9	HLA-A*02:01	1008	55	0.0014498	55	0.0014	TRUE
1	VSKPMGTQT	140	148	9	HLA-A*02:01	140	55	0.00144303	55	0.0014	TRUE
1	ESLTTTSTA	918	926	9	HLA-A*02:01	918	55	0.00144043	55	0.0014	TRUE
1	TSQKSIVAY	669	677	9	HLA-A*02:01	669	55	0.00142787	55	0.0014	TRUE
1	PLGINITNF	223	231	9	HLA-A*02:01	223	56	0.00141636	56	0.0014	TRUE
1	SAAAYFVGY	248	256	9	HLA-A*02:01	248	56	0.00141536	56	0.0014	TRUE
1	NVLYENQKQ	896	904	9	HLA-A*02:01	896	56	0.001414	56	0.0014	TRUE
1	ADSSIAYSN	683	691	9	HLA-A*02:01	683	56	0.00140634	56	0.0014	TRUE
1	HTMIFDNAF	149	157	9	HLA-A*02:01	149	56	0.00140164	56	0.0014	TRUE
1	GACSCGSCC	1228	1236	9	HLA-A*02:01	1228	56	0.00139701	56	0.0014	TRUE
1	EQDRNTREV	755	763	9	HLA-A*02:01	755	56	0.00139287	56	0.0014	TRUE
1	KRSFIEDLL	796	804	9	HLA-A*02:01	796	56	0.00138595	56	0.0014	TRUE
1	YTQHTSSMR	30	38	9	HLA-A*02:01	30	56	0.00138174	56	0.0014	TRUE
1	TTTGIGYQP	485	493	9	HLA-A*02:01	485	56	0.00137888	56	0.0014	TRUE
1	FSPQIITTD	1092	1100	9	HLA-A*02:01	1092	56	0.00137583	56	0.0014	TRUE
1	LIKNQCVNF	519	527	9	HLA-A*02:01	519	56	0.00136834	56	0.0014	TRUE
1	GVVFLHVTY	1041	1049	9	HLA-A*02:01	1041	56	0.00135797	56	0.0014	TRUE
1	CKFDEDDSE	1236	1244	9	HLA-A*02:01	1236	56	0.00134664	56	0.0013	TRUE
1	GFHTINHTF	68	76	9	HLA-A*02:01	68	57	0.00132734	57	0.0013	TRUE
1	CANLLLQYG	731	739	9	HLA-A*02:01	731	57	0.00132647	57	0.0013	TRUE
1	VDTSYECDI	642	650	9	HLA-A*02:01	642	57	0.00131768	57	0.0013	TRUE
1	SPDGKPCTP	461	469	9	HLA-A*02:01	461	57	0.00131089	57	0.0013	TRUE
1	LTDDMIAAY	847	855	9	HLA-A*02:01	847	57	0.00129931	57	0.0013	TRUE
1	LDVSEKSGN	170	178	9	HLA-A*02:01	170	57	0.00128269	57	0.0013	TRUE
1	LGADSSIAY	681	689	9	HLA-A*02:01	681	57	0.00128031	57	0.0013	TRUE
1	GDDVRQIAP	391	399	9	HLA-A*02:01	391	57	0.0012789	57	0.0013	TRUE
1	ITDAVDCSQ	272	280	9	HLA-A*02:01	272	57	0.00127762	57	0.0013	TRUE
1	LDISPCSFG	571	579	9	HLA-A*02:01	571	57	0.00127676	57	0.0013	TRUE
1	TSTALGKLQ	923	931	9	HLA-A*02:01	923	57	0.0012717	57	0.0013	TRUE
1	CGPKLSTDL	511	519	9	HLA-A*02:01	511	57	0.00127053	57	0.0013	TRUE
1	SVDCNMYIC	717	725	9	HLA-A*02:01	717	57	0.00126144	57	0.0013	TRUE
1	IRASANLAA	1000	1008	9	HLA-A*02:01	1000	58	0.00125312	58	0.0013	TRUE
1	TINHTFGNP	71	79	9	HLA-A*02:01	71	58	0.00125269	58	0.0013	TRUE
1	CAQKFNGLT	833	841	9	HLA-A*02:01	833	58	0.00124734	58	0.0012	TRUE
1	CTDVSTAIH	603	611	9	HLA-A*02:01	603	58	0.00124072	58	0.0012	TRUE
1	CCSCLKGAC	1222	1230	9	HLA-A*02:01	1222	58	0.00123483	58	0.0012	TRUE
1	KDGIYFAAT	84	92	9	HLA-A*02:01	84	58	0.00122792	58	0.0012	TRUE
1	RDLICAQKF	829	837	9	HLA-A*02:01	829	58	0.00121728	58	0.0012	TRUE
1	GSFCTQLNR	739	747	9	HLA-A*02:01	739	58	0.00121413	58	0.0012	TRUE
1	YTTTGIGYQ	484	492	9	HLA-A*02:01	484	58	0.00120904	58	0.0012	TRUE
1	LCFSNVYAD	377	385	9	HLA-A*02:01	377	58	0.00120604	58	0.0012	TRUE
1	IGAGICASY	652	660	9	HLA-A*02:01	652	58	0.00120039	58	0.0012	TRUE
1	AELKCSVKS	284	292	9	HLA-A*02:01	284	58	0.00119208	58	0.0012	TRUE
1	MSFPQAAPH	1032	1040	9	HLA-A*02:01	1032	59	0.00117437	59	0.0012	TRUE
1	QTQAGCLIG	630	638	9	HLA-A*02:01	630	59	0.00117429	59	0.0012	TRUE
1	TTEVMPVSM	705	713	9	HLA-A*02:01	705	59	0.0011735	59	0.0012	TRUE
1	TQHTSSMRG	31	39	9	HLA-A*02:01	31	59	0.00116106	5.90E+01	1.20E-03	TRUE
1	VSDFTDSVR	555	563	9	HLA-A*02:01	555	59	0.00114973	5.90E+01	1.10E-03	TRUE
1	ADYNYKLPD	406	414	9	HLA-A*02:01	406	59	0.00114219	5.90E+01	1.10E-03	TRUE
1	PKTSEILDI	565	573	9	HLA-A*02:01	565	59	0.0011396	5.90E+01	1.10E-03	TRUE
1	SEVAVLYQD	592	600	9	HLA-A*02:01	592	59	0.00113637	5.90E+01	1.10E-03	TRUE
1	VQAPNYTQH	25	33	9	HLA-A*02:01	25	59	0.00113559	5.90E+01	1.10E-03	TRUE
1	KGIYQTSNF	297	305	9	HLA-A*02:01	297	59	0.00112003	5.90E+01	1.10E-03	TRUE
1	NCVADYSVL	347	355	9	HLA-A*02:01	347	59	0.00111366	5.90E+01	1.10E-03	TRUE
1	ADSFVVKGD	384	392	9	HLA-A*02:01	384	59	0.00111161	5.90E+01	1.10E-03	TRUE
1	VVLSFELLN	497	505	9	HLA-A*02:01	497	59	0.00110825	5.90E+01	1.10E-03	TRUE
1	NESLIDLQE	1176	1184	9	HLA-A*02:01	1176	59	0.00109771	5.90E+01	1.10E-03	TRUE
1	YRFNGIGVT	886	894	9	HLA-A*02:01	886	59	0.00109517	5.90E+01	1.10E-03	TRUE
1	LSFELLNAP	499	507	9	HLA-A*02:01	499	59	0.00109408	5.90E+01	1.10E-03	TRUE
1	CSCLKGACS	1223	1231	9	HLA-A*02:01	1223	59	0.00109173	5.90E+01	1.10E-03	TRUE
1	GNFKHLREF	177	185	9	HLA-A*02:01	177	59	0.00108749	5.90E+01	1.10E-03	TRUE
1	LSTDLIKNQ	515	523	9	HLA-A*02:01	515	60	0.00108009	6.00E+01	1.10E-03	TRUE
1	AYTAALVSG	854	862	9	HLA-A*02:01	854	60	0.00107903	6.00E+01	1.10E-03	TRUE
1	GYQPIDVVR	199	207	9	HLA-A*02:01	199	60	0.00107256	6.00E+01	1.10E-03	TRUE
1	FNKAISQIQ	909	917	9	HLA-A*02:01	909	60	0.00106924	6.00E+01	1.10E-03	TRUE
1	TMSLGADSS	678	686	9	HLA-A*02:01	678	60	0.00106006	6.00E+01	1.10E-03	TRUE
1	EHVDTSYEC	640	648	9	HLA-A*02:01	640	60	0.00102688	6.00E+01	1.00E-03	TRUE
1	QKSIVAYTM	671	679	9	HLA-A*02:01	671	60	0.00102534	6.00E+01	1.00E-03	TRUE
1	VNIQKEIDR	1159	1167	9	HLA-A*02:01	1159	60	0.00102199	6.00E+01	1.00E-03	TRUE
1	TQDLFLPFY	55	63	9	HLA-A*02:01	55	60	0.00102057	6.00E+01	1.00E-03	TRUE
1	GKLRPFERD	446	454	9	HLA-A*02:01	446	60	0.00101989	6.00E+01	1.00E-03	TRUE
1	GIINNTVYD	1113	1121	9	HLA-A*02:01	1113	60	0.00101593	6.00E+01	1.00E-03	TRUE

seq #	peptide	start	end	peptide length	allele	peptide index	median binding	mhcnp score	mhcnp percent	legacy score	scores equal
1	VVRFPNITN	313	321	9	HLA-A*02:01	313	60	0.00100851	6.00E+01	1.00E-03	TRUE
1	IINNTVYDP	1114	1122	9	HLA-A*02:01	1114	60	0.00100015	6.00E+01	1.00E-03	TRUE
1	AYSNNTIAI	688	696	9	HLA-A*02:01	688	60	0.00099739	6.00E+01	1.00E-03	TRUE
1	AWNTRNIDA	422	430	9	HLA-A*02:01	422	60	0.00099688	6.00E+01	1.00E-03	TRUE
1	KNLNESLID	1173	1181	9	HLA-A*02:01	1173	61	0.0009953	6.10E+01	1.00E-03	TRUE
1	SVLYNSTFF	353	361	9	HLA-A*02:01	353	61	0.00099099	6.10E+01	1.00E-03	TRUE
1	LAELKCSVK	283	291	9	HLA-A*02:01	283	61	0.00098156	6.10E+01	1.00E-03	TRUE
1	INNTVYDPL	1115	1123	9	HLA-A*02:01	1115	61	0.00097974	6.10E+01	1.00E-03	TRUE
1	CGDSTECAN	725	733	9	HLA-A*02:01	725	61	0.00097708	6.10E+01	1.00E-03	TRUE
1	TKRSFIEDL	795	803	9	HLA-A*02:01	795	61	0.0009726	6.10E+01	1.00E-03	TRUE
1	KRFQPFQQF	543	551	9	HLA-A*02:01	543	61	0.00096778	6.10E+01	1.00E-03	TRUE
1	HGKLRPFER	445	453	9	HLA-A*02:01	445	61	0.00095279	6.10E+01	1.00E-03	TRUE
1	LGKLQDVVN	927	935	9	HLA-A*02:01	927	61	0.00094554	6.10E+01	9.00E-04	TRUE
1	VRDLPSGFN	206	214	9	HLA-A*02:01	206	61	0.00094057	6.10E+01	9.00E-04	TRUE
1	QDLFLPFYS	56	64	9	HLA-A*02:01	56	61	0.00093861	6.10E+01	9.00E-04	TRUE
1	NNKSQSVII	108	116	9	HLA-A*02:01	108	61	0.00092771	6.10E+01	9.00E-04	TRUE
1	DAFSLDVSE	166	174	9	HLA-A*02:01	166	62	0.00092512	6.20E+01	9.00E-04	TRUE
1	FGEVFNATK	325	333	9	HLA-A*02:01	325	62	0.00092397	6.20E+01	9.00E-04	TRUE
1	APATVCGPK	506	514	9	HLA-A*02:01	506	62	0.00092128	6.20E+01	9.00E-04	TRUE
1	LQELGKYEQ	1182	1190	9	HLA-A*02:01	1182	62	0.00091617	6.20E+01	9.00E-04	TRUE
1	FKEELDKYF	1130	1138	9	HLA-A*02:01	1130	62	0.00091205	6.20E+01	9.00E-04	TRUE
1	KHLREFVFK	180	188	9	HLA-A*02:01	180	62	0.000912	6.20E+01	9.00E-04	TRUE
1	SNNTIAIPT	690	698	9	HLA-A*02:01	690	62	0.00090908	6.20E+01	9.00E-04	TRUE
1	FKCYGVSAT	364	372	9	HLA-A*02:01	364	62	0.00090486	6.20E+01	9.00E-04	TRUE
1	GKGYHLMSF	1026	1034	9	HLA-A*02:01	1026	62	0.00089785	6.20E+01	9.00E-04	TRUE
1	EVAVLYQDV	593	601	9	HLA-A*02:01	593	62	0.0008859	6.20E+01	9.00E-04	TRUE
1	DSTECANLL	727	735	9	HLA-A*02:01	727	62	0.00088521	6.20E+01	9.00E-04	TRUE
1	LNESLIDLQ	1175	1183	9	HLA-A*02:01	1175	62	0.00088434	6.20E+01	9.00E-04	TRUE
1	VTGFHTINH	66	74	9	HLA-A*02:01	66	62	0.00088191	6.20E+01	9.00E-04	TRUE
1	MNNKSQSVI	107	115	9	HLA-A*02:01	107	62	0.00087091	6.20E+01	9.00E-04	TRUE
1	NNTIAIPTN	691	699	9	HLA-A*02:01	691	62	0.00086937	6.20E+01	9.00E-04	TRUE
1	MLKYDENGT	263	271	9	HLA-A*02:01	263	62	0.00086821	6.20E+01	9.00E-04	TRUE
1	KNHTSPDVD	1139	1147	9	HLA-A*02:01	1139	62	0.0008673	6.20E+01	9.00E-04	TRUE
1	GNPVIPFKD	77	85	9	HLA-A*02:01	77	62	0.00086478	6.20E+01	9.00E-04	TRUE
1	RFQPFQQFG	544	552	9	HLA-A*02:01	544	63	0.00086282	6.30E+01	9.00E-04	TRUE
1	QIITTDNTF	1095	1103	9	HLA-A*02:01	1095	63	0.00085788	6.30E+01	9.00E-04	TRUE
1	FRAILTAFS	231	239	9	HLA-A*02:01	231	63	0.00085626	6.30E+01	9.00E-04	TRUE
1	IFLLFLTLT	3	11	9	HLA-A*02:01	3	63	0.0008561	6.30E+01	9.00E-04	TRUE
1	TTDNTFVSG	1098	1106	9	HLA-A*02:01	1098	63	0.00085339	6.30E+01	9.00E-04	TRUE
1	YTMSLGADS	677	685	9	HLA-A*02:01	677	63	0.00085136	6.30E+01	9.00E-04	TRUE
1	KISNCVADY	344	352	9	HLA-A*02:01	344	63	0.00084427	6.30E+01	8.00E-04	TRUE
1	TFVSGNCDV	1102	1110	9	HLA-A*02:01	1102	63	0.00083941	6.30E+01	8.00E-04	TRUE
1	PSQERNFTT	1051	1059	9	HLA-A*02:01	1051	63	0.00083025	6.30E+01	8.00E-04	TRUE
1	TSSMRGVYY	34	42	9	HLA-A*02:01	34	63	0.00082802	6.30E+01	8.00E-04	TRUE
1	TAGWTFGAG	865	873	9	HLA-A*02:01	865	63	0.00082737	6.30E+01	8.00E-04	TRUE
1	KTPTLKYFG	772	780	9	HLA-A*02:01	772	63	0.00082145	6.30E+01	8.00E-04	TRUE
1	SGIAAEQDR	750	758	9	HLA-A*02:01	750	63	0.000812	6.30E+01	8.00E-04	TRUE
1	FERDISNVP	451	459	9	HLA-A*02:01	451	63	0.00081197	6.30E+01	8.00E-04	TRUE
1	EVAKNLNES	1170	1178	9	HLA-A*02:01	1170	63	0.00081181	6.30E+01	8.00E-04	TRUE
1	VSGTATAGW	860	868	9	HLA-A*02:01	860	63	0.00081167	6.30E+01	8.00E-04	TRUE
1	MAYRFNGIG	884	892	9	HLA-A*02:01	884	64	0.00080622	6.40E+01	8.00E-04	TRUE
1	IDATSTGNY	428	436	9	HLA-A*02:01	428	64	0.00079614	6.40E+01	8.00E-04	TRUE
1	RAILTAFSP	232	240	9	HLA-A*02:01	232	64	0.0007948	6.40E+01	8.00E-04	TRUE
1	NMYICGDST	721	729	9	HLA-A*02:01	721	64	0.00079474	6.40E+01	8.00E-04	TRUE
1	FMGCVLAWN	416	424	9	HLA-A*02:01	416	64	0.00079153	6.40E+01	8.00E-04	TRUE
1	NKVTLADAG	806	814	9	HLA-A*02:01	806	64	0.00078716	6.40E+01	8.00E-04	TRUE
1	ASANLAATK	1002	1010	9	HLA-A*02:01	1002	64	0.00078503	6.40E+01	8.00E-04	TRUE
1	LGQSKRVDF	1016	1024	9	HLA-A*02:01	1016	64	0.00078194	6.40E+01	8.00E-04	TRUE
1	NTVYDPLQP	1117	1125	9	HLA-A*02:01	1117	64	0.00078166	6.40E+01	8.00E-04	TRUE
1	VVKGDDVRQ	388	396	9	HLA-A*02:01	388	64	0.00078075	6.40E+01	8.00E-04	TRUE
1	IADYNYKLP	405	413	9	HLA-A*02:01	405	64	0.00077841	6.40E+01	8.00E-04	TRUE
1	NFFSPQIIT	1090	1098	9	HLA-A*02:01	1090	64	0.00077466	6.40E+01	8.00E-04	TRUE
1	LLCCMTSCC	1215	1223	9	HLA-A*02:01	1215	64	0.00077174	6.40E+01	8.00E-04	TRUE
1	SGNFKHLRE	176	184	9	HLA-A*02:01	176	64	0.00076981	6.40E+01	8.00E-04	TRUE
1	SGINASVVN	1152	1160	9	HLA-A*02:01	1152	64	0.00076907	6.40E+01	8.00E-04	TRUE
1	KPCTPPALN	465	473	9	HLA-A*02:01	465	64	0.00076565	6.40E+01	8.00E-04	TRUE
1	VVRGWVFGS	97	105	9	HLA-A*02:01	97	64	0.00075757	6.40E+01	8.00E-04	TRUE
1	NARDLICAQ	827	835	9	HLA-A*02:01	827	64	0.00075739	6.40E+01	8.00E-04	TRUE
1	KSQSVIIIN	110	118	9	HLA-A*02:01	110	64	0.00074867	6.40E+01	7.00E-04	TRUE
1	HTINHTFGN	70	78	9	HLA-A*02:01	70	65	0.00074568	6.50E+01	7.00E-04	TRUE
1	ADAGFMKQY	811	819	9	HLA-A*02:01	811	65	0.000731	6.50E+01	7.00E-04	TRUE
1	PFGEVFNAT	324	332	9	HLA-A*02:01	324	65	0.00073021	6.50E+01	7.00E-04	TRUE
1	LRSTSQKSI	666	674	9	HLA-A*02:01	666	65	0.00072647	6.50E+01	7.00E-04	TRUE
1	TTTSTALGK	921	929	9	HLA-A*02:01	921	65	0.00071759	6.50E+01	7.00E-04	TRUE
1	AAEIRASAN	997	1005	9	HLA-A*02:01	997	65	0.00071746	6.50E+01	7.00E-04	TRUE
1	PFSPDGKPC	459	467	9	HLA-A*02:01	459	65	0.0007102	6.50E+01	7.00E-04	TRUE
1	DDVRQIAPG	392	400	9	HLA-A*02:01	392	65	0.00070958	6.50E+01	7.00E-04	TRUE
1	GRDVSDFTD	552	560	9	HLA-A*02:01	552	65	0.00070524	6.50E+01	7.00E-04	TRUE
1	SGTATAGWT	861	869	9	HLA-A*02:01	861	65	0.00070038	6.50E+01	7.00E-04	TRUE
1	QCVNFNFNG	523	531	9	HLA-A*02:01	523	65	0.00069986	6.50E+01	7.00E-04	TRUE

seq #	peptide	start	end	peptide length	allele	peptide index	median binding	mhcnp score	mhcnp percent	legacy score	scores equal
1	LHVTYVPSQ	1045	1053	9	HLA-A*02:01	1045	66	0.00069503	6.60E+01	7.00E-04	TRUE
1	RACNFELCD	126	134	9	HLA-A*02:01	126	66	0.00068923	6.60E+01	7.00E-04	TRUE
1	AFSPAQDIW	237	245	9	HLA-A*02:01	237	66	0.00068865	6.60E+01	7.00E-04	TRUE
1	IQKEIDRLN	1161	1169	9	HLA-A*02:01	1161	66	0.00068424	6.60E+01	7.00E-04	TRUE
1	NDYGFYTTT	479	487	9	HLA-A*02:01	479	66	0.00068312	6.60E+01	7.00E-04	TRUE
1	YFAATEKSN	88	96	9	HLA-A*02:01	88	66	0.00068054	6.60E+01	7.00E-04	TRUE
1	CDNPFFAVS	133	141	9	HLA-A*02:01	133	66	0.00067811	6.60E+01	7.00E-04	TRUE
1	CTPPALNCY	467	475	9	HLA-A*02:01	467	66	0.00067351	6.60E+01	7.00E-04	TRUE
1	ARDLICAQK	828	836	9	HLA-A*02:01	828	66	0.0006695	6.60E+01	7.00E-04	TRUE
1	AYFPREGVF	1069	1077	9	HLA-A*02:01	1069	66	0.00066215	6.60E+01	7.00E-04	TRUE
1	RDVSDFTDS	553	561	9	HLA-A*02:01	553	66	0.0006574	6.60E+01	7.00E-04	TRUE
1	EAEVQIDRL	970	978	9	HLA-A*02:01	970	66	0.00065692	6.60E+01	7.00E-04	TRUE
1	WFITQRNFF	1084	1092	9	HLA-A*02:01	1084	66	0.00065579	6.60E+01	7.00E-04	TRUE
1	ITGRLQSLQ	979	987	9	HLA-A*02:01	979	66	0.00065426	6.60E+01	7.00E-04	TRUE
1	YSNNTIAIP	689	697	9	HLA-A*02:01	689	67	0.00065283	6.70E+01	7.00E-04	TRUE
1	TATAGWTFG	863	871	9	HLA-A*02:01	863	67	0.00064646	6.70E+01	6.00E-04	TRUE
1	SDAFSLDVS	165	173	9	HLA-A*02:01	165	67	0.00064551	6.70E+01	6.00E-04	TRUE
1	LFLTLTSGS	6	14	9	HLA-A*02:01	6	67	0.00064521	6.70E+01	6.00E-04	TRUE
1	EVMPVSMAK	707	715	9	HLA-A*02:01	707	67	0.00064471	6.70E+01	6.00E-04	TRUE
1	DIPIGAGIC	649	657	9	HLA-A*02:01	649	67	0.00064039	6.70E+01	6.00E-04	TRUE
1	FPNITNLCP	316	324	9	HLA-A*02:01	316	67	0.00063231	6.70E+01	6.00E-04	TRUE
1	NVPFSPDGK	457	465	9	HLA-A*02:01	457	67	0.00063207	6.70E+01	6.00E-04	TRUE
1	YPDEIFRSD	42	50	9	HLA-A*02:01	42	67	0.00062516	6.70E+01	6.00E-04	TRUE
1	LPLGINITN	222	230	9	HLA-A*02:01	222	67	0.00062496	6.70E+01	6.00E-04	TRUE
1	APNYTQHTS	27	35	9	HLA-A*02:01	27	67	0.00062057	6.70E+01	6.00E-04	TRUE
1	TQTHTMIFD	146	154	9	HLA-A*02:01	146	67	0.00061984	6.70E+01	6.00E-04	TRUE
1	GTSWFITQR	1081	1089	9	HLA-A*02:01	1081	67	0.0006164	6.70E+01	6.00E-04	TRUE
1	HVDTSYECD	641	649	9	HLA-A*02:01	641	67	0.0006137	6.70E+01	6.00E-04	TRUE
1	KSGNFKHLR	175	183	9	HLA-A*02:01	175	67	0.00060944	6.70E+01	6.00E-04	TRUE
1	AFNCTFEYI	156	164	9	HLA-A*02:01	156	68	0.00060526	6.80E+01	6.00E-04	TRUE
1	DDMIAAYTA	849	857	9	HLA-A*02:01	849	68	0.00059503	6.80E+01	6.00E-04	TRUE
1	LQPELDSFK	1123	1131	9	HLA-A*02:01	1123	68	0.00059188	6.80E+01	6.00E-04	TRUE
1	IDLQELGKY	1180	1188	9	HLA-A*02:01	1180	68	0.00059093	6.80E+01	6.00E-04	TRUE
1	IYFAATEKS	87	95	9	HLA-A*02:01	87	68	0.0005857	6.80E+01	6.00E-04	TRUE
1	SSEVAVLYQ	591	599	9	HLA-A*02:01	591	68	0.00058563	6.80E+01	6.00E-04	TRUE
1	TNFSISITT	698	706	9	HLA-A*02:01	698	68	0.00058141	6.80E+01	6.00E-04	TRUE
1	RGWVFGSTM	99	107	9	HLA-A*02:01	99	68	0.00058121	6.80E+01	6.00E-04	TRUE
1	GKAYFPREG	1067	1075	9	HLA-A*02:01	1067	68	0.00058055	6.80E+01	6.00E-04	TRUE
1	PGQTGVIAD	399	407	9	HLA-A*02:01	399	68	0.00058047	6.80E+01	6.00E-04	TRUE
1	VIIINNSTN	114	122	9	HLA-A*02:01	114	68	0.00058006	6.80E+01	6.00E-04	TRUE
1	ANQFNKAIS	906	914	9	HLA-A*02:01	906	68	0.00057805	6.80E+01	6.00E-04	TRUE
1	LDKVEAEVQ	966	974	9	HLA-A*02:01	966	68	0.00057626	6.80E+01	6.00E-04	TRUE
1	GKYEQYIKW	1186	1194	9	HLA-A*02:01	1186	68	0.00057519	6.80E+01	6.00E-04	TRUE
1	FFSTFKCYG	360	368	9	HLA-A*02:01	360	68	0.0005712	6.80E+01	6.00E-04	TRUE
1	NATKFPSVY	330	338	9	HLA-A*02:01	330	68	0.00056904	6.80E+01	6.00E-04	TRUE
1	LNTLVKQLS	941	949	9	HLA-A*02:01	941	68	0.00056394	6.80E+01	6.00E-04	TRUE
1	TEVMPVSMA	706	714	9	HLA-A*02:01	706	69	0.00055597	6.90E+01	6.00E-04	TRUE
1	DEIFRSDTL	44	52	9	HLA-A*02:01	44	69	0.00055443	6.90E+01	6.00E-04	TRUE
1	ITTDNTFVS	1097	1105	9	HLA-A*02:01	1097	69	0.00055276	6.90E+01	6.00E-04	TRUE
1	KCYGVSATK	365	373	9	HLA-A*02:01	365	69	0.00054955	6.90E+01	5.00E-04	TRUE
1	GSCCKFDED	1233	1241	9	HLA-A*02:01	1233	69	0.00054899	6.90E+01	5.00E-04	TRUE
1	GVFVFNGTS	1075	1083	9	HLA-A*02:01	1075	69	0.00054607	6.90E+01	5.00E-04	TRUE
1	YNYKLPDDF	408	416	9	HLA-A*02:01	408	69	0.00054148	6.90E+01	5.00E-04	TRUE
1	APHGVVFLH	1038	1046	9	HLA-A*02:01	1038	69	0.00053973	6.90E+01	5.00E-04	TRUE
1	NKDGFLYVY	189	197	9	HLA-A*02:01	189	69	0.00053744	6.90E+01	5.00E-04	TRUE
1	NCDVVIGII	1107	1115	9	HLA-A*02:01	1107	69	0.00053726	6.90E+01	5.00E-04	TRUE
1	KKISNCVAD	343	351	9	HLA-A*02:01	343	69	0.00053707	6.90E+01	5.00E-04	TRUE
1	CLKGACSCG	1225	1233	9	HLA-A*02:01	1225	69	0.00053047	6.90E+01	5.00E-04	TRUE
1	NYTQHTSSM	29	37	9	HLA-A*02:01	29	69	0.00052869	6.90E+01	5.00E-04	TRUE
1	DTLYLTQDL	50	58	9	HLA-A*02:01	50	69	0.00052706	6.90E+01	5.00E-04	TRUE
1	LDSFKEELD	1127	1135	9	HLA-A*02:01	1127	69	0.00052563	6.90E+01	5.00E-04	TRUE
1	EYISDAFSL	162	170	9	HLA-A*02:01	162	69	0.00052051	6.90E+01	5.00E-04	TRUE
1	CTFEYISDA	159	167	9	HLA-A*02:01	159	69	0.00052044	6.90E+01	5.00E-04	TRUE
1	NFSISITTE	699	707	9	HLA-A*02:01	699	69	0.00051987	6.90E+01	5.00E-04	TRUE
1	AGCLIGAEH	633	641	9	HLA-A*02:01	633	69	0.00051745	6.90E+01	5.00E-04	TRUE
1	VIPFKDGIY	80	88	9	HLA-A*02:01	80	69	0.00051719	6.90E+01	5.00E-04	TRUE
1	QYGECLGDI	818	826	9	HLA-A*02:01	818	69	0.00051691	6.90E+01	5.00E-04	TRUE
1	AEVQIDRLI	971	979	9	HLA-A*02:01	971	70	0.00051171	7.00E+01	5.00E-04	TRUE
1	TFKCYGVSA	363	371	9	HLA-A*02:01	363	70	0.00051004	7.00E+01	5.00E-04	TRUE
1	GFYTTTGIG	482	490	9	HLA-A*02:01	482	70	0.00050835	7.00E+01	5.00E-04	TRUE
1	SNVVRGWVF	95	103	9	HLA-A*02:01	95	70	0.00050776	7.00E+01	5.00E-04	TRUE
1	LSSNFGAIS	948	956	9	HLA-A*02:01	948	70	0.00050745	7.00E+01	5.00E-04	TRUE
1	SVYAWERKK	336	344	9	HLA-A*02:01	336	70	0.00050365	7.00E+01	5.00E-04	TRUE
1	GPKLSTDLI	512	520	9	HLA-A*02:01	512	70	0.00050349	7.00E+01	5.00E-04	TRUE
1	SKRFQPFQQ	542	550	9	HLA-A*02:01	542	70	0.00050229	7.00E+01	5.00E-04	TRUE
1	YFKNHTSPD	1137	1145	9	HLA-A*02:01	1137	70	0.00049104	7.00E+01	5.00E-04	TRUE
1	LPFYSNVTG	60	68	9	HLA-A*02:01	60	70	0.00049101	7.00E+01	5.00E-04	TRUE
1	DRNTREVFA	757	765	9	HLA-A*02:01	757	70	0.00048438	7.00E+01	5.00E-04	TRUE
1	QKEIDRLNE	1162	1170	9	HLA-A*02:01	1162	70	0.00048267	7.00E+01	5.00E-04	TRUE
1	CTQLNRALS	742	750	9	HLA-A*02:01	742	70	0.00048252	7.00E+01	5.00E-04	TRUE

seq #	peptide	start	end	peptide length	allele	peptide index	median binding	mhcnp score	mhcnp percent	legacy score	scores equal
1	SNVYADSFV	380	388	9	HLA-A*02:01	380	70	0.00048071	7.00E+01	5.00E-04	TRUE
1	NVVRGWVFG	96	104	9	HLA-A*02:01	96	70	0.0004772	7.00E+01	5.00E-04	TRUE
1	KPTKRSFIE	793	801	9	HLA-A*02:01	793	70	0.00047558	7.00E+01	5.00E-04	TRUE
1	DVQAPNYTQ	24	32	9	HLA-A*02:01	24	70	0.00047557	7.00E+01	5.00E-04	TRUE
1	IVAYTMSLG	674	682	9	HLA-A*02:01	674	71	0.00047062	7.10E+01	5.00E-04	TRUE
1	FPSVYAWER	334	342	9	HLA-A*02:01	334	71	0.00046911	7.10E+01	5.00E-04	TRUE
1	YDENGTITD	266	274	9	HLA-A*02:01	266	71	0.00046492	7.10E+01	5.00E-04	TRUE
1	ITQRNFFSP	1086	1094	9	HLA-A*02:01	1086	71	0.0004647	7.10E+01	5.00E-04	TRUE
1	AAYFVGYLK	250	258	9	HLA-A*02:01	250	71	0.00046412	7.10E+01	5.00E-04	TRUE
1	SGDVVRFPN	310	318	9	HLA-A*02:01	310	71	0.00046365	7.10E+01	5.00E-04	TRUE
1	LKGACSCGS	1226	1234	9	HLA-A*02:01	1226	71	0.00046071	7.10E+01	5.00E-04	TRUE
1	NVTGFHTIN	65	73	9	HLA-A*02:01	65	71	0.00045993	7.10E+01	5.00E-04	TRUE
1	LGINITNFR	224	232	9	HLA-A*02:01	224	71	0.00045883	7.10E+01	5.00E-04	TRUE
1	VTQNVLYEN	893	901	9	HLA-A*02:01	893	71	0.00045521	7.10E+01	5.00E-04	TRUE
1	KFPSVYAWE	333	341	9	HLA-A*02:01	333	71	0.00044808	7.10E+01	4.00E-04	TRUE
1	ATSTGNYNY	430	438	9	HLA-A*02:01	430	71	0.00044661	7.10E+01	4.00E-04	TRUE
1	CGKGYHLMS	1025	1033	9	HLA-A*02:01	1025	71	0.00044605	7.10E+01	4.00E-04	TRUE
1	VKQMYKTPT	767	775	9	HLA-A*02:01	767	71	0.00043931	7.10E+01	4.00E-04	TRUE
1	PLKPTKRSF	791	799	9	HLA-A*02:01	791	71	0.00043748	7.10E+01	4.00E-04	TRUE
1	GRLQSLQTY	981	989	9	HLA-A*02:01	981	71	0.00043666	7.10E+01	4.00E-04	TRUE
1	CVNFNFNGL	524	532	9	HLA-A*02:01	524	72	0.00043565	7.20E+01	4.00E-04	TRUE
1	MAKTSVDCN	713	721	9	HLA-A*02:01	713	72	0.00043292	7.20E+01	4.00E-04	TRUE
1	TGVLTPSSK	535	543	9	HLA-A*02:01	535	72	0.00043256	7.20E+01	4.00E-04	TRUE
1	ATKLNDLCF	371	379	9	HLA-A*02:01	371	72	0.00042925	7.20E+01	4.00E-04	TRUE
1	VVIRACNFE	123	131	9	HLA-A*02:01	123	72	0.00042697	7.20E+01	4.00E-04	TRUE
1	YGVSATKLN	367	375	9	HLA-A*02:01	367	72	0.00042102	7.20E+01	4.00E-04	TRUE
1	KYFGGFNFS	777	785	9	HLA-A*02:01	777	72	0.00041986	7.20E+01	4.00E-04	TRUE
1	MVTILLCCM	1211	1219	9	HLA-A*02:01	1211	72	0.00041872	7.20E+01	4.00E-04	TRUE
1	AMQMAYRFN	881	889	9	HLA-A*02:01	881	72	0.00041714	7.20E+01	4.00E-04	TRUE
1	LCPFGEVFN	322	330	9	HLA-A*02:01	322	72	0.00041589	7.20E+01	4.00E-04	TRUE
1	LVKQLSSNF	944	952	9	HLA-A*02:01	944	72	0.00040883	7.20E+01	4.00E-04	TRUE
1	FSNVYADSF	379	387	9	HLA-A*02:01	379	72	0.00040855	7.20E+01	4.00E-04	TRUE
1	ATEKSNVVR	91	99	9	HLA-A*02:01	91	72	0.00040598	7.20E+01	4.00E-04	TRUE
1	YGSFCTQLN	738	746	9	HLA-A*02:01	738	73	0.00039723	7.30E+01	4.00E-04	TRUE
1	EDDSEPVLK	1240	1248	9	HLA-A*02:01	1240	73	0.00039559	7.30E+01	4.00E-04	TRUE
1	AEHVDTSYE	639	647	9	HLA-A*02:01	639	73	0.00039515	7.30E+01	4.00E-04	TRUE
1	WVFGSTMNN	101	109	9	HLA-A*02:01	101	73	0.00039434	7.30E+01	4.00E-04	TRUE
1	VKQLSSNFG	945	953	9	HLA-A*02:01	945	73	0.00038869	7.30E+01	4.00E-04	TRUE
1	PHGVVFLHV	1039	1047	9	HLA-A*02:01	1039	73	0.00038654	7.30E+01	4.00E-04	TRUE
1	LQYGSFCTQ	736	744	9	HLA-A*02:01	736	73	0.00038175	7.30E+01	4.00E-04	TRUE
1	MFIFLLFLT	1	9	9	HLA-A*02:01	1	73	0.00037726	7.30E+01	4.00E-04	TRUE
1	SDFTDSVRD	556	564	9	HLA-A*02:01	556	73	0.00037482	7.30E+01	4.00E-04	TRUE
1	PFFAVSKPM	136	144	9	HLA-A*02:01	136	74	0.00037089	7.40E+01	4.00E-04	TRUE
1	ICASYHTVS	656	664	9	HLA-A*02:01	656	74	0.00036543	7.40E+01	4.00E-04	TRUE
1	LAWNTRNID	421	429	9	HLA-A*02:01	421	74	0.00036475	7.40E+01	4.00E-04	TRUE
1	PVLKGVKLH	1245	1253	9	HLA-A*02:01	1245	74	0.00036047	7.40E+01	4.00E-04	TRUE
1	GWTFGAGAA	867	875	9	HLA-A*02:01	867	74	0.00035893	7.40E+01	4.00E-04	TRUE
1	IDRLNEVAK	1165	1173	9	HLA-A*02:01	1165	74	0.00035584	7.40E+01	4.00E-04	TRUE
1	EIRASANLA	999	1007	9	HLA-A*02:01	999	74	0.00035396	7.40E+01	4.00E-04	TRUE
1	KGYHLMSFP	1027	1035	9	HLA-A*02:01	1027	74	0.00034798	7.40E+01	3.00E-04	TRUE
1	CMTSCCSCL	1218	1226	9	HLA-A*02:01	1218	74	0.00034711	7.40E+01	3.00E-04	TRUE
1	STMNNKSQS	105	113	9	HLA-A*02:01	105	74	0.00034465	7.40E+01	3.00E-04	TRUE
1	PIDVVRDLP	202	210	9	HLA-A*02:01	202	74	0.00034194	7.40E+01	3.00E-04	TRUE
1	LRHGKLRPF	443	451	9	HLA-A*02:01	443	75	0.00033801	7.50E+01	3.00E-04	TRUE
1	EKSGNFKHL	174	182	9	HLA-A*02:01	174	75	0.00033531	7.50E+01	3.00E-04	TRUE
1	ISNCVADYS	345	353	9	HLA-A*02:01	345	75	0.00033521	7.50E+01	3.00E-04	TRUE
1	SMRGVYYPD	36	44	9	HLA-A*02:01	36	75	0.00033144	7.50E+01	3.00E-04	TRUE
1	NGTITDAVD	269	277	9	HLA-A*02:01	269	75	0.00033096	7.50E+01	3.00E-04	TRUE
1	APAICHEGK	1060	1068	9	HLA-A*02:01	1060	75	0.00032965	7.50E+01	3.00E-04	TRUE
1	DVVRFPNIT	312	320	9	HLA-A*02:01	312	75	0.00032903	7.50E+01	3.00E-04	TRUE
1	AIPTNFSIS	695	703	9	HLA-A*02:01	695	75	0.00032711	7.50E+01	3.00E-04	TRUE
1	WGTSAAAYF	245	253	9	HLA-A*02:01	245	75	0.00032463	7.50E+01	3.00E-04	TRUE
1	TILLCCMTS	1213	1221	9	HLA-A*02:01	1213	75	0.00032353	7.50E+01	3.00E-04	TRUE
1	ALNCYWPLN	471	479	9	HLA-A*02:01	471	75	0.00031728	7.50E+01	3.00E-04	TRUE
1	GVIADYNYK	403	411	9	HLA-A*02:01	403	75	0.00031646	7.50E+01	3.00E-04	TRUE
1	STGNYNYKY	432	440	9	HLA-A*02:01	432	75	0.00031644	7.50E+01	3.00E-04	TRUE
1	TLKYFGGFN	775	783	9	HLA-A*02:01	775	76	0.00030733	7.60E+01	3.00E-04	TRUE
1	DVRQIAPGQ	393	401	9	HLA-A*02:01	393	76	0.00030181	7.60E+01	3.00E-04	TRUE
1	VSEKSGNFK	172	180	9	HLA-A*02:01	172	76	0.00030087	7.60E+01	3.00E-04	TRUE
1	TRNIDATST	425	433	9	HLA-A*02:01	425	76	0.0002962	7.60E+01	3.00E-04	TRUE
1	AEQDRNTRE	754	762	9	HLA-A*02:01	754	76	0.00029211	7.60E+01	3.00E-04	TRUE
1	QPYRVVVLS	492	500	9	HLA-A*02:01	492	76	0.00029141	7.60E+01	3.00E-04	TRUE
1	TKLNDLCFS	372	380	9	HLA-A*02:01	372	76	0.00029069	7.60E+01	3.00E-04	TRUE
1	PFYSNVTGF	61	69	9	HLA-A*02:01	61	76	0.00028658	7.60E+01	3.00E-04	TRUE
1	RHGKLRPFE	444	452	9	HLA-A*02:01	444	77	0.00028424	7.70E+01	3.00E-04	TRUE
1	TTFMLKYDE	260	268	9	HLA-A*02:01	260	77	0.00027596	7.70E+01	3.00E-04	TRUE
1	NFNFNGLTG	526	534	9	HLA-A*02:01	526	77	0.00027272	7.70E+01	3.00E-04	TRUE
1	NNTVYDPLQ	1116	1124	9	HLA-A*02:01	1116	77	0.00027049	7.70E+01	3.00E-04	TRUE
1	KNQCVNFNF	521	529	9	HLA-A*02:01	521	77	0.00026574	7.70E+01	3.00E-04	TRUE
1	CVLAWNTRN	419	427	9	HLA-A*02:01	419	77	0.00025975	7.70E+01	3.00E-04	TRUE

seq #	peptide	start	end	peptide length	allele	peptide index	median binding	mhcnp score	mhcnp percent	legacy score	scores equal
1	TDDMIAAYT	848	856	9	HLA-A*02:01	848	78	0.00025449	7.80E+01	3.00E-04	TRUE
1	FEYISDAFS	161	169	9	HLA-A*02:01	161	78	0.00025325	7.80E+01	3.00E-04	TRUE
1	QESLTTTST	917	925	9	HLA-A*02:01	917	78	0.00025147	7.80E+01	3.00E-04	TRUE
1	YSVLYNSTF	352	360	9	HLA-A*02:01	352	78	0.00024979	7.80E+01	2.00E-04	TRUE
1	SFKEELDKY	1129	1137	9	HLA-A*02:01	1129	78	0.00024917	7.80E+01	2.00E-04	TRUE
1	MGCVLAWNT	417	425	9	HLA-A*02:01	417	78	0.00024704	7.80E+01	2.00E-04	TRUE
1	TSWFITQRN	1082	1090	9	HLA-A*02:01	1082	78	0.00024297	7.80E+01	2.00E-04	TRUE
1	LNEVAKNLN	1168	1176	9	HLA-A*02:01	1168	78	0.00024047	7.80E+01	2.00E-04	TRUE
1	DDVQAPNYT	23	31	9	HLA-A*02:01	23	78	0.00023802	7.80E+01	2.00E-04	TRUE
1	YHTVSLLRS	660	668	9	HLA-A*02:01	660	78	0.00023766	7.80E+01	2.00E-04	TRUE
1	LTSGSDLDR	10	18	9	HLA-A*02:01	10	78	0.00023756	7.80E+01	2.00E-04	TRUE
1	NFRVVPSGD	304	312	9	HLA-A*02:01	304	78	0.00023692	7.80E+01	2.00E-04	TRUE
1	RVDFCGKGY	1021	1029	9	HLA-A*02:01	1021	78	0.00023345	7.80E+01	2.00E-04	TRUE
1	VDFCGKGYH	1022	1030	9	HLA-A*02:01	1022	78	0.00023327	7.80E+01	2.00E-04	TRUE
1	TSCCSCLKG	1220	1228	9	HLA-A*02:01	1220	79	0.00022996	7.90E+01	2.00E-04	TRUE
1	WNTRNIDAT	423	431	9	HLA-A*02:01	423	79	0.00022904	7.90E+01	2.00E-04	TRUE
1	QFGRDVSDF	550	558	9	HLA-A*02:01	550	79	0.00022454	7.90E+01	2.00E-04	TRUE
1	LKCSVKSFE	286	294	9	HLA-A*02:01	286	79	0.00022267	7.90E+01	2.00E-04	TRUE
1	NFSQILPDP	783	791	9	HLA-A*02:01	783	79	0.0002225	7.90E+01	2.00E-04	TRUE
1	TSEILDISP	567	575	9	HLA-A*02:01	567	79	0.00022246	7.90E+01	2.00E-04	TRUE
1	SDTLYLTQD	49	57	9	HLA-A*02:01	49	79	0.00021695	7.90E+01	2.00E-04	TRUE
1	DENGTITDA	267	275	9	HLA-A*02:01	267	79	0.00021669	7.90E+01	2.00E-04	TRUE
1	CCMTSCCSC	1217	1225	9	HLA-A*02:01	1217	79	0.00021664	7.90E+01	2.00E-04	TRUE
1	PALNCYWPL	470	478	9	HLA-A*02:01	470	79	0.0002164	7.90E+01	2.00E-04	TRUE
1	GFNFSQILP	781	789	9	HLA-A*02:01	781	79	0.00021527	7.90E+01	2.00E-04	TRUE
1	DGFLYVYKG	191	199	9	HLA-A*02:01	191	79	0.00021364	7.90E+01	2.00E-04	TRUE
1	DGIYFAATE	85	93	9	HLA-A*02:01	85	80	0.0002069	8.00E+01	2.00E-04	TRUE
1	VAVLYQDVN	594	602	9	HLA-A*02:01	594	80	0.00020638	8.00E+01	2.00E-04	TRUE
1	REGVFVFNG	1073	1081	9	HLA-A*02:01	1073	80	0.00020308	8.00E+01	2.00E-04	TRUE
1	LRPFERDIS	448	456	9	HLA-A*02:01	448	80	0.00020225	8.00E+01	2.00E-04	TRUE
1	SVKSFEIDK	289	297	9	HLA-A*02:01	289	80	0.00020058	8.00E+01	2.00E-04	TRUE
1	YKYRYLRHG	438	446	9	HLA-A*02:01	438	80	0.00019922	8.00E+01	2.00E-04	TRUE
1	ERDISNVPF	452	460	9	HLA-A*02:01	452	80	0.00019856	8.00E+01	2.00E-04	TRUE
1	NVVIRACNF	122	130	9	HLA-A*02:01	122	80	0.00019789	8.00E+01	2.00E-04	TRUE
1	PDVDLGDIS	1144	1152	9	HLA-A*02:01	1144	80	0.00019772	8.00E+01	2.00E-04	TRUE
1	SPCSFGGVS	574	582	9	HLA-A*02:01	574	80	0.00019677	8.00E+01	2.00E-04	TRUE
1	VEAEVQIDR	969	977	9	HLA-A*02:01	969	80	0.00019404	8.00E+01	2.00E-04	TRUE
1	KSIVAYTMS	672	680	9	HLA-A*02:01	672	80	0.00019365	8.00E+01	2.00E-04	TRUE
1	KDGFLYVYK	190	198	9	HLA-A*02:01	190	81	0.00019081	8.10E+01	2.00E-04	TRUE
1	MSECVLGQS	1011	1019	9	HLA-A*02:01	1011	81	0.00018901	8.10E+01	2.00E-04	TRUE
1	QTGVIADYN	401	409	9	HLA-A*02:01	401	81	0.00018633	8.10E+01	2.00E-04	TRUE
1	DCSQNPLAE	277	285	9	HLA-A*02:01	277	81	0.00018579	8.10E+01	2.00E-04	TRUE
1	TFGAGAALQ	869	877	9	HLA-A*02:01	869	81	0.0001857	8.10E+01	2.00E-04	TRUE
1	TTGIGYQPY	486	494	9	HLA-A*02:01	486	81	0.00017797	8.10E+01	2.00E-04	TRUE
1	TPGTNASSE	585	593	9	HLA-A*02:01	585	81	0.00017574	8.10E+01	2.00E-04	TRUE
1	VFAQVKQMY	763	771	9	HLA-A*02:01	763	81	0.0001748	8.10E+01	2.00E-04	TRUE
1	SYECDIPIG	645	653	9	HLA-A*02:01	645	82	0.00017265	8.20E+01	2.00E-04	TRUE
1	DSVRDPKTS	560	568	9	HLA-A*02:01	560	82	0.00017214	8.20E+01	2.00E-04	TRUE
1	FHTINHTFG	69	77	9	HLA-A*02:01	69	82	0.00017201	8.20E+01	2.00E-04	TRUE
1	IGIINNTVY	1112	1120	9	HLA-A*02:01	1112	82	0.00017089	8.20E+01	2.00E-04	TRUE
1	NNSTNVVIR	118	126	9	HLA-A*02:01	118	82	0.00016828	8.20E+01	2.00E-04	TRUE
1	TFMLKYDEN	261	269	9	HLA-A*02:01	261	82	0.00016801	8.20E+01	2.00E-04	TRUE
1	SSKRFQPFQ	541	549	9	HLA-A*02:01	541	83	0.00016034	8.30E+01	2.00E-04	TRUE
1	DVNCTDVST	600	608	9	HLA-A*02:01	600	83	0.00015876	8.30E+01	2.00E-04	TRUE
1	ECANLLLQY	730	738	9	HLA-A*02:01	730	83	0.00015532	8.30E+01	2.00E-04	TRUE
1	VNFNFNGLT	525	533	9	HLA-A*02:01	525	83	0.00015326	8.30E+01	2.00E-04	TRUE
1	LYENQKQIA	898	906	9	HLA-A*02:01	898	83	0.00015271	8.30E+01	2.00E-04	TRUE
1	CSCGSCCKF	1230	1238	9	HLA-A*02:01	1230	83	0.0001526	8.30E+01	2.00E-04	TRUE
1	PDPLKPTKR	789	797	9	HLA-A*02:01	789	83	0.00015244	8.30E+01	2.00E-04	TRUE
1	GAEHVDTSY	638	646	9	HLA-A*02:01	638	83	0.00014558	8.30E+01	1.00E-04	TRUE
1	IFKLPLGIN	219	227	9	HLA-A*02:01	219	83	0.00014557	8.30E+01	1.00E-04	TRUE
1	QTSNFRVVP	301	309	9	HLA-A*02:01	301	83	0.00014533	8.30E+01	1.00E-04	TRUE
1	CYGVSATKL	366	374	9	HLA-A*02:01	366	83	0.00014489	8.30E+01	1.00E-04	TRUE
1	TPPALNCYW	468	476	9	HLA-A*02:01	468	83	0.0001436	8.30E+01	1.00E-04	TRUE
1	YIKWPWYVW	1191	1199	9	HLA-A*02:01	1191	84	0.00014297	8.40E+01	1.00E-04	TRUE
1	AAEQDRNTR	753	761	9	HLA-A*02:01	753	84	0.00014269	8.40E+01	1.00E-04	TRUE
1	LPSGFNTLK	209	217	9	HLA-A*02:01	209	84	0.00014249	8.40E+01	1.00E-04	TRUE
1	YENQKQIAN	899	907	9	HLA-A*02:01	899	84	0.00014239	8.40E+01	1.00E-04	TRUE
1	PQIITTDNT	1094	1102	9	HLA-A*02:01	1094	84	0.00014187	8.40E+01	1.00E-04	TRUE
1	VNQNAQALN	934	942	9	HLA-A*02:01	934	84	0.00014183	8.40E+01	1.00E-04	TRUE
1	DLDRCTTFD	15	23	9	HLA-A*02:01	15	84	0.0001413	8.40E+01	1.00E-04	TRUE
1	PNYTQHTSS	28	36	9	HLA-A*02:01	28	84	0.00014076	8.40E+01	1.00E-04	TRUE
1	PFQQFGRDV	547	555	9	HLA-A*02:01	547	84	0.00013963	8.40E+01	1.00E-04	TRUE
1	ELKCSVKSF	285	293	9	HLA-A*02:01	285	84	0.00013936	8.40E+01	1.00E-04	TRUE
1	VPFSPDGKP	458	466	9	HLA-A*02:01	458	84	0.00013867	8.40E+01	1.00E-04	TRUE
1	VPSQERNFT	1050	1058	9	HLA-A*02:01	1050	84	0.00013668	8.40E+01	1.00E-04	TRUE
1	HTSSMRGVY	33	41	9	HLA-A*02:01	33	84	0.00013476	8.40E+01	1.00E-04	TRUE
1	NQKQIANQF	901	909	9	HLA-A*02:01	901	84	0.00013422	8.40E+01	1.00E-04	TRUE
1	KWPWYVWLG	1193	1201	9	HLA-A*02:01	1193	84	0.0001339	8.40E+01	1.00E-04	TRUE
1	TDNTFVSGN	1099	1107	9	HLA-A*02:01	1099	84	0.00013218	8.40E+01	1.00E-04	TRUE

seq #	peptide	start	end	peptide length	allele	peptide index	median binding	mhcnp score	mhcnp percent	legacy score	scores equal
1	NFRAILTAF	230	238	9	HLA-A*02:01	230	84	0.000132	8.40E+01	1.00E-04	TRUE
1	KYDENGTIT	265	273	9	HLA-A*02:01	265	84	0.00013182	8.40E+01	1.00E-04	TRUE
1	NDLCFSNVY	375	383	9	HLA-A*02:01	375	84	0.00013164	8.40E+01	1.00E-04	TRUE
1	QDVVNQNAQ	931	939	9	HLA-A*02:01	931	84	0.0001314	8.40E+01	1.00E-04	TRUE
1	REVFAQVKQ	761	769	9	HLA-A*02:01	761	84	0.00012965	8.40E+01	1.00E-04	TRUE
1	CSVKSFEID	288	296	9	HLA-A*02:01	288	84	0.00012928	8.40E+01	1.00E-04	TRUE
1	QPELDSFKE	1124	1132	9	HLA-A*02:01	1124	84	0.00012876	8.40E+01	1.00E-04	TRUE
1	TDAVDCSQN	273	281	9	HLA-A*02:01	273	85	0.00012597	8.50E+01	1.00E-04	TRUE
1	FYSNVTGFH	62	70	9	HLA-A*02:01	62	85	0.00012582	8.50E+01	1.00E-04	TRUE
1	DVVIGIINN	1109	1117	9	HLA-A*02:01	1109	85	0.00012555	8.50E+01	1.00E-04	TRUE
1	NYKLPDDFM	409	417	9	HLA-A*02:01	409	85	0.00012457	8.50E+01	1.00E-04	TRUE
1	TEKSNVVRG	92	100	9	HLA-A*02:01	92	85	0.00012432	8.50E+01	1.00E-04	TRUE
1	QNVLYENQK	895	903	9	HLA-A*02:01	895	85	0.00012345	8.50E+01	1.00E-04	TRUE
1	LDKYFKNHT	1134	1142	9	HLA-A*02:01	1134	85	0.00012323	8.50E+01	1.00E-04	TRUE
1	SKPMGTQTH	141	149	9	HLA-A*02:01	141	85	0.00012238	8.50E+01	1.00E-04	TRUE
1	NTRNIDATS	424	432	9	HLA-A*02:01	424	85	0.00012004	8.50E+01	1.00E-04	TRUE
1	VFGSTMNNK	102	110	9	HLA-A*02:01	102	85	0.00011974	8.50E+01	1.00E-04	TRUE
1	TGVIADYNY	402	410	9	HLA-A*02:01	402	85	0.00011958	8.50E+01	1.00E-04	TRUE
1	DRLNEVAKN	1166	1174	9	HLA-A*02:01	1166	85	0.00011795	8.50E+01	1.00E-04	TRUE
1	QELGKYEQY	1183	1191	9	HLA-A*02:01	1183	85	0.00011577	8.50E+01	1.00E-04	TRUE
1	DDFMGCVLA	414	422	9	HLA-A*02:01	414	85	0.00011573	8.50E+01	1.00E-04	TRUE
1	NAFNCTFEY	155	163	9	HLA-A*02:01	155	85	0.00011553	8.50E+01	1.00E-04	TRUE
1	QSKRVDFCG	1018	1026	9	HLA-A*02:01	1018	85	0.00011491	8.50E+01	1.00E-04	TRUE
1	WERKKISNC	340	348	9	HLA-A*02:01	340	86	0.00011442	8.60E+01	1.00E-04	TRUE
1	TECANLLLQ	729	737	9	HLA-A*02:01	729	86	0.00011431	8.60E+01	1.00E-04	TRUE
1	VTILLCCMT	1212	1220	9	HLA-A*02:01	1212	86	0.00011345	8.60E+01	1.00E-04	TRUE
1	CGSCCKFDE	1232	1240	9	HLA-A*02:01	1232	86	0.00011332	8.60E+01	1.00E-04	TRUE
1	RSDTLYLTQ	48	56	9	HLA-A*02:01	48	86	0.00011311	8.60E+01	1.00E-04	TRUE
1	FNGTSWFIT	1079	1087	9	HLA-A*02:01	1079	86	0.00010805	8.60E+01	1.00E-04	TRUE
1	DLICAQKFN	830	838	9	HLA-A*02:01	830	86	0.00010768	8.60E+01	1.00E-04	TRUE
1	VYAWERKKI	337	345	9	HLA-A*02:01	337	86	0.00010687	8.60E+01	1.00E-04	TRUE
1	MKQYGECLG	816	824	9	HLA-A*02:01	816	86	0.00010685	8.60E+01	1.00E-04	TRUE
1	NDILSRLDK	960	968	9	HLA-A*02:01	960	86	0.00010642	8.60E+01	1.00E-04	TRUE
1	YRVVVLSFE	494	502	9	HLA-A*02:01	494	86	0.00010622	8.60E+01	1.00E-04	TRUE
1	TPSSKRFQP	539	547	9	HLA-A*02:01	539	86	0.00010592	8.60E+01	1.00E-04	TRUE
1	THTMIFDNA	148	156	9	HLA-A*02:01	148	86	0.00010534	8.60E+01	1.00E-04	TRUE
1	YNYKYRYLR	436	444	9	HLA-A*02:01	436	86	0.00010379	8.60E+01	1.00E-04	TRUE
1	RKKISNCVA	342	350	9	HLA-A*02:01	342	86	0.00010347	8.60E+01	1.00E-04	TRUE
1	LTPSSKRFQ	538	546	9	HLA-A*02:01	538	86	0.00010215	8.60E+01	1.00E-04	TRUE
1	CYWPLNDYG	474	482	9	HLA-A*02:01	474	87	9.78E-05	8.70E+01	1.00E-04	TRUE
1	EIFRSDTLY	45	53	9	HLA-A*02:01	45	87	9.74E-05	8.70E+01	1.00E-04	TRUE
1	GAGICASYH	653	661	9	HLA-A*02:01	653	87	9.65E-05	8.70E+01	1.00E-04	TRUE
1	IYSTGNNVF	621	629	9	HLA-A*02:01	621	87	9.58E-05	8.70E+01	1.00E-04	TRUE
1	NPFFAVSKP	135	143	9	HLA-A*02:01	135	87	9.48E-05	8.70E+01	1.00E-04	TRUE
1	DLFLPFYSN	57	65	9	HLA-A*02:01	57	87	9.09E-05	8.70E+01	1.00E-04	TRUE
1	SCCKFDEDD	1234	1242	9	HLA-A*02:01	1234	87	9.06E-05	8.70E+01	1.00E-04	TRUE
1	ACSCGSCCK	1229	1237	9	HLA-A*02:01	1229	87	8.75E-05	8.70E+01	1.00E-04	TRUE
1	DLIKNQCVN	518	526	9	HLA-A*02:01	518	88	8.71E-05	8.80E+01	1.00E-04	TRUE
1	QDVNCTDVS	599	607	9	HLA-A*02:01	599	88	8.70E-05	8.80E+01	1.00E-04	TRUE
1	SYHTVSLLR	659	667	9	HLA-A*02:01	659	88	8.38E-05	8.80E+01	1.00E-04	TRUE
1	VDCNMYICG	718	726	9	HLA-A*02:01	718	88	8.30E-05	8.80E+01	1.00E-04	TRUE
1	LKPTTFMLK	257	265	9	HLA-A*02:01	257	88	8.12E-05	8.80E+01	1.00E-04	TRUE
1	QDRNTREVF	756	764	9	HLA-A*02:01	756	88	8.11E-05	8.80E+01	1.00E-04	TRUE
1	NGTSWFITQ	1080	1088	9	HLA-A*02:01	1080	88	8.07E-05	8.80E+01	1.00E-04	TRUE
1	PTLKYFGGF	774	782	9	HLA-A*02:01	774	88	7.89E-05	8.80E+01	1.00E-04	TRUE
1	YWPLNDYGF	475	483	9	HLA-A*02:01	475	88	7.81E-05	8.80E+01	1.00E-04	TRUE
1	GEVFNATKF	326	334	9	HLA-A*02:01	326	88	7.80E-05	8.80E+01	1.00E-04	TRUE
1	TGNNVFQTQ	624	632	9	HLA-A*02:01	624	88	7.70E-05	8.80E+01	1.00E-04	TRUE
1	FYTTTGIGY	483	491	9	HLA-A*02:01	483	88	7.70E-05	8.80E+01	1.00E-04	TRUE
1	PDDFMGCVL	413	421	9	HLA-A*02:01	413	89	7.62E-05	8.90E+01	1.00E-04	TRUE
1	EDLLFNKVT	801	809	9	HLA-A*02:01	801	89	7.61E-05	8.90E+01	1.00E-04	TRUE
1	MYICGDSTE	722	730	9	HLA-A*02:01	722	89	7.58E-05	8.90E+01	1.00E-04	TRUE
1	TQNVLYENQ	894	902	9	HLA-A*02:01	894	89	7.43E-05	8.90E+01	1.00E-04	TRUE
1	LYQDVNCTD	597	605	9	HLA-A*02:01	597	89	7.34E-05	8.90E+01	1.00E-04	TRUE
1	DNTFVSGNC	1100	1108	9	HLA-A*02:01	1100	89	7.13E-05	8.90E+01	1.00E-04	TRUE
1	LPPLLTDDM	843	851	9	HLA-A*02:01	843	89	7.10E-05	8.90E+01	1.00E-04	TRUE
1	FNCTFEYIS	157	165	9	HLA-A*02:01	157	89	7.09E-05	8.90E+01	1.00E-04	TRUE
1	LYNSTFFST	355	363	9	HLA-A*02:01	355	89	0.00007077	8.90E+01	1.00E-04	TRUE
1	DAVDCSQNP	274	282	9	HLA-A*02:01	274	89	7.02E-05	8.90E+01	1.00E-04	TRUE
1	TQRNFFSPQ	1087	1095	9	HLA-A*02:01	1087	89	6.79E-05	8.90E+01	1.00E-04	TRUE
1	NPVIPFKDG	78	86	9	HLA-A*02:01	78	89	6.77E-05	8.90E+01	1.00E-04	TRUE
1	PSGDVVRFP	309	317	9	HLA-A*02:01	309	90	6.45E-05	9.00E+01	1.00E-04	TRUE
1	SEPVLKGVK	1243	1251	9	HLA-A*02:01	1243	90	6.38E-05	9.00E+01	1.00E-04	TRUE
1	EVQIDRLIT	972	980	9	HLA-A*02:01	972	90	6.36E-05	9.00E+01	1.00E-04	TRUE
1	IDKGIYQTS	295	303	9	HLA-A*02:01	295	90	6.26E-05	9.00E+01	1.00E-04	TRUE
1	IPFAMQMAY	878	886	9	HLA-A*02:01	878	90	6.19E-05	9.00E+01	1.00E-04	TRUE
1	GNYNYKYRY	434	442	9	HLA-A*02:01	434	90	6.15E-05	9.00E+01	1.00E-04	TRUE
1	TYVPSQERN	1048	1056	9	HLA-A*02:01	1048	90	6.06E-05	9.00E+01	1.00E-04	TRUE
1	TPTLKYFGG	773	781	9	HLA-A*02:01	773	90	6.00E-05	9.00E+01	1.00E-04	TRUE
1	YEQYIKWPW	1188	1196	9	HLA-A*02:01	1188	90	5.93E-05	9.00E+01	1.00E-04	TRUE

seq #	peptide	start	end	peptide length	allele	peptide index	median binding	mhcnp score	mhcnp percent	legacy score	scores equal
1	YNSTFFSTF	356	364	9	HLA-A*02:01	356	90	5.86E-05	9.00E+01	1.00E-04	TRUE
1	REFVFKNKD	183	191	9	HLA-A*02:01	183	90	5.79E-05	9.00E+01	1.00E-04	TRUE
1	WYVWLGFIA	1196	1204	9	HLA-A*02:01	1196	90	5.77E-05	9.00E+01	1.00E-04	TRUE
1	ACNFELCDN	127	135	9	HLA-A*02:01	127	90	5.76E-05	9.00E+01	1.00E-04	TRUE
1	STFKCYGVS	362	370	9	HLA-A*02:01	362	90	5.69E-05	9.00E+01	1.00E-04	TRUE
1	LGKYEQYIK	1185	1193	9	HLA-A*02:01	1185	90	5.64E-05	9.00E+01	1.00E-04	TRUE
1	PELDSFKEE	1125	1133	9	HLA-A*02:01	1125	90	5.52E-05	9.00E+01	1.00E-04	TRUE
1	PSGFNTLKP	210	218	9	HLA-A*02:01	210	90	5.52E-05	9.00E+01	1.00E-04	TRUE
1	PDGKPCTPP	462	470	9	HLA-A*02:01	462	90	5.51E-05	9.00E+01	1.00E-04	TRUE
1	PFAMQMAYR	879	887	9	HLA-A*02:01	879	90	5.48E-05	9.00E+01	1.00E-04	TRUE
1	EGKAYFPRE	1066	1074	9	HLA-A*02:01	1066	91	5.48E-05	9.10E+01	1.00E-04	TRUE
1	YGECLGDIN	819	827	9	HLA-A*02:01	819	91	5.45E-05	9.10E+01	1.00E-04	TRUE
1	KTSVDCNMY	715	723	9	HLA-A*02:01	715	91	5.44E-05	9.10E+01	1.00E-04	TRUE
1	GYHLMSFPQ	1028	1036	9	HLA-A*02:01	1028	91	5.36E-05	9.10E+01	1.00E-04	TRUE
1	QTHTMIFDN	147	155	9	HLA-A*02:01	147	91	5.33E-05	9.10E+01	1.00E-04	TRUE
1	PAQDIWGTS	240	248	9	HLA-A*02:01	240	91	5.32E-05	9.10E+01	1.00E-04	TRUE
1	PAWRIYSTG	617	625	9	HLA-A*02:01	617	91	5.26E-05	9.10E+01	1.00E-04	TRUE
1	IKNQCVNFN	520	528	9	HLA-A*02:01	520	91	5.25E-05	9.10E+01	1.00E-04	TRUE
1	ELDKYFKNH	1133	1141	9	HLA-A*02:01	1133	91	5.09E-05	9.10E+01	1.00E-04	TRUE
1	GSTMNNKSQ	104	112	9	HLA-A*02:01	104	91	5.06E-05	9.10E+01	1.00E-04	TRUE
1	QERNFTTAP	1053	1061	9	HLA-A*02:01	1053	91	4.92E-05	9.10E+01	0.00E+00	TRUE
1	VYADSFVVK	382	390	9	HLA-A*02:01	382	91	4.92E-05	9.10E+01	0.00E+00	TRUE
1	PSSKRFQPF	540	548	9	HLA-A*02:01	540	91	4.91E-05	9.10E+01	0.00E+00	TRUE
1	TNVVIRACN	121	129	9	HLA-A*02:01	121	91	4.89E-05	9.10E+01	0.00E+00	TRUE
1	DKYFKNHTS	1135	1143	9	HLA-A*02:01	1135	91	4.80E-05	9.10E+01	0.00E+00	TRUE
1	DPLKPTKRS	790	798	9	HLA-A*02:01	790	91	4.78E-05	9.10E+01	0.00E+00	TRUE
1	MPVSMAKTS	709	717	9	HLA-A*02:01	709	92	4.50E-05	9.20E+01	0.00E+00	TRUE
1	PTNFSISIT	697	705	9	HLA-A*02:01	697	92	4.43E-05	9.20E+01	0.00E+00	TRUE
1	LYVYKGYQP	194	202	9	HLA-A*02:01	194	92	4.42E-05	9.20E+01	0.00E+00	TRUE
1	RYLRHGKLR	441	449	9	HLA-A*02:01	441	92	4.41E-05	9.20E+01	0.00E+00	TRUE
1	SWFITQRNF	1083	1091	9	HLA-A*02:01	1083	92	4.40E-05	9.20E+01	0.00E+00	TRUE
1	ENQKQIANQ	900	908	9	HLA-A*02:01	900	92	4.38E-05	9.20E+01	0.00E+00	TRUE
1	KYFKNHTSP	1136	1144	9	HLA-A*02:01	1136	92	4.36E-05	9.20E+01	0.00E+00	TRUE
1	SCGSCCKFD	1231	1239	9	HLA-A*02:01	1231	92	4.29E-05	9.20E+01	0.00E+00	TRUE
1	LNCYWPLND	472	480	9	HLA-A*02:01	472	92	4.23E-05	9.20E+01	0.00E+00	TRUE
1	PNITNLCPF	317	325	9	HLA-A*02:01	317	92	4.14E-05	9.20E+01	0.00E+00	TRUE
1	TSTGNYNYK	431	439	9	HLA-A*02:01	431	92	4.14E-05	9.20E+01	0.00E+00	TRUE
1	NTFVSGNCD	1101	1109	9	HLA-A*02:01	1101	92	4.08E-05	9.20E+01	0.00E+00	TRUE
1	CNFELCDNP	128	136	9	HLA-A*02:01	128	92	0.00004047	9.20E+01	0.00E+00	TRUE
1	AYFVGYLKP	251	259	9	HLA-A*02:01	251	92	4.04E-05	9.20E+01	0.00E+00	TRUE
1	DFMGCVLAW	415	423	9	HLA-A*02:01	415	92	3.99E-05	9.20E+01	0.00E+00	TRUE
1	TYVTQQLIR	988	996	9	HLA-A*02:01	988	92	3.95E-05	9.20E+01	0.00E+00	TRUE
1	KPTTFMLKY	258	266	9	HLA-A*02:01	258	92	3.86E-05	9.20E+01	0.00E+00	TRUE
1	CHEGKAYFP	1064	1072	9	HLA-A*02:01	1064	92	3.86E-05	9.20E+01	0.00E+00	TRUE
1	CNMYICGDS	720	728	9	HLA-A*02:01	720	93	3.81E-05	9.30E+01	0.00E+00	TRUE
1	QKQIANQFN	902	910	9	HLA-A*02:01	902	93	3.78E-05	9.30E+01	0.00E+00	TRUE
1	IWGTSAAAY	244	252	9	HLA-A*02:01	244	93	3.48E-05	9.30E+01	0.00E+00	TRUE
1	PPLLTDDMI	844	852	9	HLA-A*02:01	844	93	3.46E-05	9.30E+01	0.00E+00	TRUE
1	CDVVIGIIN	1108	1116	9	HLA-A*02:01	1108	93	3.40E-05	9.30E+01	0.00E+00	TRUE
1	DPLQPELDS	1121	1129	9	HLA-A*02:01	1121	93	3.34E-05	9.30E+01	0.00E+00	TRUE
1	WRIYSTGNN	619	627	9	HLA-A*02:01	619	93	3.34E-05	9.30E+01	0.00E+00	TRUE
1	SEKSGNFKH	173	181	9	HLA-A*02:01	173	93	3.33E-05	9.30E+01	0.00E+00	TRUE
1	DAGFMKQYG	812	820	9	HLA-A*02:01	812	93	3.23E-05	9.30E+01	0.00E+00	TRUE
1	NYNYKYRYL	435	443	9	HLA-A*02:01	435	94	2.99E-05	9.40E+01	0.00E+00	TRUE
1	CFSNVYADS	378	386	9	HLA-A*02:01	378	94	2.94E-05	9.40E+01	0.00E+00	TRUE
1	SFEIDKGIY	292	300	9	HLA-A*02:01	292	94	2.89E-05	9.40E+01	0.00E+00	TRUE
1	GCVLAWNTR	418	426	9	HLA-A*02:01	418	94	2.87E-05	9.40E+01	0.00E+00	TRUE
1	PKLSTDLIK	513	521	9	HLA-A*02:01	513	94	2.83E-05	9.40E+01	0.00E+00	TRUE
1	DNPFFAVSK	134	142	9	HLA-A*02:01	134	94	2.70E-05	9.40E+01	0.00E+00	TRUE
1	NQCVNFNFN	522	530	9	HLA-A*02:01	522	94	2.56E-05	9.40E+01	0.00E+00	TRUE
1	DSSIAYSNN	684	692	9	HLA-A*02:01	684	95	2.52E-05	9.50E+01	0.00E+00	TRUE
1	LCCMTSCCS	1216	1224	9	HLA-A*02:01	1216	95	2.50E-05	9.50E+01	0.00E+00	TRUE
1	EVFNATKFP	327	335	9	HLA-A*02:01	327	95	2.48E-05	9.50E+01	0.00E+00	TRUE
1	MYKTPTLKY	770	778	9	HLA-A*02:01	770	95	2.44E-05	9.50E+01	0.00E+00	TRUE
1	VFVFNGTSW	1076	1084	9	HLA-A*02:01	1076	95	2.41E-05	9.50E+01	0.00E+00	TRUE
1	DSFVVKGDD	385	393	9	HLA-A*02:01	385	95	2.39E-05	9.50E+01	0.00E+00	TRUE
1	HEGKAYFPR	1065	1073	9	HLA-A*02:01	1065	95	2.32E-05	9.50E+01	0.00E+00	TRUE
1	DATSTGNYN	429	437	9	HLA-A*02:01	429	95	2.29E-05	9.50E+01	0.00E+00	TRUE
1	RCTTFDDVQ	18	26	9	HLA-A*02:01	18	95	2.23E-05	9.50E+01	0.00E+00	TRUE
1	PWYVWLGFI	1195	1203	9	HLA-A*02:01	1195	95	2.20E-05	9.50E+01	0.00E+00	TRUE
1	QPFQQFGRD	546	554	9	HLA-A*02:01	546	95	2.17E-05	9.50E+01	0.00E+00	TRUE
1	GFLYVYKGY	192	200	9	HLA-A*02:01	192	95	2.07E-05	9.50E+01	0.00E+00	TRUE
1	MTSCCSCLK	1219	1227	9	HLA-A*02:01	1219	96	2.01E-05	9.60E+01	0.00E+00	TRUE
1	EELDKYFKN	1132	1140	9	HLA-A*02:01	1132	96	1.93E-05	9.60E+01	0.00E+00	TRUE
1	VRGWVFGST	98	106	9	HLA-A*02:01	98	96	1.89E-05	9.60E+01	0.00E+00	TRUE
1	MRGVYYPDE	37	45	9	HLA-A*02:01	37	96	1.82E-05	9.60E+01	0.00E+00	TRUE
1	TFFSTFKCY	359	367	9	HLA-A*02:01	359	96	1.80E-05	9.60E+01	0.00E+00	TRUE
1	EQYIKWPWY	1189	1197	9	HLA-A*02:01	1189	96	1.79E-05	9.60E+01	0.00E+00	TRUE
1	KEELDKYFK	1131	1139	9	HLA-A*02:01	1131	96	1.79E-05	9.60E+01	0.00E+00	TRUE
1	DNAFNCTFE	154	162	9	HLA-A*02:01	154	96	1.77E-05	9.60E+01	0.00E+00	TRUE

seq #	peptide	start	end	peptide length	allele	peptide index	median binding	mhcnp score	mhcnp percent	legacy score	scores equal
1	SKRVDFCGK	1019	1027	9	HLA-A*02:01	1019	96	1.72E-05	9.60E+01	0.00E+00	TRUE
1	DSFKEELDK	1128	1136	9	HLA-A*02:01	1128	96	1.69E-05	9.60E+01	0.00E+00	TRUE
1	LDRCTTFDD	16	24	9	HLA-A*02:01	16	96	1.69E-05	9.60E+01	0.00E+00	TRUE
1	QVKQMYKTP	766	774	9	HLA-A*02:01	766	96	1.65E-05	9.60E+01	0.00E+00	TRUE
1	PSVYAWERK	335	343	9	HLA-A*02:01	335	96	1.63E-05	9.60E+01	0.00E+00	TRUE
1	AYTMSLGAD	676	684	9	HLA-A*02:01	676	96	1.62E-05	9.60E+01	0.00E+00	TRUE
1	EGVFVFNGT	1074	1082	9	HLA-A*02:01	1074	96	1.55E-05	9.60E+01	0.00E+00	TRUE
1	NCYWPLNDY	473	481	9	HLA-A*02:01	473	96	1.54E-05	9.60E+01	0.00E+00	TRUE
1	DYGFYTTTG	480	488	9	HLA-A*02:01	480	97	1.44E-05	9.70E+01	0.00E+00	TRUE
1	SPQIITTDN	1093	1101	9	HLA-A*02:01	1093	97	1.37E-05	9.70E+01	0.00E+00	TRUE
1	DRLITGRLQ	976	984	9	HLA-A*02:01	976	97	1.36E-05	9.70E+01	0.00E+00	TRUE
1	NSTFFSTFK	357	365	9	HLA-A*02:01	357	97	1.32E-05	9.70E+01	0.00E+00	TRUE
1	WPLNDYGFY	476	484	9	HLA-A*02:01	476	97	1.30E-05	9.70E+01	0.00E+00	TRUE
1	TFEYISDAF	160	168	9	HLA-A*02:01	160	97	1.28E-05	9.70E+01	0.00E+00	TRUE
1	PTKRSFIED	794	802	9	HLA-A*02:01	794	97	1.22E-05	9.70E+01	0.00E+00	TRUE
1	AWRIYSTGN	618	626	9	HLA-A*02:01	618	97	1.20E-05	9.70E+01	0.00E+00	TRUE
1	NFELCDNPF	129	137	9	HLA-A*02:01	129	97	1.19E-05	9.70E+01	0.00E+00	TRUE
1	LREFVFKNK	182	190	9	HLA-A*02:01	182	97	1.19E-05	9.70E+01	0.00E+00	TRUE
1	AWERKKISN	339	347	9	HLA-A*02:01	339	97	1.18E-05	9.70E+01	0.00E+00	TRUE
1	EFVFKNKDG	184	192	9	HLA-A*02:01	184	97	1.11E-05	9.70E+01	0.00E+00	TRUE
1	TGNYNYKYR	433	441	9	HLA-A*02:01	433	97	1.11E-05	9.70E+01	0.00E+00	TRUE
1	VYKGYQPID	196	204	9	HLA-A*02:01	196	97	1.08E-05	97	0	TRUE
1	GWVFGSTMN	100	108	9	HLA-A*02:01	100	97	1.02E-05	97	0	TRUE
1	DCNMYICGD	719	727	9	HLA-A*02:01	719	97	9.92E-06	97	0	TRUE
1	DFTDSVRDP	557	565	9	HLA-A*02:01	557	97	9.74E-06	97	0	TRUE
1	PDEIFRSDT	43	51	9	HLA-A*02:01	43	97	9.71E-06	97	0	TRUE
1	DPKTSEILD	564	572	9	HLA-A*02:01	564	98	8.80E-06	98	0	TRUE
1	TREVFAQVK	760	768	9	HLA-A*02:01	760	98	8.56E-06	98	0	TRUE
1	DYNYKLPDD	407	415	9	HLA-A*02:01	407	98	8.20E-06	98	0	TRUE
1	NCTFEYISD	158	166	9	HLA-A*02:01	158	98	7.94E-06	98	0	TRUE
1	CCKFDEDDS	1235	1243	9	HLA-A*02:01	1235	98	7.39E-06	98	0	TRUE
1	SECVLGQSK	1012	1020	9	HLA-A*02:01	1012	98	7.03E-06	98	0	TRUE
1	EKSNVVRGW	93	101	9	HLA-A*02:01	93	98	6.82E-06	98	0	TRUE
1	WPWYVWLGF	1194	1202	9	HLA-A*02:01	1194	98	6.80E-06	98	0	TRUE
1	KYEQYIKWP	1187	1195	9	HLA-A*02:01	1187	98	6.55E-06	98	0	TRUE
1	DRCTTFDDV	17	25	9	HLA-A*02:01	17	99	6.17E-06	99	0	TRUE
1	ECVLGQSKR	1013	1021	9	HLA-A*02:01	1013	99	6.07E-06	99	0	TRUE
1	ERKKISNCV	341	349	9	HLA-A*02:01	341	99	5.54E-06	99	0	TRUE
1	DTSYECDIP	643	651	9	HLA-A*02:01	643	99	5.06E-06	99	0	TRUE
1	PTTFMLKYD	259	267	9	HLA-A*02:01	259	99	4.88E-06	99	0	TRUE
1	DKGIYQTSN	296	304	9	HLA-A*02:01	296	99	3.87E-06	99	0	TRUE
1	KYRYLRHGK	439	447	9	HLA-A*02:01	439	100	2.11E-06	100	0	TRUE
1	PREGVFVFN	1072	1080	9	HLA-A*02:01	1072	100	1.51E-06	100	0	TRUE
1	PPALNCYWP	469	477	9	HLA-A*02:01	469	100	1.44E-06	100	0	TRUE
1	PYRVVVLSF	493	501	9	HLA-A*02:01	493	100	1.44E-06	100	0	TRUE
1	DYSVLYNST	351	359	9	HLA-A*02:01	351	100	1.26E-06	100	0	TRUE
1	NYKYRYLRH	437	445	9	HLA-A*02:01	437	100	1.37E-07	100	0	TRUE

seq #	peptide	start	end	peptide length	allele	peptide index	immunogenicit	legacy score	**scores equal**
1	FIAGLIAIV	1	9	9	HLA-A*02:01	1	0.27206	0.27206	TRUE
4	KAVYNFATC	1	9	9	HLA-A*02:01	4	0.16928	0.16928	TRUE
3	RLNEVAKNL	1	9	9	HLA-A*02:01	3	-0.0101	-0.0101	TRUE
2	LITGRLQSL	1	9	9	HLA-A*02:01	2	-0.10776	-0.10776	TRUE
5	FQPQNGQFI	1	9	9	HLA-A*02:01	5	-0.12392	-0.12392	TRUE

seq #	peptide	start	end	peptide length	allele	peptide index	median binding	ann IC50	ann percentile	proteasome sc	tap score	mhc score	processing sco	processing tota	legacy Proteas	legacy TAP Sco	legacy MHC Sc	legacy Process	legacy Total Sc	legacy MHC IC	equal Proteaso	equal TAP Scor	equal MHC Sco	equal Processi	equal Total Sc	equal MHC IC50
1	MIAAYTAAL	851	859	9	HLA-A*02:01	851	0.13	14.39	0.13	1.363311	0.439621452	-1.1580608	1.802932452	0.644871658	1.36	0.44	-1.16	1.8	0.64	14.4	TRUE	TRUE	TRUE	TRUE	TRUE	TRUE
1	YLKPTTFML	256	264	9	HLA-A*02:01	256	0.17	18.56	0.17	1.493115	0.40251791	-1.268578	1.89563291	0.627054943	1.49	0.4	-1.27	1.9	0.63	18.6	TRUE	TRUE	TRUE	TRUE	TRUE	TRUE
1	VIADYNYKL	404	412	9	HLA-A*02:01	404	0.46	50.27	0.46	1.679705	0.493308915	-1.7013089	2.173013915	0.471705029	1.68	0.49	-1.7	2.17	0.47	50.3	TRUE	TRUE	TRUE	TRUE	TRUE	TRUE
1	SIVAYTMSL	673	681	9	HLA-A*02:01	673	0.33	37.41	0.33	1.470263	0.57239272	-1.5729877	2.04265572	0.46966801	1.47	0.57	-1.57	2.04	0.47	37.4	TRUE	TRUE	TRUE	TRUE	TRUE	TRUE
1	KQMYKTPTL	768	776	9	HLA-A*02:01	768	0.43	47.7	0.43	1.594796	0.54361832	-1.6785184	2.13841432	0.459895937	1.59	0.54	-1.68	2.14	0.46	47.7	TRUE	TRUE	TRUE	TRUE	TRUE	TRUE
1	FIFLLFLTL	2	10	9	HLA-A*02:01	2	0.66	71.52	0.66	1.648042	0.49564342	-1.8544275	2.143685417	0.28925791	1.65	0.5	-1.85	2.14	0.29	71.5	TRUE	TRUE	TRUE	TRUE	TRUE	TRUE
1	FIAGLIAIV	1202	1210	9	HLA-A*02:01	1202	0.09	10.29	0.09	1.092875	0.14424436	-1.0124154	1.237119357	0.224703982	1.09	0.14	-1.01	1.24	0.22	10.3	TRUE	TRUE	TRUE	TRUE	TRUE	TRUE
1	VLNDILSRL	958	966	9	HLA-A*02:01	958	0.3	33.57	0.3	1.290129	0.43472249	-1.5259513	1.724851492	0.19890015	1.29	0.43	-1.53	1.72	0.2	33.6	TRUE	TRUE	TRUE	TRUE	TRUE	TRUE
1	RLQSLQTYV	982	990	9	HLA-A*02:01	982	0.16	16.66	0.16	0.84733	0.22301424	-1.221675	1.070344242	-0.1513308	0.85	0.22	-1.22	1.07	-0.15	16.7	TRUE	TRUE	TRUE	TRUE	TRUE	TRUE
1	GLTVLPPLL	839	847	9	HLA-A*02:01	839	1.2	130.4	1.2	1.568412	0.39291672	-2.1152776	1.96132872	-0.1539489	1.57	0.39	-2.12	1.96	-0.15	130.4	TRUE	TRUE	TRUE	TRUE	TRUE	TRUE
1	FSISITTEV	700	708	9	HLA-A*02:01	700	0.27	30.13	0.27	1.030121	0.203216257	-1.4789991	1.233337257	-0.2456619	1.03	0.2	-1.48	1.23	-0.25	30.1	TRUE	TRUE	TRUE	TRUE	TRUE	TRUE
1	LLTDDMIAA	846	854	9	HLA-A*02:01	846	0.29	32.91	0.29	1.417234	-0.2994442	-1.5173279	1.117789759	-0.3995381	1.42	-0.3	-1.52	1.12	-0.4	32.9	TRUE	TRUE	TRUE	TRUE	TRUE	TRUE
1	RLDKVEAEV	965	973	9	HLA-A*02:01	965	0.35	38.95	0.35	0.981913	0.19715707	-1.5905075	1.17907007	-0.4114374	0.98	0.2	-1.59	1.18	-0.41	39	TRUE	TRUE	TRUE	TRUE	TRUE	TRUE
1	NVYADSFVV	381	389	9	HLA-A*02:01	381	0.77	87.86	0.77	1.135011	0.31636519	-1.9437912	1.451376192	-0.492415	1.14	0.32	-1.94	1.45	-0.49	87.9	TRUE	TRUE	TRUE	TRUE	TRUE	TRUE
1	VVFLHVTYV	1042	1050	9	HLA-A*02:01	1042	0.33	36.56	0.33	0.801063	0.25797296	-1.5630062	1.059035957	-0.5039702	0.8	0.26	-1.56	1.06	-0.5	36.6	TRUE	TRUE	TRUE	TRUE	TRUE	TRUE
1	NLNESLIDL	1174	1182	9	HLA-A*02:01	1174	1.4	177.32	1.4	1.241955	0.44457158	-2.2487577	1.686526577	-0.5622311	1.24	0.44	-2.25	1.69	-0.56	177.3	TRUE	TRUE	TRUE	TRUE	TRUE	TRUE
1	HLMSFPQAA	1030	1038	9	HLA-A*02:01	1030	0.28	30.89	0.28	1.144943	-0.2567852	-1.4898179	0.88815784	-0.6016601	1.14	-0.26	-1.49	0.89	-0.6	30.9	TRUE	TRUE	TRUE	TRUE	TRUE	TRUE
1	YVYKGYQPI	195	203	9	HLA-A*02:01	195	0.43	47.04	0.43	0.676692	0.3849362	-1.6724673	1.0616282	-0.6108391	0.68	0.38	-1.67	1.06	-0.61	47	TRUE	TRUE	TRUE	TRUE	TRUE	TRUE
1	LLFNKVTLA	803	811	9	HLA-A*02:01	803	0.22	25.41	0.22	1.024687	-0.2322824	-1.4050047	0.792404574	-0.6126001	1.02	-0.23	-1.41	0.79	-0.61	25.4	TRUE	TRUE	TRUE	TRUE	TRUE	TRUE
1	SLTTTSTAL	919	927	9	HLA-A*02:01	919	2	300.04	2	1.436632	0.35961596	-2.4771792	1.796247959	-0.6809312	1.44	0.36	-2.48	1.8	-0.68	300	TRUE	TRUE	TRUE	TRUE	TRUE	TRUE
1	FMKQYGECL	815	823	9	HLA-A*02:01	815	3.4	698.66	3.4	1.742981	0.41393001	-2.8442659	2.156911013	-0.6873549	1.74	0.41	-2.84	2.16	-0.69	698.7	TRUE	TRUE	TRUE	TRUE	TRUE	TRUE
1	YQPYRVVVL	491	499	9	HLA-A*02:01	491	3.9	862.1	3.9	1.79966	0.42096752	-2.9355576	2.22062752	-0.7149301	1.8	0.42	-2.94	2.22	-0.71	862.1	TRUE	TRUE	TRUE	TRUE	TRUE	TRUE
1	SITTEVMPV	703	711	9	HLA-A*02:01	703	0.46	50.81	0.46	0.782897	0.202119151	-1.7059492	0.985016151	-0.720933	0.78	0.2	-1.71	0.99	-0.72	50.8	TRUE	TRUE	TRUE	TRUE	TRUE	TRUE
1	VIRACNFEL	124	132	9	HLA-A*02:01	124	3.6	786.23	3.6	1.629979	0.540450236	-2.8955496	2.17042924	-0.7251204	1.63	0.54	-2.9	2.17	-0.73	786.2	TRUE	TRUE	TRUE	TRUE	TRUE	TRUE
1	RVVVLSFEL	495	503	9	HLA-A*02:01	495	2.5	419.83	2.5	1.303217	0.59394283	-2.6230735	1.89715983	-0.7259136	1.3	0.59	-2.62	1.9	-0.73	419.8	TRUE	TRUE	TRUE	TRUE	TRUE	TRUE
1	CMTSCCSCL	1218	1226	9	HLA-A*02:01	1218	3.2	629.65	3.2	1.476518	0.432188511	-2.7990992	1.90870651	-0.8903927	1.48	0.43	-2.8	1.91	-0.89	629.6	TRUE	TRUE	TRUE	TRUE	TRUE	TRUE
1	AIVMVTILL	1208	1216	9	HLA-A*02:01	1208	3.4	711.15	3.4	1.377933	0.572576816	-2.8519612	1.950509816	-0.9014514	1.38	0.57	-2.85	1.95	-0.9	711.1	TRUE	TRUE	TRUE	TRUE	TRUE	TRUE
1	VLYENQKQI	897	905	9	HLA-A*02:01	897	3	562.95	3	1.366109	0.40959613	-2.7504698	1.775705127	-0.9747647	1.37	0.41	-2.75	1.78	-0.97	563	TRUE	TRUE	TRUE	TRUE	TRUE	TRUE
1	RLNEVAKNL	1167	1175	9	HLA-A*02:01	1167	4.1	940.92	4.1	1.556191	0.430336302	-2.9735527	1.986527302	-0.9870254	1.56	0.43	-2.97	1.99	-0.99	940.9	TRUE	TRUE	TRUE	TRUE	TRUE	TRUE
1	FVFNGTSWF	1077	1085	9	HLA-A*02:01	1077	6.1	1869.65	6.1	1.087998	1.18780224	-3.2717603	2.275800238	-0.9959601	1.09	1.19	-3.27	2.28	-1	1869.7	TRUE	TRUE	TRUE	TRUE	TRUE	TRUE
1	GTSAAAYFV	246	254	9	HLA-A*02:01	246	1.4	167.91	1.4	1.191009	0.02173758	-2.2250766	1.21274658	-1.01233	1.19	0.02	-2.23	1.21	-1.01	167.9	TRUE	TRUE	TRUE	TRUE	TRUE	TRUE
1	IIINNSTNV	115	123	9	HLA-A*02:01	115	1.7	228.99	1.7	1.079656	0.25123866	-2.3598165	1.330894664	-1.0289219	1.08	0.25	-2.36	1.33	-1.03	229	TRUE	TRUE	TRUE	TRUE	TRUE	TRUE
1	ALVSGTATA	858	866	9	HLA-A*02:01	858	0.97	122.57	0.97	1.102763	-0.0592679	-2.0883842	1.04349507	-1.0448891	1.1	-0.06	-2.09	1.04	-1.04	122.6	TRUE	TRUE	TRUE	TRUE	TRUE	TRUE
1	ALNTLVKQL	940	948	9	HLA-A*02:01	940	5.7	1633.23	5.7	1.719339	0.425882815	-3.2130473	2.145221814	-1.0678255	1.72	0.43	-3.21	2.15	-1.07	1633.2	TRUE	TRUE	TRUE	TRUE	TRUE	TRUE
1	IAIPTNFSI	694	702	9	HLA-A*02:01	694	2.8	487.32	2.8	1.299289	0.29811003	-2.6878142	1.597399026	-1.0904152	1.3	0.3	-2.69	1.6	-1.09	487.3	TRUE	TRUE	TRUE	TRUE	TRUE	TRUE
1	LITGRLQSL	978	986	9	HLA-A*02:01	978	5.7	1675.27	5.7	1.579758	0.51589074	-3.2240848	2.095648738	-1.1284361	1.58	0.52	-3.22	2.1	-1.13	1675.3	TRUE	TRUE	TRUE	TRUE	TRUE	TRUE
1	FAMQMAYRF	880	888	9	HLA-A*02:01	880	11	4263.5	11	1.45013	1.046456357	-3.6297663	2.496586357	-1.1331799	1.45	1.05	-3.63	2.5	-1.13	4263.5	TRUE	TRUE	TRUE	TRUE	TRUE	TRUE
1	IITTDNTFV	1096	1104	9	HLA-A*02:01	1096	2.2	338.82	2.2	1.220497	0.16390746	-2.529969	1.384404463	-1.1455646	1.22	0.16	-2.53	1.38	-1.15	338.8	TRUE	TRUE	TRUE	TRUE	TRUE	TRUE
1	TMNNKSQSV	106	114	9	HLA-A*02:01	106	1.8	254.01	1.8	1.073304	0.16104814	-2.4048508	1.23435214	-1.1704987	1.07	0.16	-2.4	1.23	-1.17	254	TRUE	TRUE	TRUE	TRUE	TRUE	TRUE
1	WTFGAGAAL	868	876	9	HLA-A*02:01	868	5.3	1468.2	5.3	1.4869	0.430736438	-3.1667852	1.91763644	-1.2491488	1.49	0.43	-3.17	1.92	-1.25	1468.2	TRUE	TRUE	TRUE	TRUE	TRUE	TRUE
1	FVSGNCDVV	1103	1111	9	HLA-A*02:01	1103	2	290.37	2	1.049681	0.15672869	-2.4629517	1.20640969	-1.2565421	1.05	0.16	-2.46	1.21	-1.26	290.4	TRUE	TRUE	TRUE	TRUE	TRUE	TRUE
1	LIAIVMVTI	1206	1214	9	HLA-A*02:01	1206	3.4	691.85	3.4	1.294585	0.283099249	-2.8400119	1.577684249	-1.2623277	1.29	0.28	-2.84	1.58	-1.26	691.9	TRUE	TRUE	TRUE	TRUE	TRUE	TRUE
1	GICASYHTV	655	663	9	HLA-A*02:01	655	3	546.07	3	1.282485	0.18375871	-2.7372483	1.46624371	-1.2710046	1.28	0.18	-2.74	1.47	-1.27	546.1	TRUE	TRUE	TRUE	TRUE	TRUE	TRUE
1	AISQIQESL	912	920	9	HLA-A*02:01	912	7.1	2333.11	7.1	1.499004	0.5768055	-3.3679352	2.0758095	-1.2921257	1.5	0.58	-3.37	2.08	-1.29	2333.1	TRUE	TRUE	TRUE	TRUE	TRUE	TRUE
1	QMAYRFNGI	883	891	9	HLA-A*02:01	883	3	550.3	3	1.106379	0.31230631	-2.7405995	1.41868531	-1.3219142	1.11	0.31	-2.74	1.42	-1.32	550.3	TRUE	TRUE	TRUE	TRUE	TRUE	TRUE
1	NLCPFGEVF	321	329	9	HLA-A*02:01	321	21	11598.13	21	1.608596	1.13264409	-4.064388	2.74124009	-1.3231479	1.61	1.13	-4.06	2.74	-1.32	11598.1	TRUE	TRUE	TRUE	TRUE	TRUE	TRUE
1	TQDLFLPFY	55	63	9	HLA-A*02:01	55	23	12502.84	23	1.518882	1.237551155	-4.0970087	2.75643316	-1.3405755	1.52	1.24	-4.1	2.76	-1.34	12502.8	TRUE	TRUE	TRUE	TRUE	TRUE	TRUE
1	ELLNAPATV	502	510	9	HLA-A*02:01	502	2	279.55	2	1.078965	0.0177189	-2.4464595	1.0966839	-1.3497756	1.08	0.02	-2.45	1.1	-1.35	279.6	TRUE	TRUE	TRUE	TRUE	TRUE	TRUE
1	ATAGWTFGA	864	872	9	HLA-A*02:01	864	1.6	207.35	1.6	1.101109	-0.1666238	-2.316704	0.934485184	-1.3822189	1.1	-0.17	-2.32	0.93	-1.38	207.3	TRUE	TRUE	TRUE	TRUE	TRUE	TRUE
1	MIFDNAFNC	151	159	9	HLA-A*02:01	151	2.5	421.14	2.5	1.101151	0.1345851	-2.6244265	1.235736098	-1.3886904	1.1	0.13	-2.62	1.24	-1.39	421.1	TRUE	TRUE	TRUE	TRUE	TRUE	TRUE
1	CVADYSVLY	348	356	9	HLA-A*02:01	348	31	18163.24	31	1.475542	1.380533285	-4.2591933	2.856075285	-1.403118	1.48	1.38	-4.26	2.86	-1.4	18163.2	TRUE	TRUE	TRUE	TRUE	TRUE	TRUE
1	ASSEVAVLY	590	598	9	HLA-A*02:01	590	33	19447.87	33	1.471538	1.40941929	-4.288872	2.880957294	-1.4079147	1.47	1.41	-4.29	2.88	-1.41	19447.9	TRUE	TRUE	TRUE	TRUE	TRUE	TRUE
1	VVVLSFELL	496	504	9	HLA-A*02:01	496	9.1	3574.54	9.1	1.545624	0.594644748	-3.5532202	2.140268748	-1.4129514	1.55	0.59	-3.55	2.14	-1.41	3574.5	TRUE	TRUE	TRUE	TRUE	TRUE	TRUE
1	ILDISPCSF	570	578	9	HLA-A*02:01	570	14	6194.59	14	1.399382	0.96924642	-3.7920126	2.36862842	-1.4233841	1.4	0.97	-3.79	2.37	-1.42	6194.6	TRUE	TRUE	TRUE	TRUE	TRUE	TRUE
1	AAPHGVVFL	1037	1045	9	HLA-A*02:01	1037	9.1	3528.66	9.1	1.6386	0.474321256	-3.5476098	2.11292126	-1.4346886	1.64	0.47	-3.55	2.11	-1.43	3528.7	TRUE	TRUE	TRUE	TRUE	TRUE	TRUE
1	VWLGFIAGL	1198	1206	9	HLA-A*02:01	1198	6.5	2036.12	6.5	1.334845	0.53565961	-3.3088034	1.87050461	-1.4382988	1.33	0.54	-3.31	1.87	-1.44	2036.1	TRUE	TRUE	TRUE	TRUE	TRUE	TRUE
1	SVLYNSTFF	353	361	9	HLA-A*02:01	353	22	11760.01	22	1.463696	1.16803876	-4.0704077	2.63173476	-1.4386729	1.46	1.17	-4.07	2.63	-1.44	11760	TRUE	TRUE	TRUE	TRUE	TRUE	TRUE
1	KQLSSNFGA	946	954	9	HLA-A*02:01	946	1.2	141.16	1.2	0.799879	-0.1243004	-2.1497116	0.675578648	-1.474133	0.8	-0.12	-2.15	0.68	-1.47	141.2	TRUE	TRUE	TRUE	TRUE	TRUE	TRUE
1	ALGKLQDVV	926	934	9	HLA-A*02:01	926	3.4	717.01	3.4	1.21447	0.14017614	-2.8555252	1.35464614	-1.5008791	1.21	0.14	-2.86	1.35	-1.5	717	TRUE	TRUE	TRUE	TRUE	TRUE	TRUE
1	AAAYFVGYL	249	257	9	HLA-A*02:01	249	6.4	1975.49	6.4	1.258462	0.5325764	-3.2956748	1.791038398	-1.5046364	1.26	0.53	-3.3	1.79	-1.5	1975.5	TRUE	TRUE	TRUE	TRUE	TRUE	TRUE
1	GVVFLHVTY	1041	1049	9	HLA-A*02:01	1041	31	17901.22	31	1.458749	1.27549382	-4.2528826	2.73424282	-1.5186398	1.46	1.28	-4.25	2.73	-1.52	17901.2	TRUE	TRUE	TRUE	TRUE	TRUE	TRUE
1	NDLCFSNVY	375	383	9	HLA-A*02:01	375	52	28828.1	52	1.752871	1.181364901	-4.459816	2.9342359	-1.5255801	1.75	1.18	-4.46	2.93	-1.53	28828.1	TRUE	TRUE	TRUE	TRUE	TRUE	TRUE
1	VLAWNTRNI	420	428	9	HLA-A*02:01	420	3.7	808.52	3.7	1.108695	0.27115027	-2.9076908	1.37984527	-1.5278455	1.11	0.27	-2.91	1.38	-1.53	808.5	TRUE	TRUE	TRUE	TRUE	TRUE	TRUE
1	LQIPFAMQM	876	884	9	HLA-A*02:01	876	4	896.61	4	1.156346	0.26361241	-2.9526036	1.419958408	-1.5326452	1.16	0.26	-2.95	1.42	-1.53	896.6	TRUE	TRUE	TRUE	TRUE	TRUE	TRUE
1	QLTPAWRIY	614	622	9	HLA-A*02:01	614	41	23738.87	41	1.632951	1.20808804	-4.37546	2.841039036	-1.534421	1.63	1.21	-4.38	2.84	-1.53	23738.9	TRUE	TRUE	TRUE	TRUE	TRUE	TRUE
1	AVDCSQNPL	275	283	9	HLA-A*02:01	275	5.8	1719.38	5.8	1.298059	0.402689789	-3.2353719	1.700748789	-1.5346231	1.3	0.4	-3.24	1.7	-1.53	1719.4	TRUE	TRUE	TRUE	TRUE	TRUE	TRUE
1	NKDGFLYVY	189	197	9	HLA-A*02:01	189	45	25813.9	45	1.585491	1.2748933	-4.4118536	2.860384297	-1.5514693	1.59	1.27	-4.41	2.86	-1.55	25813.9	TRUE	TRUE	TRUE	TRUE	TRUE	TRUE
1	TFFSTFKCY	359	367	9	HLA-A*02:01	359	49	27791.96	49	1.475132	1.40541872	-4.4439192	2.880550721	-1.5633685	1.48	1.41	-4.44	2.88	-1.56	27792	TRUE	TRUE	TRUE	TRUE	TRUE	TRUE
1	IKWPWYVWL	1192	1200	9	HLA-A*02:01	1192	12	4469.74	12	1.583339	0.499880705	-3.6502823	2.083219705	-1.5670626	1.58	0.5	-3.65	2.08	-1.57	4469.7	TRUE	TRUE	TRUE	TRUE	TRUE	TRUE
1	TLYLTQDLF	51	59	9	HLA-A*02:01	51	14	5741.19	14	1.07175	1.12004251	-3.7590019	2.19179251	-1.5672094	1.07	1.12	-3.76	2.19	-1.57	5741.2	TRUE	TRUE	TRUE	TRUE	TRUE	TRUE
1	NLLLQYGSF	733	741	9	HLA-A*02:01	733	23	12869.44	23	1.32195	1.19967305	-4.1095596	2.521623047	-1.5879366	1.32	1.2	-4.11	2.52	-1.59	12869.4	TRUE	TRUE	TRUE	TRUE	TRUE	TRUE
1	VLSFELLNA	498	506	9	HLA-A*02:01	498	2	273.66	2	1.022198	-0.1772962	-2.4372113	0.844901833	-1.5923095	1.02	-0.18	-2.44	0.84	-1.59	273.7	TRUE	TRUE	TRUE	TRUE	TRUE	TRUE
1	FSTFKCYGV	361	369	9	HLA-A*02:01	361	2.8	504.47	2.8	1.054766	0.05056053	-2.7028353	1.105326525	-1.5975088	1.05	0.05	-2.7	1.11	-1.6	504.5	TRUE	TRUE	TRUE	TRUE	TRUE	TRUE
1	VIGIINNTV	1111	1119	9	HLA-A*02:01	1111	5	1299.39	5	1.340143	0.16753679	-3.1137395	1.507679791	-1.6060597	1.34	0.17	-3.11	1.51	-1.61	1299.4	TRUE	TRUE	TRUE	TRUE	TRUE	TRUE
1	VLKGVKLHY	1246	1254	9	HLA-A*02:01	1246	32	18978.49	32	1.416566	1.24140368	-4.2782617	2.657969678	-1.620292	1.42	1.24	-4.28	2.66	-1.62	18978.5	TRUE	TRUE	TRUE	TRUE	TRUE	TRUE
1	SIAYSNNTI	686	694	9	HLA-A*02:01	686	8	2910.15	8	1.500719	0.34035592	-3.4639154	1.841074918	-1.6228405	1.5	0.34	-3.46	1.84	-1.62	2910.2	TRUE	TRUE	TRUE	TRUE	TRUE	TRUE
1	ILLCCMTSC	1214	1222	9	HLA-A*02:01	1214	2.5	403.87	2.5	0.945744	0.03624098	-2.6062416	0.98198498	-1.6242566	0.95	0.04	-2.61	0.98	-1.62	403.9	TRUE	TRUE	TRUE	TRUE	TRUE	TRUE
1	GTATAGWTF	862	870	9	HLA-A*02:01	862	32	19075.05	32	1.592766	1.04289159	-4.2804657	2.63565759	-1.6448081	1.59	1.04	-4.28	2.64	-1.64	19075	TRUE	TRUE	TRUE	TRUE	TRUE	TRUE
1	RSFIEDLLF	797	805	9	HLA-A*02:01	797	27	15557.23	27	1.228417	1.31565516	-4.1919323	2.54407216	-1.6478601	1.23	1.32	-4.19	2.54	-1.65	15557.2	TRUE	TRUE	TRUE	TRUE	TRUE	TRUE
1	TSYECDIPI	644	652	9	HLA-A*02:01	644	3.2	639.51	3.2	0.890322	0.26027095	-2.8058473	1.15059295	-1.6552544	0.89	0.26	-2.81	1.15	-1.66	639.5	TRUE	TRUE	TRUE	TRUE	TRUE	TRUE
1	FKHLREFVF	179	187	9	HLA-A*02:01	179	32	18887.33	32	1.443585	1.15432038	-4.2761706	2.59790538	-1.6782652	1.44	1.15	-4.28	2.6	-1.68	18887.3	TRUE	TRUE	TRUE	TRUE	TRUE	TRUE
1	RIYSTGNNV	620	628	9	HLA-A*02:01	620	4.4	1067.65	4.4	0.994041	0.35410701	-3.0284289	1.348148007	-1.6802809	0.99	0.35	-3.03	1.35	-1.68	1067.7	TRUE	TRUE	TRUE	TRUE	TRUE	TRUE
1	LTDDMIAAY	847	855	9	HLA-A*02:01	847	32	18571.42	32	1.379396	1.20689343	-4.2688451	2.58628943	-1.6825557	1.38	1.21	-4.27	2.59	-1.68	18571.4	TRUE	TRUE	TRUE	TRUE	TRUE	TRUE
1	GLIAIVMVT	1205	1213	9	HLA-A*02:01	1205	1.8	242.04	1.8	0.974212	-0.2791116	-2.3838871	0.695100405	-1.6887867	0.97	-0.28	-2.38	0.7	-1.69	242	TRUE	TRUE	TRUE	TRUE	TRUE	TRUE
1	NTIAIPTNF	692	700	9	HLA-A*02:01	692	36	21445.29	36	1.439264	1.19816516	-4.3313319	2.637429157	-1.6939028	1.44	1.2	-4.33	2.64	-1.69	21445.3	TRUE	TRUE	TRUE	TRUE	TRUE	TRUE
1	MYKTPTLKY	770	778	9	HLA-A*02:01	770	71	34974.67	71	1.464091	1.382807249	-4.5437536	2.84689825	-1.6968554	1.46	1.38	-4.54	2.85	-1.7	34974.7	TRUE	TRUE	TRUE	TRUE	TRUE	TRUE
1	DLLFNKVTL	802	810	9	HLA-A*02:01	802	17	8350.43	17	1.995344	0.22314691	-3.9217088	2.21849091	-1.7032179	2	0.22	-3.92	2.22	-1.7	8350.4	TRUE	TRUE	TRUE	TRUE	TRUE	TRUE
1	GRLQSLQTY	981	989	9	HLA-A*02:01	981	54	29702.92	54	1.472985	1.293328277	-4.4727991	2.76631328	-1.7064859	1.47	1.29	-4.47	2.77	-1.71	29702.9	TRUE	TRUE	TRUE	TRUE	TRUE	TRUE
1	RVDFCGKGY	1021	1029	9	HLA-A*02:01	1021	55	29757.6	55	1.363169	1.403431756	-4.4735979	2.76660076	-1.7069971	1.36	1.4	-4.47	2.77	-1.71	29757.6	TRUE	TRUE	TRUE	TRUE	TRUE	TRUE
1	NVVIRACNF	122	130	9	HLA-A*02:01	122	35	20683.01	35	1.392653	1.213958357	-4.3156137	2.606611357	-1.7090024	1.39	1.21	-4.32	2.61	-1.71	20683	TRUE	TRUE	TRUE	TRUE	TRUE	TRUE
1	TSVDCNMYI	716	724	9	HLA-A*02:01	716	5	1314.15	5	1.091948	0.31671413	-3.1186449	1.408662135	-1.7099828	1.09	0.32	-3.12	1.41	-1.71	1314.2	TRUE	TRUE	TRUE	TRUE	TRUE	TRUE
1	HTMIFDNAF	149	157	9	HLA-A*02:01	149	25	13940.56	25	1.348305	1.08275936	-4.1442802	2.43106436	-1.7132159	1.35	1.08	-4.14	2.43	-1.71	13940.6	TRUE	TRUE	TRUE	TRUE	TRUE	TRUE
1	LIKNQCVNF	519	527	9	HLA-A*02:01	519	43	24920.33	43	1.585627	1.09610414	-4.3965538	2.68173114	-1.7148226	1.59	1.1	-4.4	2.68	-1.71	24920.3	TRUE	TRUE	TRUE	TRUE	TRUE	TRUE
1	LGADSSIAY	681	689	9	HLA-A*02:01	681	63	32553.99	63	1.604755	1.18644997	-4.5126042	2.791204966	-1.7213993	1.6	1.19	-4.51	2.79	-1.72	32554	TRUE	TRUE	TRUE	TRUE	TRUE	TRUE
1	LTQDLFLPF	54	62	9	HLA-A*02:01	54	17	8106.15	17	1.130736	1.05570717	-3.9088146	2.18644317	-1.7223715	1.13	1.06	-3.91	2.19	-1.72	8106.1	TRUE	TRUE	TRUE	TRUE	TRUE	TRUE
1	ATKLNDLCF	371	379	9	HLA-A*02:01	371	48	26988.6	48	1.531581	1.17538601	-4.4311804	2.706967007	-1.7242133	1.53	1.18	-4.43	2.71	-1.72	26988.6	TRUE	TRUE	TRUE	TRUE	TRUE	TRUE
1	AYFPREGVF	1069	1077	9	HLA-A*02:01	1069	67	33966.61	67	1.42852	1.3697379	-4.5310522	2.7982579	-1.7327943	1.43	1.37	-4.53	2.8	-1.73	33966.6	TRUE	TRUE	TRUE	TRUE	TRUE	TRUE
1	TSSMRGVYY	34	42	9	HLA-A*02:01	34	50	28010.53	50	1.447963	1.265886368	-4.4473213	2.71384937	-1.733472	1.45	1.27	-4.45	2.71	-1.73	28010.5	TRUE	TRUE	TRUE	TRUE	TRUE	TRUE
1	ASYHTVSLL	658	666	9	HLA-A*02:01	658	12	4557.1	12	1.350033	0.57477359	-3.6586886	1.924806594	-1.733882	1.35	0.57	-3.66	1.92	-1.73	4557.1	TRUE	TRUE	TRUE	TRUE	TRUE	TRUE
1	KISNCVADY	344	352	9	HLA-A*02:01	344	35	20411.79	35	1.161182	1.413685075	-4.3098811	2.574867075	-1.735014	1.16	1.41	-4.31	2.57	-1.74	20411.8	TRUE	TRUE	TRUE	TRUE	TRUE	TRUE
1	TQAGCLIGA	631	639	9	HLA-A*02:01	631	3	551.45	3	1.2258	-0.2221748	-2.7415061	1.00362521	-1.7378809	1.23	-0.22	-2.74	1	-1.74	551.5	TRUE	TRUE	TRUE	TRUE	TRUE	TRUE
1	IYSTGNNVF	621	629	9	HLA-A*02:01	621	72	35427.51	72	1.528755	1.28001129	-4.5493406	2.80876629	-1.7405743	1.53	1.28	-4.55	2.81	-1.74	35427.5	TRUE	TRUE	TRUE	TRUE	TRUE	TRUE
1	QLNRALSGI	744	752	9	HLA-A*02:01	744	5.2	1406.79	5.2	1.189256	0.20893765	-3.1482293	1.39819365	-1.7500356	1.19	0.21	-3.15	1.4	-1.75	1406.8	TRUE	TRUE	TRUE	TRUE	TRUE	TRUE
1	YWPLNDYGF	475	483	9	HLA-A*02:01	475	42	24304.4	42	1.444704	1.188458728	-4.3856849	2.63316273	-1.7525222	1.44	1.19	-4.39	2.63	-1.75	24304.4	TRUE	TRUE	TRUE	TRUE	TRUE	TRUE
1	NTLKPIFKL	214	222	9	HLA-A*02:01	214	8.9	3454.35	8.9	1.396372	0.385903192	-3.5383663	1.782275192	-1.7560911	1.4	0.39	-3.54	1.78	-1.76	3454.3	TRUE	TRUE	TRUE	TRUE	TRUE	TRUE
1	RDLICAQKF	829	837	9	HLA-A*02:01	829	47	26602.73	47	1.519755	1.14714307	-4.4249262	2.66689807	-1.7580281	1.52	1.15	-4.42	2.67	-1.76	26602.7	TRUE	TRUE	TRUE	TRUE	TRUE	TRUE
1	WPWYVWLGF	1194	1202	9	HLA-A*02:01	1194	23	12528.43	23	1.263088	1.074012449	-4.0978967	2.337100449	-1.7607962	1.26	1.07	-4.1	2.34	-1.76	12528.4	TRUE	TRUE	TRUE	TRUE	TRUE	TRUE
1	FELCDNPFF	130	138	9	HLA-A*02:01	130	34	19881.25	34	1.467821	1.0691032	-4.2984437	2.5369242	-1.7615195	1.47	1.07	-4.3	2.54	-1.76	19881.2	TRUE	TRUE	TRUE	TRUE	TRUE	TRUE
1	KTSVDCNMY	715	723	9	HLA-A*02:01	715	48	27118.28	48	1.298569	1.37182059	-4.4332621	2.67038959	-1.7628726	1.3	1.37	-4.43	2.67	-1.76	27118.3	TRUE	TRUE	TRUE	TRUE	TRUE	TRUE
1	YQDVNCTDV	598	606	9	HLA-A*02:01	598	3.2	612.82	3.2	0.917463	0.103832515	-2.7873329	1.021295515	-1.7660374	0.92	0.1	-2.79	1.02	-1.77	612.8	TRUE	TRUE	TRUE	TRUE	TRUE	TRUE
1	VFAQVKQMY	763	771	9	HLA-A*02:01	763	56	30172.6	56	1.405748	1.304284529	-4.4796127	2.71003253	-1.7695802	1.41	1.3	-4.48	2.71	-1.77	30172.6	TRUE	TRUE	TRUE	TRUE	TRUE	TRUE
1	FYTTTGIGY	483	491	9	HLA-A*02:01	483	50	27836.81	50	1.351758	1.31489798	-4.4446195	2.66665598	-1.7779635	1.35	1.31	-4.44	2.67	-1.78	27836.8	TRUE	TRUE	TRUE	TRUE	TRUE	TRUE
1	AILTAFSPA	233	241	9	HLA-A*02:01	233	2.3	351.2	2.3	0.810435	-0.0475561	-2.5455545	0.762878938	-1.7826756	0.81	-0.05	-2.55	0.76	-1.78	351.2	TRUE	TRUE	TRUE	TRUE	TRUE	TRUE
1	STGNYNYKY	432	440	9	HLA-A*02:01	432	63	32640.03	63	1.513922	1.216583613	-4.5137505	2.730505613	-1.7832449	1.51	1.22	-4.51	2.73	-1.78	32640	TRUE	TRUE	TRUE	TRUE	TRUE	TRUE
1	VLYNSTFFS	354	362	9	HLA-A*02:01	354	0.64	70.21	0.64	0.899305	-0.8362151	-1.846399	0.06308989	-1.7833091	0.9	-0.84	-1.85	0.06	-1.78	70.2	TRUE	TRUE	TRUE	TRUE	TRUE	TRUE
1	NAFNCTFEY	155	163	9	HLA-A*02:01	155	27	15839.69	27	1.113856	1.29733438	-4.1997467	2.411190378	-1.7885563	1.11	1.3	-4.2	2.41	-1.79	15839.7	TRUE	TRUE	TRUE	TRUE	TRUE	TRUE
1	CTPPALNCY	467	475	9	HLA-A*02:01	467	52	28741.21	52	1.49599	1.172932971	-4.458505	2.668922971	-1.7895821	1.5	1.17	-4.46	2.67	-1.79	28741.2	TRUE	TRUE	TRUE	TRUE	TRUE	TRUE
1	IPFAMQMAY	878	886	9	HLA-A*02:01	878	42	24674.56	42	1.422647	1.174906725	-4.3922494	2.59755373	-1.7946957	1.42	1.17	-4.39	2.6	-1.79	24674.6	TRUE	TRUE	TRUE	TRUE	TRUE	TRUE
1	GVYYPDEIF	39	47	9	HLA-A*02:01	39	43	25003.78	43	1.382489	1.22048847	-4.3980057	2.602977474	-1.7950282	1.38	1.22	-4.4	2.6	-1.8	25003.8	TRUE	TRUE	TRUE	TRUE	TRUE	TRUE
1	VMVTILLCC	1210	1218	9	HLA-A*02:01	1210	5.9	1723.82	5.9	1.318358	0.122703233	-3.2364919	1.44106123	-1.7954307	1.32	0.12	-3.24	1.44	-1.8	1723.8	TRUE	TRUE	TRUE	TRUE	TRUE	TRUE
1	IAIVMVTIL	1207	1215	9	HLA-A*02:01	1207	9.9	4092.03	9.9	1.310935	0.50011128	-3.6119388	1.811046282	-1.8008925	1.31	0.5	-3.61	1.81	-1.8	4092	TRUE	TRUE	TRUE	TRUE	TRUE	TRUE
1	LQTYVTQQL	986	994	9	HLA-A*02:01	986	9.6	3910.02	9.6	1.330605	0.458611248	-3.592179	1.789216248	-1.8029627	1.33	0.46	-3.59	1.79	-1.8	3910	TRUE	TRUE	TRUE	TRUE	TRUE	TRUE
1	YSVLYNSTF	352	360	9	HLA-A*02:01	352	27	15759.18	27	1.30436	1.08184382	-4.1975336	2.38620382	-1.8113298	1.3	1.08	-4.2	2.39	-1.81	15759.2	TRUE	TRUE	TRUE	TRUE	TRUE	TRUE
1	VVRDLPSGF	205	213	9	HLA-A*02:01	205	52	28871.49	52	1.501338	1.14751912	-4.4604692	2.64885712	-1.8116121	1.5	1.15	-4.46	2.65	-1.81	28871.5	TRUE	TRUE	TRUE	TRUE	TRUE	TRUE
1	AICHEGKAY	1062	1070	9	HLA-A*02:01	1062	69	34617.76	69	1.397938	1.328905881	-4.539299	2.726843881	-1.8124551	1.4	1.33	-4.54	2.73	-1.81	34617.8	TRUE	TRUE	TRUE	TRUE	TRUE	TRUE
1	WLGFIAGLI	1199	1207	9	HLA-A*02:01	1199	5.6	1603.03	5.6	1.214521	0.177586745	-3.2049417	1.392107745	-1.8128339	1.21	0.18	-3.2	1.39	-1.81	1603	TRUE	TRUE	TRUE	TRUE	TRUE	TRUE
1	NFRAILTAF	230	238	9	HLA-A*02:01	230	38	22628.84	38	1.324247	1.21419926	-4.3546623	2.53844626	-1.816216	1.32	1.21	-4.35	2.54	-1.82	22628.8	TRUE	TRUE	TRUE	TRUE	TRUE	TRUE
1	ADAGFMKQY	811	819	9	HLA-A*02:01	811	65	33196.35	65	1.476554	1.22707747	-4.5210903	2.70363147	-1.8174589	1.48	1.23	-4.52	2.7	-1.82	33196.3	TRUE	TRUE	TRUE	TRUE	TRUE	TRUE
1	ELKCSVKSF	285	293	9	HLA-A*02:01	285	47	26646.22	47	1.520731	1.085658	-4.4256356	2.606389005	-1.8192466	1.52	1.09	-4.43	2.61	-1.82	26646.2	TRUE	TRUE	TRUE	TRUE	TRUE	TRUE
1	VGYLKPTTF	254	262	9	HLA-A*02:01	254	45	25654.35	45	1.541901	1.04655643	-4.409161	2.588457435	-1.8207036	1.54	1.05	-4.41	2.59	-1.82	25654.3	TRUE	TRUE	TRUE	TRUE	TRUE	TRUE
1	QIITTDNTF	1095	1103	9	HLA-A*02:01	1095	43	24771.39	43	1.444468	1.12799029	-4.3939504	2.572458293	-1.8214921	1.44	1.13	-4.39	2.57	-1.82	24771.4	TRUE	TRUE	TRUE	TRUE	TRUE	TRUE
1	TSQKSIVAY	669	677	9	HLA-A*02:01	669	55	29996.51	55	1.366782	1.28704004	-4.4770707	2.65382204	-1.8232487	1.37	1.29	-4.48	2.65	-1.82	29996.5	TRUE	TRUE	TRUE	TRUE	TRUE	TRUE
1	EQYIKWPWY	1189	1197	9	HLA-A*02:01	1189	40	23404.77	40	1.27106	1.272347949	-4.3693044	2.54340795	-1.8258964	1.27	1.27	-4.37	2.54	-1.83	23404.8	TRUE	TRUE	TRUE	TRUE	TRUE	TRUE
1	YQTSNFRVV	300	308	9	HLA-A*02:01	300	4.6	1149.79	4.6	1.086564	0.147571076	-3.0606185	1.234135076	-1.8264835	1.09	0.15	-3.06	1.23	-1.83	1149.8	TRUE	TRUE	TRUE	TRUE	TRUE	TRUE
1	IGIINNTVY	1112	1120	9	HLA-A*02:01	1112	56	30252.03	56	1.436018	1.214121154	-4.4807545	2.650139154	-1.8306154	1.44	1.21	-4.48	2.65	-1.83	30252	TRUE	TRUE	TRUE	TRUE	TRUE	TRUE
1	LKYFGGFNF	776	784	9	HLA-A*02:01	776	36	21449.47	36	1.291444	1.20836934	-4.3314166	2.49981334	-1.8316032	1.29	1.21	-4.33	2.5	-1.83	21449.5	TRUE	TRUE	TRUE	TRUE	TRUE	TRUE
1	WFITQRNFF	1084	1092	9	HLA-A*02:01	1084	56	30347.76	56	1.430926	1.21410356	-4.4821266	2.64502956	-1.8370971	1.43	1.21	-4.48	2.65	-1.84	30347.8	TRUE	TRUE	TRUE	TRUE	TRUE	TRUE
1	GINASVVNI	1153	1161	9	HLA-A*02:01	1153	5.1	1346.83	5.1	1.093186	0.1915608	-3.1293128	1.2847468	-1.844566	1.09	0.19	-3.13	1.28	-1.84	1346.8	TRUE	TRUE	TRUE	TRUE	TRUE	TRUE
1	NATKFPSVY	330	338	9	HLA-A*02:01	330	68	34201.53	68	1.440317	1.24775158	-4.5340455	2.688068579	-1.845977	1.44	1.25	-4.53	2.69	-1.85	34201.5	TRUE	TRUE	TRUE	TRUE	TRUE	TRUE
1	SNVVRGWVF	95	103	9	HLA-A*02:01	95	41	23953.54	41	1.399307	1.13386983	-4.3793697	2.53317683	-1.8461929	1.4	1.13	-4.38	2.53	-1.85	23953.5	TRUE	TRUE	TRUE	TRUE	TRUE	TRUE
1	QAAPHGVVF	1036	1044	9	HLA-A*02:01	1036	57	30725.73	57	1.561146	1.07225404	-4.4875022	2.633400036	-1.8541022	1.56	1.07	-4.49	2.63	-1.85	30725.7	TRUE	TRUE	TRUE	TRUE	TRUE	TRUE
1	FLPFYSNVT	59	67	9	HLA-A*02:01	59	2.4	394.17	2.4	1.062295	-0.3245702	-2.5956836	0.73772484	-1.8579587	1.06	-0.32	-2.6	0.74	-1.86	394.2	TRUE	TRUE	TRUE	TRUE	TRUE	TRUE
1	ATSTGNYNY	430	438	9	HLA-A*02:01	430	61	31900.22	61	1.383309	1.26097604	-4.5037937	2.644285039	-1.8595086	1.38	1.26	-4.5	2.64	-1.86	31900.2	TRUE	TRUE	TRUE	TRUE	TRUE	TRUE
1	NCYWPLNDY	473	481	9	HLA-A*02:01	473	49	27759.81	49	1.184532	1.39682623	-4.4434165	2.58135823	-1.8620583	1.18	1.4	-4.44	2.58	-1.86	27759.8	TRUE	TRUE	TRUE	TRUE	TRUE	TRUE
1	SAAAYFVGY	248	256	9	HLA-A*02:01	248	36	20956.49	36	1.132515	1.32672669	-4.3213185	2.45924169	-1.8620769	1.13	1.33	-4.32	2.46	-1.86	20956.5	TRUE	TRUE	TRUE	TRUE	TRUE	TRUE
1	SWFITQRNF	1083	1091	9	HLA-A*02:01	1083	44	25376.08	44	1.25396	1.28207728	-4.4044245	2.53603728	-1.8683873	1.25	1.28	-4.4	2.54	-1.87	25376.1	TRUE	TRUE	TRUE	TRUE	TRUE	TRUE
1	GFHTINHTF	68	76	9	HLA-A*02:01	68	49	27677.33	49	1.455266	1.11159183	-4.4421242	2.56685783	-1.8752664	1.46	1.11	-4.44	2.57	-1.88	27677.3	TRUE	TRUE	TRUE	TRUE	TRUE	TRUE
1	GFLYVYKGY	192	200	9	HLA-A*02:01	192	47	26952.42	47	1.338975	1.21238129	-4.4305978	2.551356289	-1.8792415	1.34	1.21	-4.43	2.55	-1.88	26952.4	TRUE	TRUE	TRUE	TRUE	TRUE	TRUE
1	IINNSTNVV	116	124	9	HLA-A*02:01	116	4.4	1073.84	4.4	0.984725	0.16415223	-3.0309396	1.148877227	-1.8820624	0.98	0.16	-3.03	1.15	-1.88	1073.8	TRUE	TRUE	TRUE	TRUE	TRUE	TRUE
1	SDLDRCTTF	14	22	9	HLA-A*02:01	14	53	29070.21	53	1.596841	0.98369479	-4.4634482	2.58053579	-1.8829124	1.6	0.98	-4.46	2.58	-1.88	29070.2	TRUE	TRUE	TRUE	TRUE	TRUE	TRUE
1	IWGTSAAAY	244	252	9	HLA-A*02:01	244	58	31084.19	58	1.352709	1.2554346	-4.4925396	2.6081436	-1.884396	1.35	1.26	-4.49	2.61	-1.88	31084.2	TRUE	TRUE	TRUE	TRUE	TRUE	TRUE
1	VLTPSSKRF	537	545	9	HLA-A*02:01	537	40	23675.51	40	1.379908	1.10656681	-4.3742993	2.486474813	-1.8878245	1.38	1.11	-4.37	2.49	-1.89	23675.5	TRUE	TRUE	TRUE	TRUE	TRUE	TRUE
1	KPTTFMLKY	258	266	9	HLA-A*02:01	258	52	28725.66	52	1.38102	1.18919362	-4.45827	2.57021362	-1.8880564	1.38	1.19	-4.46	2.57	-1.89	28725.7	TRUE	TRUE	TRUE	TRUE	TRUE	TRUE
1	KLPDDFMGC	411	419	9	HLA-A*02:01	411	4.3	1013.85	4.3	1.083102	0.03181904	-3.0059737	1.11492104	-1.8910527	1.08	0.03	-3.01	1.11	-1.89	1013.9	TRUE	TRUE	TRUE	TRUE	TRUE	TRUE
1	KVTLADAGF	807	815	9	HLA-A*02:01	807	39	23159.67	39	1.219275	1.251849106	-4.3647324	2.471124106	-1.8936083	1.22	1.25	-4.36	2.47	-1.89	23159.7	TRUE	TRUE	TRUE	TRUE	TRUE	TRUE
1	ELCDNPFFA	131	139	9	HLA-A*02:01	131	2.5	408.87	2.5	1.068746	-0.3531314	-2.6115852	0.715614592	-1.8959707	1.07	-0.35	-2.61	0.72	-1.9	408.9	TRUE	TRUE	TRUE	TRUE	TRUE	TRUE
1	YNSTFFSTF	356	364	9	HLA-A*02:01	356	35	20820.89	35	1.376617	1.04411286	-4.3184993	2.42072986	-1.8977694	1.38	1.04	-4.32	2.42	-1.9	20820.9	TRUE	TRUE	TRUE	TRUE	TRUE	TRUE
1	ECANLLLQY	730	738	9	HLA-A*02:01	730	49	27775.73	49	1.342854	1.20092963	-4.4436655	2.54378363	-1.8998819	1.34	1.2	-4.44	2.54	-1.9	27775.7	TRUE	TRUE	TRUE	TRUE	TRUE	TRUE
1	IPFKDGIYF	81	89	9	HLA-A*02:01	81	37	22014.23	37	1.390972	1.04639905	-4.3427035	2.43737105	-1.9053325	1.39	1.05	-4.34	2.44	-1.91	22014.2	TRUE	TRUE	TRUE	TRUE	TRUE	TRUE
1	DLPSGFNTL	208	216	9	HLA-A*02:01	208	15	6837.1	15	1.589408	0.3389798	-3.8348719	1.928387799	-1.9064841	1.59	0.34	-3.83	1.93	-1.91	6837.1	TRUE	TRUE	TRUE	TRUE	TRUE	TRUE
1	IGAGICASY	652	660	9	HLA-A*02:01	652	43	24799.83	43	1.377307	1.10799482	-4.3944487	2.48530182	-1.9091469	1.38	1.11	-4.39	2.49	-1.91	24799.8	TRUE	TRUE	TRUE	TRUE	TRUE	TRUE
1	KRFQPFQQF	543	551	9	HLA-A*02:01	543	51	28583.66	51	1.239377	1.30754442	-4.4561178	2.54692142	-1.9091964	1.24	1.31	-4.46	2.55	-1.91	28583.7	TRUE	TRUE	TRUE	TRUE	TRUE	TRUE
1	IGVTQNVLY	891	899	9	HLA-A*02:01	891	58	31081.5	58	1.397652	1.18435173	-4.492502	2.58200373	-1.9104982	1.4	1.18	-4.49	2.58	-1.91	31081.5	TRUE	TRUE	TRUE	TRUE	TRUE	TRUE
1	FPREGVFVF	1071	1079	9	HLA-A*02:01	1071	32	19145.96	32	1.439859	0.92519006	-4.2820771	2.365049059	-1.9170281	1.44	0.93	-4.28	2.37	-1.92	19146	TRUE	TRUE	TRUE	TRUE	TRUE	TRUE
1	KNQCVNFNF	521	529	9	HLA-A*02:01	521	44	25221.43	44	1.414723	1.06989599	-4.4017697	2.48461899	-1.9171507	1.41	1.07	-4.4	2.48	-1.92	25221.4	TRUE	TRUE	TRUE	TRUE	TRUE	TRUE
1	YKTPTLKYF	771	779	9	HLA-A*02:01	771	47	26702.51	47	1.384623	1.112478	-4.4265521	2.497101	-1.9294511	1.38	1.11	-4.43	2.5	-1.93	26702.5	TRUE	TRUE	TRUE	TRUE	TRUE	TRUE
1	SFEIDKGIY	292	300	9	HLA-A*02:01	292	75	36254.25	75	1.281516	1.34663268	-4.5593589	2.628148684	-1.9312102	1.28	1.35	-4.56	2.63	-1.93	36254.2	TRUE	TRUE	TRUE	TRUE	TRUE	TRUE
1	YVPSQERNF	1049	1057	9	HLA-A*02:01	1049	62	32175.1	62	1.402242	1.14895491	-4.5075199	2.55119691	-1.956323	1.4	1.15	-4.51	2.55	-1.96	32175.1	TRUE	TRUE	TRUE	TRUE	TRUE	TRUE
1	TGVIADYNY	402	410	9	HLA-A*02:01	402	63	32654.88	63	1.398027	1.15694339	-4.5139481	2.55497039	-1.9589777	1.4	1.16	-4.51	2.55	-1.96	32654.9	TRUE	TRUE	TRUE	TRUE	TRUE	TRUE
1	QLSSNFGAI	947	955	9	HLA-A*02:01	947	6	1808.71	6	1.009996	0.287436474	-3.2573689	1.297432474	-1.9599365	1.01	0.29	-3.26	1.3	-1.96	1808.7	TRUE	TRUE	TRUE	TRUE	TRUE	TRUE
1	CSCGSCCKF	1230	1238	9	HLA-A*02:01	1230	46	26097.54	46	1.285707	1.16975464	-4.4165996	2.455461642	-1.9611379	1.29	1.17	-4.42	2.46	-1.96	26097.5	TRUE	TRUE	TRUE	TRUE	TRUE	TRUE
1	PYRVVVLSF	493	501	9	HLA-A*02:01	493	41	24115.02	41	1.411832	1.00573496	-4.3822876	2.41756696	-1.9647207	1.41	1.01	-4.38	2.42	-1.96	24115	TRUE	TRUE	TRUE	TRUE	TRUE	TRUE
1	ELDSFKEEL	1126	1134	9	HLA-A*02:01	1126	8.1	3001.36	8.1	1.302767	0.197026	-3.4773181	1.499793001	-1.9775251	1.3	0.2	-3.48	1.5	-1.98	3001.4	TRUE	TRUE	TRUE	TRUE	TRUE	TRUE
1	GIGYQPYRV	488	496	9	HLA-A*02:01	488	5.2	1383.29	5.2	1.115893	0.04644052	-3.1409132	1.162333524	-1.9785797	1.12	0.05	-3.14	1.16	-1.98	1383.3	TRUE	TRUE	TRUE	TRUE	TRUE	TRUE
1	FQTQAGCLI	629	637	9	HLA-A*02:01	629	6.7	2136.71	6.7	1.118635	0.22744332	-3.3297456	1.34607832	-1.9836673	1.12	0.23	-3.33	1.35	-1.98	2136.7	TRUE	TRUE	TRUE	TRUE	TRUE	TRUE
1	VIPFKDGIY	80	88	9	HLA-A*02:01	80	66	33640.38	66	1.257794	1.283009904	-4.5268609	2.540803904	-1.986057	1.26	1.28	-4.53	2.54	-1.99	33640.4	TRUE	TRUE	TRUE	TRUE	TRUE	TRUE
1	QIAPGQTGV	396	404	9	HLA-A*02:01	396	5.4	1487.84	5.4	0.920901	0.26131273	-3.1725562	1.18221373	-1.9903425	0.92	0.26	-3.17	1.18	-1.99	1487.8	TRUE	TRUE	TRUE	TRUE	TRUE	TRUE
1	PLGINITNF	223	231	9	HLA-A*02:01	223	32	18949.15	32	1.420475	0.850036319	-4.2775897	2.270511319	-2.0070784	1.42	0.85	-4.28	2.27	-2.01	18949.2	TRUE	TRUE	TRUE	TRUE	TRUE	TRUE
1	FDNAFNCTF	153	161	9	HLA-A*02:01	153	36	21212.68	36	1.424857	0.888380095	-4.3265955	2.31323709	-2.0133584	1.42	0.89	-4.33	2.31	-2.01	21212.7	TRUE	TRUE	TRUE	TRUE	TRUE	TRUE
1	GIGVTQNVL	890	898	9	HLA-A*02:01	890	23	12465.29	23	1.697265	0.38489423	-4.0957024	2.08215923	-2.0135432	1.7	0.38	-4.1	2.08	-2.01	12465.3	TRUE	TRUE	TRUE	TRUE	TRUE	TRUE
1	QYIKWPWYV	1190	1198	9	HLA-A*02:01	1190	5.6	1620.14	5.6	0.978301	0.21316229	-3.2095525	1.191463293	-2.0180893	0.98	0.21	-3.21	1.19	-2.02	1620.1	TRUE	TRUE	TRUE	TRUE	TRUE	TRUE
1	HTSSMRGVY	33	41	9	HLA-A*02:01	33	85	39053.48	85	1.36849	1.19754863	-4.5916597	2.566038627	-2.0256211	1.37	1.2	-4.59	2.57	-2.03	39053.5	TRUE	TRUE	TRUE	TRUE	TRUE	TRUE
1	VPSGDVVRF	308	316	9	HLA-A*02:01	308	55	29967.95	55	1.431676	1.01292275	-4.476657	2.444598746	-2.0320583	1.43	1.01	-4.48	2.44	-2.03	29968	TRUE	TRUE	TRUE	TRUE	TRUE	TRUE
1	TFEYISDAF	160	168	9	HLA-A*02:01	160	54	29355.3	54	1.336543	1.09763587	-4.4676865	2.43417887	-2.0335077	1.34	1.1	-4.47	2.43	-2.03	29355.3	TRUE	TRUE	TRUE	TRUE	TRUE	TRUE
1	TFGNPVIPF	75	83	9	HLA-A*02:01	75	43	24984.58	43	1.27037	1.08256958	-4.3976721	2.35293958	-2.0447325	1.27	1.08	-4.4	2.35	-2.04	24984.6	TRUE	TRUE	TRUE	TRUE	TRUE	TRUE
1	LLQYGSFCT	735	743	9	HLA-A*02:01	735	2.9	541.11	2.9	0.976286	-0.2893787	-2.7332856	0.686907346	-2.0463782	0.98	-0.29	-2.73	0.69	-2.05	541.1	TRUE	TRUE	TRUE	TRUE	TRUE	TRUE
1	VVNQNAQAL	933	941	9	HLA-A*02:01	933	24	13609.4	24	1.683313	0.402523431	-4.133839	2.08583643	-2.0480025	1.68	0.4	-4.13	2.09	-2.05	13609.4	TRUE	TRUE	TRUE	TRUE	TRUE	TRUE
1	LYLTQDLFL	52	60	9	HLA-A*02:01	52	23	12607.43	23	1.499216	0.545339622	-4.1006266	2.044555622	-2.0560709	1.5	0.55	-4.1	2.04	-2.06	12607.4	TRUE	TRUE	TRUE	TRUE	TRUE	TRUE

seq #	peptide	start	end	peptide length	allele	peptide index	median binding	ann IC50	ann percentile	proteasome sc	tap score	mhc score	processing scor	processing tota	legacy Proteas	legacy TAP Sco	legacy MHC Sc	legacy Process	legacy Total Sc	legacy MHC IC	equal Proteaso	equal TAP Scor	equal MHC Sco	equal Processi	equal Total Sc	equal MHC IC50
1	GFNTLKPIF	212	220	9	HLA-A*02:01	212	70	34840.97	70	1.407576	1.07724377	-4.5420902	2.484819771	-2.0572705	1.41	1.08	-4.54	2.48	-2.06	34841	TRUE	TRUE	TRUE	TRUE	TRUE	TRUE
1	SFKEELDKY	1129	1137	9	HLA-A*02:01	1129	71	35165.92	71	1.181177	1.30428145	-4.546122	2.48545845	-2.0606635	1.18	1.3	-4.55	2.49	-2.06	35165.9	TRUE	TRUE	TRUE	TRUE	TRUE	TRUE
1	QDRNTREVF	756	764	9	HLA-A*02:01	756	84	38725.25	84	1.602401	0.92002811	-4.5879942	2.52242911	-2.0655651	1.6	0.92	-4.59	2.52	-2.07	38725.2	TRUE	TRUE	TRUE	TRUE	TRUE	TRUE
1	GTQTHTMIF	145	153	9	HLA-A*02:01	145	44	25429.95	44	1.315587	1.01394436	-4.4053455	2.329531357	-2.0758141	1.32	1.01	-4.41	2.33	-2.08	25430	TRUE	TRUE	TRUE	TRUE	TRUE	TRUE
1	FSNVYADSF	379	387	9	HLA-A*02:01	379	33	19687.32	33	1.185498	1.0323135	-4.2941866	2.217811504	-2.0763751	1.19	1.03	-4.29	2.22	-2.08	19687.3	TRUE	TRUE	TRUE	TRUE	TRUE	TRUE
1	IDATSTGNY	428	436	9	HLA-A*02:01	428	87	39580.54	87	1.304886	1.214717674	-4.5974817	2.51960367	-2.077878	1.3	1.21	-4.6	2.52	-2.08	39580.5	TRUE	TRUE	TRUE	TRUE	TRUE	TRUE
1	NQKQIANQF	901	909	9	HLA-A*02:01	901	54	29420.82	54	1.311806	1.07497041	-4.4686548	2.386776407	-2.0818784	1.31	1.07	-4.47	2.39	-2.08	29420.8	TRUE	TRUE	TRUE	TRUE	TRUE	TRUE
1	WPLNDYGFY	476	484	9	HLA-A*02:01	476	68	34113.21	68	1.338859	1.11114788	-4.5329226	2.45000688	-2.0829157	1.34	1.11	-4.53	2.45	-2.08	34113.2	TRUE	TRUE	TRUE	TRUE	TRUE	TRUE
1	LVKQLSSNF	944	952	9	HLA-A*02:01	944	55	29725.11	55	1.223801	1.16489914	-4.4731235	2.38870014	-2.0844233	1.22	1.16	-4.47	2.39	-2.08	29725.1	TRUE	TRUE	TRUE	TRUE	TRUE	TRUE
1	GFIAGLIAI	1201	1209	9	HLA-A*02:01	1201	9	3496.47	9	1.180292	0.27722371	-3.5436298	1.45751571	-2.0861141	1.18	0.28	-3.54	1.46	-2.09	3496.5	TRUE	TRUE	TRUE	TRUE	TRUE	TRUE
1	IAAYTAALV	852	860	9	HLA-A*02:01	852	6	1800.7	6	0.975547	0.19095499	-3.2554414	1.16650199	-2.0889394	0.98	0.19	-3.26	1.17	-2.09	1800.7	TRUE	TRUE	TRUE	TRUE	TRUE	TRUE
1	GAEHVDTSY	638	646	9	HLA-A*02:01	638	68	34254.86	68	1.277587	1.155890091	-4.5347222	2.43347709	-2.1012451	1.28	1.16	-4.53	2.43	-2.1	34254.9	TRUE	TRUE	TRUE	TRUE	TRUE	TRUE
1	FKEELDKYF	1130	1138	9	HLA-A*02:01	1130	53	29251.31	53	1.317057	1.04076802	-4.4661453	2.357825023	-2.1083203	1.32	1.04	-4.47	2.36	-2.11	29251.3	TRUE	TRUE	TRUE	TRUE	TRUE	TRUE
1	EIFRSDTLY	45	53	9	HLA-A*02:01	45	63	32632.98	63	1.179508	1.22514214	-4.5136567	2.40465014	-2.1090066	1.18	1.23	-4.51	2.4	-2.11	32633	TRUE	TRUE	TRUE	TRUE	TRUE	TRUE
1	YVTQQLIRA	989	997	9	HLA-A*02:01	989	5.6	1577.6	5.6	1.278887	-0.1914222	-3.1979969	1.087464792	-2.1105321	1.28	-0.19	-3.2	1.09	-2.11	1577.6	TRUE	TRUE	TRUE	TRUE	TRUE	TRUE
1	NITNFRAIL	227	235	9	HLA-A*02:01	227	20	10801.48	20	1.443326	0.4768578	-4.0334833	1.9201838	-2.1132995	1.44	0.48	-4.03	1.92	-2.11	10801.5	TRUE	TRUE	TRUE	TRUE	TRUE	TRUE
1	FLLFLTLTS	4	12	9	HLA-A*02:01	4	1.2	141.86	1.2	1.030455	-0.9967819	-2.15186	0.03367311	-2.1181868	1.03	-1	-2.15	0.03	-2.12	141.9	TRUE	TRUE	TRUE	TRUE	TRUE	TRUE
1	GQTGVIADY	400	408	9	HLA-A*02:01	400	39	22737.81	39	1.091718	1.145577	-4.3567486	2.237294998	-2.1194536	1.09	1.15	-4.36	2.24	-2.12	22737.8	TRUE	TRUE	TRUE	TRUE	TRUE	TRUE
1	PFYSNVTGF	61	69	9	HLA-A*02:01	61	64	32973	64	1.326483	1.06625329	-4.5181585	2.39273629	-2.1254222	1.33	1.07	-4.52	2.39	-2.13	32973	TRUE	TRUE	TRUE	TRUE	TRUE	TRUE
1	QKFNGLTVL	835	843	9	HLA-A*02:01	835	29	16919.84	29	1.533951	0.559081475	-4.2283963	2.09303248	-2.1353638	1.53	0.56	-4.23	2.09	-2.14	16919.8	TRUE	TRUE	TRUE	TRUE	TRUE	TRUE
1	AGAALQIPF	872	880	9	HLA-A*02:01	872	38	22418.29	38	1.168913	1.04073894	-4.3506025	2.20965194	-2.1409505	1.17	1.04	-4.35	2.21	-2.14	22418.3	TRUE	TRUE	TRUE	TRUE	TRUE	TRUE
1	QELGKYEQY	1183	1191	9	HLA-A*02:01	1183	73	35596.19	73	1.164372	1.24520571	-4.5514035	2.409577709	-2.1418258	1.16	1.25	-4.55	2.41	-2.14	35596.2	TRUE	TRUE	TRUE	TRUE	TRUE	TRUE
1	FKNKDGFLY	187	195	9	HLA-A*02:01	187	54	29570.81	54	1.097049	1.2297014	-4.4708632	2.3267504	-2.1441128	1.1	1.23	-4.47	2.33	-2.14	29570.8	TRUE	TRUE	TRUE	TRUE	TRUE	TRUE
1	PALNCYWPL	470	478	9	HLA-A*02:01	470	13	5293.19	13	1.401444	0.175547817	-3.7237175	1.576991817	-2.1467257	1.4	0.18	-3.72	1.58	-2.15	5293.2	TRUE	TRUE	TRUE	TRUE	TRUE	TRUE
1	NASSEVAVL	589	597	9	HLA-A*02:01	589	26	15001.98	26	1.569711	0.45788344	-4.1761486	2.02759444	-2.1485541	1.57	0.46	-4.18	2.03	-2.15	15002	TRUE	TRUE	TRUE	TRUE	TRUE	TRUE
1	NFGAISSVL	951	959	9	HLA-A*02:01	951	38	22196.23	38	1.735776	0.458427125	-4.3462792	2.19420313	-2.1520761	1.74	0.46	-4.35	2.19	-2.15	22196.2	TRUE	TRUE	TRUE	TRUE	TRUE	TRUE
1	CVNFNFNGL	524	532	9	HLA-A*02:01	524	18	8779.02	18	1.39451	0.39651592	-3.943446	1.791025921	-2.1524201	1.39	0.4	-3.94	1.79	-2.15	8779	TRUE	TRUE	TRUE	TRUE	TRUE	TRUE
1	STECANLLL	728	736	9	HLA-A*02:01	728	16	7497.25	16	1.413589	0.30695993	-3.874902	1.720548926	-2.1543531	1.41	0.31	-3.87	1.72	-2.15	7497.2	TRUE	TRUE	TRUE	TRUE	TRUE	TRUE
1	FDDVQAPNY	22	30	9	HLA-A*02:01	22	68	34126.86	68	1.35561	1.02297426	-4.5330963	2.37858426	-2.1545121	1.36	1.02	-4.53	2.38	-2.15	34126.9	TRUE	TRUE	TRUE	TRUE	TRUE	TRUE
1	IDLQELGKY	1180	1188	9	HLA-A*02:01	1180	69	34397.11	69	1.18587	1.174864334	-4.536522	2.36073433	-2.1757876	1.19	1.17	-4.54	2.36	-2.18	34397.1	TRUE	TRUE	TRUE	TRUE	TRUE	TRUE
1	VRFPNITNL	314	322	9	HLA-A*02:01	314	29	16857.71	29	1.447356	0.60190686	-4.2267986	2.04926286	-2.1775357	1.45	0.6	-4.23	2.05	-2.18	16857.7	TRUE	TRUE	TRUE	TRUE	TRUE	TRUE
1	GNYNYKYRY	434	442	9	HLA-A*02:01	434	83	38339.22	83	1.18748	1.21111144	-4.5836433	2.39859144	-2.1850518	1.19	1.21	-4.58	2.4	-2.19	38339.2	TRUE	TRUE	TRUE	TRUE	TRUE	TRUE
1	FVFKNKDGF	185	193	9	HLA-A*02:01	185	36	21125.18	36	1.041524	1.094862	-4.3248004	2.136386	-2.1884144	1.04	1.09	-4.32	2.14	-2.19	21125.2	TRUE	TRUE	TRUE	TRUE	TRUE	TRUE
1	TTGIGYQPY	486	494	9	HLA-A*02:01	486	54	29348.32	54	1.080833	1.184461317	-4.4675832	2.265294317	-2.2022889	1.08	1.18	-4.47	2.27	-2.2	29348.3	TRUE	TRUE	TRUE	TRUE	TRUE	TRUE
1	FSQILPDPL	784	792	9	HLA-A*02:01	784	13	5056.67	13	1.056838	0.44022307	-3.7038646	1.497061072	-2.2068035	1.06	0.44	-3.7	1.5	-2.21	5056.7	TRUE	TRUE	TRUE	TRUE	TRUE	TRUE
1	ATKMSECVL	1008	1016	9	HLA-A*02:01	1008	38	22175.6	38	1.601714	0.53583348	-4.3458754	2.13754748	-2.2083279	1.6	0.54	-4.35	2.14	-2.21	22175.6	TRUE	TRUE	TRUE	TRUE	TRUE	TRUE
1	PLAELKCSV	282	290	9	HLA-A*02:01	282	5.3	1451.87	5.3	0.945257	0.00414688	-3.1619277	0.949403883	-2.2125238	0.95	0	-3.16	0.95	-2.21	1451.9	TRUE	TRUE	TRUE	TRUE	TRUE	TRUE
1	DVSEKSGNF	171	179	9	HLA-A*02:01	171	82	38114.63	82	1.320814	1.04156176	-4.5810917	2.362375756	-2.218716	1.32	1.04	-4.58	2.36	-2.22	38114.6	TRUE	TRUE	TRUE	TRUE	TRUE	TRUE
1	MVTILLCCM	1211	1219	9	HLA-A*02:01	1211	8.4	3125.64	8.4	1.058815	0.217182443	-3.494939	1.27599744	-2.2189415	1.06	0.22	-3.49	1.28	-2.22	3125.6	TRUE	TRUE	TRUE	TRUE	TRUE	TRUE
1	CYGVSATKL	366	374	9	HLA-A*02:01	366	54	29625.88	54	1.762192	0.486357803	-4.4716713	2.248549803	-2.2231215	1.76	0.49	-4.47	2.25	-2.22	29625.9	TRUE	TRUE	TRUE	TRUE	TRUE	TRUE
1	AFNCTFEYI	156	164	9	HLA-A*02:01	156	17	8641.06	17	1.320093	0.38765341	-3.936567	1.70774641	-2.2288206	1.32	0.39	-3.94	1.71	-2.23	8641.1	TRUE	TRUE	TRUE	TRUE	TRUE	TRUE
1	FCTQLNRAL	741	749	9	HLA-A*02:01	741	24	13758.94	24	1.514358	0.39167599	-4.138585	1.90603399	-2.232551	1.51	0.39	-4.14	1.91	-2.23	13758.9	TRUE	TRUE	TRUE	TRUE	TRUE	TRUE
1	NIQKEIDRL	1160	1168	9	HLA-A*02:01	1160	28	15911.67	28	1.471282	0.49474605	-4.2017158	1.966028045	-2.2356877	1.47	0.49	-4.2	1.97	-2.24	15911.7	TRUE	TRUE	TRUE	TRUE	TRUE	TRUE
1	FNATKFPSV	329	337	9	HLA-A*02:01	329	7.6	2673.48	7.6	1.134809	0.05587171	-3.4270769	1.190680713	-2.2363962	1.13	0.06	-3.43	1.19	-2.24	2673.5	TRUE	TRUE	TRUE	TRUE	TRUE	TRUE
1	CASYHTVSL	657	665	9	HLA-A*02:01	657	19	9770.02	19	1.327473	0.42335028	-3.9898955	1.75082328	-2.2390722	1.33	0.42	-3.99	1.75	-2.24	9770	TRUE	TRUE	TRUE	TRUE	TRUE	TRUE
1	ISSVLNDIL	955	963	9	HLA-A*02:01	955	29	16683.15	29	1.472029	0.508827159	-4.2222781	1.98085616	-2.2414219	1.47	0.51	-4.22	1.98	-2.24	16683.2	TRUE	TRUE	TRUE	TRUE	TRUE	TRUE
1	QQLIRAAEI	992	1000	9	HLA-A*02:01	992	13	5078.32	13	1.182431	0.28143479	-3.7057201	1.46386579	-2.2418543	1.18	0.28	-3.71	1.46	-2.24	5078.3	TRUE	TRUE	TRUE	TRUE	TRUE	TRUE
1	VFNGTSWFI	1078	1086	9	HLA-A*02:01	1078	12	4422.26	12	1.154079	0.24497588	-3.6456443	1.399054878	-2.2465894	1.15	0.24	-3.65	1.4	-2.25	4422.3	TRUE	TRUE	TRUE	TRUE	TRUE	TRUE
1	AISSVLNDI	954	962	9	HLA-A*02:01	954	6.1	1829.77	6.1	0.675158	0.334750424	-3.2623965	1.009908424	-2.2524881	0.68	0.33	-3.26	1.01	-2.25	1829.8	TRUE	TRUE	TRUE	TRUE	TRUE	TRUE
1	ICHEGKAYF	1063	1071	9	HLA-A*02:01	1063	43	25121.74	43	0.946316	1.19787614	-4.4000497	2.14419214	-2.2558576	0.95	1.2	-4.4	2.14	-2.26	25121.7	TRUE	TRUE	TRUE	TRUE	TRUE	TRUE
1	IVMVTILLC	1209	1217	9	HLA-A*02:01	1209	7.5	2591.76	7.5	0.977742	0.17057665	-3.4135948	1.14831865	-2.2652761	0.98	0.17	-3.41	1.15	-2.27	2591.8	TRUE	TRUE	TRUE	TRUE	TRUE	TRUE
1	VYYPDEIFR	40	48	9	HLA-A*02:01	40	60	31699.61	60	1.379415	0.85399118	-4.5010539	2.23340618	-2.2676477	1.38	0.85	-4.5	2.23	-2.27	31699.6	TRUE	TRUE	TRUE	TRUE	TRUE	TRUE
1	STAIHADQL	607	615	9	HLA-A*02:01	607	27	15277.66	27	1.458942	0.45741541	-4.1840568	1.916357413	-2.2676994	1.46	0.46	-4.18	1.92	-2.27	15277.7	TRUE	TRUE	TRUE	TRUE	TRUE	TRUE
1	GNFKHLREF	177	185	9	HLA-A*02:01	177	59	31333.05	59	1.200032	1.02767603	-4.4960027	2.227708029	-2.2682946	1.2	1.03	-4.5	2.23	-2.27	31333	TRUE	TRUE	TRUE	TRUE	TRUE	TRUE
1	GKGYHLMSF	1026	1034	9	HLA-A*02:01	1026	52	28695.83	52	1.215889	0.969290027	-4.4578188	2.18517903	-2.2726398	1.22	0.97	-4.46	2.19	-2.27	28695.8	TRUE	TRUE	TRUE	TRUE	TRUE	TRUE
1	AGLIAIVMV	1204	1212	9	HLA-A*02:01	1204	13	5595.06	13	1.379628	0.0941038	-3.7478047	1.4737318	-2.2740729	1.38	0.09	-3.75	1.47	-2.27	5595.1	TRUE	TRUE	TRUE	TRUE	TRUE	TRUE
1	NFELCDNPF	129	137	9	HLA-A*02:01	129	46	26153.79	46	1.017756	1.12333903	-4.4175346	2.14109503	-2.2764396	1.02	1.12	-4.42	2.14	-2.28	26153.8	TRUE	TRUE	TRUE	TRUE	TRUE	TRUE
1	PLQPELDSF	1122	1130	9	HLA-A*02:01	1122	43	25026.51	43	1.295009	0.825477977	-4.3984003	2.120486977	-2.2779133	1.3	0.83	-4.4	2.12	-2.28	25026.5	TRUE	TRUE	TRUE	TRUE	TRUE	TRUE
1	KGIYQTSNF	297	305	9	HLA-A*02:01	297	77	36804.81	77	1.286877	0.999952365	-4.5659046	2.286829365	-2.2790752	1.29	1	-4.57	2.29	-2.28	36804.8	TRUE	TRUE	TRUE	TRUE	TRUE	TRUE
1	AALQIPFAM	874	882	9	HLA-A*02:01	874	7.4	2535.79	7.4	0.905857	0.212388351	-3.4041133	1.11824535	-2.2858679	0.91	0.21	-3.4	1.12	-2.29	2535.8	TRUE	TRUE	TRUE	TRUE	TRUE	TRUE
1	VYDPLQPEL	1119	1127	9	HLA-A*02:01	1119	26	14708.49	26	1.3929	0.486068557	-4.1675681	1.87896856	-2.2885995	1.39	0.49	-4.17	1.88	-2.29	14708.5	TRUE	TRUE	TRUE	TRUE	TRUE	TRUE
1	ICAQKFNGL	832	840	9	HLA-A*02:01	832	27	15524.28	27	1.435741	0.46305422	-4.1910115	1.89879522	-2.2922162	1.44	0.46	-4.19	1.9	-2.29	15524.3	TRUE	TRUE	TRUE	TRUE	TRUE	TRUE
1	LFLPFYSNV	58	66	9	HLA-A*02:01	58	7.3	2506	7.3	0.943593	0.15788258	-3.3989811	1.10147558	-2.2975055	0.94	0.16	-3.4	1.1	-2.3	2506	TRUE	TRUE	TRUE	TRUE	TRUE	TRUE
1	WGTSAAAYF	245	253	9	HLA-A*02:01	245	48	26974.89	48	1.170948	0.960184807	-4.4309597	2.13113281	-2.2998269	1.17	0.96	-4.43	2.13	-2.3	26974.9	TRUE	TRUE	TRUE	TRUE	TRUE	TRUE
1	VAKNLNESL	1171	1179	9	HLA-A*02:01	1171	38	22543.31	38	1.622088	0.39256055	-4.3530177	2.01464855	-2.3383691	1.62	0.39	-4.35	2.01	-2.34	22543.3	TRUE	TRUE	TRUE	TRUE	TRUE	TRUE
1	KSFEIDKGI	291	299	9	HLA-A*02:01	291	16	7219.35	16	1.146921	0.36391219	-3.8584981	1.510833195	-2.3476649	1.15	0.36	-3.86	1.51	-2.35	7219.4	TRUE	TRUE	TRUE	TRUE	TRUE	TRUE
1	YIKWPWYVW	1191	1199	9	HLA-A*02:01	1191	29	17003.34	29	1.468131	0.40819991	-4.2305342	1.876330911	-2.3542033	1.47	0.41	-4.23	1.88	-2.35	17003.3	TRUE	TRUE	TRUE	TRUE	TRUE	TRUE
1	GGFNFSQIL	780	788	9	HLA-A*02:01	780	25	14001.63	25	1.564912	0.225835749	-4.1461786	1.79074775	-2.3554308	1.56	0.23	-4.15	1.79	-2.36	14001.6	TRUE	TRUE	TRUE	TRUE	TRUE	TRUE
1	YNYKLPDDF	408	416	9	HLA-A*02:01	408	58	30823.62	58	1.085707	1.0460352	-4.4888836	2.1317422	-2.3571414	1.09	1.05	-4.49	2.13	-2.36	30823.6	TRUE	TRUE	TRUE	TRUE	TRUE	TRUE
1	IFRSDTLYL	46	54	9	HLA-A*02:01	46	25	14269.57	25	1.348174	0.44768932	-4.1544109	1.79586332	-2.3585476	1.35	0.45	-4.15	1.8	-2.36	14269.6	TRUE	TRUE	TRUE	TRUE	TRUE	TRUE
1	TTSTALGKL	922	930	9	HLA-A*02:01	922	34	19868.36	34	1.537884	0.400015803	-4.298162	1.937899803	-2.3602622	1.54	0.4	-4.3	1.94	-2.36	19868.4	TRUE	TRUE	TRUE	TRUE	TRUE	TRUE
1	LLLQYGSFC	734	742	9	HLA-A*02:01	734	7.7	2742.92	7.7	0.990096	0.08561687	-3.4382131	1.07571287	-2.3625003	0.99	0.09	-3.44	1.08	-2.36	2742.9	TRUE	TRUE	TRUE	TRUE	TRUE	TRUE
1	QFGRDVSDF	550	558	9	HLA-A*02:01	550	77	36760.24	77	1.113745	1.08622025	-4.5653783	2.19996525	-2.3654131	1.11	1.09	-4.57	2.2	-2.37	36760.2	TRUE	TRUE	TRUE	TRUE	TRUE	TRUE
1	YRYLRHGKL	440	448	9	HLA-A*02:01	440	50	28152.73	50	1.455551	0.62239158	-4.4495205	2.077942581	-2.3715779	1.46	0.62	-4.45	2.08	-2.37	28152.7	TRUE	TRUE	TRUE	TRUE	TRUE	TRUE
1	FKLPLGINI	220	228	9	HLA-A*02:01	220	15	6821.73	15	1.238304	0.22357762	-3.8338945	1.46188162	-2.3720129	1.24	0.22	-3.83	1.46	-2.37	6821.7	TRUE	TRUE	TRUE	TRUE	TRUE	TRUE
1	AEIRASANL	998	1006	9	HLA-A*02:01	998	53	29065.5	53	1.569233	0.51729548	-4.4633778	2.086528479	-2.3768493	1.57	0.52	-4.46	2.09	-2.38	29065.5	TRUE	TRUE	TRUE	TRUE	TRUE	TRUE
1	NGLTVLPPL	838	846	9	HLA-A*02:01	838	14	6240.94	14	1.135475	0.279433108	-3.79525	1.414908108	-2.3803419	1.14	0.28	-3.8	1.41	-2.38	6240.9	TRUE	TRUE	TRUE	TRUE	TRUE	TRUE
1	GCVLAWNTR	418	426	9	HLA-A*02:01	418	47	26795.69	47	1.422851	0.623928367	-4.4280649	2.04677937	-2.3812856	1.42	0.62	-4.43	2.05	-2.38	26795.7	TRUE	TRUE	TRUE	TRUE	TRUE	TRUE
1	NYNYKYRYL	435	443	9	HLA-A*02:01	435	60	31686.25	60	1.610486	0.49388584	-4.5008708	2.10437184	-2.396499	1.61	0.49	-4.5	2.1	-2.4	31686.2	TRUE	TRUE	TRUE	TRUE	TRUE	TRUE
1	NCVADYSVL	347	355	9	HLA-A*02:01	347	47	26658.34	47	1.529555	0.49835882	-4.4258331	2.02791382	-2.3979193	1.53	0.5	-4.43	2.03	-2.4	26658.3	TRUE	TRUE	TRUE	TRUE	TRUE	TRUE
1	ERDISNVPF	452	460	9	HLA-A*02:01	452	61	32045.86	61	1.119876	0.97862703	-4.5057719	2.09850303	-2.4072689	1.12	0.98	-4.51	2.1	-2.41	32045.9	TRUE	TRUE	TRUE	TRUE	TRUE	TRUE
1	LKPIFKLPL	216	224	9	HLA-A*02:01	216	28	15981.2	28	1.357731	0.437543122	-4.2036094	1.795274122	-2.4083353	1.36	0.44	-4.2	1.8	-2.41	15981.2	TRUE	TRUE	TRUE	TRUE	TRUE	TRUE
1	CSFGGVSVI	576	584	9	HLA-A*02:01	576	15	7045.19	15	1.222172	0.21623879	-3.8478927	1.438410791	-2.4094819	1.22	0.22	-3.85	1.44	-2.41	7045.2	TRUE	TRUE	TRUE	TRUE	TRUE	TRUE
1	QYGSFCTQL	737	745	9	HLA-A*02:01	737	38	22157.85	38	1.44708	0.487125169	-4.3455276	1.934205169	-2.4113224	1.45	0.49	-4.35	1.93	-2.41	22157.8	TRUE	TRUE	TRUE	TRUE	TRUE	TRUE
1	DFMGCVLAW	415	423	9	HLA-A*02:01	415	34	20000.57	34	1.591448	0.28677273	-4.3010424	1.87822073	-2.4228216	1.59	0.29	-4.3	1.88	-2.42	20000.6	TRUE	TRUE	TRUE	TRUE	TRUE	TRUE
1	YFPREGVFV	1070	1078	9	HLA-A*02:01	1070	18	8723.82	18	1.256452	0.26030467	-3.9407067	1.516756672	-2.42395	1.26	0.26	-3.94	1.52	-2.42	8723.8	TRUE	TRUE	TRUE	TRUE	TRUE	TRUE
1	LGQSKRVDF	1016	1024	9	HLA-A*02:01	1016	75	36146.53	75	1.135349	0.991972466	-4.5580666	2.127321466	-2.4307451	1.14	0.99	-4.56	2.13	-2.43	36146.5	TRUE	TRUE	TRUE	TRUE	TRUE	TRUE
1	GEVFNATKF	326	334	9	HLA-A*02:01	326	67	33991.99	67	1.148715	0.95091232	-4.5313766	2.099627316	-2.4317493	1.15	0.95	-4.53	2.1	-2.43	33992	TRUE	TRUE	TRUE	TRUE	TRUE	TRUE
1	ESLIDLQEL	1177	1185	9	HLA-A*02:01	1177	32	18638.46	32	1.424032	0.41442275	-4.27041	1.838454746	-2.4319553	1.42	0.41	-4.27	1.84	-2.43	18638.5	TRUE	TRUE	TRUE	TRUE	TRUE	TRUE
1	LRHGKLRPF	443	451	9	HLA-A*02:01	443	54	29702.92	54	0.858994	1.18150149	-4.4727991	2.04049549	-2.4323037	0.86	1.18	-4.47	2.04	-2.43	29702.9	TRUE	TRUE	TRUE	TRUE	TRUE	TRUE
1	PTLKYFGGF	774	782	9	HLA-A*02:01	774	63	32705.44	63	1.187465	0.89377499	-4.51462	2.081239994	-2.43338	1.19	0.89	-4.51	2.08	-2.43	32705.4	TRUE	TRUE	TRUE	TRUE	TRUE	TRUE
1	CSQNPLAEL	278	286	9	HLA-A*02:01	278	24	13747.62	24	1.362391	0.34145627	-4.1382275	1.70384727	-2.4343802	1.36	0.34	-4.14	1.7	-2.43	13747.6	TRUE	TRUE	TRUE	TRUE	TRUE	TRUE
1	SYHTVSLLR	659	667	9	HLA-A*02:01	659	47	26881.06	47	1.125099	0.861470038	-4.4294464	1.986569038	-2.4428774	1.13	0.86	-4.43	1.99	-2.44	26881.1	TRUE	TRUE	TRUE	TRUE	TRUE	TRUE
1	MSLGADSSI	679	687	9	HLA-A*02:01	679	16	7625.78	16	1.158075	0.2811132	-3.8822843	1.4391882	-2.4430961	1.16	0.28	-3.88	1.44	-2.44	7625.8	TRUE	TRUE	TRUE	TRUE	TRUE	TRUE
1	VLYQDVNCT	596	604	9	HLA-A*02:01	596	5.3	1463.83	5.3	0.838016	-0.1229843	-3.1654906	0.715031691	-2.450459	0.84	-0.12	-3.17	0.72	-2.45	1463.8	TRUE	TRUE	TRUE	TRUE	TRUE	TRUE
1	QTYVTQQLI	987	995	9	HLA-A*02:01	987	19	9449.7	19	1.190571	0.329616	-3.975418	1.520187	-2.455231	1.19	0.33	-3.98	1.52	-2.46	9449.7	TRUE	TRUE	TRUE	TRUE	TRUE	TRUE
1	KEIDRLNEV	1163	1171	9	HLA-A*02:01	1163	14	6029.01	14	1.191037	0.1314414	-3.780246	1.3224784	-2.4577676	1.19	0.13	-3.78	1.32	-2.46	6029	TRUE	TRUE	TRUE	TRUE	TRUE	TRUE
1	LCDNPFFAV	132	140	9	HLA-A*02:01	132	6.9	2259.03	6.9	0.894296	-0.0032324	-3.353922	0.89106363	-2.4628584	0.89	0	-3.35	0.89	-2.46	2259	TRUE	TRUE	TRUE	TRUE	TRUE	TRUE
1	SVLNDILSR	957	965	9	HLA-A*02:01	957	33	19677.1	33	1.071495	0.758153792	-4.2939611	1.82964879	-2.4643123	1.07	0.76	-4.29	1.83	-2.46	19677.1	TRUE	TRUE	TRUE	TRUE	TRUE	TRUE
1	VSGNCDVVI	1104	1112	9	HLA-A*02:01	1104	26	14932.5	26	1.546878	0.15939478	-4.1741325	1.706272778	-2.4678597	1.55	0.16	-4.17	1.71	-2.47	14932.5	TRUE	TRUE	TRUE	TRUE	TRUE	TRUE
1	YLTQDLFLP	53	61	9	HLA-A*02:01	53	4.2	975.48	4.2	0.457004	0.0493491	-2.9892184	0.5063531	-2.4828653	0.46	0.05	-2.99	0.51	-2.48	975.5	TRUE	TRUE	TRUE	TRUE	TRUE	TRUE
1	VVPSGDVVR	307	315	9	HLA-A*02:01	307	74	35980.7	74	1.283964	0.78215463	-4.5560696	2.066118633	-2.489951	1.28	0.78	-4.56	2.07	-2.49	35980.7	TRUE	TRUE	TRUE	TRUE	TRUE	TRUE
1	DVSDFTDSV	554	562	9	HLA-A*02:01	554	17	7938.29	17	1.286061	0.1137182	-3.899727	1.3997792	-2.4999478	1.29	0.11	-3.9	1.4	-2.5	7938.3	TRUE	TRUE	TRUE	TRUE	TRUE	TRUE
1	PIFKLPLGI	218	226	9	HLA-A*02:01	218	18	9026.57	18	1.284304	0.17084947	-3.9555228	1.455153469	-2.5003693	1.28	0.17	-3.96	1.46	-2.5	9026.6	TRUE	TRUE	TRUE	TRUE	TRUE	TRUE
1	SVVNIQKEI	1157	1165	9	HLA-A*02:01	1157	21	11039.07	21	1.126516	0.414813452	-4.0429325	1.541329452	-2.501603	1.13	0.41	-4.04	1.54	-2.5	11039.1	TRUE	TRUE	TRUE	TRUE	TRUE	TRUE
1	DFCGKGYHL	1023	1031	9	HLA-A*02:01	1023	54	29661.17	54	1.594187	0.37617587	-4.4721883	1.97036287	-2.5018254	1.59	0.38	-4.47	1.97	-2.5	29661.2	TRUE	TRUE	TRUE	TRUE	TRUE	TRUE
1	EKSGNFKHL	174	182	9	HLA-A*02:01	174	77	36853.81	77	1.684171	0.37053666	-4.5664824	2.05470766	-2.5117747	1.68	0.37	-4.57	2.05	-2.51	36853.8	TRUE	TRUE	TRUE	TRUE	TRUE	TRUE
1	VFVFNGTSW	1076	1084	9	HLA-A*02:01	1076	55	30048.8	55	1.461418	0.50385644	-4.4778271	1.96527444	-2.5125527	1.46	0.5	-4.48	1.97	-2.51	30048.8	TRUE	TRUE	TRUE	TRUE	TRUE	TRUE
1	YGFYTTTGI	481	489	9	HLA-A*02:01	481	12	4807.2	12	1.073963	0.09522672	-3.6818922	1.16918972	-2.5127025	1.07	0.1	-3.68	1.17	-2.51	4807.2	TRUE	TRUE	TRUE	TRUE	TRUE	TRUE
1	QLIRAAEIR	993	1001	9	HLA-A*02:01	993	44	25251.46	44	1.219908	0.66707104	-4.4022865	1.88697904	-2.5153075	1.22	0.67	-4.4	1.89	-2.52	25251.5	TRUE	TRUE	TRUE	TRUE	TRUE	TRUE
1	AFSPAQDIW	237	245	9	HLA-A*02:01	237	76	36447.77	76	1.516929	0.521327735	-4.561671	2.03825674	-2.5234142	1.52	0.52	-4.56	2.04	-2.52	36447.8	TRUE	TRUE	TRUE	TRUE	TRUE	TRUE
1	PLKPTKRSF	791	799	9	HLA-A*02:01	791	77	36857.81	77	1.201945	0.838585176	-4.5665295	2.04053018	-2.5259994	1.2	0.84	-4.57	2.04	-2.53	36857.8	TRUE	TRUE	TRUE	TRUE	TRUE	TRUE
1	VFQTQAGCL	628	636	9	HLA-A*02:01	628	63	32732	63	1.419336	0.56373695	-4.5149725	1.983072948	-2.5318996	1.42	0.56	-4.51	1.98	-2.53	32732	TRUE	TRUE	TRUE	TRUE	TRUE	TRUE
1	YTAALVSGT	855	863	9	HLA-A*02:01	855	6.3	1924.22	6.3	0.996467	-0.2506145	-3.2842547	0.74585249	-2.5384022	1	-0.25	-3.28	0.75	-2.54	1924.2	TRUE	TRUE	TRUE	TRUE	TRUE	TRUE
1	TYVTQQLIR	988	996	9	HLA-A*02:01	988	90	40441.99	90	1.301911	0.76192121	-4.6068325	2.06383221	-2.5430003	1.3	0.76	-4.61	2.06	-2.54	40442	TRUE	TRUE	TRUE	TRUE	TRUE	TRUE
1	KRSFIEDLL	796	804	9	HLA-A*02:01	796	42	24463.23	42	1.274736	0.554575973	-4.3885138	1.829311973	-2.5592018	1.27	0.55	-4.39	1.83	-2.56	24463.2	TRUE	TRUE	TRUE	TRUE	TRUE	TRUE
1	CTFEYISDA	159	167	9	HLA-A*02:01	159	5.4	1478.53	5.4	0.748467	-0.1486665	-3.1698301	0.59980054	-2.5700296	0.75	-0.15	-3.17	0.6	-2.57	1478.5	TRUE	TRUE	TRUE	TRUE	TRUE	TRUE
1	NNSTNVVIR	118	126	9	HLA-A*02:01	118	77	36860.99	77	1.394894	0.59648686	-4.566567	1.991380862	-2.5751861	1.39	0.6	-4.57	1.99	-2.58	36861	TRUE	TRUE	TRUE	TRUE	TRUE	TRUE
1	RVVPSGDVV	306	314	9	HLA-A*02:01	306	22	11982.21	22	1.212802	0.289776936	-4.0785369	1.50257894	-2.575958	1.21	0.29	-4.08	1.5	-2.58	11982.2	TRUE	TRUE	TRUE	TRUE	TRUE	TRUE
1	LGINITNFR	224	232	9	HLA-A*02:01	224	55	29924.53	55	1.401179	0.497127896	-4.4760273	1.898306896	-2.5777204	1.4	0.5	-4.48	1.9	-2.58	29924.5	TRUE	TRUE	TRUE	TRUE	TRUE	TRUE
1	NTREVFAQV	759	767	9	HLA-A*02:01	759	13	5448.1	13	0.934669	0.2207911	-3.7362451	1.1554601	-2.580785	0.93	0.22	-3.74	1.16	-2.58	5448.1	TRUE	TRUE	TRUE	TRUE	TRUE	TRUE
1	LLCCMTSCC	1215	1223	9	HLA-A*02:01	1215	13	5179.09	13	1.10762	0.02401792	-3.7142535	1.13163792	-2.5826155	1.11	0.02	-3.71	1.13	-2.58	5179.1	TRUE	TRUE	TRUE	TRUE	TRUE	TRUE
1	VSATKLNDL	369	377	9	HLA-A*02:01	369	33	19298.01	33	1.245325	0.4549617	-4.2855125	1.7002867	-2.5852258	1.25	0.45	-4.29	1.7	-2.59	19298	TRUE	TRUE	TRUE	TRUE	TRUE	TRUE
1	GAALQIPFA	873	881	9	HLA-A*02:01	873	12	4492.48	12	1.279059	-0.2123347	-3.6524862	1.06672435	-2.5857618	1.28	-0.21	-3.65	1.07	-2.59	4492.5	TRUE	TRUE	TRUE	TRUE	TRUE	TRUE
1	IAGLIAIVM	1203	1211	9	HLA-A*02:01	1203	18	9186.67	18	1.328454	0.04851654	-3.9631581	1.37697054	-2.5861876	1.33	0.05	-3.96	1.38	-2.59	9186.7	TRUE	TRUE	TRUE	TRUE	TRUE	TRUE
1	NHTSPDVDL	1140	1148	9	HLA-A*02:01	1140	79	37318.09	79	1.520887	0.463856103	-4.5719194	1.9847431	-2.5871763	1.52	0.46	-4.57	1.98	-2.59	37318.1	TRUE	TRUE	TRUE	TRUE	TRUE	TRUE
1	QNAQALNTL	936	944	9	HLA-A*02:01	936	45	25973.03	45	1.400055	0.424709102	-4.4145226	1.824764102	-2.5897585	1.4	0.42	-4.41	1.82	-2.59	25973	TRUE	TRUE	TRUE	TRUE	TRUE	TRUE
1	GYQPIDVVR	199	207	9	HLA-A*02:01	199	69	34494.39	69	1.236541	0.71055893	-4.5377485	1.947099931	-2.5906485	1.24	0.71	-4.54	1.95	-2.59	34494.4	TRUE	TRUE	TRUE	TRUE	TRUE	TRUE
1	HADQLTPAW	611	619	9	HLA-A*02:01	611	58	30797.97	58	1.60361	0.294113538	-4.4885221	1.897723538	-2.5907986	1.6	0.29	-4.49	1.9	-2.59	30798	TRUE	TRUE	TRUE	TRUE	TRUE	TRUE
1	GKPCTPPAL	464	472	9	HLA-A*02:01	464	50	27833.79	50	1.559801	0.293194722	-4.4445723	1.852995722	-2.5915766	1.56	0.29	-4.44	1.85	-2.59	27833.8	TRUE	TRUE	TRUE	TRUE	TRUE	TRUE
1	VFKNKDGFL	186	194	9	HLA-A*02:01	186	72	35311.93	72	1.459256	0.469949608	-4.5479215	1.92920561	-2.6187158	1.46	0.47	-4.55	1.93	-2.62	35311.9	TRUE	TRUE	TRUE	TRUE	TRUE	TRUE
1	TKFPSVYAW	332	340	9	HLA-A*02:01	332	47	26960.59	47	1.29932	0.51106553	-4.4307294	1.810385525	-2.6203439	1.3	0.51	-4.43	1.81	-2.62	26960.6	TRUE	TRUE	TRUE	TRUE	TRUE	TRUE
1	GTSWFITQR	1081	1089	9	HLA-A*02:01	1081	39	22934.24	39	1.121831	0.61618453	-4.3604844	1.73801553	-2.6224688	1.12	0.62	-4.36	1.74	-2.62	22934.2	TRUE	TRUE	TRUE	TRUE	TRUE	TRUE
1	RDPKTSEIL	563	571	9	HLA-A*02:01	563	67	33810.05	67	1.502061	0.40293201	-4.5290458	1.90499301	-2.6240528	1.5	0.4	-4.53	1.9	-2.62	33810.1	TRUE	TRUE	TRUE	TRUE	TRUE	TRUE
1	KLRPFERDI	447	455	9	HLA-A*02:01	447	18	9035.84	18	1.025648	0.29424902	-3.9559685	1.319897018	-2.6360715	1.03	0.29	-3.96	1.32	-2.64	9035.8	TRUE	TRUE	TRUE	TRUE	TRUE	TRUE
1	EPVLKGVKL	1244	1252	9	HLA-A*02:01	1244	59	31296.46	59	1.601173	0.25258779	-4.4954952	1.853760793	-2.6417344	1.6	0.25	-4.5	1.85	-2.64	31296.5	TRUE	TRUE	TRUE	TRUE	TRUE	TRUE
1	ISDAFSLDV	164	172	9	HLA-A*02:01	164	8.4	3153.15	8.4	0.789526	0.06373101	-3.4987446	0.853257013	-2.6454876	0.79	0.06	-3.5	0.85	-2.65	3153.2	TRUE	TRUE	TRUE	TRUE	TRUE	TRUE
1	AYSNNTIAI	688	696	9	HLA-A*02:01	688	36	20948.33	36	1.263951	0.41099819	-4.3211494	1.674949192	-2.6462002	1.26	0.41	-4.32	1.67	-2.65	20948.3	TRUE	TRUE	TRUE	TRUE	TRUE	TRUE
1	PATVCGPKL	507	515	9	HLA-A*02:01	507	72	35258.12	72	1.618391	0.28197671	-4.5472592	1.900367706	-2.6468914	1.62	0.28	-4.55	1.9	-2.65	35258.1	TRUE	TRUE	TRUE	TRUE	TRUE	TRUE
1	GIYQTSNFR	298	306	9	HLA-A*02:01	298	39	22662.42	39	0.956903	0.746724285	-4.3553063	1.70362728	-2.651679	0.96	0.75	-4.36	1.7	-2.65	22662.4	TRUE	TRUE	TRUE	TRUE	TRUE	TRUE
1	KGYQPIDVV	198	206	9	HLA-A*02:01	198	26	15107.21	26	1.399044	0.124563623	-4.1791843	1.52360762	-2.6555766	1.4	0.12	-4.18	1.52	-2.66	15107.2	TRUE	TRUE	TRUE	TRUE	TRUE	TRUE
1	PNITNLCPF	317	325	9	HLA-A*02:01	317	58	30830.97	58	1.023694	0.809689795	-4.4889872	1.83338379	-2.6556034	1.02	0.81	-4.49	1.83	-2.66	30831	TRUE	TRUE	TRUE	TRUE	TRUE	TRUE
1	EYISDAFSL	162	170	9	HLA-A*02:01	162	42	24469.58	42	1.305808	0.42719034	-4.3886265	1.73299834	-2.6556282	1.31	0.43	-4.39	1.73	-2.66	24469.6	TRUE	TRUE	TRUE	TRUE	TRUE	TRUE
1	ELGKYEQYI	1184	1192	9	HLA-A*02:01	1184	12	4787.32	12	0.962231	0.05921207	-3.6800925	1.021443072	-2.6586494	0.96	0.06	-3.68	1.02	-2.66	4787.3	TRUE	TRUE	TRUE	TRUE	TRUE	TRUE
1	LLFLTLTSG	5	13	9	HLA-A*02:01	5	5.7	1676.81	5.7	1.15504	-0.5914806	-3.2244839	0.563559423	-2.6609244	1.16	-0.59	-3.22	0.56	-2.66	1676.8	TRUE	TRUE	TRUE	TRUE	TRUE	TRUE
1	GSFCTQLNR	739	747	9	HLA-A*02:01	739	53	29050.72	53	1.190039	0.60960467	-4.4631569	1.799643666	-2.6635132	1.19	0.61	-4.46	1.8	-2.66	29050.7	TRUE	TRUE	TRUE	TRUE	TRUE	TRUE
1	ATEKSNVVR	91	99	9	HLA-A*02:01	91	84	38828.49	84	1.218391	0.70170147	-4.5891505	1.920092472	-2.669058	1.22	0.7	-4.59	1.92	-2.67	38828.5	TRUE	TRUE	TRUE	TRUE	TRUE	TRUE
1	IAPGQTGVI	397	405	9	HLA-A*02:01	397	50	28014.16	50	1.541604	0.23624863	-4.4473776	1.77785263	-2.669525	1.54	0.24	-4.45	1.78	-2.67	28014.2	TRUE	TRUE	TRUE	TRUE	TRUE	TRUE
1	FVVKGDDVR	387	395	9	HLA-A*02:01	387	72	35416.78	72	1.149755	0.72015611	-4.5492091	1.86991111	-2.679298	1.15	0.72	-4.55	1.87	-2.68	35416.8	TRUE	TRUE	TRUE	TRUE	TRUE	TRUE
1	LKYDENGTI	264	272	9	HLA-A*02:01	264	51	28355.41	51	1.417286	0.35154373	-4.4526359	1.76882973	-2.6838062	1.42	0.35	-4.45	1.77	-2.68	28355.4	TRUE	TRUE	TRUE	TRUE	TRUE	TRUE
1	NAQALNTLV	937	945	9	HLA-A*02:01	937	15	6555.94	15	0.980289	0.13908582	-3.816635	1.119374818	-2.6972602	0.98	0.14	-3.82	1.12	-2.7	6555.9	TRUE	TRUE	TRUE	TRUE	TRUE	TRUE
1	FIEDLLFNK	799	807	9	HLA-A*02:01	799	17	8286.79	17	1.042804	0.17798078	-3.9183863	1.22078478	-2.6976016	1.04	0.18	-3.92	1.22	-2.7	8286.8	TRUE	TRUE	TRUE	TRUE	TRUE	TRUE
1	SNVTGFHTI	64	72	9	HLA-A*02:01	64	36	21127.24	36	1.42135	0.19922962	-4.3248428	1.62057962	-2.7042631	1.42	0.2	-4.32	1.62	-2.7	21127.2	TRUE	TRUE	TRUE	TRUE	TRUE	TRUE
1	AATEKSNVV	90	98	9	HLA-A*02:01	90	22	11753.9	22	1.205417	0.15966508	-4.070182	1.365082076	-2.7050999	1.21	0.16	-4.07	1.37	-2.71	11753.9	TRUE	TRUE	TRUE	TRUE	TRUE	TRUE
1	YICGDSTEC	723	731	9	HLA-A*02:01	723	20	10316.7	20	1.232386	0.07219555	-4.0135408	1.304581553	-2.7089592	1.23	0.07	-4.01	1.3	-2.71	10316.7	TRUE	TRUE	TRUE	TRUE	TRUE	TRUE
1	STFFSTFKC	358	366	9	HLA-A*02:01	358	16	7194.17	16	1.017605	0.129132473	-3.8569807	1.14673747	-2.7102432	1.02	0.13	-3.86	1.15	-2.71	7194.2	TRUE	TRUE	TRUE	TRUE	TRUE	TRUE
1	VYADSFVVK	382	390	9	HLA-A*02:01	382	67	33936.86	67	1.403336	0.41306042	-4.5306717	1.81639642	-2.7142752	1.4	0.41	-4.53	1.82	-2.71	33936.9	TRUE	TRUE	TRUE	TRUE	TRUE	TRUE
1	PWYVWLGFI	1195	1203	9	HLA-A*02:01	1195	26	15025.04	26	1.275957	0.1863973	-4.1768156	1.462354295	-2.7144613	1.28	0.19	-4.18	1.46	-2.71	15025	TRUE	TRUE	TRUE	TRUE	TRUE	TRUE
1	VTYVPSQER	1047	1055	9	HLA-A*02:01	1047	66	33633.46	66	1.07244	0.732781266	-4.5267715	1.805221266	-2.7215503	1.07	0.73	-4.53	1.81	-2.72	33633.5	TRUE	TRUE	TRUE	TRUE	TRUE	TRUE
1	EAEVQIDRL	970	978	9	HLA-A*02:01	970	61	31976.93	61	1.470504	0.3081231	-4.5048368	1.778627101	-2.7262097	1.47	0.31	-4.5	1.78	-2.73	31976.9	TRUE	TRUE	TRUE	TRUE	TRUE	TRUE
1	IDRLITGRL	975	983	9	HLA-A*02:01	975	46	26304.77	46	1.396065	0.29248197	-4.4200345	1.688546973	-2.7314875	1.4	0.29	-4.42	1.69	-2.73	26304.8	TRUE	TRUE	TRUE	TRUE	TRUE	TRUE
1	PDDFMGCVL	413	421	9	HLA-A*02:01	413	64	32969.43	64	1.766087	0.018860705	-4.5181114	1.78494771	-2.7331637	1.77	0.02	-4.52	1.78	-2.73	32969.4	TRUE	TRUE	TRUE	TRUE	TRUE	TRUE
1	GKYEQYIKW	1186	1194	9	HLA-A*02:01	1186	62	32395.51	62	1.335214	0.42888572	-4.5104848	1.76409972	-2.7463851	1.34	0.43	-4.51	1.76	-2.75	32395.5	TRUE	TRUE	TRUE	TRUE	TRUE	TRUE
1	VSGTATAGW	860	868	9	HLA-A*02:01	860	64	32971.21	64	1.391631	0.37788363	-4.5181349	1.76951463	-2.7486203	1.39	0.38	-4.52	1.77	-2.75	32971.2	TRUE	TRUE	TRUE	TRUE	TRUE	TRUE
1	QKSIVAYTM	671	679	9	HLA-A*02:01	671	32	18678.62	32	1.341474	0.1804416	-4.2713448	1.521915595	-2.7494292	1.34	0.18	-4.27	1.52	-2.75	18678.6	TRUE	TRUE	TRUE	TRUE	TRUE	TRUE
1	KLPLGINIT	221	229	9	HLA-A*02:01	221	8.5	3200.59	8.5	1.057985	-0.3036833	-3.50523	0.754301695	-2.7509283	1.06	-0.3	-3.51	0.75	-2.75	3200.6	TRUE	TRUE	TRUE	TRUE	TRUE	TRUE
1	DEDDSEPVL	1239	1247	9	HLA-A*02:01	1239	86	39362.72	86	1.744389	0.09839206	-4.5950851	1.84278106	-2.752304	1.74	0.1	-4.6	1.84	-2.75	39362.7	TRUE	TRUE	TRUE	TRUE	TRUE	TRUE
1	FGAGAALQI	870	878	9	HLA-A*02:01	870	20	10636.91	20	1.181447	0.08584543	-4.0268155	1.267292431	-2.7595231	1.18	0.09	-4.03	1.27	-2.76	10636.9	TRUE	TRUE	TRUE	TRUE	TRUE	TRUE
1	NGLTGTGVL	530	538	9	HLA-A*02:01	530	63	32558.56	63	1.473345	0.279433108	-4.5126652	1.752778108	-2.7598871	1.47	0.28	-4.51	1.75	-2.76	32558.6	TRUE	TRUE	TRUE	TRUE	TRUE	TRUE
1	SISITTEVM	701	709	9	HLA-A*02:01	701	18	9359.54	18	1.046933	0.16321488	-3.9712545	1.21014788	-2.7611066	1.05	0.16	-3.97	1.21	-2.76	9359.5	TRUE	TRUE	TRUE	TRUE	TRUE	TRUE
1	GCLIGAEHV	634	642	9	HLA-A*02:01	634	20	10325.63	20	1.111764	0.14074292	-4.0139166	1.252506915	-2.7614096	1.11	0.14	-4.01	1.25	-2.76	10325.6	TRUE	TRUE	TRUE	TRUE	TRUE	TRUE
1	KAYFPREGV	1068	1076	9	HLA-A*02:01	1068	19	9501.68	19	0.943539	0.27256929	-3.9778004	1.216108288	-2.7616921	0.94	0.27	-3.98	1.22	-2.76	9501.7	TRUE	TRUE	TRUE	TRUE	TRUE	TRUE
1	SLGADSSIA	680	688	9	HLA-A*02:01	680	9.7	3949.05	9.7	1.086823	-0.2548506	-3.5964926	0.831972365	-2.7645203	1.09	-0.25	-3.6	0.83	-2.76	3949.1	TRUE	TRUE	TRUE	TRUE	TRUE	TRUE
1	GNCDVVIGI	1106	1114	9	HLA-A*02:01	1106	23	12675.55	23	1.218044	0.11430901	-4.1029668	1.33235301	-2.7706138	1.22	0.11	-4.1	1.33	-2.77	12675.5	TRUE	TRUE	TRUE	TRUE	TRUE	TRUE
1	IPTNFSISI	696	704	9	HLA-A*02:01	696	29	16964.38	29	1.294703	0.160308296	-4.229538	1.455011296	-2.7745267	1.29	0.16	-4.23	1.46	-2.77	16964.4	TRUE	TRUE	TRUE	TRUE	TRUE	TRUE
1	VAYTMSLGA	675	683	9	HLA-A*02:01	675	12	4409.45	12	0.96601	-0.0963957	-3.6443844	0.869614263	-2.7747702	0.97	-0.1	-3.64	0.87	-2.77	4409.4	TRUE	TRUE	TRUE	TRUE	TRUE	TRUE
1	SNFGAISSV	950	958	9	HLA-A*02:01	950	14	6207.81	14	0.851479	0.16409474	-3.7929384	1.01557374	-2.7773647	0.85	0.16	-3.79	1.02	-2.78	6207.8	TRUE	TRUE	TRUE	TRUE	TRUE	TRUE
1	KMSECVLGQ	1010	1018	9	HLA-A*02:01	1010	9.8	4047.12	9.8	0.772917	0.05397584	-3.6071461	0.826892841	-2.7802532	0.77	0.05	-3.61	0.83	-2.78	4047.1	TRUE	TRUE	TRUE	TRUE	TRUE	TRUE
1	KCSVKSFEI	287	295	9	HLA-A*02:01	287	20	10279.26	20	0.924707	0.30516415	-4.0119619	1.229871149	-2.7820907	0.92	0.31	-4.01	1.23	-2.78	10279.3	TRUE	TRUE	TRUE	TRUE	TRUE	TRUE
1	RNFTTAPAI	1055	1063	9	HLA-A*02:01	1055	35	20541.18	35	1.281342	0.24742039	-4.3126254	1.528762394	-2.783863	1.28	0.25	-4.31	1.53	-2.78	20541.2	TRUE	TRUE	TRUE	TRUE	TRUE	TRUE
1	PSSKRFQPF	540	548	9	HLA-A*02:01	540	70	34641.36	70	0.844586	0.910741863	-4.5395949	1.75532786	-2.7842671	0.84	0.91	-4.54	1.76	-2.78	34641.4	TRUE	TRUE	TRUE	TRUE	TRUE	TRUE
1	RYLRHGKLR	441	449	9	HLA-A*02:01	441	95	42373.53	95	0.991002	0.850499743	-4.6270946	1.841501743	-2.7855929	0.99	0.85	-4.63	1.84	-2.79	42373.5	TRUE	TRUE	TRUE	TRUE	TRUE	TRUE
1	SVDCNMYIC	717	725	9	HLA-A*02:01	717	22	11729.76	22	1.266622	0.016285145	-4.0692891	1.282907144	-2.786382	1.27	0.02	-4.07	1.28	-2.79	11729.8	TRUE	TRUE	TRUE	TRUE	TRUE	TRUE
1	DEIFRSDTL	44	52	9	HLA-A*02:01	44	69	34545.54	69	1.562865	0.1872381	-4.538392	1.750103103	-2.7882889	1.56	0.19	-4.54	1.75	-2.79	34545.5	TRUE	TRUE	TRUE	TRUE	TRUE	TRUE
1	NCTDVSTAI	602	610	9	HLA-A*02:01	602	30	17630.39	30	1.189928	0.260741048	-4.2462619	1.45066905	-2.7955929	1.19	0.26	-4.25	1.45	-2.8	17630.4	TRUE	TRUE	TRUE	TRUE	TRUE	TRUE
1	DISGINASV	1150	1158	9	HLA-A*02:01	1150	15	6745.18	15	0.994029	0.038781025	-3.8289935	1.03281003	-2.7961835	0.99	0.04	-3.83	1.03	-2.8	6745.2	TRUE	TRUE	TRUE	TRUE	TRUE	TRUE
1	IANQFNKAI	905	913	9	HLA-A*02:01	905	30	17632.87	30	1.243128	0.2018765	-4.246323	1.445004495	-2.8013185	1.24	0.2	-4.25	1.45	-2.8	17632.9	TRUE	TRUE	TRUE	TRUE	TRUE	TRUE
1	NKSQSVIII	109	117	9	HLA-A*02:01	109	42	24658.54	42	1.261531	0.32855065	-4.3919674	1.590081645	-2.8018857	1.26	0.33	-4.39	1.59	-2.8	24658.5	TRUE	TRUE	TRUE	TRUE	TRUE	TRUE

seq #	peptide	start	end	peptide length	allele	peptide index	median binding	ann IC50	ann percentile	proteasome sc	tap score	mhc score	processing sco	processing tota	legacy Proteas	legacy TAP Sco	legacy MHC Sc	legacy Process	legacy Total Sc	legacy MHC IC5	equal Proteaso	equal TAP Scor	equal MHC Sco	equal Processi	equal Total Sc	equal MHC IC50
1	INNSTNVVI	117	125	9	HLA-A*02:01	117	47	26634.4	47	1.484328	0.138364021	-4.4254429	1.622692021	-2.8027509	1.48	0.14	-4.43	1.62	-2.8	26634.4	TRUE	TRUE	TRUE	TRUE	TRUE	TRUE
1	TALGKLQDV	925	933	9	HLA-A*02:01	925	14	6323.53	14	0.812132	0.18000289	-3.8009596	0.99213489	-2.8088247	0.81	0.18	-3.8	0.99	-2.81	6323.5	TRUE	TRUE	TRUE	TRUE	TRUE	TRUE
1	YTQHTSSMR	30	38	9	HLA-A*02:01	30	66	33660.41	66	1.039302	0.67336886	-4.5271194	1.71267086	-2.8144485	1.04	0.67	-4.53	1.71	-2.81	33660.4	TRUE	TRUE	TRUE	TRUE	TRUE	TRUE
1	DQLTPAWRI	613	621	9	HLA-A*02:01	613	29	16621.54	29	1.201246	0.20143436	-4.2206713	1.40268036	-2.8179909	1.2	0.2	-4.22	1.4	-2.82	16621.5	TRUE	TRUE	TRUE	TRUE	TRUE	TRUE
1	AQDIWGTSA	241	249	9	HLA-A*02:01	241	15	6897.95	15	1.293722	-0.2754824	-3.83872	1.01823961	-2.8204804	1.29	-0.28	-3.84	1.02	-2.82	6897.9	TRUE	TRUE	TRUE	TRUE	TRUE	TRUE
1	FAATEKSNV	89	97	9	HLA-A*02:01	89	15	6445.23	15	0.879504	0.1019408	-3.8092384	0.9814448	-2.8277936	0.88	0.1	-3.81	0.98	-2.83	6445.2	TRUE	TRUE	TRUE	TRUE	TRUE	TRUE
1	RNFFSPQII	1089	1097	9	HLA-A*02:01	1089	40	23369.09	40	1.238416	0.29879062	-4.3686418	1.53720662	-2.8314352	1.24	0.3	-4.37	1.54	-2.83	23369.1	TRUE	TRUE	TRUE	TRUE	TRUE	TRUE
1	AQKFNGLTV	834	842	9	HLA-A*02:01	834	20	10368.17	20	0.951272	0.226586733	-4.0157021	1.177858733	-2.8378434	0.95	0.23	-4.02	1.18	-2.84	10368.2	TRUE	TRUE	TRUE	TRUE	TRUE	TRUE
1	EKSNVVRGW	93	101	9	HLA-A*02:01	93	74	35969.43	74	1.399851	0.318228094	-4.5559336	1.71807909	-2.8378545	1.4	0.32	-4.56	1.72	-2.84	35969.4	TRUE	TRUE	TRUE	TRUE	TRUE	TRUE
1	FQPFQQFGR	545	553	9	HLA-A*02:01	545	37	21801.37	37	0.805848	0.68669385	-4.3384838	1.492541847	-2.8459419	0.81	0.69	-4.34	1.49	-2.85	21801.4	TRUE	TRUE	TRUE	TRUE	TRUE	TRUE
1	DSTECANLL	727	735	9	HLA-A*02:01	727	43	24740.59	43	1.273548	0.26776662	-4.3934101	1.541314622	-2.8520954	1.27	0.27	-4.39	1.54	-2.85	24740.6	TRUE	TRUE	TRUE	TRUE	TRUE	TRUE
1	VTLADAGFM	808	816	9	HLA-A*02:01	808	21	11049.95	21	0.988545	0.201286887	-4.0433603	1.189831887	-2.8535284	0.99	0.2	-4.04	1.19	-2.85	11050	TRUE	TRUE	TRUE	TRUE	TRUE	TRUE
1	DTLYLTQDL	50	58	9	HLA-A*02:01	50	45	25831.77	45	1.257507	0.301041647	-4.4121542	1.558548647	-2.8536055	1.26	0.3	-4.41	1.56	-2.85	25831.8	TRUE	TRUE	TRUE	TRUE	TRUE	TRUE
1	VKGDDVRQI	389	397	9	HLA-A*02:01	389	65	33393.06	65	1.435136	0.2291334	-4.5236562	1.664269396	-2.8593868	1.44	0.23	-4.52	1.66	-2.86	33393.1	TRUE	TRUE	TRUE	TRUE	TRUE	TRUE
1	CVLGQSKRV	1014	1022	9	HLA-A*02:01	1014	22	12186.56	22	1.103462	0.112642934	-4.0858811	1.216104934	-2.8697762	1.1	0.11	-4.09	1.22	-2.87	12186.6	TRUE	TRUE	TRUE	TRUE	TRUE	TRUE
1	NCDVVIGII	1107	1115	9	HLA-A*02:01	1107	26	15178.31	26	1.139666	0.16671292	-4.1812234	1.30637892	-2.8748445	1.14	0.17	-4.18	1.31	-2.87	15178.3	TRUE	TRUE	TRUE	TRUE	TRUE	TRUE
1	VYAWERKKI	337	345	9	HLA-A*02:01	337	93	41556.26	93	1.337282	0.4036783	-4.6186365	1.7409603	-2.8776762	1.34	0.4	-4.62	1.74	-2.88	41556.3	TRUE	TRUE	TRUE	TRUE	TRUE	TRUE
1	RGWVFGSTM	99	107	9	HLA-A*02:01	99	25	14422.78	25	1.062772	0.217269204	-4.159049	1.280041204	-2.8790078	1.06	0.22	-4.16	1.28	-2.88	14422.8	TRUE	TRUE	TRUE	TRUE	TRUE	TRUE
1	AAEQDRNTR	753	761	9	HLA-A*02:01	753	93	41711.67	93	1.068671	0.67087376	-4.6202576	1.739544759	-2.8807128	1.07	0.67	-4.62	1.74	-2.88	41711.7	TRUE	TRUE	TRUE	TRUE	TRUE	TRUE
1	QPIDVVRDL	201	209	9	HLA-A*02:01	201	47	26923.86	47	1.25139	0.29753137	-4.4301373	1.548921366	-2.881216	1.25	0.3	-4.43	1.55	-2.88	26923.9	TRUE	TRUE	TRUE	TRUE	TRUE	TRUE
1	TKRSFIEDL	795	803	9	HLA-A*02:01	795	51	28320.15	51	1.183557	0.386492469	-4.4520955	1.57004947	-2.8820461	1.18	0.39	-4.45	1.57	-2.88	28320.2	TRUE	TRUE	TRUE	TRUE	TRUE	TRUE
1	ISGINASVV	1151	1159	9	HLA-A*02:01	1151	29	16723.28	29	1.294589	0.0315665	-4.2233215	1.326155503	-2.897166	1.29	0.03	-4.22	1.33	-2.9	16723.3	TRUE	TRUE	TRUE	TRUE	TRUE	TRUE
1	YECDIPIGA	646	654	9	HLA-A*02:01	646	19	9971.65	19	1.370826	-0.2718628	-3.998767	1.09896319	-2.8998038	1.37	-0.27	-4	1.1	-2.9	9971.6	TRUE	TRUE	TRUE	TRUE	TRUE	TRUE
1	LTLTSGSDL	8	16	9	HLA-A*02:01	8	32	18833.46	32	0.99387	0.37148258	-4.2749301	1.36535258	-2.9095775	0.99	0.37	-4.27	1.37	-2.91	18833.5	TRUE	TRUE	TRUE	TRUE	TRUE	TRUE
1	YNYKYRYLR	436	444	9	HLA-A*02:01	436	62	32407.8	62	0.888583	0.71205574	-4.5106495	1.60063874	-2.9100108	0.89	0.71	-4.51	1.6	-2.91	32407.8	TRUE	TRUE	TRUE	TRUE	TRUE	TRUE
1	ECVLGQSKR	1013	1021	9	HLA-A*02:01	1013	97	43337.02	97	1.099899	0.62077515	-4.636859	1.720674152	-2.9161849	1.1	0.62	-4.64	1.72	-2.92	43337	TRUE	TRUE	TRUE	TRUE	TRUE	TRUE
1	AVLYQDVNC	595	603	9	HLA-A*02:01	595	41	24028.54	41	1.305238	0.15664555	-4.3807274	1.461883552	-2.9188438	1.31	0.16	-4.38	1.46	-2.92	24028.5	TRUE	TRUE	TRUE	TRUE	TRUE	TRUE
1	VVIGIINNT	1110	1118	9	HLA-A*02:01	1110	9.5	3845.5	9.5	0.906543	-0.2500208	-3.5849528	0.656522218	-2.9284306	0.91	-0.25	-3.58	0.66	-2.93	3845.5	TRUE	TRUE	TRUE	TRUE	TRUE	TRUE
1	KNKDGFLYV	188	196	9	HLA-A*02:01	188	21	11074.97	21	1.043421	0.06916591	-4.0443426	1.112586911	-2.9317556	1.04	0.07	-4.04	1.11	-2.93	11075	TRUE	TRUE	TRUE	TRUE	TRUE	TRUE
1	TPPALNCYW	468	476	9	HLA-A*02:01	468	65	33304.3	65	1.358096	0.232499416	-4.5225003	1.59059542	-2.9319049	1.36	0.23	-4.52	1.59	-2.93	33304.3	TRUE	TRUE	TRUE	TRUE	TRUE	TRUE
1	GDSTECANL	726	734	9	HLA-A*02:01	726	57	30559.64	57	1.352299	0.199956595	-4.4851482	1.5522556	-2.9328926	1.35	0.2	-4.49	1.55	-2.93	30559.6	TRUE	TRUE	TRUE	TRUE	TRUE	TRUE
1	MFIFLLFLT	1	9	9	HLA-A*02:01	1	7.1	2359.69	7.1	0.675908	-0.2499209	-3.372855	0.425987133	-2.9468678	0.68	-0.25	-3.37	0.43	-2.95	2359.7	TRUE	TRUE	TRUE	TRUE	TRUE	TRUE
1	AYRFNGIGV	885	893	9	HLA-A*02:01	885	40	23472.23	40	1.069995	0.34968956	-4.3705544	1.419684563	-2.9508698	1.07	0.35	-4.37	1.42	-2.95	23472.2	TRUE	TRUE	TRUE	TRUE	TRUE	TRUE
1	ECLGDINAR	821	829	9	HLA-A*02:01	821	75	36322.96	75	1.04096	0.558609976	-4.5601812	1.59956998	-2.9606113	1.04	0.56	-4.56	1.6	-2.96	36323	TRUE	TRUE	TRUE	TRUE	TRUE	TRUE
1	AAYFVGYLK	250	258	9	HLA-A*02:01	250	26	15222.23	26	0.785264	0.4362923	-4.1824783	1.2215563	-2.960922	0.79	0.44	-4.18	1.22	-2.96	15222.2	TRUE	TRUE	TRUE	TRUE	TRUE	TRUE
1	FPSVYAWER	334	342	9	HLA-A*02:01	334	44	25390.08	44	0.930776	0.51282469	-4.4046641	1.443600686	-2.9610634	0.93	0.51	-4.4	1.44	-2.96	25390.1	TRUE	TRUE	TRUE	TRUE	TRUE	TRUE
1	YVWLGFIAG	1197	1205	9	HLA-A*02:01	1197	7.5	2568.76	7.5	0.950793	-0.5035387	-3.4097235	0.447254332	-2.9624692	0.95	-0.5	-3.41	0.45	-2.96	2568.8	TRUE	TRUE	TRUE	TRUE	TRUE	TRUE
1	AATKMSECV	1007	1015	9	HLA-A*02:01	1007	24	13572.35	24	0.943638	0.22605586	-4.1326551	1.169693864	-2.9629612	0.94	0.23	-4.13	1.17	-2.96	13572.4	TRUE	TRUE	TRUE	TRUE	TRUE	TRUE
1	GINITNFRA	225	233	9	HLA-A*02:01	225	12	4954.26	12	1.004986	-0.2734737	-3.6949788	0.731512306	-2.9634665	1	-0.27	-3.69	0.73	-2.96	4954.3	TRUE	TRUE	TRUE	TRUE	TRUE	TRUE
1	ILTAFSPAQ	234	242	9	HLA-A*02:01	234	17	8494.96	17	0.94146	0.022791944	-3.9291613	0.96425194	-2.9649094	0.94	0.02	-3.93	0.96	-2.96	8495	TRUE	TRUE	TRUE	TRUE	TRUE	TRUE
1	LRSTSQKSI	666	674	9	HLA-A*02:01	666	90	40674.57	90	1.300131	0.34178763	-4.609323	1.641918628	-2.9674043	1.3	0.34	-4.61	1.64	-2.97	40674.6	TRUE	TRUE	TRUE	TRUE	TRUE	TRUE
1	LQDVVNQNA	930	938	9	HLA-A*02:01	930	16	7661.76	16	1.144026	-0.2294617	-3.8843285	0.91456427	-2.9697643	1.14	-0.23	-3.88	0.91	-2.97	7661.8	TRUE	TRUE	TRUE	TRUE	TRUE	TRUE
1	HGKLRPFER	445	453	9	HLA-A*02:01	445	79	37287	79	1.053936	0.54496585	-4.5715574	1.59890185	-2.9726556	1.05	0.54	-4.57	1.6	-2.97	37287	TRUE	TRUE	TRUE	TRUE	TRUE	TRUE
1	FKDGIYFAA	83	91	9	HLA-A*02:01	83	9.3	3682.77	9.3	0.981919	-0.3903676	-3.5661746	0.591551352	-2.9746232	0.98	-0.39	-3.57	0.59	-2.97	3682.8	TRUE	TRUE	TRUE	TRUE	TRUE	TRUE
1	ALQIPFAMQ	875	883	9	HLA-A*02:01	875	23	12721.72	23	1.050869	0.07726573	-4.1045458	1.128134726	-2.9764111	1.05	0.08	-4.1	1.13	-2.98	12721.7	TRUE	TRUE	TRUE	TRUE	TRUE	TRUE
1	IYQTSNFRV	299	307	9	HLA-A*02:01	299	26	14767.02	26	0.958176	0.232884961	-4.1692929	1.19106096	-2.9782319	0.96	0.23	-4.17	1.19	-2.98	14767	TRUE	TRUE	TRUE	TRUE	TRUE	TRUE
1	TLADAGFMK	809	817	9	HLA-A*02:01	809	25	14227.49	25	0.94531	0.22946596	-4.1531283	1.174775962	-2.9783523	0.95	0.23	-4.15	1.17	-2.98	14227.5	TRUE	TRUE	TRUE	TRUE	TRUE	TRUE
1	SMAKTSVDC	712	720	9	HLA-A*02:01	712	22	12272.58	22	1.00034	0.108421738	-4.0889359	1.10876174	-2.9801741	1	0.11	-4.09	1.11	-2.98	12272.6	TRUE	TRUE	TRUE	TRUE	TRUE	TRUE
1	INNTVYDPL	1115	1123	9	HLA-A*02:01	1115	36	21442.75	36	1.022816	0.32357309	-4.3312805	1.34638909	-2.9848914	1.02	0.32	-4.33	1.35	-2.98	21442.8	TRUE	TRUE	TRUE	TRUE	TRUE	TRUE
1	NMYICGDST	721	729	9	HLA-A*02:01	721	12	4715.71	12	0.844156	-0.159827	-3.6735471	0.684329022	-2.9892181	0.84	-0.16	-3.67	0.68	-2.99	4715.7	TRUE	TRUE	TRUE	TRUE	TRUE	TRUE
1	YEQYIKWPW	1188	1196	9	HLA-A*02:01	1188	42	24270.51	42	1.041353	0.34546578	-4.3850789	1.386818783	-2.9982601	1.04	0.35	-4.39	1.39	-3	24270.5	TRUE	TRUE	TRUE	TRUE	TRUE	TRUE
1	RAAEIRASA	996	1004	9	HLA-A*02:01	996	21	11342.37	21	1.147058	-0.0911665	-4.0547038	1.055891531	-2.9988123	1.15	-0.09	-4.05	1.06	-3	11342.4	TRUE	TRUE	TRUE	TRUE	TRUE	TRUE
1	STSQKSIVA	668	676	9	HLA-A*02:01	668	28	16136	28	1.363403	-0.1623797	-4.2077959	1.20102335	-3.0067725	1.36	-0.16	-4.21	1.2	-3.01	16136	TRUE	TRUE	TRUE	TRUE	TRUE	TRUE
1	ILPDPLKPT	787	795	9	HLA-A*02:01	787	6.5	2037.13	6.5	0.611578	-0.3159612	-3.3090187	0.29561677	-3.013402	0.61	-0.32	-3.31	0.3	-3.01	2037.1	TRUE	TRUE	TRUE	TRUE	TRUE	TRUE
1	AQALNTLVK	938	946	9	HLA-A*02:01	938	44	25491.37	44	1.025133	0.36652527	-4.4063932	1.391658271	-3.0147349	1.03	0.37	-4.41	1.39	-3.01	25491.4	TRUE	TRUE	TRUE	TRUE	TRUE	TRUE
1	PLNDYGFYT	477	485	9	HLA-A*02:01	477	7.6	2662.8	7.6	0.980331	-0.5718007	-3.4253385	0.408530259	-3.0168083	0.98	-0.57	-3.43	0.41	-3.02	2662.8	TRUE	TRUE	TRUE	TRUE	TRUE	TRUE
1	TGNYNYKYR	433	441	9	HLA-A*02:01	433	91	40874.42	91	1.089944	0.501412102	-4.6114516	1.591356102	-3.0200955	1.09	0.5	-4.61	1.59	-3.02	40874.4	TRUE	TRUE	TRUE	TRUE	TRUE	TRUE
1	VTILLCCMT	1212	1220	9	HLA-A*02:01	1212	15	6570.5	15	1.039706	-0.24352	-3.8175984	0.79618603	-3.0214124	1.04	-0.24	-3.82	0.8	-3.02	6570.5	TRUE	TRUE	TRUE	TRUE	TRUE	TRUE
1	LTSGSDLDR	10	18	9	HLA-A*02:01	10	72	35389.2	72	0.887805	0.63883937	-4.5488707	1.526644372	-3.0222264	0.89	0.64	-4.55	1.53	-3.02	35389.2	TRUE	TRUE	TRUE	TRUE	TRUE	TRUE
1	DILSRLDKV	961	969	9	HLA-A*02:01	961	24	13420.04	24	0.981448	0.12307916	-4.1277538	1.10452716	-3.0232267	0.98	0.12	-4.13	1.1	-3.02	13420	TRUE	TRUE	TRUE	TRUE	TRUE	TRUE
1	ISPCSFGGV	573	581	9	HLA-A*02:01	573	21	11554.05	21	0.935688	0.09591265	-4.0627342	1.031600648	-3.0311336	0.94	0.1	-4.06	1.03	-3.03	11554	TRUE	TRUE	TRUE	TRUE	TRUE	TRUE
1	QIDRLITGR	974	982	9	HLA-A*02:01	974	55	29966.98	55	0.794028	0.649214737	-4.476643	1.443242737	-3.0334002	0.79	0.65	-4.48	1.44	-3.03	29967	TRUE	TRUE	TRUE	TRUE	TRUE	TRUE
1	LIRAAEIRA	994	1002	9	HLA-A*02:01	994	27	15695.36	27	1.334854	-0.1739585	-4.1957713	1.16089545	-3.0348758	1.33	-0.17	-4.2	1.16	-3.03	15695.4	TRUE	TRUE	TRUE	TRUE	TRUE	TRUE
1	INITNFRAI	226	234	9	HLA-A*02:01	226	42	24450.26	42	1.156714	0.19208619	-4.3882835	1.34880019	-3.0394833	1.16	0.19	-4.39	1.35	-3.04	24450.3	TRUE	TRUE	TRUE	TRUE	TRUE	TRUE
1	MGTQTHTMI	144	152	9	HLA-A*02:01	144	50	28143.29	50	1.347964	0.05710801	-4.4493749	1.40507201	-3.0443029	1.35	0.06	-4.45	1.41	-3.04	28143.3	TRUE	TRUE	TRUE	TRUE	TRUE	TRUE
1	FLYVYKGYQ	193	201	9	HLA-A*02:01	193	14	5906.21	14	0.737611	-0.0111756	-3.7713089	0.726435394	-3.0448735	0.74	-0.01	-3.77	0.73	-3.04	5906.2	TRUE	TRUE	TRUE	TRUE	TRUE	TRUE
1	LNDLCFSNV	374	382	9	HLA-A*02:01	374	24	13337.68	24	1.040667	0.03863839	-4.1250803	1.079305393	-3.0457749	1.04	0.04	-4.13	1.08	-3.05	13337.7	TRUE	TRUE	TRUE	TRUE	TRUE	TRUE
1	WYVWLGFIA	1196	1204	9	HLA-A*02:01	1196	22	12220.1	22	1.195028	-0.1578273	-4.0870748	1.037200655	-3.0498741	1.2	-0.16	-4.09	1.04	-3.05	12220.1	TRUE	TRUE	TRUE	TRUE	TRUE	TRUE
1	QRNFFSPQI	1088	1096	9	HLA-A*02:01	1088	51	28504.91	51	1.11493	0.28974564	-4.4549197	1.40467564	-3.050244	1.11	0.29	-4.45	1.4	-3.05	28504.9	TRUE	TRUE	TRUE	TRUE	TRUE	TRUE
1	GVLTPSSKR	536	544	9	HLA-A*02:01	536	91	40964.3	91	0.917184	0.63816571	-4.6124055	1.555349713	-3.0570558	0.92	0.64	-4.61	1.56	-3.06	40964.3	TRUE	TRUE	TRUE	TRUE	TRUE	TRUE
1	ERKKISNCV	341	349	9	HLA-A*02:01	341	77	36918.09	77	1.393168	0.11078291	-4.5672392	1.50395091	-3.0632883	1.39	0.11	-4.57	1.5	-3.06	36918.1	TRUE	TRUE	TRUE	TRUE	TRUE	TRUE
1	QIQESLTTT	915	923	9	HLA-A*02:01	915	17	8616.79	17	1.106271	-0.2363521	-3.9353455	0.869918857	-3.0654267	1.11	-0.24	-3.94	0.87	-3.07	8616.8	TRUE	TRUE	TRUE	TRUE	TRUE	TRUE
1	IEDLLFNKV	800	808	9	HLA-A*02:01	800	26	14990.3	26	1.145868	-0.0356086	-4.1758103	1.110259431	-3.0655509	1.15	-0.04	-4.18	1.11	-3.07	14990.3	TRUE	TRUE	TRUE	TRUE	TRUE	TRUE
1	YHLMSFPQA	1029	1037	9	HLA-A*02:01	1029	23	12764.45	23	1.274061	-0.2421926	-4.1060021	1.03186839	-3.0741337	1.27	-0.24	-4.11	1.03	-3.07	12764.5	TRUE	TRUE	TRUE	TRUE	TRUE	TRUE
1	FPQAAPHGV	1034	1042	9	HLA-A*02:01	1034	21	11255.93	21	1.004368	-0.0285179	-4.0513814	0.975850098	-3.0755313	1	-0.03	-4.05	0.98	-3.08	11255.9	TRUE	TRUE	TRUE	TRUE	TRUE	TRUE
1	FMLKYDENG	262	270	9	HLA-A*02:01	262	7.3	2462.27	7.3	0.896169	-0.5817956	-3.3913357	0.314373387	-3.0769623	0.9	-0.58	-3.39	0.31	-3.08	2462.3	TRUE	TRUE	TRUE	TRUE	TRUE	TRUE
1	FFSPQIITT	1091	1099	9	HLA-A*02:01	1091	17	8456.81	17	1.063412	-0.2153077	-3.9272066	0.848104264	-3.0791023	1.06	-0.22	-3.93	0.85	-3.08	8456.8	TRUE	TRUE	TRUE	TRUE	TRUE	TRUE
1	DIWGTSAAA	243	251	9	HLA-A*02:01	243	17	8398.91	17	1.100034	-0.2595972	-3.9242229	0.840436843	-3.0837861	1.1	-0.26	-3.92	0.84	-3.08	8398.9	TRUE	TRUE	TRUE	TRUE	TRUE	TRUE
1	PFAMQMAYR	879	887	9	HLA-A*02:01	879	70	34814.22	70	0.935026	0.519675238	-4.5417567	1.45470124	-3.0870554	0.94	0.52	-4.54	1.45	-3.09	34814.2	TRUE	TRUE	TRUE	TRUE	TRUE	TRUE
1	VRDPKTSEI	562	570	9	HLA-A*02:01	562	58	30979.1	58	1.108709	0.29470477	-4.4910688	1.40341377	-3.087655	1.11	0.29	-4.49	1.4	-3.09	30979.1	TRUE	TRUE	TRUE	TRUE	TRUE	TRUE
1	KQIANQFNK	903	911	9	HLA-A*02:01	903	48	27104.49	48	1.053696	0.2901959	-4.4330412	1.343891905	-3.0891493	1.05	0.29	-4.43	1.34	-3.09	27104.5	TRUE	TRUE	TRUE	TRUE	TRUE	TRUE
1	VEAEVQIDR	969	977	9	HLA-A*02:01	969	73	35588.89	73	0.798208	0.663017254	-4.5513144	1.461225253	-3.0900892	0.8	0.66	-4.55	1.46	-3.09	35588.9	TRUE	TRUE	TRUE	TRUE	TRUE	TRUE
1	LKPTKRSFI	792	800	9	HLA-A*02:01	792	85	38900.8	85	1.310858	0.18515643	-4.5899585	1.49601443	-3.0939441	1.31	0.19	-4.59	1.5	-3.09	38900.8	TRUE	TRUE	TRUE	TRUE	TRUE	TRUE
1	SGIAAEQDR	750	758	9	HLA-A*02:01	750	95	42452.91	95	0.931032	0.60193744	-4.6279075	1.532969439	-3.094938	0.93	0.6	-4.63	1.53	-3.09	42452.9	TRUE	TRUE	TRUE	TRUE	TRUE	TRUE
1	QIPFAMQMA	877	885	9	HLA-A*02:01	877	20	10423.73	20	1.078532	-0.1555284	-4.0180232	0.923003608	-3.0950195	1.08	-0.16	-4.02	0.92	-3.1	10423.7	TRUE	TRUE	TRUE	TRUE	TRUE	TRUE
1	KSGNFKHLR	175	183	9	HLA-A*02:01	175	80	37600.97	80	0.915365	0.56431899	-4.575199	1.479683988	-3.0955151	0.92	0.56	-4.58	1.48	-3.1	37601	TRUE	TRUE	TRUE	TRUE	TRUE	TRUE
1	STNVVIRAC	120	128	9	HLA-A*02:01	120	30	17494.33	30	1.107292	0.039703429	-4.2428973	1.146995429	-3.0959019	1.11	0.04	-4.24	1.15	-3.1	17494.3	TRUE	TRUE	TRUE	TRUE	TRUE	TRUE
1	GYQPYRVVV	490	498	9	HLA-A*02:01	490	42	24362.86	42	1.123479	0.16210039	-4.3867283	1.28557939	-3.1011489	1.12	0.16	-4.39	1.29	-3.1	24362.9	TRUE	TRUE	TRUE	TRUE	TRUE	TRUE
1	QMYKTPTLK	769	777	9	HLA-A*02:01	769	36	21182.63	36	0.835714	0.38559895	-4.3259799	1.22131295	-3.1046669	0.84	0.39	-4.33	1.22	-3.1	21182.6	TRUE	TRUE	TRUE	TRUE	TRUE	TRUE
1	AKTSVDCNM	714	722	9	HLA-A*02:01	714	59	31303.24	59	1.174451	0.216177752	-4.4955893	1.39062875	-3.1049605	1.17	0.22	-4.5	1.39	-3.1	31303.2	TRUE	TRUE	TRUE	TRUE	TRUE	TRUE
1	LGDINARDL	823	831	9	HLA-A*02:01	823	57	30499.51	57	1.155485	0.22316925	-4.4842929	1.378654247	-3.1056386	1.16	0.22	-4.48	1.38	-3.11	30499.5	TRUE	TRUE	TRUE	TRUE	TRUE	TRUE
1	TAFSPAQDI	236	244	9	HLA-A*02:01	236	34	20332.01	34	0.887883	0.31432024	-4.3081803	1.20220324	-3.1059771	0.89	0.31	-4.31	1.2	-3.11	20332	TRUE	TRUE	TRUE	TRUE	TRUE	TRUE
1	RFNGIGVTQ	887	895	9	HLA-A*02:01	887	56	30166.72	56	1.302494	0.06884335	-4.4795281	1.371337351	-3.1081907	1.3	0.07	-4.48	1.37	-3.11	30166.7	TRUE	TRUE	TRUE	TRUE	TRUE	TRUE
1	TGIGYQPYR	487	495	9	HLA-A*02:01	487	89	40161.18	89	0.941075	0.55302296	-4.6038065	1.494097958	-3.1097085	0.94	0.55	-4.6	1.49	-3.11	40161.2	TRUE	TRUE	TRUE	TRUE	TRUE	TRUE
1	KLNDLCFSN	373	381	9	HLA-A*02:01	373	8.7	3317	8.7	0.961706	-0.5508095	-3.5207455	0.410896516	-3.109849	0.96	-0.55	-3.52	0.41	-3.11	3317	TRUE	TRUE	TRUE	TRUE	TRUE	TRUE
1	SANLAATKM	1003	1011	9	HLA-A*02:01	1003	44	25191.42	44	1.086072	0.20386791	-4.4012526	1.289939908	-3.1113127	1.09	0.2	-4.4	1.29	-3.11	25191.4	TRUE	TRUE	TRUE	TRUE	TRUE	TRUE
1	FRSDTLYLT	47	55	9	HLA-A*02:01	47	19	9602.76	19	1.122341	-0.2561874	-3.9823961	0.86615357	-3.1162425	1.12	-0.26	-3.98	0.87	-3.12	9602.8	TRUE	TRUE	TRUE	TRUE	TRUE	TRUE
1	NVFQTQAGC	627	635	9	HLA-A*02:01	627	32	18867.94	32	0.965659	0.19220763	-4.2757245	1.157866633	-3.1178579	0.97	0.19	-4.28	1.16	-3.12	18867.9	TRUE	TRUE	TRUE	TRUE	TRUE	TRUE
1	IFLLFLTLT	3	11	9	HLA-A*02:01	3	14	5801.83	14	0.89757	-0.2615932	-3.763565	0.63597678	-3.1275882	0.9	-0.26	-3.76	0.64	-3.13	5801.8	TRUE	TRUE	TRUE	TRUE	TRUE	TRUE
1	TFVSGNCDV	1102	1110	9	HLA-A*02:01	1102	41	23803.95	41	0.954995	0.29404183	-4.376649	1.249036826	-3.1276122	0.95	0.29	-4.38	1.25	-3.13	23804	TRUE	TRUE	TRUE	TRUE	TRUE	TRUE
1	LLNAPATVC	503	511	9	HLA-A*02:01	503	25	13968.64	25	1.107099	-0.0971294	-4.1451541	1.00996958	-3.1351845	1.11	-0.1	-4.15	1.01	-3.14	13968.6	TRUE	TRUE	TRUE	TRUE	TRUE	TRUE
1	AGWTFGAGA	866	874	9	HLA-A*02:01	866	24	13569.85	24	1.202282	-0.2057183	-4.132575	0.996563653	-3.1360114	1.2	-0.21	-4.13	1	-3.14	13569.9	TRUE	TRUE	TRUE	TRUE	TRUE	TRUE
1	TTEVMPVSM	705	713	9	HLA-A*02:01	705	36	20919.11	36	1.1225	0.0546806	-4.3205432	1.177180603	-3.1433626	1.12	0.05	-4.32	1.18	-3.14	20919.1	TRUE	TRUE	TRUE	TRUE	TRUE	TRUE
1	PVIPFKDGI	79	87	9	HLA-A*02:01	79	54	29497.31	54	1.151673	0.17429552	-4.4697824	1.32596852	-3.1438139	1.15	0.17	-4.47	1.33	-3.14	29497.3	TRUE	TRUE	TRUE	TRUE	TRUE	TRUE
1	VSDFTDSVR	555	563	9	HLA-A*02:01	555	79	37451.97	79	0.877856	0.55170321	-4.5734747	1.429559208	-3.1439155	0.88	0.55	-4.57	1.43	-3.14	37452	TRUE	TRUE	TRUE	TRUE	TRUE	TRUE
1	EVFAQVKQM	762	770	9	HLA-A*02:01	762	43	24977.28	43	1.04376	0.20954433	-4.3975451	1.25330433	-3.1442408	1.04	0.21	-4.4	1.25	-3.14	24977.3	TRUE	TRUE	TRUE	TRUE	TRUE	TRUE
1	NNKSQSVII	108	116	9	HLA-A*02:01	108	80	37599.35	80	1.233455	0.19635985	-4.5751803	1.429814848	-3.1453655	1.23	0.2	-4.58	1.43	-3.15	37599.3	TRUE	TRUE	TRUE	TRUE	TRUE	TRUE
1	GDVVRFPNI	311	319	9	HLA-A*02:01	311	48	26991.82	48	1.208691	0.07664636	-4.4312322	1.285337358	-3.1458948	1.21	0.08	-4.43	1.29	-3.15	26991.8	TRUE	TRUE	TRUE	TRUE	TRUE	TRUE
1	IGYQPYRVV	489	497	9	HLA-A*02:01	489	39	22954.84	39	1.117101	0.09748332	-4.3608743	1.214584319	-3.14629	1.12	0.1	-4.36	1.21	-3.15	22954.8	TRUE	TRUE	TRUE	TRUE	TRUE	TRUE
1	NFKHLREFV	178	186	9	HLA-A*02:01	178	49	27665.96	49	1.080175	0.210591084	-4.4419457	1.29076608	-3.1511797	1.08	0.21	-4.44	1.29	-3.15	27666	TRUE	TRUE	TRUE	TRUE	TRUE	TRUE
1	ACSCGSCCK	1229	1237	9	HLA-A*02:01	1229	81	37886.03	81	1.158391	0.25884848	-4.5784791	1.417239483	-3.1612396	1.16	0.26	-4.58	1.42	-3.16	37886	TRUE	TRUE	TRUE	TRUE	TRUE	TRUE
1	ASANLAATK	1002	1010	9	HLA-A*02:01	1002	63	32658.41	63	1.002122	0.34735918	-4.513995	1.34948118	-3.1645139	1	0.35	-4.51	1.35	-3.16	32658.4	TRUE	TRUE	TRUE	TRUE	TRUE	TRUE
1	ARDLICAQK	828	836	9	HLA-A*02:01	828	50	27992.06	50	0.934145	0.33986989	-4.4470349	1.27401489	-3.17302	0.93	0.34	-4.45	1.27	-3.17	27992.1	TRUE	TRUE	TRUE	TRUE	TRUE	TRUE
1	ALSGIAAEQ	748	756	9	HLA-A*02:01	748	20	10705.48	20	0.770056	0.07994183	-4.0296061	0.849997827	-3.1796083	0.77	0.08	-4.03	0.85	-3.18	10705.5	TRUE	TRUE	TRUE	TRUE	TRUE	TRUE
1	TDLIKNQCV	517	525	9	HLA-A*02:01	517	57	30660.31	57	1.282458	0.023617629	-4.4865765	1.30607563	-3.1805009	1.28	0.02	-4.49	1.31	-3.18	30660.3	TRUE	TRUE	TRUE	TRUE	TRUE	TRUE
1	VDLGDISGI	1146	1154	9	HLA-A*02:01	1146	40	23234.21	40	1.111387	0.0722543	-4.3661279	1.183641296	-3.1824866	1.11	0.07	-4.37	1.18	-3.18	23234.2	TRUE	TRUE	TRUE	TRUE	TRUE	TRUE
1	LQYGSFCTQ	736	744	9	HLA-A*02:01	736	28	16475.79	28	0.94295	0.08958426	-4.2168462	1.03253426	-3.184312	0.94	0.09	-4.22	1.03	-3.18	16475.8	TRUE	TRUE	TRUE	TRUE	TRUE	TRUE
1	NASVVNIQK	1155	1163	9	HLA-A*02:01	1155	56	30371.75	56	1.052895	0.243824997	-4.4824698	1.296719997	-3.1857498	1.05	0.24	-4.48	1.3	-3.19	30371.8	TRUE	TRUE	TRUE	TRUE	TRUE	TRUE
1	SLLRSTSQK	664	672	9	HLA-A*02:01	664	50	27932.15	50	0.997573	0.26223867	-4.4461044	1.25981167	-3.1862927	1	0.26	-4.45	1.26	-3.19	27932.2	TRUE	TRUE	TRUE	TRUE	TRUE	TRUE
1	NSTNVVIRA	119	127	9	HLA-A*02:01	119	28	16593.15	28	1.183921	-0.1518985	-4.2199288	1.032022453	-3.1879064	1.18	-0.15	-4.22	1.03	-3.19	16593.2	TRUE	TRUE	TRUE	TRUE	TRUE	TRUE
1	SGFNTLKPI	211	219	9	HLA-A*02:01	211	24	13799.49	24	0.824808	0.123838176	-4.139863	0.948646176	-3.1912169	0.82	0.12	-4.14	0.95	-3.19	13799.5	TRUE	TRUE	TRUE	TRUE	TRUE	TRUE
1	NHTFGNPVI	73	81	9	HLA-A*02:01	73	84	38744.54	84	1.149822	0.238887276	-4.5882105	1.38870928	-3.1995012	1.15	0.24	-4.59	1.39	-3.2	38744.5	TRUE	TRUE	TRUE	TRUE	TRUE	TRUE
1	MNNKSQSVI	107	115	9	HLA-A*02:01	107	77	36893.31	77	1.218121	0.149068488	-4.5669476	1.36718949	-3.1997581	1.22	0.15	-4.57	1.37	-3.2	36893.3	TRUE	TRUE	TRUE	TRUE	TRUE	TRUE
1	PDPLKPTKR	789	797	9	HLA-A*02:01	789	97	43228.83	97	1.116107	0.316390494	-4.6357735	1.43249749	-3.203276	1.12	0.32	-4.64	1.43	-3.2	43228.8	TRUE	TRUE	TRUE	TRUE	TRUE	TRUE
1	LSFELLNAP	499	507	9	HLA-A*02:01	499	26	15054.17	26	0.840715	0.13228688	-4.1776568	0.973001878	-3.2046549	0.84	0.13	-4.18	0.97	-3.2	15054.2	TRUE	TRUE	TRUE	TRUE	TRUE	TRUE
1	SNCVADYSV	346	354	9	HLA-A*02:01	346	31	18476.62	31	0.962021	0.09982013	-4.2666225	1.061841134	-3.2047814	0.96	0.1	-4.27	1.06	-3.2	18476.6	TRUE	TRUE	TRUE	TRUE	TRUE	TRUE
1	SLIDLQELG	1178	1186	9	HLA-A*02:01	1178	8.1	2973.3	8.1	0.882815	-0.6164582	-3.4732387	0.266356798	-3.2068819	0.88	-0.62	-3.47	0.27	-3.21	2973.3	TRUE	TRUE	TRUE	TRUE	TRUE	TRUE
1	RGVYYPDEI	38	46	9	HLA-A*02:01	38	49	27529.51	49	0.961727	0.27095735	-4.4397985	1.232684347	-3.2071141	0.96	0.27	-4.44	1.23	-3.21	27529.5	TRUE	TRUE	TRUE	TRUE	TRUE	TRUE
1	SVITPGTNA	582	590	9	HLA-A*02:01	582	31	18160.3	31	1.149961	-0.0981247	-4.259123	1.05183633	-3.2072867	1.15	-0.1	-4.26	1.05	-3.21	18160.3	TRUE	TRUE	TRUE	TRUE	TRUE	TRUE
1	RSTSQKSIV	667	675	9	HLA-A*02:01	667	65	33182.72	65	1.046332	0.26406709	-4.520912	1.31039909	-3.2105129	1.05	0.26	-4.52	1.31	-3.21	33182.7	TRUE	TRUE	TRUE	TRUE	TRUE	TRUE
1	CGPKLSTDL	511	519	9	HLA-A*02:01	511	62	32286.7	62	1.001769	0.296493692	-4.5090237	1.298262692	-3.210761	1	0.3	-4.51	1.3	-3.21	32286.7	TRUE	TRUE	TRUE	TRUE	TRUE	TRUE
1	ADQLTPAWR	612	620	9	HLA-A*02:01	612	69	34504.09	69	0.776889	0.549016597	-4.5378706	1.3259056	-3.211965	0.78	0.55	-4.54	1.33	-3.21	34504.1	TRUE	TRUE	TRUE	TRUE	TRUE	TRUE
1	QFNKAISQI	908	916	9	HLA-A*02:01	908	51	28375.05	51	0.906127	0.33049221	-4.4529366	1.23661921	-3.2163174	0.91	0.33	-4.45	1.24	-3.22	28375	TRUE	TRUE	TRUE	TRUE	TRUE	TRUE
1	LQSLQTYVT	983	991	9	HLA-A*02:01	983	23	12769.85	23	1.10497	-0.2167936	-4.1061858	0.88817645	-3.2180094	1.1	-0.22	-4.11	0.89	-3.22	12769.9	TRUE	TRUE	TRUE	TRUE	TRUE	TRUE
1	NGIGVTQNV	889	897	9	HLA-A*02:01	889	27	15660.25	27	0.955337	0.02042873	-4.1947987	0.975765734	-3.219033	0.96	0.02	-4.19	0.98	-3.22	15660.2	TRUE	TRUE	TRUE	TRUE	TRUE	TRUE
1	FVGYLKPTT	253	261	9	HLA-A*02:01	253	18	9139.98	18	1.121007	-0.3809028	-3.9609452	0.740104153	-3.2208411	1.12	-0.38	-3.96	0.74	-3.22	9140	TRUE	TRUE	TRUE	TRUE	TRUE	TRUE
1	FCGKGYHLM	1024	1032	9	HLA-A*02:01	1024	23	12753.41	23	0.9135	-0.031362	-4.1056263	0.882138041	-3.2234883	0.91	-0.03	-4.11	0.88	-3.22	12753.4	TRUE	TRUE	TRUE	TRUE	TRUE	TRUE
1	CCMTSCCSC	1217	1225	9	HLA-A*02:01	1217	27	15758.83	27	0.928749	0.045027233	-4.197524	0.973776233	-3.2237477	0.93	0.05	-4.2	0.97	-3.22	15758.8	TRUE	TRUE	TRUE	TRUE	TRUE	TRUE
1	TREVFAQVK	760	768	9	HLA-A*02:01	760	68	34335.01	68	1.029502	0.28151202	-4.5357372	1.311014023	-3.2247232	1.03	0.28	-4.54	1.31	-3.22	34335	TRUE	TRUE	TRUE	TRUE	TRUE	TRUE
1	VNIQKEIDR	1159	1167	9	HLA-A*02:01	1159	87	39707.94	87	0.713892	0.65528109	-4.5988774	1.369173094	-3.2297043	0.71	0.66	-4.6	1.37	-3.23	39707.9	TRUE	TRUE	TRUE	TRUE	TRUE	TRUE
1	LPDDFMGCV	412	420	9	HLA-A*02:01	412	27	15509.17	27	0.988324	-0.0274567	-4.1905886	0.960867259	-3.2297213	0.99	-0.03	-4.19	0.96	-3.23	15509.2	TRUE	TRUE	TRUE	TRUE	TRUE	TRUE
1	SVIIINNST	113	121	9	HLA-A*02:01	113	19	10133.05	19	0.989865	-0.2152091	-4.0057402	0.774655875	-3.2310843	0.99	-0.22	-4.01	0.77	-3.23	10133	TRUE	TRUE	TRUE	TRUE	TRUE	TRUE
1	FKNHTSPDV	1138	1146	9	HLA-A*02:01	1138	26	14692.27	26	0.880188	0.052516539	-4.1670889	0.93270454	-3.2343844	0.88	0.05	-4.17	0.93	-3.23	14692.3	TRUE	TRUE	TRUE	TRUE	TRUE	TRUE
1	KCYGVSATK	365	373	9	HLA-A*02:01	365	38	22514.3	38	0.823051	0.29316926	-4.3524584	1.116220258	-3.2362382	0.82	0.29	-4.35	1.12	-3.24	22514.3	TRUE	TRUE	TRUE	TRUE	TRUE	TRUE
1	ICGDSTECA	724	732	9	HLA-A*02:01	724	41	23965.71	41	1.448707	-0.3061452	-4.3795903	1.14256176	-3.2370285	1.45	-0.31	-4.38	1.14	-3.24	23965.7	TRUE	TRUE	TRUE	TRUE	TRUE	TRUE
1	CDIPIGAGI	648	656	9	HLA-A*02:01	648	38	22171.28	38	1.070685	0.037029957	-4.3457908	1.107714957	-3.2380758	1.07	0.04	-4.35	1.11	-3.24	22171.3	TRUE	TRUE	TRUE	TRUE	TRUE	TRUE
1	VFLHVTYVP	1043	1051	9	HLA-A*02:01	1043	37	21929.13	37	0.930406	0.17212054	-4.3410214	1.102526538	-3.2384949	0.93	0.17	-4.34	1.1	-3.24	21929.1	TRUE	TRUE	TRUE	TRUE	TRUE	TRUE
1	DLCFSNVYA	376	384	9	HLA-A*02:01	376	17	8197.17	17	0.960556	-0.2947699	-3.9136639	0.665786101	-3.2478778	0.96	-0.29	-3.91	0.67	-3.25	8197.2	TRUE	TRUE	TRUE	TRUE	TRUE	TRUE
1	QYGECLGDI	818	826	9	HLA-A*02:01	818	46	26378.87	46	0.848487	0.32475055	-4.4212562	1.173237546	-3.2480186	0.85	0.32	-4.42	1.17	-3.25	26378.9	TRUE	TRUE	TRUE	TRUE	TRUE	TRUE
1	FNGLTGTGV	529	537	9	HLA-A*02:01	529	28	16061.8	28	0.937114	0.01904414	-4.2057942	0.956158138	-3.2496361	0.94	0.02	-4.21	0.96	-3.25	16061.8	TRUE	TRUE	TRUE	TRUE	TRUE	TRUE
1	DKVEAEVQI	967	975	9	HLA-A*02:01	967	77	36658.16	77	1.13716	0.16992026	-4.5641707	1.307080255	-3.2570904	1.14	0.17	-4.56	1.31	-3.26	36658.2	TRUE	TRUE	TRUE	TRUE	TRUE	TRUE
1	LKPTTFMLK	257	265	9	HLA-A*02:01	257	58	31007.26	58	1.035149	0.19702732	-4.4914634	1.232176323	-3.2592871	1.04	0.2	-4.49	1.23	-3.26	31007.3	TRUE	TRUE	TRUE	TRUE	TRUE	TRUE
1	AEVQIDRLI	971	979	9	HLA-A*02:01	971	46	26456.61	46	0.964452	0.19602685	-4.4225342	1.16047885	-3.2620553	0.96	0.2	-4.42	1.16	-3.26	26456.6	TRUE	TRUE	TRUE	TRUE	TRUE	TRUE
1	AKNLNESLI	1172	1180	9	HLA-A*02:01	1172	64	32835.58	64	0.953027	0.2948904	-4.5163447	1.2479174	-3.2684273	0.95	0.29	-4.52	1.25	-3.27	32835.6	TRUE	TRUE	TRUE	TRUE	TRUE	TRUE
1	MTSCCSCLK	1219	1227	9	HLA-A*02:01	1219	37	21595.01	37	0.856866	0.20869423	-4.3343534	1.06556023	-3.2687932	0.86	0.21	-4.33	1.07	-3.27	21595	TRUE	TRUE	TRUE	TRUE	TRUE	TRUE
1	EQDRNTREV	755	763	9	HLA-A*02:01	755	41	23909.52	41	1.036678	0.06974703	-4.3785709	1.10642503	-3.2721458	1.04	0.07	-4.38	1.11	-3.27	23909.5	TRUE	TRUE	TRUE	TRUE	TRUE	TRUE
1	VMPVSMAKT	708	716	9	HLA-A*02:01	708	15	7054.87	15	0.878115	-0.3064843	-3.848489	0.571630668	-3.2768583	0.88	-0.31	-3.85	0.57	-3.28	7054.9	TRUE	TRUE	TRUE	TRUE	TRUE	TRUE
1	GIYFAATEK	86	94	9	HLA-A*02:01	86	42	24518.35	42	0.907042	0.2012337	-4.3894912	1.108275697	-3.2812155	0.91	0.2	-4.39	1.11	-3.28	24518.3	TRUE	TRUE	TRUE	TRUE	TRUE	TRUE
1	PPLLTDDMI	844	852	9	HLA-A*02:01	844	70	34833.8	70	1.340019	-0.080591	-4.5420009	1.259427952	-3.2825729	1.34	-0.08	-4.54	1.26	-3.28	34833.8	TRUE	TRUE	TRUE	TRUE	TRUE	TRUE
1	NYKLPDDFM	409	417	9	HLA-A*02:01	409	69	34426.89	69	1.012606	0.239857861	-4.5368978	1.25246386	-3.2844339	1.01	0.24	-4.54	1.25	-3.28	34426.9	TRUE	TRUE	TRUE	TRUE	TRUE	TRUE
1	TNFRAILTA	229	237	9	HLA-A*02:01	229	28	16482.03	28	1.18301	-0.2540386	-4.2170107	0.928971357	-3.2880393	1.18	-0.25	-4.22	0.93	-3.29	16482	TRUE	TRUE	TRUE	TRUE	TRUE	TRUE
1	RAILTAFSP	232	240	9	HLA-A*02:01	232	30	17324.99	30	0.791268	0.157547112	-4.238673	0.948815112	-3.2898579	0.79	0.16	-4.24	0.95	-3.29	17325	TRUE	TRUE	TRUE	TRUE	TRUE	TRUE
1	KHLREFVFK	180	188	9	HLA-A*02:01	180	62	32426.38	62	1.002108	0.214264188	-4.5108985	1.216372188	-3.2945263	1	0.21	-4.51	1.22	-3.29	32426.4	TRUE	TRUE	TRUE	TRUE	TRUE	TRUE
1	EVAVLYQDV	593	601	9	HLA-A*02:01	593	32	18618.09	32	0.837819	0.137260781	-4.2699351	0.975079781	-3.2948553	0.84	0.14	-4.27	0.98	-3.29	18618.1	TRUE	TRUE	TRUE	TRUE	TRUE	TRUE
1	TAALVSGTA	856	864	9	HLA-A*02:01	856	33	19532.65	33	1.224282	-0.233116	-4.2907612	0.991166024	-3.2995951	1.22	-0.23	-4.29	0.99	-3.3	19532.7	TRUE	TRUE	TRUE	TRUE	TRUE	TRUE
1	IAYSNNTIA	687	695	9	HLA-A*02:01	687	25	14156.55	25	0.944954	-0.0941456	-4.1509574	0.850808394	-3.300149	0.94	-0.09	-4.15	0.85	-3.3	14156.5	TRUE	TRUE	TRUE	TRUE	TRUE	TRUE
1	VQIDRLITG	973	981	9	HLA-A*02:01	973	17	8170.43	17	1.19841	-0.5884552	-3.9122449	0.609954776	-3.3022901	1.2	-0.59	-3.91	0.61	-3.3	8170.4	TRUE	TRUE	TRUE	TRUE	TRUE	TRUE
1	AFSLDVSEK	167	175	9	HLA-A*02:01	167	69	34479.82	69	0.955371	0.2761158	-4.537565	1.231486799	-3.3060782	0.96	0.28	-4.54	1.23	-3.31	34479.8	TRUE	TRUE	TRUE	TRUE	TRUE	TRUE
1	SLQTYVTQQ	985	993	9	HLA-A*02:01	985	27	15487.54	27	0.931702	-0.0509623	-4.1899824	0.880739702	-3.3092427	0.93	-0.05	-4.19	0.88	-3.31	15487.5	TRUE	TRUE	TRUE	TRUE	TRUE	TRUE
1	VTQQLIRAA	990	998	9	HLA-A*02:01	990	28	16105.66	28	1.147289	-0.2503444	-4.2069785	0.896944559	-3.310034	1.15	-0.25	-4.21	0.9	-3.31	16105.7	TRUE	TRUE	TRUE	TRUE	TRUE	TRUE
1	SQIQESLTT	914	922	9	HLA-A*02:01	914	20	10249.72	20	0.912443	-0.2174711	-4.010712	0.694971862	-3.3157401	0.91	-0.22	-4.01	0.69	-3.32	10249.7	TRUE	TRUE	TRUE	TRUE	TRUE	TRUE
1	GLTGTGVLT	531	539	9	HLA-A*02:01	531	14	6273.84	14	0.809062	-0.3304359	-3.7975334	0.478626087	-3.3189074	0.81	-0.33	-3.8	0.48	-3.32	6273.8	TRUE	TRUE	TRUE	TRUE	TRUE	TRUE
1	LIDLQELGK	1179	1187	9	HLA-A*02:01	1179	49	27789.56	49	0.91557	0.207178686	-4.4438817	1.122748686	-3.321133	0.92	0.21	-4.44	1.12	-3.32	27789.6	TRUE	TRUE	TRUE	TRUE	TRUE	TRUE
1	RFPNITNLC	315	323	9	HLA-A*02:01	315	52	28681.25	52	0.935771	0.19865117	-4.4575981	1.13442217	-3.3231759	0.94	0.2	-4.46	1.13	-3.32	28681.2	TRUE	TRUE	TRUE	TRUE	TRUE	TRUE
1	IDRLNEVAK	1165	1173	9	HLA-A*02:01	1165	72	35252	72	1.195916	0.027186423	-4.5471838	1.223102423	-3.3240813	1.2	0.03	-4.55	1.22	-3.32	35252	TRUE	TRUE	TRUE	TRUE	TRUE	TRUE
1	DSEPVLKGV	1242	1250	9	HLA-A*02:01	1242	46	26372.02	46	1.203973	-0.107525	-4.4211434	1.096448004	-3.3246954	1.2	-0.11	-4.42	1.1	-3.32	26372	TRUE	TRUE	TRUE	TRUE	TRUE	TRUE
1	QIANQFNKA	904	912	9	HLA-A*02:01	904	25	14253.52	25	0.946183	-0.1190615	-4.1539221	0.827121526	-3.3268006	0.95	-0.12	-4.15	0.83	-3.33	14253.5	TRUE	TRUE	TRUE	TRUE	TRUE	TRUE
1	PHGVVFLHV	1039	1047	9	HLA-A*02:01	1039	36	21063.09	36	1.059394	-0.0740313	-4.3235221	0.985362695	-3.3381594	1.06	-0.07	-4.32	0.99	-3.34	21063.1	TRUE	TRUE	TRUE	TRUE	TRUE	TRUE
1	GECLGDINA	820	828	9	HLA-A*02:01	820	54	29579.45	54	1.517598	-0.3870499	-4.4709901	1.130548086	-3.340442	1.52	-0.39	-4.47	1.13	-3.34	29579.5	TRUE	TRUE	TRUE	TRUE	TRUE	TRUE
1	NFFSPQIIT	1090	1098	9	HLA-A*02:01	1090	49	27581.68	49	1.208193	-0.1113996	-4.4406207	1.096793409	-3.3438273	1.21	-0.11	-4.44	1.1	-3.34	27581.7	TRUE	TRUE	TRUE	TRUE	TRUE	TRUE
1	ATKFPSVYA	331	339	9	HLA-A*02:01	331	30	17684.07	30	1.021148	-0.1175678	-4.2475822	0.903580172	-3.3440021	1.02	-0.12	-4.25	0.9	-3.34	17684.1	TRUE	TRUE	TRUE	TRUE	TRUE	TRUE
1	NFTTAPAIC	1056	1064	9	HLA-A*02:01	1056	78	37194.32	78	1.088481	0.13671281	-4.5704766	1.225193809	-3.3452828	1.09	0.14	-4.57	1.23	-3.35	37194.3	TRUE	TRUE	TRUE	TRUE	TRUE	TRUE
1	FGGFNFSQI	779	787	9	HLA-A*02:01	779	28	16136.18	28	0.880153	-0.0187237	-4.2078007	0.86142934	-3.3463714	0.88	-0.02	-4.21	0.86	-3.35	16136.2	TRUE	TRUE	TRUE	TRUE	TRUE	TRUE
1	TNLCPFGEV	320	328	9	HLA-A*02:01	320	39	23102.36	39	0.944562	0.07186774	-4.3636563	1.01642974	-3.3472266	0.94	0.07	-4.36	1.02	-3.35	23102.4	TRUE	TRUE	TRUE	TRUE	TRUE	TRUE
1	MQMAYRFNG	882	890	9	HLA-A*02:01	882	9.3	3716	9.3	0.672525	-0.4573829	-3.5700757	0.215142055	-3.3549336	0.67	-0.46	-3.57	0.22	-3.35	3716	TRUE	TRUE	TRUE	TRUE	TRUE	TRUE
1	PLLTDDMIA	845	853	9	HLA-A*02:01	845	23	12869.44	23	1.238055	-0.4863567	-4.1095596	0.751698331	-3.3578613	1.24	-0.49	-4.11	0.75	-3.36	12869.4	TRUE	TRUE	TRUE	TRUE	TRUE	TRUE
1	EIDRLNEVA	1164	1172	9	HLA-A*02:01	1164	47	26770.47	47	1.363989	-0.2970092	-4.427656	1.066979819	-3.3606762	1.36	-0.3	-4.43	1.07	-3.36	26770.5	TRUE	TRUE	TRUE	TRUE	TRUE	TRUE
1	QNVLYENQK	895	903	9	HLA-A*02:01	895	89	40252.09	89	1.06269	0.1798865	-4.6047884	1.242576504	-3.3622119	1.06	0.18	-4.6	1.24	-3.36	40252.1	TRUE	TRUE	TRUE	TRUE	TRUE	TRUE

seq #	peptide	start	end	peptide length	allele	peptide index	median binding	ann IC50	ann percentile	proteasome sc	tap score	mhc score	processing scor	processing tota	legacy Proteas	legacy TAP Sco	legacy MHC Sc	legacy Process	legacy Total Sc	legacy MHC IC5	equal Proteaso	equal TAP Scor	equal MHC Sco	equal Processin	equal Total Sco	equal MHC IC50
1	LAATKMSEC	1006	1014	9	HLA-A*02:01	1006	60	31588.01	60	1.035805	0.09903276	-4.4995223	1.13483776	-3.3646845	1.04	0.1	-4.5	1.13	-3.36	31588	TRUE	TRUE	TRUE	TRUE	TRUE	TRUE
1	PFKDGIYFA	82	90	9	HLA-A*02:01	82	62	32291.24	62	1.507953	-0.3639439	-4.5090847	1.1440091	-3.3650756	1.51	-0.36	-4.51	1.14	-3.37	32291.2	TRUE	TRUE	TRUE	TRUE	TRUE	TRUE
1	SVKSFEIDK	289	297	9	HLA-A*02:01	289	64	32849.79	64	0.864542	0.281429341	-4.5165326	1.14597134	-3.3705613	0.86	0.28	-4.52	1.15	-3.37	32849.8	TRUE	TRUE	TRUE	TRUE	TRUE	TRUE
1	TNASSEVAV	588	596	9	HLA-A*02:01	588	43	25035.73	43	0.972819	0.05413108	-4.3985603	1.02695008	-3.3716102	0.97	0.05	-4.4	1.03	-3.37	25035.7	TRUE	TRUE	TRUE	TRUE	TRUE	TRUE
1	AYFVGYLKP	251	259	9	HLA-A*02:01	251	49	27730.7	49	0.733521	0.337293368	-4.4429608	1.07081437	-3.3721465	0.73	0.34	-4.44	1.07	-3.37	27730.7	TRUE	TRUE	TRUE	TRUE	TRUE	TRUE
1	PMGTQTHTM	143	151	9	HLA-A*02:01	143	39	22784.86	39	1.033893	-0.0509982	-4.3576464	0.982894791	-3.3747516	1.03	-0.05	-4.36	0.98	-3.37	22784.9	TRUE	TRUE	TRUE	TRUE	TRUE	TRUE
1	LAELKCSVK	283	291	9	HLA-A*02:01	283	60	31732.22	60	1.024236	0.101579339	-4.5015005	1.125815339	-3.3756851	1.02	0.1	-4.5	1.13	-3.38	31732.2	TRUE	TRUE	TRUE	TRUE	TRUE	TRUE
1	SQKSIVAYT	670	678	9	HLA-A*02:01	670	18	9150.26	18	0.833982	-0.2483818	-3.9614334	0.585600221	-3.3758332	0.83	-0.25	-3.96	0.59	-3.38	9150.3	TRUE	TRUE	TRUE	TRUE	TRUE	TRUE
1	QSLQTYVTQ	984	992	9	HLA-A*02:01	984	56	30072.86	56	1.090982	0.00468674	-4.4781747	1.09566874	-3.382506	1.09	0	-4.48	1.1	-3.38	30072.9	TRUE	TRUE	TRUE	TRUE	TRUE	TRUE
1	DMIAAYTAA	850	858	9	HLA-A*02:01	850	17	8489.72	17	0.888469	-0.3463736	-3.9288934	0.54209542	-3.3867979	0.89	-0.35	-3.93	0.54	-3.39	8489.7	TRUE	TRUE	TRUE	TRUE	TRUE	TRUE
1	IRACNFELC	125	133	9	HLA-A*02:01	125	46	26460.33	46	0.902494	0.132543454	-4.4225953	1.03503745	-3.3875578	0.9	0.13	-4.42	1.04	-3.39	26460.3	TRUE	TRUE	TRUE	TRUE	TRUE	TRUE
1	GTITDAVDC	270	278	9	HLA-A*02:01	270	58	30963.34	58	1.093162	0.00972133	-4.4908478	1.102883333	-3.3879645	1.09	0.01	-4.49	1.1	-3.39	30963.3	TRUE	TRUE	TRUE	TRUE	TRUE	TRUE
1	KEELDKYFK	1131	1139	9	HLA-A*02:01	1131	85	39045.02	85	1.119509	0.08178358	-4.5915656	1.20129258	-3.3902731	1.12	0.08	-4.59	1.2	-3.39	39045	TRUE	TRUE	TRUE	TRUE	TRUE	TRUE
1	NSTFFSTFK	357	365	9	HLA-A*02:01	357	57	30580.14	57	0.891681	0.1981279	-4.4854395	1.089808902	-3.3956306	0.89	0.2	-4.49	1.09	-3.4	30580.1	TRUE	TRUE	TRUE	TRUE	TRUE	TRUE
1	SCCSCLKGA	1221	1229	9	HLA-A*02:01	1221	33	19298.42	33	1.080051	-0.1921759	-4.2855218	0.887875062	-3.3976467	1.08	-0.19	-4.29	0.89	-3.4	19298.4	TRUE	TRUE	TRUE	TRUE	TRUE	TRUE
1	FMGCVLAWN	416	424	9	HLA-A*02:01	416	7.6	2674.95	7.6	0.717872	-0.6886922	-3.4273157	0.029179816	-3.3981359	0.72	-0.69	-3.43	0.03	-3.4	2674.9	TRUE	TRUE	TRUE	TRUE	TRUE	TRUE
1	LGFIAGLIA	1200	1208	9	HLA-A*02:01	1200	23	12989.46	23	1.036235	-0.3209053	-4.1135911	0.71532972	-3.3982614	1.04	-0.32	-4.11	0.72	-3.4	12989.5	TRUE	TRUE	TRUE	TRUE	TRUE	TRUE
1	SECVLGQSK	1012	1020	9	HLA-A*02:01	1012	69	34546.66	69	0.950956	0.185841426	-4.5384061	1.136797426	-3.4016086	0.95	0.19	-4.54	1.14	-3.4	34546.7	TRUE	TRUE	TRUE	TRUE	TRUE	TRUE
1	RCTTFDDVQ	18	26	9	HLA-A*02:01	18	84	38852.43	84	1.195797	-0.0083621	-4.5894182	1.187434863	-3.4019833	1.2	-0.01	-4.59	1.19	-3.4	38852.4	TRUE	TRUE	TRUE	TRUE	TRUE	TRUE
1	AIHADQLTP	609	617	9	HLA-A*02:01	609	48	27240.91	48	0.853192	0.179389365	-4.4352216	1.03258137	-3.4026402	0.85	0.18	-4.44	1.03	-3.4	27240.9	TRUE	TRUE	TRUE	TRUE	TRUE	TRUE
1	EIRASANLA	999	1007	9	HLA-A*02:01	999	51	28550.28	51	1.229307	-0.1763785	-4.4556104	1.052928548	-3.4026818	1.23	-0.18	-4.46	1.05	-3.4	28550.3	TRUE	TRUE	TRUE	TRUE	TRUE	TRUE
1	STDLIKNQC	516	524	9	HLA-A*02:01	516	51	28417.45	51	1.086588	-0.036417	-4.4535851	1.05017104	-3.4034141	1.09	-0.04	-4.45	1.05	-3.4	28417.5	TRUE	TRUE	TRUE	TRUE	TRUE	TRUE
1	SQNPLAELK	279	287	9	HLA-A*02:01	279	48	27395.49	48	0.819217	0.21411068	-4.4376791	1.033327679	-3.4043514	0.82	0.21	-4.44	1.03	-3.4	27395.5	TRUE	TRUE	TRUE	TRUE	TRUE	TRUE
1	NARDLICAQ	827	835	9	HLA-A*02:01	827	63	32720.67	63	1.092604	0.01710107	-4.5148222	1.10970507	-3.4051171	1.09	0.02	-4.51	1.11	-3.41	32720.7	TRUE	TRUE	TRUE	TRUE	TRUE	TRUE
1	SCLKGACSC	1224	1232	9	HLA-A*02:01	1224	51	28589.23	51	1.008389	0.041803532	-4.4562025	1.050192532	-3.4060099	1.01	0.04	-4.46	1.05	-3.41	28589.2	TRUE	TRUE	TRUE	TRUE	TRUE	TRUE
1	NQFNKAISQ	907	915	9	HLA-A*02:01	907	44	25462.72	44	0.881961	0.11751645	-4.4059048	0.99947745	-3.4064273	0.88	0.12	-4.41	1	-3.41	25462.7	TRUE	TRUE	TRUE	TRUE	TRUE	TRUE
1	QHTSSMRGV	32	40	9	HLA-A*02:01	32	70	34928.15	70	1.003626	0.13227347	-4.5431756	1.135899472	-3.4072761	1	0.13	-4.54	1.14	-3.41	34928.2	TRUE	TRUE	TRUE	TRUE	TRUE	TRUE
1	IPIGAGICA	650	658	9	HLA-A*02:01	650	42	24485.46	42	1.364353	-0.3838163	-4.3889083	0.980536694	-3.4083716	1.36	-0.38	-4.39	0.98	-3.41	24485.5	TRUE	TRUE	TRUE	TRUE	TRUE	TRUE
1	KSNVVRGWV	94	102	9	HLA-A*02:01	94	33	19318.48	33	0.791318	0.08559421	-4.285973	0.876912208	-3.4090607	0.79	0.09	-4.29	0.88	-3.41	19318.5	TRUE	TRUE	TRUE	TRUE	TRUE	TRUE
1	NRALSGIAA	746	754	9	HLA-A*02:01	746	56	30377.66	56	1.169544	-0.0972885	-4.4825543	1.07225554	-3.4102988	1.17	-0.1	-4.48	1.07	-3.41	30377.7	TRUE	TRUE	TRUE	TRUE	TRUE	TRUE
1	LSTDLIKNQ	515	523	9	HLA-A*02:01	515	75	36267.2	75	1.147235	0.00029101	-4.559514	1.147526008	-3.411988	1.15	0	-4.56	1.15	-3.41	36267.2	TRUE	TRUE	TRUE	TRUE	TRUE	TRUE
1	EVMPVSMAK	707	715	9	HLA-A*02:01	707	46	26207.61	46	0.798433	0.20602657	-4.4184274	1.00445957	-3.4139678	0.8	0.21	-4.42	1	-3.41	26207.6	TRUE	TRUE	TRUE	TRUE	TRUE	TRUE
1	SNVYADSFV	380	388	9	HLA-A*02:01	380	41	24017.38	41	0.884562	0.0802728	-4.3805256	0.964834797	-3.4156908	0.88	0.08	-4.38	0.96	-3.42	24017.4	TRUE	TRUE	TRUE	TRUE	TRUE	TRUE
1	FGNPVIPFK	76	84	9	HLA-A*02:01	76	40	23501.19	40	0.939614	0.00912458	-4.3710899	0.948738577	-3.4223513	0.94	0.01	-4.37	0.95	-3.42	23501.2	TRUE	TRUE	TRUE	TRUE	TRUE	TRUE
1	GPKLSTDLI	512	520	9	HLA-A*02:01	512	61	32025.05	61	1.065009	0.01712565	-4.5054898	1.08213465	-3.4233552	1.07	0.02	-4.51	1.08	-3.42	32025	TRUE	TRUE	TRUE	TRUE	TRUE	TRUE
1	SQILPDPLK	785	793	9	HLA-A*02:01	785	56	30169.99	56	0.813459	0.24249069	-4.4795752	1.05594969	-3.4236255	0.81	0.24	-4.48	1.06	-3.42	30170	TRUE	TRUE	TRUE	TRUE	TRUE	TRUE
1	YKGYQPIDV	197	205	9	HLA-A*02:01	197	30	17741.19	30	0.72623	0.09763239	-4.2489827	0.823862388	-3.4251204	0.73	0.1	-4.25	0.82	-3.43	17741.2	TRUE	TRUE	TRUE	TRUE	TRUE	TRUE
1	CTTFDDVQA	19	27	9	HLA-A*02:01	19	40	23283.28	40	1.15647	-0.2170238	-4.3670442	0.939446215	-3.4275979	1.16	-0.22	-4.37	0.94	-3.43	23283.3	TRUE	TRUE	TRUE	TRUE	TRUE	TRUE
1	SQERNFTTA	1052	1060	9	HLA-A*02:01	1052	32	18978.7	32	1.151639	-0.301718	-4.2782665	0.84992103	-3.4283454	1.15	-0.3	-4.28	0.85	-3.43	18978.7	TRUE	TRUE	TRUE	TRUE	TRUE	TRUE
1	EHVDTSYEC	640	648	9	HLA-A*02:01	640	92	41210.14	92	1.165952	0.020461469	-4.6150041	1.18641347	-3.4285906	1.17	0.02	-4.62	1.19	-3.43	41210.1	TRUE	TRUE	TRUE	TRUE	TRUE	TRUE
1	SKRVDFCGK	1019	1027	9	HLA-A*02:01	1019	74	35888.16	74	0.877199	0.246441955	-4.5549512	1.12364096	-3.4313102	0.88	0.25	-4.55	1.12	-3.43	35888.2	TRUE	TRUE	TRUE	TRUE	TRUE	TRUE
1	LREFVFKNK	182	190	9	HLA-A*02:01	182	62	32338.44	62	0.856255	0.221327803	-4.5097191	1.077582803	-3.4321363	0.86	0.22	-4.51	1.08	-3.43	32338.4	TRUE	TRUE	TRUE	TRUE	TRUE	TRUE
1	SFVVKGDDV	386	394	9	HLA-A*02:01	386	50	28124.71	50	0.809015	0.204470564	-4.4490881	1.013485564	-3.4356025	0.81	0.2	-4.45	1.01	-3.44	28124.7	TRUE	TRUE	TRUE	TRUE	TRUE	TRUE
1	NYTQHTSSM	29	37	9	HLA-A*02:01	29	84	38610.22	84	0.963342	0.18455329	-4.5867023	1.147895289	-3.438807	0.96	0.18	-4.59	1.15	-3.44	38610.2	TRUE	TRUE	TRUE	TRUE	TRUE	TRUE
1	APAICHEGK	1060	1068	9	HLA-A*02:01	1060	76	36488.79	76	0.976695	0.144553918	-4.5621595	1.12124892	-3.4409105	0.98	0.14	-4.56	1.12	-3.44	36488.8	TRUE	TRUE	TRUE	TRUE	TRUE	TRUE
1	FNTLKPIFK	213	221	9	HLA-A*02:01	213	51	28399	51	0.945457	0.06678252	-4.453303	1.01223952	-3.4410635	0.95	0.07	-4.45	1.01	-3.44	28399	TRUE	TRUE	TRUE	TRUE	TRUE	TRUE
1	TFKCYGVSA	363	371	9	HLA-A*02:01	363	53	29307.39	53	1.168741	-0.1453756	-4.4669771	1.02336544	-3.4436117	1.17	-0.15	-4.47	1.02	-3.44	29307.4	TRUE	TRUE	TRUE	TRUE	TRUE	TRUE
1	VDTSYECDI	642	650	9	HLA-A*02:01	642	76	36567.44	76	1.020457	0.09887328	-4.5630946	1.11933028	-3.4437643	1.02	0.1	-4.56	1.12	-3.44	36567.4	TRUE	TRUE	TRUE	TRUE	TRUE	TRUE
1	FLHVTYVPS	1044	1052	9	HLA-A*02:01	1044	4.7	1187.08	4.7	0.605095	-0.9756133	-3.07448	-0.3705183	-3.4449983	0.61	-0.98	-3.07	-0.37	-3.44	1187.1	TRUE	TRUE	TRUE	TRUE	TRUE	TRUE
1	PCSFGGVSV	575	583	9	HLA-A*02:01	575	54	29463.18	54	1.06725	-0.0432709	-4.4692796	1.023979148	-3.4453005	1.07	-0.04	-4.47	1.02	-3.45	29463.2	TRUE	TRUE	TRUE	TRUE	TRUE	TRUE
1	GQSKRVDFC	1017	1025	9	HLA-A*02:01	1017	42	24274.18	42	0.956735	-0.0207784	-4.3851446	0.93595664	-3.4491879	0.96	-0.02	-4.39	0.94	-3.45	24274.2	TRUE	TRUE	TRUE	TRUE	TRUE	TRUE
1	VFGSTMNNK	102	110	9	HLA-A*02:01	102	74	35957.34	74	0.897743	0.207432004	-4.5557876	1.105175	-3.4506126	0.9	0.21	-4.56	1.11	-3.45	35957.3	TRUE	TRUE	TRUE	TRUE	TRUE	TRUE
1	DDMIAAYTA	849	857	9	HLA-A*02:01	849	49	27516.69	49	1.454936	-0.4701344	-4.4395962	0.9848016	-3.4547946	1.45	-0.47	-4.44	0.98	-3.45	27516.7	TRUE	TRUE	TRUE	TRUE	TRUE	TRUE
1	GYLKPTTFM	255	263	9	HLA-A*02:01	255	48	26974	48	0.776132	0.19711221	-4.4309454	0.973244213	-3.4577011	0.78	0.2	-4.43	0.97	-3.46	26974	TRUE	TRUE	TRUE	TRUE	TRUE	TRUE
1	YSTGNNVFQ	622	630	9	HLA-A*02:01	622	60	31534.74	60	1.09059	-0.0500205	-4.4987893	1.04056954	-3.4582197	1.09	-0.05	-4.5	1.04	-3.46	31534.7	TRUE	TRUE	TRUE	TRUE	TRUE	TRUE
1	GVSVITPGT	580	588	9	HLA-A*02:01	580	21	11201.74	21	0.93001	-0.3397301	-4.0492855	0.590279874	-3.4590056	0.93	-0.34	-4.05	0.59	-3.46	11201.7	TRUE	TRUE	TRUE	TRUE	TRUE	TRUE
1	FAVSKPMGT	138	146	9	HLA-A*02:01	138	19	9917.21	19	0.861793	-0.3368635	-3.9963895	0.524929475	-3.47146	0.86	-0.34	-4	0.52	-3.47	9917.2	TRUE	TRUE	TRUE	TRUE	TRUE	TRUE
1	AQVKQMYKT	765	773	9	HLA-A*02:01	765	28	16403.57	28	0.966766	-0.2233979	-4.2149384	0.743368075	-3.4715703	0.97	-0.22	-4.21	0.74	-3.47	16403.6	TRUE	TRUE	TRUE	TRUE	TRUE	TRUE
1	FNFNGLTGT	527	535	9	HLA-A*02:01	527	22	11889.74	22	0.906498	-0.3037477	-4.0751724	0.60275033	-3.472422	0.91	-0.3	-4.08	0.6	-3.47	11889.7	TRUE	TRUE	TRUE	TRUE	TRUE	TRUE
1	SVYAWERKK	336	344	9	HLA-A*02:01	336	72	35478.91	72	0.773719	0.3036416	-4.5499703	1.0773606	-3.4726097	0.77	0.3	-4.55	1.08	-3.47	35478.9	TRUE	TRUE	TRUE	TRUE	TRUE	TRUE
1	FGGVSVITP	578	586	9	HLA-A*02:01	578	36	21023.71	36	1.009386	-0.1601895	-4.3227094	0.849196522	-3.4735128	1.01	-0.16	-4.32	0.85	-3.47	21023.7	TRUE	TRUE	TRUE	TRUE	TRUE	TRUE
1	GDINARDLI	824	832	9	HLA-A*02:01	824	80	37590.82	80	1.029133	0.07064613	-4.5750818	1.09977913	-3.4753027	1.03	0.07	-4.58	1.1	-3.48	37590.8	TRUE	TRUE	TRUE	TRUE	TRUE	TRUE
1	RSDTLYLTQ	48	56	9	HLA-A*02:01	48	52	28777.91	52	1.02743	-0.0453885	-4.4590593	0.982041494	-3.4770178	1.03	-0.05	-4.46	0.98	-3.48	28777.9	TRUE	TRUE	TRUE	TRUE	TRUE	TRUE
1	PFFAVSKPM	136	144	9	HLA-A*02:01	136	44	25491.37	44	0.822029	0.10656619	-4.4063932	0.92859519	-3.477798	0.82	0.11	-4.41	0.93	-3.48	25491.4	TRUE	TRUE	TRUE	TRUE	TRUE	TRUE
1	LSRLDKVEA	963	971	9	HLA-A*02:01	963	59	31247.39	59	1.19732	-0.1870138	-4.4948137	1.01030623	-3.4845075	1.2	-0.19	-4.49	1.01	-3.48	31247.4	TRUE	TRUE	TRUE	TRUE	TRUE	TRUE
1	ITNFRAILT	228	236	9	HLA-A*02:01	228	26	15172.4	26	0.980452	-0.2857105	-4.1810543	0.694741513	-3.4863128	0.98	-0.29	-4.18	0.69	-3.49	15172.4	TRUE	TRUE	TRUE	TRUE	TRUE	TRUE
1	KDGFLYVYK	190	198	9	HLA-A*02:01	190	54	29404.59	54	0.875323	0.105973448	-4.4684151	0.981296448	-3.4871187	0.88	0.11	-4.47	0.98	-3.49	29404.6	TRUE	TRUE	TRUE	TRUE	TRUE	TRUE
1	DINARDLIC	825	833	9	HLA-A*02:01	825	64	32828.11	64	1.15813	-0.1297918	-4.5162459	1.028338195	-3.4879077	1.16	-0.13	-4.52	1.03	-3.49	32828.1	TRUE	TRUE	TRUE	TRUE	TRUE	TRUE
1	SFCTQLNRA	740	748	9	HLA-A*02:01	740	44	25434.9	44	1.056827	-0.1449497	-4.40543	0.911877266	-3.4935528	1.06	-0.14	-4.41	0.91	-3.49	25434.9	TRUE	TRUE	TRUE	TRUE	TRUE	TRUE
1	PQAAPHGVV	1035	1043	9	HLA-A*02:01	1035	67	34029.52	67	1.116178	-0.0789055	-4.5318558	1.037272466	-3.4945834	1.12	-0.08	-4.53	1.04	-3.49	34029.5	TRUE	TRUE	TRUE	TRUE	TRUE	TRUE
1	QNPLAELKC	280	288	9	HLA-A*02:01	280	83	38354.16	83	1.121982	-0.0378802	-4.5838125	1.08410185	-3.4997106	1.12	-0.04	-4.58	1.08	-3.5	38354.2	TRUE	TRUE	TRUE	TRUE	TRUE	TRUE
1	LTVLPPLLT	840	848	9	HLA-A*02:01	840	27	15684.84	27	0.982075	-0.2937426	-4.1954801	0.688332434	-3.5071477	0.98	-0.29	-4.2	0.69	-3.51	15684.8	TRUE	TRUE	TRUE	TRUE	TRUE	TRUE
1	ITDAVDCSQ	272	280	9	HLA-A*02:01	272	47	26618.27	47	1.03677	-0.1188291	-4.4251798	0.917940852	-3.507239	1.04	-0.12	-4.43	0.92	-3.51	26618.3	TRUE	TRUE	TRUE	TRUE	TRUE	TRUE
1	HEGKAYFPR	1065	1073	9	HLA-A*02:01	1065	76	36465.12	76	0.579268	0.47162488	-4.5618776	1.050892881	-3.5109848	0.58	0.47	-4.56	1.05	-3.51	36465.1	TRUE	TRUE	TRUE	TRUE	TRUE	TRUE
1	GTNASSEVA	587	595	9	HLA-A*02:01	587	40	23454.2	40	1.27834	-0.4225404	-4.3702206	0.85579959	-3.514421	1.28	-0.42	-4.37	0.86	-3.51	23454.2	TRUE	TRUE	TRUE	TRUE	TRUE	TRUE
1	DIPIGAGIC	649	657	9	HLA-A*02:01	649	70	34698	70	1.110746	-0.0853705	-4.5403044	1.025375502	-3.5149289	1.11	-0.09	-4.54	1.03	-3.51	34698	TRUE	TRUE	TRUE	TRUE	TRUE	TRUE
1	YFGGFNFSQ	778	786	9	HLA-A*02:01	778	34	19948.27	34	0.792314	-0.0096748	-4.2999052	0.78263916	-3.5172661	0.79	-0.01	-4.3	0.78	-3.52	19948.3	TRUE	TRUE	TRUE	TRUE	TRUE	TRUE
1	LQPELDSFK	1123	1131	9	HLA-A*02:01	1123	53	29286.15	53	0.780367	0.167635657	-4.4666623	0.948002657	-3.5186596	0.78	0.17	-4.47	0.95	-3.52	29286.2	TRUE	TRUE	TRUE	TRUE	TRUE	TRUE
1	KLQDVVNQN	929	937	9	HLA-A*02:01	929	26	14583.15	26	1.185109	-0.5413714	-4.1638513	0.643737608	-3.5201137	1.19	-0.54	-4.16	0.64	-3.52	14583.1	TRUE	TRUE	TRUE	TRUE	TRUE	TRUE
1	GACSCGSCC	1228	1236	9	HLA-A*02:01	1228	66	33435.36	66	1.003749	-0.0021068	-4.524206	1.001642198	-3.5225638	1	0	-4.52	1	-3.52	33435.4	TRUE	TRUE	TRUE	TRUE	TRUE	TRUE
1	FDEDDSEPV	1238	1246	9	HLA-A*02:01	1238	21	11257.51	21	0.584356	-0.0581403	-4.0514423	0.52621569	-3.5252267	0.58	-0.06	-4.05	0.53	-3.53	11257.5	TRUE	TRUE	TRUE	TRUE	TRUE	TRUE
1	REVFAQVKQ	761	769	9	HLA-A*02:01	761	89	40139.9	89	1.04033	0.03789854	-4.6035763	1.078228543	-3.5253477	1.04	0.04	-4.6	1.08	-3.53	40139.9	TRUE	TRUE	TRUE	TRUE	TRUE	TRUE
1	TTFDDVQAP	20	28	9	HLA-A*02:01	20	45	25888.57	45	0.811769	0.07093953	-4.4131081	0.88270853	-3.5303995	0.81	0.07	-4.41	0.88	-3.53	25888.6	TRUE	TRUE	TRUE	TRUE	TRUE	TRUE
1	CCSCLKGAC	1222	1230	9	HLA-A*02:01	1222	72	35357.05	72	1.000002	0.017569299	-4.548476	1.0175713	-3.5309047	1	0.02	-4.55	1.02	-3.53	35357.1	TRUE	TRUE	TRUE	TRUE	TRUE	TRUE
1	PVSMAKTSV	710	718	9	HLA-A*02:01	710	58	31052.59	58	0.958423	0.00071214	-4.4920978	0.959135138	-3.5329627	0.96	0	-4.49	0.96	-3.53	31052.6	TRUE	TRUE	TRUE	TRUE	TRUE	TRUE
1	CPFGEVFNA	323	331	9	HLA-A*02:01	323	26	14616.32	26	0.942049	-0.3119053	-4.164838	0.630143657	-3.5346944	0.94	-0.31	-4.16	0.63	-3.53	14616.3	TRUE	TRUE	TRUE	TRUE	TRUE	TRUE
1	QTSNFRVVP	301	309	9	HLA-A*02:01	301	70	34763.78	70	0.988489	0.015867336	-4.541127	1.004356336	-3.5367707	0.99	0.02	-4.54	1	-3.54	34763.8	TRUE	TRUE	TRUE	TRUE	TRUE	TRUE
1	LPDPLKPTK	788	796	9	HLA-A*02:01	788	86	39216.92	86	1.053815	1.60E-05	-4.5934735	1.053830961	-3.5396425	1.05	0	-4.59	1.05	-3.54	39216.9	TRUE	TRUE	TRUE	TRUE	TRUE	TRUE
1	TSGSDLDRC	11	19	9	HLA-A*02:01	11	79	37324.12	79	1.066473	-0.0361469	-4.5719896	1.030326072	-3.5416635	1.07	-0.04	-4.57	1.03	-3.54	37324.1	TRUE	TRUE	TRUE	TRUE	TRUE	TRUE
1	NDYGFYTTT	479	487	9	HLA-A*02:01	479	51	28561.71	51	1.232687	-0.3219501	-4.4557842	0.910736874	-3.5450473	1.23	-0.32	-4.46	0.91	-3.55	28561.7	TRUE	TRUE	TRUE	TRUE	TRUE	TRUE
1	GKLQDVVNQ	928	936	9	HLA-A*02:01	928	76	36571.8	76	1.092814	-0.0748913	-4.5631463	1.01792269	-3.5452236	1.09	-0.07	-4.56	1.02	-3.55	36571.8	TRUE	TRUE	TRUE	TRUE	TRUE	TRUE
1	TILLCCMTS	1213	1221	9	HLA-A*02:01	1213	12	4600.05	12	1.018739	-0.9018815	-3.6627626	0.116857507	-3.545905	1.02	-0.9	-3.66	0.12	-3.55	4600.1	TRUE	TRUE	TRUE	TRUE	TRUE	TRUE
1	CTDVSTAIH	603	611	9	HLA-A*02:01	603	60	31564.1	60	1.22691	-0.2737836	-4.4991934	0.953126408	-3.546067	1.23	-0.27	-4.5	0.95	-3.55	31564.1	TRUE	TRUE	TRUE	TRUE	TRUE	TRUE
1	INHTFGNPV	72	80	9	HLA-A*02:01	72	28	16426.31	28	0.573143	0.09609406	-4.21554	0.669237063	-3.546303	0.57	0.1	-4.22	0.67	-3.55	16426.3	TRUE	TRUE	TRUE	TRUE	TRUE	TRUE
1	NGTSWFITQ	1080	1088	9	HLA-A*02:01	1080	60	31728.1	60	1.15924	-0.2077334	-4.5014441	0.9515066	-3.5499375	1.16	-0.21	-4.5	0.95	-3.55	31728.1	TRUE	TRUE	TRUE	TRUE	TRUE	TRUE
1	YLRHGKLRP	442	450	9	HLA-A*02:01	442	40	23509.85	40	0.694609	0.122611786	-4.3712499	0.81722079	-3.5540291	0.69	0.12	-4.37	0.82	-3.55	23509.8	TRUE	TRUE	TRUE	TRUE	TRUE	TRUE
1	YFVGYLKPT	252	260	9	HLA-A*02:01	252	20	10835.89	20	0.620899	-0.1418591	-4.0348646	0.479039939	-3.5558246	0.62	-0.14	-4.03	0.48	-3.56	10835.9	TRUE	TRUE	TRUE	TRUE	TRUE	TRUE
1	TSTGNYNYK	431	439	9	HLA-A*02:01	431	90	40422.3	90	0.788214	0.26187428	-4.606621	1.05008828	-3.5565327	0.79	0.26	-4.61	1.05	-3.56	40422.3	TRUE	TRUE	TRUE	TRUE	TRUE	TRUE
1	SPDVDLGDI	1143	1151	9	HLA-A*02:01	1143	57	30737.37	57	0.877564	0.04970507	-4.4876667	0.927269067	-3.5603976	0.88	0.05	-4.49	0.93	-3.56	30737.4	TRUE	TRUE	TRUE	TRUE	TRUE	TRUE
1	PSVYAWERK	335	343	9	HLA-A*02:01	335	90	40462.57	90	1.046658	-0.000465	-4.6070535	1.046192989	-3.5608605	1.05	0	-4.61	1.05	-3.56	40462.6	TRUE	TRUE	TRUE	TRUE	TRUE	TRUE
1	TGNNVFQTQ	624	632	9	HLA-A*02:01	624	71	35004.95	71	1.172605	-0.1896876	-4.5441295	0.982917372	-3.5612121	1.17	-0.19	-4.54	0.98	-3.56	35004.9	TRUE	TRUE	TRUE	TRUE	TRUE	TRUE
1	LYENQKQIA	898	906	9	HLA-A*02:01	898	94	41927.51	94	1.207779	-0.156917	-4.6224991	1.05086196	-3.5716371	1.21	-0.16	-4.62	1.05	-3.57	41927.5	TRUE	TRUE	TRUE	TRUE	TRUE	TRUE
1	DNPFFAVSK	134	142	9	HLA-A*02:01	134	68	34192.66	68	0.971718	-0.0101743	-4.5339329	0.96154369	-3.5723892	0.97	-0.01	-4.53	0.96	-3.57	34192.7	TRUE	TRUE	TRUE	TRUE	TRUE	TRUE
1	SQSVIIINN	111	119	9	HLA-A*02:01	111	34	19817.48	34	1.193992	-0.4720003	-4.2970484	0.72199174	-3.5750567	1.19	-0.47	-4.3	0.72	-3.58	19817.5	TRUE	TRUE	TRUE	TRUE	TRUE	TRUE
1	RKKISNCVA	342	350	9	HLA-A*02:01	342	80	37609.51	80	1.174275	-0.1774883	-4.5752977	0.99678671	-3.578511	1.17	-0.18	-4.58	1	-3.58	37609.5	TRUE	TRUE	TRUE	TRUE	TRUE	TRUE
1	FAQVKQMYK	764	772	9	HLA-A*02:01	764	48	27026.59	48	0.658882	0.19056149	-4.4317913	0.849443486	-3.5823478	0.66	0.19	-4.43	0.85	-3.58	27026.6	TRUE	TRUE	TRUE	TRUE	TRUE	TRUE
1	PFQQFGRDV	547	555	9	HLA-A*02:01	547	64	33049.07	64	0.971394	-0.0356627	-4.5191592	0.935731314	-3.5834279	0.97	-0.04	-4.52	0.94	-3.58	33049.1	TRUE	TRUE	TRUE	TRUE	TRUE	TRUE
1	HLREFVFKN	181	189	9	HLA-A*02:01	181	24	13623.84	24	1.083231	-0.535389	-4.1342995	0.547842022	-3.5864575	1.08	-0.54	-4.13	0.55	-3.59	13623.8	TRUE	TRUE	TRUE	TRUE	TRUE	TRUE
1	LQELGKYEQ	1182	1190	9	HLA-A*02:01	1182	72	35339.09	72	1.096511	-0.1354982	-4.5482554	0.96101276	-3.5872426	1.1	-0.14	-4.55	0.96	-3.59	35339.1	TRUE	TRUE	TRUE	TRUE	TRUE	TRUE
1	FEIDKGIYQ	293	301	9	HLA-A*02:01	293	54	29461.27	54	0.97346	-0.0936145	-4.4692515	0.879845483	-3.589406	0.97	-0.09	-4.47	0.88	-3.59	29461.3	TRUE	TRUE	TRUE	TRUE	TRUE	TRUE
1	ISITTEVMP	702	710	9	HLA-A*02:01	702	54	29481.03	54	0.75285	0.124638959	-4.4695427	0.877488959	-3.5920537	0.75	0.12	-4.47	0.88	-3.59	29481	TRUE	TRUE	TRUE	TRUE	TRUE	TRUE
1	SSEVAVLYQ	591	599	9	HLA-A*02:01	591	44	25490.27	44	0.816868	-0.0025924	-4.4063744	0.814275587	-3.5920988	0.82	0	-4.41	0.81	-3.59	25490.3	TRUE	TRUE	TRUE	TRUE	TRUE	TRUE
1	VSEKSGNFK	172	180	9	HLA-A*02:01	172	84	38843.19	84	0.872795	0.123945158	-4.5893149	0.996740158	-3.5925747	0.87	0.12	-4.59	1	-3.59	38843.2	TRUE	TRUE	TRUE	TRUE	TRUE	TRUE
1	STGNNVFQT	623	631	9	HLA-A*02:01	623	24	13116.85	24	0.911073	-0.3866512	-4.1178296	0.52442179	-3.5934078	0.91	-0.39	-4.12	0.52	-3.59	13116.9	TRUE	TRUE	TRUE	TRUE	TRUE	TRUE
1	RASANLAAT	1001	1009	9	HLA-A*02:01	1001	30	17348.62	30	0.817828	-0.1758133	-4.2392649	0.64201466	-3.5972503	0.82	-0.18	-4.24	0.64	-3.6	17348.6	TRUE	TRUE	TRUE	TRUE	TRUE	TRUE
1	KFNGLTVLP	836	844	9	HLA-A*02:01	836	49	27519.37	49	0.73952	0.101266376	-4.4396385	0.840786376	-3.5988521	0.74	0.1	-4.44	0.84	-3.6	27519.4	TRUE	TRUE	TRUE	TRUE	TRUE	TRUE
1	TGVLTPSSK	535	543	9	HLA-A*02:01	535	85	38994.79	85	0.894119	0.09793211	-4.5910066	0.99205111	-3.5989555	0.89	0.1	-4.59	0.99	-3.6	38994.8	TRUE	TRUE	TRUE	TRUE	TRUE	TRUE
1	TEVMPVSMA	706	714	9	HLA-A*02:01	706	49	27646.81	49	1.101298	-0.2618731	-4.441645	0.83942493	-3.6022201	1.1	-0.26	-4.44	0.84	-3.6	27646.8	TRUE	TRUE	TRUE	TRUE	TRUE	TRUE
1	QALNTLVKQ	939	947	9	HLA-A*02:01	939	56	30397.04	56	0.82439	0.056168384	-4.4828313	0.880558384	-3.6022729	0.82	0.06	-4.48	0.88	-3.6	30397	TRUE	TRUE	TRUE	TRUE	TRUE	TRUE
1	QVKQMYKTP	766	774	9	HLA-A*02:01	766	87	39719.54	87	0.819678	0.17570364	-4.5990042	0.995381635	-3.6036226	0.82	0.18	-4.6	1	-3.6	39719.5	TRUE	TRUE	TRUE	TRUE	TRUE	TRUE
1	HTFGNPVIP	74	82	9	HLA-A*02:01	74	48	27167.31	48	0.723263	0.105071885	-4.4340466	0.828334885	-3.6057118	0.72	0.11	-4.43	0.83	-3.61	27167.3	TRUE	TRUE	TRUE	TRUE	TRUE	TRUE
1	SATKLNDLC	370	378	9	HLA-A*02:01	370	70	34697.64	70	0.878687	0.05576409	-4.5402999	0.934451085	-3.6058489	0.88	0.06	-4.54	0.93	-3.61	34697.6	TRUE	TRUE	TRUE	TRUE	TRUE	TRUE
1	FTTAPAICH	1057	1065	9	HLA-A*02:01	1057	54	29529.57	54	1.098962	-0.2360465	-4.4702571	0.862915514	-3.6073416	1.1	-0.24	-4.47	0.86	-3.61	29529.6	TRUE	TRUE	TRUE	TRUE	TRUE	TRUE
1	SSMRGVYYP	35	43	9	HLA-A*02:01	35	30	17887.29	30	0.528656	0.11628818	-4.2525445	0.64494418	-3.6076004	0.53	0.12	-4.25	0.64	-3.61	17887.3	TRUE	TRUE	TRUE	TRUE	TRUE	TRUE
1	AVSKPMGTQ	139	147	9	HLA-A*02:01	139	79	37425.24	79	0.93675	0.028418919	-4.5731646	0.965168919	-3.6079957	0.94	0.03	-4.57	0.97	-3.61	37425.2	TRUE	TRUE	TRUE	TRUE	TRUE	TRUE
1	TDVSTAIHA	604	612	9	HLA-A*02:01	604	53	29031.24	53	1.192935	-0.3436823	-4.4628656	0.8492527	-3.6136129	1.19	-0.34	-4.46	0.85	-3.61	29031.2	TRUE	TRUE	TRUE	TRUE	TRUE	TRUE
1	GVIADYNYK	403	411	9	HLA-A*02:01	403	56	30202.66	56	0.647278	0.218852784	-4.4800452	0.866130784	-3.6139144	0.65	0.22	-4.48	0.87	-3.61	30202.7	TRUE	TRUE	TRUE	TRUE	TRUE	TRUE
1	YRFNGIGVT	886	894	9	HLA-A*02:01	886	44	25356.32	44	0.89986	-0.1102839	-4.4040862	0.78957608	-3.6145101	0.9	-0.11	-4.4	0.79	-3.61	25356.3	TRUE	TRUE	TRUE	TRUE	TRUE	TRUE
1	TQNVLYENQ	894	902	9	HLA-A*02:01	894	61	31997.7	61	0.932602	-0.0443299	-4.5051188	0.888272078	-3.6168467	0.93	-0.04	-4.51	0.89	-3.62	31997.7	TRUE	TRUE	TRUE	TRUE	TRUE	TRUE
1	FYSNVTGFH	62	70	9	HLA-A*02:01	62	60	31744.93	60	1.103791	-0.2224366	-4.5016744	0.88135436	-3.62032	1.1	-0.22	-4.5	0.88	-3.62	31744.9	TRUE	TRUE	TRUE	TRUE	TRUE	TRUE
1	APHGVVFLH	1038	1046	9	HLA-A*02:01	1038	42	24249.51	4.20E+01	9.84E-01	-0.2210303	-4.384703	0.76314269	-3.6215603	0.98	-0.22	-4.38	0.76	-3.62	24249.5	TRUE	TRUE	TRUE	TRUE	TRUE	TRUE
1	EVFNATKFP	327	335	9	HLA-A*02:01	327	76	36410.7	7.60E+01	8.85E-01	0.05096313	-4.561229	0.936062133	-3.6251669	0.89	0.05	-4.56	0.94	-3.63	36410.7	TRUE	TRUE	TRUE	TRUE	TRUE	TRUE
1	VSLLRSTSQ	663	671	9	HLA-A*02:01	663	68	34169.36	6.80E+01	8.94E-01	0.00927276	-4.5336368	0.903121759	-3.6305151	0.89	0.01	-4.53	0.9	-3.63	34169.4	TRUE	TRUE	TRUE	TRUE	TRUE	TRUE
1	SEILDISPC	568	576	9	HLA-A*02:01	568	42	24300.47	4.20E+01	7.51E-01	0.00363415	-4.3856147	0.754643151	-3.6309715	0.75	0	-4.39	0.75	-3.63	24300.5	TRUE	TRUE	TRUE	TRUE	TRUE	TRUE
1	PKLSTDLIK	513	521	9	HLA-A*02:01	513	90	40408.3	9.00E+01	9.63E-01	0.010161097	-4.6064706	0.973303097	-3.6331675	0.96	0.01	-4.61	0.97	-3.63	40408.3	TRUE	TRUE	TRUE	TRUE	TRUE	TRUE
1	QDVVNQNAQ	931	939	9	HLA-A*02:01	931	98	43558.91	9.80E+01	1.13E+00	-0.1205355	-4.639077	1.005907495	-3.6331695	1.13	-0.12	-4.64	1.01	-3.63	43558.9	TRUE	TRUE	TRUE	TRUE	TRUE	TRUE
1	LTGTGVLTP	532	540	9	HLA-A*02:01	532	43	25018.39	4.30E+01	8.11E-01	-0.0472301	-4.3982594	0.764226866	-3.6340325	0.81	-0.05	-4.4	0.76	-3.63	25018.4	TRUE	TRUE	TRUE	TRUE	TRUE	TRUE
1	MLKYDENGT	263	271	9	HLA-A*02:01	263	26	14673.52	2.60E+01	8.56E-01	-0.3241709	-4.1665343	0.531796144	-3.6347382	0.86	-0.32	-4.17	0.53	-3.63	14673.5	TRUE	TRUE	TRUE	TRUE	TRUE	TRUE
1	LDKVEAEVQ	966	974	9	HLA-A*02:01	966	85	39019.69	8.50E+01	1.08E+00	-0.1222683	-4.5912838	0.95619175	-3.6350921	1.08	-0.12	-4.59	0.96	-3.64	39019.7	TRUE	TRUE	TRUE	TRUE	TRUE	TRUE
1	LMSFPQAAP	1031	1039	9	HLA-A*02:01	1031	33	19502.44	3.30E+01	5.65E-01	0.08652933	-4.290089	0.651241328	-3.6388476	0.56	0.09	-4.29	0.65	-3.64	19502.4	TRUE	TRUE	TRUE	TRUE	TRUE	TRUE
1	LADAGFMKQ	810	818	9	HLA-A*02:01	810	56	30307.06	5.60E+01	9.36E-01	-0.0945847	-4.4815438	0.84107428	-3.6404695	0.94	-0.09	-4.48	0.84	-3.64	30307.1	TRUE	TRUE	TRUE	TRUE	TRUE	TRUE
1	TLKPIFKLP	215	223	9	HLA-A*02:01	215	39	23087.61	3.90E+01	6.19E-01	0.10383944	-4.363379	0.72284644	-3.6405325	0.62	0.1	-4.36	0.72	-3.64	23087.6	TRUE	TRUE	TRUE	TRUE	TRUE	TRUE
1	TTTSTALGK	921	929	9	HLA-A*02:01	921	72	35298.57	7.20E+01	7.14E-01	0.1890577	-4.5477571	0.903197704	-3.6445594	0.71	0.19	-4.55	0.9	-3.64	35298.6	TRUE	TRUE	TRUE	TRUE	TRUE	TRUE
1	VVKGDDVRQ	388	396	9	HLA-A*02:01	388	84	38863.79	8.40E+01	9.51E-01	-0.0064716	-4.5895452	0.94489439	-3.6446508	0.95	-0.01	-4.59	0.94	-3.64	38863.8	TRUE	TRUE	TRUE	TRUE	TRUE	TRUE
1	APGQTGVIA	398	406	9	HLA-A*02:01	398	58	30789.29	5.80E+01	1.21E+00	-0.3674707	-4.4883997	0.843251321	-3.6451484	1.21	-0.37	-4.49	0.84	-3.65	30789.3	TRUE	TRUE	TRUE	TRUE	TRUE	TRUE
1	SKPMGTQTH	141	149	9	HLA-A*02:01	141	89	40337.56	8.90E+01	1.13E+00	-0.1733819	-4.6057096	0.95773713	-3.6479725	1.13	-0.17	-4.61	0.96	-3.65	40337.6	TRUE	TRUE	TRUE	TRUE	TRUE	TRUE
1	FGEVFNATK	325	333	9	HLA-A*02:01	325	46	26393.42	4.60E+01	8.53E-01	-0.0799614	-4.4214957	0.773498597	-3.6479971	0.85	-0.08	-4.42	0.77	-3.65	26393.4	TRUE	TRUE	TRUE	TRUE	TRUE	TRUE
1	FRVVPSGDV	305	313	9	HLA-A*02:01	305	51	28510.15	5.10E+01	5.25E-01	0.27896756	-4.4549995	0.80368056	-3.6513189	0.52	0.28	-4.45	0.8	-3.65	28510.2	TRUE	TRUE	TRUE	TRUE	TRUE	TRUE
1	PKTSEILDI	565	573	9	HLA-A*02:01	565	77	36750.69	7.70E+01	9.45E-01	-0.0319087	-4.5652655	0.913369296	-3.6518962	0.95	-0.03	-4.57	0.91	-3.65	36750.7	TRUE	TRUE	TRUE	TRUE	TRUE	TRUE
1	LYVYKGYQP	194	202	9	HLA-A*02:01	194	83	38323.45	8.30E+01	7.81E-01	0.14902633	-4.5834646	0.93029633	-3.6531683	0.78	0.15	-4.58	0.93	-3.65	38323.4	TRUE	TRUE	TRUE	TRUE	TRUE	TRUE
1	SFGGVSVIT	577	585	9	HLA-A*02:01	577	38	22588.96	3.80E+01	9.84E-01	-0.2848741	-4.3538962	0.69939086	-3.6545054	0.98	-0.28	-4.35	0.7	-3.65	22589	TRUE	TRUE	TRUE	TRUE	TRUE	TRUE
1	SEPVLKGVK	1243	1251	9	HLA-A*02:01	1243	93	41635.02	9.30E+01	8.61E-01	0.09973334	-4.6194588	0.96102434	-3.6584344	0.86	0.1	-4.62	0.96	-3.66	41635	TRUE	TRUE	TRUE	TRUE	TRUE	TRUE
1	INARDLICA	826	834	9	HLA-A*02:01	826	43	24958.64	4.30E+01	1.08E+00	-0.340525	-4.3972209	0.737537015	-3.6596839	1.08	-0.34	-4.4	0.74	-3.66	24958.6	TRUE	TRUE	TRUE	TRUE	TRUE	TRUE
1	LGDISGINA	1148	1156	9	HLA-A*02:01	1148	46	26533.44	4.60E+01	1.24E+00	-0.4818964	-4.4237936	0.76174864	-3.6620449	1.24	-0.48	-4.42	0.76	-3.66	26533.4	TRUE	TRUE	TRUE	TRUE	TRUE	TRUE
1	TECANLLLQ	729	737	9	HLA-A*02:01	729	51	28365.83	5.10E+01	8.79E-01	-0.0883142	-4.4527955	0.79034681	-3.6624487	0.88	-0.09	-4.45	0.79	-3.66	28365.8	TRUE	TRUE	TRUE	TRUE	TRUE	TRUE
1	QDIWGTSAA	242	250	9	HLA-A*02:01	242	65	33298.16	6.50E+01	1.12E+00	-0.2623429	-4.5224202	0.859452063	-3.6629682	1.12	-0.26	-4.52	0.86	-3.66	33298.2	TRUE	TRUE	TRUE	TRUE	TRUE	TRUE
1	MGCVLAWNT	417	425	9	HLA-A*02:01	417	30	17484.31	3.00E+01	1.02E+00	-0.4440186	-4.2426485	0.579324353	-3.6633241	1.02	-0.44	-4.24	0.58	-3.66	17484.3	TRUE	TRUE	TRUE	TRUE	TRUE	TRUE
1	INASVVNIQ	1154	1162	9	HLA-A*02:01	1154	60	31603.04	6.00E+01	9.44E-01	-0.1090673	-4.4997289	0.835187734	-3.6645411	0.94	-0.11	-4.5	0.84	-3.66	31603	TRUE	TRUE	TRUE	TRUE	TRUE	TRUE
1	WERKKISNC	340	348	9	HLA-A*02:01	340	84	38679.21	8.40E+01	8.99E-01	0.022727308	-4.5874776	0.92217231	-3.6653053	0.9	0.02	-4.59	0.92	-3.67	38679.2	TRUE	TRUE	TRUE	TRUE	TRUE	TRUE
1	NTVYDPLQP	1117	1125	9	HLA-A*02:01	1117	72	35261.55	7.20E+01	7.33E-01	0.14608305	-4.5473014	0.879417046	-3.6678844	0.73	0.15	-4.55	0.88	-3.67	35261.6	TRUE	TRUE	TRUE	TRUE	TRUE	TRUE
1	YSNNTIAIP	689	697	9	HLA-A*02:01	689	41	23733.48	4.10E+01	6.24E-01	0.07902028	-4.3753614	0.70302828	-3.6723331	0.62	0.08	-4.38	0.7	-3.67	23733.5	TRUE	TRUE	TRUE	TRUE	TRUE	TRUE
1	KYRYLRHGK	439	447	9	HLA-A*02:01	439	89	40214.24	8.90E+01	5.69E-01	0.362321926	-4.6043799	0.931477926	-3.6729019	0.57	0.36	-4.6	0.93	-3.67	40214.2	TRUE	TRUE	TRUE	TRUE	TRUE	TRUE
1	VNCTDVSTA	601	609	9	HLA-A*02:01	601	61	32123.62	6.10E+01	1.16E+00	-0.3269639	-4.5068245	0.83021208	-3.6766124	1.16	-0.33	-4.51	0.83	-3.68	32123.6	TRUE	TRUE	TRUE	TRUE	TRUE	TRUE
1	VTGFHTINH	66	74	9	HLA-A*02:01	66	65	33299.62	6.50E+01	1.07E+00	-0.2231609	-4.5224393	0.84549806	-3.6769412	1.07	-0.22	-4.52	0.85	-3.68	33299.6	TRUE	TRUE	TRUE	TRUE	TRUE	TRUE
1	PCTPPALNC	466	474	9	HLA-A*02:01	466	87	39667.99	8.70E+01	1.09E+00	-0.1663674	-4.5984402	0.920910615	-3.6775296	1.09	-0.17	-4.6	0.92	-3.68	39668	TRUE	TRUE	TRUE	TRUE	TRUE	TRUE
1	QERNFTTAP	1053	1061	9	HLA-A*02:01	1053	83	38424.34	8.30E+01	8.72E-01	0.03498309	-4.5846064	0.90707009	-3.6775363	0.87	0.03	-4.58	0.91	-3.68	38424.3	TRUE	TRUE	TRUE	TRUE	TRUE	TRUE
1	NVPFSPDGK	457	465	9	HLA-A*02:01	457	87	39529.6	8.70E+01	6.21E-01	0.29598545	-4.5969224	0.917302451	-3.67962	0.62	0.3	-4.6	0.92	-3.68	39529.6	TRUE	TRUE	TRUE	TRUE	TRUE	TRUE
1	DRCTTFDDV	17	25	9	HLA-A*02:01	17	63	32729.51	6.30E+01	7.21E-01	0.112214747	-4.5149395	0.832766747	-3.6821728	0.72	0.11	-4.51	0.83	-3.68	32729.5	TRUE	TRUE	TRUE	TRUE	TRUE	TRUE
1	DVNCTDVST	600	608	9	HLA-A*02:01	600	67	34025.48	6.70E+01	1.29E+00	-0.4460944	-4.5318043	0.848460612	-3.6833436	1.29	-0.45	-4.53	0.85	-3.68	34025.5	TRUE	TRUE	TRUE	TRUE	TRUE	TRUE
1	AALVSGTAT	857	865	9	HLA-A*02:01	857	51	28262.9	5.10E+01	9.74E-01	-0.2093234	-4.4512167	0.764494614	-3.6867221	0.97	-0.21	-4.45	0.76	-3.69	28262.9	TRUE	TRUE	TRUE	TRUE	TRUE	TRUE
1	NYKYRYLRH	437	445	9	HLA-A*02:01	437	84	38624.84	8.40E+01	1.02E+00	-0.1236302	-4.5868667	0.899885761	-3.6869809	1.02	-0.12	-4.59	0.9	-3.69	38624.8	TRUE	TRUE	TRUE	TRUE	TRUE	TRUE
1	SKRFQPFQQ	542	550	9	HLA-A*02:01	542	80	37749.77	8.00E+01	8.48E-01	0.04143123	-4.5769143	0.88972423	-3.6871901	0.85	0.04	-4.58	0.89	-3.69	37749.8	TRUE	TRUE	TRUE	TRUE	TRUE	TRUE
1	IRASANLAA	1000	1008	9	HLA-A*02:01	1000	48	27393.42	4.80E+01	9.47E-01	-0.1973766	-4.4376463	0.749973447	-3.6876728	0.95	-0.2	-4.44	0.75	-3.69	27393.4	TRUE	TRUE	TRUE	TRUE	TRUE	TRUE
1	SSKRFQPFQ	541	549	9	HLA-A*02:01	541	88	39802.98	8.80E+01	9.79E-01	-0.0709561	-4.5999156	0.908360925	-3.6915547	0.98	-0.07	-4.6	0.91	-3.69	39803	TRUE	TRUE	TRUE	TRUE	TRUE	TRUE
1	AGCLIGAEH	633	641	9	HLA-A*02:01	633	56	30100.86	5.60E+01	1.09E+00	-0.3021099	-4.4785789	0.785486054	-3.6930929	1.09	-0.3	-4.48	0.79	-3.69	30100.9	TRUE	TRUE	TRUE	TRUE	TRUE	TRUE
1	DDFMGCVLA	414	422	9	HLA-A*02:01	414	33	19239.01	3.30E+01	1.11E+00	-0.5166185	-4.2841827	0.590532517	-3.6936502	1.11	-0.52	-4.28	0.59	-3.69	19239	TRUE	TRUE	TRUE	TRUE	TRUE	TRUE
1	FITQRNFFS	1085	1093	9	HLA-A*02:01	1085	8.7	3306.75	8.70E+00	8.27E-01	-1.00171	-3.5194014	-0.174356	-3.6937574	0.83	-1	-3.52	-0.17	-3.69	3306.8	TRUE	TRUE	TRUE	TRUE	TRUE	TRUE
1	NVLYENQKQ	896	904	9	HLA-A*02:01	896	94	41995.6	9.40E+01	9.12E-01	0.016012257	-4.6232038	0.928124257	-3.6950795	0.91	0.02	-4.62	0.93	-3.7	41995.6	TRUE	TRUE	TRUE	TRUE	TRUE	TRUE
1	LNRALSGIA	745	753	9	HLA-A*02:01	745	67	33798.71	6.70E+01	1.13E+00	-0.2956955	-4.5289001	0.832851543	-3.6960486	1.13	-0.3	-4.53	0.83	-3.7	33798.7	TRUE	TRUE	TRUE	TRUE	TRUE	TRUE
1	LHVTYVPSQ	1045	1053	9	HLA-A*02:01	1045	77	36891.72	7.70E+01	9.23E-01	-0.0539924	-4.5669289	0.86851858	-3.6984103	0.92	-0.05	-4.57	0.87	-3.7	36891.7	TRUE	TRUE	TRUE	TRUE	TRUE	TRUE
1	VRGWVFGST	98	106	9	HLA-A*02:01	98	53	29024.65	5.30E+01	1.00E+00	-0.2408488	-4.462767	0.76022818	-3.7025388	1	-0.24	-4.46	0.76	-3.7	29024.7	TRUE	TRUE	TRUE	TRUE	TRUE	TRUE
1	LPSGFNTLK	209	217	9	HLA-A*02:01	209	71	35029.59	7.10E+01	8.30E-01	0.010240289	-4.5444351	0.840459289	-3.7039758	0.83	0.01	-4.54	0.84	-3.7	35029.6	TRUE	TRUE	TRUE	TRUE	TRUE	TRUE
1	GSDLDRCTT	13	21	9	HLA-A*02:01	13	69	34475.35	6.90E+01	1.25E+00	-0.4205466	-4.5375087	0.83281938	-3.7046893	1.25	-0.42	-4.54	0.83	-3.7	34475.3	TRUE	TRUE	TRUE	TRUE	TRUE	TRUE
1	THTMIFDNA	148	156	9	HLA-A*02:01	148	53	29252.59	5.30E+01	1.03E+00	-0.274737	-4.4661643	0.758490009	-3.7076743	1.03	-0.27	-4.47	0.76	-3.71	29252.6	TRUE	TRUE	TRUE	TRUE	TRUE	TRUE
1	KGDDVRQIA	390	398	9	HLA-A*02:01	390	60	31581.51	6.00E+01	1.15E+00	-0.3569824	-4.4994329	0.79128162	-3.7081513	1.15	-0.36	-4.5	0.79	-3.71	31581.5	TRUE	TRUE	TRUE	TRUE	TRUE	TRUE
1	TVCGPKLST	509	517	9	HLA-A*02:01	509	51	28543.17	5.10E+01	9.31E-01	-0.1853706	-4.4555022	0.74605945	-3.7094428	0.93	-0.19	-4.46	0.75	-3.71	28543.2	TRUE	TRUE	TRUE	TRUE	TRUE	TRUE
1	GYHLMSFPQ	1028	1036	9	HLA-A*02:01	1028	46	26455.47	4.60E+01	6.65E-01	0.046347366	-4.4225155	0.711026366	-3.7114891	0.66	0.05	-4.42	0.71	-3.71	26455.5	TRUE	TRUE	TRUE	TRUE	TRUE	TRUE
1	TNFSISITT	698	706	9	HLA-A*02:01	698	35	20403.19	3.50E+01	9.93E-01	-0.3965976	-4.3096981	0.596846407	-3.7128517	0.99	-0.4	-4.31	0.6	-3.71	20403.2	TRUE	TRUE	TRUE	TRUE	TRUE	TRUE

seq #	peptide	start	end	peptide length	allele	peptide index	median binding	ann IC50	ann percentile	proteasome sc	tap score	mhc score	processing sco	processing tota	legacy Proteas	legacy TAP Sco	legacy MHC Sc	legacy Process	legacy Total Sc	legacy MHC IC5	equal Proteaso	equal TAP Scor	equal MHC Sco	equal Processi	equal Total Sc	equal MHC IC50
1	CHEGKAYFP	1064	1072	9	HLA-A*02:01	1064	78	36956.85	7.80E+01	8.83E-01	-0.0297222	-4.5676949	0.85317179	-3.7145232	0.88	-0.03	-4.57	0.85	-3.71	36956.8	TRUE	TRUE	TRUE	TRUE	TRUE	TRUE
1	KWPWYVWLG	1193	1201	9	HLA-A*02:01	1193	27	15755.43	2.70E+01	9.37E-01	-0.4546304	-4.1974303	0.482123598	-3.7153067	0.94	-0.45	-4.2	0.48	-3.72	15755.4	TRUE	TRUE	TRUE	TRUE	TRUE	TRUE
1	PGTNASSEV	586	594	9	HLA-A*02:01	586	94	41899.83	9.40E+01	1.11E+00	-0.2006747	-4.6222123	0.906281318	-3.7159309	1.11	-0.2	-4.62	0.91	-3.72	41899.8	TRUE	TRUE	TRUE	TRUE	TRUE	TRUE
1	SFELLNAPA	500	508	9	HLA-A*02:01	500	29	17238.6	2.90E+01	6.98E-01	-0.1773317	-4.236502	0.52032134	-3.7161807	0.7	-0.18	-4.24	0.52	-3.72	17238.6	TRUE	TRUE	TRUE	TRUE	TRUE	TRUE
1	IFDNAFNCT	152	160	9	HLA-A*02:01	152	47	26625.76	4.70E+01	9.93E-01	-0.2846688	-4.425302	0.708577209	-3.7167248	0.99	-0.28	-4.43	0.71	-3.72	26625.8	TRUE	TRUE	TRUE	TRUE	TRUE	TRUE
1	ALNCYWPLN	471	479	9	HLA-A*02:01	471	18	9365.42	1.80E+01	8.56E-01	-0.601575	-3.9715273	0.254584029	-3.7169432	0.86	-0.6	-3.97	0.25	-3.72	9365.4	TRUE	TRUE	TRUE	TRUE	TRUE	TRUE
1	DVQAPNYTQ	24	32	9	HLA-A*02:01	24	88	40000.72	8.80E+01	1.08E+00	-0.1986155	-4.6020678	0.884962473	-3.7171053	1.08	-0.2	-4.6	0.88	-3.72	40000.7	TRUE	TRUE	TRUE	TRUE	TRUE	TRUE
1	DRLITGRLQ	976	984	9	HLA-A*02:01	976	80	37593.65	8.00E+01	9.38E-01	-0.0829416	-4.5751145	0.854979399	-3.7201351	0.94	-0.08	-4.58	0.85	-3.72	37593.7	TRUE	TRUE	TRUE	TRUE	TRUE	TRUE
1	KPMGTQTHT	142	150	9	HLA-A*02:01	142	69	34350.25	6.90E+01	1.16E+00	-0.3413571	-4.5359299	0.815528932	-3.720401	1.16	-0.34	-4.54	0.82	-3.72	34350.2	TRUE	TRUE	TRUE	TRUE	TRUE	TRUE
1	AWNTRNIDA	422	430	9	HLA-A*02:01	422	65	33381.5	6.50E+01	8.83E-01	-0.0816679	-4.5235058	0.801681067	-3.7218248	0.88	-0.08	-4.52	0.8	-3.72	33381.5	TRUE	TRUE	TRUE	TRUE	TRUE	TRUE
1	ITQRNFFSP	1086	1094	9	HLA-A*02:01	1086	45	25732.19	4.50E+01	7.02E-01	-0.0150579	-4.4104767	0.686804135	-3.7236726	0.7	-0.02	-4.41	0.69	-3.72	25732.2	TRUE	TRUE	TRUE	TRUE	TRUE	TRUE
1	TGFHTINHT	67	75	9	HLA-A*02:01	67	49	27520.56	4.90E+01	1.08E+00	-0.3631052	-4.4396573	0.714121836	-3.7255354	1.08	-0.36	-4.44	0.71	-3.73	27520.6	TRUE	TRUE	TRUE	TRUE	TRUE	TRUE
1	LGKYEQYIK	1185	1193	9	HLA-A*02:01	1185	95	42340.53	9.50E+01	8.83E-01	0.015000083	-4.6267563	0.898276083	-3.7284802	0.88	0.02	-4.63	0.9	-3.73	42340.5	TRUE	TRUE	TRUE	TRUE	TRUE	TRUE
1	RFQPFQQFG	544	552	9	HLA-A*02:01	544	64	32959.44	6.40E+01	1.18E+00	-0.3965587	-4.5179798	0.78431129	-3.7336685	1.18	-0.4	-4.52	0.78	-3.73	32959.4	TRUE	TRUE	TRUE	TRUE	TRUE	TRUE
1	GFMKQYGEC	814	822	9	HLA-A*02:01	814	87	39736.73	8.70E+01	7.69E-01	0.09556833	-4.5991921	0.864928332	-3.7342638	0.77	0.1	-4.6	0.86	-3.73	39736.7	TRUE	TRUE	TRUE	TRUE	TRUE	TRUE
1	DLFLPFYSN	57	65	9	HLA-A*02:01	57	27	15671.27	2.70E+01	1.12E+00	-0.6704141	-4.1951042	0.452484887	-3.7426193	1.12	-0.67	-4.2	0.45	-3.74	15671.3	TRUE	TRUE	TRUE	TRUE	TRUE	TRUE
1	HGVVFLHVT	1040	1048	9	HLA-A*02:01	1040	31	18214.8	3.10E+01	1.01E+00	-0.4917872	-4.2604244	0.515997762	-3.7444266	1.01	-0.49	-4.26	0.52	-3.74	18214.8	TRUE	TRUE	TRUE	TRUE	TRUE	TRUE
1	QILPDPLKP	786	794	9	HLA-A*02:01	786	52	28770.45	5.20E+01	5.78E-01	0.13653512	-4.4589467	0.714484119	-3.7444625	0.58	0.14	-4.46	0.71	-3.74	28770.5	TRUE	TRUE	TRUE	TRUE	TRUE	TRUE
1	FSPDGKPCT	460	468	9	HLA-A*02:01	460	36	21197.76	3.60E+01	9.80E-01	-0.3998884	-4.32629	0.580139563	-3.7461504	0.98	-0.4	-4.33	0.58	-3.75	21197.8	TRUE	TRUE	TRUE	TRUE	TRUE	TRUE
1	PFERDISNV	450	458	9	HLA-A*02:01	450	78	37047.72	7.80E+01	8.27E-01	-0.005483	-4.5687615	0.821240036	-3.7475214	0.83	-0.01	-4.57	0.82	-3.75	37047.7	TRUE	TRUE	TRUE	TRUE	TRUE	TRUE
1	AMQMAYRFN	881	889	9	HLA-A*02:01	881	45	25625.23	4.50E+01	1.17E+00	-0.5135719	-4.4086678	0.65995708	-3.7487107	1.17	-0.51	-4.41	0.66	-3.75	25625.2	TRUE	TRUE	TRUE	TRUE	TRUE	TRUE
1	DRNTREVFA	757	765	9	HLA-A*02:01	757	87	39509.08	8.70E+01	1.19E+00	-0.3411681	-4.5966969	0.845395926	-3.751301	1.19	-0.34	-4.6	0.85	-3.75	39509.1	TRUE	TRUE	TRUE	TRUE	TRUE	TRUE
1	VVLSFELLN	497	505	9	HLA-A*02:01	497	33	19418.86	3.30E+01	9.88E-01	-0.4539843	-4.2882237	0.533545693	-3.754678	0.99	-0.45	-4.29	0.53	-3.75	19418.9	TRUE	TRUE	TRUE	TRUE	TRUE	TRUE
1	LPPLLTDDM	843	851	9	HLA-A*02:01	843	55	29935.23	5.50E+01	7.05E-01	0.015786547	-4.4761826	0.72036755	-3.7558151	0.7	0.02	-4.48	0.72	-3.76	29935.2	TRUE	TRUE	TRUE	TRUE	TRUE	TRUE
1	IHADQLTPA	610	618	9	HLA-A*02:01	610	35	20551.63	3.50E+01	6.96E-01	-0.1442928	-4.3128463	0.551770159	-3.7610761	0.7	-0.14	-4.31	0.55	-3.76	20551.6	TRUE	TRUE	TRUE	TRUE	TRUE	TRUE
1	IADYNYKLP	405	413	9	HLA-A*02:01	405	77	36641.5	7.70E+01	7.80E-01	0.02057601	-4.5639732	0.800612005	-3.7633612	0.78	0.02	-4.56	0.8	-3.76	36641.5	TRUE	TRUE	TRUE	TRUE	TRUE	TRUE
1	GSTMNNKSQ	104	112	9	HLA-A*02:01	104	98	43840.71	9.80E+01	1.05E+00	-0.168885	-4.6418776	0.877944994	-3.7639326	1.05	-0.17	-4.64	0.88	-3.76	43840.7	TRUE	TRUE	TRUE	TRUE	TRUE	TRUE
1	NVVRGWVFG	96	104	9	HLA-A*02:01	96	46	26163.97	4.60E+01	1.12E+00	-0.4718935	-4.4177036	0.652479543	-3.7652241	1.12	-0.47	-4.42	0.65	-3.77	26164	TRUE	TRUE	TRUE	TRUE	TRUE	TRUE
1	FELLNAPAT	501	509	9	HLA-A*02:01	501	39	22796.44	3.90E+01	9.68E-01	-0.3755618	-4.357867	0.592306168	-3.7655609	0.97	-0.38	-4.36	0.59	-3.77	22796.4	TRUE	TRUE	TRUE	TRUE	TRUE	TRUE
1	PSGDVVRFP	309	317	9	HLA-A*02:01	309	78	37075.39	7.80E+01	9.91E-01	-0.1976364	-4.5690857	0.793406608	-3.7756791	0.99	-0.2	-4.57	0.79	-3.78	37075.4	TRUE	TRUE	TRUE	TRUE	TRUE	TRUE
1	KGYHLMSFP	1027	1035	9	HLA-A*02:01	1027	53	29221.27	5.30E+01	6.46E-01	0.04262958	-4.4656991	0.68860158	-3.7770975	0.65	0.04	-4.47	0.69	-3.78	29221.3	TRUE	TRUE	TRUE	TRUE	TRUE	TRUE
1	VSKPMGTQT	140	148	9	HLA-A*02:01	140	80	37639.65	8.00E+01	9.94E-01	-0.1965525	-4.5756456	0.797508546	-3.778137	0.99	-0.2	-4.58	0.8	-3.78	37639.7	TRUE	TRUE	TRUE	TRUE	TRUE	TRUE
1	FPNITNLCP	316	324	9	HLA-A*02:01	316	53	28975.07	5.30E+01	7.96E-01	-0.1123577	-4.4620245	0.68342535	-3.7785991	0.8	-0.11	-4.46	0.68	-3.78	28975.1	TRUE	TRUE	TRUE	TRUE	TRUE	TRUE
1	NDILSRLDK	960	968	9	HLA-A*02:01	960	82	38099.37	8.20E+01	6.65E-01	0.13630127	-4.5809178	0.80093027	-3.7799875	0.66	0.14	-4.58	0.8	-3.78	38099.4	TRUE	TRUE	TRUE	TRUE	TRUE	TRUE
1	NAPATVCGP	505	513	9	HLA-A*02:01	505	68	34149.03	6.80E+01	6.52E-01	0.09771264	-4.5333784	0.749803637	-3.7835747	0.65	0.1	-4.53	0.75	-3.78	34149	TRUE	TRUE	TRUE	TRUE	TRUE	TRUE
1	QESLTTTST	917	925	9	HLA-A*02:01	917	79	37456.03	7.90E+01	1.15E+00	-0.3581468	-4.5735217	0.789302232	-3.7842195	1.15	-0.36	-4.57	0.79	-3.78	37456	TRUE	TRUE	TRUE	TRUE	TRUE	TRUE
1	SFIEDLLFN	798	806	9	HLA-A*02:01	798	36	20919.11	3.60E+01	9.20E-01	-0.3859537	-4.3205432	0.533739271	-3.7868039	0.92	-0.39	-4.32	0.53	-3.79	20919.1	TRUE	TRUE	TRUE	TRUE	TRUE	TRUE
1	YSNVTGFHT	63	71	9	HLA-A*02:01	63	28	16241.45	2.80E+01	8.07E-01	-0.3843271	-4.2106248	0.422676918	-3.7879479	0.81	-0.38	-4.21	0.42	-3.79	16241.5	TRUE	TRUE	TRUE	TRUE	TRUE	TRUE
1	YADSFVVKG	383	391	9	HLA-A*02:01	383	26	15190.79	2.60E+01	1.04E+00	-0.6472682	-4.1815804	0.39319683	-3.7883835	1.04	-0.65	-4.18	0.39	-3.79	15190.8	TRUE	TRUE	TRUE	TRUE	TRUE	TRUE
1	TSTALGKLQ	923	931	9	HLA-A*02:01	923	84	38823.03	8.40E+01	8.50E-01	-0.0506182	-4.5890894	0.799675816	-3.7894136	0.85	-0.05	-4.59	0.8	-3.79	38823	TRUE	TRUE	TRUE	TRUE	TRUE	TRUE
1	KGACSCGSC	1227	1235	9	HLA-A*02:01	1227	76	36522.36	7.60E+01	8.08E-01	-0.0357341	-4.5625588	0.772643919	-3.7899149	0.81	-0.04	-4.56	0.77	-3.79	36522.4	TRUE	TRUE	TRUE	TRUE	TRUE	TRUE
1	HTVSLLRST	661	669	9	HLA-A*02:01	661	38	22285.99	3.80E+01	9.01E-01	-0.34473	-4.3480319	0.555918987	-3.7921129	0.9	-0.34	-4.35	0.56	-3.79	22286	TRUE	TRUE	TRUE	TRUE	TRUE	TRUE
1	LNESLIDLQ	1175	1183	9	HLA-A*02:01	1175	66	33458.87	6.60E+01	8.43E-01	-0.112385	-4.5245113	0.73044203	-3.7940692	0.84	-0.11	-4.52	0.73	-3.79	33458.9	TRUE	TRUE	TRUE	TRUE	TRUE	TRUE
1	FNKAISQIQ	909	917	9	HLA-A*02:01	909	70	34883.6	7.00E+01	9.23E-01	-0.1763392	-4.5426213	0.746324812	-3.7962965	0.92	-0.18	-4.54	0.75	-3.8	34883.6	TRUE	TRUE	TRUE	TRUE	TRUE	TRUE
1	EIDKGIYQT	294	302	9	HLA-A*02:01	294	35	20874.11	3.50E+01	9.80E-01	-0.4617489	-4.319608	0.51796113	-3.8016468	0.98	-0.46	-4.32	0.52	-3.8	20874.1	TRUE	TRUE	TRUE	TRUE	TRUE	TRUE
1	ENGTITDAV	268	276	9	HLA-A*02:01	268	63	32618.15	6.30E+01	8.62E-01	-0.1504473	-4.5134593	0.71132169	-3.8021376	0.86	-0.15	-4.51	0.71	-3.8	32618.2	TRUE	TRUE	TRUE	TRUE	TRUE	TRUE
1	FKCYGVSAT	364	372	9	HLA-A*02:01	364	44	25388.98	4.40E+01	9.17E-01	-0.3186027	-4.4046453	0.598746279	-3.805899	0.92	-0.32	-4.4	0.6	-3.81	25389	TRUE	TRUE	TRUE	TRUE	TRUE	TRUE
1	TPAWRIYST	616	624	9	HLA-A*02:01	616	51	28460.22	5.10E+01	1.07E+00	-0.4201474	-4.4542383	0.64804964	-3.8061886	1.07	-0.42	-4.45	0.65	-3.81	28460.2	TRUE	TRUE	TRUE	TRUE	TRUE	TRUE
1	GNNVFQTQA	625	633	9	HLA-A*02:01	625	69	34511.19	6.90E+01	1.14E+00	-0.4120028	-4.5379599	0.731754213	-3.8062057	1.14	-0.41	-4.54	0.73	-3.81	34511.2	TRUE	TRUE	TRUE	TRUE	TRUE	TRUE
1	DSFKEELDK	1128	1136	9	HLA-A*02:01	1128	82	38174.06	8.20E+01	6.22E-01	0.152234672	-4.5817684	0.773779672	-3.8079887	0.62	0.15	-4.58	0.77	-3.81	38174.1	TRUE	TRUE	TRUE	TRUE	TRUE	TRUE
1	CNFELCDNP	128	136	9	HLA-A*02:01	128	54	29648.97	5.40E+01	5.84E-01	0.07563192	-4.4720096	0.659677919	-3.8123317	0.58	0.08	-4.47	0.66	-3.81	29649	TRUE	TRUE	TRUE	TRUE	TRUE	TRUE
1	AAYTAALVS	853	861	9	HLA-A*02:01	853	36	21229.66	3.60E+01	1.32E+00	-0.8056965	-4.326943	0.51240551	-3.8145375	1.32	-0.81	-4.33	0.51	-3.81	21229.7	TRUE	TRUE	TRUE	TRUE	TRUE	TRUE
1	TSEILDISP	567	575	9	HLA-A*02:01	567	60	31543.95	6.00E+01	6.51E-01	0.033148652	-4.4989161	0.684243652	-3.8146724	0.65	0.03	-4.5	0.68	-3.81	31544	TRUE	TRUE	TRUE	TRUE	TRUE	TRUE
1	QAPNYTQHT	26	34	9	HLA-A*02:01	26	62	32273.42	6.20E+01	9.52E-01	-0.2646226	-4.508845	0.687184427	-3.8216606	0.95	-0.26	-4.51	0.69	-3.82	32273.4	TRUE	TRUE	TRUE	TRUE	TRUE	TRUE
1	LYNSTFFST	355	363	9	HLA-A*02:01	355	36	21282.56	3.60E+01	7.17E-01	-0.2105231	-4.3280239	0.506156868	-3.821867	0.72	-0.21	-4.33	0.51	-3.82	21282.6	TRUE	TRUE	TRUE	TRUE	TRUE	TRUE
1	KYEQYIKWP	1187	1195	9	HLA-A*02:01	1187	76	36506.96	7.60E+01	6.11E-01	0.1294654	-4.5623757	0.7404424	-3.8219333	0.61	0.13	-4.56	0.74	-3.82	36507	TRUE	TRUE	TRUE	TRUE	TRUE	TRUE
1	TINHTFGNP	71	79	9	HLA-A*02:01	71	71	35173.16	7.10E+01	6.79E-01	0.03960675	-4.5462114	0.71856275	-3.8276486	0.68	0.04	-4.55	0.72	-3.83	35173.2	TRUE	TRUE	TRUE	TRUE	TRUE	TRUE
1	TRNIDATST	425	433	9	HLA-A*02:01	425	88	39930.66	8.80E+01	9.79E-01	-0.2060069	-4.6013065	0.772957069	-3.8283494	0.98	-0.21	-4.6	0.77	-3.83	39930.7	TRUE	TRUE	TRUE	TRUE	TRUE	TRUE
1	LCPFGEVFN	322	330	9	HLA-A*02:01	322	75	36267.2	7.50E+01	1.23E+00	-0.5036998	-4.559514	0.730953185	-3.8285608	1.23	-0.5	-4.56	0.73	-3.83	36267.2	TRUE	TRUE	TRUE	TRUE	TRUE	TRUE
1	VDCSQNPLA	276	284	9	HLA-A*02:01	276	77	36849.44	7.70E+01	1.00E+00	-0.267288	-4.5664309	0.736520968	-3.8299099	1	-0.27	-4.57	0.74	-3.83	36849.4	TRUE	TRUE	TRUE	TRUE	TRUE	TRUE
1	KSQSVIIIN	110	118	9	HLA-A*02:01	110	44	25261.57	4.40E+01	1.03E+00	-0.4582046	-4.4024603	0.570386442	-3.8320739	1.03	-0.46	-4.4	0.57	-3.83	25261.6	TRUE	TRUE	TRUE	TRUE	TRUE	TRUE
1	DVVRFPNIT	312	320	9	HLA-A*02:01	312	71	34972.39	7.10E+01	1.07E+00	-0.3634854	-4.5437253	0.71052662	-3.8331987	1.07	-0.36	-4.54	0.71	-3.83	34972.4	TRUE	TRUE	TRUE	TRUE	TRUE	TRUE
1	AGICASYHT	654	662	9	HLA-A*02:01	654	48	27116.22	4.80E+01	9.40E-01	-0.3430239	-4.4332291	0.5972961	-3.8359331	0.94	-0.34	-4.43	0.6	-3.84	27116.2	TRUE	TRUE	TRUE	TRUE	TRUE	TRUE
1	LTPSSKRFQ	538	546	9	HLA-A*02:01	538	96	42578.95	9.60E+01	8.31E-01	-0.0406155	-4.6291949	0.790582487	-3.8386125	0.83	-0.04	-4.63	0.79	-3.84	42578.9	TRUE	TRUE	TRUE	TRUE	TRUE	TRUE
1	KLSTDLIKN	514	522	9	HLA-A*02:01	514	28	16471.51	2.80E+01	9.57E-01	-0.5791149	-4.2167334	0.377913087	-3.8388203	0.96	-0.58	-4.22	0.38	-3.84	16471.5	TRUE	TRUE	TRUE	TRUE	TRUE	TRUE
1	FERDISNVP	451	459	9	HLA-A*02:01	451	72	35290.17	7.20E+01	8.06E-01	-0.0979207	-4.5476538	0.708208332	-3.8394454	0.81	-0.1	-4.55	0.71	-3.84	35290.2	TRUE	TRUE	TRUE	TRUE	TRUE	TRUE
1	SSIAYSNNT	685	693	9	HLA-A*02:01	685	41	23840.54	4.10E+01	8.28E-01	-0.2969262	-4.3773161	0.53068077	-3.8466353	0.83	-0.3	-4.38	0.53	-3.85	23840.5	TRUE	TRUE	TRUE	TRUE	TRUE	TRUE
1	GWTFGAGAA	867	875	9	HLA-A*02:01	867	71	34945.54	7.10E+01	8.48E-01	-0.1512882	-4.5433918	0.6964118	-3.84698	0.85	-0.15	-4.54	0.7	-3.85	34945.5	TRUE	TRUE	TRUE	TRUE	TRUE	TRUE
1	TMIFDNAFN	150	158	9	HLA-A*02:01	150	32	18736.71	3.20E+01	9.31E-01	-0.5060593	-4.2726933	0.424731653	-3.8479617	0.93	-0.51	-4.27	0.42	-3.85	18736.7	TRUE	TRUE	TRUE	TRUE	TRUE	TRUE
1	TTTGIGYQP	485	493	9	HLA-A*02:01	485	66	33655.68	6.60E+01	6.90E-01	-0.0128626	-4.5270584	0.677484428	-3.8495739	0.69	-0.01	-4.53	0.68	-3.85	33655.7	TRUE	TRUE	TRUE	TRUE	TRUE	TRUE
1	LNDYGFYTT	478	486	9	HLA-A*02:01	478	43	25027.33	4.30E+01	1.06E+00	-0.5103607	-4.3984145	0.548490281	-3.8499242	1.06	-0.51	-4.4	0.55	-3.85	25027.3	TRUE	TRUE	TRUE	TRUE	TRUE	TRUE
1	EVQIDRLIT	972	980	9	HLA-A*02:01	972	68	34180.82	6.80E+01	9.63E-01	-0.2826197	-4.5337825	0.680197273	-3.8535852	0.96	-0.28	-4.53	0.68	-3.85	34180.8	TRUE	TRUE	TRUE	TRUE	TRUE	TRUE
1	RQIAPGQTG	395	403	9	HLA-A*02:01	395	67	33731.12	6.70E+01	1.09E+00	-0.416306	-4.5280308	0.674298011	-3.8537328	1.09	-0.42	-4.53	0.67	-3.85	33731.1	TRUE	TRUE	TRUE	TRUE	TRUE	TRUE
1	VQAPNYTQH	25	33	9	HLA-A*02:01	25	78	37120.35	7.80E+01	9.41E-01	-0.2249356	-4.5696121	0.715795405	-3.8538167	0.94	-0.22	-4.57	0.72	-3.85	37120.3	TRUE	TRUE	TRUE	TRUE	TRUE	TRUE
1	ESLTTTSTA	918	926	9	HLA-A*02:01	918	64	33016.54	6.40E+01	9.80E-01	-0.3161365	-4.5187316	0.663578517	-3.855153	0.98	-0.32	-4.52	0.66	-3.86	33016.5	TRUE	TRUE	TRUE	TRUE	TRUE	TRUE
1	VNFNFNGLT	525	533	9	HLA-A*02:01	525	54	29496.36	5.40E+01	8.93E-01	-0.300307	-4.4697684	0.59317098	-3.8765974	0.89	-0.3	-4.47	0.59	-3.88	29496.4	TRUE	TRUE	TRUE	TRUE	TRUE	TRUE
1	KYFGGFNFS	777	785	9	HLA-A*02:01	777	36	21087.51	3.60E+01	1.19E+00	-0.7456446	-4.3240253	0.445027358	-3.8789979	1.19	-0.75	-4.32	0.45	-3.88	21087.5	TRUE	TRUE	TRUE	TRUE	TRUE	TRUE
1	CDVVIGIIN	1108	1116	9	HLA-A*02:01	1108	49	27453.06	4.90E+01	1.18E+00	-0.6257801	-4.4385908	0.55728887	-3.8813019	1.18	-0.63	-4.44	0.56	-3.88	27453.1	TRUE	TRUE	TRUE	TRUE	TRUE	TRUE
1	DVRQIAPGQ	393	401	9	HLA-A*02:01	393	79	37288.62	7.90E+01	8.54E-01	-0.1634913	-4.5715763	0.69020968	-3.8813666	0.85	-0.16	-4.57	0.69	-3.88	37288.6	TRUE	TRUE	TRUE	TRUE	TRUE	TRUE
1	WNTRNIDAT	423	431	9	HLA-A*02:01	423	89	40249.06	8.90E+01	1.06E+00	-0.3335596	-4.6047557	0.72317842	-3.8815773	1.06	-0.33	-4.6	0.72	-3.88	40249.1	TRUE	TRUE	TRUE	TRUE	TRUE	TRUE
1	IAAEQDRNT	752	760	9	HLA-A*02:01	752	66	33599.64	6.60E+01	9.14E-01	-0.2800567	-4.5263346	0.634132285	-3.8922023	0.91	-0.28	-4.53	0.63	-3.89	33599.6	TRUE	TRUE	TRUE	TRUE	TRUE	TRUE
1	IVAYTMSLG	674	682	9	HLA-A*02:01	674	36	21038.28	3.60E+01	9.34E-01	-0.5047927	-4.3230102	0.429176319	-3.8938339	0.93	-0.5	-4.32	0.43	-3.89	21038.3	TRUE	TRUE	TRUE	TRUE	TRUE	TRUE
1	LIGAEHVDT	636	644	9	HLA-A*02:01	636	45	25695.74	4.50E+01	8.36E-01	-0.3229738	-4.4098611	0.512968245	-3.8968929	0.84	-0.32	-4.41	0.51	-3.9	25695.7	TRUE	TRUE	TRUE	TRUE	TRUE	TRUE
1	EDDSEPVLK	1240	1248	9	HLA-A*02:01	1240	88	39851.25	8.80E+01	8.58E-01	-0.1564383	-4.6004419	0.701457724	-3.8989842	0.86	-0.16	-4.6	0.7	-3.9	39851.2	TRUE	TRUE	TRUE	TRUE	TRUE	TRUE
1	FNKVTLADA	805	813	9	HLA-A*02:01	805	40	23598.53	4.00E+01	7.90E-01	-0.3184014	-4.372885	0.471641644	-3.9012433	0.79	-0.32	-4.37	0.47	-3.9	23598.5	TRUE	TRUE	TRUE	TRUE	TRUE	TRUE
1	DISNVPFSP	454	462	9	HLA-A*02:01	454	52	28664.5	5.20E+01	5.24E-01	0.018015667	-4.4573444	0.542383667	-3.9149607	0.52	0.02	-4.46	0.54	-3.91	28664.5	TRUE	TRUE	TRUE	TRUE	TRUE	TRUE
1	YTTTGIGYQ	484	492	9	HLA-A*02:01	484	47	26932.3	4.70E+01	6.40E-01	-0.1262356	-4.4302734	0.51408636	-3.9161871	0.64	-0.13	-4.43	0.51	-3.92	26932.3	TRUE	TRUE	TRUE	TRUE	TRUE	TRUE
1	DVVNQNAQA	932	940	9	HLA-A*02:01	932	74	36030.58	7.40E+01	9.32E-01	-0.2961767	-4.5566713	0.636318326	-3.9203529	0.93	-0.3	-4.56	0.64	-3.92	36030.6	TRUE	TRUE	TRUE	TRUE	TRUE	TRUE
1	MSFPQAAPH	1032	1040	9	HLA-A*02:01	1032	49	27676.13	4.90E+01	6.43E-01	-0.1240211	-4.4421054	0.51928785	-3.9228175	0.64	-0.12	-4.44	0.52	-3.92	27676.1	TRUE	TRUE	TRUE	TRUE	TRUE	TRUE
1	KYFKNHTSP	1136	1144	9	HLA-A*02:01	1136	92	41172.26	9.20E+01	4.95E-01	0.19362455	-4.6146047	0.689042552	-3.9255622	0.5	0.19	-4.61	0.69	-3.93	41172.3	TRUE	TRUE	TRUE	TRUE	TRUE	TRUE
1	SPAQDIWGT	239	247	9	HLA-A*02:01	239	46	26342.09	4.60E+01	9.49E-01	-0.4544159	-4.4206502	0.494782148	-3.9258681	0.95	-0.45	-4.42	0.49	-3.93	26342.1	TRUE	TRUE	TRUE	TRUE	TRUE	TRUE
1	NQNAQALNT	935	943	9	HLA-A*02:01	935	44	25212.69	4.40E+01	7.52E-01	-0.2784207	-4.4016192	0.473919329	-3.9276999	0.75	-0.28	-4.4	0.47	-3.93	25212.7	TRUE	TRUE	TRUE	TRUE	TRUE	TRUE
1	NQCVNFNFN	522	530	9	HLA-A*02:01	522	52	28739.02	5.20E+01	1.00E+00	-0.4728065	-4.458472	0.52985454	-3.9286174	1	-0.47	-4.46	0.53	-3.93	28739	TRUE	TRUE	TRUE	TRUE	TRUE	TRUE
1	SPDGKPCTP	461	469	9	HLA-A*02:01	461	84	38724.01	8.40E+01	8.46E-01	-0.1871474	-4.5879803	0.6586736	-3.9293067	0.85	-0.19	-4.59	0.66	-3.93	38724	TRUE	TRUE	TRUE	TRUE	TRUE	TRUE
1	PVLKGVKLH	1245	1253	9	HLA-A*02:01	1245	87	39673.57	8.70E+01	1.11E+00	-0.4446497	-4.5985013	0.66699729	-3.931504	1.11	-0.44	-4.6	0.67	-3.93	39673.6	TRUE	TRUE	TRUE	TRUE	TRUE	TRUE
1	KYDENGTIT	265	273	9	HLA-A*02:01	265	79	37386.38	7.90E+01	8.42E-01	-0.201121	-4.5727134	0.640843973	-3.9318694	0.84	-0.2	-4.57	0.64	-3.93	37386.4	TRUE	TRUE	TRUE	TRUE	TRUE	TRUE
1	AYTAALVSG	854	862	9	HLA-A*02:01	854	61	31815.41	6.10E+01	9.43E-01	-0.3764609	-4.5026375	0.566826128	-3.9358114	0.94	-0.38	-4.5	0.57	-3.94	31815.4	TRUE	TRUE	TRUE	TRUE	TRUE	TRUE
1	GFNFSQILP	781	789	9	HLA-A*02:01	781	65	33147.55	6.50E+01	5.98E-01	-0.0148501	-4.5204514	0.583078885	-3.9373725	0.6	-0.01	-4.52	0.58	-3.94	33147.6	TRUE	TRUE	TRUE	TRUE	TRUE	TRUE
1	TAIHADQLT	608	616	9	HLA-A*02:01	608	60	31619.13	6.00E+01	8.02E-01	-0.2400386	-4.4999499	0.56179843	-3.9381515	0.8	-0.24	-4.5	0.56	-3.94	31619.1	TRUE	TRUE	TRUE	TRUE	TRUE	TRUE
1	GAGAALQIP	871	879	9	HLA-A*02:01	871	64	32841.25	6.40E+01	7.05E-01	-0.1273957	-4.5164197	0.57716832	-3.9392514	0.7	-0.13	-4.52	0.58	-3.94	32841.2	TRUE	TRUE	TRUE	TRUE	TRUE	TRUE
1	PAICHEGKA	1061	1069	9	HLA-A*02:01	1061	97	43186.28	9.70E+01	1.02E+00	-0.3201003	-4.6353458	0.695235716	-3.9401101	1.02	-0.32	-4.64	0.7	-3.94	43186.3	TRUE	TRUE	TRUE	TRUE	TRUE	TRUE
1	NNTVYDPLQ	1116	1124	9	HLA-A*02:01	1116	94	42034.7	9.40E+01	7.89E-01	-0.1084378	-4.623608	0.680168168	-3.9434398	0.79	-0.11	-4.62	0.68	-3.94	42034.7	TRUE	TRUE	TRUE	TRUE	TRUE	TRUE
1	TDDMIAAYT	848	856	9	HLA-A*02:01	848	45	25956.16	4.50E+01	9.74E-01	-0.5052543	-4.4142404	0.468737734	-3.9455027	0.97	-0.51	-4.41	0.47	-3.95	25956.2	TRUE	TRUE	TRUE	TRUE	TRUE	TRUE
1	SGSDLDRCT	12	20	9	HLA-A*02:01	12	86	39362.72	8.60E+01	1.05E+00	-0.3976846	-4.5950851	0.648527381	-3.9465577	1.05	-0.4	-4.6	0.65	-3.95	39362.7	TRUE	TRUE	TRUE	TRUE	TRUE	TRUE
1	FNGTSWFIT	1079	1087	9	HLA-A*02:01	1079	36	21236.78	3.60E+01	8.35E-01	-0.4643018	-4.3270887	0.371147242	-3.9559414	0.84	-0.46	-4.33	0.37	-3.96	21236.8	TRUE	TRUE	TRUE	TRUE	TRUE	TRUE
1	RNTREVFAQ	758	766	9	HLA-A*02:01	758	80	37617.25	8.00E+01	7.02E-01	-0.0829134	-4.575387	0.61924765	-3.9561394	0.7	-0.08	-4.58	0.62	-3.96	37617.2	TRUE	TRUE	TRUE	TRUE	TRUE	TRUE
1	ITGRLQSLQ	979	987	9	HLA-A*02:01	979	80	37757.54	8.00E+01	7.16E-01	-0.0963943	-4.5770037	0.61983367	-3.95717	0.72	-0.1	-4.58	0.62	-3.96	37757.5	TRUE	TRUE	TRUE	TRUE	TRUE	TRUE
1	TGRLQSLQT	980	988	9	HLA-A*02:01	980	75	36143.01	7.50E+01	1.01E+00	-0.4070306	-4.5580243	0.600383438	-3.9576409	1.01	-0.41	-4.56	0.6	-3.96	36143	TRUE	TRUE	TRUE	TRUE	TRUE	TRUE
1	DYSVLYNST	351	359	9	HLA-A*02:01	351	78	36987.65	7.80E+01	8.62E-01	-0.2641739	-4.5680567	0.59747812	-3.9705786	0.86	-0.26	-4.57	0.6	-3.97	36987.7	TRUE	TRUE	TRUE	TRUE	TRUE	TRUE
1	VKQLSSNFG	945	953	9	HLA-A*02:01	945	80	37601.39	8.00E+01	1.15E+00	-0.5502141	-4.5752039	0.598919867	-3.976284	1.15	-0.55	-4.58	0.6	-3.98	37601.4	TRUE	TRUE	TRUE	TRUE	TRUE	TRUE
1	VRQIAPGQT	394	402	9	HLA-A*02:01	394	85	39099.54	8.50E+01	8.78E-01	-0.2647599	-4.5921716	0.613059121	-3.9791125	0.88	-0.26	-4.59	0.61	-3.98	39099.5	TRUE	TRUE	TRUE	TRUE	TRUE	TRUE
1	TVLPPLLTD	841	849	9	HLA-A*02:01	841	47	26932.89	4.70E+01	1.16E+00	-0.7091711	-4.430283	0.44754792	-3.982735	1.16	-0.71	-4.43	0.45	-3.98	26932.9	TRUE	TRUE	TRUE	TRUE	TRUE	TRUE
1	VVRGWVFGS	97	105	9	HLA-A*02:01	97	26	14834.27	2.60E+01	9.95E-01	-0.8069233	-4.1712662	0.18844272	-3.9828235	1	-0.81	-4.17	0.19	-3.98	14834.3	TRUE	TRUE	TRUE	TRUE	TRUE	TRUE
1	TYVPSQERN	1048	1056	9	HLA-A*02:01	1048	100	44953.18	1.00E+02	1.08E+00	-0.4097551	-4.6527604	0.66743893	-3.9853215	1.08	-0.41	-4.65	0.67	-3.99	44953.2	TRUE	TRUE	TRUE	TRUE	TRUE	TRUE
1	WVFGSTMNN	101	109	9	HLA-A*02:01	101	39	22782.39	3.90E+01	8.57E-01	-0.4854745	-4.3575993	0.371226451	-3.9863728	0.86	-0.49	-4.36	0.37	-3.99	22782.4	TRUE	TRUE	TRUE	TRUE	TRUE	TRUE
1	PFSPDGKPC	459	467	9	HLA-A*02:01	459	99	44025.61	9.90E+01	7.67E-01	-0.1115786	-4.6437054	0.655330414	-3.988375	0.77	-0.11	-4.64	0.66	-3.99	44025.6	TRUE	TRUE	TRUE	TRUE	TRUE	TRUE
1	DAVDCSQNP	274	282	9	HLA-A*02:01	274	93	41421.14	9.30E+01	6.61E-01	-0.0327061	-4.617222	0.628067866	-3.9891542	0.66	-0.03	-4.62	0.63	-3.99	41421.1	TRUE	TRUE	TRUE	TRUE	TRUE	TRUE
1	ITTEVMPVS	704	712	9	HLA-A*02:01	704	27	15266.09	2.70E+01	1.16E+00	-0.9683309	-4.1837278	0.194278068	-3.9894498	1.16	-0.97	-4.18	0.19	-3.99	15266.1	TRUE	TRUE	TRUE	TRUE	TRUE	TRUE
1	KRVDFCGKG	1020	1028	9	HLA-A*02:01	1020	83	38415.62	8.30E+01	1.01E+00	-0.4125307	-4.5845078	0.594447257	-3.9900606	1.01	-0.41	-4.58	0.59	-3.99	38415.6	TRUE	TRUE	TRUE	TRUE	TRUE	TRUE
1	IINNTVYDP	1114	1122	9	HLA-A*02:01	1114	36	21192.48	3.60E+01	2.80E-01	0.05576082	-4.3261818	0.335422824	-3.990759	0.28	0.06	-4.33	0.34	-3.99	21192.5	TRUE	TRUE	TRUE	TRUE	TRUE	TRUE
1	ISQIQESLT	913	921	9	HLA-A*02:01	913	69	34591.56	6.90E+01	7.73E-01	-0.2267402	-4.5389701	0.546469794	-3.9925004	0.77	-0.23	-4.54	0.55	-3.99	34591.6	TRUE	TRUE	TRUE	TRUE	TRUE	TRUE
1	VPFSPDGKP	458	466	9	HLA-A*02:01	458	95	42477.72	9.50E+01	5.81E-01	0.048953682	-4.6281612	0.629564682	-3.9985965	0.58	0.05	-4.63	0.63	-4	42477.7	TRUE	TRUE	TRUE	TRUE	TRUE	TRUE
1	ILSRLDKVE	962	970	9	HLA-A*02:01	962	64	33069.45	6.40E+01	1.24E+00	-0.7217119	-4.519427	0.52055612	-3.9988709	1.24	-0.72	-4.52	0.52	-4	33069.4	TRUE	TRUE	TRUE	TRUE	TRUE	TRUE
1	LKGVKLHYT	1247	1255	9	HLA-A*02:01	1247	51	28305.75	5.10E+01	7.85E-01	-0.3340108	-4.4518747	0.45119024	-4.0006844	0.79	-0.33	-4.45	0.45	-4	28305.8	TRUE	TRUE	TRUE	TRUE	TRUE	TRUE
1	DNTFVSGNC	1100	1108	9	HLA-A*02:01	1100	91	40989.56	9.10E+01	8.21E-01	-0.2092621	-4.6126733	0.61137588	-4.0012974	0.82	-0.21	-4.61	0.61	-4	40989.6	TRUE	TRUE	TRUE	TRUE	TRUE	TRUE
1	TFGAGAALQ	869	877	9	HLA-A*02:01	869	75	36119.96	7.50E+01	6.34E-01	-0.0784845	-4.5577473	0.55564545	-4.0021018	0.63	-0.08	-4.56	0.56	-4	36120	TRUE	TRUE	TRUE	TRUE	TRUE	TRUE
1	NPFFAVSKP	135	143	9	HLA-A*02:01	135	72	35250.09	7.20E+01	6.23E-01	-0.0833199	-4.5471602	0.539404138	-4.0077561	0.62	-0.08	-4.55	0.54	-4.01	35250.1	TRUE	TRUE	TRUE	TRUE	TRUE	TRUE
1	LAWNTRNID	421	429	9	HLA-A*02:01	421	79	37362.11	7.90E+01	1.23E+00	-0.6691405	-4.5724314	0.56396351	-4.0084679	1.23	-0.67	-4.57	0.56	-4.01	37362.1	TRUE	TRUE	TRUE	TRUE	TRUE	TRUE
1	VADYSVLYN	349	357	9	HLA-A*02:01	349	45	25729.12	4.50E+01	1.00E+00	-0.5989009	-4.4104249	0.400663116	-4.0097618	1	-0.6	-4.41	0.4	-4.01	25729.1	TRUE	TRUE	TRUE	TRUE	TRUE	TRUE
1	PSQERNFTT	1051	1059	9	HLA-A*02:01	1051	80	37494.94	8.00E+01	1.06E+00	-0.4918946	-4.5739727	0.564207371	-4.0097653	1.06	-0.49	-4.57	0.56	-4.01	37494.9	TRUE	TRUE	TRUE	TRUE	TRUE	TRUE
1	VAVLYQDVN	594	602	9	HLA-A*02:01	594	84	38756.29	8.40E+01	1.14E+00	-0.5585061	-4.5883422	0.578151876	-4.0101903	1.14	-0.56	-4.59	0.58	-4.01	38756.3	TRUE	TRUE	TRUE	TRUE	TRUE	TRUE
1	EDLLFNKVT	801	809	9	HLA-A*02:01	801	81	37820.48	8.10E+01	1.08E+00	-0.5173294	-4.577727	0.56700057	-4.0107265	1.08	-0.52	-4.58	0.57	-4.01	37820.5	TRUE	TRUE	TRUE	TRUE	TRUE	TRUE
1	ELDKYFKNH	1133	1141	9	HLA-A*02:01	1133	64	32887.84	6.40E+01	9.66E-01	-0.4605013	-4.5170354	0.50580265	-4.0112327	0.97	-0.46	-4.52	0.51	-4.01	32887.8	TRUE	TRUE	TRUE	TRUE	TRUE	TRUE
1	RACNFELCD	126	134	9	HLA-A*02:01	126	64	32881.07	6.40E+01	1.15E+00	-0.652104	-4.5169459	0.49494804	-4.0219979	1.15	-0.65	-4.52	0.49	-4.02	32881.1	TRUE	TRUE	TRUE	TRUE	TRUE	TRUE
1	TQTHTMIFD	146	154	9	HLA-A*02:01	146	56	30327.42	5.60E+01	1.25E+00	-0.7943444	-4.4818355	0.459132572	-4.0227029	1.25	-0.79	-4.48	0.46	-4.02	30327.4	TRUE	TRUE	TRUE	TRUE	TRUE	TRUE
1	TQRNFFSPQ	1087	1095	9	HLA-A*02:01	1087	54	29378.83	5.40E+01	4.43E-01	0.00117372	-4.4680345	0.44457972	-4.0234548	0.44	0	-4.47	0.44	-4.02	29378.8	TRUE	TRUE	TRUE	TRUE	TRUE	TRUE
1	EGVFVFNGT	1074	1082	9	HLA-A*02:01	1074	49	27694.7	4.90E+01	8.37E-01	-0.4203769	-4.4423967	0.416674071	-4.0257226	0.84	-0.42	-4.44	0.42	-4.03	27694.7	TRUE	TRUE	TRUE	TRUE	TRUE	TRUE
1	FGRDVSDFT	551	559	9	HLA-A*02:01	551	45	25822.56	4.50E+01	8.49E-01	-0.4639462	-4.4119993	0.385314815	-4.0266845	0.85	-0.46	-4.41	0.39	-4.03	25822.6	TRUE	TRUE	TRUE	TRUE	TRUE	TRUE
1	LGKLQDVVN	927	935	9	HLA-A*02:01	927	89	40330.12	8.90E+01	1.19E+00	-0.6171255	-4.6056295	0.576923527	-4.028706	1.19	-0.62	-4.61	0.58	-4.03	40330.1	TRUE	TRUE	TRUE	TRUE	TRUE	TRUE
1	RALSGIAAE	747	755	9	HLA-A*02:01	747	52	28907.43	5.20E+01	9.10E-01	-0.4798796	-4.4610095	0.42991736	-4.0310921	0.91	-0.48	-4.46	0.43	-4.03	28907.4	TRUE	TRUE	TRUE	TRUE	TRUE	TRUE
1	FSLDVSEKS	168	176	9	HLA-A*02:01	168	27	15627.92	2.70E+01	1.09E+00	-0.9251382	-4.1939012	0.162801823	-4.0310994	1.09	-0.93	-4.19	0.16	-4.03	15627.9	TRUE	TRUE	TRUE	TRUE	TRUE	TRUE
1	MAKTSVDCN	713	721	9	HLA-A*02:01	713	89	40194.22	8.90E+01	1.08E+00	-0.503661	-4.6041636	0.573002969	-4.0311606	1.08	-0.5	-4.6	0.57	-4.03	40194.2	TRUE	TRUE	TRUE	TRUE	TRUE	TRUE
1	FGAISSVLN	952	960	9	HLA-A*02:01	952	54	29448.2	5.40E+01	1.09E+00	-0.6573548	-4.4690588	0.43751616	-4.0315426	1.09	-0.66	-4.47	0.44	-4.03	29448.2	TRUE	TRUE	TRUE	TRUE	TRUE	TRUE
1	FTDSVRDPK	558	566	9	HLA-A*02:01	558	49	27644.12	4.90E+01	3.88E-01	0.015580399	-4.4416028	0.403135399	-4.0384674	0.39	0.02	-4.44	0.4	-4.04	27644.1	TRUE	TRUE	TRUE	TRUE	TRUE	TRUE
1	YKYRYLRHG	438	446	9	HLA-A*02:01	438	60	31705.46	6.00E+01	9.10E-01	-0.4499358	-4.5011341	0.459837189	-4.0412969	0.91	-0.45	-4.5	0.46	-4.04	31705.5	TRUE	TRUE	TRUE	TRUE	TRUE	TRUE
1	VIIINNSTN	114	122	9	HLA-A*02:01	114	66	33642.57	6.60E+01	9.19E-01	-0.4331756	-4.5268892	0.485568381	-4.0413208	0.92	-0.43	-4.53	0.49	-4.04	33642.6	TRUE	TRUE	TRUE	TRUE	TRUE	TRUE
1	KFDEDDSEP	1237	1245	9	HLA-A*02:01	1237	84	38805.8	8.40E+01	4.57E-01	0.08390465	-4.5888966	0.540440649	-4.048456	0.46	0.08	-4.59	0.54	-4.05	38805.8	TRUE	TRUE	TRUE	TRUE	TRUE	TRUE
1	DENGTITDA	267	275	9	HLA-A*02:01	267	79	37248.7	7.90E+01	1.01E+00	-0.4908214	-4.5711111	0.51830164	-4.0528095	1.01	-0.49	-4.57	0.52	-4.05	37248.7	TRUE	TRUE	TRUE	TRUE	TRUE	TRUE
1	DLICAQKFN	830	838	9	HLA-A*02:01	830	73	35556.93	7.30E+01	1.11E+00	-0.6102892	-4.5509243	0.496600803	-4.0543235	1.11	-0.61	-4.55	0.5	-4.05	35556.9	TRUE	TRUE	TRUE	TRUE	TRUE	TRUE
1	SYECDIPIG	645	653	9	HLA-A*02:01	645	79	37442.64	7.90E+01	1.04E+00	-0.5262194	-4.5733665	0.51750563	-4.0558608	1.04	-0.53	-4.57	0.52	-4.06	37442.6	TRUE	TRUE	TRUE	TRUE	TRUE	TRUE
1	TFMLKYDEN	261	269	9	HLA-A*02:01	261	88	40041.86	8.80E+01	1.02E+00	-0.4734694	-4.6025142	0.546213629	-4.0563006	1.02	-0.47	-4.6	0.55	-4.06	40041.9	TRUE	TRUE	TRUE	TRUE	TRUE	TRUE
1	NVTGFHTIN	65	73	9	HLA-A*02:01	65	85	39132.14	8.50E+01	1.02E+00	-0.4851194	-4.5925336	0.534177582	-4.058356	1.02	-0.49	-4.59	0.53	-4.06	39132.1	TRUE	TRUE	TRUE	TRUE	TRUE	TRUE
1	CVLAWNTRN	419	427	9	HLA-A*02:01	419	65	33208.94	6.50E+01	9.93E-01	-0.5323286	-4.521255	0.461127406	-4.0601276	0.99	-0.53	-4.52	0.46	-4.06	33208.9	TRUE	TRUE	TRUE	TRUE	TRUE	TRUE
1	APATVCGPK	506	514	9	HLA-A*02:01	506	66	33500.89	6.60E+01	2.61E-01	0.20184985	-4.5250563	0.462834854	-4.0622215	0.26	0.2	-4.53	0.46	-4.06	33500.9	TRUE	TRUE	TRUE	TRUE	TRUE	TRUE
1	ENQKQIANQ	900	908	9	HLA-A*02:01	900	92	41078.38	9.20E+01	7.87E-01	-0.2384096	-4.6136133	0.548792359	-4.0648209	0.79	-0.24	-4.61	0.55	-4.06	41078.4	TRUE	TRUE	TRUE	TRUE	TRUE	TRUE
1	RHGKLRPFE	444	452	9	HLA-A*02:01	444	95	42239.87	9.50E+01	1.18E+00	-0.6188414	-4.6257226	0.560719579	-4.065003	1.18	-0.62	-4.63	0.56	-4.07	42239.9	TRUE	TRUE	TRUE	TRUE	TRUE	TRUE
1	DVVIGIINN	1109	1117	9	HLA-A*02:01	1109	55	29811.1	5.50E+01	1.02E+00	-0.6157889	-4.474378	0.406939145	-4.0674389	1.02	-0.62	-4.47	0.41	-4.07	29811.1	TRUE	TRUE	TRUE	TRUE	TRUE	TRUE
1	VSVITPGTN	581	589	9	HLA-A*02:01	581	78	36972.44	7.80E+01	1.00E+00	-0.5043449	-4.5678781	0.49937508	-4.068503	1	-0.5	-4.57	0.5	-4.07	36972.4	TRUE	TRUE	TRUE	TRUE	TRUE	TRUE
1	KTPTLKYFG	772	780	9	HLA-A*02:01	772	58	30942.92	5.80E+01	1.02E+00	-0.5972561	-4.4905613	0.420406933	-4.0701544	1.02	-0.6	-4.49	0.42	-4.07	30942.9	TRUE	TRUE	TRUE	TRUE	TRUE	TRUE
1	QKQIANQFN	902	910	9	HLA-A*02:01	902	95	42122.57	9.50E+01	1.05E+00	-0.4949585	-4.6245149	0.553761487	-4.0707534	1.05	-0.49	-4.62	0.55	-4.07	42122.6	TRUE	TRUE	TRUE	TRUE	TRUE	TRUE
1	DFTDSVRDP	557	565	9	HLA-A*02:01	557	97	43054.72	9.70E+01	5.76E-01	-0.0126723	-4.6340208	0.563007714	-4.0710131	0.58	-0.01	-4.63	0.56	-4.07	43054.7	TRUE	TRUE	TRUE	TRUE	TRUE	TRUE
1	KQYGECLGD	817	825	9	HLA-A*02:01	817	43	24814.85	4.30E+01	9.36E-01	-0.6129365	-4.3947117	0.323512544	-4.0711991	0.94	-0.61	-4.39	0.32	-4.07	24814.8	TRUE	TRUE	TRUE	TRUE	TRUE	TRUE
1	QQFGRDVSD	549	557	9	HLA-A*02:01	549	68	34129.45	6.80E+01	1.20E+00	-0.7474369	-4.5331293	0.455547098	-4.0775822	1.2	-0.75	-4.53	0.46	-4.08	34129.4	TRUE	TRUE	TRUE	TRUE	TRUE	TRUE
1	RNIDATSTG	426	434	9	HLA-A*02:01	426	93	41642.68	9.30E+01	1.07E+00	-0.5310312	-4.6195387	0.541207829	-4.0783308	1.07	-0.53	-4.62	0.54	-4.08	41642.7	TRUE	TRUE	TRUE	TRUE	TRUE	TRUE
1	FSPQIITTD	1092	1100	9	HLA-A*02:01	1092	45	25962.63	4.50E+01	1.20E+00	-0.864213	-4.4143487	0.334640025	-4.0797087	1.2	-0.86	-4.41	0.33	-4.08	25962.6	TRUE	TRUE	TRUE	TRUE	TRUE	TRUE
1	YRVVVLSFE	494	502	9	HLA-A*02:01	494	31	18002.03	3.10E+01	8.02E-01	-0.6266246	-4.2553215	0.175318433	-4.080003	0.8	-0.63	-4.26	0.18	-4.08	18002	TRUE	TRUE	TRUE	TRUE	TRUE	TRUE
1	LVSGTATAG	859	867	9	HLA-A*02:01	859	56	30236.33	5.60E+01	8.77E-01	-0.4781836	-4.4805291	0.399097359	-4.0814317	0.88	-0.48	-4.48	0.4	-4.08	30236.3	TRUE	TRUE	TRUE	TRUE	TRUE	TRUE
1	NITNLCPFG	318	326	9	HLA-A*02:01	318	35	20657.74	3.50E+01	8.46E-01	-0.6120221	-4.3150828	0.233566943	-4.0815159	0.85	-0.61	-4.32	0.23	-4.08	20657.7	TRUE	TRUE	TRUE	TRUE	TRUE	TRUE
1	FNGLTVLPP	837	845	9	HLA-A*02:01	837	32	18547.33	3.20E+01	2.72E-01	-0.0899303	-4.2682814	0.182435681	-4.0858457	0.27	-0.09	-4.27	0.18	-4.09	18547.3	TRUE	TRUE	TRUE	TRUE	TRUE	TRUE
1	GAGICASYH	653	661	9	HLA-A*02:01	653	58	30921.84	5.80E+01	7.54E-01	-0.3533769	-4.4902653	0.401068105	-4.0891972	0.75	-0.35	-4.49	0.4	-4.09	30921.8	TRUE	TRUE	TRUE	TRUE	TRUE	TRUE
1	YKLPDDFMG	410	418	9	HLA-A*02:01	410	52	28812.51	5.20E+01	8.93E-01	-0.5232651	-4.4595811	0.369692872	-4.0898882	0.89	-0.52	-4.46	0.37	-4.09	28812.5	TRUE	TRUE	TRUE	TRUE	TRUE	TRUE
1	MYICGDSTE	722	730	9	HLA-A*02:01	722	89	40210.32	8.90E+01	9.65E-01	-0.4513505	-4.6043375	0.51389652	-4.090441	0.97	-0.45	-4.6	0.51	-4.09	40210.3	TRUE	TRUE	TRUE	TRUE	TRUE	TRUE
1	TSWFITQRN	1082	1090	9	HLA-A*02:01	1082	57	30457.96	5.70E+01	8.90E-01	-0.4996512	-4.4837008	0.390159825	-4.093541	0.89	-0.5	-4.48	0.39	-4.09	30458	TRUE	TRUE	TRUE	TRUE	TRUE	TRUE
1	IKNQCVNFN	520	528	9	HLA-A*02:01	520	68	34145.33	6.80E+01	9.92E-01	-0.5578221	-4.5333313	0.434319932	-4.0990114	0.99	-0.56	-4.53	0.43	-4.1	34145.3	TRUE	TRUE	TRUE	TRUE	TRUE	TRUE
1	LPLGINITN	222	230	9	HLA-A*02:01	222	59	31351.71	5.90E+01	1.04E+00	-0.6477326	-4.4962612	0.394173416	-4.1020878	1.04	-0.65	-4.5	0.39	-4.1	31351.7	TRUE	TRUE	TRUE	TRUE	TRUE	TRUE
1	ADSSIAYSN	683	691	9	HLA-A*02:01	683	82	38134.84	8.20E+01	1.13E+00	-0.6550635	-4.5813219	0.47805552	-4.1032664	1.13	-0.66	-4.58	0.48	-4.1	38134.8	TRUE	TRUE	TRUE	TRUE	TRUE	TRUE
1	GDDVRQIAP	391	399	9	HLA-A*02:01	391	86	39282.3	8.60E+01	6.90E-01	-0.2001983	-4.5941969	0.489373666	-4.1048232	0.69	-0.2	-4.59	0.49	-4.1	39282.3	TRUE	TRUE	TRUE	TRUE	TRUE	TRUE
1	VVNIQKEID	1158	1166	9	HLA-A*02:01	1158	93	41595.84	9.30E+01	1.23E+00	-0.7191376	-4.6190499	0.51304943	-4.1060005	1.23	-0.72	-4.62	0.51	-4.11	41595.8	TRUE	TRUE	TRUE	TRUE	TRUE	TRUE
1	AWRIYSTGN	618	626	9	HLA-A*02:01	618	84	38639.46	8.40E+01	9.21E-01	-0.4417232	-4.587031	0.479263832	-4.1077672	0.92	-0.44	-4.59	0.48	-4.11	38639.5	TRUE	TRUE	TRUE	TRUE	TRUE	TRUE
1	VLPPLLTDD	842	850	9	HLA-A*02:01	842	41	23812.18	4.10E+01	1.02E+00	-0.7553591	-4.3767992	0.26780788	-4.1089913	1.02	-0.76	-4.38	0.27	-4.11	23812.2	TRUE	TRUE	TRUE	TRUE	TRUE	TRUE
1	ITTDNTFVS	1097	1105	9	HLA-A*02:01	1097	35	20447.83	3.50E+01	1.18E+00	-0.9876616	-4.3106472	0.193635384	-4.1170118	1.18	-0.99	-4.31	0.19	-4.12	20447.8	TRUE	TRUE	TRUE	TRUE	TRUE	TRUE
1	DTSYECDIP	643	651	9	HLA-A*02:01	643	88	39887.91	8.80E+01	5.66E-01	-0.0842568	-4.6008413	0.481595222	-4.1192461	0.57	-0.08	-4.6	0.48	-4.12	39887.9	TRUE	TRUE	TRUE	TRUE	TRUE	TRUE
1	TLVKQLSSN	943	951	9	HLA-A*02:01	943	51	28337	5.10E+01	8.07E-01	-0.4747643	-4.4523539	0.332283699	-4.1200702	0.81	-0.47	-4.45	0.33	-4.12	28337	TRUE	TRUE	TRUE	TRUE	TRUE	TRUE
1	LYQDVNCTD	597	605	9	HLA-A*02:01	597	84	38718.14	8.40E+01	1.13E+00	-0.6657692	-4.5879145	0.46576277	-4.1221517	1.13	-0.67	-4.59	0.47	-4.12	38718.1	TRUE	TRUE	TRUE	TRUE	TRUE	TRUE
1	TPSSKRFQP	539	547	9	HLA-A*02:01	539	96	42904.07	9.60E+01	5.89E-01	-0.0783901	-4.6324985	0.510276926	-4.1222216	0.59	-0.08	-4.63	0.51	-4.12	42904.1	TRUE	TRUE	TRUE	TRUE	TRUE	TRUE
1	VSTAIHADQ	606	614	9	HLA-A*02:01	606	80	37750.99	8.00E+01	5.32E-01	-0.0787327	-4.5769283	0.453450345	-4.123478	0.53	-0.08	-4.58	0.45	-4.12	37751	TRUE	TRUE	TRUE	TRUE	TRUE	TRUE
1	NFSISITTE	699	707	9	HLA-A*02:01	699	64	32784.1	6.40E+01	9.78E-01	-0.5865551	-4.5156633	0.391632935	-4.1240303	0.98	-0.59	-4.52	0.39	-4.12	32784.1	TRUE	TRUE	TRUE	TRUE	TRUE	TRUE
1	QTQAGCLIG	630	638	9	HLA-A*02:01	630	54	29464.46	5.40E+01	1.01E+00	-0.6687279	-4.4692985	0.344246059	-4.1250524	1.01	-0.67	-4.47	0.34	-4.13	29464.5	TRUE	TRUE	TRUE	TRUE	TRUE	TRUE

seq #	peptide	start	end	peptide length	allele	peptide index	median binding	ann IC50	ann percentile	proteasome sc	tap score	mhc score	processing sco	processing tota	legacy Proteas	legacy TAP Sco	legacy MHC Sc	legacy Process	legacy Total Sc	legacy MHC IC5	equal Proteaso	equal TAP Scor	equal MHC Sco	equal Processi	equal Total Sc	equal MHC IC50
1	YYPDEIFRS	41	49	9	HLA-A*02:01	41	43	24746.75	4.30E+01	1.12E+00	-0.856272	-4.3935182	0.267957994	-4.1255602	1.12	-0.86	-4.39	0.27	-4.13	24746.8	TRUE	TRUE	TRUE	TRUE	TRUE	TRUE
1	SCGSCCKFD	1231	1239	9	HLA-A*02:01	1231	75	36094.95	7.50E+01	1.26E+00	-0.8312233	-4.5574464	0.431682736	-4.1257637	1.26	-0.83	-4.56	0.43	-4.13	36094.9	TRUE	TRUE	TRUE	TRUE	TRUE	TRUE
1	CLIGAEHVD	635	643	9	HLA-A*02:01	635	51	28318.62	5.10E+01	1.11E+00	-0.7811222	-4.4520721	0.326215772	-4.1258563	1.11	-0.78	-4.45	0.33	-4.13	28318.6	TRUE	TRUE	TRUE	TRUE	TRUE	TRUE
1	IFKLPLGIN	219	227	9	HLA-A*02:01	219	66	33608.72	6.60E+01	9.51E-01	-0.5504152	-4.526452	0.400541762	-4.1259102	0.95	-0.55	-4.53	0.4	-4.13	33608.7	TRUE	TRUE	TRUE	TRUE	TRUE	TRUE
1	RLITGRLQS	977	985	9	HLA-A*02:01	977	24	13798.74	2.40E+01	9.04E-01	-0.8917772	-4.1398394	0.012379789	-4.1274596	0.9	-0.89	-4.14	0.01	-4.13	13798.7	TRUE	TRUE	TRUE	TRUE	TRUE	TRUE
1	ASVVNIQKE	1156	1164	9	HLA-A*02:01	1156	74	35861.37	7.40E+01	9.30E-01	-0.5029722	-4.5546269	0.426686833	-4.12794	0.93	-0.5	-4.55	0.43	-4.13	35861.4	TRUE	TRUE	TRUE	TRUE	TRUE	TRUE
1	AWERKKISN	339	347	9	HLA-A*02:01	339	98	43798.97	9.80E+01	9.98E-01	-0.4848261	-4.6414639	0.51293095	-4.1285329	1	-0.48	-4.64	0.51	-4.13	43799	TRUE	TRUE	TRUE	TRUE	TRUE	TRUE
1	TTDNTFVSG	1098	1106	9	HLA-A*02:01	1098	48	26994.74	4.80E+01	9.99E-01	-0.6964415	-4.4312791	0.302552499	-4.1287266	1	-0.7	-4.43	0.3	-4.13	26994.7	TRUE	TRUE	TRUE	TRUE	TRUE	TRUE
1	VVIRACNFE	123	131	9	HLA-A*02:01	123	50	27893.79	5.00E+01	8.12E-01	-0.4996054	-4.4455075	0.312495586	-4.1330119	0.81	-0.5	-4.45	0.31	-4.13	27893.8	TRUE	TRUE	TRUE	TRUE	TRUE	TRUE
1	LFNKVTLAD	804	812	9	HLA-A*02:01	804	58	31058.64	5.80E+01	1.09E+00	-0.733681	-4.4921824	0.358543957	-4.1336385	1.09	-0.73	-4.49	0.36	-4.13	31058.6	TRUE	TRUE	TRUE	TRUE	TRUE	TRUE
1	LPFYSNVTG	60	68	9	HLA-A*02:01	60	63	32581.82	6.30E+01	1.11E+00	-0.7323139	-4.5129753	0.378344128	-4.1346312	1.11	-0.73	-4.51	0.38	-4.13	32581.8	TRUE	TRUE	TRUE	TRUE	TRUE	TRUE
1	TSAAAYFVG	247	255	9	HLA-A*02:01	247	57	30772.65	5.70E+01	9.56E-01	-0.6031568	-4.4881649	0.3532022	-4.1349627	0.96	-0.6	-4.49	0.35	-4.13	30772.7	TRUE	TRUE	TRUE	TRUE	TRUE	TRUE
1	CLKGACSCG	1225	1233	9	HLA-A*02:01	1225	45	25829.26	4.50E+01	8.67E-01	-0.5923282	-4.412112	0.27464578	-4.1374662	0.87	-0.59	-4.41	0.27	-4.14	25829.3	TRUE	TRUE	TRUE	TRUE	TRUE	TRUE
1	LDKYFKNHT	1134	1142	9	HLA-A*02:01	1134	92	41068.6	9.20E+01	9.88E-01	-0.5121779	-4.6135099	0.475922064	-4.1375878	0.99	-0.51	-4.61	0.48	-4.14	41068.6	TRUE	TRUE	TRUE	TRUE	TRUE	TRUE
1	TATAGWTFG	863	871	9	HLA-A*02:01	863	67	33992.36	6.70E+01	1.01E+00	-0.6206994	-4.5313813	0.391221587	-4.1401597	1.01	-0.62	-4.53	0.39	-4.14	33992.4	TRUE	TRUE	TRUE	TRUE	TRUE	TRUE
1	YTMSLGADS	677	685	9	HLA-A*02:01	677	24	13160.35	2.40E+01	8.78E-01	-0.8994529	-4.1192674	-0.0210769	-4.1403444	0.88	-0.9	-4.12	-0.02	-4.14	13160.4	TRUE	TRUE	TRUE	TRUE	TRUE	TRUE
1	CAQKFNGLT	833	841	9	HLA-A*02:01	833	58	30773.64	5.80E+01	6.59E-01	-0.3122171	-4.4881789	0.347091915	-4.141087	0.66	-0.31	-4.49	0.35	-4.14	30773.6	TRUE	TRUE	TRUE	TRUE	TRUE	TRUE
1	CYWPLNDYG	474	482	9	HLA-A*02:01	474	52	28640	5.20E+01	6.88E-01	-0.3724541	-4.456973	0.315495917	-4.1414771	0.69	-0.37	-4.46	0.32	-4.14	28640	TRUE	TRUE	TRUE	TRUE	TRUE	TRUE
1	ITNLCPFGE	319	327	9	HLA-A*02:01	319	42	24643.61	4.20E+01	9.07E-01	-0.6569887	-4.3917043	0.250051323	-4.141653	0.91	-0.66	-4.39	0.25	-4.14	24643.6	TRUE	TRUE	TRUE	TRUE	TRUE	TRUE
1	LCFSNVYAD	377	385	9	HLA-A*02:01	377	46	26128.61	4.60E+01	1.06E+00	-0.7898874	-4.4171163	0.273919623	-4.1431967	1.06	-0.79	-4.42	0.27	-4.14	26128.6	TRUE	TRUE	TRUE	TRUE	TRUE	TRUE
1	NFNFNGLTG	526	534	9	HLA-A*02:01	526	69	34432.86	6.90E+01	9.30E-01	-0.5364869	-4.5369731	0.393599112	-4.143374	0.93	-0.54	-4.54	0.39	-4.14	34432.9	TRUE	TRUE	TRUE	TRUE	TRUE	TRUE
1	SNNTIAIPT	690	698	9	HLA-A*02:01	690	48	27088.95	4.80E+01	7.02E-01	-0.4133172	-4.4327922	0.288474799	-4.1443174	0.7	-0.41	-4.43	0.29	-4.14	27089	TRUE	TRUE	TRUE	TRUE	TRUE	TRUE
1	SMRGVYYPD	36	44	9	HLA-A*02:01	36	31	18303.31	3.10E+01	7.87E-01	-0.6691463	-4.2625296	0.118050689	-4.1444789	0.79	-0.67	-4.26	0.12	-4.14	18303.3	TRUE	TRUE	TRUE	TRUE	TRUE	TRUE
1	KKISNCVAD	343	351	9	HLA-A*02:01	343	82	38117.93	8.20E+01	1.07E+00	-0.6335849	-4.5811293	0.4364911	-4.1446382	1.07	-0.63	-4.58	0.44	-4.14	38117.9	TRUE	TRUE	TRUE	TRUE	TRUE	TRUE
1	NIDATSTGN	427	435	9	HLA-A*02:01	427	76	36508.13	7.60E+01	9.31E-01	-0.5160578	-4.5623896	0.414487203	-4.1479024	0.93	-0.52	-4.56	0.41	-4.15	36508.1	TRUE	TRUE	TRUE	TRUE	TRUE	TRUE
1	PREGVFVFN	1072	1080	9	HLA-A*02:01	1072	70	34676.61	7.00E+01	1.19E+00	-0.7964001	-4.5400366	0.389850865	-4.1501858	1.19	-0.8	-4.54	0.39	-4.15	34676.6	TRUE	TRUE	TRUE	TRUE	TRUE	TRUE
1	NLAATKMSE	1005	1013	9	HLA-A*02:01	1005	52	28723.47	5.20E+01	8.85E-01	-0.5788222	-4.4582369	0.305893787	-4.1523431	0.88	-0.58	-4.46	0.31	-4.15	28723.5	TRUE	TRUE	TRUE	TRUE	TRUE	TRUE
1	SRLDKVEAE	964	972	9	HLA-A*02:01	964	84	38777.26	8.40E+01	9.70E-01	-0.5370116	-4.5885771	0.432774443	-4.1558027	0.97	-0.54	-4.59	0.43	-4.16	38777.3	TRUE	TRUE	TRUE	TRUE	TRUE	TRUE
1	TAGWTFGAG	865	873	9	HLA-A*02:01	865	67	33791.04	6.70E+01	9.69E-01	-0.5956422	-4.5288016	0.372994782	-4.1558068	0.97	-0.6	-4.53	0.37	-4.16	33791	TRUE	TRUE	TRUE	TRUE	TRUE	TRUE
1	VVRFPNITN	313	321	9	HLA-A*02:01	313	67	33879.26	6.70E+01	8.95E-01	-0.5214002	-4.5299339	0.37365882	-4.1562751	0.9	-0.52	-4.53	0.37	-4.16	33879.3	TRUE	TRUE	TRUE	TRUE	TRUE	TRUE
1	SEKSGNFKH	173	181	9	HLA-A*02:01	173	95	42420.78	9.50E+01	7.19E-01	-0.2483159	-4.6275786	0.470773074	-4.1568056	0.72	-0.25	-4.63	0.47	-4.16	42420.8	TRUE	TRUE	TRUE	TRUE	TRUE	TRUE
1	VPSQERNFT	1050	1058	9	HLA-A*02:01	1050	93	41725.66	9.30E+01	8.99E-01	-0.4363793	-4.6204032	0.46291671	-4.1574865	0.9	-0.44	-4.62	0.46	-4.16	41725.7	TRUE	TRUE	TRUE	TRUE	TRUE	TRUE
1	EILDISPCS	569	577	9	HLA-A*02:01	569	42	24271.04	4.20E+01	1.20E+00	-0.9782503	-4.3850884	0.224534656	-4.1605537	1.2	-0.98	-4.39	0.22	-4.16	24271	TRUE	TRUE	TRUE	TRUE	TRUE	TRUE
1	TSCCSCLKG	1220	1228	9	HLA-A*02:01	1220	61	32027.82	6.10E+01	9.21E-01	-0.5778594	-4.5055274	0.343524554	-4.1620028	0.92	-0.58	-4.51	0.34	-4.16	32027.8	TRUE	TRUE	TRUE	TRUE	TRUE	TRUE
1	MAYRFNGIG	884	892	9	HLA-A*02:01	884	56	30357.94	5.60E+01	8.19E-01	-0.5021941	-4.4822723	0.316342895	-4.1659294	0.82	-0.5	-4.48	0.32	-4.17	30357.9	TRUE	TRUE	TRUE	TRUE	TRUE	TRUE
1	QCVNFNFNG	523	531	9	HLA-A*02:01	523	53	29163.46	5.30E+01	7.95E-01	-0.4961764	-4.464839	0.298527621	-4.1663114	0.79	-0.5	-4.46	0.3	-4.17	29163.5	TRUE	TRUE	TRUE	TRUE	TRUE	TRUE
1	PQIITTDNT	1094	1102	9	HLA-A*02:01	1094	71	35048.16	7.10E+01	8.05E-01	-0.4295004	-4.5446652	0.375623588	-4.1690416	0.81	-0.43	-4.54	0.38	-4.17	35048.2	TRUE	TRUE	TRUE	TRUE	TRUE	TRUE
1	NNTIAIPTN	691	699	9	HLA-A*02:01	691	87	39615.25	8.70E+01	1.04E+00	-0.6112537	-4.5978624	0.426004294	-4.1718581	1.04	-0.61	-4.6	0.43	-4.17	39615.2	TRUE	TRUE	TRUE	TRUE	TRUE	TRUE
1	CANLLLQYG	731	739	9	HLA-A*02:01	731	35	20566.76	3.50E+01	8.54E-01	-0.7140195	-4.3131659	0.139996514	-4.1731694	0.85	-0.71	-4.31	0.14	-4.17	20566.8	TRUE	TRUE	TRUE	TRUE	TRUE	TRUE
1	QTHTMIFDN	147	155	9	HLA-A*02:01	147	60	31519.73	6.00E+01	8.81E-01	-0.5561111	-4.4985825	0.324827904	-4.1737546	0.88	-0.56	-4.5	0.32	-4.17	31519.7	TRUE	TRUE	TRUE	TRUE	TRUE	TRUE
1	PFGEVFNAT	324	332	9	HLA-A*02:01	324	59	31396.51	5.90E+01	8.36E-01	-0.5162341	-4.4968814	0.319337904	-4.1775435	0.84	-0.52	-4.5	0.32	-4.18	31396.5	TRUE	TRUE	TRUE	TRUE	TRUE	TRUE
1	YFAATEKSN	88	96	9	HLA-A*02:01	88	94	42045.16	9.40E+01	9.39E-01	-0.4936025	-4.623716	0.445812457	-4.1779036	0.94	-0.49	-4.62	0.45	-4.18	42045.2	TRUE	TRUE	TRUE	TRUE	TRUE	TRUE
1	VDFCGKGYH	1022	1030	9	HLA-A*02:01	1022	90	40454.69	9.00E+01	6.59E-01	-0.2319542	-4.6069689	0.427088819	-4.1798801	0.66	-0.23	-4.61	0.43	-4.18	40454.7	TRUE	TRUE	TRUE	TRUE	TRUE	TRUE
1	LSGIAAEQD	749	757	9	HLA-A*02:01	749	77	36737.96	7.70E+01	1.16E+00	-0.7731953	-4.565115	0.38494473	-4.1801703	1.16	-0.77	-4.57	0.38	-4.18	36738	TRUE	TRUE	TRUE	TRUE	TRUE	TRUE
1	SGNCDVVIG	1105	1113	9	HLA-A*02:01	1105	68	34084.8	6.80E+01	1.07E+00	-0.7188946	-4.5325608	0.349567368	-4.1829934	1.07	-0.72	-4.53	0.35	-4.18	34084.8	TRUE	TRUE	TRUE	TRUE	TRUE	TRUE
1	PIDVVRDLP	202	210	9	HLA-A*02:01	202	73	35611.23	7.30E+01	5.74E-01	-0.2065018	-4.551587	0.367775181	-4.1838118	0.57	-0.21	-4.55	0.37	-4.18	35611.2	TRUE	TRUE	TRUE	TRUE	TRUE	TRUE
1	VTQNVLYEN	893	901	9	HLA-A*02:01	893	40	23365.3	4.00E+01	7.59E-01	-0.5788404	-4.3685714	0.180373578	-4.1881978	0.76	-0.58	-4.37	0.18	-4.19	23365.3	TRUE	TRUE	TRUE	TRUE	TRUE	TRUE
1	TQLNRALSG	743	751	9	HLA-A*02:01	743	46	26252.46	4.60E+01	8.03E-01	-0.5772666	-4.41917	0.225728367	-4.1934416	0.8	-0.58	-4.42	0.23	-4.19	26252.5	TRUE	TRUE	TRUE	TRUE	TRUE	TRUE
1	REFVFKNKD	183	191	9	HLA-A*02:01	183	88	39915.55	8.80E+01	1.08E+00	-0.671981	-4.6011421	0.40702604	-4.1941161	1.08	-0.67	-4.6	0.41	-4.19	39915.6	TRUE	TRUE	TRUE	TRUE	TRUE	TRUE
1	DLIKNQCVN	518	526	9	HLA-A*02:01	518	80	37729.35	8.00E+01	1.04E+00	-0.6636096	-4.5766793	0.38062637	-4.196053	1.04	-0.66	-4.58	0.38	-4.2	37729.3	TRUE	TRUE	TRUE	TRUE	TRUE	TRUE
1	FLTLTSGSD	7	15	9	HLA-A*02:01	7	37	21544.6	3.70E+01	9.52E-01	-0.8157986	-4.3333384	0.13574237	-4.1975961	0.95	-0.82	-4.33	0.14	-4.2	21544.6	TRUE	TRUE	TRUE	TRUE	TRUE	TRUE
1	SSVLNDILS	956	964	9	HLA-A*02:01	956	44	25608.59	4.40E+01	1.11E+00	-0.8986362	-4.4083857	0.209681815	-4.1987039	1.11	-0.9	-4.41	0.21	-4.2	25608.6	TRUE	TRUE	TRUE	TRUE	TRUE	TRUE
1	FRAILTAFS	231	239	9	HLA-A*02:01	231	30	17540.01	3.00E+01	9.01E-01	-0.8591953	-4.2440298	0.04221574	-4.2018141	0.9	-0.86	-4.24	0.04	-4.2	17540	TRUE	TRUE	TRUE	TRUE	TRUE	TRUE
1	GWVFGSTMN	100	108	9	HLA-A*02:01	100	87	39513.36	8.70E+01	8.40E-01	-0.4493379	-4.596744	0.390980115	-4.2057638	0.84	-0.45	-4.6	0.39	-4.21	39513.4	TRUE	TRUE	TRUE	TRUE	TRUE	TRUE
1	RDLPSGFNT	207	215	9	HLA-A*02:01	207	66	33510.68	6.60E+01	6.23E-01	-0.3061377	-4.5251832	0.316879252	-4.208304	0.62	-0.31	-4.53	0.32	-4.21	33510.7	TRUE	TRUE	TRUE	TRUE	TRUE	TRUE
1	CSVKSFEID	288	296	9	HLA-A*02:01	288	70	34770.53	7.00E+01	1.04E+00	-0.7120299	-4.5412113	0.332242149	-4.2089692	1.04	-0.71	-4.54	0.33	-4.21	34770.5	TRUE	TRUE	TRUE	TRUE	TRUE	TRUE
1	RPFERDISN	449	457	9	HLA-A*02:01	449	72	35468.18	7.20E+01	8.64E-01	-0.5255834	-4.5498389	0.338201582	-4.2116373	0.86	-0.53	-4.55	0.34	-4.21	35468.2	TRUE	TRUE	TRUE	TRUE	TRUE	TRUE
1	NKAISQIQE	910	918	9	HLA-A*02:01	910	93	41485.28	9.30E+01	1.08E+00	-0.6786332	-4.617894	0.405155771	-4.2127383	1.08	-0.68	-4.62	0.41	-4.21	41485.3	TRUE	TRUE	TRUE	TRUE	TRUE	TRUE
1	TDSVRDPKT	559	567	9	HLA-A*02:01	559	98	43854	9.80E+01	9.25E-01	-0.4971562	-4.6420092	0.427718776	-4.2142904	0.92	-0.5	-4.64	0.43	-4.21	43854	TRUE	TRUE	TRUE	TRUE	TRUE	TRUE
1	SEVAVLYQD	592	600	9	HLA-A*02:01	592	53	29296.92	5.30E+01	1.02E+00	-0.7679712	-4.466822	0.251702787	-4.2151192	1.02	-0.77	-4.47	0.25	-4.22	29296.9	TRUE	TRUE	TRUE	TRUE	TRUE	TRUE
1	KVEAEVQID	968	976	9	HLA-A*02:01	968	81	37782.04	8.10E+01	1.17E+00	-0.8052703	-4.5772854	0.359882749	-4.2174027	1.17	-0.81	-4.58	0.36	-4.22	37782	TRUE	TRUE	TRUE	TRUE	TRUE	TRUE
1	KDGIYFAAT	84	92	9	HLA-A*02:01	84	43	24766.84	4.30E+01	6.85E-01	-0.5103484	-4.3938706	0.174694595	-4.219176	0.69	-0.51	-4.39	0.17	-4.22	24766.8	TRUE	TRUE	TRUE	TRUE	TRUE	TRUE
1	FFSTFKCYG	360	368	9	HLA-A*02:01	360	41	24035.57	4.10E+01	7.33E-01	-0.5710864	-4.3808544	0.161631608	-4.2192228	0.73	-0.57	-4.38	0.16	-4.22	24035.6	TRUE	TRUE	TRUE	TRUE	TRUE	TRUE
1	SGINASVVN	1152	1160	9	HLA-A*02:01	1152	88	39814.61	8.80E+01	1.01E+00	-0.6282342	-4.6000425	0.380584846	-4.2194576	1.01	-0.63	-4.6	0.38	-4.22	39814.6	TRUE	TRUE	TRUE	TRUE	TRUE	TRUE
1	YGSFCTQLN	738	746	9	HLA-A*02:01	738	61	32006	6.10E+01	1.00E+00	-0.7168538	-4.5052314	0.285699176	-4.2195322	1	-0.72	-4.51	0.29	-4.22	32006	TRUE	TRUE	TRUE	TRUE	TRUE	TRUE
1	IQKEIDRLN	1161	1169	9	HLA-A*02:01	1161	69	34496.25	6.90E+01	7.82E-01	-0.4663582	-4.5377719	0.315505815	-4.2222661	0.78	-0.47	-4.54	0.32	-4.22	34496.2	TRUE	TRUE	TRUE	TRUE	TRUE	TRUE
1	TAPAICHEG	1059	1067	9	HLA-A*02:01	1059	57	30642.74	5.70E+01	8.57E-01	-0.5941551	-4.4863276	0.262594865	-4.2237327	0.86	-0.59	-4.49	0.26	-4.22	30642.7	TRUE	TRUE	TRUE	TRUE	TRUE	TRUE
1	SLDVSEKSG	169	177	9	HLA-A*02:01	169	54	29514.23	5.40E+01	9.41E-01	-0.6956865	-4.4700315	0.245542477	-4.224489	0.94	-0.7	-4.47	0.25	-4.22	29514.2	TRUE	TRUE	TRUE	TRUE	TRUE	TRUE
1	VRDLPSGFN	206	214	9	HLA-A*02:01	206	89	40238.16	8.90E+01	8.88E-01	-0.5088441	-4.6046381	0.378669899	-4.2259682	0.89	-0.51	-4.6	0.38	-4.23	40238.2	TRUE	TRUE	TRUE	TRUE	TRUE	TRUE
1	STFKCYGVS	362	370	9	HLA-A*02:01	362	44	25229.61	4.40E+01	1.13E+00	-0.9598256	-4.4019105	0.173131416	-4.2287791	1.13	-0.96	-4.4	0.17	-4.23	25229.6	TRUE	TRUE	TRUE	TRUE	TRUE	TRUE
1	YQPIDVVRD	200	208	9	HLA-A*02:01	200	48	27125.91	4.80E+01	9.81E-01	-0.7842887	-4.4333843	0.197177329	-4.236207	0.98	-0.78	-4.43	0.2	-4.24	27125.9	TRUE	TRUE	TRUE	TRUE	TRUE	TRUE
1	YAWERKKIS	338	346	9	HLA-A*02:01	338	56	30141.6	5.60E+01	1.11E+00	-0.8673579	-4.4791663	0.24073705	-4.2384293	1.11	-0.87	-4.48	0.24	-4.24	30141.6	TRUE	TRUE	TRUE	TRUE	TRUE	TRUE
1	STALGKLQD	924	932	9	HLA-A*02:01	924	84	38810.84	8.40E+01	1.11E+00	-0.7639857	-4.588953	0.348704328	-4.2402487	1.11	-0.76	-4.59	0.35	-4.24	38810.8	TRUE	TRUE	TRUE	TRUE	TRUE	TRUE
1	VITPGTNAS	583	591	9	HLA-A*02:01	583	44	25457.2	4.40E+01	1.05E+00	-0.8861535	-4.4058106	0.162010532	-4.2438001	1.05	-0.89	-4.41	0.16	-4.24	25457.2	TRUE	TRUE	TRUE	TRUE	TRUE	TRUE
1	SGTATAGWT	861	869	9	HLA-A*02:01	861	86	39380.62	8.60E+01	7.44E-01	-0.3953647	-4.5952825	0.349051315	-4.2462312	0.74	-0.4	-4.6	0.35	-4.25	39380.6	TRUE	TRUE	TRUE	TRUE	TRUE	TRUE
1	TNVVIRACN	121	129	9	HLA-A*02:01	121	91	40770.19	9.10E+01	9.51E-01	-0.5876008	-4.6103427	0.363733165	-4.2466096	0.95	-0.59	-4.61	0.36	-4.25	40770.2	TRUE	TRUE	TRUE	TRUE	TRUE	TRUE
1	CLGDINARD	822	830	9	HLA-A*02:01	822	39	22643.78	3.90E+01	1.05E+00	-0.9414806	-4.3549489	0.108321372	-4.2466276	1.05	-0.94	-4.35	0.11	-4.25	22643.8	TRUE	TRUE	TRUE	TRUE	TRUE	TRUE
1	YDENGTITD	266	274	9	HLA-A*02:01	266	86	39298.04	8.60E+01	1.30E+00	-0.9498842	-4.5943709	0.347449755	-4.2469211	1.3	-0.95	-4.59	0.35	-4.25	39298	TRUE	TRUE	TRUE	TRUE	TRUE	TRUE
1	QSVIIINNS	112	120	9	HLA-A*02:01	112	38	22566.25	3.80E+01	1.00E+00	-0.8965229	-4.3534594	0.104938147	-4.2485212	1	-0.9	-4.35	0.1	-4.25	22566.2	TRUE	TRUE	TRUE	TRUE	TRUE	TRUE
1	LNCYWPLND	472	480	9	HLA-A*02:01	472	73	35692.62	7.30E+01	1.08E+00	-0.7821258	-4.5525784	0.302656165	-4.2499223	1.08	-0.78	-4.55	0.3	-4.25	35692.6	TRUE	TRUE	TRUE	TRUE	TRUE	TRUE
1	TVYDPLQPE	1118	1126	9	HLA-A*02:01	1118	47	26763.81	4.70E+01	6.62E-01	-0.4847126	-4.4275479	0.17753135	-4.2500166	0.66	-0.48	-4.43	0.18	-4.25	26763.8	TRUE	TRUE	TRUE	TRUE	TRUE	TRUE
1	IRAAEIRAS	995	1003	9	HLA-A*02:01	995	81	37828.68	8.10E+01	1.18E+00	-0.8488728	-4.5778212	0.327353223	-4.250468	1.18	-0.85	-4.58	0.33	-4.25	37828.7	TRUE	TRUE	TRUE	TRUE	TRUE	TRUE
1	TKLNDLCFS	372	380	9	HLA-A*02:01	372	61	32104.86	6.10E+01	1.14E+00	-0.8895194	-4.5065708	0.254306572	-4.2522642	1.14	-0.89	-4.51	0.25	-4.25	32104.9	TRUE	TRUE	TRUE	TRUE	TRUE	TRUE
1	GIINNTVYD	1113	1121	9	HLA-A*02:01	1113	49	27614.52	4.90E+01	9.64E-01	-0.7770086	-4.4411375	0.186527417	-4.2546101	0.96	-0.78	-4.44	0.19	-4.25	27614.5	TRUE	TRUE	TRUE	TRUE	TRUE	TRUE
1	WRIYSTGNN	619	627	9	HLA-A*02:01	619	98	43914.28	9.80E+01	7.80E-01	-0.3946049	-4.6426058	0.385506118	-4.2570996	0.78	-0.39	-4.64	0.39	-4.26	43914.3	TRUE	TRUE	TRUE	TRUE	TRUE	TRUE
1	NEVAKNLNE	1169	1177	9	HLA-A*02:01	1169	94	41858.59	9.40E+01	1.03E+00	-0.6661704	-4.6217846	0.362832641	-4.258952	1.03	-0.67	-4.62	0.36	-4.26	41858.6	TRUE	TRUE	TRUE	TRUE	TRUE	TRUE
1	DAFSLDVSE	166	174	9	HLA-A*02:01	166	72	35367.77	7.20E+01	1.01E+00	-0.7206507	-4.5486077	0.289272318	-4.2593354	1.01	-0.72	-4.55	0.29	-4.26	35367.8	TRUE	TRUE	TRUE	TRUE	TRUE	TRUE
1	FNGIGVTQN	888	896	9	HLA-A*02:01	888	72	35277.95	7.20E+01	9.70E-01	-0.6894789	-4.5475033	0.280413131	-4.2670902	0.97	-0.69	-4.55	0.28	-4.27	35277.9	TRUE	TRUE	TRUE	TRUE	TRUE	TRUE
1	DSSIAYSNN	684	692	9	HLA-A*02:01	684	96	42545.33	9.60E+01	9.81E-01	-0.6240353	-4.6288519	0.35659374	-4.2722582	0.98	-0.62	-4.63	0.36	-4.27	42545.3	TRUE	TRUE	TRUE	TRUE	TRUE	TRUE
1	ATVCGPKLS	508	516	9	HLA-A*02:01	508	68	34335.76	6.80E+01	1.21E+00	-0.9524865	-4.5357467	0.260453471	-4.2752932	1.21	-0.95	-4.54	0.26	-4.28	34335.8	TRUE	TRUE	TRUE	TRUE	TRUE	TRUE
1	IYFAATEKS	87	95	9	HLA-A*02:01	87	63	32529.33	6.30E+01	1.05E+00	-0.8161076	-4.5122751	0.234547389	-4.2777277	1.05	-0.82	-4.51	0.23	-4.28	32529.3	TRUE	TRUE	TRUE	TRUE	TRUE	TRUE
1	QSKRVDFCG	1018	1026	9	HLA-A*02:01	1018	79	37251.5	7.90E+01	8.90E-01	-0.5964918	-4.5711438	0.293375192	-4.2777686	0.89	-0.6	-4.57	0.29	-4.28	37251.5	TRUE	TRUE	TRUE	TRUE	TRUE	TRUE
1	DLGDISGIN	1147	1155	9	HLA-A*02:01	1147	72	35481.22	7.20E+01	1.02E+00	-0.7482472	-4.5499985	0.270323845	-4.2796747	1.02	-0.75	-4.55	0.27	-4.28	35481.2	TRUE	TRUE	TRUE	TRUE	TRUE	TRUE
1	YISDAFSLD	163	171	9	HLA-A*02:01	163	35	20839.81	3.50E+01	8.52E-01	-0.816208	-4.3188938	0.03596903	-4.2829247	0.85	-0.82	-4.32	0.04	-4.28	20839.8	TRUE	TRUE	TRUE	TRUE	TRUE	TRUE
1	AEQDRNTRE	754	762	9	HLA-A*02:01	754	99	44452.1	9.90E+01	9.81E-01	-0.6214863	-4.6478923	0.359601695	-4.2882906	0.98	-0.62	-4.65	0.36	-4.29	44452.1	TRUE	TRUE	TRUE	TRUE	TRUE	TRUE
1	NCTFEYISD	158	166	9	HLA-A*02:01	158	86	39218.62	8.60E+01	1.13E+00	-0.8250496	-4.5934923	0.304495432	-4.2889969	1.13	-0.83	-4.59	0.3	-4.29	39218.6	TRUE	TRUE	TRUE	TRUE	TRUE	TRUE
1	FSPAQDIWG	238	246	9	HLA-A*02:01	238	53	29147.7	5.30E+01	7.44E-01	-0.5683345	-4.4646043	0.175328523	-4.2892758	0.74	-0.57	-4.46	0.18	-4.29	29147.7	TRUE	TRUE	TRUE	TRUE	TRUE	TRUE
1	TQHTSSMRG	31	39	9	HLA-A*02:01	31	68	34306.04	6.80E+01	8.37E-01	-0.5917963	-4.5353706	0.245302691	-4.2900679	0.84	-0.59	-4.54	0.25	-4.29	34306	TRUE	TRUE	TRUE	TRUE	TRUE	TRUE
1	MPVSMAKTS	709	717	9	HLA-A*02:01	709	82	38075.89	8.20E+01	1.32E+00	-1.0341913	-4.5806501	0.290259655	-4.2903904	1.32	-1.03	-4.58	0.29	-4.29	38075.9	TRUE	TRUE	TRUE	TRUE	TRUE	TRUE
1	DGFLYVYKG	191	199	9	HLA-A*02:01	191	49	27625.28	4.90E+01	9.16E-01	-0.7660607	-4.4413067	0.150024306	-4.2912824	0.92	-0.77	-4.44	0.15	-4.29	27625.3	TRUE	TRUE	TRUE	TRUE	TRUE	TRUE
1	FEYISDAFS	161	169	9	HLA-A*02:01	161	33	19569.46	3.30E+01	9.90E-01	-0.9909994	-4.2915788	-0.0013204	-4.2928992	0.99	-0.99	-4.29	0	-4.29	19569.5	TRUE	TRUE	TRUE	TRUE	TRUE	TRUE
1	LNAPATVCG	504	512	9	HLA-A*02:01	504	82	38123.7	8.20E+01	9.28E-01	-0.6412524	-4.581195	0.286350611	-4.2948444	0.93	-0.64	-4.58	0.29	-4.29	38123.7	TRUE	TRUE	TRUE	TRUE	TRUE	TRUE
1	EELDKYFKN	1132	1140	9	HLA-A*02:01	1132	98	43467.57	9.80E+01	1.01E+00	-0.6652833	-4.6381654	0.34140471	-4.2967607	1.01	-0.67	-4.64	0.34	-4.3	43467.6	TRUE	TRUE	TRUE	TRUE	TRUE	TRUE
1	MKQYGECLG	816	824	9	HLA-A*02:01	816	86	39183.83	8.60E+01	9.18E-01	-0.6231141	-4.5931069	0.29485487	-4.298252	0.92	-0.62	-4.59	0.29	-4.3	39183.8	TRUE	TRUE	TRUE	TRUE	TRUE	TRUE
1	VYKGYQPID	196	204	9	HLA-A*02:01	196	100	45094.44	1.00E+02	1.03E+00	-0.6703968	-4.654123	0.355796189	-4.2983268	1.03	-0.67	-4.65	0.36	-4.3	45094.4	TRUE	TRUE	TRUE	TRUE	TRUE	TRUE
1	IQESLTTTS	916	924	9	HLA-A*02:01	916	43	24817.54	4.30E+01	1.11E+00	-1.0179689	-4.3947587	0.09625609	-4.2985026	1.11	-1.02	-4.39	0.1	-4.3	24817.5	TRUE	TRUE	TRUE	TRUE	TRUE	TRUE
1	SFPQAAPHG	1033	1041	9	HLA-A*02:01	1033	87	39736.73	8.70E+01	8.00E-01	-0.500243	-4.5991921	0.300082964	-4.2991092	0.8	-0.5	-4.6	0.3	-4.3	39736.7	TRUE	TRUE	TRUE	TRUE	TRUE	TRUE
1	TLKYFGGFN	775	783	9	HLA-A*02:01	775	53	29288.68	5.30E+01	8.01E-01	-0.6338765	-4.4666998	0.167298539	-4.2994013	0.8	-0.63	-4.47	0.17	-4.3	29288.7	TRUE	TRUE	TRUE	TRUE	TRUE	TRUE
1	ERNFTTAPA	1054	1062	9	HLA-A*02:01	1054	68	34164.18	6.80E+01	5.26E-01	-0.292635	-4.533571	0.232990956	-4.30058	0.53	-0.29	-4.53	0.23	-4.3	34164.2	TRUE	TRUE	TRUE	TRUE	TRUE	TRUE
1	AYTMSLGAD	676	684	9	HLA-A*02:01	676	84	38812.52	8.40E+01	8.94E-01	-0.6065959	-4.5889718	0.287692131	-4.3012797	0.89	-0.61	-4.59	0.29	-4.3	38812.5	TRUE	TRUE	TRUE	TRUE	TRUE	TRUE
1	TTAPAICHE	1058	1066	9	HLA-A*02:01	1058	67	33724.55	6.70E+01	9.61E-01	-0.7350812	-4.5279462	0.225522792	-4.3024234	0.96	-0.74	-4.53	0.23	-4.3	33724.6	TRUE	TRUE	TRUE	TRUE	TRUE	TRUE
1	LTPAWRIYS	615	623	9	HLA-A*02:01	615	33	19751.97	3.30E+01	1.00E+00	-1.0115487	-4.2956104	-0.0074027	-4.3030132	1	-1.01	-4.3	-0.01	-4.3	19752	TRUE	TRUE	TRUE	TRUE	TRUE	TRUE
1	GFYTTTGIG	482	490	9	HLA-A*02:01	482	75	36178.62	7.50E+01	8.03E-01	-0.5516093	-4.558452	0.251881685	-4.3065703	0.8	-0.55	-4.56	0.25	-4.31	36178.6	TRUE	TRUE	TRUE	TRUE	TRUE	TRUE
1	TEKSNVVRG	92	100	9	HLA-A*02:01	92	89	40182.92	8.90E+01	9.04E-01	-0.6076498	-4.6040415	0.296759217	-4.3072823	0.9	-0.61	-4.6	0.3	-4.31	40182.9	TRUE	TRUE	TRUE	TRUE	TRUE	TRUE
1	NTLVKQLSS	942	950	9	HLA-A*02:01	942	57	30731.04	5.70E+01	1.12E+00	-0.940628	-4.4875773	0.179930048	-4.3076472	1.12	-0.94	-4.49	0.18	-4.31	30731	TRUE	TRUE	TRUE	TRUE	TRUE	TRUE
1	YENQKQIAN	899	907	9	HLA-A*02:01	899	88	39925.05	8.80E+01	9.47E-01	-0.6566204	-4.6012455	0.290098603	-4.3111469	0.95	-0.66	-4.6	0.29	-4.31	39925.1	TRUE	TRUE	TRUE	TRUE	TRUE	TRUE
1	NFSQILPDP	783	791	9	HLA-A*02:01	783	65	33242.37	6.50E+01	1.22E-01	0.08601422	-4.521692	0.208476224	-4.3132158	0.12	0.09	-4.52	0.21	-4.31	33242.4	TRUE	TRUE	TRUE	TRUE	TRUE	TRUE
1	VLGQSKRVD	1015	1023	9	HLA-A*02:01	1015	88	39768.55	8.80E+01	1.11E+00	-0.8268755	-4.5995398	0.286253537	-4.3132862	1.11	-0.83	-4.6	0.29	-4.31	39768.6	TRUE	TRUE	TRUE	TRUE	TRUE	TRUE
1	GVFVFNGTS	1075	1083	9	HLA-A*02:01	1075	30	17587.14	3.00E+01	9.50E-01	-1.0183185	-4.2451952	-0.0683605	-4.3135558	0.95	-1.02	-4.25	-0.07	-4.31	17587.1	TRUE	TRUE	TRUE	TRUE	TRUE	TRUE
1	PPALNCYWP	469	477	9	HLA-A*02:01	469	77	36731.61	7.70E+01	5.35E-01	-0.2852875	-4.56504	0.249644496	-4.3153955	0.53	-0.29	-4.57	0.25	-4.32	36731.6	TRUE	TRUE	TRUE	TRUE	TRUE	TRUE
1	SSNFGAISS	949	957	9	HLA-A*02:01	949	54	29431.96	5.40E+01	1.11E+00	-0.9561141	-4.4688192	0.151621856	-4.3171973	1.11	-0.96	-4.47	0.15	-4.32	29432	TRUE	TRUE	TRUE	TRUE	TRUE	TRUE
1	TKMSECVLG	1009	1017	9	HLA-A*02:01	1009	65	33321.23	6.50E+01	7.04E-01	-0.4983524	-4.522721	0.205384637	-4.3173364	0.7	-0.5	-4.52	0.21	-4.32	33321.2	TRUE	TRUE	TRUE	TRUE	TRUE	TRUE
1	SNFRVVPSG	303	311	9	HLA-A*02:01	303	61	31825.06	6.10E+01	7.81E-01	-0.5957804	-4.5027692	0.184794603	-4.3179746	0.78	-0.6	-4.5	0.18	-4.32	31825.1	TRUE	TRUE	TRUE	TRUE	TRUE	TRUE
1	AAEIRASAN	997	1005	9	HLA-A*02:01	997	84	38754.61	8.40E+01	7.55E-01	-0.488919	-4.5883234	0.266160021	-4.3221633	0.76	-0.49	-4.59	0.27	-4.32	38754.6	TRUE	TRUE	TRUE	TRUE	TRUE	TRUE
1	NFNGLTGTG	528	536	9	HLA-A*02:01	528	82	38048.69	8.20E+01	8.59E-01	-0.6022304	-4.5803397	0.256605611	-4.3237341	0.86	-0.6	-4.58	0.26	-4.32	38048.7	TRUE	TRUE	TRUE	TRUE	TRUE	TRUE
1	PSGFNTLKP	210	218	9	HLA-A*02:01	210	92	41085.48	9.20E+01	4.87E-01	-0.1969891	-4.6136884	0.289949895	-4.3237385	0.49	-0.2	-4.61	0.29	-4.32	41085.5	TRUE	TRUE	TRUE	TRUE	TRUE	TRUE
1	LCCMTSCCS	1216	1224	9	HLA-A*02:01	1216	46	26320.99	4.60E+01	1.04E+00	-0.9413072	-4.4203022	0.09476976	-4.3255325	1.04	-0.94	-4.42	0.09	-4.33	26321	TRUE	TRUE	TRUE	TRUE	TRUE	TRUE
1	DSVRDPKTS	560	568	9	HLA-A*02:01	560	99	44596.13	9.90E+01	1.38E+00	-1.0585799	-4.6492972	0.321143093	-4.3281541	1.38	-1.06	-4.65	0.32	-4.33	44596.1	TRUE	TRUE	TRUE	TRUE	TRUE	TRUE
1	HVTYVPSQE	1046	1054	9	HLA-A*02:01	1046	84	38644.9	8.40E+01	9.10E-01	-0.6524873	-4.5870922	0.257204715	-4.3298875	0.91	-0.65	-4.59	0.26	-4.33	38644.9	TRUE	TRUE	TRUE	TRUE	TRUE	TRUE
1	FHTINHTFG	69	77	9	HLA-A*02:01	69	80	37507.51	8.00E+01	9.16E-01	-0.672152	-4.5741182	0.244188951	-4.3299293	0.92	-0.67	-4.57	0.24	-4.33	37507.5	TRUE	TRUE	TRUE	TRUE	TRUE	TRUE
1	GVTQNVLYE	892	900	9	HLA-A*02:01	892	51	28264.12	5.10E+01	8.28E-01	-0.707747	-4.4512355	0.120017967	-4.3312175	0.83	-0.71	-4.45	0.12	-4.33	28264.1	TRUE	TRUE	TRUE	TRUE	TRUE	TRUE
1	KAISQIQES	911	919	9	HLA-A*02:01	911	46	26201.66	4.60E+01	9.02E-01	-0.8158555	-4.4183288	0.08654752	-4.3317813	0.9	-0.82	-4.42	0.09	-4.33	26201.7	TRUE	TRUE	TRUE	TRUE	TRUE	TRUE
1	ADYSVLYNS	350	358	9	HLA-A*02:01	350	37	21505.25	3.70E+01	9.44E-01	-0.9458037	-4.3325445	-0.0018847	-4.3344292	0.94	-0.95	-4.33	0	-4.33	21505.2	TRUE	TRUE	TRUE	TRUE	TRUE	TRUE
1	PAWRIYSTG	617	625	9	HLA-A*02:01	617	78	37058.54	7.80E+01	9.46E-01	-0.7123848	-4.5688883	0.234083226	-4.3348051	0.95	-0.71	-4.57	0.23	-4.33	37058.5	TRUE	TRUE	TRUE	TRUE	TRUE	TRUE
1	NTFVSGNCD	1101	1109	9	HLA-A*02:01	1101	83	38545.93	8.30E+01	1.04E+00	-0.793378	-4.5859785	0.249012012	-4.3369665	1.04	-0.79	-4.59	0.25	-4.34	38545.9	TRUE	TRUE	TRUE	TRUE	TRUE	TRUE
1	AGFMKQYGE	813	821	9	HLA-A*02:01	813	77	36696.27	7.70E+01	9.65E-01	-0.7428597	-4.5646219	0.221744274	-4.3428776	0.96	-0.74	-4.56	0.22	-4.34	36696.3	TRUE	TRUE	TRUE	TRUE	TRUE	TRUE
1	HTSPDVDLG	1141	1149	9	HLA-A*02:01	1141	67	33753.4	6.70E+01	7.76E-01	-0.5948574	-4.5283175	0.181475609	-4.3468419	0.78	-0.59	-4.53	0.18	-4.35	33753.4	TRUE	TRUE	TRUE	TRUE	TRUE	TRUE
1	PTNFSISIT	697	705	9	HLA-A*02:01	697	75	36209.17	7.50E+01	7.71E-01	-0.5615798	-4.5588186	0.209051197	-4.3497674	0.77	-0.56	-4.56	0.21	-4.35	36209.2	TRUE	TRUE	TRUE	TRUE	TRUE	TRUE
1	LTTTSTALG	920	928	9	HLA-A*02:01	920	64	32971.21	6.40E+01	7.72E-01	-0.6051644	-4.5181349	0.166938607	-4.3511963	0.77	-0.61	-4.52	0.17	-4.35	32971.2	TRUE	TRUE	TRUE	TRUE	TRUE	TRUE
1	DCNMYICGD	719	727	9	HLA-A*02:01	719	74	36068.01	7.40E+01	1.14E+00	-9.38E-01	-4.5571222	0.205818994	-4.3513032	1.14	-0.94	-4.56	0.21	-4.35	36068	TRUE	TRUE	TRUE	TRUE	TRUE	TRUE
1	LICAQKFNG	831	839	9	HLA-A*02:01	831	53	29108.29	5.30E+01	7.12E-01	-0.6042248	-4.4640167	0.107723223	-4.3562935	0.71	-0.6	-4.46	0.11	-4.36	29108.3	TRUE	TRUE	TRUE	TRUE	TRUE	TRUE
1	TDNTFVSGN	1099	1107	9	HLA-A*02:01	1099	87	39556.99	8.70E+01	9.86E-01	-0.7487825	-4.5972232	0.237585545	-4.3596377	0.99	-0.75	-4.6	0.24	-4.36	39557	TRUE	TRUE	TRUE	TRUE	TRUE	TRUE
1	TMSLGADSS	678	686	9	HLA-A*02:01	678	36	21103.48	3.60E+01	9.19E-01	-0.9576319	-4.3243541	-0.0385619	-4.3629159	0.92	-0.96	-4.32	-0.04	-4.36	21103.5	TRUE	TRUE	TRUE	TRUE	TRUE	TRUE
1	CKFDEDDSE	1236	1244	9	HLA-A*02:01	1236	93	41626	9.30E+01	8.84E-01	-0.6291804	-4.6193647	0.255211578	-4.3641531	0.88	-0.63	-4.62	0.26	-4.36	41626	TRUE	TRUE	TRUE	TRUE	TRUE	TRUE
1	QPYRVVVLS	492	500	9	HLA-A*02:01	492	39	22831.25	3.90E+01	1.04E+00	-1.0442147	-4.3585297	-0.0058907	-4.3644203	1.04	-1.04	-4.36	-0.01	-4.36	22831.2	TRUE	TRUE	TRUE	TRUE	TRUE	TRUE
1	YGVSATKLN	367	375	9	HLA-A*02:01	367	90	40452.95	9.00E+01	9.00E-01	-0.6630678	-4.6069502	0.236705162	-4.370245	0.9	-0.66	-4.61	0.24	-4.37	40452.9	TRUE	TRUE	TRUE	TRUE	TRUE	TRUE
1	LSSNFGAIS	948	956	9	HLA-A*02:01	948	53	29264.61	5.30E+01	1.07E+00	-0.980299	-4.4663427	0.08670201	-4.3796407	1.07	-0.98	-4.47	0.09	-4.38	29264.6	TRUE	TRUE	TRUE	TRUE	TRUE	TRUE
1	TIAIPTNFS	693	701	9	HLA-A*02:01	693	43	25042.49	4.30E+01	8.78E-01	-0.8613809	-4.3986775	0.016532133	-4.3821454	0.88	-0.86	-4.4	0.02	-4.38	25042.5	TRUE	TRUE	TRUE	TRUE	TRUE	TRUE
1	FFAVSKPMG	137	145	9	HLA-A*02:01	137	77	36757.07	7.70E+01	8.11E-01	-0.6291316	-4.5653409	0.181992404	-4.3833485	0.81	-0.63	-4.57	0.18	-4.38	36757.1	TRUE	TRUE	TRUE	TRUE	TRUE	TRUE
1	NKVTLADAG	806	814	9	HLA-A*02:01	806	88	39965.68	8.80E+01	7.95E-01	-0.5794391	-4.6016872	0.215362898	-4.3863243	0.79	-0.58	-4.6	0.22	-4.39	39965.7	TRUE	TRUE	TRUE	TRUE	TRUE	TRUE
1	SVRDPKTSE	561	569	9	HLA-A*02:01	561	95	42281.92	9.50E+01	8.74E-01	-0.6344866	-4.6261547	0.239494417	-4.3866603	0.87	-0.63	-4.63	0.24	-4.39	42281.9	TRUE	TRUE	TRUE	TRUE	TRUE	TRUE
1	VCGPKLSTD	510	518	9	HLA-A*02:01	510	84	38796.97	8.40E+01	1.01E+00	-0.8198922	-4.5887978	0.193224789	-4.395573	1.01	-0.82	-4.59	0.19	-4.4	38797	TRUE	TRUE	TRUE	TRUE	TRUE	TRUE
1	VDCNMYICG	718	726	9	HLA-A*02:01	718	71	35133.21	7.10E+01	8.33E-01	-0.6849467	-4.5457178	0.148375301	-4.3973425	0.83	-0.68	-4.55	0.15	-4.4	35133.2	TRUE	TRUE	TRUE	TRUE	TRUE	TRUE
1	VSMAKTSVD	711	719	9	HLA-A*02:01	711	93	41690.02	9.30E+01	1.00E+00	-0.7795218	-4.6200321	0.222233157	-4.3977989	1	-0.78	-4.62	0.22	-4.4	41690	TRUE	TRUE	TRUE	TRUE	TRUE	TRUE
1	DGIYFAATE	85	93	9	HLA-A*02:01	85	71	35226.09	7.10E+01	1.01E+00	-0.8574768	-4.5468644	0.148697211	-4.3981672	1.01	-0.86	-4.55	0.15	-4.4	35226.1	TRUE	TRUE	TRUE	TRUE	TRUE	TRUE
1	GIAAEQDRN	751	759	9	HLA-A*02:01	751	96	42900.81	9.60E+01	7.88E-01	-0.5609308	-4.6324655	0.227207198	-4.4052583	0.79	-0.56	-4.63	0.23	-4.41	42900.8	TRUE	TRUE	TRUE	TRUE	TRUE	TRUE
1	HTINHTFGN	70	78	9	HLA-A*02:01	70	63	32735.88	6.30E+01	7.42E-01	-0.6336856	-4.515024	0.108641375	-4.4063826	0.74	-0.63	-4.52	0.11	-4.41	32735.9	TRUE	TRUE	TRUE	TRUE	TRUE	TRUE
1	QTGVIADYN	401	409	9	HLA-A*02:01	401	66	33446.57	6.60E+01	7.72E-01	-0.6568501	-4.5243516	0.115567906	-4.4087837	0.77	-0.66	-4.52	0.12	-4.41	33446.6	TRUE	TRUE	TRUE	TRUE	TRUE	TRUE
1	AEHVDTSYE	639	647	9	HLA-A*02:01	639	87	39550.99	8.70E+01	8.63E-01	-0.6746766	-4.5971574	0.187932433	-4.4092249	0.86	-0.67	-4.6	0.19	-4.41	39551	TRUE	TRUE	TRUE	TRUE	TRUE	TRUE
1	REGVFVFNG	1073	1081	9	HLA-A*02:01	1073	44	25500.48	4.40E+01	6.89E-01	-0.691933	-4.4065484	-0.003206	-4.4097543	0.69	-0.69	-4.41	0	-4.41	25500.5	TRUE	TRUE	TRUE	TRUE	TRUE	TRUE
1	NESLIDLQE	1176	1184	9	HLA-A*02:01	1176	67	34027.3	6.70E+01	8.24E-01	-0.7017014	-4.5318275	0.121822568	-4.4100049	0.82	-0.7	-4.53	0.12	-4.41	34027.3	TRUE	TRUE	TRUE	TRUE	TRUE	TRUE
1	VNQNAQALN	934	942	9	HLA-A*02:01	934	97	43440.3	9.70E+01	8.29E-01	-0.6021162	-4.6378928	0.227007846	-4.410885	0.83	-0.6	-4.64	0.23	-4.41	43440.3	TRUE	TRUE	TRUE	TRUE	TRUE	TRUE
1	DVVRDLPSG	204	212	9	HLA-A*02:01	204	90	40510.75	9.00E+01	8.40E-01	-0.6448819	-4.6075703	0.194993147	-4.4125771	0.84	-0.64	-4.61	0.19	-4.41	40510.8	TRUE	TRUE	TRUE	TRUE	TRUE	TRUE
1	FNCTFEYIS	157	165	9	HLA-A*02:01	157	44	25384.04	4.40E+01	1.00E+00	-1.0126978	-4.4045607	-0.0080938	-4.4126545	1	-1.01	-4.4	-0.01	-4.41	25384	TRUE	TRUE	TRUE	TRUE	TRUE	TRUE
1	DDVQAPNYT	23	31	9	HLA-A*02:01	23	93	41454.76	9.30E+01	7.94E-01	-0.5895797	-4.6175744	0.204186302	-4.4133881	0.79	-0.59	-4.62	0.2	-4.41	41454.8	TRUE	TRUE	TRUE	TRUE	TRUE	TRUE
1	KFPSVYAWE	333	341	9	HLA-A*02:01	333	63	32617.81	6.30E+01	6.61E-01	-0.5611043	-4.5134548	0.09987073	-4.4135841	0.66	-0.56	-4.51	0.1	-4.41	32617.8	TRUE	TRUE	TRUE	TRUE	TRUE	TRUE
1	QKEIDRLNE	1162	1170	9	HLA-A*02:01	1162	92	41357.99	9.20E+01	9.06E-01	-0.7098789	-4.6165594	0.196050067	-4.4205094	0.91	-0.71	-4.62	0.2	-4.42	41358	TRUE	TRUE	TRUE	TRUE	TRUE	TRUE
1	TITDAVDCS	271	279	9	HLA-A*02:01	271	54	29470.52	5.40E+01	1.01E+00	-0.9588535	-4.4693878	0.04689345	-4.4224943	1.01	-0.96	-4.47	0.05	-4.42	29470.5	TRUE	TRUE	TRUE	TRUE	TRUE	TRUE
1	YGECLGDIN	819	827	9	HLA-A*02:01	819	78	37142.86	7.80E+01	9.24E-01	-0.7776344	-4.5698753	0.146836625	-4.4230387	0.92	-0.78	-4.57	0.15	-4.42	37142.9	TRUE	TRUE	TRUE	TRUE	TRUE	TRUE
1	KNLNESLID	1173	1181	9	HLA-A*02:01	1173	90	40606.43	9.00E+01	9.17E-01	-0.7335845	-4.6085948	0.183769523	-4.4248253	0.92	-0.73	-4.61	0.18	-4.42	40606.4	TRUE	TRUE	TRUE	TRUE	TRUE	TRUE
1	NNVFQTQAG	626	634	9	HLA-A*02:01	626	95	42210.62	9.50E+01	8.35E-01	-0.6344447	-4.6254217	0.200092324	-4.4253294	0.83	-0.63	-4.63	0.2	-4.43	42210.6	TRUE	TRUE	TRUE	TRUE	TRUE	TRUE
1	NTRNIDATS	424	432	9	HLA-A*02:01	424	67	33778.62	6.70E+01	1.07E+00	-0.9727457	-4.5286419	0.102185284	-4.4264566	1.07	-0.97	-4.53	0.1	-4.43	33778.6	TRUE	TRUE	TRUE	TRUE	TRUE	TRUE
1	RDISNVPFS	453	461	9	HLA-A*02:01	453	71	34970.14	7.10E+01	1.16E+00	-1.0464939	-4.5436974	0.115533114	-4.4281643	1.16	-1.05	-4.54	0.12	-4.43	34970.1	TRUE	TRUE	TRUE	TRUE	TRUE	TRUE
1	DYGFYTTTG	480	488	9	HLA-A*02:01	480	91	40790.5	9.10E+01	8.16E-01	-0.6370659	-4.610559	0.179197075	-4.431362	0.82	-0.64	-4.61	0.18	-4.43	40790.5	TRUE	TRUE	TRUE	TRUE	TRUE	TRUE
1	DGKPCTPPA	463	471	9	HLA-A*02:01	463	81	37845.47	8.10E+01	6.90E-01	-0.5485322	-4.5780139	0.141006836	-4.4370071	0.69	-0.55	-4.58	0.14	-4.44	37845.5	TRUE	TRUE	TRUE	TRUE	TRUE	TRUE
1	TSPDVDLGD	1142	1150	9	HLA-A*02:01	1142	81	37899.55	8.10E+01	9.39E-01	-0.7971616	-4.5786341	0.14140142	-4.4372326	0.94	-0.8	-4.58	0.14	-4.44	37899.6	TRUE	TRUE	TRUE	TRUE	TRUE	TRUE
1	LNEVAKNLN	1168	1176	9	HLA-A*02:01	1168	96	42959.8	9.60E+01	8.31E-01	-0.638507	-4.6330623	0.192635966	-4.4404263	0.83	-0.64	-4.63	0.19	-4.44	42959.8	TRUE	TRUE	TRUE	TRUE	TRUE	TRUE
1	TFDDVQAPN	21	29	9	HLA-A*02:01	21	78	37104.7	7.80E+01	7.05E-01	-0.5778215	-4.5694289	0.127287485	-4.4421414	0.71	-0.58	-4.57	0.13	-4.44	37104.7	TRUE	TRUE	TRUE	TRUE	TRUE	TRUE
1	AIPTNFSIS	695	703	9	HLA-A*02:01	695	54	29416.37	5.40E+01	8.85E-01	-0.8603762	-4.4685891	0.024347757	-4.4442413	0.88	-0.86	-4.47	0.02	-4.44	29416.4	TRUE	TRUE	TRUE	TRUE	TRUE	TRUE
1	GRDVSDFTD	552	560	9	HLA-A*02:01	552	89	40193.78	8.90E+01	1.06E+00	-0.8990899	-4.6041589	0.158917117	-4.4452417	1.06	-0.9	-4.6	0.16	-4.45	40193.8	TRUE	TRUE	TRUE	TRUE	TRUE	TRUE
1	KSIVAYTMS	672	680	9	HLA-A*02:01	672	48	27234.13	4.80E+01	8.93E-01	-0.9039913	-4.4351135	-0.0111463	-4.4462598	0.89	-0.9	-4.44	-0.01	-4.45	27234.1	TRUE	TRUE	TRUE	TRUE	TRUE	TRUE
1	TLTSGSDLD	9	17	9	HLA-A*02:01	9	70	34918.33	7.00E+01	8.78E-01	-0.784175	-4.5430535	0.09403	-4.4490235	0.88	-0.78	-4.54	0.09	-4.45	34918.3	TRUE	TRUE	TRUE	TRUE	TRUE	TRUE
1	SDTLYLTQD	49	57	9	HLA-A*02:01	49	81	37884.79	8.10E+01	9.86E-01	-0.8578434	-4.5784649	0.127759588	-4.4507053	0.99	-0.86	-4.58	0.13	-4.45	37884.8	TRUE	TRUE	TRUE	TRUE	TRUE	TRUE
1	ACNFELCDN	127	135	9	HLA-A*02:01	127	74	35823	7.40E+01	5.85E-01	-0.4812639	-4.554162	0.103384132	-4.4507778	0.58	-0.48	-4.55	0.1	-4.45	35823	TRUE	TRUE	TRUE	TRUE	TRUE	TRUE
1	GNPVIPFKD	77	85	9	HLA-A*02:01	77	84	38710.6	8.40E+01	1.12E+00	-0.9846473	-4.5878299	0.135576744	-4.4522532	1.12	-0.98	-4.59	0.14	-4.45	38710.6	TRUE	TRUE	TRUE	TRUE	TRUE	TRUE
1	DISPCSFGG	572	580	9	HLA-A*02:01	572	55	29948.49	5.50E+01	6.92E-01	-0.667885	-4.4763749	0.023947	-4.4524279	0.69	-0.67	-4.48	0.02	-4.45	29948.5	TRUE	TRUE	TRUE	TRUE	TRUE	TRUE
1	GADSSIAYS	682	690	9	HLA-A*02:01	682	30	17677.38	3.00E+01	8.82E-01	-1.0871065	-4.2474179	-0.2054505	-4.4528684	0.88	-1.09	-4.25	-0.21	-4.45	17677.4	TRUE	TRUE	TRUE	TRUE	TRUE	TRUE
1	NFRVVPSGD	304	312	9	HLA-A*02:01	304	79	37424.01	7.90E+01	7.75E-01	-0.6550734	-4.5731503	0.120137608	-4.4530127	0.78	-0.66	-4.57	0.12	-4.45	37424	TRUE	TRUE	TRUE	TRUE	TRUE	TRUE

seq #	peptide	start	end	peptide length	allele	peptide index	median binding	ann IC50	ann percentile	proteasome sc	tap score	mhc score	processing scor	processing tota	legacy Proteas	legacy TAP Sco	legacy MHC Sc	legacy Processi	legacy Total Sc	legacy MHC IC5	equal Proteaso	equal TAP Scor	equal MHC Sco	equal Processin	equal Total Sc	equal MHC IC50
1	ISNCVADYS	345	353	9	HLA-A*02:01	345	42	24266.04	4.20E+01	8.73E-01	-0.9461994	-4.3849989	-0.0732244	-4.4582233	0.87	-0.95	-4.38	-0.07	-4.46	24266	TRUE	TRUE	TRUE	TRUE	TRUE	TRUE
1	KPCTPPALN	465	473	9	HLA-A*02:01	465	88	40014.13	8.80E+01	8.05E-01	-0.664377	-4.6022134	0.140881972	-4.4613314	0.81	-0.66	-4.6	0.14	-4.46	40014.1	TRUE	TRUE	TRUE	TRUE	TRUE	TRUE
1	EFVFKNKDG	184	192	9	HLA-A*02:01	184	100	44753.7	1.00E+02	7.10E-01	-0.5300328	-4.6508289	0.179731226	-4.4710977	0.71	-0.53	-4.65	0.18	-4.47	44753.7	TRUE	TRUE	TRUE	TRUE	TRUE	TRUE
1	TQQLIRAAE	991	999	9	HLA-A*02:01	991	59	31400.25	5.90E+01	6.74E-01	-0.6487446	-4.4969331	0.025599433	-4.4713337	0.67	-0.65	-4.5	0.03	-4.47	31400.2	TRUE	TRUE	TRUE	TRUE	TRUE	TRUE
1	KPIFKLPLG	217	225	9	HLA-A*02:01	217	57	30437.87	5.70E+01	6.49E-01	-0.6401723	-4.4834143	0.00930975	-4.4741045	0.65	-0.64	-4.48	0.01	-4.47	30437.9	TRUE	TRUE	TRUE	TRUE	TRUE	TRUE
1	DRLNEVAKN	1166	1174	9	HLA-A*02:01	1166	98	44014.65	9.80E+01	7.75E-01	-0.6050882	-4.6435973	0.169468841	-4.4741284	0.77	-0.61	-4.64	0.17	-4.47	44014.7	TRUE	TRUE	TRUE	TRUE	TRUE	TRUE
1	CSCLKGACS	1223	1231	9	HLA-A*02:01	1223	60	31784.79	6.00E+01	1.00E+00	-0.9764106	-4.5022193	0.026978425	-4.4752409	1	-0.98	-4.5	0.03	-4.48	31784.8	TRUE	TRUE	TRUE	TRUE	TRUE	TRUE
1	AELKCSVKS	284	292	9	HLA-A*02:01	284	74	36000.57	7.40E+01	1.02E+00	-0.9438467	-4.5563094	0.08028127	-4.4760281	1.02	-0.94	-4.56	0.08	-4.48	36000.6	TRUE	TRUE	TRUE	TRUE	TRUE	TRUE
1	GAISSVLND	953	961	9	HLA-A*02:01	953	65	33184.52	6.50E+01	9.03E-01	-0.8594065	-4.5209355	0.04369449	-4.4772411	0.9	-0.86	-4.52	0.04	-4.48	33184.5	TRUE	TRUE	TRUE	TRUE	TRUE	TRUE
1	DNAFNCTFE	154	162	9	HLA-A*02:01	154	87	39602.37	8.70E+01	1.04E+00	-0.9196758	-4.5977212	0.119309248	-4.4784119	1.04	-0.92	-4.6	0.12	-4.48	39602.4	TRUE	TRUE	TRUE	TRUE	TRUE	TRUE
1	YHTVSLLRS	660	668	9	HLA-A*02:01	660	48	27108	4.80E+01	9.27E-01	-0.9744672	-4.4330975	-0.0472202	-4.4803177	0.93	-0.97	-4.43	-0.05	-4.48	27108	TRUE	TRUE	TRUE	TRUE	TRUE	TRUE
1	TTFMLKYDE	260	268	9	HLA-A*02:01	260	62	32422.52	6.20E+01	7.40E-01	-0.7112358	-4.5108468	0.029022214	-4.4818246	0.74	-0.71	-4.51	0.03	-4.48	32422.5	TRUE	TRUE	TRUE	TRUE	TRUE	TRUE
1	KNHTSPDVD	1139	1147	9	HLA-A*02:01	1139	99	44272.57	9.90E+01	1.00E+00	-0.8411515	-4.6461347	0.163582465	-4.4825523	1	-0.84	-4.65	0.16	-4.48	44272.6	TRUE	TRUE	TRUE	TRUE	TRUE	TRUE
1	LLRSTSQKS	665	673	9	HLA-A*02:01	665	47	26617.12	4.70E+01	8.64E-01	-0.9214184	-4.4251611	-0.0574784	-4.4826394	0.86	-0.92	-4.43	-0.06	-4.48	26617.1	TRUE	TRUE	TRUE	TRUE	TRUE	TRUE
1	KTSEILDIS	566	574	9	HLA-A*02:01	566	30	17276.51	3.00E+01	7.59E-01	-1.0044297	-4.237456	-0.2453237	-4.4827798	0.76	-1	-4.24	-0.25	-4.48	17276.5	TRUE	TRUE	TRUE	TRUE	TRUE	TRUE
1	SDFTDSVRD	556	564	9	HLA-A*02:01	556	90	40603.78	9.00E+01	9.56E-01	-0.8306371	-4.6085665	0.125235873	-4.4833306	0.96	-0.83	-4.61	0.13	-4.48	40603.8	TRUE	TRUE	TRUE	TRUE	TRUE	TRUE
1	CGDSTECAN	725	733	9	HLA-A*02:01	725	88	39969.15	8.80E+01	8.93E-01	-0.7754784	-4.6017249	0.11702662	-4.4846983	0.89	-0.78	-4.6	0.12	-4.48	39969.2	TRUE	TRUE	TRUE	TRUE	TRUE	TRUE
1	DVSTAIHAD	605	613	9	HLA-A*02:01	605	89	40090.41	8.90E+01	9.82E-01	-0.8637006	-4.6030405	0.11785936	-4.4851811	0.98	-0.86	-4.6	0.12	-4.49	40090.4	TRUE	TRUE	TRUE	TRUE	TRUE	TRUE
1	ITPGTNASS	584	592	9	HLA-A*02:01	584	50	28153.33	5.00E+01	9.25E-01	-0.9620587	-4.4495298	-0.0368007	-4.4863305	0.93	-0.96	-4.45	-0.04	-4.49	28153.3	TRUE	TRUE	TRUE	TRUE	TRUE	TRUE
1	PGQTGVIAD	399	407	9	HLA-A*02:01	399	91	40853.65	9.10E+01	1.18E+00	-1.0603038	-4.6112309	0.124556199	-4.4866747	1.18	-1.06	-4.61	0.12	-4.49	40853.7	TRUE	TRUE	TRUE	TRUE	TRUE	TRUE
1	SGNFKHLRE	176	184	9	HLA-A*02:01	176	89	40201.17	8.90E+01	8.84E-01	-0.7682084	-4.6042387	0.115322635	-4.4889161	0.88	-0.77	-4.6	0.12	-4.49	40201.2	TRUE	TRUE	TRUE	TRUE	TRUE	TRUE
1	DKYFKNHTS	1135	1143	9	HLA-A*02:01	1135	96	42652.28	9.60E+01	1.13E+00	-0.9902115	-4.6299423	0.140626465	-4.4893158	1.13	-0.99	-4.63	0.14	-4.49	42652.3	TRUE	TRUE	TRUE	TRUE	TRUE	TRUE
1	QAGCLIGAE	632	640	9	HLA-A*02:01	632	67	33915.94	6.70E+01	7.57E-01	-0.7163918	-4.5304039	0.0406242	-4.4897797	0.76	-0.72	-4.53	0.04	-4.49	33915.9	TRUE	TRUE	TRUE	TRUE	TRUE	TRUE
1	CTQLNRALS	742	750	9	HLA-A*02:01	742	58	30813.62	5.80E+01	1.06E+00	-1.0634163	-4.4887427	-0.0024843	-4.4912271	1.06	-1.06	-4.49	0	-4.49	30813.6	TRUE	TRUE	TRUE	TRUE	TRUE	TRUE
1	PAQDIWGTS	240	248	9	HLA-A*02:01	240	87	39609.68	8.70E+01	1.26E+00	-1.1529965	-4.5978013	0.102805508	-4.4949958	1.26	-1.15	-4.6	0.1	-4.49	39609.7	TRUE	TRUE	TRUE	TRUE	TRUE	TRUE
1	YDPLQPELD	1120	1128	9	HLA-A*02:01	1120	85	38947.56	8.50E+01	1.01E+00	-0.9170855	-4.5904803	0.08925452	-4.5012257	1.01	-0.92	-4.59	0.09	-4.5	38947.6	TRUE	TRUE	TRUE	TRUE	TRUE	TRUE
1	LKCSVKSFE	286	294	9	HLA-A*02:01	286	89	40361.14	8.90E+01	8.27E-01	-0.7237701	-4.6059634	0.103132937	-4.5028305	0.83	-0.72	-4.61	0.1	-4.5	40361.1	TRUE	TRUE	TRUE	TRUE	TRUE	TRUE
1	PDGKPCTPP	462	470	9	HLA-A*02:01	462	95	42198.75	9.50E+01	4.68E-01	-0.3455542	-4.6252996	0.122206787	-4.5030928	0.47	-0.35	-4.63	0.12	-4.5	42198.8	TRUE	TRUE	TRUE	TRUE	TRUE	TRUE
1	DLQELGKYE	1181	1189	9	HLA-A*02:01	1181	62	32334.58	6.20E+01	8.22E-01	-0.8168193	-4.5096672	0.00478775	-4.5048795	0.82	-0.82	-4.51	0	-4.5	32334.6	TRUE	TRUE	TRUE	TRUE	TRUE	TRUE
1	KPTKRSFIE	793	801	9	HLA-A*02:01	793	93	41390.22	9.30E+01	8.69E-01	-0.7587289	-4.6168977	0.110214086	-4.5066836	0.87	-0.76	-4.62	0.11	-4.51	41390.2	TRUE	TRUE	TRUE	TRUE	TRUE	TRUE
1	ANLLLQYGS	732	740	9	HLA-A*02:01	732	57	30491.27	5.70E+01	9.52E-01	-0.9748673	-4.4841755	-0.0225783	-4.5067539	0.95	-0.97	-4.48	-0.02	-4.51	30491.3	TRUE	TRUE	TRUE	TRUE	TRUE	TRUE
1	LNDILSRLD	959	967	9	HLA-A*02:01	959	75	36107.46	7.50E+01	9.60E-01	-0.9089418	-4.5575969	0.05079424	-4.5068027	0.96	-0.91	-4.56	0.05	-4.51	36107.5	TRUE	TRUE	TRUE	TRUE	TRUE	TRUE
1	TPTLKYFGG	773	781	9	HLA-A*02:01	773	78	36921.68	7.80E+01	7.71E-01	-0.718753	-4.5672815	0.05240599	-4.5148755	0.77	-0.72	-4.57	0.05	-4.51	36921.7	TRUE	TRUE	TRUE	TRUE	TRUE	TRUE
1	STMNNKSQS	105	113	9	HLA-A*02:01	105	76	36444.59	7.60E+01	9.95E-01	-0.9481399	-4.5616331	0.04654011	-4.515093	0.99	-0.95	-4.56	0.05	-4.52	36444.6	TRUE	TRUE	TRUE	TRUE	TRUE	TRUE
1	DVDLGDISG	1145	1153	9	HLA-A*02:01	1145	71	35003.05	7.10E+01	8.51E-01	-0.8219463	-4.5441059	0.028805744	-4.5153001	0.85	-0.82	-4.54	0.03	-4.52	35003.1	TRUE	TRUE	TRUE	TRUE	TRUE	TRUE
1	ANLAATKMS	1004	1012	9	HLA-A*02:01	1004	89	40136.84	8.90E+01	1.04E+00	-0.9484996	-4.6035432	0.08819641	-4.5153468	1.04	-0.95	-4.6	0.09	-4.52	40136.8	TRUE	TRUE	TRUE	TRUE	TRUE	TRUE
1	HVDTSYECD	641	649	9	HLA-A*02:01	641	89	40344.98	8.90E+01	1.01E+00	-0.917364	-4.6057895	0.08917496	-4.5166145	1.01	-0.92	-4.61	0.09	-4.52	40345	TRUE	TRUE	TRUE	TRUE	TRUE	TRUE
1	VKSFEIDKG	290	298	9	HLA-A*02:01	290	88	39790.07	8.80E+01	6.16E-01	-0.535594	-4.5997747	0.08033598	-4.5194387	0.62	-0.54	-4.6	0.08	-4.52	39790.1	TRUE	TRUE	TRUE	TRUE	TRUE	TRUE
1	PELDSFKEE	1125	1133	9	HLA-A*02:01	1125	98	43608.41	9.80E+01	1.08E+00	-0.9590767	-4.6395703	0.119987274	-4.519583	1.08	-0.96	-4.64	0.12	-4.52	43608.4	TRUE	TRUE	TRUE	TRUE	TRUE	TRUE
1	GKLRPFERD	446	454	9	HLA-A*02:01	446	96	42617.22	9.60E+01	9.57E-01	-0.8475518	-4.6295851	0.109333195	-4.5202519	0.96	-0.85	-4.63	0.11	-4.52	42617.2	TRUE	TRUE	TRUE	TRUE	TRUE	TRUE
1	LFLTLTSGS	6	14	9	HLA-A*02:01	6	56	30240.25	5.60E+01	8.25E-01	-0.8726918	-4.4805854	-0.0479148	-4.5285001	0.82	-0.87	-4.48	-0.05	-4.53	30240.2	TRUE	TRUE	TRUE	TRUE	TRUE	TRUE
1	GKAYFPREG	1067	1075	9	HLA-A*02:01	1067	92	41086.38	9.20E+01	8.12E-01	-0.7274059	-4.6136979	0.08509412	-4.5286038	0.81	-0.73	-4.61	0.09	-4.53	41086.4	TRUE	TRUE	TRUE	TRUE	TRUE	TRUE
1	LRPFERDIS	448	456	9	HLA-A*02:01	448	98	43836.44	9.80E+01	9.56E-01	-0.8475908	-4.6418353	0.108211164	-4.5336241	0.96	-0.85	-4.64	0.11	-4.53	43836.4	TRUE	TRUE	TRUE	TRUE	TRUE	TRUE
1	TDAVDCSQN	273	281	9	HLA-A*02:01	273	97	43062.66	9.70E+01	8.00E-01	-0.700911	-4.6341009	0.09939203	-4.5347088	0.8	-0.7	-4.63	0.1	-4.53	43062.7	TRUE	TRUE	TRUE	TRUE	TRUE	TRUE
1	QPELDSFKE	1124	1132	9	HLA-A*02:01	1124	87	39732.43	8.70E+01	9.10E-01	-0.8464336	-4.5991451	0.06357143	-4.5355737	0.91	-0.85	-4.6	0.06	-4.54	39732.4	TRUE	TRUE	TRUE	TRUE	TRUE	TRUE
1	LTAFSPAQD	235	243	9	HLA-A*02:01	235	84	38654.94	8.40E+01	8.36E-01	-0.7849585	-4.587205	0.05100347	-4.5362015	0.84	-0.78	-4.59	0.05	-4.54	38654.9	TRUE	TRUE	TRUE	TRUE	TRUE	TRUE
1	LNTLVKQLS	941	949	9	HLA-A*02:01	941	73	35561.17	7.30E+01	1.00E+00	-0.9892295	-4.550976	0.014617527	-4.5363585	1	-0.99	-4.55	0.01	-4.54	35561.2	TRUE	TRUE	TRUE	TRUE	TRUE	TRUE
1	LDISPCSFG	571	579	9	HLA-A*02:01	571	76	36413.86	7.60E+01	7.28E-01	-0.7029547	-4.5612667	0.024864326	-4.5364024	0.73	-0.7	-4.56	0.02	-4.54	36413.9	TRUE	TRUE	TRUE	TRUE	TRUE	TRUE
1	DATSTGNYN	429	437	9	HLA-A*02:01	429	97	43200.32	9.70E+01	7.93E-01	-0.6954376	-4.635487	0.09787236	-4.5376146	0.79	-0.7	-4.64	0.1	-4.54	43200.3	TRUE	TRUE	TRUE	TRUE	TRUE	TRUE
1	ADSFVVKGD	384	392	9	HLA-A*02:01	384	81	37934.41	8.10E+01	9.17E-01	-0.8839855	-4.5790333	0.03281549	-4.5462178	0.92	-0.88	-4.58	0.03	-4.55	37934.4	TRUE	TRUE	TRUE	TRUE	TRUE	TRUE
1	YPDEIFRSD	42	50	9	HLA-A*02:01	42	64	32999.4	6.40E+01	9.98E-01	-1.028324	-4.518506	-0.029841	-4.548347	1	-1.03	-4.52	-0.03	-4.55	32999.4	TRUE	TRUE	TRUE	TRUE	TRUE	TRUE
1	MSECVLGQS	1011	1019	9	HLA-A*02:01	1011	44	25473.74	4.40E+01	8.11E-01	-0.9575364	-4.4060927	-0.1461074	-4.5522001	0.81	-0.96	-4.41	-0.15	-4.55	25473.7	TRUE	TRUE	TRUE	TRUE	TRUE	TRUE
1	PTTFMLKYD	259	267	9	HLA-A*02:01	259	87	39744.89	8.70E+01	1.02E+00	-0.9786764	-4.5992813	0.04280662	-4.5564747	1.02	-0.98	-4.6	0.04	-4.56	39744.9	TRUE	TRUE	TRUE	TRUE	TRUE	TRUE
1	IDKGIYQTS	295	303	9	HLA-A*02:01	295	76	36593.56	7.60E+01	1.18E+00	-1.1738272	-4.5634047	0.0047568	-4.5586479	1.18	-1.17	-4.56	0	-4.56	36593.6	TRUE	TRUE	TRUE	TRUE	TRUE	TRUE
1	CDNPFFAVS	133	141	9	HLA-A*02:01	133	53	29209.89	5.30E+01	1.05E+00	-1.1479038	-4.4655299	-0.0936518	-4.5591817	1.05	-1.15	-4.47	-0.09	-4.56	29209.9	TRUE	TRUE	TRUE	TRUE	TRUE	TRUE
1	DLDRCTTFD	15	23	9	HLA-A*02:01	15	64	32877.15	6.40E+01	9.27E-01	-0.970388	-4.5168942	-0.043138	-4.5600322	0.93	-0.97	-4.52	-0.04	-4.56	32877.2	TRUE	TRUE	TRUE	TRUE	TRUE	TRUE
1	EGKAYFPRE	1066	1074	9	HLA-A*02:01	1066	89	40195.52	8.90E+01	9.34E-01	-0.893785	-4.6041777	0.040463	-4.5637146	0.93	-0.89	-4.6	0.04	-4.56	40195.5	TRUE	TRUE	TRUE	TRUE	TRUE	TRUE
1	TSNFRVVPS	302	310	9	HLA-A*02:01	302	47	26719.56	4.70E+01	8.93E-01	-1.0304537	-4.4268293	-0.1377537	-4.564583	0.89	-1.03	-4.43	-0.14	-4.56	26719.6	TRUE	TRUE	TRUE	TRUE	TRUE	TRUE
1	DYNYKLPDD	407	415	9	HLA-A*02:01	407	98	43630.61	9.80E+01	8.57E-01	-0.7849496	-4.6397913	0.07163837	-4.5681529	0.86	-0.78	-4.64	0.07	-4.57	43630.6	TRUE	TRUE	TRUE	TRUE	TRUE	TRUE
1	SPCSFGGVS	574	582	9	HLA-A*02:01	574	78	37109.91	7.80E+01	1.08E+00	-1.0780392	-4.5694899	-0.0020272	-4.5715171	1.08	-1.08	-4.57	0	-4.57	37109.9	TRUE	TRUE	TRUE	TRUE	TRUE	TRUE
1	YFKNHTSPD	1137	1145	9	HLA-A*02:01	1137	97	43067.3	9.70E+01	7.40E-01	-0.6816336	-4.6341476	0.057964353	-4.5761833	0.74	-0.68	-4.63	0.06	-4.58	43067.3	TRUE	TRUE	TRUE	TRUE	TRUE	TRUE
1	IGAEHVDTS	637	645	9	HLA-A*02:01	637	70	34878.31	7.00E+01	1.02E+00	-1.054249	-4.5425554	-0.038874	-4.5814294	1.02	-1.05	-4.54	-0.04	-4.58	34878.3	TRUE	TRUE	TRUE	TRUE	TRUE	TRUE
1	SNVPFSPDG	456	464	9	HLA-A*02:01	456	88	39779.32	8.80E+01	6.27E-01	-0.6109393	-4.5996574	0.015661702	-4.5839957	0.63	-0.61	-4.6	0.02	-4.58	39779.3	TRUE	TRUE	TRUE	TRUE	TRUE	TRUE
1	VKQMYKTPT	767	775	9	HLA-A*02:01	767	80	37668.16	8.00E+01	2.85E-01	-0.2939487	-4.5759744	-0.0087987	-4.5847731	0.29	-0.29	-4.58	-0.01	-4.58	37668.2	TRUE	TRUE	TRUE	TRUE	TRUE	TRUE
1	DKGIYQTSN	296	304	9	HLA-A*02:01	296	100	45031.55	1.00E+02	8.16E-01	-0.7518785	-4.6535169	0.06459748	-4.5889194	0.82	-0.75	-4.65	0.06	-4.59	45031.6	TRUE	TRUE	TRUE	TRUE	TRUE	TRUE
1	QDLFLPFYS	56	64	9	HLA-A*02:01	56	38	22507.74	3.80E+01	8.54E-01	-1.0924391	-4.3523319	-0.2385281	-4.59086	0.85	-1.09	-4.35	-0.24	-4.59	22507.7	TRUE	TRUE	TRUE	TRUE	TRUE	TRUE
1	ICASYHTVS	656	664	9	HLA-A*02:01	656	80	37504.27	8.00E+01	9.45E-01	-0.9638793	-4.5740807	-0.0185873	-4.592668	0.95	-0.96	-4.57	-0.02	-4.59	37504.3	TRUE	TRUE	TRUE	TRUE	TRUE	TRUE
1	NGTITDAVD	269	277	9	HLA-A*02:01	269	94	42077.01	9.40E+01	1.01E+00	-0.9766276	-4.6240449	0.029832391	-4.5942125	1.01	-0.98	-4.62	0.03	-4.59	42077	TRUE	TRUE	TRUE	TRUE	TRUE	TRUE
1	APNYTQHTS	27	35	9	HLA-A*02:01	27	94	41937.92	9.40E+01	1.13E+00	-1.1078724	-4.6226069	0.024759586	-4.5978473	1.13	-1.11	-4.62	0.02	-4.6	41937.9	TRUE	TRUE	TRUE	TRUE	TRUE	TRUE
1	EVAKNLNES	1170	1178	9	HLA-A*02:01	1170	81	37763.23	8.10E+01	9.13E-01	-0.936405	-4.5770691	-0.023086	-4.6001551	0.91	-0.94	-4.58	-0.02	-4.6	37763.2	TRUE	TRUE	TRUE	TRUE	TRUE	TRUE
1	FQQFGRDVS	548	556	9	HLA-A*02:01	548	57	30674.57	5.70E+01	9.45E-01	-1.0619818	-4.4867785	-0.1174438	-4.6042223	0.94	-1.06	-4.49	-0.12	-4.6	30674.6	TRUE	TRUE	TRUE	TRUE	TRUE	TRUE
1	GVSATKLND	368	376	9	HLA-A*02:01	368	88	39892.67	8.80E+01	8.27E-01	-0.8310048	-4.6008931	-0.0040338	-4.6049269	0.83	-0.83	-4.6	0	-4.6	39892.7	TRUE	TRUE	TRUE	TRUE	TRUE	TRUE
1	DAGFMKQYG	812	820	9	HLA-A*02:01	812	94	41792.53	9.40E+01	7.27E-01	-0.7235968	-4.6210987	0.00366923	-4.6174294	0.73	-0.72	-4.62	0	-4.62	41792.5	TRUE	TRUE	TRUE	TRUE	TRUE	TRUE
1	PIGAGICAS	651	659	9	HLA-A*02:01	651	62	32266.78	6.20E+01	1.08E+00	-1.1960262	-4.5087556	-0.1120352	-4.6207908	1.08	-1.2	-4.51	-0.11	-4.62	32266.8	TRUE	TRUE	TRUE	TRUE	TRUE	TRUE
1	SCCKFDEDD	1234	1242	9	HLA-A*02:01	1234	81	37914.31	8.10E+01	7.24E-01	-0.7668133	-4.5788032	-0.0430603	-4.6218635	0.72	-0.77	-4.58	-0.04	-4.62	37914.3	TRUE	TRUE	TRUE	TRUE	TRUE	TRUE
1	GSCCKFDED	1233	1241	9	HLA-A*02:01	1233	88	39782.32	8.80E+01	8.23E-01	-0.8487162	-4.5996901	-0.0255232	-4.6252133	0.82	-0.85	-4.6	-0.03	-4.63	39782.3	TRUE	TRUE	TRUE	TRUE	TRUE	TRUE
1	PTKRSFIED	794	802	9	HLA-A*02:01	794	94	41965.17	9.40E+01	9.57E-01	-0.9639589	-4.622889	-0.0066949	-4.6295839	0.96	-0.96	-4.62	-0.01	-4.63	41965.2	TRUE	TRUE	TRUE	TRUE	TRUE	TRUE
1	PDVDLGDIS	1144	1152	9	HLA-A*02:01	1144	99	44249.11	9.90E+01	1.26E+00	-1.2616359	-4.6459045	0.00141305	-4.6444915	1.26	-1.26	-4.65	0	-4.64	44249.1	TRUE	TRUE	TRUE	TRUE	TRUE	TRUE
1	QDVNCTDVS	599	607	9	HLA-A*02:01	599	93	41627.37	9.30E+01	1.07E+00	-1.0932814	-4.619379	-0.0252404	-4.6446194	1.07	-1.09	-4.62	-0.03	-4.64	41627.4	TRUE	TRUE	TRUE	TRUE	TRUE	TRUE
1	TVSLLRSTS	662	670	9	HLA-A*02:01	662	70	34758.52	7.00E+01	8.23E-01	-0.9375827	-4.5410613	-0.1141277	-4.655189	0.82	-0.94	-4.54	-0.11	-4.66	34758.5	TRUE	TRUE	TRUE	TRUE	TRUE	TRUE
1	LDVSEKSGN	170	178	9	HLA-A*02:01	170	100	45758.45	1.00E+02	6.47E-01	-0.6438991	-4.6604713	0.00273693	-4.6577344	0.65	-0.64	-4.66	0	-4.66	45758.4	TRUE	TRUE	TRUE	TRUE	TRUE	TRUE
1	ANQFNKAIS	906	914	9	HLA-A*02:01	906	95	42237.58	9.50E+01	9.57E-01	-0.9898278	-4.625699	-0.0327558	-4.6584549	0.96	-0.99	-4.63	-0.03	-4.66	42237.6	TRUE	TRUE	TRUE	TRUE	TRUE	TRUE
1	FNFSQILPD	782	790	9	HLA-A*02:01	782	40	23189.76	4.00E+01	5.64E-01	-0.8601665	-4.3652963	-0.2960175	-4.6613138	0.56	-0.86	-4.37	-0.3	-4.66	23189.8	TRUE	TRUE	TRUE	TRUE	TRUE	TRUE
1	SPQIITTDN	1093	1101	9	HLA-A*02:01	1093	80	37627.03	8.00E+01	6.49E-01	-0.7381156	-4.5754999	-0.0891886	-4.6646886	0.65	-0.74	-4.58	-0.09	-4.66	37627	TRUE	TRUE	TRUE	TRUE	TRUE	TRUE
1	CGKGYHLMS	1025	1033	9	HLA-A*02:01	1025	74	35943.73	7.40E+01	1.03E+00	-1.1567792	-4.5556231	-0.1248462	-4.6804694	1.03	-1.16	-4.56	-0.12	-4.68	35943.7	TRUE	TRUE	TRUE	TRUE	TRUE	TRUE
1	MRGVYYPDE	37	45	9	HLA-A*02:01	37	88	39952.27	8.80E+01	5.30E-01	-0.6155836	-4.6015415	-0.0855346	-4.687076	0.53	-0.62	-4.6	-0.09	-4.69	39952.3	TRUE	TRUE	TRUE	TRUE	TRUE	TRUE
1	TPGTNASSE	585	593	9	HLA-A*02:01	585	99	44042.28	9.90E+01	8.43E-01	-0.8863295	-4.6438698	-0.0435445	-4.6874143	0.84	-0.89	-4.64	-0.04	-4.69	44042.3	TRUE	TRUE	TRUE	TRUE	TRUE	TRUE
1	CGSCCKFDE	1232	1240	9	HLA-A*02:01	1232	82	38289.48	8.20E+01	6.63E-01	-0.7905844	-4.5830795	-0.1280594	-4.7111389	0.66	-0.79	-4.58	-0.13	-4.71	38289.5	TRUE	TRUE	TRUE	TRUE	TRUE	TRUE
1	NPVIPFKDG	78	86	9	HLA-A*02:01	78	84	38761.31	8.40E+01	5.75E-01	-0.7005398	-4.5883984	-0.1256618	-4.7140603	0.57	-0.7	-4.59	-0.13	-4.71	38761.3	TRUE	TRUE	TRUE	TRUE	TRUE	TRUE
1	QPFQQFGRD	546	554	9	HLA-A*02:01	546	91	40844.8	9.10E+01	8.07E-01	-0.9121123	-4.6111368	-0.1055963	-4.7167331	0.81	-0.91	-4.61	-0.11	-4.72	40844.8	TRUE	TRUE	TRUE	TRUE	TRUE	TRUE
1	LKGACSCGS	1226	1234	9	HLA-A*02:01	1226	76	36447.37	7.60E+01	8.61E-01	-1.0268952	-4.5616662	-0.1654262	-4.7270923	0.86	-1.03	-4.56	-0.17	-4.73	36447.4	TRUE	TRUE	TRUE	TRUE	TRUE	TRUE
1	ECDIPIGAG	647	655	9	HLA-A*02:01	647	75	36205.64	7.50E+01	6.16E-01	-0.7844092	-4.5587762	-0.1686592	-4.7274354	0.62	-0.78	-4.56	-0.17	-4.73	36205.6	TRUE	TRUE	TRUE	TRUE	TRUE	TRUE
1	GTGVLTPSS	534	542	9	HLA-A*02:01	534	54	29349.91	5.40E+01	8.70E-01	-1.1304205	-4.4676068	-0.2601925	-4.7277993	0.87	-1.13	-4.47	-0.26	-4.73	29349.9	TRUE	TRUE	TRUE	TRUE	TRUE	TRUE
1	LDSFKEELD	1127	1135	9	HLA-A*02:01	1127	96	42578.05	96	0.896014	-0.994974	-4.6291858	-0.09896	-4.7281457	0.9	-0.99	-4.63	-0.1	-4.73	42578.1	TRUE	TRUE	TRUE	TRUE	TRUE	TRUE
1	GDISGINAS	1149	1157	9	HLA-A*02:01	1149	79	37216.88	79	0.978094	-1.1370667	-4.57074	-0.1589727	-4.7297127	0.98	-1.14	-4.57	-0.16	-4.73	37216.9	TRUE	TRUE	TRUE	TRUE	TRUE	TRUE
1	PNYTQHTSS	28	36	9	HLA-A*02:01	28	100	45424.99	100	1.027628	-1.1028126	-4.6572948	-0.0751846	-4.7324794	1.03	-1.1	-4.66	-0.08	-4.73	45425	TRUE	TRUE	TRUE	TRUE	TRUE	TRUE
1	NPLAELKCS	281	289	9	HLA-A*02:01	281	86	39244.93	86	0.962388	-1.1055804	-4.5937836	-0.1431924	-4.7369759	0.96	-1.11	-4.59	-0.14	-4.74	39244.9	TRUE	TRUE	TRUE	TRUE	TRUE	TRUE
1	CFSNVYADS	378	386	9	HLA-A*02:01	378	60	31506.42	60	0.65927	-0.902454	-4.4983991	-0.243184	-4.7415831	0.66	-0.9	-4.5	-0.24	-4.74	31506.4	TRUE	TRUE	TRUE	TRUE	TRUE	TRUE
1	DCSQNPLAE	277	285	9	HLA-A*02:01	277	85	38994.79	85	0.629341	-0.7885135	-4.5910066	-0.1591725	-4.750179	0.63	-0.79	-4.59	-0.16	-4.75	38994.8	TRUE	TRUE	TRUE	TRUE	TRUE	TRUE
1	DPLKPTKRS	790	798	9	HLA-A*02:01	790	100	45117.39	100	1.196053	-1.3009906	-4.654344	-0.1049376	-4.7592816	1.2	-1.3	-4.65	-0.1	-4.76	45117.4	TRUE	TRUE	TRUE	TRUE	TRUE	TRUE
1	SDAFSLDVS	165	173	9	HLA-A*02:01	165	74	36070.75	74	0.853454	-1.0760053	-4.5571552	-0.2225513	-4.7797065	0.85	-1.08	-4.56	-0.22	-4.78	36070.8	TRUE	TRUE	TRUE	TRUE	TRUE	TRUE
1	SGDVVRFPN	310	318	9	HLA-A*02:01	310	61	31978.66	61	0.525246	-0.8017037	-4.5048603	-0.2764577	-4.781318	0.53	-0.8	-4.5	-0.28	-4.78	31978.7	TRUE	TRUE	TRUE	TRUE	TRUE	TRUE
1	GGVSVITPG	579	587	9	HLA-A*02:01	579	48	27304.35	48	0.449767	-0.8110254	-4.4362318	-0.3612584	-4.7974902	0.45	-0.81	-4.44	-0.36	-4.8	27304.3	TRUE	TRUE	TRUE	TRUE	TRUE	TRUE
1	FGSTMNNKS	103	111	9	HLA-A*02:01	103	88	39781.04	88	0.913404	-1.1148549	-4.5996761	-0.2014509	-4.8011271	0.91	-1.11	-4.6	-0.2	-4.8	39781	TRUE	TRUE	TRUE	TRUE	TRUE	TRUE
1	ADYNYKLPD	406	414	9	HLA-A*02:01	406	88	39881.46	88	0.569045	-0.774416	-4.600771	-0.205371	-4.8061421	0.57	-0.77	-4.6	-0.21	-4.81	39881.5	TRUE	TRUE	TRUE	TRUE	TRUE	TRUE
1	DPKTSEILD	564	572	9	HLA-A*02:01	564	97	43461.46	97	0.829363	-1.0017172	-4.6381043	-0.1723542	-4.8104585	0.83	-1	-4.64	-0.17	-4.81	43461.5	TRUE	TRUE	TRUE	TRUE	TRUE	TRUE
1	DDSEPVLKG	1241	1249	9	HLA-A*02:01	1241	87	39693.76	87	0.736419	-0.9507902	-4.5987222	-0.2143712	-4.8130935	0.74	-0.95	-4.6	-0.21	-4.81	39693.8	TRUE	TRUE	TRUE	TRUE	TRUE	TRUE
1	LDRCTTFDD	16	24	9	HLA-A*02:01	16	85	39054.73	85	0.685526	-0.9596668	-4.5916736	-0.2741408	-4.8658144	0.69	-0.96	-4.59	-0.27	-4.87	39054.7	TRUE	TRUE	TRUE	TRUE	TRUE	TRUE
1	PDEIFRSDT	43	51	9	HLA-A*02:01	43	97	43030.04	97	0.498893	-0.7341663	-4.6337717	-0.2352733	-4.8690451	0.5	-0.73	-4.63	-0.24	-4.87	43030	TRUE	TRUE	TRUE	TRUE	TRUE	TRUE
1	DSFVVKGDD	385	393	9	HLA-A*02:01	385	91	40916.46	91	0.488477	-0.7930293	-4.6118981	-0.3045523	-4.9164504	0.49	-0.79	-4.61	-0.3	-4.92	40916.5	TRUE	TRUE	TRUE	TRUE	TRUE	TRUE
1	IDVVRDLPS	203	211	9	HLA-A*02:01	203	64	32888.9	64	0.683371	-1.1295709	-4.5170493	-0.4461999	-4.9632492	0.68	-1.13	-4.52	-0.45	-4.96	32888.9	TRUE	TRUE	TRUE	TRUE	TRUE	TRUE
1	VFNATKFPS	328	336	9	HLA-A*02:01	328	60	31603.74	60	0.525755	-0.9899337	-4.4997385	-0.4641787	-4.9639172	0.53	-0.99	-4.5	-0.46	-4.96	31603.7	TRUE	TRUE	TRUE	TRUE	TRUE	TRUE
1	DDVRQIAPG	392	400	9	HLA-A*02:01	392	95	42151.29	95	0.512151	-0.8556832	-4.6248109	-0.3435322	-4.9683431	0.51	-0.86	-4.62	-0.34	-4.97	42151.3	TRUE	TRUE	TRUE	TRUE	TRUE	TRUE
1	CCKFDEDDS	1235	1243	9	HLA-A*02:01	1235	95	42377.66	95	0.592468	-0.9636018	-4.627137	-0.3711338	-4.9982707	0.59	-0.96	-4.63	-0.37	-5	42377.7	TRUE	TRUE	TRUE	TRUE	TRUE	TRUE
1	ISNVPFSPD	455	463	9	HLA-A*02:01	455	73	35527.32	73	0.400328	-0.8625579	-4.5505624	-0.4622299	-5.0127924	0.4	-0.86	-4.55	-0.46	-5.01	35527.3	TRUE	TRUE	TRUE	TRUE	TRUE	TRUE
1	CNMYICGDS	720	728	9	HLA-A*02:01	720	63	32751.83	63	0.5905	-1.0927773	-4.5152356	-0.5022773	-5.0175129	0.59	-1.09	-4.52	-0.5	-5.02	32751.8	TRUE	TRUE	TRUE	TRUE	TRUE	TRUE
1	RDVSDFTDS	553	561	9	HLA-A*02:01	553	91	40982.93	91	0.558358	-0.9833231	-4.612603	-0.4249651	-5.0375681	0.56	-0.98	-4.61	-0.42	-5.04	40982.9	TRUE	TRUE	TRUE	TRUE	TRUE	TRUE
1	TGTGVLTPS	533	541	9	HLA-A*02:01	533	57	30540.45	57	0.53127	-1.096409	-4.4848754	-0.565139	-5.0500144	0.53	-1.1	-4.48	-0.57	-5.05	30540.5	TRUE	TRUE	TRUE	TRUE	TRUE	TRUE
1	DPLQPELDS	1121	1129	9	HLA-A*02:01	1121	91	40914.69	91	0.601722	-1.2604035	-4.6118793	-0.6586815	-5.2705607	0.6	-1.26	-4.61	-0.66	-5.27	40914.7	TRUE	TRUE	TRUE	TRUE	TRUE	TRUE

seq #	position	amino acid	NetChop Predic	legacy score	scores equal
1	1	M	0.298279	0.298279	TRUE
1	2	F	0.330884	0.330884	TRUE
1	3	I	0.114689	0.114689	TRUE
1	4	F	0.442811	0.442811	TRUE
1	5	L	0.410182	0.410182	TRUE
1	6	L	0.931958	0.931958	TRUE
1	7	F	0.621056	0.621056	TRUE
1	8	L	0.972544	0.972544	TRUE
1	9	T	0.041485	0.041485	TRUE
1	10	L	0.97615	0.97615	TRUE
1	11	T	0.241841	0.241841	TRUE
1	12	S	0.457062	0.457062	TRUE
1	13	G	0.422642	0.422642	TRUE
1	14	S	0.046707	0.046707	TRUE
1	15	D	0.036781	0.036781	TRUE
1	16	L	0.879275	0.879275	TRUE
1	17	D	0.126103	0.126103	TRUE
1	18	R	0.71261	0.71261	TRUE
1	19	C	0.02337	0.02337	TRUE
1	20	T	0.043221	0.043221	TRUE
1	21	T	0.086554	0.086554	TRUE
1	22	F	0.930575	0.930575	TRUE
1	23	D	0.04825	0.04825	TRUE
1	24	D	0.028366	0.028366	TRUE
1	25	V	0.590699	0.590699	TRUE
1	26	Q	0.027362	0.027362	TRUE
1	27	A	0.169519	0.169519	TRUE
1	28	P	0.140523	0.140523	TRUE
1	29	N	0.123554	0.123554	TRUE
1	30	Y	0.694831	0.694831	TRUE
1	31	T	0.050037	0.050037	TRUE
1	32	Q	0.614091	0.614091	TRUE
1	33	H	0.418012	0.418012	TRUE
1	34	T	0.155867	0.155867	TRUE
1	35	S	0.265637	0.265637	TRUE
1	36	S	0.048827	0.048827	TRUE
1	37	M	0.940259	0.940259	TRUE
1	38	R	0.241518	0.241518	TRUE
1	39	G	0.047992	0.047992	TRUE
1	40	V	0.109214	0.109214	TRUE
1	41	Y	0.57864	0.57864	TRUE
1	42	Y	0.247408	0.247408	TRUE
1	43	P	0.03909	0.03909	TRUE
1	44	D	0.120699	0.120699	TRUE

seq #	position	amino acid	NetChop Predic	legacy score	scores equal
1	45	E	0.039457	0.039457	TRUE
1	46	I	0.240977	0.240977	TRUE
1	47	F	0.59066	0.59066	TRUE
1	48	R	0.401686	0.401686	TRUE
1	49	S	0.205822	0.205822	TRUE
1	50	D	0.327179	0.327179	TRUE
1	51	T	0.024547	0.024547	TRUE
1	52	L	0.954987	0.954987	TRUE
1	53	Y	0.961645	0.961645	TRUE
1	54	L	0.90775	0.90775	TRUE
1	55	T	0.122765	0.122765	TRUE
1	56	Q	0.212951	0.212951	TRUE
1	57	D	0.055997	0.055997	TRUE
1	58	L	0.730354	0.730354	TRUE
1	59	F	0.195046	0.195046	TRUE
1	60	L	0.536814	0.536814	TRUE
1	61	P	0.043812	0.043812	TRUE
1	62	F	0.610865	0.610865	TRUE
1	63	Y	0.812533	0.812533	TRUE
1	64	S	0.035535	0.035535	TRUE
1	65	N	0.188157	0.188157	TRUE
1	66	V	0.64158	0.64158	TRUE
1	67	T	0.292719	0.292719	TRUE
1	68	G	0.306453	0.306453	TRUE
1	69	F	0.458486	0.458486	TRUE
1	70	H	0.241619	0.241619	TRUE
1	71	T	0.03629	0.03629	TRUE
1	72	I	0.955396	0.955396	TRUE
1	73	N	0.222865	0.222865	TRUE
1	74	H	0.075778	0.075778	TRUE
1	75	T	0.132782	0.132782	TRUE
1	76	F	0.952139	0.952139	TRUE
1	77	G	0.028787	0.028787	TRUE
1	78	N	0.024169	0.024169	TRUE
1	79	P	0.028202	0.028202	TRUE
1	80	V	0.366345	0.366345	TRUE
1	81	I	0.557503	0.557503	TRUE
1	82	P	0.060982	0.060982	TRUE
1	83	F	0.444012	0.444012	TRUE
1	84	K	0.228498	0.228498	TRUE
1	85	D	0.02951	0.02951	TRUE
1	86	G	0.046407	0.046407	TRUE
1	87	I	0.266909	0.266909	TRUE
1	88	Y	0.897143	0.897143	TRUE

seq #	position	amino acid	NetChop Predic	legacy score	scores equal
1	89	F	0.974964	0.974964	TRUE
1	90	A	0.688128	0.688128	TRUE
1	91	A	0.214246	0.214246	TRUE
1	92	T	0.045014	0.045014	TRUE
1	93	E	0.02331	0.02331	TRUE
1	94	K	0.898666	0.898666	TRUE
1	95	S	0.056475	0.056475	TRUE
1	96	N	0.022442	0.022442	TRUE
1	97	V	0.084067	0.084067	TRUE
1	98	V	0.934328	0.934328	TRUE
1	99	R	0.155471	0.155471	TRUE
1	100	G	0.039359	0.039359	TRUE
1	101	W	0.439895	0.439895	TRUE
1	102	V	0.111968	0.111968	TRUE
1	103	F	0.526021	0.526021	TRUE
1	104	G	0.026782	0.026782	TRUE
1	105	S	0.053796	0.053796	TRUE
1	106	T	0.18755	0.18755	TRUE
1	107	M	0.960354	0.960354	TRUE
1	108	N	0.120122	0.120122	TRUE
1	109	N	0.044832	0.044832	TRUE
1	110	K	0.159299	0.159299	TRUE
1	111	S	0.026398	0.026398	TRUE
1	112	Q	0.02401	0.02401	TRUE
1	113	S	0.023503	0.023503	TRUE
1	114	V	0.627336	0.627336	TRUE
1	115	I	0.073603	0.073603	TRUE
1	116	I	0.091071	0.091071	TRUE
1	117	I	0.229186	0.229186	TRUE
1	118	N	0.028382	0.028382	TRUE
1	119	N	0.029111	0.029111	TRUE
1	120	S	0.026213	0.026213	TRUE
1	121	T	0.065161	0.065161	TRUE
1	122	N	0.023567	0.023567	TRUE
1	123	V	0.509248	0.509248	TRUE
1	124	V	0.833598	0.833598	TRUE
1	125	I	0.529456	0.529456	TRUE
1	126	R	0.228765	0.228765	TRUE
1	127	A	0.715995	0.715995	TRUE
1	128	C	0.05255	0.05255	TRUE
1	129	N	0.02768	0.02768	TRUE
1	130	F	0.895396	0.895396	TRUE
1	131	E	0.025198	0.025198	TRUE
1	132	L	0.817254	0.817254	TRUE

seq #	position	amino acid	NetChop Predic	legacy score	scores equal
1	133	C	0.027073	0.027073	TRUE
1	134	D	0.022693	0.022693	TRUE
1	135	N	0.022771	0.022771	TRUE
1	136	P	0.02369	0.02369	TRUE
1	137	F	0.308533	0.308533	TRUE
1	138	F	0.509872	0.509872	TRUE
1	139	A	0.247508	0.247508	TRUE
1	140	V	0.849922	0.849922	TRUE
1	141	S	0.051815	0.051815	TRUE
1	142	K	0.675559	0.675559	TRUE
1	143	P	0.697041	0.697041	TRUE
1	144	M	0.751781	0.751781	TRUE
1	145	G	0.022949	0.022949	TRUE
1	146	T	0.02961	0.02961	TRUE
1	147	Q	0.136275	0.136275	TRUE
1	148	T	0.034222	0.034222	TRUE
1	149	H	0.059441	0.059441	TRUE
1	150	T	0.519607	0.519607	TRUE
1	151	M	0.855392	0.855392	TRUE
1	152	I	0.312052	0.312052	TRUE
1	153	F	0.552876	0.552876	TRUE
1	154	D	0.02296	0.02296	TRUE
1	155	N	0.027825	0.027825	TRUE
1	156	A	0.080007	0.080007	TRUE
1	157	F	0.669097	0.669097	TRUE
1	158	N	0.024506	0.024506	TRUE
1	159	C	0.060218	0.060218	TRUE
1	160	T	0.051831	0.051831	TRUE
1	161	F	0.714746	0.714746	TRUE
1	162	E	0.025004	0.025004	TRUE
1	163	Y	0.950771	0.950771	TRUE
1	164	I	0.277502	0.277502	TRUE
1	165	S	0.024333	0.024333	TRUE
1	166	D	0.033505	0.033505	TRUE
1	167	A	0.680183	0.680183	TRUE
1	168	F	0.424088	0.424088	TRUE
1	169	S	0.02449	0.02449	TRUE
1	170	L	0.973902	0.973902	TRUE
1	171	D	0.067756	0.067756	TRUE
1	172	V	0.9316	0.9316	TRUE
1	173	S	0.027624	0.027624	TRUE
1	174	E	0.03023	0.03023	TRUE
1	175	K	0.902816	0.902816	TRUE
1	176	S	0.044918	0.044918	TRUE

seq #	position	amino acid	NetChop Predic	legacy score	scores equal
1	177	G	0.116457	0.116457	TRUE
1	178	N	0.025271	0.025271	TRUE
1	179	F	0.669527	0.669527	TRUE
1	180	K	0.15922	0.15922	TRUE
1	181	H	0.250362	0.250362	TRUE
1	182	L	0.850045	0.850045	TRUE
1	183	R	0.221547	0.221547	TRUE
1	184	E	0.029922	0.029922	TRUE
1	185	F	0.376835	0.376835	TRUE
1	186	V	0.094246	0.094246	TRUE
1	187	F	0.550837	0.550837	TRUE
1	188	K	0.711411	0.711411	TRUE
1	189	N	0.066849	0.066849	TRUE
1	190	K	0.5457	0.5457	TRUE
1	191	D	0.037645	0.037645	TRUE
1	192	G	0.023651	0.023651	TRUE
1	193	F	0.098569	0.098569	TRUE
1	194	L	0.441503	0.441503	TRUE
1	195	Y	0.530218	0.530218	TRUE
1	196	V	0.918859	0.918859	TRUE
1	197	Y	0.976524	0.976524	TRUE
1	198	K	0.56138	0.56138	TRUE
1	199	G	0.111051	0.111051	TRUE
1	200	Y	0.28048	0.28048	TRUE
1	201	Q	0.030039	0.030039	TRUE
1	202	P	0.028102	0.028102	TRUE
1	203	I	0.948151	0.948151	TRUE
1	204	D	0.058137	0.058137	TRUE
1	205	V	0.573075	0.573075	TRUE
1	206	V	0.663401	0.663401	TRUE
1	207	R	0.952684	0.952684	TRUE
1	208	D	0.043886	0.043886	TRUE
1	209	L	0.928221	0.928221	TRUE
1	210	P	0.026053	0.026053	TRUE
1	211	S	0.03213	0.03213	TRUE
1	212	G	0.050678	0.050678	TRUE
1	213	F	0.570902	0.570902	TRUE
1	214	N	0.064953	0.064953	TRUE
1	215	T	0.04171	0.04171	TRUE
1	216	L	0.975396	0.975396	TRUE
1	217	K	0.952359	0.952359	TRUE
1	218	P	0.122798	0.122798	TRUE
1	219	I	0.541863	0.541863	TRUE
1	220	F	0.603907	0.603907	TRUE

seq #	position	amino acid	NetChop Predic	legacy score	scores equal
1	221	K	0.09149	0.09149	TRUE
1	222	L	0.943169	0.943169	TRUE
1	223	P	0.03772	0.03772	TRUE
1	224	L	0.468708	0.468708	TRUE
1	225	G	0.030692	0.030692	TRUE
1	226	I	0.363069	0.363069	TRUE
1	227	N	0.025305	0.025305	TRUE
1	228	I	0.069297	0.069297	TRUE
1	229	T	0.526435	0.526435	TRUE
1	230	N	0.179957	0.179957	TRUE
1	231	F	0.812431	0.812431	TRUE
1	232	R	0.233772	0.233772	TRUE
1	233	A	0.554724	0.554724	TRUE
1	234	I	0.113207	0.113207	TRUE
1	235	L	0.880313	0.880313	TRUE
1	236	T	0.123577	0.123577	TRUE
1	237	A	0.470811	0.470811	TRUE
1	238	F	0.858928	0.858928	TRUE
1	239	S	0.027377	0.027377	TRUE
1	240	P	0.045365	0.045365	TRUE
1	241	A	0.196678	0.196678	TRUE
1	242	Q	0.358339	0.358339	TRUE
1	243	D	0.024171	0.024171	TRUE
1	244	I	0.529729	0.529729	TRUE
1	245	W	0.560925	0.560925	TRUE
1	246	G	0.023412	0.023412	TRUE
1	247	T	0.139366	0.139366	TRUE
1	248	S	0.062018	0.062018	TRUE
1	249	A	0.364143	0.364143	TRUE
1	250	A	0.058284	0.058284	TRUE
1	251	A	0.792759	0.792759	TRUE
1	252	Y	0.500223	0.500223	TRUE
1	253	F	0.258039	0.258039	TRUE
1	254	V	0.234938	0.234938	TRUE
1	255	G	0.03359	0.03359	TRUE
1	256	Y	0.92326	0.92326	TRUE
1	257	L	0.506742	0.506742	TRUE
1	258	K	0.566436	0.566436	TRUE
1	259	P	0.0702	0.0702	TRUE
1	260	T	0.036152	0.036152	TRUE
1	261	T	0.025607	0.025607	TRUE
1	262	F	0.957999	0.957999	TRUE
1	263	M	0.959639	0.959639	TRUE
1	264	L	0.978806	0.978806	TRUE

seq #	position	amino acid	NetChop Predic	legacy score	scores equal
1	265	K	0.705928	0.705928	TRUE
1	266	Y	0.974316	0.974316	TRUE
1	267	D	0.028749	0.028749	TRUE
1	268	E	0.023337	0.023337	TRUE
1	269	N	0.023189	0.023189	TRUE
1	270	G	0.027052	0.027052	TRUE
1	271	T	0.027602	0.027602	TRUE
1	272	I	0.521234	0.521234	TRUE
1	273	T	0.515176	0.515176	TRUE
1	274	D	0.294219	0.294219	TRUE
1	275	A	0.807139	0.807139	TRUE
1	276	V	0.76772	0.76772	TRUE
1	277	D	0.031619	0.031619	TRUE
1	278	C	0.030834	0.030834	TRUE
1	279	S	0.034977	0.034977	TRUE
1	280	Q	0.047626	0.047626	TRUE
1	281	N	0.027426	0.027426	TRUE
1	282	P	0.036112	0.036112	TRUE
1	283	L	0.94996	0.94996	TRUE
1	284	A	0.077707	0.077707	TRUE
1	285	E	0.025825	0.025825	TRUE
1	286	L	0.955093	0.955093	TRUE
1	287	K	0.597372	0.597372	TRUE
1	288	C	0.02746	0.02746	TRUE
1	289	S	0.030354	0.030354	TRUE
1	290	V	0.80365	0.80365	TRUE
1	291	K	0.871101	0.871101	TRUE
1	292	S	0.032877	0.032877	TRUE
1	293	F	0.929975	0.929975	TRUE
1	294	E	0.022638	0.022638	TRUE
1	295	I	0.324161	0.324161	TRUE
1	296	D	0.024696	0.024696	TRUE
1	297	K	0.695444	0.695444	TRUE
1	298	G	0.023966	0.023966	TRUE
1	299	I	0.485693	0.485693	TRUE
1	300	Y	0.813724	0.813724	TRUE
1	301	Q	0.041192	0.041192	TRUE
1	302	T	0.276841	0.276841	TRUE
1	303	S	0.08906	0.08906	TRUE
1	304	N	0.060892	0.060892	TRUE
1	305	F	0.517332	0.517332	TRUE
1	306	R	0.526027	0.526027	TRUE
1	307	V	0.858292	0.858292	TRUE
1	308	V	0.135357	0.135357	TRUE

seq #	position	amino acid	NetChop Predic	legacy score	scores equal
1	309	P	0.073471	0.073471	TRUE
1	310	S	0.029979	0.029979	TRUE
1	311	G	0.095444	0.095444	TRUE
1	312	D	0.023383	0.023383	TRUE
1	313	V	0.83755	0.83755	TRUE
1	314	V	0.626508	0.626508	TRUE
1	315	R	0.200367	0.200367	TRUE
1	316	F	0.927429	0.927429	TRUE
1	317	P	0.025395	0.025395	TRUE
1	318	N	0.033767	0.033767	TRUE
1	319	I	0.255958	0.255958	TRUE
1	320	T	0.403684	0.403684	TRUE
1	321	N	0.06777	0.06777	TRUE
1	322	L	0.976336	0.976336	TRUE
1	323	C	0.043462	0.043462	TRUE
1	324	P	0.045318	0.045318	TRUE
1	325	F	0.161583	0.161583	TRUE
1	326	G	0.024743	0.024743	TRUE
1	327	E	0.022608	0.022608	TRUE
1	328	V	0.690091	0.690091	TRUE
1	329	F	0.914123	0.914123	TRUE
1	330	N	0.028159	0.028159	TRUE
1	331	A	0.461187	0.461187	TRUE
1	332	T	0.05843	0.05843	TRUE
1	333	K	0.352542	0.352542	TRUE
1	334	F	0.622537	0.622537	TRUE
1	335	P	0.025711	0.025711	TRUE
1	336	S	0.023055	0.023055	TRUE
1	337	V	0.526172	0.526172	TRUE
1	338	Y	0.95409	0.95409	TRUE
1	339	A	0.718438	0.718438	TRUE
1	340	W	0.970353	0.970353	TRUE
1	341	E	0.023981	0.023981	TRUE
1	342	R	0.68481	0.68481	TRUE
1	343	K	0.171851	0.171851	TRUE
1	344	K	0.432424	0.432424	TRUE
1	345	I	0.359387	0.359387	TRUE
1	346	S	0.050129	0.050129	TRUE
1	347	N	0.02714	0.02714	TRUE
1	348	C	0.02406	0.02406	TRUE
1	349	V	0.88767	0.88767	TRUE
1	350	A	0.085746	0.085746	TRUE
1	351	D	0.027013	0.027013	TRUE
1	352	Y	0.903522	0.903522	TRUE

seq #	position	amino acid	NetChop Predic	legacy score	scores equal
1	353	S	0.023063	0.023063	TRUE
1	354	V	0.18937	0.18937	TRUE
1	355	L	0.227026	0.227026	TRUE
1	356	Y	0.972184	0.972184	TRUE
1	357	N	0.024368	0.024368	TRUE
1	358	S	0.029946	0.029946	TRUE
1	359	T	0.100809	0.100809	TRUE
1	360	F	0.877285	0.877285	TRUE
1	361	F	0.722699	0.722699	TRUE
1	362	S	0.063057	0.063057	TRUE
1	363	T	0.17051	0.17051	TRUE
1	364	F	0.957783	0.957783	TRUE
1	365	K	0.741123	0.741123	TRUE
1	366	C	0.079893	0.079893	TRUE
1	367	Y	0.915784	0.915784	TRUE
1	368	G	0.027617	0.027617	TRUE
1	369	V	0.832312	0.832312	TRUE
1	370	S	0.045343	0.045343	TRUE
1	371	A	0.118323	0.118323	TRUE
1	372	T	0.027034	0.027034	TRUE
1	373	K	0.900998	0.900998	TRUE
1	374	L	0.864084	0.864084	TRUE
1	375	N	0.029447	0.029447	TRUE
1	376	D	0.027773	0.027773	TRUE
1	377	L	0.781806	0.781806	TRUE
1	378	C	0.022939	0.022939	TRUE
1	379	F	0.368403	0.368403	TRUE
1	380	S	0.026191	0.026191	TRUE
1	381	N	0.056863	0.056863	TRUE
1	382	V	0.26046	0.26046	TRUE
1	383	Y	0.891087	0.891087	TRUE
1	384	A	0.09784	0.09784	TRUE
1	385	D	0.034196	0.034196	TRUE
1	386	S	0.045141	0.045141	TRUE
1	387	F	0.193622	0.193622	TRUE
1	388	V	0.062268	0.062268	TRUE
1	389	V	0.959718	0.959718	TRUE
1	390	K	0.94059	0.94059	TRUE
1	391	G	0.292812	0.292812	TRUE
1	392	D	0.050489	0.050489	TRUE
1	393	D	0.025601	0.025601	TRUE
1	394	V	0.130458	0.130458	TRUE
1	395	R	0.658855	0.658855	TRUE
1	396	Q	0.216641	0.216641	TRUE

seq #	position	amino acid	NetChop Predic	legacy score	scores equal
1	397	I	0.44954	0.44954	TRUE
1	398	A	0.125725	0.125725	TRUE
1	399	P	0.025618	0.025618	TRUE
1	400	G	0.077366	0.077366	TRUE
1	401	Q	0.025415	0.025415	TRUE
1	402	T	0.033406	0.033406	TRUE
1	403	G	0.032892	0.032892	TRUE
1	404	V	0.790139	0.790139	TRUE
1	405	I	0.64151	0.64151	TRUE
1	406	A	0.445296	0.445296	TRUE
1	407	D	0.028465	0.028465	TRUE
1	408	Y	0.975887	0.975887	TRUE
1	409	N	0.024589	0.024589	TRUE
1	410	Y	0.950603	0.950603	TRUE
1	411	K	0.692475	0.692475	TRUE
1	412	L	0.952592	0.952592	TRUE
1	413	P	0.023052	0.023052	TRUE
1	414	D	0.032753	0.032753	TRUE
1	415	D	0.022908	0.022908	TRUE
1	416	F	0.173811	0.173811	TRUE
1	417	M	0.179024	0.179024	TRUE
1	418	G	0.037648	0.037648	TRUE
1	419	C	0.071828	0.071828	TRUE
1	420	V	0.87174	0.87174	TRUE
1	421	L	0.325177	0.325177	TRUE
1	422	A	0.239884	0.239884	TRUE
1	423	W	0.629587	0.629587	TRUE
1	424	N	0.0245	0.0245	TRUE
1	425	T	0.02327	0.02327	TRUE
1	426	R	0.407908	0.407908	TRUE
1	427	N	0.036727	0.036727	TRUE
1	428	I	0.765822	0.765822	TRUE
1	429	D	0.09212	0.09212	TRUE
1	430	A	0.072744	0.072744	TRUE
1	431	T	0.024103	0.024103	TRUE
1	432	S	0.034989	0.034989	TRUE
1	433	T	0.032867	0.032867	TRUE
1	434	G	0.037172	0.037172	TRUE
1	435	N	0.025766	0.025766	TRUE
1	436	Y	0.55055	0.55055	TRUE
1	437	N	0.023242	0.023242	TRUE
1	438	Y	0.960883	0.960883	TRUE
1	439	K	0.930985	0.930985	TRUE
1	440	Y	0.970695	0.970695	TRUE

seq #	position	amino acid	NetChop Predic	legacy score	scores equal
1	441	R	0.060704	0.060704	TRUE
1	442	Y	0.966982	0.966982	TRUE
1	443	L	0.620103	0.620103	TRUE
1	444	R	0.612315	0.612315	TRUE
1	445	H	0.10743	0.10743	TRUE
1	446	G	0.117311	0.117311	TRUE
1	447	K	0.463407	0.463407	TRUE
1	448	L	0.976216	0.976216	TRUE
1	449	R	0.260032	0.260032	TRUE
1	450	P	0.162685	0.162685	TRUE
1	451	F	0.930752	0.930752	TRUE
1	452	E	0.022801	0.022801	TRUE
1	453	R	0.579923	0.579923	TRUE
1	454	D	0.042931	0.042931	TRUE
1	455	I	0.818478	0.818478	TRUE
1	456	S	0.030776	0.030776	TRUE
1	457	N	0.309362	0.309362	TRUE
1	458	V	0.954019	0.954019	TRUE
1	459	P	0.288313	0.288313	TRUE
1	460	F	0.97307	0.97307	TRUE
1	461	S	0.025092	0.025092	TRUE
1	462	P	0.711924	0.711924	TRUE
1	463	D	0.103156	0.103156	TRUE
1	464	G	0.254354	0.254354	TRUE
1	465	K	0.55946	0.55946	TRUE
1	466	P	0.53455	0.53455	TRUE
1	467	C	0.024604	0.024604	TRUE
1	468	T	0.029194	0.029194	TRUE
1	469	P	0.258902	0.258902	TRUE
1	470	P	0.044817	0.044817	TRUE
1	471	A	0.284091	0.284091	TRUE
1	472	L	0.78605	0.78605	TRUE
1	473	N	0.17637	0.17637	TRUE
1	474	C	0.027934	0.027934	TRUE
1	475	Y	0.927795	0.927795	TRUE
1	476	W	0.849349	0.849349	TRUE
1	477	P	0.045543	0.045543	TRUE
1	478	L	0.455832	0.455832	TRUE
1	479	N	0.027666	0.027666	TRUE
1	480	D	0.022798	0.022798	TRUE
1	481	Y	0.878771	0.878771	TRUE
1	482	G	0.099431	0.099431	TRUE
1	483	F	0.7302	0.7302	TRUE
1	484	Y	0.922937	0.922937	TRUE

seq #	position	amino acid	NetChop Predic	legacy score	scores equal
1	485	T	0.191482	0.191482	TRUE
1	486	T	0.056504	0.056504	TRUE
1	487	T	0.424839	0.424839	TRUE
1	488	G	0.292035	0.292035	TRUE
1	489	I	0.383624	0.383624	TRUE
1	490	G	0.04091	0.04091	TRUE
1	491	Y	0.725587	0.725587	TRUE
1	492	Q	0.028077	0.028077	TRUE
1	493	P	0.030747	0.030747	TRUE
1	494	Y	0.793641	0.793641	TRUE
1	495	R	0.062749	0.062749	TRUE
1	496	V	0.23712	0.23712	TRUE
1	497	V	0.579547	0.579547	TRUE
1	498	V	0.90967	0.90967	TRUE
1	499	L	0.965873	0.965873	TRUE
1	500	S	0.567005	0.567005	TRUE
1	501	F	0.954144	0.954144	TRUE
1	502	E	0.037025	0.037025	TRUE
1	503	L	0.944547	0.944547	TRUE
1	504	L	0.777858	0.777858	TRUE
1	505	N	0.049561	0.049561	TRUE
1	506	A	0.214618	0.214618	TRUE
1	507	P	0.059194	0.059194	TRUE
1	508	A	0.307513	0.307513	TRUE
1	509	T	0.036431	0.036431	TRUE
1	510	V	0.976864	0.976864	TRUE
1	511	C	0.05408	0.05408	TRUE
1	512	G	0.035651	0.035651	TRUE
1	513	P	0.048091	0.048091	TRUE
1	514	K	0.919262	0.919262	TRUE
1	515	L	0.828358	0.828358	TRUE
1	516	S	0.023896	0.023896	TRUE
1	517	T	0.058876	0.058876	TRUE
1	518	D	0.028528	0.028528	TRUE
1	519	L	0.430414	0.430414	TRUE
1	520	I	0.610574	0.610574	TRUE
1	521	K	0.214085	0.214085	TRUE
1	522	N	0.063282	0.063282	TRUE
1	523	Q	0.051454	0.051454	TRUE
1	524	C	0.026447	0.026447	TRUE
1	525	V	0.364193	0.364193	TRUE
1	526	N	0.031319	0.031319	TRUE
1	527	F	0.905618	0.905618	TRUE
1	528	N	0.024457	0.024457	TRUE

seq #	position	amino acid	NetChop Predic	legacy score	scores equal
1	529	F	0.628572	0.628572	TRUE
1	530	N	0.025701	0.025701	TRUE
1	531	G	0.030044	0.030044	TRUE
1	532	L	0.944746	0.944746	TRUE
1	533	T	0.072811	0.072811	TRUE
1	534	G	0.066785	0.066785	TRUE
1	535	T	0.053856	0.053856	TRUE
1	536	G	0.130306	0.130306	TRUE
1	537	V	0.142428	0.142428	TRUE
1	538	L	0.901788	0.901788	TRUE
1	539	T	0.499404	0.499404	TRUE
1	540	P	0.158185	0.158185	TRUE
1	541	S	0.071489	0.071489	TRUE
1	542	S	0.06724	0.06724	TRUE
1	543	K	0.794428	0.794428	TRUE
1	544	R	0.878876	0.878876	TRUE
1	545	F	0.968579	0.968579	TRUE
1	546	Q	0.023294	0.023294	TRUE
1	547	P	0.100258	0.100258	TRUE
1	548	F	0.889344	0.889344	TRUE
1	549	Q	0.084716	0.084716	TRUE
1	550	Q	0.106743	0.106743	TRUE
1	551	F	0.973297	0.973297	TRUE
1	552	G	0.049227	0.049227	TRUE
1	553	R	0.606064	0.606064	TRUE
1	554	D	0.253257	0.253257	TRUE
1	555	V	0.314875	0.314875	TRUE
1	556	S	0.023162	0.023162	TRUE
1	557	D	0.172477	0.172477	TRUE
1	558	F	0.489672	0.489672	TRUE
1	559	T	0.038578	0.038578	TRUE
1	560	D	0.404181	0.404181	TRUE
1	561	S	0.080225	0.080225	TRUE
1	562	V	0.970205	0.970205	TRUE
1	563	R	0.314918	0.314918	TRUE
1	564	D	0.054671	0.054671	TRUE
1	565	P	0.072717	0.072717	TRUE
1	566	K	0.130178	0.130178	TRUE
1	567	T	0.058179	0.058179	TRUE
1	568	S	0.111461	0.111461	TRUE
1	569	E	0.075691	0.075691	TRUE
1	570	I	0.910032	0.910032	TRUE
1	571	L	0.945552	0.945552	TRUE
1	572	D	0.059687	0.059687	TRUE

seq #	position	amino acid	NetChop Predic	legacy score	scores equal
1	573	I	0.876851	0.876851	TRUE
1	574	S	0.036155	0.036155	TRUE
1	575	P	0.02834	0.02834	TRUE
1	576	C	0.050315	0.050315	TRUE
1	577	S	0.034015	0.034015	TRUE
1	578	F	0.952896	0.952896	TRUE
1	579	G	0.042027	0.042027	TRUE
1	580	G	0.039285	0.039285	TRUE
1	581	V	0.393687	0.393687	TRUE
1	582	S	0.094861	0.094861	TRUE
1	583	V	0.37755	0.37755	TRUE
1	584	I	0.873363	0.873363	TRUE
1	585	T	0.074429	0.074429	TRUE
1	586	P	0.036559	0.036559	TRUE
1	587	G	0.140912	0.140912	TRUE
1	588	T	0.27981	0.27981	TRUE
1	589	N	0.045791	0.045791	TRUE
1	590	A	0.134216	0.134216	TRUE
1	591	S	0.093685	0.093685	TRUE
1	592	S	0.05924	0.05924	TRUE
1	593	E	0.032483	0.032483	TRUE
1	594	V	0.784925	0.784925	TRUE
1	595	A	0.128982	0.128982	TRUE
1	596	V	0.068761	0.068761	TRUE
1	597	L	0.837271	0.837271	TRUE
1	598	Y	0.971939	0.971939	TRUE
1	599	Q	0.044814	0.044814	TRUE
1	600	D	0.058009	0.058009	TRUE
1	601	V	0.802544	0.802544	TRUE
1	602	N	0.033166	0.033166	TRUE
1	603	C	0.023004	0.023004	TRUE
1	604	T	0.095759	0.095759	TRUE
1	605	D	0.130669	0.130669	TRUE
1	606	V	0.827049	0.827049	TRUE
1	607	S	0.024234	0.024234	TRUE
1	608	T	0.092326	0.092326	TRUE
1	609	A	0.230868	0.230868	TRUE
1	610	I	0.922045	0.922045	TRUE
1	611	H	0.087678	0.087678	TRUE
1	612	A	0.13594	0.13594	TRUE
1	613	D	0.115649	0.115649	TRUE
1	614	Q	0.047421	0.047421	TRUE
1	615	L	0.272737	0.272737	TRUE
1	616	T	0.023863	0.023863	TRUE

seq #	position	amino acid	NetChop Predic	legacy score	scores equal
1	617	P	0.044619	0.044619	TRUE
1	618	A	0.1486	0.1486	TRUE
1	619	W	0.900175	0.900175	TRUE
1	620	R	0.061712	0.061712	TRUE
1	621	I	0.555948	0.555948	TRUE
1	622	Y	0.889904	0.889904	TRUE
1	623	S	0.027428	0.027428	TRUE
1	624	T	0.161186	0.161186	TRUE
1	625	G	0.292039	0.292039	TRUE
1	626	N	0.029385	0.029385	TRUE
1	627	N	0.025945	0.025945	TRUE
1	628	V	0.761649	0.761649	TRUE
1	629	F	0.967718	0.967718	TRUE
1	630	Q	0.041237	0.041237	TRUE
1	631	T	0.271776	0.271776	TRUE
1	632	Q	0.53096	0.53096	TRUE
1	633	A	0.299565	0.299565	TRUE
1	634	G	0.041774	0.041774	TRUE
1	635	C	0.03004	0.03004	TRUE
1	636	L	0.284911	0.284911	TRUE
1	637	I	0.434564	0.434564	TRUE
1	638	G	0.045258	0.045258	TRUE
1	639	A	0.074878	0.074878	TRUE
1	640	E	0.024303	0.024303	TRUE
1	641	H	0.036338	0.036338	TRUE
1	642	V	0.614183	0.614183	TRUE
1	643	D	0.054992	0.054992	TRUE
1	644	T	0.055043	0.055043	TRUE
1	645	S	0.053106	0.053106	TRUE
1	646	Y	0.974135	0.974135	TRUE
1	647	E	0.049364	0.049364	TRUE
1	648	C	0.206886	0.206886	TRUE
1	649	D	0.0233	0.0233	TRUE
1	650	I	0.075256	0.075256	TRUE
1	651	P	0.022901	0.022901	TRUE
1	652	I	0.219864	0.219864	TRUE
1	653	G	0.032424	0.032424	TRUE
1	654	A	0.254982	0.254982	TRUE
1	655	G	0.040918	0.040918	TRUE
1	656	I	0.580928	0.580928	TRUE
1	657	C	0.026806	0.026806	TRUE
1	658	A	0.609998	0.609998	TRUE
1	659	S	0.025877	0.025877	TRUE
1	660	Y	0.944756	0.944756	TRUE

seq #	position	amino acid	NetChop Predic	legacy score	scores equal
1	661	H	0.040062	0.040062	TRUE
1	662	T	0.073953	0.073953	TRUE
1	663	V	0.733936	0.733936	TRUE
1	664	S	0.054444	0.054444	TRUE
1	665	L	0.945789	0.945789	TRUE
1	666	L	0.970319	0.970319	TRUE
1	667	R	0.972625	0.972625	TRUE
1	668	S	0.029803	0.029803	TRUE
1	669	T	0.0283	0.0283	TRUE
1	670	S	0.036397	0.036397	TRUE
1	671	Q	0.034305	0.034305	TRUE
1	672	K	0.897781	0.897781	TRUE
1	673	S	0.040035	0.040035	TRUE
1	674	I	0.408338	0.408338	TRUE
1	675	V	0.419109	0.419109	TRUE
1	676	A	0.423187	0.423187	TRUE
1	677	Y	0.963311	0.963311	TRUE
1	678	T	0.072785	0.072785	TRUE
1	679	M	0.972172	0.972172	TRUE
1	680	S	0.051602	0.051602	TRUE
1	681	L	0.974925	0.974925	TRUE
1	682	G	0.037909	0.037909	TRUE
1	683	A	0.132639	0.132639	TRUE
1	684	D	0.023279	0.023279	TRUE
1	685	S	0.024635	0.024635	TRUE
1	686	S	0.029896	0.029896	TRUE
1	687	I	0.772053	0.772053	TRUE
1	688	A	0.170974	0.170974	TRUE
1	689	Y	0.941604	0.941604	TRUE
1	690	S	0.037215	0.037215	TRUE
1	691	N	0.035356	0.035356	TRUE
1	692	N	0.024889	0.024889	TRUE
1	693	T	0.041227	0.041227	TRUE
1	694	I	0.711025	0.711025	TRUE
1	695	A	0.257003	2.57E-01	TRUE
1	696	I	0.603389	6.03E-01	TRUE
1	697	P	0.042974	4.30E-02	TRUE
1	698	T	0.034006	3.40E-02	TRUE
1	699	N	0.030469	3.05E-02	TRUE
1	700	F	0.729809	7.30E-01	TRUE
1	701	S	0.023533	2.35E-02	TRUE
1	702	I	0.950572	9.51E-01	TRUE
1	703	S	0.058093	5.81E-02	TRUE
1	704	I	0.969885	9.70E-01	TRUE

seq #	position	amino acid	NetChop Predic	legacy score	scores equal
1	705	T	0.031262	3.13E-02	TRUE
1	706	T	0.197827	1.98E-01	TRUE
1	707	E	0.026379	2.64E-02	TRUE
1	708	V	0.788997	7.89E-01	TRUE
1	709	M	0.650257	6.50E-01	TRUE
1	710	P	0.024191	2.42E-02	TRUE
1	711	V	0.894645	8.95E-01	TRUE
1	712	S	0.036549	3.65E-02	TRUE
1	713	M	0.701527	7.02E-01	TRUE
1	714	A	0.279285	2.79E-01	TRUE
1	715	K	0.922634	9.23E-01	TRUE
1	716	T	0.088447	8.84E-02	TRUE
1	717	S	0.037791	3.78E-02	TRUE
1	718	V	0.446528	4.47E-01	TRUE
1	719	D	0.027185	2.72E-02	TRUE
1	720	C	0.02949	2.95E-02	TRUE
1	721	N	0.023222	2.32E-02	TRUE
1	722	M	0.868094	8.68E-01	TRUE
1	723	Y	0.954373	9.54E-01	TRUE
1	724	I	0.908371	9.08E-01	TRUE
1	725	C	0.02949	2.95E-02	TRUE
1	726	G	0.026566	2.66E-02	TRUE
1	727	D	0.023588	2.36E-02	TRUE
1	728	S	0.023712	2.37E-02	TRUE
1	729	T	0.034589	3.46E-02	TRUE
1	730	E	0.052655	5.27E-02	TRUE
1	731	C	0.044193	4.42E-02	TRUE
1	732	A	0.164582	1.65E-01	TRUE
1	733	N	0.023876	2.39E-02	TRUE
1	734	L	0.83189	8.32E-01	TRUE
1	735	L	0.438693	4.39E-01	TRUE
1	736	L	0.904153	9.04E-01	TRUE
1	737	Q	0.038279	3.83E-02	TRUE
1	738	Y	0.356686	3.57E-01	TRUE
1	739	G	0.042149	4.21E-02	TRUE
1	740	S	0.030362	3.04E-02	TRUE
1	741	F	0.934105	9.34E-01	TRUE
1	742	C	0.026481	2.65E-02	TRUE
1	743	T	0.131498	1.31E-01	TRUE
1	744	Q	0.408316	4.08E-01	TRUE
1	745	L	0.97369	9.74E-01	TRUE
1	746	N	0.035365	3.54E-02	TRUE
1	747	R	0.819448	8.19E-01	TRUE
1	748	A	0.075418	7.54E-02	TRUE

seq #	position	amino acid	NetChop Predic	legacy score	scores equal
1	749	L	0.103822	1.04E-01	TRUE
1	750	S	0.027961	2.80E-02	TRUE
1	751	G	0.039042	3.90E-02	TRUE
1	752	I	0.861289	8.61E-01	TRUE
1	753	A	0.091589	9.16E-02	TRUE
1	754	A	0.411931	4.12E-01	TRUE
1	755	E	0.040861	4.09E-02	TRUE
1	756	Q	0.526878	5.27E-01	TRUE
1	757	D	0.026529	2.65E-02	TRUE
1	758	R	0.046903	4.69E-02	TRUE
1	759	N	0.022757	2.28E-02	TRUE
1	760	T	0.024059	2.41E-02	TRUE
1	761	R	0.157262	1.57E-01	TRUE
1	762	E	0.033798	3.38E-02	TRUE
1	763	V	0.542081	5.42E-01	TRUE
1	764	F	0.277268	2.77E-01	TRUE
1	765	A	0.124033	1.24E-01	TRUE
1	766	Q	0.026944	2.69E-02	TRUE
1	767	V	0.920954	9.21E-01	TRUE
1	768	K	0.890121	8.90E-01	TRUE
1	769	Q	0.089397	8.94E-02	TRUE
1	770	M	0.816854	8.17E-01	TRUE
1	771	Y	0.804842	8.05E-01	TRUE
1	772	K	0.262501	2.63E-01	TRUE
1	773	T	0.031892	3.19E-02	TRUE
1	774	P	0.02956	2.96E-02	TRUE
1	775	T	0.024987	2.50E-02	TRUE
1	776	L	0.784027	7.84E-01	TRUE
1	777	K	0.897863	8.98E-01	TRUE
1	778	Y	0.976568	9.77E-01	TRUE
1	779	F	0.707914	7.08E-01	TRUE
1	780	G	0.024069	2.41E-02	TRUE
1	781	G	0.14651	1.47E-01	TRUE
1	782	F	0.897725	8.98E-01	TRUE
1	783	N	0.028456	2.85E-02	TRUE
1	784	F	0.844785	8.45E-01	TRUE
1	785	S	0.659551	6.60E-01	TRUE
1	786	Q	0.430723	4.31E-01	TRUE
1	787	I	0.712608	7.13E-01	TRUE
1	788	L	0.906211	9.06E-01	TRUE
1	789	P	0.034428	3.44E-02	TRUE
1	790	D	0.038318	3.83E-02	TRUE
1	791	P	0.027408	2.74E-02	TRUE
1	792	L	0.552918	5.53E-01	TRUE

seq #	position	amino acid	NetChop Predic	legacy score	scores equal
1	793	K	0.224716	2.25E-01	TRUE
1	794	P	0.073894	7.39E-02	TRUE
1	795	T	0.663258	6.63E-01	TRUE
1	796	K	0.95129	9.51E-01	TRUE
1	797	R	0.581704	5.82E-01	TRUE
1	798	S	0.276055	2.76E-01	TRUE
1	799	F	0.764333	7.64E-01	TRUE
1	800	I	0.079442	7.94E-02	TRUE
1	801	E	0.059759	5.98E-02	TRUE
1	802	D	0.030148	3.01E-02	TRUE
1	803	L	0.576903	5.77E-01	TRUE
1	804	L	0.581771	5.82E-01	TRUE
1	805	F	0.593781	5.94E-01	TRUE
1	806	N	0.025937	2.59E-02	TRUE
1	807	K	0.473942	4.74E-01	TRUE
1	808	V	0.944355	9.44E-01	TRUE
1	809	T	0.045954	4.60E-02	TRUE
1	810	L	0.940324	9.40E-01	TRUE
1	811	A	0.814134	8.14E-01	TRUE
1	812	D	0.047203	4.72E-02	TRUE
1	813	A	0.048203	4.82E-02	TRUE
1	814	G	0.023904	2.39E-02	TRUE
1	815	F	0.668321	6.68E-01	TRUE
1	816	M	0.106202	1.06E-01	TRUE
1	817	K	0.953949	9.54E-01	TRUE
1	818	Q	0.480575	4.81E-01	TRUE
1	819	Y	0.953202	9.53E-01	TRUE
1	820	G	0.032301	3.23E-02	TRUE
1	821	E	0.030774	3.08E-02	TRUE
1	822	C	0.024793	2.48E-02	TRUE
1	823	L	0.190746	1.91E-01	TRUE
1	824	G	0.0283	2.83E-02	TRUE
1	825	D	0.034729	3.47E-02	TRUE
1	826	I	0.254506	2.55E-01	TRUE
1	827	N	0.023316	2.33E-02	TRUE
1	828	A	0.088055	8.81E-02	TRUE
1	829	R	0.083107	8.31E-02	TRUE
1	830	D	0.090586	9.06E-02	TRUE
1	831	L	0.426692	4.27E-01	TRUE
1	832	I	0.164412	1.64E-01	TRUE
1	833	C	0.023633	2.36E-02	TRUE
1	834	A	0.056116	5.61E-02	TRUE
1	835	Q	0.077224	7.72E-02	TRUE
1	836	K	0.960105	9.60E-01	TRUE

seq #	position	amino acid	NetChop Predic	legacy score	scores equal
1	837	F	0.585716	5.86E-01	TRUE
1	838	N	0.025846	2.58E-02	TRUE
1	839	G	0.027668	2.77E-02	TRUE
1	840	L	0.36922	3.69E-01	TRUE
1	841	T	0.038104	3.81E-02	TRUE
1	842	V	0.872465	8.72E-01	TRUE
1	843	L	0.934773	9.35E-01	TRUE
1	844	P	0.083586	8.36E-02	TRUE
1	845	P	0.031457	3.15E-02	TRUE
1	846	L	0.789843	7.90E-01	TRUE
1	847	L	0.916968	9.17E-01	TRUE
1	848	T	0.027225	2.72E-02	TRUE
1	849	D	0.03582	3.58E-02	TRUE
1	850	D	0.062073	6.21E-02	TRUE
1	851	M	0.684001	6.84E-01	TRUE
1	852	I	0.435847	4.36E-01	TRUE
1	853	A	0.192659	1.93E-01	TRUE
1	854	A	0.23881	2.39E-01	TRUE
1	855	Y	0.972437	9.72E-01	TRUE
1	856	T	0.030316	3.03E-02	TRUE
1	857	A	0.510478	5.10E-01	TRUE
1	858	A	0.262063	2.62E-01	TRUE
1	859	L	0.776558	7.77E-01	TRUE
1	860	V	0.316902	3.17E-01	TRUE
1	861	S	0.052568	5.26E-02	TRUE
1	862	G	0.06201	6.20E-02	TRUE
1	863	T	0.031701	3.17E-02	TRUE
1	864	A	0.413518	4.14E-01	TRUE
1	865	T	0.044145	4.41E-02	TRUE
1	866	A	0.923514	9.24E-01	TRUE
1	867	G	0.063721	6.37E-02	TRUE
1	868	W	0.739938	7.40E-01	TRUE
1	869	T	0.024875	2.49E-02	TRUE
1	870	F	0.770046	7.70E-01	TRUE
1	871	G	0.06126	6.13E-02	TRUE
1	872	A	0.702723	7.03E-01	TRUE
1	873	G	0.072756	7.28E-02	TRUE
1	874	A	0.266096	2.66E-01	TRUE
1	875	A	0.061859	6.19E-02	TRUE
1	876	L	0.822451	8.22E-01	TRUE
1	877	Q	0.023342	2.33E-02	TRUE
1	878	I	0.136952	1.37E-01	TRUE
1	879	P	0.024595	2.46E-02	TRUE
1	880	F	0.108552	1.09E-01	TRUE

seq #	position	amino acid	NetChop Predic	legacy score	scores equal
1	881	A	0.033441	3.34E-02	TRUE
1	882	M	0.770697	7.71E-01	TRUE
1	883	Q	0.228864	2.29E-01	TRUE
1	884	M	0.881233	8.81E-01	TRUE
1	885	A	0.129659	1.30E-01	TRUE
1	886	Y	0.969849	9.70E-01	TRUE
1	887	R	0.02954	2.95E-02	TRUE
1	888	F	0.197499	1.97E-01	TRUE
1	889	N	0.022408	2.24E-02	TRUE
1	890	G	0.023449	2.34E-02	TRUE
1	891	I	0.141404	1.41E-01	TRUE
1	892	G	0.030843	3.08E-02	TRUE
1	893	V	0.288981	2.89E-01	TRUE
1	894	T	0.518429	5.18E-01	TRUE
1	895	Q	0.106569	1.07E-01	TRUE
1	896	N	0.027635	2.76E-02	TRUE
1	897	V	0.551045	5.51E-01	TRUE
1	898	L	0.954729	9.55E-01	TRUE
1	899	Y	0.93115	9.31E-01	TRUE
1	900	E	0.041446	4.14E-02	TRUE
1	901	N	0.031548	3.15E-02	TRUE
1	902	Q	0.02411	2.41E-02	TRUE
1	903	K	0.55703	5.57E-01	TRUE
1	904	Q	0.028414	2.84E-02	TRUE
1	905	I	0.838986	8.39E-01	TRUE
1	906	A	0.124648	1.25E-01	TRUE
1	907	N	0.029432	2.94E-02	TRUE
1	908	Q	0.042486	4.25E-02	TRUE
1	909	F	0.502933	5.03E-01	TRUE
1	910	N	0.023247	2.32E-02	TRUE
1	911	K	0.853372	8.53E-01	TRUE
1	912	A	0.376269	3.76E-01	TRUE
1	913	I	0.411448	4.11E-01	TRUE
1	914	S	0.027482	2.75E-02	TRUE
1	915	Q	0.053932	5.39E-02	TRUE
1	916	I	0.957298	9.57E-01	TRUE
1	917	Q	0.040057	4.01E-02	TRUE
1	918	E	0.029313	2.93E-02	TRUE
1	919	S	0.056215	5.62E-02	TRUE
1	920	L	0.946281	9.46E-01	TRUE
1	921	T	0.030763	3.08E-02	TRUE
1	922	T	0.069401	6.94E-02	TRUE
1	923	T	0.188388	1.88E-01	TRUE
1	924	S	0.055941	5.59E-02	TRUE

seq #	position	amino acid	NetChop Predic	legacy score	scores equal
1	925	T	0.619699	6.20E-01	TRUE
1	926	A	0.876172	8.76E-01	TRUE
1	927	L	0.975042	9.75E-01	TRUE
1	928	G	0.048388	4.84E-02	TRUE
1	929	K	0.260085	2.60E-01	TRUE
1	930	L	0.824919	8.25E-01	TRUE
1	931	Q	0.024016	2.40E-02	TRUE
1	932	D	0.033321	3.33E-02	TRUE
1	933	V	0.211061	2.11E-01	TRUE
1	934	V	0.333255	3.33E-01	TRUE
1	935	N	0.038346	3.83E-02	TRUE
1	936	Q	0.037196	3.72E-02	TRUE
1	937	N	0.390622	3.91E-01	TRUE
1	938	A	0.167218	1.67E-01	TRUE
1	939	Q	0.02954	2.95E-02	TRUE
1	940	A	0.458651	4.59E-01	TRUE
1	941	L	0.600474	6.00E-01	TRUE
1	942	N	0.029915	2.99E-02	TRUE
1	943	T	0.027802	2.78E-02	TRUE
1	944	L	0.644207	6.44E-01	TRUE
1	945	V	0.243592	2.44E-01	TRUE
1	946	K	0.966362	9.66E-01	TRUE
1	947	Q	0.19586	1.96E-01	TRUE
1	948	L	0.943025	9.43E-01	TRUE
1	949	S	0.02678	2.68E-02	TRUE
1	950	S	0.068149	6.81E-02	TRUE
1	951	N	0.042391	4.24E-02	TRUE
1	952	F	0.228361	2.28E-01	TRUE
1	953	G	0.031411	3.14E-02	TRUE
1	954	A	0.069086	6.91E-02	TRUE
1	955	I	0.803067	8.03E-01	TRUE
1	956	S	0.045024	4.50E-02	TRUE
1	957	S	0.052221	5.22E-02	TRUE
1	958	V	0.904941	9.05E-01	TRUE
1	959	L	0.490709	4.91E-01	TRUE
1	960	N	0.028867	2.89E-02	TRUE
1	961	D	0.028704	2.87E-02	TRUE
1	962	I	0.798926	7.99E-01	TRUE
1	963	L	0.418824	4.19E-01	TRUE
1	964	S	0.05145	5.15E-02	TRUE
1	965	R	0.753344	7.53E-01	TRUE
1	966	L	0.97061	9.71E-01	TRUE
1	967	D	0.026311	2.63E-02	TRUE
1	968	K	0.113252	1.13E-01	TRUE

seq #	position	amino acid	NetChop Predic	legacy score	scores equal
1	969	V	0.447484	4.47E-01	TRUE
1	970	E	0.034525	3.45E-02	TRUE
1	971	A	0.213053	2.13E-01	TRUE
1	972	E	0.029859	2.99E-02	TRUE
1	973	V	0.969105	9.69E-01	TRUE
1	974	Q	0.026643	2.66E-02	TRUE
1	975	I	0.271561	2.72E-01	TRUE
1	976	D	0.024025	2.40E-02	TRUE
1	977	R	0.658108	6.58E-01	TRUE
1	978	L	0.500008	5.00E-01	TRUE
1	979	I	0.416883	4.17E-01	TRUE
1	980	T	0.028135	2.81E-02	TRUE
1	981	G	0.117726	1.18E-01	TRUE
1	982	R	0.663197	6.63E-01	TRUE
1	983	L	0.921652	9.22E-01	TRUE
1	984	Q	0.041021	4.10E-02	TRUE
1	985	S	0.034682	3.47E-02	TRUE
1	986	L	0.891839	8.92E-01	TRUE
1	987	Q	0.02913	2.91E-02	TRUE
1	988	T	0.048198	4.82E-02	TRUE
1	989	Y	0.962117	9.62E-01	TRUE
1	990	V	0.748401	7.48E-01	TRUE
1	991	T	0.075954	7.60E-02	TRUE
1	992	Q	0.09213	9.21E-02	TRUE
1	993	Q	0.238855	2.39E-01	TRUE
1	994	L	0.83313	8.33E-01	TRUE
1	995	I	0.846957	8.47E-01	TRUE
1	996	R	0.481206	4.81E-01	TRUE
1	997	A	0.386661	3.87E-01	TRUE
1	998	A	0.066948	6.69E-02	TRUE
1	999	E	0.023338	2.33E-02	TRUE
1	1000	I	0.768889	7.69E-01	TRUE
1	1001	R	0.141956	1.42E-01	TRUE
1	1002	A	0.11725	1.17E-01	TRUE
1	1003	S	0.024755	2.48E-02	TRUE
1	1004	A	0.471833	4.72E-01	TRUE
1	1005	N	0.023983	2.40E-02	TRUE
1	1006	L	0.964218	9.64E-01	TRUE
1	1007	A	0.121699	1.22E-01	TRUE
1	1008	A	0.113098	1.13E-01	TRUE
1	1009	T	0.039329	3.93E-02	TRUE
1	1010	K	0.696234	6.96E-01	TRUE
1	1011	M	0.959289	9.59E-01	TRUE
1	1012	S	0.038261	3.83E-02	TRUE

seq #	position	amino acid	NetChop Predic	legacy score	scores equal
1	1013	E	0.043905	4.39E-02	TRUE
1	1014	C	0.025332	2.53E-02	TRUE
1	1015	V	0.263389	2.63E-01	TRUE
1	1016	L	0.54987	5.50E-01	TRUE
1	1017	G	0.035102	3.51E-02	TRUE
1	1018	Q	0.052213	5.22E-02	TRUE
1	1019	S	0.041658	4.17E-02	TRUE
1	1020	K	0.419425	4.19E-01	TRUE
1	1021	R	0.072851	7.29E-02	TRUE
1	1022	V	0.925498	9.25E-01	TRUE
1	1023	D	0.028547	2.85E-02	TRUE
1	1024	F	0.290255	2.90E-01	TRUE
1	1025	C	0.022726	2.27E-02	TRUE
1	1026	G	0.033717	3.37E-02	TRUE
1	1027	K	0.46223	4.62E-01	TRUE
1	1028	G	0.280733	2.81E-01	TRUE
1	1029	Y	0.752882	7.53E-01	TRUE
1	1030	H	0.057232	5.72E-02	TRUE
1	1031	L	0.951797	9.52E-01	TRUE
1	1032	M	0.57062	5.71E-01	TRUE
1	1033	S	0.035124	3.51E-02	TRUE
1	1034	F	0.464732	4.65E-01	TRUE
1	1035	P	0.034905	3.49E-02	TRUE
1	1036	Q	0.161931	1.62E-01	TRUE
1	1037	A	0.067245	6.72E-02	TRUE
1	1038	A	0.046386	4.64E-02	TRUE
1	1039	P	0.048829	4.88E-02	TRUE
1	1040	H	0.095767	9.58E-02	TRUE
1	1041	G	0.026512	2.65E-02	TRUE
1	1042	V	0.691118	6.91E-01	TRUE
1	1043	V	0.428755	4.29E-01	TRUE
1	1044	F	0.39586	3.96E-01	TRUE
1	1045	L	0.906343	9.06E-01	TRUE
1	1046	H	0.768182	7.68E-01	TRUE
1	1047	V	0.891233	8.91E-01	TRUE
1	1048	T	0.067518	6.75E-02	TRUE
1	1049	Y	0.975435	9.75E-01	TRUE
1	1050	V	0.831125	8.31E-01	TRUE
1	1051	P	0.118875	1.19E-01	TRUE
1	1052	S	0.103419	1.03E-01	TRUE
1	1053	Q	0.33477	3.35E-01	TRUE
1	1054	E	0.029356	2.94E-02	TRUE
1	1055	R	0.934669	9.35E-01	TRUE
1	1056	N	0.044138	4.41E-02	TRUE

seq #	position	amino acid	NetChop Predic	legacy score	scores equal
1	1057	F	0.266788	2.67E-01	TRUE
1	1058	T	0.033806	3.38E-02	TRUE
1	1059	T	0.125051	1.25E-01	TRUE
1	1060	A	0.396717	3.97E-01	TRUE
1	1061	P	0.586716	5.87E-01	TRUE
1	1062	A	0.717699	7.18E-01	TRUE
1	1063	I	0.363895	3.64E-01	TRUE
1	1064	C	0.029536	2.95E-02	TRUE
1	1065	H	0.114708	1.15E-01	TRUE
1	1066	E	0.029617	2.96E-02	TRUE
1	1067	G	0.135454	1.35E-01	TRUE
1	1068	K	0.858847	8.59E-01	TRUE
1	1069	A	0.100562	1.01E-01	TRUE
1	1070	Y	0.252364	2.52E-01	TRUE
1	1071	F	0.165143	1.65E-01	TRUE
1	1072	P	0.02356	2.36E-02	TRUE
1	1073	R	0.13628	1.36E-01	TRUE
1	1074	E	0.024663	2.47E-02	TRUE
1	1075	G	0.025334	2.53E-02	TRUE
1	1076	V	0.126397	1.26E-01	TRUE
1	1077	F	0.860201	8.60E-01	TRUE
1	1078	V	0.164595	1.65E-01	TRUE
1	1079	F	0.967898	9.68E-01	TRUE
1	1080	N	0.051682	5.17E-02	TRUE
1	1081	G	0.030304	3.03E-02	TRUE
1	1082	T	0.028272	2.83E-02	TRUE
1	1083	S	0.039627	3.96E-02	TRUE
1	1084	W	0.873003	8.73E-01	TRUE
1	1085	F	0.729527	7.30E-01	TRUE
1	1086	I	0.372711	3.73E-01	TRUE
1	1087	T	0.032544	3.25E-02	TRUE
1	1088	Q	0.091586	9.16E-02	TRUE
1	1089	R	0.942443	9.42E-01	TRUE
1	1090	N	0.364672	3.65E-01	TRUE
1	1091	F	0.617906	6.18E-01	TRUE
1	1092	F	0.092193	9.22E-02	TRUE
1	1093	S	0.02257	2.26E-02	TRUE
1	1094	P	0.033203	3.32E-02	TRUE
1	1095	Q	0.065053	6.51E-02	TRUE
1	1096	I	0.885718	8.86E-01	TRUE
1	1097	I	0.284734	2.85E-01	TRUE
1	1098	T	0.030961	3.10E-02	TRUE
1	1099	T	0.454893	4.55E-01	TRUE
1	1100	D	0.098122	9.81E-02	TRUE

seq #	position	amino acid	NetChop Predic	legacy score	scores equal
1	1101	N	0.238006	2.38E-01	TRUE
1	1102	T	0.028133	2.81E-02	TRUE
1	1103	F	0.75359	7.54E-01	TRUE
1	1104	V	0.756614	7.57E-01	TRUE
1	1105	S	0.138216	1.38E-01	TRUE
1	1106	G	0.293253	2.93E-01	TRUE
1	1107	N	0.029876	2.99E-02	TRUE
1	1108	C	0.024077	2.41E-02	TRUE
1	1109	D	0.023624	2.36E-02	TRUE
1	1110	V	0.21595	2.16E-01	TRUE
1	1111	V	0.095821	9.58E-02	TRUE
1	1112	I	0.058928	5.89E-02	TRUE
1	1113	G	0.024097	2.41E-02	TRUE
1	1114	I	0.182334	1.82E-01	TRUE
1	1115	I	0.123132	1.23E-01	TRUE
1	1116	N	0.030843	3.08E-02	TRUE
1	1117	N	0.063526	6.35E-02	TRUE
1	1118	T	0.169801	1.70E-01	TRUE
1	1119	V	0.944194	9.44E-01	TRUE
1	1120	Y	0.948128	9.48E-01	TRUE
1	1121	D	0.162761	1.63E-01	TRUE
1	1122	P	0.046338	4.63E-02	TRUE
1	1123	L	0.839742	8.40E-01	TRUE
1	1124	Q	0.024294	2.43E-02	TRUE
1	1125	P	0.052431	5.24E-02	TRUE
1	1126	E	0.060839	6.08E-02	TRUE
1	1127	L	0.97446	9.74E-01	TRUE
1	1128	D	0.045904	4.59E-02	TRUE
1	1129	S	0.124699	1.25E-01	TRUE
1	1130	F	0.865602	8.66E-01	TRUE
1	1131	K	0.262564	2.63E-01	TRUE
1	1132	E	0.243921	2.44E-01	TRUE
1	1133	E	0.027792	2.78E-02	TRUE
1	1134	L	0.826679	8.27E-01	TRUE
1	1135	D	0.026734	2.67E-02	TRUE
1	1136	K	0.684596	6.85E-01	TRUE
1	1137	Y	0.937522	9.38E-01	TRUE
1	1138	F	0.33136	3.31E-01	TRUE
1	1139	K	0.599992	6.00E-01	TRUE
1	1140	N	0.290353	2.90E-01	TRUE
1	1141	H	0.456622	4.57E-01	TRUE
1	1142	T	0.091321	9.13E-02	TRUE
1	1143	S	0.10854	1.09E-01	TRUE
1	1144	P	0.751396	7.51E-01	TRUE

seq #	position	amino acid	NetChop Predic	legacy score	scores equal
1	1145	D	0.036278	3.63E-02	TRUE
1	1146	V	0.878126	8.78E-01	TRUE
1	1147	D	0.025611	2.56E-02	TRUE
1	1148	L	0.643203	6.43E-01	TRUE
1	1149	G	0.069166	6.92E-02	TRUE
1	1150	D	0.03142	3.14E-02	TRUE
1	1151	I	0.690329	6.90E-01	TRUE
1	1152	S	0.023405	2.34E-02	TRUE
1	1153	G	0.041312	4.13E-02	TRUE
1	1154	I	0.28429	2.84E-01	TRUE
1	1155	N	0.039967	4.00E-02	TRUE
1	1156	A	0.060691	6.07E-02	TRUE
1	1157	S	0.029025	2.90E-02	TRUE
1	1158	V	0.738783	7.39E-01	TRUE
1	1159	V	0.628456	6.28E-01	TRUE
1	1160	N	0.034644	3.46E-02	TRUE
1	1161	I	0.54906	5.49E-01	TRUE
1	1162	Q	0.031321	3.13E-02	TRUE
1	1163	K	0.949286	9.49E-01	TRUE
1	1164	E	0.035373	3.54E-02	TRUE
1	1165	I	0.707235	7.07E-01	TRUE
1	1166	D	0.024403	2.44E-02	TRUE
1	1167	R	0.046798	4.68E-02	TRUE
1	1168	L	0.392924	3.93E-01	TRUE
1	1169	N	0.027963	2.80E-02	TRUE
1	1170	E	0.022482	2.25E-02	TRUE
1	1171	V	0.956933	9.57E-01	TRUE
1	1172	A	0.271839	2.72E-01	TRUE
1	1173	K	0.348497	3.48E-01	TRUE
1	1174	N	0.090368	9.04E-02	TRUE
1	1175	L	0.9655	9.66E-01	TRUE
1	1176	N	0.025771	2.58E-02	TRUE
1	1177	E	0.025344	2.53E-02	TRUE
1	1178	S	0.091616	9.16E-02	TRUE
1	1179	L	0.725554	7.26E-01	TRUE
1	1180	I	0.3904	3.90E-01	TRUE
1	1181	D	0.028871	2.89E-02	TRUE
1	1182	L	0.945978	9.46E-01	TRUE
1	1183	Q	0.028869	2.89E-02	TRUE
1	1184	E	0.032809	3.28E-02	TRUE
1	1185	L	0.712329	7.12E-01	TRUE
1	1186	G	0.029705	2.97E-02	TRUE
1	1187	K	0.523584	5.24E-01	TRUE
1	1188	Y	0.552839	5.53E-01	TRUE

seq #	position	amino acid	NetChop Predic	legacy score	scores equal
1	1189	E	0.025313	2.53E-02	TRUE
1	1190	Q	0.057362	5.74E-02	TRUE
1	1191	Y	0.538812	5.39E-01	TRUE
1	1192	I	0.165847	1.66E-01	TRUE
1	1193	K	0.056961	5.70E-02	TRUE
1	1194	W	0.447654	4.48E-01	TRUE
1	1195	P	0.026251	2.63E-02	TRUE
1	1196	W	0.412339	4.12E-01	TRUE
1	1197	Y	0.206132	2.06E-01	TRUE
1	1198	V	0.829028	8.29E-01	TRUE
1	1199	W	0.88866	8.89E-01	TRUE
1	1200	L	0.81462	8.15E-01	TRUE
1	1201	G	0.072581	7.26E-02	TRUE
1	1202	F	0.863428	8.63E-01	TRUE
1	1203	I	0.055383	5.54E-02	TRUE
1	1204	A	0.038881	3.89E-02	TRUE
1	1205	G	0.107165	1.07E-01	TRUE
1	1206	L	0.262228	2.62E-01	TRUE
1	1207	I	0.161537	1.62E-01	TRUE
1	1208	A	0.027828	2.78E-02	TRUE
1	1209	I	0.090463	9.05E-02	TRUE
1	1210	V	0.214371	2.14E-01	TRUE
1	1211	M	0.125271	1.25E-01	TRUE
1	1212	V	0.472139	4.72E-01	TRUE
1	1213	T	0.070452	7.05E-02	TRUE
1	1214	I	0.77209	7.72E-01	TRUE
1	1215	L	0.866205	8.66E-01	TRUE
1	1216	L	0.934318	9.34E-01	TRUE
1	1217	C	0.045495	4.55E-02	TRUE
1	1218	C	0.046086	4.61E-02	TRUE
1	1219	M	0.407928	4.08E-01	TRUE
1	1220	T	0.098008	9.80E-02	TRUE
1	1221	S	0.156547	1.57E-01	TRUE
1	1222	C	0.079352	0.079352	TRUE
1	1223	C	0.033914	0.033914	TRUE
1	1224	S	0.027149	0.027149	TRUE
1	1225	C	0.025507	0.025507	TRUE
1	1226	L	0.829758	0.829758	TRUE
1	1227	K	0.709149	0.709149	TRUE
1	1228	G	0.059028	0.059028	TRUE
1	1229	A	0.039098	0.039098	TRUE
1	1230	C	0.023379	0.023379	TRUE
1	1231	S	0.025706	0.025706	TRUE
1	1232	C	0.032775	0.032775	TRUE

seq #	position	amino acid	NetChop Predic	legacy score	scores equal
1	1233	G	0.318992	0.318992	TRUE
1	1234	S	0.034369	0.034369	TRUE
1	1235	C	0.034304	0.034304	TRUE
1	1236	C	0.028926	0.028926	TRUE
1	1237	K	0.805768	0.805768	TRUE
1	1238	F	0.921571	0.921571	TRUE
1	1239	D	0.023119	0.023119	TRUE
1	1240	E	0.022167	0.022167	TRUE
1	1241	D	0.025901	0.025901	TRUE
1	1242	D	0.024311	0.024311	TRUE
1	1243	S	0.022436	0.022436	TRUE
1	1244	E	0.023009	0.023009	TRUE
1	1245	P	0.036752	0.036752	TRUE
1	1246	V	0.274641	0.274641	TRUE
1	1247	L	0.950554	0.950554	TRUE
1	1248	K	0.310251	0.310251	TRUE
1	1249	G	0.152658	0.152658	TRUE
1	1250	V	0.623653	0.623653	TRUE
1	1251	K	0.357531	0.357531	TRUE
1	1252	L	0.97679	0.97679	TRUE
1	1253	H	0.386486	0.386486	TRUE
1	1254	Y	0.978325	0.978325	TRUE
1	1255	T	0.118749	0.118749	TRUE

seq #	peptide	start	end	peptide length	allele	peptide index	NetCTL Predict	NetCTL Rescale	NetCTL c termi	NetCTL TAP tra	NetCTL Predict	NetCTL Superty	legacy predicte	legacy rescale_	legacy c_termi	legacy tap_tran	legacy predicti	equal predicted	equal rescale_	equal c_termin	equal tap_tran	equal predictions_score
1	MFIFLLFLT	1	9	9	HLA-A*02:01	1	0.0729	0.1744	0.0118	-0.424	0.1549	A2	0.0729	0.1744	0.0118	-0.424	0.1549	TRUE	TRUE	TRUE	TRUE	TRUE
1	FIFLLFLTL	2	10	9	HLA-A*02:01	2	0.2016	0.4824	0.999	1.136	0.6891	A2	0.2016	0.4824	0.999	1.136	0.6891	TRUE	TRUE	TRUE	TRUE	TRUE
1	IFLLFLTLT	3	11	9	HLA-A*02:01	3	0.0342	0.0819	0.122	-0.602	0.0701	A2	0.0342	0.0819	0.122	-0.602	0.0701	TRUE	TRUE	TRUE	TRUE	TRUE
1	FLLFLTLTS	4	12	9	HLA-A*02:01	4	0.2806	0.6715	0.7072	-2.294	0.6629	A2	0.2806	0.6715	0.7072	-2.294	0.6629	TRUE	TRUE	TRUE	TRUE	TRUE
1	LLFLTLTSG	5	13	9	HLA-A*02:01	5	0.0811	0.1942	0.372	-1.365	0.1817	A2	0.0811	0.1942	0.372	-1.365	0.1817	TRUE	TRUE	TRUE	TRUE	TRUE
1	LFLTLTSGS	6	14	9	HLA-A*02:01	6	0.046	0.1101	0.0208	-2.008	0.0128	A2	0.046	0.1101	0.0208	-2.008	0.0128	TRUE	TRUE	TRUE	TRUE	TRUE
1	FLTLTSGSD	7	15	9	HLA-A*02:01	7	0.0777	0.186	0.0634	-1.879	0.1016	A2	0.0777	0.186	0.0634	-1.879	0.1016	TRUE	TRUE	TRUE	TRUE	TRUE
1	LTLTSGSDL	8	16	9	HLA-A*02:01	8	0.0706	0.1691	0.8173	0.85	0.3341	A2	0.0706	0.1691	0.8173	0.85	0.3341	TRUE	TRUE	TRUE	TRUE	TRUE
1	TLTSGSDLD	9	17	9	HLA-A*02:01	9	0.0735	0.176	0.0778	-1.805	0.0974	A2	0.0735	0.176	0.0778	-1.805	0.0974	TRUE	TRUE	TRUE	TRUE	TRUE
1	LTSGSDLDR	10	18	9	HLA-A*02:01	10	0.0391	0.0936	0.5684	1.473	0.2525	A2	0.0391	0.0936	0.5684	1.473	0.2525	TRUE	TRUE	TRUE	TRUE	TRUE
1	TSGSDLDRC	11	19	9	HLA-A*02:01	11	0.0265	0.0634	0.0096	-0.081	0.0608	A2	0.0265	0.0634	0.0096	-0.081	0.0608	TRUE	TRUE	TRUE	TRUE	TRUE
1	SGSDLDRCT	12	20	9	HLA-A*02:01	12	0.0222	0.0532	0.0183	-0.913	0.0103	A2	0.0222	0.0532	0.0183	-0.913	0.0103	TRUE	TRUE	TRUE	TRUE	TRUE
1	GSDLDRCTT	13	21	9	HLA-A*02:01	13	0.0183	0.0437	0.0319	-0.966	0.0002	A2	0.0183	0.0437	0.0319	-0.966	0.0002	TRUE	TRUE	TRUE	TRUE	TRUE
1	SDLDRCTTF	14	22	9	HLA-A*02:01	14	0.0222	0.0532	0.9803	2.268	0.3136	A2	0.0222	0.0532	0.9803	2.268	0.3136	TRUE	TRUE	TRUE	TRUE	TRUE
1	DLDRCTTFD	15	23	9	HLA-A*02:01	15	0.047	0.1124	0.0129	-2.234	0.0026	A2	0.047	0.1124	0.0129	-2.234	0.0026	TRUE	TRUE	TRUE	TRUE	TRUE
1	LDRCTTFDD	16	24	9	HLA-A*02:01	16	0.017	0.0407	0.0174	-2.209	-0.0672	A2	0.017	0.0407	0.0174	-2.209	-0.0672	TRUE	TRUE	TRUE	TRUE	TRUE
1	DRCTTFDDV	17	25	9	HLA-A*02:01	17	0.0231	0.0553	0.8567	0.262	0.1969	A2	0.0231	0.0553	0.8567	0.262	0.1969	TRUE	TRUE	TRUE	TRUE	TRUE
1	RCTTFDDVQ	18	26	9	HLA-A*02:01	18	0.0224	0.0536	0.0124	-0.016	0.0546	A2	0.0224	0.0536	0.0124	-0.016	0.0546	TRUE	TRUE	TRUE	TRUE	TRUE
1	CTTFDDVQA	19	27	9	HLA-A*02:01	19	0.0289	0.0691	0.1818	-0.502	0.0713	A2	0.0289	0.0691	0.1818	-0.502	0.0713	TRUE	TRUE	TRUE	TRUE	TRUE
1	TTFDDVQAP	20	28	9	HLA-A*02:01	20	0.0926	0.2215	0.044	0.167	0.2365	A2	0.0926	0.2215	0.044	0.167	0.2365	TRUE	TRUE	TRUE	TRUE	TRUE
1	TFDDVQAPN	21	29	9	HLA-A*02:01	21	0.0297	0.0711	0.0826	-1.335	0.0167	A2	0.0297	0.0711	0.0826	-1.335	0.0167	TRUE	TRUE	TRUE	TRUE	TRUE
1	FDDVQAPNY	22	30	9	HLA-A*02:01	22	0.0185	0.0444	0.7209	2.356	0.2703	A2	0.0185	0.0444	0.7209	2.356	0.2703	TRUE	TRUE	TRUE	TRUE	TRUE
1	DDVQAPNYT	23	31	9	HLA-A*02:01	23	0.0173	0.0414	0.0178	-1.356	-0.0237	A2	0.0173	0.0414	0.0178	-1.356	-0.0237	TRUE	TRUE	TRUE	TRUE	TRUE
1	DVQAPNYTQ	24	32	9	HLA-A*02:01	24	0.0335	0.0802	0.699	-0.454	0.1624	A2	0.0335	0.0802	0.699	-0.454	0.1624	TRUE	TRUE	TRUE	TRUE	TRUE
1	VQAPNYTQH	25	33	9	HLA-A*02:01	25	0.0242	0.0579	0.711	-0.52	0.1385	A2	0.0242	0.0579	0.711	-0.52	0.1385	TRUE	TRUE	TRUE	TRUE	TRUE
1	QAPNYTQHT	26	34	9	HLA-A*02:01	26	0.0359	0.086	0.5145	-0.608	0.1328	A2	0.0359	0.086	0.5145	-0.608	0.1328	TRUE	TRUE	TRUE	TRUE	TRUE
1	APNYTQHTS	27	35	9	HLA-A*02:01	27	0.0205	0.0491	0.2055	-2.549	-0.0475	A2	0.0205	0.0491	0.2055	-2.549	-0.0475	TRUE	TRUE	TRUE	TRUE	TRUE
1	PNYTQHTSS	28	36	9	HLA-A*02:01	28	0.0171	0.041	0.021	-2.537	-0.0827	A2	0.0171	0.041	0.021	-2.537	-0.0827	TRUE	TRUE	TRUE	TRUE	TRUE
1	NYTQHTSSM	29	37	9	HLA-A*02:01	29	0.0239	0.0573	0.9903	0.425	0.2271	A2	0.0239	0.0573	0.9903	0.425	0.2271	TRUE	TRUE	TRUE	TRUE	TRUE
1	YTQHTSSMR	30	38	9	HLA-A*02:01	30	0.0429	0.1027	0.4729	1.552	0.2512	A2	0.0429	0.1027	0.4729	1.552	0.2512	TRUE	TRUE	TRUE	TRUE	TRUE
1	TQHTSSMRG	31	39	9	HLA-A*02:01	31	0.0241	0.0576	0.0824	-1.363	0.0018	A2	0.0241	0.0576	0.0824	-1.363	0.0018	TRUE	TRUE	TRUE	TRUE	TRUE
1	QHTSSMRGV	32	40	9	HLA-A*02:01	32	0.0301	0.0722	0.0209	0.309	0.0907	A2	0.0301	0.0722	0.0209	0.309	0.0907	TRUE	TRUE	TRUE	TRUE	TRUE
1	HTSSMRGVY	33	41	9	HLA-A*02:01	33	0.0209	0.0501	0.7345	2.759	0.2982	A2	0.0209	0.0501	0.7345	2.759	0.2982	TRUE	TRUE	TRUE	TRUE	TRUE
1	TSSMRGVYY	34	42	9	HLA-A*02:01	34	0.0178	0.0427	0.0478	2.917	0.1957	A2	0.0178	0.0427	0.0478	2.917	0.1957	TRUE	TRUE	TRUE	TRUE	TRUE
1	SSMRGVYYP	35	43	9	HLA-A*02:01	35	0.0473	0.1131	0.0131	0.273	0.1287	A2	0.0473	0.1131	0.0131	0.273	0.1287	TRUE	TRUE	TRUE	TRUE	TRUE
1	SMRGVYYPD	36	44	9	HLA-A*02:01	36	0.1076	0.2576	0.0852	-1.538	0.1935	A2	0.1076	0.2576	0.0852	-1.538	0.1935	TRUE	TRUE	TRUE	TRUE	TRUE
1	MRGVYYPDE	37	45	9	HLA-A*02:01	37	0.016	0.0382	0.0165	-1.421	-0.0304	A2	0.016	0.0382	0.0165	-1.421	-0.0304	TRUE	TRUE	TRUE	TRUE	TRUE
1	RGVYYPDEI	38	46	9	HLA-A*02:01	38	0.0217	0.0521	0.2207	0.626	0.1165	A2	0.0217	0.0521	0.2207	0.626	0.1165	TRUE	TRUE	TRUE	TRUE	TRUE
1	GVYYPDEIF	39	47	9	HLA-A*02:01	39	0.0358	0.0856	0.3526	2.813	0.2791	A2	0.0358	0.0856	0.3526	2.813	0.2791	TRUE	TRUE	TRUE	TRUE	TRUE
1	VYYPDEIFR	40	48	9	HLA-A*02:01	40	0.0495	0.1184	0.0903	1.968	0.2303	A2	0.0495	0.1184	0.0903	1.968	0.2303	TRUE	TRUE	TRUE	TRUE	TRUE
1	YYPDEIFRS	41	49	9	HLA-A*02:01	41	0.2624	0.6281	0.0907	-1.969	0.5432	A2	0.2624	0.6281	0.0907	-1.969	0.5432	TRUE	TRUE	TRUE	TRUE	TRUE
1	YPDEIFRSD	42	50	9	HLA-A*02:01	42	0.0223	0.0534	0.7515	-2.368	0.0477	A2	0.0223	0.0534	0.7515	-2.368	0.0477	TRUE	TRUE	TRUE	TRUE	TRUE
1	PDEIFRSDT	43	51	9	HLA-A*02:01	43	0.0163	0.039	0.0102	-1.689	-0.0439	A2	0.0163	0.039	0.0102	-1.689	-0.0439	TRUE	TRUE	TRUE	TRUE	TRUE
1	DEIFRSDTL	44	52	9	HLA-A*02:01	44	0.0201	0.0481	0.9958	0.427	0.2188	A2	0.0201	0.0481	0.9958	0.427	0.2188	TRUE	TRUE	TRUE	TRUE	TRUE
1	EIFRSDTLY	45	53	9	HLA-A*02:01	45	0.0311	0.0745	0.9968	2.822	0.3651	A2	0.0311	0.0745	0.9968	2.822	0.3651	TRUE	TRUE	TRUE	TRUE	TRUE
1	IFRSDTLYL	46	54	9	HLA-A*02:01	46	0.1223	0.2928	0.9967	1.027	0.4936	A2	0.1223	0.2928	0.9967	1.027	0.4936	TRUE	TRUE	TRUE	TRUE	TRUE
1	FRSDTLYLT	47	55	9	HLA-A*02:01	47	0.0858	0.2052	0.035	-0.589	0.181	A2	0.0858	0.2052	0.035	-0.589	0.181	TRUE	TRUE	TRUE	TRUE	TRUE
1	RSDTLYLTQ	48	56	9	HLA-A*02:01	48	0.0195	0.0466	0.3087	-0.102	0.0878	A2	0.0195	0.0466	0.3087	-0.102	0.0878	TRUE	TRUE	TRUE	TRUE	TRUE
1	SDTLYLTQD	49	57	9	HLA-A*02:01	49	0.0144	0.0346	0.0345	-1.974	-0.059	A2	0.0144	0.0346	0.0345	-1.974	-0.059	TRUE	TRUE	TRUE	TRUE	TRUE
1	DTLYLTQDL	50	58	9	HLA-A*02:01	50	0.0417	0.0998	0.4214	0.689	0.1974	A2	0.0417	0.0998	0.4214	0.689	0.1974	TRUE	TRUE	TRUE	TRUE	TRUE
1	TLYLTQDLF	51	59	9	HLA-A*02:01	51	0.0716	0.1713	0.0279	2.581	0.3046	A2	0.0716	0.1713	0.0279	2.581	0.3046	TRUE	TRUE	TRUE	TRUE	TRUE
1	LYLTQDLFL	52	60	9	HLA-A*02:01	52	0.0658	0.1574	0.7209	1.251	0.3281	A2	0.0658	0.1574	0.7209	1.251	0.3281	TRUE	TRUE	TRUE	TRUE	TRUE
1	YLTQDLFLP	53	61	9	HLA-A*02:01	53	0.3053	0.7307	0.0133	0.116	0.7385	A2	0.3053	0.7307	0.0133	0.116	0.7385	TRUE	TRUE	TRUE	TRUE	TRUE
1	LTQDLFLPF	54	62	9	HLA-A*02:01	54	0.081	0.1939	0.8155	2.433	0.4378	A2	0.081	0.1939	0.8155	2.433	0.4378	TRUE	TRUE	TRUE	TRUE	TRUE
1	TQDLFLPFY	55	63	9	HLA-A*02:01	55	0.0176	0.0421	0.8118	2.851	0.3064	A2	0.0176	0.0421	0.8118	2.851	0.3064	TRUE	TRUE	TRUE	TRUE	TRUE
1	QDLFLPFYS	56	64	9	HLA-A*02:01	56	0.0207	0.0495	0.0262	-2.512	-0.0722	A2	0.0207	0.0495	0.0262	-2.512	-0.0722	TRUE	TRUE	TRUE	TRUE	TRUE
1	DLFLPFYSN	57	65	9	HLA-A*02:01	57	0.0389	0.0931	0.1111	-1.549	0.0323	A2	0.0389	0.0931	0.1111	-1.549	0.0323	TRUE	TRUE	TRUE	TRUE	TRUE
1	LFLPFYSNV	58	66	9	HLA-A*02:01	58	0.1773	0.4242	0.6642	0.365	0.5421	A2	0.1773	0.4242	0.6642	0.365	0.5421	TRUE	TRUE	TRUE	TRUE	TRUE
1	FLPFYSNVT	59	67	9	HLA-A*02:01	59	0.2703	0.6469	0.3428	-0.748	0.6609	A2	0.2703	0.6469	0.3428	-0.748	0.6609	TRUE	TRUE	TRUE	TRUE	TRUE
1	LPFYSNVTG	60	68	9	HLA-A*02:01	60	0.017	0.0407	0.118	-1.689	-0.0261	A2	0.017	0.0407	0.118	-1.689	-0.0261	TRUE	TRUE	TRUE	TRUE	TRUE
1	PFYSNVTGF	61	69	9	HLA-A*02:01	61	0.0373	0.0894	0.4168	2.457	0.2748	A2	0.0373	0.0894	0.4168	2.457	0.2748	TRUE	TRUE	TRUE	TRUE	TRUE
1	FYSNVTGFH	62	70	9	HLA-A*02:01	62	0.0399	0.0956	0.4857	-0.515	0.1427	A2	0.0399	0.0956	0.4857	-0.515	0.1427	TRUE	TRUE	TRUE	TRUE	TRUE
1	YSNVTGFHT	63	71	9	HLA-A*02:01	63	0.0438	0.1049	0.0145	-0.884	0.0629	A2	0.0438	0.1049	0.0145	-0.884	0.0629	TRUE	TRUE	TRUE	TRUE	TRUE
1	SNVTGFHTI	64	72	9	HLA-A*02:01	64	0.0319	0.0763	0.9955	0.461	0.2486	A2	0.0319	0.0763	0.9955	0.461	0.2486	TRUE	TRUE	TRUE	TRUE	TRUE
1	NVTGFHTIN	65	73	9	HLA-A*02:01	65	0.0212	0.0508	0.4838	-1.122	0.0672	A2	0.0212	0.0508	0.4838	-1.122	0.0672	TRUE	TRUE	TRUE	TRUE	TRUE
1	VTGFHTINH	66	74	9	HLA-A*02:01	66	0.0211	0.0504	0.0217	-0.517	0.0278	A2	0.0211	0.0504	0.0217	-0.517	0.0278	TRUE	TRUE	TRUE	TRUE	TRUE
1	TGFHTINHT	67	75	9	HLA-A*02:01	67	0.025	0.0599	0.0217	-0.834	0.0215	A2	0.025	0.0599	0.0217	-0.834	0.0215	TRUE	TRUE	TRUE	TRUE	TRUE
1	GFHTINHTF	68	76	9	HLA-A*02:01	68	0.0554	0.1325	0.9917	2.563	0.4094	A2	0.0554	0.1325	0.9917	2.563	0.4094	TRUE	TRUE	TRUE	TRUE	TRUE
1	FHTINHTFG	69	77	9	HLA-A*02:01	69	0.0174	0.0417	0.0109	-1.549	-0.0341	A2	0.0174	0.0417	0.0109	-1.549	-0.0341	TRUE	TRUE	TRUE	TRUE	TRUE
1	HTINHTFGN	70	78	9	HLA-A*02:01	70	0.0298	0.0714	0.0114	-1.464	-0.0001	A2	0.0298	0.0714	0.0114	-1.464	-0.0001	TRUE	TRUE	TRUE	TRUE	TRUE
1	TINHTFGNP	71	79	9	HLA-A*02:01	71	0.0388	0.0929	0.0139	0.095	0.0998	A2	0.0388	0.0929	0.0139	0.095	0.0998	TRUE	TRUE	TRUE	TRUE	TRUE
1	INHTFGNPV	72	80	9	HLA-A*02:01	72	0.04	0.0957	0.6719	0.225	0.2077	A2	0.04	0.0957	0.6719	0.225	0.2077	TRUE	TRUE	TRUE	TRUE	TRUE
1	NHTFGNPVI	73	81	9	HLA-A*02:01	73	0.0213	0.0509	0.8613	0.552	0.2077	A2	0.0213	0.0509	0.8613	0.552	0.2077	TRUE	TRUE	TRUE	TRUE	TRUE
1	HTFGNPVIP	74	82	9	HLA-A*02:01	74	0.0325	0.0778	0.0228	0.245	0.0935	A2	0.0325	0.0778	0.0228	0.245	0.0935	TRUE	TRUE	TRUE	TRUE	TRUE
1	TFGNPVIPF	75	83	9	HLA-A*02:01	75	0.0273	0.0653	0.2372	2.495	0.2256	A2	0.0273	0.0653	0.2372	2.495	0.2256	TRUE	TRUE	TRUE	TRUE	TRUE
1	FGNPVIPFK	76	84	9	HLA-A*02:01	76	0.0426	0.102	0.5024	0.019	0.1783	A2	0.0426	0.102	0.5024	0.019	0.1783	TRUE	TRUE	TRUE	TRUE	TRUE
1	GNPVIPFKD	77	85	9	HLA-A*02:01	77	0.0155	0.0371	0.0119	-2.267	-0.0744	A2	0.0155	0.0371	0.0119	-2.267	-0.0744	TRUE	TRUE	TRUE	TRUE	TRUE
1	NPVIPFKDG	78	86	9	HLA-A*02:01	78	0.0147	0.0353	0.0181	-1.615	-0.0427	A2	0.0147	0.0353	0.0181	-1.615	-0.0427	TRUE	TRUE	TRUE	TRUE	TRUE
1	PVIPFKDGI	79	87	9	HLA-A*02:01	79	0.087	0.2081	0.19	0.403	0.2568	A2	0.087	0.2081	0.19	0.403	0.2568	TRUE	TRUE	TRUE	TRUE	TRUE
1	VIPFKDGIY	80	88	9	HLA-A*02:01	80	0.0285	0.0681	0.9816	2.955	0.3631	A2	0.0285	0.0681	0.9816	2.955	0.3631	TRUE	TRUE	TRUE	TRUE	TRUE
1	IPFKDGIYF	81	89	9	HLA-A*02:01	81	0.0303	0.0725	0.9957	2.412	0.3425	A2	0.0303	0.0725	0.9957	2.412	0.3425	TRUE	TRUE	TRUE	TRUE	TRUE
1	PFKDGIYFA	82	90	9	HLA-A*02:01	82	0.1952	0.4672	0.9212	-0.841	0.5634	A2	0.1952	0.4672	0.9212	-0.841	0.5634	TRUE	TRUE	TRUE	TRUE	TRUE
1	FKDGIYFAA	83	91	9	HLA-A*02:01	83	0.22	0.5265	0.5655	-0.902	0.5662	A2	0.22	0.5265	0.5655	-0.902	0.5662	TRUE	TRUE	TRUE	TRUE	TRUE
1	KDGIYFAAT	84	92	9	HLA-A*02:01	84	0.0172	0.0412	0.0155	-1.174	-0.0152	A2	0.0172	0.0412	0.0155	-1.174	-0.0152	TRUE	TRUE	TRUE	TRUE	TRUE
1	DGIYFAATE	85	93	9	HLA-A*02:01	85	0.0149	0.0357	0.0098	-1.978	-0.0618	A2	0.0149	0.0357	0.0098	-1.978	-0.0618	TRUE	TRUE	TRUE	TRUE	TRUE
1	GIYFAATEK	86	94	9	HLA-A*02:01	86	0.0549	0.1314	0.9965	0.462	0.304	A2	0.0549	0.1314	0.9965	0.462	0.304	TRUE	TRUE	TRUE	TRUE	TRUE
1	IYFAATEKS	87	95	9	HLA-A*02:01	87	0.0481	0.1151	0.1221	-1.876	0.0397	A2	0.0481	0.1151	0.1221	-1.876	0.0397	TRUE	TRUE	TRUE	TRUE	TRUE
1	YFAATEKSN	88	96	9	HLA-A*02:01	88	0.023	0.0551	0.0105	-1.142	-0.0004	A2	0.023	0.0551	0.0105	-1.142	-0.0004	TRUE	TRUE	TRUE	TRUE	TRUE
1	FAATEKSNV	89	97	9	HLA-A*02:01	89	0.0944	0.2259	0.0265	0.237	0.2417	A2	0.0944	0.2259	0.0265	0.237	0.2417	TRUE	TRUE	TRUE	TRUE	TRUE
1	AATEKSNVV	90	98	9	HLA-A*02:01	90	0.1997	0.478	0.9986	0.37	0.6463	A2	0.1997	0.478	0.9986	0.37	0.6463	TRUE	TRUE	TRUE	TRUE	TRUE
1	ATEKSNVVR	91	99	9	HLA-A*02:01	91	0.0286	0.0684	0.0766	1.617	0.1608	A2	0.0286	0.0684	0.0766	1.617	0.1608	TRUE	TRUE	TRUE	TRUE	TRUE
1	TEKSNVVRG	92	100	9	HLA-A*02:01	92	0.0169	0.0404	0.0167	-1.4	-0.0271	A2	0.0169	0.0404	0.0167	-1.4	-0.0271	TRUE	TRUE	TRUE	TRUE	TRUE
1	EKSNVVRGW	93	101	9	HLA-A*02:01	93	0.0149	0.0358	0.4842	0.736	0.1452	A2	0.0149	0.0358	0.4842	0.736	0.1452	TRUE	TRUE	TRUE	TRUE	TRUE
1	KSNVVRGWV	94	102	9	HLA-A*02:01	94	0.0361	0.0863	0.0257	0.201	0.1002	A2	0.0361	0.0863	0.0257	0.201	0.1002	TRUE	TRUE	TRUE	TRUE	TRUE
1	SNVVRGWVF	95	103	9	HLA-A*02:01	95	0.0185	0.0443	0.2791	2.614	0.2168	A2	0.0185	0.0443	0.2791	2.614	0.2168	TRUE	TRUE	TRUE	TRUE	TRUE
1	NVVRGWVFG	96	104	9	HLA-A*02:01	96	0.0361	0.0865	0.0101	-1.088	0.0336	A2	0.0361	0.0865	0.0101	-1.088	0.0336	TRUE	TRUE	TRUE	TRUE	TRUE
1	VVRGWVFGS	97	105	9	HLA-A*02:01	97	0.2749	0.6578	0.0198	-1.855	0.568	A2	0.2749	0.6578	0.0198	-1.855	0.568	TRUE	TRUE	TRUE	TRUE	TRUE
1	VRGWVFGST	98	106	9	HLA-A*02:01	98	0.0241	0.0577	0.2625	-0.553	0.0694	A2	0.0241	0.0577	0.2625	-0.553	0.0694	TRUE	TRUE	TRUE	TRUE	TRUE
1	RGWVFGSTM	99	107	9	HLA-A*02:01	99	0.0191	0.0458	0.9981	0.501	0.2206	A2	0.0191	0.0458	0.9981	0.501	0.2206	TRUE	TRUE	TRUE	TRUE	TRUE
1	GWVFGSTMN	100	108	9	HLA-A*02:01	100	0.0161	0.0384	0.3621	-1.038	0.0409	A2	0.0161	0.0384	0.3621	-1.038	0.0409	TRUE	TRUE	TRUE	TRUE	TRUE
1	WVFGSTMNN	101	109	9	HLA-A*02:01	101	0.1616	0.3868	0.0151	-1.122	0.3329	A2	0.1616	0.3868	0.0151	-1.122	0.3329	TRUE	TRUE	TRUE	TRUE	TRUE
1	VFGSTMNNK	102	110	9	HLA-A*02:01	102	0.0307	0.0734	0.0276	0.475	0.1013	A2	0.0307	0.0734	0.0276	0.475	0.1013	TRUE	TRUE	TRUE	TRUE	TRUE
1	FGSTMNNKS	103	111	9	HLA-A*02:01	103	0.0233	0.0558	0.0106	-2.564	-0.0708	A2	0.0233	0.0558	0.0106	-2.564	-0.0708	TRUE	TRUE	TRUE	TRUE	TRUE
1	GSTMNNKSQ	104	112	9	HLA-A*02:01	104	0.017	0.0408	0.0101	-0.385	0.023	A2	0.017	0.0408	0.0101	-0.385	0.023	TRUE	TRUE	TRUE	TRUE	TRUE
1	STMNNKSQS	105	113	9	HLA-A*02:01	105	0.0288	0.069	0.0097	-2.179	-0.0385	A2	0.0288	0.069	0.0097	-2.179	-0.0385	TRUE	TRUE	TRUE	TRUE	TRUE
1	TMNNKSQSV	106	114	9	HLA-A*02:01	106	0.4024	0.9631	0.89	0.375	1.1154	A2	0.4024	0.9631	0.89	0.375	1.1154	TRUE	TRUE	TRUE	TRUE	TRUE
1	MNNKSQSVI	107	115	9	HLA-A*02:01	107	0.0238	0.0569	0.0379	0.345	0.0798	A2	0.0238	0.0569	0.0379	0.345	0.0798	TRUE	TRUE	TRUE	TRUE	TRUE
1	NNKSQSVII	108	116	9	HLA-A*02:01	108	0.0176	0.0421	0.2326	0.453	0.0996	A2	0.0176	0.0421	0.2326	0.453	0.0996	TRUE	TRUE	TRUE	TRUE	TRUE
1	NKSQSVIII	109	117	9	HLA-A*02:01	109	0.0283	0.0678	0.2838	0.758	0.1483	A2	0.0283	0.0678	0.2838	0.758	0.1483	TRUE	TRUE	TRUE	TRUE	TRUE
1	KSQSVIIIN	110	118	9	HLA-A*02:01	110	0.0186	0.0444	0.0101	-1.059	-0.007	A2	0.0186	0.0444	0.0101	-1.059	-0.007	TRUE	TRUE	TRUE	TRUE	TRUE
1	SQSVIIINN	111	119	9	HLA-A*02:01	111	0.0169	0.0403	0.0099	-1.09	-0.0127	A2	0.0169	0.0403	0.0099	-1.09	-0.0127	TRUE	TRUE	TRUE	TRUE	TRUE
1	QSVIIINNS	112	120	9	HLA-A*02:01	112	0.0208	0.0497	0.0098	-2.06	-0.0518	A2	0.0208	0.0497	0.0098	-2.06	-0.0518	TRUE	TRUE	TRUE	TRUE	TRUE
1	SVIIINNST	113	121	9	HLA-A*02:01	113	0.0425	0.1016	0.0363	-0.494	0.0824	A2	0.0425	0.1016	0.0363	-0.494	0.0824	TRUE	TRUE	TRUE	TRUE	TRUE
1	VIIINNSTN	114	122	9	HLA-A*02:01	114	0.0293	0.0702	0.0097	-1.002	0.0215	A2	0.0293	0.0702	0.0097	-1.002	0.0215	TRUE	TRUE	TRUE	TRUE	TRUE
1	IIINNSTNV	115	123	9	HLA-A*02:01	115	0.2725	0.6521	0.8658	0.582	0.811	A2	0.2725	0.6521	0.8658	0.582	0.811	TRUE	TRUE	TRUE	TRUE	TRUE
1	IINNSTNVV	116	124	9	HLA-A*02:01	116	0.2453	0.587	0.9665	0.381	0.751	A2	0.2453	0.587	0.9665	0.381	0.751	TRUE	TRUE	TRUE	TRUE	TRUE
1	INNSTNVVI	117	125	9	HLA-A*02:01	117	0.0256	0.0612	0.9266	0.32	0.2162	A2	0.0256	0.0612	0.9266	0.32	0.2162	TRUE	TRUE	TRUE	TRUE	TRUE
1	NNSTNVVIR	118	126	9	HLA-A*02:01	118	0.0145	0.0348	0.4129	1.374	0.1654	A2	0.0145	0.0348	0.4129	1.374	0.1654	TRUE	TRUE	TRUE	TRUE	TRUE
1	NSTNVVIRA	119	127	9	HLA-A*02:01	119	0.0222	0.0532	0.9447	-0.353	0.1773	A2	0.0222	0.0532	0.9447	-0.353	0.1773	TRUE	TRUE	TRUE	TRUE	TRUE
1	STNVVIRAC	120	128	9	HLA-A*02:01	120	0.0322	0.0771	0.0113	0.093	0.0834	A2	0.0322	0.0771	0.0113	0.093	0.0834	TRUE	TRUE	TRUE	TRUE	TRUE
1	TNVVIRACN	121	129	9	HLA-A*02:01	121	0.0148	0.0355	0.0099	-1.357	-0.0309	A2	0.0148	0.0355	0.0099	-1.357	-0.0309	TRUE	TRUE	TRUE	TRUE	TRUE
1	NVVIRACNF	122	130	9	HLA-A*02:01	122	0.0266	0.0636	0.9202	2.798	0.3415	A2	0.0266	0.0636	0.9202	2.798	0.3415	TRUE	TRUE	TRUE	TRUE	TRUE
1	VVIRACNFE	123	131	9	HLA-A*02:01	123	0.0798	0.1909	0.0099	-1.155	0.1346	A2	0.0798	0.1909	0.0099	-1.155	0.1346	TRUE	TRUE	TRUE	TRUE	TRUE
1	VIRACNFEL	124	132	9	HLA-A*02:01	124	0.1795	0.4297	0.9783	1.241	0.6385	A2	0.1795	0.4297	0.9783	1.241	0.6385	TRUE	TRUE	TRUE	TRUE	TRUE
1	IRACNFELC	125	133	9	HLA-A*02:01	125	0.0195	0.0468	0.0128	0.308	0.0641	A2	0.0195	0.0468	0.0128	0.308	0.0641	TRUE	TRUE	TRUE	TRUE	TRUE
1	RACNFELCD	126	134	9	HLA-A*02:01	126	0.0192	0.046	0.0099	-1.501	-0.0276	A2	0.0192	0.046	0.0099	-1.501	-0.0276	TRUE	TRUE	TRUE	TRUE	TRUE
1	ACNFELCDN	127	135	9	HLA-A*02:01	127	0.0204	0.0488	0.0095	-1.113	-0.0055	A2	0.0204	0.0488	0.0095	-1.113	-0.0055	TRUE	TRUE	TRUE	TRUE	TRUE
1	CNFELCDNP	128	136	9	HLA-A*02:01	128	0.0213	0.0509	0.0097	0.177	0.0613	A2	0.0213	0.0509	0.0097	0.177	0.0613	TRUE	TRUE	TRUE	TRUE	TRUE
1	NFELCDNPF	129	137	9	HLA-A*02:01	129	0.0185	0.0443	0.2642	2.588	0.2134	A2	0.0185	0.0443	0.2642	2.588	0.2134	TRUE	TRUE	TRUE	TRUE	TRUE
1	FELCDNPFF	130	138	9	HLA-A*02:01	130	0.0283	0.0678	0.7842	2.463	0.3086	A2	0.0283	0.0678	0.7842	2.463	0.3086	TRUE	TRUE	TRUE	TRUE	TRUE
1	ELCDNPFFA	131	139	9	HLA-A*02:01	131	0.3485	0.8341	0.3268	-0.816	0.8423	A2	0.3485	0.8341	0.3268	-0.816	0.8423	TRUE	TRUE	TRUE	TRUE	TRUE
1	LCDNPFFAV	132	140	9	HLA-A*02:01	132	0.2616	0.626	0.9916	-0.004	0.7745	A2	0.2616	0.626	0.9916	-0.004	0.7745	TRUE	TRUE	TRUE	TRUE	TRUE
1	CDNPFFAVS	133	141	9	HLA-A*02:01	133	0.0188	0.045	0.0986	-2.64	-0.0722	A2	0.0188	0.045	0.0986	-2.64	-0.0722	TRUE	TRUE	TRUE	TRUE	TRUE
1	DNPFFAVSK	134	142	9	HLA-A*02:01	134	0.0146	0.035	0.6098	-0.026	0.1252	A2	0.0146	0.035	0.6098	-0.026	0.1252	TRUE	TRUE	TRUE	TRUE	TRUE
1	NPFFAVSKP	135	143	9	HLA-A*02:01	135	0.0153	0.0367	0.7801	-0.19	0.1442	A2	0.0153	0.0367	0.7801	-0.19	0.1442	TRUE	TRUE	TRUE	TRUE	TRUE
1	PFFAVSKPM	136	144	9	HLA-A*02:01	136	0.0383	0.0918	0.9381	0.245	0.2447	A2	0.0383	0.0918	0.9381	0.245	0.2447	TRUE	TRUE	TRUE	TRUE	TRUE
1	FFAVSKPMG	137	145	9	HLA-A*02:01	137	0.0254	0.0607	0.0096	-1.451	-0.0104	A2	0.0254	0.0607	0.0096	-1.451	-0.0104	TRUE	TRUE	TRUE	TRUE	TRUE
1	FAVSKPMGT	138	146	9	HLA-A*02:01	138	0.052	0.1245	0.0109	-0.775	0.0874	A2	0.052	0.1245	0.0109	-0.775	0.0874	TRUE	TRUE	TRUE	TRUE	TRUE
1	AVSKPMGTQ	139	147	9	HLA-A*02:01	139	0.0509	0.1219	0.0333	0.068	0.1303	A2	0.0509	0.1219	0.0333	0.068	0.1303	TRUE	TRUE	TRUE	TRUE	TRUE
1	VSKPMGTQT	140	148	9	HLA-A*02:01	140	0.0299	0.0716	0.0127	-0.451	0.0509	A2	0.0299	0.0716	0.0127	-0.451	0.0509	TRUE	TRUE	TRUE	TRUE	TRUE
1	SKPMGTQTH	141	149	9	HLA-A*02:01	141	0.0204	0.0489	0.0455	-0.401	0.0357	A2	0.0204	0.0489	0.0455	-0.401	0.0357	TRUE	TRUE	TRUE	TRUE	TRUE
1	KPMGTQTHT	142	150	9	HLA-A*02:01	142	0.025	0.0597	0.6776	-0.784	0.1222	A2	0.025	0.0597	0.6776	-0.784	0.1222	TRUE	TRUE	TRUE	TRUE	TRUE
1	PMGTQTHTM	143	151	9	HLA-A*02:01	143	0.2294	0.5491	0.9159	-0.116	0.6806	A2	0.2294	0.5491	0.9159	-0.116	0.6806	TRUE	TRUE	TRUE	TRUE	TRUE
1	MGTQTHTMI	144	152	9	HLA-A*02:01	144	0.0272	0.0651	0.7298	0.134	0.1813	A2	0.0272	0.0651	0.7298	0.134	0.1813	TRUE	TRUE	TRUE	TRUE	TRUE
1	GTQTHTMIF	145	153	9	HLA-A*02:01	145	0.0379	0.0908	0.5753	2.338	0.294	A2	0.0379	0.0908	0.5753	2.338	0.294	TRUE	TRUE	TRUE	TRUE	TRUE
1	TQTHTMIFD	146	154	9	HLA-A*02:01	146	0.0222	0.0531	0.0097	-1.827	-0.0368	A2	0.0222	0.0531	0.0097	-1.827	-0.0368	TRUE	TRUE	TRUE	TRUE	TRUE
1	QTHTMIFDN	147	155	9	HLA-A*02:01	147	0.0391	0.0935	0.0185	-1.284	0.0321	A2	0.0391	0.0935	0.0185	-1.284	0.0321	TRUE	TRUE	TRUE	TRUE	TRUE
1	THTMIFDNA	148	156	9	HLA-A*02:01	148	0.0188	0.045	0.13	-0.634	0.0328	A2	0.0188	0.045	0.13	-0.634	0.0328	TRUE	TRUE	TRUE	TRUE	TRUE
1	HTMIFDNAF	149	157	9	HLA-A*02:01	149	0.0249	0.0596	0.5966	2.497	0.274	A2	0.0249	0.0596	0.5966	2.497	0.274	TRUE	TRUE	TRUE	TRUE	TRUE
1	TMIFDNAFN	150	158	9	HLA-A*02:01	150	0.0727	0.174	0.0103	-1.169	0.1171	A2	0.0727	0.174	0.0103	-1.169	0.1171	TRUE	TRUE	TRUE	TRUE	TRUE
1	MIFDNAFNC	151	159	9	HLA-A*02:01	151	0.4452	1.0654	0.0195	0.312	1.0839	A2	0.4452	1.0654	0.0195	0.312	1.0839	TRUE	TRUE	TRUE	TRUE	TRUE
1	IFDNAFNCT	152	160	9	HLA-A*02:01	152	0.0537	0.1284	0.0363	-0.655	0.1011	A2	0.0537	0.1284	0.0363	-0.655	0.1011	TRUE	TRUE	TRUE	TRUE	TRUE
1	FDNAFNCTF	153	161	9	HLA-A*02:01	153	0.022	0.0527	0.8757	2.047	0.2864	A2	0.022	0.0527	0.8757	2.047	0.2864	TRUE	TRUE	TRUE	TRUE	TRUE
1	DNAFNCTFE	154	162	9	HLA-A*02:01	154	0.0155	0.0372	0.0096	-2.122	-0.0674	A2	0.0155	0.0372	0.0096	-2.122	-0.0674	TRUE	TRUE	TRUE	TRUE	TRUE
1	NAFNCTFEY	155	163	9	HLA-A*02:01	155	0.0286	0.0684	0.9791	2.987	0.3646	A2	0.0286	0.0684	0.9791	2.987	0.3646	TRUE	TRUE	TRUE	TRUE	TRUE
1	AFNCTFEYI	156	164	9	HLA-A*02:01	156	0.0621	0.1487	0.3747	0.893	0.2495	A2	0.0621	0.1487	0.3747	0.893	0.2495	TRUE	TRUE	TRUE	TRUE	TRUE
1	FNCTFEYIS	157	165	9	HLA-A*02:01	157	0.0213	0.051	0.0106	-2.33	-0.0639	A2	0.0213	0.051	0.0106	-2.33	-0.0639	TRUE	TRUE	TRUE	TRUE	TRUE
1	NCTFEYISD	158	166	9	HLA-A*02:01	158	0.0172	0.0412	0.0298	-1.899	-0.0493	A2	0.0172	0.0412	0.0298	-1.899	-0.0493	TRUE	TRUE	TRUE	TRUE	TRUE
1	CTFEYISDA	159	167	9	HLA-A*02:01	159	0.1554	0.3718	0.834	-0.345	0.4797	A2	0.1554	0.3718	0.834	-0.345	0.4797	TRUE	TRUE	TRUE	TRUE	TRUE
1	TFEYISDAF	160	168	9	HLA-A*02:01	160	0.0306	0.0731	0.2991	2.53	0.2445	A2	0.0306	0.0731	0.2991	2.53	0.2445	TRUE	TRUE	TRUE	TRUE	TRUE
1	FEYISDAFS	161	169	9	HLA-A*02:01	161	0.036	0.086	0.0101	-2.279	-0.0264	A2	0.036	0.086	0.0101	-2.279	-0.0264	TRUE	TRUE	TRUE	TRUE	TRUE

seq #	peptide	start	end	peptide length	allele	peptide index	NetCTL Predict	NetCTL Rescale	NetCTL c termi	NetCTL TAP tra	NetCTL Predict	NetCTL Superty	legacy predicte	legacy rescale_	legacy c_termi	legacy tap_tra	legacy predicti	equal predicte	equal rescale_	equal c_termir	equal tap_tran	equal predictions_score
1	EYISDAFSL	162	170	9	HLA-A*02:01	162	0.0725	0.1734	0.9978	0.979	0.372	A2	0.0725	0.1734	0.9978	0.979	0.372	TRUE	TRUE	TRUE	TRUE	TRUE
1	YISDAFSLD	163	171	9	HLA-A*02:01	163	0.0874	0.2091	0.0838	-1.878	0.1278	A2	0.0874	0.2091	0.0838	-1.878	0.1278	TRUE	TRUE	TRUE	TRUE	TRUE
1	ISDAFSLDV	164	172	9	HLA-A*02:01	164	0.0743	0.1779	0.9978	0.15	0.3351	A2	0.0743	0.1779	0.9978	0.15	0.3351	TRUE	TRUE	TRUE	TRUE	TRUE
1	SDAFSLDVS	165	173	9	HLA-A*02:01	165	0.0181	0.0434	0.0111	-2.474	-0.0786	A2	0.0181	0.0434	0.0111	-2.474	-0.0786	TRUE	TRUE	TRUE	TRUE	TRUE
1	DAFSLDVSE	166	174	9	HLA-A*02:01	166	0.0189	0.0451	0.0114	-1.663	-0.0363	A2	0.0189	0.0451	0.0114	-1.663	-0.0363	TRUE	TRUE	TRUE	TRUE	TRUE
1	AFSLDVSEK	167	175	9	HLA-A*02:01	167	0.0254	0.0608	0.9854	0.633	0.2402	A2	0.0254	0.0608	0.9854	0.633	0.2402	TRUE	TRUE	TRUE	TRUE	TRUE
1	FSLDVSEKS	168	176	9	HLA-A*02:01	168	0.1154	0.2761	0.0467	-2.128	0.1767	A2	0.1154	0.2761	0.0467	-2.128	0.1767	TRUE	TRUE	TRUE	TRUE	TRUE
1	SLDVSEKSG	169	177	9	HLA-A*02:01	169	0.0322	0.0769	0.0568	-1.604	0.0053	A2	0.0322	0.0769	0.0568	-1.604	0.0053	TRUE	TRUE	TRUE	TRUE	TRUE
1	LDVSEKSGN	170	178	9	HLA-A*02:01	170	0.0148	0.0354	0.0101	-1.487	-0.0374	A2	0.0148	0.0354	0.0101	-1.487	-0.0374	TRUE	TRUE	TRUE	TRUE	TRUE
1	DVSEKSGNF	171	179	9	HLA-A*02:01	171	0.0458	0.1096	0.8309	2.401	0.3543	A2	0.0458	0.1096	0.8309	2.401	0.3543	TRUE	TRUE	TRUE	TRUE	TRUE
1	VSEKSGNFK	172	180	9	HLA-A*02:01	172	0.0264	0.0632	0.0599	0.284	0.0864	A2	0.0264	0.0632	0.0599	0.284	0.0864	TRUE	TRUE	TRUE	TRUE	TRUE
1	SEKSGNFKH	173	181	9	HLA-A*02:01	173	0.0214	0.0512	0.2063	-0.573	0.0535	A2	0.0214	0.0512	0.2063	-0.573	0.0535	TRUE	TRUE	TRUE	TRUE	TRUE
1	EKSGNFKHL	174	182	9	HLA-A*02:01	174	0.0261	0.0624	0.9722	0.85	0.2507	A2	0.0261	0.0624	0.9722	0.85	0.2507	TRUE	TRUE	TRUE	TRUE	TRUE
1	KSGNFKHLR	175	183	9	HLA-A*02:01	175	0.0163	0.0391	0.0962	1.302	0.1186	A2	0.0163	0.0391	0.0962	1.302	0.1186	TRUE	TRUE	TRUE	TRUE	TRUE
1	SGNFKHLRE	176	184	9	HLA-A*02:01	176	0.0152	0.0364	0.0137	-1.772	-0.0502	A2	0.0152	0.0364	0.0137	-1.772	-0.0502	TRUE	TRUE	TRUE	TRUE	TRUE
1	GNFKHLREF	177	185	9	HLA-A*02:01	177	0.0183	0.0438	0.1062	2.369	0.1782	A2	0.0183	0.0438	0.1062	2.369	0.1782	TRUE	TRUE	TRUE	TRUE	TRUE
1	NFKHLREFV	178	186	9	HLA-A*02:01	178	0.0641	0.1535	0.0528	0.487	0.1858	A2	0.0641	0.1535	0.0528	0.487	0.1858	TRUE	TRUE	TRUE	TRUE	TRUE
1	FKHLREFVF	179	187	9	HLA-A*02:01	179	0.0278	0.0665	0.8001	2.66	0.3195	A2	0.0278	0.0665	0.8001	2.66	0.3195	TRUE	TRUE	TRUE	TRUE	TRUE
1	KHLREFVFK	180	188	9	HLA-A*02:01	180	0.0194	0.0464	0.7954	0.492	0.1903	A2	0.0194	0.0464	0.7954	0.492	0.1903	TRUE	TRUE	TRUE	TRUE	TRUE
1	HLREFVFKN	181	189	9	HLA-A*02:01	181	0.1423	0.3405	0.0383	-1.237	0.2844	A2	0.1423	0.3405	0.0383	-1.237	0.2844	TRUE	TRUE	TRUE	TRUE	TRUE
1	LREFVFKNK	182	190	9	HLA-A*02:01	182	0.016	0.0384	0.8198	0.508	0.1868	A2	0.016	0.0384	0.8198	0.508	0.1868	TRUE	TRUE	TRUE	TRUE	TRUE
1	REFVFKNKD	183	191	9	HLA-A*02:01	183	0.0147	0.0353	0.0161	-1.547	-0.0397	A2	0.0147	0.0353	0.0161	-1.547	-0.0397	TRUE	TRUE	TRUE	TRUE	TRUE
1	EFVFKNKDG	184	192	9	HLA-A*02:01	184	0.0189	0.0452	0.011	-1.222	-0.0142	A2	0.0189	0.0452	0.011	-1.222	-0.0142	TRUE	TRUE	TRUE	TRUE	TRUE
1	FVFKNKDGF	185	193	9	HLA-A*02:01	185	0.0944	0.226	0.0141	2.523	0.3542	A2	0.0944	0.226	0.0141	2.523	0.3542	TRUE	TRUE	TRUE	TRUE	TRUE
1	VFKNKDGFL	186	194	9	HLA-A*02:01	186	0.0572	0.1369	0.6934	1.077	0.2948	A2	0.0572	0.1369	0.6934	1.077	0.2948	TRUE	TRUE	TRUE	TRUE	TRUE
1	FKNKDGFLY	187	195	9	HLA-A*02:01	187	0.0314	0.0752	0.6916	2.832	0.3206	A2	0.0314	0.0752	0.6916	2.832	0.3206	TRUE	TRUE	TRUE	TRUE	TRUE
1	KNKDGFLYV	188	196	9	HLA-A*02:01	188	0.0827	0.198	0.9941	0.162	0.3552	A2	0.0827	0.198	0.9941	0.162	0.3552	TRUE	TRUE	TRUE	TRUE	TRUE
1	NKDGFLYVY	189	197	9	HLA-A*02:01	189	0.0187	0.0447	0.999	2.936	0.3413	A2	0.0187	0.0447	0.999	2.936	0.3413	TRUE	TRUE	TRUE	TRUE	TRUE
1	KDGFLYVYK	190	198	9	HLA-A*02:01	190	0.016	0.0382	0.6657	0.242	0.1501	A2	0.016	0.0382	0.6657	0.242	0.1501	TRUE	TRUE	TRUE	TRUE	TRUE
1	DGFLYVYKG	191	199	9	HLA-A*02:01	191	0.015	0.0359	0.0304	-1.765	-0.0478	A2	0.015	0.0359	0.0304	-1.765	-0.0478	TRUE	TRUE	TRUE	TRUE	TRUE
1	GFLYVYKGY	192	200	9	HLA-A*02:01	192	0.0219	0.0523	0.0865	2.792	0.2049	A2	0.0219	0.0523	0.0865	2.792	0.2049	TRUE	TRUE	TRUE	TRUE	TRUE
1	FLYVYKGYQ	193	201	9	HLA-A*02:01	193	0.1553	0.3716	0.0112	-0.024	0.3721	A2	0.1553	0.3716	0.0112	-0.024	0.3721	TRUE	TRUE	TRUE	TRUE	TRUE
1	LYVYKGYQP	194	202	9	HLA-A*02:01	194	0.0265	0.0634	0.0099	0.346	0.0821	A2	0.0265	0.0634	0.0099	0.346	0.0821	TRUE	TRUE	TRUE	TRUE	TRUE
1	YVYKGYQPI	195	203	9	HLA-A*02:01	195	0.4132	0.9889	0.9942	0.888	1.1824	A2	0.4132	0.9889	0.9942	0.888	1.1824	TRUE	TRUE	TRUE	TRUE	TRUE
1	VYKGYQPID	196	204	9	HLA-A*02:01	196	0.0257	0.0616	0.0271	-1.543	-0.0115	A2	0.0257	0.0616	0.0271	-1.543	-0.0115	TRUE	TRUE	TRUE	TRUE	TRUE
1	YKGYQPIDV	197	205	9	HLA-A*02:01	197	0.0419	0.1002	0.71	0.228	0.2181	A2	0.0419	0.1002	0.71	0.228	0.2181	TRUE	TRUE	TRUE	TRUE	TRUE
1	KGYQPIDVV	198	206	9	HLA-A*02:01	198	0.1422	0.3402	0.9535	0.29	0.4977	A2	0.1422	0.3402	0.9535	0.29	0.4977	TRUE	TRUE	TRUE	TRUE	TRUE
1	GYQPIDVVR	199	207	9	HLA-A*02:01	199	0.0289	0.0692	0.9972	1.639	0.3008	A2	0.0289	0.0692	0.9972	1.639	0.3008	TRUE	TRUE	TRUE	TRUE	TRUE
1	YQPIDVVRD	200	208	9	HLA-A*02:01	200	0.0203	0.0485	0.0196	-1.805	-0.0388	A2	0.0203	0.0485	0.0196	-1.805	-0.0388	TRUE	TRUE	TRUE	TRUE	TRUE
1	QPIDVVRDL	201	209	9	HLA-A*02:01	201	0.0323	0.0772	0.9846	0.68	0.2589	A2	0.0323	0.0772	0.9846	0.68	0.2589	TRUE	TRUE	TRUE	TRUE	TRUE
1	PIDVVRDLP	202	210	9	HLA-A*02:01	202	0.0237	0.0568	0.0113	-0.472	0.0349	A2	0.0237	0.0568	0.0113	-0.472	0.0349	TRUE	TRUE	TRUE	TRUE	TRUE
1	IDVVRDLPS	203	211	9	HLA-A*02:01	203	0.0168	0.0402	0.0126	-2.597	-0.0878	A2	0.0168	0.0402	0.0126	-2.597	-0.0878	TRUE	TRUE	TRUE	TRUE	TRUE
1	DVVRDLPSG	204	212	9	HLA-A*02:01	204	0.0273	0.0653	0.0123	-1.486	-0.0071	A2	0.0273	0.0653	0.0123	-1.486	-0.0071	TRUE	TRUE	TRUE	TRUE	TRUE
1	VVRDLPSGF	205	213	9	HLA-A*02:01	205	0.0677	0.1619	0.5178	2.645	0.3718	A2	0.0677	0.1619	0.5178	2.645	0.3718	TRUE	TRUE	TRUE	TRUE	TRUE
1	VRDLPSGFN	206	214	9	HLA-A*02:01	206	0.0161	0.0386	0.0505	-1.176	-0.0126	A2	0.0161	0.0386	0.0505	-1.176	-0.0126	TRUE	TRUE	TRUE	TRUE	TRUE
1	RDLPSGFNT	207	215	9	HLA-A*02:01	207	0.0396	0.0947	0.0139	-0.704	0.0616	A2	0.0396	0.0947	0.0139	-0.704	0.0616	TRUE	TRUE	TRUE	TRUE	TRUE
1	DLPSGFNTL	208	216	9	HLA-A*02:01	208	0.1202	0.2877	0.9984	0.775	0.4762	A2	0.1202	0.2877	0.9984	0.775	0.4762	TRUE	TRUE	TRUE	TRUE	TRUE
1	LPSGFNTLK	209	217	9	HLA-A*02:01	209	0.0194	0.0464	0.997	0.021	0.197	A2	0.0194	0.0464	0.997	0.021	0.197	TRUE	TRUE	TRUE	TRUE	TRUE
1	PSGFNTLKP	210	218	9	HLA-A*02:01	210	0.0163	0.0389	0.1013	-0.45	0.0316	A2	0.0163	0.0389	0.1013	-0.45	0.0316	TRUE	TRUE	TRUE	TRUE	TRUE
1	SGFNTLKPI	211	219	9	HLA-A*02:01	211	0.0265	0.0633	0.667	0.288	0.1778	A2	0.0265	0.0633	0.667	0.288	0.1778	TRUE	TRUE	TRUE	TRUE	TRUE
1	GFNTLKPIF	212	220	9	HLA-A*02:01	212	0.0229	0.0547	0.4877	2.483	0.252	A2	0.0229	0.0547	0.4877	2.483	0.252	TRUE	TRUE	TRUE	TRUE	TRUE
1	FNTLKPIFK	213	221	9	HLA-A*02:01	213	0.0177	0.0423	0.0299	0.151	0.0543	A2	0.0177	0.0423	0.0299	0.151	0.0543	TRUE	TRUE	TRUE	TRUE	TRUE
1	NTLKPIFKL	214	222	9	HLA-A*02:01	214	0.1727	0.4133	0.9936	0.883	0.6065	A2	0.1727	0.4133	0.9936	0.883	0.6065	TRUE	TRUE	TRUE	TRUE	TRUE
1	TLKPIFKLP	215	223	9	HLA-A*02:01	215	0.0576	0.1379	0.0127	0.242	0.1519	A2	0.0576	0.1379	0.0127	0.242	0.1519	TRUE	TRUE	TRUE	TRUE	TRUE
1	LKPIFKLPL	216	224	9	HLA-A*02:01	216	0.0215	0.0514	0.4825	1.003	0.1739	A2	0.0215	0.0514	0.4825	1.003	0.1739	TRUE	TRUE	TRUE	TRUE	TRUE
1	KPIFKLPLG	217	225	9	HLA-A*02:01	217	0.0162	0.0389	0.0107	-1.476	-0.0333	A2	0.0162	0.0389	0.0107	-1.476	-0.0333	TRUE	TRUE	TRUE	TRUE	TRUE
1	PIFKLPLGI	218	226	9	HLA-A*02:01	218	0.1229	0.2942	0.564	0.396	0.3986	A2	0.1229	0.2942	0.564	0.396	0.3986	TRUE	TRUE	TRUE	TRUE	TRUE
1	IFKLPLGIN	219	227	9	HLA-A*02:01	219	0.0195	0.0467	0.0098	-1.272	-0.0155	A2	0.0195	0.0467	0.0098	-1.272	-0.0155	TRUE	TRUE	TRUE	TRUE	TRUE
1	FKLPLGINI	220	228	9	HLA-A*02:01	220	0.1924	0.4604	0.0203	0.516	0.4893	A2	0.1924	0.4604	0.0203	0.516	0.4893	TRUE	TRUE	TRUE	TRUE	TRUE
1	KLPLGINIT	221	229	9	HLA-A*02:01	221	0.1236	0.2958	0.504	-0.699	0.3364	A2	0.1236	0.2958	0.504	-0.699	0.3364	TRUE	TRUE	TRUE	TRUE	TRUE
1	LPLGINITN	222	230	9	HLA-A*02:01	222	0.0203	0.0485	0.1266	-1.497	-0.0074	A2	0.0203	0.0485	0.1266	-1.497	-0.0074	TRUE	TRUE	TRUE	TRUE	TRUE
1	PLGINITNF	223	231	9	HLA-A*02:01	223	0.0661	0.1581	0.7956	1.959	0.3754	A2	0.0661	0.1581	0.7956	1.959	0.3754	TRUE	TRUE	TRUE	TRUE	TRUE
1	LGINITNFR	224	232	9	HLA-A*02:01	224	0.0204	0.0487	0.1458	1.146	0.1279	A2	0.0204	0.0487	0.1458	1.146	0.1279	TRUE	TRUE	TRUE	TRUE	TRUE
1	GINITNFRA	225	233	9	HLA-A*02:01	225	0.1104	0.2642	0.7322	-0.632	0.3424	A2	0.1104	0.2642	0.7322	-0.632	0.3424	TRUE	TRUE	TRUE	TRUE	TRUE
1	INITNFRAI	226	234	9	HLA-A*02:01	226	0.0285	0.0683	0.0357	0.444	0.0959	A2	0.0285	0.0683	0.0357	0.444	0.0959	TRUE	TRUE	TRUE	TRUE	TRUE
1	NITNFRAIL	227	235	9	HLA-A*02:01	227	0.0496	0.1187	0.9965	1.093	0.3228	A2	0.0496	0.1187	0.9965	1.093	0.3228	TRUE	TRUE	TRUE	TRUE	TRUE
1	ITNFRAILT	228	236	9	HLA-A*02:01	228	0.032	0.0767	0.0601	-0.657	0.0529	A2	0.032	0.0767	0.0601	-0.657	0.0529	TRUE	TRUE	TRUE	TRUE	TRUE
1	TNFRAILTA	229	237	9	HLA-A*02:01	229	0.029	0.0695	0.6704	-0.588	0.1406	A2	0.029	0.0695	0.6704	-0.588	0.1406	TRUE	TRUE	TRUE	TRUE	TRUE
1	NFRAILTAF	230	238	9	HLA-A*02:01	230	0.0232	0.0556	0.9637	2.798	0.34	A2	0.0232	0.0556	0.9637	2.798	0.34	TRUE	TRUE	TRUE	TRUE	TRUE
1	FRAILTAFS	231	239	9	HLA-A*02:01	231	0.0326	0.0779	0.0148	-1.976	-0.0187	A2	0.0326	0.0779	0.0148	-1.976	-0.0187	TRUE	TRUE	TRUE	TRUE	TRUE
1	RAILTAFSP	232	240	9	HLA-A*02:01	232	0.0258	0.0618	0.028	0.365	0.0843	A2	0.0258	0.0618	0.028	0.365	0.0843	TRUE	TRUE	TRUE	TRUE	TRUE
1	AILTAFSPA	233	241	9	HLA-A*02:01	233	0.0952	0.2278	0.3356	-0.113	0.2725	A2	0.0952	0.2278	0.3356	-0.113	0.2725	TRUE	TRUE	TRUE	TRUE	TRUE
1	ILTAFSPAQ	234	242	9	HLA-A*02:01	234	0.13	0.3111	0.8175	0.055	0.4365	A2	0.13	0.3111	0.8175	0.055	0.4365	TRUE	TRUE	TRUE	TRUE	TRUE
1	LTAFSPAQD	235	243	9	HLA-A*02:01	235	0.0212	0.0506	0.0099	-1.807	-0.0382	A2	0.0212	0.0506	0.0099	-1.807	-0.0382	TRUE	TRUE	TRUE	TRUE	TRUE
1	TAFSPAQDI	236	244	9	HLA-A*02:01	236	0.0782	0.1872	0.2957	0.726	0.2678	A2	0.0782	0.1872	0.2957	0.726	0.2678	TRUE	TRUE	TRUE	TRUE	TRUE
1	AFSPAQDIW	237	245	9	HLA-A*02:01	237	0.0254	0.0608	0.1594	1.202	0.1448	A2	0.0254	0.0608	0.1594	1.202	0.1448	TRUE	TRUE	TRUE	TRUE	TRUE
1	FSPAQDIWG	238	246	9	HLA-A*02:01	238	0.0347	0.0831	0.0096	-1.311	0.019	A2	0.0347	0.0831	0.0096	-1.311	0.019	TRUE	TRUE	TRUE	TRUE	TRUE
1	SPAQDIWGT	239	247	9	HLA-A*02:01	239	0.0469	0.1122	0.0441	-1.045	0.0666	A2	0.0469	0.1122	0.0441	-1.045	0.0666	TRUE	TRUE	TRUE	TRUE	TRUE
1	PAQDIWGTS	240	248	9	HLA-A*02:01	240	0.0405	0.097	0.1599	-2.651	-0.0116	A2	0.0405	0.097	0.1599	-2.651	-0.0116	TRUE	TRUE	TRUE	TRUE	TRUE
1	AQDIWGTSA	241	249	9	HLA-A*02:01	241	0.0374	0.0894	0.6294	-0.637	0.152	A2	0.0374	0.0894	0.6294	-0.637	0.152	TRUE	TRUE	TRUE	TRUE	TRUE
1	QDIWGTSAA	242	250	9	HLA-A*02:01	242	0.0331	0.0793	0.1833	-0.607	0.0764	A2	0.0331	0.0793	0.1833	-0.607	0.0764	TRUE	TRUE	TRUE	TRUE	TRUE
1	DIWGTSAAA	243	251	9	HLA-A*02:01	243	0.2142	0.5125	0.9831	-0.6	0.63	A2	0.2142	0.5125	0.9831	-0.6	0.63	TRUE	TRUE	TRUE	TRUE	TRUE
1	IWGTSAAAY	244	252	9	HLA-A*02:01	244	0.018	0.0431	0.4284	2.892	0.2519	A2	0.018	0.0431	0.4284	2.892	0.2519	TRUE	TRUE	TRUE	TRUE	TRUE
1	WGTSAAAYF	245	253	9	HLA-A*02:01	245	0.031	0.0743	0.1486	2.214	0.2073	A2	0.031	0.0743	0.1486	2.214	0.2073	TRUE	TRUE	TRUE	TRUE	TRUE
1	GTSAAAYFV	246	254	9	HLA-A*02:01	246	0.2328	0.5572	0.1184	0.054	0.5777	A2	0.2328	0.5572	0.1184	0.054	0.5777	TRUE	TRUE	TRUE	TRUE	TRUE
1	TSAAAYFVG	247	255	9	HLA-A*02:01	247	0.0235	0.0562	0.0324	-1.389	-0.0084	A2	0.0235	0.0562	0.0324	-1.389	-0.0084	TRUE	TRUE	TRUE	TRUE	TRUE
1	SAAAYFVGY	248	256	9	HLA-A*02:01	248	0.0212	0.0508	0.9669	3.057	0.3487	A2	0.0212	0.0508	0.9669	3.057	0.3487	TRUE	TRUE	TRUE	TRUE	TRUE
1	AAAYFVGYL	249	257	9	HLA-A*02:01	249	0.0515	0.1232	0.4654	1.222	0.2541	A2	0.0515	0.1232	0.4654	1.222	0.2541	TRUE	TRUE	TRUE	TRUE	TRUE
1	AAYFVGYLK	250	258	9	HLA-A*02:01	250	0.0347	0.083	0.598	1.002	0.2228	A2	0.0347	0.083	0.598	1.002	0.2228	TRUE	TRUE	TRUE	TRUE	TRUE
1	AYFVGYLKP	251	259	9	HLA-A*02:01	251	0.0293	0.0701	0.0336	0.779	0.1141	A2	0.0293	0.0701	0.0336	0.779	0.1141	TRUE	TRUE	TRUE	TRUE	TRUE
1	YFVGYLKPT	252	260	9	HLA-A*02:01	252	0.0917	0.2194	0.0178	-0.326	0.2058	A2	0.0917	0.2194	0.0178	-0.326	0.2058	TRUE	TRUE	TRUE	TRUE	TRUE
1	FVGYLKPTT	253	261	9	HLA-A*02:01	253	0.0912	0.2182	0.0104	-0.877	0.1759	A2	0.0912	0.2182	0.0104	-0.877	0.1759	TRUE	TRUE	TRUE	TRUE	TRUE
1	VGYLKPTTF	254	262	9	HLA-A*02:01	254	0.0166	0.0397	0.9841	2.412	0.308	A2	0.0166	0.0397	0.9841	2.412	0.308	TRUE	TRUE	TRUE	TRUE	TRUE
1	GYLKPTTFM	255	263	9	HLA-A*02:01	255	0.06	0.1435	0.9902	0.455	0.3148	A2	0.06	0.1435	0.9902	0.455	0.3148	TRUE	TRUE	TRUE	TRUE	TRUE
1	YLKPTTFML	256	264	9	HLA-A*02:01	256	0.5837	1.3969	0.9991	0.922	1.5928	A2	0.5837	1.3969	0.9991	0.922	1.5928	TRUE	TRUE	TRUE	TRUE	TRUE
1	LKPTTFMLK	257	265	9	HLA-A*02:01	257	0.0215	0.0515	0.9436	0.451	0.2155	A2	0.0215	0.0515	0.9436	0.451	0.2155	TRUE	TRUE	TRUE	TRUE	TRUE
1	KPTTFMLKY	258	266	9	HLA-A*02:01	258	0.0166	0.0396	0.9988	2.739	0.3264	A2	0.0166	0.0396	0.9988	2.739	0.3264	TRUE	TRUE	TRUE	TRUE	TRUE
1	PTTFMLKYD	259	267	9	HLA-A*02:01	259	0.0192	0.046	0.0105	-2.252	-0.065	A2	0.0192	0.046	0.0105	-2.252	-0.065	TRUE	TRUE	TRUE	TRUE	TRUE
1	TTFMLKYDE	260	268	9	HLA-A*02:01	260	0.0249	0.0596	0.0097	-1.641	-0.021	A2	0.0249	0.0596	0.0097	-1.641	-0.021	TRUE	TRUE	TRUE	TRUE	TRUE
1	TFMLKYDEN	261	269	9	HLA-A*02:01	261	0.0199	0.0476	0.0097	-1.094	-0.0057	A2	0.0199	0.0476	0.0097	-1.094	-0.0057	TRUE	TRUE	TRUE	TRUE	TRUE
1	FMLKYDENG	262	270	9	HLA-A*02:01	262	0.2029	0.4857	0.0167	-1.341	0.4211	A2	0.2029	0.4857	0.0167	-1.341	0.4211	TRUE	TRUE	TRUE	TRUE	TRUE
1	MLKYDENGT	263	271	9	HLA-A*02:01	263	0.0941	0.2252	0.01	-0.746	0.1894	A2	0.0941	0.2252	0.01	-0.746	0.1894	TRUE	TRUE	TRUE	TRUE	TRUE
1	LKYDENGTI	264	272	9	HLA-A*02:01	264	0.0805	0.1927	0.8944	0.811	0.3674	A2	0.0805	0.1927	0.8944	0.811	0.3674	TRUE	TRUE	TRUE	TRUE	TRUE
1	KYDENGTIT	265	273	9	HLA-A*02:01	265	0.0513	0.1228	0.6043	-0.462	0.1904	A2	0.0513	0.1228	0.6043	-0.462	0.1904	TRUE	TRUE	TRUE	TRUE	TRUE
1	YDENGTITD	266	274	9	HLA-A*02:01	266	0.0202	0.0484	0.735	-2.186	0.0494	A2	0.0202	0.0484	0.735	-2.186	0.0494	TRUE	TRUE	TRUE	TRUE	TRUE
1	DENGTITDA	267	275	9	HLA-A*02:01	267	0.0233	0.0557	0.7173	-1.133	0.1066	A2	0.0233	0.0557	0.7173	-1.133	0.1066	TRUE	TRUE	TRUE	TRUE	TRUE
1	ENGTITDAV	268	276	9	HLA-A*02:01	268	0.0251	0.0601	0.9763	-0.344	0.1893	A2	0.0251	0.0601	0.9763	-0.344	0.1893	TRUE	TRUE	TRUE	TRUE	TRUE
1	NGTITDAVD	269	277	9	HLA-A*02:01	269	0.0147	0.0351	0.0199	-2.248	-0.0743	A2	0.0147	0.0351	0.0199	-2.248	-0.0743	TRUE	TRUE	TRUE	TRUE	TRUE
1	GTITDAVDC	270	278	9	HLA-A*02:01	270	0.0513	0.1228	0.0116	0.024	0.1258	A2	0.0513	0.1228	0.0116	0.024	0.1258	TRUE	TRUE	TRUE	TRUE	TRUE
1	TITDAVDCS	271	279	9	HLA-A*02:01	271	0.122	0.2919	0.0126	-2.204	0.1836	A2	0.122	0.2919	0.0126	-2.204	0.1836	TRUE	TRUE	TRUE	TRUE	TRUE
1	ITDAVDCSQ	272	280	9	HLA-A*02:01	272	0.032	0.0765	0.0224	-0.27	0.0664	A2	0.032	0.0765	0.0224	-0.27	0.0664	TRUE	TRUE	TRUE	TRUE	TRUE
1	TDAVDCSQN	273	281	9	HLA-A*02:01	273	0.0149	0.0356	0.0107	-1.618	-0.0437	A2	0.0149	0.0356	0.0107	-1.618	-0.0437	TRUE	TRUE	TRUE	TRUE	TRUE
1	DAVDCSQNP	274	282	9	HLA-A*02:01	274	0.0242	0.0578	0.0122	-0.071	0.0561	A2	0.0242	0.0578	0.0122	-0.071	0.0561	TRUE	TRUE	TRUE	TRUE	TRUE
1	AVDCSQNPL	275	283	9	HLA-A*02:01	275	0.0722	0.1727	0.9921	0.922	0.3676	A2	0.0722	0.1727	0.9921	0.922	0.3676	TRUE	TRUE	TRUE	TRUE	TRUE
1	VDCSQNPLA	276	284	9	HLA-A*02:01	276	0.0219	0.0525	0.0486	-0.618	0.0289	A2	0.0219	0.0525	0.0486	-0.618	0.0289	TRUE	TRUE	TRUE	TRUE	TRUE
1	DCSQNPLAE	277	285	9	HLA-A*02:01	277	0.0196	0.0468	0.0098	-1.818	-0.0426	A2	0.0196	0.0468	0.0098	-1.818	-0.0426	TRUE	TRUE	TRUE	TRUE	TRUE
1	CSQNPLAEL	278	286	9	HLA-A*02:01	278	0.0461	0.1104	0.9982	0.783	0.2993	A2	0.0461	0.1104	0.9982	0.783	0.2993	TRUE	TRUE	TRUE	TRUE	TRUE
1	SQNPLAELK	279	287	9	HLA-A*02:01	279	0.0198	0.0475	0.7942	0.492	0.1912	A2	0.0198	0.0475	0.7942	0.492	0.1912	TRUE	TRUE	TRUE	TRUE	TRUE
1	QNPLAELKC	280	288	9	HLA-A*02:01	280	0.0173	0.0414	0.014	-0.086	0.0392	A2	0.0173	0.0414	0.014	-0.086	0.0392	TRUE	TRUE	TRUE	TRUE	TRUE
1	NPLAELKCS	281	289	9	HLA-A*02:01	281	0.0167	0.0399	0.0106	-2.544	-0.0857	A2	0.0167	0.0399	0.0106	-2.544	-0.0857	TRUE	TRUE	TRUE	TRUE	TRUE
1	PLAELKCSV	282	290	9	HLA-A*02:01	282	0.44	1.053	0.9563	0.012	1.1971	A2	0.44	1.053	0.9563	0.012	1.1971	TRUE	TRUE	TRUE	TRUE	TRUE
1	LAELKCSVK	283	291	9	HLA-A*02:01	283	0.0201	0.0482	0.9976	0.232	0.2094	A2	0.0201	0.0482	0.9976	0.232	0.2094	TRUE	TRUE	TRUE	TRUE	TRUE
1	AELKCSVKS	284	292	9	HLA-A*02:01	284	0.0241	0.0578	0.0142	-2.171	-0.0487	A2	0.0241	0.0578	0.0142	-2.171	-0.0487	TRUE	TRUE	TRUE	TRUE	TRUE
1	ELKCSVKSF	285	293	9	HLA-A*02:01	285	0.0354	0.0847	0.9751	2.502	0.3561	A2	0.0354	0.0847	0.9751	2.502	0.3561	TRUE	TRUE	TRUE	TRUE	TRUE
1	LKCSVKSFE	286	294	9	HLA-A*02:01	286	0.0181	0.0434	0.0094	-1.67	-0.0387	A2	0.0181	0.0434	0.0094	-1.67	-0.0387	TRUE	TRUE	TRUE	TRUE	TRUE
1	KCSVKSFEI	287	295	9	HLA-A*02:01	287	0.1101	0.2634	0.282	0.706	0.341	A2	0.1101	0.2634	0.282	0.706	0.341	TRUE	TRUE	TRUE	TRUE	TRUE
1	CSVKSFEID	288	296	9	HLA-A*02:01	288	0.017	0.0406	0.0101	-1.637	-0.0397	A2	0.017	0.0406	0.0101	-1.637	-0.0397	TRUE	TRUE	TRUE	TRUE	TRUE
1	SVKSFEIDK	289	297	9	HLA-A*02:01	289	0.0316	0.0755	0.8709	0.647	0.2385	A2	0.0316	0.0755	0.8709	0.647	0.2385	TRUE	TRUE	TRUE	TRUE	TRUE
1	VKSFEIDKG	290	298	9	HLA-A*02:01	290	0.0184	0.0441	0.0097	-1.234	-0.0162	A2	0.0184	0.0441	0.0097	-1.234	-0.0162	TRUE	TRUE	TRUE	TRUE	TRUE
1	KSFEIDKGI	291	299	9	HLA-A*02:01	291	0.0853	0.2042	0.8129	0.841	0.3682	A2	0.0853	0.2042	0.8129	0.841	0.3682	TRUE	TRUE	TRUE	TRUE	TRUE
1	SFEIDKGIY	292	300	9	HLA-A*02:01	292	0.0176	0.0421	0.9403	3.102	0.3382	A2	0.0176	0.0421	0.9403	3.102	0.3382	TRUE	TRUE	TRUE	TRUE	TRUE
1	FEIDKGIYQ	293	301	9	HLA-A*02:01	293	0.0582	0.1394	0.0156	-0.213	0.1311	A2	0.0582	0.1394	0.0156	-0.213	0.1311	TRUE	TRUE	TRUE	TRUE	TRUE
1	EIDKGIYQT	294	302	9	HLA-A*02:01	294	0.1405	0.3363	0.2174	-1.062	0.3158	A2	0.1405	0.3363	0.2174	-1.062	0.3158	TRUE	TRUE	TRUE	TRUE	TRUE
1	IDKGIYQTS	295	303	9	HLA-A*02:01	295	0.0241	0.0578	0.042	-2.699	-0.0709	A2	0.0241	0.0578	0.042	-2.699	-0.0709	TRUE	TRUE	TRUE	TRUE	TRUE
1	DKGIYQTSN	296	304	9	HLA-A*02:01	296	0.0137	0.0328	0.0173	-1.736	-0.0514	A2	0.0137	0.0328	0.0173	-1.736	-0.0514	TRUE	TRUE	TRUE	TRUE	TRUE
1	KGIYQTSNF	297	305	9	HLA-A*02:01	297	0.0176	0.0421	0.3452	2.305	0.2091	A2	0.0176	0.0421	0.3452	2.305	0.2091	TRUE	TRUE	TRUE	TRUE	TRUE
1	GIYQTSNFR	298	306	9	HLA-A*02:01	298	0.1083	0.2591	0.6637	1.722	0.4448	A2	0.1083	0.2591	0.6637	1.722	0.4448	TRUE	TRUE	TRUE	TRUE	TRUE
1	IYQTSNFRV	299	307	9	HLA-A*02:01	299	0.2343	0.5607	0.988	0.54	0.7359	A2	0.2343	0.5607	0.988	0.54	0.7359	TRUE	TRUE	TRUE	TRUE	TRUE
1	YQTSNFRVV	300	308	9	HLA-A*02:01	300	0.0753	0.1803	0.1779	0.343	0.2241	A2	0.0753	0.1803	0.1779	0.343	0.2241	TRUE	TRUE	TRUE	TRUE	TRUE
1	QTSNFRVVP	301	309	9	HLA-A*02:01	301	0.0211	0.0504	0.0311	0.04	0.0571	A2	0.0211	0.0504	0.0311	0.04	0.0571	TRUE	TRUE	TRUE	TRUE	TRUE
1	TSNFRVVPS	302	310	9	HLA-A*02:01	302	0.0176	0.042	0.0108	-2.369	-0.0748	A2	0.0176	0.042	0.0108	-2.369	-0.0748	TRUE	TRUE	TRUE	TRUE	TRUE
1	SNFRVVPSG	303	311	9	HLA-A*02:01	303	0.0176	0.0421	0.0234	-1.373	-0.023	A2	0.0176	0.0421	0.0234	-1.373	-0.023	TRUE	TRUE	TRUE	TRUE	TRUE
1	NFRVVPSGD	304	312	9	HLA-A*02:01	304	0.0156	0.0374	0.0098	-1.508	-0.0366	A2	0.0156	0.0374	0.0098	-1.508	-0.0366	TRUE	TRUE	TRUE	TRUE	TRUE
1	FRVVPSGDV	305	313	9	HLA-A*02:01	305	0.043	0.103	0.9625	0.645	0.2796	A2	0.043	0.103	0.9625	0.645	0.2796	TRUE	TRUE	TRUE	TRUE	TRUE
1	RVVPSGDVV	306	314	9	HLA-A*02:01	306	0.1817	0.4348	0.594	0.67	0.5574	A2	0.1817	0.4348	0.594	0.67	0.5574	TRUE	TRUE	TRUE	TRUE	TRUE
1	VVPSGDVVR	307	315	9	HLA-A*02:01	307	0.0399	0.0956	0.1061	1.802	0.2016	A2	0.0399	0.0956	0.1061	1.802	0.2016	TRUE	TRUE	TRUE	TRUE	TRUE
1	VPSGDVVRF	308	316	9	HLA-A*02:01	308	0.0257	0.0615	0.9436	2.335	0.3198	A2	0.0257	0.0615	0.9436	2.335	0.3198	TRUE	TRUE	TRUE	TRUE	TRUE
1	PSGDVVRFP	309	317	9	HLA-A*02:01	309	0.0221	0.0528	0.0099	-0.451	0.0318	A2	0.0221	0.0528	0.0099	-0.451	0.0318	TRUE	TRUE	TRUE	TRUE	TRUE
1	SGDVVRFPN	310	318	9	HLA-A*02:01	310	0.0161	0.0386	0.0121	-1.85	-0.0521	A2	0.0161	0.0386	0.0121	-1.85	-0.0521	TRUE	TRUE	TRUE	TRUE	TRUE
1	GDVVRFPNI	311	319	9	HLA-A*02:01	311	0.0196	0.047	0.4152	0.18	0.1183	A2	0.0196	0.047	0.4152	0.18	0.1183	TRUE	TRUE	TRUE	TRUE	TRUE
1	DVVRFPNIT	312	320	9	HLA-A*02:01	312	0.0249	0.0597	0.818	-0.835	0.1406	A2	0.0249	0.0597	0.818	-0.835	0.1406	TRUE	TRUE	TRUE	TRUE	TRUE
1	VVRFPNITN	313	321	9	HLA-A*02:01	313	0.0252	0.0603	0.0208	-1.205	0.0032	A2	0.0252	0.0603	0.0208	-1.205	0.0032	TRUE	TRUE	TRUE	TRUE	TRUE
1	VRFPNITNL	314	322	9	HLA-A*02:01	314	0.06	0.1436	0.9987	1.382	0.3625	A2	0.06	0.1436	0.9987	1.382	0.3625	TRUE	TRUE	TRUE	TRUE	TRUE
1	RFPNITNLC	315	323	9	HLA-A*02:01	315	0.05	0.1196	0.0119	0.458	0.1443	A2	0.05	0.1196	0.0119	0.458	0.1443	TRUE	TRUE	TRUE	TRUE	TRUE
1	FPNITNLCP	316	324	9	HLA-A*02:01	316	0.0185	0.0444	0.0201	-0.257	0.0345	A2	0.0185	0.0444	0.0201	-0.257	0.0345	TRUE	TRUE	TRUE	TRUE	TRUE
1	PNITNLCPF	317	325	9	HLA-A*02:01	317	0.0188	0.0451	0.0559	1.866	0.1467	A2	0.0188	0.0451	0.0559	1.866	0.1467	TRUE	TRUE	TRUE	TRUE	TRUE
1	NITNLCPFG	318	326	9	HLA-A*02:01	318	0.0378	0.0905	0.0103	-1.411	0.0215	A2	0.0378	0.0905	0.0103	-1.411	0.0215	TRUE	TRUE	TRUE	TRUE	TRUE
1	ITNLCPFGE	319	327	9	HLA-A*02:01	319	0.0228	0.0546	0.0095	-1.517	-0.0198	A2	0.0228	0.0546	0.0095	-1.517	-0.0198	TRUE	TRUE	TRUE	TRUE	TRUE
1	TNLCPFGEV	320	328	9	HLA-A*02:01	320	0.026	0.0622	0.9204	0.168	0.2087	A2	0.026	0.0622	0.9204	0.168	0.2087	TRUE	TRUE	TRUE	TRUE	TRUE
1	NLCPFGEVF	321	329	9	HLA-A*02:01	321	0.08	0.1915	0.9807	2.61	0.4691	A2	0.08	0.1915	0.9807	2.61	0.4691	TRUE	TRUE	TRUE	TRUE	TRUE
1	LCPFGEVFN	322	330	9	HLA-A*02:01	322	0.0196	0.047	0.0105	-1.165	-0.0097	A2	0.0196	0.047	0.0105	-1.165	-0.0097	TRUE	TRUE	TRUE	TRUE	TRUE

seq #	peptide	start	end	peptide length	allele	peptide index	NetCTL Predict	NetCTL Rescale	NetCTL c termi	NetCTL TAP tra	NetCTL Predict	NetCTL Superty	legacy predicte	legacy rescale_	legacy c_termi	legacy tap_tran	legacy predicti	equal predicted	equal rescale_	equal c_termin	equal tap_tran	equal predictions_score
1	CPFGEVFNA	323	331	9	HLA-A*02:01	323	0.0456	0.1092	0.4427	-0.721	0.1396	A2	0.0456	0.1092	0.4427	-0.721	0.1396	TRUE	TRUE	TRUE	TRUE	TRUE
1	PFGEVFNAT	324	332	9	HLA-A*02:01	324	0.04	0.0958	0.0775	-1.188	0.0481	A2	0.04	0.0958	0.0775	-1.188	0.0481	TRUE	TRUE	TRUE	TRUE	TRUE
1	FGEVFNATK	325	333	9	HLA-A*02:01	325	0.0186	0.0446	0.6784	-0.186	0.137	A2	0.0186	0.0446	0.6784	-0.186	0.137	TRUE	TRUE	TRUE	TRUE	TRUE
1	GEVFNATKF	326	334	9	HLA-A*02:01	326	0.0173	0.0415	0.6856	2.193	0.254	A2	0.0173	0.0415	0.6856	2.193	0.254	TRUE	TRUE	TRUE	TRUE	TRUE
1	EVFNATKFP	327	335	9	HLA-A*02:01	327	0.0617	0.1477	0.0161	0.121	0.1562	A2	0.0617	0.1477	0.0161	0.121	0.1562	TRUE	TRUE	TRUE	TRUE	TRUE
1	VFNATKFPS	328	336	9	HLA-A*02:01	328	0.0515	0.1233	0.0095	-2.277	0.0109	A2	0.0515	0.1233	0.0095	-2.277	0.0109	TRUE	TRUE	TRUE	TRUE	TRUE
1	FNATKFPSV	329	337	9	HLA-A*02:01	329	0.0518	0.1239	0.9701	0.131	0.276	A2	0.0518	0.1239	0.9701	0.131	0.276	TRUE	TRUE	TRUE	TRUE	TRUE
1	NATKFPSVY	330	338	9	HLA-A*02:01	330	0.0166	0.0398	0.9981	2.873	0.3332	A2	0.0166	0.0398	0.9981	2.873	0.3332	TRUE	TRUE	TRUE	TRUE	TRUE
1	ATKFPSVYA	331	339	9	HLA-A*02:01	331	0.0586	0.1402	0.7787	-0.274	0.2434	A2	0.0586	0.1402	0.7787	-0.274	0.2434	TRUE	TRUE	TRUE	TRUE	TRUE
1	TKFPSVYAW	332	340	9	HLA-A*02:01	332	0.0209	0.0499	0.9956	1.179	0.2582	A2	0.0209	0.0499	0.9956	1.179	0.2582	TRUE	TRUE	TRUE	TRUE	TRUE
1	KFPSVYAWE	333	341	9	HLA-A*02:01	333	0.0432	0.1033	0.0096	-1.296	0.04	A2	0.0432	0.1033	0.0096	-1.296	0.04	TRUE	TRUE	TRUE	TRUE	TRUE
1	FPSVYAWER	334	342	9	HLA-A*02:01	334	0.0266	0.0637	0.7624	1.182	0.2371	A2	0.0266	0.0637	0.7624	1.182	0.2371	TRUE	TRUE	TRUE	TRUE	TRUE
1	PSVYAWERK	335	343	9	HLA-A*02:01	335	0.0178	0.0425	0.0615	-0.002	0.0517	A2	0.0178	0.0425	0.0615	-0.002	0.0517	TRUE	TRUE	TRUE	TRUE	TRUE
1	SVYAWERKK	336	344	9	HLA-A*02:01	336	0.0555	0.1327	0.1751	0.698	0.1939	A2	0.0555	0.1327	0.1751	0.698	0.1939	TRUE	TRUE	TRUE	TRUE	TRUE
1	VYAWERKKI	337	345	9	HLA-A*02:01	337	0.0793	0.1897	0.1426	0.932	0.2577	A2	0.0793	0.1897	0.1426	0.932	0.2577	TRUE	TRUE	TRUE	TRUE	TRUE
1	YAWERKKIS	338	346	9	HLA-A*02:01	338	0.0555	0.1328	0.03	-1.994	0.0376	A2	0.0555	0.1328	0.03	-1.994	0.0376	TRUE	TRUE	TRUE	TRUE	TRUE
1	AWERKKISN	339	347	9	HLA-A*02:01	339	0.0158	0.0379	0.0116	-1.121	-0.0164	A2	0.0158	0.0379	0.0116	-1.121	-0.0164	TRUE	TRUE	TRUE	TRUE	TRUE
1	WERKKISNC	340	348	9	HLA-A*02:01	340	0.0382	0.0914	0.0098	0.055	0.0956	A2	0.0382	0.0914	0.0098	0.055	0.0956	TRUE	TRUE	TRUE	TRUE	TRUE
1	ERKKISNCV	341	349	9	HLA-A*02:01	341	0.0316	0.0757	0.9667	0.259	0.2336	A2	0.0316	0.0757	0.9667	0.259	0.2336	TRUE	TRUE	TRUE	TRUE	TRUE
1	RKKISNCVA	342	350	9	HLA-A*02:01	342	0.0203	0.0485	0.0791	-0.411	0.0399	A2	0.0203	0.0485	0.0791	-0.411	0.0399	TRUE	TRUE	TRUE	TRUE	TRUE
1	KKISNCVAD	343	351	9	HLA-A*02:01	343	0.0181	0.0433	0.0117	-1.458	-0.0278	A2	0.0181	0.0433	0.0117	-1.458	-0.0278	TRUE	TRUE	TRUE	TRUE	TRUE
1	KISNCVADY	344	352	9	HLA-A*02:01	344	0.0382	0.0913	0.9479	3.257	0.3964	A2	0.0382	0.0913	0.9479	3.257	0.3964	TRUE	TRUE	TRUE	TRUE	TRUE
1	ISNCVADYS	345	353	9	HLA-A*02:01	345	0.0208	0.0497	0.0096	-2.175	-0.0576	A2	0.0208	0.0497	0.0096	-2.175	-0.0576	TRUE	TRUE	TRUE	TRUE	TRUE
1	SNCVADYSV	346	354	9	HLA-A*02:01	346	0.0286	0.0685	0.237	0.233	0.1157	A2	0.0286	0.0685	0.237	0.233	0.1157	TRUE	TRUE	TRUE	TRUE	TRUE
1	NCVADYSVL	347	355	9	HLA-A*02:01	347	0.0282	0.0674	0.4798	1.144	0.1965	A2	0.0282	0.0674	0.4798	1.144	0.1965	TRUE	TRUE	TRUE	TRUE	TRUE
1	CVADYSVLY	348	356	9	HLA-A*02:01	348	0.0465	0.1112	0.9981	3.18	0.4199	A2	0.0465	0.1112	0.9981	3.18	0.4199	TRUE	TRUE	TRUE	TRUE	TRUE
1	VADYSVLYN	349	357	9	HLA-A*02:01	349	0.0245	0.0586	0.0102	-1.384	-0.0091	A2	0.0245	0.0586	0.0102	-1.384	-0.0091	TRUE	TRUE	TRUE	TRUE	TRUE
1	ADYSVLYNS	350	358	9	HLA-A*02:01	350	0.0313	0.0749	0.0127	-2.175	-0.0319	A2	0.0313	0.0749	0.0127	-2.175	-0.0319	TRUE	TRUE	TRUE	TRUE	TRUE
1	DYSVLYNST	351	359	9	HLA-A*02:01	351	0.0194	0.0464	0.0187	-0.607	0.0189	A2	0.0194	0.0464	0.0187	-0.607	0.0189	TRUE	TRUE	TRUE	TRUE	TRUE
1	YSVLYNSTF	352	360	9	HLA-A*02:01	352	0.0206	0.0492	0.8762	2.494	0.3054	A2	0.0206	0.0492	0.8762	2.494	0.3054	TRUE	TRUE	TRUE	TRUE	TRUE
1	SVLYNSTFF	353	361	9	HLA-A*02:01	353	0.0556	0.1331	0.5293	2.692	0.3471	A2	0.0556	0.1331	0.5293	2.692	0.3471	TRUE	TRUE	TRUE	TRUE	TRUE
1	VLYNSTFFS	354	362	9	HLA-A*02:01	354	0.5057	1.2102	0.0672	-1.923	1.1242	A2	0.5057	1.2102	0.0672	-1.923	1.1242	TRUE	TRUE	TRUE	TRUE	TRUE
1	LYNSTFFST	355	363	9	HLA-A*02:01	355	0.0647	0.1549	0.0991	-0.484	0.1455	A2	0.0647	0.1549	0.0991	-0.484	0.1455	TRUE	TRUE	TRUE	TRUE	TRUE
1	YNSTFFSTF	356	364	9	HLA-A*02:01	356	0.0191	0.0458	0.9979	2.406	0.3158	A2	0.0191	0.0458	0.9979	2.406	0.3158	TRUE	TRUE	TRUE	TRUE	TRUE
1	NSTFFSTFK	357	365	9	HLA-A*02:01	357	0.0168	0.0403	0.8845	0.454	0.1956	A2	0.0168	0.0403	0.8845	0.454	0.1956	TRUE	TRUE	TRUE	TRUE	TRUE
1	STFFSTFKC	358	366	9	HLA-A*02:01	358	0.1107	0.2649	0.0152	0.299	0.2822	A2	0.1107	0.2649	0.0152	0.299	0.2822	TRUE	TRUE	TRUE	TRUE	TRUE
1	TFFSTFKCY	359	367	9	HLA-A*02:01	359	0.0254	0.0608	0.9775	3.237	0.3693	A2	0.0254	0.0608	0.9775	3.237	0.3693	TRUE	TRUE	TRUE	TRUE	TRUE
1	FFSTFKCYG	360	368	9	HLA-A*02:01	360	0.0377	0.0903	0.0104	-1.317	0.026	A2	0.0377	0.0903	0.0104	-1.317	0.026	TRUE	TRUE	TRUE	TRUE	TRUE
1	FSTFKCYGV	361	369	9	HLA-A*02:01	361	0.0983	0.2353	0.9542	0.119	0.3843	A2	0.0983	0.2353	0.9542	0.119	0.3843	TRUE	TRUE	TRUE	TRUE	TRUE
1	STFKCYGVS	362	370	9	HLA-A*02:01	362	0.0341	0.0815	0.013	-2.207	-0.0269	A2	0.0341	0.0815	0.013	-2.207	-0.0269	TRUE	TRUE	TRUE	TRUE	TRUE
1	TFKCYGVSA	363	371	9	HLA-A*02:01	363	0.0275	0.0659	0.1655	-0.337	0.0738	A2	0.0275	0.0659	0.1655	-0.337	0.0738	TRUE	TRUE	TRUE	TRUE	TRUE
1	FKCYGVSAT	364	372	9	HLA-A*02:01	364	0.0322	0.0771	0.0109	-0.732	0.0421	A2	0.0322	0.0771	0.0109	-0.732	0.0421	TRUE	TRUE	TRUE	TRUE	TRUE
1	KCYGVSATK	365	373	9	HLA-A*02:01	365	0.0661	0.1582	0.969	0.674	0.3373	A2	0.0661	0.1582	0.969	0.674	0.3373	TRUE	TRUE	TRUE	TRUE	TRUE
1	CYGVSATKL	366	374	9	HLA-A*02:01	366	0.0244	0.0584	0.8533	1.116	0.2421	A2	0.0244	0.0584	0.8533	1.116	0.2421	TRUE	TRUE	TRUE	TRUE	TRUE
1	YGVSATKLN	367	375	9	HLA-A*02:01	367	0.0169	0.0404	0.0113	-1.531	-0.0344	A2	0.0169	0.0404	0.0113	-1.531	-0.0344	TRUE	TRUE	TRUE	TRUE	TRUE
1	GVSATKLND	368	376	9	HLA-A*02:01	368	0.028	0.0671	0.0107	-1.912	-0.0269	A2	0.028	0.0671	0.0107	-1.912	-0.0269	TRUE	TRUE	TRUE	TRUE	TRUE
1	VSATKLNDL	369	377	9	HLA-A*02:01	369	0.0377	0.0902	0.5157	1.044	0.2198	A2	0.0377	0.0902	0.5157	1.044	0.2198	TRUE	TRUE	TRUE	TRUE	TRUE
1	SATKLNDLC	370	378	9	HLA-A*02:01	370	0.0367	0.0879	0.0099	0.131	0.0959	A2	0.0367	0.0879	0.0099	0.131	0.0959	TRUE	TRUE	TRUE	TRUE	TRUE
1	ATKLNDLCF	371	379	9	HLA-A*02:01	371	0.0251	0.06	0.2241	2.709	0.2291	A2	0.0251	0.06	0.2241	2.709	0.2291	TRUE	TRUE	TRUE	TRUE	TRUE
1	TKLNDLCFS	372	380	9	HLA-A*02:01	372	0.0311	0.0745	0.0111	-2.045	-0.0261	A2	0.0311	0.0745	0.0111	-2.045	-0.0261	TRUE	TRUE	TRUE	TRUE	TRUE
1	KLNDLCFSN	373	381	9	HLA-A*02:01	373	0.2004	0.4795	0.028	-1.273	0.4201	A2	0.2004	0.4795	0.028	-1.273	0.4201	TRUE	TRUE	TRUE	TRUE	TRUE
1	LNDLCFSNV	374	382	9	HLA-A*02:01	374	0.0222	0.0531	0.4554	0.091	0.126	A2	0.0222	0.0531	0.4554	0.091	0.126	TRUE	TRUE	TRUE	TRUE	TRUE
1	NDLCFSNVY	375	383	9	HLA-A*02:01	375	0.0147	0.0352	0.9419	2.72	0.3124	A2	0.0147	0.0352	0.9419	2.72	0.3124	TRUE	TRUE	TRUE	TRUE	TRUE
1	DLCFSNVYA	376	384	9	HLA-A*02:01	376	0.0824	0.1972	0.0447	-0.682	0.1698	A2	0.0824	0.1972	0.0447	-0.682	0.1698	TRUE	TRUE	TRUE	TRUE	TRUE
1	LCFSNVYAD	377	385	9	HLA-A*02:01	377	0.0363	0.0868	0.0172	-1.818	-0.0016	A2	0.0363	0.0868	0.0172	-1.818	-0.0016	TRUE	TRUE	TRUE	TRUE	TRUE
1	CFSNVYADS	378	386	9	HLA-A*02:01	378	0.0364	0.0871	0.0193	-2.075	-0.0138	A2	0.0364	0.0871	0.0193	-2.075	-0.0138	TRUE	TRUE	TRUE	TRUE	TRUE
1	FSNVYADSF	379	387	9	HLA-A*02:01	379	0.0248	0.0594	0.0793	2.379	0.1902	A2	0.0248	0.0594	0.0793	2.379	0.1902	TRUE	TRUE	TRUE	TRUE	TRUE
1	SNVYADSFV	380	388	9	HLA-A*02:01	380	0.0282	0.0676	0.0389	0.188	0.0828	A2	0.0282	0.0676	0.0389	0.188	0.0828	TRUE	TRUE	TRUE	TRUE	TRUE
1	NVYADSFVV	381	389	9	HLA-A*02:01	381	0.3466	0.8295	0.9981	0.731	1.0158	A2	0.3466	0.8295	0.9981	0.731	1.0158	TRUE	TRUE	TRUE	TRUE	TRUE
1	VYADSFVVK	382	390	9	HLA-A*02:01	382	0.0472	0.1129	0.9939	0.949	0.3094	A2	0.0472	0.1129	0.9939	0.949	0.3094	TRUE	TRUE	TRUE	TRUE	TRUE
1	YADSFVVKG	383	391	9	HLA-A*02:01	383	0.0409	0.0979	0.1536	-1.492	0.0463	A2	0.0409	0.0979	0.1536	-1.492	0.0463	TRUE	TRUE	TRUE	TRUE	TRUE
1	ADSFVVKGD	384	392	9	HLA-A*02:01	384	0.0151	0.0362	0.0367	-2.035	-0.0601	A2	0.0151	0.0362	0.0367	-2.035	-0.0601	TRUE	TRUE	TRUE	TRUE	TRUE
1	DSFVVKGDD	385	393	9	HLA-A*02:01	385	0.0148	0.0355	0.0099	-1.825	-0.0543	A2	0.0148	0.0355	0.0099	-1.825	-0.0543	TRUE	TRUE	TRUE	TRUE	TRUE
1	SFVVKGDDV	386	394	9	HLA-A*02:01	386	0.0577	0.1382	0.0498	0.474	0.1694	A2	0.0577	0.1382	0.0498	0.474	0.1694	TRUE	TRUE	TRUE	TRUE	TRUE
1	FVVKGDDVR	387	395	9	HLA-A*02:01	387	0.0593	0.1418	0.9423	1.66	0.3662	A2	0.0593	0.1418	0.9423	1.66	0.3662	TRUE	TRUE	TRUE	TRUE	TRUE
1	VVKGDDVRQ	388	396	9	HLA-A*02:01	388	0.0722	0.1728	0.1638	-0.012	0.1967	A2	0.0722	0.1728	0.1638	-0.012	0.1967	TRUE	TRUE	TRUE	TRUE	TRUE
1	VKGDDVRQI	389	397	9	HLA-A*02:01	389	0.0622	0.1489	0.9454	0.53	0.3172	A2	0.0622	0.1489	0.9454	0.53	0.3172	TRUE	TRUE	TRUE	TRUE	TRUE
1	KGDDVRQIA	390	398	9	HLA-A*02:01	390	0.0278	0.0664	0.3103	-0.824	0.0718	A2	0.0278	0.0664	0.3103	-0.824	0.0718	TRUE	TRUE	TRUE	TRUE	TRUE
1	GDDVRQIAP	391	399	9	HLA-A*02:01	391	0.0148	0.0354	0.013	-0.457	0.0145	A2	0.0148	0.0354	0.013	-0.457	0.0145	TRUE	TRUE	TRUE	TRUE	TRUE
1	DDVRQIAPG	392	400	9	HLA-A*02:01	392	0.0151	0.0362	0.0271	-1.971	-0.0583	A2	0.0151	0.0362	0.0271	-1.971	-0.0583	TRUE	TRUE	TRUE	TRUE	TRUE
1	DVRQIAPGQ	393	401	9	HLA-A*02:01	393	0.0348	0.0833	0.0099	-0.373	0.0661	A2	0.0348	0.0833	0.0099	-0.373	0.0661	TRUE	TRUE	TRUE	TRUE	TRUE
1	VRQIAPGQT	394	402	9	HLA-A*02:01	394	0.0192	0.046	0.0141	-0.608	0.0177	A2	0.0192	0.046	0.0141	-0.608	0.0177	TRUE	TRUE	TRUE	TRUE	TRUE
1	RQIAPGQTG	395	403	9	HLA-A*02:01	395	0.0193	0.0461	0.0116	-0.96	-0.0001	A2	0.0193	0.0461	0.0116	-0.96	-0.0001	TRUE	TRUE	TRUE	TRUE	TRUE
1	QIAPGQTGV	396	404	9	HLA-A*02:01	396	0.2482	0.594	0.8707	0.605	0.7549	A2	0.2482	0.594	0.8707	0.605	0.7549	TRUE	TRUE	TRUE	TRUE	TRUE
1	IAPGQTGVI	397	405	9	HLA-A*02:01	397	0.073	0.1747	0.8234	0.546	0.3255	A2	0.073	0.1747	0.8234	0.546	0.3255	TRUE	TRUE	TRUE	TRUE	TRUE
1	APGQTGVIA	398	406	9	HLA-A*02:01	398	0.0203	0.0487	0.3595	-0.85	0.0601	A2	0.0203	0.0487	0.3595	-0.85	0.0601	TRUE	TRUE	TRUE	TRUE	TRUE
1	PGQTGVIAD	399	407	9	HLA-A*02:01	399	0.0164	0.0393	0.0142	-2.44	-0.0806	A2	0.0164	0.0393	0.0142	-2.44	-0.0806	TRUE	TRUE	TRUE	TRUE	TRUE
1	GQTGVIADY	400	408	9	HLA-A*02:01	400	0.0271	0.0648	0.9988	2.64	0.3466	A2	0.0271	0.0648	0.9988	2.64	0.3466	TRUE	TRUE	TRUE	TRUE	TRUE
1	QTGVIADYN	401	409	9	HLA-A*02:01	401	0.0177	0.0422	0.0106	-1.517	-0.032	A2	0.0177	0.0422	0.0106	-1.517	-0.032	TRUE	TRUE	TRUE	TRUE	TRUE
1	TGVIADYNY	402	410	9	HLA-A*02:01	402	0.0161	0.0384	0.9985	2.666	0.3215	A2	0.0161	0.0384	0.9985	2.666	0.3215	TRUE	TRUE	TRUE	TRUE	TRUE
1	GVIADYNYK	403	411	9	HLA-A*02:01	403	0.0525	0.1256	0.9024	0.503	0.2861	A2	0.0525	0.1256	0.9024	0.503	0.2861	TRUE	TRUE	TRUE	TRUE	TRUE
1	VIADYNYKL	404	412	9	HLA-A*02:01	404	0.3762	0.9002	0.9974	1.132	1.1065	A2	0.3762	0.9002	0.9974	1.132	1.1065	TRUE	TRUE	TRUE	TRUE	TRUE
1	IADYNYKLP	405	413	9	HLA-A*02:01	405	0.0198	0.0474	0.01	0.051	0.0515	A2	0.0198	0.0474	0.01	0.051	0.0515	TRUE	TRUE	TRUE	TRUE	TRUE
1	ADYNYKLPD	406	414	9	HLA-A*02:01	406	0.0173	0.0414	0.0171	-1.783	-0.0452	A2	0.0173	0.0414	0.0171	-1.783	-0.0452	TRUE	TRUE	TRUE	TRUE	TRUE
1	DYNYKLPDD	407	415	9	HLA-A*02:01	407	0.017	0.0406	0.0099	-1.807	-0.0482	A2	0.017	0.0406	0.0099	-1.807	-0.0482	TRUE	TRUE	TRUE	TRUE	TRUE
1	YNYKLPDDF	408	416	9	HLA-A*02:01	408	0.0266	0.0636	0.0547	2.41	0.1923	A2	0.0266	0.0636	0.0547	2.41	0.1923	TRUE	TRUE	TRUE	TRUE	TRUE
1	NYKLPDDFM	409	417	9	HLA-A*02:01	409	0.0204	0.0489	0.0998	0.552	0.0915	A2	0.0204	0.0489	0.0998	0.552	0.0915	TRUE	TRUE	TRUE	TRUE	TRUE
1	YKLPDDFMG	410	418	9	HLA-A*02:01	410	0.0456	0.1092	0.0392	-1.207	0.0548	A2	0.0456	0.1092	0.0392	-1.207	0.0548	TRUE	TRUE	TRUE	TRUE	TRUE
1	KLPDDFMGC	411	419	9	HLA-A*02:01	411	0.3954	0.9463	0.0335	0.074	0.955	A2	0.3954	0.9463	0.0335	0.074	0.955	TRUE	TRUE	TRUE	TRUE	TRUE
1	LPDDFMGCV	412	420	9	HLA-A*02:01	412	0.0596	0.1427	0.9876	-0.061	0.2878	A2	0.0596	0.1427	0.9876	-0.061	0.2878	TRUE	TRUE	TRUE	TRUE	TRUE
1	PDDFMGCVL	413	421	9	HLA-A*02:01	413	0.0211	0.0505	0.772	0.039	0.1683	A2	0.0211	0.0505	0.772	0.039	0.1683	TRUE	TRUE	TRUE	TRUE	TRUE
1	DDFMGCVLA	414	422	9	HLA-A*02:01	414	0.0181	0.0432	0.1242	-1.192	0.0022	A2	0.0181	0.0432	0.1242	-1.192	0.0022	TRUE	TRUE	TRUE	TRUE	TRUE
1	DFMGCVLAW	415	423	9	HLA-A*02:01	415	0.021	0.0502	0.2681	0.662	0.1235	A2	0.021	0.0502	0.2681	0.662	0.1235	TRUE	TRUE	TRUE	TRUE	TRUE
1	FMGCVLAWN	416	424	9	HLA-A*02:01	416	0.1274	0.3048	0.01	-1.591	0.2268	A2	0.1274	0.3048	0.01	-1.591	0.2268	TRUE	TRUE	TRUE	TRUE	TRUE
1	MGCVLAWNT	417	425	9	HLA-A*02:01	417	0.0291	0.0697	0.0094	-1.021	0.0201	A2	0.0291	0.0697	0.0094	-1.021	0.0201	TRUE	TRUE	TRUE	TRUE	TRUE
1	GCVLAWNTR	418	426	9	HLA-A*02:01	418	0.0185	0.0444	0.4417	1.44	0.1826	A2	0.0185	0.0444	0.4417	1.44	0.1826	TRUE	TRUE	TRUE	TRUE	TRUE
1	CVLAWNTRN	419	427	9	HLA-A*02:01	419	0.0377	0.0901	0.0112	-1.23	0.0303	A2	0.0377	0.0901	0.0112	-1.23	0.0303	TRUE	TRUE	TRUE	TRUE	TRUE
1	VLAWNTRNI	420	428	9	HLA-A*02:01	420	0.4221	1.0102	0.551	0.626	1.1242	A2	0.4221	1.0102	0.551	0.626	1.1242	TRUE	TRUE	TRUE	TRUE	TRUE
1	LAWNTRNID	421	429	9	HLA-A*02:01	421	0.0244	0.0585	0.1591	-1.54	0.0054	A2	0.0244	0.0585	0.1591	-1.54	0.0054	TRUE	TRUE	TRUE	TRUE	TRUE
1	AWNTRNIDA	422	430	9	HLA-A*02:01	422	0.025	0.0598	0.0522	-0.191	0.0581	A2	0.025	0.0598	0.0522	-0.191	0.0581	TRUE	TRUE	TRUE	TRUE	TRUE
1	WNTRNIDAT	423	431	9	HLA-A*02:01	423	0.0307	0.0734	0.0099	-0.767	0.0366	A2	0.0307	0.0734	0.0099	-0.767	0.0366	TRUE	TRUE	TRUE	TRUE	TRUE
1	NTRNIDATS	424	432	9	HLA-A*02:01	424	0.0231	0.0553	0.0179	-2.237	-0.0539	A2	0.0231	0.0553	0.0179	-2.237	-0.0539	TRUE	TRUE	TRUE	TRUE	TRUE
1	TRNIDATST	425	433	9	HLA-A*02:01	425	0.0167	0.04	0.0129	-0.473	0.0183	A2	0.0167	0.04	0.0129	-0.473	0.0183	TRUE	TRUE	TRUE	TRUE	TRUE
1	RNIDATSTG	426	434	9	HLA-A*02:01	426	0.0202	0.0483	0.0403	-1.225	-0.0069	A2	0.0202	0.0483	0.0403	-1.225	-0.0069	TRUE	TRUE	TRUE	TRUE	TRUE
1	NIDATSTGN	427	435	9	HLA-A*02:01	427	0.0274	0.0655	0.0124	-1.194	0.0077	A2	0.0274	0.0655	0.0124	-1.194	0.0077	TRUE	TRUE	TRUE	TRUE	TRUE
1	IDATSTGNY	428	436	9	HLA-A*02:01	428	0.0176	0.0422	0.5338	2.798	0.2622	A2	0.0176	0.0422	0.5338	2.798	0.2622	TRUE	TRUE	TRUE	TRUE	TRUE
1	DATSTGNYN	429	437	9	HLA-A*02:01	429	0.0181	0.0434	0.0099	-1.606	-0.0354	A2	0.0181	0.0434	0.0099	-1.606	-0.0354	TRUE	TRUE	TRUE	TRUE	TRUE
1	ATSTGNYNY	430	438	9	HLA-A*02:01	430	0.034	0.0815	0.9973	2.904	0.3763	A2	0.034	0.0815	0.9973	2.904	0.3763	TRUE	TRUE	TRUE	TRUE	TRUE
1	TSTGNYNYK	431	439	9	HLA-A*02:01	431	0.0278	0.0664	0.9932	0.602	0.2455	A2	0.0278	0.0664	0.9932	0.602	0.2455	TRUE	TRUE	TRUE	TRUE	TRUE
1	STGNYNYKY	432	440	9	HLA-A*02:01	432	0.0219	0.0523	0.9985	2.803	0.3423	A2	0.0219	0.0523	0.9985	2.803	0.3423	TRUE	TRUE	TRUE	TRUE	TRUE
1	TGNYNYKYR	433	441	9	HLA-A*02:01	433	0.0156	0.0374	0.0806	1.157	0.1073	A2	0.0156	0.0374	0.0806	1.157	0.1073	TRUE	TRUE	TRUE	TRUE	TRUE
1	GNYNYKYRY	434	442	9	HLA-A*02:01	434	0.0181	0.0432	0.9985	2.79	0.3325	A2	0.0181	0.0432	0.9985	2.79	0.3325	TRUE	TRUE	TRUE	TRUE	TRUE
1	NYNYKYRYL	435	443	9	HLA-A*02:01	435	0.0258	0.0618	0.5313	1.132	0.198	A2	0.0258	0.0618	0.5313	1.132	0.198	TRUE	TRUE	TRUE	TRUE	TRUE
1	YNYKYRYLR	436	444	9	HLA-A*02:01	436	0.0258	0.0618	0.6426	1.64	0.2401	A2	0.0258	0.0618	0.6426	1.64	0.2401	TRUE	TRUE	TRUE	TRUE	TRUE
1	NYKYRYLRH	437	445	9	HLA-A*02:01	437	0.017	0.0407	0.0469	-0.288	0.0333	A2	0.017	0.0407	0.0469	-0.288	0.0333	TRUE	TRUE	TRUE	TRUE	TRUE
1	YKYRYLRHG	438	446	9	HLA-A*02:01	438	0.0318	0.0762	0.1271	-1.038	0.0433	A2	0.0318	0.0762	0.1271	-1.038	0.0433	TRUE	TRUE	TRUE	TRUE	TRUE
1	KYRYLRHGK	439	447	9	HLA-A*02:01	439	0.0237	0.0568	0.1439	0.833	0.1201	A2	0.0237	0.0568	0.1439	0.833	0.1201	TRUE	TRUE	TRUE	TRUE	TRUE
1	YRYLRHGKL	440	448	9	HLA-A*02:01	440	0.0273	0.0654	0.9986	1.429	0.2867	A2	0.0273	0.0654	0.9986	1.429	0.2867	TRUE	TRUE	TRUE	TRUE	TRUE
1	RYLRHGKLR	441	449	9	HLA-A*02:01	441	0.0194	0.0465	0.0806	1.959	0.1566	A2	0.0194	0.0465	0.0806	1.959	0.1566	TRUE	TRUE	TRUE	TRUE	TRUE
1	YLRHGKLRP	442	450	9	HLA-A*02:01	442	0.1451	0.3473	0.1262	0.285	0.3804	A2	0.1451	0.3473	0.1262	0.285	0.3804	TRUE	TRUE	TRUE	TRUE	TRUE
1	LRHGKLRPF	443	451	9	HLA-A*02:01	443	0.0221	0.0528	0.8739	2.723	0.32	A2	0.0221	0.0528	0.8739	2.723	0.32	TRUE	TRUE	TRUE	TRUE	TRUE
1	RHGKLRPFE	444	452	9	HLA-A*02:01	444	0.0164	0.0392	0.0095	-1.429	-0.0308	A2	0.0164	0.0392	0.0095	-1.429	-0.0308	TRUE	TRUE	TRUE	TRUE	TRUE
1	HGKLRPFER	445	453	9	HLA-A*02:01	445	0.0159	0.038	0.5656	1.257	0.1857	A2	0.0159	0.038	0.5656	1.257	0.1857	TRUE	TRUE	TRUE	TRUE	TRUE
1	GKLRPFERD	446	454	9	HLA-A*02:01	446	0.02	0.0479	0.015	-1.95	-0.0474	A2	0.02	0.0479	0.015	-1.95	-0.0474	TRUE	TRUE	TRUE	TRUE	TRUE
1	KLRPFERDI	447	455	9	HLA-A*02:01	447	0.1873	0.4482	0.9446	0.68	0.6239	A2	0.1873	0.4482	0.9446	0.68	0.6239	TRUE	TRUE	TRUE	TRUE	TRUE
1	LRPFERDIS	448	456	9	HLA-A*02:01	448	0.0169	0.0405	0.0118	-1.949	-0.0552	A2	0.0169	0.0405	0.0118	-1.949	-0.0552	TRUE	TRUE	TRUE	TRUE	TRUE
1	RPFERDISN	449	457	9	HLA-A*02:01	449	0.0175	0.0418	0.3175	-1.216	0.0286	A2	0.0175	0.0418	0.3175	-1.216	0.0286	TRUE	TRUE	TRUE	TRUE	TRUE
1	PFERDISNV	450	458	9	HLA-A*02:01	450	0.1027	0.2459	0.998	-0.01	0.3951	A2	0.1027	0.2459	0.998	-0.01	0.3951	TRUE	TRUE	TRUE	TRUE	TRUE
1	FERDISNVP	451	459	9	HLA-A*02:01	451	0.032	0.0766	0.7547	-0.222	0.1787	A2	0.032	0.0766	0.7547	-0.222	0.1787	TRUE	TRUE	TRUE	TRUE	TRUE
1	ERDISNVPF	452	460	9	HLA-A*02:01	452	0.0161	0.0386	0.9983	2.256	0.3012	A2	0.0161	0.0386	0.9983	2.256	0.3012	TRUE	TRUE	TRUE	TRUE	TRUE
1	RDISNVPFS	453	461	9	HLA-A*02:01	453	0.0212	0.0507	0.0098	-2.407	-0.0682	A2	0.0212	0.0507	0.0098	-2.407	-0.0682	TRUE	TRUE	TRUE	TRUE	TRUE
1	DISNVPFSP	454	462	9	HLA-A*02:01	454	0.0306	0.0732	0.7747	0.045	0.1916	A2	0.0306	0.0732	0.7747	0.045	0.1916	TRUE	TRUE	TRUE	TRUE	TRUE
1	ISNVPFSPD	455	463	9	HLA-A*02:01	455	0.0161	0.0386	0.0622	-1.985	-0.0513	A2	0.0161	0.0386	0.0622	-1.985	-0.0513	TRUE	TRUE	TRUE	TRUE	TRUE
1	SNVPFSPDG	456	464	9	HLA-A*02:01	456	0.0182	0.0437	0.2033	-1.408	0.0038	A2	0.0182	0.0437	0.2033	-1.408	0.0038	TRUE	TRUE	TRUE	TRUE	TRUE
1	NVPFSPDGK	457	465	9	HLA-A*02:01	457	0.022	0.0528	0.6255	0.679	0.1805	A2	0.022	0.0528	0.6255	0.679	0.1805	TRUE	TRUE	TRUE	TRUE	TRUE
1	VPFSPDGKP	458	466	9	HLA-A*02:01	458	0.0207	0.0497	0.7492	0.115	0.1678	A2	0.0207	0.0497	0.7492	0.115	0.1678	TRUE	TRUE	TRUE	TRUE	TRUE
1	PFSPDGKPC	459	467	9	HLA-A*02:01	459	0.0498	0.1192	0.0098	-0.255	0.1079	A2	0.0498	0.1192	0.0098	-0.255	0.1079	TRUE	TRUE	TRUE	TRUE	TRUE
1	FSPDGKPCT	460	468	9	HLA-A*02:01	460	0.0636	0.1522	0.0108	-0.92	0.1079	A2	0.0636	0.1522	0.0108	-0.92	0.1079	TRUE	TRUE	TRUE	TRUE	TRUE
1	SPDGKPCTP	461	469	9	HLA-A*02:01	461	0.0167	0.0401	0.1562	-0.428	0.0421	A2	0.0167	0.0401	0.1562	-0.428	0.0421	TRUE	TRUE	TRUE	TRUE	TRUE
1	PDGKPCTPP	462	470	9	HLA-A*02:01	462	0.0157	0.0375	0.0698	-0.792	0.0083	A2	0.0157	0.0375	0.0698	-0.792	0.0083	TRUE	TRUE	TRUE	TRUE	TRUE
1	DGKPCTPPA	463	471	9	HLA-A*02:01	463	0.0197	0.0471	0.2817	-1.265	0.0261	A2	0.0197	0.0471	0.2817	-1.265	0.0261	TRUE	TRUE	TRUE	TRUE	TRUE
1	GKPCTPPAL	464	472	9	HLA-A*02:01	464	0.0213	0.0509	0.9431	0.671	0.2259	A2	0.0213	0.0509	0.9431	0.671	0.2259	TRUE	TRUE	TRUE	TRUE	TRUE
1	KPCTPPALN	465	473	9	HLA-A*02:01	465	0.0151	0.0362	0.1894	-1.534	-0.0121	A2	0.0151	0.0362	0.1894	-1.534	-0.0121	TRUE	TRUE	TRUE	TRUE	TRUE
1	PCTPPALNC	466	474	9	HLA-A*02:01	466	0.0553	0.1323	0.0107	-0.38	0.1149	A2	0.0553	0.1323	0.0107	-0.38	0.1149	TRUE	TRUE	TRUE	TRUE	TRUE
1	CTPPALNCY	467	475	9	HLA-A*02:01	467	0.0296	0.0709	0.849	2.702	0.3333	A2	0.0296	0.0709	0.849	2.702	0.3333	TRUE	TRUE	TRUE	TRUE	TRUE
1	TPPALNCYW	468	476	9	HLA-A*02:01	468	0.0165	0.0394	0.9388	0.537	0.2071	A2	0.0165	0.0394	0.9388	0.537	0.2071	TRUE	TRUE	TRUE	TRUE	TRUE
1	PPALNCYWP	469	477	9	HLA-A*02:01	469	0.019	0.0454	0.0173	-0.653	0.0154	A2	0.019	0.0454	0.0173	-0.653	0.0154	TRUE	TRUE	TRUE	TRUE	TRUE
1	PALNCYWPL	470	478	9	HLA-A*02:01	470	0.0731	0.1749	0.4815	0.4	0.2672	A2	0.0731	0.1749	0.4815	0.4	0.2672	TRUE	TRUE	TRUE	TRUE	TRUE
1	ALNCYWPLN	471	479	9	HLA-A*02:01	471	0.0406	0.0972	0.0109	-1.391	0.0293	A2	0.0406	0.0972	0.0109	-1.391	0.0293	TRUE	TRUE	TRUE	TRUE	TRUE
1	LNCYWPLND	472	480	9	HLA-A*02:01	472	0.0173	0.0414	0.0095	-1.801	-0.0472	A2	0.0173	0.0414	0.0095	-1.801	-0.0472	TRUE	TRUE	TRUE	TRUE	TRUE
1	NCYWPLNDY	473	481	9	HLA-A*02:01	473	0.0324	0.0775	0.9798	3.217	0.3853	A2	0.0324	0.0775	0.9798	3.217	0.3853	TRUE	TRUE	TRUE	TRUE	TRUE
1	CYWPLNDYG	474	482	9	HLA-A*02:01	474	0.0439	0.105	0.0212	-0.859	0.0652	A2	0.0439	0.105	0.0212	-0.859	0.0652	TRUE	TRUE	TRUE	TRUE	TRUE
1	YWPLNDYGF	475	483	9	HLA-A*02:01	475	0.0234	0.056	0.4377	2.739	0.2586	A2	0.0234	0.056	0.4377	2.739	0.2586	TRUE	TRUE	TRUE	TRUE	TRUE
1	WPLNDYGFY	476	484	9	HLA-A*02:01	476	0.024	0.0573	0.9946	2.559	0.3345	A2	0.024	0.0573	0.9946	2.559	0.3345	TRUE	TRUE	TRUE	TRUE	TRUE
1	PLNDYGFYT	477	485	9	HLA-A*02:01	477	0.4345	1.0398	0.3669	-1.316	1.029	A2	0.4345	1.0398	0.3669	-1.316	1.029	TRUE	TRUE	TRUE	TRUE	TRUE
1	LNDYGFYTT	478	486	9	HLA-A*02:01	478	0.0231	0.0554	0.049	-1.175	0.004	A2	0.0231	0.0554	0.049	-1.175	0.004	TRUE	TRUE	TRUE	TRUE	TRUE
1	NDYGFYTTT	479	487	9	HLA-A*02:01	479	0.0207	0.0495	0.5676	-0.741	0.0976	A2	0.0207	0.0495	0.5676	-0.741	0.0976	TRUE	TRUE	TRUE	TRUE	TRUE
1	DYGFYTTTG	480	488	9	HLA-A*02:01	480	0.0156	0.0373	0.1394	-1.469	-0.0153	A2	0.0156	0.0373	0.1394	-1.469	-0.0153	TRUE	TRUE	TRUE	TRUE	TRUE
1	YGFYTTTGI	481	489	9	HLA-A*02:01	481	0.0657	0.1572	0.3643	0.221	0.2229	A2	0.0657	0.1572	0.3643	0.221	0.2229	TRUE	TRUE	TRUE	TRUE	TRUE
1	GFYTTTGIG	482	490	9	HLA-A*02:01	482	0.0273	0.0654	0.0209	-1.272	0.0049	A2	0.0273	0.0654	0.0209	-1.272	0.0049	TRUE	TRUE	TRUE	TRUE	TRUE
1	FYTTTGIGY	483	491	9	HLA-A*02:01	483	0.037	0.0885	0.8012	3.028	0.36	A2	0.037	0.0885	0.8012	3.028	0.36	TRUE	TRUE	TRUE	TRUE	TRUE

seq #	peptide	start	end	peptide length	allele	peptide index	NetCTL Predict	NetCTL Rescale	NetCTL c termi	NetCTL TAP tra	NetCTL Predict	NetCTL Superty	legacy predicte	legacy rescale_	legacy c_termi	legacy tap_tra	legacy predicti	equal predicte	equal rescale_	equal c_termi	equal tap_tran	equal predictions_score
1	YTTTGIGYQ	484	492	9	HLA-A*02:01	484	0.08	0.1915	0.0103	-0.288	0.1787	A2	0.08	0.1915	0.0103	-0.288	0.1787	TRUE	TRUE	TRUE	TRUE	TRUE
1	TTTGIGYQP	485	493	9	HLA-A*02:01	485	0.0455	0.109	0.0105	-0.026	0.1093	A2	0.0455	0.109	0.0105	-0.026	0.1093	TRUE	TRUE	TRUE	TRUE	TRUE
1	TTGIGYQPY	486	494	9	HLA-A*02:01	486	0.0206	0.0492	0.8089	2.729	0.307	A2	0.0206	0.0492	0.8089	2.729	0.307	TRUE	TRUE	TRUE	TRUE	TRUE
1	TGIGYQPYR	487	495	9	HLA-A*02:01	487	0.0223	0.0534	0.0212	1.276	0.1204	A2	0.0223	0.0534	0.0212	1.276	0.1204	TRUE	TRUE	TRUE	TRUE	TRUE
1	GIGYQPYRV	488	496	9	HLA-A*02:01	488	0.1196	0.2861	0.3346	0.111	0.3419	A2	0.1196	0.2861	0.3346	0.111	0.3419	TRUE	TRUE	TRUE	TRUE	TRUE
1	IGYQPYRVV	489	497	9	HLA-A*02:01	489	0.0644	0.1541	0.6602	0.228	0.2645	A2	0.0644	0.1541	0.6602	0.228	0.2645	TRUE	TRUE	TRUE	TRUE	TRUE
1	GYQPYRVVV	490	498	9	HLA-A*02:01	490	0.1123	0.2689	0.9643	0.377	0.4324	A2	0.1123	0.2689	0.9643	0.377	0.4324	TRUE	TRUE	TRUE	TRUE	TRUE
1	YQPYRVVVL	491	499	9	HLA-A*02:01	491	0.0392	0.0938	0.9925	0.965	0.291	A2	0.0392	0.0938	0.9925	0.965	0.291	TRUE	TRUE	TRUE	TRUE	TRUE
1	QPYRVVVLS	492	500	9	HLA-A*02:01	492	0.0197	0.0472	0.3782	-2.402	-0.0161	A2	0.0197	0.0472	0.3782	-2.402	-0.0161	TRUE	TRUE	TRUE	TRUE	TRUE
1	PYRVVVLSF	493	501	9	HLA-A*02:01	493	0.0196	0.0469	0.9846	2.319	0.3105	A2	0.0196	0.0469	0.9846	2.319	0.3105	TRUE	TRUE	TRUE	TRUE	TRUE
1	YRVVVLSFE	494	502	9	HLA-A*02:01	494	0.0191	0.0456	0.0112	-1.446	-0.025	A2	0.0191	0.0456	0.0112	-1.446	-0.025	TRUE	TRUE	TRUE	TRUE	TRUE
1	RVVVLSFEL	495	503	9	HLA-A*02:01	495	0.1212	0.29	0.9965	1.363	0.5076	A2	0.1212	0.29	0.9965	1.363	0.5076	TRUE	TRUE	TRUE	TRUE	TRUE
1	VVVLSFELL	496	504	9	HLA-A*02:01	496	0.0558	0.1335	0.9401	1.365	0.3428	A2	0.0558	0.1335	0.9401	1.365	0.3428	TRUE	TRUE	TRUE	TRUE	TRUE
1	VVLSFELLN	497	505	9	HLA-A*02:01	497	0.0304	0.0728	0.0133	-1.05	0.0223	A2	0.0304	0.0728	0.0133	-1.05	0.0223	TRUE	TRUE	TRUE	TRUE	TRUE
1	VLSFELLNA	498	506	9	HLA-A*02:01	498	0.1798	0.4304	0.0766	-0.411	0.4213	A2	0.1798	0.4304	0.0766	-0.411	0.4213	TRUE	TRUE	TRUE	TRUE	TRUE
1	LSFELLNAP	499	507	9	HLA-A*02:01	499	0.057	0.1364	0.0274	0.308	0.1559	A2	0.057	0.1364	0.0274	0.308	0.1559	TRUE	TRUE	TRUE	TRUE	TRUE
1	SFELLNAPA	500	508	9	HLA-A*02:01	500	0.0312	0.0747	0.4319	-0.411	0.1189	A2	0.0312	0.0747	0.4319	-0.411	0.1189	TRUE	TRUE	TRUE	TRUE	TRUE
1	FELLNAPAT	501	509	9	HLA-A*02:01	501	0.0281	0.0673	0.0186	-0.864	0.0269	A2	0.0281	0.0673	0.0186	-0.864	0.0269	TRUE	TRUE	TRUE	TRUE	TRUE
1	ELLNAPATV	502	510	9	HLA-A*02:01	502	0.315	0.7538	0.999	0.043	0.9058	A2	0.315	0.7538	0.999	0.043	0.9058	TRUE	TRUE	TRUE	TRUE	TRUE
1	LLNAPATVC	503	511	9	HLA-A*02:01	503	0.1721	0.4118	0.0135	-0.223	0.4027	A2	0.1721	0.4118	0.0135	-0.223	0.4027	TRUE	TRUE	TRUE	TRUE	TRUE
1	LNAPATVCG	504	512	9	HLA-A*02:01	504	0.0189	0.0453	0.0124	-1.479	-0.0268	A2	0.0189	0.0453	0.0124	-1.479	-0.0268	TRUE	TRUE	TRUE	TRUE	TRUE
1	NAPATVCGP	505	513	9	HLA-A*02:01	505	0.0193	0.0461	0.0142	0.227	0.0596	A2	0.0193	0.0461	0.0142	0.227	0.0596	TRUE	TRUE	TRUE	TRUE	TRUE
1	APATVCGPK	506	514	9	HLA-A*02:01	506	0.0195	0.0466	0.9809	0.462	0.2169	A2	0.0195	0.0466	0.9809	0.462	0.2169	TRUE	TRUE	TRUE	TRUE	TRUE
1	PATVCGPKL	507	515	9	HLA-A*02:01	507	0.0285	0.0683	0.9056	0.645	0.2364	A2	0.0285	0.0683	0.9056	0.645	0.2364	TRUE	TRUE	TRUE	TRUE	TRUE
1	ATVCGPKLS	508	516	9	HLA-A*02:01	508	0.0235	0.0562	0.0098	-2.19	-0.0518	A2	0.0235	0.0562	0.0098	-2.19	-0.0518	TRUE	TRUE	TRUE	TRUE	TRUE
1	TVCGPKLST	509	517	9	HLA-A*02:01	509	0.0617	0.1476	0.0233	-0.425	0.1299	A2	0.0617	0.1476	0.0233	-0.425	0.1299	TRUE	TRUE	TRUE	TRUE	TRUE
1	VCGPKLSTD	510	518	9	HLA-A*02:01	510	0.0226	0.054	0.0114	-1.886	-0.0385	A2	0.0226	0.054	0.0114	-1.886	-0.0385	TRUE	TRUE	TRUE	TRUE	TRUE
1	CGPKLSTDL	511	519	9	HLA-A*02:01	511	0.0237	0.0567	0.111	0.679	0.1073	A2	0.0237	0.0567	0.111	0.679	0.1073	TRUE	TRUE	TRUE	TRUE	TRUE
1	GPKLSTDLI	512	520	9	HLA-A*02:01	512	0.0198	0.0475	0.6234	0.042	0.1431	A2	0.0198	0.0475	0.6234	0.042	0.1431	TRUE	TRUE	TRUE	TRUE	TRUE
1	PKLSTDLIK	513	521	9	HLA-A*02:01	513	0.0184	0.0441	0.3525	0.022	0.0981	A2	0.0184	0.0441	0.3525	0.022	0.0981	TRUE	TRUE	TRUE	TRUE	TRUE
1	KLSTDLIKN	514	522	9	HLA-A*02:01	514	0.0605	0.1447	0.0262	-1.338	0.0817	A2	0.0605	0.1447	0.0262	-1.338	0.0817	TRUE	TRUE	TRUE	TRUE	TRUE
1	LSTDLIKNQ	515	523	9	HLA-A*02:01	515	0.0321	0.0769	0.0244	0.004	0.0808	A2	0.0321	0.0769	0.0244	0.004	0.0808	TRUE	TRUE	TRUE	TRUE	TRUE
1	STDLIKNQC	516	524	9	HLA-A*02:01	516	0.0251	0.0601	0.0105	-0.082	0.0576	A2	0.0251	0.0601	0.0105	-0.082	0.0576	TRUE	TRUE	TRUE	TRUE	TRUE
1	TDLIKNQCV	517	525	9	HLA-A*02:01	517	0.0282	0.0676	0.8403	0.058	0.1965	A2	0.0282	0.0676	0.8403	0.058	0.1965	TRUE	TRUE	TRUE	TRUE	TRUE
1	DLIKNQCVN	518	526	9	HLA-A*02:01	518	0.0382	0.0913	0.0107	-1.533	0.0163	A2	0.0382	0.0913	0.0107	-1.533	0.0163	TRUE	TRUE	TRUE	TRUE	TRUE
1	LIKNQCVNF	519	527	9	HLA-A*02:01	519	0.0598	0.1432	0.9956	2.526	0.4188	A2	0.0598	0.1432	0.9956	2.526	0.4188	TRUE	TRUE	TRUE	TRUE	TRUE
1	IKNQCVNFN	520	528	9	HLA-A*02:01	520	0.0189	0.0452	0.0097	-1.289	-0.0178	A2	0.0189	0.0452	0.0097	-1.289	-0.0178	TRUE	TRUE	TRUE	TRUE	TRUE
1	KNQCVNFNF	521	529	9	HLA-A*02:01	521	0.0215	0.0514	0.8505	2.466	0.3023	A2	0.0215	0.0514	0.8505	2.466	0.3023	TRUE	TRUE	TRUE	TRUE	TRUE
1	NQCVNFNFN	522	530	9	HLA-A*02:01	522	0.0158	0.0379	0.0123	-1.093	-0.015	A2	0.0158	0.0379	0.0123	-1.093	-0.015	TRUE	TRUE	TRUE	TRUE	TRUE
1	QCVNFNFNG	523	531	9	HLA-A*02:01	523	0.0277	0.0662	0.0118	-1.143	0.0108	A2	0.0277	0.0662	0.0118	-1.143	0.0108	TRUE	TRUE	TRUE	TRUE	TRUE
1	CVNFNFNGL	524	532	9	HLA-A*02:01	524	0.0528	0.1264	0.9901	0.909	0.3204	A2	0.0528	0.1264	0.9901	0.909	0.3204	TRUE	TRUE	TRUE	TRUE	TRUE
1	VNFNFNGLT	525	533	9	HLA-A*02:01	525	0.0235	0.0562	0.0446	-0.691	0.0284	A2	0.0235	0.0562	0.0446	-0.691	0.0284	TRUE	TRUE	TRUE	TRUE	TRUE
1	NFNFNGLTG	526	534	9	HLA-A*02:01	526	0.0184	0.044	0.0287	-1.238	-0.0136	A2	0.0184	0.044	0.0287	-1.238	-0.0136	TRUE	TRUE	TRUE	TRUE	TRUE
1	FNFNGLTGT	527	535	9	HLA-A*02:01	527	0.0441	0.1055	0.0382	-0.7	0.0762	A2	0.0441	0.1055	0.0382	-0.7	0.0762	TRUE	TRUE	TRUE	TRUE	TRUE
1	NFNGLTGTG	528	536	9	HLA-A*02:01	528	0.023	0.055	0.073	-1.39	-0.0036	A2	0.023	0.055	0.073	-1.39	-0.0036	TRUE	TRUE	TRUE	TRUE	TRUE
1	FNGLTGTGV	529	537	9	HLA-A*02:01	529	0.0304	0.0727	0.0998	0.045	0.0899	A2	0.0304	0.0727	0.0998	0.045	0.0899	TRUE	TRUE	TRUE	TRUE	TRUE
1	NGLTGTGVL	530	538	9	HLA-A*02:01	530	0.0218	0.0522	0.9959	0.638	0.2335	A2	0.0218	0.0522	0.9959	0.638	0.2335	TRUE	TRUE	TRUE	TRUE	TRUE
1	GLTGTGVLT	531	539	9	HLA-A*02:01	531	0.2168	0.519	0.4931	-0.76	0.5549	A2	0.2168	0.519	0.4931	-0.76	0.5549	TRUE	TRUE	TRUE	TRUE	TRUE
1	LTGTGVLTP	532	540	9	HLA-A*02:01	532	0.0624	0.1493	0.0927	-0.106	0.1579	A2	0.0624	0.1493	0.0927	-0.106	0.1579	TRUE	TRUE	TRUE	TRUE	TRUE
1	TGTGVLTPS	533	541	9	HLA-A*02:01	533	0.0219	0.0524	0.0492	-2.521	-0.0663	A2	0.0219	0.0524	0.0492	-2.521	-0.0663	TRUE	TRUE	TRUE	TRUE	TRUE
1	GTGVLTPSS	534	542	9	HLA-A*02:01	534	0.0341	0.0815	0.0327	-2.599	-0.0436	A2	0.0341	0.0815	0.0327	-2.599	-0.0436	TRUE	TRUE	TRUE	TRUE	TRUE
1	TGVLTPSSK	535	543	9	HLA-A*02:01	535	0.0147	0.0353	0.9752	0.225	0.1928	A2	0.0147	0.0353	0.9752	0.225	0.1928	TRUE	TRUE	TRUE	TRUE	TRUE
1	GVLTPSSKR	536	544	9	HLA-A*02:01	536	0.0392	0.0938	0.9836	1.472	0.315	A2	0.0392	0.0938	0.9836	1.472	0.315	TRUE	TRUE	TRUE	TRUE	TRUE
1	VLTPSSKRF	537	545	9	HLA-A*02:01	537	0.1718	0.4112	0.9965	2.55	0.6881	A2	0.1718	0.4112	0.9965	2.55	0.6881	TRUE	TRUE	TRUE	TRUE	TRUE
1	LTPSSKRFQ	538	546	9	HLA-A*02:01	538	0.0295	0.0705	0.0098	-0.091	0.0675	A2	0.0295	0.0705	0.0098	-0.091	0.0675	TRUE	TRUE	TRUE	TRUE	TRUE
1	TPSSKRFQP	539	547	9	HLA-A*02:01	539	0.0181	0.0433	0.0331	-0.177	0.0394	A2	0.0181	0.0433	0.0331	-0.177	0.0394	TRUE	TRUE	TRUE	TRUE	TRUE
1	PSSKRFQPF	540	548	9	HLA-A*02:01	540	0.0195	0.0468	0.9739	2.101	0.2979	A2	0.0195	0.0468	0.9739	2.101	0.2979	TRUE	TRUE	TRUE	TRUE	TRUE
1	SSKRFQPFQ	541	549	9	HLA-A*02:01	541	0.0206	0.0494	0.0282	-0.159	0.0456	A2	0.0206	0.0494	0.0282	-0.159	0.0456	TRUE	TRUE	TRUE	TRUE	TRUE
1	SKRFQPFQQ	542	550	9	HLA-A*02:01	542	0.0177	0.0423	0.0895	0.1	0.0607	A2	0.0177	0.0423	0.0895	0.1	0.0607	TRUE	TRUE	TRUE	TRUE	TRUE
1	KRFQPFQQF	543	551	9	HLA-A*02:01	543	0.0229	0.0549	0.9987	3.014	0.3554	A2	0.0229	0.0549	0.9987	3.014	0.3554	TRUE	TRUE	TRUE	TRUE	TRUE
1	RFQPFQQFG	544	552	9	HLA-A*02:01	544	0.0364	0.087	0.0125	-0.915	0.0431	A2	0.0364	0.087	0.0125	-0.915	0.0431	TRUE	TRUE	TRUE	TRUE	TRUE
1	FQPFQQFGR	545	553	9	HLA-A*02:01	545	0.0248	0.0593	0.6327	1.582	0.2333	A2	0.0248	0.0593	0.6327	1.582	0.2333	TRUE	TRUE	TRUE	TRUE	TRUE
1	QPFQQFGRD	546	554	9	HLA-A*02:01	546	0.0169	0.0405	0.4628	-2.1	0.0049	A2	0.0169	0.0405	0.4628	-2.1	0.0049	TRUE	TRUE	TRUE	TRUE	TRUE
1	PFQQFGRDV	547	555	9	HLA-A*02:01	547	0.1234	0.2954	0.253	-0.079	0.3294	A2	0.1234	0.2954	0.253	-0.079	0.3294	TRUE	TRUE	TRUE	TRUE	TRUE
1	FQQFGRDVS	548	556	9	HLA-A*02:01	548	0.0307	0.0735	0.0101	-2.442	-0.0471	A2	0.0307	0.0735	0.0101	-2.442	-0.0471	TRUE	TRUE	TRUE	TRUE	TRUE
1	QQFGRDVSD	549	557	9	HLA-A*02:01	549	0.0192	0.0459	0.0395	-1.72	-0.0342	A2	0.0192	0.0459	0.0395	-1.72	-0.0342	TRUE	TRUE	TRUE	TRUE	TRUE
1	QFGRDVSDF	550	558	9	HLA-A*02:01	550	0.0244	0.0583	0.0834	2.503	0.196	A2	0.0244	0.0583	0.0834	2.503	0.196	TRUE	TRUE	TRUE	TRUE	TRUE
1	FGRDVSDFT	551	559	9	HLA-A*02:01	551	0.0642	0.1536	0.0147	-1.067	0.1024	A2	0.0642	0.1536	0.0147	-1.067	0.1024	TRUE	TRUE	TRUE	TRUE	TRUE
1	GRDVSDFTD	552	560	9	HLA-A*02:01	552	0.0167	0.04	0.7493	-2.069	0.049	A2	0.0167	0.04	0.7493	-2.069	0.049	TRUE	TRUE	TRUE	TRUE	TRUE
1	RDVSDFTDS	553	561	9	HLA-A*02:01	553	0.0183	0.0438	0.0643	-2.261	-0.0596	A2	0.0183	0.0438	0.0643	-2.261	-0.0596	TRUE	TRUE	TRUE	TRUE	TRUE
1	DVSDFTDSV	554	562	9	HLA-A*02:01	554	0.1985	0.475	0.9986	0.265	0.6381	A2	0.1985	0.475	0.9986	0.265	0.6381	TRUE	TRUE	TRUE	TRUE	TRUE
1	VSDFTDSVR	555	563	9	HLA-A*02:01	555	0.0171	0.0409	0.1787	1.272	0.1313	A2	0.0171	0.0409	0.1787	1.272	0.1313	TRUE	TRUE	TRUE	TRUE	TRUE
1	SDFTDSVRD	556	564	9	HLA-A*02:01	556	0.017	0.0408	0.0178	-1.911	-0.0521	A2	0.017	0.0408	0.0178	-1.911	-0.0521	TRUE	TRUE	TRUE	TRUE	TRUE
1	DFTDSVRDP	557	565	9	HLA-A*02:01	557	0.0258	0.0618	0.0196	-0.026	0.0634	A2	0.0258	0.0618	0.0196	-0.026	0.0634	TRUE	TRUE	TRUE	TRUE	TRUE
1	FTDSVRDPK	558	566	9	HLA-A*02:01	558	0.0416	0.0996	0.0834	0.033	0.1137	A2	0.0416	0.0996	0.0834	0.033	0.1137	TRUE	TRUE	TRUE	TRUE	TRUE
1	TDSVRDPKT	559	567	9	HLA-A*02:01	559	0.017	0.0407	0.0246	-1.143	-0.0127	A2	0.017	0.0407	0.0246	-1.143	-0.0127	TRUE	TRUE	TRUE	TRUE	TRUE
1	DSVRDPKTS	560	568	9	HLA-A*02:01	560	0.0165	0.0394	0.0584	-2.433	-0.0735	A2	0.0165	0.0394	0.0584	-2.433	-0.0735	TRUE	TRUE	TRUE	TRUE	TRUE
1	SVRDPKTSE	561	569	9	HLA-A*02:01	561	0.0374	0.0894	0.0149	-1.464	0.0184	A2	0.0374	0.0894	0.0149	-1.464	0.0184	TRUE	TRUE	TRUE	TRUE	TRUE
1	VRDPKTSEI	562	570	9	HLA-A*02:01	562	0.0437	0.1046	0.9534	0.681	0.2816	A2	0.0437	0.1046	0.9534	0.681	0.2816	TRUE	TRUE	TRUE	TRUE	TRUE
1	RDPKTSEIL	563	571	9	HLA-A*02:01	563	0.0222	0.0531	0.9892	0.923	0.2477	A2	0.0222	0.0531	0.9892	0.923	0.2477	TRUE	TRUE	TRUE	TRUE	TRUE
1	DPKTSEILD	564	572	9	HLA-A*02:01	564	0.0146	0.0349	0.0215	-2.306	-0.0772	A2	0.0146	0.0349	0.0215	-2.306	-0.0772	TRUE	TRUE	TRUE	TRUE	TRUE
1	PKTSEILDI	565	573	9	HLA-A*02:01	565	0.0337	0.0806	0.9816	-0.071	0.2243	A2	0.0337	0.0806	0.9816	-0.071	0.2243	TRUE	TRUE	TRUE	TRUE	TRUE
1	KTSEILDIS	566	574	9	HLA-A*02:01	566	0.0666	0.1593	0.0134	-2.309	0.0458	A2	0.0666	0.1593	0.0134	-2.309	0.0458	TRUE	TRUE	TRUE	TRUE	TRUE
1	TSEILDISP	567	575	9	HLA-A*02:01	567	0.0163	0.039	0.0172	0.081	0.0456	A2	0.0163	0.039	0.0172	0.081	0.0456	TRUE	TRUE	TRUE	TRUE	TRUE
1	SEILDISPC	568	576	9	HLA-A*02:01	568	0.0229	0.0548	0.0177	0.011	0.058	A2	0.0229	0.0548	0.0177	0.011	0.058	TRUE	TRUE	TRUE	TRUE	TRUE
1	EILDISPCS	569	577	9	HLA-A*02:01	569	0.1388	0.3323	0.0135	-2.249	0.2218	A2	0.1388	0.3323	0.0135	-2.249	0.2218	TRUE	TRUE	TRUE	TRUE	TRUE
1	ILDISPCSF	570	578	9	HLA-A*02:01	570	0.091	0.2177	0.9005	2.234	0.4645	A2	0.091	0.2177	0.9005	2.234	0.4645	TRUE	TRUE	TRUE	TRUE	TRUE
1	LDISPCSFG	571	579	9	HLA-A*02:01	571	0.0184	0.0441	0.0157	-1.621	-0.0346	A2	0.0184	0.0441	0.0157	-1.621	-0.0346	TRUE	TRUE	TRUE	TRUE	TRUE
1	DISPCSFGG	572	580	9	HLA-A*02:01	572	0.0517	0.1238	0.0118	-1.539	0.0486	A2	0.0517	0.1238	0.0118	-1.539	0.0486	TRUE	TRUE	TRUE	TRUE	TRUE
1	ISPCSFGGV	573	581	9	HLA-A*02:01	573	0.0366	0.0877	0.846	0.224	0.2258	A2	0.0366	0.0877	0.846	0.224	0.2258	TRUE	TRUE	TRUE	TRUE	TRUE
1	SPCSFGGVS	574	582	9	HLA-A*02:01	574	0.0175	0.0418	0.0557	-2.479	-0.0738	A2	0.0175	0.0418	0.0557	-2.479	-0.0738	TRUE	TRUE	TRUE	TRUE	TRUE
1	PCSFGGVSV	575	583	9	HLA-A*02:01	575	0.0566	0.1355	0.5765	-0.095	0.2173	A2	0.0566	0.1355	0.5765	-0.095	0.2173	TRUE	TRUE	TRUE	TRUE	TRUE
1	CSFGGVSVI	576	584	9	HLA-A*02:01	576	0.0685	0.1639	0.9808	0.501	0.336	A2	0.0685	0.1639	0.9808	0.501	0.336	TRUE	TRUE	TRUE	TRUE	TRUE
1	SFGGVSVIT	577	585	9	HLA-A*02:01	577	0.0401	0.0959	0.0131	-0.655	0.0651	A2	0.0401	0.0959	0.0131	-0.655	0.0651	TRUE	TRUE	TRUE	TRUE	TRUE
1	FGGVSVITP	578	586	9	HLA-A*02:01	578	0.0188	0.0449	0.0123	-0.366	0.0285	A2	0.0188	0.0449	0.0123	-0.366	0.0285	TRUE	TRUE	TRUE	TRUE	TRUE
1	GGVSVITPG	579	587	9	HLA-A*02:01	579	0.0165	0.0394	0.1953	-1.868	-0.0247	A2	0.0165	0.0394	0.1953	-1.868	-0.0247	TRUE	TRUE	TRUE	TRUE	TRUE
1	GVSVITPGT	580	588	9	HLA-A*02:01	580	0.0548	0.1312	0.3676	-0.78	0.1473	A2	0.0548	0.1312	0.3676	-0.78	0.1473	TRUE	TRUE	TRUE	TRUE	TRUE
1	VSVITPGTN	581	589	9	HLA-A*02:01	581	0.0171	0.0408	0.0161	-1.165	-0.015	A2	0.0171	0.0408	0.0161	-1.165	-0.015	TRUE	TRUE	TRUE	TRUE	TRUE
1	SVITPGTNA	582	590	9	HLA-A*02:01	582	0.0649	0.1552	0.0351	-0.228	0.1491	A2	0.0649	0.1552	0.0351	-0.228	0.1491	TRUE	TRUE	TRUE	TRUE	TRUE
1	VITPGTNAS	583	591	9	HLA-A*02:01	583	0.1611	0.3855	0.0219	-2.037	0.2869	A2	0.1611	0.3855	0.0219	-2.037	0.2869	TRUE	TRUE	TRUE	TRUE	TRUE
1	ITPGTNASS	584	592	9	HLA-A*02:01	584	0.0927	0.2219	0.0175	-2.212	0.114	A2	0.0927	0.2219	0.0175	-2.212	0.114	TRUE	TRUE	TRUE	TRUE	TRUE
1	TPGTNASSE	585	593	9	HLA-A*02:01	585	0.0153	0.0366	0.0117	-2.045	-0.0639	A2	0.0153	0.0366	0.0117	-2.045	-0.0639	TRUE	TRUE	TRUE	TRUE	TRUE
1	PGTNASSEV	586	594	9	HLA-A*02:01	586	0.0357	0.0855	0.9933	-0.458	0.2116	A2	0.0357	0.0855	0.9933	-0.458	0.2116	TRUE	TRUE	TRUE	TRUE	TRUE
1	GTNASSEVA	587	595	9	HLA-A*02:01	587	0.0445	0.1065	0.1187	-0.975	0.0756	A2	0.0445	0.1065	0.1187	-0.975	0.0756	TRUE	TRUE	TRUE	TRUE	TRUE
1	TNASSEVAV	588	596	9	HLA-A*02:01	588	0.0268	0.0642	0.0603	0.128	0.0797	A2	0.0268	0.0642	0.0603	0.128	0.0797	TRUE	TRUE	TRUE	TRUE	TRUE
1	NASSEVAVL	589	597	9	HLA-A*02:01	589	0.0303	0.0724	0.929	1.05	0.2643	A2	0.0303	0.0724	0.929	1.05	0.2643	TRUE	TRUE	TRUE	TRUE	TRUE
1	ASSEVAVLY	590	598	9	HLA-A*02:01	590	0.0232	0.0556	0.9972	3.246	0.3674	A2	0.0232	0.0556	0.9972	3.246	0.3674	TRUE	TRUE	TRUE	TRUE	TRUE
1	SSEVAVLYQ	591	599	9	HLA-A*02:01	591	0.0205	0.0491	0.0284	-0.002	0.0533	A2	0.0205	0.0491	0.0284	-0.002	0.0533	TRUE	TRUE	TRUE	TRUE	TRUE
1	SEVAVLYQD	592	600	9	HLA-A*02:01	592	0.0175	0.0418	0.0136	-1.766	-0.0444	A2	0.0175	0.0418	0.0136	-1.766	-0.0444	TRUE	TRUE	TRUE	TRUE	TRUE
1	EVAVLYQDV	593	601	9	HLA-A*02:01	593	0.1059	0.2534	0.6299	0.32	0.3638	A2	0.1059	0.2534	0.6299	0.32	0.3638	TRUE	TRUE	TRUE	TRUE	TRUE
1	VAVLYQDVN	594	602	9	HLA-A*02:01	594	0.0173	0.0413	0.0125	-1.29	-0.0213	A2	0.0173	0.0413	0.0125	-1.29	-0.0213	TRUE	TRUE	TRUE	TRUE	TRUE
1	AVLYQDVNC	595	603	9	HLA-A*02:01	595	0.0653	0.1562	0.0096	0.362	0.1757	A2	0.0653	0.1562	0.0096	0.362	0.1757	TRUE	TRUE	TRUE	TRUE	TRUE
1	VLYQDVNCT	596	604	9	HLA-A*02:01	596	0.3174	0.7596	0.0208	-0.283	0.7485	A2	0.3174	0.7596	0.0208	-0.283	0.7485	TRUE	TRUE	TRUE	TRUE	TRUE
1	LYQDVNCTD	597	605	9	HLA-A*02:01	597	0.0414	0.099	0.129	-1.532	0.0417	A2	0.0414	0.099	0.129	-1.532	0.0417	TRUE	TRUE	TRUE	TRUE	TRUE
1	YQDVNCTDV	598	606	9	HLA-A*02:01	598	0.0741	0.1774	0.9556	0.242	0.3328	A2	0.0741	0.1774	0.9556	0.242	0.3328	TRUE	TRUE	TRUE	TRUE	TRUE
1	QDVNCTDVS	599	607	9	HLA-A*02:01	599	0.0172	0.0411	0.0097	-2.514	-0.0831	A2	0.0172	0.0411	0.0097	-2.514	-0.0831	TRUE	TRUE	TRUE	TRUE	TRUE
1	DVNCTDVST	600	608	9	HLA-A*02:01	600	0.0229	0.0548	0.0586	-1.026	0.0123	A2	0.0229	0.0548	0.0586	-1.026	0.0123	TRUE	TRUE	TRUE	TRUE	TRUE
1	VNCTDVSTA	601	609	9	HLA-A*02:01	601	0.0218	0.0521	0.3669	-0.756	0.0693	A2	0.0218	0.0521	0.3669	-0.756	0.0693	TRUE	TRUE	TRUE	TRUE	TRUE
1	NCTDVSTAI	602	610	9	HLA-A*02:01	602	0.0659	0.1577	0.9977	0.603	0.3375	A2	0.0659	0.1577	0.9977	0.603	0.3375	TRUE	TRUE	TRUE	TRUE	TRUE
1	CTDVSTAIH	603	611	9	HLA-A*02:01	603	0.0224	0.0537	0.0631	-0.633	0.0315	A2	0.0224	0.0537	0.0631	-0.633	0.0315	TRUE	TRUE	TRUE	TRUE	TRUE
1	TDVSTAIHA	604	612	9	HLA-A*02:01	604	0.0206	0.0493	0.2284	-0.793	0.0439	A2	0.0206	0.0493	0.2284	-0.793	0.0439	TRUE	TRUE	TRUE	TRUE	TRUE
1	DVSTAIHAD	605	613	9	HLA-A*02:01	605	0.0348	0.0832	0.2035	-1.987	0.0144	A2	0.0348	0.0832	0.2035	-1.987	0.0144	TRUE	TRUE	TRUE	TRUE	TRUE
1	VSTAIHADQ	606	614	9	HLA-A*02:01	606	0.0239	0.0572	0.038	-0.178	0.054	A2	0.0239	0.0572	0.038	-0.178	0.054	TRUE	TRUE	TRUE	TRUE	TRUE
1	STAIHADQL	607	615	9	HLA-A*02:01	607	0.0325	0.0779	0.0574	1.05	0.139	A2	0.0325	0.0779	0.0574	1.05	0.139	TRUE	TRUE	TRUE	TRUE	TRUE
1	TAIHADQLT	608	616	9	HLA-A*02:01	608	0.0324	0.0776	0.0104	-0.551	0.0516	A2	0.0324	0.0776	0.0104	-0.551	0.0516	TRUE	TRUE	TRUE	TRUE	TRUE
1	AIHADQLTP	609	617	9	HLA-A*02:01	609	0.0538	0.1287	0.0182	0.417	0.1523	A2	0.0538	0.1287	0.0182	0.417	0.1523	TRUE	TRUE	TRUE	TRUE	TRUE
1	IHADQLTPA	610	618	9	HLA-A*02:01	610	0.0327	0.0783	0.1096	-0.334	0.0781	A2	0.0327	0.0783	0.1096	-0.334	0.0781	TRUE	TRUE	TRUE	TRUE	TRUE
1	HADQLTPAW	611	619	9	HLA-A*02:01	611	0.0288	0.0688	0.9807	0.679	0.2499	A2	0.0288	0.0688	0.9807	0.679	0.2499	TRUE	TRUE	TRUE	TRUE	TRUE
1	ADQLTPAWR	612	620	9	HLA-A*02:01	612	0.0173	0.0413	0.0167	1.266	0.1071	A2	0.0173	0.0413	0.0167	1.266	0.1071	TRUE	TRUE	TRUE	TRUE	TRUE
1	DQLTPAWRI	613	621	9	HLA-A*02:01	613	0.0645	0.1544	0.5967	0.466	0.2672	A2	0.0645	0.1544	0.5967	0.466	0.2672	TRUE	TRUE	TRUE	TRUE	TRUE
1	QLTPAWRIY	614	622	9	HLA-A*02:01	614	0.0638	0.1526	0.9848	2.782	0.4395	A2	0.0638	0.1526	0.9848	2.782	0.4395	TRUE	TRUE	TRUE	TRUE	TRUE
1	LTPAWRIYS	615	623	9	HLA-A*02:01	615	0.0806	0.1928	0.0113	-2.327	0.0782	A2	0.0806	0.1928	0.0113	-2.327	0.0782	TRUE	TRUE	TRUE	TRUE	TRUE
1	TPAWRIYST	616	624	9	HLA-A*02:01	616	0.0323	0.0774	0.098	-0.966	0.0438	A2	0.0323	0.0774	0.098	-0.966	0.0438	TRUE	TRUE	TRUE	TRUE	TRUE
1	PAWRIYSTG	617	625	9	HLA-A*02:01	617	0.0235	0.0562	0.114	-1.641	-0.0087	A2	0.0235	0.0562	0.114	-1.641	-0.0087	TRUE	TRUE	TRUE	TRUE	TRUE
1	AWRIYSTGN	618	626	9	HLA-A*02:01	618	0.016	0.0383	0.0266	-1.021	-0.0088	A2	0.016	0.0383	0.0266	-1.021	-0.0088	TRUE	TRUE	TRUE	TRUE	TRUE
1	WRIYSTGNN	619	627	9	HLA-A*02:01	619	0.0194	0.0463	0.0147	-0.913	0.0029	A2	0.0194	0.0463	0.0147	-0.913	0.0029	TRUE	TRUE	TRUE	TRUE	TRUE
1	RIYSTGNNV	620	628	9	HLA-A*02:01	620	0.3034	0.726	0.8501	0.818	0.8944	A2	0.3034	0.726	0.8501	0.818	0.8944	TRUE	TRUE	TRUE	TRUE	TRUE
1	IYSTGNNVF	621	629	9	HLA-A*02:01	621	0.031	0.0741	0.9977	2.95	0.3713	A2	0.031	0.0741	0.9977	2.95	0.3713	TRUE	TRUE	TRUE	TRUE	TRUE
1	YSTGNNVFQ	622	630	9	HLA-A*02:01	622	0.045	0.1077	0.0154	-0.112	0.1044	A2	0.045	0.1077	0.0154	-0.112	0.1044	TRUE	TRUE	TRUE	TRUE	TRUE
1	STGNNVFQT	623	631	9	HLA-A*02:01	623	0.0612	0.1465	0.1695	-0.889	0.1275	A2	0.0612	0.1465	0.1695	-0.889	0.1275	TRUE	TRUE	TRUE	TRUE	TRUE
1	TGNNVFQTQ	624	632	9	HLA-A*02:01	624	0.0171	0.0408	0.829	-0.433	0.1435	A2	0.0171	0.0408	0.829	-0.433	0.1435	TRUE	TRUE	TRUE	TRUE	TRUE
1	GNNVFQTQA	625	633	9	HLA-A*02:01	625	0.0154	0.037	0.6385	-0.951	0.0852	A2	0.0154	0.037	0.6385	-0.951	0.0852	TRUE	TRUE	TRUE	TRUE	TRUE
1	NNVFQTQAG	626	634	9	HLA-A*02:01	626	0.0146	0.0349	0.0356	-1.463	-0.0329	A2	0.0146	0.0349	0.0356	-1.463	-0.0329	TRUE	TRUE	TRUE	TRUE	TRUE
1	NVFQTQAGC	627	635	9	HLA-A*02:01	627	0.0807	0.1931	0.013	0.443	0.2172	A2	0.0807	0.1931	0.013	0.443	0.2172	TRUE	TRUE	TRUE	TRUE	TRUE
1	VFQTQAGCL	628	636	9	HLA-A*02:01	628	0.0641	0.1535	0.1653	1.293	0.2429	A2	0.0641	0.1535	0.1653	1.293	0.2429	TRUE	TRUE	TRUE	TRUE	TRUE
1	FQTQAGCLI	629	637	9	HLA-A*02:01	629	0.1224	0.2928	0.344	0.526	0.3707	A2	0.1224	0.2928	0.344	0.526	0.3707	TRUE	TRUE	TRUE	TRUE	TRUE
1	QTQAGCLIG	630	638	9	HLA-A*02:01	630	0.0275	0.0659	0.0242	-1.541	-0.0075	A2	0.0275	0.0659	0.0242	-1.541	-0.0075	TRUE	TRUE	TRUE	TRUE	TRUE
1	TQAGCLIGA	631	639	9	HLA-A*02:01	631	0.0375	0.0897	0.0188	-0.513	0.0669	A2	0.0375	0.0897	0.0188	-0.513	0.0669	TRUE	TRUE	TRUE	TRUE	TRUE
1	QAGCLIGAE	632	640	9	HLA-A*02:01	632	0.0173	0.0413	0.0104	-1.653	-0.0398	A2	0.0173	0.0413	0.0104	-1.653	-0.0398	TRUE	TRUE	TRUE	TRUE	TRUE
1	AGCLIGAEH	633	641	9	HLA-A*02:01	633	0.017	0.0406	0.0117	-0.698	0.0075	A2	0.017	0.0406	0.0117	-0.698	0.0075	TRUE	TRUE	TRUE	TRUE	TRUE
1	GCLIGAEHV	634	642	9	HLA-A*02:01	634	0.0643	0.154	0.7711	0.328	0.286	A2	0.0643	0.154	0.7711	0.328	0.286	TRUE	TRUE	TRUE	TRUE	TRUE
1	CLIGAEHVD	635	643	9	HLA-A*02:01	635	0.1283	0.307	0.0337	-1.798	0.2222	A2	0.1283	0.307	0.0337	-1.798	0.2222	TRUE	TRUE	TRUE	TRUE	TRUE
1	LIGAEHVDT	636	644	9	HLA-A*02:01	636	0.0515	0.1233	0.0227	-0.743	0.0896	A2	0.0515	0.1233	0.0227	-0.743	0.0896	TRUE	TRUE	TRUE	TRUE	TRUE
1	IGAEHVDTS	637	645	9	HLA-A*02:01	637	0.0249	0.0595	0.0185	-2.424	-0.0589	A2	0.0249	0.0595	0.0185	-2.424	-0.0589	TRUE	TRUE	TRUE	TRUE	TRUE
1	GAEHVDTSY	638	646	9	HLA-A*02:01	638	0.0198	0.0473	0.999	2.663	0.3303	A2	0.0198	0.0473	0.999	2.663	0.3303	TRUE	TRUE	TRUE	TRUE	TRUE
1	AEHVDTSYE	639	647	9	HLA-A*02:01	639	0.02	0.0479	0.0179	-1.557	-0.0272	A2	0.02	0.0479	0.0179	-1.557	-0.0272	TRUE	TRUE	TRUE	TRUE	TRUE
1	EHVDTSYEC	640	648	9	HLA-A*02:01	640	0.0459	0.1099	0.3635	0.05	0.1669	A2	0.0459	0.1099	0.3635	0.05	0.1669	TRUE	TRUE	TRUE	TRUE	TRUE
1	HVDTSYECD	641	649	9	HLA-A*02:01	641	0.0272	0.0651	0.0139	-2.111	-0.0384	A2	0.0272	0.0651	0.0139	-2.111	-0.0384	TRUE	TRUE	TRUE	TRUE	TRUE
1	VDTSYECDI	642	650	9	HLA-A*02:01	642	0.0273	0.0654	0.046	0.23	0.0838	A2	0.0273	0.0654	0.046	0.23	0.0838	TRUE	TRUE	TRUE	TRUE	TRUE
1	DTSYECDIP	643	651	9	HLA-A*02:01	643	0.0178	0.0427	0.0096	-0.19	0.0346	A2	0.0178	0.0427	0.0096	-0.19	0.0346	TRUE	TRUE	TRUE	TRUE	TRUE
1	TSYECDIPI	644	652	9	HLA-A*02:01	644	0.0585	0.14	0.1443	0.602	0.1918	A2	0.0585	0.14	0.1443	0.602	0.1918	TRUE	TRUE	TRUE	TRUE	TRUE

seq #	peptide	start	end	peptide length	allele	peptide index	NetCTL Predict	NetCTL Rescale	NetCTL c termi	NetCTL TAP tra	NetCTL Predict	NetCTL Superty	legacy predicte	legacy rescale_	legacy c_termi	legacy tap_tran	legacy predicti	equal predicted	equal rescale_	equal c_termin	equal tap_tran	equal predictions_score
1	SYECDIPIG	645	653	9	HLA-A*02:01	645	0.0187	0.0447	0.0142	-1.212	-0.0138	A2	0.0187	0.0447	0.0142	-1.212	-0.0138	TRUE	TRUE	TRUE	TRUE	TRUE
1	YECDIPIGA	646	654	9	HLA-A*02:01	646	0.05	0.1197	0.3276	-0.628	0.1374	A2	0.05	0.1197	0.3276	-0.628	0.1374	TRUE	TRUE	TRUE	TRUE	TRUE
1	ECDIPIGAG	647	655	9	HLA-A*02:01	647	0.0191	0.0456	0.0195	-1.807	-0.0418	A2	0.0191	0.0456	0.0195	-1.807	-0.0418	TRUE	TRUE	TRUE	TRUE	TRUE
1	CDIPIGAGI	648	656	9	HLA-A*02:01	648	0.0392	0.0937	0.7155	0.088	0.2054	A2	0.0392	0.0937	0.7155	0.088	0.2054	TRUE	TRUE	TRUE	TRUE	TRUE
1	DIPIGAGIC	649	657	9	HLA-A*02:01	649	0.0363	0.0869	0.0099	-0.195	0.0786	A2	0.0363	0.0869	0.0099	-0.195	0.0786	TRUE	TRUE	TRUE	TRUE	TRUE
1	IPIGAGICA	650	658	9	HLA-A*02:01	650	0.0406	0.0971	0.5005	-0.887	0.1279	A2	0.0406	0.0971	0.5005	-0.887	0.1279	TRUE	TRUE	TRUE	TRUE	TRUE
1	PIGAGICAS	651	659	9	HLA-A*02:01	651	0.0725	0.1735	0.0103	-2.751	0.0375	A2	0.0725	0.1735	0.0103	-2.751	0.0375	TRUE	TRUE	TRUE	TRUE	TRUE
1	IGAGICASY	652	660	9	HLA-A*02:01	652	0.0235	0.0563	0.9958	2.553	3.33E-01	A2	0.0235	0.0563	0.9958	2.553	0.3333	TRUE	TRUE	TRUE	TRUE	TRUE
1	GAGICASYH	653	661	9	HLA-A*02:01	653	0.0173	0.0414	0.0111	-0.816	0.0023	A2	0.0173	0.0414	0.0111	-0.816	0.0023	TRUE	TRUE	TRUE	TRUE	TRUE
1	AGICASYHT	654	662	9	HLA-A*02:01	654	0.0252	0.0603	0.0397	-0.789	0.0268	A2	0.0252	0.0603	0.0397	-0.789	0.0268	TRUE	TRUE	TRUE	TRUE	TRUE
1	GICASYHTV	655	663	9	HLA-A*02:01	655	0.2986	0.7146	0.9729	0.427	0.8819	A2	0.2986	0.7146	0.9729	0.427	0.8819	TRUE	TRUE	TRUE	TRUE	TRUE
1	ICASYHTVS	656	664	9	HLA-A*02:01	656	0.0306	0.0732	0.0524	-2.215	-0.0297	A2	0.0306	0.0732	0.0524	-2.215	-0.0297	TRUE	TRUE	TRUE	TRUE	TRUE
1	CASYHTVSL	657	665	9	HLA-A*02:01	657	0.0272	0.065	0.9599	0.971	0.2576	A2	0.0272	0.065	0.9599	0.971	0.2576	TRUE	TRUE	TRUE	TRUE	TRUE
1	ASYHTVSLL	658	666	9	HLA-A*02:01	658	0.051	0.1222	0.9979	1.319	0.3378	A2	0.051	0.1222	0.9979	1.319	0.3378	TRUE	TRUE	TRUE	TRUE	TRUE
1	SYHTVSLLR	659	667	9	HLA-A*02:01	659	0.0256	0.0613	0.9987	1.986	0.3104	A2	0.0256	0.0613	0.9987	1.986	0.3104	TRUE	TRUE	TRUE	TRUE	TRUE
1	YHTVSLLRS	660	668	9	HLA-A*02:01	660	0.023	0.055	0.0119	-2.24	-0.0552	A2	0.023	0.055	0.0119	-2.24	-0.0552	TRUE	TRUE	TRUE	TRUE	TRUE
1	HTVSLLRST	661	669	9	HLA-A*02:01	661	0.033	0.079	0.0103	-0.792	0.0409	A2	0.033	0.079	0.0103	-0.792	0.0409	TRUE	TRUE	TRUE	TRUE	TRUE
1	TVSLLRSTS	662	670	9	HLA-A*02:01	662	0.0262	0.0626	0.0123	-2.155	-0.0433	A2	0.0262	0.0626	0.0123	-2.155	-0.0433	TRUE	TRUE	TRUE	TRUE	TRUE
1	VSLLRSTSQ	663	671	9	HLA-A*02:01	663	0.0194	0.0465	0.0113	0.025	0.0494	A2	0.0194	0.0465	0.0113	0.025	0.0494	TRUE	TRUE	TRUE	TRUE	TRUE
1	SLLRSTSQK	664	672	9	HLA-A*02:01	664	0.0943	0.2258	0.9824	0.602	0.4032	A2	0.0943	0.2258	0.9824	0.602	0.4032	TRUE	TRUE	TRUE	TRUE	TRUE
1	LLRSTSQKS	665	673	9	HLA-A*02:01	665	0.1811	0.4334	0.0253	-2.119	0.3312	A2	0.1811	0.4334	0.0253	-2.119	0.3312	TRUE	TRUE	TRUE	TRUE	TRUE
1	LRSTSQKSI	666	674	9	HLA-A*02:01	666	0.0224	0.0536	0.3477	0.789	0.1452	A2	0.0224	0.0536	0.3477	0.789	0.1452	TRUE	TRUE	TRUE	TRUE	TRUE
1	RSTSQKSIV	667	675	9	HLA-A*02:01	667	0.0257	0.0614	0.4987	0.611	0.1668	A2	0.0257	0.0614	0.4987	0.611	0.1668	TRUE	TRUE	TRUE	TRUE	TRUE
1	STSQKSIVA	668	676	9	HLA-A*02:01	668	0.0622	0.1489	0.7296	-0.376	0.2396	A2	0.0622	0.1489	0.7296	-0.376	0.2396	TRUE	TRUE	TRUE	TRUE	TRUE
1	TSQKSIVAY	669	677	9	HLA-A*02:01	669	0.0225	0.054	0.9941	2.966	0.3514	A2	0.0225	0.054	0.9941	2.966	0.3514	TRUE	TRUE	TRUE	TRUE	TRUE
1	SQKSIVAYT	670	678	9	HLA-A*02:01	670	0.029	0.0694	0.0181	-0.569	0.0436	A2	0.029	0.0694	0.0181	-0.569	0.0436	TRUE	TRUE	TRUE	TRUE	TRUE
1	QKSIVAYTM	671	679	9	HLA-A*02:01	671	0.0205	0.049	0.999	0.417	0.2197	A2	0.0205	0.049	0.999	0.417	0.2197	TRUE	TRUE	TRUE	TRUE	TRUE
1	KSIVAYTMS	672	680	9	HLA-A*02:01	672	0.0197	0.0472	0.0149	-2.078	-0.0545	A2	0.0197	0.0472	0.0149	-2.078	-0.0545	TRUE	TRUE	TRUE	TRUE	TRUE
1	SIVAYTMSL	673	681	9	HLA-A*02:01	673	0.1666	0.3986	0.9989	1.315	0.6142	A2	0.1666	0.3986	0.9989	1.315	0.6142	TRUE	TRUE	TRUE	TRUE	TRUE
1	IVAYTMSLG	674	682	9	HLA-A*02:01	674	0.0315	0.0755	0.0101	-1.163	0.0189	A2	0.0315	0.0755	0.0101	-1.163	0.0189	TRUE	TRUE	TRUE	TRUE	TRUE
1	VAYTMSLGA	675	683	9	HLA-A*02:01	675	0.0784	0.1876	0.061	-0.225	0.1855	A2	0.0784	0.1876	0.061	-0.225	0.1855	TRUE	TRUE	TRUE	TRUE	TRUE
1	AYTMSLGAD	676	684	9	HLA-A*02:01	676	0.0209	0.0499	0.0102	-1.396	-0.0183	A2	0.0209	0.0499	0.0102	-1.396	-0.0183	TRUE	TRUE	TRUE	TRUE	TRUE
1	YTMSLGADS	677	685	9	HLA-A*02:01	677	0.1311	0.3137	0.01	-2.068	0.2118	A2	0.1311	0.3137	0.01	-2.068	0.2118	TRUE	TRUE	TRUE	TRUE	TRUE
1	TMSLGADSS	678	686	9	HLA-A*02:01	678	0.0934	0.2234	0.0113	-2.201	0.1151	A2	0.0934	0.2234	0.0113	-2.201	0.1151	TRUE	TRUE	TRUE	TRUE	TRUE
1	MSLGADSSI	679	687	9	HLA-A*02:01	679	0.0637	0.1524	0.9272	0.65	0.3239	A2	0.0637	0.1524	0.9272	0.65	0.3239	TRUE	TRUE	TRUE	TRUE	TRUE
1	SLGADSSIA	680	688	9	HLA-A*02:01	680	0.107	0.256	0.0773	-0.59	0.2381	A2	0.107	0.256	0.0773	-0.59	0.2381	TRUE	TRUE	TRUE	TRUE	TRUE
1	LGADSSIAY	681	689	9	HLA-A*02:01	681	0.0249	0.0597	0.9978	2.733	0.346	A2	0.0249	0.0597	0.9978	2.733	0.346	TRUE	TRUE	TRUE	TRUE	TRUE
1	GADSSIAYS	682	690	9	HLA-A*02:01	682	0.0625	0.1497	0.0118	-2.5	0.0264	A2	0.0625	0.1497	0.0118	-2.5	0.0264	TRUE	TRUE	TRUE	TRUE	TRUE
1	ADSSIAYSN	683	691	9	HLA-A*02:01	683	0.0172	0.0413	0.013	-1.513	-0.0324	A2	0.0172	0.0413	0.013	-1.513	-0.0324	TRUE	TRUE	TRUE	TRUE	TRUE
1	DSSIAYSNN	684	692	9	HLA-A*02:01	684	0.0149	0.0355	0.0101	-1.441	-0.035	A2	0.0149	0.0355	0.0101	-1.441	-0.035	TRUE	TRUE	TRUE	TRUE	TRUE
1	SSIAYSNNT	685	693	9	HLA-A*02:01	685	0.0264	0.0631	0.0115	-0.682	0.0307	A2	0.0264	0.0631	0.0115	-0.682	0.0307	TRUE	TRUE	TRUE	TRUE	TRUE
1	SIAYSNNTI	686	694	9	HLA-A*02:01	686	0.092	0.2201	0.7134	0.787	0.3665	A2	0.092	0.2201	0.7134	0.787	0.3665	TRUE	TRUE	TRUE	TRUE	TRUE
1	IAYSNNTIA	687	695	9	HLA-A*02:01	687	0.0456	0.1092	0.3263	-0.219	0.1472	A2	0.0456	0.1092	0.3263	-0.219	0.1472	TRUE	TRUE	TRUE	TRUE	TRUE
1	AYSNNTIAI	688	696	9	HLA-A*02:01	688	0.0861	0.2061	0.5433	0.948	0.335	A2	0.0861	0.2061	0.5433	0.948	0.335	TRUE	TRUE	TRUE	TRUE	TRUE
1	YSNNTIAIP	689	697	9	HLA-A*02:01	689	0.0296	0.0709	0.0367	0.185	0.0857	A2	0.0296	0.0709	0.0367	0.185	0.0857	TRUE	TRUE	TRUE	TRUE	TRUE
1	SNNTIAIPT	690	698	9	HLA-A*02:01	690	0.0192	0.0459	0.0128	-0.951	0.0003	A2	0.0192	0.0459	0.0128	-0.951	0.0003	TRUE	TRUE	TRUE	TRUE	TRUE
1	NNTIAIPTN	691	699	9	HLA-A*02:01	691	0.0146	0.0349	0.0133	-1.413	-0.0338	A2	0.0146	0.0349	0.0133	-1.413	-0.0338	TRUE	TRUE	TRUE	TRUE	TRUE
1	NTIAIPTNF	692	700	9	HLA-A*02:01	692	0.0237	0.0568	0.8208	2.76	0.3179	A2	0.0237	0.0568	0.8208	2.76	0.3179	TRUE	TRUE	TRUE	TRUE	TRUE
1	TIAIPTNFS	693	701	9	HLA-A*02:01	693	0.0773	0.185	0.0096	-1.98	0.0874	A2	0.0773	0.185	0.0096	-1.98	0.0874	TRUE	TRUE	TRUE	TRUE	TRUE
1	IAIPTNFSI	694	702	9	HLA-A*02:01	694	0.19	0.4548	0.9961	0.689	0.6386	A2	0.19	0.4548	0.9961	0.689	0.6386	TRUE	TRUE	TRUE	TRUE	TRUE
1	AIPTNFSIS	695	703	9	HLA-A*02:01	695	0.0552	0.132	1.29E-02	-1.98E+00	0.035	A2	0.0552	0.132	0.0129	-1.979	0.035	TRUE	TRUE	TRUE	TRUE	TRUE
1	IPTNFSISI	696	704	9	HLA-A*02:01	696	0.0348	0.0832	9.95E-01	3.71E-01	0.251	A2	0.0348	0.0832	0.995	0.371	0.251	TRUE	TRUE	TRUE	TRUE	TRUE
1	PTNFSISIT	697	705	9	HLA-A*02:01	697	0.0254	0.0608	1.06E-02	-1.29E+00	-0.0022	A2	0.0254	0.0608	0.0106	-1.292	-0.0022	TRUE	TRUE	TRUE	TRUE	TRUE
1	TNFSISITT	698	706	9	HLA-A*02:01	698	0.0248	0.0594	3.57E-01	-9.12E-01	0.0673	A2	0.0248	0.0594	0.3566	-0.912	0.0673	TRUE	TRUE	TRUE	TRUE	TRUE
1	NFSISITTE	699	707	9	HLA-A*02:01	699	0.02	0.0478	9.80E-03	-1.36E+00	-0.0185	A2	0.02	0.0478	0.0098	-1.355	-0.0185	TRUE	TRUE	TRUE	TRUE	TRUE
1	FSISITTEV	700	708	9	HLA-A*02:01	700	0.1549	0.3706	9.58E-01	4.70E-01	0.5378	A2	0.1549	0.3706	0.958	0.47	0.5378	TRUE	TRUE	TRUE	TRUE	TRUE
1	SISITTEVM	701	709	9	HLA-A*02:01	701	0.0593	0.1418	7.54E-01	3.77E-01	0.2737	A2	0.0593	0.1418	0.7539	0.377	0.2737	TRUE	TRUE	TRUE	TRUE	TRUE
1	ISITTEVMP	702	710	9	HLA-A*02:01	702	0.0178	0.0427	9.70E-03	2.91E-01	0.0587	A2	0.0178	0.0427	0.0097	0.291	0.0587	TRUE	TRUE	TRUE	TRUE	TRUE
1	SITTEVMPV	703	711	9	HLA-A*02:01	703	0.2035	0.487	9.93E-01	4.69E-01	0.6594	A2	0.2035	0.487	0.9932	0.469	0.6594	TRUE	TRUE	TRUE	TRUE	TRUE
1	ITTEVMPVS	704	712	9	HLA-A*02:01	704	0.0825	0.1975	1.12E-02	-2.23E+00	0.0878	A2	0.0825	0.1975	0.0112	-2.226	0.0878	TRUE	TRUE	TRUE	TRUE	TRUE
1	TTEVMPVSM	705	713	9	HLA-A*02:01	705	0.023	0.055	6.13E-01	1.27E-01	0.1533	A2	0.023	0.055	0.6129	0.127	0.1533	TRUE	TRUE	TRUE	TRUE	TRUE
1	TEVMPVSMA	706	714	9	HLA-A*02:01	706	0.0186	0.0445	5.35E-01	-6.04E-01	0.0946	A2	0.0186	0.0445	0.5348	-0.604	0.0946	TRUE	TRUE	TRUE	TRUE	TRUE
1	EVMPVSMAK	707	715	9	HLA-A*02:01	707	0.0781	0.1868	9.95E-01	4.74E-01	0.3598	A2	0.0781	0.1868	0.995	0.474	0.3598	TRUE	TRUE	TRUE	TRUE	TRUE
1	VMPVSMAKT	708	716	9	HLA-A*02:01	708	0.2157	0.5162	6.01E-02	-7.04E-01	0.49	A2	0.2157	0.5162	0.0601	-0.704	0.49	TRUE	TRUE	TRUE	TRUE	TRUE
1	MPVSMAKTS	709	717	9	HLA-A*02:01	709	0.0191	0.0458	1.18E-02	-2.38E+00	-0.0713	A2	0.0191	0.0458	0.0118	-2.378	-0.0713	TRUE	TRUE	TRUE	TRUE	TRUE
1	PVSMAKTSV	710	718	9	HLA-A*02:01	710	0.0685	0.1639	6.56E-01	5.00E-03	0.2625	A2	0.0685	0.1639	0.6557	0.005	0.2625	TRUE	TRUE	TRUE	TRUE	TRUE
1	VSMAKTSVD	711	719	9	HLA-A*02:01	711	0.0192	0.0459	1.16E-02	-1.79E+00	-0.0421	A2	0.0192	0.0459	0.0116	-1.793	-0.0421	TRUE	TRUE	TRUE	TRUE	TRUE
1	SMAKTSVDC	712	720	9	HLA-A*02:01	712	0.2699	0.6459	1.37E-02	2.53E-01	0.6606	A2	0.2699	0.6459	0.0137	0.253	0.6606	TRUE	TRUE	TRUE	TRUE	TRUE
1	MAKTSVDCN	713	721	9	HLA-A*02:01	713	0.0204	0.0488	9.60E-03	-1.16E+00	-0.0079	A2	0.0204	0.0488	0.0096	-1.164	-0.0079	TRUE	TRUE	TRUE	TRUE	TRUE
1	AKTSVDCNM	714	722	9	HLA-A*02:01	714	0.0306	0.0731	9.93E-01	4.98E-01	0.2469	A2	0.0306	0.0731	0.9926	0.498	0.2469	TRUE	TRUE	TRUE	TRUE	TRUE
1	KTSVDCNMY	715	723	9	HLA-A*02:01	715	0.0214	0.0511	9.89E-01	3.16E+00	0.3575	A2	0.0214	0.0511	0.9892	3.16	0.3575	TRUE	TRUE	TRUE	TRUE	TRUE
1	TSVDCNMYI	716	724	9	HLA-A*02:01	716	0.0841	0.2012	9.86E-01	7.33E-01	0.3858	A2	0.0841	0.2012	0.9864	0.733	0.3858	TRUE	TRUE	TRUE	TRUE	TRUE
1	SVDCNMYIC	717	725	9	HLA-A*02:01	717	0.0571	0.1367	1.08E-02	4.00E-02	0.1403	A2	0.0571	0.1367	0.0108	0.04	0.1403	TRUE	TRUE	TRUE	TRUE	TRUE
1	VDCNMYICG	718	726	9	HLA-A*02:01	718	0.0174	0.0417	1.01E-02	-1.58E+00	-0.0357	A2	0.0174	0.0417	0.0101	-1.578	-0.0357	TRUE	TRUE	TRUE	TRUE	TRUE
1	DCNMYICGD	719	727	9	HLA-A*02:01	719	0.0183	0.0438	9.70E-03	-2.16E+00	-0.0626	A2	0.0183	0.0438	0.0097	-2.158	-0.0626	TRUE	TRUE	TRUE	TRUE	TRUE
1	CNMYICGDS	720	728	9	HLA-A*02:01	720	0.0206	0.0492	9.60E-03	-2.51E+00	-0.075	A2	0.0206	0.0492	0.0096	-2.513	-0.075	TRUE	TRUE	TRUE	TRUE	TRUE
1	NMYICGDST	721	729	9	HLA-A*02:01	721	0.0892	0.2134	1.24E-02	-3.67E-01	0.1969	A2	0.0892	0.2134	0.0124	-0.367	0.1969	TRUE	TRUE	TRUE	TRUE	TRUE
1	MYICGDSTE	722	730	9	HLA-A*02:01	722	0.0223	0.0534	4.18E-02	-1.04E+00	0.0075	A2	0.0223	0.0534	0.0418	-1.044	0.0075	TRUE	TRUE	TRUE	TRUE	TRUE
1	YICGDSTEC	723	731	9	HLA-A*02:01	723	0.3074	0.7358	7.92E-02	1.68E-01	0.7561	A2	0.3074	0.7358	0.0792	0.168	0.7561	TRUE	TRUE	TRUE	TRUE	TRUE
1	ICGDSTECA	724	732	9	HLA-A*02:01	724	0.0761	0.1822	2.56E-01	-7.07E-01	0.1852	A2	0.0761	0.1822	0.2558	-0.707	0.1852	TRUE	TRUE	TRUE	TRUE	TRUE
1	CGDSTECAN	725	733	9	HLA-A*02:01	725	0.0149	0.0357	1.10E-02	-1.79E+00	-0.0521	A2	0.0149	0.0357	0.011	-1.79	-0.0521	TRUE	TRUE	TRUE	TRUE	TRUE
1	GDSTECANL	726	734	9	HLA-A*02:01	726	0.0249	0.0595	9.79E-01	4.57E-01	0.2293	A2	0.0249	0.0595	0.9793	0.457	0.2293	TRUE	TRUE	TRUE	TRUE	TRUE
1	DSTECANLL	727	735	9	HLA-A*02:01	727	0.0253	0.0606	4.16E-01	6.13E-01	0.1537	A2	0.0253	0.0606	0.416	0.613	0.1537	TRUE	TRUE	TRUE	TRUE	TRUE
1	STECANLLL	728	736	9	HLA-A*02:01	728	0.0357	0.0853	9.94E-01	7.03E-01	0.2695	A2	0.0357	0.0853	0.9936	0.703	0.2695	TRUE	TRUE	TRUE	TRUE	TRUE
1	TECANLLLQ	729	737	9	HLA-A*02:01	729	0.0198	0.0475	1.02E-02	-1.99E-01	0.0391	A2	0.0198	0.0475	0.0102	-0.199	0.0391	TRUE	TRUE	TRUE	TRUE	TRUE
1	ECANLLLQY	730	738	9	HLA-A*02:01	730	0.0219	0.0525	6.59E-01	2.77E+00	0.2898	A2	0.0219	0.0525	0.6594	2.768	0.2898	TRUE	TRUE	TRUE	TRUE	TRUE
1	CANLLLQYG	731	739	9	HLA-A*02:01	731	0.0195	0.0467	1.17E-02	-1.65E+00	-0.0337	A2	0.0195	0.0467	0.0117	-1.645	-0.0337	TRUE	TRUE	TRUE	TRUE	TRUE
1	ANLLLQYGS	732	740	9	HLA-A*02:01	732	0.0186	0.0445	1.08E-02	-2.24E+00	-0.066	A2	0.0186	0.0445	0.0108	-2.243	-0.066	TRUE	TRUE	TRUE	TRUE	TRUE
1	NLLLQYGSF	733	741	9	HLA-A*02:01	733	0.0417	0.0997	9.83E-01	2.76E+00	0.3853	A2	0.0417	0.0997	0.9828	2.763	0.3853	TRUE	TRUE	TRUE	TRUE	TRUE
1	LLLQYGSFC	734	742	9	HLA-A*02:01	734	0.3888	0.9305	1.02E-02	1.97E-01	0.9418	A2	0.3888	0.9305	0.0102	0.197	0.9418	TRUE	TRUE	TRUE	TRUE	TRUE
1	LLQYGSFCT	735	743	9	HLA-A*02:01	735	0.3863	0.9244	1.05E-01	-6.66E-01	0.9068	A2	0.3863	0.9244	0.1046	-0.666	0.9068	TRUE	TRUE	TRUE	TRUE	TRUE
1	LQYGSFCTQ	736	744	9	HLA-A*02:01	736	0.0429	0.1027	4.19E-01	2.09E-01	0.176	A2	0.0429	0.1027	0.419	0.209	0.176	TRUE	TRUE	TRUE	TRUE	TRUE
1	QYGSFCTQL	737	745	9	HLA-A*02:01	737	0.0286	0.0684	9.99E-01	1.12E+00	0.2741	A2	0.0286	0.0684	0.9989	1.117	0.2741	TRUE	TRUE	TRUE	TRUE	TRUE
1	YGSFCTQLN	738	746	9	HLA-A*02:01	738	0.016	0.0383	1.05E-02	-1.66E+00	-0.0428	A2	0.016	0.0383	0.0105	-1.655	-0.0428	TRUE	TRUE	TRUE	TRUE	TRUE
1	GSFCTQLNR	739	747	9	HLA-A*02:01	739	0.0173	0.0413	9.57E-01	1.41E+00	0.2552	A2	0.0173	0.0413	0.9569	1.406	0.2552	TRUE	TRUE	TRUE	TRUE	TRUE
1	SFCTQLNRA	740	748	9	HLA-A*02:01	740	0.0418	0.1001	3.75E-02	-3.36E-01	0.0889	A2	0.0418	0.1001	0.0375	-0.336	0.0889	TRUE	TRUE	TRUE	TRUE	TRUE
1	FCTQLNRAL	741	749	9	HLA-A*02:01	741	0.1205	0.2885	3.11E-02	8.98E-01	0.3381	A2	0.1205	0.2885	0.0311	0.898	0.3381	TRUE	TRUE	TRUE	TRUE	TRUE
1	CTQLNRALS	742	750	9	HLA-A*02:01	742	0.0241	0.0577	1.08E-02	-2.45E+00	-0.0629	A2	0.0241	0.0577	0.0108	-2.445	-0.0629	TRUE	TRUE	TRUE	TRUE	TRUE
1	TQLNRALSG	743	751	9	HLA-A*02:01	743	0.0186	0.0444	1.34E-02	-1.33E+00	-0.02	A2	0.0186	0.0444	0.0134	-1.33	-0.02	TRUE	TRUE	TRUE	TRUE	TRUE
1	QLNRALSGI	744	752	9	HLA-A*02:01	744	0.2181	0.522	9.80E-01	4.83E-01	0.6932	A2	0.2181	0.522	0.9801	0.483	0.6932	TRUE	TRUE	TRUE	TRUE	TRUE
1	LNRALSGIA	745	753	9	HLA-A*02:01	745	0.0197	0.0471	2.55E-01	-6.84E-01	0.0511	A2	0.0197	0.0471	0.2547	-0.684	0.0511	TRUE	TRUE	TRUE	TRUE	TRUE
1	NRALSGIAA	746	754	9	HLA-A*02:01	746	0.0169	0.0403	4.92E-01	-2.27E-01	0.1027	A2	0.0169	0.0403	0.4916	-0.227	0.1027	TRUE	TRUE	TRUE	TRUE	TRUE
1	RALSGIAAE	747	755	9	HLA-A*02:01	747	0.0325	0.0779	1.50E-02	-1.11E+00	0.0246	A2	0.0325	0.0779	0.015	-1.11	0.0246	TRUE	TRUE	TRUE	TRUE	TRUE
1	ALSGIAAEQ	748	756	9	HLA-A*02:01	748	0.2737	0.6549	4.34E-01	1.86E-01	0.7293	A2	0.2737	0.6549	0.4337	0.186	0.7293	TRUE	TRUE	TRUE	TRUE	TRUE
1	LSGIAAEQD	749	757	9	HLA-A*02:01	749	0.0153	0.0366	1.12E-02	-1.78E+00	-0.0507	A2	0.0153	0.0366	0.0112	-1.78	-0.0507	TRUE	TRUE	TRUE	TRUE	TRUE
1	SGIAAEQDR	750	758	9	HLA-A*02:01	750	0.0164	0.0392	2.00E-02	1.39E+00	0.1116	A2	0.0164	0.0392	0.02	1.388	0.1116	TRUE	TRUE	TRUE	TRUE	TRUE
1	GIAAEQDRN	751	759	9	HLA-A*02:01	751	0.029	0.0693	9.70E-03	-1.30E+00	0.006	A2	0.029	0.0693	0.0097	-1.295	0.006	TRUE	TRUE	TRUE	TRUE	TRUE
1	IAAEQDRNT	752	760	9	HLA-A*02:01	752	0.044	0.1052	9.70E-03	-6.43E-01	0.0745	A2	0.044	0.1052	0.0097	-0.643	0.0745	TRUE	TRUE	TRUE	TRUE	TRUE
1	AAEQDRNTR	753	761	9	HLA-A*02:01	753	0.0253	0.0606	8.95E-02	1.55E+00	0.1513	A2	0.0253	0.0606	0.0895	1.546	0.1513	TRUE	TRUE	TRUE	TRUE	TRUE
1	AEQDRNTRE	754	762	9	HLA-A*02:01	754	0.0267	0.064	1.60E-02	-1.44E+00	-0.0054	A2	0.0267	0.064	0.016	-1.435	-0.0054	TRUE	TRUE	TRUE	TRUE	TRUE
1	EQDRNTREV	755	763	9	HLA-A*02:01	755	0.0462	0.1105	7.25E-01	1.64E-01	0.2274	A2	0.0462	0.1105	0.7246	0.164	0.2274	TRUE	TRUE	TRUE	TRUE	TRUE
1	QDRNTREVF	756	764	9	HLA-A*02:01	756	0.0163	0.0391	5.25E-01	2.12E+00	0.224	A2	0.0163	0.0391	0.5253	2.122	0.224	TRUE	TRUE	TRUE	TRUE	TRUE
1	DRNTREVFA	757	765	9	HLA-A*02:01	757	0.0159	0.0381	4.94E-02	-7.88E-01	0.0061	A2	0.0159	0.0381	0.0494	-0.788	0.0061	TRUE	TRUE	TRUE	TRUE	TRUE
1	RNTREVFAQ	758	766	9	HLA-A*02:01	758	0.0214	0.0511	1.06E-02	-1.89E-01	0.0432	A2	0.0214	0.0511	0.0106	-0.189	0.0432	TRUE	TRUE	TRUE	TRUE	TRUE
1	NTREVFAQV	759	767	9	HLA-A*02:01	759	0.1084	0.2595	9.98E-01	5.11E-01	0.4346	A2	0.1084	0.2595	0.9976	0.511	0.4346	TRUE	TRUE	TRUE	TRUE	TRUE
1	TREVFAQVK	760	768	9	HLA-A*02:01	760	0.0154	0.037	9.87E-01	6.47E-01	0.2173	A2	0.0154	0.037	0.9865	0.647	0.2173	TRUE	TRUE	TRUE	TRUE	TRUE
1	REVFAQVKQ	761	769	9	HLA-A*02:01	761	0.0171	0.041	3.33E-02	9.10E-02	0.0506	A2	0.0171	0.041	0.0333	0.091	0.0506	TRUE	TRUE	TRUE	TRUE	TRUE
1	EVFAQVKQM	762	770	9	HLA-A*02:01	762	0.0605	0.1448	8.28E-01	4.83E-01	0.2931	A2	0.0605	0.1448	0.828	0.483	0.2931	TRUE	TRUE	TRUE	TRUE	TRUE
1	VFAQVKQMY	763	771	9	HLA-A*02:01	763	0.0278	0.0665	8.65E-01	3.00E+00	0.3465	A2	0.0278	0.0665	0.8652	3.004	0.3465	TRUE	TRUE	TRUE	TRUE	TRUE
1	FAQVKQMYK	764	772	9	HLA-A*02:01	764	0.0394	0.0942	2.91E-01	4.37E-01	0.1598	A2	0.0394	0.0942	0.2913	0.437	0.1598	TRUE	TRUE	TRUE	TRUE	TRUE
1	AQVKQMYKT	765	773	9	HLA-A*02:01	765	0.0425	0.1017	1.31E-02	-5.13E-01	0.0781	A2	0.0425	0.1017	0.0131	-0.513	0.0781	TRUE	TRUE	TRUE	TRUE	TRUE
1	QVKQMYKTP	766	774	9	HLA-A*02:01	766	0.0322	0.077	2.27E-02	4.08E-01	0.1008	A2	0.0322	0.077	0.0227	0.408	0.1008	TRUE	TRUE	TRUE	TRUE	TRUE
1	VKQMYKTPT	767	775	9	HLA-A*02:01	767	0.0192	0.0461	1.22E-02	-6.75E-01	0.0141	A2	0.0192	0.0461	0.0122	-0.675	0.0141	TRUE	TRUE	TRUE	TRUE	TRUE
1	KQMYKTPTL	768	776	9	HLA-A*02:01	768	0.0459	0.1098	9.20E-01	1.25E+00	0.3102	A2	0.0459	0.1098	0.92	1.249	0.3102	TRUE	TRUE	TRUE	TRUE	TRUE
1	QMYKTPTLK	769	777	9	HLA-A*02:01	769	0.0872	0.2086	9.98E-01	8.87E-01	0.4027	A2	0.0872	0.2086	0.9981	0.887	0.4027	TRUE	TRUE	TRUE	TRUE	TRUE
1	MYKTPTLKY	770	778	9	HLA-A*02:01	770	0.0271	0.0649	9.99E-01	3.19E+00	0.374	A2	0.0271	0.0649	0.999	3.185	0.374	TRUE	TRUE	TRUE	TRUE	TRUE
1	YKTPTLKYF	771	779	9	HLA-A*02:01	771	0.0428	0.1023	7.61E-01	2.56E+00	0.3446	A2	0.0428	0.1023	0.7606	2.564	0.3446	TRUE	TRUE	TRUE	TRUE	TRUE
1	KTPTLKYFG	772	780	9	HLA-A*02:01	772	0.0247	0.059	1.04E-02	-1.38E+00	-0.0083	A2	0.0247	0.059	0.0104	-1.377	-0.0083	TRUE	TRUE	TRUE	TRUE	TRUE
1	TPTLKYFGG	773	781	9	HLA-A*02:01	773	0.0166	0.0398	1.71E-01	-1.66E+00	-0.0173	A2	0.0166	0.0398	0.1714	-1.656	-0.0173	TRUE	TRUE	TRUE	TRUE	TRUE
1	PTLKYFGGF	774	782	9	HLA-A*02:01	774	0.0308	0.0737	9.41E-01	2.06E+00	0.3179	A2	0.0308	0.0737	0.9409	2.061	0.3179	TRUE	TRUE	TRUE	TRUE	TRUE
1	TLKYFGGFN	775	783	9	HLA-A*02:01	775	0.042	0.1006	9.90E-03	-1.46E+00	0.0288	A2	0.042	0.1006	0.0099	-1.464	0.0288	TRUE	TRUE	TRUE	TRUE	TRUE
1	LKYFGGFNF	776	784	9	HLA-A*02:01	776	0.0439	0.105	9.47E-01	2.79E+00	0.3863	A2	0.0439	0.105	0.9469	2.785	0.3863	TRUE	TRUE	TRUE	TRUE	TRUE
1	KYFGGFNFS	777	785	9	HLA-A*02:01	777	0.0849	0.2032	8.55E-01	-1.71E+00	0.2458	A2	0.0849	0.2032	0.8554	-1.714	0.2458	TRUE	TRUE	TRUE	TRUE	TRUE
1	YFGGFNFSQ	778	786	9	HLA-A*02:01	778	0.1097	0.2626	4.76E-01	-2.00E-02	0.333	A2	0.1097	0.2626	0.4758	-0.02	0.333	TRUE	TRUE	TRUE	TRUE	TRUE
1	FGGFNFSQI	779	787	9	HLA-A*02:01	779	0.0255	0.061	9.11E-01	-4.20E-02	0.1955	A2	0.0255	0.061	0.9109	-0.042	0.1955	TRUE	TRUE	TRUE	TRUE	TRUE
1	GGFNFSQIL	780	788	9	HLA-A*02:01	780	0.029	0.0693	9.96E-01	5.16E-01	0.2445	A2	0.029	0.0693	0.9958	0.516	0.2445	TRUE	TRUE	TRUE	TRUE	TRUE
1	GFNFSQILP	781	789	9	HLA-A*02:01	781	0.0203	0.0486	1.19E-02	-3.10E-02	0.0489	A2	0.0203	0.0486	0.0119	-0.031	0.0489	TRUE	TRUE	TRUE	TRUE	TRUE
1	FNFSQILPD	782	790	9	HLA-A*02:01	782	0.0236	0.0566	1.40E-02	-1.98E+00	-0.0404	A2	0.0236	0.0566	0.014	-1.981	-0.0404	TRUE	TRUE	TRUE	TRUE	TRUE
1	NFSQILPDP	783	791	9	HLA-A*02:01	783	0.03	0.0718	1.06E-02	2.00E-01	0.0834	A2	0.03	0.0718	0.0106	0.2	0.0834	TRUE	TRUE	TRUE	TRUE	TRUE
1	FSQILPDPL	784	792	9	HLA-A*02:01	784	0.0361	0.0865	5.45E-01	1.01E+00	0.2187	A2	0.0361	0.0865	0.5448	1.009	0.2187	TRUE	TRUE	TRUE	TRUE	TRUE
1	SQILPDPLK	785	793	9	HLA-A*02:01	785	0.0183	0.0438	1.81E-01	5.57E-01	0.0988	A2	0.0183	0.0438	0.1807	0.557	0.0988	TRUE	TRUE	TRUE	TRUE	TRUE
1	QILPDPLKP	786	794	9	HLA-A*02:01	786	0.0525	0.1256	1.60E-01	3.18E-01	0.1655	A2	0.0525	0.1256	0.1603	0.318	0.1655	TRUE	TRUE	TRUE	TRUE	TRUE
1	ILPDPLKPT	787	795	9	HLA-A*02:01	787	0.2769	0.6627	2.90E-01	-7.27E-01	0.6698	A2	0.2769	0.6627	0.2898	-0.727	0.6698	TRUE	TRUE	TRUE	TRUE	TRUE
1	LPDPLKPTK	788	796	9	HLA-A*02:01	788	0.0199	0.0476	9.94E-01	-3.00E-03	0.1966	A2	0.0199	0.0476	0.9943	-0.003	0.1966	TRUE	TRUE	TRUE	TRUE	TRUE
1	PDPLKPTKR	789	797	9	HLA-A*02:01	789	0.0142	0.0341	7.90E-01	7.30E-01	0.189	A2	0.0142	0.0341	0.7897	0.73	0.189	TRUE	TRUE	TRUE	TRUE	TRUE
1	DPLKPTKRS	790	798	9	HLA-A*02:01	790	0.0176	0.0422	2.06E-01	-2.99E+00	-0.0765	A2	0.0176	0.0422	0.2063	-2.993	-0.0765	TRUE	TRUE	TRUE	TRUE	TRUE
1	PLKPTKRSF	791	799	9	HLA-A*02:01	791	0.0659	0.1578	4.36E-01	1.93E+00	0.3198	A2	0.0659	0.1578	0.4357	1.933	0.3198	TRUE	TRUE	TRUE	TRUE	TRUE
1	LKPTKRSFI	792	800	9	HLA-A*02:01	792	0.0283	0.0677	2.90E-02	4.28E-01	0.0934	A2	0.0283	0.0677	0.029	0.428	0.0934	TRUE	TRUE	TRUE	TRUE	TRUE
1	KPTKRSFIE	793	801	9	HLA-A*02:01	793	0.0206	0.0493	2.16E-02	-1.75E+00	-0.0351	A2	0.0206	0.0493	0.0216	-1.751	-0.0351	TRUE	TRUE	TRUE	TRUE	TRUE
1	PTKRSFIED	794	802	9	HLA-A*02:01	794	0.023	0.055	1.16E-02	-2.22E+00	-0.0542	A2	0.023	0.055	0.0116	-2.218	-0.0542	TRUE	TRUE	TRUE	TRUE	TRUE
1	TKRSFIEDL	795	803	9	HLA-A*02:01	795	0.0268	0.0641	7.20E-01	8.87E-01	0.2164	A2	0.0268	0.0641	0.7198	0.887	0.2164	TRUE	TRUE	TRUE	TRUE	TRUE
1	KRSFIEDLL	796	804	9	HLA-A*02:01	796	0.019	0.0454	5.62E-01	1.27E+00	0.1934	A2	0.019	0.0454	0.5621	1.274	0.1934	TRUE	TRUE	TRUE	TRUE	TRUE
1	RSFIEDLLF	797	805	9	HLA-A*02:01	797	0.0188	0.045	1.69E-01	3.03E+00	0.222	A2	0.0188	0.045	0.1694	3.032	0.222	TRUE	TRUE	TRUE	TRUE	TRUE
1	SFIEDLLFN	798	806	9	HLA-A*02:01	798	0.0488	0.1169	9.80E-03	-8.94E-01	0.0737	A2	0.0488	0.1169	0.0098	-0.894	0.0737	TRUE	TRUE	TRUE	TRUE	TRUE
1	FIEDLLFNK	799	807	9	HLA-A*02:01	799	0.3042	0.728	5.48E-01	4.08E-01	0.8306	A2	0.3042	0.728	0.5476	0.408	0.8306	TRUE	TRUE	TRUE	TRUE	TRUE
1	IEDLLFNKV	800	808	9	HLA-A*02:01	800	0.0332	0.0794	9.99E-01	-7.90E-02	0.2253	A2	0.0332	0.0794	0.9989	-0.079	0.2253	TRUE	TRUE	TRUE	TRUE	TRUE
1	EDLLFNKVT	801	809	9	HLA-A*02:01	801	0.0159	0.0381	1.87E-02	-1.19E+00	-0.0186	A2	0.0159	0.0381	0.0187	-1.19	-0.0186	TRUE	TRUE	TRUE	TRUE	TRUE
1	DLLFNKVTL	802	810	9	HLA-A*02:01	802	0.0699	0.1673	9.97E-01	5.09E-01	0.3422	A2	0.0699	0.1673	0.9965	0.509	0.3422	TRUE	TRUE	TRUE	TRUE	TRUE
1	LLFNKVTLA	803	811	9	HLA-A*02:01	803	0.2445	0.5852	9.03E-01	-5.39E-01	0.6936	A2	0.2445	0.5852	0.9026	-0.539	0.6936	TRUE	TRUE	TRUE	TRUE	TRUE
1	LFNKVTLAD	804	812	9	HLA-A*02:01	804	0.0333	0.0796	2.41E-02	-1.69E+00	-0.0013	A2	0.0333	0.0796	0.0241	-1.69	-0.0013	TRUE	TRUE	TRUE	TRUE	TRUE
1	FNKVTLADA	805	813	9	HLA-A*02:01	805	0.0253	0.0604	6.02E-02	-7.37E-01	0.0326	A2	0.0253	0.0604	0.0602	-0.737	0.0326	TRUE	TRUE	TRUE	TRUE	TRUE

seq #	peptide	start	end	peptide length	allele	peptide index	NetCTL Predict	NetCTL Rescale	NetCTL c termi	NetCTL TAP tra	NetCTL Predict	NetCTL Superty	legacy predicte	legacy rescale_	legacy c_termi	legacy tap_trar	legacy predicti	equal predicte	equal rescale_	equal c_termir	equal tap_tran	equal predictions_score
1	NKVTLADAG	806	814	9	HLA-A*02:01	806	0.0151	0.0362	1.10E-02	-1.34E+00	-0.029	A2	0.0151	0.0362	0.011	-1.336	-0.029	TRUE	TRUE	TRUE	TRUE	TRUE
1	KVTLADAGF	807	815	9	HLA-A*02:01	807	0.0362	0.0865	6.75E-01	2.89E+00	0.332	A2	0.0362	0.0865	0.6747	2.885	0.332	TRUE	TRUE	TRUE	TRUE	TRUE
1	VTLADAGFM	808	816	9	HLA-A*02:01	808	0.0622	0.1488	2.48E-02	4.64E-01	0.1758	A2	0.0622	0.1488	0.0248	0.464	0.1758	TRUE	TRUE	TRUE	TRUE	TRUE
1	TLADAGFMK	809	817	9	HLA-A*02:01	809	0.2599	0.622	9.98E-01	5.27E-01	0.7979	A2	0.2599	0.622	0.9976	0.527	0.7979	TRUE	TRUE	TRUE	TRUE	TRUE
1	LADAGFMKQ	810	818	9	HLA-A*02:01	810	0.0424	0.1014	9.33E-01	-2.15E-01	0.2305	A2	0.0424	0.1014	0.9327	-0.215	0.2305	TRUE	TRUE	TRUE	TRUE	TRUE
1	ADAGFMKQY	811	819	9	HLA-A*02:01	811	0.0178	0.0426	9.93E-01	2.83E+00	0.3328	A2	0.0178	0.0426	0.9925	2.826	0.3328	TRUE	TRUE	TRUE	TRUE	TRUE
1	DAGFMKQYG	812	820	9	HLA-A*02:01	812	0.0154	0.0369	1.21E-02	-1.67E+00	-0.0447	A2	0.0154	0.0369	0.0121	-1.668	-0.0447	TRUE	TRUE	TRUE	TRUE	TRUE
1	AGFMKQYGE	813	821	9	HLA-A*02:01	813	0.0174	0.0417	1.21E-02	-1.72E+00	-0.0423	A2	0.0174	0.0417	0.0121	-1.715	-0.0423	TRUE	TRUE	TRUE	TRUE	TRUE
1	GFMKQYGEC	814	822	9	HLA-A*02:01	814	0.0679	0.1624	1.01E-02	2.22E-01	0.175	A2	0.0679	0.1624	0.0101	0.222	0.175	TRUE	TRUE	TRUE	TRUE	TRUE
1	FMKQYGECL	815	823	9	HLA-A*02:01	815	0.4219	1.0097	1.53E-01	9.49E-01	1.0801	A2	0.4219	1.0097	0.1527	0.949	1.0801	TRUE	TRUE	TRUE	TRUE	TRUE
1	MKQYGECLG	816	824	9	HLA-A*02:01	816	0.0173	0.0413	1.51E-02	-1.44E+00	-0.0282	A2	0.0173	0.0413	0.0151	-1.436	-0.0282	TRUE	TRUE	TRUE	TRUE	TRUE
1	KQYGECLGD	817	825	9	HLA-A*02:01	817	0.0286	0.0684	1.29E-02	-1.41E+00	-0.0002	A2	0.0286	0.0684	0.0129	-1.41	-0.0002	TRUE	TRUE	TRUE	TRUE	TRUE
1	QYGECLGDI	818	826	9	HLA-A*02:01	818	0.0467	0.1117	1.63E-01	7.50E-01	0.1737	A2	0.0467	0.1117	0.163	0.75	0.1737	TRUE	TRUE	TRUE	TRUE	TRUE
1	YGECLGDIN	819	827	9	HLA-A*02:01	819	0.0152	0.0363	9.70E-03	-1.80E+00	-0.052	A2	0.0152	0.0363	0.0097	-1.795	-0.052	TRUE	TRUE	TRUE	TRUE	TRUE
1	GECLGDINA	820	828	9	HLA-A*02:01	820	0.0204	0.0489	2.77E-02	-8.93E-01	0.0084	A2	0.0204	0.0489	0.0277	-0.893	0.0084	TRUE	TRUE	TRUE	TRUE	TRUE
1	ECLGDINAR	821	829	9	HLA-A*02:01	821	0.0314	0.0752	3.24E-02	1.29E+00	0.1445	A2	0.0314	0.0752	0.0324	1.289	0.1445	TRUE	TRUE	TRUE	TRUE	TRUE
1	CLGDINARD	822	830	9	HLA-A*02:01	822	0.1203	0.2878	6.89E-02	-2.17E+00	0.1898	A2	0.1203	0.2878	0.0689	-2.167	0.1898	TRUE	TRUE	TRUE	TRUE	TRUE
1	LGDINARDL	823	831	9	HLA-A*02:01	823	0.022	0.0526	2.12E-01	5.09E-01	0.1099	A2	0.022	0.0526	0.2124	0.509	0.1099	TRUE	TRUE	TRUE	TRUE	TRUE
1	GDINARDLI	824	832	9	HLA-A*02:01	824	0.024	0.0573	3.17E-01	1.65E-01	0.1132	A2	0.024	0.0573	0.3174	0.165	0.1132	TRUE	TRUE	TRUE	TRUE	TRUE
1	DINARDLIC	825	833	9	HLA-A*02:01	825	0.0352	0.0843	1.01E-02	-2.97E-01	0.071	A2	0.0352	0.0843	0.0101	-0.297	0.071	TRUE	TRUE	TRUE	TRUE	TRUE
1	INARDLICA	826	834	9	HLA-A*02:01	826	0.0273	0.0654	1.49E-02	-7.87E-01	0.0283	A2	0.0273	0.0654	0.0149	-0.787	0.0283	TRUE	TRUE	TRUE	TRUE	TRUE
1	NARDLICAQ	827	835	9	HLA-A*02:01	827	0.0372	0.089	5.90E-02	4.20E-02	0.0999	A2	0.0372	0.089	0.059	0.042	0.0999	TRUE	TRUE	TRUE	TRUE	TRUE
1	ARDLICAQK	828	836	9	HLA-A*02:01	828	0.0172	0.0413	9.98E-01	7.80E-01	0.23	A2	0.0172	0.0413	0.9981	0.78	0.23	TRUE	TRUE	TRUE	TRUE	TRUE
1	RDLICAQKF	829	837	9	HLA-A*02:01	829	0.0168	0.0403	7.12E-02	2.64E+00	0.1831	A2	0.0168	0.0403	0.0712	2.643	0.1831	TRUE	TRUE	TRUE	TRUE	TRUE
1	DLICAQKFN	830	838	9	HLA-A*02:01	830	0.0262	0.0627	1.04E-02	-1.41E+00	-0.0063	A2	0.0262	0.0627	0.0104	-1.411	-0.0063	TRUE	TRUE	TRUE	TRUE	TRUE
1	LICAQKFNG	831	839	9	HLA-A*02:01	831	0.0466	0.1114	9.80E-03	-1.39E+00	0.0432	A2	0.0466	0.1114	0.0098	-1.393	0.0432	TRUE	TRUE	TRUE	TRUE	TRUE
1	ICAQKFNGL	832	840	9	HLA-A*02:01	832	0.0632	0.1512	5.90E-01	1.06E+00	0.2929	A2	0.0632	0.1512	0.5902	1.063	0.2929	TRUE	TRUE	TRUE	TRUE	TRUE
1	CAQKFNGLT	833	841	9	HLA-A*02:01	833	0.0284	0.0679	1.74E-02	-7.17E-01	0.0346	A2	0.0284	0.0679	0.0174	-0.717	0.0346	TRUE	TRUE	TRUE	TRUE	TRUE
1	AQKFNGLTV	834	842	9	HLA-A*02:01	834	0.0527	0.1261	9.54E-01	5.25E-01	0.2954	A2	0.0527	0.1261	0.9539	0.525	0.2954	TRUE	TRUE	TRUE	TRUE	TRUE
1	QKFNGLTVL	835	843	9	HLA-A*02:01	835	0.0386	0.0924	9.94E-01	1.28E+00	0.3056	A2	0.0386	0.0924	0.9939	1.283	0.3056	TRUE	TRUE	TRUE	TRUE	TRUE
1	KFNGLTVLP	836	844	9	HLA-A*02:01	836	0.0366	0.0875	3.58E-02	2.36E-01	0.1047	A2	0.0366	0.0875	0.0358	0.236	0.1047	TRUE	TRUE	TRUE	TRUE	TRUE
1	FNGLTVLPP	837	845	9	HLA-A*02:01	837	0.0179	0.0428	1.32E-02	-2.05E-01	0.0345	A2	0.0179	0.0428	0.0132	-0.205	0.0345	TRUE	TRUE	TRUE	TRUE	TRUE
1	NGLTVLPPL	838	846	9	HLA-A*02:01	838	0.0225	0.0538	8.69E-01	6.38E-01	0.216	A2	0.0225	0.0538	0.8693	0.638	0.216	TRUE	TRUE	TRUE	TRUE	TRUE
1	GLTVLPPLL	839	847	9	HLA-A*02:01	839	0.0982	0.2351	9.28E-01	9.00E-01	0.4193	A2	0.0982	0.2351	0.9283	0.9	0.4193	TRUE	TRUE	TRUE	TRUE	TRUE
1	LTVLPPLLT	840	848	9	HLA-A*02:01	840	0.0281	0.0672	9.90E-03	-6.75E-01	0.0349	A2	0.0281	0.0672	0.0099	-0.675	0.0349	TRUE	TRUE	TRUE	TRUE	TRUE
1	TVLPPLLTD	841	849	9	HLA-A*02:01	841	0.0486	0.1163	1.34E-02	-1.63E+00	0.0367	A2	0.0486	0.1163	0.0134	-1.632	0.0367	TRUE	TRUE	TRUE	TRUE	TRUE
1	VLPPLLTDD	842	850	9	HLA-A*02:01	842	0.1207	0.2889	1.62E-02	-1.74E+00	0.2044	A2	0.1207	0.2889	0.0162	-1.739	0.2044	TRUE	TRUE	TRUE	TRUE	TRUE
1	LPPLLTDDM	843	851	9	HLA-A*02:01	843	0.0198	0.0474	5.35E-01	3.60E-02	0.1294	A2	0.0198	0.0474	0.5351	0.036	0.1294	TRUE	TRUE	TRUE	TRUE	TRUE
1	PPLLTDDMI	844	852	9	HLA-A*02:01	844	0.0199	0.0477	7.92E-01	-1.84E-01	0.1573	A2	0.0199	0.0477	0.7917	-0.184	0.1573	TRUE	TRUE	TRUE	TRUE	TRUE
1	PLLTDDMIA	845	853	9	HLA-A*02:01	845	0.1343	0.3213	2.80E-01	-1.12E+00	0.3071	A2	0.1343	0.3213	0.2797	-1.123	0.3071	TRUE	TRUE	TRUE	TRUE	TRUE
1	LLTDDMIAA	846	854	9	HLA-A*02:01	846	0.4534	1.085	2.51E-01	-6.93E-01	1.088	A2	0.4534	1.085	0.251	-0.693	1.088	TRUE	TRUE	TRUE	TRUE	TRUE
1	LTDDMIAAY	847	855	9	HLA-A*02:01	847	0.0841	0.2012	9.99E-01	2.78E+00	0.4899	A2	0.0841	0.2012	0.9989	2.779	0.4899	TRUE	TRUE	TRUE	TRUE	TRUE
1	TDDMIAAYT	848	856	9	HLA-A*02:01	848	0.0219	0.0523	1.33E-02	-1.16E+00	-0.0038	A2	0.0219	0.0523	0.0133	-1.162	-0.0038	TRUE	TRUE	TRUE	TRUE	TRUE
1	DDMIAAYTA	849	857	9	HLA-A*02:01	849	0.0184	0.044	7.94E-01	-1.08E+00	0.1088	A2	0.0184	0.044	0.7936	-1.084	0.1088	TRUE	TRUE	TRUE	TRUE	TRUE
1	DMIAAYTAA	850	858	9	HLA-A*02:01	850	0.1313	0.3143	5.34E-01	-8.00E-01	0.3545	A2	0.1313	0.3143	0.5342	-0.8	0.3545	TRUE	TRUE	TRUE	TRUE	TRUE
1	MIAAYTAAL	851	859	9	HLA-A*02:01	851	0.2533	0.6063	8.31E-01	1.01E+00	0.7813	A2	0.2533	0.6063	0.8308	1.008	0.7813	TRUE	TRUE	TRUE	TRUE	TRUE
1	IAAYTAALV	852	860	9	HLA-A*02:01	852	0.0926	0.2216	1.96E-01	4.43E-01	0.273	A2	0.0926	0.2216	0.1955	0.443	0.273	TRUE	TRUE	TRUE	TRUE	TRUE
1	AAYTAALVS	853	861	9	HLA-A*02:01	853	0.052	0.1244	2.33E-02	-1.85E+00	0.0353	A2	0.052	0.1244	0.0233	-1.853	0.0353	TRUE	TRUE	TRUE	TRUE	TRUE
1	AYTAALVSG	854	862	9	HLA-A*02:01	854	0.025	0.0598	3.82E-02	-8.69E-01	0.0221	A2	0.025	0.0598	0.0382	-0.869	0.0221	TRUE	TRUE	TRUE	TRUE	TRUE
1	YTAALVSGT	855	863	9	HLA-A*02:01	855	0.0789	0.1888	1.73E-02	-5.76E-01	0.1626	A2	0.0789	0.1888	0.0173	-0.576	0.1626	TRUE	TRUE	TRUE	TRUE	TRUE
1	TAALVSGTA	856	864	9	HLA-A*02:01	856	0.0273	0.0654	8.16E-01	-5.39E-01	0.1609	A2	0.0273	0.0654	0.816	-0.539	0.1609	TRUE	TRUE	TRUE	TRUE	TRUE
1	AALVSGTAT	857	865	9	HLA-A*02:01	857	0.0317	0.0759	4.25E-02	-4.81E-01	0.0583	A2	0.0317	0.0759	0.0425	-0.481	0.0583	TRUE	TRUE	TRUE	TRUE	TRUE
1	ALVSGTATA	858	866	9	HLA-A*02:01	858	0.3084	0.738	9.98E-01	-1.40E-01	0.8807	A2	0.3084	0.738	0.9976	-0.14	0.8807	TRUE	TRUE	TRUE	TRUE	TRUE
1	LVSGTATAG	859	867	9	HLA-A*02:01	859	0.0528	0.1264	2.13E-02	-1.10E+00	0.0745	A2	0.0528	0.1264	0.0213	-1.103	0.0745	TRUE	TRUE	TRUE	TRUE	TRUE
1	VSGTATAGW	860	868	9	HLA-A*02:01	860	0.0174	0.0416	6.50E-01	8.72E-01	0.1827	A2	0.0174	0.0416	0.6499	0.872	0.1827	TRUE	TRUE	TRUE	TRUE	TRUE
1	SGTATAGWT	861	869	9	HLA-A*02:01	861	0.0224	0.0535	9.80E-03	-9.08E-01	0.0096	A2	0.0224	0.0535	0.0098	-0.908	0.0096	TRUE	TRUE	TRUE	TRUE	TRUE
1	GTATAGWTF	862	870	9	HLA-A*02:01	862	0.0707	0.1692	5.99E-01	2.41E+00	0.3793	A2	0.0707	0.1692	0.599	2.405	0.3793	TRUE	TRUE	TRUE	TRUE	TRUE
1	TATAGWTFG	863	871	9	HLA-A*02:01	863	0.0259	0.062	2.25E-02	-1.43E+00	-0.0062	A2	0.0259	0.062	0.0225	-1.43	-0.0062	TRUE	TRUE	TRUE	TRUE	TRUE
1	ATAGWTFGA	864	872	9	HLA-A*02:01	864	0.4044	0.9678	6.45E-01	-3.86E-01	1.0452	A2	0.4044	0.9678	0.6446	-0.386	1.0452	TRUE	TRUE	TRUE	TRUE	TRUE
1	TAGWTFGAG	865	873	9	HLA-A*02:01	865	0.0228	0.0547	8.63E-02	-1.37E+00	-0.001	A2	0.0228	0.0547	0.0863	-1.373	-0.001	TRUE	TRUE	TRUE	TRUE	TRUE
1	AGWTFGAGA	866	874	9	HLA-A*02:01	866	0.0362	0.0867	7.36E-01	-4.76E-01	0.1733	A2	0.0362	0.0867	0.7359	-0.476	0.1733	TRUE	TRUE	TRUE	TRUE	TRUE
1	GWTFGAGAA	867	875	9	HLA-A*02:01	867	0.0247	0.0591	3.41E-01	-3.50E-01	0.0927	A2	0.0247	0.0591	0.3405	-0.35	0.0927	TRUE	TRUE	TRUE	TRUE	TRUE
1	WTFGAGAAL	868	876	9	HLA-A*02:01	868	0.3747	0.8968	9.46E-01	9.88E-01	1.0881	A2	0.3747	0.8968	0.9456	0.988	1.0881	TRUE	TRUE	TRUE	TRUE	TRUE
1	TFGAGAALQ	869	877	9	HLA-A*02:01	869	0.0279	0.0667	9.80E-03	-1.78E-01	0.0592	A2	0.0279	0.0667	0.0098	-0.178	0.0592	TRUE	TRUE	TRUE	TRUE	TRUE
1	FGAGAALQI	870	878	9	HLA-A*02:01	870	0.0892	0.2135	1.34E-01	2.00E-01	0.2436	A2	0.0892	0.2135	0.1337	0.2	0.2436	TRUE	TRUE	TRUE	TRUE	TRUE
1	GAGAALQIP	871	879	9	HLA-A*02:01	871	0.0221	0.0528	1.05E-02	-2.90E-01	0.0399	A2	0.0221	0.0528	0.0105	-0.29	0.0399	TRUE	TRUE	TRUE	TRUE	TRUE
1	AGAALQIPF	872	880	9	HLA-A*02:01	872	0.0207	0.0495	1.38E-02	2.40E+00	0.1715	A2	0.0207	0.0495	0.0138	2.399	0.1715	TRUE	TRUE	TRUE	TRUE	TRUE
1	GAALQIPFA	873	881	9	HLA-A*02:01	873	0.0386	0.0924	1.13E-02	-4.91E-01	0.0695	A2	0.0386	0.0924	0.0113	-0.491	0.0695	TRUE	TRUE	TRUE	TRUE	TRUE
1	AALQIPFAM	874	882	9	HLA-A*02:01	874	0.086	0.2059	8.72E-01	4.89E-01	0.3611	A2	0.086	0.2059	0.8719	0.489	0.3611	TRUE	TRUE	TRUE	TRUE	TRUE
1	ALQIPFAMQ	875	883	9	HLA-A*02:01	875	0.1064	0.2548	5.23E-01	1.80E-01	0.3421	A2	0.1064	0.2548	0.5226	0.18	0.3421	TRUE	TRUE	TRUE	TRUE	TRUE
1	LQIPFAMQM	876	884	9	HLA-A*02:01	876	0.0514	0.123	9.96E-01	6.07E-01	0.3026	A2	0.0514	0.123	0.9955	0.607	0.3026	TRUE	TRUE	TRUE	TRUE	TRUE
1	QIPFAMQMA	877	885	9	HLA-A*02:01	877	0.0619	0.148	2.16E-01	-3.61E-01	0.1624	A2	0.0619	0.148	0.2159	-0.361	0.1624	TRUE	TRUE	TRUE	TRUE	TRUE
1	IPFAMQMAY	878	886	9	HLA-A*02:01	878	0.0191	0.0457	9.97E-01	2.71E+00	0.3305	A2	0.0191	0.0457	0.9965	2.706	0.3305	TRUE	TRUE	TRUE	TRUE	TRUE
1	PFAMQMAYR	879	887	9	HLA-A*02:01	879	0.0255	0.061	1.09E-02	1.20E+00	0.1226	A2	0.0255	0.061	0.0109	1.198	0.1226	TRUE	TRUE	TRUE	TRUE	TRUE
1	FAMQMAYRF	880	888	9	HLA-A*02:01	880	0.1734	0.4149	1.26E-01	2.41E+00	0.5545	A2	0.1734	0.4149	0.1263	2.412	0.5545	TRUE	TRUE	TRUE	TRUE	TRUE
1	AMQMAYRFN	881	889	9	HLA-A*02:01	881	0.0894	0.214	9.80E-03	-1.19E+00	0.1561	A2	0.0894	0.214	0.0098	-1.187	0.1561	TRUE	TRUE	TRUE	TRUE	TRUE
1	MQMAYRFNG	882	890	9	HLA-A*02:01	882	0.0298	0.0712	9.60E-03	-1.05E+00	0.02	A2	0.0298	0.0712	0.0096	-1.054	0.02	TRUE	TRUE	TRUE	TRUE	TRUE
1	QMAYRFNGI	883	891	9	HLA-A*02:01	883	0.1759	0.421	8.17E-02	7.22E-01	0.4693	A2	0.1759	0.421	0.0817	0.722	0.4693	TRUE	TRUE	TRUE	TRUE	TRUE
1	MAYRFNGIG	884	892	9	HLA-A*02:01	884	0.0274	0.0656	1.31E-02	-1.16E+00	0.0097	A2	0.0274	0.0656	0.0131	-1.158	0.0097	TRUE	TRUE	TRUE	TRUE	TRUE
1	AYRFNGIGV	885	893	9	HLA-A*02:01	885	0.0823	0.1969	1.64E-01	8.08E-01	0.2619	A2	0.0823	0.1969	0.1637	0.808	0.2619	TRUE	TRUE	TRUE	TRUE	TRUE
1	YRFNGIGVT	886	894	9	HLA-A*02:01	886	0.0414	0.099	7.20E-01	-2.53E-01	0.1943	A2	0.0414	0.099	0.7198	-0.253	0.1943	TRUE	TRUE	TRUE	TRUE	TRUE
1	RFNGIGVTQ	887	895	9	HLA-A*02:01	887	0.0375	0.0897	6.18E-02	1.60E-01	0.107	A2	0.0375	0.0897	0.0618	0.16	0.107	TRUE	TRUE	TRUE	TRUE	TRUE
1	FNGIGVTQN	888	896	9	HLA-A*02:01	888	0.015	0.036	1.18E-02	-1.59E+00	-0.042	A2	0.015	0.036	0.0118	-1.594	-0.042	TRUE	TRUE	TRUE	TRUE	TRUE
1	NGIGVTQNV	889	897	9	HLA-A*02:01	889	0.0484	0.1158	7.36E-01	4.90E-02	0.2286	A2	0.0484	0.1158	0.7358	0.049	0.2286	TRUE	TRUE	TRUE	TRUE	TRUE
1	GIGVTQNVL	890	898	9	HLA-A*02:01	890	0.0502	0.1202	9.96E-01	8.82E-01	0.3136	A2	0.0502	0.1202	0.9956	0.882	0.3136	TRUE	TRUE	TRUE	TRUE	TRUE
1	IGVTQNVLY	891	899	9	HLA-A*02:01	891	0.0158	0.0378	9.95E-01	2.73E+00	0.3235	A2	0.0158	0.0378	0.995	2.729	0.3235	TRUE	TRUE	TRUE	TRUE	TRUE
1	GVTQNVLYE	892	900	9	HLA-A*02:01	892	0.0673	0.1612	1.45E-02	-1.63E+00	0.0817	A2	0.0673	0.1612	0.0145	-1.633	0.0817	TRUE	TRUE	TRUE	TRUE	TRUE
1	VTQNVLYEN	893	901	9	HLA-A*02:01	893	0.0385	0.0921	1.13E-02	-1.34E+00	0.0269	A2	0.0385	0.0921	0.0113	-1.337	0.0269	TRUE	TRUE	TRUE	TRUE	TRUE
1	TQNVLYENQ	894	902	9	HLA-A*02:01	894	0.0174	0.0417	1.00E-02	-9.80E-02	0.0383	A2	0.0174	0.0417	0.01	-0.098	0.0383	TRUE	TRUE	TRUE	TRUE	TRUE
1	QNVLYENQK	895	903	9	HLA-A*02:01	895	0.0145	0.0348	9.14E-01	4.13E-01	0.1926	A2	0.0145	0.0348	0.9144	0.413	0.1926	TRUE	TRUE	TRUE	TRUE	TRUE
1	NVLYENQKQ	896	904	9	HLA-A*02:01	896	0.0286	0.0684	1.13E-02	3.90E-02	0.0721	A2	0.0286	0.0684	0.0113	0.039	0.0721	TRUE	TRUE	TRUE	TRUE	TRUE
1	VLYENQKQI	897	905	9	HLA-A*02:01	897	0.4306	1.0306	9.84E-01	9.44E-01	1.2253	A2	0.4306	1.0306	0.9838	0.944	1.2253	TRUE	TRUE	TRUE	TRUE	TRUE
1	LYENQKQIA	898	906	9	HLA-A*02:01	898	0.0272	0.0651	2.99E-01	-3.64E-01	0.0918	A2	0.0272	0.0651	0.299	-0.364	0.0918	TRUE	TRUE	TRUE	TRUE	TRUE
1	YENQKQIAN	899	907	9	HLA-A*02:01	899	0.0174	0.0416	1.16E-02	-1.52E+00	-0.0326	A2	0.0174	0.0416	0.0116	-1.517	-0.0326	TRUE	TRUE	TRUE	TRUE	TRUE
1	ENQKQIANQ	900	908	9	HLA-A*02:01	900	0.0197	0.0471	1.22E-02	-5.46E-01	0.0216	A2	0.0197	0.0471	0.0122	-0.546	0.0216	TRUE	TRUE	TRUE	TRUE	TRUE
1	NQKQIANQF	901	909	9	HLA-A*02:01	901	0.0185	0.0444	5.02E-01	2.48E+00	0.2436	A2	0.0185	0.0444	0.5023	2.478	0.2436	TRUE	TRUE	TRUE	TRUE	TRUE
1	QKQIANQFN	902	910	9	HLA-A*02:01	902	0.0162	0.0389	9.60E-03	-1.14E+00	-0.0169	A2	0.0162	0.0389	0.0096	-1.144	-0.0169	TRUE	TRUE	TRUE	TRUE	TRUE
1	KQIANQFNK	903	911	9	HLA-A*02:01	903	0.0261	0.0624	9.49E-01	6.67E-01	0.238	A2	0.0261	0.0624	0.9485	0.667	0.238	TRUE	TRUE	TRUE	TRUE	TRUE
1	QIANQFNKA	904	912	9	HLA-A*02:01	904	0.0673	0.1611	5.72E-01	-2.76E-01	0.2331	A2	0.0673	0.1611	0.5716	-0.276	0.2331	TRUE	TRUE	TRUE	TRUE	TRUE
1	IANQFNKAI	905	913	9	HLA-A*02:01	905	0.0551	0.1318	8.34E-01	4.67E-01	0.2803	A2	0.0551	0.1318	0.8344	0.467	0.2803	TRUE	TRUE	TRUE	TRUE	TRUE
1	ANQFNKAIS	906	914	9	HLA-A*02:01	906	0.0169	0.0403	1.04E-02	-2.28E+00	-0.0719	A2	0.0169	0.0403	0.0104	-2.277	-0.0719	TRUE	TRUE	TRUE	TRUE	TRUE
1	NQFNKAISQ	907	915	9	HLA-A*02:01	907	0.019	0.0455	1.84E-02	2.73E-01	0.0619	A2	0.019	0.0455	0.0184	0.273	0.0619	TRUE	TRUE	TRUE	TRUE	TRUE
1	QFNKAISQI	908	916	9	HLA-A*02:01	908	0.0689	0.1649	9.94E-01	7.62E-01	0.3521	A2	0.0689	0.1649	0.9936	0.762	0.3521	TRUE	TRUE	TRUE	TRUE	TRUE
1	FNKAISQIQ	909	917	9	HLA-A*02:01	909	0.0211	0.0504	1.55E-02	-4.04E-01	0.0325	A2	0.0211	0.0504	0.0155	-0.404	0.0325	TRUE	TRUE	TRUE	TRUE	TRUE
1	NKAISQIQE	910	918	9	HLA-A*02:01	910	0.0154	0.0369	1.15E-02	-1.57E+00	-0.0398	A2	0.0154	0.0369	0.0115	-1.567	-0.0398	TRUE	TRUE	TRUE	TRUE	TRUE
1	KAISQIQES	911	919	9	HLA-A*02:01	911	0.0431	0.1031	1.43E-02	-1.88E+00	0.0114	A2	0.0431	0.1031	0.0143	-1.876	0.0114	TRUE	TRUE	TRUE	TRUE	TRUE
1	AISQIQESL	912	920	9	HLA-A*02:01	912	0.176	0.4211	9.97E-01	1.32E+00	0.6369	A2	0.176	0.4211	0.9973	1.324	0.6369	TRUE	TRUE	TRUE	TRUE	TRUE
1	ISQIQESLT	913	921	9	HLA-A*02:01	913	0.0181	0.0432	1.11E-02	-5.20E-01	0.0189	A2	0.0181	0.0432	0.0111	-0.52	0.0189	TRUE	TRUE	TRUE	TRUE	TRUE
1	SQIQESLTT	914	922	9	HLA-A*02:01	914	0.0326	0.0781	2.24E-02	-4.99E-01	0.0565	A2	0.0326	0.0781	0.0224	-0.499	0.0565	TRUE	TRUE	TRUE	TRUE	TRUE
1	QIQESLTTT	915	923	9	HLA-A*02:01	915	0.1631	0.3903	4.69E-01	-5.42E-01	0.4335	A2	0.1631	0.3903	0.4685	-0.542	0.4335	TRUE	TRUE	TRUE	TRUE	TRUE
1	IQESLTTTS	916	924	9	HLA-A*02:01	916	0.0242	0.0579	3.40E-02	-2.34E+00	-0.054	A2	0.0242	0.0579	0.034	-2.34	-0.054	TRUE	TRUE	TRUE	TRUE	TRUE
1	QESLTTTST	917	925	9	HLA-A*02:01	917	0.0171	0.0409	9.54E-01	-8.23E-01	0.1428	A2	0.0171	0.0409	0.954	-0.823	0.1428	TRUE	TRUE	TRUE	TRUE	TRUE
1	ESLTTTSTA	918	926	9	HLA-A*02:01	918	0.0228	0.0545	9.57E-01	-7.30E-01	0.1614	A2	0.0228	0.0545	0.9566	-0.73	0.1614	TRUE	TRUE	TRUE	TRUE	TRUE
1	SLTTTSTAL	919	927	9	HLA-A*02:01	919	0.2247	0.5378	9.99E-01	8.24E-01	0.7289	A2	0.2247	0.5378	0.999	0.824	0.7289	TRUE	TRUE	TRUE	TRUE	TRUE
1	LTTTSTALG	920	928	9	HLA-A*02:01	920	0.029	0.0693	1.82E-02	-1.40E+00	0.0023	A2	0.029	0.0693	0.0182	-1.395	0.0023	TRUE	TRUE	TRUE	TRUE	TRUE
1	TTTSTALGK	921	929	9	HLA-A*02:01	921	0.031	0.0743	6.49E-02	4.34E-01	0.1057	A2	0.031	0.0743	0.0649	0.434	0.1057	TRUE	TRUE	TRUE	TRUE	TRUE
1	TTSTALGKL	922	930	9	HLA-A*02:01	922	0.0515	0.1233	7.96E-01	9.18E-01	0.2886	A2	0.0515	0.1233	0.7962	0.918	0.2886	TRUE	TRUE	TRUE	TRUE	TRUE
1	TSTALGKLQ	923	931	9	HLA-A*02:01	923	0.0177	0.0423	9.70E-03	-1.12E-01	0.0381	A2	0.0177	0.0423	0.0097	-0.112	0.0381	TRUE	TRUE	TRUE	TRUE	TRUE
1	STALGKLQD	924	932	9	HLA-A*02:01	924	0.0181	0.0432	1.51E-02	-1.76E+00	-0.0424	A2	0.0181	0.0432	0.0151	-1.757	-0.0424	TRUE	TRUE	TRUE	TRUE	TRUE
1	TALGKLQDV	925	933	9	HLA-A*02:01	925	0.1466	0.3508	3.38E-02	4.18E-01	0.3767	A2	0.1466	0.3508	0.0338	0.418	0.3767	TRUE	TRUE	TRUE	TRUE	TRUE
1	ALGKLQDVV	926	934	9	HLA-A*02:01	926	0.3626	0.8677	2.36E-01	3.25E-01	0.9193	A2	0.3626	0.8677	0.2361	0.325	0.9193	TRUE	TRUE	TRUE	TRUE	TRUE
1	LGKLQDVVN	927	935	9	HLA-A*02:01	927	0.0143	0.0343	1.11E-02	-1.43E+00	-0.0354	A2	0.0143	0.0343	0.0111	-1.426	-0.0354	TRUE	TRUE	TRUE	TRUE	TRUE
1	GKLQDVVNQ	928	936	9	HLA-A*02:01	928	0.0247	0.0592	1.35E-02	-1.69E-01	0.0528	A2	0.0247	0.0592	0.0135	-0.169	0.0528	TRUE	TRUE	TRUE	TRUE	TRUE
1	KLQDVVNQN	929	937	9	HLA-A*02:01	929	0.1262	0.302	6.29E-01	-1.25E+00	0.3339	A2	0.1262	0.302	0.6291	-1.251	0.3339	TRUE	TRUE	TRUE	TRUE	TRUE
1	LQDVVNQNA	930	938	9	HLA-A*02:01	930	0.0269	0.0644	3.75E-01	-5.31E-01	0.0941	A2	0.0269	0.0644	0.3747	-0.531	0.0941	TRUE	TRUE	TRUE	TRUE	TRUE
1	QDVVNQNAQ	931	939	9	HLA-A*02:01	931	0.0154	0.0369	1.12E-02	-2.74E-01	0.0249	A2	0.0154	0.0369	0.0112	-0.274	0.0249	TRUE	TRUE	TRUE	TRUE	TRUE
1	DVVNQNAQA	932	940	9	HLA-A*02:01	932	0.0351	0.0841	4.97E-01	-6.84E-01	0.1245	A2	0.0351	0.0841	0.4974	-0.684	0.1245	TRUE	TRUE	TRUE	TRUE	TRUE
1	VVNQNAQAL	933	941	9	HLA-A*02:01	933	0.1685	0.4032	6.65E-01	9.22E-01	0.5491	A2	0.1685	0.4032	0.6654	0.922	0.5491	TRUE	TRUE	TRUE	TRUE	TRUE
1	VNQNAQALN	934	942	9	HLA-A*02:01	934	0.016	0.0384	1.09E-02	-1.39E+00	-0.0296	A2	0.016	0.0384	0.0109	-1.391	-0.0296	TRUE	TRUE	TRUE	TRUE	TRUE
1	NQNAQALNT	935	943	9	HLA-A*02:01	935	0.0189	0.0452	1.50E-02	-6.40E-01	0.0155	A2	0.0189	0.0452	0.015	-0.64	0.0155	TRUE	TRUE	TRUE	TRUE	TRUE
1	QNAQALNTL	936	944	9	HLA-A*02:01	936	0.0263	0.0629	5.19E-01	9.73E-01	0.1894	A2	0.0263	0.0629	0.519	0.973	0.1894	TRUE	TRUE	TRUE	TRUE	TRUE
1	NAQALNTLV	937	945	9	HLA-A*02:01	937	0.0436	0.1043	4.64E-01	3.23E-01	0.1901	A2	0.0436	0.1043	0.4641	0.323	0.1901	TRUE	TRUE	TRUE	TRUE	TRUE
1	AQALNTLVK	938	946	9	HLA-A*02:01	938	0.0204	0.0489	9.98E-01	8.42E-01	0.2407	A2	0.0204	0.0489	0.9984	0.842	0.2407	TRUE	TRUE	TRUE	TRUE	TRUE
1	QALNTLVKQ	939	947	9	HLA-A*02:01	939	0.0268	0.0641	3.30E-01	1.32E-01	0.1202	A2	0.0268	0.0641	0.3299	0.132	0.1202	TRUE	TRUE	TRUE	TRUE	TRUE
1	ALNTLVKQL	940	948	9	HLA-A*02:01	940	0.1933	0.4625	9.97E-01	9.75E-01	0.6608	A2	0.1933	0.4625	0.9974	0.975	0.6608	TRUE	TRUE	TRUE	TRUE	TRUE
1	LNTLVKQLS	941	949	9	HLA-A*02:01	941	0.0164	0.0391	1.08E-02	-2.28E+00	-0.073	A2	0.0164	0.0391	0.0108	-2.276	-0.073	TRUE	TRUE	TRUE	TRUE	TRUE
1	NTLVKQLSS	942	950	9	HLA-A*02:01	942	0.0197	0.0471	1.88E-02	-2.16E+00	-0.0583	A2	0.0197	0.0471	0.0188	-2.164	-0.0583	TRUE	TRUE	TRUE	TRUE	TRUE
1	TLVKQLSSN	943	951	9	HLA-A*02:01	943	0.0481	0.1151	1.10E-02	-1.10E+00	0.0618	A2	0.0481	0.1151	0.011	-1.098	0.0618	TRUE	TRUE	TRUE	TRUE	TRUE
1	LVKQLSSNF	944	952	9	HLA-A*02:01	944	0.0654	0.1565	1.56E-01	2.69E+00	0.3141	A2	0.0654	0.1565	0.1559	2.685	0.3141	TRUE	TRUE	TRUE	TRUE	TRUE
1	VKQLSSNFG	945	953	9	HLA-A*02:01	945	0.0191	0.0457	1.22E-02	-1.27E+00	-0.0159	A2	0.0191	0.0457	0.0122	-1.268	-0.0159	TRUE	TRUE	TRUE	TRUE	TRUE
1	KQLSSNFGA	946	954	9	HLA-A*02:01	946	0.0574	0.1374	3.30E-02	-2.88E-01	0.128	A2	0.0574	0.1374	0.033	-0.288	0.128	TRUE	TRUE	TRUE	TRUE	TRUE
1	QLSSNFGAI	947	955	9	HLA-A*02:01	947	0.1631	0.3904	9.55E-01	6.64E-01	0.5668	A2	0.1631	0.3904	0.9546	0.664	0.5668	TRUE	TRUE	TRUE	TRUE	TRUE
1	LSSNFGAIS	948	956	9	HLA-A*02:01	948	0.023	0.055	1.69E-02	-2.25E+00	-0.0552	A2	0.023	0.055	0.0169	-2.254	-0.0552	TRUE	TRUE	TRUE	TRUE	TRUE
1	SSNFGAISS	949	957	9	HLA-A*02:01	949	0.0203	0.0486	1.46E-02	-2.20E+00	-0.0591	A2	0.0203	0.0486	0.0146	-2.198	-0.0591	TRUE	TRUE	TRUE	TRUE	TRUE
1	SNFGAISSV	950	958	9	HLA-A*02:01	950	0.0839	0.2009	8.99E-01	3.81E-01	0.3548	A2	0.0839	0.2009	0.8992	0.381	0.3548	TRUE	TRUE	TRUE	TRUE	TRUE
1	NFGAISSVL	951	959	9	HLA-A*02:01	951	0.0314	0.0753	7.02E-01	1.05E+00	0.2331	A2	0.0314	0.0753	0.7024	1.05	0.2331	TRUE	TRUE	TRUE	TRUE	TRUE
1	FGAISSVLN	952	960	9	HLA-A*02:01	952	0.0169	0.0404	1.06E-02	-1.52E+00	-0.034	A2	0.0169	0.0404	0.0106	-1.519	-0.034	TRUE	TRUE	TRUE	TRUE	TRUE
1	GAISSVLND	953	961	9	HLA-A*02:01	953	0.0247	0.0591	1.18E-02	-1.98E+00	-0.038	A2	0.0247	0.0591	0.0118	-1.978	-0.038	TRUE	TRUE	TRUE	TRUE	TRUE
1	AISSVLNDI	954	962	9	HLA-A*02:01	954	0.1808	0.4326	8.83E-01	7.73E-01	0.6038	A2	0.1808	0.4326	0.8833	0.773	0.6038	TRUE	TRUE	TRUE	TRUE	TRUE
1	ISSVLNDIL	955	963	9	HLA-A*02:01	955	0.0242	0.058	5.35E-01	1.17E+00	0.1966	A2	0.0242	0.058	0.5347	1.168	0.1966	TRUE	TRUE	TRUE	TRUE	TRUE
1	SSVLNDILS	956	964	9	HLA-A*02:01	956	0.0182	0.0435	2.26E-02	-2.07E+00	-0.0563	A2	0.0182	0.0435	0.0226	-2.065	-0.0563	TRUE	TRUE	TRUE	TRUE	TRUE
1	SVLNDILSR	957	965	9	HLA-A*02:01	957	0.0463	0.1109	7.35E-01	1.75E+00	0.3085	A2	0.0463	0.1109	0.7352	1.748	0.3085	TRUE	TRUE	TRUE	TRUE	TRUE
1	VLNDILSRL	958	966	9	HLA-A*02:01	958	0.4231	1.0125	9.98E-01	9.96E-01	1.212	A2	0.4231	1.0125	0.9981	0.996	1.212	TRUE	TRUE	TRUE	TRUE	TRUE
1	LNDILSRLD	959	967	9	HLA-A*02:01	959	0.0153	0.0365	1.17E-02	-2.09E+00	-0.0664	A2	0.0153	0.0365	0.0117	-2.093	-0.0664	TRUE	TRUE	TRUE	TRUE	TRUE
1	NDILSRLDK	960	968	9	HLA-A*02:01	960	0.0156	0.0374	5.67E-02	3.11E-01	0.0614	A2	0.0156	0.0374	0.0567	0.311	0.0614	TRUE	TRUE	TRUE	TRUE	TRUE
1	DILSRLDKV	961	969	9	HLA-A*02:01	961	0.1119	0.2678	2.26E-01	2.86E-01	0.316	A2	0.1119	0.2678	0.2261	0.286	0.316	TRUE	TRUE	TRUE	TRUE	TRUE
1	ILSRLDKVE	962	970	9	HLA-A*02:01	962	0.0628	0.1502	1.10E-02	-1.67E+00	0.0686	A2	0.0628	0.1502	0.011	-1.666	0.0686	TRUE	TRUE	TRUE	TRUE	TRUE
1	LSRLDKVEA	963	971	9	HLA-A*02:01	963	0.0197	0.0471	2.43E-01	-4.33E-01	0.0619	A2	0.0197	0.0471	0.2428	-0.433	0.0619	TRUE	TRUE	TRUE	TRUE	TRUE
1	SRLDKVEAE	964	972	9	HLA-A*02:01	964	0.0195	0.0466	1.12E-02	-1.24E+00	-0.0137	A2	0.0195	0.0466	0.0112	-1.24	-0.0137	TRUE	TRUE	TRUE	TRUE	TRUE
1	RLDKVEAEV	965	973	9	HLA-A*02:01	965	0.39	0.9333	9.99E-01	4.56E-01	1.1059	A2	0.39	0.9333	0.9986	0.456	1.1059	TRUE	TRUE	TRUE	TRUE	TRUE
1	LDKVEAEVQ	966	974	9	HLA-A*02:01	966	0.0158	0.0377	1.04E-02	-2.79E-01	0.0253	A2	0.0158	0.0377	0.0104	-0.279	0.0253	TRUE	TRUE	TRUE	TRUE	TRUE

seq #	peptide	start	end	peptide length	allele	peptide index	NetCTL Predict	NetCTL Rescale	NetCTL c termi	NetCTL TAP tra	NetCTL Predict	NetCTL Superty	legacy predicte	legacy rescale_	legacy c_termi	legacy tap_tra	legacy predicti	equal predicted	equal rescale_	equal c_termin	equal tap_tran	equal predictions_score
1	DKVEAEVQI	967	975	9	HLA-A*02:01	967	0.0254	0.0608	5.21E-01	3.94E-01	0.1587	A2	0.0254	0.0608	0.5212	0.394	0.1587	TRUE	TRUE	TRUE	TRUE	TRUE
1	KVEAEVQID	968	976	9	HLA-A*02:01	968	0.0254	0.0608	1.04E-02	-1.85E+00	-0.0303	A2	0.0254	0.0608	0.0104	-1.853	-0.0303	TRUE	TRUE	TRUE	TRUE	TRUE
1	VEAEVQIDR	969	977	9	HLA-A*02:01	969	0.0236	0.0564	6.67E-01	1.53E+00	0.233	A2	0.0236	0.0564	0.6673	1.529	0.233	TRUE	TRUE	TRUE	TRUE	TRUE
1	EAEVQIDRL	970	978	9	HLA-A*02:01	970	0.0312	0.0746	4.24E-01	7.06E-01	0.1735	A2	0.0312	0.0746	0.4238	0.706	0.1735	TRUE	TRUE	TRUE	TRUE	TRUE
1	AEVQIDRLI	971	979	9	HLA-A*02:01	971	0.0309	0.0739	7.03E-01	4.54E-01	0.202	A2	0.0309	0.0739	0.703	0.454	0.202	TRUE	TRUE	TRUE	TRUE	TRUE
1	EVQIDRLIT	972	980	9	HLA-A*02:01	972	0.0324	0.0775	1.08E-02	-6.49E-01	0.0466	A2	0.0324	0.0775	0.0108	-0.649	0.0466	TRUE	TRUE	TRUE	TRUE	TRUE
1	VQIDRLITG	973	981	9	HLA-A*02:01	973	0.0297	0.0712	3.78E-02	-1.36E+00	0.0091	A2	0.0297	0.0712	0.0378	-1.356	0.0091	TRUE	TRUE	TRUE	TRUE	TRUE
1	QIDRLITGR	974	982	9	HLA-A*02:01	974	0.0469	0.1123	7.42E-01	1.50E+00	0.2984	A2	0.0469	0.1123	0.7415	1.497	0.2984	TRUE	TRUE	TRUE	TRUE	TRUE
1	IDRLITGRL	975	983	9	HLA-A*02:01	975	0.0225	0.0539	9.96E-01	6.70E-01	0.2368	A2	0.0225	0.0539	0.996	0.67	0.2368	TRUE	TRUE	TRUE	TRUE	TRUE
1	DRLITGRLQ	976	984	9	HLA-A*02:01	976	0.0149	0.0357	1.17E-02	-1.88E-01	0.028	A2	0.0149	0.0357	0.0117	-0.188	0.028	TRUE	TRUE	TRUE	TRUE	TRUE
1	RLITGRLQS	977	985	9	HLA-A*02:01	977	0.1221	0.2923	1.38E-02	-2.05E+00	0.1918	A2	0.1221	0.2923	0.0138	-2.052	0.1918	TRUE	TRUE	TRUE	TRUE	TRUE
1	LITGRLQSL	978	986	9	HLA-A*02:01	978	0.24	0.5744	9.88E-01	1.18E+00	0.7818	A2	0.24	0.5744	0.9882	1.183	0.7818	TRUE	TRUE	TRUE	TRUE	TRUE
1	ITGRLQSLQ	979	987	9	HLA-A*02:01	979	0.0223	0.0533	1.04E-02	-2.19E-01	0.0439	A2	0.0223	0.0533	0.0104	-0.219	0.0439	TRUE	TRUE	TRUE	TRUE	TRUE
1	TGRLQSLQT	980	988	9	HLA-A*02:01	980	0.0166	0.0397	2.46E-02	-9.35E-01	-0.0034	A2	0.0166	0.0397	0.0246	-0.935	-0.0034	TRUE	TRUE	TRUE	TRUE	TRUE
1	GRLQSLQTY	981	989	9	HLA-A*02:01	981	0.0186	0.0446	9.95E-01	2.98E+00	0.3429	A2	0.0186	0.0446	0.9953	2.98	0.3429	TRUE	TRUE	TRUE	TRUE	TRUE
1	RLQSLQTYV	982	990	9	HLA-A*02:01	982	0.399	0.955	9.28E-01	5.16E-01	1.1199	A2	0.399	0.955	0.9276	0.516	1.1199	TRUE	TRUE	TRUE	TRUE	TRUE
1	LQSLQTYVT	983	991	9	HLA-A*02:01	983	0.0255	0.061	1.26E-01	-4.98E-01	0.0549	A2	0.0255	0.061	0.1255	-0.498	0.0549	TRUE	TRUE	TRUE	TRUE	TRUE
1	QSLQTYVTQ	984	992	9	HLA-A*02:01	984	0.0212	0.0508	5.33E-02	1.40E-02	0.0595	A2	0.0212	0.0508	0.0533	0.014	0.0595	TRUE	TRUE	TRUE	TRUE	TRUE
1	SLQTYVTQQ	985	993	9	HLA-A*02:01	985	0.0935	0.2239	1.75E-01	-1.14E-01	0.2444	A2	0.0935	0.2239	0.1751	-0.114	0.2444	TRUE	TRUE	TRUE	TRUE	TRUE
1	LQTYVTQQL	986	994	9	HLA-A*02:01	986	0.0395	0.0947	8.89E-01	1.05E+00	0.2806	A2	0.0395	0.0947	0.8892	1.052	0.2806	TRUE	TRUE	TRUE	TRUE	TRUE
1	QTYVTQQLI	987	995	9	HLA-A*02:01	987	0.0661	0.1583	7.53E-01	7.61E-01	0.3093	A2	0.0661	0.1583	0.7531	0.761	0.3093	TRUE	TRUE	TRUE	TRUE	TRUE
1	TYVTQQLIR	988	996	9	HLA-A*02:01	988	0.0188	0.0449	6.89E-01	1.76E+00	0.2361	A2	0.0188	0.0449	0.6894	1.757	0.2361	TRUE	TRUE	TRUE	TRUE	TRUE
1	YVTQQLIRA	989	997	9	HLA-A*02:01	989	0.2622	0.6274	7.19E-01	-4.43E-01	0.7132	A2	0.2622	0.6274	0.7193	-0.443	0.7132	TRUE	TRUE	TRUE	TRUE	TRUE
1	VTQQLIRAA	990	998	9	HLA-A*02:01	990	0.1405	0.3362	1.90E-02	-5.79E-01	0.3101	A2	0.1405	0.3362	0.019	-0.579	0.3101	TRUE	TRUE	TRUE	TRUE	TRUE
1	TQQLIRAAE	991	999	9	HLA-A*02:01	991	0.0177	0.0424	9.60E-03	-1.50E+00	-0.031	A2	0.0177	0.0424	0.0096	-1.496	-0.031	TRUE	TRUE	TRUE	TRUE	TRUE
1	QQLIRAAEI	992	1000	9	HLA-A*02:01	992	0.0327	0.0784	6.98E-01	6.51E-01	0.2155	A2	0.0327	0.0784	0.6975	0.651	0.2155	TRUE	TRUE	TRUE	TRUE	TRUE
1	QLIRAAEIR	993	1001	9	HLA-A*02:01	993	0.0568	0.1358	1.34E-01	1.54E+00	0.2327	A2	0.0568	0.1358	0.1337	1.537	0.2327	TRUE	TRUE	TRUE	TRUE	TRUE
1	LIRAAEIRA	994	1002	9	HLA-A*02:01	994	0.0799	0.1913	4.00E-01	-4.03E-01	0.2312	A2	0.0799	0.1913	0.4002	-0.403	0.2312	TRUE	TRUE	TRUE	TRUE	TRUE
1	IRAAEIRAS	995	1003	9	HLA-A*02:01	995	0.0206	0.0492	9.60E-03	-1.95E+00	-0.0469	A2	0.0206	0.0492	0.0096	-1.951	-0.0469	TRUE	TRUE	TRUE	TRUE	TRUE
1	RAAEIRASA	996	1004	9	HLA-A*02:01	996	0.0659	0.1578	8.25E-01	-2.13E-01	0.271	A2	0.0659	0.1578	0.8254	-0.213	0.271	TRUE	TRUE	TRUE	TRUE	TRUE
1	AAEIRASAN	997	1005	9	HLA-A*02:01	997	0.0173	0.0414	9.80E-03	-1.13E+00	-0.0136	A2	0.0173	0.0414	0.0098	-1.131	-0.0136	TRUE	TRUE	TRUE	TRUE	TRUE
1	AEIRASANL	998	1006	9	HLA-A*02:01	998	0.0519	0.1243	9.95E-01	1.19E+00	0.3329	A2	0.0519	0.1243	0.9954	1.186	0.3329	TRUE	TRUE	TRUE	TRUE	TRUE
1	EIRASANLA	999	1007	9	HLA-A*02:01	999	0.0455	0.109	1.18E-01	-4.08E-01	0.1062	A2	0.0455	0.109	0.1176	-0.408	0.1062	TRUE	TRUE	TRUE	TRUE	TRUE
1	IRASANLAA	1000	1008	9	HLA-A*02:01	1000	0.0267	0.0638	1.37E-01	-4.56E-01	0.0616	A2	0.0267	0.0638	0.137	-0.456	0.0616	TRUE	TRUE	TRUE	TRUE	TRUE
1	RASANLAAT	1001	1009	9	HLA-A*02:01	1001	0.0348	0.0832	1.22E-02	-4.04E-01	0.0649	A2	0.0348	0.0832	0.0122	-0.404	0.0649	TRUE	TRUE	TRUE	TRUE	TRUE
1	ASANLAATK	1002	1010	9	HLA-A*02:01	1002	0.0233	0.0558	7.70E-01	7.98E-01	0.2112	A2	0.0233	0.0558	0.7704	0.798	0.2112	TRUE	TRUE	TRUE	TRUE	TRUE
1	SANLAATKM	1003	1011	9	HLA-A*02:01	1003	0.0233	0.0557	9.97E-01	4.70E-01	0.2287	A2	0.0233	0.0557	0.9968	0.47	0.2287	TRUE	TRUE	TRUE	TRUE	TRUE
1	ANLAATKMS	1004	1012	9	HLA-A*02:01	1004	0.0197	0.0472	1.38E-02	-2.18E+00	-0.0598	A2	0.0197	0.0472	0.0138	-2.182	-0.0598	TRUE	TRUE	TRUE	TRUE	TRUE
1	NLAATKMSE	1005	1013	9	HLA-A*02:01	1005	0.0568	0.1358	1.90E-02	-1.34E+00	0.0718	A2	0.0568	0.1358	0.019	-1.338	0.0718	TRUE	TRUE	TRUE	TRUE	TRUE
1	LAATKMSEC	1006	1014	9	HLA-A*02:01	1006	0.0416	0.0996	1.05E-02	2.29E-01	0.1126	A2	0.0416	0.0996	0.0105	0.229	0.1126	TRUE	TRUE	TRUE	TRUE	TRUE
1	AATKMSECV	1007	1015	9	HLA-A*02:01	1007	0.1079	0.2581	5.53E-01	5.23E-01	0.3672	A2	0.1079	0.2581	0.553	0.523	0.3672	TRUE	TRUE	TRUE	TRUE	TRUE
1	ATKMSECVL	1008	1016	9	HLA-A*02:01	1008	0.0481	0.115	7.01E-01	1.23E+00	0.2816	A2	0.0481	0.115	0.7006	1.229	0.2816	TRUE	TRUE	TRUE	TRUE	TRUE
1	TKMSECVLG	1009	1017	9	HLA-A*02:01	1009	0.0181	0.0433	1.40E-02	-1.15E+00	-0.012	A2	0.0181	0.0433	0.014	-1.148	-0.012	TRUE	TRUE	TRUE	TRUE	TRUE
1	KMSECVLGQ	1010	1018	9	HLA-A*02:01	1010	0.1817	0.4347	3.24E-02	1.29E-01	0.446	A2	0.1817	0.4347	0.0324	0.129	0.446	TRUE	TRUE	TRUE	TRUE	TRUE
1	MSECVLGQS	1011	1019	9	HLA-A*02:01	1011	0.019	0.0455	2.59E-02	-2.20E+00	-0.0607	A2	0.019	0.0455	0.0259	-2.201	-0.0607	TRUE	TRUE	TRUE	TRUE	TRUE
1	SECVLGQSK	1012	1020	9	HLA-A*02:01	1012	0.0162	0.0389	5.45E-01	4.27E-01	0.1419	A2	0.0162	0.0389	0.5447	0.427	0.1419	TRUE	TRUE	TRUE	TRUE	TRUE
1	ECVLGQSKR	1013	1021	9	HLA-A*02:01	1013	0.0175	0.0419	1.19E-01	1.43E+00	0.1314	A2	0.0175	0.0419	0.119	1.433	0.1314	TRUE	TRUE	TRUE	TRUE	TRUE
1	CVLGQSKRV	1014	1022	9	HLA-A*02:01	1014	0.1775	0.4247	9.97E-01	2.63E-01	0.5875	A2	0.1775	0.4247	0.9971	0.263	0.5875	TRUE	TRUE	TRUE	TRUE	TRUE
1	VLGQSKRVD	1015	1023	9	HLA-A*02:01	1015	0.0486	0.1164	1.37E-02	-1.90E+00	0.0233	A2	0.0486	0.1164	0.0137	-1.904	0.0233	TRUE	TRUE	TRUE	TRUE	TRUE
1	LGQSKRVDF	1016	1024	9	HLA-A*02:01	1016	0.0194	0.0465	3.27E-01	2.29E+00	0.21	A2	0.0194	0.0465	0.3274	2.287	0.21	TRUE	TRUE	TRUE	TRUE	TRUE
1	GQSKRVDFC	1017	1025	9	HLA-A*02:01	1017	0.0363	0.0868	9.70E-03	-4.50E-02	0.0861	A2	0.0363	0.0868	0.0097	-0.045	0.0861	TRUE	TRUE	TRUE	TRUE	TRUE
1	QSKRVDFCG	1018	1026	9	HLA-A*02:01	1018	0.0195	0.0467	1.31E-02	-1.37E+00	-0.02	A2	0.0195	0.0467	0.0131	-1.374	-0.02	TRUE	TRUE	TRUE	TRUE	TRUE
1	SKRVDFCGK	1019	1027	9	HLA-A*02:01	1019	0.0168	0.0402	7.09E-01	5.67E-01	0.1749	A2	0.0168	0.0402	0.709	0.567	0.1749	TRUE	TRUE	TRUE	TRUE	TRUE
1	KRVDFCGKG	1020	1028	9	HLA-A*02:01	1020	0.0194	0.0464	5.71E-01	-9.50E-01	0.0846	A2	0.0194	0.0464	0.5709	-0.95	0.0846	TRUE	TRUE	TRUE	TRUE	TRUE
1	RVDFCGKGY	1021	1029	9	HLA-A*02:01	1021	0.0222	0.0532	8.07E-01	3.23E+00	0.3359	A2	0.0222	0.0532	0.8071	3.232	0.3359	TRUE	TRUE	TRUE	TRUE	TRUE
1	VDFCGKGYH	1022	1030	9	HLA-A*02:01	1022	0.0165	0.0395	3.52E-02	-5.37E-01	0.018	A2	0.0165	0.0395	0.0352	-0.537	0.018	TRUE	TRUE	TRUE	TRUE	TRUE
1	DFCGKGYHL	1023	1031	9	HLA-A*02:01	1023	0.0571	0.1366	9.97E-01	8.62E-01	0.3292	A2	0.0571	0.1366	0.9967	0.862	0.3292	TRUE	TRUE	TRUE	TRUE	TRUE
1	FCGKGYHLM	1024	1032	9	HLA-A*02:01	1024	0.1096	0.2622	8.21E-01	-7.20E-02	0.3817	A2	0.1096	0.2622	0.821	-0.072	0.3817	TRUE	TRUE	TRUE	TRUE	TRUE
1	CGKGYHLMS	1025	1033	9	HLA-A*02:01	1025	0.019	0.0454	1.27E-02	-2.66E+00	-0.0857	A2	0.019	0.0454	0.0127	-2.66	-0.0857	TRUE	TRUE	TRUE	TRUE	TRUE
1	GKGYHLMSF	1026	1034	9	HLA-A*02:01	1026	0.0196	0.0469	5.21E-01	2.24E+00	0.2368	A2	0.0196	0.0469	0.5208	2.235	0.2368	TRUE	TRUE	TRUE	TRUE	TRUE
1	KGYHLMSFP	1027	1035	9	HLA-A*02:01	1027	0.0225	0.0538	1.20E-02	1.02E-01	0.0607	A2	0.0225	0.0538	0.012	0.102	0.0607	TRUE	TRUE	TRUE	TRUE	TRUE
1	GYHLMSFPQ	1028	1036	9	HLA-A*02:01	1028	0.0336	0.0803	3.54E-01	1.11E-01	0.139	A2	0.0336	0.0803	0.3541	0.111	0.139	TRUE	TRUE	TRUE	TRUE	TRUE
1	YHLMSFPQA	1029	1037	9	HLA-A*02:01	1029	0.0319	0.0763	4.62E-02	-5.60E-01	0.0553	A2	0.0319	0.0763	0.0462	-0.56	0.0553	TRUE	TRUE	TRUE	TRUE	TRUE
1	HLMSFPQAA	1030	1038	9	HLA-A*02:01	1030	0.1891	0.4525	3.99E-02	-5.94E-01	0.4288	A2	0.1891	0.4525	0.0399	-0.594	0.4288	TRUE	TRUE	TRUE	TRUE	TRUE
1	LMSFPQAAP	1031	1039	9	HLA-A*02:01	1031	0.0676	0.1618	1.84E-02	2.03E-01	0.1747	A2	0.0676	0.1618	0.0184	0.203	0.1747	TRUE	TRUE	TRUE	TRUE	TRUE
1	MSFPQAAPH	1032	1040	9	HLA-A*02:01	1032	0.0271	0.0649	3.31E-02	-2.88E-01	0.0554	A2	0.0271	0.0649	0.0331	-0.288	0.0554	TRUE	TRUE	TRUE	TRUE	TRUE
1	SFPQAAPHG	1033	1041	9	HLA-A*02:01	1033	0.031	0.0741	1.04E-02	-1.15E+00	0.0179	A2	0.031	0.0741	0.0104	-1.154	0.0179	TRUE	TRUE	TRUE	TRUE	TRUE
1	FPQAAPHGV	1034	1042	9	HLA-A*02:01	1034	0.1107	0.2648	7.93E-01	-6.30E-02	0.3806	A2	0.1107	0.2648	0.793	-0.063	0.3806	TRUE	TRUE	TRUE	TRUE	TRUE
1	PQAAPHGVV	1035	1043	9	HLA-A*02:01	1035	0.033	0.0791	7.55E-01	-1.78E-01	0.1834	A2	0.033	0.0791	0.7548	-0.178	0.1834	TRUE	TRUE	TRUE	TRUE	TRUE
1	QAAPHGVVF	1036	1044	9	HLA-A*02:01	1036	0.0295	0.0706	9.75E-02	2.47E+00	0.2088	A2	0.0295	0.0706	0.0975	2.472	0.2088	TRUE	TRUE	TRUE	TRUE	TRUE
1	AAPHGVVFL	1037	1045	9	HLA-A*02:01	1037	0.095	0.2274	7.55E-01	1.09E+00	0.395	A2	0.095	0.2274	0.7547	1.087	0.395	TRUE	TRUE	TRUE	TRUE	TRUE
1	APHGVVFLH	1038	1046	9	HLA-A*02:01	1038	0.0309	0.074	7.78E-01	-5.12E-01	0.165	A2	0.0309	0.074	0.7778	-0.512	0.165	TRUE	TRUE	TRUE	TRUE	TRUE
1	PHGVVFLHV	1039	1047	9	HLA-A*02:01	1039	0.022	0.0526	9.92E-01	-1.67E-01	0.1931	A2	0.022	0.0526	0.9923	-0.167	0.1931	TRUE	TRUE	TRUE	TRUE	TRUE
1	HGVVFLHVT	1040	1048	9	HLA-A*02:01	1040	0.0196	0.0469	2.28E-02	-1.13E+00	-0.0062	A2	0.0196	0.0469	0.0228	-1.13	-0.0062	TRUE	TRUE	TRUE	TRUE	TRUE
1	GVVFLHVTY	1041	1049	9	HLA-A*02:01	1041	0.0243	0.0582	9.99E-01	2.94E+00	0.3549	A2	0.0243	0.0582	0.9987	2.939	0.3549	TRUE	TRUE	TRUE	TRUE	TRUE
1	VVFLHVTYV	1042	1050	9	HLA-A*02:01	1042	0.1724	0.4126	9.76E-01	5.97E-01	0.5888	A2	0.1724	0.4126	0.9757	0.597	0.5888	TRUE	TRUE	TRUE	TRUE	TRUE
1	VFLHVTYVP	1043	1051	9	HLA-A*02:01	1043	0.057	0.1363	5.27E-02	3.99E-01	0.1642	A2	0.057	0.1363	0.0527	0.399	0.1642	TRUE	TRUE	TRUE	TRUE	TRUE
1	FLHVTYVPS	1044	1052	9	HLA-A*02:01	1044	0.2096	0.5016	7.42E-02	-2.24E+00	0.4006	A2	0.2096	0.5016	0.0742	-2.244	0.4006	TRUE	TRUE	TRUE	TRUE	TRUE
1	LHVTYVPSQ	1045	1053	9	HLA-A*02:01	1045	0.0188	0.0449	3.73E-01	-1.21E-01	0.0948	A2	0.0188	0.0449	0.373	-0.121	0.0948	TRUE	TRUE	TRUE	TRUE	TRUE
1	HVTYVPSQE	1046	1054	9	HLA-A*02:01	1046	0.0224	0.0536	1.17E-02	-1.51E+00	-0.0199	A2	0.0224	0.0536	0.0117	-1.506	-0.0199	TRUE	TRUE	TRUE	TRUE	TRUE
1	VTYVPSQER	1047	1055	9	HLA-A*02:01	1047	0.0414	0.0992	9.82E-01	1.69E+00	0.3309	A2	0.0414	0.0992	0.9816	1.689	0.3309	TRUE	TRUE	TRUE	TRUE	TRUE
1	TYVPSQERN	1048	1056	9	HLA-A*02:01	1048	0.021	0.0503	1.42E-02	-9.47E-01	0.0051	A2	0.021	0.0503	0.0142	-0.947	0.0051	TRUE	TRUE	TRUE	TRUE	TRUE
1	YVPSQERNF	1049	1057	9	HLA-A*02:01	1049	0.0495	0.1184	6.20E-02	2.65E+00	0.2601	A2	0.0495	0.1184	0.062	2.648	0.2601	TRUE	TRUE	TRUE	TRUE	TRUE
1	VPSQERNFT	1050	1058	9	HLA-A*02:01	1050	0.0248	0.0594	1.21E-02	-1.00E+00	0.011	A2	0.0248	0.0594	0.0121	-1.004	0.011	TRUE	TRUE	TRUE	TRUE	TRUE
1	PSQERNFTT	1051	1059	9	HLA-A*02:01	1051	0.0505	0.1207	3.41E-01	-1.13E+00	0.1154	A2	0.0505	0.1207	0.341	-1.13	0.1154	TRUE	TRUE	TRUE	TRUE	TRUE
1	SQERNFTTA	1052	1060	9	HLA-A*02:01	1052	0.0236	0.0565	7.35E-01	-6.96E-01	0.132	A2	0.0236	0.0565	0.7354	-0.696	0.132	TRUE	TRUE	TRUE	TRUE	TRUE
1	QERNFTTAP	1053	1061	9	HLA-A*02:01	1053	0.0161	0.0386	9.47E-01	8.50E-02	0.1849	A2	0.0161	0.0386	0.9466	0.085	0.1849	TRUE	TRUE	TRUE	TRUE	TRUE
1	ERNFTTAPA	1054	1062	9	HLA-A*02:01	1054	0.0185	0.0444	7.81E-01	-6.76E-01	0.1277	A2	0.0185	0.0444	0.7806	-0.676	0.1277	TRUE	TRUE	TRUE	TRUE	TRUE
1	RNFTTAPAI	1055	1063	9	HLA-A*02:01	1055	0.0313	0.075	6.65E-01	5.71E-01	0.2032	A2	0.0313	0.075	0.6647	0.571	0.2032	TRUE	TRUE	TRUE	TRUE	TRUE
1	NFTTAPAIC	1056	1064	9	HLA-A*02:01	1056	0.0277	0.0664	1.21E-02	3.15E-01	0.084	A2	0.0277	0.0664	0.0121	0.315	0.084	TRUE	TRUE	TRUE	TRUE	TRUE
1	FTTAPAICH	1057	1065	9	HLA-A*02:01	1057	0.0415	0.0993	3.80E-02	-5.47E-01	0.0777	A2	0.0415	0.0993	0.038	-0.547	0.0777	TRUE	TRUE	TRUE	TRUE	TRUE
1	TTAPAICHE	1058	1066	9	HLA-A*02:01	1058	0.0566	0.1354	1.18E-02	-1.70E+00	0.0524	A2	0.0566	0.1354	0.0118	-1.696	0.0524	TRUE	TRUE	TRUE	TRUE	TRUE
1	TAPAICHEG	1059	1067	9	HLA-A*02:01	1059	0.0299	0.0716	1.61E-01	-1.37E+00	0.0273	A2	0.0299	0.0716	0.1609	-1.369	0.0273	TRUE	TRUE	TRUE	TRUE	TRUE
1	APAICHEGK	1060	1068	9	HLA-A*02:01	1060	0.0167	0.04	9.74E-01	3.31E-01	0.2026	A2	0.0167	0.04	0.9738	0.331	0.2026	TRUE	TRUE	TRUE	TRUE	TRUE
1	PAICHEGKA	1061	1069	9	HLA-A*02:01	1061	0.0205	0.049	6.52E-01	-7.40E-01	0.1097	A2	0.0205	0.049	0.6515	-0.74	0.1097	TRUE	TRUE	TRUE	TRUE	TRUE
1	AICHEGKAY	1062	1070	9	HLA-A*02:01	1062	0.0401	0.0959	2.28E-01	3.06E+00	0.283	A2	0.0401	0.0959	0.2275	3.061	0.283	TRUE	TRUE	TRUE	TRUE	TRUE
1	ICHEGKAYF	1063	1071	9	HLA-A*02:01	1063	0.092	0.2201	2.28E-01	2.76E+00	0.3924	A2	0.092	0.2201	0.2278	2.762	0.3924	TRUE	TRUE	TRUE	TRUE	TRUE
1	CHEGKAYFP	1064	1072	9	HLA-A*02:01	1064	0.0204	0.0488	9.70E-03	-6.40E-02	0.0471	A2	0.0204	0.0488	0.0097	-0.064	0.0471	TRUE	TRUE	TRUE	TRUE	TRUE
1	HEGKAYFPR	1065	1073	9	HLA-A*02:01	1065	0.0199	0.0476	4.83E-02	1.09E+00	0.1093	A2	0.0199	0.0476	0.0483	1.088	0.1093	TRUE	TRUE	TRUE	TRUE	TRUE
1	EGKAYFPRE	1066	1074	9	HLA-A*02:01	1066	0.0169	0.0405	1.18E-02	-2.06E+00	-0.0608	A2	0.0169	0.0405	0.0118	-2.061	-0.0608	TRUE	TRUE	TRUE	TRUE	TRUE
1	GKAYFPREG	1067	1075	9	HLA-A*02:01	1067	0.0156	0.0374	1.01E-02	-1.68E+00	-0.0448	A2	0.0156	0.0374	0.0101	-1.675	-0.0448	TRUE	TRUE	TRUE	TRUE	TRUE
1	KAYFPREGV	1068	1076	9	HLA-A*02:01	1068	0.0734	0.1756	2.48E-01	6.31E-01	0.2444	A2	0.0734	0.1756	0.2479	0.631	0.2444	TRUE	TRUE	TRUE	TRUE	TRUE
1	AYFPREGVF	1069	1077	9	HLA-A*02:01	1069	0.0353	0.0845	9.33E-01	3.16E+00	0.3822	A2	0.0353	0.0845	0.9327	3.156	0.3822	TRUE	TRUE	TRUE	TRUE	TRUE
1	YFPREGVFV	1070	1078	9	HLA-A*02:01	1070	0.272	0.6509	6.12E-02	6.01E-01	0.6901	A2	0.272	0.6509	0.0612	0.601	0.6901	TRUE	TRUE	TRUE	TRUE	TRUE
1	FPREGVFVF	1071	1079	9	HLA-A*02:01	1071	0.0601	0.1438	9.99E-01	2.13E+00	0.4001	A2	0.0601	0.1438	0.9986	2.132	0.4001	TRUE	TRUE	TRUE	TRUE	TRUE
1	PREGVFVFN	1072	1080	9	HLA-A*02:01	1072	0.0171	0.0409	1.46E-02	-1.84E+00	-0.0488	A2	0.0171	0.0409	0.0146	-1.838	-0.0488	TRUE	TRUE	TRUE	TRUE	TRUE
1	REGVFVFNG	1073	1081	9	HLA-A*02:01	1073	0.0162	0.0389	1.44E-02	-1.60E+00	-0.0387	A2	0.0162	0.0389	0.0144	-1.595	-0.0387	TRUE	TRUE	TRUE	TRUE	TRUE
1	EGVFVFNGT	1074	1082	9	HLA-A*02:01	1074	0.017	0.0407	1.20E-02	-9.66E-01	-0.0058	A2	0.017	0.0407	0.012	-0.966	-0.0058	TRUE	TRUE	TRUE	TRUE	TRUE
1	GVFVFNGTS	1075	1083	9	HLA-A*02:01	1075	0.0355	0.0849	1.25E-02	-2.34E+00	-0.0303	A2	0.0355	0.0849	0.0125	-2.341	-0.0303	TRUE	TRUE	TRUE	TRUE	TRUE
1	VFVFNGTSW	1076	1084	9	HLA-A*02:01	1076	0.0212	0.0507	8.81E-01	1.16E+00	0.2409	A2	0.0212	0.0507	0.8809	1.162	0.2409	TRUE	TRUE	TRUE	TRUE	TRUE
1	FVFNGTSWF	1077	1085	9	HLA-A*02:01	1077	0.267	0.639	6.88E-01	2.74E+00	0.8791	A2	0.267	0.639	0.6883	2.737	0.8791	TRUE	TRUE	TRUE	TRUE	TRUE
1	VFNGTSWFI	1078	1086	9	HLA-A*02:01	1078	0.3885	0.9298	4.24E-01	5.65E-01	1.0216	A2	0.3885	0.9298	0.4235	0.565	1.0216	TRUE	TRUE	TRUE	TRUE	TRUE
1	FNGTSWFIT	1079	1087	9	HLA-A*02:01	1079	0.0267	0.0638	1.13E-02	-1.07E+00	0.0121	A2	0.0267	0.0638	0.0113	-1.069	0.0121	TRUE	TRUE	TRUE	TRUE	TRUE
1	NGTSWFITQ	1080	1088	9	HLA-A*02:01	1080	0.0187	0.0448	8.27E-02	-4.76E-01	3.35E-02	A2	0.0187	0.0448	0.0827	-0.476	0.0335	TRUE	TRUE	TRUE	TRUE	TRUE
1	GTSWFITQR	1081	1089	9	HLA-A*02:01	1081	0.0342	0.0818	9.94E-01	1.42E+00	0.302	A2	0.0342	0.0818	0.9941	1.421	0.302	TRUE	TRUE	TRUE	TRUE	TRUE
1	TSWFITQRN	1082	1090	9	HLA-A*02:01	1082	0.0185	0.0444	5.20E-01	-1.15E+00	0.0647	A2	0.0185	0.0444	0.5203	-1.154	0.0647	TRUE	TRUE	TRUE	TRUE	TRUE
1	SWFITQRNF	1083	1091	9	HLA-A*02:01	1083	0.018	0.0431	7.89E-02	2.96E+00	0.2027	A2	0.018	0.0431	0.0789	2.956	0.2027	TRUE	TRUE	TRUE	TRUE	TRUE
1	WFITQRNFF	1084	1092	9	HLA-A*02:01	1084	0.0532	0.1273	1.57E-02	2.80E+00	0.2696	A2	0.0532	0.1273	0.0157	2.798	0.2696	TRUE	TRUE	TRUE	TRUE	TRUE
1	FITQRNFFS	1085	1093	9	HLA-A*02:01	1085	0.3357	0.8034	9.50E-03	-2.30E+00	0.6896	A2	0.3357	0.8034	0.0095	-2.304	0.6896	TRUE	TRUE	TRUE	TRUE	TRUE
1	ITQRNFFSP	1086	1094	9	HLA-A*02:01	1086	0.054	0.1293	1.22E-02	-3.10E-02	0.1296	A2	0.054	0.1293	0.0122	-0.031	0.1296	TRUE	TRUE	TRUE	TRUE	TRUE
1	TQRNFFSPQ	1087	1095	9	HLA-A*02:01	1087	0.0187	0.0447	2.09E-02	7.00E-03	0.0482	A2	0.0187	0.0447	0.0209	0.007	0.0482	TRUE	TRUE	TRUE	TRUE	TRUE
1	QRNFFSPQI	1088	1096	9	HLA-A*02:01	1088	0.0219	0.0525	9.92E-01	6.70E-01	0.2347	A2	0.0219	0.0525	0.9915	0.67	0.2347	TRUE	TRUE	TRUE	TRUE	TRUE
1	RNFFSPQII	1089	1097	9	HLA-A*02:01	1089	0.0234	0.0559	3.67E-01	6.89E-01	0.1455	A2	0.0234	0.0559	0.3674	0.689	0.1455	TRUE	TRUE	TRUE	TRUE	TRUE
1	NFFSPQIIT	1090	1098	9	HLA-A*02:01	1090	0.0297	0.0711	1.12E-02	-2.57E-01	0.0599	A2	0.0297	0.0711	0.0112	-0.257	0.0599	TRUE	TRUE	TRUE	TRUE	TRUE
1	FFSPQIITT	1091	1099	9	HLA-A*02:01	1091	0.1807	0.4325	3.61E-01	-4.96E-01	0.4619	A2	0.1807	0.4325	0.3612	-0.496	0.4619	TRUE	TRUE	TRUE	TRUE	TRUE
1	FSPQIITTD	1092	1100	9	HLA-A*02:01	1092	0.0303	0.0725	6.33E-02	-1.99E+00	-0.0175	A2	0.0303	0.0725	0.0633	-1.99	-0.0175	TRUE	TRUE	TRUE	TRUE	TRUE
1	SPQIITTDN	1093	1101	9	HLA-A*02:01	1093	0.0157	0.0376	2.63E-01	-1.70E+00	-0.0081	A2	0.0157	0.0376	0.2631	-1.704	-0.0081	TRUE	TRUE	TRUE	TRUE	TRUE
1	PQIITTDNT	1094	1102	9	HLA-A*02:01	1094	0.0222	0.0531	1.34E-02	-9.87E-01	0.0057	A2	0.0222	0.0531	0.0134	-0.987	0.0057	TRUE	TRUE	TRUE	TRUE	TRUE
1	QIITTDNTF	1095	1103	9	HLA-A*02:01	1095	0.0468	0.112	8.21E-01	2.60E+00	0.3651	A2	0.0468	0.112	0.8208	2.6	0.3651	TRUE	TRUE	TRUE	TRUE	TRUE
1	IITTDNTFV	1096	1104	9	HLA-A*02:01	1096	0.228	0.5457	9.82E-01	3.81E-01	0.7121	A2	0.228	0.5457	0.9824	0.381	0.7121	TRUE	TRUE	TRUE	TRUE	TRUE
1	ITTDNTFVS	1097	1105	9	HLA-A*02:01	1097	0.1781	0.4261	8.27E-02	-2.27E+00	0.325	A2	0.1781	0.4261	0.0827	-2.271	0.325	TRUE	TRUE	TRUE	TRUE	TRUE
1	TTDNTFVSG	1098	1106	9	HLA-A*02:01	1098	0.0229	0.0548	2.96E-01	-1.61E+00	0.0189	A2	0.0229	0.0548	0.2955	-1.605	0.0189	TRUE	TRUE	TRUE	TRUE	TRUE
1	TDNTFVSGN	1099	1107	9	HLA-A*02:01	1099	0.0146	0.0349	1.08E-02	-1.73E+00	-0.0499	A2	0.0146	0.0349	0.0108	-1.728	-0.0499	TRUE	TRUE	TRUE	TRUE	TRUE
1	DNTFVSGNC	1100	1108	9	HLA-A*02:01	1100	0.0183	0.0439	9.80E-03	-4.80E-01	0.0213	A2	0.0183	0.0439	0.0098	-0.48	0.0213	TRUE	TRUE	TRUE	TRUE	TRUE
1	NTFVSGNCD	1101	1109	9	HLA-A*02:01	1101	0.0192	0.046	9.90E-03	-1.83E+00	-0.0439	A2	0.0192	0.046	0.0099	-1.827	-0.0439	TRUE	TRUE	TRUE	TRUE	TRUE
1	TFVSGNCDV	1102	1110	9	HLA-A*02:01	1102	0.1178	0.2818	1.19E-01	6.80E-01	0.3336	A2	0.1178	0.2818	0.1186	0.68	0.3336	TRUE	TRUE	TRUE	TRUE	TRUE
1	FVSGNCDVV	1103	1111	9	HLA-A*02:01	1103	0.3946	0.9443	9.74E-02	3.64E-01	0.9771	A2	0.3946	0.9443	0.0974	0.364	0.9771	TRUE	TRUE	TRUE	TRUE	TRUE
1	VSGNCDVVI	1104	1112	9	HLA-A*02:01	1104	0.0256	0.0613	1.50E-02	3.69E-01	0.082	A2	0.0256	0.0613	0.015	0.369	0.082	TRUE	TRUE	TRUE	TRUE	TRUE
1	SGNCDVVIG	1105	1113	9	HLA-A*02:01	1105	0.0141	0.0339	9.90E-03	-1.66E+00	-0.0475	A2	0.0141	0.0339	0.0099	-1.656	-0.0475	TRUE	TRUE	TRUE	TRUE	TRUE
1	GNCDVVIGI	1106	1114	9	HLA-A*02:01	1106	0.0459	0.1098	6.39E-02	2.66E-01	0.1327	A2	0.0459	0.1098	0.0639	0.266	0.1327	TRUE	TRUE	TRUE	TRUE	TRUE
1	NCDVVIGII	1107	1115	9	HLA-A*02:01	1107	0.0258	0.0617	6.54E-02	3.86E-01	0.0908	A2	0.0258	0.0617	0.0654	0.386	0.0908	TRUE	TRUE	TRUE	TRUE	TRUE
1	CDVVIGIIN	1108	1116	9	HLA-A*02:01	1108	0.0145	0.0347	1.06E-02	-1.45E+00	-0.036	A2	0.0145	0.0347	0.0106	-1.445	-0.036	TRUE	TRUE	TRUE	TRUE	TRUE
1	DVVIGIINN	1109	1117	9	HLA-A*02:01	1109	0.0224	0.0535	4.51E-02	-1.42E+00	-0.0108	A2	0.0224	0.0535	0.0451	-1.422	-0.0108	TRUE	TRUE	TRUE	TRUE	TRUE
1	VVIGIINNT	1110	1118	9	HLA-A*02:01	1110	0.185	0.4428	3.67E-02	-5.75E-01	0.4195	A2	0.185	0.4428	0.0367	-0.575	0.4195	TRUE	TRUE	TRUE	TRUE	TRUE
1	VIGIINNTV	1111	1119	9	HLA-A*02:01	1111	0.1228	0.294	9.93E-01	3.89E-01	0.4624	A2	0.1228	0.294	0.9934	0.389	0.4624	TRUE	TRUE	TRUE	TRUE	TRUE
1	IGIINNTVY	1112	1120	9	HLA-A*02:01	1112	0.0169	0.0405	9.96E-01	2.80E+00	0.3298	A2	0.0169	0.0405	0.9963	2.797	0.3298	TRUE	TRUE	TRUE	TRUE	TRUE
1	GIINNTVYD	1113	1121	9	HLA-A*02:01	1113	0.0553	0.1323	2.28E-01	-1.79E+00	0.0771	A2	0.0553	0.1323	0.2278	-1.788	0.0771	TRUE	TRUE	TRUE	TRUE	TRUE
1	IINNTVYDP	1114	1122	9	HLA-A*02:01	1114	0.1094	0.2618	2.08E-02	1.32E-01	0.2716	A2	0.1094	0.2618	0.0208	0.132	0.2716	TRUE	TRUE	TRUE	TRUE	TRUE
1	INNTVYDPL	1115	1123	9	HLA-A*02:01	1115	0.0238	0.057	9.87E-01	7.40E-01	0.242	A2	0.0238	0.057	0.9865	0.74	0.242	TRUE	TRUE	TRUE	TRUE	TRUE
1	NNTVYDPLQ	1116	1124	9	HLA-A*02:01	1116	0.0151	0.0361	1.02E-02	-2.48E-01	0.0252	A2	0.0151	0.0361	0.0102	-0.248	0.0252	TRUE	TRUE	TRUE	TRUE	TRUE
1	NTVYDPLQP	1117	1125	9	HLA-A*02:01	1117	0.0194	0.0463	4.29E-02	3.39E-01	0.0697	A2	0.0194	0.0463	0.0429	0.339	0.0697	TRUE	TRUE	TRUE	TRUE	TRUE
1	TVYDPLQPE	1118	1126	9	HLA-A*02:01	1118	0.1133	0.2711	1.48E-02	-1.12E+00	0.2173	A2	0.1133	0.2711	0.0148	-1.12	0.2173	TRUE	TRUE	TRUE	TRUE	TRUE
1	VYDPLQPEL	1119	1127	9	HLA-A*02:01	1119	0.0955	0.2286	9.98E-01	1.12E+00	0.4341	A2	0.0955	0.2286	0.9983	1.115	0.4341	TRUE	TRUE	TRUE	TRUE	TRUE
1	YDPLQPELD	1120	1128	9	HLA-A*02:01	1120	0.0146	0.0348	1.88E-02	-2.11E+00	-0.0679	A2	0.0146	0.0348	0.0188	-2.111	-0.0679	TRUE	TRUE	TRUE	TRUE	TRUE
1	DPLQPELDS	1121	1129	9	HLA-A*02:01	1121	0.0177	0.0423	3.21E-02	-2.90E+00	-0.0979	A2	0.0177	0.0423	0.0321	-2.9	-0.0979	TRUE	TRUE	TRUE	TRUE	TRUE
1	PLQPELDSF	1122	1130	9	HLA-A*02:01	1122	0.1656	0.3962	8.96E-01	1.90E+00	0.6257	A2	0.1656	0.3962	0.8956	1.903	0.6257	TRUE	TRUE	TRUE	TRUE	TRUE
1	LQPELDSFK	1123	1131	9	HLA-A*02:01	1123	0.0287	0.0688	4.97E-01	3.84E-01	0.1626	A2	0.0287	0.0688	0.4974	0.384	0.1626	TRUE	TRUE	TRUE	TRUE	TRUE
1	QPELDSFKE	1124	1132	9	HLA-A*02:01	1124	0.0177	0.0425	5.25E-01	-1.95E+00	0.0235	A2	0.0177	0.0425	0.5245	-1.953	0.0235	TRUE	TRUE	TRUE	TRUE	TRUE
1	PELDSFKEE	1125	1133	9	HLA-A*02:01	1125	0.0215	0.0514	1.19E-02	-2.21E+00	-0.0574	A2	0.0215	0.0514	0.0119	-2.212	-0.0574	TRUE	TRUE	TRUE	TRUE	TRUE
1	ELDSFKEEL	1126	1134	9	HLA-A*02:01	1126	0.1198	0.2867	9.47E-01	4.49E-01	0.4512	A2	0.1198	0.2867	0.947	0.449	0.4512	TRUE	TRUE	TRUE	TRUE	TRUE
1	LDSFKEELD	1127	1135	9	HLA-A*02:01	1127	0.0143	0.0343	1.23E-02	-2.29E+00	-0.0783	A2	0.0143	0.0343	0.0123	-2.29	-0.0783	TRUE	TRUE	TRUE	TRUE	TRUE

seq #	peptide	start	end	peptide length	allele	peptide index	NetCTL Predict	NetCTL Rescale	NetCTL c termi	NetCTL TAP tra	NetCTL Predict	NetCTL Superty	legacy predicte	legacy rescale_	legacy c_termi	legacy tap_tran	legacy predicti	equal predicted	equal rescale_	equal c_termin	equal tap_tran	equal predictions_score
1	DSFKEELDK	1128	1136	9	HLA-A*02:01	1128	0.0179	0.0429	5.16E-01	3.49E-01	0.1377	A2	0.0179	0.0429	0.5155	0.349	0.1377	TRUE	TRUE	TRUE	TRUE	TRUE
1	SFKEELDKY	1129	1137	9	HLA-A*02:01	1129	0.0345	0.0826	9.92E-01	3.00E+00	0.3816	A2	0.0345	0.0826	0.9923	3.004	0.3816	TRUE	TRUE	TRUE	TRUE	TRUE
1	FKEELDKYF	1130	1138	9	HLA-A*02:01	1130	0.0434	0.1038	2.99E-01	2.40E+00	0.2686	A2	0.0434	0.1038	0.2986	2.399	0.2686	TRUE	TRUE	TRUE	TRUE	TRUE
1	KEELDKYFK	1131	1139	9	HLA-A*02:01	1131	0.017	0.0407	7.07E-01	1.87E-01	0.1561	A2	0.017	0.0407	0.707	0.187	0.1561	TRUE	TRUE	TRUE	TRUE	TRUE
1	EELDKYFKN	1132	1140	9	HLA-A*02:01	1132	0.0222	0.053	4.47E-01	-1.54E+00	0.0433	A2	0.0222	0.053	0.447	-1.536	0.0433	TRUE	TRUE	TRUE	TRUE	TRUE
1	ELDKYFKNH	1133	1141	9	HLA-A*02:01	1133	0.05	0.1196	5.87E-01	-1.06E+00	0.1545	A2	0.05	0.1196	0.5866	-1.063	0.1545	TRUE	TRUE	TRUE	TRUE	TRUE
1	LDKYFKNHT	1134	1142	9	HLA-A*02:01	1134	0.0162	0.0387	2.79E-02	-1.18E+00	-0.016	A2	0.0162	0.0387	0.0279	-1.178	-0.016	TRUE	TRUE	TRUE	TRUE	TRUE
1	DKYFKNHTS	1135	1143	9	HLA-A*02:01	1135	0.0196	0.047	3.80E-02	-2.28E+00	-0.0611	A2	0.0196	0.047	0.038	-2.277	-0.0611	TRUE	TRUE	TRUE	TRUE	TRUE
1	KYFKNHTSP	1136	1144	9	HLA-A*02:01	1136	0.0237	0.0568	7.72E-01	4.49E-01	0.195	A2	0.0237	0.0568	0.7719	0.449	0.195	TRUE	TRUE	TRUE	TRUE	TRUE
1	YFKNHTSPD	1137	1145	9	HLA-A*02:01	1137	0.0284	0.0681	2.29E-02	-1.57E+00	-0.007	A2	0.0284	0.0681	0.0229	-1.569	-0.007	TRUE	TRUE	TRUE	TRUE	TRUE
1	FKNHTSPDV	1138	1146	9	HLA-A*02:01	1138	0.1476	0.3533	9.91E-01	1.23E-01	0.5081	A2	0.1476	0.3533	0.9911	0.123	0.5081	TRUE	TRUE	TRUE	TRUE	TRUE
1	KNHTSPDVD	1139	1147	9	HLA-A*02:01	1139	0.0157	0.0375	1.15E-02	-1.94E+00	-0.0575	A2	0.0157	0.0375	0.0115	-1.936	-0.0575	TRUE	TRUE	TRUE	TRUE	TRUE
1	NHTSPDVDL	1140	1148	9	HLA-A*02:01	1140	0.0188	0.0449	6.20E-01	1.06E+00	0.1911	A2	0.0188	0.0449	0.6204	1.064	0.1911	TRUE	TRUE	TRUE	TRUE	TRUE
1	HTSPDVDLG	1141	1149	9	HLA-A*02:01	1141	0.0321	0.0768	2.29E-02	-1.37E+00	0.0117	A2	0.0321	0.0768	0.0229	-1.371	0.0117	TRUE	TRUE	TRUE	TRUE	TRUE
1	TSPDVDLGD	1142	1150	9	HLA-A*02:01	1142	0.0239	0.0572	1.20E-02	-1.83E+00	-0.0327	A2	0.0239	0.0572	0.012	-1.834	-0.0327	TRUE	TRUE	TRUE	TRUE	TRUE
1	SPDVDLGDI	1143	1151	9	HLA-A*02:01	1143	0.0219	0.0524	6.45E-01	1.17E-01	0.155	A2	0.0219	0.0524	0.6452	0.117	0.155	TRUE	TRUE	TRUE	TRUE	TRUE
1	PDVDLGDIS	1144	1152	9	HLA-A*02:01	1144	0.021	0.0502	9.70E-03	-2.90E+00	-0.0934	A2	0.021	0.0502	0.0097	-2.901	-0.0934	TRUE	TRUE	TRUE	TRUE	TRUE
1	DVDLGDISG	1145	1153	9	HLA-A*02:01	1145	0.0195	0.0466	1.36E-02	-1.89E+00	-0.046	A2	0.0195	0.0466	0.0136	-1.894	-0.046	TRUE	TRUE	TRUE	TRUE	TRUE
1	VDLGDISGI	1146	1154	9	HLA-A*02:01	1146	0.0577	0.1381	1.57E-01	1.68E-01	0.17	A2	0.0577	0.1381	0.1568	0.168	0.17	TRUE	TRUE	TRUE	TRUE	TRUE
1	DLGDISGIN	1147	1155	9	HLA-A*02:01	1147	0.0377	0.0901	1.26E-02	-1.73E+00	0.0056	A2	0.0377	0.0901	0.0126	-1.728	0.0056	TRUE	TRUE	TRUE	TRUE	TRUE
1	LGDISGINA	1148	1156	9	HLA-A*02:01	1148	0.0209	0.05	1.83E-02	-1.11E+00	-0.0029	A2	0.0209	0.05	0.0183	-1.113	-0.0029	TRUE	TRUE	TRUE	TRUE	TRUE
1	GDISGINAS	1149	1157	9	HLA-A*02:01	1149	0.023	0.055	1.14E-02	-2.62E+00	-0.074	A2	0.023	0.055	0.0114	-2.615	-0.074	TRUE	TRUE	TRUE	TRUE	TRUE
1	DISGINASV	1150	1158	9	HLA-A*02:01	1150	0.2253	0.5391	9.70E-01	9.30E-02	0.6893	A2	0.2253	0.5391	0.9702	0.093	0.6893	TRUE	TRUE	TRUE	TRUE	TRUE
1	ISGINASVV	1151	1159	9	HLA-A*02:01	1151	0.0331	0.0791	5.29E-01	7.60E-02	0.1623	A2	0.0331	0.0791	0.529	0.076	0.1623	TRUE	TRUE	TRUE	TRUE	TRUE
1	SGINASVVN	1152	1160	9	HLA-A*02:01	1152	0.0159	0.038	1.03E-02	-1.45E+00	-0.033	A2	0.0159	0.038	0.0103	-1.451	-0.033	TRUE	TRUE	TRUE	TRUE	TRUE
1	GINASVVNI	1153	1161	9	HLA-A*02:01	1153	0.1274	0.3049	4.78E-01	4.44E-01	0.3988	A2	0.1274	0.3049	0.4779	0.444	0.3988	TRUE	TRUE	TRUE	TRUE	TRUE
1	INASVVNIQ	1154	1162	9	HLA-A*02:01	1154	0.0183	0.0438	1.06E-02	-2.48E-01	0.033	A2	0.0183	0.0438	0.0106	-0.248	0.033	TRUE	TRUE	TRUE	TRUE	TRUE
1	NASVVNIQK	1155	1163	9	HLA-A*02:01	1155	0.0179	0.0428	9.99E-01	5.59E-01	0.2205	A2	0.0179	0.0428	0.9988	0.559	0.2205	TRUE	TRUE	TRUE	TRUE	TRUE
1	ASVVNIQKE	1156	1164	9	HLA-A*02:01	1156	0.0205	0.0491	1.12E-02	-1.16E+00	-0.0073	A2	0.0205	0.0491	0.0112	-1.162	-0.0073	TRUE	TRUE	TRUE	TRUE	TRUE
1	SVVNIQKEI	1157	1165	9	HLA-A*02:01	1157	0.0675	0.1615	7.99E-01	9.58E-01	0.3293	A2	0.0675	0.1615	0.7989	0.958	0.3293	TRUE	TRUE	TRUE	TRUE	TRUE
1	VVNIQKEID	1158	1166	9	HLA-A*02:01	1158	0.0192	0.046	1.03E-02	-1.66E+00	-0.0352	A2	0.0192	0.046	0.0103	-1.655	-0.0352	TRUE	TRUE	TRUE	TRUE	TRUE
1	VNIQKEIDR	1159	1167	9	HLA-A*02:01	1159	0.0181	0.0433	1.04E-02	1.51E+00	0.1204	A2	0.0181	0.0433	0.0104	1.51	0.1204	TRUE	TRUE	TRUE	TRUE	TRUE
1	NIQKEIDRL	1160	1168	9	HLA-A*02:01	1160	0.131	0.3136	4.15E-01	1.14E+00	0.4326	A2	0.131	0.3136	0.415	1.135	0.4326	TRUE	TRUE	TRUE	TRUE	TRUE
1	IQKEIDRLN	1161	1169	9	HLA-A*02:01	1161	0.0185	0.0443	1.12E-02	-1.08E+00	-0.0079	A2	0.0185	0.0443	0.0112	-1.078	-0.0079	TRUE	TRUE	TRUE	TRUE	TRUE
1	QKEIDRLNE	1162	1170	9	HLA-A*02:01	1162	0.0165	0.0395	9.70E-03	-1.64E+00	-0.0409	A2	0.0165	0.0395	0.0097	-1.638	-0.0409	TRUE	TRUE	TRUE	TRUE	TRUE
1	KEIDRLNEV	1163	1171	9	HLA-A*02:01	1163	0.091	0.2179	9.98E-01	3.06E-01	0.3829	A2	0.091	0.2179	0.998	0.306	0.3829	TRUE	TRUE	TRUE	TRUE	TRUE
1	EIDRLNEVA	1164	1172	9	HLA-A*02:01	1164	0.0666	0.1593	4.14E-01	-6.86E-01	0.1871	A2	0.0666	0.1593	0.414	-0.686	0.1871	TRUE	TRUE	TRUE	TRUE	TRUE
1	IDRLNEVAK	1165	1173	9	HLA-A*02:01	1165	0.0153	0.0366	4.45E-01	6.20E-02	0.1065	A2	0.0153	0.0366	0.4452	0.062	0.1065	TRUE	TRUE	TRUE	TRUE	TRUE
1	DRLNEVAKN	1166	1174	9	HLA-A*02:01	1166	0.0148	0.0355	1.62E-02	-1.40E+00	-0.032	A2	0.0148	0.0355	0.0162	-1.398	-0.032	TRUE	TRUE	TRUE	TRUE	TRUE
1	RLNEVAKNL	1167	1175	9	HLA-A*02:01	1167	0.2671	0.6391	9.98E-01	9.85E-01	0.838	A2	0.2671	0.6391	0.9979	0.985	0.838	TRUE	TRUE	TRUE	TRUE	TRUE
1	LNEVAKNLN	1168	1176	9	HLA-A*02:01	1168	0.0141	0.0338	1.01E-02	-1.48E+00	-0.0385	A2	0.0141	0.0338	0.0101	-1.476	-0.0385	TRUE	TRUE	TRUE	TRUE	TRUE
1	NEVAKNLNE	1169	1177	9	HLA-A*02:01	1169	0.0156	0.0372	1.16E-02	-1.54E+00	-0.0379	A2	0.0156	0.0372	0.0116	-1.538	-0.0379	TRUE	TRUE	TRUE	TRUE	TRUE
1	EVAKNLNES	1170	1178	9	HLA-A*02:01	1170	0.052	0.1246	1.62E-02	-2.15E+00	0.0193	A2	0.052	0.1246	0.0162	-2.153	0.0193	TRUE	TRUE	TRUE	TRUE	TRUE
1	VAKNLNESL	1171	1179	9	HLA-A*02:01	1171	0.0454	0.1088	9.05E-01	9.00E-01	0.2895	A2	0.0454	0.1088	0.9046	0.9	0.2895	TRUE	TRUE	TRUE	TRUE	TRUE
1	AKNLNESLI	1172	1180	9	HLA-A*02:01	1172	0.0209	0.0499	6.10E-01	6.81E-01	0.1755	A2	0.0209	0.0499	0.6103	0.681	0.1755	TRUE	TRUE	TRUE	TRUE	TRUE
1	KNLNESLID	1173	1181	9	HLA-A*02:01	1173	0.0164	0.0392	1.33E-02	-1.69E+00	-0.0432	A2	0.0164	0.0392	0.0133	-1.689	-0.0432	TRUE	TRUE	TRUE	TRUE	TRUE
1	NLNESLIDL	1174	1182	9	HLA-A*02:01	1174	0.2788	0.6673	9.97E-01	1.02E+00	0.8677	A2	0.2788	0.6673	0.9971	1.018	0.8677	TRUE	TRUE	TRUE	TRUE	TRUE
1	LNESLIDLQ	1175	1183	9	HLA-A*02:01	1175	0.0195	0.0467	1.33E-02	-2.57E-01	0.0358	A2	0.0195	0.0467	0.0133	-0.257	0.0358	TRUE	TRUE	TRUE	TRUE	TRUE
1	NESLIDLQE	1176	1184	9	HLA-A*02:01	1176	0.0152	0.0363	1.62E-02	-1.62E+00	-0.0422	A2	0.0152	0.0363	0.0162	-1.62	-0.0422	TRUE	TRUE	TRUE	TRUE	TRUE
1	ESLIDLQEL	1177	1185	9	HLA-A*02:01	1177	0.0284	0.0681	9.06E-01	9.50E-01	0.2515	A2	0.0284	0.0681	0.906	0.95	0.2515	TRUE	TRUE	TRUE	TRUE	TRUE
1	SLIDLQELG	1178	1186	9	HLA-A*02:01	1178	0.0911	0.218	1.49E-02	-1.42E+00	0.1492	A2	0.0911	0.218	0.0149	-1.421	0.1492	TRUE	TRUE	TRUE	TRUE	TRUE
1	LIDLQELGK	1179	1187	9	HLA-A*02:01	1179	0.0311	0.0745	8.92E-01	4.75E-01	0.232	A2	0.0311	0.0745	0.8918	0.475	0.232	TRUE	TRUE	TRUE	TRUE	TRUE
1	IDLQELGKY	1180	1188	9	HLA-A*02:01	1180	0.019	0.0454	8.28E-01	2.71E+00	0.305	A2	0.019	0.0454	0.8284	2.706	0.305	TRUE	TRUE	TRUE	TRUE	TRUE
1	DLQELGKYE	1181	1189	9	HLA-A*02:01	1181	0.1126	0.2694	1.10E-02	-1.89E+00	0.1768	A2	0.1126	0.2694	0.011	-1.885	0.1768	TRUE	TRUE	TRUE	TRUE	TRUE
1	LQELGKYEQ	1182	1190	9	HLA-A*02:01	1182	0.0213	0.051	1.14E-01	-3.09E-01	0.0527	A2	0.0213	0.051	0.114	-0.309	0.0527	TRUE	TRUE	TRUE	TRUE	TRUE
1	QELGKYEQY	1183	1191	9	HLA-A*02:01	1183	0.0178	0.0425	9.77E-01	2.87E+00	0.3324	A2	0.0178	0.0425	0.9769	2.868	0.3324	TRUE	TRUE	TRUE	TRUE	TRUE
1	ELGKYEQYI	1184	1192	9	HLA-A*02:01	1184	0.2194	0.525	7.56E-02	1.38E-01	0.5433	A2	0.2194	0.525	0.0756	0.138	0.5433	TRUE	TRUE	TRUE	TRUE	TRUE
1	LGKYEQYIK	1185	1193	9	HLA-A*02:01	1185	0.0157	0.0377	5.05E-02	3.30E-02	0.0469	A2	0.0157	0.0377	0.0505	0.033	0.0469	TRUE	TRUE	TRUE	TRUE	TRUE
1	GKYEQYIKW	1186	1194	9	HLA-A*02:01	1186	0.0224	0.0536	8.43E-01	9.90E-01	0.2295	A2	0.0224	0.0536	0.8429	0.99	0.2295	TRUE	TRUE	TRUE	TRUE	TRUE
1	KYEQYIKWP	1187	1195	9	HLA-A*02:01	1187	0.0469	0.1123	1.00E-02	3.02E-01	0.1289	A2	0.0469	0.1123	0.01	0.302	0.1289	TRUE	TRUE	TRUE	TRUE	TRUE
1	YEQYIKWPW	1188	1196	9	HLA-A*02:01	1188	0.0209	0.05	1.96E-01	7.98E-01	0.1193	A2	0.0209	0.05	0.1956	0.798	0.1193	TRUE	TRUE	TRUE	TRUE	TRUE
1	EQYIKWPWY	1189	1197	9	HLA-A*02:01	1189	0.0205	0.0491	5.15E-02	2.93E+00	0.2033	A2	0.0205	0.0491	0.0515	2.931	0.2033	TRUE	TRUE	TRUE	TRUE	TRUE
1	QYIKWPWYV	1190	1198	9	HLA-A*02:01	1190	0.2697	0.6454	8.28E-01	4.94E-01	0.7942	A2	0.2697	0.6454	0.8277	0.494	0.7942	TRUE	TRUE	TRUE	TRUE	TRUE
1	YIKWPWYVW	1191	1199	9	HLA-A*02:01	1191	0.0849	0.2032	8.61E-01	9.42E-01	0.3794	A2	0.0849	0.2032	0.8611	0.942	0.3794	TRUE	TRUE	TRUE	TRUE	TRUE
1	IKWPWYVWL	1192	1200	9	HLA-A*02:01	1192	0.2216	0.5304	9.77E-01	1.15E+00	0.7342	A2	0.2216	0.5304	0.9765	1.147	0.7342	TRUE	TRUE	TRUE	TRUE	TRUE
1	KWPWYVWLG	1193	1201	9	HLA-A*02:01	1193	0.0522	0.1249	1.68E-02	-1.05E+00	0.0751	A2	0.0522	0.1249	0.0168	-1.048	0.0751	TRUE	TRUE	TRUE	TRUE	TRUE
1	WPWYVWLGF	1194	1202	9	HLA-A*02:01	1194	0.0286	0.0685	8.23E-01	2.48E+00	0.3158	A2	0.0286	0.0685	0.8228	2.476	0.3158	TRUE	TRUE	TRUE	TRUE	TRUE
1	PWYVWLGFI	1195	1203	9	HLA-A*02:01	1195	0.0552	0.132	1.13E-02	4.32E-01	0.1553	A2	0.0552	0.132	0.0113	0.432	0.1553	TRUE	TRUE	TRUE	TRUE	TRUE
1	WYVWLGFIA	1196	1204	9	HLA-A*02:01	1196	0.1788	0.4279	2.45E-02	-3.65E-01	0.4133	A2	0.1788	0.4279	0.0245	-0.365	0.4133	TRUE	TRUE	TRUE	TRUE	TRUE
1	YVWLGFIAG	1197	1205	9	HLA-A*02:01	1197	0.0745	0.1784	2.32E-02	-1.16E+00	0.1238	A2	0.0745	0.1784	0.0232	-1.161	0.1238	TRUE	TRUE	TRUE	TRUE	TRUE
1	VWLGFIAGL	1198	1206	9	HLA-A*02:01	1198	0.0925	0.2215	7.84E-02	1.23E+00	0.2947	A2	0.0925	0.2215	0.0784	1.229	0.2947	TRUE	TRUE	TRUE	TRUE	TRUE
1	WLGFIAGLI	1199	1207	9	HLA-A*02:01	1199	0.1772	0.4241	1.36E-01	4.11E-01	0.4651	A2	0.1772	0.4241	0.136	0.411	0.4651	TRUE	TRUE	TRUE	TRUE	TRUE
1	LGFIAGLIA	1200	1208	9	HLA-A*02:01	1200	0.0227	0.0543	1.04E-02	-7.42E-01	0.0188	A2	0.0227	0.0543	0.0104	-0.742	0.0188	TRUE	TRUE	TRUE	TRUE	TRUE
1	GFIAGLIAI	1201	1209	9	HLA-A*02:01	1201	0.1032	0.2471	1.61E-02	6.40E-01	0.2815	A2	0.1032	0.2471	0.0161	0.64	0.2815	TRUE	TRUE	TRUE	TRUE	TRUE
1	FIAGLIAIV	1202	1210	9	HLA-A*02:01	1202	0.5704	1.3651	1.84E-01	3.35E-01	1.4094	A2	0.5704	1.3651	0.1838	0.335	1.4094	TRUE	TRUE	TRUE	TRUE	TRUE
1	IAGLIAIVM	1203	1211	9	HLA-A*02:01	1203	0.0269	0.0644	1.24E-01	1.12E-01	0.0886	A2	0.0269	0.0644	0.124	0.112	0.0886	TRUE	TRUE	TRUE	TRUE	TRUE
1	AGLIAIVMV	1204	1212	9	HLA-A*02:01	1204	0.0439	0.1051	5.63E-01	2.19E-01	0.2005	A2	0.0439	0.1051	0.5626	0.219	0.2005	TRUE	TRUE	TRUE	TRUE	TRUE
1	GLIAIVMVT	1205	1213	9	HLA-A*02:01	1205	0.2296	0.5494	2.09E-02	-6.42E-01	0.5205	A2	0.2296	0.5494	0.0209	-0.642	0.5205	TRUE	TRUE	TRUE	TRUE	TRUE
1	LIAIVMVTI	1206	1214	9	HLA-A*02:01	1206	0.1249	0.2989	8.63E-01	6.54E-01	0.4611	A2	0.1249	0.2989	0.8629	0.654	0.4611	TRUE	TRUE	TRUE	TRUE	TRUE
1	IAIVMVTIL	1207	1215	9	HLA-A*02:01	1207	0.0337	0.0807	9.52E-01	1.15E+00	0.2808	A2	0.0337	0.0807	0.952	1.147	0.2808	TRUE	TRUE	TRUE	TRUE	TRUE
1	AIVMVTILL	1208	1216	9	HLA-A*02:01	1208	0.0991	0.2373	9.94E-01	1.31E+00	0.4521	A2	0.0991	0.2373	0.9942	1.314	0.4521	TRUE	TRUE	TRUE	TRUE	TRUE
1	IVMVTILLC	1209	1217	9	HLA-A*02:01	1209	0.1024	0.2451	1.10E-02	3.95E-01	0.2665	A2	0.1024	0.2451	0.011	0.395	0.2665	TRUE	TRUE	TRUE	TRUE	TRUE
1	VMVTILLCC	1210	1218	9	HLA-A*02:01	1210	0.2488	0.5954	1.60E-02	2.85E-01	0.612	A2	0.2488	0.5954	0.016	0.285	0.612	TRUE	TRUE	TRUE	TRUE	TRUE
1	MVTILLCCM	1211	1219	9	HLA-A*02:01	1211	0.0606	0.1451	3.28E-01	5.01E-01	0.2194	A2	0.0606	0.1451	0.3281	0.501	0.2194	TRUE	TRUE	TRUE	TRUE	TRUE
1	VTILLCCMT	1212	1220	9	HLA-A*02:01	1212	0.0385	0.0921	4.28E-02	-5.60E-01	0.0705	A2	0.0385	0.0921	0.0428	-0.56	0.0705	TRUE	TRUE	TRUE	TRUE	TRUE
1	TILLCCMTS	1213	1221	9	HLA-A*02:01	1213	0.0522	0.1249	9.99E-02	-2.07E+00	0.0362	A2	0.0522	0.1249	0.0999	-2.073	0.0362	TRUE	TRUE	TRUE	TRUE	TRUE
1	ILLCCMTSC	1214	1222	9	HLA-A*02:01	1214	0.1296	0.3102	2.01E-02	8.50E-02	0.3174	A2	0.1296	0.3102	0.0201	0.085	0.3174	TRUE	TRUE	TRUE	TRUE	TRUE
1	LLCCMTSCC	1215	1223	9	HLA-A*02:01	1215	0.1173	0.2806	1.63E-02	5.60E-02	0.2859	A2	0.1173	0.2806	0.0163	0.056	0.2859	TRUE	TRUE	TRUE	TRUE	TRUE
1	LCCMTSCCS	1216	1224	9	HLA-A*02:01	1216	0.0356	0.0853	1.06E-02	-2.16E+00	-0.0213	A2	0.0356	0.0853	0.0106	-2.164	-0.0213	TRUE	TRUE	TRUE	TRUE	TRUE
1	CCMTSCCSC	1217	1225	9	HLA-A*02:01	1217	0.0479	0.1146	9.80E-03	1.07E-01	0.1214	A2	0.0479	0.1146	0.0098	0.107	0.1214	TRUE	TRUE	TRUE	TRUE	TRUE
1	CMTSCCSCL	1218	1226	9	HLA-A*02:01	1218	0.1259	0.3014	9.04E-01	9.92E-01	0.4866	A2	0.1259	0.3014	0.9043	0.992	0.4866	TRUE	TRUE	TRUE	TRUE	TRUE
1	MTSCCSCLK	1219	1227	9	HLA-A*02:01	1219	0.0246	0.0588	9.16E-01	4.79E-01	0.2202	A2	0.0246	0.0588	0.9161	0.479	0.2202	TRUE	TRUE	TRUE	TRUE	TRUE
1	TSCCSCLKG	1220	1228	9	HLA-A*02:01	1220	0.0168	0.0401	3.77E-01	-1.33E+00	0.03	A2	0.0168	0.0401	0.3765	-1.331	0.03	TRUE	TRUE	TRUE	TRUE	TRUE
1	SCCSCLKGA	1221	1229	9	HLA-A*02:01	1221	0.0273	0.0654	1.02E-02	-4.43E-01	0.0448	A2	0.0273	0.0654	0.0102	-0.443	0.0448	TRUE	TRUE	TRUE	TRUE	TRUE
1	CCSCLKGAC	1222	1230	9	HLA-A*02:01	1222	0.0227	0.0543	0.0096	0.044	0.0579	A2	0.0227	0.0543	0.0096	0.044	0.0579	TRUE	TRUE	TRUE	TRUE	TRUE
1	CSCLKGACS	1223	1231	9	HLA-A*02:01	1223	0.0184	0.044	0.0114	-2.244	-0.0665	A2	0.0184	0.044	0.0114	-2.244	-0.0665	TRUE	TRUE	TRUE	TRUE	TRUE
1	SCLKGACSC	1224	1232	9	HLA-A*02:01	1224	0.0608	0.1456	0.0121	0.099	0.1523	A2	0.0608	0.1456	0.0121	0.099	0.1523	TRUE	TRUE	TRUE	TRUE	TRUE
1	CLKGACSCG	1225	1233	9	HLA-A*02:01	1225	0.0791	0.1892	0.7632	-1.365	0.2355	A2	0.0791	0.1892	0.7632	-1.365	0.2355	TRUE	TRUE	TRUE	TRUE	TRUE
1	LKGACSCGS	1226	1234	9	HLA-A*02:01	1226	0.0202	0.0483	0.0125	-2.362	-0.0679	A2	0.0202	0.0483	0.0125	-2.362	-0.0679	TRUE	TRUE	TRUE	TRUE	TRUE
1	KGACSCGSC	1227	1235	9	HLA-A*02:01	1227	0.0204	0.0488	0.0173	-0.08	0.0474	A2	0.0204	0.0488	0.0173	-0.08	0.0474	TRUE	TRUE	TRUE	TRUE	TRUE
1	GACSCGSCC	1228	1236	9	HLA-A*02:01	1228	0.0315	0.0753	0.0103	-0.002	0.0767	A2	0.0315	0.0753	0.0103	-0.002	0.0767	TRUE	TRUE	TRUE	TRUE	TRUE
1	ACSCGSCCK	1229	1237	9	HLA-A*02:01	1229	0.0291	0.0695	0.9804	0.595	0.2463	A2	0.0291	0.0695	0.9804	0.595	0.2463	TRUE	TRUE	TRUE	TRUE	TRUE
1	CSCGSCCKF	1230	1238	9	HLA-A*02:01	1230	0.0287	0.0686	0.9636	2.697	0.348	A2	0.0287	0.0686	0.9636	2.697	0.348	TRUE	TRUE	TRUE	TRUE	TRUE
1	SCGSCCKFD	1231	1239	9	HLA-A*02:01	1231	0.0185	0.0443	0.0096	-1.912	-0.0498	A2	0.0185	0.0443	0.0096	-1.912	-0.0498	TRUE	TRUE	TRUE	TRUE	TRUE
1	CGSCCKFDE	1232	1240	9	HLA-A*02:01	1232	0.0161	0.0384	0.0094	-1.823	-0.0513	A2	0.0161	0.0384	0.0094	-1.823	-0.0513	TRUE	TRUE	TRUE	TRUE	TRUE
1	GSCCKFDED	1233	1241	9	HLA-A*02:01	1233	0.0155	0.0372	0.0129	-1.952	-0.0585	A2	0.0155	0.0372	0.0129	-1.952	-0.0585	TRUE	TRUE	TRUE	TRUE	TRUE
1	SCCKFDEDD	1234	1242	9	HLA-A*02:01	1234	0.0199	0.0477	0.01	-1.763	-0.039	A2	0.0199	0.0477	0.01	-1.763	-0.039	TRUE	TRUE	TRUE	TRUE	TRUE
1	CCKFDEDDS	1235	1243	9	HLA-A*02:01	1235	0.0216	0.0516	0.0096	-2.214	-0.0577	A2	0.0216	0.0516	0.0096	-2.214	-0.0577	TRUE	TRUE	TRUE	TRUE	TRUE
1	CKFDEDDSE	1236	1244	9	HLA-A*02:01	1236	0.0263	0.063	0.0096	-1.452	-0.0081	A2	0.0263	0.063	0.0096	-1.452	-0.0081	TRUE	TRUE	TRUE	TRUE	TRUE
1	KFDEDDSEP	1237	1245	9	HLA-A*02:01	1237	0.04	0.0958	0.0411	0.196	0.1118	A2	0.04	0.0958	0.0411	0.196	0.1118	TRUE	TRUE	TRUE	TRUE	TRUE
1	FDEDDSEPV	1238	1246	9	HLA-A*02:01	1238	0.1492	0.3572	0.479	-0.131	0.4225	A2	0.1492	0.3572	0.479	-0.131	0.4225	TRUE	TRUE	TRUE	TRUE	TRUE
1	DEDDSEPVL	1239	1247	9	HLA-A*02:01	1239	0.0268	0.0642	0.9972	0.222	0.2249	A2	0.0268	0.0642	0.9972	0.222	0.2249	TRUE	TRUE	TRUE	TRUE	TRUE
1	EDDSEPVLK	1240	1248	9	HLA-A*02:01	1240	0.0152	0.0364	0.365	-0.362	0.073	A2	0.0152	0.0364	0.365	-0.362	0.073	TRUE	TRUE	TRUE	TRUE	TRUE
1	DDSEPVLKG	1241	1249	9	HLA-A*02:01	1241	0.0161	0.0385	0.124	-2.19	-0.0524	A2	0.0161	0.0385	0.124	-2.19	-0.0524	TRUE	TRUE	TRUE	TRUE	TRUE
1	DSEPVLKGV	1242	1250	9	HLA-A*02:01	1242	0.0335	0.0801	0.8448	-0.244	0.1946	A2	0.0335	0.0801	0.8448	-0.244	0.1946	TRUE	TRUE	TRUE	TRUE	TRUE
1	SEPVLKGVK	1243	1251	9	HLA-A*02:01	1243	0.0151	0.0362	0.3209	0.228	0.0958	A2	0.0151	0.0362	0.3209	0.228	0.0958	TRUE	TRUE	TRUE	TRUE	TRUE
1	EPVLKGVKL	1244	1252	9	HLA-A*02:01	1244	0.0174	0.0417	0.9988	0.578	0.2204	A2	0.0174	0.0417	0.9988	0.578	0.2204	TRUE	TRUE	TRUE	TRUE	TRUE
1	PVLKGVKLH	1245	1253	9	HLA-A*02:01	1245	0.0296	0.071	0.388	-1.026	0.0779	A2	0.0296	0.071	0.388	-1.026	0.0779	TRUE	TRUE	TRUE	TRUE	TRUE
1	VLKGVKLHY	1246	1254	9	HLA-A*02:01	1246	0.1035	0.2478	0.9991	2.859	0.5406	A2	0.1035	0.2478	0.9991	2.859	0.5406	TRUE	TRUE	TRUE	TRUE	TRUE
1	LKGVKLHYT	1247	1255	9	HLA-A*02:01	1247	0.0327	0.0783	0.0794	-0.768	0.0518	A2	0.0327	0.0783	0.0794	-0.768	0.0518	TRUE	TRUE	TRUE	TRUE	TRUE

seq #	peptide	start	end	peptide length	allele	peptide index	NetCTLpan MH	NetCTLpan tap	NetCTLpan clea	NetCTLpan com	NetCTLpan per	legacy mhc_pr	legacy tap_pre	legacy cleavag	legacy combine	legacy %_rank	equal mhc_pre	equal tap_pred	equal cleavage	equal combine	equal %_rank
1	MFIFLLFLT	1	9	9	HLA-A*02:01	1	0.191	-0.43	0.04149	0.18958	50	0.191	-0.43	0.04149	0.18958	50	TRUE	TRUE	TRUE	TRUE	TRUE
1	FIFLLFLTL	2	10	9	HLA-A*02:01	2	0.524	1.136	0.97615	0.77203	1.5	0.524	1.136	0.97615	0.77203	1.5	TRUE	TRUE	TRUE	TRUE	TRUE
1	IFLLFLTLT	3	11	9	HLA-A*02:01	3	0.117	-0.602	0.24184	0.15636	50	0.117	-0.602	0.24184	0.15636	50	TRUE	TRUE	TRUE	TRUE	TRUE
1	FLLFLTLTS	4	12	9	HLA-A*02:01	4	0.476	-2.294	0.45706	0.52149	5	0.476	-2.294	0.45706	0.52149	5	TRUE	TRUE	TRUE	TRUE	TRUE
1	LLFLTLTSG	5	13	9	HLA-A*02:01	5	0.206	-1.365	0.42264	0.26697	32	0.206	-1.365	0.42264	0.26697	32	TRUE	TRUE	TRUE	TRUE	TRUE
1	LFLTLTSGS	6	14	9	HLA-A*02:01	6	0.039	-2.008	0.04671	-0.00069	50	0.039	-2.008	0.04671	-0.00069	50	TRUE	TRUE	TRUE	TRUE	TRUE
1	FLTLTSGSD	7	15	9	HLA-A*02:01	7	0.071	-1.879	0.03678	0.0323	50	0.071	-1.879	0.03678	0.0323	50	TRUE	TRUE	TRUE	TRUE	TRUE
1	LTLTSGSDL	8	16	9	HLA-A*02:01	8	0.083	0.85	0.87928	0.30209	16	0.083	0.85	0.87928	0.30209	16	TRUE	TRUE	TRUE	TRUE	TRUE
1	TLTSGSDLD	9	17	9	HLA-A*02:01	9	0.024	-1.805	0.1261	0.00725	50	0.024	-1.805	0.1261	0.00725	50	TRUE	TRUE	TRUE	TRUE	TRUE
1	LTSGSDLDR	10	18	9	HLA-A*02:01	10	0.015	1.473	0.71261	0.21216	32	0.015	1.473	0.71261	0.21216	32	TRUE	TRUE	TRUE	TRUE	TRUE
1	TSGSDLDRC	11	19	9	HLA-A*02:01	11	0.016	-0.081	0.02337	0.01923	50	0.016	-0.081	0.02337	0.01923	50	TRUE	TRUE	TRUE	TRUE	TRUE
1	SGSDLDRCT	12	20	9	HLA-A*02:01	12	0.011	-0.913	0.04322	-0.0021	50	0.011	-0.913	0.04322	-0.0021	50	TRUE	TRUE	TRUE	TRUE	TRUE
1	GSDLDRCTT	13	21	9	HLA-A*02:01	13	0.026	-0.966	0.08655	0.02132	50	0.026	-0.966	0.08655	0.02132	50	TRUE	TRUE	TRUE	TRUE	TRUE
1	SDLDRCTTF	14	22	9	HLA-A*02:01	14	0.036	2.268	0.93058	0.30208	16	0.036	2.268	0.93058	0.30208	16	TRUE	TRUE	TRUE	TRUE	TRUE
1	DLDRCTTFD	15	23	9	HLA-A*02:01	15	0.019	-2.234	0.04825	-0.02599	50	0.019	-2.234	0.04825	-0.02599	50	TRUE	TRUE	TRUE	TRUE	TRUE
1	LDRCTTFDD	16	24	9	HLA-A*02:01	16	0.007	-2.209	0.02837	-0.04184	50	0.007	-2.209	0.02837	-0.04184	50	TRUE	TRUE	TRUE	TRUE	TRUE
1	DRCTTFDDV	17	25	9	HLA-A*02:01	17	0.011	0.262	0.5907	0.15046	50	0.011	0.262	0.5907	0.15046	50	TRUE	TRUE	TRUE	TRUE	TRUE
1	RCTTFDDVQ	18	26	9	HLA-A*02:01	18	0.006	-0.016	0.02736	0.01176	50	0.006	-0.016	0.02736	0.01176	50	TRUE	TRUE	TRUE	TRUE	TRUE
1	CTTFDDVQA	19	27	9	HLA-A*02:01	19	0.073	-0.502	0.16952	0.09859	50	0.073	-0.502	0.16952	0.09859	50	TRUE	TRUE	TRUE	TRUE	TRUE
1	TTFDDVQAP	20	28	9	HLA-A*02:01	20	0.028	0.167	0.14052	0.06379	50	0.028	0.167	0.14052	0.06379	50	TRUE	TRUE	TRUE	TRUE	TRUE
1	TFDDVQAPN	21	29	9	HLA-A*02:01	21	0.028	-1.335	0.12355	0.02242	50	0.028	-1.335	0.12355	0.02242	50	TRUE	TRUE	TRUE	TRUE	TRUE
1	FDDVQAPNY	22	30	9	HLA-A*02:01	22	0.02	2.356	0.69483	0.23524	32	0.02	2.356	0.69483	0.23524	32	TRUE	TRUE	TRUE	TRUE	TRUE
1	DDVQAPNYT	23	31	9	HLA-A*02:01	23	0.008	-1.356	0.05004	-0.01464	50	0.008	-1.356	0.05004	-0.01464	50	TRUE	TRUE	TRUE	TRUE	TRUE
1	DVQAPNYTQ	24	32	9	HLA-A*02:01	24	0.014	-0.454	0.61409	0.14082	50	0.014	-0.454	0.61409	0.14082	50	TRUE	TRUE	TRUE	TRUE	TRUE
1	VQAPNYTQH	25	33	9	HLA-A*02:01	25	0.044	-0.52	0.41801	0.12505	50	0.044	-0.52	0.41801	0.12505	50	TRUE	TRUE	TRUE	TRUE	TRUE
1	QAPNYTQHT	26	34	9	HLA-A*02:01	26	0.025	-0.608	0.15587	0.04487	50	0.025	-0.608	0.15587	0.04487	50	TRUE	TRUE	TRUE	TRUE	TRUE
1	APNYTQHTS	27	35	9	HLA-A*02:01	27	0.014	-2.549	0.26564	0.01004	50	0.014	-2.549	0.26564	0.01004	50	TRUE	TRUE	TRUE	TRUE	TRUE
1	PNYTQHTSS	28	36	9	HLA-A*02:01	28	0.007	-2.537	0.04883	-0.04544	50	0.007	-2.537	0.04883	-0.04544	50	TRUE	TRUE	TRUE	TRUE	TRUE
1	NYTQHTSSM	29	37	9	HLA-A*02:01	29	0.023	0.425	0.94026	0.24518	32	0.023	0.425	0.94026	0.24518	32	TRUE	TRUE	TRUE	TRUE	TRUE
1	YTQHTSSMR	30	38	9	HLA-A*02:01	30	0.033	1.552	0.24152	0.12614	50	0.033	1.552	0.24152	0.12614	50	TRUE	TRUE	TRUE	TRUE	TRUE
1	TQHTSSMRG	31	39	9	HLA-A*02:01	31	0.016	-1.363	0.04799	-0.00728	50	0.016	-1.363	0.04799	-0.00728	50	TRUE	TRUE	TRUE	TRUE	TRUE
1	QHTSSMRGV	32	40	9	HLA-A*02:01	32	0.022	0.309	0.10921	0.0543	50	0.022	0.309	0.10921	0.0543	50	TRUE	TRUE	TRUE	TRUE	TRUE
1	HTSSMRGVY	33	41	9	HLA-A*02:01	33	0.035	2.759	0.57864	0.23417	32	0.035	2.759	0.57864	0.23417	32	TRUE	TRUE	TRUE	TRUE	TRUE
1	TSSMRGVYY	34	42	9	HLA-A*02:01	34	0.038	2.917	0.24741	0.16659	50	0.038	2.917	0.24741	0.16659	50	TRUE	TRUE	TRUE	TRUE	TRUE
1	SSMRGVYYP	35	43	9	HLA-A*02:01	35	0.063	0.273	0.03909	0.07862	50	0.063	0.273	0.03909	0.07862	50	TRUE	TRUE	TRUE	TRUE	TRUE
1	SMRGVYYPD	36	44	9	HLA-A*02:01	36	0.071	-1.538	0.1207	0.05971	50	0.071	-1.538	0.1207	0.05971	50	TRUE	TRUE	TRUE	TRUE	TRUE
1	MRGVYYPDE	37	45	9	HLA-A*02:01	37	0.007	-1.421	0.03946	-0.01965	50	0.007	-1.421	0.03946	-0.01965	50	TRUE	TRUE	TRUE	TRUE	TRUE
1	RGVYYPDEI	38	46	9	HLA-A*02:01	38	0.058	0.626	0.24098	0.12787	50	0.058	0.626	0.24098	0.12787	50	TRUE	TRUE	TRUE	TRUE	TRUE
1	GVYYPDEIF	39	47	9	HLA-A*02:01	39	0.06	2.813	0.59066	0.26322	32	0.06	2.813	0.59066	0.26322	32	TRUE	TRUE	TRUE	TRUE	TRUE
1	VYYPDEIFR	40	48	9	HLA-A*02:01	40	0.032	1.968	0.40169	0.17158	50	0.032	1.968	0.40169	0.17158	50	TRUE	TRUE	TRUE	TRUE	TRUE
1	YYPDEIFRS	41	49	9	HLA-A*02:01	41	0.064	-1.969	0.20582	0.06108	50	0.064	-1.969	0.20582	0.06108	50	TRUE	TRUE	TRUE	TRUE	TRUE
1	YPDEIFRSD	42	50	9	HLA-A*02:01	42	0.024	-2.368	0.32718	0.03842	50	0.024	-2.368	0.32718	0.03842	50	TRUE	TRUE	TRUE	TRUE	TRUE
1	PDEIFRSDT	43	51	9	HLA-A*02:01	43	0.005	-1.689	0.02455	-0.0317	50	0.005	-1.689	0.02455	-0.0317	50	TRUE	TRUE	TRUE	TRUE	TRUE
1	DEIFRSDTL	44	52	9	HLA-A*02:01	44	0.024	0.427	0.95499	0.24955	32	0.024	0.427	0.95499	0.24955	32	TRUE	TRUE	TRUE	TRUE	TRUE
1	EIFRSDTLY	45	53	9	HLA-A*02:01	45	0.042	2.822	0.96165	0.32892	16	0.042	2.822	0.96165	0.32892	16	TRUE	TRUE	TRUE	TRUE	TRUE
1	IFRSDTLYL	46	54	9	HLA-A*02:01	46	0.099	1.027	0.90775	0.32892	16	0.099	1.027	0.90775	0.32892	16	TRUE	TRUE	TRUE	TRUE	TRUE
1	FRSDTLYLT	47	55	9	HLA-A*02:01	47	0.092	-0.589	0.12276	0.1049	50	0.092	-0.589	0.12276	0.1049	50	TRUE	TRUE	TRUE	TRUE	TRUE
1	RSDTLYLTQ	48	56	9	HLA-A*02:01	48	0.03	-0.102	0.21295	0.07536	50	0.03	-0.102	0.21295	0.07536	50	TRUE	TRUE	TRUE	TRUE	TRUE
1	SDTLYLTQD	49	57	9	HLA-A*02:01	49	0.011	-1.974	0.056	-0.02575	50	0.011	-1.974	0.056	-0.02575	50	TRUE	TRUE	TRUE	TRUE	TRUE
1	DTLYLTQDL	50	58	9	HLA-A*02:01	50	0.048	0.689	0.73035	0.22955	32	0.048	0.689	0.73035	0.22955	32	TRUE	TRUE	TRUE	TRUE	TRUE
1	TLYLTQDLF	51	59	9	HLA-A*02:01	51	0.146	2.581	0.19505	0.25441	32	0.146	2.581	0.19505	0.25441	32	TRUE	TRUE	TRUE	TRUE	TRUE
1	LYLTQDLFL	52	60	9	HLA-A*02:01	52	0.092	1.251	0.53681	0.24406	32	0.092	1.251	0.53681	0.24406	32	TRUE	TRUE	TRUE	TRUE	TRUE
1	YLTQDLFLP	53	61	9	HLA-A*02:01	53	0.318	0.116	0.04381	0.33076	16	0.318	0.116	0.04381	0.33076	16	TRUE	TRUE	TRUE	TRUE	TRUE
1	LTQDLFLPF	54	62	9	HLA-A*02:01	54	0.13	2.433	0.61086	0.32827	16	0.13	2.433	0.61086	0.32827	16	TRUE	TRUE	TRUE	TRUE	TRUE
1	TQDLFLPFY	55	63	9	HLA-A*02:01	55	0.097	2.851	0.81253	0.35109	10	0.097	2.851	0.81253	0.35109	10	TRUE	TRUE	TRUE	TRUE	TRUE
1	QDLFLPFYS	56	64	9	HLA-A*02:01	56	0.032	-2.512	0.03554	-0.0228	50	0.032	-2.512	0.03554	-0.0228	50	TRUE	TRUE	TRUE	TRUE	TRUE
1	DLFLPFYSN	57	65	9	HLA-A*02:01	57	0.048	-1.549	0.18816	0.05161	50	0.048	-1.549	0.18816	0.05161	50	TRUE	TRUE	TRUE	TRUE	TRUE
1	LFLPFYSNV	58	66	9	HLA-A*02:01	58	0.293	0.365	0.64158	0.44648	6	0.293	0.365	0.64158	0.44648	6	TRUE	TRUE	TRUE	TRUE	TRUE
1	FLPFYSNVT	59	67	9	HLA-A*02:01	59	0.522	-0.748	0.29272	0.56916	4	0.522	-0.748	0.29272	0.56916	4	TRUE	TRUE	TRUE	TRUE	TRUE
1	LPFYSNVTG	60	68	9	HLA-A*02:01	60	0.018	-1.689	0.30645	0.04473	50	0.018	-1.689	0.30645	0.04473	50	TRUE	TRUE	TRUE	TRUE	TRUE
1	PFYSNVTGF	61	69	9	HLA-A*02:01	61	0.024	2.457	0.45849	0.18858	50	0.024	2.457	0.45849	0.18858	50	TRUE	TRUE	TRUE	TRUE	TRUE
1	FYSNVTGFH	62	70	9	HLA-A*02:01	62	0.039	-0.515	0.24162	0.08049	50	0.039	-0.515	0.24162	0.08049	50	TRUE	TRUE	TRUE	TRUE	TRUE
1	YSNVTGFHT	63	71	9	HLA-A*02:01	63	0.071	-0.884	0.03629	0.05707	50	0.071	-0.884	0.03629	0.05707	50	TRUE	TRUE	TRUE	TRUE	TRUE
1	SNVTGFHTI	64	72	9	HLA-A*02:01	64	0.117	0.461	0.9554	0.34349	16	0.117	0.461	0.9554	0.34349	16	TRUE	TRUE	TRUE	TRUE	TRUE
1	NVTGFHTIN	65	73	9	HLA-A*02:01	65	0.022	-1.122	0.22287	0.04409	50	0.022	-1.122	0.22287	0.04409	50	TRUE	TRUE	TRUE	TRUE	TRUE
1	VTGFHTINH	66	74	9	HLA-A*02:01	66	0.026	-0.517	0.07578	0.03013	50	0.026	-0.517	0.07578	0.03013	50	TRUE	TRUE	TRUE	TRUE	TRUE
1	TGFHTINHT	67	75	9	HLA-A*02:01	67	0.027	-0.834	0.13278	0.03603	50	0.027	-0.834	0.13278	0.03603	50	TRUE	TRUE	TRUE	TRUE	TRUE
1	GFHTINHTF	68	76	9	HLA-A*02:01	68	0.052	2.563	0.95214	0.33031	16	0.052	2.563	0.95214	0.33031	16	TRUE	TRUE	TRUE	TRUE	TRUE
1	FHTINHTFG	69	77	9	HLA-A*02:01	69	0.014	-1.549	0.02879	-0.01825	50	0.014	-1.549	0.02879	-0.01825	50	TRUE	TRUE	TRUE	TRUE	TRUE
1	HTINHTFGN	70	78	9	HLA-A*02:01	70	0.051	-1.464	0.02417	0.01984	50	0.051	-1.464	0.02417	0.01984	50	TRUE	TRUE	TRUE	TRUE	TRUE
1	TINHTFGNP	71	79	9	HLA-A*02:01	71	0.022	0.095	0.0282	0.03072	50	0.022	0.095	0.0282	0.03072	50	TRUE	TRUE	TRUE	TRUE	TRUE
1	INHTFGNPV	72	80	9	HLA-A*02:01	72	0.067	0.225	0.36634	0.15505	50	0.067	0.225	0.36634	0.15505	50	TRUE	TRUE	TRUE	TRUE	TRUE
1	NHTFGNPVI	73	81	9	HLA-A*02:01	73	0.038	0.552	0.5575	0.17724	50	0.038	0.552	0.5575	0.17724	50	TRUE	TRUE	TRUE	TRUE	TRUE
1	HTFGNPVIP	74	82	9	HLA-A*02:01	74	0.026	0.245	0.06098	0.04585	50	0.026	0.245	0.06098	0.04585	50	TRUE	TRUE	TRUE	TRUE	TRUE
1	TFGNPVIPF	75	83	9	HLA-A*02:01	75	0.05	2.495	0.44401	0.21228	32	0.05	2.495	0.44401	0.21228	32	TRUE	TRUE	TRUE	TRUE	TRUE
1	FGNPVIPFK	76	84	9	HLA-A*02:01	76	0.041	0.019	0.2285	0.09289	50	0.041	0.019	0.2285	0.09289	50	TRUE	TRUE	TRUE	TRUE	TRUE
1	GNPVIPFKD	77	85	9	HLA-A*02:01	77	0.008	-2.267	0.02951	-0.04204	50	0.008	-2.267	0.02951	-0.04204	50	TRUE	TRUE	TRUE	TRUE	TRUE
1	NPVIPFKDG	78	86	9	HLA-A*02:01	78	0.007	-1.615	0.04641	-0.02293	50	0.007	-1.615	0.04641	-0.02293	50	TRUE	TRUE	TRUE	TRUE	TRUE
1	PVIPFKDGI	79	87	9	HLA-A*02:01	79	0.057	0.403	0.26691	0.12713	50	0.057	0.403	0.26691	0.12713	50	TRUE	TRUE	TRUE	TRUE	TRUE
1	VIPFKDGIY	80	88	9	HLA-A*02:01	80	0.029	2.955	0.89714	0.30473	16	0.029	2.955	0.89714	0.30473	16	TRUE	TRUE	TRUE	TRUE	TRUE
1	IPFKDGIYF	81	89	9	HLA-A*02:01	81	0.041	2.412	0.97496	0.32067	16	0.041	2.412	0.97496	0.32067	16	TRUE	TRUE	TRUE	TRUE	TRUE
1	PFKDGIYFA	82	90	9	HLA-A*02:01	82	0.04	-0.841	0.68813	0.1738	50	0.04	-0.841	0.68813	0.1738	50	TRUE	TRUE	TRUE	TRUE	TRUE
1	FKDGIYFAA	83	91	9	HLA-A*02:01	83	0.226	-0.902	0.21425	0.25166	32	0.226	-0.902	0.21425	0.25166	32	TRUE	TRUE	TRUE	TRUE	TRUE
1	KDGIYFAAT	84	92	9	HLA-A*02:01	84	0.032	-1.174	0.04501	0.01278	50	0.032	-1.174	0.04501	0.01278	50	TRUE	TRUE	TRUE	TRUE	TRUE
1	DGIYFAATE	85	93	9	HLA-A*02:01	85	0.011	-1.978	0.02331	-0.03321	50	0.011	-1.978	0.02331	-0.03321	50	TRUE	TRUE	TRUE	TRUE	TRUE
1	GIYFAATEK	86	94	9	HLA-A*02:01	86	0.058	0.462	0.89867	0.27175	32	0.058	0.462	0.89867	0.27175	32	TRUE	TRUE	TRUE	TRUE	TRUE
1	IYFAATEKS	87	95	9	HLA-A*02:01	87	0.025	-1.876	0.05647	-0.00919	50	0.025	-1.876	0.05647	-0.00919	50	TRUE	TRUE	TRUE	TRUE	TRUE
1	YFAATEKSN	88	96	9	HLA-A*02:01	88	0.018	-1.142	0.02244	-0.0055	50	0.018	-1.142	0.02244	-0.0055	50	TRUE	TRUE	TRUE	TRUE	TRUE
1	FAATEKSNV	89	97	9	HLA-A*02:01	89	0.174	0.237	0.08407	0.19884	50	0.174	0.237	0.08407	0.19884	50	TRUE	TRUE	TRUE	TRUE	TRUE
1	AATEKSNVV	90	98	9	HLA-A*02:01	90	0.106	0.37	0.93433	0.32547	16	0.106	0.37	0.93433	0.32547	16	TRUE	TRUE	TRUE	TRUE	TRUE
1	ATEKSNVVR	91	99	9	HLA-A*02:01	91	0.014	1.617	0.15547	0.08941	50	0.014	1.617	0.15547	0.08941	50	TRUE	TRUE	TRUE	TRUE	TRUE
1	TEKSNVVRG	92	100	9	HLA-A*02:01	92	0.006	-1.4	0.03936	-0.02014	50	0.006	-1.4	0.03936	-0.02014	50	TRUE	TRUE	TRUE	TRUE	TRUE
1	EKSNVVRGW	93	101	9	HLA-A*02:01	93	0.012	0.736	0.4399	0.12938	50	0.012	0.736	0.4399	0.12938	50	TRUE	TRUE	TRUE	TRUE	TRUE
1	KSNVVRGWV	94	102	9	HLA-A*02:01	94	0.099	0.201	0.11197	0.12922	50	0.099	0.201	0.11197	0.12922	50	TRUE	TRUE	TRUE	TRUE	TRUE
1	SNVVRGWVF	95	103	9	HLA-A*02:01	95	0.034	2.614	0.52602	0.2177	32	0.034	2.614	0.52602	0.2177	32	TRUE	TRUE	TRUE	TRUE	TRUE
1	NVVRGWVFG	96	104	9	HLA-A*02:01	96	0.044	-1.088	0.02678	0.02283	50	0.044	-1.088	0.02678	0.02283	50	TRUE	TRUE	TRUE	TRUE	TRUE
1	VVRGWVFGS	97	105	9	HLA-A*02:01	97	0.116	-1.855	0.0538	0.08173	50	0.116	-1.855	0.0538	0.08173	50	TRUE	TRUE	TRUE	TRUE	TRUE
1	VRGWVFGST	98	106	9	HLA-A*02:01	98	0.034	-0.553	0.18755	0.06237	50	0.034	-0.553	0.18755	0.06237	50	TRUE	TRUE	TRUE	TRUE	TRUE
1	RGWVFGSTM	99	107	9	HLA-A*02:01	99	0.13	0.501	0.96035	0.3586	10	0.13	0.501	0.96035	0.3586	10	TRUE	TRUE	TRUE	TRUE	TRUE
1	GWVFGSTMN	100	108	9	HLA-A*02:01	100	0.012	-1.038	0.12012	0.01308	50	0.012	-1.038	0.12012	0.01308	50	TRUE	TRUE	TRUE	TRUE	TRUE
1	WVFGSTMNN	101	109	9	HLA-A*02:01	101	0.068	-1.122	0.04483	0.05004	50	0.068	-1.122	0.04483	0.05004	50	TRUE	TRUE	TRUE	TRUE	TRUE
1	VFGSTMNNK	102	110	9	HLA-A*02:01	102	0.017	0.475	0.1593	0.06472	50	0.017	0.475	0.1593	0.06472	50	TRUE	TRUE	TRUE	TRUE	TRUE
1	FGSTMNNKS	103	111	9	HLA-A*02:01	103	0.017	-2.564	0.0264	-0.04116	50	0.017	-2.564	0.0264	-0.04116	50	TRUE	TRUE	TRUE	TRUE	TRUE
1	GSTMNNKSQ	104	112	9	HLA-A*02:01	104	0.005	-0.385	0.02401	0.00078	50	0.005	-0.385	0.02401	0.00078	50	TRUE	TRUE	TRUE	TRUE	TRUE
1	STMNNKSQS	105	113	9	HLA-A*02:01	105	0.023	-2.179	0.0235	-0.02619	50	0.023	-2.179	0.0235	-0.02619	50	TRUE	TRUE	TRUE	TRUE	TRUE
1	TMNNKSQSV	106	114	9	HLA-A*02:01	106	0.507	0.375	0.62734	0.65753	3	0.507	0.375	0.62734	0.65753	3	TRUE	TRUE	TRUE	TRUE	TRUE
1	MNNKSQSVI	107	115	9	HLA-A*02:01	107	0.032	0.345	0.0736	0.05719	50	0.032	0.345	0.0736	0.05719	50	TRUE	TRUE	TRUE	TRUE	TRUE
1	NNKSQSVII	108	116	9	HLA-A*02:01	108	0.015	0.453	0.09107	0.04682	50	0.015	0.453	0.09107	0.04682	50	TRUE	TRUE	TRUE	TRUE	TRUE
1	NKSQSVIII	109	117	9	HLA-A*02:01	109	0.031	0.758	0.22919	0.10152	50	0.031	0.758	0.22919	0.10152	50	TRUE	TRUE	TRUE	TRUE	TRUE
1	KSQSVIIIN	110	118	9	HLA-A*02:01	110	0.02	-1.059	0.02838	-0.00009	50	0.02	-1.059	0.02838	-0.00009	50	TRUE	TRUE	TRUE	TRUE	TRUE
1	SQSVIIINN	111	119	9	HLA-A*02:01	111	0.047	-1.09	0.02911	0.0263	50	0.047	-1.09	0.02911	0.0263	50	TRUE	TRUE	TRUE	TRUE	TRUE
1	QSVIIINNS	112	120	9	HLA-A*02:01	112	0.028	-2.06	0.02621	-0.0176	50	0.028	-2.06	0.02621	-0.0176	50	TRUE	TRUE	TRUE	TRUE	TRUE
1	SVIIINNST	113	121	9	HLA-A*02:01	113	0.131	-0.494	0.06516	0.13331	50	0.131	-0.494	0.06516	0.13331	50	TRUE	TRUE	TRUE	TRUE	TRUE
1	VIIINNSTN	114	122	9	HLA-A*02:01	114	0.04	-1.002	0.02357	0.02025	50	0.04	-1.002	0.02357	0.02025	50	TRUE	TRUE	TRUE	TRUE	TRUE
1	IIINNSTNV	115	123	9	HLA-A*02:01	115	0.56	0.582	0.50925	0.68913	2	0.56	0.582	0.50925	0.68913	2	TRUE	TRUE	TRUE	TRUE	TRUE
1	IINNSTNVV	116	124	9	HLA-A*02:01	116	0.325	0.381	0.8336	0.52208	5	0.325	0.381	0.8336	0.52208	5	TRUE	TRUE	TRUE	TRUE	TRUE
1	INNSTNVVI	117	125	9	HLA-A*02:01	117	0.044	0.32	0.52946	0.17113	50	0.044	0.32	0.52946	0.17113	50	TRUE	TRUE	TRUE	TRUE	TRUE
1	NNSTNVVIR	118	126	9	HLA-A*02:01	118	0.008	1.374	0.22876	0.09382	50	0.008	1.374	0.22876	0.09382	50	TRUE	TRUE	TRUE	TRUE	TRUE
1	NSTNVVIRA	119	127	9	HLA-A*02:01	119	0.076	-0.353	0.716	0.22827	32	0.076	-0.353	0.716	0.22827	32	TRUE	TRUE	TRUE	TRUE	TRUE
1	STNVVIRAC	120	128	9	HLA-A*02:01	120	0.062	0.093	0.05255	0.07615	50	0.062	0.093	0.05255	0.07615	50	TRUE	TRUE	TRUE	TRUE	TRUE
1	TNVVIRACN	121	129	9	HLA-A*02:01	121	0.007	-1.357	0.02768	-0.0207	50	0.007	-1.357	0.02768	-0.0207	50	TRUE	TRUE	TRUE	TRUE	TRUE
1	NVVIRACNF	122	130	9	HLA-A*02:01	122	0.046	2.798	0.8954	0.31741	16	0.046	2.798	0.8954	0.31741	16	TRUE	TRUE	TRUE	TRUE	TRUE
1	VVIRACNFE	123	131	9	HLA-A*02:01	123	0.026	-1.155	0.0252	0.00279	50	0.026	-1.155	0.0252	0.00279	50	TRUE	TRUE	TRUE	TRUE	TRUE
1	VIRACNFEL	124	132	9	HLA-A*02:01	124	0.374	1.241	0.81725	0.58891	4	0.374	1.241	0.81725	0.58891	4	TRUE	TRUE	TRUE	TRUE	TRUE
1	IRACNFELC	125	133	9	HLA-A*02:01	125	0.025	0.308	0.02707	0.03879	50	0.025	0.308	0.02707	0.03879	50	TRUE	TRUE	TRUE	TRUE	TRUE
1	RACNFELCD	126	134	9	HLA-A*02:01	126	0.015	-1.501	0.02269	-0.01742	50	0.015	-1.501	0.02269	-0.01742	50	TRUE	TRUE	TRUE	TRUE	TRUE
1	ACNFELCDN	127	135	9	HLA-A*02:01	127	0.007	-1.113	0.02277	-0.0157	50	0.007	-1.113	0.02277	-0.0157	50	TRUE	TRUE	TRUE	TRUE	TRUE
1	CNFELCDNP	128	136	9	HLA-A*02:01	128	0.009	0.177	0.02369	0.01876	50	0.009	0.177	0.02369	0.01876	50	TRUE	TRUE	TRUE	TRUE	TRUE
1	NFELCDNPF	129	137	9	HLA-A*02:01	129	0.032	2.588	0.30853	0.16612	50	0.032	2.588	0.30853	0.16612	50	TRUE	TRUE	TRUE	TRUE	TRUE
1	FELCDNPFF	130	138	9	HLA-A*02:01	130	0.067	2.463	0.50987	0.2433	32	0.067	2.463	0.50987	0.2433	32	TRUE	TRUE	TRUE	TRUE	TRUE
1	ELCDNPFFA	131	139	9	HLA-A*02:01	131	0.398	-0.816	0.24751	0.43329	7	0.398	-0.816	0.24751	0.43329	7	TRUE	TRUE	TRUE	TRUE	TRUE
1	LCDNPFFAV	132	140	9	HLA-A*02:01	132	0.294	-0.004	0.84992	0.48513	6	0.294	-0.004	0.84992	0.48513	6	TRUE	TRUE	TRUE	TRUE	TRUE
1	CDNPFFAVS	133	141	9	HLA-A*02:01	133	0.023	-2.64	0.05181	-0.03134	50	0.023	-2.64	0.05181	-0.03134	50	TRUE	TRUE	TRUE	TRUE	TRUE
1	DNPFFAVSK	134	142	9	HLA-A*02:01	134	0.015	-0.026	0.67556	0.16635	50	0.015	-0.026	0.67556	0.16635	50	TRUE	TRUE	TRUE	TRUE	TRUE
1	NPFFAVSKP	135	143	9	HLA-A*02:01	135	0.01	-0.19	0.69704	0.16208	50	0.01	-0.19	0.69704	0.16208	50	TRUE	TRUE	TRUE	TRUE	TRUE
1	PFFAVSKPM	136	144	9	HLA-A*02:01	136	0.032	0.245	0.75178	0.20728	32	0.032	0.245	0.75178	0.20728	32	TRUE	TRUE	TRUE	TRUE	TRUE
1	FFAVSKPMG	137	145	9	HLA-A*02:01	137	0.024	-1.451	0.02295	-0.00711	50	0.024	-1.451	0.02295	-0.00711	50	TRUE	TRUE	TRUE	TRUE	TRUE
1	FAVSKPMGT	138	146	9	HLA-A*02:01	138	0.113	-0.775	0.02961	0.10029	50	0.113	-0.775	0.02961	0.10029	50	TRUE	TRUE	TRUE	TRUE	TRUE
1	AVSKPMGTQ	139	147	9	HLA-A*02:01	139	0.024	0.068	0.13627	0.05636	50	0.024	0.068	0.13627	0.05636	50	TRUE	TRUE	TRUE	TRUE	TRUE
1	VSKPMGTQT	140	148	9	HLA-A*02:01	140	0.019	-0.451	0.03422	0.01542	50	0.019	-0.451	0.03422	0.01542	50	TRUE	TRUE	TRUE	TRUE	TRUE
1	SKPMGTQTH	141	149	9	HLA-A*02:01	141	0.008	-0.401	0.05944	0.01135	50	0.008	-0.401	0.05944	0.01135	50	TRUE	TRUE	TRUE	TRUE	TRUE
1	KPMGTQTHT	142	150	9	HLA-A*02:01	142	0.027	-0.784	0.51961	0.12431	50	0.027	-0.784	0.51961	0.12431	50	TRUE	TRUE	TRUE	TRUE	TRUE
1	PMGTQTHTM	143	151	9	HLA-A*02:01	143	0.082	-0.116	0.85539	0.27156	32	0.082	-0.116	0.85539	0.27156	32	TRUE	TRUE	TRUE	TRUE	TRUE
1	MGTQTHTMI	144	152	9	HLA-A*02:01	144	0.056	0.134	0.31205	0.12956	50	0.056	0.134	0.31205	0.12956	50	TRUE	TRUE	TRUE	TRUE	TRUE
1	GTQTHTMIF	145	153	9	HLA-A*02:01	145	0.052	2.338	0.55288	0.23485	32	0.052	2.338	0.55288	0.23485	32	TRUE	TRUE	TRUE	TRUE	TRUE
1	TQTHTMIFD	146	154	9	HLA-A*02:01	146	0.033	-1.827	0.02296	-0.00751	50	0.033	-1.827	0.02296	-0.00751	50	TRUE	TRUE	TRUE	TRUE	TRUE
1	QTHTMIFDN	147	155	9	HLA-A*02:01	147	0.017	-1.284	0.02782	-0.00884	50	0.017	-1.284	0.02782	-0.00884	50	TRUE	TRUE	TRUE	TRUE	TRUE
1	THTMIFDNA	148	156	9	HLA-A*02:01	148	0.03	-0.634	0.08001	0.03215	50	0.03	-0.634	0.08001	0.03215	50	TRUE	TRUE	TRUE	TRUE	TRUE
1	HTMIFDNAF	149	157	9	HLA-A*02:01	149	0.122	2.497	0.6691	0.33497	16	0.122	2.497	0.6691	0.33497	16	TRUE	TRUE	TRUE	TRUE	TRUE
1	TMIFDNAFN	150	158	9	HLA-A*02:01	150	0.129	-1.169	0.02451	0.10529	50	0.129	-1.169	0.02451	0.10529	50	TRUE	TRUE	TRUE	TRUE	TRUE

seq #	peptide	start	end	peptide length	allele	peptide index	NetCTLpan MH	NetCTLpan tap	NetCTLpan clea	NetCTLpan com	NetCTLpan per	legacy mhc_pr	legacy tap_pre	legacy cleavag	legacy combine	legacy %_rank	equal mhc_pre	equal tap_pred	equal cleavage	equal combine	equal %_rank
1	MIFDNAFNC	151	159	9	HLA-A*02:01	151	0.434	0.312	0.06022	0.45535	6	0.434	0.312	0.06022	0.45535	6	TRUE	TRUE	TRUE	TRUE	TRUE
1	IFDNAFNCT	152	160	9	HLA-A*02:01	152	0.055	-0.655	0.05183	0.05029	50	0.055	-0.655	0.05183	0.05029	50	TRUE	TRUE	TRUE	TRUE	TRUE
1	FDNAFNCTF	153	161	9	HLA-A*02:01	153	0.051	2.047	0.71475	0.26299	32	0.051	2.047	0.71475	0.26299	32	TRUE	TRUE	TRUE	TRUE	TRUE
1	DNAFNCTFE	154	162	9	HLA-A*02:01	154	0.006	-2.122	0.025	-0.04142	50	0.006	-2.122	0.025	-0.04142	50	TRUE	TRUE	TRUE	TRUE	TRUE
1	NAFNCTFEY	155	163	9	HLA-A*02:01	155	0.104	2.987	0.95077	0.3926	8	0.104	2.987	0.95077	0.3926	8	TRUE	TRUE	TRUE	TRUE	TRUE
1	AFNCTFEYI	156	164	9	HLA-A*02:01	156	0.187	0.893	0.2775	0.27176	32	0.187	0.893	0.2775	0.27176	32	TRUE	TRUE	TRUE	TRUE	TRUE
1	FNCTFEYIS	157	165	9	HLA-A*02:01	157	0.021	-2.33	0.02433	-0.03178	50	0.021	-2.33	0.02433	-0.03178	50	TRUE	TRUE	TRUE	TRUE	TRUE
1	NCTFEYISD	158	166	9	HLA-A*02:01	158	0.007	-1.899	0.03351	-0.03294	50	0.007	-1.899	0.03351	-0.03294	50	TRUE	TRUE	TRUE	TRUE	TRUE
1	CTFEYISDA	159	167	9	HLA-A*02:01	159	0.312	-0.345	0.68018	0.45642	6	0.312	-0.345	0.68018	0.45642	6	TRUE	TRUE	TRUE	TRUE	TRUE
1	TFEYISDAF	160	168	9	HLA-A*02:01	160	0.042	2.53	0.42409	0.20067	32	0.042	2.53	0.42409	0.20067	32	TRUE	TRUE	TRUE	TRUE	TRUE
1	FEYISDAFS	161	169	9	HLA-A*02:01	161	0.034	-2.279	0.02449	-0.01746	50	0.034	-2.279	0.02449	-0.01746	50	TRUE	TRUE	TRUE	TRUE	TRUE
1	EYISDAFSL	162	170	9	HLA-A*02:01	162	0.061	0.979	0.9739	0.3046	16	0.061	0.979	0.9739	0.3046	16	TRUE	TRUE	TRUE	TRUE	TRUE
1	YISDAFSLD	163	171	9	HLA-A*02:01	163	0.107	-1.878	0.06776	0.0753	50	0.107	-1.878	0.06776	0.0753	50	TRUE	TRUE	TRUE	TRUE	TRUE
1	ISDAFSLDV	164	172	9	HLA-A*02:01	164	0.325	0.15	0.9316	0.53836	5	0.325	0.15	0.9316	0.53836	5	TRUE	TRUE	TRUE	TRUE	TRUE
1	SDAFSLDVS	165	173	9	HLA-A*02:01	165	0.014	-2.474	0.02762	-0.04163	50	0.014	-2.474	0.02762	-0.04163	50	TRUE	TRUE	TRUE	TRUE	TRUE
1	DAFSLDVSE	166	174	9	HLA-A*02:01	166	0.013	-1.663	0.03023	-0.02177	50	0.013	-1.663	0.03023	-0.02177	50	TRUE	TRUE	TRUE	TRUE	TRUE
1	AFSLDVSEK	167	175	9	HLA-A*02:01	167	0.018	0.633	0.90282	0.23696	32	0.018	0.633	0.90282	0.23696	32	TRUE	TRUE	TRUE	TRUE	TRUE
1	FSLDVSEKS	168	176	9	HLA-A*02:01	168	0.074	-2.128	0.04492	0.03091	50	0.074	-2.128	0.04492	0.03091	50	TRUE	TRUE	TRUE	TRUE	TRUE
1	SLDVSEKSG	169	177	9	HLA-A*02:01	169	0.035	-1.604	0.11646	0.0211	50	0.035	-1.604	0.11646	0.0211	50	TRUE	TRUE	TRUE	TRUE	TRUE
1	LDVSEKSGN	170	178	9	HLA-A*02:01	170	0.004	-1.487	0.02527	-0.02749	50	0.004	-1.487	0.02527	-0.02749	50	TRUE	TRUE	TRUE	TRUE	TRUE
1	DVSEKSGNF	171	179	9	HLA-A*02:01	171	0.019	2.401	0.66953	0.22967	32	0.019	2.401	0.66953	0.22967	32	TRUE	TRUE	TRUE	TRUE	TRUE
1	VSEKSGNFK	172	180	9	HLA-A*02:01	172	0.011	0.284	0.15922	0.05392	50	0.011	0.284	0.15922	0.05392	50	TRUE	TRUE	TRUE	TRUE	TRUE
1	SEKSGNFKH	173	181	9	HLA-A*02:01	173	0.009	-0.573	0.25036	0.05101	50	0.009	-0.573	0.25036	0.05101	50	TRUE	TRUE	TRUE	TRUE	TRUE
1	EKSGNFKHL	174	182	9	HLA-A*02:01	174	0.014	0.85	0.85005	0.22651	32	0.014	0.85	0.85005	0.22651	32	TRUE	TRUE	TRUE	TRUE	TRUE
1	KSGNFKHLR	175	183	9	HLA-A*02:01	175	0.031	1.302	0.22155	0.1134	50	0.031	1.302	0.22155	0.1134	50	TRUE	TRUE	TRUE	TRUE	TRUE
1	SGNFKHLRE	176	184	9	HLA-A*02:01	176	0.009	-1.772	0.02992	-0.02857	50	0.009	-1.772	0.02992	-0.02857	50	TRUE	TRUE	TRUE	TRUE	TRUE
1	GNFKHLREF	177	185	9	HLA-A*02:01	177	0.027	2.369	0.37683	0.17101	50	0.027	2.369	0.37683	0.17101	50	TRUE	TRUE	TRUE	TRUE	TRUE
1	NFKHLREFV	178	186	9	HLA-A*02:01	178	0.047	0.487	0.09425	0.08038	50	0.047	0.487	0.09425	0.08038	50	TRUE	TRUE	TRUE	TRUE	TRUE
1	FKHLREFVF	179	187	9	HLA-A*02:01	179	0.027	2.66	0.55084	0.21744	32	0.027	2.66	0.55084	0.21744	32	TRUE	TRUE	TRUE	TRUE	TRUE
1	KHLREFVFK	180	188	9	HLA-A*02:01	180	0.026	0.492	0.71141	0.19837	50	0.026	0.492	0.71141	0.19837	50	TRUE	TRUE	TRUE	TRUE	TRUE
1	HLREFVFKN	181	189	9	HLA-A*02:01	181	0.09	-1.237	0.06685	0.07412	50	0.09	-1.237	0.06685	0.07412	50	TRUE	TRUE	TRUE	TRUE	TRUE
1	LREFVFKNK	182	190	9	HLA-A*02:01	182	0.01	0.508	0.5457	0.14548	50	0.01	0.508	0.5457	0.14548	50	TRUE	TRUE	TRUE	TRUE	TRUE
1	REFVFKNKD	183	191	9	HLA-A*02:01	183	0.012	-1.547	0.03765	-0.0182	50	0.012	-1.547	0.03765	-0.0182	50	TRUE	TRUE	TRUE	TRUE	TRUE
1	EFVFKNKDG	184	192	9	HLA-A*02:01	184	0.007	-1.222	0.02365	-0.01823	50	0.007	-1.222	0.02365	-0.01823	50	TRUE	TRUE	TRUE	TRUE	TRUE
1	FVFKNKDGF	185	193	9	HLA-A*02:01	185	0.092	2.523	0.09857	0.17725	50	0.092	2.523	0.09857	0.17725	50	TRUE	TRUE	TRUE	TRUE	TRUE
1	VFKNKDGFL	186	194	9	HLA-A*02:01	186	0.037	1.077	0.4415	0.16326	50	0.037	1.077	0.4415	0.16326	50	TRUE	TRUE	TRUE	TRUE	TRUE
1	FKNKDGFLY	187	195	9	HLA-A*02:01	187	0.031	2.832	0.53022	0.2211	32	0.031	2.832	0.53022	0.2211	32	TRUE	TRUE	TRUE	TRUE	TRUE
1	KNKDGFLYV	188	196	9	HLA-A*02:01	188	0.124	0.162	0.91886	0.33479	16	0.124	0.162	0.91886	0.33479	16	TRUE	TRUE	TRUE	TRUE	TRUE
1	NKDGFLYVY	189	197	9	HLA-A*02:01	189	0.045	2.936	0.97652	0.33812	16	0.045	2.936	0.97652	0.33812	16	TRUE	TRUE	TRUE	TRUE	TRUE
1	KDGFLYVYK	190	198	9	HLA-A*02:01	190	0.024	0.242	0.56138	0.15636	50	0.024	0.242	0.56138	0.15636	50	TRUE	TRUE	TRUE	TRUE	TRUE
1	DGFLYVYKG	191	199	9	HLA-A*02:01	191	0.017	-1.765	0.11105	-0.00214	50	0.017	-1.765	0.11105	-0.00214	50	TRUE	TRUE	TRUE	TRUE	TRUE
1	GFLYVYKGY	192	200	9	HLA-A*02:01	192	0.03	2.792	0.28048	0.16291	50	0.03	2.792	0.28048	0.16291	50	TRUE	TRUE	TRUE	TRUE	TRUE
1	FLYVYKGYQ	193	201	9	HLA-A*02:01	193	0.221	-0.024	0.03004	0.22716	32	0.221	-0.024	0.03004	0.22716	32	TRUE	TRUE	TRUE	TRUE	TRUE
1	LYVYKGYQP	194	202	9	HLA-A*02:01	194	0.014	0.346	0.0281	0.02897	50	0.014	0.346	0.0281	0.02897	50	TRUE	TRUE	TRUE	TRUE	TRUE
1	YVYKGYQPI	195	203	9	HLA-A*02:01	195	0.689	0.888	0.94815	0.92453	0.8	0.689	0.888	0.94815	0.92453	0.8	TRUE	TRUE	TRUE	TRUE	TRUE
1	VYKGYQPID	196	204	9	HLA-A*02:01	196	0.008	-1.543	0.05814	-0.01749	50	0.008	-1.543	0.05814	-0.01749	50	TRUE	TRUE	TRUE	TRUE	TRUE
1	YKGYQPIDV	197	205	9	HLA-A*02:01	197	0.044	0.228	0.57307	0.17864	50	0.044	0.228	0.57307	0.17864	50	TRUE	TRUE	TRUE	TRUE	TRUE
1	KGYQPIDVV	198	206	9	HLA-A*02:01	198	0.138	0.29	0.6634	0.29452	16	0.138	0.29	0.6634	0.29452	16	TRUE	TRUE	TRUE	TRUE	TRUE
1	GYQPIDVVR	199	207	9	HLA-A*02:01	199	0.022	1.639	0.95268	0.27733	32	0.022	1.639	0.95268	0.27733	32	TRUE	TRUE	TRUE	TRUE	TRUE
1	YQPIDVVRD	200	208	9	HLA-A*02:01	200	0.026	-1.805	0.04389	-0.00925	50	0.026	-1.805	0.04389	-0.00925	50	TRUE	TRUE	TRUE	TRUE	TRUE
1	QPIDVVRDL	201	209	9	HLA-A*02:01	201	0.035	0.68	0.92822	0.26085	32	0.035	0.68	0.92822	0.26085	32	TRUE	TRUE	TRUE	TRUE	TRUE
1	PIDVVRDLP	202	210	9	HLA-A*02:01	202	0.008	-0.472	0.02605	0.00206	50	0.008	-0.472	0.02605	0.00206	50	TRUE	TRUE	TRUE	TRUE	TRUE
1	IDVVRDLPS	203	211	9	HLA-A*02:01	203	0.012	-2.597	0.03213	-0.0457	50	0.012	-2.597	0.03213	-0.0457	50	TRUE	TRUE	TRUE	TRUE	TRUE
1	DVVRDLPSG	204	212	9	HLA-A*02:01	204	0.015	-1.486	0.05068	-0.01075	50	0.015	-1.486	0.05068	-0.01075	50	TRUE	TRUE	TRUE	TRUE	TRUE
1	VVRDLPSGF	205	213	9	HLA-A*02:01	205	0.053	2.645	0.5709	0.24758	32	0.053	2.645	0.5709	0.24758	32	TRUE	TRUE	TRUE	TRUE	TRUE
1	VRDLPSGFN	206	214	9	HLA-A*02:01	206	0.006	-1.176	0.06495	-0.00879	50	0.006	-1.176	0.06495	-0.00879	50	TRUE	TRUE	TRUE	TRUE	TRUE
1	RDLPSGFNT	207	215	9	HLA-A*02:01	207	0.026	-0.704	0.04171	0.01778	50	0.026	-0.704	0.04171	0.01778	50	TRUE	TRUE	TRUE	TRUE	TRUE
1	DLPSGFNTL	208	216	9	HLA-A*02:01	208	0.182	0.775	0.9754	0.42084	7	0.182	0.775	0.9754	0.42084	7	TRUE	TRUE	TRUE	TRUE	TRUE
1	LPSGFNTLK	209	217	9	HLA-A*02:01	209	0.017	0.021	0.95236	0.23181	32	0.017	0.021	0.95236	0.23181	32	TRUE	TRUE	TRUE	TRUE	TRUE
1	PSGFNTLKP	210	218	9	HLA-A*02:01	210	0.004	-0.45	0.1228	0.02038	50	0.004	-0.45	0.1228	0.02038	50	TRUE	TRUE	TRUE	TRUE	TRUE
1	SGFNTLKPI	211	219	9	HLA-A*02:01	211	0.114	0.288	0.54186	0.24312	32	0.114	0.288	0.54186	0.24312	32	TRUE	TRUE	TRUE	TRUE	TRUE
1	GFNTLKPIF	212	220	9	HLA-A*02:01	212	0.022	2.483	0.60391	0.21995	32	0.022	2.483	0.60391	0.21995	32	TRUE	TRUE	TRUE	TRUE	TRUE
1	FNTLKPIFK	213	221	9	HLA-A*02:01	213	0.017	0.151	0.09149	0.04136	50	0.017	0.151	0.09149	0.04136	50	TRUE	TRUE	TRUE	TRUE	TRUE
1	NTLKPIFKL	214	222	9	HLA-A*02:01	214	0.284	0.883	0.94317	0.51829	5	0.284	0.883	0.94317	0.51829	5	TRUE	TRUE	TRUE	TRUE	TRUE
1	TLKPIFKLP	215	223	9	HLA-A*02:01	215	0.039	0.242	0.03772	0.05354	50	0.039	0.242	0.03772	0.05354	50	TRUE	TRUE	TRUE	TRUE	TRUE
1	LKPIFKLPL	216	224	9	HLA-A*02:01	216	0.041	1.003	0.46871	0.17153	50	0.041	1.003	0.46871	0.17153	50	TRUE	TRUE	TRUE	TRUE	TRUE
1	KPIFKLPLG	217	225	9	HLA-A*02:01	217	0.018	-1.476	0.03069	-0.01199	50	0.018	-1.476	0.03069	-0.01199	50	TRUE	TRUE	TRUE	TRUE	TRUE
1	PIFKLPLGI	218	226	9	HLA-A*02:01	218	0.184	0.396	0.36307	0.27559	32	0.184	0.396	0.36307	0.27559	32	TRUE	TRUE	TRUE	TRUE	TRUE
1	IFKLPLGIN	219	227	9	HLA-A*02:01	219	0.014	-1.272	0.0253	-0.01211	50	0.014	-1.272	0.0253	-0.01211	50	TRUE	TRUE	TRUE	TRUE	TRUE
1	FKLPLGINI	220	228	9	HLA-A*02:01	220	0.102	0.516	0.0693	0.13049	50	0.102	0.516	0.0693	0.13049	50	TRUE	TRUE	TRUE	TRUE	TRUE
1	KLPLGINIT	221	229	9	HLA-A*02:01	221	0.231	-0.699	0.52644	0.33197	16	0.231	-0.699	0.52644	0.33197	16	TRUE	TRUE	TRUE	TRUE	TRUE
1	LPLGINITN	222	230	9	HLA-A*02:01	222	0.017	-1.497	0.17996	0.02007	50	0.017	-1.497	0.17996	0.02007	50	TRUE	TRUE	TRUE	TRUE	TRUE
1	PLGINITNF	223	231	9	HLA-A*02:01	223	0.041	1.959	0.81243	0.27277	32	0.041	1.959	0.81243	0.27277	32	TRUE	TRUE	TRUE	TRUE	TRUE
1	LGINITNFR	224	232	9	HLA-A*02:01	224	0.03	1.146	0.23377	0.11125	50	0.03	1.146	0.23377	0.11125	50	TRUE	TRUE	TRUE	TRUE	TRUE
1	GINITNFRA	225	233	9	HLA-A*02:01	225	0.184	-0.632	0.55472	0.29301	16	0.184	-0.632	0.55472	0.29301	16	TRUE	TRUE	TRUE	TRUE	TRUE
1	INITNFRAI	226	234	9	HLA-A*02:01	226	0.049	0.444	0.11321	0.08557	50	0.049	0.444	0.11321	0.08557	50	TRUE	TRUE	TRUE	TRUE	TRUE
1	NITNFRAIL	227	235	9	HLA-A*02:01	227	0.149	1.093	0.88031	0.3744	9	0.149	1.093	0.88031	0.3744	9	TRUE	TRUE	TRUE	TRUE	TRUE
1	ITNFRAILT	228	236	9	HLA-A*02:01	228	0.087	-0.657	0.12358	0.09838	50	0.087	-0.657	0.12358	0.09838	50	TRUE	TRUE	TRUE	TRUE	TRUE
1	TNFRAILTA	229	237	9	HLA-A*02:01	229	0.071	-0.588	0.47081	0.16223	50	0.071	-0.588	0.47081	0.16223	50	TRUE	TRUE	TRUE	TRUE	TRUE
1	NFRAILTAF	230	238	9	HLA-A*02:01	230	0.049	2.798	0.85893	0.31221	16	0.049	2.798	0.85893	0.31221	16	TRUE	TRUE	TRUE	TRUE	TRUE
1	FRAILTAFS	231	239	9	HLA-A*02:01	231	0.035	-1.976	0.02738	-0.00824	50	0.035	-1.976	0.02738	-0.00824	50	TRUE	TRUE	TRUE	TRUE	TRUE
1	RAILTAFSP	232	240	9	HLA-A*02:01	232	0.046	0.365	0.04536	0.06533	50	0.046	0.365	0.04536	0.06533	50	TRUE	TRUE	TRUE	TRUE	TRUE
1	AILTAFSPA	233	241	9	HLA-A*02:01	233	0.428	-0.113	0.19668	0.46943	6	0.428	-0.113	0.19668	0.46943	6	TRUE	TRUE	TRUE	TRUE	TRUE
1	ILTAFSPAQ	234	242	9	HLA-A*02:01	234	0.164	0.055	0.35834	0.246	32	0.164	0.055	0.35834	0.246	32	TRUE	TRUE	TRUE	TRUE	TRUE
1	LTAFSPAQD	235	243	9	HLA-A*02:01	235	0.023	-1.807	0.02417	-0.01674	50	0.023	-1.807	0.02417	-0.01674	50	TRUE	TRUE	TRUE	TRUE	TRUE
1	TAFSPAQDI	236	244	9	HLA-A*02:01	236	0.053	0.726	0.52973	0.19034	50	0.053	0.726	0.52973	0.19034	50	TRUE	TRUE	TRUE	TRUE	TRUE
1	AFSPAQDIW	237	245	9	HLA-A*02:01	237	0.029	1.202	0.56093	0.18526	50	0.029	1.202	0.56093	0.18526	50	TRUE	TRUE	TRUE	TRUE	TRUE
1	FSPAQDIWG	238	246	9	HLA-A*02:01	238	0.031	-1.311	0.02341	0.00349	50	0.031	-1.311	0.02341	0.00349	50	TRUE	TRUE	TRUE	TRUE	TRUE
1	SPAQDIWGT	239	247	9	HLA-A*02:01	239	0.031	-1.045	0.13937	0.03623	50	0.031	-1.045	0.13937	0.03623	50	TRUE	TRUE	TRUE	TRUE	TRUE
1	PAQDIWGTS	240	248	9	HLA-A*02:01	240	0.013	-2.651	0.06202	-0.03932	50	0.013	-2.651	0.06202	-0.03932	50	TRUE	TRUE	TRUE	TRUE	TRUE
1	AQDIWGTSA	241	249	9	HLA-A*02:01	241	0.304	-0.637	0.36414	0.37001	9	0.304	-0.637	0.36414	0.37001	9	TRUE	TRUE	TRUE	TRUE	TRUE
1	QDIWGTSAA	242	250	9	HLA-A*02:01	242	0.035	-0.607	0.05828	0.03294	50	0.035	-0.607	0.05828	0.03294	50	TRUE	TRUE	TRUE	TRUE	TRUE
1	DIWGTSAAA	243	251	9	HLA-A*02:01	243	0.142	-0.6	0.79276	0.30537	16	0.142	-0.6	0.79276	0.30537	16	TRUE	TRUE	TRUE	TRUE	TRUE
1	IWGTSAAAY	244	252	9	HLA-A*02:01	244	0.024	2.892	0.50022	0.20885	32	0.024	2.892	0.50022	0.20885	32	TRUE	TRUE	TRUE	TRUE	TRUE
1	WGTSAAAYF	245	253	9	HLA-A*02:01	245	0.048	2.214	0.25804	0.16141	50	0.048	2.214	0.25804	0.16141	50	TRUE	TRUE	TRUE	TRUE	TRUE
1	GTSAAAYFV	246	254	9	HLA-A*02:01	246	0.537	0.054	0.23494	0.59121	4	0.537	0.054	0.23494	0.59121	4	TRUE	TRUE	TRUE	TRUE	TRUE
1	TSAAAYFVG	247	255	9	HLA-A*02:01	247	0.032	-1.389	0.03359	0.00483	50	0.032	-1.389	0.03359	0.00483	50	TRUE	TRUE	TRUE	TRUE	TRUE
1	SAAAYFVGY	248	256	9	HLA-A*02:01	248	0.12	3.057	0.92326	0.40416	8	0.12	3.057	0.92326	0.40416	8	TRUE	TRUE	TRUE	TRUE	TRUE
1	AAAYFVGYL	249	257	9	HLA-A*02:01	249	0.292	1.222	0.50674	0.43657	7	0.292	1.222	0.50674	0.43657	7	TRUE	TRUE	TRUE	TRUE	TRUE
1	AAYFVGYLK	250	258	9	HLA-A*02:01	250	0.07	1.002	0.56644	0.2225	32	0.07	1.002	0.56644	0.2225	32	TRUE	TRUE	TRUE	TRUE	TRUE
1	AYFVGYLKP	251	259	9	HLA-A*02:01	251	0.016	0.779	0.0702	0.05127	50	0.016	0.779	0.0702	0.05127	50	TRUE	TRUE	TRUE	TRUE	TRUE
1	YFVGYLKPT	252	260	9	HLA-A*02:01	252	0.149	-0.326	0.03615	0.14898	50	0.149	-0.326	0.03615	0.14898	50	TRUE	TRUE	TRUE	TRUE	TRUE
1	FVGYLKPTT	253	261	9	HLA-A*02:01	253	0.107	-0.877	0.02561	0.09084	50	0.107	-0.877	0.02561	0.09084	50	TRUE	TRUE	TRUE	TRUE	TRUE
1	VGYLKPTTF	254	262	9	HLA-A*02:01	254	0.043	2.412	0.958	0.31885	16	0.043	2.412	0.958	0.31885	16	TRUE	TRUE	TRUE	TRUE	TRUE
1	GYLKPTTFM	255	263	9	HLA-A*02:01	255	0.054	0.455	0.95964	0.28129	32	0.054	0.455	0.95964	0.28129	32	TRUE	TRUE	TRUE	TRUE	TRUE
1	YLKPTTFML	256	264	9	HLA-A*02:01	256	0.798	0.922	0.97881	1.04128	0.15	0.798	0.922	0.97881	1.04128	0.15	TRUE	TRUE	TRUE	TRUE	TRUE
1	LKPTTFMLK	257	265	9	HLA-A*02:01	257	0.017	0.451	0.70593	0.18711	50	0.017	0.451	0.70593	0.18711	50	TRUE	TRUE	TRUE	TRUE	TRUE
1	KPTTFMLKY	258	266	9	HLA-A*02:01	258	0.028	2.739	0.97432	0.3157	16	0.028	2.739	0.97432	0.3157	16	TRUE	TRUE	TRUE	TRUE	TRUE
1	PTTFMLKYD	259	267	9	HLA-A*02:01	259	0.007	-2.252	0.02875	-0.04283	50	0.007	-2.252	0.02875	-0.04283	50	TRUE	TRUE	TRUE	TRUE	TRUE
1	TTFMLKYDE	260	268	9	HLA-A*02:01	260	0.017	-1.641	0.02334	-0.01877	50	0.017	-1.641	0.02334	-0.01877	50	TRUE	TRUE	TRUE	TRUE	TRUE
1	TFMLKYDEN	261	269	9	HLA-A*02:01	261	0.016	-1.094	0.02319	-0.00613	50	0.016	-1.094	0.02319	-0.00613	50	TRUE	TRUE	TRUE	TRUE	TRUE
1	FMLKYDENG	262	270	9	HLA-A*02:01	262	0.248	-1.341	0.02705	0.22056	32	0.248	-1.341	0.02705	0.22056	32	TRUE	TRUE	TRUE	TRUE	TRUE
1	MLKYDENGT	263	271	9	HLA-A*02:01	263	0.086	-0.746	0.0276	0.07356	50	0.086	-0.746	0.0276	0.07356	50	TRUE	TRUE	TRUE	TRUE	TRUE
1	LKYDENGTI	264	272	9	HLA-A*02:01	264	0.048	0.811	0.52123	0.18555	50	0.048	0.811	0.52123	0.18555	50	TRUE	TRUE	TRUE	TRUE	TRUE
1	KYDENGTIT	265	273	9	HLA-A*02:01	265	0.029	-0.462	0.51518	0.13336	50	0.029	-0.462	0.51518	0.13336	50	TRUE	TRUE	TRUE	TRUE	TRUE
1	YDENGTITD	266	274	9	HLA-A*02:01	266	0.009	-2.186	0.29422	0.02055	50	0.009	-2.186	0.29422	0.02055	50	TRUE	TRUE	TRUE	TRUE	TRUE
1	DENGTITDA	267	275	9	HLA-A*02:01	267	0.013	-1.133	0.80714	0.16628	50	0.013	-1.133	0.80714	0.16628	50	TRUE	TRUE	TRUE	TRUE	TRUE
1	ENGTITDAV	268	276	9	HLA-A*02:01	268	0.027	-0.344	0.76772	0.19114	50	0.027	-0.344	0.76772	0.19114	50	TRUE	TRUE	TRUE	TRUE	TRUE
1	NGTITDAVD	269	277	9	HLA-A*02:01	269	0.005	-2.248	0.03162	-0.04409	50	0.005	-2.248	0.03162	-0.04409	50	TRUE	TRUE	TRUE	TRUE	TRUE
1	GTITDAVDC	270	278	9	HLA-A*02:01	270	0.043	0.024	0.03083	0.05054	50	0.043	0.024	0.03083	0.05054	50	TRUE	TRUE	TRUE	TRUE	TRUE
1	TITDAVDCS	271	279	9	HLA-A*02:01	271	0.028	-2.204	0.03498	-0.01923	50	0.028	-2.204	0.03498	-0.01923	50	TRUE	TRUE	TRUE	TRUE	TRUE
1	ITDAVDCSQ	272	280	9	HLA-A*02:01	272	0.036	-0.27	0.04763	0.03997	50	0.036	-0.27	0.04763	0.03997	50	TRUE	TRUE	TRUE	TRUE	TRUE
1	TDAVDCSQN	273	281	9	HLA-A*02:01	273	0.005	-1.618	0.02743	-0.02928	50	0.005	-1.618	0.02743	-0.02928	50	TRUE	TRUE	TRUE	TRUE	TRUE
1	DAVDCSQNP	274	282	9	HLA-A*02:01	274	0.008	-0.071	0.03611	0.01435	50	0.008	-0.071	0.03611	0.01435	50	TRUE	TRUE	TRUE	TRUE	TRUE
1	AVDCSQNPL	275	283	9	HLA-A*02:01	275	0.287	0.922	0.94996	0.52379	5	0.287	0.922	0.94996	0.52379	5	TRUE	TRUE	TRUE	TRUE	TRUE
1	VDCSQNPLA	276	284	9	HLA-A*02:01	276	0.018	-0.618	0.07771	0.02003	50	0.018	-0.618	0.07771	0.02003	50	TRUE	TRUE	TRUE	TRUE	TRUE
1	DCSQNPLAE	277	285	9	HLA-A*02:01	277	0.005	-1.818	0.02582	-0.03464	50	0.005	-1.818	0.02582	-0.03464	50	TRUE	TRUE	TRUE	TRUE	TRUE
1	CSQNPLAEL	278	286	9	HLA-A*02:01	278	0.111	0.783	0.95509	0.34547	10	0.111	0.783	0.95509	0.34547	10	TRUE	TRUE	TRUE	TRUE	TRUE
1	SQNPLAELK	279	287	9	HLA-A*02:01	279	0.045	0.492	0.59737	0.19171	50	0.045	0.492	0.59737	0.19171	50	TRUE	TRUE	TRUE	TRUE	TRUE
1	QNPLAELKC	280	288	9	HLA-A*02:01	280	0.011	-0.086	0.02746	0.01503	50	0.011	-0.086	0.02746	0.01503	50	TRUE	TRUE	TRUE	TRUE	TRUE
1	NPLAELKCS	281	289	9	HLA-A*02:01	281	0.009	-2.544	0.03035	-0.04777	50	0.009	-2.544	0.03035	-0.04777	50	TRUE	TRUE	TRUE	TRUE	TRUE
1	PLAELKCSV	282	290	9	HLA-A*02:01	282	0.39	0.012	0.80365	0.57112	4	0.39	0.012	0.80365	0.57112	4	TRUE	TRUE	TRUE	TRUE	TRUE
1	LAELKCSVK	283	291	9	HLA-A*02:01	283	0.012	0.232	0.8711	0.2138	32	0.012	0.232	0.8711	0.2138	32	TRUE	TRUE	TRUE	TRUE	TRUE
1	AELKCSVKS	284	292	9	HLA-A*02:01	284	0.023	-2.171	0.03288	-0.02388	50	0.023	-2.171	0.03288	-0.02388	50	TRUE	TRUE	TRUE	TRUE	TRUE
1	ELKCSVKSF	285	293	9	HLA-A*02:01	285	0.038	2.502	0.92997	0.30979	16	0.038	2.502	0.92997	0.30979	16	TRUE	TRUE	TRUE	TRUE	TRUE
1	LKCSVKSFE	286	294	9	HLA-A*02:01	286	0.005	-1.67	0.02264	-0.03166	50	0.005	-1.67	0.02264	-0.03166	50	TRUE	TRUE	TRUE	TRUE	TRUE
1	KCSVKSFEI	287	295	9	HLA-A*02:01	287	0.085	0.706	0.32416	0.17559	50	0.085	0.706	0.32416	0.17559	50	TRUE	TRUE	TRUE	TRUE	TRUE
1	CSVKSFEID	288	296	9	HLA-A*02:01	288	0.014	-1.637	0.0247	-0.02137	50	0.014	-1.637	0.0247	-0.02137	50	TRUE	TRUE	TRUE	TRUE	TRUE
1	SVKSFEIDK	289	297	9	HLA-A*02:01	289	0.016	0.647	0.69544	0.18865	50	0.016	0.647	0.69544	0.18865	50	TRUE	TRUE	TRUE	TRUE	TRUE
1	VKSFEIDKG	290	298	9	HLA-A*02:01	290	0.005	-1.234	0.02397	-0.02046	50	0.005	-1.234	0.02397	-0.02046	50	TRUE	TRUE	TRUE	TRUE	TRUE
1	KSFEIDKGI	291	299	9	HLA-A*02:01	291	0.183	0.841	0.48569	0.31331	16	0.183	0.841	0.48569	0.31331	16	TRUE	TRUE	TRUE	TRUE	TRUE
1	SFEIDKGIY	292	300	9	HLA-A*02:01	292	0.018	3.102	0.81372	0.27864	32	0.018	3.102	0.81372	0.27864	32	TRUE	TRUE	TRUE	TRUE	TRUE
1	FEIDKGIYQ	293	301	9	HLA-A*02:01	293	0.03	-0.213	0.04119	0.03394	50	0.03	-0.213	0.04119	0.03394	50	TRUE	TRUE	TRUE	TRUE	TRUE
1	EIDKGIYQT	294	302	9	HLA-A*02:01	294	0.089	-1.062	0.27684	0.12474	50	0.089	-1.062	0.27684	0.12474	50	TRUE	TRUE	TRUE	TRUE	TRUE
1	IDKGIYQTS	295	303	9	HLA-A*02:01	295	0.013	-2.699	0.08906	-0.03444	50	0.013	-2.699	0.08906	-0.03444	50	TRUE	TRUE	TRUE	TRUE	TRUE
1	DKGIYQTSN	296	304	9	HLA-A*02:01	296	0.004	-1.736	0.06089	-0.0257	50	0.004	-1.736	0.06089	-0.0257	50	TRUE	TRUE	TRUE	TRUE	TRUE
1	KGIYQTSNF	297	305	9	HLA-A*02:01	297	0.038	2.305	0.51733	0.21202	32	0.038	2.305	0.51733	0.21202	32	TRUE	TRUE	TRUE	TRUE	TRUE
1	GIYQTSNFR	298	306	9	HLA-A*02:01	298	0.037	1.722	0.52603	0.19841	50	0.037	1.722	0.52603	0.19841	50	TRUE	TRUE	TRUE	TRUE	TRUE
1	IYQTSNFRV	299	307	9	HLA-A*02:01	299	0.098	0.54	0.85829	0.30462	16	0.098	0.54	0.85829	0.30462	16	TRUE	TRUE	TRUE	TRUE	TRUE
1	YQTSNFRVV	300	308	9	HLA-A*02:01	300	0.37	0.343	0.13536	0.40903	8	0.37	0.343	0.13536	0.40903	8	TRUE	TRUE	TRUE	TRUE	TRUE

seq #	peptide	start	end	peptide length	allele	peptide index	NetCTLpan MH	NetCTLpan tap	NetCTLpan clea	NetCTLpan con	NetCTLpan per	legacy mhc_pr	legacy tap_pre	legacy cleavag	legacy combine	legacy %_rank	equal mhc_pre	equal tap_pred	equal cleavage	equal combine	equal %_rank
1	QTSNFRVVP	301	309	9	HLA-A*02:01	301	0.015	0.04	0.07347	0.03253	50	0.015	0.04	0.07347	0.03253	50	TRUE	TRUE	TRUE	TRUE	TRUE
1	TSNFRVVPS	302	310	9	HLA-A*02:01	302	0.043	-2.369	0.02998	-0.00948	50	0.043	-2.369	0.02998	-0.00948	50	TRUE	TRUE	TRUE	TRUE	TRUE
1	SNFRVVPSG	303	311	9	HLA-A*02:01	303	0.023	-1.373	0.09544	0.01015	50	0.023	-1.373	0.09544	0.01015	50	TRUE	TRUE	TRUE	TRUE	TRUE
1	NFRVVPSGD	304	312	9	HLA-A*02:01	304	0.01	-1.508	0.02338	-0.02244	50	0.01	-1.508	0.02338	-0.02244	50	TRUE	TRUE	TRUE	TRUE	TRUE
1	FRVVPSGDV	305	313	9	HLA-A*02:01	305	0.032	0.645	0.83755	0.23657	32	0.032	0.645	0.83755	0.23657	32	TRUE	TRUE	TRUE	TRUE	TRUE
1	RVVPSGDVV	306	314	9	HLA-A*02:01	306	0.195	0.67	0.62651	0.35271	10	0.195	0.67	0.62651	0.35271	10	TRUE	TRUE	TRUE	TRUE	TRUE
1	VVPSGDVVR	307	315	9	HLA-A*02:01	307	0.023	1.802	0.20037	0.11313	50	0.023	1.802	0.20037	0.11313	50	TRUE	TRUE	TRUE	TRUE	TRUE
1	VPSGDVVRF	308	316	9	HLA-A*02:01	308	0.029	2.335	0.92743	0.29605	16	0.029	2.335	0.92743	0.29605	16	TRUE	TRUE	TRUE	TRUE	TRUE
1	PSGDVVRFP	309	317	9	HLA-A*02:01	309	0.005	-0.451	0.0254	-0.00056	50	0.005	-0.451	0.0254	-0.00056	50	TRUE	TRUE	TRUE	TRUE	TRUE
1	SGDVVRFPN	310	318	9	HLA-A*02:01	310	0.017	-1.85	0.03377	-0.02165	50	0.017	-1.85	0.03377	-0.02165	50	TRUE	TRUE	TRUE	TRUE	TRUE
1	GDVVRFPNI	311	319	9	HLA-A*02:01	311	0.03	0.18	0.25596	0.09209	50	0.03	0.18	0.25596	0.09209	50	TRUE	TRUE	TRUE	TRUE	TRUE
1	DVVRFPNIT	312	320	9	HLA-A*02:01	312	0.023	-0.835	0.40368	0.09295	50	0.023	-0.835	0.40368	0.09295	50	TRUE	TRUE	TRUE	TRUE	TRUE
1	VVRFPNITN	313	321	9	HLA-A*02:01	313	0.021	-1.205	0.06777	0.00612	50	0.021	-1.205	0.06777	0.00612	50	TRUE	TRUE	TRUE	TRUE	TRUE
1	VRFPNITNL	314	322	9	HLA-A*02:01	314	0.069	1.382	0.97634	0.32323	16	0.069	1.382	0.97634	0.32323	16	TRUE	TRUE	TRUE	TRUE	TRUE
1	RFPNITNLC	315	323	9	HLA-A*02:01	315	0.052	0.458	0.04346	0.07323	50	0.052	0.458	0.04346	0.07323	50	TRUE	TRUE	TRUE	TRUE	TRUE
1	FPNITNLCP	316	324	9	HLA-A*02:01	316	0.015	-0.257	0.04532	0.01877	50	0.015	-0.257	0.04532	0.01877	50	TRUE	TRUE	TRUE	TRUE	TRUE
1	PNITNLCPF	317	325	9	HLA-A*02:01	317	0.015	1.866	0.16158	0.09801	50	0.015	1.866	0.16158	0.09801	50	TRUE	TRUE	TRUE	TRUE	TRUE
1	NITNLCPFG	318	326	9	HLA-A*02:01	318	0.032	-1.411	0.02474	0.00229	50	0.032	-1.411	0.02474	0.00229	50	TRUE	TRUE	TRUE	TRUE	TRUE
1	ITNLCPFGE	319	327	9	HLA-A*02:01	319	0.042	-1.517	0.02261	0.00916	50	0.042	-1.517	0.02261	0.00916	50	TRUE	TRUE	TRUE	TRUE	TRUE
1	TNLCPFGEV	320	328	9	HLA-A*02:01	320	0.092	0.168	0.69009	0.25147	32	0.092	0.168	0.69009	0.25147	32	TRUE	TRUE	TRUE	TRUE	TRUE
1	NLCPFGEVF	321	329	9	HLA-A*02:01	321	0.115	2.61	0.91412	0.38593	8	0.115	2.61	0.91412	0.38593	8	TRUE	TRUE	TRUE	TRUE	TRUE
1	LCPFGEVFN	322	330	9	HLA-A*02:01	322	0.009	-1.165	0.02816	-0.01379	50	0.009	-1.165	0.02816	-0.01379	50	TRUE	TRUE	TRUE	TRUE	TRUE
1	CPFGEVFNA	323	331	9	HLA-A*02:01	323	0.079	-0.721	0.46119	0.16474	50	0.079	-0.721	0.46119	0.16474	50	TRUE	TRUE	TRUE	TRUE	TRUE
1	PFGEVFNAT	324	332	9	HLA-A*02:01	324	0.024	-1.188	0.05843	0.00745	50	0.024	-1.188	0.05843	0.00745	50	TRUE	TRUE	TRUE	TRUE	TRUE
1	FGEVFNATK	325	333	9	HLA-A*02:01	325	0.025	-0.186	0.35254	0.09967	50	0.025	-0.186	0.35254	0.09967	50	TRUE	TRUE	TRUE	TRUE	TRUE
1	GEVFNATKF	326	334	9	HLA-A*02:01	326	0.02	2.193	0.62254	0.2149	32	0.02	2.193	0.62254	0.2149	32	TRUE	TRUE	TRUE	TRUE	TRUE
1	EVFNATKFP	327	335	9	HLA-A*02:01	327	0.014	0.121	0.02571	0.02281	50	0.014	0.121	0.02571	0.02281	50	TRUE	TRUE	TRUE	TRUE	TRUE
1	VFNATKFPS	328	336	9	HLA-A*02:01	328	0.03	-2.277	0.02306	-0.02174	50	0.03	-2.277	0.02306	-0.02174	50	TRUE	TRUE	TRUE	TRUE	TRUE
1	FNATKFPSV	329	337	9	HLA-A*02:01	329	0.257	0.131	0.52617	0.37866	9	0.257	0.131	0.52617	0.37866	9	TRUE	TRUE	TRUE	TRUE	TRUE
1	NATKFPSVY	330	338	9	HLA-A*02:01	330	0.033	2.873	0.95409	0.3195	16	0.033	2.873	0.95409	0.3195	16	TRUE	TRUE	TRUE	TRUE	TRUE
1	ATKFPSVYA	331	339	9	HLA-A*02:01	331	0.112	-0.274	0.71844	0.2668	32	0.112	-0.274	0.71844	0.2668	32	TRUE	TRUE	TRUE	TRUE	TRUE
1	TKFPSVYAW	332	340	9	HLA-A*02:01	332	0.036	1.179	0.97035	0.2838	32	0.036	1.179	0.97035	0.2838	32	TRUE	TRUE	TRUE	TRUE	TRUE
1	KFPSVYAWE	333	341	9	HLA-A*02:01	333	0.034	-1.296	0.02398	0.007	50	0.034	-1.296	0.02398	0.007	50	TRUE	TRUE	TRUE	TRUE	TRUE
1	FPSVYAWER	334	342	9	HLA-A*02:01	334	0.038	1.182	0.68481	0.22163	32	0.038	1.182	0.68481	0.22163	32	TRUE	TRUE	TRUE	TRUE	TRUE
1	PSVYAWERK	335	343	9	HLA-A*02:01	335	0.009	-0.002	0.17185	0.04762	50	0.009	-0.002	0.17185	0.04762	50	TRUE	TRUE	TRUE	TRUE	TRUE
1	SVYAWERKK	336	344	9	HLA-A*02:01	336	0.034	0.698	0.43242	0.14875	50	0.034	0.698	0.43242	0.14875	50	TRUE	TRUE	TRUE	TRUE	TRUE
1	VYAWERKKI	337	345	9	HLA-A*02:01	337	0.022	0.932	0.35939	0.12616	50	0.022	0.932	0.35939	0.12616	50	TRUE	TRUE	TRUE	TRUE	TRUE
1	YAWERKKIS	338	346	9	HLA-A*02:01	338	0.035	-1.994	0.05013	-0.00357	50	0.035	-1.994	0.05013	-0.00357	50	TRUE	TRUE	TRUE	TRUE	TRUE
1	AWERKKISN	339	347	9	HLA-A*02:01	339	0.006	-1.121	0.02714	-0.01592	50	0.006	-1.121	0.02714	-0.01592	50	TRUE	TRUE	TRUE	TRUE	TRUE
1	WERKKISNC	340	348	9	HLA-A*02:01	340	0.014	0.055	0.02406	0.02079	50	0.014	0.055	0.02406	0.02079	50	TRUE	TRUE	TRUE	TRUE	TRUE
1	ERKKISNCV	341	349	9	HLA-A*02:01	341	0.014	0.259	0.88767	0.2202	32	0.014	0.259	0.88767	0.2202	32	TRUE	TRUE	TRUE	TRUE	TRUE
1	RKKISNCVA	342	350	9	HLA-A*02:01	342	0.012	-0.411	0.08575	0.02102	50	0.012	-0.411	0.08575	0.02102	50	TRUE	TRUE	TRUE	TRUE	TRUE
1	KKISNCVAD	343	351	9	HLA-A*02:01	343	0.007	-1.458	0.02701	-0.02337	50	0.007	-1.458	0.02701	-0.02337	50	TRUE	TRUE	TRUE	TRUE	TRUE
1	KISNCVADY	344	352	9	HLA-A*02:01	344	0.078	3.257	0.90352	0.36272	9	0.078	3.257	0.90352	0.36272	9	TRUE	TRUE	TRUE	TRUE	TRUE
1	ISNCVADYS	345	353	9	HLA-A*02:01	345	0.035	-2.175	0.02306	-0.01419	50	0.035	-2.175	0.02306	-0.01419	50	TRUE	TRUE	TRUE	TRUE	TRUE
1	SNCVADYSV	346	354	9	HLA-A*02:01	346	0.053	0.233	0.18937	0.10143	50	0.053	0.233	0.18937	0.10143	50	TRUE	TRUE	TRUE	TRUE	TRUE
1	NCVADYSVL	347	355	9	HLA-A*02:01	347	0.032	1.144	0.22703	0.11168	50	0.032	1.144	0.22703	0.11168	50	TRUE	TRUE	TRUE	TRUE	TRUE
1	CVADYSVLY	348	356	9	HLA-A*02:01	348	0.1	3.18	0.97218	0.39824	8	0.1	3.18	0.97218	0.39824	8	TRUE	TRUE	TRUE	TRUE	TRUE
1	VADYSVLYN	349	357	9	HLA-A*02:01	349	0.037	-1.384	0.02437	0.00788	50	0.037	-1.384	0.02437	0.00788	50	TRUE	TRUE	TRUE	TRUE	TRUE
1	ADYSVLYNS	350	358	9	HLA-A*02:01	350	0.05	-2.175	0.02995	0.00236	50	0.05	-2.175	0.02995	0.00236	50	TRUE	TRUE	TRUE	TRUE	TRUE
1	DYSVLYNST	351	359	9	HLA-A*02:01	351	0.011	-0.607	0.10081	0.01851	50	0.011	-0.607	0.10081	0.01851	50	TRUE	TRUE	TRUE	TRUE	TRUE
1	YSVLYNSTF	352	360	9	HLA-A*02:01	352	0.084	2.494	0.87729	0.34374	16	0.084	2.494	0.87729	0.34374	16	TRUE	TRUE	TRUE	TRUE	TRUE
1	SVLYNSTFF	353	361	9	HLA-A*02:01	353	0.173	2.692	0.7227	0.40291	8	0.173	2.692	0.7227	0.40291	8	TRUE	TRUE	TRUE	TRUE	TRUE
1	VLYNSTFFS	354	362	9	HLA-A*02:01	354	0.526	-1.923	0.06306	0.49211	5	0.526	-1.923	0.06306	0.49211	5	TRUE	TRUE	TRUE	TRUE	TRUE
1	LYNSTFFST	355	363	9	HLA-A*02:01	355	0.055	-0.484	0.17051	0.08126	50	0.055	-0.484	0.17051	0.08126	50	TRUE	TRUE	TRUE	TRUE	TRUE
1	YNSTFFSTF	356	364	9	HLA-A*02:01	356	0.066	2.406	0.95778	0.34165	16	0.066	2.406	0.95778	0.34165	16	TRUE	TRUE	TRUE	TRUE	TRUE
1	NSTFFSTFK	357	365	9	HLA-A*02:01	357	0.041	0.454	0.74112	0.2191	32	0.041	0.454	0.74112	0.2191	32	TRUE	TRUE	TRUE	TRUE	TRUE
1	STFFSTFKC	358	366	9	HLA-A*02:01	358	0.154	0.299	0.07989	0.17945	50	0.154	0.299	0.07989	0.17945	50	TRUE	TRUE	TRUE	TRUE	TRUE
1	TFFSTFKCY	359	367	9	HLA-A*02:01	359	0.031	3.237	0.91578	0.31798	16	0.031	3.237	0.91578	0.31798	16	TRUE	TRUE	TRUE	TRUE	TRUE
1	FFSTFKCYG	360	368	9	HLA-A*02:01	360	0.044	-1.317	0.02762	0.01729	50	0.044	-1.317	0.02762	0.01729	50	TRUE	TRUE	TRUE	TRUE	TRUE
1	FSTFKCYGV	361	369	9	HLA-A*02:01	361	0.372	0.119	0.83231	0.56225	4	0.372	0.119	0.83231	0.56225	4	TRUE	TRUE	TRUE	TRUE	TRUE
1	STFKCYGVS	362	370	9	HLA-A*02:01	362	0.042	-2.207	0.04534	-0.00297	50	0.042	-2.207	0.04534	-0.00297	50	TRUE	TRUE	TRUE	TRUE	TRUE
1	TFKCYGVSA	363	371	9	HLA-A*02:01	363	0.053	-0.337	0.11832	0.0712	50	0.053	-0.337	0.11832	0.0712	50	TRUE	TRUE	TRUE	TRUE	TRUE
1	FKCYGVSAT	364	372	9	HLA-A*02:01	364	0.022	-0.732	0.02703	0.00978	50	0.022	-0.732	0.02703	0.00978	50	TRUE	TRUE	TRUE	TRUE	TRUE
1	KCYGVSATK	365	373	9	HLA-A*02:01	365	0.024	0.674	0.901	0.24357	32	0.024	0.674	0.901	0.24357	32	TRUE	TRUE	TRUE	TRUE	TRUE
1	CYGVSATKL	366	374	9	HLA-A*02:01	366	0.025	1.116	0.86408	0.24732	32	0.025	1.116	0.86408	0.24732	32	TRUE	TRUE	TRUE	TRUE	TRUE
1	YGVSATKLN	367	375	9	HLA-A*02:01	367	0.008	-1.531	0.02945	-0.02365	50	0.008	-1.531	0.02945	-0.02365	50	TRUE	TRUE	TRUE	TRUE	TRUE
1	GVSATKLND	368	376	9	HLA-A*02:01	368	0.012	-1.912	0.02777	-0.02955	50	0.012	-1.912	0.02777	-0.02955	50	TRUE	TRUE	TRUE	TRUE	TRUE
1	VSATKLNDL	369	377	9	HLA-A*02:01	369	0.062	1.044	0.78181	0.26401	32	0.062	1.044	0.78181	0.26401	32	TRUE	TRUE	TRUE	TRUE	TRUE
1	SATKLNDLC	370	378	9	HLA-A*02:01	370	0.03	0.131	0.02294	0.03844	50	0.03	0.131	0.02294	0.03844	50	TRUE	TRUE	TRUE	TRUE	TRUE
1	ATKLNDLCF	371	379	9	HLA-A*02:01	371	0.027	2.709	0.3684	0.17762	50	0.027	2.709	0.3684	0.17762	50	TRUE	TRUE	TRUE	TRUE	TRUE
1	TKLNDLCFS	372	380	9	HLA-A*02:01	372	0.017	-2.045	0.02619	-0.02823	50	0.017	-2.045	0.02619	-0.02823	50	TRUE	TRUE	TRUE	TRUE	TRUE
1	KLNDLCFSN	373	381	9	HLA-A*02:01	373	0.267	-1.273	0.05686	0.24797	32	0.267	-1.273	0.05686	0.24797	32	TRUE	TRUE	TRUE	TRUE	TRUE
1	LNDLCFSNV	374	382	9	HLA-A*02:01	374	0.12	0.091	0.26046	0.18088	50	0.12	0.091	0.26046	0.18088	50	TRUE	TRUE	TRUE	TRUE	TRUE
1	NDLCFSNVY	375	383	9	HLA-A*02:01	375	0.021	2.72	0.89109	0.28949	32	0.021	2.72	0.89109	0.28949	32	TRUE	TRUE	TRUE	TRUE	TRUE
1	DLCFSNVYA	376	384	9	HLA-A*02:01	376	0.149	-0.682	0.09784	0.15396	50	0.149	-0.682	0.09784	0.15396	50	TRUE	TRUE	TRUE	TRUE	TRUE
1	LCFSNVYAD	377	385	9	HLA-A*02:01	377	0.021	-1.818	0.0342	-0.01676	50	0.021	-1.818	0.0342	-0.01676	50	TRUE	TRUE	TRUE	TRUE	TRUE
1	CFSNVYADS	378	386	9	HLA-A*02:01	378	0.031	-2.075	0.04514	-0.01072	50	0.031	-2.075	0.04514	-0.01072	50	TRUE	TRUE	TRUE	TRUE	TRUE
1	FSNVYADSF	379	387	9	HLA-A*02:01	379	0.08	2.379	0.19362	0.18304	50	0.08	2.379	0.19362	0.18304	50	TRUE	TRUE	TRUE	TRUE	TRUE
1	SNVYADSFV	380	388	9	HLA-A*02:01	380	0.07	0.188	0.06227	0.08871	50	0.07	0.188	0.06227	0.08871	50	TRUE	TRUE	TRUE	TRUE	TRUE
1	NVYADSFVV	381	389	9	HLA-A*02:01	381	0.611	0.731	0.95972	0.84521	1	0.611	0.731	0.95972	0.84521	1	TRUE	TRUE	TRUE	TRUE	TRUE
1	VYADSFVVK	382	390	9	HLA-A*02:01	382	0.033	0.949	0.94059	0.26836	32	0.033	0.949	0.94059	0.26836	32	TRUE	TRUE	TRUE	TRUE	TRUE
1	YADSFVVKG	383	391	9	HLA-A*02:01	383	0.071	-1.492	0.29281	0.09958	50	0.071	-1.492	0.29281	0.09958	50	TRUE	TRUE	TRUE	TRUE	TRUE
1	ADSFVVKGD	384	392	9	HLA-A*02:01	384	0.008	-2.035	0.05049	-0.03151	50	0.008	-2.035	0.05049	-0.03151	50	TRUE	TRUE	TRUE	TRUE	TRUE
1	DSFVVKGDD	385	393	9	HLA-A*02:01	385	0.004	-1.825	0.0256	-0.03586	50	0.004	-1.825	0.0256	-0.03586	50	TRUE	TRUE	TRUE	TRUE	TRUE
1	SFVVKGDDV	386	394	9	HLA-A*02:01	386	0.065	0.474	0.13046	0.1062	50	0.065	0.474	0.13046	0.1062	50	TRUE	TRUE	TRUE	TRUE	TRUE
1	FVVKGDDVR	387	395	9	HLA-A*02:01	387	0.031	1.66	0.65886	0.22074	32	0.031	1.66	0.65886	0.22074	32	TRUE	TRUE	TRUE	TRUE	TRUE
1	VVKGDDVRQ	388	396	9	HLA-A*02:01	388	0.01	-0.012	0.21664	0.05844	50	0.01	-0.012	0.21664	0.05844	50	TRUE	TRUE	TRUE	TRUE	TRUE
1	VKGDDVRQI	389	397	9	HLA-A*02:01	389	0.023	0.53	0.44954	0.1374	50	0.023	0.53	0.44954	0.1374	50	TRUE	TRUE	TRUE	TRUE	TRUE
1	KGDDVRQIA	390	398	9	HLA-A*02:01	390	0.041	-0.824	0.12573	0.04869	50	0.041	-0.824	0.12573	0.04869	50	TRUE	TRUE	TRUE	TRUE	TRUE
1	GDDVRQIAP	391	399	9	HLA-A*02:01	391	0.007	-0.457	0.02562	0.00134	50	0.007	-0.457	0.02562	0.00134	50	TRUE	TRUE	TRUE	TRUE	TRUE
1	DDVRQIAPG	392	400	9	HLA-A*02:01	392	0.006	-1.971	0.07737	-0.02587	50	0.006	-1.971	0.07737	-0.02587	50	TRUE	TRUE	TRUE	TRUE	TRUE
1	DVRQIAPGQ	393	401	9	HLA-A*02:01	393	0.01	-0.373	0.02541	0.00639	50	0.01	-0.373	0.02541	0.00639	50	TRUE	TRUE	TRUE	TRUE	TRUE
1	VRQIAPGQT	394	402	9	HLA-A*02:01	394	0.014	-0.608	0.03341	0.00632	50	0.014	-0.608	0.03341	0.00632	50	TRUE	TRUE	TRUE	TRUE	TRUE
1	RQIAPGQTG	395	403	9	HLA-A*02:01	395	0.052	-0.96	0.03289	0.0354	50	0.052	-0.96	0.03289	0.0354	50	TRUE	TRUE	TRUE	TRUE	TRUE
1	QIAPGQTGV	396	404	9	HLA-A*02:01	396	0.386	0.605	0.79014	0.57891	4	0.386	0.605	0.79014	0.57891	4	TRUE	TRUE	TRUE	TRUE	TRUE
1	IAPGQTGVI	397	405	9	HLA-A*02:01	397	0.065	0.546	0.64151	0.22299	32	0.065	0.546	0.64151	0.22299	32	TRUE	TRUE	TRUE	TRUE	TRUE
1	APGQTGVIA	398	406	9	HLA-A*02:01	398	0.024	-0.85	0.4453	0.10294	50	0.024	-0.85	0.4453	0.10294	50	TRUE	TRUE	TRUE	TRUE	TRUE
1	PGQTGVIAD	399	407	9	HLA-A*02:01	399	0.004	-2.44	0.02847	-0.0506	50	0.004	-2.44	0.02847	-0.0506	50	TRUE	TRUE	TRUE	TRUE	TRUE
1	GQTGVIADY	400	408	9	HLA-A*02:01	400	0.076	2.64	0.97589	0.36157	10	0.076	2.64	0.97589	0.36157	10	TRUE	TRUE	TRUE	TRUE	TRUE
1	QTGVIADYN	401	409	9	HLA-A*02:01	401	0.013	-1.517	0.02459	-0.01939	50	0.013	-1.517	0.02459	-0.01939	50	TRUE	TRUE	TRUE	TRUE	TRUE
1	TGVIADYNY	402	410	9	HLA-A*02:01	402	0.021	2.666	0.9506	0.30154	16	0.021	2.666	0.9506	0.30154	16	TRUE	TRUE	TRUE	TRUE	TRUE
1	GVIADYNYK	403	411	9	HLA-A*02:01	403	0.044	0.503	0.69248	0.21238	32	0.044	0.503	0.69248	0.21238	32	TRUE	TRUE	TRUE	TRUE	TRUE
1	VIADYNYKL	404	412	9	HLA-A*02:01	404	0.701	1.132	0.95259	0.94363	0.8	0.701	1.132	0.95259	0.94363	0.8	TRUE	TRUE	TRUE	TRUE	TRUE
1	IADYNYKLP	405	413	9	HLA-A*02:01	405	0.014	0.051	0.02305	0.02046	50	0.014	0.051	0.02305	0.02046	50	TRUE	TRUE	TRUE	TRUE	TRUE
1	ADYNYKLPD	406	414	9	HLA-A*02:01	406	0.016	-1.783	0.03275	-0.02121	50	0.016	-1.783	0.03275	-0.02121	50	TRUE	TRUE	TRUE	TRUE	TRUE
1	DYNYKLPDD	407	415	9	HLA-A*02:01	407	0.004	-1.807	0.02291	-0.03602	50	0.004	-1.807	0.02291	-0.03602	50	TRUE	TRUE	TRUE	TRUE	TRUE
1	YNYKLPDDF	408	416	9	HLA-A*02:01	408	0.028	2.41	0.17381	0.12736	50	0.028	2.41	0.17381	0.12736	50	TRUE	TRUE	TRUE	TRUE	TRUE
1	NYKLPDDFM	409	417	9	HLA-A*02:01	409	0.015	0.552	0.17902	0.06908	50	0.015	0.552	0.17902	0.06908	50	TRUE	TRUE	TRUE	TRUE	TRUE
1	YKLPDDFMG	410	418	9	HLA-A*02:01	410	0.018	-1.207	0.03765	-0.0037	50	0.018	-1.207	0.03765	-0.0037	50	TRUE	TRUE	TRUE	TRUE	TRUE
1	KLPDDFMGC	411	419	9	HLA-A*02:01	411	0.51	0.074	0.07183	0.52801	5	0.51	0.074	0.07183	0.52801	5	TRUE	TRUE	TRUE	TRUE	TRUE
1	LPDDFMGCV	412	420	9	HLA-A*02:01	412	0.059	-0.061	0.87174	0.25362	32	0.059	-0.061	0.87174	0.25362	32	TRUE	TRUE	TRUE	TRUE	TRUE
1	PDDFMGCVL	413	421	9	HLA-A*02:01	413	0.016	0.039	0.32518	0.09014	50	0.016	0.039	0.32518	0.09014	50	TRUE	TRUE	TRUE	TRUE	TRUE
1	DDFMGCVLA	414	422	9	HLA-A*02:01	414	0.03	-1.192	0.23988	0.05417	50	0.03	-1.192	0.23988	0.05417	50	TRUE	TRUE	TRUE	TRUE	TRUE
1	DFMGCVLAW	415	423	9	HLA-A*02:01	415	0.047	0.662	0.62959	0.20521	32	0.047	0.662	0.62959	0.20521	32	TRUE	TRUE	TRUE	TRUE	TRUE
1	FMGCVLAWN	416	424	9	HLA-A*02:01	416	0.366	-1.591	0.0245	0.33174	16	0.366	-1.591	0.0245	0.33174	16	TRUE	TRUE	TRUE	TRUE	TRUE
1	MGCVLAWNT	417	425	9	HLA-A*02:01	417	0.032	-1.021	0.02327	0.01171	50	0.032	-1.021	0.02327	0.01171	50	TRUE	TRUE	TRUE	TRUE	TRUE
1	GCVLAWNTR	418	426	9	HLA-A*02:01	418	0.012	1.44	0.40791	0.13978	50	0.012	1.44	0.40791	0.13978	50	TRUE	TRUE	TRUE	TRUE	TRUE
1	CVLAWNTRN	419	427	9	HLA-A*02:01	419	0.043	-1.23	0.03673	0.02051	50	0.043	-1.23	0.03673	0.02051	50	TRUE	TRUE	TRUE	TRUE	TRUE
1	VLAWNTRNI	420	428	9	HLA-A*02:01	420	0.471	0.626	0.76582	0.65896	3	0.471	0.626	0.76582	0.65896	3	TRUE	TRUE	TRUE	TRUE	TRUE
1	LAWNTRNID	421	429	9	HLA-A*02:01	421	0.016	-1.54	0.09212	-0.00177	50	0.016	-1.54	0.09212	-0.00177	50	TRUE	TRUE	TRUE	TRUE	TRUE
1	AWNTRNIDA	422	430	9	HLA-A*02:01	422	0.029	-0.191	0.07274	0.04059	50	0.029	-0.191	0.07274	0.04059	50	TRUE	TRUE	TRUE	TRUE	TRUE
1	WNTRNIDAT	423	431	9	HLA-A*02:01	423	0.013	-0.767	0.0241	-0.00075	50	0.013	-0.767	0.0241	-0.00075	50	TRUE	TRUE	TRUE	TRUE	TRUE
1	NTRNIDATS	424	432	9	HLA-A*02:01	424	0.018	-2.237	0.03499	-0.03005	50	0.018	-2.237	0.03499	-0.03005	50	TRUE	TRUE	TRUE	TRUE	TRUE
1	TRNIDATST	425	433	9	HLA-A*02:01	425	0.008	-0.473	0.03287	0.00357	50	0.008	-0.473	0.03287	0.00357	50	TRUE	TRUE	TRUE	TRUE	TRUE
1	RNIDATSTG	426	434	9	HLA-A*02:01	426	0.015	-1.225	0.03717	-0.00726	50	0.015	-1.225	0.03717	-0.00726	50	TRUE	TRUE	TRUE	TRUE	TRUE
1	NIDATSTGN	427	435	9	HLA-A*02:01	427	0.022	-1.194	0.02577	-0.00205	50	0.022	-1.194	0.02577	-0.00205	50	TRUE	TRUE	TRUE	TRUE	TRUE
1	IDATSTGNY	428	436	9	HLA-A*02:01	428	0.012	2.798	0.55055	0.20582	32	0.012	2.798	0.55055	0.20582	32	TRUE	TRUE	TRUE	TRUE	TRUE
1	DATSTGNYN	429	437	9	HLA-A*02:01	429	0.007	-1.606	0.02324	-0.02792	50	0.007	-1.606	0.02324	-0.02792	50	TRUE	TRUE	TRUE	TRUE	TRUE
1	ATSTGNYNY	430	438	9	HLA-A*02:01	430	0.051	2.904	0.96088	0.3398	16	0.051	2.904	0.96088	0.3398	16	TRUE	TRUE	TRUE	TRUE	TRUE
1	TSTGNYNYK	431	439	9	HLA-A*02:01	431	0.022	0.602	0.93098	0.24652	32	0.022	0.602	0.93098	0.24652	32	TRUE	TRUE	TRUE	TRUE	TRUE
1	STGNYNYKY	432	440	9	HLA-A*02:01	432	0.042	2.803	0.9707	0.33048	16	0.042	2.803	0.9707	0.33048	16	TRUE	TRUE	TRUE	TRUE	TRUE
1	TGNYNYKYR	433	441	9	HLA-A*02:01	433	0.01	1.157	0.0607	0.05258	50	0.01	1.157	0.0607	0.05258	50	TRUE	TRUE	TRUE	TRUE	TRUE
1	GNYNYKYRY	434	442	9	HLA-A*02:01	434	0.032	2.79	0.96698	0.31932	16	0.032	2.79	0.96698	0.31932	16	TRUE	TRUE	TRUE	TRUE	TRUE
1	NYNYKYRYL	435	443	9	HLA-A*02:01	435	0.026	1.132	0.6201	0.19382	50	0.026	1.132	0.6201	0.19382	50	TRUE	TRUE	TRUE	TRUE	TRUE
1	YNYKYRYLR	436	444	9	HLA-A*02:01	436	0.056	1.64	0.61231	0.23477	32	0.056	1.64	0.61231	0.23477	32	TRUE	TRUE	TRUE	TRUE	TRUE
1	NYKYRYLRH	437	445	9	HLA-A*02:01	437	0.012	-0.288	0.10743	0.02897	50	0.012	-0.288	0.10743	0.02897	50	TRUE	TRUE	TRUE	TRUE	TRUE
1	YKYRYLRHG	438	446	9	HLA-A*02:01	438	0.023	-1.038	0.11731	0.02344	50	0.023	-1.038	0.11731	0.02344	50	TRUE	TRUE	TRUE	TRUE	TRUE
1	KYRYLRHGK	439	447	9	HLA-A*02:01	439	0.016	0.833	0.46341	0.14109	50	0.016	0.833	0.46341	0.14109	50	TRUE	TRUE	TRUE	TRUE	TRUE
1	YRYLRHGKL	440	448	9	HLA-A*02:01	440	0.055	1.429	0.97622	0.31037	16	0.055	1.429	0.97622	0.31037	16	TRUE	TRUE	TRUE	TRUE	TRUE
1	RYLRHGKLR	441	449	9	HLA-A*02:01	441	0.021	1.959	0.26003	0.12848	50	0.021	1.959	0.26003	0.12848	50	TRUE	TRUE	TRUE	TRUE	TRUE
1	YLRHGKLRP	442	450	9	HLA-A*02:01	442	0.033	0.285	0.16269	0.07673	50	0.033	0.285	0.16269	0.07673	50	TRUE	TRUE	TRUE	TRUE	TRUE
1	LRHGKLRPF	443	451	9	HLA-A*02:01	443	0.022	2.723	0.93075	0.29949	16	0.022	2.723	0.93075	0.29949	16	TRUE	TRUE	TRUE	TRUE	TRUE
1	RHGKLRPFE	444	452	9	HLA-A*02:01	444	0.007	-1.429	0.0228	-0.02359	50	0.007	-1.429	0.0228	-0.02359	50	TRUE	TRUE	TRUE	TRUE	TRUE
1	HGKLRPFER	445	453	9	HLA-A*02:01	445	0.015	1.257	0.57992	0.17691	50	0.015	1.257	0.57992	0.17691	50	TRUE	TRUE	TRUE	TRUE	TRUE
1	GKLRPFERD	446	454	9	HLA-A*02:01	446	0.005	-1.95	0.04293	-0.03409	50	0.005	-1.95	0.04293	-0.03409	50	TRUE	TRUE	TRUE	TRUE	TRUE
1	KLRPFERDI	447	455	9	HLA-A*02:01	447	0.226	0.68	0.81848	0.42716	7	0.226	0.68	0.81848	0.42716	7	TRUE	TRUE	TRUE	TRUE	TRUE
1	LRPFERDIS	448	456	9	HLA-A*02:01	448	0.006	-1.949	0.03078	-0.0358	50	0.006	-1.949	0.03078	-0.0358	50	TRUE	TRUE	TRUE	TRUE	TRUE
1	RPFERDISN	449	457	9	HLA-A*02:01	449	0.018	-1.216	0.30936	0.05721	50	0.018	-1.216	0.30936	0.05721	50	TRUE	TRUE	TRUE	TRUE	TRUE
1	PFERDISNV	450	458	9	HLA-A*02:01	450	0.017	-0.01	0.95402	0.2314	32	0.017	-0.01	0.95402	0.2314	32	TRUE	TRUE	TRUE	TRUE	TRUE

seq #	peptide	start	end	peptide length	allele	peptide index	NetCTLpan MH	NetCTLpan tap	NetCTLpan clea	NetCTLpan con	NetCTLpan per	legacy mhc_pr	legacy tap_pre	legacy cleavag	legacy combine	legacy %_rank	equal mhc_pre	equal tap_pred	equal cleavage	equal combine	equal %_rank
1	FERDISNVP	451	459	9	HLA-A*02:01	451	0.011	-0.222	0.28831	0.07032	50	0.011	-0.222	0.28831	0.07032	50	TRUE	TRUE	TRUE	TRUE	TRUE
1	ERDISNVPF	452	460	9	HLA-A*02:01	452	0.018	2.256	0.97307	0.29334	16	0.018	2.256	0.97307	0.29334	16	TRUE	TRUE	TRUE	TRUE	TRUE
1	RDISNVPFS	453	461	9	HLA-A*02:01	453	0.028	-2.407	0.02509	-0.02653	50	0.028	-2.407	0.02509	-0.02653	50	TRUE	TRUE	TRUE	TRUE	TRUE
1	DISNVPFSP	454	462	9	HLA-A*02:01	454	0.025	0.045	0.71192	0.18631	50	0.025	0.045	0.71192	0.18631	50	TRUE	TRUE	TRUE	TRUE	TRUE
1	ISNVPFSPD	455	463	9	HLA-A*02:01	455	0.017	-1.985	0.10316	-0.00941	50	0.017	-1.985	0.10316	-0.00941	50	TRUE	TRUE	TRUE	TRUE	TRUE
1	SNVPFSPDG	456	464	9	HLA-A*02:01	456	0.012	-1.408	0.25435	0.03403	50	0.012	-1.408	0.25435	0.03403	50	TRUE	TRUE	TRUE	TRUE	TRUE
1	NVPFSPDGK	457	465	9	HLA-A*02:01	457	0.02	0.679	0.55946	0.16285	50	0.02	0.679	0.55946	0.16285	50	TRUE	TRUE	TRUE	TRUE	TRUE
1	VPFSPDGKP	458	466	9	HLA-A*02:01	458	0.007	0.115	0.53455	0.13015	50	0.007	0.115	0.53455	0.13015	50	TRUE	TRUE	TRUE	TRUE	TRUE
1	PFSPDGKPC	459	467	9	HLA-A*02:01	459	0.008	-0.255	0.0246	0.00716	50	0.008	-0.255	0.0246	0.00716	50	TRUE	TRUE	TRUE	TRUE	TRUE
1	FSPDGKPCT	460	468	9	HLA-A*02:01	460	0.03	-0.92	0.02919	0.01357	50	0.03	-0.92	0.02919	0.01357	50	TRUE	TRUE	TRUE	TRUE	TRUE
1	SPDGKPCTP	461	469	9	HLA-A*02:01	461	0.009	-0.428	0.2589	0.05655	50	0.009	-0.428	0.2589	0.05655	50	TRUE	TRUE	TRUE	TRUE	TRUE
1	PDGKPCTPP	462	470	9	HLA-A*02:01	462	0.003	-0.792	0.04482	-0.00672	50	0.003	-0.792	0.04482	-0.00672	50	TRUE	TRUE	TRUE	TRUE	TRUE
1	DGKPCTPPA	463	471	9	HLA-A*02:01	463	0.015	-1.265	0.28409	0.0473	50	0.015	-1.265	0.28409	0.0473	50	TRUE	TRUE	TRUE	TRUE	TRUE
1	GKPCTPPAL	464	472	9	HLA-A*02:01	464	0.029	0.671	0.78605	0.22264	32	0.029	0.671	0.78605	0.22264	32	TRUE	TRUE	TRUE	TRUE	TRUE
1	KPCTPPALN	465	473	9	HLA-A*02:01	465	0.007	-1.534	0.17637	0.00833	50	0.007	-1.534	0.17637	0.00833	50	TRUE	TRUE	TRUE	TRUE	TRUE
1	PCTPPALNC	466	474	9	HLA-A*02:01	466	0.009	-0.38	0.02793	0.00579	50	0.009	-0.38	0.02793	0.00579	50	TRUE	TRUE	TRUE	TRUE	TRUE
1	CTPPALNCY	467	475	9	HLA-A*02:01	467	0.046	2.702	0.92779	0.3223	16	0.046	2.702	0.92779	0.3223	16	TRUE	TRUE	TRUE	TRUE	TRUE
1	TPPALNCYW	468	476	9	HLA-A*02:01	468	0.023	0.537	0.84935	0.22753	32	0.023	0.537	0.84935	0.22753	32	TRUE	TRUE	TRUE	TRUE	TRUE
1	PPALNCYWP	469	477	9	HLA-A*02:01	469	0.007	-0.653	0.04554	0.00092	50	0.007	-0.653	0.04554	0.00092	50	TRUE	TRUE	TRUE	TRUE	TRUE
1	PALNCYWPL	470	478	9	HLA-A*02:01	470	0.134	0.4	0.45583	0.24656	32	0.134	0.4	0.45583	0.24656	32	TRUE	TRUE	TRUE	TRUE	TRUE
1	ALNCYWPLN	471	479	9	HLA-A*02:01	471	0.151	-1.391	0.02767	0.12245	50	0.151	-1.391	0.02767	0.12245	50	TRUE	TRUE	TRUE	TRUE	TRUE
1	LNCYWPLND	472	480	9	HLA-A*02:01	472	0.01	-1.801	0.0228	-0.0299	50	0.01	-1.801	0.0228	-0.0299	50	TRUE	TRUE	TRUE	TRUE	TRUE
1	NCYWPLNDY	473	481	9	HLA-A*02:01	473	0.024	3.217	0.87877	0.30215	16	0.024	3.217	0.87877	0.30215	16	TRUE	TRUE	TRUE	TRUE	TRUE
1	CYWPLNDYG	474	482	9	HLA-A*02:01	474	0.025	-0.859	0.09943	0.0259	50	0.025	-0.859	0.09943	0.0259	50	TRUE	TRUE	TRUE	TRUE	TRUE
1	YWPLNDYGF	475	483	9	HLA-A*02:01	475	0.047	2.739	0.7302	0.27977	32	0.047	2.739	0.7302	0.27977	32	TRUE	TRUE	TRUE	TRUE	TRUE
1	WPLNDYGFY	476	484	9	HLA-A*02:01	476	0.019	2.559	0.92294	0.29064	16	0.019	2.559	0.92294	0.29064	16	TRUE	TRUE	TRUE	TRUE	TRUE
1	PLNDYGFYT	477	485	9	HLA-A*02:01	477	0.356	-1.316	0.19148	0.36618	9	0.356	-1.316	0.19148	0.36618	9	TRUE	TRUE	TRUE	TRUE	TRUE
1	LNDYGFYTT	478	486	9	HLA-A*02:01	478	0.07	-1.175	0.0565	0.05334	50	0.07	-1.175	0.0565	0.05334	50	TRUE	TRUE	TRUE	TRUE	TRUE
1	NDYGFYTTT	479	487	9	HLA-A*02:01	479	0.052	-0.741	0.42484	0.12906	50	0.052	-0.741	0.42484	0.12906	50	TRUE	TRUE	TRUE	TRUE	TRUE
1	DYGFYTTTG	480	488	9	HLA-A*02:01	480	0.009	-1.469	0.29204	0.03798	50	0.009	-1.469	0.29204	0.03798	50	TRUE	TRUE	TRUE	TRUE	TRUE
1	YGFYTTTGI	481	489	9	HLA-A*02:01	481	0.21	0.221	0.38362	0.30184	16	0.21	0.221	0.38362	0.30184	16	TRUE	TRUE	TRUE	TRUE	TRUE
1	GFYTTTGIG	482	490	9	HLA-A*02:01	482	0.02	-1.272	0.04091	-0.0026	50	0.02	-1.272	0.04091	-0.0026	50	TRUE	TRUE	TRUE	TRUE	TRUE
1	FYTTTGIGY	483	491	9	HLA-A*02:01	483	0.033	3.028	0.72559	0.27196	32	0.033	3.028	0.72559	0.27196	32	TRUE	TRUE	TRUE	TRUE	TRUE
1	YTTTGIGYQ	484	492	9	HLA-A*02:01	484	0.041	-0.288	0.02808	0.04012	50	0.041	-0.288	0.02808	0.04012	50	TRUE	TRUE	TRUE	TRUE	TRUE
1	TTTGIGYQP	485	493	9	HLA-A*02:01	485	0.024	-0.026	0.03075	0.03027	50	0.024	-0.026	0.03075	0.03027	50	TRUE	TRUE	TRUE	TRUE	TRUE
1	TTGIGYQPY	486	494	9	HLA-A*02:01	486	0.029	2.729	0.79364	0.27579	32	0.029	2.729	0.79364	0.27579	32	TRUE	TRUE	TRUE	TRUE	TRUE
1	TGIGYQPYR	487	495	9	HLA-A*02:01	487	0.027	1.276	0.06275	0.07302	50	0.027	1.276	0.06275	0.07302	50	TRUE	TRUE	TRUE	TRUE	TRUE
1	GIGYQPYRV	488	496	9	HLA-A*02:01	488	0.276	0.111	0.23712	0.33213	16	0.276	0.111	0.23712	0.33213	16	TRUE	TRUE	TRUE	TRUE	TRUE
1	IGYQPYRVV	489	497	9	HLA-A*02:01	489	0.071	0.228	0.57955	0.2071	32	0.071	0.228	0.57955	0.2071	32	TRUE	TRUE	TRUE	TRUE	TRUE
1	GYQPYRVVV	490	498	9	HLA-A*02:01	490	0.056	0.377	0.90967	0.2701	32	0.056	0.377	0.90967	0.2701	32	TRUE	TRUE	TRUE	TRUE	TRUE
1	YQPYRVVVL	491	499	9	HLA-A*02:01	491	0.318	0.965	0.96587	0.55945	4	0.318	0.965	0.96587	0.55945	4	TRUE	TRUE	TRUE	TRUE	TRUE
1	QPYRVVVLS	492	500	9	HLA-A*02:01	492	0.026	-2.402	0.567	0.09353	50	0.026	-2.402	0.567	0.09353	50	TRUE	TRUE	TRUE	TRUE	TRUE
1	PYRVVVLSF	493	501	9	HLA-A*02:01	493	0.018	2.319	0.95414	0.29066	16	0.018	2.319	0.95414	0.29066	16	TRUE	TRUE	TRUE	TRUE	TRUE
1	YRVVVLSFE	494	502	9	HLA-A*02:01	494	0.015	-1.446	0.03703	-0.01282	50	0.015	-1.446	0.03703	-0.01282	50	TRUE	TRUE	TRUE	TRUE	TRUE
1	RVVVLSFEL	495	503	9	HLA-A*02:01	495	0.43	1.363	0.94455	0.6766	3	0.43	1.363	0.94455	0.6766	3	TRUE	TRUE	TRUE	TRUE	TRUE
1	VVVLSFELL	496	504	9	HLA-A*02:01	496	0.238	1.365	0.77786	0.44714	6	0.238	1.365	0.77786	0.44714	6	TRUE	TRUE	TRUE	TRUE	TRUE
1	VVLSFELLN	497	505	9	HLA-A*02:01	497	0.052	-1.05	0.04956	0.0369	50	0.052	-1.05	0.04956	0.0369	50	TRUE	TRUE	TRUE	TRUE	TRUE
1	VLSFELLNA	498	506	9	HLA-A*02:01	498	0.592	-0.411	0.21462	0.63001	3	0.592	-0.411	0.21462	0.63001	3	TRUE	TRUE	TRUE	TRUE	TRUE
1	LSFELLNAP	499	507	9	HLA-A*02:01	499	0.046	0.308	0.05919	0.06702	50	0.046	0.308	0.05919	0.06702	50	TRUE	TRUE	TRUE	TRUE	TRUE
1	SFELLNAPA	500	508	9	HLA-A*02:01	500	0.094	-0.411	0.30751	0.15292	50	0.094	-0.411	0.30751	0.15292	50	TRUE	TRUE	TRUE	TRUE	TRUE
1	FELLNAPAT	501	509	9	HLA-A*02:01	501	0.058	-0.864	0.03643	0.0446	50	0.058	-0.864	0.03643	0.0446	50	TRUE	TRUE	TRUE	TRUE	TRUE
1	ELLNAPATV	502	510	9	HLA-A*02:01	502	0.436	0.043	0.97686	0.65687	3	0.436	0.043	0.97686	0.65687	3	TRUE	TRUE	TRUE	TRUE	TRUE
1	LLNAPATVC	503	511	9	HLA-A*02:01	503	0.175	-0.223	0.05408	0.18159	50	0.175	-0.223	0.05408	0.18159	50	TRUE	TRUE	TRUE	TRUE	TRUE
1	LNAPATVCG	504	512	9	HLA-A*02:01	504	0.014	-1.479	0.03565	-0.01495	50	0.014	-1.479	0.03565	-0.01495	50	TRUE	TRUE	TRUE	TRUE	TRUE
1	NAPATVCGP	505	513	9	HLA-A*02:01	505	0.017	0.227	0.04809	0.0335	50	0.017	0.227	0.04809	0.0335	50	TRUE	TRUE	TRUE	TRUE	TRUE
1	APATVCGPK	506	514	9	HLA-A*02:01	506	0.01	0.462	0.91926	0.22838	32	0.01	0.462	0.91926	0.22838	32	TRUE	TRUE	TRUE	TRUE	TRUE
1	PATVCGPKL	507	515	9	HLA-A*02:01	507	0.021	0.645	0.82836	0.22351	32	0.021	0.645	0.82836	0.22351	32	TRUE	TRUE	TRUE	TRUE	TRUE
1	ATVCGPKLS	508	516	9	HLA-A*02:01	508	0.02	-2.19	0.0239	-0.02937	50	0.02	-2.19	0.0239	-0.02937	50	TRUE	TRUE	TRUE	TRUE	TRUE
1	TVCGPKLST	509	517	9	HLA-A*02:01	509	0.032	-0.425	0.05888	0.03462	50	0.032	-0.425	0.05888	0.03462	50	TRUE	TRUE	TRUE	TRUE	TRUE
1	VCGPKLSTD	510	518	9	HLA-A*02:01	510	0.008	-1.886	0.02853	-0.03273	50	0.008	-1.886	0.02853	-0.03273	50	TRUE	TRUE	TRUE	TRUE	TRUE
1	CGPKLSTDL	511	519	9	HLA-A*02:01	511	0.036	0.679	0.43041	0.14982	50	0.036	0.679	0.43041	0.14982	50	TRUE	TRUE	TRUE	TRUE	TRUE
1	GPKLSTDLI	512	520	9	HLA-A*02:01	512	0.018	0.042	0.61057	0.15643	50	0.018	0.042	0.61057	0.15643	50	TRUE	TRUE	TRUE	TRUE	TRUE
1	PKLSTDLIK	513	521	9	HLA-A*02:01	513	0.004	0.022	0.21408	0.05272	50	0.004	0.022	0.21408	0.05272	50	TRUE	TRUE	TRUE	TRUE	TRUE
1	KLSTDLIKN	514	522	9	HLA-A*02:01	514	0.13	-1.338	0.06328	0.11079	50	0.13	-1.338	0.06328	0.11079	50	TRUE	TRUE	TRUE	TRUE	TRUE
1	LSTDLIKNQ	515	523	9	HLA-A*02:01	515	0.01	0.004	0.05145	0.02168	50	0.01	0.004	0.05145	0.02168	50	TRUE	TRUE	TRUE	TRUE	TRUE
1	STDLIKNQC	516	524	9	HLA-A*02:01	516	0.051	-0.082	0.02645	0.0549	50	0.051	-0.082	0.02645	0.0549	50	TRUE	TRUE	TRUE	TRUE	TRUE
1	TDLIKNQCV	517	525	9	HLA-A*02:01	517	0.026	0.058	0.36419	0.10939	50	0.026	0.058	0.36419	0.10939	50	TRUE	TRUE	TRUE	TRUE	TRUE
1	DLIKNQCVN	518	526	9	HLA-A*02:01	518	0.014	-1.533	0.03132	-0.01728	50	0.014	-1.533	0.03132	-0.01728	50	TRUE	TRUE	TRUE	TRUE	TRUE
1	LIKNQCVNF	519	527	9	HLA-A*02:01	519	0.055	2.526	0.90562	0.32191	16	0.055	2.526	0.90562	0.32191	16	TRUE	TRUE	TRUE	TRUE	TRUE
1	IKNQCVNFN	520	528	9	HLA-A*02:01	520	0.007	-1.289	0.02446	-0.01972	50	0.007	-1.289	0.02446	-0.01972	50	TRUE	TRUE	TRUE	TRUE	TRUE
1	KNQCVNFNF	521	529	9	HLA-A*02:01	521	0.052	2.466	0.62857	0.25508	32	0.052	2.466	0.62857	0.25508	32	TRUE	TRUE	TRUE	TRUE	TRUE
1	NQCVNFNFN	522	530	9	HLA-A*02:01	522	0.022	-1.093	0.0257	0.00046	50	0.022	-1.093	0.0257	0.00046	50	TRUE	TRUE	TRUE	TRUE	TRUE
1	QCVNFNFNG	523	531	9	HLA-A*02:01	523	0.02	-1.143	0.03004	-0.00182	50	0.02	-1.143	0.03004	-0.00182	50	TRUE	TRUE	TRUE	TRUE	TRUE
1	CVNFNFNGL	524	532	9	HLA-A*02:01	524	0.258	0.909	0.94475	0.49329	5	0.258	0.909	0.94475	0.49329	5	TRUE	TRUE	TRUE	TRUE	TRUE
1	VNFNFNGLT	525	533	9	HLA-A*02:01	525	0.037	-0.691	0.07281	0.03611	50	0.037	-0.691	0.07281	0.03611	50	TRUE	TRUE	TRUE	TRUE	TRUE
1	NFNFNGLTG	526	534	9	HLA-A*02:01	526	0.021	-1.238	0.06679	0.00508	50	0.021	-1.238	0.06679	0.00508	50	TRUE	TRUE	TRUE	TRUE	TRUE
1	FNFNGLTGT	527	535	9	HLA-A*02:01	527	0.112	-0.7	0.05386	0.10662	50	0.112	-0.7	0.05386	0.10662	50	TRUE	TRUE	TRUE	TRUE	TRUE
1	NFNGLTGTG	528	536	9	HLA-A*02:01	528	0.014	-1.39	0.13031	0.00857	50	0.014	-1.39	0.13031	0.00857	50	TRUE	TRUE	TRUE	TRUE	TRUE
1	FNGLTGTGV	529	537	9	HLA-A*02:01	529	0.098	0.045	0.14243	0.13117	50	0.098	0.045	0.14243	0.13117	50	TRUE	TRUE	TRUE	TRUE	TRUE
1	NGLTGTGVL	530	538	9	HLA-A*02:01	530	0.037	0.638	0.90179	0.25585	32	0.037	0.638	0.90179	0.25585	32	TRUE	TRUE	TRUE	TRUE	TRUE
1	GLTGTGVLT	531	539	9	HLA-A*02:01	531	0.152	-0.76	0.4994	0.24537	32	0.152	-0.76	0.4994	0.24537	32	TRUE	TRUE	TRUE	TRUE	TRUE
1	LTGTGVLTP	532	540	9	HLA-A*02:01	532	0.021	-0.106	0.15819	0.05394	50	0.021	-0.106	0.15819	0.05394	50	TRUE	TRUE	TRUE	TRUE	TRUE
1	TGTGVLTPS	533	541	9	HLA-A*02:01	533	0.027	-2.521	0.07149	-0.01994	50	0.027	-2.521	0.07149	-0.01994	50	TRUE	TRUE	TRUE	TRUE	TRUE
1	GTGVLTPSS	534	542	9	HLA-A*02:01	534	0.023	-2.599	0.06724	-0.02685	50	0.023	-2.599	0.06724	-0.02685	50	TRUE	TRUE	TRUE	TRUE	TRUE
1	TGVLTPSSK	535	543	9	HLA-A*02:01	535	0.01	0.225	0.79443	0.19437	50	0.01	0.225	0.79443	0.19437	50	TRUE	TRUE	TRUE	TRUE	TRUE
1	GVLTPSSKR	536	544	9	HLA-A*02:01	536	0.018	1.472	0.87888	0.25255	32	0.018	1.472	0.87888	0.25255	32	TRUE	TRUE	TRUE	TRUE	TRUE
1	VLTPSSKRF	537	545	9	HLA-A*02:01	537	0.102	2.55	0.96858	0.38368	8	0.102	2.55	0.96858	0.38368	8	TRUE	TRUE	TRUE	TRUE	TRUE
1	LTPSSKRFQ	538	546	9	HLA-A*02:01	538	0.007	-0.091	0.02329	0.00997	50	0.007	-0.091	0.02329	0.00997	50	TRUE	TRUE	TRUE	TRUE	TRUE
1	TPSSKRFQP	539	547	9	HLA-A*02:01	539	0.009	-0.177	0.10026	0.02713	50	0.009	-0.177	0.10026	0.02713	50	TRUE	TRUE	TRUE	TRUE	TRUE
1	PSSKRFQPF	540	548	9	HLA-A*02:01	540	0.023	2.101	0.88934	0.27563	32	0.023	2.101	0.88934	0.27563	32	TRUE	TRUE	TRUE	TRUE	TRUE
1	SSKRFQPFQ	541	549	9	HLA-A*02:01	541	0.015	-0.159	0.08472	0.03009	50	0.015	-0.159	0.08472	0.03009	50	TRUE	TRUE	TRUE	TRUE	TRUE
1	SKRFQPFQQ	542	550	9	HLA-A*02:01	542	0.009	0.1	0.10674	0.03552	50	0.009	0.1	0.10674	0.03552	50	TRUE	TRUE	TRUE	TRUE	TRUE
1	KRFQPFQQF	543	551	9	HLA-A*02:01	543	0.054	3.014	0.9733	0.34834	10	0.054	3.014	0.9733	0.34834	10	TRUE	TRUE	TRUE	TRUE	TRUE
1	RFQPFQQFG	544	552	9	HLA-A*02:01	544	0.03	-0.915	0.04923	0.0182	50	0.03	-0.915	0.04923	0.0182	50	TRUE	TRUE	TRUE	TRUE	TRUE
1	FQPFQQFGR	545	553	9	HLA-A*02:01	545	0.081	1.582	0.60606	0.25691	32	0.081	1.582	0.60606	0.25691	32	TRUE	TRUE	TRUE	TRUE	TRUE
1	QPFQQFGRD	546	554	9	HLA-A*02:01	546	0.008	-2.1	0.25326	0.01248	50	0.008	-2.1	0.25326	0.01248	50	TRUE	TRUE	TRUE	TRUE	TRUE
1	PFQQFGRDV	547	555	9	HLA-A*02:01	547	0.019	-0.079	0.31488	0.08787	50	0.019	-0.079	0.31488	0.08787	50	TRUE	TRUE	TRUE	TRUE	TRUE
1	FQQFGRDVS	548	556	9	HLA-A*02:01	548	0.045	-2.442	0.02316	-0.01084	50	0.045	-2.442	0.02316	-0.01084	50	TRUE	TRUE	TRUE	TRUE	TRUE
1	QQFGRDVSD	549	557	9	HLA-A*02:01	549	0.033	-1.72	0.17248	0.02881	50	0.033	-1.72	0.17248	0.02881	50	TRUE	TRUE	TRUE	TRUE	TRUE
1	QFGRDVSDF	550	558	9	HLA-A*02:01	550	0.02	2.503	0.48967	0.19275	50	0.02	2.503	0.48967	0.19275	50	TRUE	TRUE	TRUE	TRUE	TRUE
1	FGRDVSDFT	551	559	9	HLA-A*02:01	551	0.042	-1.067	0.03858	0.02401	50	0.042	-1.067	0.03858	0.02401	50	TRUE	TRUE	TRUE	TRUE	TRUE
1	GRDVSDFTD	552	560	9	HLA-A*02:01	552	0.006	-2.069	0.40418	0.04522	50	0.006	-2.069	0.40418	0.04522	50	TRUE	TRUE	TRUE	TRUE	TRUE
1	RDVSDFTDS	553	561	9	HLA-A*02:01	553	0.01	-2.261	0.08022	-0.02847	50	0.01	-2.261	0.08022	-0.02847	50	TRUE	TRUE	TRUE	TRUE	TRUE
1	DVSDFTDSV	554	562	9	HLA-A*02:01	554	0.154	0.265	0.97021	0.37892	9	0.154	0.265	0.97021	0.37892	9	TRUE	TRUE	TRUE	TRUE	TRUE
1	VSDFTDSVR	555	563	9	HLA-A*02:01	555	0.018	1.272	0.31492	0.12066	50	0.018	1.272	0.31492	0.12066	50	TRUE	TRUE	TRUE	TRUE	TRUE
1	SDFTDSVRD	556	564	9	HLA-A*02:01	556	0.007	-1.911	0.05467	-0.02847	50	0.007	-1.911	0.05467	-0.02847	50	TRUE	TRUE	TRUE	TRUE	TRUE
1	DFTDSVRDP	557	565	9	HLA-A*02:01	557	0.005	-0.026	0.07272	0.02071	50	0.005	-0.026	0.07272	0.02071	50	TRUE	TRUE	TRUE	TRUE	TRUE
1	FTDSVRDPK	558	566	9	HLA-A*02:01	558	0.038	0.033	0.13018	0.06812	50	0.038	0.033	0.13018	0.06812	50	TRUE	TRUE	TRUE	TRUE	TRUE
1	TDSVRDPKT	559	567	9	HLA-A*02:01	559	0.007	-1.143	0.05818	-0.00848	50	0.007	-1.143	0.05818	-0.00848	50	TRUE	TRUE	TRUE	TRUE	TRUE
1	DSVRDPKTS	560	568	9	HLA-A*02:01	560	0.005	-2.433	0.11146	-0.03075	50	0.005	-2.433	0.11146	-0.03075	50	TRUE	TRUE	TRUE	TRUE	TRUE
1	SVRDPKTSE	561	569	9	HLA-A*02:01	561	0.008	-1.464	0.07569	-0.01157	50	0.008	-1.464	0.07569	-0.01157	50	TRUE	TRUE	TRUE	TRUE	TRUE
1	VRDPKTSEI	562	570	9	HLA-A*02:01	562	0.035	0.681	0.91003	0.25678	32	0.035	0.681	0.91003	0.25678	32	TRUE	TRUE	TRUE	TRUE	TRUE
1	RDPKTSEIL	563	571	9	HLA-A*02:01	563	0.015	0.923	0.94555	0.25082	32	0.015	0.923	0.94555	0.25082	32	TRUE	TRUE	TRUE	TRUE	TRUE
1	DPKTSEILD	564	572	9	HLA-A*02:01	564	0.004	-2.306	0.05969	-0.04022	50	0.004	-2.306	0.05969	-0.04022	50	TRUE	TRUE	TRUE	TRUE	TRUE
1	PKTSEILDI	565	573	9	HLA-A*02:01	565	0.009	-0.071	0.87685	0.20452	32	0.009	-0.071	0.87685	0.20452	32	TRUE	TRUE	TRUE	TRUE	TRUE
1	KTSEILDIS	566	574	9	HLA-A*02:01	566	0.074	-2.309	0.03616	0.02441	50	0.074	-2.309	0.03616	0.02441	50	TRUE	TRUE	TRUE	TRUE	TRUE
1	TSEILDISP	567	575	9	HLA-A*02:01	567	0.01	0.081	0.02834	0.0184	50	0.01	0.081	0.02834	0.0184	50	TRUE	TRUE	TRUE	TRUE	TRUE
1	SEILDISPC	568	576	9	HLA-A*02:01	568	0.032	0.011	0.05032	0.0436	50	0.032	0.011	0.05032	0.0436	50	TRUE	TRUE	TRUE	TRUE	TRUE
1	EILDISPCS	569	577	9	HLA-A*02:01	569	0.043	-2.249	0.03401	-0.00557	50	0.043	-2.249	0.03401	-0.00557	50	TRUE	TRUE	TRUE	TRUE	TRUE
1	ILDISPCSF	570	578	9	HLA-A*02:01	570	0.258	2.234	0.9529	0.52825	5	0.258	2.234	0.9529	0.52825	5	TRUE	TRUE	TRUE	TRUE	TRUE
1	LDISPCSFG	571	579	9	HLA-A*02:01	571	0.012	-1.621	0.04203	-0.01907	50	0.012	-1.621	0.04203	-0.01907	50	TRUE	TRUE	TRUE	TRUE	TRUE
1	DISPCSFGG	572	580	9	HLA-A*02:01	572	0.028	-1.539	0.03929	-0.00164	50	0.028	-1.539	0.03929	-0.00164	50	TRUE	TRUE	TRUE	TRUE	TRUE
1	ISPCSFGGV	573	581	9	HLA-A*02:01	573	0.193	0.224	0.39369	0.28718	32	0.193	0.224	0.39369	0.28718	32	TRUE	TRUE	TRUE	TRUE	TRUE
1	SPCSFGGVS	574	582	9	HLA-A*02:01	574	0.012	-2.479	0.09486	-0.02863	50	0.012	-2.479	0.09486	-0.02863	50	TRUE	TRUE	TRUE	TRUE	TRUE
1	PCSFGGVSV	575	583	9	HLA-A*02:01	575	0.03	-0.095	0.37755	0.11257	50	0.03	-0.095	0.37755	0.11257	50	TRUE	TRUE	TRUE	TRUE	TRUE
1	CSFGGVSVI	576	584	9	HLA-A*02:01	576	0.201	0.501	0.87336	0.41003	7	0.201	0.501	0.87336	0.41003	7	TRUE	TRUE	TRUE	TRUE	TRUE
1	SFGGVSVIT	577	585	9	HLA-A*02:01	577	0.067	-0.655	0.07443	0.06737	50	0.067	-0.655	0.07443	0.06737	50	TRUE	TRUE	TRUE	TRUE	TRUE
1	FGGVSVITP	578	586	9	HLA-A*02:01	578	0.02	-0.366	0.03656	0.01908	50	0.02	-0.366	0.03656	0.01908	50	TRUE	TRUE	TRUE	TRUE	TRUE
1	GGVSVITPG	579	587	9	HLA-A*02:01	579	0.019	-1.868	0.14091	0.00401	50	0.019	-1.868	0.14091	0.00401	50	TRUE	TRUE	TRUE	TRUE	TRUE
1	GVSVITPGT	580	588	9	HLA-A*02:01	580	0.102	-0.78	0.27981	0.14546	50	0.102	-0.78	0.27981	0.14546	50	TRUE	TRUE	TRUE	TRUE	TRUE
1	VSVITPGTN	581	589	9	HLA-A*02:01	581	0.013	-1.165	0.04579	-0.00582	50	0.013	-1.165	0.04579	-0.00582	50	TRUE	TRUE	TRUE	TRUE	TRUE
1	SVITPGTNA	582	590	9	HLA-A*02:01	582	0.15	-0.228	0.13422	0.1745	50	0.15	-0.228	0.13422	0.1745	50	TRUE	TRUE	TRUE	TRUE	TRUE
1	VITPGTNAS	583	591	9	HLA-A*02:01	583	0.055	-2.037	0.09369	0.02515	50	0.055	-2.037	0.09369	0.02515	50	TRUE	TRUE	TRUE	TRUE	TRUE
1	ITPGTNASS	584	592	9	HLA-A*02:01	584	0.041	-2.212	0.05924	-0.00097	50	0.041	-2.212	0.05924	-0.00097	50	TRUE	TRUE	TRUE	TRUE	TRUE
1	TPGTNASSE	585	593	9	HLA-A*02:01	585	0.006	-2.045	0.03248	-0.03782	50	0.006	-2.045	0.03248	-0.03782	50	TRUE	TRUE	TRUE	TRUE	TRUE
1	PGTNASSEV	586	594	9	HLA-A*02:01	586	0.013	-0.458	0.78492	0.17816	50	0.013	-0.458	0.78492	0.17816	50	TRUE	TRUE	TRUE	TRUE	TRUE
1	GTNASSEVA	587	595	9	HLA-A*02:01	587	0.06	-0.975	0.12898	0.06465	50	0.06	-0.975	0.12898	0.06465	50	TRUE	TRUE	TRUE	TRUE	TRUE
1	TNASSEVAV	588	596	9	HLA-A*02:01	588	0.046	0.128	0.06876	0.06467	50	0.046	0.128	0.06876	0.06467	50	TRUE	TRUE	TRUE	TRUE	TRUE
1	NASSEVAVL	589	597	9	HLA-A*02:01	589	0.082	1.05	0.83727	0.29664	16	0.082	1.05	0.83727	0.29664	16	TRUE	TRUE	TRUE	TRUE	TRUE
1	ASSEVAVLY	590	598	9	HLA-A*02:01	590	0.077	3.246	0.97194	0.37684	9	0.077	3.246	0.97194	0.37684	9	TRUE	TRUE	TRUE	TRUE	TRUE
1	SSEVAVLYQ	591	599	9	HLA-A*02:01	591	0.016	-0.002	0.04481	0.02603	50	0.016	-0.002	0.04481	0.02603	50	TRUE	TRUE	TRUE	TRUE	TRUE
1	SEVAVLYQD	592	600	9	HLA-A*02:01	592	0.025	-1.766	0.05801	-0.0061	50	0.025	-1.766	0.05801	-0.0061	50	TRUE	TRUE	TRUE	TRUE	TRUE
1	EVAVLYQDV	593	601	9	HLA-A*02:01	593	0.083	0.32	0.80254	0.27157	32	0.083	0.32	0.80254	0.27157	32	TRUE	TRUE	TRUE	TRUE	TRUE
1	VAVLYQDVN	594	602	9	HLA-A*02:01	594	0.012	-1.29	0.03317	-0.01279	50	0.012	-1.29	0.03317	-0.01279	50	TRUE	TRUE	TRUE	TRUE	TRUE
1	AVLYQDVNC	595	603	9	HLA-A*02:01	595	0.063	0.362	0.023	0.07723	50	0.063	0.362	0.023	0.07723	50	TRUE	TRUE	TRUE	TRUE	TRUE
1	VLYQDVNCT	596	604	9	HLA-A*02:01	596	0.313	-0.283	0.09576	0.32747	16	0.313	-0.283	0.09576	0.32747	16	TRUE	TRUE	TRUE	TRUE	TRUE
1	LYQDVNCTD	597	605	9	HLA-A*02:01	597	0.017	-1.532	0.13067	0.0081	50	0.017	-1.532	0.13067	0.0081	50	TRUE	TRUE	TRUE	TRUE	TRUE
1	YQDVNCTDV	598	606	9	HLA-A*02:01	598	0.365	0.242	0.82705	0.55714	4	0.365	0.242	0.82705	0.55714	4	TRUE	TRUE	TRUE	TRUE	TRUE
1	QDVNCTDVS	599	607	9	HLA-A*02:01	599	0.006	-2.514	0.02423	-0.0514	50	0.006	-2.514	0.02423	-0.0514	50	TRUE	TRUE	TRUE	TRUE	TRUE
1	DVNCTDVST	600	608	9	HLA-A*02:01	600	0.025	-1.026	0.09233	0.02012	50	0.025	-1.026	0.09233	0.02012	50	TRUE	TRUE	TRUE	TRUE	TRUE

seq #	peptide	start	end	peptide length	allele	peptide index	NetCTLpan MH	NetCTLpan tap	NetCTLpan clea	NetCTLpan con	NetCTLpan per	legacy mhc_pr	legacy tap_pre	legacy cleavag	legacy combine	legacy %_rank	equal mhc_pre	equal tap_pred	equal cleavage	equal combine	equal %_rank
1	VNCTDVSTA	601	609	9	HLA-A*02:01	601	0.025	-0.756	0.23087	0.05805	50	0.025	-0.756	0.23087	0.05805	50	TRUE	TRUE	TRUE	TRUE	TRUE
1	NCTDVSTAI	602	610	9	HLA-A*02:01	602	0.069	0.603	0.92204	0.29154	16	0.069	0.603	0.92204	0.29154	16	TRUE	TRUE	TRUE	TRUE	TRUE
1	CTDVSTAIH	603	611	9	HLA-A*02:01	603	0.032	-0.633	0.08768	0.0359	50	0.032	-0.633	0.08768	0.0359	50	TRUE	TRUE	TRUE	TRUE	TRUE
1	TDVSTAIHA	604	612	9	HLA-A*02:01	604	0.018	-0.793	0.13594	0.02876	50	0.018	-0.793	0.13594	0.02876	50	TRUE	TRUE	TRUE	TRUE	TRUE
1	DVSTAIHAD	605	613	9	HLA-A*02:01	605	0.013	-1.987	0.11565	-0.01065	50	0.013	-1.987	0.11565	-0.01065	50	TRUE	TRUE	TRUE	TRUE	TRUE
1	VSTAIHADQ	606	614	9	HLA-A*02:01	606	0.017	-0.178	0.04742	0.02322	50	0.017	-0.178	0.04742	0.02322	50	TRUE	TRUE	TRUE	TRUE	TRUE
1	STAIHADQL	607	615	9	HLA-A*02:01	607	0.12	1.05	0.27274	0.20762	32	0.12	1.05	0.27274	0.20762	32	TRUE	TRUE	TRUE	TRUE	TRUE
1	TAIHADQLT	608	616	9	HLA-A*02:01	608	0.028	-0.551	0.02386	0.01959	50	0.028	-0.551	0.02386	0.01959	50	TRUE	TRUE	TRUE	TRUE	TRUE
1	AIHADQLTP	609	617	9	HLA-A*02:01	609	0.026	0.417	0.04462	0.04646	50	0.026	0.417	0.04462	0.04646	50	TRUE	TRUE	TRUE	TRUE	TRUE
1	IHADQLTPA	610	618	9	HLA-A*02:01	610	0.099	-0.334	0.1486	0.12409	50	0.099	-0.334	0.1486	0.12409	50	TRUE	TRUE	TRUE	TRUE	TRUE
1	HADQLTPAW	611	619	9	HLA-A*02:01	611	0.05	0.679	0.90017	0.26951	32	0.05	0.679	0.90017	0.26951	32	TRUE	TRUE	TRUE	TRUE	TRUE
1	ADQLTPAWR	612	620	9	HLA-A*02:01	612	0.013	1.266	0.06171	0.05854	50	0.013	1.266	0.06171	0.05854	50	TRUE	TRUE	TRUE	TRUE	TRUE
1	DQLTPAWRI	613	621	9	HLA-A*02:01	613	0.174	0.466	0.55595	0.31074	16	0.174	0.466	0.55595	0.31074	16	TRUE	TRUE	TRUE	TRUE	TRUE
1	QLTPAWRIY	614	622	9	HLA-A*02:01	614	0.048	2.782	0.8899	0.31778	16	0.048	2.782	0.8899	0.31778	16	TRUE	TRUE	TRUE	TRUE	TRUE
1	LTPAWRIYS	615	623	9	HLA-A*02:01	615	0.08	-2.327	0.02743	0.028	50	0.08	-2.327	0.02743	0.028	50	TRUE	TRUE	TRUE	TRUE	TRUE
1	TPAWRIYST	616	624	9	HLA-A*02:01	616	0.053	-0.966	0.16119	0.06512	50	0.053	-0.966	0.16119	0.06512	50	TRUE	TRUE	TRUE	TRUE	TRUE
1	PAWRIYSTG	617	625	9	HLA-A*02:01	617	0.014	-1.641	0.29204	0.03868	50	0.014	-1.641	0.29204	0.03868	50	TRUE	TRUE	TRUE	TRUE	TRUE
1	AWRIYSTGN	618	626	9	HLA-A*02:01	618	0.012	-1.021	0.02939	-0.00691	50	0.012	-1.021	0.02939	-0.00691	50	TRUE	TRUE	TRUE	TRUE	TRUE
1	WRIYSTGNN	619	627	9	HLA-A*02:01	619	0.006	-0.913	0.02595	-0.01099	50	0.006	-0.913	0.02595	-0.01099	50	TRUE	TRUE	TRUE	TRUE	TRUE
1	RIYSTGNNV	620	628	9	HLA-A*02:01	620	0.378	0.818	0.76165	0.56982	4	0.378	0.818	0.76165	0.56982	4	TRUE	TRUE	TRUE	TRUE	TRUE
1	IYSTGNNVF	621	629	9	HLA-A*02:01	621	0.028	2.95	0.96772	0.31949	16	0.028	2.95	0.96772	0.31949	16	TRUE	TRUE	TRUE	TRUE	TRUE
1	YSTGNNVFQ	622	630	9	HLA-A*02:01	622	0.027	-0.112	0.04124	0.03348	50	0.027	-0.112	0.04124	0.03348	50	TRUE	TRUE	TRUE	TRUE	TRUE
1	STGNNVFQT	623	631	9	HLA-A*02:01	623	0.087	-0.889	0.27178	0.12592	50	0.087	-0.889	0.27178	0.12592	50	TRUE	TRUE	TRUE	TRUE	TRUE
1	TGNNVFQTQ	624	632	9	HLA-A*02:01	624	0.012	-0.433	0.53096	0.12064	50	0.012	-0.433	0.53096	0.12064	50	TRUE	TRUE	TRUE	TRUE	TRUE
1	GNNVFQTQA	625	633	9	HLA-A*02:01	625	0.03	-0.951	0.29956	0.07363	50	0.03	-0.951	0.29956	0.07363	50	TRUE	TRUE	TRUE	TRUE	TRUE
1	NNVFQTQAG	626	634	9	HLA-A*02:01	626	0.008	-1.463	0.04177	-0.01918	50	0.008	-1.463	0.04177	-0.01918	50	TRUE	TRUE	TRUE	TRUE	TRUE
1	NVFQTQAGC	627	635	9	HLA-A*02:01	627	0.064	0.443	0.03004	0.08183	50	0.064	0.443	0.03004	0.08183	50	TRUE	TRUE	TRUE	TRUE	TRUE
1	VFQTQAGCL	628	636	9	HLA-A*02:01	628	0.048	1.293	0.28491	0.14443	50	0.048	1.293	0.28491	0.14443	50	TRUE	TRUE	TRUE	TRUE	TRUE
1	FQTQAGCLI	629	637	9	HLA-A*02:01	629	0.306	0.526	0.43456	0.41693	7	0.306	0.526	0.43456	0.41693	7	TRUE	TRUE	TRUE	TRUE	TRUE
1	QTQAGCLIG	630	638	9	HLA-A*02:01	630	0.019	-1.541	0.04526	-0.00934	50	0.019	-1.541	0.04526	-0.00934	50	TRUE	TRUE	TRUE	TRUE	TRUE
1	TQAGCLIGA	631	639	9	HLA-A*02:01	631	0.488	-0.513	0.07488	0.49202	5	0.488	-0.513	0.07488	0.49202	5	TRUE	TRUE	TRUE	TRUE	TRUE
1	QAGCLIGAE	632	640	9	HLA-A*02:01	632	0.013	-1.653	0.0243	-0.02286	50	0.013	-1.653	0.0243	-0.02286	50	TRUE	TRUE	TRUE	TRUE	TRUE
1	AGCLIGAEH	633	641	9	HLA-A*02:01	633	0.015	-0.698	0.03634	0.00573	50	0.015	-0.698	0.03634	0.00573	50	TRUE	TRUE	TRUE	TRUE	TRUE
1	GCLIGAEHV	634	642	9	HLA-A*02:01	634	0.069	0.328	0.61418	0.21539	32	0.069	0.328	0.61418	0.21539	32	TRUE	TRUE	TRUE	TRUE	TRUE
1	CLIGAEHVD	635	643	9	HLA-A*02:01	635	0.069	-1.798	0.05499	0.03642	50	0.069	-1.798	0.05499	0.03642	50	TRUE	TRUE	TRUE	TRUE	TRUE
1	LIGAEHVDT	636	644	9	HLA-A*02:01	636	0.066	-0.743	0.05504	0.05981	50	0.066	-0.743	0.05504	0.05981	50	TRUE	TRUE	TRUE	TRUE	TRUE
1	IGAEHVDTS	637	645	9	HLA-A*02:01	637	0.025	-2.424	0.05311	-0.02365	50	0.025	-2.424	0.05311	-0.02365	50	TRUE	TRUE	TRUE	TRUE	TRUE
1	GAEHVDTSY	638	646	9	HLA-A*02:01	638	0.027	2.663	0.97413	0.31276	16	0.027	2.663	0.97413	0.31276	16	TRUE	TRUE	TRUE	TRUE	TRUE
1	AEHVDTSYE	639	647	9	HLA-A*02:01	639	0.006	-1.557	0.04936	-0.02182	50	0.006	-1.557	0.04936	-0.02182	50	TRUE	TRUE	TRUE	TRUE	TRUE
1	EHVDTSYEC	640	648	9	HLA-A*02:01	640	0.017	0.05	0.20689	0.0648	50	0.017	0.05	0.20689	0.0648	50	TRUE	TRUE	TRUE	TRUE	TRUE
1	HVDTSYECD	641	649	9	HLA-A*02:01	641	0.011	-2.111	0.0233	-0.03653	50	0.011	-2.111	0.0233	-0.03653	50	TRUE	TRUE	TRUE	TRUE	TRUE
1	VDTSYECDI	642	650	9	HLA-A*02:01	642	0.02	0.23	0.07526	0.04268	50	0.02	0.23	0.07526	0.04268	50	TRUE	TRUE	TRUE	TRUE	TRUE
1	DTSYECDIP	643	651	9	HLA-A*02:01	643	0.005	-0.19	0.0229	0.0054	50	0.005	-0.19	0.0229	0.0054	50	TRUE	TRUE	TRUE	TRUE	TRUE
1	TSYECDIPI	644	652	9	HLA-A*02:01	644	0.248	0.602	0.21986	0.31252	16	0.248	0.602	0.21986	0.31252	16	TRUE	TRUE	TRUE	TRUE	TRUE
1	SYECDIPIG	645	653	9	HLA-A*02:01	645	0.012	-1.212	0.03242	-0.011	50	0.012	-1.212	0.03242	-0.011	50	TRUE	TRUE	TRUE	TRUE	TRUE
1	YECDIPIGA	646	654	9	HLA-A*02:01	646	0.086	-0.628	0.25498	0.12767	50	0.086	-0.628	0.25498	0.12767	50	TRUE	TRUE	TRUE	TRUE	TRUE
1	ECDIPIGAG	647	655	9	HLA-A*02:01	647	0.01	-1.807	0.04092	-0.02597	50	0.01	-1.807	0.04092	-0.02597	50	TRUE	TRUE	TRUE	TRUE	TRUE
1	CDIPIGAGI	648	656	9	HLA-A*02:01	648	0.055	0.088	0.58093	0.18791	50	0.055	0.088	0.58093	0.18791	50	TRUE	TRUE	TRUE	TRUE	TRUE
1	DIPIGAGIC	649	657	9	HLA-A*02:01	649	0.019	-0.195	0.02681	0.02016	50	0.019	-0.195	0.02681	0.02016	50	TRUE	TRUE	TRUE	TRUE	TRUE
1	IPIGAGICA	650	658	9	HLA-A*02:01	650	0.047	-0.887	0.61	0.16207	50	0.047	-0.887	0.61	0.16207	50	TRUE	TRUE	TRUE	TRUE	TRUE
1	PIGAGICAS	651	659	9	HLA-A*02:01	651	0.024	-2.751	0.02588	-0.03895	50	0.024	-2.751	0.02588	-0.03895	50	TRUE	TRUE	TRUE	TRUE	TRUE
1	IGAGICASY	652	660	9	HLA-A*02:01	652	0.061	2.553	0.94476	0.3374	1.60E+01	0.061	2.553	0.94476	0.3374	16	TRUE	TRUE	TRUE	TRUE	TRUE
1	GAGICASYH	653	661	9	HLA-A*02:01	653	0.024	-0.816	0.04006	0.01261	50	0.024	-0.816	0.04006	0.01261	50	TRUE	TRUE	TRUE	TRUE	TRUE
1	AGICASYHT	654	662	9	HLA-A*02:01	654	0.041	-0.789	0.07395	0.03791	50	0.041	-0.789	0.07395	0.03791	50	TRUE	TRUE	TRUE	TRUE	TRUE
1	GICASYHTV	655	663	9	HLA-A*02:01	655	0.465	0.427	0.73394	0.64081	3	0.465	0.427	0.73394	0.64081	3	TRUE	TRUE	TRUE	TRUE	TRUE
1	ICASYHTVS	656	664	9	HLA-A*02:01	656	0.019	-2.215	0.05444	-0.02413	50	0.019	-2.215	0.05444	-0.02413	50	TRUE	TRUE	TRUE	TRUE	TRUE
1	CASYHTVSL	657	665	9	HLA-A*02:01	657	0.168	0.971	0.94579	0.40508	8	0.168	0.971	0.94579	0.40508	8	TRUE	TRUE	TRUE	TRUE	TRUE
1	ASYHTVSLL	658	666	9	HLA-A*02:01	658	0.221	1.319	0.97032	0.4723	6	0.221	1.319	0.97032	0.4723	6	TRUE	TRUE	TRUE	TRUE	TRUE
1	SYHTVSLLR	659	667	9	HLA-A*02:01	659	0.033	1.986	0.97263	0.30149	16	0.033	1.986	0.97263	0.30149	16	TRUE	TRUE	TRUE	TRUE	TRUE
1	YHTVSLLRS	660	668	9	HLA-A*02:01	660	0.019	-2.24	0.0298	-0.03029	50	0.019	-2.24	0.0298	-0.03029	50	TRUE	TRUE	TRUE	TRUE	TRUE
1	HTVSLLRST	661	669	9	HLA-A*02:01	661	0.07	-0.792	0.0283	0.05657	50	0.07	-0.792	0.0283	0.05657	50	TRUE	TRUE	TRUE	TRUE	TRUE
1	TVSLLRSTS	662	670	9	HLA-A*02:01	662	0.033	-2.155	0.0364	-0.01269	50	0.033	-2.155	0.0364	-0.01269	50	TRUE	TRUE	TRUE	TRUE	TRUE
1	VSLLRSTSQ	663	671	9	HLA-A*02:01	663	0.021	0.025	0.0343	0.02934	50	0.021	0.025	0.0343	0.02934	50	TRUE	TRUE	TRUE	TRUE	TRUE
1	SLLRSTSQK	664	672	9	HLA-A*02:01	664	0.036	0.602	0.89778	0.25305	32	0.036	0.602	0.89778	0.25305	32	TRUE	TRUE	TRUE	TRUE	TRUE
1	LLRSTSQKS	665	673	9	HLA-A*02:01	665	0.046	-2.119	0.04003	0.00203	50	0.046	-2.119	0.04003	0.00203	50	TRUE	TRUE	TRUE	TRUE	TRUE
1	LRSTSQKSI	666	674	9	HLA-A*02:01	666	0.018	0.789	0.40834	0.1296	50	0.018	0.789	0.40834	0.1296	50	TRUE	TRUE	TRUE	TRUE	TRUE
1	RSTSQKSIV	667	675	9	HLA-A*02:01	667	0.04	0.611	0.41911	0.14957	50	0.04	0.611	0.41911	0.14957	50	TRUE	TRUE	TRUE	TRUE	TRUE
1	STSQKSIVA	668	676	9	HLA-A*02:01	668	0.076	-0.376	0.42319	0.16182	50	0.076	-0.376	0.42319	0.16182	50	TRUE	TRUE	TRUE	TRUE	TRUE
1	TSQKSIVAY	669	677	9	HLA-A*02:01	669	0.04	2.966	0.96331	0.33089	16	0.04	2.966	0.96331	0.33089	16	TRUE	TRUE	TRUE	TRUE	TRUE
1	SQKSIVAYT	670	678	9	HLA-A*02:01	670	0.149	-0.569	0.07278	0.15115	50	0.149	-0.569	0.07278	0.15115	50	TRUE	TRUE	TRUE	TRUE	TRUE
1	QKSIVAYTM	671	679	9	HLA-A*02:01	671	0.042	0.417	0.97217	0.27116	32	0.042	0.417	0.97217	0.27116	32	TRUE	TRUE	TRUE	TRUE	TRUE
1	KSIVAYTMS	672	680	9	HLA-A*02:01	672	0.034	-2.078	0.0516	-0.00634	50	0.034	-2.078	0.0516	-0.00634	50	TRUE	TRUE	TRUE	TRUE	TRUE
1	SIVAYTMSL	673	681	9	HLA-A*02:01	673	0.674	1.315	0.97492	0.92623	0.8	0.674	1.315	0.97492	0.92623	0.8	TRUE	TRUE	TRUE	TRUE	TRUE
1	IVAYTMSLG	674	682	9	HLA-A*02:01	674	0.061	-1.163	0.03791	0.04045	50	0.061	-1.163	0.03791	0.04045	50	TRUE	TRUE	TRUE	TRUE	TRUE
1	VAYTMSLGA	675	683	9	HLA-A*02:01	675	0.26	-0.225	0.13264	0.28422	32	0.26	-0.225	0.13264	0.28422	32	TRUE	TRUE	TRUE	TRUE	TRUE
1	AYTMSLGAD	676	684	9	HLA-A*02:01	676	0.009	-1.396	0.02328	-0.02066	50	0.009	-1.396	0.02328	-0.02066	50	TRUE	TRUE	TRUE	TRUE	TRUE
1	YTMSLGADS	677	685	9	HLA-A*02:01	677	0.085	-2.068	0.02464	0.03884	50	0.085	-2.068	0.02464	0.03884	50	TRUE	TRUE	TRUE	TRUE	TRUE
1	TMSLGADSS	678	686	9	HLA-A*02:01	678	0.073	-2.201	0.0299	0.0247	50	0.073	-2.201	0.0299	0.0247	50	TRUE	TRUE	TRUE	TRUE	TRUE
1	MSLGADSSI	679	687	9	HLA-A*02:01	679	0.202	0.65	0.77205	0.39196	8	0.202	0.65	0.77205	0.39196	8	TRUE	TRUE	TRUE	TRUE	TRUE
1	SLGADSSIA	680	688	9	HLA-A*02:01	680	0.196	-0.59	0.17097	0.21972	32	0.196	-0.59	0.17097	0.21972	32	TRUE	TRUE	TRUE	TRUE	TRUE
1	LGADSSIAY	681	689	9	HLA-A*02:01	681	0.036	2.733	0.9416	0.31619	16	0.036	2.733	0.9416	0.31619	16	TRUE	TRUE	TRUE	TRUE	TRUE
1	GADSSIAYS	682	690	9	HLA-A*02:01	682	0.059	-2.5	0.03721	0.00487	50	0.059	-2.5	0.03721	0.00487	50	TRUE	TRUE	TRUE	TRUE	TRUE
1	ADSSIAYSN	683	691	9	HLA-A*02:01	683	0.014	-1.513	0.03536	-0.01587	50	0.014	-1.513	0.03536	-0.01587	50	TRUE	TRUE	TRUE	TRUE	TRUE
1	DSSIAYSNN	684	692	9	HLA-A*02:01	684	0.008	-1.441	0.02489	-0.02242	50	0.008	-1.441	0.02489	-0.02242	50	TRUE	TRUE	TRUE	TRUE	TRUE
1	SSIAYSNNT	685	693	9	HLA-A*02:01	685	0.078	-0.682	0.04123	0.07023	50	0.078	-0.682	0.04123	0.07023	50	TRUE	TRUE	TRUE	TRUE	TRUE
1	SIAYSNNTI	686	694	9	HLA-A*02:01	686	0.299	0.787	0.71102	0.47866	6	0.299	0.787	0.71102	0.47866	6	TRUE	TRUE	TRUE	TRUE	TRUE
1	IAYSNNTIA	687	695	9	HLA-A*02:01	687	0.131	-0.219	0.257	0.18335	50	0.131	-0.219	0.257	0.18335	50	TRUE	TRUE	TRUE	TRUE	TRUE
1	AYSNNTIAI	688	696	9	HLA-A*02:01	688	0.079	0.948	0.60339	0.23846	32	0.079	0.948	0.60339	0.23846	32	TRUE	TRUE	TRUE	TRUE	TRUE
1	YSNNTIAIP	689	697	9	HLA-A*02:01	689	0.025	0.185	0.04297	0.03929	50	0.025	0.185	0.04297	0.03929	50	TRUE	TRUE	TRUE	TRUE	TRUE
1	SNNTIAIPT	690	698	9	HLA-A*02:01	690	0.033	-0.951	0.03401	0.01688	50	0.033	-0.951	0.03401	0.01688	50	TRUE	TRUE	TRUE	TRUE	TRUE
1	NNTIAIPTN	691	699	9	HLA-A*02:01	691	0.007	-1.413	0.03047	-0.02147	50	0.007	-1.413	0.03047	-0.02147	50	TRUE	TRUE	TRUE	TRUE	TRUE
1	NTIAIPTNF	692	700	9	HLA-A*02:01	692	0.106	2.76	0.72981	0.33921	16	0.106	2.76	0.72981	0.33921	16	TRUE	TRUE	TRUE	TRUE	TRUE
1	TIAIPTNFS	693	701	9	HLA-A*02:01	693	0.057	-1.98	0.02353	0.01279	50	0.057	-1.98	0.02353	0.01279	50	TRUE	TRUE	TRUE	TRUE	TRUE
1	IAIPTNFSI	694	702	9	HLA-A*02:01	694	0.383	0.689	0.95057	0.6141	3	0.383	0.689	0.95057	0.6141	3	TRUE	TRUE	TRUE	TRUE	TRUE
1	AIPTNFSIS	695	703	9	HLA-A*02:01	695	0.039	-1.979	5.81E-02	2.60E-03	50	0.039	-1.979	0.05809	0.0026	50	TRUE	TRUE	TRUE	TRUE	TRUE
1	IPTNFSISI	696	704	9	HLA-A*02:01	696	0.084	0.371	9.70E-01	3.12E-01	16	0.084	0.371	0.96988	0.3115	16	TRUE	TRUE	TRUE	TRUE	TRUE
1	PTNFSISIT	697	705	9	HLA-A*02:01	697	0.015	-1.292	3.13E-02	-1.03E-02	50	0.015	-1.292	0.03126	-0.01027	50	TRUE	TRUE	TRUE	TRUE	TRUE
1	TNFSISITT	698	706	9	HLA-A*02:01	698	0.049	-0.912	1.98E-01	7.07E-02	50	0.049	-0.912	0.19783	0.07071	50	TRUE	TRUE	TRUE	TRUE	TRUE
1	NFSISITTE	699	707	9	HLA-A*02:01	699	0.016	-1.355	2.64E-02	-1.19E-02	50	0.016	-1.355	0.02638	-0.01194	50	TRUE	TRUE	TRUE	TRUE	TRUE
1	FSISITTEV	700	708	9	HLA-A*02:01	700	0.666	0.47	7.89E-01	8.55E-01	0.8	0.666	0.47	0.789	0.85527	0.8	TRUE	TRUE	TRUE	TRUE	TRUE
1	SISITTEVM	701	709	9	HLA-A*02:01	701	0.138	0.377	6.50E-01	2.94E-01	16	0.138	0.377	0.65026	0.29373	16	TRUE	TRUE	TRUE	TRUE	TRUE
1	ISITTEVMP	702	710	9	HLA-A*02:01	702	0.016	0.291	2.42E-02	2.87E-02	50	0.016	0.291	0.02419	0.02872	50	TRUE	TRUE	TRUE	TRUE	TRUE
1	SITTEVMPV	703	711	9	HLA-A*02:01	703	0.603	0.469	8.95E-01	8.16E-01	1.5	0.603	0.469	0.89464	0.81602	1.5	TRUE	TRUE	TRUE	TRUE	TRUE
1	ITTEVMPVS	704	712	9	HLA-A*02:01	704	0.066	-2.226	3.66E-02	1.86E-02	50	0.066	-2.226	0.03655	0.01857	50	TRUE	TRUE	TRUE	TRUE	TRUE
1	TTEVMPVSM	705	713	9	HLA-A*02:01	705	0.066	0.127	7.02E-01	2.27E-01	32	0.066	0.127	0.70153	0.22702	32	TRUE	TRUE	TRUE	TRUE	TRUE
1	TEVMPVSMA	706	714	9	HLA-A*02:01	706	0.03	-0.604	2.79E-01	7.77E-02	50	0.03	-0.604	0.27929	0.07774	50	TRUE	TRUE	TRUE	TRUE	TRUE
1	EVMPVSMAK	707	715	9	HLA-A*02:01	707	0.062	0.474	9.23E-01	2.81E-01	32	0.062	0.474	0.92263	0.28144	32	TRUE	TRUE	TRUE	TRUE	TRUE
1	VMPVSMAKT	708	716	9	HLA-A*02:01	708	0.175	-0.704	8.85E-02	1.77E-01	50	0.175	-0.704	0.08845	0.1773	50	TRUE	TRUE	TRUE	TRUE	TRUE
1	MPVSMAKTS	709	717	9	HLA-A*02:01	709	0.016	-2.378	3.78E-02	-3.50E-02	50	0.016	-2.378	0.03779	-0.03495	50	TRUE	TRUE	TRUE	TRUE	TRUE
1	PVSMAKTSV	710	718	9	HLA-A*02:01	710	0.037	0.005	4.47E-01	1.38E-01	50	0.037	0.005	0.44653	0.13759	50	TRUE	TRUE	TRUE	TRUE	TRUE
1	VSMAKTSVD	711	719	9	HLA-A*02:01	711	0.018	-1.793	2.72E-02	-2.07E-02	50	0.018	-1.793	0.02719	-0.02071	50	TRUE	TRUE	TRUE	TRUE	TRUE
1	SMAKTSVDC	712	720	9	HLA-A*02:01	712	0.175	0.253	2.95E-02	1.88E-01	50	0.175	0.253	0.02949	0.18796	50	TRUE	TRUE	TRUE	TRUE	TRUE
1	MAKTSVDCN	713	721	9	HLA-A*02:01	713	0.008	-1.164	2.32E-02	-1.59E-02	50	0.008	-1.164	0.02322	-0.01588	50	TRUE	TRUE	TRUE	TRUE	TRUE
1	AKTSVDCNM	714	722	9	HLA-A*02:01	714	0.019	0.498	8.68E-01	2.27E-01	32	0.019	0.498	0.86809	0.22677	32	TRUE	TRUE	TRUE	TRUE	TRUE
1	KTSVDCNMY	715	723	9	HLA-A*02:01	715	0.038	3.16	9.54E-01	3.32E-01	16	0.038	3.16	0.95437	0.33173	16	TRUE	TRUE	TRUE	TRUE	TRUE
1	TSVDCNMYI	716	724	9	HLA-A*02:01	716	0.219	0.733	9.08E-01	4.42E-01	7	0.219	0.733	0.90837	0.44171	7	TRUE	TRUE	TRUE	TRUE	TRUE
1	SVDCNMYIC	717	725	9	HLA-A*02:01	717	0.211	0.04	2.95E-02	2.19E-01	32	0.211	0.04	0.02949	0.21864	32	TRUE	TRUE	TRUE	TRUE	TRUE
1	VDCNMYICG	718	726	9	HLA-A*02:01	718	0.008	-1.578	2.66E-02	-2.55E-02	50	0.008	-1.578	0.02657	-0.02547	50	TRUE	TRUE	TRUE	TRUE	TRUE
1	DCNMYICGD	719	727	9	HLA-A*02:01	719	0.005	-2.158	2.36E-02	-4.36E-02	50	0.005	-2.158	0.02359	-0.04364	50	TRUE	TRUE	TRUE	TRUE	TRUE
1	CNMYICGDS	720	728	9	HLA-A*02:01	720	0.017	-2.513	2.37E-02	-4.05E-02	50	0.017	-2.513	0.02371	-0.04049	50	TRUE	TRUE	TRUE	TRUE	TRUE
1	NMYICGDST	721	729	9	HLA-A*02:01	721	0.23	-0.367	3.46E-02	2.29E-01	32	0.23	-0.367	0.03459	0.22861	32	TRUE	TRUE	TRUE	TRUE	TRUE
1	MYICGDSTE	722	730	9	HLA-A*02:01	722	0.019	-1.044	5.27E-02	4.75E-03	50	0.019	-1.044	0.05266	0.00475	50	TRUE	TRUE	TRUE	TRUE	TRUE
1	YICGDSTEC	723	731	9	HLA-A*02:01	723	0.133	0.168	4.42E-02	1.47E-01	50	0.133	0.168	0.04419	0.14714	50	TRUE	TRUE	TRUE	TRUE	TRUE
1	ICGDSTECA	724	732	9	HLA-A*02:01	724	0.023	-0.707	1.65E-01	4.24E-02	50	0.023	-0.707	0.16458	0.04236	50	TRUE	TRUE	TRUE	TRUE	TRUE
1	CGDSTECAN	725	733	9	HLA-A*02:01	725	0.006	-1.79	2.39E-02	-3.34E-02	50	0.006	-1.79	0.02388	-0.03338	50	TRUE	TRUE	TRUE	TRUE	TRUE
1	GDSTECANL	726	734	9	HLA-A*02:01	726	0.024	0.457	8.32E-01	2.23E-01	32	0.024	0.457	0.83189	0.2226	32	TRUE	TRUE	TRUE	TRUE	TRUE
1	DSTECANLL	727	735	9	HLA-A*02:01	727	0.053	0.613	4.39E-01	1.67E-01	50	0.053	0.613	0.43869	0.16703	50	TRUE	TRUE	TRUE	TRUE	TRUE
1	STECANLLL	728	736	9	HLA-A*02:01	728	0.161	0.703	9.04E-01	3.82E-01	9	0.161	0.703	0.90415	0.38201	9	TRUE	TRUE	TRUE	TRUE	TRUE
1	TECANLLLQ	729	737	9	HLA-A*02:01	729	0.012	-0.199	3.83E-02	1.56E-02	50	0.012	-0.199	0.03828	0.01564	50	TRUE	TRUE	TRUE	TRUE	TRUE
1	ECANLLLQY	730	738	9	HLA-A*02:01	730	0.026	2.768	3.57E-01	1.75E-01	50	0.026	2.768	0.35669	0.17545	50	TRUE	TRUE	TRUE	TRUE	TRUE
1	CANLLLQYG	731	739	9	HLA-A*02:01	731	0.035	-1.645	4.22E-02	3.36E-03	50	0.035	-1.645	0.04215	0.00336	50	TRUE	TRUE	TRUE	TRUE	TRUE
1	ANLLLQYGS	732	740	9	HLA-A*02:01	732	0.022	-2.243	3.04E-02	-2.72E-02	50	0.022	-2.243	0.03036	-0.02724	50	TRUE	TRUE	TRUE	TRUE	TRUE
1	NLLLQYGSF	733	741	9	HLA-A*02:01	733	0.105	2.763	9.34E-01	3.84E-01	8	0.105	2.763	0.9341	0.38425	8	TRUE	TRUE	TRUE	TRUE	TRUE
1	LLLQYGSFC	734	742	9	HLA-A*02:01	734	0.293	0.197	2.65E-02	3.04E-01	16	0.293	0.197	0.02648	0.30388	16	TRUE	TRUE	TRUE	TRUE	TRUE
1	LLQYGSFCT	735	743	9	HLA-A*02:01	735	0.416	-0.666	1.32E-01	4.29E-01	7	0.416	-0.666	0.1315	0.42894	7	TRUE	TRUE	TRUE	TRUE	TRUE
1	LQYGSFCTQ	736	744	9	HLA-A*02:01	736	0.127	0.209	4.08E-01	2.24E-01	32	0.127	0.209	0.40832	0.2241	32	TRUE	TRUE	TRUE	TRUE	TRUE
1	QYGSFCTQL	737	745	9	HLA-A*02:01	737	0.039	1.117	9.74E-01	2.86E-01	32	0.039	1.117	0.97369	0.28601	32	TRUE	TRUE	TRUE	TRUE	TRUE
1	YGSFCTQLN	738	746	9	HLA-A*02:01	738	0.018	-1.655	3.54E-02	-1.54E-02	50	0.018	-1.655	0.03537	-0.01542	50	TRUE	TRUE	TRUE	TRUE	TRUE
1	GSFCTQLNR	739	747	9	HLA-A*02:01	739	0.037	1.406	8.19E-01	2.57E-01	32	0.037	1.406	0.81945	0.25653	32	TRUE	TRUE	TRUE	TRUE	TRUE
1	SFCTQLNRA	740	748	9	HLA-A*02:01	740	0.043	-0.336	7.54E-02	5.16E-02	50	0.043	-0.336	0.07542	0.05157	50	TRUE	TRUE	TRUE	TRUE	TRUE
1	FCTQLNRAL	741	749	9	HLA-A*02:01	741	0.062	0.898	1.04E-01	1.08E-01	50	0.062	0.898	0.10382	0.10781	50	TRUE	TRUE	TRUE	TRUE	TRUE
1	CTQLNRALS	742	750	9	HLA-A*02:01	742	0.027	-2.445	2.80E-02	-2.78E-02	50	0.027	-2.445	0.02796	-0.02783	50	TRUE	TRUE	TRUE	TRUE	TRUE
1	TQLNRALSG	743	751	9	HLA-A*02:01	743	0.061	-1.33	3.90E-02	3.65E-02	50	0.061	-1.33	0.03904	0.03653	50	TRUE	TRUE	TRUE	TRUE	TRUE
1	QLNRALSGI	744	752	9	HLA-A*02:01	744	0.399	0.483	8.61E-01	6.05E-01	4	0.399	0.483	0.86129	0.60487	4	TRUE	TRUE	TRUE	TRUE	TRUE
1	LNRALSGIA	745	753	9	HLA-A*02:01	745	0.023	-0.684	9.16E-02	2.65E-02	50	0.023	-0.684	0.09159	0.02651	50	TRUE	TRUE	TRUE	TRUE	TRUE
1	NRALSGIAA	746	754	9	HLA-A*02:01	746	0.022	-0.227	4.12E-01	1.09E-01	50	0.022	-0.227	0.41193	0.10901	50	TRUE	TRUE	TRUE	TRUE	TRUE
1	RALSGIAAE	747	755	9	HLA-A*02:01	747	0.045	-1.11	4.09E-02	2.64E-02	50	0.045	-1.11	0.04086	0.02644	50	TRUE	TRUE	TRUE	TRUE	TRUE
1	ALSGIAAEQ	748	756	9	HLA-A*02:01	748	0.137	0.186	5.27E-01	2.60E-01	32	0.137	0.186	0.52688	0.2602	32	TRUE	TRUE	TRUE	TRUE	TRUE
1	LSGIAAEQD	749	757	9	HLA-A*02:01	749	0.008	-1.78	2.65E-02	-3.05E-02	50	0.008	-1.78	0.02653	-0.03053	50	TRUE	TRUE	TRUE	TRUE	TRUE
1	SGIAAEQDR	750	758	9	HLA-A*02:01	750	0.008	1.388	4.69E-02	5.33E-02	50	0.008	1.388	0.0469	0.05325	50	TRUE	TRUE	TRUE	TRUE	TRUE

seq #	peptide	start	end	peptide length	allele	peptide index	NetCTLpan MH	NetCTLpan tap	NetCTLpan clea	NetCTLpan con	NetCTLpan per	legacy mhc_pr	legacy tap_pre	legacy cleavag	legacy combine	legacy %_rank	equal mhc_pre	equal tap_pred	equal cleavage	equal combine	equal %_rank
1	GIAAEQDRN	751	759	9	HLA-A*02:01	751	0.013	-1.295	2.28E-02	-1.43E-02	50	0.013	-1.295	0.02276	-0.01425	50	TRUE	TRUE	TRUE	TRUE	TRUE
1	IAAEQDRNT	752	760	9	HLA-A*02:01	752	0.024	-0.643	2.41E-02	1.33E-02	50	0.024	-0.643	0.02406	0.01334	50	TRUE	TRUE	TRUE	TRUE	TRUE
1	AAEQDRNTR	753	761	9	HLA-A*02:01	753	0.009	1.546	1.57E-01	8.30E-02	50	0.009	1.546	0.15726	0.08303	50	TRUE	TRUE	TRUE	TRUE	TRUE
1	AEQDRNTRE	754	762	9	HLA-A*02:01	754	0.007	-1.435	3.38E-02	-2.13E-02	50	0.007	-1.435	0.0338	-0.02127	50	TRUE	TRUE	TRUE	TRUE	TRUE
1	EQDRNTREV	755	763	9	HLA-A*02:01	755	0.104	0.164	5.42E-01	2.30E-01	32	0.104	0.164	0.54208	0.23007	32	TRUE	TRUE	TRUE	TRUE	TRUE
1	QDRNTREVF	756	764	9	HLA-A*02:01	756	0.009	2.122	2.77E-01	1.24E-01	50	0.009	2.122	0.27727	0.12444	50	TRUE	TRUE	TRUE	TRUE	TRUE
1	DRNTREVFA	757	765	9	HLA-A*02:01	757	0.01	-0.788	1.24E-01	1.82E-02	50	0.01	-0.788	0.12403	0.01821	50	TRUE	TRUE	TRUE	TRUE	TRUE
1	RNTREVFAQ	758	766	9	HLA-A*02:01	758	0.01	-0.189	2.69E-02	1.13E-02	50	0.01	-0.189	0.02694	0.01134	50	TRUE	TRUE	TRUE	TRUE	TRUE
1	NTREVFAQV	759	767	9	HLA-A*02:01	759	0.199	0.511	9.21E-01	4.19E-01	7	0.199	0.511	0.92095	0.41899	7	TRUE	TRUE	TRUE	TRUE	TRUE
1	TREVFAQVK	760	768	9	HLA-A*02:01	760	0.008	0.647	8.90E-01	2.24E-01	32	0.008	0.647	0.89012	0.22445	32	TRUE	TRUE	TRUE	TRUE	TRUE
1	REVFAQVKQ	761	769	9	HLA-A*02:01	761	0.011	0.091	8.94E-02	3.34E-02	50	0.011	0.091	0.0894	0.03339	50	TRUE	TRUE	TRUE	TRUE	TRUE
1	EVFAQVKQM	762	770	9	HLA-A*02:01	762	0.053	0.483	8.17E-01	2.49E-01	32	0.053	0.483	0.81685	0.24887	32	TRUE	TRUE	TRUE	TRUE	TRUE
1	VFAQVKQMY	763	771	9	HLA-A*02:01	763	0.034	3.004	8.05E-01	2.90E-01	16	0.034	3.004	0.80484	0.29019	16	TRUE	TRUE	TRUE	TRUE	TRUE
1	FAQVKQMYK	764	772	9	HLA-A*02:01	764	0.036	0.437	2.63E-01	1.06E-01	50	0.036	0.437	0.2625	0.10599	50	TRUE	TRUE	TRUE	TRUE	TRUE
1	AQVKQMYKT	765	773	9	HLA-A*02:01	765	0.127	-0.513	3.19E-02	1.21E-01	50	0.127	-0.513	0.03189	0.12135	50	TRUE	TRUE	TRUE	TRUE	TRUE
1	QVKQMYKTP	766	774	9	HLA-A*02:01	766	0.008	0.408	2.96E-02	2.49E-02	50	0.008	0.408	0.02956	0.02485	50	TRUE	TRUE	TRUE	TRUE	TRUE
1	VKQMYKTPT	767	775	9	HLA-A*02:01	767	0.014	-0.675	2.50E-02	2.75E-03	50	0.014	-0.675	0.02499	0.00275	50	TRUE	TRUE	TRUE	TRUE	TRUE
1	KQMYKTPTL	768	776	9	HLA-A*02:01	768	0.581	1.249	7.84E-01	7.89E-01	1.5	0.581	1.249	0.78403	0.78863	1.5	TRUE	TRUE	TRUE	TRUE	TRUE
1	QMYKTPTLK	769	777	9	HLA-A*02:01	769	0.069	0.887	8.98E-01	2.93E-01	16	0.069	0.887	0.89786	0.29319	16	TRUE	TRUE	TRUE	TRUE	TRUE
1	MYKTPTLKY	770	778	9	HLA-A*02:01	770	0.024	3.185	9.77E-01	3.23E-01	16	0.024	3.185	0.97657	0.32335	16	TRUE	TRUE	TRUE	TRUE	TRUE
1	YKTPTLKYF	771	779	9	HLA-A*02:01	771	0.031	2.564	7.08E-01	2.54E-01	32	0.031	2.564	0.70791	0.25438	32	TRUE	TRUE	TRUE	TRUE	TRUE
1	KTPTLKYFG	772	780	9	HLA-A*02:01	772	0.024	-1.377	2.41E-02	-5.01E-03	50	0.024	-1.377	0.02407	-0.00501	50	TRUE	TRUE	TRUE	TRUE	TRUE
1	TPTLKYFGG	773	781	9	HLA-A*02:01	773	0.011	-1.656	1.47E-01	2.56E-03	50	0.011	-1.656	0.14651	0.00256	50	TRUE	TRUE	TRUE	TRUE	TRUE
1	PTLKYFGGF	774	782	9	HLA-A*02:01	774	0.037	2.061	8.98E-01	2.91E-01	16	0.037	2.061	0.89772	0.29051	16	TRUE	TRUE	TRUE	TRUE	TRUE
1	TLKYFGGFN	775	783	9	HLA-A*02:01	775	0.026	-1.464	2.85E-02	-4.20E-03	50	0.026	-1.464	0.02846	-0.0042	50	TRUE	TRUE	TRUE	TRUE	TRUE
1	LKYFGGFNF	776	784	9	HLA-A*02:01	776	0.055	2.785	8.45E-01	3.15E-01	16	0.055	2.785	0.84478	0.3147	16	TRUE	TRUE	TRUE	TRUE	TRUE
1	KYFGGFNFS	777	785	9	HLA-A*02:01	777	0.098	-1.714	6.60E-01	2.04E-01	32	0.098	-1.714	0.65955	0.20355	32	TRUE	TRUE	TRUE	TRUE	TRUE
1	YFGGFNFSQ	778	786	9	HLA-A*02:01	778	0.065	-0.02	4.31E-01	1.61E-01	50	0.065	-0.02	0.43072	0.16141	50	TRUE	TRUE	TRUE	TRUE	TRUE
1	FGGFNFSQI	779	787	9	HLA-A*02:01	779	0.166	-0.042	7.13E-01	3.25E-01	16	0.166	-0.042	0.71261	0.32529	16	TRUE	TRUE	TRUE	TRUE	TRUE
1	GGFNFSQIL	780	788	9	HLA-A*02:01	780	0.085	0.516	9.06E-01	3.02E-01	16	0.085	0.516	0.90621	0.3018	16	TRUE	TRUE	TRUE	TRUE	TRUE
1	GFNFSQILP	781	789	9	HLA-A*02:01	781	0.018	-0.031	3.44E-02	2.50E-02	50	0.018	-0.031	0.03443	0.02497	50	TRUE	TRUE	TRUE	TRUE	TRUE
1	FNFSQILPD	782	790	9	HLA-A*02:01	782	0.032	-1.981	3.83E-02	-8.90E-03	50	0.032	-1.981	0.03832	-0.0089	50	TRUE	TRUE	TRUE	TRUE	TRUE
1	NFSQILPDP	783	791	9	HLA-A*02:01	783	0.015	0.2	2.74E-02	2.62E-02	50	0.015	0.2	0.02741	0.02617	50	TRUE	TRUE	TRUE	TRUE	TRUE
1	FSQILPDPL	784	792	9	HLA-A*02:01	784	0.172	1.009	5.53E-01	3.22E-01	16	0.172	1.009	0.55292	0.32163	16	TRUE	TRUE	TRUE	TRUE	TRUE
1	SQILPDPLK	785	793	9	HLA-A*02:01	785	0.05	0.557	2.25E-01	1.14E-01	50	0.05	0.557	0.22472	0.11449	50	TRUE	TRUE	TRUE	TRUE	TRUE
1	QILPDPLKP	786	794	9	HLA-A*02:01	786	0.02	0.318	7.39E-02	4.46E-02	50	0.02	0.318	0.07389	0.04458	50	TRUE	TRUE	TRUE	TRUE	TRUE
1	ILPDPLKPT	787	795	9	HLA-A*02:01	787	0.281	-0.727	6.63E-01	4.12E-01	7	0.281	-0.727	0.66326	0.41206	7	TRUE	TRUE	TRUE	TRUE	TRUE
1	LPDPLKPTK	788	796	9	HLA-A*02:01	788	0.01	-0.003	9.51E-01	2.24E-01	32	0.01	-0.003	0.95129	0.22397	32	TRUE	TRUE	TRUE	TRUE	TRUE
1	PDPLKPTKR	789	797	9	HLA-A*02:01	789	0.004	0.73	5.82E-01	1.53E-01	50	0.004	0.73	0.5817	0.15313	50	TRUE	TRUE	TRUE	TRUE	TRUE
1	DPLKPTKRS	790	798	9	HLA-A*02:01	790	0.006	-2.993	2.76E-01	-6.71E-03	50	0.006	-2.993	0.27606	-0.00671	50	TRUE	TRUE	TRUE	TRUE	TRUE
1	PLKPTKRSF	791	799	9	HLA-A*02:01	791	0.017	1.933	7.64E-01	2.37E-01	32	0.017	1.933	0.76433	0.2373	32	TRUE	TRUE	TRUE	TRUE	TRUE
1	LKPTKRSFI	792	800	9	HLA-A*02:01	792	0.015	0.428	7.94E-02	4.36E-02	50	0.015	0.428	0.07944	0.04357	50	TRUE	TRUE	TRUE	TRUE	TRUE
1	KPTKRSFIE	793	801	9	HLA-A*02:01	793	0.01	-1.751	5.98E-02	-2.03E-02	50	0.01	-1.751	0.05976	-0.02033	50	TRUE	TRUE	TRUE	TRUE	TRUE
1	PTKRSFIED	794	802	9	HLA-A*02:01	794	0.005	-2.218	3.02E-02	-4.37E-02	50	0.005	-2.218	0.03015	-0.04367	50	TRUE	TRUE	TRUE	TRUE	TRUE
1	TKRSFIEDL	795	803	9	HLA-A*02:01	795	0.019	0.887	5.77E-01	1.71E-01	50	0.019	0.887	0.5769	0.17098	50	TRUE	TRUE	TRUE	TRUE	TRUE
1	KRSFIEDLL	796	804	9	HLA-A*02:01	796	0.054	1.274	5.82E-01	2.17E-01	32	0.054	1.274	0.58177	0.21675	32	TRUE	TRUE	TRUE	TRUE	TRUE
1	RSFIEDLLF	797	805	9	HLA-A*02:01	797	0.075	3.032	5.94E-01	2.84E-01	32	0.075	3.032	0.59378	0.2844	32	TRUE	TRUE	TRUE	TRUE	TRUE
1	SFIEDLLFN	798	806	9	HLA-A*02:01	798	0.046	-0.894	2.59E-02	2.95E-02	50	0.046	-0.894	0.02594	0.02949	50	TRUE	TRUE	TRUE	TRUE	TRUE
1	FIEDLLFNK	799	807	9	HLA-A*02:01	799	0.109	0.408	4.74E-01	2.26E-01	32	0.109	0.408	0.47394	0.22584	32	TRUE	TRUE	TRUE	TRUE	TRUE
1	IEDLLFNKV	800	808	9	HLA-A*02:01	800	0.085	-0.079	9.44E-01	2.96E-01	16	0.085	-0.079	0.94436	0.2955	16	TRUE	TRUE	TRUE	TRUE	TRUE
1	EDLLFNKVT	801	809	9	HLA-A*02:01	801	0.009	-1.19	4.60E-02	-1.04E-02	50	0.009	-1.19	0.04595	-0.01041	50	TRUE	TRUE	TRUE	TRUE	TRUE
1	DLLFNKVTL	802	810	9	HLA-A*02:01	802	0.145	0.509	9.40E-01	3.69E-01	9	0.145	0.509	0.94032	0.3693	9	TRUE	TRUE	TRUE	TRUE	TRUE
1	LLFNKVTLA	803	811	9	HLA-A*02:01	803	0.742	-0.539	8.14E-01	9.12E-01	0.8	0.742	-0.539	0.81413	0.91171	0.8	TRUE	TRUE	TRUE	TRUE	TRUE
1	LFNKVTLAD	804	812	9	HLA-A*02:01	804	0.027	-1.69	4.72E-02	-4.63E-03	50	0.027	-1.69	0.0472	-0.00463	50	TRUE	TRUE	TRUE	TRUE	TRUE
1	FNKVTLADA	805	813	9	HLA-A*02:01	805	0.034	-0.737	4.82E-02	2.64E-02	50	0.034	-0.737	0.0482	0.02642	50	TRUE	TRUE	TRUE	TRUE	TRUE
1	NKVTLADAG	806	814	9	HLA-A*02:01	806	0.005	-1.336	2.39E-02	-2.30E-02	50	0.005	-1.336	0.0239	-0.02302	50	TRUE	TRUE	TRUE	TRUE	TRUE
1	KVTLADAGF	807	815	9	HLA-A*02:01	807	0.075	2.885	6.68E-01	2.98E-01	16	0.075	2.885	0.66832	0.2975	16	TRUE	TRUE	TRUE	TRUE	TRUE
1	VTLADAGFM	808	816	9	HLA-A*02:01	808	0.129	0.464	1.06E-01	1.65E-01	50	0.129	0.464	0.1062	0.1645	50	TRUE	TRUE	TRUE	TRUE	TRUE
1	TLADAGFMK	809	817	9	HLA-A*02:01	809	0.11	0.527	9.54E-01	3.38E-01	16	0.11	0.527	0.95395	0.33781	16	TRUE	TRUE	TRUE	TRUE	TRUE
1	LADAGFMKQ	810	818	9	HLA-A*02:01	810	0.035	-0.215	4.81E-01	1.38E-01	50	0.035	-0.215	0.48057	0.13775	50	TRUE	TRUE	TRUE	TRUE	TRUE
1	ADAGFMKQY	811	819	9	HLA-A*02:01	811	0.022	2.826	9.53E-01	3.07E-01	16	0.022	2.826	0.9532	0.30712	16	TRUE	TRUE	TRUE	TRUE	TRUE
1	DAGFMKQYG	812	820	9	HLA-A*02:01	812	0.009	-1.668	3.23E-02	-2.54E-02	50	0.009	-1.668	0.0323	-0.02543	50	TRUE	TRUE	TRUE	TRUE	TRUE
1	AGFMKQYGE	813	821	9	HLA-A*02:01	813	0.015	-1.715	3.08E-02	-2.10E-02	50	0.015	-1.715	0.03077	-0.02095	50	TRUE	TRUE	TRUE	TRUE	TRUE
1	GFMKQYGEC	814	822	9	HLA-A*02:01	814	0.032	0.222	2.48E-02	4.31E-02	50	0.032	0.222	0.02479	0.04313	50	TRUE	TRUE	TRUE	TRUE	TRUE
1	FMKQYGECL	815	823	9	HLA-A*02:01	815	0.479	0.949	1.91E-01	5.46E-01	5	0.479	0.949	0.19075	0.54564	5	TRUE	TRUE	TRUE	TRUE	TRUE
1	MKQYGECLG	816	824	9	HLA-A*02:01	816	0.008	-1.436	2.83E-02	-2.15E-02	50	0.008	-1.436	0.0283	-0.02153	50	TRUE	TRUE	TRUE	TRUE	TRUE
1	KQYGECLGD	817	825	9	HLA-A*02:01	817	0.064	-1.41	3.47E-02	3.66E-02	50	0.064	-1.41	0.03473	0.03656	50	TRUE	TRUE	TRUE	TRUE	TRUE
1	QYGECLGDI	818	826	9	HLA-A*02:01	818	0.045	0.75	2.55E-01	1.21E-01	50	0.045	0.75	0.25451	0.12101	50	TRUE	TRUE	TRUE	TRUE	TRUE
1	YGECLGDIN	819	827	9	HLA-A*02:01	819	0.006	-1.795	2.33E-02	-3.36E-02	50	0.006	-1.795	0.02332	-0.03363	50	TRUE	TRUE	TRUE	TRUE	TRUE
1	GECLGDINA	820	828	9	HLA-A*02:01	820	0.017	-0.893	8.81E-02	1.45E-02	50	0.017	-0.893	0.08805	0.01449	50	TRUE	TRUE	TRUE	TRUE	TRUE
1	ECLGDINAR	821	829	9	HLA-A*02:01	821	0.007	1.289	8.31E-02	5.79E-02	50	0.007	1.289	0.08311	0.05792	50	TRUE	TRUE	TRUE	TRUE	TRUE
1	CLGDINARD	822	830	9	HLA-A*02:01	822	0.074	-2.167	9.06E-02	4.02E-02	50	0.074	-2.167	0.09059	0.04021	50	TRUE	TRUE	TRUE	TRUE	TRUE
1	LGDINARDL	823	831	9	HLA-A*02:01	823	0.039	0.509	4.27E-01	1.48E-01	50	0.039	0.509	0.42669	0.14773	50	TRUE	TRUE	TRUE	TRUE	TRUE
1	GDINARDLI	824	832	9	HLA-A*02:01	824	0.029	0.165	1.64E-01	7.01E-02	50	0.029	0.165	0.16441	0.07012	50	TRUE	TRUE	TRUE	TRUE	TRUE
1	DINARDLIC	825	833	9	HLA-A*02:01	825	0.022	-0.297	2.36E-02	1.99E-02	50	0.022	-0.297	0.02363	0.01989	50	TRUE	TRUE	TRUE	TRUE	TRUE
1	INARDLICA	826	834	9	HLA-A*02:01	826	0.045	-0.787	5.61E-02	3.80E-02	50	0.045	-0.787	0.05612	0.03795	50	TRUE	TRUE	TRUE	TRUE	TRUE
1	NARDLICAQ	827	835	9	HLA-A*02:01	827	0.016	0.042	7.72E-02	3.44E-02	50	0.016	0.042	0.07722	0.03443	50	TRUE	TRUE	TRUE	TRUE	TRUE
1	ARDLICAQK	828	836	9	HLA-A*02:01	828	0.018	0.78	9.60E-01	2.54E-01	32	0.018	0.78	0.96011	0.25352	32	TRUE	TRUE	TRUE	TRUE	TRUE
1	RDLICAQKF	829	837	9	HLA-A*02:01	829	0.023	2.643	5.86E-01	2.21E-01	32	0.023	2.643	0.58572	0.22086	32	TRUE	TRUE	TRUE	TRUE	TRUE
1	DLICAQKFN	830	838	9	HLA-A*02:01	830	0.016	-1.411	2.59E-02	-1.35E-02	50	0.016	-1.411	0.02585	-0.01346	50	TRUE	TRUE	TRUE	TRUE	TRUE
1	LICAQKFNG	831	839	9	HLA-A*02:01	831	0.025	-1.393	2.77E-02	-3.60E-03	50	0.025	-1.393	0.02767	-0.0036	50	TRUE	TRUE	TRUE	TRUE	TRUE
1	ICAQKFNGL	832	840	9	HLA-A*02:01	832	0.072	1.063	3.69E-01	1.82E-01	50	0.072	1.063	0.36922	0.18165	50	TRUE	TRUE	TRUE	TRUE	TRUE
1	CAQKFNGLT	833	841	9	HLA-A*02:01	833	0.045	-0.717	3.81E-02	3.57E-02	50	0.045	-0.717	0.0381	0.03565	50	TRUE	TRUE	TRUE	TRUE	TRUE
1	AQKFNGLTV	834	842	9	HLA-A*02:01	834	0.192	0.525	8.72E-01	4.01E-01	8	0.192	0.525	0.87247	0.40143	8	TRUE	TRUE	TRUE	TRUE	TRUE
1	QKFNGLTVL	835	843	9	HLA-A*02:01	835	0.059	1.283	9.35E-01	3.01E-01	16	0.059	1.283	0.93477	0.3014	16	TRUE	TRUE	TRUE	TRUE	TRUE
1	KFNGLTVLP	836	844	9	HLA-A*02:01	836	0.022	0.236	8.36E-02	4.67E-02	50	0.022	0.236	0.08359	0.04671	50	TRUE	TRUE	TRUE	TRUE	TRUE
1	FNGLTVLPP	837	845	9	HLA-A*02:01	837	0.022	-0.205	3.15E-02	2.40E-02	50	0.022	-0.205	0.03146	0.02395	50	TRUE	TRUE	TRUE	TRUE	TRUE
1	NGLTVLPPL	838	846	9	HLA-A*02:01	838	0.181	0.638	7.90E-01	3.75E-01	9	0.181	0.638	0.78984	0.37466	9	TRUE	TRUE	TRUE	TRUE	TRUE
1	GLTVLPPLL	839	847	9	HLA-A*02:01	839	0.48	0.9	9.17E-01	7.09E-01	2	0.48	0.9	0.91697	0.70882	2	TRUE	TRUE	TRUE	TRUE	TRUE
1	LTVLPPLLT	840	848	9	HLA-A*02:01	840	0.063	-0.675	2.72E-02	5.23E-02	50	0.063	-0.675	0.02723	0.05225	50	TRUE	TRUE	TRUE	TRUE	TRUE
1	TVLPPLLTD	841	849	9	HLA-A*02:01	841	0.049	-1.632	3.58E-02	1.63E-02	50	0.049	-1.632	0.03582	0.01626	50	TRUE	TRUE	TRUE	TRUE	TRUE
1	VLPPLLTDD	842	850	9	HLA-A*02:01	842	0.064	-1.739	6.21E-02	3.45E-02	50	0.064	-1.739	0.06207	0.03449	50	TRUE	TRUE	TRUE	TRUE	TRUE
1	LPPLLTDDM	843	851	9	HLA-A*02:01	843	0.023	0.036	6.84E-01	1.78E-01	50	0.023	0.036	0.684	0.1778	50	TRUE	TRUE	TRUE	TRUE	TRUE
1	PPLLTDDMI	844	852	9	HLA-A*02:01	844	0.02	-0.184	4.36E-01	1.13E-01	50	0.02	-0.184	0.43585	0.11347	50	TRUE	TRUE	TRUE	TRUE	TRUE
1	PLLTDDMIA	845	853	9	HLA-A*02:01	845	0.104	-1.123	1.93E-01	1.19E-01	50	0.104	-1.123	0.19266	0.11927	50	TRUE	TRUE	TRUE	TRUE	TRUE
1	LLTDDMIAA	846	854	9	HLA-A*02:01	846	0.669	-0.693	2.39E-01	7.05E-01	2	0.669	-0.693	0.23881	0.70541	2	TRUE	TRUE	TRUE	TRUE	TRUE
1	LTDDMIAAY	847	855	9	HLA-A*02:01	847	0.097	2.779	9.72E-01	3.85E-01	8	0.097	2.779	0.97244	0.38527	8	TRUE	TRUE	TRUE	TRUE	TRUE
1	TDDMIAAYT	848	856	9	HLA-A*02:01	848	0.03	-1.162	3.03E-02	7.77E-03	50	0.03	-1.162	0.03032	0.00777	50	TRUE	TRUE	TRUE	TRUE	TRUE
1	DDMIAAYTA	849	857	9	HLA-A*02:01	849	0.026	-1.084	5.10E-01	1.14E-01	50	0.026	-1.084	0.51048	0.11376	50	TRUE	TRUE	TRUE	TRUE	TRUE
1	DMIAAYTAA	850	858	9	HLA-A*02:01	850	0.222	-0.8	2.62E-01	2.61E-01	32	0.222	-0.8	0.26206	0.26096	32	TRUE	TRUE	TRUE	TRUE	TRUE
1	MIAAYTAAL	851	859	9	HLA-A*02:01	851	0.815	1.008	7.77E-01	1.01E+00	0.2	0.815	1.008	0.77656	1.01493	0.2	TRUE	TRUE	TRUE	TRUE	TRUE
1	IAAYTAALV	852	860	9	HLA-A*02:01	852	0.429	0.443	3.17E-01	5.11E-01	5	0.429	0.443	0.3169	0.51138	5	TRUE	TRUE	TRUE	TRUE	TRUE
1	AAYTAALVS	853	861	9	HLA-A*02:01	853	0.046	-1.853	5.26E-02	1.15E-02	50	0.046	-1.853	0.05257	0.0115	50	TRUE	TRUE	TRUE	TRUE	TRUE
1	AYTAALVSG	854	862	9	HLA-A*02:01	854	0.02	-0.869	6.20E-02	1.22E-02	50	0.02	-0.869	0.06201	0.01223	50	TRUE	TRUE	TRUE	TRUE	TRUE
1	YTAALVSGT	855	863	9	HLA-A*02:01	855	0.296	-0.576	3.17E-02	2.89E-01	32	0.296	-0.576	0.0317	0.28873	32	TRUE	TRUE	TRUE	TRUE	TRUE
1	TAALVSGTA	856	864	9	HLA-A*02:01	856	0.115	-0.539	4.14E-01	1.95E-01	50	0.115	-0.539	0.41352	0.19457	50	TRUE	TRUE	TRUE	TRUE	TRUE
1	AALVSGTAT	857	865	9	HLA-A*02:01	857	0.05	-0.481	4.41E-02	4.79E-02	50	0.05	-0.481	0.04414	0.04791	50	TRUE	TRUE	TRUE	TRUE	TRUE
1	ALVSGTATA	858	866	9	HLA-A*02:01	858	0.546	-0.14	9.24E-01	7.50E-01	1.5	0.546	-0.14	0.92351	0.75029	1.5	TRUE	TRUE	TRUE	TRUE	TRUE
1	LVSGTATAG	859	867	9	HLA-A*02:01	859	0.032	-1.103	6.37E-02	1.88E-02	50	0.032	-1.103	0.06372	0.01876	50	TRUE	TRUE	TRUE	TRUE	TRUE
1	VSGTATAGW	860	868	9	HLA-A*02:01	860	0.03	0.872	7.40E-01	2.18E-01	32	0.03	0.872	0.73994	0.21829	32	TRUE	TRUE	TRUE	TRUE	TRUE
1	SGTATAGWT	861	869	9	HLA-A*02:01	861	0.015	-0.908	2.49E-02	-2.10E-03	50	0.015	-0.908	0.02488	-0.0021	50	TRUE	TRUE	TRUE	TRUE	TRUE
1	GTATAGWTF	862	870	9	HLA-A*02:01	862	0.093	2.405	7.70E-01	3.26E-01	16	0.093	2.405	0.77005	0.32639	16	TRUE	TRUE	TRUE	TRUE	TRUE
1	TATAGWTFG	863	871	9	HLA-A*02:01	863	0.03	-1.43	6.13E-02	8.03E-03	50	0.03	-1.43	0.06126	0.00803	50	TRUE	TRUE	TRUE	TRUE	TRUE
1	ATAGWTFGA	864	872	9	HLA-A*02:01	864	0.549	-0.386	7.03E-01	6.97E-01	2	0.549	-0.386	0.70272	0.69746	2	TRUE	TRUE	TRUE	TRUE	TRUE
1	TAGWTFGAG	865	873	9	HLA-A*02:01	865	0.024	-1.373	7.28E-02	6.05E-03	50	0.024	-1.373	0.07276	0.00605	50	TRUE	TRUE	TRUE	TRUE	TRUE
1	AGWTFGAGA	866	874	9	HLA-A*02:01	866	0.154	-0.476	2.66E-01	2.02E-01	32	0.154	-0.476	0.2661	0.20197	32	TRUE	TRUE	TRUE	TRUE	TRUE
1	GWTFGAGAA	867	875	9	HLA-A*02:01	867	0.03	-0.35	6.19E-02	3.52E-02	50	0.03	-0.35	0.06186	0.03517	50	TRUE	TRUE	TRUE	TRUE	TRUE
1	WTFGAGAAL	868	876	9	HLA-A*02:01	868	0.356	0.988	8.22E-01	5.66E-01	4	0.356	0.988	0.82245	0.56575	4	TRUE	TRUE	TRUE	TRUE	TRUE
1	TFGAGAALQ	869	877	9	HLA-A*02:01	869	0.022	-0.178	2.33E-02	2.28E-02	50	0.022	-0.178	0.02334	0.0228	50	TRUE	TRUE	TRUE	TRUE	TRUE
1	FGAGAALQI	870	878	9	HLA-A*02:01	870	0.194	0.2	1.37E-01	2.30E-01	32	0.194	0.2	0.13695	0.22981	32	TRUE	TRUE	TRUE	TRUE	TRUE
1	GAGAALQIP	871	879	9	HLA-A*02:01	871	0.011	-0.29	2.46E-02	9.28E-03	50	0.011	-0.29	0.02459	0.00928	50	TRUE	TRUE	TRUE	TRUE	TRUE
1	AGAALQIPF	872	880	9	HLA-A*02:01	872	0.047	2.399	1.09E-01	1.31E-01	50	0.047	2.399	0.10855	0.1314	50	TRUE	TRUE	TRUE	TRUE	TRUE
1	GAALQIPFA	873	881	9	HLA-A*02:01	873	0.173	-0.491	3.34E-02	1.68E-01	50	0.173	-0.491	0.03344	0.16825	50	TRUE	TRUE	TRUE	TRUE	TRUE
1	AALQIPFAM	874	882	9	HLA-A*02:01	874	0.247	0.489	7.71E-01	4.33E-01	7	0.247	0.489	0.7707	0.43263	7	TRUE	TRUE	TRUE	TRUE	TRUE
1	ALQIPFAMQ	875	883	9	HLA-A*02:01	875	0.086	0.18	2.29E-01	1.42E-01	50	0.086	0.18	0.22886	0.14199	50	TRUE	TRUE	TRUE	TRUE	TRUE
1	LQIPFAMQM	876	884	9	HLA-A*02:01	876	0.345	0.607	8.81E-01	5.58E-01	4	0.345	0.607	0.88123	0.55845	4	TRUE	TRUE	TRUE	TRUE	TRUE
1	QIPFAMQMA	877	885	9	HLA-A*02:01	877	0.123	-0.361	1.30E-01	1.43E-01	50	0.123	-0.361	0.12966	0.14315	50	TRUE	TRUE	TRUE	TRUE	TRUE
1	IPFAMQMAY	878	886	9	HLA-A*02:01	878	0.029	2.706	9.70E-01	3.15E-01	16	0.029	2.706	0.96985	0.31487	16	TRUE	TRUE	TRUE	TRUE	TRUE
1	PFAMQMAYR	879	887	9	HLA-A*02:01	879	0.018	1.198	2.95E-02	5.46E-02	50	0.018	1.198	0.02954	0.0546	50	TRUE	TRUE	TRUE	TRUE	TRUE
1	FAMQMAYRF	880	888	9	HLA-A*02:01	880	0.203	2.412	1.98E-01	3.08E-01	16	0.203	2.412	0.1975	0.30774	16	TRUE	TRUE	TRUE	TRUE	TRUE
1	AMQMAYRFN	881	889	9	HLA-A*02:01	881	0.039	-1.187	2.24E-02	1.44E-02	50	0.039	-1.187	0.02241	0.01437	50	TRUE	TRUE	TRUE	TRUE	TRUE
1	MQMAYRFNG	882	890	9	HLA-A*02:01	882	0.243	-1.054	2.35E-02	2.22E-01	32	0.243	-1.054	0.02345	0.22193	32	TRUE	TRUE	TRUE	TRUE	TRUE
1	QMAYRFNGI	883	891	9	HLA-A*02:01	883	0.397	0.722	1.41E-01	4.47E-01	6	0.397	0.722	0.1414	0.44687	6	TRUE	TRUE	TRUE	TRUE	TRUE
1	MAYRFNGIG	884	892	9	HLA-A*02:01	884	0.041	-1.158	3.08E-02	1.90E-02	50	0.041	-1.158	0.03084	0.01899	50	TRUE	TRUE	TRUE	TRUE	TRUE
1	AYRFNGIGV	885	893	9	HLA-A*02:01	885	0.052	0.808	2.89E-01	1.37E-01	50	0.052	0.808	0.28898	0.13722	50	TRUE	TRUE	TRUE	TRUE	TRUE
1	YRFNGIGVT	886	894	9	HLA-A*02:01	886	0.043	-0.253	5.18E-01	1.53E-01	50	0.043	-0.253	0.51843	0.15332	50	TRUE	TRUE	TRUE	TRUE	TRUE
1	RFNGIGVTQ	887	895	9	HLA-A*02:01	887	0.026	0.16	1.07E-01	5.40E-02	50	0.026	0.16	0.10657	0.05398	50	TRUE	TRUE	TRUE	TRUE	TRUE
1	FNGIGVTQN	888	896	9	HLA-A*02:01	888	0.016	-1.594	2.76E-02	-1.76E-02	50	0.016	-1.594	0.02764	-0.01763	50	TRUE	TRUE	TRUE	TRUE	TRUE
1	NGIGVTQNV	889	897	9	HLA-A*02:01	889	0.119	0.049	5.51E-01	2.44E-01	32	0.119	0.049	0.55105	0.24421	32	TRUE	TRUE	TRUE	TRUE	TRUE
1	GIGVTQNVL	890	898	9	HLA-A*02:01	890	0.086	0.882	9.55E-01	3.23E-01	16	0.086	0.882	0.95473	0.32286	16	TRUE	TRUE	TRUE	TRUE	TRUE
1	IGVTQNVLY	891	899	9	HLA-A*02:01	891	0.023	2.729	9.31E-01	3.01E-01	16	0.023	2.729	0.93115	0.30073	16	TRUE	TRUE	TRUE	TRUE	TRUE
1	GVTQNVLYE	892	900	9	HLA-A*02:01	892	0.024	-1.633	4.15E-02	-7.50E-03	50	0.024	-1.633	0.04145	-0.0075	50	TRUE	TRUE	TRUE	TRUE	TRUE
1	VTQNVLYEN	893	901	9	HLA-A*02:01	893	0.035	-1.337	3.16E-02	8.67E-03	50	0.035	-1.337	0.03155	0.00867	50	TRUE	TRUE	TRUE	TRUE	TRUE
1	TQNVLYENQ	894	902	9	HLA-A*02:01	894	0.016	-0.098	2.41E-02	1.90E-02	50	0.016	-0.098	0.02411	0.01897	50	TRUE	TRUE	TRUE	TRUE	TRUE
1	QNVLYENQK	895	903	9	HLA-A*02:01	895	0.007	0.413	5.57E-01	1.43E-01	50	0.007	0.413	0.55703	0.14266	50	TRUE	TRUE	TRUE	TRUE	TRUE
1	NVLYENQKQ	896	904	9	HLA-A*02:01	896	0.012	0.039	2.84E-02	1.94E-02	50	0.012	0.039	0.02841	0.01937	50	TRUE	TRUE	TRUE	TRUE	TRUE
1	VLYENQKQI	897	905	9	HLA-A*02:01	897	0.416	0.944	8.39E-01	6.28E-01	3	0.416	0.944	0.83899	0.62837	3	TRUE	TRUE	TRUE	TRUE	TRUE
1	LYENQKQIA	898	906	9	HLA-A*02:01	898	0.012	-0.364	1.25E-01	3.10E-02	50	0.012	-0.364	0.12465	0.03095	50	TRUE	TRUE	TRUE	TRUE	TRUE
1	YENQKQIAN	899	907	9	HLA-A*02:01	899	0.009	-1.517	2.94E-02	-2.23E-02	50	0.009	-1.517	0.02943	-0.0223	50	TRUE	TRUE	TRUE	TRUE	TRUE
1	ENQKQIANQ	900	908	9	HLA-A*02:01	900	0.005	-0.546	4.25E-02	9.10E-04	50	0.005	-0.546	0.04249	0.00091	50	TRUE	TRUE	TRUE	TRUE	TRUE

seq #	peptide	start	end	peptide length	allele	peptide index	NetCTLpan MH	NetCTLpan tap	NetCTLpan clea	NetCTLpan con	NetCTLpan per	legacy mhc_pr	legacy tap_pre	legacy cleavag	legacy combine	legacy %_rank	equal mhc_pre	equal tap_pred	equal cleavage	equal combine	equal %_rank
1	NQKQIANQF	901	909	9	HLA-A*02:01	901	0.047	2.478	5.03E-01	2.22E-01	32	0.047	2.478	0.50293	0.22211	32	TRUE	TRUE	TRUE	TRUE	TRUE
1	QKQIANQFN	902	910	9	HLA-A*02:01	902	0.004	-1.144	2.33E-02	-1.94E-02	50	0.004	-1.144	0.02325	-0.01937	50	TRUE	TRUE	TRUE	TRUE	TRUE
1	KQIANQFNK	903	911	9	HLA-A*02:01	903	0.112	0.667	8.53E-01	3.21E-01	16	0.112	0.667	0.85337	0.32068	16	TRUE	TRUE	TRUE	TRUE	TRUE
1	QIANQFNKA	904	912	9	HLA-A*02:01	904	0.104	-0.276	3.76E-01	1.82E-01	50	0.104	-0.276	0.37627	0.18176	50	TRUE	TRUE	TRUE	TRUE	TRUE
1	IANQFNKAI	905	913	9	HLA-A*02:01	905	0.092	0.467	4.11E-01	1.96E-01	50	0.092	0.467	0.41145	0.19625	50	TRUE	TRUE	TRUE	TRUE	TRUE
1	ANQFNKAIS	906	914	9	HLA-A*02:01	906	0.007	-2.277	2.75E-02	-4.37E-02	50	0.007	-2.277	0.02748	-0.04374	50	TRUE	TRUE	TRUE	TRUE	TRUE
1	NQFNKAISQ	907	915	9	HLA-A*02:01	907	0.052	0.273	5.39E-02	7.10E-02	50	0.052	0.273	0.05393	0.07096	50	TRUE	TRUE	TRUE	TRUE	TRUE
1	QFNKAISQI	908	916	9	HLA-A*02:01	908	0.06	0.762	9.57E-01	2.94E-01	16	0.06	0.762	0.9573	0.29444	16	TRUE	TRUE	TRUE	TRUE	TRUE
1	FNKAISQIQ	909	917	9	HLA-A*02:01	909	0.011	-0.404	4.01E-02	9.91E-03	50	0.011	-0.404	0.04006	0.00991	50	TRUE	TRUE	TRUE	TRUE	TRUE
1	NKAISQIQE	910	918	9	HLA-A*02:01	910	0.005	-1.567	2.93E-02	-2.76E-02	50	0.005	-1.567	0.02931	-0.02758	50	TRUE	TRUE	TRUE	TRUE	TRUE
1	KAISQIQES	911	919	9	HLA-A*02:01	911	0.044	-1.876	5.62E-02	9.75E-03	50	0.044	-1.876	0.05621	0.00975	50	TRUE	TRUE	TRUE	TRUE	TRUE
1	AISQIQESL	912	920	9	HLA-A*02:01	912	0.267	1.324	9.46E-01	5.13E-01	5	0.267	1.324	0.94628	0.51301	5	TRUE	TRUE	TRUE	TRUE	TRUE
1	ISQIQESLT	913	921	9	HLA-A*02:01	913	0.028	-0.52	3.08E-02	2.19E-02	50	0.028	-0.52	0.03076	0.02192	50	TRUE	TRUE	TRUE	TRUE	TRUE
1	SQIQESLTT	914	922	9	HLA-A*02:01	914	0.116	-0.499	6.94E-02	1.19E-01	50	0.116	-0.499	0.0694	0.11914	50	TRUE	TRUE	TRUE	TRUE	TRUE
1	QIQESLTTT	915	923	9	HLA-A*02:01	915	0.142	-0.542	1.88E-01	1.71E-01	50	0.142	-0.542	0.18839	0.17084	50	TRUE	TRUE	TRUE	TRUE	TRUE
1	IQESLTTTS	916	924	9	HLA-A*02:01	916	0.039	-2.34	5.59E-02	-6.91E-03	50	0.039	-2.34	0.05594	-0.00691	50	TRUE	TRUE	TRUE	TRUE	TRUE
1	QESLTTTST	917	925	9	HLA-A*02:01	917	0.013	-0.823	6.20E-01	1.32E-01	50	0.013	-0.823	0.6197	0.13186	50	TRUE	TRUE	TRUE	TRUE	TRUE
1	ESLTTTSTA	918	926	9	HLA-A*02:01	918	0.044	-0.73	8.76E-01	2.23E-01	32	0.044	-0.73	0.87617	0.22289	32	TRUE	TRUE	TRUE	TRUE	TRUE
1	SLTTTSTAL	919	927	9	HLA-A*02:01	919	0.387	0.824	9.75E-01	6.27E-01	3	0.387	0.824	0.97504	0.62698	3	TRUE	TRUE	TRUE	TRUE	TRUE
1	LTTTSTALG	920	928	9	HLA-A*02:01	920	0.024	-1.395	4.84E-02	1.00E-05	50	0.024	-1.395	0.04839	0.00001	50	TRUE	TRUE	TRUE	TRUE	TRUE
1	TTTSTALGK	921	929	9	HLA-A*02:01	921	0.019	0.434	2.60E-01	8.84E-02	50	0.019	0.434	0.26008	0.08837	50	TRUE	TRUE	TRUE	TRUE	TRUE
1	TTSTALGKL	922	930	9	HLA-A*02:01	922	0.078	0.918	8.25E-01	2.87E-01	32	0.078	0.918	0.82492	0.28656	32	TRUE	TRUE	TRUE	TRUE	TRUE
1	TSTALGKLQ	923	931	9	HLA-A*02:01	923	0.009	-0.112	2.40E-02	1.16E-02	50	0.009	-0.112	0.02402	0.0116	50	TRUE	TRUE	TRUE	TRUE	TRUE
1	STALGKLQD	924	932	9	HLA-A*02:01	924	0.012	-1.757	3.33E-02	-2.44E-02	50	0.012	-1.757	0.03332	-0.02443	50	TRUE	TRUE	TRUE	TRUE	TRUE
1	TALGKLQDV	925	933	9	HLA-A*02:01	925	0.182	0.418	2.11E-01	2.40E-01	32	0.182	0.418	0.21106	0.23994	32	TRUE	TRUE	TRUE	TRUE	TRUE
1	ALGKLQDVV	926	934	9	HLA-A*02:01	926	0.397	0.325	3.33E-01	4.80E-01	6	0.397	0.325	0.33325	0.48011	6	TRUE	TRUE	TRUE	TRUE	TRUE
1	LGKLQDVVN	927	935	9	HLA-A*02:01	927	0.005	-1.426	3.84E-02	-2.20E-02	50	0.005	-1.426	0.03835	-0.02202	50	TRUE	TRUE	TRUE	TRUE	TRUE
1	GKLQDVVNQ	928	936	9	HLA-A*02:01	928	0.007	-0.169	3.72E-02	1.11E-02	50	0.007	-0.169	0.0372	0.01114	50	TRUE	TRUE	TRUE	TRUE	TRUE
1	KLQDVVNQN	929	937	9	HLA-A*02:01	929	0.199	-1.251	3.91E-01	2.56E-01	32	0.199	-1.251	0.39062	0.25561	32	TRUE	TRUE	TRUE	TRUE	TRUE
1	LQDVVNQNA	930	938	9	HLA-A*02:01	930	0.17	-0.531	1.67E-01	1.94E-01	50	0.17	-0.531	0.16722	0.19435	50	TRUE	TRUE	TRUE	TRUE	TRUE
1	QDVVNQNAQ	931	939	9	HLA-A*02:01	931	0.004	-0.274	2.95E-02	3.80E-03	50	0.004	-0.274	0.02954	0.0038	50	TRUE	TRUE	TRUE	TRUE	TRUE
1	DVVNQNAQA	932	940	9	HLA-A*02:01	932	0.029	-0.684	4.59E-01	1.15E-01	50	0.029	-0.684	0.45865	0.1151	50	TRUE	TRUE	TRUE	TRUE	TRUE
1	VVNQNAQAL	933	941	9	HLA-A*02:01	933	0.127	0.922	6.00E-01	2.85E-01	32	0.127	0.922	0.60047	0.28516	32	TRUE	TRUE	TRUE	TRUE	TRUE
1	VNQNAQALN	934	942	9	HLA-A*02:01	934	0.007	-1.391	2.99E-02	-2.10E-02	50	0.007	-1.391	0.02991	-0.02104	50	TRUE	TRUE	TRUE	TRUE	TRUE
1	NQNAQALNT	935	943	9	HLA-A*02:01	935	0.06	-0.64	2.78E-02	5.03E-02	50	0.06	-0.64	0.0278	0.05026	50	TRUE	TRUE	TRUE	TRUE	TRUE
1	QNAQALNTL	936	944	9	HLA-A*02:01	936	0.043	0.973	6.44E-01	2.12E-01	32	0.043	0.973	0.64421	0.21227	32	TRUE	TRUE	TRUE	TRUE	TRUE
1	NAQALNTLV	937	945	9	HLA-A*02:01	937	0.191	0.323	2.44E-01	2.54E-01	32	0.191	0.323	0.24359	0.25388	32	TRUE	TRUE	TRUE	TRUE	TRUE
1	AQALNTLVK	938	946	9	HLA-A*02:01	938	0.039	0.842	9.66E-01	2.77E-01	32	0.039	0.842	0.96636	0.27748	32	TRUE	TRUE	TRUE	TRUE	TRUE
1	QALNTLVKQ	939	947	9	HLA-A*02:01	939	0.021	0.132	1.96E-01	6.84E-02	50	0.021	0.132	0.19586	0.06837	50	TRUE	TRUE	TRUE	TRUE	TRUE
1	ALNTLVKQL	940	948	9	HLA-A*02:01	940	0.443	0.975	9.43E-01	6.80E-01	3	0.443	0.975	0.94302	0.67956	3	TRUE	TRUE	TRUE	TRUE	TRUE
1	LNTLVKQLS	941	949	9	HLA-A*02:01	941	0.011	-2.276	2.68E-02	-3.99E-02	50	0.011	-2.276	0.02678	-0.03987	50	TRUE	TRUE	TRUE	TRUE	TRUE
1	NTLVKQLSS	942	950	9	HLA-A*02:01	942	0.029	-2.164	6.82E-02	-9.77E-03	50	0.029	-2.164	0.06815	-0.00977	50	TRUE	TRUE	TRUE	TRUE	TRUE
1	TLVKQLSSN	943	951	9	HLA-A*02:01	943	0.052	-1.098	4.24E-02	3.41E-02	50	0.052	-1.098	0.04239	0.03409	50	TRUE	TRUE	TRUE	TRUE	TRUE
1	LVKQLSSNF	944	952	9	HLA-A*02:01	944	0.04	2.685	2.28E-01	1.59E-01	50	0.04	2.685	0.22836	0.15851	50	TRUE	TRUE	TRUE	TRUE	TRUE
1	VKQLSSNFG	945	953	9	HLA-A*02:01	945	0.007	-1.268	3.14E-02	-1.76E-02	50	0.007	-1.268	0.03141	-0.01763	50	TRUE	TRUE	TRUE	TRUE	TRUE
1	KQLSSNFGA	946	954	9	HLA-A*02:01	946	0.498	-0.288	6.91E-02	5.06E-01	5	0.498	-0.288	0.06909	0.50634	5	TRUE	TRUE	TRUE	TRUE	TRUE
1	QLSSNFGAI	947	955	9	HLA-A*02:01	947	0.34	0.664	8.03E-01	5.37E-01	5	0.34	0.664	0.80307	0.53729	5	TRUE	TRUE	TRUE	TRUE	TRUE
1	LSSNFGAIS	948	956	9	HLA-A*02:01	948	0.038	-2.254	4.50E-02	-8.22E-03	50	0.038	-2.254	0.04502	-0.00822	50	TRUE	TRUE	TRUE	TRUE	TRUE
1	SSNFGAISS	949	957	9	HLA-A*02:01	949	0.036	-2.198	5.22E-02	-7.20E-03	50	0.036	-2.198	0.05222	-0.0072	50	TRUE	TRUE	TRUE	TRUE	TRUE
1	SNFGAISSV	950	958	9	HLA-A*02:01	950	0.197	0.381	9.05E-01	4.10E-01	7	0.197	0.381	0.90494	0.41014	7	TRUE	TRUE	TRUE	TRUE	TRUE
1	NFGAISSVL	951	959	9	HLA-A*02:01	951	0.089	1.05	4.91E-01	2.26E-01	32	0.089	1.05	0.49071	0.22566	32	TRUE	TRUE	TRUE	TRUE	TRUE
1	FGAISSVLN	952	960	9	HLA-A*02:01	952	0.025	-1.519	2.89E-02	-6.48E-03	50	0.025	-1.519	0.02887	-0.00648	50	TRUE	TRUE	TRUE	TRUE	TRUE
1	GAISSVLND	953	961	9	HLA-A*02:01	953	0.018	-1.978	2.87E-02	-2.50E-02	50	0.018	-1.978	0.0287	-0.02499	50	TRUE	TRUE	TRUE	TRUE	TRUE
1	AISSVLNDI	954	962	9	HLA-A*02:01	954	0.377	0.773	7.99E-01	5.76E-01	4	0.377	0.773	0.79893	0.57608	4	TRUE	TRUE	TRUE	TRUE	TRUE
1	ISSVLNDIL	955	963	9	HLA-A*02:01	955	0.084	1.168	4.19E-01	2.07E-01	32	0.084	1.168	0.41882	0.20744	32	TRUE	TRUE	TRUE	TRUE	TRUE
1	SSVLNDILS	956	964	9	HLA-A*02:01	956	0.031	-2.065	5.15E-02	-9.05E-03	50	0.031	-2.065	0.05145	-0.00905	50	TRUE	TRUE	TRUE	TRUE	TRUE
1	SVLNDILSR	957	965	9	HLA-A*02:01	957	0.048	1.748	7.53E-01	2.61E-01	32	0.048	1.748	0.75334	0.2612	32	TRUE	TRUE	TRUE	TRUE	TRUE
1	VLNDILSRL	958	966	9	HLA-A*02:01	958	0.741	0.996	9.71E-01	9.84E-01	0.3	0.741	0.996	0.97061	0.98429	0.3	TRUE	TRUE	TRUE	TRUE	TRUE
1	LNDILSRLD	959	967	9	HLA-A*02:01	959	0.009	-2.093	2.63E-02	-3.74E-02	50	0.009	-2.093	0.02631	-0.03741	50	TRUE	TRUE	TRUE	TRUE	TRUE
1	NDILSRLDK	960	968	9	HLA-A*02:01	960	0.006	0.311	1.13E-01	3.93E-02	50	0.006	0.311	0.11325	0.03926	50	TRUE	TRUE	TRUE	TRUE	TRUE
1	DILSRLDKV	961	969	9	HLA-A*02:01	961	0.124	0.286	4.47E-01	2.32E-01	32	0.124	0.286	0.44748	0.23183	32	TRUE	TRUE	TRUE	TRUE	TRUE
1	ILSRLDKVE	962	970	9	HLA-A*02:01	962	0.021	-1.666	3.45E-02	-1.29E-02	50	0.021	-1.666	0.03452	-0.01288	50	TRUE	TRUE	TRUE	TRUE	TRUE
1	LSRLDKVEA	963	971	9	HLA-A*02:01	963	0.029	-0.433	2.13E-01	6.61E-02	50	0.029	-0.433	0.21305	0.06611	50	TRUE	TRUE	TRUE	TRUE	TRUE
1	SRLDKVEAE	964	972	9	HLA-A*02:01	964	0.011	-1.24	2.99E-02	-1.33E-02	50	0.011	-1.24	0.02986	-0.01328	50	TRUE	TRUE	TRUE	TRUE	TRUE
1	RLDKVEAEV	965	973	9	HLA-A*02:01	965	0.624	0.456	9.69E-01	8.53E-01	0.8	0.624	0.456	0.96911	0.85345	0.8	TRUE	TRUE	TRUE	TRUE	TRUE
1	LDKVEAEVQ	966	974	9	HLA-A*02:01	966	0.004	-0.279	2.66E-02	3.02E-03	50	0.004	-0.279	0.02664	0.00302	50	TRUE	TRUE	TRUE	TRUE	TRUE
1	DKVEAEVQI	967	975	9	HLA-A*02:01	967	0.017	0.394	2.72E-01	8.80E-02	50	0.017	0.394	0.27156	0.08795	50	TRUE	TRUE	TRUE	TRUE	TRUE
1	KVEAEVQID	968	976	9	HLA-A*02:01	968	0.013	-1.853	2.40E-02	-2.79E-02	50	0.013	-1.853	0.02403	-0.02792	50	TRUE	TRUE	TRUE	TRUE	TRUE
1	VEAEVQIDR	969	977	9	HLA-A*02:01	969	0.021	1.529	6.58E-01	2.07E-01	32	0.021	1.529	0.65811	0.2073	32	TRUE	TRUE	TRUE	TRUE	TRUE
1	EAEVQIDRL	970	978	9	HLA-A*02:01	970	0.021	0.706	5.00E-01	1.51E-01	50	0.021	0.706	0.50001	0.15115	50	TRUE	TRUE	TRUE	TRUE	TRUE
1	AEVQIDRLI	971	979	9	HLA-A*02:01	971	0.038	0.454	4.17E-01	1.43E-01	50	0.038	0.454	0.41688	0.14315	50	TRUE	TRUE	TRUE	TRUE	TRUE
1	EVQIDRLIT	972	980	9	HLA-A*02:01	972	0.018	-0.649	2.81E-02	8.11E-03	50	0.018	-0.649	0.02813	0.00811	50	TRUE	TRUE	TRUE	TRUE	TRUE
1	VQIDRLITG	973	981	9	HLA-A*02:01	973	0.129	-1.356	1.18E-01	1.22E-01	50	0.129	-1.356	0.11773	0.12159	50	TRUE	TRUE	TRUE	TRUE	TRUE
1	QIDRLITGR	974	982	9	HLA-A*02:01	974	0.026	1.497	6.63E-01	2.13E-01	32	0.026	1.497	0.6632	0.21264	32	TRUE	TRUE	TRUE	TRUE	TRUE
1	IDRLITGRL	975	983	9	HLA-A*02:01	975	0.026	0.67	9.22E-01	2.50E-01	32	0.026	0.67	0.92165	0.25012	32	TRUE	TRUE	TRUE	TRUE	TRUE
1	DRLITGRLQ	976	984	9	HLA-A*02:01	976	0.006	-0.188	4.10E-02	1.05E-02	50	0.006	-0.188	0.04102	0.01053	50	TRUE	TRUE	TRUE	TRUE	TRUE
1	RLITGRLQS	977	985	9	HLA-A*02:01	977	0.123	-2.052	3.47E-02	7.95E-02	50	0.123	-2.052	0.03468	0.0795	50	TRUE	TRUE	TRUE	TRUE	TRUE
1	LITGRLQSL	978	986	9	HLA-A*02:01	978	0.279	1.183	8.92E-01	5.09E-01	5	0.279	1.183	0.89184	0.50924	5	TRUE	TRUE	TRUE	TRUE	TRUE
1	ITGRLQSLQ	979	987	9	HLA-A*02:01	979	0.013	-0.219	2.91E-02	1.41E-02	50	0.013	-0.219	0.02913	0.01408	50	TRUE	TRUE	TRUE	TRUE	TRUE
1	TGRLQSLQT	980	988	9	HLA-A*02:01	980	0.014	-0.935	4.82E-02	1.47E-03	50	0.014	-0.935	0.0482	0.00147	50	TRUE	TRUE	TRUE	TRUE	TRUE
1	GRLQSLQTY	981	989	9	HLA-A*02:01	981	0.02	2.98	9.62E-01	3.11E-01	16	0.02	2.98	0.96212	0.31098	16	TRUE	TRUE	TRUE	TRUE	TRUE
1	RLQSLQTYV	982	990	9	HLA-A*02:01	982	0.731	0.516	7.48E-01	9.12E-01	0.8	0.731	0.516	0.7484	0.91229	0.8	TRUE	TRUE	TRUE	TRUE	TRUE
1	LQSLQTYVT	983	991	9	HLA-A*02:01	983	0.109	-0.498	7.60E-02	1.14E-01	50	0.109	-0.498	0.07595	0.11364	50	TRUE	TRUE	TRUE	TRUE	TRUE
1	QSLQTYVTQ	984	992	9	HLA-A*02:01	984	0.021	0.014	9.21E-02	4.21E-02	50	0.021	0.014	0.09213	0.04208	50	TRUE	TRUE	TRUE	TRUE	TRUE
1	SLQTYVTQQ	985	993	9	HLA-A*02:01	985	0.146	-0.114	2.39E-01	1.97E-01	50	0.146	-0.114	0.23886	0.19689	50	TRUE	TRUE	TRUE	TRUE	TRUE
1	LQTYVTQQL	986	994	9	HLA-A*02:01	986	0.265	1.052	8.33E-01	4.79E-01	6	0.265	1.052	0.83313	0.47875	6	TRUE	TRUE	TRUE	TRUE	TRUE
1	QTYVTQQLI	987	995	9	HLA-A*02:01	987	0.127	0.761	8.47E-01	3.37E-01	16	0.127	0.761	0.84696	0.33659	16	TRUE	TRUE	TRUE	TRUE	TRUE
1	TYVTQQLIR	988	996	9	HLA-A*02:01	988	0.014	1.757	4.81E-01	1.66E-01	50	0.014	1.757	0.48121	0.1662	50	TRUE	TRUE	TRUE	TRUE	TRUE
1	YVTQQLIRA	989	997	9	HLA-A*02:01	989	0.218	-0.443	3.87E-01	2.94E-01	16	0.218	-0.443	0.38666	0.29392	16	TRUE	TRUE	TRUE	TRUE	TRUE
1	VTQQLIRAA	990	998	9	HLA-A*02:01	990	0.075	-0.579	6.70E-02	7.56E-02	50	0.075	-0.579	0.06695	0.07559	50	TRUE	TRUE	TRUE	TRUE	TRUE
1	TQQLIRAAE	991	999	9	HLA-A*02:01	991	0.023	-1.496	2.33E-02	-9.15E-03	50	0.023	-1.496	0.02334	-0.00915	50	TRUE	TRUE	TRUE	TRUE	TRUE
1	QQLIRAAEI	992	1000	9	HLA-A*02:01	992	0.234	0.651	7.69E-01	4.23E-01	7	0.234	0.651	0.76889	0.42327	7	TRUE	TRUE	TRUE	TRUE	TRUE
1	QLIRAAEIR	993	1001	9	HLA-A*02:01	993	0.032	1.537	1.42E-01	1.02E-01	50	0.032	1.537	0.14196	0.10237	50	TRUE	TRUE	TRUE	TRUE	TRUE
1	LIRAAEIRA	994	1002	9	HLA-A*02:01	994	0.102	-0.403	1.17E-01	1.18E-01	50	0.102	-0.403	0.11725	0.11831	50	TRUE	TRUE	TRUE	TRUE	TRUE
1	IRAAEIRAS	995	1003	9	HLA-A*02:01	995	0.014	-1.951	2.48E-02	-2.92E-02	50	0.014	-1.951	0.02475	-0.02921	50	TRUE	TRUE	TRUE	TRUE	TRUE
1	RAAEIRASA	996	1004	9	HLA-A*02:01	996	0.185	-0.213	4.72E-01	2.86E-01	32	0.185	-0.213	0.47183	0.28584	32	TRUE	TRUE	TRUE	TRUE	TRUE
1	AAEIRASAN	997	1005	9	HLA-A*02:01	997	0.011	-1.131	2.40E-02	-1.19E-02	50	0.011	-1.131	0.02398	-0.01188	50	TRUE	TRUE	TRUE	TRUE	TRUE
1	AEIRASANL	998	1006	9	HLA-A*02:01	998	0.056	1.186	9.64E-01	3.03E-01	16	0.056	1.186	0.96422	0.3026	16	TRUE	TRUE	TRUE	TRUE	TRUE
1	EIRASANLA	999	1007	9	HLA-A*02:01	999	0.037	-0.408	1.22E-01	5.42E-02	50	0.037	-0.408	0.1217	0.05418	50	TRUE	TRUE	TRUE	TRUE	TRUE
1	IRASANLAA	1000	1008	9	HLA-A*02:01	1000	0.032	-0.456	1.13E-01	4.61E-02	50	0.032	-0.456	0.1131	0.04605	50	TRUE	TRUE	TRUE	TRUE	TRUE
1	RASANLAAT	1001	1009	9	HLA-A*02:01	1001	0.13	-0.404	3.93E-02	1.29E-01	50	0.13	-0.404	0.03933	0.12875	50	TRUE	TRUE	TRUE	TRUE	TRUE
1	ASANLAATK	1002	1010	9	HLA-A*02:01	1002	0.026	0.798	6.96E-01	2.03E-01	32	0.026	0.798	0.69623	0.2026	32	TRUE	TRUE	TRUE	TRUE	TRUE
1	SANLAATKM	1003	1011	9	HLA-A*02:01	1003	0.049	0.47	9.59E-01	2.77E-01	32	0.049	0.47	0.95929	0.27659	32	TRUE	TRUE	TRUE	TRUE	TRUE
1	ANLAATKMS	1004	1012	9	HLA-A*02:01	1004	0.01	-2.182	3.83E-02	-3.59E-02	50	0.01	-2.182	0.03826	-0.03594	50	TRUE	TRUE	TRUE	TRUE	TRUE
1	NLAATKMSE	1005	1013	9	HLA-A*02:01	1005	0.033	-1.338	4.39E-02	9.43E-03	50	0.033	-1.338	0.04391	0.00943	50	TRUE	TRUE	TRUE	TRUE	TRUE
1	LAATKMSEC	1006	1014	9	HLA-A*02:01	1006	0.056	0.229	2.53E-02	6.74E-02	50	0.056	0.229	0.02533	0.06742	50	TRUE	TRUE	TRUE	TRUE	TRUE
1	AATKMSECV	1007	1015	9	HLA-A*02:01	1007	0.11	0.523	2.63E-01	1.82E-01	50	0.11	0.523	0.26339	0.18234	50	TRUE	TRUE	TRUE	TRUE	TRUE
1	ATKMSECVL	1008	1016	9	HLA-A*02:01	1008	0.042	1.229	5.50E-01	1.96E-01	50	0.042	1.229	0.54987	0.19645	50	TRUE	TRUE	TRUE	TRUE	TRUE
1	TKMSECVLG	1009	1017	9	HLA-A*02:01	1009	0.012	-1.148	3.51E-02	-8.80E-03	50	0.012	-1.148	0.0351	-0.0088	50	TRUE	TRUE	TRUE	TRUE	TRUE
1	KMSECVLGQ	1010	1018	9	HLA-A*02:01	1010	0.388	0.129	5.22E-02	4.03E-01	8	0.388	0.129	0.05221	0.40297	8	TRUE	TRUE	TRUE	TRUE	TRUE
1	MSECVLGQS	1011	1019	9	HLA-A*02:01	1011	0.036	-2.201	4.17E-02	-9.65E-03	50	0.036	-2.201	0.04166	-0.00965	50	TRUE	TRUE	TRUE	TRUE	TRUE
1	SECVLGQSK	1012	1020	9	HLA-A*02:01	1012	0.01	0.427	4.19E-01	1.15E-01	50	0.01	0.427	0.41943	0.11505	50	TRUE	TRUE	TRUE	TRUE	TRUE
1	ECVLGQSKR	1013	1021	9	HLA-A*02:01	1013	0.006	1.433	7.29E-02	5.82E-02	50	0.006	1.433	0.07285	0.05822	50	TRUE	TRUE	TRUE	TRUE	TRUE
1	CVLGQSKRV	1014	1022	9	HLA-A*02:01	1014	0.174	0.263	9.26E-01	3.89E-01	8	0.174	0.263	0.9255	0.38881	8	TRUE	TRUE	TRUE	TRUE	TRUE
1	VLGQSKRVD	1015	1023	9	HLA-A*02:01	1015	0.011	-1.904	2.86E-02	-3.02E-02	50	0.011	-1.904	0.02855	-0.03018	50	TRUE	TRUE	TRUE	TRUE	TRUE
1	LGQSKRVDF	1016	1024	9	HLA-A*02:01	1016	0.013	2.287	2.90E-01	1.35E-01	50	0.013	2.287	0.29026	0.13548	50	TRUE	TRUE	TRUE	TRUE	TRUE
1	GQSKRVDFC	1017	1025	9	HLA-A*02:01	1017	0.077	-0.045	2.27E-02	8.10E-02	50	0.077	-0.045	0.02273	0.08099	50	TRUE	TRUE	TRUE	TRUE	TRUE
1	QSKRVDFCG	1018	1026	9	HLA-A*02:01	1018	0.008	-1.374	3.37E-02	-1.88E-02	50	0.008	-1.374	0.03372	-0.01876	50	TRUE	TRUE	TRUE	TRUE	TRUE
1	SKRVDFCGK	1019	1027	9	HLA-A*02:01	1019	0.007	0.567	4.62E-01	1.25E-01	50	0.007	0.567	0.46223	0.12518	50	TRUE	TRUE	TRUE	TRUE	TRUE
1	KRVDFCGKG	1020	1028	9	HLA-A*02:01	1020	0.011	-0.95	2.81E-01	5.04E-02	50	0.011	-0.95	0.28073	0.05041	50	TRUE	TRUE	TRUE	TRUE	TRUE
1	RVDFCGKGY	1021	1029	9	HLA-A*02:01	1021	0.048	3.232	7.53E-01	2.98E-01	16	0.048	3.232	0.75288	0.2982	16	TRUE	TRUE	TRUE	TRUE	TRUE
1	VDFCGKGYH	1022	1030	9	HLA-A*02:01	1022	0.015	-0.537	5.72E-02	1.45E-02	50	0.015	-0.537	0.05723	0.01445	50	TRUE	TRUE	TRUE	TRUE	TRUE
1	DFCGKGYHL	1023	1031	9	HLA-A*02:01	1023	0.032	0.862	9.52E-01	2.68E-01	32	0.032	0.862	0.9518	0.2677	32	TRUE	TRUE	TRUE	TRUE	TRUE
1	FCGKGYHLM	1024	1032	9	HLA-A*02:01	1024	0.086	-0.072	5.71E-01	2.13E-01	32	0.086	-0.072	0.57062	0.21259	32	TRUE	TRUE	TRUE	TRUE	TRUE
1	CGKGYHLMS	1025	1033	9	HLA-A*02:01	1025	0.015	-2.66	3.51E-02	-4.36E-02	50	0.015	-2.66	0.03512	-0.0436	50	TRUE	TRUE	TRUE	TRUE	TRUE
1	GKGYHLMSF	1026	1034	9	HLA-A*02:01	1026	0.025	2.235	4.65E-01	1.85E-01	50	0.025	2.235	0.46473	0.18544	50	TRUE	TRUE	TRUE	TRUE	TRUE
1	KGYHLMSFP	1027	1035	9	HLA-A*02:01	1027	0.028	0.102	3.49E-02	3.84E-02	50	0.028	0.102	0.03491	0.0384	50	TRUE	TRUE	TRUE	TRUE	TRUE
1	GYHLMSFPQ	1028	1036	9	HLA-A*02:01	1028	0.015	0.111	1.62E-01	5.42E-02	50	0.015	0.111	0.16193	0.05421	50	TRUE	TRUE	TRUE	TRUE	TRUE
1	YHLMSFPQA	1029	1037	9	HLA-A*02:01	1029	0.128	-0.56	6.72E-02	1.29E-01	50	0.128	-0.56	0.06724	0.12913	50	TRUE	TRUE	TRUE	TRUE	TRUE
1	HLMSFPQAA	1030	1038	9	HLA-A*02:01	1030	0.639	-0.594	4.64E-02	6.35E-01	3	0.639	-0.594	0.04639	0.63459	3	TRUE	TRUE	TRUE	TRUE	TRUE
1	LMSFPQAAP	1031	1039	9	HLA-A*02:01	1031	0.094	0.203	4.88E-02	1.10E-01	50	0.094	0.203	0.04883	0.11006	50	TRUE	TRUE	TRUE	TRUE	TRUE
1	MSFPQAAPH	1032	1040	9	HLA-A*02:01	1032	0.064	-0.288	9.58E-02	7.84E-02	50	0.064	-0.288	0.09577	0.07835	50	TRUE	TRUE	TRUE	TRUE	TRUE
1	SFPQAAPHG	1033	1041	9	HLA-A*02:01	1033	0.014	-1.154	2.65E-02	-8.88E-03	50	0.014	-1.154	0.02651	-0.00888	50	TRUE	TRUE	TRUE	TRUE	TRUE
1	FPQAAPHGV	1034	1042	9	HLA-A*02:01	1034	0.118	-0.063	6.91E-01	2.72E-01	32	0.118	-0.063	0.69112	0.27193	32	TRUE	TRUE	TRUE	TRUE	TRUE
1	PQAAPHGVV	1035	1043	9	HLA-A*02:01	1035	0.053	-0.178	4.29E-01	1.45E-01	50	0.053	-0.178	0.42875	0.14502	50	TRUE	TRUE	TRUE	TRUE	TRUE
1	QAAPHGVVF	1036	1044	9	HLA-A*02:01	1036	0.047	2.472	3.96E-01	1.98E-01	50	0.047	2.472	0.39586	0.19787	50	TRUE	TRUE	TRUE	TRUE	TRUE
1	AAPHGVVFL	1037	1045	9	HLA-A*02:01	1037	0.209	1.087	9.06E-01	4.40E-01	7	0.209	1.087	0.90634	0.4401	7	TRUE	TRUE	TRUE	TRUE	TRUE
1	APHGVVFLH	1038	1046	9	HLA-A*02:01	1038	0.022	-0.512	7.68E-01	1.82E-01	50	0.022	-0.512	0.76818	0.18204	50	TRUE	TRUE	TRUE	TRUE	TRUE
1	PHGVVFLHV	1039	1047	9	HLA-A*02:01	1039	0.026	-0.167	8.91E-01	2.22E-01	32	0.026	-0.167	0.89123	0.22235	32	TRUE	TRUE	TRUE	TRUE	TRUE
1	HGVVFLHVT	1040	1048	9	HLA-A*02:01	1040	0.043	-1.13	6.75E-02	2.99E-02	50	0.043	-1.13	0.06752	0.02994	50	TRUE	TRUE	TRUE	TRUE	TRUE
1	GVVFLHVTY	1041	1049	9	HLA-A*02:01	1041	0.056	2.939	9.75E-01	3.49E-01	10	0.056	2.939	0.97544	0.34895	10	TRUE	TRUE	TRUE	TRUE	TRUE
1	VVFLHVTYV	1042	1050	9	HLA-A*02:01	1042	0.751	0.597	8.31E-01	9.53E-01	0.4	0.751	0.597	0.83113	0.95293	0.4	TRUE	TRUE	TRUE	TRUE	TRUE
1	VFLHVTYVP	1043	1051	9	HLA-A*02:01	1043	0.035	0.399	1.19E-01	7.17E-02	50	0.035	0.399	0.11887	0.07172	50	TRUE	TRUE	TRUE	TRUE	TRUE
1	FLHVTYVPS	1044	1052	9	HLA-A*02:01	1044	0.335	-2.244	1.03E-01	3.02E-01	16	0.335	-2.244	0.10342	0.30217	16	TRUE	TRUE	TRUE	TRUE	TRUE
1	LHVTYVPSQ	1045	1053	9	HLA-A*02:01	1045	0.016	-0.121	3.35E-01	8.83E-02	50	0.016	-0.121	0.33477	0.0883	50	TRUE	TRUE	TRUE	TRUE	TRUE
1	HVTYVPSQE	1046	1054	9	HLA-A*02:01	1046	0.02	-1.506	2.94E-02	-1.10E-02	50	0.02	-1.506	0.02936	-0.01104	50	TRUE	TRUE	TRUE	TRUE	TRUE
1	VTYVPSQER	1047	1055	9	HLA-A*02:01	1047	0.023	1.689	9.35E-01	2.76E-01	32	0.023	1.689	0.93467	0.27553	32	TRUE	TRUE	TRUE	TRUE	TRUE
1	TYVPSQERN	1048	1056	9	HLA-A*02:01	1048	0.007	-0.947	4.41E-02	-6.74E-03	50	0.007	-0.947	0.04414	-0.00674	50	TRUE	TRUE	TRUE	TRUE	TRUE
1	YVPSQERNF	1049	1057	9	HLA-A*02:01	1049	0.031	2.648	2.67E-01	1.57E-01	50	0.031	2.648	0.26679	0.15723	50	TRUE	TRUE	TRUE	TRUE	TRUE
1	VPSQERNFT	1050	1058	9	HLA-A*02:01	1050	0.013	-1.004	3.38E-02	-4.49E-03	50	0.013	-1.004	0.03381	-0.00449	50	TRUE	TRUE	TRUE	TRUE	TRUE

seq #	peptide	start	end	peptide length	allele	peptide index	NetCTLpan MH	NetCTLpan tap	NetCTLpan cle	NetCTLpan com	NetCTLpan per	legacy mhc_pr	legacy tap_pre	legacy cleavag	legacy combin	legacy %_rank	equal mhc_pre	equal tap_pred	equal cleavage	equal combine	equal %_rank
1	PSQERNFTT	1051	1059	9	HLA-A*02:01	1051	0.017	-1.13	1.25E-01	1.69E-02	50	0.017	-1.13	0.12505	0.01689	50	TRUE	TRUE	TRUE	TRUE	TRUE
1	SQERNFTTA	1052	1060	9	HLA-A*02:01	1052	0.159	-0.696	3.97E-01	2.31E-01	32	0.159	-0.696	0.39672	0.23086	32	TRUE	TRUE	TRUE	TRUE	TRUE
1	QERNFTTAP	1053	1061	9	HLA-A*02:01	1053	0.008	0.085	5.87E-01	1.42E-01	50	0.008	0.085	0.58672	0.14214	50	TRUE	TRUE	TRUE	TRUE	TRUE
1	ERNFTTAPA	1054	1062	9	HLA-A*02:01	1054	0.021	-0.676	7.18E-01	1.66E-01	50	0.021	-0.676	0.7177	0.16558	50	TRUE	TRUE	TRUE	TRUE	TRUE
1	RNFTTAPAI	1055	1063	9	HLA-A*02:01	1055	0.101	0.571	3.64E-01	1.97E-01	50	0.101	0.571	0.36389	0.19715	50	TRUE	TRUE	TRUE	TRUE	TRUE
1	NFTTAPAIC	1056	1064	9	HLA-A*02:01	1056	0.023	0.315	2.95E-02	3.75E-02	50	0.023	0.315	0.02954	0.03752	50	TRUE	TRUE	TRUE	TRUE	TRUE
1	FTTAPAICH	1057	1065	9	HLA-A*02:01	1057	0.051	-0.547	1.15E-01	6.31E-02	50	0.051	-0.547	0.11471	0.06313	50	TRUE	TRUE	TRUE	TRUE	TRUE
1	TTAPAICHE	1058	1066	9	HLA-A*02:01	1058	0.023	-1.696	2.96E-02	-1.27E-02	50	0.023	-1.696	0.02962	-0.01274	50	TRUE	TRUE	TRUE	TRUE	TRUE
1	TAPAICHEG	1059	1067	9	HLA-A*02:01	1059	0.028	-1.369	1.35E-01	2.43E-02	50	0.028	-1.369	0.13545	0.02425	50	TRUE	TRUE	TRUE	TRUE	TRUE
1	APAICHEGK	1060	1068	9	HLA-A*02:01	1060	0.01	0.331	8.59E-01	2.12E-01	32	0.01	0.331	0.85885	0.21152	32	TRUE	TRUE	TRUE	TRUE	TRUE
1	PAICHEGKA	1061	1069	9	HLA-A*02:01	1061	0.015	-0.74	1.01E-01	1.91E-02	50	0.015	-0.74	0.10056	0.01913	50	TRUE	TRUE	TRUE	TRUE	TRUE
1	AICHEGKAY	1062	1070	9	HLA-A*02:01	1062	0.021	3.061	2.52E-01	1.54E-01	50	0.021	3.061	0.25236	0.15431	50	TRUE	TRUE	TRUE	TRUE	TRUE
1	ICHEGKAYF	1063	1071	9	HLA-A*02:01	1063	0.023	2.762	1.65E-01	1.29E-01	50	0.023	2.762	0.16514	0.12921	50	TRUE	TRUE	TRUE	TRUE	TRUE
1	CHEGKAYFP	1064	1072	9	HLA-A*02:01	1064	0.01	-0.064	2.36E-02	1.37E-02	50	0.01	-0.064	0.02356	0.0137	50	TRUE	TRUE	TRUE	TRUE	TRUE
1	HEGKAYFPR	1065	1073	9	HLA-A*02:01	1065	0.022	1.088	1.36E-01	7.99E-02	50	0.022	1.088	0.13628	0.07986	50	TRUE	TRUE	TRUE	TRUE	TRUE
1	EGKAYFPRE	1066	1074	9	HLA-A*02:01	1066	0.007	-2.061	2.47E-02	-3.90E-02	50	0.007	-2.061	0.02466	-0.03898	50	TRUE	TRUE	TRUE	TRUE	TRUE
1	GKAYFPREG	1067	1075	9	HLA-A*02:01	1067	0.008	-1.675	2.53E-02	-2.82E-02	50	0.008	-1.675	0.02533	-0.02817	50	TRUE	TRUE	TRUE	TRUE	TRUE
1	KAYFPREGV	1068	1076	9	HLA-A*02:01	1068	0.235	0.631	1.26E-01	2.79E-01	32	0.235	0.631	0.1264	0.27921	32	TRUE	TRUE	TRUE	TRUE	TRUE
1	AYFPREGVF	1069	1077	9	HLA-A*02:01	1069	0.032	3.156	8.60E-01	3.04E-01	16	0.032	3.156	0.8602	0.30445	16	TRUE	TRUE	TRUE	TRUE	TRUE
1	YFPREGVFV	1070	1078	9	HLA-A*02:01	1070	0.166	0.601	1.65E-01	2.18E-01	32	0.166	0.601	0.16459	0.21806	32	TRUE	TRUE	TRUE	TRUE	TRUE
1	FPREGVFVF	1071	1079	9	HLA-A*02:01	1071	0.064	2.132	9.68E-01	3.35E-01	16	0.064	2.132	0.9679	0.33508	16	TRUE	TRUE	TRUE	TRUE	TRUE
1	PREGVFVFN	1072	1080	9	HLA-A*02:01	1072	0.005	-1.838	5.17E-02	-2.93E-02	50	0.005	-1.838	0.05168	-0.02932	50	TRUE	TRUE	TRUE	TRUE	TRUE
1	REGVFVFNG	1073	1081	9	HLA-A*02:01	1073	0.019	-1.595	3.03E-02	-1.41E-02	50	0.019	-1.595	0.0303	-0.01406	50	TRUE	TRUE	TRUE	TRUE	TRUE
1	EGVFVFNGT	1074	1082	9	HLA-A*02:01	1074	0.032	-0.966	2.83E-02	1.42E-02	50	0.032	-0.966	0.02827	0.01421	50	TRUE	TRUE	TRUE	TRUE	TRUE
1	GVFVFNGTS	1075	1083	9	HLA-A*02:01	1075	0.071	-2.341	3.96E-02	2.14E-02	50	0.071	-2.341	0.03963	0.02139	50	TRUE	TRUE	TRUE	TRUE	TRUE
1	VFVFNGTSW	1076	1084	9	HLA-A*02:01	1076	0.036	1.162	8.73E-01	2.61E-01	32	0.036	1.162	0.873	0.26148	32	TRUE	TRUE	TRUE	TRUE	TRUE
1	FVFNGTSWF	1077	1085	9	HLA-A*02:01	1077	0.289	2.737	7.30E-01	5.22E-01	5	0.289	2.737	0.72953	0.52157	5	TRUE	TRUE	TRUE	TRUE	TRUE
1	VFNGTSWFI	1078	1086	9	HLA-A*02:01	1078	0.262	0.565	3.73E-01	3.60E-01	10	0.262	0.565	0.37271	0.35998	10	TRUE	TRUE	TRUE	TRUE	TRUE
1	FNGTSWFIT	1079	1087	9	HLA-A*02:01	1079	0.045	-1.069	3.25E-02	2.56E-02	50	0.045	-1.069	0.03254	0.0256	50	TRUE	TRUE	TRUE	TRUE	TRUE
1	NGTSWFITQ	1080	1088	9	HLA-A*02:01	1080	0.022	-0.476	9.16E-02	3.07E-02	5.00E+01	0.022	-0.476	0.09159	0.03071	50	TRUE	TRUE	TRUE	TRUE	TRUE
1	GTSWFITQR	1081	1089	9	HLA-A*02:01	1081	0.066	1.421	9.42E-01	3.14E-01	16	0.066	1.421	0.94244	0.31357	16	TRUE	TRUE	TRUE	TRUE	TRUE
1	TSWFITQRN	1082	1090	9	HLA-A*02:01	1082	0.022	-1.154	3.65E-01	7.52E-02	50	0.022	-1.154	0.36467	0.0752	50	TRUE	TRUE	TRUE	TRUE	TRUE
1	SWFITQRNF	1083	1091	9	HLA-A*02:01	1083	0.03	2.956	6.18E-01	2.43E-01	32	0.03	2.956	0.61791	0.24293	32	TRUE	TRUE	TRUE	TRUE	TRUE
1	WFITQRNFF	1084	1092	9	HLA-A*02:01	1084	0.045	2.798	9.22E-02	1.36E-01	50	0.045	2.798	0.09219	0.13569	50	TRUE	TRUE	TRUE	TRUE	TRUE
1	FITQRNFFS	1085	1093	9	HLA-A*02:01	1085	0.159	-2.304	2.26E-02	1.06E-01	50	0.159	-2.304	0.02257	0.10648	50	TRUE	TRUE	TRUE	TRUE	TRUE
1	ITQRNFFSP	1086	1094	9	HLA-A*02:01	1086	0.033	-0.031	3.32E-02	3.97E-02	50	0.033	-0.031	0.0332	0.0397	50	TRUE	TRUE	TRUE	TRUE	TRUE
1	TQRNFFSPQ	1087	1095	9	HLA-A*02:01	1087	0.046	0.007	6.51E-02	6.08E-02	50	0.046	0.007	0.06505	0.06081	50	TRUE	TRUE	TRUE	TRUE	TRUE
1	QRNFFSPQI	1088	1096	9	HLA-A*02:01	1088	0.058	0.67	8.86E-01	2.74E-01	32	0.058	0.67	0.88572	0.27404	32	TRUE	TRUE	TRUE	TRUE	TRUE
1	RNFFSPQII	1089	1097	9	HLA-A*02:01	1089	0.06	0.689	2.85E-01	1.41E-01	50	0.06	0.689	0.28473	0.14129	50	TRUE	TRUE	TRUE	TRUE	TRUE
1	NFFSPQIIT	1090	1098	9	HLA-A*02:01	1090	0.044	-0.257	3.10E-02	4.45E-02	50	0.044	-0.257	0.03096	0.04454	50	TRUE	TRUE	TRUE	TRUE	TRUE
1	FFSPQIITT	1091	1099	9	HLA-A*02:01	1091	0.147	-0.496	4.55E-01	2.37E-01	32	0.147	-0.496	0.45489	0.23695	32	TRUE	TRUE	TRUE	TRUE	TRUE
1	FSPQIITTD	1092	1100	9	HLA-A*02:01	1092	0.033	-1.99	9.81E-02	5.33E-03	50	0.033	-1.99	0.09812	0.00533	50	TRUE	TRUE	TRUE	TRUE	TRUE
1	SPQIITTDN	1093	1101	9	HLA-A*02:01	1093	0.009	-1.704	2.38E-01	2.00E-02	50	0.009	-1.704	0.23801	0.01995	50	TRUE	TRUE	TRUE	TRUE	TRUE
1	PQIITTDNT	1094	1102	9	HLA-A*02:01	1094	0.016	-0.987	2.81E-02	-2.35E-03	50	0.016	-0.987	0.02813	-0.00235	50	TRUE	TRUE	TRUE	TRUE	TRUE
1	QIITTDNTF	1095	1103	9	HLA-A*02:01	1095	0.064	2.6	7.54E-01	2.99E-01	16	0.064	2.6	0.75359	0.29856	16	TRUE	TRUE	TRUE	TRUE	TRUE
1	IITTDNTFV	1096	1104	9	HLA-A*02:01	1096	0.471	0.381	7.57E-01	6.51E-01	3	0.471	0.381	0.75661	0.65076	3	TRUE	TRUE	TRUE	TRUE	TRUE
1	ITTDNTFVS	1097	1105	9	HLA-A*02:01	1097	0.061	-2.271	1.38E-01	3.53E-02	50	0.061	-2.271	0.13822	0.03532	50	TRUE	TRUE	TRUE	TRUE	TRUE
1	TTDNTFVSG	1098	1106	9	HLA-A*02:01	1098	0.04	-1.605	2.93E-01	6.59E-02	50	0.04	-1.605	0.29325	0.06586	50	TRUE	TRUE	TRUE	TRUE	TRUE
1	TDNTFVSGN	1099	1107	9	HLA-A*02:01	1099	0.01	-1.728	2.99E-02	-2.65E-02	50	0.01	-1.728	0.02988	-0.02648	50	TRUE	TRUE	TRUE	TRUE	TRUE
1	DNTFVSGNC	1100	1108	9	HLA-A*02:01	1100	0.01	-0.48	2.41E-02	3.42E-03	50	0.01	-0.48	0.02408	0.00342	50	TRUE	TRUE	TRUE	TRUE	TRUE
1	NTFVSGNCD	1101	1109	9	HLA-A*02:01	1101	0.009	-1.827	2.36E-02	-3.14E-02	50	0.009	-1.827	0.02362	-0.03136	50	TRUE	TRUE	TRUE	TRUE	TRUE
1	TFVSGNCDV	1102	1110	9	HLA-A*02:01	1102	0.097	0.68	2.16E-01	1.63E-01	50	0.097	0.68	0.21595	0.16259	50	TRUE	TRUE	TRUE	TRUE	TRUE
1	FVSGNCDVV	1103	1111	9	HLA-A*02:01	1103	0.496	0.364	9.58E-02	5.27E-01	5	0.496	0.364	0.09582	0.52666	5	TRUE	TRUE	TRUE	TRUE	TRUE
1	VSGNCDVVI	1104	1112	9	HLA-A*02:01	1104	0.078	0.369	5.89E-02	1.00E-01	50	0.078	0.369	0.05893	0.10048	50	TRUE	TRUE	TRUE	TRUE	TRUE
1	SGNCDVVIG	1105	1113	9	HLA-A*02:01	1105	0.012	-1.656	2.41E-02	-2.40E-02	50	0.012	-1.656	0.02411	-0.02398	50	TRUE	TRUE	TRUE	TRUE	TRUE
1	GNCDVVIGI	1106	1114	9	HLA-A*02:01	1106	0.095	0.266	1.82E-01	1.43E-01	50	0.095	0.266	0.18233	0.14268	50	TRUE	TRUE	TRUE	TRUE	TRUE
1	NCDVVIGII	1107	1115	9	HLA-A*02:01	1107	0.074	0.386	1.23E-01	1.11E-01	50	0.074	0.386	0.12313	0.11135	50	TRUE	TRUE	TRUE	TRUE	TRUE
1	CDVVIGIIN	1108	1116	9	HLA-A*02:01	1108	0.009	-1.445	3.08E-02	-2.02E-02	50	0.009	-1.445	0.03084	-0.02019	50	TRUE	TRUE	TRUE	TRUE	TRUE
1	DVVIGIINN	1109	1117	9	HLA-A*02:01	1109	0.015	-1.422	6.35E-02	-6.26E-03	50	0.015	-1.422	0.06353	-0.00626	50	TRUE	TRUE	TRUE	TRUE	TRUE
1	VVIGIINNT	1110	1118	9	HLA-A*02:01	1110	0.186	-0.575	1.70E-01	2.10E-01	32	0.186	-0.575	0.1698	0.20983	32	TRUE	TRUE	TRUE	TRUE	TRUE
1	VIGIINNTV	1111	1119	9	HLA-A*02:01	1111	0.393	0.389	9.44E-01	6.15E-01	3	0.393	0.389	0.94419	0.61517	3	TRUE	TRUE	TRUE	TRUE	TRUE
1	IGIINNTVY	1112	1120	9	HLA-A*02:01	1112	0.02	2.797	9.48E-01	3.03E-01	16	0.02	2.797	0.94813	0.30325	16	TRUE	TRUE	TRUE	TRUE	TRUE
1	GIINNTVYD	1113	1121	9	HLA-A*02:01	1113	0.044	-1.788	1.63E-01	3.59E-02	50	0.044	-1.788	0.16276	0.03592	50	TRUE	TRUE	TRUE	TRUE	TRUE
1	IINNTVYDP	1114	1122	9	HLA-A*02:01	1114	0.039	0.132	4.63E-02	5.27E-02	50	0.039	0.132	0.04634	0.05273	50	TRUE	TRUE	TRUE	TRUE	TRUE
1	INNTVYDPL	1115	1123	9	HLA-A*02:01	1115	0.05	0.74	8.40E-01	2.57E-01	32	0.05	0.74	0.83974	0.25744	32	TRUE	TRUE	TRUE	TRUE	TRUE
1	NNTVYDPLQ	1116	1124	9	HLA-A*02:01	1116	0.006	-0.248	2.43E-02	5.27E-03	50	0.006	-0.248	0.02429	0.00527	50	TRUE	TRUE	TRUE	TRUE	TRUE
1	NTVYDPLQP	1117	1125	9	HLA-A*02:01	1117	0.013	0.339	5.24E-02	3.33E-02	50	0.013	0.339	0.05243	0.03327	50	TRUE	TRUE	TRUE	TRUE	TRUE
1	TVYDPLQPE	1118	1126	9	HLA-A*02:01	1118	0.055	-1.12	6.08E-02	4.07E-02	50	0.055	-1.12	0.06084	0.04069	50	TRUE	TRUE	TRUE	TRUE	TRUE
1	VYDPLQPEL	1119	1127	9	HLA-A*02:01	1119	0.083	1.115	9.74E-01	3.30E-01	16	0.083	1.115	0.97446	0.33013	16	TRUE	TRUE	TRUE	TRUE	TRUE
1	YDPLQPELD	1120	1128	9	HLA-A*02:01	1120	0.006	-2.111	4.59E-02	-3.65E-02	50	0.006	-2.111	0.0459	-0.03645	50	TRUE	TRUE	TRUE	TRUE	TRUE
1	DPLQPELDS	1121	1129	9	HLA-A*02:01	1121	0.006	-2.9	1.25E-01	-3.84E-02	50	0.006	-2.9	0.1247	-0.03844	50	TRUE	TRUE	TRUE	TRUE	TRUE
1	PLQPELDSF	1122	1130	9	HLA-A*02:01	1122	0.055	1.903	8.66E-01	2.97E-01	16	0.055	1.903	0.8656	0.29734	16	TRUE	TRUE	TRUE	TRUE	TRUE
1	LQPELDSFK	1123	1131	9	HLA-A*02:01	1123	0.06	0.384	2.63E-01	1.29E-01	50	0.06	0.384	0.26256	0.12868	50	TRUE	TRUE	TRUE	TRUE	TRUE
1	QPELDSFKE	1124	1132	9	HLA-A*02:01	1124	0.005	-1.953	2.44E-01	1.11E-02	50	0.005	-1.953	0.24392	0.01106	50	TRUE	TRUE	TRUE	TRUE	TRUE
1	PELDSFKEE	1125	1133	9	HLA-A*02:01	1125	0.005	-2.212	2.78E-02	-4.41E-02	50	0.005	-2.212	0.02779	-0.04405	50	TRUE	TRUE	TRUE	TRUE	TRUE
1	ELDSFKEEL	1126	1134	9	HLA-A*02:01	1126	0.2	0.449	8.27E-01	3.97E-01	8	0.2	0.449	0.82668	0.39723	8	TRUE	TRUE	TRUE	TRUE	TRUE
1	LDSFKEELD	1127	1135	9	HLA-A*02:01	1127	0.006	-2.29	2.67E-02	-4.52E-02	50	0.006	-2.29	0.02673	-0.04523	50	TRUE	TRUE	TRUE	TRUE	TRUE
1	DSFKEELDK	1128	1136	9	HLA-A*02:01	1128	0.008	0.349	6.85E-01	1.71E-01	50	0.008	0.349	0.6846	0.17076	50	TRUE	TRUE	TRUE	TRUE	TRUE
1	SFKEELDKY	1129	1137	9	HLA-A*02:01	1129	0.023	3.004	9.38E-01	3.09E-01	16	0.023	3.004	0.93752	0.30904	16	TRUE	TRUE	TRUE	TRUE	TRUE
1	FKEELDKYF	1130	1138	9	HLA-A*02:01	1130	0.024	2.399	3.31E-01	1.59E-01	50	0.024	2.399	0.33136	0.15853	50	TRUE	TRUE	TRUE	TRUE	TRUE
1	KEELDKYFK	1131	1139	9	HLA-A*02:01	1131	0.017	0.187	6.00E-01	1.57E-01	50	0.017	0.187	0.59999	0.15667	50	TRUE	TRUE	TRUE	TRUE	TRUE
1	EELDKYFKN	1132	1140	9	HLA-A*02:01	1132	0.008	-1.536	2.90E-01	3.49E-02	50	0.008	-1.536	0.29035	0.03493	50	TRUE	TRUE	TRUE	TRUE	TRUE
1	ELDKYFKNH	1133	1141	9	HLA-A*02:01	1133	0.035	-1.063	4.57E-01	1.11E-01	50	0.035	-1.063	0.45662	0.11116	50	TRUE	TRUE	TRUE	TRUE	TRUE
1	LDKYFKNHT	1134	1142	9	HLA-A*02:01	1134	0.011	-1.178	9.13E-02	2.10E-03	50	0.011	-1.178	0.09132	0.0021	50	TRUE	TRUE	TRUE	TRUE	TRUE
1	DKYFKNHTS	1135	1143	9	HLA-A*02:01	1135	0.01	-2.277	1.09E-01	-2.25E-02	50	0.01	-2.277	0.10854	-0.0225	50	TRUE	TRUE	TRUE	TRUE	TRUE
1	KYFKNHTSP	1136	1144	9	HLA-A*02:01	1136	0.014	0.449	7.51E-01	1.94E-01	50	0.014	0.449	0.7514	0.19429	50	TRUE	TRUE	TRUE	TRUE	TRUE
1	YFKNHTSPD	1137	1145	9	HLA-A*02:01	1137	0.016	-1.569	3.63E-02	-1.51E-02	50	0.016	-1.569	0.03628	-0.01506	50	TRUE	TRUE	TRUE	TRUE	TRUE
1	FKNHTSPDV	1138	1146	9	HLA-A*02:01	1138	0.074	0.123	8.78E-01	2.75E-01	32	0.074	0.123	0.87813	0.27465	32	TRUE	TRUE	TRUE	TRUE	TRUE
1	KNHTSPDVD	1139	1147	9	HLA-A*02:01	1139	0.004	-1.936	2.56E-02	-3.86E-02	50	0.004	-1.936	0.02561	-0.03864	50	TRUE	TRUE	TRUE	TRUE	TRUE
1	NHTSPDVDL	1140	1148	9	HLA-A*02:01	1140	0.018	1.064	6.43E-01	1.89E-01	50	0.018	1.064	0.6432	0.18932	50	TRUE	TRUE	TRUE	TRUE	TRUE
1	HTSPDVDLG	1141	1149	9	HLA-A*02:01	1141	0.019	-1.371	6.92E-02	2.90E-04	50	0.019	-1.371	0.06917	0.00029	50	TRUE	TRUE	TRUE	TRUE	TRUE
1	TSPDVDLGD	1142	1150	9	HLA-A*02:01	1142	0.013	-1.834	3.14E-02	-2.58E-02	50	0.013	-1.834	0.03142	-0.02578	50	TRUE	TRUE	TRUE	TRUE	TRUE
1	SPDVDLGDI	1143	1151	9	HLA-A*02:01	1143	0.029	0.117	6.90E-01	1.87E-01	50	0.029	0.117	0.69033	0.18725	50	TRUE	TRUE	TRUE	TRUE	TRUE
1	PDVDLGDIS	1144	1152	9	HLA-A*02:01	1144	0.004	-2.901	2.34E-02	-6.33E-02	50	0.004	-2.901	0.02341	-0.06326	50	TRUE	TRUE	TRUE	TRUE	TRUE
1	DVDLGDISG	1145	1153	9	HLA-A*02:01	1145	0.02	-1.894	4.13E-02	-1.81E-02	50	0.02	-1.894	0.04131	-0.01805	50	TRUE	TRUE	TRUE	TRUE	TRUE
1	VDLGDISGI	1146	1154	9	HLA-A*02:01	1146	0.061	0.168	2.84E-01	1.29E-01	50	0.061	0.168	0.28429	0.12917	50	TRUE	TRUE	TRUE	TRUE	TRUE
1	DLGDISGIN	1147	1155	9	HLA-A*02:01	1147	0.014	-1.728	4.00E-02	-2.02E-02	50	0.014	-1.728	0.03997	-0.02021	50	TRUE	TRUE	TRUE	TRUE	TRUE
1	LGDISGINA	1148	1156	9	HLA-A*02:01	1148	0.063	-1.113	6.07E-02	4.88E-02	50	0.063	-1.113	0.06069	0.04883	50	TRUE	TRUE	TRUE	TRUE	TRUE
1	GDISGINAS	1149	1157	9	HLA-A*02:01	1149	0.018	-2.615	2.90E-02	-4.08E-02	50	0.018	-2.615	0.02902	-0.04084	50	TRUE	TRUE	TRUE	TRUE	TRUE
1	DISGINASV	1150	1158	9	HLA-A*02:01	1150	0.21	0.093	7.39E-01	3.79E-01	9	0.21	0.093	0.73878	0.37855	9	TRUE	TRUE	TRUE	TRUE	TRUE
1	ISGINASVV	1151	1159	9	HLA-A*02:01	1151	0.107	0.076	6.28E-01	2.50E-01	32	0.107	0.076	0.62846	0.2503	32	TRUE	TRUE	TRUE	TRUE	TRUE
1	SGINASVVN	1152	1160	9	HLA-A*02:01	1152	0.01	-1.451	3.46E-02	-1.85E-02	50	0.01	-1.451	0.03464	-0.01848	50	TRUE	TRUE	TRUE	TRUE	TRUE
1	GINASVVNI	1153	1161	9	HLA-A*02:01	1153	0.421	0.444	5.49E-01	5.56E-01	4	0.421	0.444	0.54906	0.55564	4	TRUE	TRUE	TRUE	TRUE	TRUE
1	INASVVNIQ	1154	1162	9	HLA-A*02:01	1154	0.015	-0.248	3.13E-02	1.59E-02	50	0.015	-0.248	0.03132	0.01585	50	TRUE	TRUE	TRUE	TRUE	TRUE
1	NASVVNIQK	1155	1163	9	HLA-A*02:01	1155	0.019	0.559	9.49E-01	2.47E-01	32	0.019	0.559	0.94929	0.24656	32	TRUE	TRUE	TRUE	TRUE	TRUE
1	ASVVNIQKE	1156	1164	9	HLA-A*02:01	1156	0.011	-1.162	3.54E-02	-1.01E-02	50	0.011	-1.162	0.03537	-0.01009	50	TRUE	TRUE	TRUE	TRUE	TRUE
1	SVVNIQKEI	1157	1165	9	HLA-A*02:01	1157	0.145	0.958	7.07E-01	3.28E-01	16	0.145	0.958	0.70723	0.32808	16	TRUE	TRUE	TRUE	TRUE	TRUE
1	VVNIQKEID	1158	1166	9	HLA-A*02:01	1158	0.008	-1.655	2.44E-02	-2.79E-02	50	0.008	-1.655	0.0244	-0.02788	50	TRUE	TRUE	TRUE	TRUE	TRUE
1	VNIQKEIDR	1159	1167	9	HLA-A*02:01	1159	0.007	1.51	4.68E-02	5.53E-02	50	0.007	1.51	0.0468	0.05528	50	TRUE	TRUE	TRUE	TRUE	TRUE
1	NIQKEIDRL	1160	1168	9	HLA-A*02:01	1160	0.111	1.135	3.93E-01	2.28E-01	32	0.111	1.135	0.39292	0.22778	32	TRUE	TRUE	TRUE	TRUE	TRUE
1	IQKEIDRLN	1161	1169	9	HLA-A*02:01	1161	0.016	-1.078	2.80E-02	-4.66E-03	50	0.016	-1.078	0.02796	-0.00466	50	TRUE	TRUE	TRUE	TRUE	TRUE
1	QKEIDRLNE	1162	1170	9	HLA-A*02:01	1162	0.003	-1.638	2.25E-02	-3.29E-02	50	0.003	-1.638	0.02248	-0.03289	50	TRUE	TRUE	TRUE	TRUE	TRUE
1	KEIDRLNEV	1163	1171	9	HLA-A*02:01	1163	0.197	0.306	9.57E-01	4.20E-01	7	0.197	0.306	0.95693	0.41996	7	TRUE	TRUE	TRUE	TRUE	TRUE
1	EIDRLNEVA	1164	1172	9	HLA-A*02:01	1164	0.071	-0.686	2.72E-01	1.15E-01	50	0.071	-0.686	0.27184	0.11501	50	TRUE	TRUE	TRUE	TRUE	TRUE
1	IDRLNEVAK	1165	1173	9	HLA-A*02:01	1165	0.007	0.062	3.49E-01	8.70E-02	50	0.007	0.062	0.3485	0.08696	50	TRUE	TRUE	TRUE	TRUE	TRUE
1	DRLNEVAKN	1166	1174	9	HLA-A*02:01	1166	0.003	-1.398	9.04E-02	-1.16E-02	50	0.003	-1.398	0.09037	-0.01162	50	TRUE	TRUE	TRUE	TRUE	TRUE
1	RLNEVAKNL	1167	1175	9	HLA-A*02:01	1167	0.484	0.985	9.66E-01	7.26E-01	2	0.484	0.985	0.9655	0.72586	2	TRUE	TRUE	TRUE	TRUE	TRUE
1	LNEVAKNLN	1168	1176	9	HLA-A*02:01	1168	0.004	-1.476	2.58E-02	-2.71E-02	50	0.004	-1.476	0.02577	-0.0271	50	TRUE	TRUE	TRUE	TRUE	TRUE
1	NEVAKNLNE	1169	1177	9	HLA-A*02:01	1169	0.007	-1.538	2.53E-02	-2.58E-02	50	0.007	-1.538	0.02534	-0.02575	50	TRUE	TRUE	TRUE	TRUE	TRUE
1	EVAKNLNES	1170	1178	9	HLA-A*02:01	1170	0.029	-2.153	9.16E-02	-4.21E-03	50	0.029	-2.153	0.09162	-0.00421	50	TRUE	TRUE	TRUE	TRUE	TRUE
1	VAKNLNESL	1171	1179	9	HLA-A*02:01	1171	0.048	0.9	7.26E-01	2.34E-01	32	0.048	0.9	0.72555	0.23375	32	TRUE	TRUE	TRUE	TRUE	TRUE
1	AKNLNESLI	1172	1180	9	HLA-A*02:01	1172	0.032	0.681	3.90E-01	1.37E-01	50	0.032	0.681	0.3904	0.13687	50	TRUE	TRUE	TRUE	TRUE	TRUE
1	KNLNESLID	1173	1181	9	HLA-A*02:01	1173	0.009	-1.689	2.89E-02	-2.67E-02	50	0.009	-1.689	0.02887	-0.02673	50	TRUE	TRUE	TRUE	TRUE	TRUE
1	NLNESLIDL	1174	1182	9	HLA-A*02:01	1174	0.612	1.018	9.46E-01	8.50E-01	1	0.612	1.018	0.94598	0.8503	1	TRUE	TRUE	TRUE	TRUE	TRUE
1	LNESLIDLQ	1175	1183	9	HLA-A*02:01	1175	0.007	-0.257	2.89E-02	7.07E-03	50	0.007	-0.257	0.02887	0.00707	50	TRUE	TRUE	TRUE	TRUE	TRUE
1	NESLIDLQE	1176	1184	9	HLA-A*02:01	1176	0.01	-1.62	3.28E-02	-2.31E-02	50	0.01	-1.62	0.03281	-0.02312	50	TRUE	TRUE	TRUE	TRUE	TRUE
1	ESLIDLQEL	1177	1185	9	HLA-A*02:01	1177	0.048	0.95	7.12E-01	2.32E-01	32	0.048	0.95	0.71233	0.23202	32	TRUE	TRUE	TRUE	TRUE	TRUE
1	SLIDLQELG	1178	1186	9	HLA-A*02:01	1178	0.144	-1.421	2.97E-02	1.15E-01	50	0.144	-1.421	0.0297	0.11516	50	TRUE	TRUE	TRUE	TRUE	TRUE
1	LIDLQELGK	1179	1187	9	HLA-A*02:01	1179	0.02	0.475	5.24E-01	1.50E-01	50	0.02	0.475	0.52358	0.14968	50	TRUE	TRUE	TRUE	TRUE	TRUE
1	IDLQELGKY	1180	1188	9	HLA-A*02:01	1180	0.011	2.706	5.53E-01	2.03E-01	32	0.011	2.706	0.55284	0.20304	32	TRUE	TRUE	TRUE	TRUE	TRUE
1	DLQELGKYE	1181	1189	9	HLA-A*02:01	1181	0.014	-1.885	2.53E-02	-2.74E-02	50	0.014	-1.885	0.02531	-0.02743	50	TRUE	TRUE	TRUE	TRUE	TRUE
1	LQELGKYEQ	1182	1190	9	HLA-A*02:01	1182	0.022	-0.309	5.74E-02	2.72E-02	50	0.022	-0.309	0.05736	0.02718	50	TRUE	TRUE	TRUE	TRUE	TRUE
1	QELGKYEQY	1183	1191	9	HLA-A*02:01	1183	0.015	2.868	5.39E-01	2.08E-01	32	0.015	2.868	0.53881	0.20793	32	TRUE	TRUE	TRUE	TRUE	TRUE
1	ELGKYEQYI	1184	1192	9	HLA-A*02:01	1184	0.21	0.138	1.66E-01	2.51E-01	32	0.21	0.138	0.16585	0.25077	32	TRUE	TRUE	TRUE	TRUE	TRUE
1	LGKYEQYIK	1185	1193	9	HLA-A*02:01	1185	0.008	0.033	5.70E-02	2.16E-02	50	0.008	0.033	0.05696	0.02164	50	TRUE	TRUE	TRUE	TRUE	TRUE
1	GKYEQYIKW	1186	1194	9	HLA-A*02:01	1186	0.029	0.99	4.48E-01	1.54E-01	50	0.029	0.99	0.44765	0.15447	50	TRUE	TRUE	TRUE	TRUE	TRUE
1	KYEQYIKWP	1187	1195	9	HLA-A*02:01	1187	0.01	0.302	2.63E-02	2.35E-02	50	0.01	0.302	0.02625	0.02346	50	TRUE	TRUE	TRUE	TRUE	TRUE
1	YEQYIKWPW	1188	1196	9	HLA-A*02:01	1188	0.058	0.798	4.12E-01	1.71E-01	50	0.058	0.798	0.41234	0.17073	50	TRUE	TRUE	TRUE	TRUE	TRUE
1	EQYIKWPWY	1189	1197	9	HLA-A*02:01	1189	0.056	2.931	2.06E-01	1.76E-01	50	0.056	2.931	0.20613	0.17565	50	TRUE	TRUE	TRUE	TRUE	TRUE
1	QYIKWPWYV	1190	1198	9	HLA-A*02:01	1190	0.44	0.494	8.29E-01	6.39E-01	3	0.44	0.494	0.82903	0.63888	3	TRUE	TRUE	TRUE	TRUE	TRUE
1	YIKWPWYVW	1191	1199	9	HLA-A*02:01	1191	0.081	0.942	8.89E-01	3.05E-01	16	0.081	0.942	0.88866	0.3045	16	TRUE	TRUE	TRUE	TRUE	TRUE
1	IKWPWYVWL	1192	1200	9	HLA-A*02:01	1192	0.218	1.147	8.15E-01	4.30E-01	7	0.218	1.147	0.81462	0.42996	7	TRUE	TRUE	TRUE	TRUE	TRUE
1	KWPWYVWLG	1193	1201	9	HLA-A*02:01	1193	0.067	-1.048	7.26E-02	5.71E-02	50	0.067	-1.048	0.07258	0.05713	50	TRUE	TRUE	TRUE	TRUE	TRUE
1	WPWYVWLGF	1194	1202	9	HLA-A*02:01	1194	0.071	2.476	8.63E-01	3.27E-01	16	0.071	2.476	0.86343	0.32717	16	TRUE	TRUE	TRUE	TRUE	TRUE
1	PWYVWLGFI	1195	1203	9	HLA-A*02:01	1195	0.081	0.432	5.54E-02	1.04E-01	50	0.081	0.432	0.05538	0.10426	50	TRUE	TRUE	TRUE	TRUE	TRUE
1	WYVWLGFIA	1196	1204	9	HLA-A*02:01	1196	0.09	-0.365	3.89E-02	8.96E-02	50	0.09	-0.365	0.03888	0.08962	50	TRUE	TRUE	TRUE	TRUE	TRUE
1	YVWLGFIAG	1197	1205	9	HLA-A*02:01	1197	0.201	-1.161	1.07E-01	1.96E-01	50	0.201	-1.161	0.10717	0.19609	50	TRUE	TRUE	TRUE	TRUE	TRUE
1	VWLGFIAGL	1198	1206	9	HLA-A*02:01	1198	0.283	1.229	2.62E-01	3.73E-01	9	0.283	1.229	0.26223	0.37273	9	TRUE	TRUE	TRUE	TRUE	TRUE
1	WLGFIAGLI	1199	1207	9	HLA-A*02:01	1199	0.409	0.411	1.62E-01	4.56E-01	6	0.409	0.411	0.16154	0.45562	6	TRUE	TRUE	TRUE	TRUE	TRUE
1	LGFIAGLIA	1200	1208	9	HLA-A*02:01	1200	0.078	-0.742	2.78E-02	6.57E-02	50	0.078	-0.742	0.02783	0.06571	50	TRUE	TRUE	TRUE	TRUE	TRUE

seq #	peptide	start	end	peptide length	allele	peptide index	NetCTLpan MH	NetCTLpan tap	NetCTLpan clea	NetCTLpan con	NetCTLpan per	legacy mhc_pre	legacy tap_pre	legacy cleavage	legacy combine	legacy %_rank	equal mhc_pre	equal tap_pred	equal cleavage	equal combine	equal %_rank
1	GFIAGLIAI	1201	1209	9	HLA-A*02:01	1201	0.321	0.64	9.05E-02	3.57E-01	10	0.321	0.64	0.09046	0.35735	10	TRUE	TRUE	TRUE	TRUE	TRUE
1	FIAGLIAIV	1202	1210	9	HLA-A*02:01	1202	0.845	0.335	2.14E-01	9.02E-01	0.8	0.845	0.335	0.21437	0.90161	0.8	TRUE	TRUE	TRUE	TRUE	TRUE
1	IAGLIAIVM	1203	1211	9	HLA-A*02:01	1203	0.116	0.112	1.25E-01	1.47E-01	50	0.116	0.112	0.12527	0.14699	50	TRUE	TRUE	TRUE	TRUE	TRUE
1	AGLIAIVMV	1204	1212	9	HLA-A*02:01	1204	0.208	0.219	4.72E-01	3.20E-01	16	0.208	0.219	0.47214	0.31971	16	TRUE	TRUE	TRUE	TRUE	TRUE
1	GLIAIVMVT	1205	1213	9	HLA-A*02:01	1205	0.453	-0.642	7.05E-02	4.53E-01	6	0.453	-0.642	0.07045	0.4528	6	TRUE	TRUE	TRUE	TRUE	TRUE
1	LIAIVMVTI	1206	1214	9	HLA-A*02:01	1206	0.444	0.654	7.72E-01	6.34E-01	3	0.444	0.654	0.77209	0.63407	3	TRUE	TRUE	TRUE	TRUE	TRUE
1	IAIVMVTIL	1207	1215	9	HLA-A*02:01	1207	0.181	1.147	8.66E-01	4.05E-01	8	0.181	1.147	0.8662	0.40457	8	TRUE	TRUE	TRUE	TRUE	TRUE
1	AIVMVTILL	1208	1216	9	HLA-A*02:01	1208	0.365	1.314	9.34E-01	6.08E-01	3	0.365	1.314	0.93432	0.60807	3	TRUE	TRUE	TRUE	TRUE	TRUE
1	IVMVTILLC	1209	1217	9	HLA-A*02:01	1209	0.221	0.395	4.55E-02	2.41E-01	32	0.221	0.395	0.04549	0.24111	32	TRUE	TRUE	TRUE	TRUE	TRUE
1	VMVTILLCC	1210	1218	9	HLA-A*02:01	1210	0.281	0.285	4.61E-02	2.98E-01	16	0.281	0.285	0.04609	0.29849	16	TRUE	TRUE	TRUE	TRUE	TRUE
1	MVTILLCCM	1211	1219	9	HLA-A*02:01	1211	0.195	0.501	4.08E-01	2.99E-01	16	0.195	0.501	0.40793	0.29931	16	TRUE	TRUE	TRUE	TRUE	TRUE
1	VTILLCCMT	1212	1220	9	HLA-A*02:01	1212	0.106	-0.56	9.80E-02	1.14E-01	50	0.106	-0.56	0.09801	0.11405	50	TRUE	TRUE	TRUE	TRUE	TRUE
1	TILLCCMTS	1213	1221	9	HLA-A*02:01	1213	0.137	-2.073	1.57E-01	1.20E-01	50	0.137	-2.073	0.15655	0.1204	50	TRUE	TRUE	TRUE	TRUE	TRUE
1	ILLCCMTSC	1214	1222	9	HLA-A*02:01	1214	0.43	0.085	7.94E-02	4.50E-01	6	0.43	0.085	0.07935	0.44998	6	TRUE	TRUE	TRUE	TRUE	TRUE
1	LLCCMTSCC	1215	1223	9	HLA-A*02:01	1215	0.171	0.056	3.39E-02	1.80E-01	50	0.171	0.056	0.03391	0.18003	50	TRUE	TRUE	TRUE	TRUE	TRUE
1	LCCMTSCCS	1216	1224	9	HLA-A*02:01	1216	0.008	-2.164	2.72E-02	-4.00E-02	50	0.008	-2.164	0.02715	-0.03999	50	TRUE	TRUE	TRUE	TRUE	TRUE
1	CCMTSCCSC	1217	1225	9	HLA-A*02:01	1217	0.043	0.107	2.55E-02	5.14E-02	50	0.043	0.107	0.02551	0.05141	50	TRUE	TRUE	TRUE	TRUE	TRUE
1	CMTSCCSCL	1218	1226	9	HLA-A*02:01	1218	0.359	0.992	8.30E-01	5.71E-01	4	0.359	0.992	0.82976	0.5705	4	TRUE	TRUE	TRUE	TRUE	TRUE
1	MTSCCSCLK	1219	1227	9	HLA-A*02:01	1219	0.058	0.479	7.09E-01	2.30E-01	32	0.058	0.479	0.70915	0.22953	32	TRUE	TRUE	TRUE	TRUE	TRUE
1	TSCCSCLKG	1220	1228	9	HLA-A*02:01	1220	0.012	-1.331	5.90E-02	-7.99E-03	50	0.012	-1.331	0.05903	-0.00799	50	TRUE	TRUE	TRUE	TRUE	TRUE
1	SCCSCLKGA	1221	1229	9	HLA-A*02:01	1221	0.038	-0.443	3.91E-02	3.57E-02	50	0.038	-0.443	0.0391	0.03572	50	TRUE	TRUE	TRUE	TRUE	TRUE
1	CCSCLKGAC	1222	1230	9	HLA-A*02:01	1222	0.015	0.044	0.02338	0.02136	50	0.015	0.044	0.02338	0.02136	50	TRUE	TRUE	TRUE	TRUE	TRUE
1	CSCLKGACS	1223	1231	9	HLA-A*02:01	1223	0.013	-2.244	0.02571	-0.03732	50	0.013	-2.244	0.02571	-0.03732	50	TRUE	TRUE	TRUE	TRUE	TRUE
1	SCLKGACSC	1224	1232	9	HLA-A*02:01	1224	0.044	0.099	0.03277	0.05385	50	0.044	0.099	0.03277	0.05385	50	TRUE	TRUE	TRUE	TRUE	TRUE
1	CLKGACSCG	1225	1233	9	HLA-A*02:01	1225	0.03	-1.365	0.31899	0.06765	50	0.03	-1.365	0.31899	0.06765	50	TRUE	TRUE	TRUE	TRUE	TRUE
1	LKGACSCGS	1226	1234	9	HLA-A*02:01	1226	0.01	-2.362	0.03437	-0.04132	50	0.01	-2.362	0.03437	-0.04132	50	TRUE	TRUE	TRUE	TRUE	TRUE
1	KGACSCGSC	1227	1235	9	HLA-A*02:01	1227	0.024	-0.08	0.0343	0.02972	50	0.024	-0.08	0.0343	0.02972	50	TRUE	TRUE	TRUE	TRUE	TRUE
1	GACSCGSCC	1228	1236	9	HLA-A*02:01	1228	0.02	-0.002	0.02893	0.02646	50	0.02	-0.002	0.02893	0.02646	50	TRUE	TRUE	TRUE	TRUE	TRUE
1	ACSCGSCCK	1229	1237	9	HLA-A*02:01	1229	0.008	0.595	0.80577	0.20417	32	0.008	0.595	0.80577	0.20417	32	TRUE	TRUE	TRUE	TRUE	TRUE
1	CSCGSCCKF	1230	1238	9	HLA-A*02:01	1230	0.028	2.697	0.92157	0.30278	16	0.028	2.697	0.92157	0.30278	16	TRUE	TRUE	TRUE	TRUE	TRUE
1	SCGSCCKFD	1231	1239	9	HLA-A*02:01	1231	0.005	-1.912	0.02312	-0.0376	50	0.005	-1.912	0.02312	-0.0376	50	TRUE	TRUE	TRUE	TRUE	TRUE
1	CGSCCKFDE	1232	1240	9	HLA-A*02:01	1232	0.006	-1.823	0.02217	-0.03459	50	0.006	-1.823	0.02217	-0.03459	50	TRUE	TRUE	TRUE	TRUE	TRUE
1	GSCCKFDED	1233	1241	9	HLA-A*02:01	1233	0.008	-1.952	0.0259	-0.03497	50	0.008	-1.952	0.0259	-0.03497	50	TRUE	TRUE	TRUE	TRUE	TRUE
1	SCCKFDEDD	1234	1242	9	HLA-A*02:01	1234	0.004	-1.763	0.02431	-0.03461	50	0.004	-1.763	0.02431	-0.03461	50	TRUE	TRUE	TRUE	TRUE	TRUE
1	CCKFDEDDS	1235	1243	9	HLA-A*02:01	1235	0.004	-2.214	0.02244	-0.0463	50	0.004	-2.214	0.02244	-0.0463	50	TRUE	TRUE	TRUE	TRUE	TRUE
1	CKFDEDDSE	1236	1244	9	HLA-A*02:01	1236	0.004	-1.452	0.02301	-0.02712	50	0.004	-1.452	0.02301	-0.02712	50	TRUE	TRUE	TRUE	TRUE	TRUE
1	KFDEDDSEP	1237	1245	9	HLA-A*02:01	1237	0.015	0.196	0.03675	0.02817	50	0.015	0.196	0.03675	0.02817	50	TRUE	TRUE	TRUE	TRUE	TRUE
1	FDEDDSEPV	1238	1246	9	HLA-A*02:01	1238	0.07	-0.131	0.27464	0.12852	50	0.07	-0.131	0.27464	0.12852	50	TRUE	TRUE	TRUE	TRUE	TRUE
1	DEDDSEPVL	1239	1247	9	HLA-A*02:01	1239	0.019	0.222	0.95055	0.23842	32	0.019	0.222	0.95055	0.23842	32	TRUE	TRUE	TRUE	TRUE	TRUE
1	EDDSEPVLK	1240	1248	9	HLA-A*02:01	1240	0.008	-0.362	0.31025	0.06876	50	0.008	-0.362	0.31025	0.06876	50	TRUE	TRUE	TRUE	TRUE	TRUE
1	DDSEPVLKG	1241	1249	9	HLA-A*02:01	1241	0.005	-2.19	0.15266	-0.0154	50	0.005	-2.19	0.15266	-0.0154	50	TRUE	TRUE	TRUE	TRUE	TRUE
1	DSEPVLKGV	1242	1250	9	HLA-A*02:01	1242	0.027	-0.244	0.62365	0.16122	50	0.027	-0.244	0.62365	0.16122	50	TRUE	TRUE	TRUE	TRUE	TRUE
1	SEPVLKGVK	1243	1251	9	HLA-A*02:01	1243	0.008	0.228	0.35753	0.09414	50	0.008	0.228	0.35753	0.09414	50	TRUE	TRUE	TRUE	TRUE	TRUE
1	EPVLKGVKL	1244	1252	9	HLA-A*02:01	1244	0.021	0.578	0.97679	0.25523	32	0.021	0.578	0.97679	0.25523	32	TRUE	TRUE	TRUE	TRUE	TRUE
1	PVLKGVKLH	1245	1253	9	HLA-A*02:01	1245	0.014	-1.026	0.38649	0.07531	50	0.014	-1.026	0.38649	0.07531	50	TRUE	TRUE	TRUE	TRUE	TRUE
1	VLKGVKLHY	1246	1254	9	HLA-A*02:01	1246	0.056	2.859	0.97833	0.3476	10	0.056	2.859	0.97833	0.3476	10	TRUE	TRUE	TRUE	TRUE	TRUE
1	LKGVKLHYT	1247	1255	9	HLA-A*02:01	1247	0.02	-0.768	0.11875	0.02752	50	0.02	-0.768	0.11875	0.02752	50	TRUE	TRUE	TRUE	TRUE	TRUE

Cluster.Sub-Clu	Peptide Numbe	Alignment	Position	Input seq id	Peptide	Cluster Consensus		legacy Cluster.S	legacy Peptide	legacy Alignme	legacy Position	legacy Descript	legacy Peptide	equal Cluster.S	equal Peptide N	equal Alignme	equal Position	equal Descripti	equal Peptide
1.1	Consensus	TFQLMXLYGRX	-	-	-	TFQLMXLYGRXXDLSSDIKEKFAKLCEA		1.1	Consensus	TFQLMXLYGRX	-	-	-	TRUE	TRUE	TRUE	TRUE	TRUE	TRUE
1.1	1	TFQLMVLYGRT	1	Rat Pep17	TFQLMVLYGRT	TFQLMXLYGRXXDLSSDIKEKFAKLCEA		1.1	1	TFQLMVLYGRT	1	Rat Pep17	TFQLMVLYGRT	TRUE	TRUE	TRUE	TRUE	TRUE	TRUE
1.1	2	-----GLYGREPD	6	Mus Pep3	GLYGREPDLSSI	TFQLMXLYGRXXDLSSDIKEKFAKLCEA		1.1	2	-----GLYGREPD	6	Mus Pep3	GLYGREPDLSSI	TRUE	TRUE	TRUE	TRUE	TRUE	TRUE
1.1	3	-------YGREPDL	8	Mus Pep7	YGREPDLSLDIK	TFQLMXLYGRXXDLSSDIKEKFAKLCEA		1.1	3	-------YGREPDL	8	Mus Pep7	YGREPDLSLDIK	TRUE	TRUE	TRUE	TRUE	TRUE	TRUE
1.1	4	RTKDLSSDIKEK	9	Rat Pep9	GRTKDLSSDIKE	TFQLMXLYGRXXDLSSDIKEKFAKLCEA		1.1	4	RTKDLSSDIKEK	9	Rat Pep9	GRTKDLSSDIKE	TRUE	TRUE	TRUE	TRUE	TRUE	TRUE
2.1	Consensus	YDRYVMXHLIN	-	-	-	YDRYVMXHLINXKXGETFQLMXLYGRTK		2.1	Consensus	YDRYVMXHLIN	-	-	-	TRUE	TRUE	TRUE	TRUE	TRUE	TRUE
2.1	1	YDRYVMFHLIN	1	Rat Pep19	YDRYVMFHLIN	YDRYVMXHLINXKXGETFQLMXLYGRTK		2.1	1	YDRYVMFHLIN	1	Rat Pep19	YDRYVMFHLIN	TRUE	TRUE	TRUE	TRUE	TRUE	TRUE
2.1	2	------AHLINEKD	7	Mus Pep9	AHLINEKDGETF	YDRYVMXHLINXKXGETFQLMXLYGRTK		2.1	2	------AHLINEKD	7	Mus Pep9	AHLINEKDGETF	TRUE	TRUE	TRUE	TRUE	TRUE	TRUE
2.1	3	NFKNGETFQLM	9	Rat Pep12	LINFKNGETFQL	YDRYVMXHLINXKXGETFQLMXLYGRTK		2.1	3	NFKNGETFQLM	9	Rat Pep12	LINFKNGETFQL	TRUE	TRUE	TRUE	TRUE	TRUE	TRUE
2.1	4	----------NEKDG	11	Mus Pep6	NEKDGETFQLM	YDRYVMXHLINXKXGETFQLMXLYGRTK		2.1	4	----------NEKDG	11	Mus Pep6	NEKDGETFQLM	TRUE	TRUE	TRUE	TRUE	TRUE	TRUE
3.1	Consensus	FXXHIXVLENSL	-	-	-	FXXHIXVLENSLXFKFRIKE		3.1	Consensus	FXXHIXVLENSL	-	-	-	TRUE	TRUE	TRUE	TRUE	TRUE	TRUE
3.1	1	FMQHIDVLENS	1	Rat Pep6	FMQHIDVLENS	FXXHIXVLENSLXFKFRIKE		3.1	1	FMQHIDVLENS	1	Rat Pep6	FMQHIDVLENS	TRUE	TRUE	TRUE	TRUE	TRUE	TRUE
3.1	2	FVEHIHVLENSL	1	Mus Pep2	FVEHIHVLENSL	FXXHIXVLENSLXFKFRIKE		3.1	2	FVEHIHVLENSL	1	Mus Pep2	FVEHIHVLENSL	TRUE	TRUE	TRUE	TRUE	TRUE	TRUE
4.1	Consensus	EENGSMRVFX	-	-	-	EENGSMRVFXXHIXVLENSL		4.1	Consensus	EENGSMRVFX	-	-	-	TRUE	TRUE	TRUE	TRUE	TRUE	TRUE
4.1	1	EENGSMRVFM	1	Rat Pep4	EENGSMRVFM	EENGSMRVFXXHIXVLENSL		4.1	1	EENGSMRVFM	1	Rat Pep4	EENGSMRVFM	TRUE	TRUE	TRUE	TRUE	TRUE	TRUE
4.1	2	---GSMRVFVEH	4	Mus Pep16	GSMRVFVEHIH	EENGSMRVFXXHIXVLENSL		4.1	2	---GSMRVFVEH	4	Mus Pep16	GSMRVFVEHIH	TRUE	TRUE	TRUE	TRUE	TRUE	TRUE
5.1	Consensus	EEASSTGRNFN	-	-	-	EEASSTGRNFNVXKINGEWHTIIL		5.1	Consensus	EEASSTGRNFN	-	-	-	TRUE	TRUE	TRUE	TRUE	TRUE	TRUE
5.1	1	EEASSTGRNFN	1	Mus Pep10	EEASSTGRNFN	EEASSTGRNFNVXKINGEWHTIIL		5.1	1	EEASSTGRNFN	1	Mus Pep10	EEASSTGRNFN	TRUE	TRUE	TRUE	TRUE	TRUE	TRUE
5.1	2	---NVEKINGEW	11	Mus Pep13	NVEKINGEWHT	EEASSTGRNFNVXKINGEWHTIIL		5.1	2	---NVEKINGEW	11	Mus Pep13	NVEKINGEWHT	TRUE	TRUE	TRUE	TRUE	TRUE	TRUE
6.1	Consensus	GXWFSIXXASX	-	-	-	GXWFSIXXASXKREKIEENG		6.1	Consensus	GXWFSIXXASX	-	-	-	TRUE	TRUE	TRUE	TRUE	TRUE	TRUE
6.1	1	GDWFSIVVASN	1	Rat Pep8	GDWFSIVVASN	GXWFSIXXASXKREKIEENG		6.1	1	GDWFSIVVASN	1	Rat Pep8	GDWFSIVVASN	TRUE	TRUE	TRUE	TRUE	TRUE	TRUE
6.1	2	-EWFSILLASDK	2	Mus Pep4	EWFSILLASDKR	GXWFSIXXASXKREKIEENG		6.1	2	-EWFSILLASDK	2	Mus Pep4	EWFSILLASDKR	TRUE	TRUE	TRUE	TRUE	TRUE	TRUE
7.1	Consensus	FVEYDGXNTFT	-	-	-	FVEYDGXNTFTILKTDYDXY		7.1	Consensus	FVEYDGXNTFT	-	-	-	TRUE	TRUE	TRUE	TRUE	TRUE	TRUE
7.1	1	FVEYDGGNTFT	1	Rat Pep7	FVEYDGGNTFT	FVEYDGXNTFTILKTDYDXY		7.1	1	FVEYDGGNTFT	1	Rat Pep7	FVEYDGGNTFT	TRUE	TRUE	TRUE	TRUE	TRUE	TRUE
7.1	2	----DGFNTFTIL	5	Mus Pep5	DGFNTFTILKTD	FVEYDGXNTFTILKTDYDXY		7.1	2	----DGFNTFTIL	5	Mus Pep5	DGFNTFTILKTD	TRUE	TRUE	TRUE	TRUE	TRUE	TRUE
8.1	Consensus	RXNIIDLTKTXR	-	-	-	RXNIIDLTKTXRCLXARG		8.1	Consensus	RXNIIDLTKTXR	-	-	-	TRUE	TRUE	TRUE	TRUE	TRUE	TRUE
8.1	1	RDNIIDLTKTDR	1	Rat Pep14	RDNIIDLTKTDR	RXNIIDLTKTXRCLXARG		8.1	1	RDNIIDLTKTDR	1	Rat Pep14	RDNIIDLTKTDR	TRUE	TRUE	TRUE	TRUE	TRUE	TRUE
8.1	2	-ENIIDLTKTNR	2	Mus Pep17	ENIIDLTKTNRC	RXNIIDLTKTXRCLXARG		8.1	2	-ENIIDLTKTNR	2	Mus Pep17	ENIIDLTKTNRC	TRUE	TRUE	TRUE	TRUE	TRUE	TRUE
9.1	Singleton	LEQIHVLENSLV	-	Mus Pep1	LEQIHVLENSLV	LEQIHVLENSLVL		9.1	Singleton	LEQIHVLENSLV	-	Mus Pep1	LEQIHVLENSLV	TRUE	TRUE	TRUE	TRUE	TRUE	TRUE
10.1	Singleton	LENSLVLKFHTV	-	Mus Pep8	LENSLVLKFHTV	LENSLVLKFHTVRDE		10.1	Singleton	LENSLVLKFHTV	-	Mus Pep8	LENSLVLKFHTV	TRUE	TRUE	TRUE	TRUE	TRUE	TRUE
11.1	Singleton	SRPFIFQEVIDL	-	Mus Pep12	SRPFIFQEVIDL	SRPFIFQEVIDLGGE		11.1	Singleton	SRPFIFQEVIDL	-	Mus Pep12	SRPFIFQEVIDL	TRUE	TRUE	TRUE	TRUE	TRUE	TRUE
12.1	Singleton	EELRRLAPITSD	-	Mus Pep22	EELRRLAPITSD	EELRRLAPITSDPTE		12.1	Singleton	EELRRLAPITSD	-	Mus Pep22	EELRRLAPITSD	TRUE	TRUE	TRUE	TRUE	TRUE	TRUE
13.1	Singleton	EEASSTRGNLD	-	Rat Pep3	EEASSTRGNLD	EEASSTRGNLDVAKLNGDWF		13.1	Singleton	EEASSTRGNLD	-	Rat Pep3	EEASSTRGNLD	TRUE	TRUE	TRUE	TRUE	TRUE	TRUE
14.1	Singleton	ENSLGFKFRIKE	-	Rat Pep5	ENSLGFKFRIKE	ENSLGFKFRIKENGECRELY		14.1	Singleton	ENSLGFKFRIKE	-	Rat Pep5	ENSLGFKFRIKE	TRUE	TRUE	TRUE	TRUE	TRUE	TRUE
15.1	Singleton	DIKEKFAKLCEA	-	Rat Pep2	DIKEKFAKLCEA	DIKEKFAKLCEAHGITRDNI		15.1	Singleton	DIKEKFAKLCEA	-	Rat Pep2	DIKEKFAKLCEA	TRUE	TRUE	TRUE	TRUE	TRUE	TRUE
16.1	Singleton	NLDVAKLNGD	-	Rat Pep13	NLDVAKLNGD	NLDVAKLNGDWFSIVVASNK		16.1	Singleton	NLDVAKLNGD	-	Rat Pep13	NLDVAKLNGD	TRUE	TRUE	TRUE	TRUE	TRUE	TRUE
17.1	Singleton	GIYYLNYDGFN	-	Mus Pep14	GIYYLNYDGFN	GIYYLNYDGFNTFTI		17.1	Singleton	GIYYLNYDGFN	-	Mus Pep14	GIYYLNYDGFN	TRUE	TRUE	TRUE	TRUE	TRUE	TRUE
18.1	Singleton	KTPEDGEYFVE	-	Rat Pep10	KTPEDGEYFVE	KTPEDGEYFVEYDGGNTFTI		18.1	Singleton	KTPEDGEYFVE	-	Rat Pep10	KTPEDGEYFVE	TRUE	TRUE	TRUE	TRUE	TRUE	TRUE
19.1	Singleton	LCEAHGITRDNI	-	Rat Pep11	LCEAHGITRDNI	LCEAHGITRDNIIDLTKTDR		19.1	Singleton	LCEAHGITRDNI	-	Rat Pep11	LCEAHGITRDNI	TRUE	TRUE	TRUE	TRUE	TRUE	TRUE
20.1	Singleton	TFTILKTDYDRY	-	Rat Pep18	TFTILKTDYDRY	TFTILKTDYDRYVMFHLINF		20.1	Singleton	TFTILKTDYDRY	-	Rat Pep18	TFTILKTDYDRY	TRUE	TRUE	TRUE	TRUE	TRUE	TRUE
21.1	Singleton	RELYLVAYKTPE	-	Rat Pep15	RELYLVAYKTPE	RELYLVAYKTPEDGEYFVEY		21.1	Singleton	RELYLVAYKTPE	-	Rat Pep15	RELYLVAYKTPE	TRUE	TRUE	TRUE	TRUE	TRUE	TRUE
22.1	Singleton	RIKENGECRELY	-	Rat Pep16	RIKENGECRELY	RIKENGECRELYLVAYKTPE		22.1	Singleton	RIKENGECRELY	-	Rat Pep16	RIKENGECRELY	TRUE	TRUE	TRUE	TRUE	TRUE	TRUE
23.1	Singleton	ASNKREKIEEN	-	Rat Pep1	ASNKREKIEEN	ASNKREKIEENGSMRVFMQH		23.1	Singleton	ASNKREKIEEN	-	Rat Pep1	ASNKREKIEEN	TRUE	TRUE	TRUE	TRUE	TRUE	TRUE
24.1	Singleton	EKALVSSVRQR	-	Mus Pep11	EKALVSSVRQR	EKALVSSVRQRMKCS		24.1	Singleton	EKALVSSVRQR	-	Mus Pep11	EKALVSSVRQR	TRUE	TRUE	TRUE	TRUE	TRUE	TRUE
25.1	Singleton	DDVVASEALNS	-	Mus Pep15	DDVVASEALNS	DDVVASEALNSVWSGF		25.1	Singleton	DDVVASEALNS	-	Mus Pep15	DDVVASEALNS	TRUE	TRUE	TRUE	TRUE	TRUE	TRUE
26.1	Singleton	ILGKLVKDYHLQ	-	Mus Pep18	ILGKLVKDYHLQ	ILGKLVKDYHLQFHR		26.1	Singleton	ILGKLVKDYHLQ	-	Mus Pep18	ILGKLVKDYHLQ	TRUE	TRUE	TRUE	TRUE	TRUE	TRUE
27.1	Singleton	IGGPDDGVITP	-	Mus Pep19	IGGPDDGVITP	IGGPDDGVITPWQSSF		27.1	Singleton	IGGPDDGVITP	-	Mus Pep19	IGGPDDGVITP	TRUE	TRUE	TRUE	TRUE	TRUE	TRUE
28.1	Singleton	DKETLSLEELKA	-	Mus Pep20	DKETLSLEELKA	DKETLSLEELKALLL		28.1	Singleton	DKETLSLEELKA	-	Mus Pep20	DKETLSLEELKA	TRUE	TRUE	TRUE	TRUE	TRUE	TRUE
29.1	Singleton	LQSGFYSLSSLV	-	Mus Pep21	LQSGFYSLSSLV	LQSGFYSLSSLVTVP		29.1	Singleton	LQSGFYSLSSLV	-	Mus Pep21	LQSGFYSLSSLV	TRUE	TRUE	TRUE	TRUE	TRUE	TRUE
30.1	Singleton	TIFISLFLLSVCY	-	Mus Pep23	TIFISLFLLSVCY	TIFISLFLLSVCYSA		30.1	Singleton	TIFISLFLLSVCY	-	Mus Pep23	TIFISLFLLSVCY	TRUE	TRUE	TRUE	TRUE	TRUE	TRUE
